# A semantic classification of nominal technical terms in secondary school biology textbooks

**DOI:** 10.1371/journal.pone.0312040

**Published:** 2024-11-11

**Authors:** Rurong Le, Sheng Yu, Xianhe Zhang

**Affiliations:** 1 School of Foreign Studies, Guangzhou University, Guangzhou, Guangdong, People’s Republic of China; 2 School of the English Language & Culture, Xiamen University Tan Kah Kee College, Zhangzhou, Fujian, People’s Republic of China; Indiana University Bloomington, UNITED STATES OF AMERICA

## Abstract

Nominal technical terms (NTTs), as crucial builders for disciplinary knowledge, can cause difficulties for students. However, previous studies have rarely associated NTTs with disciplinary knowledge construction. Apart from that, scholars of English for Specific Purposes (ESP) have mainly focused on making wordlists for one specific discipline based on corpora rather than on meanings. Moreover, the current categorization of technical terms cannot reveal their role in constructing disciplinary knowledge. Against this backdrop, we carried out a corpus-based study to classify NTTs in secondary school biology textbooks and to unveil the knowledge constructed by different types of those NTTs. As a result, we found that NTTs in those textbooks could fall into five major categories: Thing, Activity, Semiotic, Place, and Time. We also found intra-disciplinary differences in NTT distributions. The lexicogrammatical analysis indicates that the five types of NTTs can construct different knowledge. With the findings, we put forward implications for teaching biology in secondary schools.

## Introduction

Language has long been recognized as a crucial resource for learning disciplinary knowledge [1,2] and plays a vital role in students’ mastery of disciplinary-specific literacy [3]. While linguistic resources, such as metadiscourse [4], nominalizations [5,6], clause complex [7], and interpersonal resources [8] have been the focus of extensive scholarly attention, technical terms as a type of linguistic resource for constructing disciplinary knowledge have been comparatively underexplored. While such terms are crucial to students’ conceptual learning [9] and can cause cognitive load for students, particularly those at higher levels of study [10], only some researchers, mainly in the field of Systemic Functional Linguistics (SFL), have devoted their attention to this topic. Wignell [11], for example, revealed the gradual development of technical terms in the process of slowly constructing a hierarchical knowledge structure in economics. Doran [12] delved into the knowledge structure of physics and unveiled the process of knowledge construction by analyzing the taxonomic relations between technical terms and the activities contained in technical terms. In effect, linguistic resources, such as metadiscourse, do not directly build disciplinary knowledge, even though they do facilitate the expression of the propositional contents. In addition to that, the corpus-based method, while highlighting the distributional features of the linguistic resources studied in question, usually obscures disciplinary knowledge.

Technical terms have been investigated by ESP researchers in tertiary education contexts. They have made wordlists that benefit disciplines including medicine [13], economics [14,15], plumbing [16], and engineering [17] and that contribute to specialized educational contexts such as trades training [18], rugby instruction [19], and aviation radiotelephony communication [20]. There is also one study on secondary school wordlists made by Green & Lambert [21], who included eight secondary school subjects and developed word families and associations for the lexis. All these statistically-made unclassified wordlists, however, have dwarfed the meanings of technical terms when disciplinary knowledge is considered, let alone explicitating the bond between the meanings of terms and the content of a particular discipline [22]. Moreover, those wordlists are quite inclusive, and this inclusiveness can cause problems. Terms of multi-word units (MWUs), for example, engender more learning difficulties for students than single-word ones since the former type involves more semantic relations [23]. Furthermore, the existing wordlists include both nouns and verbs, which construe the human experience of things, qualities, and actions [24] and therefore require different ways to understand. Even nouns, such as ‘respiration’, can be derived from grammatical metaphors [25] or can reconstrue activity sequences [26], for example, ‘photosynthesis’. Classifying terms is also suggested as a strategy to improve students’ understanding of how language constructs scientific knowledge [27]. A proper classification of technical terms is thus needed.

An early classification of technical terms was made by Jacobson [28], who proposed three types of terminology based on the degree of the one-to-one correspondence between terms and realities and on the use of terminology in different contexts. Jacobson’s [28] categorization recognizes the existence of term variation but deviates from the standardization of terminology. A recent classification can be found in ESP studies, where the classification of technical terms is related to the identification of the terms. Considering levels of specialization, for example, Chung & Nation’s [29] four-scale rating and Ha and Hyland’s [14] Technicality Analysis Model can discriminate technical terms from less or the least technical ones. Besides, some researchers have used formal criteria. Drayton & Coxhead [20], for instance, put the aviation radiotelephony vocabulary into five categories: technical words, multi-word units, proper nouns, acronyms, and numbers. ESP researchers’ technicality-based categorizations, however, filter technical terms from non-technical ones.

Up to now, there has been no categorization of technical terms in relation to disciplinary knowledge and the present study is a trailblazer in this regard. In this study, we classify the nominal technical terms (NTTs) in secondary school biology textbooks. We draw upon Hao’s [26] entity system to categorize the NTTs from a discourse semantic perspective. In particular, we aim to address the following research questions:

1. How can NTTs in secondary school biology textbooks be classified according to Hao’s [26] entity system?
2. What are the intra-disciplinary distributions of the different types of NTTs in secondary school biology?
3. How do different types of NTTs build knowledge in secondary school biology textbooks?

## Theoretical framework

**Fig 1:**
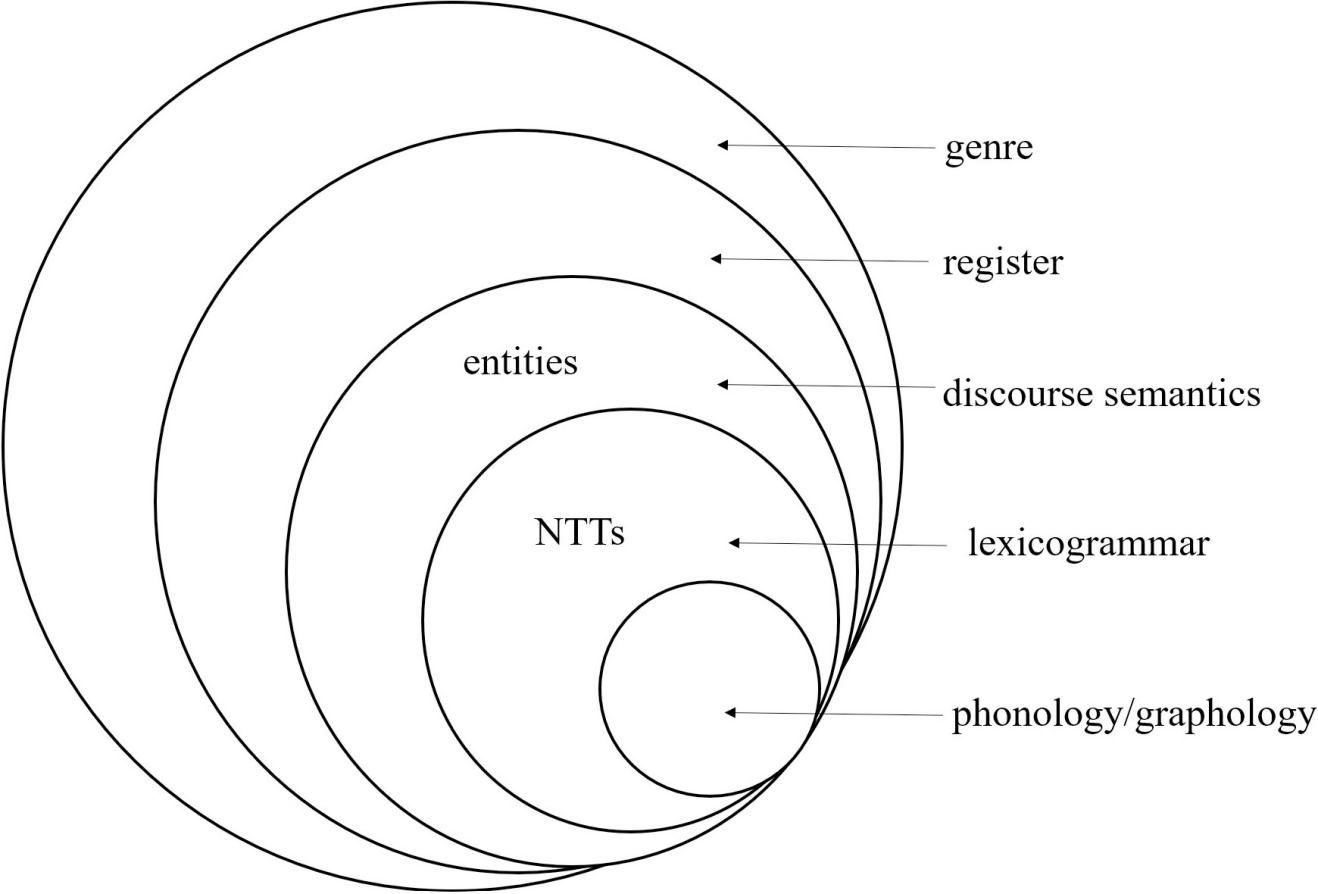
The stratification model (based on Martin [30]).

This study approaches the classification of NTTs from a discourse semantic perspective. Discourse semantics is included in SFL’s stratification model [30] (see Fig 1) where the meaning becomes more and more abstract upwards. In this model, Discourse semantics is at a higher stratum than Lexicogrammar, thus construing more abstract or generalized meaning.

In Fig 1, NTTs are the resources at the Lexicogrammar stratum and entities at the Discourse semantics stratum. Since entities are more abstract than the resources at the Lexicogrammar stratum, consistent semantic classification of NTTs can be guaranteed. This study employs Hao’s [26] biology-text-based system of entities to classify NTTs, because according to Hao [26], entities are the discourse semantic resources that can construe taxonomies.

Take *cell*, for example (Table 1). At the Discourse semantics stratum, the term *cell* realizes Thing entities. At the Lexicogrammar stratum, however, it plays several participant roles (token, actor, and minor participant) in different clauses, according to Halliday and Matthiessen’s [31] functional grammar. Fortunately, Hao’s [26] entity system can avoid this inconsistent categorization. In this study, *cell* is a Thing NTT, where ‘Thing’ means the entities realized by the term and is the semantic category of the term.

**Table 1:** A stratificational description of NTTs.

Strata	Semantic description	Words
Discourse semantics	thing entities	cell
Lexicogrammar	1) A *cell* is the basic unit…. (token) 2) All *cells* share the same …. (actor) 3) It controls … into and out of the *cell*. (minor participant) (Sub-corpus Cell)	cell

SFL also offers us a theoretical understanding of the relationship between disciplinary knowledge and linguistic resources. Knowledge is both a social-semiotic activity and a dynamically unfolding process, and the study of knowledge should be dependent on the linguistic system whereby knowledge is constructed [32]. It is, therefore, theoretically possible to lexicogrammatically analyze NTTs to find out the knowledge they construct.

## Methodology

### Data collection

Secondary school biology textbooks were collected by two criteria. First, the scope of learning levels or school years. Secondary school ranges from Years 7 to 12. Second, the quality of the textbooks. We only selected those published by world-renowned publishing houses, such as Oxford, McGraw Hill Education, and Pearson Education. As another quality guarantee, the textbooks we selected had been reviewed by secondary school biology teachers whose information can be found in the preface.

Finally, 9 textbooks were collected, all in PDF format. Five of them were biology textbooks while the other four were science textbooks in which biology was accompanied by physics, chemistry, and geography. The textbooks are listed in S1 Appendix.

### Building the corpora

The corpora were built in two steps. **Step 1**. Converting the PDF textbooks into editable Word documents. We used Adobe Acrobat Pro to make the PDF textbooks editable and then copied the main contents to Word documents. We did not, however, encompass everything in the textbooks.

First, visual elements such as images, pictures, and graphs were deleted, though we acknowledge their interaction with the running verbal texts in science textbooks. For one thing, because this is a mono-modal study of the NTTs in the running verbal texts, and thus a multi-modal study where images and graphs are analyzed would be beyond the scope of this study. For another, the deletion of the visual elements would not affect the semantic classification of NTTs because we follow Hao’s [26] completely grammar-based entity system (transitivity).

Second, output-oriented curriculum activities like Exercises, Chapter Reviews, and Deeper Understanding were excluded, leaving only the input-oriented passages of each chapter or section. On the one hand, these passages belong to factual genres usually used in science textbooks, such as reports, explanations, observations, and procedural recounts [33]. In these genres, biology NTTs play a central role in classification, description, recount, and interpretation. On the other hand, those output-oriented curriculum activities, which involve classroom interactions, may affect the proportions of NTTs used to describe or present biological facts. Leaving only the passages about biological facts will thus generate more statistically precise input-focused data.

**Step 2.** Dividing the textbooks into four topics. The biology textbooks were divided into several topics according to their common contents. After comparing the tables of contents of all the textbooks, we got four topics, which we named Cell, Ecosystem, Life Systems, and Genetics & Evolution. Accordingly, our corpora were composed of four sub-corpora. Table 2 presents the number of tokens of each sub-corpus and the main contents of each topic.

**Table 2:** Basic information about each sub-corpus.

	Cell	Ecosystem	Life Systems	Genetics & Evolution
**No. of tokens**	70,612	74,055	197,686	205,067
**Main contents**	cell structure and function, organization of living organism	biodiversity and ecosystem	structural organization of living organisms and physiological processes	DNA, genetic inheritance, evolution of species

### Retrieving nominal technical terms

For the reliability of the retrieval, two tools were employed. One was a webpage-based tool TermStat Web 3.0 [34], used as an NTT extractor. The other was a specialized dictionary, *A Dictionary of Biology* [35], used as a biology expert for consultation.

After the extraction, we deleted some lexical bundles like ‘type of cell’ and ‘series of reaction’ because such non-terminological bundles do not refer to any specific biological things or activities. However, we reserved the sub-types of the more general terms, for example, *plant cell*, and *animal cell*, although they are not listed in the dictionary. We also reserved all the candidates that included ‘theory, hypothesis, principle, and model’, since they indicate abstract meanings and can be difficult for students to learn. In the end, all the NTTs were put in four separate Excel documents, in descending order of frequencies. Table 3 shows the numbers and the frequencies of all the NTTs in the four sub-corpora.

**Table 3:** Numbers and frequencies of all the NTTs.

	Total numbers of NTTs	Total frequencies of NTTs
**Cell**	427	6,949
**Ecosystem**	550	8,263
**Life Systems**	1,292	19,774
**Genetics & Evolution**	846	15,861
**Total**	3,115	50,847

### Annotation of NTTs

The annotation was carried out in two steps. In the first step, the first author independently annotated all the NTTs in the Excel documents, following Hao’s [26] entity system. The NTTs were labeled with ‘Thing’, ‘Activity’, ‘Place’, ‘Semiotic’, and ‘Time’, excluding Hao’s [26] source entities because they were people and publications and could not be regarded as terms. Table 4 shows the five types and some examples of our annotation.

**Table 4:** Examples of annotation.

NTTs	Categories	Remarks
*cell wall*	(observational) Thing	biological things that can be observed by human beings through either bare eyes or instruments
*microscope*	(instrumental) Thing	instruments used in biological experiments
*photosynthesis*	(observational) Activity	biological activities that are done by biological things and can be observed by human beings
*Gram staining*	(enacted) Activity	biological experiments carried out by human beings
*tropical rain forest*	place	
*endosymbiont theory*	semiotic	
*Mesozoic era*	time	

Activity NTTs realize activity entities that encapsulate a series of activities [26,36]. Although the relations among activities can be either expected (temporal) or implicated (causal) [30], we chose not to consider this view to further categorize Activity NTTs. The reason is that it could be very difficult for us to decide whether the relations were expected or implicated since they could be different for the same Activity NTT in the textbooks.

During the annotation, we encountered some confusing cases, for instance, *anaphase* and *interphase*, two of the stages of cell division. However, the description of the two in the textbooks was fully based on various activities rather than time. According to the first author’s email communication with Hao, the two terms are activity NTTs from the perspective of discourse semantics and we accepted her opinion.

In the next step, 120 NTTs (30 from each sub-corpus) were selected for annotation by the second author, to ensure inter-rater reliability. Inter-rater reliability was then calculated by using Cohen’s kappa (κ) through SPSS 18. The Cohen’s Kappa coefficient was 0.914 and *p*. = .000, indicating a high level of agreement. We then discussed our disagreement and reached a consensus.

### Analytical methods

This study takes a mixed-method approach. The quantitative method is corpus-based, presenting the raw frequencies and the percentages of NTTs for comparison; the qualitative method is concerned with a lexicogrammatical analysis of the NTTs in clauses. To be specific, we employed Halliday and Matthiessen’s [31] transitivity to uncover the knowledge constructed by different types of NTTs.

## Results

### A general distribution

Table 5 displays the general distribution of the five major categories of NTTs across the four topics, with the raw frequencies and proportions. Among the five categories, Thing and Activity NTTs were two major ones used to construct biological knowledge. However, the proportions of Thing NTTs (over 80%) far surpassed those of Activity ones (ranging from 8.77% to 16.37%), suggesting that the Thing NTTs play a dominant role in constructing biological knowledge. Although the gap between Activity NTTs and Thing ones was enormous, the former also supported the construction of biological knowledge in our corpora.

**Table 5:** An overall distribution of NTTs.

	Activity	Thing	Place	Semiotic	Time	Total
**Cell**	1,045*	5,877	1	26	0	6,949
	15.04%	84.57%	0.01%	0.38%	0	100%
**Ecosystem**	725	6,626	**735**	**177**	0	8,263
	8.77%	80.19%	**8.89%**	**2.14%**	0	100%
**Life Systems**	1,947	**17,755**	16	56	0	19,774
	9.85%	**89.79%**	0.08%	0.28%	0	100%
**Genetics & Evolution**	**2,596**	12,904	76	111	**174**	15,861
	**16.37%**	81.35%	0.48%	0.70%	1.10%	100%
**Total**	6,313	43,162	828	370	174	50,847
	12.42%	84.87%	1.63%	0.73%	0.34%	100%

Note: For all topics, the numbers in their first rows refer to the frequencies of the type of NTTs; the percentages presented in their second rows were calculated based on the total frequencies of NTTs within each topic.

The other three categories, namely, Place, Semiotic, and Time NTTs were minor ones, accounting only for a small fraction. They were, however, the critical minorities that could distinguish between the four topics. Time NTTs, as a unique category to Genetics & Evolution, indicated that the knowledge of this topic also pertains to the geological time of the earth (Mesozoic era and Triassic period, for example). Place NTTs reached nearly 10% in Ecosystem, showing that they can differentiate Ecosystem from the other three topics. Others had less than 1%, indicating that locations or places are crucial knowledge in Ecosystem. Semiotic NTTs, on the other hand, were particularly preferred in Ecosystem and Genetics & Evolution.

There were also differences among the four topics. The frequencies showed that the topic of Life Systems was heavily dependent on Thing NTTs, topping those in the other three topics, in terms of both raw frequencies and percentages. The other three topics, however, witnessed fewer Thing NTTs, whose proportions were eroded by other types. Genetics & Evolution employed the most frequent Activity NTTs, followed by Cell, implying that the NTTs containing activities are more important in the two topics. Although Ecosystem employed the least Activity NTTs, its Place NTTs outnumbered the other three topics.

### The sub-types and major frequencies of NTTs

This subsection presents the sub-types of Thing, Activity, and Semiotic NTTs in Table 6 and the most frequent NTTs of each (sub)category in Table 7. The two tables unveil the nuances and the complexity of knowledge construction among the four topics.

**Table 6:** The sub-types of Activity, Thing, and Semiotic NTTs.

Major types	Sub-types	Cell	Ecosystem	Life Systems	Genetics & Evolution
**Activity NTTs**	enacted	7/0.10%*	34/0.41%	19/0.10%	**293/1.84%**
	observational	**1,038/14.94%**	691/8.36%	1,928/9.75%	2,303/14.53%
**Thing NTTs**	instrumental	**163/2.31%**	14/0.17%	37/0.19%	77/0.49%
	observational	5,714/82.23%	6,612/80.02%	**17,718/89.60%**	12,827/80.86%
**Semiotic NTTs**	theory	20/0.29%	0	11/0.06%	**58/0.37%**
	model	6/0.09%	**169/2.05%**	45/0.23%	47/0.30%
	hypothesis	0	2/0.02%	0	**6/0.04%**
	principle	0	**6/0.07%**	0	0

Note: The data in Table 6 report the frequencies/percentages of each subcategory. The percentages were calculated based on the total number of NTTs within each topic. The highest percentages are in bold type.

**Table 7:** The top ten most frequent NTTs of each category in each topic*.

Topics Categories	Cell	Ecosystem	Life Systems	Genetics & Evolution
**observational Thing NTTs**	cell (1152) energy (446) membrane (142) cell membrane (125) plasma membrane (105) DNA (104) nucleus (101) glucose (83) bacteria (69) organism (67)	organism (577) population (574) ecosystem (488) plant (389) species (282) animal (259) biodiversity (144) community (139) nutrient (95) predator (86)	plant (1035) animal (696) heart (343) organ (335) bone (303) sperm (227) lung (214) skin (206) mammal (198) brain (185)	gene (979) chromosome (844) plant (819) DNA (737) allele (582) animal (473) offspring (349) phenotype (273) gamete (221) amino acid (212)
**instrumental Thing NTTs**	microscope (115) microscopy (22) electron microscopy (6) radioactive isotope (5) light microscopy (4)	isotope (11) carbon isotope (3)	microscope (16) telescope (11) optical instrument (5)	microscope (17) dye (13) DNA microarrays (9) fluorescent dye (6) bacteriophage (5)
**observational Activity NTTs**	photosynthesis (129) diffusion (91) cellular respiration (84) mitosis (79) cell cycle (49) cell division (49) glycolysis (46) interphase (42) prophase (26) osmosis (26)	population growth (55) photosynthesis (44) precipitation (27) predation (26) climate change (25) parasitism (20) adaptation (17) secondary succession (17) symbiosis (16) migration (16)	reproduction (134) photosynthesis (122) fertilization (121) infection (86) contraction (66) cell division (65) immunity (58) respiration (57) life cycle (50) meiosis (49)	evolution (280) meiosis (216) natural selection (151) fertilization (139) mitosis (104) genetic variation (82) cell division (73) transcription (59) synthesis (57) sexual reproduction (45)
**enacted Activity NTTs**	autoradiography (5) Gram staining (2)	quadrat (16) sampling (15) carbon dating (3)	dialysis (10) vaccination (9)	cloning (83) genetics (49) gene therapy (37)
**Semiotic NTTs**	cell theory (18) fluid mosaic model (6) endosymbiont theory (2)	food chain (80) food web (60) ecological pyramid (9) edge effect (8) competitive exclusion principle (4) growth curve (3)	evolutionary tree (33) cladogram (12) sliding filament theory (5) transpiration-cohesion-tension theory (4) germ theory (2)	cladogram (27) law of segregation (14) phylogenetic tree (13) theory of natural selection (9) punctuated equilibrium (9) chromosome map (7)
**Place NTTs**	rainforest (1)	habitat (235) forest (166) rain forest/rainforest (104) desert (47) wetland (20) tropical rain forest (20) taiga (12) woodland (12) photic zone (12) prairie (10)	freshwater habitat (6) temperate region (4)	habitat (59) rainforest (13) tropical forest (4)
**Time NTTs**	N/A	N/A	N/A	Precambrian (25) Mesozoic era (21) Permian (17) Cenozoic era (16) Cretaceous period (12) Ordovician (9) Triassic period (9) Proterozoic eon (9) Silurian (7) Jurassic Period (7)

Note: Categories of NTTs that do not exceed ten are exhausted. For a full list of all the NTTs across the four topics, refer to S1 Table.

As indicated by Table 6, the sub-types of Activity, Thing, and Semiotic NTTs were also distributed differently among the four topics. In general, these three categories had their own dominating sub-types.

Since the NTTs of all the topics were dominated by Thing NTTs (see Table 5), it may be interesting to check the details from a zooming-in perspective. As the results in Table 6 show, Thing NTTs were further dominated by observational Thing NTTs, exceeding 80% in all the topics. Life Systems, however, still owned the most frequent observational Thing NTTs. Although the other sub-type, instrumental Thing NTTs, accounted for a minute proportion (2.31%) in Cell, it still far surpassed those in Genetics & Evolution (nearly 0.5%) and those in Ecosystem and Life Systems (less than 0.2%).

The dominance of observational Thing NTTs suggests that the things represented by these terms, visible through bare eyes or instruments, comprise the major content and are the most attention-deserving objects of study in secondary biology classrooms. Meanwhile, the instrumental ones were a minority because the things represented by them facilitated the study of biology. They were the pragmatic tools utilized by biologists to observe and do experiments with those observational things.

When it comes to Activity NTTs, they were categorized into observational and enacted ones, with proportion features quite similar to those of the sub-categories of Thing NTTs. The observational ones were dominant while enacted ones were much less used in all four topics, indicating that biology at secondary school levels stresses the knowledge of observable biological activities.

Observational Activity NTTs and observational Thing NTTs were similarly dominant since the former are by definition about processes involving the latter. When the latter is in the majority, the former naturally becomes the main part of Activity NTTs. The same is true for enacted Activity NTTs and instrumental Thing NTTs because instrumental Thing NTTs represent the tools and instruments utilized in biological experiments represented by enacted Activity NTTs.

Semiotic NTTs were also unevenly distributed among the four sub-types. Only the model sub-type could be found in all four topics and the other three sub-types in some. Another feature was that the dominating sub-types were model ones, theory ones, or even both. Specifically, theory ones were dominant in Cell and model ones in Ecosystem. Genetics & Evolution mostly used theory and model sub-types; Life Systems only these two.

In addition to the frequencies and percentages, we also list the top ten most frequent NTTs for each topic (Table 7).

Table 7 presents the top ten most frequently used NTTs of each (sub)category, with their frequencies in parentheses. We can easily find intra-disciplinary differences, particularly manifested by Thing, Activity, and Semiotic NTTs. The reason may be that the four topics are concerned with their own biological things and activities and therefore biologists propose corresponding theories or models to explain and summarize these phenomena. In other words, the four topics rarely share highly frequent NTTs.

We can see from Table 7 that Time NTTs were exclusively used in Genetics & Evolution, making this topic distinctive from the other three. Place NTTs were most salient in Ecosystem which by nature encompasses numerous locations.

A comparison of the frequencies showed that the observational Thing NTTs were always the most crucial in each topic, followed by observational Activity NTTs. In particular, Ecosystem and Life Systems relied more on the former than the latter, since the proportion of the tenth most frequent observational Thing NTTs in Ecosystem (86) and in Life Systems (185) exceeded those of the most frequent observational Activity NTTs in Ecosystem (55) and in Life Systems (134). In addition, the top Place NTTs (235) and Semiotic ones (80) were more favored than the top observational Activity ones in Ecosystem. In Genetics & evolution, the most frequent observational Activity NTTs barely squeezed into the top 10. Meanwhile, the top 10 observational Thing and Activity NTTs had similar frequencies in Cell; all the other categories of NTTs were much less used.

Notably, some of these top frequent NTTs were used across the topics. Although the top ten observational Thing NTTs were quite distinctive among the four topics, indicating that the four topics can be clearly differentiated by these terms, some other types of NTTs showed that the four topics shared something in common. *Microscope*, one of the other sub-categories of Thing NTTs, was shared by three of the four topics (except Ecosystem), implying that the tool is fundamentally important in learning secondary biology. When it comes to Activity NTTs, some of the top ten observational Activity NTTs, such as *photosynthesis*, *respiration*, *mitosis*, *cell division*, occurred across Cell, Life Systems, and/or Genetics & Evolution. The same is true for some Semiotic NTTs. For example, *cladogram*, *phylogenetic tree*, and *evolutionary tree* were used by Life Systems and Genetics & Evolution.

Interestingly, only one Place NTT *rainforest* appeared in Cell. This term, according to the sub-corpus Cell, was mentioned in a section titled “Cell requirements” where the environments for living organisms were introduced.

### Knowledge construction via NTTs

#### Thing NTTs

As shown above, Thing NTTs played a dominating role in constructing biology knowledge in our corpora. Observational Thing NTTs were found to be tokens, actors, possessors, and minor participants in clauses, implying that they can construct the knowledge of features, functions, and compositions of biological things (see Examples 1–4). All the terms to be linguistically analyzed are in italics; they are not so, however, when they are used semantically.

(1) Example 1 *Mitochondria* are rod-shaped organelles with …. (token, relational clause) (Sub-corpus Cell)
(2) Example 2 In this process, *glucose* and oxygen react to form water, …. (actor, material clause) (Sub-corpus Cell)
(3) Example 3 Many *prokaryotes* have small hair-like projections called *pili*, …. (possessor, possessed, possessive clause) (Sub-corpus Cell)
(4) Examples 4 cytoplasm—the ‘jelly-like’ fluid inside the *cell* between the *membrane* and the *nucleus*. (minor participant, prepositional phrase) (Sub-corpus Cell)

Relational clauses are used for characterization and identification [31]. In Example 1, the Thing NTT *mitochondria* is a token, and the “rod-shaped organelles” are the values about the shape of mitochondria. In Example 2, *glucose* is an actor, indicating the functions of the thing glucose.

Examples 3 and 4, however, construct the knowledge of composition, or a part-whole relationship. In Example 3, *prokaryotes* is the possessor, and *pili* is possessed by *prokaryotes*, showing that pili is a part of prokaryotes. In Example 4, the Thing NTTs *cell*, *membrane*, and *nucleus* are minor participants used after the prepositions ‘inside’ and ‘between’, indicating circumstantial meaning in the clause. Nevertheless, it does not mean they are places or locations but the prepositions indicate the relative positions among cell, membrane, and nucleus. This is also a part-whole relationship where the membrane and the nucleus are parts of a cell.

The other sub-category, instrumental Thing NTTs, can be goals and minor participants in clauses. These participant roles highlight their function of being tools (see Examples 5 and 6).

(5) Example 5 The *compound light microscope* is used to observe thin slices of specimens, …. (goal, passive material clause) (Sub-corpus Cell)
(6) Example 6 Major structures within individual cells can be seen with a *compound light microscope*. (minor participant) (Sub-corpus Cell)

The two examples show that instrumental Thing NTTs construct the knowledge of the thing being tools. In Example 5, *compound light microscope* is the goal in the passive material clause. In Example 6, the term plays the same participant role as that in Example 4. The difference, however, is that in Example 6, the preposition ‘with’ introduces a tool or method term while the prepositions in Example 4 introduce location terms.

#### Activity NTTs

Activity NTTs can construct the knowledge of a series of activities that involve Thing NTTs. Activity NTTs realize activity entities, which, according to Hao [26,36], reconstrue a series of activities at the stratum of Register (field) (see Tables 8 and 9). The two sub-types of Activity NTTs construct different knowledge in the corpora. Observational ones are used for biological phenomena but enacted ones for procedures of biological experiments. This section cites *endocytosis* and *autoradiography* as examples.

**Table 8:** A stratificational interpretation of *endocytosis* (Sub-corpus Cell).

Stratum	Ideational resources	Example
Register (field)	a series of activities	…, (1) a small area of the *cell membrane* sinks inwards to form a pocket. …, (2) materials near the cell membrane are enclosed by the *membrane*, which then… (3) pinches off to form a *vesicle*. (4) The *vesicle* then transports the substance to ….
Discourse semantics	entities	observational Activity
Lexicogrammar	NTTs	*endocytosis*

**Table 9:** A stratificational interpretation of *autoradiography* (Sub-corpus Cell).

Stratum	Ideational resources	Example
Register (field)	a series of activities	The *tissue* (1) is first treated with… (2) is then taken up to … (3) is then sliced into… (4) is then placed against … (5) is then stained to …
Discourse semantics	entities	enacted Activity
Lexicogrammar	NTTs	*autoradiography*

From Tables 8 and 9, we can see that at the Register (field) stratum, a set of activities is reconstrued by the entities at the Discourse semantics stratum. In turn, the entities are realized by Activity NTTs at the Lexicogrammar stratum.

Although the process is the same, the distinctions between observational and enacted ones can be attributed to the clauses that realize the activity series. In both tables, the activities are all realized by material clauses that describe doings and happenings [31], yet by active ones in Table 8 and passive ones in Table 9. Also, the actors and goals in these clauses are different. In Table 8, both the actors and the goals are observational Thing NTTs. In Table 9, however, the actors that initiate the activities are omitted and the goal *tissue* is used as the subject of the clauses. Indeed, the omitted actors are human beings who are doing experiments.

Accordingly, the kind of knowledge built by the two sub-categories of Activity NTTs is also distinctive. Observational ones encapsulate the activities done by biology-related things and describe the happenings observable in the biological world. Such kind of knowledge makes up the major content for students because they need to learn how the biology activities take place and learn to explain these processes. In terms of enacted ones, the series of activities are experimental instructions or procedures, with which students can perform experiments by themselves.

In addition, the participant roles of Activity NTTs show that they can build more complex knowledge than Thing ones. Activity NTTs can be actors, goals, or tokens (see examples 7–9). In these examples, the activity sequences encapsulated in the NTTs are taken as a whole, and new knowledge is built upon them in the clauses.

(7) Example 7 Animals cells … and do not perform *photosynthesis*. (goal, material clause) (Sub-corpus Cell)
(8) Example 8 *Photosynthesis* is critically important for life on Earth. (token, relational clause) (Sub-corpus Cell)
(9) Example 9 *Sexual reproduction* results in increased genetic diversity because …. (actor, material clause) (Sub-corpus Genetics & Evolution)

In Example 8, the Activity NTT *photosynthesis* is taken as a whole and functions as a token to which the value “critically important” is assigned. In other words, the completion of the whole process is important. Activity NTTs can also perform actions or be acted upon, like the two material clauses in examples 7 and 9.

### Semiotic NTTs

This category of NTTs is unique compared with the other categories. They do not represent the material world, but human thought and non-material things constructed by signs. In our corpora, theory, principle, and hypothesis Semiotic NTTs usually act as sayers in verbal clauses, which can be told by the verbs in the clauses. The lexicogrammatical features of model ones are similar to those of Thing and Activity NTTs.

(10) Example 10 The *cell theory* states that all organisms are composed of one or more cells. (sayer, verbal clause) (Sub-corpus Cell)
(11) Example 11 The *intermediate disturbance hypothesis* proposes that high and low levels of …. (sayer, verbal clause) (Sub-corpus Ecosystem)
(12) Example 12 According to the *law of segregation*, the two factors for each trait segregate or separate from each other …. (minor participant) (Sub-corpus Genetics & Evolution)

The above three examples deal with the participant roles of theory, hypothesis, and principle Semiotic NTTs in clauses. Examples 10 and 11 are verbal clauses, in which the two verbs ‘states’ and ‘proposes’ have the meaning of saying and the that-clauses are the content of the theory or hypothesis. The theories and hypotheses are saying or reporting biologists’ ideas or thoughts. Although in Example 12 the theory Semiotic NTT is a minor participant, the prepositional phrase ‘according to’ represents the source of saying.

The knowledge built by theory, hypothesis, and principle Semiotic NTTs is focused on the content expressed by them. The content represents biologists’ possible explanations for or generalization of biological phenomena, which can result in predictions that are based on observations and experiments [37].

Model Semiotic NTTs, on the other hand, show quite similar lexicogrammatical features to those of Activity and Thing NTTs, although they are usually represented by diagrams or figures in the textbooks. Model Semiotic NTTs can be tokens and goals in clauses.

(13) Example 13 Energy is passed through ecosystems via *food chains* and *food webs*. (minor participant, circumstance) (Sub-corpus Ecosystem)
(14) Example 14 *Detritivore and decomposer food chains* are most abundant in forests. (token, relational clause) (Sub-corpus Ecosystem)

In Example 13, the preposition ‘via’ signifies the means or methods used, and therefore the knowledge built here is about the functions of food webs and chains. The model Semiotic NTT *detritivore and decomposer food chains* is a token in Example 14 where the comparative and evaluative features are assigned to the two food chains respectively. The knowledge built here is about the features of the models.

### Time and Place NTTs

Time NTTs represent a period in the geographical history and they play the role of minor participants in the circumstance of a clause. When the characteristics of the period are discussed, Time NTTs can be tokens and goals in relational and material clauses where the features of the period are assigned to the tokens.

(15) Example 15 During the *Mesozoic era*, Earth gradually rebounded from the toll of the Permian. (minor participant, circumstance) (Sub-corpus Genetics & Evolution)
(16) Example 16 The *Precambrian* accounts for about 87 percent of Earth’s history…. (token, relational clause) (Sub-corpus Genetics & Evolution)

In Example 15, the Time NTT *Mesozoic era* follows the temporal preposition ‘during’, and the main clause is about the activity of Earth that took place in this period. Example 16 focuses on the features of the period called Precambrian. The Time NTT *Precambrian* is a token and its value is its numerical proportion in the Earth’s history.

As for Place NTTs, they represent not only spatial meanings realized by minor participants in circumstances but also the characteristics associated with the locations.

(17) Example 17 In a *rain forest*, a tree grows from nutrients released by decomposers. (minor participant, circumstance) (Sub-corpus Ecosystem)
(18) Example 18 *Tropical rain forests* have warm temperatures, wet weather, and lush plant growth. (possessor, possessive relational clause) (Sub-corpus Ecosystem)

In Example 17, the Place NTT *rain forest* indicates a location since it is the minor participant in the circumstance that contains the location preposition *in*. The next two examples present the traits of tropical rain forest in two relational clauses. The clause in Example 18 is a possessive relational one and the Place NTT *tropical rain forest* is the possessor of ‘warm temperatures, wet weather, and lush plant growth’.

## Discussions

### Classification of NTTs

Our wordlist includes five major categories of NTTs, namely, Thing, Activity, Semiotic, Place, and Time. Our quantitative analysis indicates intra-disciplinary distributional variations of the NTTs among the four biology sub-topics of Cell, Ecosystem, Life Systems, and Genetics & Evolution. Moreover, our study unveils the knowledge constructed by each type of NTTs through qualitative analysis of their participant roles in different clauses.

Our study contributes to the current research on specialized wordlists. First, our wordlist remedies the situation where the only concern over statistical factors downplays the meaning of NTTs [13] and different parts of speech and forms of technical terms are mixed [13,16]. Second, our lexicogrammatical analysis reveals the knowledge constructed by each kind of NTT. Through such an analysis, our study relates knowledge construction with linguistic resources, instead of reducing to a mere quantitative description of linguistic resources that only facilitate knowledge construction. Our lexicogrammatical analysis is also a response to the criticism that ESP studies on wordlists have ignored the lexical or grammatical features of the wordlists [38,39]. Third, our quantitative analysis, which shows varied distributions of the five types of NTTs among the four topics, may draw attention to intra-disciplinary differences.

### Pedagogical implications

Learning a specialized linguistic resource, like NTTs, is the final stage of literacy development for middle school and high school students [3]. Our input-oriented, fact-targeted research is particularly conducive to enculturing students into discipline-specific, output-oriented knowledge practices. In this section, we offer some implications for both language teachers and biology teachers to apply our categorization to output-oriented classroom teaching settings.

First, for observational Thing NTTs, which are dominant in all four biological topics, we suggest that teachers pair students to make up dialogues [40] incorporating such NTTs taught in a specific biology lesson, so that they can better apprehend the meanings of such NTTs and develop the grammar related to them. According to our transitivity analysis, we also advise teachers to focus on the various features related to biological things. They can, for example, offer clauses containing such NTTs as contexts for students to understand the usages of the NTTs since these features are important knowledge represented by the functional roles played by such NTTs in different clauses. Considering the critical role of clauses, we suggest that teachers should prepare teaching materials with clauses that contain observational Thing NTTs and use such materials to help students practice those NTTs in class. One of the teaching activities can be a match game where the observational Thing NTTs are replaced with blanks and these NTTs act as candidates (Table 10).

**Table 10:** A match game example.

Candidates	Clauses with blanks
cytoplasm	____________ do not have any true organelles but still contain DNA.
cell	___________ is the powerhouse of the cell.
Mitochondria	___________ contains all the cell organelles (mini organs), dissolved nutrients and wastes, and helps provide structure for the cell.
bacteria	A ___________ is the basic unit of life.

A more intriguing yet challenging activity is to prepare, at the end of each chapter, section or unit, a crossword puzzle containing adequate clauses that provide definitions of observational Thing NTTs. Teamwork may also help if the puzzle is excessively difficult for students at lower language levels. Since a crossword puzzle can stimulate thinking and increase vocabulary [41], a purposefully designed one for observational Thing NTTs can be of some, if not great, help.

Notably, the topic of Life Systems possesses the most observational Thing NTTs and students may get confused when faced with so many terms, for example, the Participant errors made by students’ writing [27]. Considering the features of the knowledge in this topic, we think that teachers can outline or highlight the compositional relationships among these terms. For instrumental Thing NTTs, teachers may incorporate such instruments as microscopy [42] to strengthen students’ command of such terms when students do experiments. A teaching example can be a hands-on activity of showing students how to use a microscope to observe the structures of a plant cell and an animal cell, and then arrange for the students to orally report their structural differences using some top-frequent NTTs (like in Table 7) they have learned. In this way, students may master key NTTs and relevant biological knowledge more efficiently.

Second, for Activity NTTs, teachers may help students unpack the series of activities involved in a specific NTT [43]. Unpacking them into activity sequences should be the initial step. After that, teachers need to repack them as a whole and recontextualize them in different clauses, because our lexicogrammatical analysis finds out that first, Activity NTTs can be taken as a whole, and second, they can acquire functional features and perform actions to bring about more biological activities. The above suggestion also applies to all of the four topics because Thing NTTs plus Activity ones account for the overwhelming majority of all the NTTs in the four topics. *Endocytosis* is taken as a concrete example here. The term *endocytosis* represents a series of activities that take place in time order (see Table 8). Biology teachers can first mess up the sequence of the activities and then ask students to reorder them and highlight the biological things involved in the activities, for example, plasma membrane. After that, teachers can encourage students to make sentences by using the term *endocytosis*, and a sample sentence can be “The cell uses endocytosis to move large molecules into it”. Such an interesting way of teaching may help students internalize the NTT and relevant biological knowledge relaxingly.

Third, despite less frequency of use, the other three types of NTTs have unique features and therefore we also give our suggestions. Digital story-telling is one of the approaches that can facilitate students’ learning of both Time and Place NTTs, since using digital story-telling in EFL students’ learning science knowledge has proven effective [44]. Teachers may encourage students to make short videos to tell biology-themed stories to describe a certain period of geological time using Time and/or Place NTTs. This suggestion also works for the topics of Ecosystem and Genetics & evolution, as the former topic features the most frequent Place NTTs and the latter exclusively owns Time NTTs. When it comes to Semiotic NTTs, teachers need to help students apply or testify them in specific situations, by choosing a particular case either from their textbooks or from daily life, because theories are useful when students react to specific instances [45]. These methods also apply to the teaching of Ecosystem and Genetics & evolution, which contain more Semiotic NTTs than the other two topics do.

Besides, for each category, teachers also need to pay special attention to the NTTs that occur across the topics, especially those top frequent ones. For example, *cell division* and *photosynthesis* used in Cell can be taken as a connection for, or transition to, new knowledge in Genetics & Evolution and Life Systems.

Interestingly, teachers and students might be purposefully guided by our categorization in their daily teaching and learning, if textbook editors or curriculum developers could organize, in our way, the categorized NTT wordlists at the end of textbooks or in the curriculum, instead of the traditional alphabetically ordered wordlists.

Despite the implications, we failed to list as many examples as may be wanted due to the lenght limit. A manual of NTT-classification-based suggestions for secondary school teachers may be complied under a large-scale interdisciplinary project involving teachers of biology and the English language.

## Conclusion

In this study, we classified NTTs from a discourse semantic perspective by adopting Hao’s [26] entity system. From secondary school biology textbooks, we discovered that NTTs can be categorized into Thing, Activity, Semiotic, Place, and Time and that the former three types could be further classified. We also found intra-disciplinary differences among the four topics of Cell, Ecosystem, Life Systems, and Genetics & Evolution, across which the five categories of NTTs showed varied distribution patterns. Moreover, through our lexicogrammatical analysis, we found that the five types of NTTs could build different knowledge.

These findings contribute to a growing body of literature on knowledge construction and technical terms. Given the central role of technical terms in knowledge construction, it is necessary to empirically describe their discourse meanings, upon which the technical terms are classified in the real context. Thus, our corpus-based method and lexicogrammatical analysis make it possible for us to depict knowledge construction from the inside of a discipline and differentiate the various topics within it.

Future studies may expand the current research by exploring the relationships among different types of NTTs from three directions: 1) in the form of schema since (nominal) technical terms are interrelated [9]; 2) from the perspective of field relations formed by (nominal) technical terms since such relations underpin the organization of scientific knowledge, such as classification, composition, and activities [46]; 3) from a multimodal perspective to see how semiotic resources like images, graphs, and pictures build disciplinary knowledge [47].

## Supporting information

S1 Table (PDF)

S1 Appendix (PDF)

S1 File Cell. (DOCX)

S2 File Ecosystem. (DOCX)

S3 File Genetics & evolution. (DOCX)

S4 File Life systems. (DOCX)
