# Supplementary material for: A semantic classification of nominal technical terms in secondary school biology textbooks
Source: PLoS One. 2024 Nov 11;19(11):e0312040. doi: 10.1371/journal.pone.0312040 (PMC11554214; doi:10.1371/journal.pone.0312040)
Supplement: S1 Table — (PDF) [file pone.0312040.s001.pdf]

CELL		
**NTTs**	**Frequencies**	**Categories**
cell	1152	Thing: observational
energy	446	Thing: observational
membrane	142	Thing: observational
photosynthesis	129	Activity: observational
cell membrane	125	Thing: observational
microscope	115	Thing: instrumental
plasma membrane	105	Thing: observational
DNA	104	Thing: observational
nucleus	101	Thing: observational
diffusion	91	Activity: observational
cellular respiration	84	Activity: observational
glucose	83	Thing: observational
mitosis	79	Activity: observational
bacteria	69	Thing: observational
organism	67	Thing: observational
cytoplasm	65	Thing: observational
cell wall	63	Thing: observational
eukaryotic cell	62	Thing: observational
plant cell	55	Thing: observational
cell cycle	49	Activity: observational
cell division	49	Activity: observational
animal cell	48	Thing: observational
glycolysis	46	Activity: observational
prokaryotic cell	46	Thing: observational
nutrient	43	Thing: observational
interphase	42	Activity: observational
phosphate	42	Thing: observational
fungus	42	Thing: observational
pigment	41	Thing: observational
chlorophyll	40	Thing: observational
amino acid	39	Thing: observational
substrate	37	Thing: observational
enzyme	36	Thing: observational
phospholipid	35	Thing: observational
endoplasmic reticulum	35	Thing: observational
lysosome	34	Thing: observational
vesicle	33	Thing: observational
skin	30	Thing: observational
prokaryote	30	Thing: observational
eukaryote	30	Thing: observational
multicellular organism	30	Thing: observational
nucleic acid	30	Thing: observational
organic compound	29	Thing: observational
flagellum	28	Thing: observational
spindle	28	Thing: observational
Golgi apparatus	28	Thing: observational
chromosome	27	Thing: observational
covalent bond	27	Thing: observational
prophase	26	Activity: observational
cilium	26	Thing: observational
osmosis	26	Activity: observational
eukaryotes	26	Thing: observational
mammal	26	Thing: observational
concentration gradient	26	Thing: observational
tissue	25	Thing: observational
cytokinesis	25	Activity: observational

prokaryotes	24	Thing: observational
stem	24	Thing: observational
membrane-bound organelle	24	Thing: observational
sister chromatid	24	Thing: observational
organelle	23	Thing: observational
archaea	23	Thing: observational
autotrophs	23	Thing: observational
aerobic respiration	23	Activity: observational
centriole	22	Thing: observational
protist	22	Thing: observational
microscopy	22	Thing: instrumental
stem cell	22	Thing: observational
vacuole	21	Thing: observational
exocytosis	21	Activity: observational
adaptation	21	Activity: observational
vitamin	21	Thing: observational
unicellular organism	21	Thing: observational
homologous chromosome	21	Thing: observational
fatty acid	21	Thing: observational
homeostasis	20	Activity: observational
cellulose	20	Thing: observational
cytosol	19	Thing: observational
telophase	19	Activity: observational
cytoskeleton	19	Thing: observational
meiosis	19	Activity: observational
isotope	19	Thing: observational
neutron	19	Thing: observational
fat	19	Thing: observational
lipid	18	Thing: observational
organ	18	Thing: observational
solvent	18	Thing: observational
cell theory	18	Semiotic: theory
endocytosis	17	Activity: observational
solute	17	Thing: observational
transport protein	17	Thing: observational
daughter cell	17	Thing: observational
blood cell	17	Thing: observational
anaphase	16	Activity: observational
pyruvate	16	Thing: observational
yeast	16	Thing: observational
polymer	16	Thing: observational
nitrogen	16	Thing: observational
metaphase	15	Activity: observational
centromere	15	Thing: observational
phylum	15	Thing: observational
starch	15	Thing: observational
cholesterol	15	Thing: observational
spindle fiber	15	Thing: observational
chloroplast	14	Thing: observational
adenosine	14	Thing: observational
invertebrate	14	Thing: observational
nucleotide	14	Thing: observational
liver	14	Thing: observational
stomach	14	Thing: observational
nerve	14	Thing: observational
species	14	Thing: observational
thylakoid	14	Thing: observational
light-independent reaction	14	Activity: observational

electron transport chain	14	Thing: observational
anaerobic respiration	14	Activity: observational
mitochondrion	13	Thing: observational
muscle	13	Thing: observational
heterotrophs	13	Thing: observational
metabolism	13	Activity: observational
skeleton	13	Thing: observational
kidney	13	Thing: observational
lung	13	Thing: observational
urine	13	Thing: observational
urea	13	Thing: observational
carrier protein	13	Thing: observational
red blood cell	13	Thing: observational
lactic acid fermentation	13	Activity: observational
skin cell	13	Thing: observational
muscle cell	13	Thing: observational
stroma	12	Thing: observational
gamete	12	Thing: observational
pump	12	Thing: observational
light-dependent reaction	12	Activity: observational
cell organelle	12	Thing: observational
organ system	12	Thing: observational
thylakoid membrane	12	Thing: observational
epidermis	11	Thing: observational
plastid	11	Thing: observational
coenzyme	11	Thing: observational
chromatin	11	Thing: observational
digestive enzyme	11	Thing: observational
photosystem	10	Thing: observational
pore	10	Thing: observational
chloride	10	Thing: observational
digestion	10	Activity: observational
syrup	10	Thing: observational
rough endoplasmic reticulum	10	Thing: observational
phospholipid bilayer	10	Thing: observational
citric acid	10	Thing: observational
lactic acid	10	Thing: observational
fermentation	9	Activity: observational
dermis	9	Thing: observational
cisternae	9	Thing: observational
grana	9	Thing: observational
ionic bond	9	Thing: observational
nuclear membrane	9	Thing: observational
phospholipid molecule	9	Thing: observational
electron carrier	9	Thing: observational
algae	8	Thing: observational
glycerol	8	Thing: observational
apoptosis	8	Activity: observational
stain	8	Thing: observational
intestine	8	Thing: observational
nuclear envelope	8	Thing: observational
hypertonic solution	8	Thing: observational
activation energy	8	Thing: observational
cancer cell	8	Thing: observational
adult stem cell	8	Thing: observational
carotenoid	7	Thing: observational
amoeba	7	Thing: observational
ecosystem	7	Thing: observational

cyclin	7	Thing: observational
cell plate	7	Thing: observational
cell compartmentalisation	7	Activity: observational
citric acid cycle	7	Activity: observational
spindle apparatus	7	Thing: observational
net movement	7	Activity: observational
sister chromatid	7	Thing: observational
adenosine triphosphate	7	Thing: observational
parent cell	7	Thing: observational
muscle tissue	7	Thing: observational
bacterial cell	7	Thing: observational
protein synthesis	7	Activity: observational
nerve cell	7	Thing: observational
nervous system	7	Thing: observational
gland	6	Thing: observational
monosaccharide	6	Thing: observational
inhibitor	6	Thing: observational
thylakoids	6	Thing: observational
chemiosmosis	6	Activity: observational
cyanobacteria	6	Thing: observational
phosphorylation	6	Activity: observational
polysaccharide	6	Thing: observational
herbicide	6	Thing: observational
cork	6	Thing: observational
potassium	6	Thing: observational
mRNA	6	Thing: observational
permeable membrane	6	Thing: observational
fluid mosaic model	6	Semiotic: model
hypotonic solution	6	Thing: observational
isotonic solution	6	Thing: observational
hydrogen bond	6	Thing: observational
electron microscopy	6	Thing: instrumental
body system	6	Thing: observational
digestive system	6	Thing: observational
blood vessel	6	Thing: observational
dynamic equilibrium	6	Activity: observational
glycogen	5	Thing: observational
microorganism	5	Thing: observational
nephron	5	Thing: observational
sperm	5	Thing: observational
chitin	5	Thing: observational
amylase	5	Thing: observational
disaccharide	5	Thing: observational
autoradiography	5	Activity: enacted
biomolecules	5	Thing: observational
microfilaments	5	Thing: observational
peptidoglycan	5	Thing: observational
smooth endoplasmic reticulum	5	Thing: observational
channel protein	5	Thing: observational
eukaryotic organism	5	Thing: observational
plant enzyme	5	Thing: observational
hydrophobic tail	5	Thing: observational
pyruvic acid	5	Thing: observational
embryonic stem	5	Thing: observational
photosynthetic organism	5	Thing: observational
chemical bond	5	Thing: observational
radioactive isotope	5	Thing: instrumental
plasma	4	Thing: observational

vertebrate	4	Thing: observational
gill	4	Thing: observational
proteasomes	4	Thing: observational
photolysis	4	Activity: observational
protease	4	Thing: observational
acetyl CoA	4	Thing: observational
excretion	4	Activity: observational
filtrate	4	Thing: observational
appendage	4	Thing: observational
tadpole	4	Thing: observational
ammonia	4	Thing: observational
phosphorus	4	Thing: observational
haemoglobin	4	Thing: observational
buffer	4	Thing: observational
secretion	4	Activity: observational
colloid	4	Thing: observational
mesophyll cell	4	Thing: observational
binary fission	4	Activity: observational
peptide bond	4	Thing: observational
embryonic stem cell	4	Thing: observational
motor protein	4	Thing: observational
light microscopy	4	Thing: instrumental
fatty acid tail	4	Thing: observational
mitochondrial matrix	4	Thing: observational
alcoholic fermentation	4	Activity: observational
osmotic gradient	4	Thing: observational
DNA replication	4	Activity: observational
double-stranded DNA	4	Thing: observational
gram-negative bacterium	4	Thing: observational
adenosine diphosphate	4	Thing: observational
synthesis of protein	4	Activity: observational
biological system	4	Thing: observational
circulatory system	4	Thing: observational
sodium chloride	4	Thing: observational
sequence of amino	4	Thing: observational
deoxyribonucleic acid	4	Thing: observational
sexual reproduction	4	Activity: observational
life cycle	4	Activity: observational
sooty grunter	4	Thing: observational
organ system	4	Thing: observational
bacterium	3	Thing: observational
ribosome	3	Thing: observational
adenine	3	Thing: observational
offspring	3	Thing: observational
phycobiliproteins	3	Thing: observational
pili	3	Thing: observational
cyclins	3	Thing: observational
thermophiles	3	Thing: observational
synapsis	3	Activity: observational
cristae	3	Thing: observational
turgor	3	Activity: observational
epithelium	3	Thing: observational
pleat	3	Thing: observational
amphibian	3	Thing: observational
carcinogen	3	Thing: observational
sucrose	3	Thing: observational
fertilization	3	Activity: observational
filtration	3	Activity: observational

excretory organ	3	Thing: observational
surface-area-to-volume ratio	3	Thing: observational
semipermeable membrane	3	Thing: observational
transmission electron	3	Thing: observational
vascular plant	3	Thing: observational
processing of protein	3	Thing: observational
heterotrophic cell	3	Thing: observational
mitochondrial membrane	3	Thing: observational
protein production	3	Activity: observational
bile pigment	3	Thing: observational
glycogen synthase	3	Thing: observational
prokaryotic organism	3	Thing: observational
confocal microscopy	3	Thing: instrumental
membrane-bound organelle	3	Thing: observational
thermal energy	3	Thing: observational
ocular lens	3	Thing: observational
confocal microscope	3	Thing: instrumental
stereo microscope	3	Thing: instrumental
pigment chlorophyll	3	Thing: observational
anabolic pathway	3	Thing: observational
irreversible inhibitor	3	Thing: observational
protein channel	3	Thing: observational
integral protein	3	Thing: observational
sugar solution	3	Thing: observational
amino acid chain	3	Thing: observational
final electron acceptor	3	Thing: observational
cellular transport	3	Thing: observational
yeast cell	3	Thing: observational
third phosphate group	3	Thing: observational
lipid synthesis	3	Activity: observational
concentration of enzyme	3	Activity: observational
oxygen nucleus	3	Thing: observational
amino group	3	Thing: observational
fatty acid tail	3	Thing: observational
secretory vesicle	3	Thing: observational
competitive inhibition	3	Activity: observational
placental mammal	3	Thing: observational
transport vesicle	3	Thing: observational
enzyme-substrate complex	3	Thing: observational
skeletal muscle	3	Thing: observational
anaerobic pathway	3	Thing: observational
egg cell	3	Thing: observational
metabolic pathway	3	Thing: observational
ribonucleic acid	3	Thing: observational
carboxyl group	3	Thing: observational
liver cell	3	Thing: observational
human cell	3	Thing: observational
brain cell	3	Thing: observational
tubule	2	Thing: observational
leucoplast	2	Thing: observational
ferrodoxin	2	Thing: observational
chromoplast	2	Thing: observational
nucleoid	2	Thing: observational
rubisco	2	Thing: observational
acidophile	2	Thing: observational
extremophiles	2	Thing: observational
xylem	2	Thing: observational
dephosphorylation	2	Activity: observational

papillae	2	Thing: observational
glucagon	2	Thing: observational
oxyhaemoglobin	2	Thing: observational
paramecium	2	Thing: observational
halophiles	2	Thing: observational
maltose	2	Thing: observational
pinocytosis	2	Activity: observational
glycoproteins	2	Thing: observational
endosome	2	Thing: observational
triglyceride	2	Thing: observational
sap	2	Thing: observational
lignin	2	Thing: observational
kinase	2	Thing: observational
pepsin	2	Thing: observational
guanine	2	Thing: observational
cytosine	2	Thing: observational
on-membrane-bound organell	2	Thing: observational
bond of organic compound	2	Thing: observational
transport chain	2	Thing: observational
nicotinamide adenine	2	Thing: observational
nephron tubule	2	Thing: observational
begonia plant	2	Thing: observational
singular mitochondrion	2	Thing: observational
transmembrane protein	2	Thing: observational
nicotinarnide adenine	2	Thing: observational
hydrogen chloride	2	Thing: observational
feedback inhibition	2	Activity: observational
membrane system	2	Thing: observational
peripheral protein	2	Thing: observational
cylindrical structure	2	Thing: observational
epidermal cell	2	Thing: observational
flavin adenine dinucleotide	2	Thing: observational
adaptive pathway	2	Thing: observational
cell recognition	2	Activity: observational
enzyme-substrate interaction	2	Activity: observational
tertiary structure	2	Thing: observational
excretory structure	2	Thing: observational
microscopic organism	2	Thing: observational
homogeneous mixture	2	Thing: observational
subcutaneous fat layer	2	Thing: observational
nuclear pore	2	Thing: observational
autotrophic cell	2	Thing: observational
green pigment chlorophyll	2	Thing: observational
vesicle membrane	2	Thing: observational
glucose polymer	2	Thing: observational
regulatory mechanism	2	Activity: observational
aster fiber	2	Thing: observational
phagocytic cell	2	Thing: observational
enzyme concentration	2	Activity: observational
diploid cell	2	Thing: observational
muscle fiber	2	Thing: observational
primary filtrate	2	Thing: observational
yellow pigment	2	Thing: observational
protein complex	2	Thing: observational
accessory pigment	2	Thing: observational
endosymbiont theory	2	Semiotic: theory
glucose concentration	2	Activity: observational
plant vacuole	2	Thing: observational

adenosine monophosphate	2	Thing: observational
compound light microscope	2	Thing: instrumental
dinucleotide phosphate	2	Thing: observational
fungal cell	2	Thing: observational
integumentary system	2	Thing: observational
non-vascular plant	2	Thing: observational
haploid gamete	2	Thing: observational
secondary structure	2	Thing: observational
enzyme production	2	Activity: observational
unsaturated fat	2	Thing: observational
pollen grain	2	Thing: observational
membrane protein	2	Thing: observational
gram-positive bacterium	2	Thing: observational
haploid cell	2	Thing: observational
asexual reproduction	2	Activity: observational
cell differentiation	2	Activity: observational
sex cell	2	Thing: observational
ribose sugar	2	Thing: observational
glycerol backbone	2	Thing: observational
ATP synthase	2	Thing: observational
Gram staining	2	Activity: enacted
rainforest	1	Place
sperm cell	1	Thing: observational

ECOSYSTEM		
**NTTs**	**Frequencies**	**Categories**
organism	577	Thing: observational
population	574	Thing: observational
ecosystem	488	Thing: observational
plant	389	Thing: observational
species	282	Thing: observational
animal	259	Thing: observational
habitat	235	Place
forest	166	Place
biodiversity	144	Thing: observational
community	139	Thing: observational
nutrient	95	Thing: observational
predator	86	Thing: observational
bacterium	83	Thing: observational
food chain	80	Semiotic: model
disease	77	Thing: observational
nitrogen	75	Thing: observational
abiotic factor	70	Thing: observational
insect	66	Thing: observational
atmosphere	64	Thing: observational
microorganism	63	Thing: observational
food web	60	Semiotic: model
population growth	55	Activity: observational
consumer	52	Thing: observational
biosphere	50	Thing: observational
producer	49	Thing: observational
rain forest	76	Place
algae	48	Thing: observational
fungus	47	Thing: observational
photosynthesis	44	Activity: observational
trophic level	44	Thing: observational
parasite	41	Thing: observational
seed	41	Thing: observational
crop	41	Thing: observational
decomposer	40	Thing: observational
herbivore	40	Thing: observational
desert	47	Place
earth	38	Thing: observational
prey	35	Thing: observational
toad	34	Thing: observational
pollution	34	Thing: observational
autotrophs	33	Thing: observational
mammal	33	Thing: observational
cat	30	Thing: observational
coral reef	28	Thing: observational
precipitation	27	Activity: observational
predation	26	Activity: observational
tundra	26	Thing: observational
coral	25	Thing: observational
mouse	25	Thing: observational
climate change	25	Activity: observational
heterotrophs	24	Thing: observational
glucose	24	Thing: observational
rainforest	28	Place
fertiliser	23	Thing: observational
pond	23	Thing: observational
biotic factor	23	Thing: observational

phosphorus	22	Thing: observational
invasive species	22	Thing: observational
carnivore	21	Thing: observational
kangaroo	21	Thing: observational
cane	21	Thing: observational
parasitism	20	Activity: observational
lichen	20	Thing: observational
wetland	20	Place
tropical rain forest	20	Place
root	19	Thing: observational
biological community	19	Thing: observational
hare	18	Thing: observational
fox	18	Thing: observational
pest	18	Thing: observational
beetle	17	Thing: observational
plankton	17	Thing: observational
dung	17	Thing: observational
adaptation	17	Activity: observational
secondary succession	17	Activity: observational
plant species	17	Thing: observational
quadrat	16	Activity: enacted
symbiosis	16	Activity: observational
shrub	16	Thing: observational
mosquito	16	Thing: observational
offspring	16	Thing: observational
migration	16	Activity: observational
cow	16	Thing: observational
competitor	16	Thing: observational
commensalism	15	Activity: observational
lynx	15	Thing: observational
grazing	15	Activity: observational
flower	15	Thing: observational
sampling	15	Activity: enacted
natural ecosystem	15	Thing: observational
population growth rate	15	Thing: observational
cane toad	15	Thing: observational
respiration	14	Activity: observational
koala	14	Thing: observational
sediment	14	Thing: observational
erosion	14	Activity: observational
mineral	14	Thing: observational
dog	14	Thing: observational
cactus	13	Thing: observational
squirrel	13	Thing: observational
rabbit	13	Thing: observational
protein	13	Thing: observational
density-dependent factor	13	Thing: observational
primary succession	13	Activity: observational
death rate	13	Thing: observational
eutrophication	12	Activity: observational
taiga	12	Place
detritivores	12	Thing: observational
eucalypt	12	Thing: observational
anemone	12	Thing: observational
moth	12	Thing: observational
emigration	12	Activity: observational
woodland	12	Place
deer	12	Thing: observational

bioprospecting	12	Activity: observational
aquatic ecosystem	12	Thing: observational
photic zone	12	Place
isotope	11	Thing: instrumental
toxin	11	Thing: observational
frog	11	Thing: observational
elephant	11	Thing: observational
rat	11	Thing: observational
cattle	11	Thing: observational
whale	11	Thing: observational
immigration	11	Activity: observational
habitat fragmentation	11	Activity: observational
human population growth	11	Activity: observational
species diversity	11	Thing: observational
climax community	11	Thing: observational
habitat loss	11	Activity: observational
ecological niche	11	Thing: observational
mutualism	10	Activity: observational
cowbird	10	Thing: observational
protozoon	10	Thing: observational
seedling	10	Thing: observational
birth rate	10	Thing: observational
equilibrium	10	Activity: observational
degradation	10	Activity: observational
pathogen	10	Thing: observational
prairie	10	Place
ant	10	Thing: observational
mortality	10	Thing: observational
marsh	10	Thing: observational
bear	10	Thing: observational
wildlife	10	Thing: observational
consumption	10	Activity: observational
animal species	10	Thing: observational
genetic diversity	10	Thing: observational
midden	9	Thing: observational
dingo	9	Thing: observational
urchin	9	Thing: observational
steelhead	9	Thing: observational
aphid	9	Thing: observational
ferret	9	Thing: observational
worm	9	Thing: observational
wolf	9	Thing: observational
horn	9	Thing: observational
pollutant	9	Thing: observational
exponential growth	9	Activity: observational
species extinction	9	Activity: observational
ecological pyramid	9	Semiotic: model
intertidal zone	9	Place
selection pressure	9	Thing: observational
life cycle	9	Activity: observational
heterotroph	8	Thing: observational
grasshopper	8	Thing: observational
fern	8	Thing: observational
pollen	8	Thing: observational
phosphate	8	Thing: observational
intestine	8	Thing: observational
humidity	8	Thing: observational
glacier	8	Thing: observational

larva	8	Thing: observational
buffalo	8	Thing: observational
breeding	8	Activity: observational
trunk	8	Thing: observational
lion	8	Thing: observational
trout	8	Thing: observational
local extinction	8	Activity: observational
nitrogen cycle	8	Activity: observational
dung beetle	8	Thing: observational
edge effect	8	Semiotic: model
density-independent factor	8	Thing: observational
temperate forest	8	Place
mass extinction	8	Activity: observational
sea urchin	8	Thing: observational
genetic variation	8	Activity: observational
bird species	8	Thing: observational
skink	7	Thing: observational
cyanobacteria	7	Thing: observational
chlorophyll	7	Thing: observational
acidification	7	Activity: observational
rhinoceros	7	Thing: observational
nectar	7	Thing: observational
reptile	7	Thing: observational
hawk	7	Thing: observational
bison	7	Thing: observational
turtle	7	Thing: observational
pesticide	7	Thing: observational
lawn	7	Thing: observational
marine biome	7	Thing: observational
deciduous forest	7	Place
invasive species	7	Thing: observational
biotic and abiotic factor	7	Thing: observational
pest species	7	Thing: observational
marine ecosystem	7	Thing: observational
clownfish	6	Thing: observational
sclerophyll	6	Thing: observational
pollinator	6	Thing: observational
tapeworm	6	Thing: observational
sulfur	6	Thing: observational
organelle	6	Thing: observational
crustacean	6	Thing: observational
mangrove	6	Thing: observational
savanna	6	Thing: observational
acorn	6	Thing: observational
traps	6	Thing: observational
beaver	6	Thing: observational
fossil	6	Thing: observational
swamp	6	Thing: observational
litter	6	Thing: observational
moss	6	Thing: observational
arrow	6	Thing: observational
fertilizer	6	Thing: observational
owl	6	Thing: observational
crab	6	Thing: observational
contamination	6	Activity: observational
shrimp	6	Thing: observational
herd	6	Thing: observational
snail	6	Thing: observational

long-term cycle	6	Activity: observational
abiotic and biotic factor	6	Thing: observational
sea anemone	6	Thing: observational
native species	6	Thing: observational
eucalypt tree	6	Thing: observational
algal bloom	6	Activity: observational
acid precipitation	6	Activity: observational
intraspecific competition	6	Activity: observational
oxygen cycle	6	Activity: observational
prairie dog	6	Thing: observational
energy flow	6	Thing: observational
biological control	6	Activity: observational
flow of energy	6	Thing: observational
salt marsh	6	Thing: observational
water cycle	6	Activity: observational
flowering plant	6	Thing: observational
zooxanthellae	5	Thing: observational
detritivore	5	Thing: observational
salamander	5	Thing: observational
sulfide	5	Thing: observational
triage	5	Thing: observational
omnivore	5	Thing: observational
earthworm	5	Thing: observational
overexploitation	5	Activity: observational
wombat	5	Thing: observational
phytoplankton	5	Thing: observational
catfish	5	Thing: observational
invertebrate	5	Thing: observational
cyclone	5	Thing: observational
clam	5	Thing: observational
silt	5	Thing: observational
nitrate	5	Thing: observational
shoreline	5	Thing: observational
weed	5	Thing: observational
snake	5	Thing: observational
mass extinction	5	Activity: observational
cellular respiration	5	Activity: observational
land degradation	5	Activity: observational
sclerophyll plant	5	Thing: observational
boreal forest	5	Place
aphotic zone	5	Place
black-footed ferret	5	Thing: observational
plant species	5	Thing: observational
brown-headed cowbird	5	Thing: observational
biogeochemical cycle	5	Activity: observational
interspecific competition	5	Activity: observational
endemic species	5	Thing: observational
aquatic plant	5	Thing: observational
snowshoe hare	5	Thing: observational
feedback loop	5	Activity: observational
possum	4	Thing: observational
amensalism	4	Activity: observational
macroinvertebrates	4	Thing: observational
protist	4	Thing: observational
mantis	4	Thing: observational
calcification	4	Activity: observational
grevillea	4	Thing: observational
ponderosa	4	Thing: observational

bushfire	4	Thing: observational
quandong	4	Thing: observational
pollination	4	Activity: observational
ladybugs	4	Thing: observational
lamprey	4	Thing: observational
cilium	4	Thing: observational
kookaburra	4	Thing: observational
flagellum	4	Thing: observational
permafrost	4	Thing: observational
lemming	4	Thing: observational
emu	4	Thing: observational
grub	4	Thing: observational
ethanol	4	Thing: observational
elk	4	Thing: observational
deforestation	4	Activity: observational
scavenger	4	Thing: observational
detritus	4	Thing: observational
pelican	4	Thing: observational
raccoon	4	Thing: observational
seaweed	4	Thing: observational
falcon	4	Thing: observational
panda	4	Thing: observational
evaporation	4	Activity: observational
rodent	4	Thing: observational
moose	4	Thing: observational
spider	4	Thing: observational
dinosaur	4	Thing: observational
pine	4	Thing: observational
canopy	4	Thing: observational
yeast	4	Thing: observational
potassium	4	Thing: observational
red squirrel	4	Thing: observational
ocean acidification	4	Activity: observational
temperate zone	4	Place
grey squirrel	4	Thing: observational
organic fertiliser	4	Thing: observational
benthic zone	4	Place
hydrogen sulfide	4	Thing: observational
tropical zone	4	Place
short-term cycle	4	Activity: observational
seasonal forest	4	Place
tropical savanna	4	Thing: observational
ompetitive exclusion principl	4	Semiotic: principle
coral cover	4	Thing: observational
first trophic level	4	Thing: observational
temperate deciduous forest	4	Place
brown pelican	4	Thing: observational
green algae	4	Thing: observational
ecological succession	4	Activity: observational
mammal species	4	Thing: observational
aquatic biome	4	Thing: observational
terrestrial biome	4	Thing: observational
feral cat	4	Thing: observational
coral polyp	4	Thing: observational
photosynthetic organism	4	Thing: observational
reef ecosystem	4	Thing: observational
plant tissue	4	Thing: observational
water buffalo	4	Thing: observational

selective breeding	4	Activity: observational
nitrogen-fixing bacterium	4	Thing: observational
giant panda	4	Thing: observational
exotic species	4	Thing: observational
tropical rainforest	4	Place
tectonic plate	4	Thing: observational
peregrine falcon	4	Thing: observational
negative feedback	4	Activity: observational
marine organism	4	Thing: observational
cell wall	4	Activity: observational
epiphyte	3	Thing: observational
ginkgo	3	Thing: observational
extremophiles	3	Thing: observational
housefly	3	Thing: observational
savannah	3	Thing: observational
transpiration	3	Activity: observational
quolls	3	Thing: observational
stromatolite	3	Thing: observational
autotroph	3	Thing: observational
krill	3	Thing: observational
cactorum	3	Thing: observational
thylacine	3	Thing: observational
natality	3	Thing: observational
hypha	3	Thing: observational
magpie	3	Thing: observational
humus	3	Thing: observational
songbird	3	Thing: observational
chloroplast	3	Thing: observational
amphibian	3	Thing: observational
orchid	3	Thing: observational
cellulose	3	Thing: observational
foliage	3	Thing: observational
stubble	3	Thing: observational
sprawl	3	Thing: observational
sleet	3	Thing: observational
goldfish	3	Thing: observational
tadpole	3	Thing: observational
mating	3	Activity: observational
lizard	3	Thing: observational
shellfish	3	Thing: observational
fauna	3	Thing: observational
dispersion	3	Activity: observational
heartworm	3	Thing: observational
ammonia	3	Thing: observational
mat	3	Thing: observational
pelagic zone	3	Place
limnetic zone	3	Place
aquatic organism	3	Thing: observational
photosynthetic autotrophs	3	Thing: observational
arid area	3	Place
third-order consumer	3	Activity: observational
habitat degradation	3	Activity: observational
climate zone	3	Place
polar zone	3	Place
phosphorus cycle	3	Activity: observational
ponderosa pine	3	Thing: observational
tropical seasonal forest	3	Place
growth curve	3	Semiotic: model

species of plant	3	Thing: observational
first-order consumer	3	Thing: observational
rhinoceros horn	3	Thing: observational
golden toad	3	Thing: observational
white-tailed deer	3	Thing: observational
terrestrial ecosystem	3	Thing: observational
musky rat kangaroo	3	Thing: observational
brood parasitism	3	Activity: observational
cactus moth	3	Thing: observational
eastern grey kangaroo	3	Thing: observational
eucalypt forest	3	Place
enhanced greenhouse effect	3	Semiotic: model
reef species	3	Thing: observational
green wedge	3	Thing: observational
aquatic insect	3	Thing: observational
passenger pigeon	3	Thing: observational
epicormic bud	3	Thing: observational
carbon cycle	3	Activity: observational
carbon isotope	3	Thing: instrumental
root system	3	Thing: observational
fruit fly	3	Thing: observational
insect larva	3	Thing: observational
marine animal	3	Thing: observational
fire ant	3	Thing: observational
bacterial cell	3	Thing: observational
fish species	3	Thing: observational
black bear	3	Thing: observational
carbon dating	3	Activity: enacted
cattails	2	Thing: observational
diatom	2	Thing: observational
millipedes	2	Thing: observational
beech-maple	2	Thing: observational
marsupial	2	Thing: observational
copepod	2	Thing: observational
microhabitat	2	Place
seastar	2	Thing: observational
fossilisation	2	Activity: observational
tuatara	2	Thing: observational
pampas	2	Thing: observational
zooplankton	2	Thing: observational
lithosphere	2	Thing: observational
broadleaf	2	Thing: observational
coelacanth	2	Thing: observational
wallaby	2	Thing: observational
ballart	2	Thing: observational
hydrosphere	2	Thing: observational
mudja	2	Thing: observational
mold	2	Thing: observational
roundworm	2	Thing: observational
microflora	2	Thing: observational
wattle	2	Thing: observational
r-strategist	2	Thing: observational
k-strategist	2	Thing: observational
termite	2	Thing: observational
weasel	2	Thing: observational
chub	2	Thing: observational
endemic	2	Thing: observational
tectonics	2	Thing: observational

coyote	2	Thing: observational
reproduction rate	2	Thing: observational
species of whale	2	Thing: observational
faecal pellet	2	Thing: observational
competitive exclusion principle	2	Semiotic: principle
third trophic level	2	Thing: observational
african teak tree	2	Thing: observational
ermediate disturbance hypothe	2	Semiotic: hypothesis
leaf litter	2	Thing: observational
photosynthetic algae	2	Thing: observational
native beetle	2	Thing: observational
cow pat	2	Thing: observational
bacterium species	2	Thing: observational
cane beetle	2	Thing: observational
food dye	2	Thing: observational
membrane-bound nucleus	2	Thing: observational
secondary consumer	2	Thing: observational
moth grub	2	Thing: observational
crop species	2	Thing: observational
logistic growth model	2	Semiotic: model
hawaiian dung beetle	2	Thing: observational
biological magnification	2	Activity: observational
freshwater habitat	2	Place
underwater habitat	2	Place
prickly pear cactus	2	Thing: observational
brown tree snake	2	Thing: observational
canadian lynx	2	Thing: observational
j-shaped growth curve	2	Semiotic: model
sea lamprey	2	Thing: observational
host bird	2	Thing: observational
beech-maple forest	2	Place
specie of insect	2	Thing: observational
forest ecosystem	2	Thing: observational
microscopic organism	2	Thing: observational
predator-prey food chain	2	Thing: observational
transitional aquatic ecosystem	2	Thing: observational
pyramid of energy	2	Semiotic: model
spruce tree	2	Thing: observational
deciduous tree	2	Thing: observational
cushion plant	2	Thing: observational
arctic fox	2	Thing: observational
sea star	2	Thing: observational
taiga community	2	Thing: observational
broadleaf ballart	2	Thing: observational
ginkgo tree	2	Thing: observational
rainforest habitat	2	Place
transitional ecosystem	2	Thing: observational
abyssal zone	2	Place
littoral zone	2	Place
land biome	2	Thing: observational
humpback chub	2	Thing: observational
species of microorganism	2	Thing: observational
rainforest ecosystem	2	Thing: observational
freshwater biome	2	Thing: observational
pest species	2	Thing: observational
coral calcification	2	Activity: observational
vegetation cover	2	Thing: observational
nitrogen fixation	2	Activity: observational

marine fish	2	Thing: observational
spider species	2	Thing: observational
indicator species	2	Thing: observational
nitrogen fertilizer	2	Thing: observational
profundal zone	2	Place
hydrothermal vent	2	Thing: observational
weedy plant	2	Thing: observational
spray zone	2	Thing: observational
membrane-bound organelle	2	Thing: observational
shallow-rooted plant	2	Thing: observational
mangrove tree	2	Thing: observational
second-order consumer	2	Thing: observational
autotrophic organism	2	Thing: observational
optimum zone	2	Place
desert biome	2	Thing: observational
specific pollinator	2	Thing: observational
species of bacterium	2	Thing: observational
population dynamics	2	Activity: observational
dominant species	2	Thing: observational
desert plant	2	Thing: observational
digestive enzyme	2	Thing: observational
host species	2	Thing: observational
seed disperser	2	Thing: observational
prey species	2	Thing: observational
top predator	2	Thing: observational
marine species	2	Thing: observational
migratory bird	2	Thing: observational
coniferous tree	2	Thing: observational
soil bacterium	2	Thing: observational
soil erosion	2	Activity: observational

LIFE SYSTEMS		
**NTTs**	**Frequencies**	**Categories**
plant	1035	Thing: observational
animal	696	Thing: observational
heart	342	Thing: observational
organ	335	Thing: observational
bone	303	Thing: observational
sperm	227	Thing: observational
lung	214	Thing: observational
skin	206	Thing: observational
mammal	198	Thing: observational
brain	185	Thing: observational
blood cell	171	Thing: observational
pathogen	167	Thing: observational
embryo	154	Thing: observational
nervous system	154	Thing: observational
arthropod	147	Thing: observational
protein	135	Thing: observational
reproduction	134	Activity: observational
bacterium	134	Thing: observational
mouth	131	Thing: observational
eye	128	Thing: observational
seed	125	Thing: observational
blood vessel	125	Thing: observational
neuron	123	Thing: observational
photosynthesis	122	Activity: observational
reptile	122	Thing: observational
fertilization	121	Activity: observational
gametophyte	120	Thing: observational
hair	120	Thing: observational
pollen	108	Thing: observational
kidney	107	Thing: observational
circulatory system	107	Thing: observational
echinoderm	105	Thing: observational
predator	104	Thing: observational
stem	102	Thing: observational
intestine	99	Thing: observational
enzyme	99	Thing: observational
impulse	96	Thing: observational
cord	95	Thing: observational
artery	95	Thing: observational
organism	93	Thing: observational
sporophyte	93	Thing: observational
ear	93	Thing: observational
amphibian	88	Thing: observational
vascular tissue	88	Thing: observational
infection	86	Activity: observational
red blood cell	86	Thing: observational
prey	85	Thing: observational
hormone	84	Thing: observational
membrane	83	Thing: observational
glucose	82	Thing: observational
fetus	79	Thing: observational
gill	79	Thing: observational
arm	79	Thing: observational
foot	78	Thing: observational
vertebrate	77	Thing: observational
fiber	76	Thing: observational

leaf	75	Thing: observational
stomach	75	Thing: observational
vein	72	Thing: observational
leg	68	Thing: observational
uterus	68	Thing: observational
contraction	66	Activity: observational
cell division	65	Activity: observational
cavity	63	Thing: observational
liver	62	Thing: observational
digestive system	61	Thing: observational
xylem	60	Thing: observational
epidermis	58	Thing: observational
immunity	58	Activity: observational
cell wall	58	Thing: observational
respiration	57	Activity: observational
vascular plant	57	Thing: observational
fat	54	Thing: observational
nucleus	51	Thing: observational
phloem	51	Thing: observational
ventricle	50	Thing: observational
sponge	50	Thing: observational
life cycle	50	Activity: observational
meiosis	49	Activity: observational
zygote	48	Thing: observational
haemoglobin	46	Thing: observational
digestive tract	46	Thing: observational
white blood cell	46	Thing: observational
offspring	45	Thing: observational
ovary	44	Thing: observational
lymph	44	Thing: observational
flowering plant	44	Thing: observational
stomata	43	Thing: observational
respiratory system	43	Thing: observational
exoskeleton	42	Thing: observational
nonvascular plant	42	Thing: observational
spinal cord	42	Thing: observational
atrium	41	Thing: observational
placenta	41	Thing: observational
tube foot	41	Thing: observational
menstrual cycle	41	Activity: observational
immune system	38	Thing: observational
mucus	37	Thing: observational
multicellular organism	37	Thing: observational
transpiration	36	Activity: observational
metabolism	36	Activity: observational
homeostasis	35	Activity: observational
invertebrate	35	Thing: observational
mesoderm	34	Thing: observational
urine	34	Thing: observational
bilateral symmetry	34	Thing: observational
reproductive system	34	Thing: observational
lever	33	Thing: observational
virus	33	Thing: observational
pregnancy	33	Activity: observational
seed plant	33	Thing: observational
evolutionary tree	33	Semiotic: model
parenchyma cell	33	Thing: observational
connective tissue	33	Thing: observational

host	32	Thing: observational
anthophytes	32	Thing: observational
pollination	32	Activity: observational
ovule	31	Thing: observational
mitosis	31	Activity: observational
dermis	30	Thing: observational
anus	30	Thing: observational
cartilage	30	Thing: observational
pollen grain	30	Thing: observational
skull	29	Thing: observational
estrogen	29	Thing: observational
central nervous system	29	Thing: observational
skeletal muscle	29	Thing: observational
lymphatic system	29	Thing: observational
plant cell	29	Thing: observational
body cell	29	Thing: observational
branch	28	Thing: observational
abdomen	28	Thing: observational
endocrine system	28	Thing: observational
internal organ	28	Thing: observational
muscle cell	28	Thing: observational
diaphragm	27	Thing: observational
coelom	27	Thing: observational
cellulose	27	Thing: observational
algae	27	Thing: observational
insulin	27	Thing: observational
cellular respiration	27	Activity: observational
female gametophyte	27	Thing: observational
metamorphosis	26	Activity: observational
pancreas	26	Thing: observational
vagina	26	Thing: observational
plasma membrane	26	Thing: observational
polyp	25	Thing: observational
auxin	25	Thing: observational
retina	25	Thing: observational
notochord	25	Thing: observational
heterotrophs	25	Thing: observational
endoderm	25	Thing: observational
nose	25	Thing: observational
placental mammal	25	Thing: observational
large intestine	25	Thing: observational
capillary	24	Thing: observational
larva	24	Thing: observational
axon	24	Thing: observational
throat	24	Thing: observational
excretion	24	Activity: observational
radial symmetry	24	Thing: observational
smooth muscle	24	Thing: observational
sea urchin	24	Thing: observational
cytoplasm	23	Thing: observational
t cell	23	Thing: observational
digestive enzyme	23	Thing: observational
body tissue	23	Thing: observational
immune response	23	Activity: observational
thyroid gland	23	Thing: observational
tongue	22	Thing: observational
gastrula	22	Thing: observational
hypothalamus	22	Thing: observational

ovulation	22	Activity: observational
trachea	22	Thing: observational
egg cell	22	Thing: observational
pituitary gland	22	Thing: observational
tracheid	21	Thing: observational
autotrophs	21	Thing: observational
cochlea	21	Thing: observational
pharynx	21	Thing: observational
reproductive structure	21	Thing: observational
sensory neuron	21	Thing: observational
invertebrate chordate	21	Thing: observational
vegetative reproduction	21	Activity: observational
body cavity	21	Thing: observational
pollen tube	21	Thing: observational
middle ear	21	Thing: observational
cell membrane	21	Thing: observational
motor neuron	21	Thing: observational
cone	20	Thing: observational
antigen	20	Thing: observational
follicle	20	Thing: observational
gut	20	Thing: observational
osmosis	20	Activity: observational
muscle fiber	20	Thing: observational
apical meristem	20	Thing: observational
steroid hormone	20	Thing: observational
muscle tissue	20	Thing: observational
inner ear	20	Thing: observational
sexual reproduction	20	Activity: observational
endometrium	19	Thing: observational
progesterone	19	Thing: observational
dispersal	19	Activity: observational
cortex	19	Thing: observational
sporophyte generation	19	Activity: observational
root system	19	Thing: observational
bone marrow	19	Thing: observational
nerve impulse	19	Thing: observational
nerve cell	19	Thing: observational
capsule	18	Thing: observational
dicots	18	Thing: observational
cuticle	18	Thing: observational
cerebrum	18	Thing: observational
ectoderm	18	Thing: observational
vessel element	18	Thing: observational
muscle contraction	18	Activity: observational
negative feedback	18	Activity: observational
chest cavity	18	Thing: observational
pigment	17	Thing: observational
thorax	17	Thing: observational
lycophytes	17	Thing: observational
fertilisation	17	Activity: observational
testosterone	17	Thing: observational
mantle	17	Thing: observational
potassium	17	Thing: observational
penis	17	Thing: observational
urethra	17	Thing: observational
right ventricle	17	Thing: observational
plant tissue	17	Thing: observational
cardiovascular system	17	Thing: observational

endoskeleton	16	Thing: observational
exhalation	16	Activity: observational
esophagus	16	Thing: observational
aorta	16	Thing: observational
microscope	16	Thing: instrumental
peripheral nervous system	16	Thing: observational
seed coat	16	Thing: observational
pharyngeal pouch	16	Thing: observational
female cone	16	Thing: observational
autonomic nervous system	16	Thing: observational
postanal tail	16	Thing: observational
spore	15	Thing: observational
medusa	15	Thing: observational
oviduct	15	Thing: observational
blastula	15	Thing: observational
cardiac muscle	15	Thing: observational
right atrium	15	Thing: observational
ground tissue	15	Thing: observational
corpus luteum	15	Thing: observational
helper t	15	Thing: observational
larval stage	15	Activity: observational
blood glucose	15	Thing: observational
fallopian tube	15	Thing: observational
sense organ	15	Thing: observational
myelin	14	Thing: observational
megaspore	14	Thing: observational
cerebellum	14	Thing: observational
subphylum	14	Thing: observational
centipede	14	Thing: observational
scrotum	14	Thing: observational
tropism	14	Activity: observational
glycogen	14	Thing: observational
mating	14	Activity: observational
antenna	14	Thing: observational
cervix	14	Thing: observational
synthesis	14	Activity: observational
sclerenchyma cell	14	Thing: observational
target cell	14	Thing: observational
helper t cell	14	Thing: observational
guard cell	14	Thing: observational
nerve tissue	14	Thing: observational
asexual reproduction	14	Activity: observational
hair cell	14	Thing: observational
limb	13	Thing: observational
neurotransmitter	13	Thing: observational
eudicots	13	Thing: observational
blastocyst	13	Thing: observational
tetrapod	13	Thing: observational
nephron	13	Thing: observational
oocyte	13	Thing: observational
rhizome	13	Thing: observational
inhalation	13	Activity: observational
germination	13	Activity: observational
ethylene	13	Thing: observational
lipid	13	Thing: observational
evaporation	13	Activity: observational
crest	13	Thing: observational
cholesterol	13	Thing: observational

endocrine gland	13	Thing: observational
capillary wall	13	Thing: observational
male gametophyte	13	Thing: observational
epidermal cell	13	Thing: observational
epithelial cell	13	Thing: observational
chordate	12	Thing: observational
antibody	12	Thing: observational
ovum	12	Thing: observational
caecilian	12	Thing: observational
interneuron	12	Thing: observational
cladogram	12	Semiotic: model
ginkgo	12	Thing: observational
nephridia	12	Thing: observational
pollinator	12	Thing: observational
omnivore	12	Thing: observational
medulla	12	Thing: observational
mitochondrion	12	Thing: observational
nectar	12	Thing: observational
saliva	12	Thing: observational
courtship	12	Activity: observational
nicotine	12	Thing: observational
mite	12	Thing: observational
bulb	12	Thing: observational
tendon	12	Thing: observational
convex lens	12	Thing: observational
skeletal system	12	Thing: observational
urinary bladder	12	Thing: observational
concentration gradient	12	Thing: observational
vertebral column	12	Thing: observational
sieve tube cell	12	Thing: observational
conifer	11	Thing: observational
pistil	11	Thing: observational
telescope	11	Thing: instrumental
therapsid	11	Thing: observational
protostome	11	Thing: observational
palisade	11	Thing: observational
microspore	11	Thing: observational
oblongata	11	Thing: observational
deuterostome	11	Thing: observational
endotherms	11	Thing: observational
phagocytosis	11	Thing: observational
peristalsis	11	Activity: observational
micropyle	11	Thing: observational
pseudocoelom	11	Thing: observational
tuatara	11	Thing: observational
deferens	11	Thing: observational
squirt	11	Thing: observational
fermentation	11	Activity: observational
semen	11	Thing: observational
moth	11	Thing: observational
faeces	11	Thing: observational
adolescence	11	Activity: observational
sieve tube	11	Thing: observational
medulla oblongata	11	Thing: observational
hair follicle	11	Thing: observational
internal fertilization	11	Activity: observational
muscular system	11	Thing: observational
excretory system	11	Thing: observational

four-chambered heart	11	Thing: observational
closed circulatory system	11	Thing: observational
reproductive tract	11	Thing: observational
vas deferens	11	Thing: observational
sperm cell	11	Thing: observational
adrenal gland	11	Thing: observational
sensory cell	11	Thing: observational
cnidarian	10	Thing: observational
archegonium	10	Thing: observational
eardrum	10	Thing: observational
thymus	10	Thing: observational
prothallus	10	Thing: observational
sepal	10	Thing: observational
radula	10	Thing: observational
myofibrils	10	Thing: observational
deuterostomes	10	Thing: observational
gymnosperm	10	Thing: observational
polychaete	10	Thing: observational
mandible	10	Thing: observational
urea	10	Thing: observational
atherosclerosis	10	Activity: observational
sternum	10	Thing: observational
dialysis	10	Activity: enacted
ejaculation	10	Activity: observational
spleen	10	Thing: observational
rectum	10	Thing: observational
fluke	10	Thing: observational
vegetation	10	Thing: observational
xylem vessel	10	Thing: observational
ympathetic nervous syster	10	Thing: observational
open circulatory system	10	Thing: observational
swim bladder	10	Thing: observational
spongy bone	10	Thing: observational
emale reproductive syster	10	Thing: observational
mammary gland	10	Thing: observational
inflammatory response	10	Activity: observational
active immunity	10	Thing: observational
menstrual flow	10	Thing: observational
protein synthesis	10	Activity: observational
skin cell	10	Thing: observational
shoot system	10	Thing: observational
jaw	9	Thing: observational
elongation	9	Activity: observational
monotreme	9	Thing: observational
photoperiodism	9	Activity: observational
chorion	9	Thing: observational
incisor	9	Thing: observational
endosperm	9	Thing: observational
cytokinins	9	Thing: observational
antheridia	9	Thing: observational
trichome	9	Thing: observational
anthophyte	9	Thing: observational
myoglobin	9	Thing: observational
keratin	9	Thing: observational
cornea	9	Thing: observational
gallbladder	9	Thing: observational
hydra	9	Thing: observational
ammonia	9	Thing: observational

burrow	9	Thing: observational
mole	9	Thing: observational
vaccination	9	Activity: enacted
green algae	9	Thing: observational
companion cell	9	Thing: observational
mitotic cell	9	Thing: observational
tympanic membrane	9	Thing: observational
filter feeder	9	Thing: observational
vascular cambium	9	Thing: observational
palisade mesophyll	9	Thing: observational
chorionic villus	9	Thing: observational
meristematic tissue	9	Thing: observational
collenchyma cell	9	Thing: observational
compound eye	9	Thing: observational
vocal cord	9	Thing: observational
sweat gland	9	Thing: observational
tissue fluid	9	Thing: observational
feedback mechanism	9	Activity: observational
reproductive organ	9	Thing: observational
cell differentiation	9	Activity: observational
human eye	9	Thing: observational
heart muscle	9	Thing: observational
natural selection	9	Activity: observational
stem cell	9	Thing: observational
stem cells	9	Thing: observational
endodermis	8	Thing: observational
lamella	8	Thing: observational
epiglottis	8	Thing: observational
feces	8	Thing: observational
anthozoan	8	Thing: observational
bronchiole	8	Thing: observational
pseudocoelomates	8	Thing: observational
millipedes	8	Thing: observational
ectotherms	8	Thing: observational
translocation	8	Activity: observational
immunization	8	Activity: observational
hinge	8	Thing: observational
phosphate	8	Activity: observational
hemisphere	8	Thing: observational
yolk	8	Thing: observational
larynx	8	Thing: observational
non-seed plant	8	Thing: observational
plant hormone	8	Thing: observational
sperm nucleus	8	Thing: observational
left ventricle	8	Thing: observational
bone tissue	8	Thing: observational
negative feedback system	8	Thing: observational
sporophyte stage	8	Activity: observational
gastrovascular cavity	8	Thing: observational
lateral line system	8	Thing: observational
parasitic flatworm	8	Thing: observational
third-class lever	8	Thing: observational
pulmonary system	8	Thing: observational
complete metamorphosis	8	Activity: observational
thymus gland	8	Thing: observational
tracheal tube	8	Thing: observational
lateral meristem	8	Thing: observational
flower organ	8	Thing: observational

male reproductive structu	8	Thing: observational
bone cell	8	Thing: observational
involuntary muscle	8	Thing: observational
muscular organ	8	Thing: observational
specialized cell	8	Thing: observational
blood plasma	8	Thing: observational
sensory organ	8	Thing: observational
umbilical cord	8	Thing: observational
fatty acid	8	Thing: observational
testis	7	Thing: observational
liverwort	7	Thing: observational
bud	7	Thing: observational
planarian	7	Thing: observational
squid	7	Thing: observational
nematocyst	7	Thing: observational
lymphocyte	7	Thing: observational
vacuole	7	Thing: observational
pedipalp	7	Thing: observational
seta	7	Thing: observational
melanin	7	Thing: observational
sorus	7	Thing: observational
sclereids	7	Thing: observational
proglottids	7	Thing: observational
platypus	7	Thing: observational
amnion	7	Thing: observational
neutrophil	7	Thing: observational
taproot	7	Thing: observational
stapes	7	Thing: observational
osteoblast	7	Thing: observational
medusae	7	Thing: observational
chelicera	7	Thing: observational
cephalothorax	7	Thing: observational
calcitonin	7	Thing: observational
myosin	7	Thing: observational
pincers	7	Thing: observational
hydroid	7	Thing: observational
chlorophyll	7	Thing: observational
allergen	7	Thing: observational
flagellum	7	Thing: observational
seesaw	7	Thing: observational
prism	7	Thing: observational
collagen	7	Thing: observational
snout	7	Thing: observational
siphon	7	Thing: observational
hibernation	7	Activity: observational
gestation	7	Activity: observational
rodent	7	Thing: observational
vesicle	7	Thing: observational
silica	7	Thing: observational
menopause	7	Activity: observational
pelvis	7	Thing: observational
foetus	7	Thing: observational
ventilation	7	Activity: observational
external fertilization	7	Activity: observational
mitotic cell division	7	Activity: observational
unicellular organism	7	Thing: observational
male reproductive system	7	Thing: observational
cartilaginous fish	7	Thing: observational

saturated fat	7	Thing: observational
leaflike structure	7	Thing: observational
outer ear	7	Thing: observational
cell elongation	7	Activity: observational
dorsal hollow nerve cord	7	Thing: observational
cone cell	7	Thing: observational
symmetrical animal	7	Thing: observational
posterior air sac	7	Thing: observational
oxygen-poor blood	7	Thing: observational
dorsal tubular nerve cord	7	Thing: observational
dermal tissue	7	Thing: observational
gill slit	7	Thing: observational
vertebrate chordate	7	Thing: observational
red bone marrow	7	Thing: observational
cognitive behavior	7	Thing: observational
cytotoxic t	7	Thing: observational
taste bud	7	Thing: observational
passive immunity	7	Thing: observational
sex cell	7	Thing: observational
marine animal	7	Thing: observational
nerve ending	7	Thing: observational
growth hormone	7	Thing: observational
salamander	6	Thing: observational
leech	6	Thing: observational
rhizoid	6	Thing: observational
inhalant	6	Thing: observational
radulas	6	Thing: observational
thallose	6	Thing: observational
amphioxus	6	Thing: observational
angiosperm	6	Thing: observational
glomerulus	6	Thing: observational
gonorrhea	6	Thing: observational
molt	6	Thing: observational
keratinocytes	6	Thing: observational
glucagon	6	Thing: observational
scyphozoan	6	Thing: observational
gemmule	6	Thing: observational
epinephrine	6	Thing: observational
anteater	6	Thing: observational
melanoma	6	Thing: observational
barnacle	6	Thing: observational
dormancy	6	Activity: observational
pepsin	6	Thing: observational
sphincter	6	Thing: observational
shrew	6	Thing: observational
nautilus	6	Thing: observational
epididymis	6	Thing: observational
tonsil	6	Thing: observational
interferon	6	Thing: observational
barb	6	Thing: observational
iodine	6	Thing: observational
primary oocyte	6	Thing: observational
photosynthetic tissue	6	Thing: observational
epidermal layer	6	Thing: observational
vascular system	6	Thing: observational
tiny bone	6	Thing: observational
plant organ	6	Thing: observational
fibrous root	6	Thing: observational

non-seed vascular plant	6	Thing: observational
pivot joint	6	Thing: observational
flame cell	6	Thing: observational
cell specialisation	6	Activity: observational
mantle cavity	6	Thing: observational
freshwater habitat	6	Place
collar cell	6	Thing: observational
somatic nervous system	6	Thing: observational
anterior air sac	6	Thing: observational
parathyroid gland	6	Thing: observational
first-class lever	6	Thing: observational
second-class lever	6	Thing: observational
posterior end	6	Thing: observational
jointed appendage	6	Thing: observational
free-swimming larva	6	Thing: observational
oxygen-rich blood	6	Thing: observational
ball-and-socket joint	6	Thing: observational
myelin sheath	6	Thing: observational
spongy mesophyll	6	Thing: observational
simple eye	6	Thing: observational
malpighian tubule	6	Thing: observational
pulmonary artery	6	Thing: observational
female gamete	6	Thing: observational
sieve cell	6	Thing: observational
leaf blade	6	Thing: observational
integumentary system	6	Thing: observational
oxygenate blood	6	Thing: observational
meiotic division	6	Activity: observational
muscular contraction	6	Activity: observational
lymph vessel	6	Thing: observational
altruistic behavior	6	Thing: observational
fight-or-flight response	6	Activity: observational
amino acid hormone	6	Thing: observational
sperm production	6	Activity: observational
luteal phase	6	Activity: observational
lymphatic tissue	6	Thing: observational
voluntary muscle	6	Thing: observational
nasal passage	6	Thing: observational
alveolus	5	Thing: observational
appendage	5	Thing: observational
urchin	5	Thing: observational
parasite	5	Thing: observational
wing	5	Thing: observational
villus	5	Thing: observational
spine	5	Thing: observational
tunicate	5	Thing: observational
fungus	5	Thing: observational
pore	5	Thing: observational
stamen	5	Thing: observational
macrophage	5	Thing: observational
phagocyte	5	Thing: observational
collenchyma	5	Thing: observational
petiole	5	Thing: observational
coelomates	5	Thing: observational
prophase	5	Activity: observational
trilobite	5	Thing: observational
ureter	5	Thing: observational
dicot	5	Thing: observational

chitin	5	Thing: observational
monocot	5	Thing: observational
amylase	5	Thing: observational
acoelomates	5	Thing: observational
zooxanthellae	5	Thing: observational
nudibranchs	5	Thing: observational
strobilus	5	Thing: observational
protostomes	5	Thing: observational
hypocotyl	5	Thing: observational
hydrozoan	5	Thing: observational
pons	5	Thing: observational
foregut	5	Thing: observational
allantois	5	Thing: observational
spongin	5	Thing: observational
coelacanth	5	Thing: observational
pedicellariae	5	Thing: observational
echidna	5	Thing: observational
iguana	5	Thing: observational
protozoans	5	Thing: observational
cloaca	5	Thing: observational
thyroxine	5	Thing: observational
arteriole	5	Thing: observational
polysaccharide	5	Thing: observational
scab	5	Thing: observational
cypress	5	Thing: observational
slime	5	Thing: observational
prokaryotes	5	Thing: observational
echolocation	5	Activity: observational
camouflage	5	Thing: observational
suffocation	5	Activity: observational
flow phase	5	Activity: observational
root cell	5	Thing: observational
two-chambered heart	5	Thing: observational
auditory canal	5	Thing: observational
optical instrument	5	Thing: instrumental
xylem tissue	5	Thing: observational
synaptic gap	5	Thing: observational
root cap	5	Thing: observational
brain stem	5	Thing: observational
water vascular system	5	Thing: observational
diploid zygote	5	Thing: observational
day-neutral plant	5	Thing: observational
immovable joint	5	Thing: observational
muscular tube	5	Thing: observational
scalelike leave	5	Thing: observational
cytoplasmic pathway	5	Thing: observational
oil gland	5	Thing: observational
fast-twitch muscle	5	Thing: observational
gametophyte stage	5	Activity: observational
haploid spore	5	Thing: observational
tetanus bacterium	5	Thing: observational
movable joint	5	Thing: observational
tubular cell	5	Thing: observational
light sensor	5	Thing: observational
biceps muscle	5	Thing: observational
plant response	5	Thing: observational
parasympathetic system	5	Thing: observational
stem reptile	5	Thing: observational

generative nucleus	5	Thing: observational
asympathetic nervous syst	5	Thing: observational
musclelike cell	5	Thing: observational
pain receptor	5	Thing: observational
three-chambered heart	5	Thing: observational
terrestrial arthropod	5	Thing: observational
tube nucleus	5	Thing: observational
parathyroid hormone	5	Thing: observational
sliding filament theory	5	Semiotic: theory
degenerative disease	5	Thing: observational
water-vascular system	5	Thing: observational
sensory receptor	5	Thing: observational
stinging cell	5	Thing: observational
aquatic plant	5	Thing: observational
reproductive cell	5	Thing: observational
embryonic cell	5	Thing: observational
upper arm	5	Thing: observational
cerebral cortex	5	Thing: observational
liver cell	5	Thing: observational
optic nerve	5	Thing: observational
coronary artery	5	Thing: observational
food chain	5	Thing: observational
carbon fixation	5	Activity: observational
gamete	4	Thing: observational
cotyledon	4	Thing: observational
chromosome	4	Thing: observational
arthrophytes	4	Thing: observational
amniotes	4	Thing: observational
peduncle	4	Thing: observational
incus	4	Thing: observational
gnetophyta	4	Thing: observational
amoebocyte	4	Thing: observational
gnetophytes	4	Thing: observational
malleus	4	Thing: observational
thigmotropism	4	Activity: observational
corm	4	Thing: observational
turbellarian	4	Thing: observational
tardigrade	4	Thing: observational
theropod	4	Thing: observational
vena	4	Thing: observational
cnidocytes	4	Thing: observational
duodenum	4	Thing: observational
gravitropism	4	Activity: observational
sarcomere	4	Thing: observational
bryophyte	4	Thing: observational
agnathans	4	Thing: observational
flytrap	4	Thing: observational
sarcomeres	4	Thing: observational
caecum	4	Thing: observational
plasmodesmata	4	Thing: observational
chemoautotrophs	4	Thing: observational
dilation	4	Activity: observational
cephalization	4	Activity: observational
autotroph	4	Thing: observational
chemoreceptor	4	Thing: observational
glycerol	4	Thing: observational
chipmunk	4	Thing: observational
grazer	4	Thing: observational

ruminant	4	Thing: observational
trichinosis	4	Thing: observational
rumen	4	Thing: observational
termite	4	Thing: observational
gizzard	4	Thing: observational
radicle	4	Thing: observational
lupus	4	Thing: observational
oxytocin	4	Thing: observational
carapace	4	Thing: observational
oesophagus	4	Thing: observational
lignin	4	Thing: observational
elegans	4	Thing: observational
tympanum	4	Thing: observational
norepinephrine	4	Thing: observational
parthenogenesis	4	Activity: observational
lumen	4	Thing: observational
lysine	4	Thing: observational
plankton	4	Thing: observational
scavenger	4	Thing: observational
ingestion	4	Activity: observational
menstruation	4	Activity: observational
receptor cell	4	Thing: observational
third trimester	4	Thing: observational
temperate region	4	Place
respiratory organ	4	Thing: observational
non-infectious disease	4	Thing: observational
hindgut fermenter	4	Thing: observational
somatic system	4	Thing: observational
waxy cuticle	4	Thing: observational
ovary wall	4	Thing: observational
fluid-filled body cavity	4	Thing: observational
follicular phase	4	Activity: observational
upper epidermis	4	Thing: observational
motile sperm	4	Thing: observational
moss gametophyte	4	Thing: observational
foregut fermenter	4	Thing: observational
human growth hormone	4	Thing: observational
cycloid scale	4	Thing: observational
leafy liverwort	4	Thing: observational
diploid sporophyte	4	Thing: observational
gametophyte plant	4	Thing: observational
cytotoxic t cell	4	Thing: observational
haploid gametophyte	4	Thing: observational
respiratory structure	4	Thing: observational
double fertilization	4	Activity: observational
circular muscle	4	Thing: observational
roundworm parasite	4	Thing: observational
duck-billed platypus	4	Thing: observational
mouth cavity	4	Thing: observational
sporophyte plant	4	Thing: observational
female reproductive organ	4	Thing: observational
fast-twitch muscle fiber	4	Thing: observational
piration-cohesion-tension t	4	Semiotic: theory
leaf litter	4	Thing: observational
deoxygenate blood	4	Thing: observational
terrestrial vertebrate	4	Thing: observational
anterior pituitary gland	4	Thing: observational
defense system	4	Thing: observational

root hair cell	4	Thing: observational
lymph capillary	4	Thing: observational
arthropod group	4	Thing: observational
left atrium	4	Thing: observational
female reproductive tract	4	Thing: observational
nonspecific immunity	4	Thing: observational
man immunodeficiency vi	4	Thing: observational
nervous tissue	4	Thing: observational
extraembryonic membrane	4	Thing: observational
tissue of root	4	Thing: observational
adult tunicate	4	Thing: observational
phloem cell	4	Thing: observational
specific immune response	4	Activity: observational
photosynthetic autotrophs	4	Thing: observational
chemosynthetic autotrophs	4	Thing: observational
peptide hormone	4	Thing: observational
tip of root	4	Thing: observational
phloem sap	4	Thing: observational
plant cell wall	4	Thing: observational
mple multicellular organis	4	Thing: observational
free-living flatworm	4	Thing: observational
companion cell	4	Thing: observational
root apical meristem	4	Thing: observational
acquired immunity	4	Thing: observational
seminiferous tubule	4	Thing: observational
horseshoe crab	4	Thing: observational
woody stem	4	Thing: observational
vein thrombosis	4	Activity: observational
haploid cell	4	Thing: observational
epithelial tissue	4	Thing: observational
spinal nerve	4	Thing: observational
memory cell	4	Thing: observational
nasal cavity	4	Thing: observational
animal cell	4	Thing: observational
fat tissue	4	Thing: observational
lactic acid	4	Thing: observational
host cell	4	Thing: observational
brain tissue	4	Thing: observational
hydrochloric acid	4	Thing: observational
herbivore	3	Thing: observational
chloroplast	3	Thing: observational
dendrite	3	Thing: observational
rotifer	3	Thing: observational
sporangium	3	Thing: observational
ganglion	3	Thing: observational
opossum	3	Thing: observational
photoheterotrophs	3	Thing: observational
antheridium	3	Thing: observational
ampulla	3	Thing: observational
depolarization	3	Activity: observational
chemoheterotrophs	3	Activity: observational
wishbone	3	Activity: observational
cubozoans	3	Activity: observational
hydrozoa	3	Activity: observational
archaeopteryx	3	Activity: observational
fibrin	3	Activity: observational
monocotyledon	3	Activity: observational
clitellum	3	Activity: observational

exocytosis	3	Activity: observational
melanocyte	3	Activity: observational
copulation	3	Activity: observational
metaphase	3	Activity: observational
regrow	3	Thing: observational
psilophytes	3	Thing: observational
strobili	3	Thing: observational
venule	3	Thing: observational
oligochaete	3	Thing: observational
insectivore	3	Thing: observational
endotherm	3	Thing: observational
cecum	3	Thing: observational
madreporite	3	Thing: observational
chyme	3	Thing: observational
sponges	3	Thing: observational
ommatidium	3	Thing: observational
protonema	3	Thing: observational
placoderm	3	Thing: observational
venation	3	Activity: observational
dicotyledon	3	Thing: observational
bighorn	3	Thing: observational
duce	3	Thing: observational
pterophytes	3	Thing: observational
operculum	3	Thing: observational
gnetum	3	Thing: observational
thallus	3	Thing: observational
eudicot	3	Thing: observational
photoautotrophs	3	Thing: observational
heterotroph	3	Thing: observational
cyanobacteria	3	Thing: observational
mechanoreceptors	3	Thing: observational
karyotype	3	Thing: instrumental
siphonophore	3	Thing: observational
embolism	3	Thing: observational
spiracle	3	Thing: observational
pika	3	Thing: observational
osteocytes	3	Thing: observational
prions	3	Thing: observational
endothermy	3	Thing: observational
guttation	3	Activity: observational
poinsettia	3	Thing: observational
sclera	3	Thing: observational
osteon	3	Thing: observational
egestion	3	Activity: observational
monocyte	3	Thing: observational
armadillo	3	Thing: observational
septum	3	Thing: observational
aldosterone	3	Thing: observational
fructose	3	Thing: observational
larch	3	Thing: observational
radish	3	Thing: observational
redwood	3	Thing: observational
bursitis	3	Thing: observational
adenosine	3	Thing: observational
sprain	3	Thing: observational
analgesic	3	Thing: observational
femur	3	Thing: observational
goitre	3	Thing: observational

garter	3	Thing: observational
aphid	3	Thing: observational
embryonic membrane	3	Thing: observational
intercalary meristem	3	Thing: observational
arthropod limb	3	Thing: observational
bony plate	3	Thing: observational
cell plate	3	Thing: observational
hormone insulin	3	Thing: observational
thallose liverwort	3	Thing: observational
double circulatory system	3	Thing: observational
liverwort gametophyte	3	Thing: observational
single circulatory system	3	Thing: observational
triceps muscle	3	Thing: observational
evolutionary adaptation	3	Activity: observational
aerobic respiration	3	Activity: observational
entire menstrual cycle	3	Activity: observational
thallose liverwort	3	Thing: observational
protective tissue	3	Thing: observational
spinal column	3	Thing: observational
aquatic larva	3	Thing: observational
blood surface antigen	3	Thing: observational
appendicular skeleton	3	Thing: observational
leaf tissue	3	Thing: observational
adaptive radiation	3	Activity: observational
digestive organ	3	Thing: observational
uterine muscle	3	Thing: observational
ancestral thyroid gland	3	Thing: observational
sensory perception	3	Activity: observational
eustachian tube	3	Thing: observational
endoderm cell	3	Thing: observational
simple nervous system	3	Thing: observational
physical digestion	3	Activity: observational
sessile animal	3	Thing: observational
vegetative propagation	3	Activity: observational
subcutaneous layer	3	Thing: observational
axial skeleton	3	Thing: observational
ellipsoid joint	3	Thing: observational
tropic response	3	Activity: observational
free nerve ending	3	Thing: observational
sensory structure	3	Thing: observational
root tissue	3	Thing: observational
plasma cell	3	Thing: observational
homeotic gene	3	Thing: observational
phloem tissue	3	Thing: observational
herbaceous stem	3	Thing: observational
sea slug	3	Thing: observational
tropical area	3	Place
second meiotic division	3	Activity: observational
cylindrical body	3	Thing: observational
visual cell	3	Thing: observational
uterine contraction	3	Activity: observational
artery branch	3	Thing: observational
hormone estrogen	3	Thing: observational
host organism	3	Thing: observational
head-footed mollusk	3	Thing: observational
innate response	3	Activity: observational
heart tissue	3	Thing: observational
nale reproductive structur	3	Thing: observational

tropical rain forest	3	Place
acoelomate animal	3	Thing: observational
renal tubule	3	Thing: observational
endothermic metabolism	3	Activity: observational
primary response	3	Activity: observational
gland cell	3	Thing: observational
sebaceous gland	3	Thing: observational
spiny anteater	3	Thing: observational
eyepiece lens	3	Thing: observational
fibrous root system	3	Thing: observational
non-vascular plant	3	Thing: observational
neck muscle	3	Thing: observational
mass extinction	3	Activity: observational
cranial nerve	3	Thing: observational
aortic valve	3	Thing: observational
gene expression	3	Activity: observational
hoofed mammal	3	Thing: observational
menstrual bleeding	3	Activity: observational
salivary gland	3	Thing: observational
sex organ	3	Thing: observational
auditory nerve	3	Thing: observational
chemical messenger	3	Thing: observational
genital herpes	3	Thing: observational
heart valve	3	Thing: observational
biological clock	3	Activity: observational
birth canal	3	Thing: observational
light-sensitive cell	3	Thing: observational
human skeleton	3	Thing: observational
genetic diversity	3	Thing: observational
upper jaw	3	Thing: observational
eye socket	3	Thing: observational
kin selection	3	Activity: observational
tooth	2	Thing: observational
microorganism	2	Thing: observational
pheromone	2	Thing: observational
hermaphrodite	2	Thing: observational
ligament	2	Thing: observational
sheath	2	Thing: observational
spinneret	2	Thing: observational
lacteal	2	Thing: observational
welwitschia	2	Thing: observational
rhipidistians	2	Thing: observational
hepaticophytes	2	Thing: observational
gecko	2	Thing: observational
osteoclast	2	Thing: observational
chlorenchyma	2	Thing: observational
mantid	2	Thing: observational
housefly	2	Thing: observational
hirudinea	2	Thing: observational
wallaby	2	Thing: observational
copepod	2	Thing: observational
pneumatophore	2	Thing: observational
chordata	2	Thing: observational
blimp	2	Thing: observational
acrosome	2	Thing: observational
preen	2	Thing: observational
midbrain	2	Thing: observational
cellulase	2	Thing: observational

flyswatter	2	Thing: observational
mutualism	2	Activity: observational
cichlid	2	Thing: observational
cytosol	2	Thing: observational
morula	2	Thing: observational
cytokines	2	Thing: observational
isoleucine	2	Thing: observational
landmass	2	Thing: observational
cestode	2	Thing: observational
lemur	2	Thing: observational
millipede	2	Thing: observational
sebum	2	Thing: observational
elastin	2	Thing: observational
plastron	2	Thing: observational
erythrocyte	2	Thing: observational
cladograms	2	Thing: observational
epiphyte	2	Thing: observational
lycophyte	2	Thing: observational
cava	2	Thing: observational
nephritis	2	Thing: observational
sclerids	2	Thing: observational
trematode	2	Thing: observational
subcutis	2	Thing: observational
luid	2	Thing: observational
swamphen	2	Thing: observational
endocytosis	2	Activity: observational
nonsteroid	2	Thing: observational
grana	2	Thing: observational
tubifex	2	Thing: observational
myofibril	2	Thing: observational
umbilicus	2	Thing: observational
placentals	2	Thing: observational
saprotrophs	2	Thing: observational
methanogens	2	Thing: observational
glucocorticoids	2	Thing: observational
gnetophyte	2	Thing: observational
perissodactyl	2	Thing: observational
cavae	2	Thing: observational
auricle	2	Thing: observational
tracheoles	2	Thing: observational
aster	2	Thing: observational
pneumoniae	2	Thing: observational
cutin	2	Thing: observational
pansy	2	Thing: observational
sugarcane	2	Thing: observational
nonchordates	2	Thing: observational
endostyle	2	Thing: observational
peony	2	Thing: observational
hemophilia	2	Thing: observational
stents	2	Thing: observational
phototropism	2	Activity: observational
exine	2	Thing: observational
cubozoa	2	Thing: observational
abalone	2	Thing: observational
scyphozoa	2	Thing: observational
whelk	2	Thing: observational
photoreceptor	2	Thing: observational
oxyhaemoglobin	2	Thing: observational

proglottid	2	Thing: observational
haemolymph	2	Thing: observational
chorionic gonadotropin	2	Thing: observational
aquatic mammal	2	Thing: observational
preen gland	2	Thing: observational
hind limb	2	Thing: observational
vessel wall	2	Thing: observational
elongate cell	2	Thing: observational
diploid cell	2	Thing: observational
uman reproductive syster	2	Thing: observational
nembrane-bound organell	2	Thing: observational
ruptured blood vessel	2	Thing: observational
iron-containing protein	2	Thing: observational
tricuspid valve	2	Thing: observational
ear canal	2	Thing: observational
male reproductive organ	2	Thing: observational
bulbourethral gland	2	Thing: observational
mitotic division (mitosis)	2	Activity: observational
poisonous nematocyst	2	Thing: observational
thorax region	2	Thing: observational
hookworm infection	2	Activity: observational
environmental antigen	2	Thing: observational
tonguelike organ	2	Thing: observational
lymphatic organ	2	Thing: observational
diploid animal	2	Thing: observational
aquatic arthropod	2	Thing: observational
overall immune response	2	Activity: observational
threadlike tube	2	Thing: observational
sponge embryo	2	Thing: observational
marine biome	2	Thing: observational
cnidarian embryo	2	Thing: observational
soft-bodied invertebrate	2	Thing: observational
photosynthetic protist	2	Thing: observational
phylum platyhelminthes	2	Thing: observational
phagocytic cell	2	Thing: observational
terrestrial chordate	2	Thing: observational
fluid-filled canal	2	Thing: observational
aquatic tetrapod	2	Thing: observational
sensory tentacle	2	Thing: observational
phylum chordata	2	Thing: observational
nerve ring	2	Thing: observational
tunicate larva	2	Thing: observational
symmetrical larva	2	Thing: observational
ndoskeleton of echinoder	2	Thing: observational
aquatic chordate	2	Thing: observational
major blood vessel	2	Thing: observational
fan-shaped membrane	2	Thing: observational
sensory system	2	Thing: observational
pair of chromosome	2	Thing: observational
brown recluse	2	Thing: observational
front limb	2	Thing: observational
arthropod species	2	Thing: observational
crustacean mandible	2	Thing: observational
arthropod blood	2	Thing: observational
germ theory	2	Semiotic: theory
male gamete	2	Thing: observational
respiratory tube	2	Thing: observational
internal respiration	2	Activity: observational

b marker	2	Thing: observational
disease-causing organism	2	Thing: observational
botulism bacterium	2	Thing: observational
scaly reptile	2	Thing: observational
biological rhythm	2	Thing: observational
acid fermentation	2	Activity: observational
slow-twitch muscle	2	Thing: observational
human digestive system	2	Thing: observational
cylindrical cell	2	Thing: observational
modified root	2	Thing: observational
nutrient agar	2	Thing: observational
wind-pollinated plant	2	Thing: observational
protein fiber	2	Thing: observational
male cricket	2	Thing: observational
n-specific immune respor	2	Thing: observational
food-storage organ	2	Thing: observational
branching root	2	Thing: observational
bone-forming cell	2	Thing: observational
cycad cone	2	Thing: observational
flower stem	2	Thing: observational
complex eye	2	Thing: observational
division gnetophyta	2	Thing: observational
genus gnetum	2	Thing: observational
monocot root	2	Thing: observational
lateral root	2	Thing: observational
stomach lining	2	Thing: observational
system of reptile	2	Thing: observational
microscopic tubule	2	Thing: observational
air capillary	2	Thing: observational
nonflagellated sperm	2	Thing: observational
corky tissue	2	Thing: observational
motic concentration gradi	2	Thing: observational
cular tissue of terrestrial p	2	Thing: observational
gymnosperm plant	2	Thing: observational
primary growth	2	Activity: observational
skull bone	2	Thing: observational
nplex multicellular organi	2	Thing: observational
natural passive immunity	2	Thing: observational
haploid nucleus	2	Thing: observational
glucagon bind	2	Thing: observational
chain of amino	2	Thing: observational
vascular tissue of root	2	Thing: observational
chrysanthemum plant	2	Thing: observational
spongin fiber	2	Thing: observational
metamorphosis insect	2	Thing: observational
human zygote	2	Thing: observational
lumbriculid worm	2	Thing: observational
tubifex worm	2	Thing: observational
organic debris	2	Thing: observational
herbivorous mammal	2	Thing: observational
chlorenchyma cell	2	Thing: observational
innate immunity	2	Thing: observational
tissue of leave	2	Thing: observational
endodermal cell	2	Thing: observational
hornwort sporophyte	2	Thing: observational
tree-sized lycophytes	2	Thing: observational
dominant sporophyte	2	Thing: observational
whooping crane	2	Thing: observational

fern gametophyte	2	Thing: observational
leafy stem	2	Thing: observational
meristematic cell	2	Thing: observational
memory b cell	2	Thing: observational
lymph vein	2	Thing: observational
epithelial-like cell	2	Thing: observational
specialized transport tissue	2	Thing: observational
heart-shaped gametophyte	2	Thing: observational
animal embryo	2	Thing: observational
ectoderm cell	2	Thing: observational
single neuron	2	Thing: observational
first meiotic division	2	Activity: observational
mesoderm cell	2	Thing: observational
fern sporophyte	2	Thing: observational
indoleacetic acid	2	Thing: observational
human red blood cell	2	Thing: observational
cellulose fiber	2	Thing: observational
male reproductive gland	2	Thing: observational
osteon system	2	Thing: observational
cardiac muscle tissue	2	Thing: observational
trachea branch	2	Thing: observational
protective ring	2	Thing: observational
meristem tissue	2	Thing: observational
negative gravitropism	2	Activity: observational
amoeba-like cell	2	Thing: observational
seed germination	2	Activity: observational
palisade mesophyll cell	2	Thing: observational
enzyme lysozyme	2	Thing: observational
fluorescence microscopy	2	Thing: instrumental
dorsal nerve cord	2	Thing: observational
coronary circulation	2	Activity: observational
aquatic ecosystem	2	Thing: observational
excretory organ	2	Thing: observational
keletal and muscular syste	2	Thing: observational
optic lobe	2	Thing: observational
microscopic blood vessel	2	Thing: observational
microscopic organism	2	Thing: observational
food vacuole	2	Thing: observational
olfactory bulb	2	Thing: observational
tubelike strand	2	Thing: observational
effector cell	2	Thing: observational
rgy-producing mitochondr	2	Thing: observational
cork tissue	2	Thing: observational
multicellular sporophyte	2	Thing: observational
jaw muscle	2	Thing: observational
cylindrical skeleton	2	Thing: observational
nspecific defense mechani	2	Activity: observational
stomach acid	2	Thing: observational
intestinal lining	2	Thing: observational
multicellular rhizoid	2	Thing: observational
nerve fiber	2	Thing: observational
leaf cell	2	Thing: observational
innate immune system	2	Thing: observational
aquatic annelid	2	Thing: observational
antiviral protein	2	Thing: observational
animal cell	2	Thing: observational
calcium carbonate plate	2	Thing: observational
aquatic organism	2	Thing: observational

cardiac muscle cell	2	Thing: observational
sympathetic system	2	Thing: observational
non-sulfur bacterium	2	Thing: observational
bicuspid valve	2	Thing: observational
xylem of vascular plant	2	Thing: observational
pulmonary valve	2	Thing: observational
flat membrane	2	Thing: observational
photosynthetic organism	2	Thing: observational
viral pathogen	2	Thing: observational
herbivorous heterotrophs	2	Thing: observational
hydrostatic skeleton	2	Thing: observational
unicellular microorganism	2	Thing: observational
stomatal pore	2	Thing: observational
carnivorous heterotrophs	2	Thing: observational
shoulder joint	2	Thing: observational
inner membrane	2	Thing: observational
flatworm cell	2	Thing: observational
circulatory organ	2	Thing: observational

GENETICS & EVOLUTION		
**NTTs**	**Frequencies**	**Categories**
gene	979	Thing: observational
chromosome	844	Thing: observational
plant	819	Thing: observational
DNA	737	Thing: observational
allele	582	Thing: observational
animal	473	Thing: observational
offspring	349	Thing: observational
evolution	280	Activity: observational
phenotype	273	Thing: observational
gamete	221	Thing: observational
meiosis	216	Activity: observational
amino acid	212	Thing: observational
nucleotide	189	Thing: observational
enzyme	186	Thing: observational
genotype	178	Thing: observational
bacterium	163	Thing: observational
natural selection	151	Activity: observational
fertilisation	139	Activity: observational
sperm	137	Thing: observational
embryo	129	Thing: observational
genome	126	Thing: observational
nucleus	113	Thing: observational
mitosis	104	Activity: observational
mRNA	100	Thing: observational
eye	94	Thing: observational
pedigree	87	Thing: observational
brain	85	Thing: observational
cloning	83	Activity: enacted
genetic variation	82	Activity: observational
polypeptide	76	Thing: observational
cell division	73	Activity: observational
zygote	72	Thing: observational
homologous chromosome	72	Thing: observational
insect	69	Thing: observational
pollen	64	Thing: observational
pea plant	63	Thing: observational
transcription	59	Activity: observational
habitat	59	Place
prokaryotes	57	Thing: observational
synthesis	57	Activity: observational
ecosystem	56	Thing: observational
isotope	56	Thing: observational
ribosome	53	Thing: observational
cytoplasm	53	Thing: observational
eukaryotic cell	53	Thing: observational
eukaryotes	52	Thing: observational
hormone	52	Thing: observational
chromatid	51	Thing: observational
reptile	51	Thing: observational
restriction enzyme	50	Thing: observational
genetics	49	Activity: enacted
predator	48	Thing: observational
DNA sequence	45	Thing: observational
sexual reproduction	45	Activity: observational
asexual reproduction	44	Activity: observational
recessive allele	43	Thing: observational

finch	42	Thing: observational
tail	42	Thing: observational
pollination	41	Activity: observational
fungus	41	Thing: observational
selective breeding	41	Activity: observational
daughter cell	41	Thing: observational
recombinant DNA	40	Thing: observational
polymerase	39	Thing: observational
mutagen	39	Thing: observational
carrier	39	Thing: observational
breeding	37	Activity: observational
sex chromosome	37	Thing: observational
gene therapy	37	Activity: enacted
fetus	36	Thing: observational
spore	35	Thing: observational
skull	35	Thing: observational
limb	35	Thing: observational
translation	35	Activity: observational
tRNA	33	Thing: observational
anthropoid	33	Thing: observational
mass extinction	33	Activity: observational
protist	32	Thing: observational
uterus	32	Thing: observational
thymine	31	Thing: observational
flowering plant	31	Thing: observational
ovary	30	Thing: observational
somatic cell	29	Thing: observational
genetic code	29	Thing: observational
taxon	28	Thing: observational
x chromosome	28	Thing: observational
bacterial cell	28	Thing: observational
hominid	27	Thing: observational
neanderthal	27	Thing: observational
ear	27	Thing: observational
cell cycle	27	Activity: observational
hominins	26	Thing: observational
polyploidy	26	Activity: observational
adenine	26	Thing: observational
nitrogenous base	26	Thing: observational
gene expression	26	Activity: observational
cytosine	25	Thing: observational
Precambrian	25	Time
prokaryotic cell	25	Thing: observational
reproductive organ	25	Thing: observational
nucleic acid	25	Thing: observational
mimicry	24	Activity: observational
heredity	24	Activity: observational
migration	24	Activity: observational
sister chromatid	24	Thing: observational
centromere	23	Thing: observational
crosse	23	Thing: observational
guanine	23	Thing: observational
metaphase	23	Activity: observational
polypeptide chain	23	Thing: observational
pollen grain	23	Thing: observational
egg cell	23	Thing: observational
gene sequence	23	Thing: observational
equilibrium	22	Activity: observational

pigment	22	Thing: observational
angiosperm	22	Thing: observational
mitochondrion	22	Thing: observational
root	22	Thing: observational
structural adaptation	22	Activity: observational
molecular clock	22	Thing: observational
interphase	21	Activity: observational
adaptive radiation	21	Activity: observational
ir of homologous chromoson	21	Thing: observational
sperm cell	21	Thing: observational
genetic mutation	21	Activity: observational
Mesozoic era	21	Time
fission	20	Activity: observational
cladogram	27	Semiotic: model
mating	20	Activity: observational
testis	20	Thing: observational
genetic marker	20	Thing: observational
parent cell	20	Thing: observational
australopithecine	19	Thing: observational
ovule	19	Thing: observational
neoplasm	19	Thing: observational
anaphase	19	Activity: observational
independent assortment	19	Activity: observational
DNA replication	19	Activity: observational
artificial selection	19	Activity: observational
introns	18	Thing: observational
cytokinesis	18	Activity: observational
ovum	18	Thing: observational
cone	18	Thing: observational
prey	18	Thing: observational
diploid cell	18	Thing: observational
amino acid sequence	18	Thing: observational
haploid cell	18	Thing: observational
cell membrane	18	Thing: observational
genetic engineering	18	Activity: enacted
exon	17	Thing: observational
pollinator	17	Thing: observational
electrophoresis	17	Activity: enacted
microscope	17	Thing: instrumental
Permian	17	Time
female gamete	17	Thing: observational
stop codon	17	Thing: observational
multicellular organism	17	Thing: observational
physiological adaptation	17	Activity: observational
protein synthesis	17	Activity: observational
organelle	16	Thing: observational
melanin	16	Thing: observational
polynucleotide	16	Thing: observational
vertebrate	16	Thing: observational
cell replication	16	Activity: observational
recessive phenotype	16	Thing: observational
point mutation	16	Activity: observational
DNA strand	16	Thing: observational
Cenozoic era	16	Time
stem	15	Thing: observational
spindle	15	Thing: observational
operon	15	Thing: observational
chloroplast	15	Thing: observational

placenta	15	Thing: observational
promoter	15	Thing: observational
multiple allele	15	Thing: observational
y chromosome	15	Thing: observational
recessive inheritance	15	Activity: observational
male gamete	15	Thing: observational
somatic mutation	15	Activity: observational
mitochondrial DNA	15	Thing: observational
nucleotide sequence	15	Thing: observational
radioactive isotope	15	Thing: observational
artificial insemination	15	Activity: enacted
stromatolite	14	Thing: observational
monotreme	14	Thing: observational
autosome	14	Thing: observational
phylogeny	14	Thing: observational
semen	14	Thing: observational
stigma	14	Thing: observational
microorganism	14	Thing: observational
proteome	14	Thing: observational
law of segregation	14	Semiotic: theory
gel electrophoresis	14	Activity: observational
plant cell	14	Thing: observational
histone	13	Thing: observational
oocyte	13	Thing: observational
gymnosperm	13	Thing: observational
phenylalanine	13	Thing: observational
hominin	13	Thing: observational
cyanobacteria	13	Thing: observational
bipedalism	13	Thing: observational
lactose	13	Thing: observational
gestation	13	Activity: observational
haemophilia	13	Thing: observational
embryology	13	Activity: enacted
rainforest	13	Place
dye	13	Thing: instrumental
polypeptide synthesis	13	Activity: observational
binary fission	13	Activity: observational
phylogenetic tree	13	Semiotic: model
mass extinction	13	Activity: observational
sequence of nucleotide	13	Thing: observational
gametophyte	12	Thing: observational
pistil	12	Thing: observational
uracil	12	Thing: observational
cytochrome	12	Thing: observational
phylum	12	Thing: observational
camouflage	12	Activity: observational
telophase	12	Activity: observational
artificial pollination	12	Activity: observational
phosphate group	12	Thing: observational
sexual selection	12	Activity: observational
reproductive tract	12	Thing: observational
Cretaceous period	12	Time
algae	11	Thing: observational
proteome	11	Thing: observational
anticodon	11	Thing: observational
diploid	11	Thing: observational
hibernation	11	Activity: observational
proton	11	Thing: observational

metabolism	11	Activity: observational
fertility	11	Thing: observational
erosion	11	Activity: observational
allopatric speciation	11	Activity: observational
meiosis i	11	Activity: observational
reproductive structure	11	Thing: observational
transgenic organism	11	Thing: observational
reproductive system	11	Thing: observational
glutamic acid	11	Thing: observational
blastocyst	10	Thing: observational
homozygote	10	Thing: observational
oviduct	10	Thing: observational
trisomy	10	Thing: observational
sporophyte	10	Thing: observational
deoxyribose	10	Thing: observational
photosynthesis	10	Activity: observational
lipid	10	Thing: observational
purine	10	Thing: observational
pyrimidine	10	Thing: observational
cervix	10	Thing: observational
female reproductive organ	10	Thing: observational
dihybrid cross	10	Thing: observational
pollen tube	10	Thing: observational
polygenic inheritance	10	Activity: observational
double-stranded DNA	10	Thing: observational
polymerase chain	10	Thing: observational
genetic screening	10	Activity: enacted
sex cell	10	Thing: observational
host cell	10	Thing: observational
sugar-phosphate backbone	10	Thing: observational
mtDNA	9	Thing: observational
tryptophan	9	Thing: observational
karyotype	9	Thing: observational
tetrapod	9	Thing: observational
stamen	9	Thing: observational
dormancy	9	Activity: observational
torpor	9	Activity: observational
arthropod	9	Thing: observational
digestion	9	Activity: observational
cleavage	9	Activity: observational
vagina	9	Thing: observational
artery	9	Thing: observational
Ordovician	9	Time
DNA polymerase	9	Thing: observational
recombinant plasmid	9	Thing: observational
endosymbiont theory	2	Semiotic: theory
nuclear membrane	9	Thing: observational
behavioural adaptation	9	Activity: observational
sex-linked inheritance	9	Activity: observational
mitotic division	9	Activity: observational
peptide bond	9	Thing: observational
theory of natural selection	9	Semiotic: theory
sister chromatid	9	Thing: observational
reproductive isolation	9	Activity: observational
chromosome pair	9	Thing: observational
vegetative reproduction	9	Activity: observational
mendelian inheritance	9	Activity: observational
frameshift mutation	9	Activity: observational

nucleotide polymorphism	9	Activity: enacted
DNA microarrays	9	Thing: instrumental
punctuated equilibrium	9	Semiotic: theory
Triassic period	9	Time
Proterozoic eon	9	Time
follicle	8	Thing: observational
ligases	8	Thing: observational
cross-pollination	8	Activity: observational
gonad	8	Thing: observational
ovulation	8	Activity: observational
invertebrate	8	Thing: observational
fauna	8	Thing: observational
larva	8	Thing: observational
mucus	8	Thing: observational
embryonic stem	8	Thing: observational
sympatric speciation	8	Activity: observational
spindle fiber	8	Thing: observational
chain of amino	8	Thing: observational
mitochondrial genome	8	Thing: observational
x-linked trait	8	Thing: observational
e allele	8	Thing: observational
oncogenic virus	8	Thing: observational
abiotic factor	8	Thing: observational
chromosomal mutation	8	Activity: observational
yellow-seed plant	8	Thing: observational
diploid zygote	8	Thing: observational
deoxyribonucleic acid	8	Thing: observational
blood cell	8	Thing: observational
archaea	7	Thing: observational
clasts	7	Thing: observational
lithosphere	7	Thing: observational
anther	7	Thing: observational
heterotrophs	7	Thing: observational
autotrophs	7	Thing: observational
archaebacteria	7	Thing: observational
systematics	7	Activity: enacted
phenylketonuria	7	Thing: observational
germination	7	Activity: observational
scavenger	7	Thing: observational
oestrogen	7	Thing: observational
puberty	7	Activity: observational
Silurian	7	Time
nuclear DNA	7	Thing: observational
chromosome map	7	Semiotic: model
cellular respiration	7	Activity: observational
x-linked recessive inheritance	7	Activity: observational
eukaryotic organism	7	Thing: observational
pyrimidine base	7	Thing: observational
chain of amino acid	7	Thing: observational
lithospheric plate	7	Thing: observational
chemical mutagen	7	Thing: observational
polynucleotide strand	7	Thing: observational
genetic factor	7	Thing: observational
food web	7	Thing: observational
haploid gamete	7	Thing: observational
green-seed plant	7	Thing: observational
t allele	7	Thing: observational
Jurassic Period	7	Time

germ	6	Thing: observational
polyploid	6	Thing: observational
xerophyte	6	Thing: observational
nucleosomes	6	Thing: observational
apoptosis	6	Activity: observational
tetrad	6	Thing: observational
ligase	6	Thing: observational
theropod	6	Thing: observational
hydrosphere	6	Thing: observational
oxytocin	6	Thing: observational
parthenogenesis	6	Activity: observational
predation	6	Activity: observational
haploid	6	Thing: observational
carboxyl	6	Thing: observational
progesterone	6	Thing: observational
savanna	6	Thing: observational
antigen	6	Thing: observational
implantation	6	Activity: observational
glucose	6	Thing: observational
amine	6	Thing: observational
synthesis of protein	6	Activity: observational
template strand	6	Thing: observational
diploid plant	6	Thing: observational
daughter isotope	6	Thing: observational
prehensile tail	6	Thing: observational
free nucleotide	6	Thing: observational
red blood cell	6	Thing: observational
female reproductive tract	6	Thing: observational
male reproductive organ	6	Thing: observational
missense mutation	6	Activity: observational
host organism	6	Thing: observational
parent isotope	6	Thing: observational
haploid sperm	6	Thing: observational
y-linked inheritance	6	Activity: observational
mRNA codon	6	Thing: observational
haploid egg	6	Thing: observational
vitamin d-resistant	6	Thing: observational
lacz gene	6	Thing: observational
recombinant plasmids	6	Thing: observational
polynucleotide chain	6	Thing: observational
biotic factor	6	Thing: observational
stem cell	6	Thing: observational
reproductive cell	6	Thing: observational
blood vessel	6	Thing: observational
fluorescent dye	6	Thing: instrumental
rezygotic isolating mechanisı	6	Activity: observational
transpiration	5	Activity: observational
monosomy	5	Activity: observational
alkaptonuria	5	Thing: observational
biogenesis	5	Semiotic: theory
valine	5	Thing: observational
ampicillin	5	Thing: observational
albinism	5	Thing: observational
trilobite	5	Thing: observational
decomposer	5	Thing: observational
auxin	5	Thing: observational
turgor	5	Thing: observational
lactation	5	Activity: observational

oncogene	5	Thing: observational
gastrula	5	Thing: observational
molar	5	Thing: observational
metamorphosis	5	Activity: observational
bacteriophage	5	Thing: instrumental
geology	5	Activity: enacted
menstruation	5	Activity: observational
fluorescence	5	Thing: observational
germ cell	5	Thing: observational
sequence of amino acid	5	Thing: observational
repair mechanism	5	Activity: observational
single strand	5	Thing: observational
DNA nucleotide	5	Thing: observational
enzyme gene	5	Thing: observational
vitamin d-resistant rickets	5	Thing: observational
ngle nucleotide polymorphisn	5	Activity: enacted
seedless plant	5	Thing: observational
germline mutation	5	Thing: observational
quaternary structure	5	Thing: observational
heterozygous offspring	5	Thing: observational
y-linked trait	5	Thing: observational
simple mendelian inheritance	5	Activity: observational
haploid spore	5	Thing: observational
tree frog	5	Thing: observational
mechanical pollination	5	Activity: enacted
complementary strand	5	Thing: observational
sequence of DNA	5	Thing: observational
artificial embryo	5	Thing: observational
intermediate phenotype	5	Thing: observational
genetic modification	5	Activity: enacted
red pigment	5	Thing: observational
cell wall	5	Thing: observational
repressor protein	5	Thing: observational
single nucleotide	5	Thing: observational
purine base	5	Thing: observational
circular DNA	5	Thing: observational
silent mutation	5	Activity: observational
animal cell	5	Thing: observational
regulatory gene	5	Thing: observational
carboxyl group	5	Thing: observational
skin cell	5	Thing: observational
Quaternary period	5	Time
Ediacaran period	5	Time
Devonian period	5	Time
hybridisation	4	Activity: observational
stratigraphy	4	Activity: enacted
brumation	4	Activity: observational
aestivation	4	Activity: observational
methionine	4	Thing: observational
primase	4	Thing: observational
cladistics	4	Activity: enacted
synapsis	4	Activity: observational
paramecium	4	Thing: observational
nucleoid	4	Thing: observational
bioluminescence	4	Activity: observational
phloem	4	Thing: observational
spliceosome	4	Thing: observational
thermoregulation	4	Activity: observational

stoma	4	Thing: observational
hypha	4	Thing: observational
australopithecus	4	Thing: observational
centriole	4	Thing: observational
endometrium	4	Thing: observational
sepal	4	Thing: observational
permineralization	4	Activity: observational
eukaryote	4	Thing: observational
galactose	4	Thing: observational
prokaryote	4	Thing: observational
inducer	4	Thing: observational
liverwort	4	Thing: observational
tubule	4	Thing: observational
herbivore	4	Thing: observational
filament	4	Thing: observational
carcinogen	4	Thing: observational
retardation	4	Activity: observational
protocell	4	Thing: observational
Hadean	4	Time
transcription factor	4	Thing: observational
dna region	4	Thing: observational
tertiary structure	4	Thing: observational
frontier toad	4	Thing: observational
embryonic stem cell	4	Thing: observational
membrane-bound nucleus	4	Thing: observational
theory of plate tectonics	4	Semiotic: theory
tumour-suppressor gene	4	Thing: observational
rock cycle	4	Activity: observational
x-linked dominant inheritance	4	Activity: observational
marine reptile	4	Thing: observational
genome sequence	4	Thing: observational
y allele	4	Thing: observational
law of heredity	4	Semiotic: theory
mutant allele	4	Thing: observational
allele code	4	Thing: observational
condensation polymerisation	4	Activity: observational
reduction division	4	Activity: observational
first division of meiosis	4	Activity: observational
eukaryotic dna	4	Thing: observational
mitotic cell	4	Thing: observational
seminiferous tubule	4	Thing: observational
parent strand	4	Thing: observational
lagging strand	4	Thing: observational
central nervous system	4	Thing: observational
strand of dna	4	Thing: observational
eukaryotic gene	4	Thing: observational
nuclear genome	4	Thing: observational
vertebrate animal	4	Thing: observational
daughter strand	4	Thing: observational
corpus luteum	4	Thing: observational
primordial follicle	4	Thing: observational
mRNA strand	4	Thing: observational
zygote cell	4	Thing: observational
defective gene	4	Thing: observational
genetic inheritance	4	Activity: observational
multicellular animal	4	Thing: observational
pelvic bone	4	Thing: observational
pituitary gland	4	Thing: observational

dna code	4	Thing: observational
light microscope	4	Thing: instrumental
gene mutation	4	Activity: observational
surface-area-to-volume ratio	4	Thing: observational
immune response	4	Activity: observational
marine organism	4	Thing: observational
tropical forest	4	Place
central dogma	4	Thing: observational
Archaean eon	4	Time
ostzygotic isolating mechanis	4	Activity: observational
respiration	3	Activity: observational
elongation	3	Activity: observational
fossilisation	3	Activity: observational
urohydrosis	3	Activity: observational
ribozyme	3	Thing: observational
pharmacogenomics	3	Activity: enacted
colchicine	3	Thing: observational
xenotransplantation	3	Activity: enacted
morula	3	Thing: observational
terminator	3	Thing: observational
spermatocyte	3	Thing: observational
brachiopod	3	Thing: observational
cuticle	3	Thing: observational
self-pollination	3	Activity: observational
thalassaemia	3	Thing: observational
macroevolution	3	Activity: observational
xylem	3	Thing: observational
zooplankton	3	Thing: observational
forensics	3	Activity: enacted
karyotyping	3	Activity: enacted
hermaphrodite	3	Thing: observational
tyrosine	3	Thing: observational
melanocyte	3	Thing: observational
brachiation	3	Activity: observational
fimbria	3	Thing: observational
megaspore	3	Thing: observational
electroporation	3	Activity: observational
macronucleus	3	Thing: observational
proteomics	3	Activity: enacted
anthocyanin	3	Thing: observational
copulation	3	Activity: observational
sporangium	3	Thing: observational
pellucida	3	Thing: observational
telomeres	3	Thing: observational
self-fertilization	3	Activity: observational
vacuole	3	Thing: observational
mosaicism	3	Activity: observational
flagellum	3	Thing: observational
self-replication	3	Activity: observational
hypothalamus	3	Thing: observational
chlorophyll	3	Thing: observational
amber	3	Thing: observational
eubacteria	3	Thing: observational
sterility	3	Activity: observational
amniotes	3	Thing: observational
Palaeozoic	3	Time
Neogene	3	Time
umbilical cord	3	Thing: observational

green algae	3	Thing: observational
vascular tissue	3	Thing: observational
vascular system	3	Thing: observational
malignant neoplasm	3	Thing: observational
semiconservative replication	3	Activity: observational
prolonged torpor	3	Thing: observational
exogenous DNA	3	Thing: observational
diploid gamete	3	Thing: observational
amino acid methionine	3	Thing: observational
mutant phenotype	3	Thing: observational
heterozygous genotype	3	Thing: observational
spacer DNA	3	Thing: observational
prokaryotic chromosome	3	Thing: observational
haemoglobin protein	3	Thing: observational
eukaryotic chromosome	3	Thing: observational
flowerless seed plant	3	Thing: observational
disease-causing mutation	3	Activity: observational
fan-shaped tail	3	Thing: observational
sickle-shaped cell	3	Thing: observational
genetic sequence	3	Thing: observational
second division of meiosis	3	Activity: observational
DNA segment	3	Thing: observational
female reproductive structure	3	Thing: observational
autosomal gene	3	Thing: observational
tile DNA	3	Thing: observational
parental DNA	3	Thing: observational
eye gene	3	Thing: observational
plasmid DNA	3	Thing: observational
ancestral hominoid	3	Thing: observational
membrane-bound organelle	3	Thing: observational
double-stranded helix	3	Thing: observational
antifreeze protein	3	Thing: observational
hydrothermal vent theory	3	Semiotic: theory
male cone	3	Thing: observational
polyploid plant	3	Thing: observational
polyploid zygote	3	Thing: observational
foramen magnum	3	Thing: observational
bacterial plasmid	3	Thing: observational
enzyme dna	3	Thing: observational
zona pellucida	3	Thing: observational
uterine contraction	3	Activity: observational
complementary dna	3	Thing: observational
dna ligases	3	Thing: observational
cell plate	3	Thing: observational
rainforest biome	3	Thing: observational
plasma membrane	3	Thing: observational
omozygous recessive genotyp	3	Thing: observational
resistant bacterium	3	Thing: observational
human gamete	3	Thing: observational
spindle fibre	3	Thing: observational
ngle nucleotide polymorphis	3	Activity: enacted
disease-causing bacterium	3	Thing: observational
second division	3	Activity: observational
protein chain	3	Thing: observational
haploid plant	3	Thing: observational
hydrogen cyanide	3	Thing: observational
digestive enzyme	3	Thing: observational
capillary electrophoresis	3	Activity: observational

plant tissue	3	Thing: observational
food chain	3	Thing: observational
captive breeding	3	Activity: observational
soil bacterium	3	Thing: observational
bacterial chromosome	3	Thing: observational
Holocene epoch	3	Time
Carboniferous period	3	Time
Cryogenian period	3	Time
mutualism	2	Activity: observational
chiasmata	2	Thing: observational
photonasty	2	Thing: observational
acrosome	2	Thing: observational
epistasis	2	Activity: observational
nucleosome	2	Thing: observational
fossilization	2	Activity: observational
chiasma	2	Thing: observational
lithification	2	Activity: observational
helicase	2	Thing: observational
genomics	2	Activity: enacted
tetraploid	2	Thing: observational
cytosol	2	Thing: observational
rRNA	2	Thing: observational
arginine	2	Thing: observational
echinoderm	2	Thing: observational
ectoderm	2	Thing: observational
diatom	2	Thing: observational
endoderm	2	Thing: observational
mycelium	2	Thing: observational
mitospores	2	Thing: observational
endosperm	2	Thing: observational
transgene	2	Thing: observational
epiphyte	2	Thing: observational
metamorphism	2	Activity: observational
radioisotope	2	Thing: instrumental
carbonization	2	Activity: observational
cytoskeleton	2	Thing: observational
planula	2	Thing: observational
epigenetics	2	Activity: observational
endotherms	2	Thing: observational
mesoderm	2	Thing: observational
dinucleotide	2	Thing: observational
cytokines	2	Activity: observational
thermocycler	2	Thing: instrumental
phototropism	2	Activity: observational
luciferin	2	Thing: observational
mutualism disruption	2	Activity: observational
immune system	2	Thing: observational
sugar-phosphate chain	2	Thing: observational
sickle-cell allele	2	Thing: observational
ostzygotic isolating mechanis	2	Activity: observational
x-linked recessive allele	2	Thing: observational
defective allele	2	Thing: observational
single x chromosome	2	Thing: observational
DNA ladder	2	Thing: observational
double helix DNA	2	Thing: observational
synthesis of tryptophan	2	Activity: observational
amino acid valine	2	Thing: observational
coral polyp	2	Thing: observational

principle of inheritance	2	Semiotic: theory
arboreal adaptation	2	Activity: observational
white blood cell	2	Thing: observational
female orangutan	2	Thing: observational
single dna sequence	2	Thing: observational
receptor gene	2	Thing: observational
w gamete	2	Thing: observational
homozygous genotype	2	Thing: observational
multiple fission	2	Activity: observational
snap-dragon plant	2	Thing: observational
diploid zygote cell	2	Thing: observational
circular dna chromosome	2	Thing: observational
seed pod	2	Thing: observational
r cell	2	Thing: observational
haploid sperm cell	2	Thing: observational
poly-a tail	2	Thing: observational
amino acid glycine	2	Thing: observational
hydrogen sulfide	2	Thing: observational
vitro fertilisation	2	Activity: observational
heirloom plant	2	Thing: observational
synthetic enzyme	2	Thing: observational
fungus cell	2	Thing: observational
second x chromosome	2	Thing: observational
vascular plant	2	Thing: observational
chromosome duplication	2	Activity: observational
amniotic egg	2	Thing: observational
pigment allele	2	Thing: observational
strand of mRNA	2	Thing: observational
primary transcript	2	Thing: observational
ribosomal protein	2	Thing: observational
non-coding strand	2	Thing: observational
non-coding sequence	2	Thing: observational
segregation of chromosome	2	Activity: observational
second filial generation	2	Thing: observational
haploid ovum	2	Thing: observational
non-ionising radiation	2	Thing: observational
regulatory protein	2	Thing: observational
internal membrane	2	Thing: observational
germ-line mutation	2	Activity: observational
transverse fission	2	Activity: observational
defective gamete	2	Thing: observational
longitudinal fission	2	Activity: observational
mitotic cell division	2	Activity: observational
single polynucleotide chain	2	Thing: observational
morphological adaptation	2	Activity: observational
physical mutagen	2	Thing: observational
blood protein	2	Thing: observational
DNA transcription	2	Activity: observational
penicillium expansum	2	Thing: observational
intron mutation	2	Activity: observational
beta thalassaemia	2	Thing: observational
seed coat	2	Thing: observational
polyploid cell	2	Thing: observational
tetraploid organism	2	Thing: observational
white eye gene	2	Thing: observational
anterior pituitary gland	2	Thing: observational
pigeon breeder	2	Thing: observational
mosquito larva	2	Thing: observational

molecular clock hypothesis	2	Semiotic: hypothesis
seed dispersal	2	Activity: observational
eukaryotic cell cycle	2	Activity: observational
biological mutagen	2	Thing: observational
nutrient cycle	2	Activity: observational
animal cell	2	Thing: observational
anaerobic respiration	2	Activity: observational
hoofed animal	2	Thing: observational
desert animal	2	Thing: observational
yeast cell	2	Thing: observational
scarlet dye	2	Thing: instrumental
intrauterine insemination	2	Activity: enacted
human mitochondrial genome	2	Thing: observational
anaphase i of meiosis	2	Activity: observational
noncoding dna	2	Thing: observational
principle of uniformitarianism	2	Semiotic: theory
homologous chromosome line	2	Thing: observational
hypothesis of common descent	2	Semiotic: hypothesis
amino acid bond	2	Thing: observational
lung cell	2	Thing: observational
eye-colour gene	2	Thing: observational
semi-conservative replication	2	Activity: observational
marine invertebrate	2	Thing: observational
skin pigment	2	Thing: observational
sugar deoxyribose	2	Thing: observational
monohybrid crosse	2	Thing: observational
nitrogen-containing base	2	Thing: observational
uterine lining	2	Thing: observational
carotid rete system	2	Thing: observational
meiotic division	2	Activity: observational
adjacent nucleotide	2	Thing: observational
invertebrate organism	2	Thing: observational
nuclear gene	2	Thing: observational
amino acid chain	2	Thing: observational
sperm nucleus	2	Thing: observational
finger bone	2	Thing: observational
single uterus	2	Thing: observational
germ-line cell	2	Thing: observational
paralytic phenotype	2	Thing: observational
primary spermatocyte	2	Thing: observational
haemophiliac allele	2	Thing: observational
defective enzyme	2	Thing: observational
ancestral anthropoid	2	Thing: observational
globular protein	2	Thing: observational
conjugated protein	2	Thing: observational
fibrous protein	2	Thing: observational
protein expression	2	Activity: observational
non-functional protein	2	Thing: observational
disulfide bridge	2	Thing: observational
uorescence in situ hybridisati	2	Activity: enacted
cell mutation	2	Activity: observational
lethal mutation	2	Activity: observational
shoulder joint	2	Thing: observational
embryonic germ	2	Thing: observational
amniotic cavity	2	Thing: observational
primordial soup hypothesis	2	Semiotic: hypothesis
ib allele	2	Thing: observational
fluorescent protein	2	Thing: observational

indirect oncogenic virus	2	Thing: observational
leaf abscission	2	Activity: observational
direct oncogenic virus	2	Thing: observational
multicellular eukaryotes	2	Thing: observational
bacterial host	2	Thing: observational
monotreme mammal	2	Thing: observational
template dna	2	Thing: observational
recombinant plasmid DNA	2	Thing: observational
accessory gland	2	Thing: observational
photosynthetic cyanobacteria	2	Thing: observational
photosynthetic organism	2	Thing: observational
non-recombinant plasmid	2	Thing: observational
tropical rainforest biome	2	Thing: observational
single parent cell	2	Thing: observational
germline cell	2	Thing: observational
nucleus of cell	2	Thing: observational
male reproductive system	2	Thing: observational
male reproductive structure	2	Thing: observational
hind limb	2	Thing: observational
circadian rhythm	2	Activity: observational
translocation mutation	2	Activity: observational
single-stranded DNA	2	Thing: observational
amino acid phenylalanine	2	Thing: observational
diploid tissue	2	Thing: observational
cephalopod eye	2	Thing: observational
human sex chromosome	2	Thing: observational
ampicillin resistance gene	2	Thing: observational
principle of evolution	2	Semiotic: theory
inversion mutation	2	Activity: observational
lominant-recessive interactio	2	Activity: observational
australopithecine skeleton	2	Thing: observational
bioluminescent organism	2	Thing: observational
primordial soup theory	2	Semiotic: theory
Pleistocene epoch	2	Time
Palaeogene	1	Time
Oligocene epoch	1	Time
