# Supplementary material for: A semantic classification of nominal technical terms in secondary school biology textbooks
Source: PLoS One. 2024 Nov 11;19(11):e0312040. doi: 10.1371/journal.pone.0312040 (PMC11554214; doi:10.1371/journal.pone.0312040)
Supplement: S1 Appendix — (PDF) [file pone.0312040.s002.pdf]

Appendix A list of secondary school biology textbooks used in this study

1. Berwald J, Fisher D, Lee KF, Mann KO, Ross DL, Zike D. *Focus on life science* (California Grade 7). Glencoe/McGraw-Hill; 2007.
2. Biggs A, Hagins WC, Holliday WG, Kapicka C, Lundgren L, MacKenzie AH, Rogers WD, Sewer MB, Zike D. *Biology* (Georgia ed.). Glencoe/McGraw-Hill; 2008.
3. Biggs A, Hagins WC, Kapicka C, Lundgren L, Rillero P, Tallman KG, Zike D. *Biology: The dynamics of life*. Glencoe/McGraw-Hill; 2004.
4. Wood R, Armstrong Z, Chapman D, Deeker W, Madden A, Maginn H, Naughton K. *Pearson biology New South Wales* (Student Book, Year 12). Pearson Australia; 2019.
5. Wood R, Armstrong Z, Deeker W, Madden A, Maginn H, McMahon K, Naughton K. *Pearson biology New South Wales (Student Book, Year 11)*. Pearson Australia; 2018.
6. Zhang J, Filan S, Hopley S, Morante R, Stanger J, Tilley C. *Oxford insight science: Australian curriculum for NSW, Year 10*. Oxford University Press; 2015.
7. Zhang J, Alford D, Hopley S, Tilley C. *Oxford insight science: Australian curriculum for NSW, Year 8*. Oxford University Press; 2014.
8. Zhang J, Alford D, McGowan D, Tilley C. *Oxford insight science: Australian curriculum for NSW, Year 7*. Oxford University Press; 2013.
9. Zhang J, Alford D, Morante R, Tilley C. *Oxford insight science: Australian curriculum for NSW, Year 9*. Oxford University Press; 2014.
