# Supplementary material for: A semantic classification of nominal technical terms in secondary school biology textbooks
Source: PLoS One. 2024 Nov 11;19(11):e0312040. doi: 10.1371/journal.pone.0312040 (PMC11554214; doi:10.1371/journal.pone.0312040)
Supplement: S1 File — (DOCX) [file pone.0312040.s003.docx]

**Textbook 1: NSW Oxford Insight Science Year 7**

3.1 Looking at cells

In 1665, Robert Hooke became the first person to discover and describe cells. Using one of the first **microscopes** ever made, Hooke observed many types of living things and made accurate drawings of what he saw. One of Hooke’s most famous scientific achievements was his diagram of very thin slices of cork. He was surprised to see that, under the **microscope**, the cork looked like a piece of honeycomb. He described the ‘holes’ and their boundaries in the ‘honeycomb’ as cells because they reminded him of the rooms in a monastery. Hooke had discovered plant cells.

Hooke also examined animals under the **microscope**, including a flea. His detailed picture of his observations can be seen in Figure 3.2. Anton van Leeuwenhoek improved the **microscope** and saw many more cells than Hooke.

It was because of Hooke and Leeuwenhoek’s important contributions to microbiology that other scientists went on to develop a further understanding of cells.

Cell theory

**Cell theory** describes the main ideas about the importance of cells and their role in living things. It was first proposed in 1839 by two German biologists, Theodor Schwann and Matthias Schleiden. In 1858, German scientist Rudolf Virchow completed the classic cell theory.

The combined **cell theory** includes the following three principles:

•All organisms are composed of one or more cells.

•Cells are the basic unit of life and structure.

•New cells are created from existing cells.

MICROSCOPES AND MICROSCOPY

Historically, the first **microscopes** were very basic. Over time their magnifying ability has improved and continues to do so. Scientists can now look at images that have been magnified thousands of times using various systems of lenses. This makes it possible to study the structure of cells.

You have probably seen people use magnifying glasses either to read or to enlarge a viewed object. The glass or plastic lenses magnify the object viewed. In the same way, **microscopes** magnify the size of the object placed under them.

Light microscopes

As a science student you will probably use two types of **light microscopes**: the **stereo microscope** and the **compound light microscope**. As the name suggests, they use light to assist viewing.

The **compound light microscope** is used to observe thin slices of specimens, such as blood cells. It can magnify up to 1500 times. Its view is flat—that is, two dimensional. The specimen must be thin enough to allow light to pass through it. Major structures within individual cells can be seen with a **compound light microscope**.

The **stereo microscope** is used for viewing larger objects, such as insects. It can magnify up to 200 times and shows the surface of the object viewed, giving the object a three-dimensional view. It cannot be used to see inside or through an object, making it difficult to see individual cells.

The **stereo microscope** has two eyepieces to look through, whereas the **compound light microscope** can have one or two eyepieces.

The word ‘monocular’ is used to describe a microscope with one eyepiece (mono meaning one). **Microscopes** with two lenses are called binocular (bi meaning two). The **compound light microscope** uses the effect of two lenses (the lens in the eyepiece and the objective lens further down the column) combined with light to give a greater magnification.

Preparing cells for microscopy

To look at cells clearly through a **compound microscope**, very thin layers of a specimen must be used. The light must be able to pass through the specimen (the object you are looking at), otherwise all you will see is a dark shadow.

Specimens are prepared by taking a very thin slice of the object, using a very sharp blade or even a laser. Most cells are clear, which makes them difficult to see, so a stain such as iodine or methylene blue is used to help make them more visible. Different stains are used to highlight different components of the cells.

Placing a cover slip over the top of the stained specimen helps keep the sample in place and helps to protect the specimen itself. The coverslip can be sealed onto the slide creating a preserved and reusable specimen. You may use a number of these prepared slides during the experiments in this book. You may also have to hone your skills and prepare some of your own slides to examine.

3.2 Cell structure and function

CELL COMPONENTS

The **cell theory** states that all organisms are composed of one or more cells.

Organisms like bacteria and amoebas, are often single celled. These tiny unicellular organisms are also called microorganisms because they are often only visible through a **microscope**.

Multicellular (multi meaning lots) organisms are made up of many cells joined together. All cells share the same basic structure regardless of the type of organism they are in. This structure includes three key components:

•cell membrane—the ‘skin’ of a cell, forming a double-layered barrier around it. It controls the entry and exit of things into and out of the cell.

•cytoplasm—the ‘jelly-like’ fluid inside the **cell** between the **membrane** and the **nucleus**. It contains all the cell organelles (mini organs), dissolved nutrients and wastes, and helps provide structure for the cell.

•DNA (deoxyribonucleic acid)—a complex chemical that provides the instructions for every job cells need to do, and is passed from one generation to the next. The code for half of your DNA came from your mother in the egg, and the other half came from your father in the sperm. The same complete set of DNA is found in each of your cells.

DIFFERENT CELLS

By looking at the characteristics of different living things, it’s fairly easy to see that they are different types of organisms. It’s not hard to tell an apple from an elephant! As more powerful **microscopes** were developed, scientists could see there were basic differences between plant and animal cells. This made sense—if cells are the basic building blocks and the basic units differ then the final living things will have different characteristics.

We can classify cell cells based on whether they have organelles or not, as well as the types of organelles they have. For example, bacteria do not have any true organelles but still contain DNA. Their **DNA** floats around inside the **cytoplasm** rather than being held within a nucleus. Many of these bacterial cells also have a cell wall, but it is made of a different chemical to the cell walls found in plant cells. Plant cells and animal cells all contain organelles, however, plant cells tend to have one large vacuole while animal cells often have many smaller vacuoles. Plant cells also contain chloroplasts whereas animal cells do not. Look back at Figure 3.13 and see if you can identify the other differences between typical plants, animal and bacterial cells.

Measuring cells and their organelles

Can you imagine measuring the distance between Melbourne and Sydney in millimetres? Selecting appropriate units of measurement means you do not need to deal with really large or really small numbers.

Because cells are microscopic, we need an appropriate unit of measurement to measure them and their parts.

Look at 1 millimetre on your ruler. Now imagine this 1 millimetre is divided into a thousand parts. One of those tiny parts is equal to 1 micrometre (μm). Cells and their parts are measured in micrometres (sometimes called microns). Cells vary in size depending on their function. A bacterial cell usually measures approximately 1 μm, whereas a plant cell may be up to 100 μm in size (equivalent to one-tenth of a millimetre).

A CLOSER LOOK AT RESPIRATION

**Mitochondria** (singular mitochondrion) are the powerhouse of cells as it is the place where a cell’s energy is produced. There may be several thousand mitochondria in a **cell**, depending on what the cell does. For example, muscle cells contain a lot of mitochondria to make sure we have enough energy to run and jump when we need to.

Mitochondria are rod-shaped organelles with an inner and outer membrane.

Mitochondria have their own DNA, make some of their own proteins, and are able to grow and divide when a cell needs more of them. They are usually too small to see with a **compound light microscope**.

**Cellular respiration** occurs inside **mitochondria**. In this process, glucose (from the food we eat) and oxygen react to form water, carbon dioxide and energy. This energy is used by our bodies to help us function.

Whenever you burn a fuel, such as wood or oil, you release energy that has been stored in that fuel. Burning is a very rapid process that requires oxygen, and produces a lot of heat energy, carbon dioxide and water. **Cellular respiration** is similar to burning. Pay attention to your breathing the next time you walk up a hill. Your body is working harder to deliver oxygen to your exercising muscle cells so they can make fuel, and you are ‘puffing’ out the waste carbon dioxide.

It is important not to confuse respiration with breathing. Respiration is a chemical reaction that occurs in the mitochondria inside cells. Breathing is a process performed by multicellular organisms that have lungs or gills to exchange gases with the environment. Breathing usually involves taking oxygen into the body and expelling carbon dioxide back into the environment.

CELL DIVISION

Cells, like organisms, need to carry out many functions to survive. They need to process many substances, harness energy and, ultimately, reproduce. Cells can also become damaged, grow old and die. New cells are made to replace old or damaged cells. The instructions for all these jobs are in the form of DNA — lengths of codes that can be ‘read’ when required to make sure jobs are done correctly. The DNA is usually stored in the **nucleus**, which is often referred to as the control centre of the cell.

When cells are ready to reproduce they divide into two identical daughter cells. If it were this simple though, each cell division would result in daughter cells with only half the required amount of DNA. So what must happen inside the parent cell before it divides? The amount of DNA in the cell doubles in a process called DNA replication. This produces two exact copies of the DNA so each daughter cell can have its own copy. Many of the organelles are also replicated and divided evenly between the daughter cells. When this type of cell division occurs in **multicellular organisms** it is for growth and repair, and is called mitosis. Unicellular organisms reproduce (produce new organisms) with a very similar type of cell division called binary fission.

3.3 Different types of cells

HOW ORGANISMS FUNCTION

Cells are often called ‘the building blocks of life’. Think of the way bricks are used to build a house. Cells build living things in a similar way, however, there are usually many more cells in living things than bricks in a house—an adult human body is made up of about ten trillion (10000000000000) cells. Elephants have even more. Any living thing that has more than one cell is referred to as multicellular (multi meaning many), but there are many living things, such as bacteria, that consist of only one cell. These are called single-celled or unicellular organisms. Figure 3.24 shows two unicellular organisms.

Cells come in lots of different shapes and sizes. Some have branches like trees. Others are more like saucers, or rods.

These differences are usually a result of the different jobs the cells need to do.

Unicellular organisms can survive on their own without the help of other cells. Many people think of them as just simple creatures but there is nothing simple about them. Their single cell is complex, making the organism itself complex. These unicellular organisms have the mechanisms to move, reproduce and sense the environment, all contained in one cell!

A multicellular organism has many different cells that are more specialised than the single cell of a unicellular organism. The cells in a **multicellular organism** have lost their independence and cannot survive on their own outside the organism. The entire organism depends upon the health of all its cells to survive. Each cell has a separate job to do; nerve cells cannot do what muscle cells do.

Input and output systems

All living things take in materials from the environment that enable them to live, grow and reproduce. Waste products are excreted back into the environment.

In **unicellular organisms**, such as amoebas, the materials simply pass across into and out of the cell across the cell membrane.

In **multicellular organisms**, most of the cells are not in direct contact with the environment so they need to pass the materials onto other cells in order to function correctly.

Multicellular organisms have systems within their structure that take nutrients and oxygen to every cell and remove waste products, such as carbon dioxide, and transport them back out to the environment.

Cells, tissues and organs

All cells in our body are very similar as they are all animal cells, but each different type has a special role and is different from the others. Cheek cells are not the same as stomach cells. Hair cells are not the same as muscle cells. But all these different types of cells have a cell membrane, nucleus, mitochondria and other organelles that enable the cell itself to survive. Even though they might have the same organelles, each cell type is specialised and necessary to do a specific job to keep you alive. For example, muscle cells have many more mitochondria than skin cells because they need more energy.

These specialist cells work together in teams. Groups of cells that do a similar task are called tissues. We have skin tissue (also called epithelial tissue), muscle tissue, nerve tissue, bone tissue and so on. Groups of tissues that work together are called organs. The liver, heart, eyes, brain and intestines are all examples of organs.

Major body systems

When groups of different organs work together they are called a body system. In humans, the brain, spinal cord and a network of nerves make up the nervous system. Our mouth, oesophagus, stomach and intestines make up the digestive system.

Each organ has a specific role but the organs work together for a greater purpose. All the systems then connect to ensure the survival of the whole organism (us!). Each body system has a particular role in keeping the body alive.

ANATOMY OF SKIN

The skin as a body system doesn’t always get a lot of attention. It is something we see every day and perhaps does not seem as hidden or mysterious as our other body systems. But it has a crucial role to play in our survival. It does a lot more for us than just keeping our insides in!

It seems incredible that the average human adult has about 1.5–2 square metres of skin. If you spread out this skin it would be enough skin to cover a double bed!

Your skin is the body system sometimes called the covering system, but known medically as the integumentary system. The Latin word integumentum means ‘covering’. The integumentary system also includes your fingernails and hair.

Skin as an organ

Skin serves various functions in the body (Figure 3.32).

Human skin consists of three layers:

•epidermis

•dermis

•subcutaneous fat layer.

Epidermis

The epidermis is the outer layer of the skin— the part you can see. The main purpose of the epidermis is to protect the inner layers from external things getting into your body and to help control body temperature. The epidermis also contains melanin, which is a pigment that gives skin its colour (and its freckles).

The epidermis is made up of a number of layers of cells. The outer surface is constantly being shed by everyday actions, such as putting on clothes and washing. We lose about 1 gram of dead skin cells each day, which adds up to approximately 20–25 kilograms of dead skin cells over a lifetime! No wonder about 90% of household dust is really dead skin cells.

The bottom layer of the epidermis is constantly making new epidermal cells to replace the lost cells. It takes about a month for the new cells to move up from the base to the top of the epidermis. As old, dead skin cells are sloughed off the surface, new ones are pushed up to replace them. So all that scrubbing of your skin when you wash your face in the morning is important for healthy skin.

Dermis

The dermis is a thicker layer under the epidermis. The main role of the dermis is to support and strengthen the skin.

The dermis contains finger-like extensions called papillae. The papillae join the cushions organs dermis and epidermis together and hold the epidermis in place. The blood vessels found in the **dermis** provide the epidermis with nutrients. The majority of the dermis is made of collagen, which is a tough fibre. The dermis also contains another type of fibre, called elastic fibre, which assists the skin to return to its original shape after it has been stretched. Sensory nerves, which detect heat, pain and pressure, are also located in the **dermis**.

Subcutaneous fat layer

The deepest layer of skin contains fat. Subcutaneous means ‘under the skin’, so subcutaneous fat refers to the layer of fat at the bottom of the skin. This layer of fat consists not only of fat cells but also more blood vessels and nerves.

The three main roles of this layer of fat are to:

•store energy

•provide the body with insulation

•cushion the skin.

Glands

The skin contains two types of glands:

•sweat glands—these glands secrete moisture up through the pores on the skin surface when the body becomes overheated; the evaporation of this moisture cools the body

•oil glands—these glands are located in the walls of the hair follicles and produce the oil called sebum, which lubricates the skin and hair.

**Textbook 2: NSW Pearson Biology Year 11**

2.1 Cell types

COMMON CELL STRUCTURES

Cells are the basic structural unit of all living things. Although there are different types of cells, the cells of plants, animals and bacteria share a number of common structures (Figure 2.1.1). These include:

a cell membrane (also known as the plasma membrane)-separates the interior of the cell from the outside environment.

cytoplasm-consists of the cytosol and, in eukaryotes, the organelles.

Cytosol is a gel-like substance, made up of more than 80% water, and contains ions, salts and organic molecules.

•DNA-carries hereditary information, directs the cell's activities and is passed from parents to offspring

•ribosomes-organelles responsible for the synthesis of proteins.

CLASSIFICATION OF CELLS

There are two fundamentally different types of cells. Organisms are classified according to the cell type of which they are composed.

•Prokaryotes are composed of prokaryotic cells. They include bacteria and archaea. Prokaryotic cells are usually unicellular and are generally smaller and less complex than eukaryotic cells. The organelles of prokaryotic cells are not membrane-bound (Figure 2.1.2).

•Eukaryotes are composed of eukaryotic cells. They include protists, fungi, plants and animals. Eukaryotic cells contain membrane-bound organelles.

In older classification systems all organisms were divided into five ranks, called kingdoms. Prokaryotic organisms were placed in the kingdom Monera and eukaryotic organisms were placed in the kingdoms Protista, Plantae, Fungi and Animalia. These systems were based on the morphology (appearance and structure) of organisms.

In the late 1970s, the use of DNA techniques led to the discovery of two different types of prokaryotic cells. This discovery resulted in the development of a system with three domains and six kingdoms (Figure 2.1.3). Domains are now the highest rank in taxonomy, instead of kingdoms. Prokaryotes are divided into two domains: Bacteria and Archaea. All eukaryotic organisms are placed in a third domain called Eukarya. The four kingdoms within the Eukarya domain remain the same: Protista, Plantae, Fungi and Animalia (Figure 2.1.3).

PROKARYOTES

Prokaryotes are organisms that are made up of a single cell (unicellular). Bacteria, cyanobacteria (photosynthetic bacteria), and archaea, such as methanogens, are examples of prokaryotes. Prokaryotic organisms can be found everywhere-even in extreme environments such as volcanoes.

Most prokaryotic cells are small and therefore have a large surface area relative to their volume. This allows the cells to take in and release materials efficiently and replicate quickly.

The structure of a typical prokaryotic cell is shown in Figure 2.1.4. Prokaryote cells lack membrane-bound organelles, and their cytoplasm contains scattered ribosomes that are involved in the synthesis of proteins. The genetic material of prokaryotic cells is usually a single, circular DNA chromosome called the genophore, which is contained in an irregularly shaped region called the **nucleoid**. The nucleoid does not have a nuclear membrane like the nucleus of eukaryotes.

The prokaryotic chromosomal DNA is attached to the cell membrane by a region of the chromosome called the **origin**. In addition to this chromosomal DNA, many prokaryotic cells also contain small rings of double-stranded DNA called plasmids.

The cell membrane of prokaryotic cells is surrounded by an **outer cell wall**. Many bacteria also have a capsule outside the **cell wall**. The capsule protects the bacterial cell from damage, dehydration and engulfment by eukaryotic cells. It also helps the bacteria stick to surfaces. These features of bacterial capsules increase the virulence (ability to cause disease) of pathogenic bacteria.

Some prokaryotes can move around using a tail-like structure called a flagellum. Many prokaryotes have small hair-like projections called pili, which can also help to generate movement. Pili are also involved in the transfer of DNA between organisms. Specialised pili that can attach to surfaces are called fimbriae.

Bacteria

Most prokaryotes in the domain Bacteria are microscopic single-celled organisms. Fossil evidence dated to between 3. 7 and 4. 3 billion years old confirms that bacteria were the first type of living organism on Earth. Today they are still the most numerous type of organism in the biosphere.

Bacteria have very diverse metabolic systems, making them extremely adaptable. They can survive in almost every environment on Earth. Bacteria are common in moist, low-salt environments of moderate temperature, where sunlight or organic compounds are plentiful, and inside or on plants and animals.

Bacteria need little oxygen to survive, because they have many ways of extracting energy and fixing carbon. Bacteria can obtain energy from sunlight (**photosynthesis**) or by reducing inorganic compounds such as sulfides or ferrous ions (**chemosynthesis**).

Bacteria play an important role in ecosystems, because they break down many kinds of substances, including plant and animal remains and wastes. Bacteria are also widely used in industry to manufacture foods, such as cheeses and yoghurt, and in medicine, to produce antibiotics, drugs and even human insulin. Some bacteria can even break down oils and plastics, making them useful for pollution control.

Gram-positive and gram-negative bacteria

Bacteria have mesh-like cell walls that are made up of a polymer called peptidoglycan (also known as murein). Different species of bacteria have different cell wall characteristics. Based on the structure of their cell walls, different species of bacteria can be classified as gram-positive or gram-negative using a technique called Gram staining.

Gram staining involves adding a purple dye called crystal violet to bacterial cells. The dye interacts differently with the cell walls of gram-positive and gram-negative bacteria, staining the cells different colours. Gram-positive bacteria have a thicker layer of peptidoglycan that absorbs and holds the stain, so they give a purple or 'positive' result. Gram-negative bacteria have a much thinner layer of peptidoglycan that does not retain the stain as well, so they give a pink or 'negative' result (Figure 2. 1.5).

There are numerous types of gram-negative and gram-positive bacteria. For example, gram-positive cocci are spherical bacteria that include Staphylococcus and Streptococcus, which can cause serious diseases or death in humans (Figure 2.1.6). An example of a gram-negative bacterium is a cyanobacterium (Figure 2.1. 7a). Cyanobacteria were once called blue-green algae because they contain chlorophyll. We now know that cyanobacteria are actually prokaryotes, and so they are placed in the Bacteria domain. Cyanobacteria often form dense colonies in shallow estuaries or fresh water (Figure 2.1. 7b). Some species form large colonies, known as blooms, which produce toxins. These toxic blooms can kill fish and other aquatic life and cause illness in humans.

Archaea

The prokaryotes in the domain Archaea include extremophiles. These are organisms that can live in extreme conditions, such as:

areas of high temperatures (thermophiles) areas of low temperatures

the upper atmosphere

alkaline environments

acidic environments (acidophiles)

salty environments (halophiles) environments with little or no oxygen areas without light

petroleum deposits deep underground.

Archaea hold records for living in the hottest places (121 °C), the most acidic environments (pH 0), and the saltiest water (about 30% salt). However, some archaea live in less extreme environments such as the open seas. There are many different types of extremophiles. Hyperthermophiles such as Pyrococcus furiosus can survive in very hot environments such as undersea vents, where temperatures are often above 100°C (Figure 2.1.8). They can also withstand extremely high pressures. Sulfolobus is a genus of archaea that live in volcanic springs. Sulfolobus are thermophiles as well as acidophiles, because they can survive in both high temperatures and high acidity (Figure 2.1. 9).

Although archaea and bacteria are now known to be very different organisms, scientists did not recognise these groups as distinct for a long time. Many species of archaea and bacteria look very similar. The extreme habitats that archaea occupy also make them difficult to find and culture in a laboratory.

The ability of archaea to live in extreme environments is due in part to their unique cell membranes. Like other living organisms, archaea possess a cell membrane composed mainly of lipids. Cell membranes need to be fluid to enable cells to rapidly respond to external conditions and allow proteins to easily move in and out of cells. You will learn more about the structure of cell membranes in Section 2.3.

The lipids that compose the cell membranes of archaea are different from the lipids **in eukaryotic cell membranes**. The cell membranes of archaea form a unique structure that remains fluid and selectively permeable (semipermeable) over a wide range of temperatures-from freezing cold to boiling hot. The lipids in eukaryotic cell membranes have fluidity and selective permeability, but only in a narrow range of temperatures.

Differences between bacteria and archaea

Despite their name, archaea are not the most ancient group of organisms. DNA studies have shown that bacteria are the most ancient group, and that archaea evolved from eukaryotic cells at a later time.

The cells of bacteria and archaea are different in several ways:

•Archaea have a different type of lipid structure in the cell membrane.

•The cell wall in bacteria contains peptidoglycan, but the cell wall in archaea does not (although there is a similar compound in some archaea).

•Both have diverse metabolic systems, but methanogenesis (in which methane is produced) is unique to archaea.

EUKARYOTES

The cells of eukaryotes are much larger and more complex than prokaryotic cells (Table 2.1.1). Eukaryotic cells have a cell (plasma) membrane that surrounds the cell's cytoplasm and internal (non-plasma) membranes that form specialised compartments within the cell. The membrane-bound structures in **eukaryotic cells** are called organelles. Cell compartmentalisation and organelles will be discussed further in Section 2.2.

Eukaryotic organisms are incredibly diverse. There are unicellular and multicellular forms and organisms that can reproduce asexually and sexually. Multicellularity and sexual reproduction are unique to eukaryotes. Eukaryotic organisms only represent a small proportion of all species on Earth, but because eukaryotes are much larger in size, their total biomass is about the same as that of prokaryotes.

Eukaryotes are divided into the four kingdoms, Protista, Fungi, Plantae and Animalia. The cells of these groups share many typical eukaryotic features, but they also have cell structures and functions that are unique (Figures 2.1.10 and 2.1.11).

Animal and plant cells

Different groups of eukaryotic organisms can have different cellular structures. The organelles are involved in specific cellular functions, so their presence depends on the needs of the cells. A good way to understand this is to compare animal and plant cells.

Animal and plant cells are very similar. They both contain a nucleus surrounded by **cytoplasm**, which is enclosed by the **cell membrane**. They have mitochondria (singular mitochondrion) for **cellular respiration**, and organelles, such as the **Golgi apparatus**, in which proteins are synthesised and processed. However, plant and animal cells also differ in several ways (Figure 2.1.11 and Table 2.1.2): Plant cells have cell walls made from cellulose outside the **cell membrane**. The cell wall provides structural support and results in a fixed shape. Animal cells do not have cell walls.

Plant cells have a large, permanent vacuole that stores minerals and nutrients in a solution called **cell sap**. The vacuole also provides structure to plant cells by maintaining turgor pressure against the cell wall. Animal cells have many small temporary fluid-filled vacuoles called vesicles, but these do not provide structural support.

Plant cells have chloroplasts, which are the site of **photosynthesis**. Animal cells do not contain chloroplasts and do not perform **photosynthesis**.

COMPARISON OF PROKARYOTIC AND EUKARYOTIC CELLS

Prokaryotic and eukaryotic cells differ in several ways (Table 2.1.1 and Table 2.1.2):

Prokaryotic cells do not have membrane-bound organelles, while eukaryotic cells have many different membrane-bound organelles, with specialised structures and functions.

Prokaryotic cells do not have a nucleus, and their DNA is in the form of a single, circular chromosome and small, circular molecules called plasmids. The DNA of eukaryotic cells is in the form of linear chromosomes and is contained in the **nucleus**.

Prokaryotic cell walls are made of peptidoglycan. The cells of some eukaryotes, such as plants, fungi (singular fungus) and protists, are surrounded by a cell wall composed of carbohydrates.

Prokaryotic and eukaryotic cells also differ significantly in size. The typical eukaryotic cell is around 10 times larger than most prokaryotic cells.

2.2 Cell organelles

In the previous section, you learnt that the two fundamentally different types of cells are prokaryotic and eukaryotic cells, and that organisms are classified into one of three domains (Bacteria, Archaea or Eukarya) according to the type of cell they have.

Bacteria and archaea are prokaryotes: their cells do not contain membrane­bound organelles. Animals, plants, fungi and protists are eukaryotes. Each represents a kingdom in taxonomy: Animalia, Plantae, Fungi and Protista. There are many different types of cells within the four eukaryotic kingdoms. Although these cells have very different appearances and functions, they all contain membrane-bound organelles (Figure 2.2.1).

In this section, you will learn about the importance of cell compartmentalisation and membrane-bound structures in eukaryotes. You will also learn more about the structure and function of organelles and the differences between plant and animal cells.

COMPARTMENTALISATION IN EUKARYOTIC CELLS

As you learnt in the previous section, the two main types of cells are prokaryotic and eukaryotic cells. •Prokaryotic cells are relatively small and lack membrane-bound organelles. Bacteria and archaea are prokaryotes.

•Eukaryotic cells are relatively large and more complex. They possess membrane­bound organelles such as a nucleus and mitochondria. Protists, fungi, plants and animals are called eukaryotes because they are composed of eukaryotic cells. As well as a cell membrane surrounding the cytoplasm, eukaryotes have internal membranes that form specialised membrane-bound compartments within the cell. This is known as cell compartmentalisation. The membrane-bound compartments are called organelles. However, not all organelles have membranes (Figure 2.2.2).

Each membrane-bound organelle has a different function. For this reason, each organelle requires a different internal composition, including a high concentration of enzymes and reactants that are needed for the organelle's particular function.

Role of organelle membranes

The membranes surrounding organelles control the movement of substances between the organelle and the cell's cytosol (the liquid part of the cytoplasm). The outer cell membrane enables the cytosol to have a different composition from the cell's surrounding environment. In the same way, membranes of membrane­bound organelles enable each organelle to have a different composition from the surrounding cytosol and other organelles.

Benefits of compartmentalisation

Cellular compartmentalisation benefits the cell by:

•allowing enzymes and reactants for a particular cellular function to be close together in high concentrations and at the right conditions, such as at optimum pH levels, so that the processes within the organelles are very efficient

•allowing processes that require different environments to occur at the same time, in the same cell

•making the cell less vulnerable to changes to its external environment, because any changes will affect the cytosol much more than the membrane-bound organelles.

MEMBRANE-BOUND AND NON-MEMBRANE-BOUND ORGANELLES

Organelles are subcellular structures that have specific functions within the cell (Table 2.2.1). Some organelles, as previously mentioned, are membrane-bound compartments within the cytoplasm. Membrane-bound organelles are only present in **eukaryotic cells**.

Prokaryotic cells have some non-membrane-bound organelles, such as ribosomes, a cell wall and sometimes flagella, although the structure and composition of these are usually different from those of eukaryotic cells.

FUNCTION AND ULTRASTRUCTURE OF ORGANELLES

Cellular organelles are involved in several different functions (Table 2.2.2). These include protein and lipid synthesis and processing, energy transformation, storage and maintaining cell structure.

Synthesis and processing of proteins and lipids

Organelles involved in the synthesis and processing of proteins and lipids in eukaryotic cells are the nucleus, ribosomes, endoplasmic reticulum, Golgi apparatus and lysosomes.

Nucleus

In eukaryotes, the nucleus is a large organelle surrounded by a double-layered nuclear membrane. The nuclear membrane contains pores that link it with the cytosol (Figure 2.2.4). The nucleus contains most of the genetic material, which is formed in linear chromosomes composed of DNA and proteins. Chromosomes are usually not clearly visible, except during cell division. The most visible structure inside the **nucleus** of a non-dividing cell is the **nucleolus**. The nucleolus is composed of proteins, DNA and RNA, and is where ribosomes are assembled.

The information for the synthesis of new proteins is present in genes within the DNA. The genes are transcribed from the DNA into copies known as messenger RNA (mRNA). The mRNA travels through the nuclear membrane's pores into the cytosol and to the ribosomes, where the new proteins are made.

Ribosomes

Cells contain many thousands of ribosomes. These are only about 30 nanometres (nm) in diameter, and can therefore only be seen under an **electron microscope**. Ribosomes are composed of proteins and ribosomal RNA (rRNA), and are the sites of protein synthesis. They translate the sequence of amino acids specified by the mRNA into proteins. Ribosomes do not have a membrane surrounding them; they are non-membrane-bound organelles.

Ribosomes consist of two subunits joined together (Figure 2.2.5). The subunits in eukaryote ribosomes are different from those in prokaryote ribosomes.

Ribosomes are found either free in the cytosol, or bound to endoplasmic reticulum. When ribosomes are bound to the endoplasmic reticulum, it is called the rough endoplasmic reticulum. Proteins produced in free ribosomes will function in the cell's cytosol, while proteins synthesised in ribosomes bound to the endoplasmic reticulum are secreted out of the cell, packaged into organelles or inserted into cell membranes.

Endoplasmic reticulum

The endoplasmic reticulum is a network of intracellular membranous sacs (cisternae) and tubules. It links with the cell membrane and other membranous organelles, including the nucleus.

The endoplasmic reticulum can be rough or smooth. Rough endoplasmic reticulum has ribosomes attached (Figure 2.2.6). After the ribosomes have translated mRNA into proteins, the proteins pass into the endoplasmic reticulum cavity, which contains enzymes. The enzymes add sugar molecules to the proteins to form glycoproteins. Rough endoplasmic reticulum is abundant in cells that actively produce and export proteins, such as pancreatic cells, which secrete digestive enzymes. From the rough endoplasmic reticulum, proteins move into the Golgi apparatus for export from the cell.

Smooth endoplasmic reticulum does not have ribosomes attached. It contains the enzymes involved in the synthesis of molecules other than proteins, such as phospholipids and steroids. Smooth endoplasmic reticulum is abundant in steroid­secreting cells in the testes, ovaries, kidneys and adrenal glands (Figure 2.2.7).

Golgi apparatus

The Golgi apparatus is also called the Golgi body or Golgi complex. It is a stack of flattened, smooth membrane sacs called cisternae (Figure 2.2.8).

Unlike the cisternae found in the **rough endoplasmic reticulum**, the cisternae in the **Golgi apparatus** are not connected. When proteins formed in the **rough endoplasmic reticulum** reach the Golgi apparatus, vesicles are formed from each cisternae. The vesicles transport the proteins from one cisternae to the next, where they are modified for use by the cell, or for transport out of the cell.

The cisternae then form transport vesicles to move the modified proteins into the cytosol, into other organelles, or out of the cell. For example, digestive enzymes sent to lysosomes are not released from the cell, while secreted hormones are exported from the cell. Other vesicles budding from the Golgi apparatus carry membrane­bound proteins to the cell membrane.

The Golgi apparatus has two faces: the cis face and the trans face (Figure 2.2.9). The cisternae of the cis face are connected to the endoplasmic reticulum, either directly or by small transport vesicles. This allows the proteins made in the **rough endoplasmic reticulum** to enter the Golgi apparatus. The cisternae of the trans face are connected to the cell membrane by large, secretory vesicles that contain proteins to be secreted outside the cell. The membranes of the cis face more closely resemble the membranes of the endoplasmic reticulum, while the membranes of the trans face more closely resemble the cell membrane.

Secretory cells have a well-developed Golgi apparatus, but in other cells, the Golgi apparatus is small.

Lysosomes

Lysosomes are the cell's recycling units-specialised vesicles that digest unwanted matter (Figure 2.2.11). They are found in animal cells, and lysosome-like structures have been found in some plant cells. Lysosomes are formed when a transport vesicle containing enzymes is released from the Golgi apparatus, and fuses with another vesicle called an endosome. The endosome contains molecules brought into the cell by the process of endocytosis.

Lysosomes fuse with vesicles containing unwanted matter, such as damaged organelles or foreign matter. The enzymes in the lysosome then digest the unwanted matter. Small molecules that the cell can reuse may diffuse back into the cytoplasm. The rest are either retained in the **lysosome**, or released from the cell by the process of exocytosis.

Summary: Synthesis and processing of proteins and lipids

Protein and lipid synthesis and processing are shown in Figure 2.2.12. DNA is transcribed inside the nucleus into mRNA, which moves out of the nucleus and binds to ribosomes. Ribosomes synthesise proteins using the information on the mRNA. Proteins that will be secreted out of the cell are made in the **ribosomes** bound to the rough endoplasmic reticulum. These proteins are modified and packaged in the **Golgi apparatus**. Vesicles arising from the Golgi apparatus can fuse with the cell membrane, releasing their contents from the cell, or can insert membrane-bound proteins into the cell membrane. Lipids are synthesised and processed in the **smooth endoplasmic reticulum**.

Energy transformations

Mitochondria and chloroplasts are the organelles involved in energy transformations within eukaryotic cells.

Mitochondria

Mitochondria are organelles composed of two membranes. The inner membrane of the mitochondria has folds called cristae (Figure 2.2.13). There are two different compartments inside **mitochondria**: an intermembrane space and the matrix. The matrix is the fluid-filled space enclosed by the inner membrane and contains a double-stranded DNA molecule. Different enzymes are found inside each compartment and on each membrane.

Mitochondria play an important role in **cellular respiration**, converting the chemical energy in organic molecules (from food) into energy that cells can use. **The inner mitochondrial membranes** are the site of the chemical reactions of **cellular respiration**. The highly folded structure of the inner membranes increases the surface area over which these chemical reactions can take place.

The number of mitochondria in a cell is related to the cell's energy requirements. Very active cells, such as heart muscle cells, have many thousands of mitochondria.

Chloroplasts

Chloroplasts are organelles involved in **photosynthesis**. They contain large amounts of a green pigment called chlorophyll (Figure 2.2.14). Like mitochondria, chloroplasts also possess a double-stranded DNA molecule. Chloroplasts are present in **plants and many protists**, but never in **animals or fungi**.

Chloroplasts are composed of a system of three membranes: the outer membrane, the inner membrane and the thylakoid system. Thylakoids are disc-shaped sacs that form compartments within the chloroplast. Different compartments contain different enzymes.

Chloroplasts trap light energy, which is used to split water molecules into hydrogen and oxygen in the process of **photosynthesis**. The hydrogen then combines with carbon dioxide to make glucose, and the oxygen is released into the atmosphere as a waste product.

Storage and cell structure

The organelles involved in storage and supporting the cell structure in eukaryotic cells are vacuoles, plastids, the cell wall, the cytoskeleton, centrioles, cilia and flagella.

Vacuoles

A vacuole is a membrane-bound, liquid-filled space that stores enzymes and other organic and inorganic molecules. Vacuoles occur in most **cells**, but the number varies. They also differ between animal cells and plant cells (Figure 2.2.17). Animal cells contain many small, temporary vacuoles, but most plant cells contain a single, large, permanent vacuole surrounded by a membrane called the **tonoplast**. Plant vacuoles provide structural support by helping to maintain turgor. They also function in a similar way to the lysosomes found in animal cells.

Plastids

Plastids are organelles involved in the synthesis and storage of different chemical compounds. They contain a double-stranded DNA molecule and are surrounded by a **double membrane**. Plastids develop from simple organelles called proplastids. Animal cells do not contain plastids. Plastids can be:

chloroplasts, which are involved in **photosynthesis** and are found only in plants and some protists leucoplasts, which are involved in storage

-amyloplasts are a type of leucoplast found in plants (Figure 2.2.18)

-they are commonly responsible for synthesising and storing starch, but can also convert the starch back to sugar when the plant requires energy chromoplasts, which contain colour pigments and occur in **petals** and fruit.

Cell wall

The cell wall is a rigid structure that surrounds the cell membrane of plant cells, fungal cells and some prokaryote cells (Figure 2.2.19). In plants, the cell wall is composed mainly of cellulose. Fungal cell walls are made of chitin.

The cell wall provides support, prevents expansion of the cell, and allows water and dissolved substances to pass freely through it. Lignin in the cell walls of woody plants, especially in the xylem, gives them additional strength.

Cytoskeleton

The cytoskeleton consists of microtubules of a protein called tubulin, and filaments of a protein called actin (Figure 2.2.20). The cytoskeleton supports the cell's structure, allows the cell to move, and helps transport organelles and vesicles within the **cell**.

Centrioles

Centrioles are a pair of small, cylindrical structures composed of microtubules (figure 2.2.21). They are present in most eukaryotic cells, but many plant cells do not have them. Centrioles are involved in cell division and the formation of cell structures such as cilia and flagella.

Cilia and flagella

Cilia and flagella (singular cilium and flagellum) are hair-like structures on the surface of cells (Figures 2.2.22 and 2.2.23). They consist of an arrangement of microtubules that are enclosed by an extension of the cell membrane. Both structures are involved in the movement of the cell or things around the cell. Cilia move with an oar-like motion, and are usually shorter and more numerous than flagella.

2.3 Cell membranes

The cell membrane encloses the contents of a cell and controls the movement of substances between the extracellular fluid outside the cell and the intracellular fluid (or cytosol) inside the cell (Figure 2.3.1). The cell membrane therefore helps maintain an environment within the cell that differs from the external environment. As well as controlling the transport of molecules into and out of the cell, the cell membrane is also involved in cell recognition and communication with other cells. In this section, you will learn about the composition and characteristics of the cell membrane, described using the **fluid mosaic model** of the cell membrane.

EXTRACELLULAR FLUID IN UNICELLULAR ORGANISMS

Cells exist in a watery environment of extracellular fluid. This can be a large amount of fluid, or just a thin surface layer. The composition of the extracellular fluid is critical to cell stability and function, because it provides them with the nutrients they need to survive.

For unicellular organisms, the extracellular fluid is simply the watery, external environment in which they live. Unicellular organisms can do little to control their environment, and may die if it changes significantly. However, some unicellular organisms, such as yeasts, can become dormant until environment conditions are favourable. Other unicellular organisms can move slowly to a place where the conditions suit their needs. For example, unicellular algae can move towards light, and some bacteria can detect chemicals, and then move towards nutrients or away from toxic substances.

EXTRACELLULAR FLUID IN MULTICELLULAR ORGANISMS

The cells of multicellular organisms are more protected from the external environment than the cells of unicellular organisms. Multicellular organisms have more control over the environment in which their cells exist, and are therefore less affected by changes in the external environment. Whether they live in water or on land, multicellular organisms have an outer layer that acts as a protective barrier (Figure 2.3.2). This outer layer creates an internal environment for the organism that differs from their external environment. In multicellular organisms, the environment of the cells is the extracellular fluid that surrounds them.

Most multicellular organisms can regulate the conditions of their internal environment very precisely. This allows multicellular organisms to provide the specific conditions needed by specialised cells and tissues. It also allows their cells to function more efficiently. Commonly regulated aspects of the internal environment are:

• temperature

• oxygen concentration

• carbon dioxide concentration

• pH (acidity or alkalinity)

• osmotic pressure ( concentrations of salts or ions)

• nitrogenous waste concentration

• glucose concentration.

The way cells interact with the extracellular fluid of the internal environment is regulated by the cell membrane.

CELL MEMBRANE COMPOSITION: THE FLUID MOSAIC MODEL

The **fluid mosaic model** describes the structure of the cell membrane. The model was first proposed by Jonathan Singer and Garth Nicholson in 1972, and is now widely accepted as the basic model of all biological membranes. According to this model, cell membranes consist of a bilayer (two layers) of phospholipid molecules. Other molecules, such as proteins, carbohydrates and cholesterol, are scattered throughout the **bilayer**.

The **fluid mosaic model** is a representation of our current knowledge of the cell membrane. It is modified and updated as developments in cellular techniques and technology allow more information to be gathered.

Cell membranes have the same basic structure in all organisms. The structure serves to separate the interior of the cell (the cytoplasm) from its external environment. Most membranes are also asymmetrical, meaning one layer has different properties from the other. For example, the pattern of proteins and carbohydrate molecules in the external surface is different from the pattern in its internal surface.

The composition and characteristics of the cell membrane are related to the cell's needs and function. The cell membrane performs many functions, such as transporting molecules into and out of the cell, cell recognition and communication with other cells (Figure 2.3.4).

Phospholipids

Phospholipid molecules have a hydrophilic (water-attracting) 'head' and two hydrophobic (water-repelling) 'tails' (Figure 2. 3. 5). The phospholipid bilayer of the cell membrane is called a bilayer because it has two layers of phospholipids. The hydrophilic heads form the outside and inside lining of the cell membrane, and the hydrophobic tails of the two layers of phospholipids meet in the middle. The hydrophilic head of a phospholipid is made up of a phosphate group and glycerol, while the two hydrophobic tails are made up of fatty acids. The tails can be made of saturated fatty acids or unsaturated fatty acids.

Because phospholipids have a hydrophilic head and two hydrophobic tails, they react to the presence of water (Figure 2.3.6). If the phospholipids are in contact with water on one side and oil on the other side, all the hydrophilic heads will be embedded in water and the tails will be embedded in the oil. However, if the phospholipids are in contact with water on both sides, all the hydrophilic heads will be embedded in water, and the hydrophobic tails will point towards each other, with oil sandwiched in between. This bilayer arrangement shelters the hydrophobic tails of the phospholipids from water, while exposing the hydrophilic heads to water.

The phospholipid nature of cell membranes makes them impermeable to water­soluble particles, ions and polar molecules. The movement of these molecules across the membrane is controlled by protein channels. The protein channels allow the cell to regulate the exchange of molecules with the exterior environment. This regulation is central to important processes that keep the cell alive, such as cell respiration, digestion and waste elimination. You will learn more about the movement of materials across cell membranes in Section 3 .1.

Cell membranes are fluid structures. This means that individual phospholipid molecules (and some proteins) are free to move about within the layers. However, they rarely cross from one side of the cell membrane to the other. The level of membrane fluidity depends on the percentage of unsaturated fatty acids in the phospholipid molecules-the greater the percentage, the more fluid the membrane.

Fluidity of the cell membrane

The molecules of the cell membrane are not fixed in place. Most of the phospholipids and some of the proteins can move about laterally, while some molecules can occasionally flip-flop transversely across the membrane (Figure 2.3.7). The rate at which the molecules move within a layer of the cell membrane varies. Proteins in the **membrane** can move sideways, but at a much slower rate than the phospholipids. The ability of the phospholipids and proteins to move gives the cell membrane its fluid nature.

The fluidity of the cell membrane is very important. It affects the permeability of the membrane to substances, and the capacity for proteins to move within the membrane to particular areas where they are required to carry out their function.

Factors that alter the fluidity of the cell membrane include: phospholipid composition and structure

temperature

the presence of cholesterol.

Temperature

As the temperature increases, the fluidity of cell membranes increases. This is because the phospholipids become less closely packed together and are able to move more freely.

As the temperature decreases, a cell membrane with a large proportion of saturated fatty acids may solidify at a certain point. This will not occur in a **cell membrane** with a large proportion of unsaturated fatty acids, because the kinks in the tails of these fatty acids prevent the phospholipids from becoming too closely packed.

Cholesterol

Membranes contain many fatty molecules, including cholesterol molecules, between the **phospholipid molecules** (Figure 2.3.8). The cholesterol found in **eukaryotic cell membranes** gives stability to the cell membrane without affecting its fluidity. It also reduces the permeability of the cell membrane to small, water-soluble molecules.

Proteins

Like phospholipid molecules, proteins in the **cell membrane** can move about to some extent. However, this movement may be limited to particular regions of the cell membrane.

Proteins that are a permanent part of the cell membrane are called integral proteins. Proteins that are a temporary part of the cell membrane are called peripheral proteins. Peripheral proteins bind to integral proteins, or penetrate into one surface of the cell membrane (Figure 2.3.8). When integral proteins span both phospholipid layers, they are also called transmembrane proteins. Transmembrane proteins are involved in several important cellular and intercellular activities (Figure 2.3.4).

Carbohydrates

Carbohydrates associated with cell membranes are usually linked to protruding proteins (forming glycoproteins) or to lipids (forming glycolipids) on the outer surface of the membrane (Figure 2.3.8). Carbohydrates play a role in recognition and adhesion between cells, and in the recognition of antibodies, hormones and viruses by cells.

2.4 Investigating cells

Cytology is the study of cells. Cytologists use a variety of tools and techniques to study cells, including several microscopy techniques. Modern microscopy techniques, including light and electron microscopy, have greatly advanced our understanding of the structure and function of cells.

CELL SIZE

Cells vary greatly in size (Figure 2.4.1). Most cells are only visible under a **light microscope** and their size is usually measured in micrometres (µm) (note: 1000 µm = 1 mm). Although most cells are microscopic, there are some exceptions. For example, the egg cell of some bird species can be many centimetres in diameter.

Some typical cell lengths are:

• bacterium: 0.1-1.Sµm

• human cell: 8-60 µm

• plant cell: 10-100 µm

• paramecium (a single-celled eukaryote): about 150 µm.

The thickness of cell membranes also differs between cells, and can be between 0.004 and 0.1 µm thick.

LIGHT MICROSCOPY

Most cells are so small that they can only be seen with a **microscope**. The **light microscope** uses light and a system of lenses to magnify the image. One lens is called the objective lens, and the other is the eyepiece or ocular lens. The total magnification of a microscope is calculated by multiplying the magnifying power of the ocular lens (eyepiece) by the magnifying power of the objective lens. For example, an ocular lens with a magnifying power of 4 times (x4) used with an objective lens with a magnifying power of 10 times (xlO) gives a total magnification of 40 times (x40).

One of the main advantages of light microscopy is that it can be used to view living cells in colour.

Preparation time is usually quick and simple, and coloured stains can highlight different components of cells. To view cells through a **light microscope**, the cells first need to be prepared and mounted on a glass slide. Different techniques for preparing and mounting cells are used for different specimen types. Some common specimen preparation techniques are:

•whole mounts, which involve placing the whole organism or structure directly on the slide, and are used for thin structures or very small organisms such as Daphnia (Figure 2.4.4)

•smears, which are used for cells suspended in fluid (e.g. blood), or cells that have been scraped from a surface, such as cervical cells collected during a Pap smear (Figure 2.4.5)

•sections, which are very thin slices of specimens prepared by embedding the specimen in paraffin wax, and using a slicing instrument called a microtome to cut sections of just one layer of cells (3-7 µm) (e.g. liver tissue) (Figure 2.4.6).

After preparing, mounting and staining a specimen, the glass slide is placed on the stage of the microscope, under the lenses. Light travels through the specimen and into the lens system. The image is then viewed by eye or with a digital camera.

The condenser lens beneath the movable stage is used to concentrate light from the light source onto the specimen, and the image is focused using the coarse and fine adjusters. Different parts of the specimen can be viewed by moving the specimen on the stage.

Light microscopy techniques used in cytology include histology, autoradiography, fluorescence and confocal microscopy. Each of these uses visible light to examine cells and tissues.

fluorescence microscopy

A **fluorescence microscope** is used to examine cells, cellular structures or any fluorescing material, such as stains, dyes or antibodies, with fluorescent molecules. fluorescent cells contain molecules that absorb light at a particular wavelength ( called the exciting wavelength) and emit light at another wavelength. Filters are used to block out the exciting wavelength, allowing the light emitted by the fluorescing molecules to be seen against a black background (Figure 2.4.8). If the cells do not contain fluorescent molecules, fluorescent dyes (called markers) can be added that attach to different cellular structures, such as DNA or cell wall components (Figure 2.4.8). Fluorescence techniques allow scientists to visualise structures and materials inside cells that are usually too small to view. They can also target and detect particular proteins, and diagnose disease.

Confocal microscopy

A **confocal microscope** allows scientists to obtain 'optical sections' of a cell or tissue, stained with fluorescent markers, without actually sectioning or slicing the cells.

Confocal microscopy can obtain high-resolution (high-quality) images of very thin sections of a specimen (Figure 2.4.9). It involves passing laser light through a pinhole and lens, which provides highly focused light onto only a tiny part of the specimen. This eliminates light reflecting from adjacent parts of the section, which normally blur the image. Slowly scanning the object in this way, together with a suitable computer, allows you to view an 'optical section' of the sample.

Thicker samples can be imaged in thin sections and then reconstructed in three dimensions using image analysis software. At present, **confocal microscopes** and the computer software required are very expensive, and the images take a long time to make. However, **confocal microscopes** can produce remarkable three-dimensional views of living structures.

ELECTRON MICROSCOPY

An **electron microscope** uses an electron beam rather than light to view objects. This allows us to see structures in far more detail than is possible using light microscopy (Figure 2.4.10). An **electron microscope** produces a narrow beam of electrons. The beam is maintained by electromagnetic lenses, which are coils that surround the tube and emit an electromagnetic field. Electrons striking the specimen are either absorbed, scattered, or pass through it.

The image obtained with an **electron microscope** has a much higher resolution and a greater depth of field than an image from a light microscope. In microscopy, the depth of field is the range of depth that a specimen is in acceptable focus; e.g. if a **microscope** has a shallow depth of field, it will be difficult to focus clearly on a thick specimen. Electron microscopy produces only black and white images, but these are often coloured later to highlight important features.

Transmission electron microscopy

In **transmission electron microscopy (TEM)** the electron beam travels through an ultra thin section (less than 100 nm thick) of a specimen. This allows very fine details of cellular structures to be seen (Figure 2.4.11).

Because the specimen must be in a vacuum in the TEM, it is first chemically fixed to stop the structures from collapsing, and then dehydrated with alcohol. It is then embedded in a plastic resin, sectioned with a diamond cutter called an ultramicrotome, and stained.

Scanning electron microscopy

In **scanning electron microscopy (SEM)** the electrons are bounced off a specimen that has been coated with an extremely thin layer of gold. This gives a high-resolution picture of the surface features, but cannot show internal details (Figure 2.4.12).

AUTORADIOGRAPHY

**Autoradiography** is a method that allows scientists to identify specific organelles or the location of molecules within a cell or tissue. In autoradiography, the tissue is first treated with a radioactively labelled substance. The substance is taken up into the part of the cell that is being investigated (Figure 2.4.13). The tissue is then sliced into very thin sections, which are then placed against a very thin, high-resolution photographic film. The radioactive substance emits beta particles, which produce an image on the film. The tissue sections are then stained to locate the cellular structures seen in the photographic image. This technique can be used to reveal the activity levels of organelles in different situations.

Although autoradiography is still sometimes used with light microscopy, it is more commonly used today with electron microscopy.

3.1 Movement of materials in and out of cells

In Section 2.3, you learnt about the composition of the cell membrane, and that one of its main functions is to control the transport of molecules between the cell's internal environment (the cytosol) and the external environment. The way in which materials cross the cell membrane depends on the:

characteristics of the materials being exchanged

permeability of the cell membrane to the material (Table 3 .1.1)

surface-area-to-volume ratio of the membrane

concentration gradient between the internal and external environments of the cell.

In this section, you will learn about the semipermeability of the cell membrane.

You will also explore the various methods cells use to control the exchange of molecules, including diffusion, osmosis, active transport, facilitated diffusion, endocytosis and exocytosis.

CELL MEMBRANE PERMEABILITY

The permeability of a cell membrane refers to its ability to allow the cell to exchange liquids and materials between the cell's internal environment and the external environment. The movement of materials in and out of a cell is critical to its function and survival, allowing essential materials to enter while keeping waste materials out. It also allows cells to communicate with other cells.

The cell membrane is selective about the materials that it allows in and out of the cell. This characteristic is known as semipermeability. Many different types of molecules can move across **cell membranes**(Figure 3 .1.1). They do so in different ways depending on their characteristics, such as size and charge, and the permeability of the cell membrane to the material (Table 3 .1.1).

Because of their lipid nature, cell membranes are permeable to small molecules and lipid-soluble molecules that can move freely through the phospholipid bilayer. However, their lipid nature also makes cell membranes impermeable to:

most water-soluble molecules

ions (atoms or groups of atoms with an overall positive or negative charge) polar molecules (molecules with charged regions but no overall charge). These substances must therefore pass through specific protein channels in the cell membrane.

DIFFUSION

Particles in a **solution** move from an area of high concentration to an area of low concentration. This process is called diffusion (Figure 3.1.3). Because many particles collide with each other during this process, the overall movement of particles is very slow.

Diffusion can be seen when a drop of ink (the solute) is placed in a jar of still water (the solvent). The dye particles in the ink move randomly through the water until the colour is homogenous (evenly spread). In other words, the solute particles move from an area of high solute concentration (the drop of ink) to the areas of low solute concentration (the rest of the jar). The solute particles are said to have moved along the concentration gradient.

Diffusion is called a passive process, because it does not require energy. It occurs only because there is a concentration gradient.

DIFFUSION ACROSS MEMBRANES

The two types of diffusion across membranes are simple diffusion and facilitated diffusion. These are both passive types of diffusion, and both move molecules along the concentration gradient.

Simple diffusion

Solute molecules can only diffuse across a membrane if that membrane is permeable to them. There is a constant movement of solute molecules backwards and forwards across the membrane.

If the concentration of solute molecules is the same on both sides of the membrane, there will always be about the same number moving across in either direction.

That is, there will be no net movement from one side to the other. However, if the concentration of the solute molecule is higher on one side of the membrane than the other, more molecules will cross from the area of higher concentration to the area of lower concentration (i.e. down its concentration gradient).

If the membrane is semipermeable-that is, it is impermeable to some molecules-there will be no movement of those molecules from the area of higher concentration to the area of lower concentration.

Factors affecting rate of diffusion

Three main factors affect the rate of diffusion across a membrane: Concentration-the greater the difference in concentration gradient, the faster the rate of diffusion. When the concentration is equal on both sides of the membrane, the net diffusion is zero, even at high temperatures.

Temperature-the higher the temperature, the higher the rate of diffusion. Increasing temperature increases the speed at which molecules move.

Particle size-the smaller the particles, the faster the rate of diffusion.

Facilitated diffusion

The phospholipid bilayer of the membrane is impermeable to certain particles (ions or molecules). However, channel proteins in the **membrane** allow for the movement of these particles. When movement is down the concentration gradient, the process is called facilitated diffusion.

In facilitated diffusion:

The membrane transport proteins are specific for particular particles, so transport is selective; some particles are transported and others are not. Transport is more rapid than by simple diffusion.

The transport proteins can become saturated (fully occupied) as the concentration of the transported substances increases.

The transport of one particle may be inhibited by the presence of another particle that uses the same transport protein.

No energy is required; the particles move down their own concentration gradient. The two main types of membrane transport proteins involved in facilitated diffusion are channel proteins and carrier proteins. Membrane proteins provide channels for the passage of water-soluble (polar) molecules and ions across the phospholipid bilayer. The channel proteins are specific for a substance. Channel proteins do not usually bind with the molecules being transported. They function like pores that open and close to allow the passage of specific molecules. Channel proteins are mainly involved in the passage of water-soluble polar particles, such as ions (e.g. Na+, K+ and CJ-).

Carrier proteins bind the molecules being transported. This causes the protein 10 undergo changes in shape (or conformation) that allow specific molecules to be transported across the membrane. After the molecule has crossed the membrane, the original shape of the protein is restored.

OSMOSIS

Osmosis refers to the net diffusion of water molecules across a semipermeable membrane.

If a diluted and a concentrated solution are separated by a semipermeable membrane that allows the movement of free water molecules across the membrane, but not the movement of the solute molecules, the free water molecules will move across the membrane from the diluted to the concentrated solution.

In osmosis, net diffusion of water occurs through a semipermeable membrane from a diluted to a concentrated solution along its own concentration gradient. This is known as the osmotic gradient (Figure 3.1.6). The pressure causing the water to move along this gradient is called osmotic pressure.

The cell membrane is permeable to water. Therefore, when cells are placed in freshwater, an osmotic gradient will draw water into the cells. This is because the cytosol is a concentrated solution containing many dissolved substances.

For example, if red blood cells are placed in freshwater, the cells absorb so much water by osmosis that they swell and may eventually burst, releasing red pigment into the water. Conversely, if red blood cells are placed in a **solution** that is more concentrated than their cytosol, water leaves the red blood cells by osmosis, causing them to shrink.

If a plant cell absorbs water, it swells to some extent, but the cell wall prevents the cell from bursting (Figure 3 .1. 7). Water will continue to enter the cell along an osmotic gradient until the internal fluid pressure equals the osmotic pressure drawing water in, at which point no more water will enter. Plant cells with high internal fluid pressures are said to have a high turgor, or be turgid.

In osmosis, we always compare solute concentration between two solutions. The terms isotonic, hypertonic and hypotonic are often used to describe the differences between solutions.

Isotonic solutions-the solutions being compared have equal concentration of solutes.

Hypertonic solution-the solution with a higher concentration of solute (hence lower concentration of free water molecules).

Hypotonic solution-the solution with a lower concentration of solute (hence higher concentration of free water molecules).

Osmosis in salty environments

There is no biological mechanism for actively transporting water molecules across cell membranes. The net movement of water across membranes occurs only by osmosis. Some bacteria, known as halophiles, are adapted to extremely salty environments, such as the pink salt lakes in western New South Wales (Figure 3.1.8). They survive by retaining much higher ion concentrations within their cells. They also produce small, osmotically active but otherwise inert molecules to reduce the osmotic gradient. This prevents the loss of water to their salty surroundings. Their proteins are also specialised to function normally, despite the high concentration of salts in the cytosol.

ACTIVE TRANSPORT

Diffusion, facilitated diffusion and osmosis are examples of passive transport, because they do not require energy to move particles across the cell membrane. Active transport involves the cell using energy to transport particles across membranes (Figure 3.1.9).

Active transport and facilitated diffusion compared

Active transport has the same properties of selectivity, saturation and competitive inhibition as facilitated diffusion, because it also occurs through transport proteins (Figure 3.1.10).

•Selectivity means that some substances are transported but others are not.

•Saturation means that there is no increase in the rate of transfer when all transport proteins are open.

•Competitive inhibition means that one substance can inhibit the transport of another substance by using the same transport protein.

However, unlike facilitated diffusion, which can occur through either channel or carrier proteins, active transport only occurs through carrier proteins. Because active transport uses energy, it can move substances against a concentration gradient (from low concentrations to high concentrations). In comparison, facilitated diffusion uses no energy, so it can only move substances down a concentration gradient.

In different situations, either facilitated diffusion or active transport may be used to transport a particular molecule. Whether a cell uses facilitated diffusion or active transport depends on the specific needs of the cell.

For example, glucose is actively transported from the gut into epithelial cells lining the gut, so it can enter the bloodstream. The regulation of this process is controlled by hormones, principally insulin and glucagon. If gut glucose levels are high, blood glucose levels will increase. If gut glucose levels are low, active transport makes sure that the little glucose that is in the **gut** gets pumped into the **epithelium**. From the epithelium, the glucose can move into the blood via facilitated diffusion.

In contrast, red blood cells move glucose by facilitated diffusion. This makes sense, because glucose concentration in the blood is usually maintained within a narrow range. In addition, cells convert glucose into other chemicals as soon as it enters the cell. This keeps the intracellular concentration of glucose lower than the blood concentration of glucose, meaning that the concentration gradient is maintained in favour of glucose entering the cell.

ENDOCYTOSIS AND EXOCYTOSIS

Some large molecules and other particles and fluids are moved into or out of the **cell** by endocytosis and exocytosis (Figure 3 .1.11). These are both forms of active transport, because they require energy.

Endocytosis

In endocytosis, the cell takes in materials in bulk by forming new vesicles from the cell membrane. During endocytosis, a small area of the cell membrane sinks inwards to form a pocket. As the pocket deepens, materials near the cell membrane are enclosed by the **membrane**, which then pinches off to form a vesicle. The vesicle then transports the substance to where it is required within the cell.

There are three types of endocytosis (Figure 3 .1.12):

In phagocytosis, a cell engulfs a solid material by wrapping temporary cytoplasmic extensions called pseudopodia (meaning 'false feet') around it. This forms a membrane-bound structure known as a phagosome (Figure 3.1.13). The material will be digested when the food vacuole fuses with a lysosome that contains enzymes.

In pinocytosis, the cell membrane engulfs liquid that contains dissolved molecules (Figure 3.1.14).

Receptor-mediated endocytosis is a type of pinocytosis that engulfs specific substances. Protein receptors located on the surface of the cell membrane respond to particular molecules, binding to the molecule and triggering the engulfment of the substance into the cell.

Exocytosis

When a secretory vesicle membrane and the cell membrane come into contact, specific proteins alter the arrangement of the phospholipids in the **phospholipid bilayer**. The fluid and dynamic nature of the cell membrane then enables the two membranes to fuse. Once the membranes have fused, the contents of the secretory vesicle are released out of the cell. This is called exocytosis.

The vesicle membrane becomes a permanent part of the cell membrane (Figure 3 .1.15). The cell membrane is continually recycled as vesicles fuse during exocytosis, and are conversely formed and released during endocytosis.

In addition to being used for secreting proteins, exocytosis is also involved in the release of cellular waste and the breakdown products from lysosomes. For example, phagocytic cells (such as unicellular protists or macrophages in multicellular organisms) engulf food or foreign matter and digest them with the aid of lysosomes. Afterwards, the waste products of this digestion are released by exocytosis.

A summary of the roles of the rough endoplasmic reticulum and Golgi apparatus in the exocytosis of proteins can be seen in Figure 3 .1.11.

SURFACE-AREA-TO-VOLUME RATIO

All cells must exchange nutrients and wastes with their environment via the cell membrane. In addition, enzymes that are bound to the cell membrane catalyse many important cellular processes. The surface area of the cell membrane around a cell affects the rate of exchange that is possible between the cell and its environment, and can affect certain processes catalysed by membrane-bound enzymes.

Larger cells have greater metabolic needs, so they need to exchange more nutrients and waste with their environment. However, as the size of a cell increases, the surface-area-to-volume ratio of the cell decreases.

Because of this surface-area-to-volume relationship, larger cells do not have a proportionally larger surface area of cell membrane for the efficient exchange of nutrients and waste. Smaller cells can exchange matter with their environment more efficiently.

Increasing the cell surface-area-to-volume ratio

Three ways of increasing the membrane surface area of cells without changing cell volume are:

• cell compartmentalisation

• a flattened shape

• cell membrane extensions.

Cell compartmentalisation

In Section 2.2, you learnt that cell compartmentalisation allows organelles to have the right conditions and concentration of enzymes and reactants for a particular function, making the processes in the organelles-and in turn, the whole cell­highly efficient.

Cell compartmentalisation also allows eukaryotic cells to be much bigger than prokaryotic cells, because it:

• reduces the amount of exchange that needs to occur across the cell membrane to maintain an environment suitable for all cell functions

• creates more space for membrane-bound enzymes, allowing increased activity in the **cell**.

A flattened shape

As a cell increases in volume, the distance from the centre of the cell to the cell membrane also increases. The rate of chemical exchange (or rate of diffusion) from the centre of the cell to the surrounding environment may then become too low to maintain the cell.

One way to counteract this effect is to be flatter. For example: flattening a cube while keeping the volume constant results in a larger surface area, and therefore a larger surface-area-to-volume ratio. This larger surface-area-to-volume ratio allows a higher rate of exchange through the cell membrane. It also reduces the distance that substances need to be transported to and from the cell membrane.

The flattened-shape solution is observed in nature in many types of cells­especially those involved in the rapid transport of substances, such as red blood cells and lung epithelium. These cell types do not usually have a high metabolism and so do not contain many organelles.

Cell membrane extensions

Instead of being larger or flatter, cells involved in absorbing nutrients or secreting wastes counteract the surface-area-to-volume ratio problem by extending the surface area of their cell membranes. For example, some animal cells have finger­like extensions of the cell membrane called microvilli (singular microvillus), which increase the surface area (Figure 3 .1.19). Another example is root hairs in plants, which are lateral extensions of root cells. The hairs increase the surface area of the root, allowing the plant to absorb more water and nutrients from the soil.

A flattened shape would not be useful for cells involved in absorbing nutrients or secreting wastes, because they require an increased surface area in particular regions of the cell. For example, in cells of the small intestine, an increased surface for exchange is only required on the inside of the intestinal tube, where the cells absorb nutrients. In addition, these cells have a high metabolism and possess many organelles. If they were flattened, the distance between the different organelles of the cell would affect the movement of substances within the cell and reduce its functionality.

3.2 Cell requirements

There is an enormous diversity of living organisms on Earth, but many of their processes and requirements for life are the same. Whether organisms are unicellular or multicellular-and whether they live at the bottom of the ocean or in a rainforest­they all need to take in nutrients and water, exchange gases, obtain energy and remove waste products.

All life on Earth needs a carbon source and other atoms (e.g. oxygen) to build organic compounds and an energy source to grow and add mass. Ultimately, most biological systems and organisms rely primarily on one source of energy for their survival: sunlight (Figure 3.2.1). Organisms can be divided into groups according to how they obtain organic compounds and how they obtain energy.

CELL REQUIREMENTS-ENERGY

Organisms can be divided into two groups depending on the strategies they use to obtain organic compounds, which are in turn their source of energy.

Autotrophs (self-feeders) make their own organic compounds from inorganic compounds found in the soil and atmosphere. The conversion of inorganic compounds into organic compounds is called carbon fixation because the autotroph 'fixes' inorganic carbon into organic molecules, such as glucose. Because autotrophs produce their own nutrients and all of the organic compounds in ecosystems, they are also called producers. Autotrophs include all of the green plants that carry out **photosynthesis**.

Heterotrophs (other-feeders) obtain organic compounds by consuming other organisms (autotrophs or other heterotrophs). Because heterotrophs consume (eat) organic compounds, they are also called consumers. Heterotrophs include all animals and fungi.

Both autotrophs and heterotrophs use matter (organic and inorganic compounds) to produce the energy required for all biological processes. **Photosynthesis and cellular respiration** are the reactions that cells use to transform matter into energy (Figure 3.2.2). Autotrophs use both **photosynthesis and cellular respiration**, while heterotrophs use only **cellular respiration**. The biochemical processes of photosynthesis and cellular respiration are examined in more detail in Section 3.3.

CELL REQUIREMENTS-MATTER

There are 92 different types of naturally occurring atoms on Earth, and each type is known as an element. The same elements that can be found in rocks, soil and air are also found in living cells. But there is a difference in the way that these atoms are organised into larger compounds in living organisms.

Organisms produce characteristic complex compounds that contain carbon and hydrogen (Figure 3.2.3). These are called organic compounds because the first ones discovered were produced by organisms or found in them. Most large organic molecules are composed of many smaller organic molecules linked together. Scientists sometimes refer to this system as a polymer made from monomers. For example, protein molecules are constructed from chains of amino acids, and DNA is made up of a series of nucleotide subunits.

All other compounds, whether in living or non-living things, are called inorganic compounds. Inorganic compounds that are important for living organisms include water, oxygen, carbon dioxide, nitrogen and minerals.

Chemical compounds required by living cells include inorganic compounds, organic compounds and structural organic molecules.

Inorganic compounds

Inorganic compounds include water, oxygen, carbon dioxide, nitrogen and minerals.

Water (H20) makes up 70-90% of most organisms. It is an important solvent and transport medium. Chemical reactions in cells take place in water-based fluid (cytosol), and some reactions include water.

Oxygen (02) is needed for efficient energy supply, achieved by the process of **cellular respiration** in almost all organisms. It is taken in as a gas by terrestrial organisms and in solution by aquatic organisms.

Carbon dioxide (CO2) is the ultimate source of the vital carbon atoms for organic molecules, usually starting with carbon fixation by **photosynthesis** in autotrophs (green plant cells). Carbon dioxide is taken into plant leaves as a gas, converted to sugars and eventually returns to the atmosphere in the **carbon cycle**. Nitrogen (N) is a key atom of the 20 types of amino acids that link together to form protein molecules and of the nucleotide subunits that form nucleic acids. Consumers derive their nitrogen atoms from plants, which obtain soluble nitrate ions (N03) that are added to the soil by nitrogen-fixing bacteria using N2 gas from the air. The **nitrogen cycle** eventually returns N 2 gas to the air. Minerals are important for building many enzymes and vitamins that are needed for the structure and function of biological systems. Calcium is used for the structural parts of vertebrates, such as bones and teeth. Sodium and potassium are important for nervous system function. Magnesium is important for muscle function, and iron is needed for haemoglobin production in red blood cells. Humans require more than 20 minerals, some in only minute quantities (trace elements).

Organic compounds

Organic compounds include carbohydrates, lipids, proteins and nucleic acids.

• Carbohydrates are important energy sources and structural components of organisms. Some important carbohydrates are glucose, sucrose, starch and cellulose. The basic subunits of carbohydrates are simple sugars called monosaccharides (single sugars), disaccharides (two sugars joined together), and polysaccharides (many sugars joined together in long chains) (Figure 3.2.4a). Carbohydrates have hydrogen atoms in a 2: 1 ratio with oxygen atoms.

• Lipids play an important role in **cell membranes**. They include fats and oils, which are important for energy storage (Figure 3.2.4b). Lipids are composed of a glycerol 'head' and fatty acid 'tails'. Compared with carbohydrates, lipids contain a much smaller proportion of oxygen, and can contain other elements, such as phosphorus and nitrogen. The role of lipids in cell membranes is covered in detail in Chapter 2.

Proteins are composed of amino acids and have many functions. Some are enzymes, while others are hormones, antibodies or carrier molecules (e.g. haemoglobin). Proteins form part of the cell membrane (Figure 3.2.4c). Organisms have their own unique proteins, unlike the more generic lipids and carbohydrates found in all **organisms**. As well as carbon, hydrogen and oxygen atoms, proteins always have nitrogen. Their diversity comes from the 20 possible amino acid subunits, linked by peptide bonds into polypeptide chains, two or more of which are folded into a complex 3D-shaped structure (called the tertiary structure). Proteins are constructed in each cell under coded directions from DNA (genetic information).

Nucleic acids carry the genetic information of cells. The two types of nucleic acids are DNA and RNA, both of which are made of long chains of nucleotides (adenine, guanine, cytosine and thymine in DNA; adenine, guanine, cytosine and uracil in RNA), sugars and phosphates (Figure 3.2.4d). DNA carries the information needed to assemble proteins from amino acid subunits. Genetic information is passed from cell to cell during cell division. RNA plays a major role in the manufacture of proteins within cells.

Structural organic molecules

Structural organic molecules and vitamins are small, organic molecules that are vital for normal cell function (Figure 3.2.5).

Some organic molecules can be synthesised, but others must be obtained in the diet. For example, most mammals can synthesise vitamin C, but humans must obtain it in their diet. Vitamins may be water soluble (such as vitamins B and C) or lipid soluble (such as vitamins A, D, E and K).

Water-soluble vitamins must be consumed regularly in the diet because they cannot be stored in body tissues. Lipid-soluble vitamins can be stored. Many vitamins are needed to work with enzymes.

CELL REQUIREMENTS-WASTE REMOVAL

Waste material for one organism may be a useful substance for another. The undigested food (faeces) removed from a vertebrate are useful nutrients for many decomposing bacteria, fungi and protozoa. Operating under strict hygiene conditions, Sydney Water and other wastewater facilities around Australia produce biosolids from activated sludge from sewage. This is the nutrient-rich material created from treating wastewater solids with suitable microorganisms. Biosolids are a rich source of phosphorus and nitrogen and are used in agriculture, horticulture and mining.

The waste products from our digestive system may be the most obvious waste that we think of. However, they are not actually the waste products of cells. They have passed through the digestive tract without actually becoming part of the body-unlike digested food, which has passed into the bloodstream.

As both autotrophic and heterotrophic cells function, they produce substances from their metabolism that are no longer useful to them. Accumulation of these waste substances, such as carbon dioxide from **cellular respiration** and nitrogenous wastes from the breakdown of proteins and nucleic acids (DNA and RNA), can prevent cells from functioning properly. The waste substances need to be removed to ensure balance is maintained in the organism. This process is known as excretion.

An important property of the cell membrane is to regulate the transport of materials-including any waste material. Depending on the size and concentration of the waste material, transport may be passive or active:

•passive e.g. osmosis of water molecules; simple diffusion of oxygen, carbon dioxide, ammonia and alcohol; facilitated diffusion of urea, glucose and ions

•active e.g. urea, toxins and ions being removed against their concentration gradient via endocytosis and exocytosis.

The function of the cell membrane in controlling the movement of materials in and out of the cell is explained in more detail in Section 3 .1.

Waste removal from autotrophs

Autotrophs produce and excrete a variety of waste products, but these are usually considered by-products rather than waste, and elimination may be in subtle ways. Plants (autotrophs) do not require specialised excretory organs, although leaves could be regarded as excreting any carbon dioxide or oxygen gas that is excess to the leaf cell requirements. Compared to heterotrophs, the autotrophic cells produce almost no true waste. This is because they have a much lower metabolic rate, can reuse gases such as oxygen and carbon dioxide, and contain fewer protein molecules, thereby producing less nitrogenous waste.

Examples of waste removal in plants include the following.

For aquatic autotrophs, such as all the algae, e.g. the seaweed Ulva (sea lettuce), waste chemicals are passed directly into the surrounding water.

Some species live in environments where they need to deal with unusual waste problems. One example is the mangrove, which is a tree that lives in the intertidal zone of estuaries (area where saltwater from the ocean meets freshwater from a river). Mangroves are periodically subjected to high levels of salt around their roots. Mangrove adaptations include concentrating excess salt in old leaves and bark, which are then shed; storing salt within **enclosed cell vacuoles**; and excreting salt crystals onto the leaf surface where they are blown or washed away.

Some terrestrial (land) plants store wastes in non-living hardwood, or in leaves and bark that are later dropped, such as deciduous trees in autumn, or eucalyptus trees with continuous leaf drop (Figure 3.2.6).

Waste removal from heterotrophs

Normal metabolic activity for animal (heterotroph) cells is more complex than in autotrophic cells. Heterotrophic cells break down and replace carbohydrates, lipids, nucleic acids (DNA and RNA) and proteins, producing waste products that usually cannot be used by the body.

In unicellular heterotrophs, waste products can be released directly into the surrounding environment. The unicellular organisms Paramecium and Amoeba are heterotrophic organisms that are common in freshwater (Figure 3.2.7). Their small size means they can expel wastes, including carbon dioxide gas, through their cell membrane directly into the surrounding water. These organisms have structures such as contractile vacuoles, which collect water and wastes internally and periodically pump them out.

More complex heterotrophs require more complex excretory structures and processes. Unlike in plants, the excretory structures of animals are obvious. Animals have specific excretory organs and systems to ensure that wastes are efficiently removed from cells.

Carbon dioxide

When carbohydrates or lipids are broken down during **cellular respiration** to release energy, carbon dioxide and water are produced. These are removed into the surrounding environment by first diffusing across moist respiratory membranes­which in mammals, are in the lungs-and are then breathed out. For aquatic animals, the removal of carbon dioxide may occur by diffusion or via a specialised organ, such as gills. Animal groups that use gills include fish, crustaceans (e.g. crabs, barnacles and krill), some echinoderms (sea stars), aquatic molluscs (shell fish), some amphibians and the **larval stage** of some insects. Ancient fossils of trilobites show the imprint of gill structures between their many legs.

An efficient removal process is particularly important for carbon dioxide. If it is allowed to build up, it lowers the pH and increases tl1e acidity of the blood and extracellular fluid.

Water

Water is not usually a waste problem. For terrestrial organisms, water is more likely to be in shortage. If extra water is produced during **cellular respiration** or by ingestion, it would first be incorporated into normal body fluids. Any excess water is then expelled from the body as water vapour when breathing (Figure 3.2.10) or with urine from the kidneys as part of the routine removal of nitrogenous waste. Urine can be produced that is more or less diluted as part of the body's homeostatic regulatory process. Freshwater animals do have to remove excess water, which is drawn in by osmosis. Paramecium (Figure 3.2.7) is an example of an organism that needs to do this.

Nitrogenous wastes

Proteins are made up of amino acid molecules, which all contain nitrogen atoms (N) as part of their structure. When proteins are broken down, the nitrogenous parts are split off and the remainder of the molecule is converted into carbohydrates or lipids, which can be used for energy. The remaining nitrogenous waste must be removed from the cell, because it can become toxic. Cells also break down and recycle nucleic acids (DNA and RNA), releasing yet more nitrogenous waste. Multicellular animals use their circulatory systems to transport wastes away from cells to the excretory organs.

In mammals, nitrogenous waste is first managed by the liver, and then regulated by the kidneys and excreted as urea in the **urine**. Specialised structures called nephrons in the **kidneys** regulate the concentration of water and soluble substances in the body by filtering the blood (Figure 3.2.11). Close interactions between blood vessels and each nephron ensure that the blood is continuously filtered to remove urea and form urine, yet retains useful substances. During the filtration process, other toxins are also removed from the blood and the salt-water balance of the body is maintained.

The biochemical management of nitrogenous waste will be explained in more detail in Section 3.3.

3.3 Biochemical processes in cells

Living things can be divided into autotrophs-those that can produce their own organic materials-and heterotrophs, which must consume the organic materials produced by others. The cells of both types of organism use the biochemical process of **cellular respiration** to obtain energy from glucose to carry out cellular activities (Figure 3.3.1). Only in autotrophs do cells use solar energy to power part of the biochemical pathway, known as **photosynthesis**, and produce their own organic material. During metabolic processes like these, cells produce some waste or excess substances that need to be removed to maintain a stable internal environment.

PHOTOSYNTHESIS IN EUKARYOTIC CELLS

Plants produce glucose by a biochemical process called **photosynthesis**. **Photosynthesis** is the process in which plants and other photoautotrophic organisms obtain energy from sunlight to make their own organic compounds. This section will focus on photosynthesis in eukaryotic cells.

Simple experiments show that when plants have light, water (H20) and carbon dioxide (CO2), they make glucose (C6H1206) in their green **tissues**, usually the leaves (Figure 3.3.2). They trap the energy from sunlight and convert it into chemical energy, which they store in the bonds of **glucose molecules**. This enzyme­controlled process is **photosynthesis** ('photo' meaning 'light', 'synthesis' meaning 'putting together').

All photosynthetic organisms, from single-celled algae to the largest trees, produce glucose in the same way (Figure 3. 3 .1). The ability to carry out **photosynthesis** means that green plants are classed as autotrophs.

Chloroplasts

**Chloroplasts** are the sites of **photosynthesis** -where light energy is converted to food (glucose) for the plant and where oxygen is released. The function of these important organelles sustains almost all life on Earth. Chloroplasts are found in the cells of the leaves and green stems of vascular plants, and throughout the leaves of non­vascular plants, such as mosses. The leaves, and sometimes stems, of plants are green because of the green pigment chlorophyll that is inside **chloroplasts**. The chlorophyll molecules absorb energy from sunlight, the essential first stage of photosynthesis.

In vascular plants, chloroplasts are found in **mesophyll cells**, which make up the upper surface of leaves (Figure 3.3.4). The **mesophyll cells** are packed with chloroplasts and are the main sites of **photosynthesis**. The location of mesophyll cells near the upper surface of the leaf means that the chloroplasts have maximum exposure to sunlight, increasing the rate of **photosynthesis**.

In plants and algae, the chloroplasts are surrounded by a **double membrane**. Inside each **chloroplast** are stacks of flattened membranes called grana (singular granum) (Figures 3.3.4 and 3.3.5).

Photosynthesis takes place inside the **chloroplasts**, which are numerous in each grunter cell. The grana stacks contain the chlorophyll pigments where reactions using light energy occur during **photosynthesis**.

Plant chloroplasts are generally shaped like a biconvex lens about 5-8 µm in diameter. The outer membrane is highly permeable. The **inner membrane** is impermeable and surrounds the area filled with a liquid called stroma. Stroma contains a number of enzymes, spherical DNA and ribosomes. The grana hold stacks of small disc-shaped structures called thylakoids where chlorophyll is made (Figure 3.3.5).

Photosynthesis in non-green plants, protists and cyanobacteria

All plants (as well as many protists and cyanobacteria) contain chlorophyll, but not all are green. Some plants have yellow, red or purple leaves, and protists and cyano bacteria can be red, blue or other colours (Figure 3. 3. 6). These organisms do contain chlorophyll, but also have other pigments, including phycobiliproteins and carotenoids, which mask the green chlorophyll.

Phycobiliproteins and carotenoids assist in **photosynthesis** by absorbing different wavelengths of light. Phycobiliproteins are especially useful in deep water, where only green wavelengths can penetrate. Pigments other than chlorophyll also protect plants from overexposure to sunlight and attract pollinators. There are six known types of chlorophyll, with chlorophyll-a being the most prevalent.

Stages of photosynthesis

**Photosynthesis** is often simplified into an equation such as the one shown in Figure 3.3.7. In reality, it is a complex biochemical process in which solar energy is converted into chemical energy stored in the form of glucose, which can be used by all organisms. **Photosynthesis** occurs in a series of chemical reactions spread over two stages: the light-dependent reactions and the light-independent reactions. In Section 3.4 you will learn how the pathway is controlled by enzymes.

Stage 1: Light-dependent reactions

In the light-dependent reactions, chlorophyll captures solar energy and uses it to produce adenosine triphosphate (ATP). During this process, photolysis occurs, where water is split into hydrogen ions and oxygen gas. The light-dependent reactions occur on the **thylakoid membranes (grana)** of the chloroplast, where chlorophyll molecules are located.

Stage 2: Light-independent reactions

The light-independent reactions (also called dark reactions) produce glucose, water and adenosine diphosphate (ADP). These reactions are called light­independent because they do not require solar energy. ATP made during Stage 1, the light-dependent stage, provides the energy for the dark reactions. This energy is needed to combine carbon dioxide with hydrogen ions (also from the light­dependent stage) to form glucose-an energy-rich, organic molecule-and water. The light-independent reactions take place in the **stroma** (fluid part) of the chloroplast.

**Photosynthesis** is critically important for life on Earth. This is because carbon fixation and the production of plant matter is essential in providing energy and biomass in both aquatic and terrestrial ecosystems. Plants can produce all the organic compounds they need from the glucose they produce by **photosynthesis**, as long as they have the necessary minerals (Figure 3.3.8).

Controlling the rate of photosynthesis

Three main factors control the rate of photosynthesis:

• light intensity

• carbon dioxide concentration

• temperature.

The availability or concentrations of these factors may limit the rate of the reactions in the photosynthesis pathway. For each factor, there is an optimum amount at which photosynthetic reactions proceed at the fastest rate. Below the optimum level, reactions are slower. At levels above the optimum, reactions do not proceed any faster-and sometimes occur more slowly.

The factor that is present in the smallest amount is the limiting factor. Therefore, only one factor will be limiting at a particular time. For example, if a plant is in the process of **photosynthesis** and there is a large amount of carbon dioxide available but not enough light, then light is a limiting factor. And if there is enough light and not enough carbon dioxide, then the concentration of carbon dioxide is the limiting factor (Figure 3. 3 .11).

Light intensity

Light intensity can affect the rate of **photosynthesis**. When light intensity is low, the light-dependent reactions in the **grana of the chloroplasts** (the production of ATP and the splitting of water molecules by photolysis) cannot occur. So at night, light intensity is the limiting factor, because **photosynthesis** cannot occur.

As light intensity increases from this point, the rate of **photosynthesis** will increase. However, at a certain light intensity, the rate of **photosynthesis** will not be affected by further increases in light intensity. This effect can be seen in Figure 3.3.12.

Plants living in shaded environments have adaptations to enable them to photosynthesise under low light conditions. They have low respiration rates, fewer cells per leaf and lower concentrations of proteins compared with species that grow in sunnier places. In terms of energy requirements, they can be thought of as very cheap to run. Shade-tolerant plants also absorb light very effectively. Some plants that grow in dark environments have a lens-like arrangement in the upper epidermal cells that focuses light onto their photosynthetic cells below.

Carbon dioxide levels

Carbon dioxide availability affects the rate of photosynthesis because **photosynthesis** uses the carbon atoms from carbon dioxide to make glucose. When no carbon dioxide is available, **photosynthesis** cannot occur, so carbon dioxide is a limiting factor. As carbon dioxide becomes available, **photosynthesis** begins. As with light, the rate of photosynthesis will increase as carbon dioxide levels increase until the carbon dioxide levels reach a certain point when the reaction rate will plateau.

Temperature

Temperature also affects the rate of photosynthesis. All light-dependent and light­independent reactions are catalysed by enzymes. Enzyme activity initially increases as temperature increases, but enzyme molecules become denatured above optimum temperatures, and can no longer function. For this reason, the rate of photosynthesis will approximately double for every increase in temperature until the optimum temperature is reached. Above the optimum temperature, the rate of photosynthesis will not stay level. It will decline steeply as the enzymes become denatured and the chemical processes involved in the light-independent reactions cannot be catalysed (see Figure 3.3.15). Different plants are adapted to different environments; hence, there is no fixed optimum temperature for plant enzymes. The enzymes in a **cactus** will have a higher optimum temperature than those in an aquatic plant in cool water. Plant enzymes are described further in Section 3.4.

Balancing photosynthesis and cellular respiration

Cells release chemical energy by breaking apart glucose molecules. This process is called **cellular respiration**. As well as producing energy in the form of ATP, **cellular respiration** produces carbon dioxide and water.

For plants, **cellular respiration** occurs continuously, whereas **photosynthesis** occurs only during daylight. So although plants and algae produce carbon dioxide and use oxygen continuously through **cellular respiration**, this is masked during the day because **photosynthesis** is also occurring, using carbon dioxide and producing oxygen.

At low levels of light intensity, the rate of cellular respiration is greater than the rate of photosynthesis. This means there is a net uptake of oxygen by plants. At high light levels, the rate of photosynthesis is greater than the rate of cellular respiration, so there is a net output of oxygen by plants. The light compensation point is the level of light at which the rates of photosynthesis and cellular respiration of a photosynthetic organism are equal and there is no net exchange of oxygen (Figure 3.3.16). At this point, the amount of oxygen used in **cellular respiration** and the amount produced by **photosynthesis** are equal.

CELLULAR RESPIRATION IN EUKARYOTIC CELLS

Cells need energy to do work-and in mammals and birds, to also generate body heat. The energy used by organisms and their cells is stored in the chemical bonds of organic compounds. There are different ways that organisms acquire energy in this form.

Energy cannot be created or destroyed-but it can be changed from one form into another. Cells obtain energy from organic compounds, such as glucose. They transform the chemical energy stored in organic compounds into a more usable form of chemical energy, which is stored in the **bonds of ATP**. This transformation of energy occurs by a biochemical process called **cellular respiration**.

The biochemical pathway of cellular respiration varies according to the availability of free oxygen. It also depends on the availability of a basic carbohydrate-the glucose molecule. Animals (heterotrophs) obtain their glucose by eating other organisms and breaking down the organic molecules. Plants (autotrophs) make their own glucose from raw materials.

**Cellular respiration** is important not only for cells, but on a much larger scale. A healthy ecosystem works by the interconnection and balance of cellular respiration and photosynthesis. These biochemical processes in plant and animal cells are part of the **carbon and oxygen cycles** that sustain life.

Cells need energy to do work

All living cells require energy to carry out their functions. Energy can be defined as the ability to cause change. For example, you are using energy right now to move your eyes to read these words, just as the cells inside your body are currently using energy to transport substances across their membranes. You, like all organisms, are constantly expending energy.

Energy exists in many forms. The energy in sunlight is solar energy. The heat generated by your body is thermal energy. When you turn a page, the movement involves kinetic (movement) energy. Chemical energy is the potential energy that can be released by a chemical reaction. Chemical energy is stored in the bonds or connections that join atoms together; for example, between atoms of carbon and hydrogen in organic compounds, such as glucose, fats and proteins. The cells of all organisms use the energy stored in these organic compounds.

When energy is required by a cell, the bonds of organic compounds are broken and energy is released and stored in ATP molecules where it is readily accessible.

ATP (adenosine triphosphate)

ATP is the universal carrier of energy in living organisms. Molecules of ATP are the cell's store of immediately usable chemical energy that is required for cell processes. They can be compared to a charged battery that releases energy when required and can be easily recharged.

The ATP molecule contains two high-energy bonds between the inorganic phosphate groups. These bonds can be easily broken to release a small 'packet' of energy. These packets of energy are used to carry out all the energy-dependent processes of cells. How many are used at once depends on how much energy is required.

When an ATP molecule gives up its energy, it splits into a molecule of ADP (adenosine diphosphate) and a phosphate ion. This process is reversible, because the ADP can readily combine back with a phosphate ion to form an ATP molecule again, using energy derived from the breakdown of glucose during **cellular respiration**. The recycling process requires much less energy than it would take to make an entirely new ATP molecule. It is like recharging an existing battery, rather than buying a new one.

Cells obtain most of their energy for making ATP by breaking apart glucose, lipids and proteins. Glucose is one of the energy-rich products of **photosynthesis**.

The chemical energy from the glucose molecule is released in a series of steps that involve many enzymes producing many packets of energy as described above. Each energy packet can be used to produce an ATP molecule from ADP and a phosphate ion.

Biochemical pathway for cellular respiration

The breakdown of glucose molecules (Figure 3.3.22) results in three products:

• carbon dioxide

• water

• energy.

Of the chemical energy released in the breakdown of glucose, almost 40% is converted to energy stored in the bonds of ATP molecules during aerobic respiration. The rest is lost as heat.

**Cellular respiration** can be divided into two main stages: Glycolysis is the first stage, which splits glucose molecules into two parts and does not require oxygen.

Aerobic respiration (if oxygen is present) or anaerobic respiration (if oxygen is absent; also called fermentation) is the second stage. In a living cell, anaerobic respiration occurs if insufficient oxygen is available, but aerobic respiration happens at the same time, using all the available oxygen. The total amount of energy that can be harvested depends on whether or not oxygen is present.

Glycolysis

Glycolysis is the first process that takes place in **cellular respiration**. It involves the splitting (]ysis) of glucose into two pyruvate molecules (Figure 3.3.23). This occurs in the cytosol of the cell and is anaerobic; that is, it does not require oxygen. Glycolysis uses two ATP molecules in breaking down one glucose molecule, but it produces four more. Therefore, there is a net gain of two ATP molecules.

The process of glycolysis is common to both aerobic and anaerobic respiration. The pathway followed after glycolysis depends on whether or not there is an adequate supply of molecular oxygen. If there is enough oxygen, aerobic respiration takes place. If there is not enough oxygen, anaerobic respiration takes place.

Aerobic respiration

When molecular oxygen is available, the next stage of **cellular respiration** after glycolysis is aerobic respiration. Aerobic respiration occurs in **mitochondria** and converts ADP to ATP. During aerobic respiration, the two pyruvate molecules produced during glycolysis (from one glucose molecule) are then broken down to produce carbon dioxide, water and 36 ATP molecules (Figure 3.3.25).

Anaerobic respiration

If there is not enough oxygen available, aerobic respiration does not occur. Instead, the pyruvate passes into an anaerobic pathway. This anaerobic pathway is known as anaerobic respiration or fermentation. Anaerobic respiration occurs in the **cytosol**, not in the mitochondria. During anaerobic respiration, there is no further formation of ATP. The purpose of anaerobic respiration is to prevent the accumulation, or build-up, of pyruvate, which in turn allows glycolysis to continue. If pyruvate accumulates, the process of glycolysis slows, and no energy is available to the cell. Accumulation of a product can slow a chemical reaction.

In anaerobic respiration, the pyruvate produced during glycolysis is converted into either lactic acid (in most animals) or carbon dioxide and alcohol (in most plants and in microorganisms such as yeasts and bacteria). These two pathways are shown in Figures 3.3.23 and 3.3.24.

Aerobic vs anaerobic respiration

Aerobic respiration and anaerobic respiration both enable organisms to maintain their energy stores, but they have different functions (Table 3.3.1). For each molecule of glucose, aerobic respiration produces almost 20 times the number of ATP molecules produced by glycolysis alone. So it is not surprising that most eukaryotic cells normally carry out aerobic respiration. Most eukaryotic cells only rely on anaerobic respiration to continue the generation of ATP by glycolysis for very short periods (seconds), or when there is not enough oxygen available for aerobic respiration.

Transporting oxygen to cells

Organisms that use aerobic respiration need to transport oxygen to their cells. In mammals, oxygen is made available to cells via the lungs. In the **lungs**, oxygen diffuses from the alveoli into the blood (Figure 3.3.26). The oxygen combines with a protein called haemoglobin to form oxyhaemoglobin, which is transported to cells by the body's circulatory system. Oxygen is then released from the oxyhaemoglobin and diffuses into the cells. The cells use the oxygen for aerobic respiration, which supplies them with the energy they need to function. In **heterotrophic cells**, a reverse process removes the waste carbon dioxide produced by **aerobic cellular respiration**. You will learn more about the respiratory system in Chapter 5.

Mitochondria

Mitochondria are often called the power plants of cells, because they produce most of the ATP used in a cell. Mitochondria are relatively large organelles with a double membrane. The inner membrane forms many folded layers called cristae (Figure 3.3.27). The **cristae** provide a large surface area on which the chemical reactions of aerobic respiration take place.

The number of mitochondria in a cell is related to the cell's energy requirements. Very active cells, such as muscle cells and neurons (nerve cells), may have thousands of mitochondria (Figure 3.3.28).

Some important points about glycolysis and aerobic respiration in mitochondria are summarised in Figure 3.3.29 and Table 3.3.2.

Just as **photosynthesis** is often simplified into a single equation (Figure 3.3.7), **cellular respiration** can also be represented by a generalised chemical equation (Figure 3.3.25). Again, in reality, **cellular respiration** is a complex biochemical pathway involving many steps, each controlled by enzymes. At the simplified level, the two processes appear to be reciprocal (opposites). While this is not strictly true, it is a useful way to remember the overall equations and the importance of both in biological systems. The difference between the two is that **photosynthesis** uses solar energy and chlorophyll, whereas **cellular respiration** uses chemical energy only, and does not require light or chlorophyll.

REMOVAL OF CELLULAR PRODUCTS AND WASTES IN

EUKARYOTIC CELLS

During the biochemical processes of **photosynthesis and cellular respiration**, by­products are produced by cells. Cells need efficient ways to remove these waste products so they don't accumulate in the cell and inhibit normal cell functions. For example, a build-up of pyruvate from glycolysis would slow further glycolysis (Figure 3.3.23). Cells, tissues, organs and systems have several ways of removing waste products from an organism.

Cellular waste removal

Eukaryotic cells have organelles that are specialised for processing and removing waste products from cells. Two important structures that process and remove wastes inside cells are lysosomes and proteasomes.

lysosomes

Lysosomes are small vesicles that are filled with digestive enzymes. The lysosomes float in the **cytosol** of the cell, fusing with vesicles that contain waste products or damaged cellular structures. The digestive enzymes inside lysosomes break down waste products into smaller molecules.

Once the waste products are small enough, they are either reused by the cell, or transported out of the cell through the cell membrane via exocytosis. The removal of waste products via exocytosis is covered in Section 3 .1.

Lysosomes are found in all animal cells and some plant cells. Lysosomes are examined in more detail in Chapter 2.

Proteasomes

Proteasomes are protein complexes that degrade damaged proteins by breaking their peptide bonds. They are found in the **cytoplasm** and **nucleus** of all eukaryotic cells, and in **archaea and some bacteria**.

Proteasomes regulate the concentrations of proteins inside the **cell** and destroy proteins that are misfolded. The chemical reaction that proteasomes use to degrade proteins is called proteolysis, and the enzymes that catalyse these reactions are called proteases.

Waste-removing organs and systems

Waste removal not only occurs at the cellular level but also within tissues, organs and systems that are specialised for this function. Two important organs in the excretory system of mammals are the liver and kidney.

The liver prepares wastes

The liver in mammals performs many different functions and has a central role in the maintenance of a stable internal environment. In addition, it is responsible for preparing various substances for excretion. It detoxifies a variety of harmful chemicals, such as alcohol and some drugs. It is also responsible for breaking down amino acids to release ammonia, which it then converts largely into urea. The waste products from these processes travel in the bloodstream to the kidneys for excretion in the urine.

The liver also destroys worn-out red blood cells, producing bile pigments from the breakdown of haemoglobin. Bile pigments (along with bile salts, which emulsify fats as part of digestion) are stored in the **gall bladder** before they are released into the lumen of the intestine. From here, the bile joins undigested food to be excreted in **the faeces**, a process known as egestion in mammals. Bile pigments are one of the few substances excreted into the gut.

The first nitrogenous waste to be formed from the breakdown of protein is ammonia. Ammonia can be converted into urea or uric acid. This process comes at an energy cost, but makes the waste less toxic.

Neither urea nor uric acid are of any further use to most animals. But a rare few animals, such as sharks, are adapted to maintain high levels of nitrogenous wastes­particularly urea-within their body to aid in water regulation.

The blood of most sharks is isotonic to their marine environment. This means that there is an equal concentration of solutes (dissolved substances) in both their body and the ocean. They maintain osmotic balance with the seawater and are known as osmoconformers. Unlike seawater, with its dissolved salts such as sodium chloride, the shark uses dissolved urea in its body fluids. The urea is retained after the production of nitrogenous wastes. The difficulty with this strategy, and the reason other vertebrates excrete their urea, is that urea destabilises many enzymes necessary for the body's chemical reactions. A shark counters this effect with another solute: tri-methyl amine oxide (TMAO). After a shark dies, the urea and TMAO in their rotting flesh convert into distinctive, foul-smelling chemicals.

The kidneys excrete wastes

The kidneys of all vertebrates perform four main steps:

•filtration of blood

•reabsorption of useful substances back into the blood

•active secretion of extra wastes into the filtrate (forming waste called urine)

•elimination of the unwanted urine from the organism.

Within the kidney are large numbers of microscopic functioning units called nephrons. Fish have the least complex nephrons, while mammals have the most complex. The two human kidneys contain approximately one million nephrons each, all within a vital organ that is roughly fist-sized in an adult.

During its routine circulation around the body, blood passing through each kidney is filtered through the blood vessel walls to form a primary filtrate in the nephron tubules. The filtrate has the same composition as blood plasma, except that large protein molecules, as well as blood cells, have been filtered out. Most of the useful substances in the primary excretion are quickly reabsorbed as it passes along each nephron tubule. Some extra unwanted substances may be secreted into the fluid in the tubule before it passes as urine from the kidney to the bladder. The formation of urine involves passive filtration, selective reabsorption and secretion, and the passive removal of water (Figure 3.3.34).

These processes regulate the concentration of different salts in the **blood**, including those responsible for maintaining the pH of body fluids within closely controlled limits. Mammals conserve water by producing urine that is more concentrated than body fluids. In some mammals, such as the desert-dwelling bilby (Figure 3.3.35) or hopping mouse (Figure 3.3.36), the ability to produce concentrated urine is related to the degree of water stress experienced in their normal environments.

3.4 Enzyme activity in cells

Cells have mechanisms that regulate biochemical reactions to ensure the final product is not overproduced or underproduced. Enzymes are protein molecules that catalyse biochemical reactions and regulate biochemical pathways. The highly specific nature of enzymes ensures that particular enzymes only bind to particular substrates, catalysing specific chemical reactions. Enzyme activity is altered by the environment around the enzyme and sometimes by the availability of cofactors.

FEATURES OF ENZYMES

Most enzymes are globular-type protein molecules. This means they are formed from long chains of amino acids that coil and fold into a compact structure. Most globular proteins are soluble. The 3D shape of an enzyme molecule is critical to the enzyme's activity. Because the molecules are so complex and have a large number of atoms, illustrations use computer-generated models to represent their structure.

The main features of enzymes are their specificity for a substrate, their catalytic power and their capacity to catalyse repeated reactions.

Specificity-different enzymes act as catalysts for different biochemical reactions by binding to a specific type of molecule, called a substrate (Figure 3 .4.1). Although some enzymes have evolved to be highly specific, binding to only one substrate and catalysing one specific reaction, other enzymes can act on multiple substrates that are similar and catalyse multiple reactions.

Catalytic power-enzymes do not make reactions occur that would not occur on their own; they only make reactions occur more quickly (sometimes more than a million times faster).

Enzymes are not consumed when they catalyse reactions-they do not form part of the products of the reactions they catalyse. At the end of a reaction, enzyme molecules are the same as they were at the beginning. This means that enzymes can be reused over and over again.

Enzyme specificity

An important structure of enzymes is their active site. This is a pocket or groove­like depression in the enzyme's structure formed from the tertiary (3D) folding of the protein. Each enzyme's active site is a complex 3D shape that interacts with a specific substrate to catalyse a specific reaction. When the active site binds to the substrate, it forms an enzyme-substrate complex (Figure 3.4.4).

Enzyme-substrate interaction models

Two models describe how enzymes and their substrates interact (Figure 3.4.5).

The lock-and-key model describes the active site and the specific substrate as fitting together like a lock and key. If the 'key' (the substrate), does not fit into the 'lock' (the enzyme's active site), then no reaction occurs. This is the older version of the interaction model, and is now thought to be less accurate.

The induced-fit model states that when a substrate binds to the active site of an enzyme, the active site changes shape. This model is a more accurate representation of enzyme-substrate interactions. We now know that the active site is flexible, capable of changing its shape to conform to the shape of the substrate and achieve a tighter fit.

Enzymes and energy in reactions

Metabolism is the collection of all the biochemical (metabolic) reactions that occur in **living cells**. Metabolic reactions can be catabolic or anabolic for energy, and each is enzyme-controlled (Figure 3.4.6).

Catabolic reactions break down substrates. Catabolic reactions are exergonic, because they release energy.

Anabolic reactions produce larger molecules from smaller substrates. Anabolic reactions are endergonic because they require energy to form bonds between molecules.

Some reactions are not energetically favourable and require an input of energy.

To address this need for energy input, biochemical pathways sometimes couple a reaction that releases energy with a reaction that requires energy.

PLANT ENZYMES

Plants also carry out many biochemical reactions. These include photosynthesis and cellular respiration. Biochemical reactions need to be regulated in **plant cells**, just as they are in animal cells. Different enzymes catalyse the biochemical pathways in plants, but many of these enzymes have similar features to those in **animal cells**.

Photosynthesis

Enzymes play a role in **photosynthesis**, particularly during the light-independent stage of photosynthesis. The first step in the cycle is when carbon dioxide combines with ribulose biphosphate. An enzyme called RuBisCo catalyses this fixation of carbon dioxide (Figure 3.4.7). The cycle can then proceed to produce glyceraldehyde-3-phosphate, which the plant uses to make carbohydrates.

Because the light-independent reactions of **photosynthesis** are catalysed by enzymes, the rate of reaction changes as enzyme activity changes. At very low and very high temperatures, enzyme activity is disrupted, and the light-independent stage of **photosynthesis** slows down or stops.

Other plant enzymes

Some herbicides (chemicals that kill plants) work by blocking plant enzyme activity. A tiny herbicide molecule can attach to the active site of an enzyme and stop it from working. Such molecules are called irreversible inhibitors. Some plants have adapted to herbicides by changing one or two amino acids in their enzymes. These amino acid changes allow the enzymes to adjust their structure and continue working. As a result, these plants have become herbicide resistant.

Insectivorous plants, such as Venus fly traps, pitcher plants and sundews, produce a sticky liquid that attracts and traps insects (Figure 3.4.8). The liquid contains digestive enzymes that break down the insect body supplying extra nutrients to the plant.

Fruit enzymes

Many fruits, such as pineapple, papaya, fig and kiwifruit, contain enzymes called proteases. Proteases speed up the breakdown of proteins. Enzymes extracted from these fruits have uses as medicines, food-processing agents and dietary supplements. For example, the protease enzyme from papaya, called papain, is used for tenderising meat, treating wounds, dietary supplements and removing sediment from cold beers (Figure 3.4.9). If you have ever tried to make jelly that includes one of these fresh fruits, you will find that it doesn't set. This is because the gelatin protein has been digested by the fruit enzyme, preventing its setting action.

FUNGI ENZYMES

Many fungi are saphrotrophs. They cause decay by releasing enzymes onto the dead animal or plant (Figure 3.4.12). These break down complex compounds into simple, soluble compounds that can be absorbed by the decomposer. For example, if a fungus grows on a piece of bread, it releases amylase enzymes to digest the bread starch into maltose and then absorbs the maltose (Figure 3.4. l 2a).

Several different fungi species cause tinea in humans using a similar action. This infection is also known as athlete's foot, because it is common around the toes and often spread through dampness in communal change rooms and shower floors at sporting facilities. Enzymes secreted by the infectious fungi soften and irritate the skin between the toes. Fungicide creams can be used for treatment, but prevention is best-by keeping the toes dry, avoiding walking barefoot on damp, communal floors and not sharing towels or socks.

Many other disease-causing fungi infect plants and animals, with their action usually coming from the enzymes secreted by the fungi to obtain the nutrients they need. The fungus group includes organisms known as mould, rot and yeast (Figure 3.4.12b).

However, fungi and their enzymes are an essential part of decomposition cycles in the environment. The combined actions of a community of diverse microbes (including fungi with their secreted exo-enzymes) convert complex organic compounds into easily assimilated nutrients for other species. These microbial communities are ubiquitous in nature, inhabiting both terrestrial and aquatic ecosystems. This cycling of elements from dead organic matter by heterotrophic soil microbes is essential for nutrient turnover and energy transfer in terrestrial ecosystems. Wood-rotting fungi are among the only organisms that produce enzymes capable of breaking down the tough lignin and cellulose of dead plant cell walls and wood fibre (Figure 3.4.12c).

FACTORS THAT AFFECT ENZYME ACTIVITY

When cells produce too much or too little of particular substances, or are unable to properly break down substances, the whole organism can suffer. Cells that produce excess substances are also wasting energy and resources. To account for this, cells have mechanisms that regulate biochemical reactions (metabolism) to ensure the final product is not overproduced or underproduced.

If the regulatory mechanisms fail, a variety of problems emerge for a human body-from mild to fatal. One example is known as maple syrup urine disease (Figure 3.4.13). The disease is caused by a defective group of enzymes that normally work together: an enzyme complex called the branched-chain ketoacid dehydrogenase complex. It results in a build-up of branched-chain amino acids that causes the urine to smell like maple syrup, and the blood to become acidic (low pH), which can result in death.

As you've already learnt, enzymes control metabolism through the catalysis of reactions at every step of a biochemical (or metabolic) pathway. A whole pathway can be regulated through control of one enzyme. For this reason, it is important to understand the ways in which enzyme activity is affected by their environment.

All enzymes have specific conditions under which they perform at their best. Factors such as temperature, pH and the concentration of the substrate and enzyme all affect the rate of enzymatic reactions. When these conditions are optimal, enzyme activity is at its highest, the rate of reaction is at its fastest, and the biochemical pathway is operating at maximum efficiency. There are times when conditions are not optimal, leading to decreased enzyme activity and slower reaction rates. One example is the protein-digesting enzyme, pepsin, which only becomes active when it enters the low-pH environment of the acidic stomach juices (pH 2). Other examples in relation to limiting factors for photosynthesis are described in Section 3.3.

The amounts of final products and the speed at which they are produced in a biochemical pathway can therefore be controlled through the regulation of the pathway's enzymes and individual reactions. Different enzymes may be used in each step of a biochemical pathway (Figure 3.4.14). It is common to have a sequence of chemical reactions in which the product from the previous reaction is used as a substrate for the next reaction. (Figure 3 .4.15). Slowing down one reaction will have an effect on all subsequent reactions.

Some biochemical pathways are linear, while some are branched, leading to many final products. Others are cycles.

Temperature

As with most chemical reactions, the rate of enzyme-catalysed reactions will generally increase as the temperature increases. This is because the warmer that particles are during a reaction, the more rapidly they move, which makes successful collisions and reactions between them more likely to occur.

However, proteins-including enzymes-can be denatured at high temperatures. When this occurs, the hydrogen bonds and hydrophobic interactions that create the coiled, globular, tertiary (3D) shape of the enzyme are broken. The shape of the enzyme molecule is changed so that the substrate cannot bind to it, and the reaction cannot occur. Even the induced-fit process does not provide an active site on the enzyme for the substrate molecule. Denaturation of a protein is usually irreversible. However, an Australian biochemist recently discovered how to return cooked egg white protein back to its clear, runny form. This has opened up new fields of research.

Human enzymes have their optimum activity at a temperature of 36-38°C, because that matches our normal body temperature (approximately 37°C). Many animal enzymes will begin to denature at temperatures above 40°C (Figure 3 .4.18). However, some enzymes have optimum temperatures much higher than this. For example, Taq polymerase is an enzyme that was originally found in bacteria living in volcanic hot springs, and it has an optimum temperature of 70°C. The enzymes of each species, like their other adaptations, have evolved over time to enable the species to live successfully in their natural environment.

If enzymes are cooled below their optimum temperature, the rate of reaction will slow down. This is because particles will move more slowly at cooler temperatures, making successful collisions less likely. Chemical bonds are also not as flexible at cooler temperatures and conformational changes do not occur. However, enzymes are not denatured at low temperatures like they are at high temperatures. If an enzyme's environment increases in temperature, its activity and reaction rate will also increase. This is why the decomposition of food and ripening of fruit is slower in a refrigerator.

Enzymes at extreme temperatures

The sooty grunter (Hephaestus juliginosus), also known as sooties or black bream, is a species of fish that inhabits coastal and inland freshwater creeks and rivers of northern Australia, from the upper Burdekin River in Queensland to the Daly River in the Northern Territory (Figure 3.4.19). Sooty grunters inhabit large freshwater streams, preferring rapidly flowing waters with a rocky bottom and sparse, aquatic plant cover. This species is also sometimes found in hot springs. Sooty grunters can tolerate acidic conditions to a pH of 4.0 and water temperatures in the range of 12-34°C. The wide tolerance range of sooty grunters is due to their metabolic enzymes being able to function in these extreme environments.

Psychrophiles are bacteria and fungi that are adapted to the extreme cold of Antarctica and the Arctic (Figure 3.4.20). These organisms have enzymes that can function in a wide range of temperatures, or two optimum temperature zones, including an optimum range as low as O-l 5°C. This allows their cells to grow and carry out metabolic processes at the constant low temperatures of their cold environments.

Thermophiles are prokaryotes belonging to the Archaea domain. They tolerate very hot environments and contain enzymes with much higher optimal temperatures than those of mammals (37°C). The enzyme shown in Figure 3.4.21 can still function in temperatures up to 135°C without being denatured, making it of interest to scientists for potential industrial use.

pH

The pH scale is used to measure acidity or alkalinity (Figure 3 .4. 22). Enzymes have a specific pH range at which they function best. If enzymes are taken too far above or below their optimum pH, then their tertiary (3D) structure is affected, the enzyme may become denatured and the substrate may not be able to bind.

If the pH of the environment is not ideal, the micro-environment of the active site may be altered to provide a different pH and allow the reaction to take place.

The optimum pH range for enzymes can be quite different, and varies depending on the function of the enzyme and where it is located. Examples include the following digestive enzymes (Figure 3.4.23).

Amylase starts the digestion of starch in the mouth and has an optimum pH of about 7. Pepsin is found in the **stomach** and has an optimum pH of about 2 from the presence of hydrochloric acid.

Trypsin is found in the **small intestine** and has an optimum pH of about 8.

Enzyme and substrate concentration

The concentration of enzyme compared to substrate affects the rate of reaction. If the enzyme concentration is high compared to that of the substrate, the reaction will occur over a short period of time. This is because the more enzyme molecules that are available, the more active sites there will be for the substrate to bind to, and so the rate of reaction will be faster until saturation point is reached (Figure 3.4.24).

If the enzyme concentration is lower than the substrate concentration, then the reaction rate will be slow and will continue for longer. This is because there are few active sites compared to the number of substrate molecules, so the substrate molecules will have to 'wait' until there is a free active site on an enzyme molecule. When all sites are taken, the saturation point is reached.

Controlling the enzyme or substrate concentration in an organism's body is a way to regulate the speed of a biochemical pathway.

Cellular compartmentalisation

Cellular compartmentalisation in **eukaryotes** helps to create an environment in which conditions are optimal for enzymes to catalyse reactions (Figure 3.4.25). Each organelle has a specific function. Within an **organelle**, the molecules involved in biochemical pathways that are related to the organelle's function are brought close together for reactions to take place. It also makes a different environment possible within each organelle, such as different pH and cofactor chemicals that support activity of particular enzymes. There will be high concentrations of the enzymes and substrates for that organelle's function, such as in the chloroplasts for **photosynthesis** and mitochondria for **cellular respiration**.

Another example in animal cells is the lysosome, which is a specialised sac containing digestive enzymes called acid hydrolases (Figure 3.4.25c). These enzymes are delivered to lysosomes via vesicles that bud off the Golgi apparatus. Lysosomes contain about 40 types of enzyme that break down and recycle proteins, lipids, nucleic acids and more. These enzymes function optimally in the acidic lysosomal environment, around pH 5. Lysosomes digest cell components that are past their use-by date. Specialist phagocytic cells, such as macrophages, use lysosomal enzymes to destroy ingested foreign matter.

Inhibition of enzyme activity

Enzyme activity can also be changed by inhibitor molecules that bind to the enzyme's active site, preventing the substrate from binding.

The inhibition of enzyme activity by an inhibitor molecule can be reversible or irreversible, depending on the strength of the bonds between the enzyme and inhibitor. Reversible inhibitors are used to control enzyme activity by switching them on or off. Many poisons are irreversible inhibitors, e.g. heavy metals such as lead and mercury, which bind permanently to the active sites of important enzymes. Scientists have used knowledge of irreversible inhibitors to manufacture pesticides that act on enzymes in the **nervous system** of insects, or herbicides that bind to enzymes involved in the photosynthesis pathway. For example, herbicides such as amitrole and glyphosate inhibit the production of chlorophyll, and paraquat and diquat block the light­dependent reactions in the first part of photosynthesis in green plants (Figure 3 .4.26).

Enzyme inhibition is also classified as being competitive inhibition or non­competitive inhibition, depending on where the inhibiting molecule binds to the enzyme (Figure 3.4.27).

Feedback inhibition occurs when a product that is produced late in a biochemical pathway is also an inhibitor of an enzyme earlier in the pathway. As the amount of the inhibiting product increases, the amount of enzyme molecules being inhibited also increases (Figure 3.4.28). This in turn reduces the amount of the inhibiting product. As the level of inhibiting product reduces, less of it will bind to the enzymes, allowing them to function again. Feedback inhibition is an important mechanism in controlling enzyme activity.

Phosphorylation

The binding of a phosphate group to a protein is known as phosphorylation. The removal of a phosphate group from a protein is known as dephosphorylation. Phosphorylation is thought to be the most common regulatory mechanism of protein function, with as many as one-third of all proteins in the human body being substrates for phosphorylation at some point.

Phosphorylation and dephosphorylation both change the structure (or conformation) of proteins, and therefore both can regulate enzymes. For example, the phosphorylation of glycogen synthase inhibits its ability to interact with glucose. The phosphorylation of glycogen synthase is performed by another enzyme, called glycogen synthase kinase 3.

Cofactors and coenzymes

Some enzymes need additional components to enable them to catalyse a reaction. These components, called cofactors, bind to the enzyme. Cofactors can be inorganic ions such as iron (Fe2+), magnesium (Mg2+) and zinc (Zn2+), or organic molecules such as proteins, vitamins and ATP.

Small, non-protein organic cofactors are known as coenzymes (Figure 3.4.29). For certain enzymes, a specific coenzyme is required to catalyse reactions. Often the coenzyme is structurally altered during the reaction, but afterwards reverts to its original form, which allows it to be reused. There are many types of coenzymes. They include vitamins, ATP, nicotinarnide adenine dinucleotide (NADH), flavin adenine dinucleotide (FADH2) and nicotinarnide adenine dinucleotide phosphate (NADPH).

Except for vitamin C, all other vitamins must be modified in the body before they can function as coenzymes. An example is vitamin A, which is a coenzyme for the production of some proteins, such as the light-absorbing pigments in the retina of the human eye. The popular belief that eating carrots is good for night vision has substance, because the carrot's orange colour indicates the presence of vitamin A, which is used by the body to improve vision in low light.

The vitamin B complex consists of eight vitamins that are used in the body to produce several coenzymes. Some examples are:

•Bl (thiamin), B2 (riboflavin) and B3 (niacin) work with enzymes in the conversion of glucose to energy ( cellular respiration).

•B9 (folate) is needed to form red blood cells, and in fetal development for the nervous system, DNA synthesis and cell growth. Therefore, it is very important for pregnant women and has been related to prevention of spina bifida in babies.

•B12 is important for maintenance of myelin (the insulation around nerve cells) and in energy production. B9 and B 12 operate together.

Controlling the availability of cofactors is another way for the body to regulate enzyme activity.

**Textbook 3: Dynamics of life Vol. 1**

The Life of a Cell

The Chemistry of Life

Atoms and Their Interactions

Elements

Everything—whether it is a rock, frog, or flower—is made of substances called elements. Suppose you find a nugget of pure gold. You could grind it into a billion bits of powder and every particle would still be gold. You could treat the gold with every known chemical, but you could never break it down into simpler substances. That’s because gold is an element. An element is a substance that can’t be broken down into simpler chemical substances.

Natural elements in living things

Of the naturally occurring elements on Earth, only about 25 are essential to living organisms. Table 6.1 on the next page lists some elements found in the human body. Notice that four of the elements—carbon, hydrogen, oxygen, and nitrogen—together make up more than 96 percent of the mass of a human body. Each element is identified by a one-or two-letter abbreviation called a symbol. For example, the symbol C represents the element carbon, Ca represents the element calcium, and Cl represents the element chlorine.

Trace elements

Some of the elements listed in Table 6.1, such as iron and copper, are present in living things in very small amounts. Such elements are known as trace elements. They play a vital role in maintaining healthy cells in all organisms, as shown by the examples in Figure 6.1. Plants obtain trace elements by absorbing them through their roots; animals get them from the foods they eat.

Atoms: The Building Blocks of Elements

Whether elements are found in living things, like cup corals, mammals, or plants, or in nonliving things, like rocks, they are made of atoms. An atom is the smallest particle of an element that has the characteristics of that element. Atoms are the basic building blocks of all matter. The way they are structured affects their properties and their chemical behavior.

The structure of an atom

All atoms have the same general structure. The center of an atom is called the nucleus (plural, nuclei). All nuclei contain positively charged particles called protons (p+). Most contain particles that have no charge, called neutrons (n0). All nuclei are positively charged because of the presence of protons. Each element has distinct characteristics that result from the number of protons in the nuclei of the atoms that compose the element. For example, the element iron differs from the element aluminum because iron atoms have a different number of protons than aluminum atoms.

The region of space surrounding the nucleus contains extremely small, negatively charged particles called electrons (e—). The electrons are held in this region by their attraction to the positively charged nucleus. You can visualize this region as an electron cloud. Although it is impossible to pinpoint the exact location of an electron, the electron cloud is the area where it is most likely to be found.

Electron energy levels

Electrons exist around the nucleus in regions known as energy levels, as indicated in Figure 6.2A. The first energy level can hold only two electrons. The second level can hold a maximum of eight electrons. The third level can hold up to 18 electrons. The oxygen atom in Figure 6.2C has a total of eight electrons. Two electrons fill the first energy level. The remaining six electrons occupy the second energy level.

Atoms contain equal numbers of electrons and protons; therefore, they have no net charge. The hydrogen (H) atom in Figure 6.2B has just one electron and one proton. Oxygen (O) has eight electrons and eight protons.

Isotopes of an Element

Atoms of the same element always have the same number of protons but may contain different numbers of neutrons. Atoms of the same element that have different numbers of neutrons are called isotopes of that element. For example, most carbon nuclei contain six neutrons. However, some have seven or eight neutrons. Each of these atoms is an isotope of the element carbon. Scientists refer to isotopes by stating the combined total of protons and neutrons in the nucleus. Thus, the most common carbon atom is referred to as carbon-12 because it has six protons and six neutrons. Other isotopes of carbon include carbon-13 and carbon-14.

Isotopes are often useful to scientists. The nuclei of some isotopes, such as carbon-14, are unstable and tend to break apart. As nuclei break, they give off radiation. These isotopes are said to be radioactive. Because radiation is detectable and can damage or kill cells, scientists have developed some useful applications for radioactive isotopes, as described in Figure 6.4.

Atomic models like those discussed in the Problem-Solving Lab on the next page help scientists and students visualize the structure of atoms and understand complex intermolecular interactions.

Compounds and Bonding

Table salt is a substance that is familiar to everyone; however, table salt is not an element. Rather, salt is a type of substance called a compound. A compound is a substance that is composed of atoms of two or more different elements that are chemically combined. Table salt (NaCl) is a compound composed of the elements sodium and chlorine. If an electric current is passed through molten salt in an industrial process, the salt breaks down into these elements. You can see in Figure 6.5 that the properties of a compound are different from those of its individual elements.

How covalent bonds form

Most elements in nature are found combined in the form of compounds. But how and why do atoms combine, and what is it that holds the atoms together in a compound? Atoms combine with other atoms only when the resulting compound is more stable than the individual atoms.

For many elements, an atom becomes stable when its outermost energy level is full, as when eight electrons are in the second level. An exception is hydrogen, which becomes stable when its first energy level is full (two electrons). How do elements fill the energy levels and become stable? One way is to share electrons with other atoms.

Look at Figure 6.6A. You will see that two hydrogen atoms can combine with each other by sharing their electrons. As you know, hydrogen atoms contain only one electron. Each atom becomes stable by sharing its electron with the other atom. The two shared electrons move about the nuclei of both atoms. The attraction of the positively charged nuclei for the shared, negatively charged electrons holds the atoms together. When two atoms share electrons, such as two hydrogen atoms sharing electrons, the force that holds them together is called a covalent bond. Most compounds in organisms have covalent bonds. Examples include sugars, fats, proteins, and water.

A molecule is a group of atoms held together by covalent bonds. It has no overall charge. In a molecule of water, Figure 6.6B, two hydrogen atoms and one oxygen atom share eight electrons. Each of the hydrogen atoms contributes one electron, and the oxygen atom contributes six electrons. Thus, all three atoms are stable.

A molecule of water is represented by the chemical formula H2O. The subscript 2 represents two atoms of hydrogen (H) combined with one atom of oxygen (O). As you will see, many compounds in living things have more complex formulas.

How ionic bonds form

Not all atoms bond with each other by sharing electrons. Sometimes atoms combine with each other by first gaining or losing electrons in their outer energy levels. An atom (or group of atoms) that gains or loses electrons has an electrical charge and is called an ion. An ion is a charged particle made of atoms.

A different type of chemical bond holds ions together. The bond formed between a sodium atom (Na) and chlorine atom (Cl) in table salt is a good example of this. A sodium atom contains 11 electrons, including one in the third energy level. A chlorine atom has 17 electrons, with the outer level holding seven electrons. The sodium atom loses one electron to the chlorine atom, and the chlorine atom gains one electron from the sodium atom. With eight electrons in its outer level, the chloride ion formed is stable and has a negative charge. The sodium ion has eight electrons in its outer energy level. The sodium ion is stable and has a positive charge. The attractive force between two ions of opposite charge is known as an ionic bond. The bond between sodium and chlorine when they combine is an ionic bond, as shown in Figure 6.7.

Ionic compounds are less abundant in living things than are covalent molecules, but ions are important in biological processes. For example, sodium and potassium ions are required for transmission of nerve impulses. Calcium ions are necessary for muscles to contract. Plant roots absorb essential minerals in the form of ions.

Chemical Reactions

Chemical reactions occur when bonds are formed or broken, causing substances to recombine into different substances. In organisms, chemical reactions occur inside cells. All of the chemical reactions that occur within an organism are referred to as that organism’s metabolism. These reactions break down and build molecules that are important for the functioning of organisms. Scientists represent chemical reactions by writing chemical equations.

Writing chemical equations

The reaction that takes place when hydrogen gas combines with oxygen gas is shown in Figure 6.8. In a chemical reaction, substances that undergo chemical reactions, such as hydrogen and oxygen, are called reactants. Substances formed by chemical reactions, such as water, are called products. It’s easy to tell how many molecules are involved in a reaction. In a chemical equation the number before each chemical formula indicates the number of molecules of each substance. The subscript numbers in a formula indicate the number of atoms of each element in a molecule of the substance.

A molecule of table sugar can be represented by the formula C12H22O11. The lack of a number before a formula or under a symbol indicates that only one molecule or atom is present.

Looking at Figure 6.8, you can see that each molecule of hydrogen gas is composed of two atoms of hydrogen. Likewise, a molecule of oxygen gas is made of two oxygen atoms. Perhaps the easiest way to understand chemical equations is to know that atoms are neither created nor destroyed in chemical reactions. They are simply rearranged. An equation is written so that the same numbers of atoms of each element appear on both sides of the arrow. In other words, equations must always be written so that they balance.

Mixtures and Solutions

When elements combine chemically to form a compound, the elements no longer have their original properties. What happens if substances are just mixed together and do not combine chemically? A mixture is a combination of substances in which the individual components retain their own properties. Figure 6.9 shows a mixture of sand and sugar crystals.

When you stir sand and sugar together, you can still tell the sand from the sugar. Neither component of the mixture changes; that is, the components would not combine chemically. You can easily separate them by adding water to dissolve the sugar and then filtering the mixture to collect the sand.

A solution is a mixture in which one or more substances (solutes) are distributed evenly in another substance (solvent). In other words, one substance is dissolved in another and will not settle out of solution. You may remember using powdered drink mix when you were younger. The sugar molecules in the powdered drink mix dissolve easily in water to form a solution, as shown in Figure 6.10.

Solutions are important in living things. In organisms, many vital substances, such as sugars and mineral ions, are dissolved in water. The more solute that is dissolved in a given amount of solvent, the greater is the solution’s concentration. The concentration of a solute is important to organisms. Organisms can’t live unless the concentration of dissolved substances stays within a specific, narrow range. Organisms have many mechanisms to keep the concentrations of molecules and ions within this range. For example, the pancreas and other organs in your body produce substances such as insulin and glucagon that keep the amount of sugar dissolved in your bloodstream within a critical range.

Acids and bases

Chemical reactions can occur only when conditions are right. A reaction may depend on available energy, temperature, or a certain concentration of a substance dissolved in solution. Chemical reactions in organisms also depend on the pH of the environment within the organism. The pH is a measure of how acidic or basic a solution is. A scale with values ranging from below 0 to above 14 is used to measure pH. Figure 6.11 shows the pH of some common substances.

Substances with a pH below 7 are acidic. An acid is any substance that forms hydrogen ions (H+) in water. When hydrogen chloride (HCl) is added to water, hydrogen ions (H+) and chloride ions (Cl—) are formed. Thus, hydrogen chloride in solution with water as a solvent is called hydrochloric acid. This acidic solution contains an abundance of H+ ions and has a pH below 7. A solution is neutral if its pH equals 7.

Substances with a pH above 7 are basic. A base is any substance that forms hydroxide ions (OH—) in water. For example, if sodium hydroxide (NaOH) is dissolved in water, it forms sodium ions (Na+) and hydroxide ions (OH—). This basic solution contains an abundance of OH— ions and has a pH above 7.

Water and Diffusion

Water and Its Importance

Water is perhaps the most important compound in living organisms. Most life processes can occur only when molecules and ions are free to move and collide with one another. This condition exists when they are dissolved in water. Water also serves to transport materials in organisms. For example, blood and plant sap, which are mostly water, transport materials in animals and plants. In fact, water makes up 70 to 95 percent of most organisms.

Water is polar

Sometimes, when atoms form covalent bonds, they do not share the electrons equally. The water molecule pictured in Figure 6.12A shows that the shared electrons are attracted by the oxygen nucleus more strongly than by the hydrogen nuclei. As a result, the electrons spend more time near the oxygen nucleus than they do near the hydrogen nuclei. When atoms in a covalent bond do not share the electrons equally, they form a polar bond. A polar molecule is a molecule with an unequal distribution of charge; that is, each molecule has a positive end and a negative end. As illustrated in Figure 6.12B, water is an example of a polar molecule. Polar water molecules attract ions as well as other polar molecules. Because of this attraction, water can dissolve many ionic compounds, such as salt, and many other polar molecules, such as sugar.

Water molecules also attract other water molecules. The positively charged hydrogen atoms of one water molecule attract the negatively charged oxygen atoms of another water molecule. This attraction of opposite charges between hydrogen and oxygen forms a weak bond called a hydrogen bond. Hydrogen bonds are important to organisms because they help hold many biomolecules, such as proteins, together.

Also because of its polarity, water has the unique property of being able to creep up thin tubes. Plants in particular take advantage of this property, called capillary action, to get water from the ground. Capillary action and the tension on the water’s surface, which is also a result of polarity, play major roles in getting water from the soil to the tops of even the tallest trees.

Water resists temperature changes

Water resists changes in temperature. Therefore, water requires more heat to increase its temperature than do most other common liquids. Likewise, water loses a lot of heat when it cools. In fact, water is like an insulator that helps maintain a steady environment when conditions fluctuate. Because cells exist in an aqueous environment, this property of water is extremely important to cellular functions as it helps cells maintain homeostasis.

Water expands when it freezes

Water is one of the few substances that expands when it freezes. Because of this property, ice is less dense than liquid water so it floats as it forms in a body of water. Use the Problem-Solving Lab on the next page to investigate this property. Water expands as it freezes inside the cracks of rocks. As it expands, it often breaks apart the rocks. Over long time periods, this process helps form soil.

The properties of water make it an excellent vehicle for carrying substances in living systems. One way to move substances is by diffusion.

Diffusion

All objects in motion have energy of motion called kinetic energy. A moving particle of matter moves in a straight line until it collides with another particle, much like the table tennis balls shown in Figure 6.13. After the collision, both particles rebound. Particles of matter, like the table tennis balls, are in constant motion, colliding with each other.

Early observations: Brownian motion

In 1827, Scottish scientist Robert Brown used a **microscope** to observe pollen grains suspended in water. He noticed that the grains moved constantly in little jerks, as if being struck by invisible objects. This motion, he thought, was the result of a life force hidden within the pollen grains. However, when he repeated his experiment using dye particles, which are nonliving, he saw the same motion. This motion is now called Brownian motion. Brown had no explanation for the motion, but today we know that Brown was observing evidence of the random motion of atoms and molecules. The random movement that Brown observed is characteristic of gases, liquids, and some solids.

The process of diffusion

Particles of different substances that are in constant motion have an effect on each other. For example, if you layer pure corn syrup on top of corn syrup colored with food coloring in a beaker as illustrated in Figure 6.14, over time you will observe that the colored corn syrup has mixed with the pure corn syrup. This mixture is the result of the random movement of corn syrup and water molecules. Diffusion is the net movement of particles from an area of higher concentration to an area of lower concentration. Diffusion results because of the random movement of particles (Brownian motion).

Diffusion is a slow process because it relies on the random motion of atoms and molecules. You will see evidence that the corn syrup in Figure 6.14 has begun to diffuse within hours but it will take months to mix completely if undisturbed.

Three key factors—concentration, temperature, and pressure—affect the rate of diffusion. The concentration of the substances involved is the primary controlling factor. The more concentrated the substances, the more rapidly diffusion occurs because there are more collisions between the particles of the substances. Two external factors—temperature and pressure—can change the rate of diffusion. An increase in temperature increases energy and will cause more rapid particle motion. This will increase the rate of diffusion. Similarly, increasing pressure will accelerate particle motion and, therefore, diffusion.

The results of diffusion

As the pure corn syrup continues to diffuse into the colored corn syrup, the two will become evenly distributed eventually. After this point, the molecules continue to move randomly and collide with one another; however, no further change in concentration will occur. This condition, in which there is continuous movement but no overall concentration change, is called dynamic equilibrium. Figure 6.15 illustrates dynamic equilibrium in a cell.

Diffusion in living systems

Most substances in and around a cell are in water solutions where the ions and molecules of solute are distributed evenly among water molecules, as in the powdered drink mix and water example. The difference in concentration of a substance across space is called a concentration gradient. Because ions and molecules diffuse from an area of higher concentration to an area of lower concentration, they are said to move with the gradient. If no other processes interfere, diffusion will continue until there is no longer a concentration gradient. At this point, dynamic equilibrium occurs. Diffusion is one of the methods by which cells move substances in and out of the cell.

Diffusion in biological systems is also evident outside of the cell and can involve substances other than molecules in an aqueous environment. For example, oxygen (a gas) diffuses into the capillaries of the lungs because there is a greater concentration of oxygen in the **air sacs** of the lungs than in the **capillaries**.

Life Substances

The Role of Carbon in Organisms

A carbon atom has four electrons available for bonding in its outer energy level. In order to become stable, a carbon atom forms four covalent bonds that fill its outer energy level. Look at the model showing carbon atoms and bond types in Figure 6.16. Carbon can bond with other carbon atoms, as well as with many other elements. When each atom shares two electrons, a double bond is formed. A double bond is represented by two bars between carbon atoms. When each atom shares three electrons, a triple bond is formed. Triple bonds are represented by three bars between carbon atoms.

When carbon atoms bond to each other, they can form straight chains, branched chains, or rings. These chains and rings can have almost any number of carbon atoms and can include atoms of other elements as well. This ability to bond in so many ways makes a huge number of carbon structures possible. In addition, compounds with the same chemical formula often differ in structure. Compounds that have the same chemical formula but different three-dimensional structures are called isomers. The glucose and colloid molecules shown in Figure 6.17 have the same formula, C6H12O6, but different structures.

Molecular chains

Carbon compounds vary greatly in size. Some compounds contain just one or two carbon atoms, whereas others contain tens, hundreds, or even thousands of carbon atoms. These large organic compounds are called biomolecules. Proteins are examples of biomolecules that are found in organisms. Cells build biomolecules by bonding small molecules together to form chains called polymers. A polymer is a large molecule formed when many smaller molecules bond together.

Many polymers are formed by a chemical reaction known as condensation. In condensation, the small molecules that are bonded together to make a polymer have an –H and an–OH group that can be removed to form H–O–H, a water molecule. The subunits become bonded by a covalent bond, as shown in Figure 6.18. These polymers can be broken apart by hydrolysis. Hydrogen and hydroxyl groups from water attach to the bonds between the subunits that make up the polymer, thus breaking the polymer as shown in Figure 6.18.

The structure of carbohydrates

You may have heard of runners eating large quantities of spaghetti or bread the day before a race. This practice is called “carbohydrate loading.” It works because carbohydrates are used by cells to provide energy. A carbohydrate is a biomolecule composed of carbon, hydrogen, and oxygen with a ratio of about two hydrogen atoms and one oxygen atom for every carbon atom.

The simplest type of carbohydrate is a simple sugar called a monosaccharide. Common examples are the isomers glucose and fructose. Two monosaccharide molecules can combine to form a disaccharide, a two-sugar carbohydrate. When glucose and fructose link together by a condensation reaction, a molecule of sucrose, known as table sugar, is formed.

The largest carbohydrate molecules are polysaccharides, polymers composed of many monosaccharide subunits. The starch, glycogen, and cellulose pictured in Figure 6.19 are examples of polysaccharides. Starch consists of branched chains of glucose units and is used as energy storage by plant cells and as food reservoirs in seeds and bulbs. Mammals store energy in the liver in the form of glycogen, a highly branched glucose polymer. Cellulose is another glucose polymer that forms the cell walls of plants and gives plants structural support. Cellulose is made of long chains of glucose units linked together in arrangements somewhat like a chain-link fence.

The structure of lipids

Lipids are large biomolecules that are made mostly of carbon and hydrogen with a small amount of oxygen. Fats, oils, waxes, and steroids are all lipids. They are insoluble in water because their molecules are nonpolar and are not attracted by water molecules.

A common type of lipid, shown in Figure 6.20, consists of three fatty acids linked with a molecule of glycerol. A fatty acid is a long chain of carbon and hydrogen. If each carbon in the chain is bonded to other carbons by single bonds, the fatty acid is said to be saturated. If a double bond is present in the chain, the fatty acid is unsaturated.

Fatty acids with more than one double bond are polyunsaturated.

Lipids are very important for the proper functioning of organisms. Cells use lipids for energy storage, insulation, and protective coverings. In fact, lipids are the major components of the membranes that surround all living cells. To learn more about lipids in your body, read the Biotechnology feature at the end of this chapter.

The structure of proteins

Proteins are essential to all life. They provide structure for tissues and organs and carry out cell metabolism. A protein is a large, complex polymer composed of carbon, hydrogen, oxygen, nitrogen, and sometimes sulfur.

The basic building blocks of proteins are called amino acids, shown in Figure 6.21A. There are about 20 common amino acids. These building blocks, in various combinations, make literally thousands of proteins.

Amino acids are linked together when an –H from the amino group of one amino acid and an –OH group from the carboxyl group of another amino acid are removed to form a water molecule. The covalent bond formed between the amino acids, like the bond labeled in Figure 6.21B, is called a peptide bond.

Proteins come in a large variety of shapes and sizes. The number and sequence of amino acids that make up a protein are important in determining its shape. Certain amino acids are acidic, some are basic, and some are not charged. These properties cause the amino acids to attract or repel each other in different ways. The amino acid chain that makes up the protein twists and turns as the amino acids interact. Many proteins consist of two or more amino acid chains that are held together by hydrogen bonds. The ultimate three-dimensional shape that the protein folds into is extremely important to the functioning of the protein. If the sequence of amino acids in the protein were to change, the protein might fold differently and not be able to carry out its function in the cell.

Proteins are the building blocks of many structural components of organisms, as illustrated in Figure 6.22. Proteins are also important in the contracting of muscle tissue, transporting oxygen in the bloodstream, providing immunity, regulating other proteins, and carrying out chemical reactions.

Enzymes are important proteins found in living things. An enzyme is a protein that changes the rate of a chemical reaction. In some cases, enzymes increase the speed of reactions that would otherwise occur slowly.

Enzymes are involved in nearly all metabolic processes. They speed the reactions in digestion of food. The activities of enzymes depend on the temperature, ionic conditions, and the pH of the surroundings.

The structure of nucleic acids

Nucleic acids are another important type of organic compound that is necessary for life. A **nucleic acid** is a complex biomolecule that stores cellular information in the form of a code. Nucleic acids are polymers made of smaller subunits called nucleotides.

Nucleotides consist of carbon, hydrogen, oxygen, nitrogen, and phosphorus atoms arranged in three groups—a nitrogenous base, a simple sugar, and a phosphate group— as shown in Figure 6.24. You have probably heard of the nucleic acid DNA, which stands for deoxyribonucleic acid. DNA is the master copy of an organism’s information code. The information coded in **DNA** contains the instructions used to form all of an organism’s enzymes and structural proteins. Thus, DNA forms the genetic code that determines how an organism looks and acts. DNA’s instructions are passed on every time a cell divides and from one generation of an organism to the next.

Another important nucleic acid is RNA, which stands for ribonucleic acid. RNA is a nucleic acid that forms a copy of DNA for use in making proteins. The chemical differences between RNA and DNA are minor but important. A later chapter discusses how DNA and RNA work together to produce proteins.

A View of the Cell

The Discovery of Cells

The History of the Cell theory

Before microscopes were invented, people believed that diseases were caused by curses and supernatural spirits. They had no idea that organisms such as bacteria existed. As scientists began using **microscopes**, they quickly realized they were entering a new world—one of microorganisms. **Microscopes** enabled scientists to view and study cells, the basic units of living organisms.

Light microscopes

The microscope Anton van Leeuwenhoek used in the 1600s is considered a simple light microscope because it contained one lens and used light to view objects. Over the next 200 years, scientists greatly improved microscopes by grinding higher quality lenses and developing the compound light microscope. **Compound light microscopes** use a series of lenses to magnify objects in steps. These microscopes can magnify objects up to about 1500 times. As the observations of organisms viewed under a microscope expanded, scientists began to draw conclusions about the organization of living matter. With the **microscope** established as a valid scientific tool, scientists had to learn the size relationship of magnified objects to their true size.

The cell theory

Robert Hooke was an English scientist who lived at the same time as van Leeuwenhoek. Hooke used a **compound light microscope** to study cork, the dead cells of oak bark. In cork, Hooke observed small geometric shapes, like those shown in Figure 7.1. Hooke gave these box-shaped structures the name cells because they reminded him of the small rooms monks lived in at a monastery. Cells are the basic units of all living things.

Several scientists extended Hooke’s observations and drew some important conclusions. In the 1830s, the German scientist Matthias Schleiden observed a variety of plants and concluded that all plants are composed of cells. Another German scientist, Theodor Schwann, made similar observations on animals. The observations and conclusions of these scientists are summarized as the **cell theory**, one of the fundamental ideas of modern biology.

The **cell theory** is made up of three main ideas:

1. All organisms are composed of one or more cells. An organism may be a single cell, such as the organisms van Leeuwenhoek saw in water. Others, like the plants and animals with which you are most familiar, are multicellular, or made up of many cells.

2. The cell is the basic unit of structure and organization of organisms. Although organisms such as humans, dogs, and trees can become very large and complex, the cell remains the simplest, most basic component of any organism.

3. All cells come from preexisting cells. Before the **cell theory**, no one knew how cells were formed, where they came from, or what determined the type of cell they became. The **cell theory** states that a cell divides to form two identical cells.

Electron microscopes

The microscopes we have discussed so far use a beam of light and can magnify an object up to about 1500 times its actual size. Although **light microscopes** continue to be valuable tools, scientists knew that another world, which they could not yet see, existed within a cell. In the 1930s and 1940s, a new type of microscope, the **electron microscope**, was developed. This **microscope** uses a beam of electrons instead of light to magnify structures up to 500 000 times their actual size, allowing scientists to see structures within a cell. Because the electrons can collide with air particles and scatter, specimens must be examined in a vacuum.

There are two basic types of **electron microscopes**. Scientists commonly use the **scanning electron microscope (SEM)** to scan the surfaces of cells to learn their three-dimensional shape. The **transmission electron microscope (TEM)** allows scientists to study the structures contained within a cell.

New types of microscopes and new techniques are continually being designed. For example, the **scanning tunneling microscope (STM)** uses the flow of electrons to create computer images of atoms on the surface of a molecule. New techniques using the **light microscope** have increased the information scientists can gain with this basic tool. Most of these new techniques seek to add contrast to structures within the cells, such as adding dyes that stain some parts of a cell, but not others.

Two Basic Cell Types

With the development of better microscopes, scientists observed that all cells contain small, specialized structures called organelles. Many, but not all, organelles are surrounded by membranes. Each organelle has a specific function in the cell.

Cells can be divided into two broad groups: those that contain membrane-bound organelles and those that do not. Cells that do not contain any membrane-bound organelles are called prokaryotic cells. Most unicellular organisms, such as bacteria, do not have membrane-bound organelles and are therefore called prokaryotes.

Cells of the other type, those containing membrane-bound organelles, are called eukaryotic cells. Most of the multicellular organisms we know are made up of eukaryotic cells and are therefore called eukaryotes. It is important to note, however, that some eukaryotes, such as amoebas, or some algae and yeast, are unicellular organisms.

The Plasma Membrane

Maintaining a Balance

You are comfortable in your house largely because the thermostat maintains the temperature within a limited range regardless of what’s happening outside. Similarly, all living cells must maintain a balance regardless of internal and external conditions. Survival depends on the cell’s ability to maintain the proper conditions within itself.

Why cells must control materials

Your cells need nutrients such as glucose, amino acids, and lipids to function. It is the job of the plasma membrane, the flexible boundary between the cell and its environment, to allow a steady supply of these nutrients to come into the cell no matter what the external conditions are. However, too much of any of these nutrients or other substances, especially ions, can be harmful to the cell. If levels become too high, the excess is removed through the plasma membrane. Waste and other products also leave the cell through the plasma membrane. Recall that this process of maintaining balance in the cell’s environment is called homeostasis.

How does the plasma membrane maintain homeostasis? One mechanism is selective permeability, a process in which a membrane allows some molecules to pass through while keeping others out. In your home, a screen in a window can perform selective permeability in a similar way. When you open the window, the screen lets fresh air in and keeps most insects out.

Some molecules, such as water, freely enter the cell through the plasma membrane, as shown in Figure 7.3. Other particles, such as sodium and calcium ions, must be allowed into the cell only at certain times, in certain amounts, and through certain channels. The plasma membrane must be selective in allowing these ions to enter. Use the Problem-Solving Lab here to analyze the plasma membrane of a yeast cell.

Structure of the Plasma Membrane

Now that you understand the basic function of the plasma membrane, you can study its structure. Recall from Chapter 6 that lipids are large molecules that are composed of glycerol and three fatty acids. If a phophate group replaces a fatty acid, a phospholipid is formed. Thus, a phospholipid has a glycerol backbone, two fatty acid chains, and a phosphate group. The plasma membrane is composed of a phospholipid bilayer, which has two layers of phospholipids back-to-back.

The phospholipid bilayer

The phosphate group is critical for the formation and function of the plasma membrane. The two fatty acid tails of the phospholipids are nonpolar, whereas the head of the phospholipid molecule containing the phosphate group is polar.

Water is a key component of living organisms, both inside and outside the cell. The polar phosphate group allows the cell membrane to interact with its watery environment because, as you recall, water is also polar. The fatty acid tails, on the other hand, avoid water. The two layers of phospholipid molecules make a sandwich with the fatty acid tails forming the interior of the membrane and the phospholipid heads facing the watery environments found inside and outside the cell. Figure 7.4 illustrates phospholipids and their place within the structure of the plasma membrane. When many phospholipid molecules come together in this manner, a barrier is created that is water-soluble at its outer surfaces and water-insoluble in the middle. Water-soluble molecules will not easily move through the membrane because they are stopped by this water-insoluble layer.

The model of the plasma membrane is called the **fluid mosaic model**. It is fluid because the phospholipids move within the membrane just as water molecules move with the currents in a lake. At the same time, proteins in the membrane also move among the phospholipids like boats with their decks above water and hulls below water. These proteins create a “mosaic,” or pattern, on the membrane surface.

Other components of the plasma membrane

Cholesterol, shown in Figure 7.5, is also found in the **plasma membrane** where it helps to stabilize the phospholipids by preventing their fatty acid tails from sticking together. Cholesterol is a common topic in health issues today because high levels are associated with reduced blood flow in blood vessels. Yet, for all the emphasis on cholesterol-free foods, it is important to recognize that cholesterol plays a critical role in the stability of the plasma membrane and is therefore a necessary part of your diet. You’ve learned that proteins are found within the lipid membrane. Proteins that span the entire membrane help form the selectively permeable membrane that regulates which molecules enter and which molecules leave a cell. These proteins are called transport proteins. Transport proteins move needed substances or waste materials through the plasma membrane. Other proteins and carbohydrates that stick out from the cell surface help cells to identify chemical signals and each other. As you will discover later, these characteristics are important in protecting your cells from infection. Proteins at the inner surface of a plasma membrane play an important role in attaching the plasma membrane to the cell’s internal support structure, giving the cell its flexibility.

Eukaryotic Cell Structure

Cellular Boundaries

When a group works together, someone on the team decides what resources are necessary for the project and provides these resources. In the cell, the plasma membrane, shown in Figure 7.6, performs this task by acting as a selectively permeable membrane. The **fluid mosaic model** describes the plasma membrane as a flexible boundary of a cell. However, plant cells, fungi, bacteria, and some protists have an additional boundary, the cell wall. The cell wall is a fairly rigid structure located outside the plasma membrane that provides additional support and protection.

The cell wall

The cell wall forms an inflexible barrier that protects the cell and gives it support. Figure 7.7 shows a plant cell wall composed of a carbohydrate called cellulose. The cellulose forms a thick, tough mesh of fibers. This fibrous cell wall is very porous and allows molecules to enter. Unlike the plasma membrane, it does not select which molecules can enter into the cell.

The Nucleus and Cell Control

Just as every team needs a leader to direct activity, so the cell needs a leader to give directions. The nucleus is the leader of the eukaryotic cell because it contains the directions to make proteins. Every part of the cell depends on proteins, so by containing the blueprint to make proteins, the nucleus controls the activity of the organelles.

The master set of directions for making proteins is contained in **chromatin**, which are strands of the genetic material, DNA. When a cell divides, the chromatin condenses to form chromosomes. Within the nucleus is a prominent organelle called the nucleolus, which makes ribosomes. Ribosomes are the sites where the cell produces proteins according to the directions of DNA. Unlike other organelles, ribosomes are not bound by a membrane. They are simple structures made of RNA and protein.

For proteins to be made, ribosomes must leave the nucleus and enter the cytoplasm, and the blueprints contained in **DNA** must be translated into RNA and sent to the cytoplasm. Cytoplasm is the clear, gelatinous fluid inside a cell. Ribosomes and translated RNA are transported to the cytoplasm through the nuclear envelope—a structure that separates the nucleus from the cytoplasm, as shown in Figure 7.8. The nuclear envelope is a double membrane made up of two phospholipid bilayers containing small nuclear pores for substances to pass through. Ribosomes and translated RNA pass into the cytoplasm through these pores in the nuclear envelope.

Assembly, Transport, and Storage

You have begun to follow the trail of protein production as directed by the cell manager—the nucleus. But what happens to the copy of the blueprints for proteins once it passes from the nucleus into the cytoplasm?

Organelles for assembly and transport of proteins

The cytoplasm suspends the cell’s organelles. One particular organelle in a eukaryotic cell, the **endoplasmic reticulum** (ER), is the site of cellular chemical reactions. Shown in Figure 7.9, the ER is arranged in a series of highly folded membranes in the **cytoplasm**. Its folds are like the folds of an accordion. If you spread the accordion out, it would take up tremendous space. By pleating and folding, the accordion fits into a compact unit. Similarly, a large amount of folded ER is available to do work in a small space.

Ribosomes in the cytoplasm are attached to the surface of the endoplasmic reticulum, called **rough endoplasmic reticulum**, where they carry out the function of protein synthesis.

The ribosome’s job is to make proteins. Each protein made in the **rough ER** has a particular function; it may become a protein that forms a part of the plasma membrane, a protein that is released from the cell, or a protein transported to other organelles. Ribosomes can also be found floating freely in the cytoplasm. They make proteins that perform tasks within the cytoplasm itself.

Areas of the ER that are not studded with ribosomes are known as smooth endoplasmic reticulum. The smooth ER is involved in numerous biochemical activities, including the production and storage of lipids.

After proteins are made, they are transferred to another organelle called the Golgi apparatus. The Golgi apparatus, as shown in Figure 7.10, is a flattened stack of tubular membranes that modifies the proteins. The Golgi apparatus sorts proteins into packages and packs them into membrane-bound structures, called vesicles, to be sent to the appropriate destination, like mail being sorted at the post office.

Vacuoles and storage

Now let’s look at some of the other members of the cell team important for the cell’s functioning. Cells have membrane-bound compartments, called vacuoles, for temporary storage of materials. A vacuole, like that in Figure 7.11A, is a sac used to store food, enzymes, and other materials needed by a cell. Some vacuoles store waste products. Animal cells usually do not contain vacuoles. If they do, the vacuoles are much smaller, as shown in Figure 7.11B.

Lysosomes and recycling

Did anyone ever ask you to take out the trash? Is that action part of a team effort? In a cell, it is. Lysosomes are organelles that contain digestive enzymes. They digest excess or worn out organelles, food particles, and engulfed viruses or bacteria. The membrane surrounding a lysosome prevents the digestive enzymes inside from destroying the cell. Lysosomes can fuse with vacuoles and dispense their enzymes into the vacuole, digesting its contents.

For example, when an amoeba engulfs food and encloses it in a vacuole, a lysosome fuses with the vacuole and releases its enzymes, which digest the food. Sometimes, lysosomes digest the cells that contain them. When a tadpole develops into a frog, lysosomes within the cells of the tadpole’s tail cause its digestion. The molecules released are used to build different cells, perhaps in the legs of the adult frog.

Energy Transformers

After learning about cell parts and what they do, it’s easy to imagine that each of these cell team members requires a lot of energy. Protein production, modification, transportation, digestion—all require energy. Two other organelles, chloroplasts and mitochondria, provide that energy.

Chloroplasts and energy

When you walk through a field or pick a vegetable from the garden, you may not think of the plants as energy generators. In fact, that is exactly what you see. Located in the **cells** of green plants and some protists, chloroplasts are the heart of the generator. Chloroplasts are cell organelles that capture light energy and convert it to chemical energy.

A chloroplast, like a nucleus, has a double membrane. The diagram and TEM photomicrograph of a chloroplast in Figure 7.12 shows an outer membrane and a folded inner membrane system. It is within these inner thylakoid membranes that the energy from sunlight is trapped. These inner membranes are arranged in stacks of membranous sacs called grana, which resemble stacks of coins. The fluid that surrounds the stacks of grana is called stroma.

The chloroplast belongs to a group of plant organelles called plastids, which are used for storage. Some plastids store starches or lipids, whereas others contain pigments, molecules that give color. Plastids are named according to their color or the pigment they contain. Chloroplasts contain the green pigment chlorophyll. Chlorophyll traps light energy and gives leaves and stems their green color.

Mitochondria and energy

The chemical energy generated by chloroplasts is stored in the **bonds of sugar molecules** until they are broken down by mitochondria, shown in Figure 7.13. Mitochondria are membrane-bound organelles in **plant and animal cells** that transform energy for the cell. This energy is then stored in the bonds of other molecules that cell organelles can access easily and quickly when energy is needed.

A mitochondrion has an outer membrane and a highly folded inner membrane. As with the endoplasmic reticulum and chloroplasts, the folds of the inner membrane provide a large surface area that fits in a small space. Energy-storing molecules are produced on the inner folds. Mitochondria occur in varying numbers depending on the function of the cell. For example, liver cells may have up to 2000 mitochondria.

Although the process by which energy is transformed and used in the cells is a technical concept that you will learn in a later chapter, the Connection to Literature at the end of this chapter explains how cellular processes can also be inspiring.

Organelles for Support and Locomotion

Scientists once thought that cell organelles just floated in a sea of cytoplasm. More recently, cell biologists have discovered that cells have a support structure called the cytoskeleton within the cytoplasm.

The cytoskeleton

The cytoskeleton forms a framework for the cell, like the skeleton that forms the framework for your body. However, unlike your bones, the cytoskeleton is a constantly changing structure. It can be dismantled in one place and reassembled somewhere else in the cell, changing the cell’s shape.

The cytoskeleton is a network of tiny rods and filaments. Microtubules are thin, hollow cylinders made of protein. Microfilaments are smaller, solid protein fibers. Together, they act as a sort of scaffold to maintain the shape of the cell in the same way that poles maintain the shape of a tent. They also anchor and support many organelles and provide a sort of highway system through which materials move within the cell.

Centrioles

Centrioles are organelles found in the **cells** of animals and most protists. They occur in pairs and are made up of microtubules. Centrioles play an important role in cell division.

Cilia and flagella

Some cell surfaces have cilia and flagella, which are organelles made of microtubules that aid the cell in locomotion or feeding. Cilia and flagella can be distinguished by their structure and by the nature of their action. Cilia are short, numerous projections that look like hairs.

Their motion is similar to that of oars in a rowboat. Flagella are longer projections that move with a whip-like motion. A cell usually has only one or two flagella. In **unicellular organisms**, cilia and flagella are the major means of locomotion.

Remember that prokaryotic cells lack the membrane-bound organelles that are found in eukaryotic cells. Table 7.1 shows a side-by-side comparison of eukaryotic and prokaryotic cells, their cell parts, and what those parts do. Figure 7.14 summarizes the structure of eukaryotic plant and animal cells.

Cellular Transport and the Cell Cycle

Cellular Transport

Osmosis: Diffusion of Water

Although the plasma membrane of a cell can act as a dam or pump for water-soluble molecules that cannot pass freely through the membrane, it does not limit the diffusion of water. Recall that diffusion is the movement of particles from an area of higher concentration to an area of lower concentration. In a cell, water always moves to reach an equal concentration on both sides of the membrane. The diffusion of water across a selectively permeable membrane is called osmosis. Regulating the water flow through the plasma membrane is an important factor in maintaining homeostasis within the cell.

What controls osmosis?

If you add sugar to water, the water becomes sweeter as you add more sugar. If a strong sugar solution and a weak sugar solution are placed in direct contact, water molecules diffuse in one direction and sugar molecules diffuse in the other direction until all molecules are evenly distributed throughout.

If the two solutions are separated by selectively permeable membrane increases.

As the pressure increases inside animal cells, the plasma membrane swells, like the red blood cells shown in Figure 8.3B. If the solution is extremely hypotonic, the plasma membrane where the water concentration is lower. The water continues to diffuse until it is in equal concentration on both sides of the membrane, as shown in Figure 8.1. Therefore, we know that unequal distribution of particles, called a concentration gradient, is one factor that controls osmosis.

Cells in an isotonic solution

It is important to understand how osmosis affects cells. Most cells, whether in multicellular or unicellular organisms, are subject to osmosis because they are surrounded by water solutions. In an isotonic solution, the concentration of dissolved substances in the solution is the same as the concentration of dissolved substances inside the cell. Likewise, the concentration of water in the solution is the same as the concentration of water inside the cell.

Cells in an isotonic solution do experience osmosis, but because water diffuses into and out of the cells at the same rate, the cells retain their normal shape, as shown in Figure 8.2.

Cells in a hypotonic solution

In the hypotonic solution in Figure 8.3A, the concentration of dissolved substances is lower in the solution outside the cell than the concentration inside the cell. Therefore, there is more water outside the cell than inside. Cells in a hypotonic solution experience osmosis. Water moves through the plasma membrane into the cell. The cell swells and its internal pressure increases.

As the pressure increases inside **animal cells**, the plasma membrane swells, like the red blood cells shown in Figure 8.3B. If the solution is extremely hypotonic, the plasma membrane may be unable to withstand this pressure and may burst.

Because plant cells contain a rigid cell wall that supports the cell, they do not burst when in a hypotonic solution. As the pressure increases inside the **cell**, the plasma membrane is pressed against the **cell wall**, as shown in Figure 8.3C. Instead of bursting, the plant cell becomes more firm. Grocers keep produce looking fresh by misting the fruits and vegetables with water.

Cells in a hypertonic solution

In a hypertonic solution, the concentration of dissolved substances outside the cell is higher than the concentration inside the cell. Cells in a hypertonic solution experience osmosis that causes water to flow out.

Animal cells in a hypertonic solution shrivel because of decreased pressure in the cells.

Plant cells in a hypertonic environment lose water, mainly from the central vacuole. The plasma membrane and cytoplasm shrink away from the cell wall, as shown in Figure 8.4C. Loss of water in a plant cell results in a drop in pressure and explains why plants wilt.

Passive Transport

Some molecules, like water, can pass through the plasma membrane by simple diffusion, as shown in Figure 8.5A. The cell uses no energy to move these particles; therefore, this movement of particles across the membrane is classified as passive transport.

Passive transport by proteins

Recall that transport proteins help substances move through the plasma membrane. Passive transport of materials across the membrane using transport proteins is called facilitated diffusion.

Some transport proteins, called channel proteins, form channels that allow specific molecules to flow through, as illustrated in Figure 8.5B.

The movement is with the concentration gradient, and requires no energy input from the cell.

Carrier proteins are another type of transport protein. Carrier proteins change shape to allow a substance to pass through the plasma membrane, as shown in Figure 8.5C. In facilitated diffusion by carrier protein, the movement is with the concentration gradient and requires no energy input from the cell.

Active Transport

A cell can move particles from a region of lower concentration to a region of higher concentration, but it must expend energy to counteract the force of diffusion that is moving the particles in the opposite direction. Movement of materials through a membrane against a concentration gradient is called active transport and requires energy from the cell.

How active transport occurs

In active transport, a transport protein called a carrier protein first binds with a particle of the substance to be transported. In general, each type of carrier protein has a shape that fits a specific molecule or ion. When the proper molecule binds with the protein, chemical energy allows the cell to change the shape of the carrier protein so that the particle to be moved is released on the other side of the membrane, something like the opening of a door. Once the particle is released, the protein’s original shape is restored, as illustrated in Figure 8.6. Active transport allows particle movement into or out of a cell against a concentration gradient. Transport of substances across the cell membrane is required for cells to maintain homeostasis. The types of transport are summarized in Table 8.1.

Transport of Large Particles

Some cells can take in large molecules, groups of molecules, or even whole cells. Endocytosis is a process by which a cell surrounds and takes in material from its environment as shown in Figure 8.7. This material does not pass directly through the membrane. Instead, it is engulfed and enclosed by a portion of the cell’s plasma membrane. That portion of the membrane then breaks away, and the resulting vacuole with its contents moves to the inside of the cell.

Figure 8.7 also shows the reverse process of endocytosis, called exocytosis. Exocytosis is the expulsion or secretion of materials from a cell. Cells use exocytosis to expel wastes. They also use this method to secrete substances, such as hormones produced by the cell. Because endocytosis and exocytosis both move masses of material, they both require energy.

With the various mechanisms the cell uses to transport materials in and out, cells must also have mechanisms to regulate size and growth.

Cell Growth and Reproduction

Cell Size Limitations

The cells that make up a multicellular organism come in a wide variety of sizes and shapes. Some cells, such as red blood cells, measure only 8 µm (micrometers) in diameter. Other cells, such as nerve cells in large animals, can reach lengths of up to 1 m but have small diameters. The cell with the largest diameter is the yolk of an ostrich egg measuring 8 cm. Most living cells, however, are between 2 and 200 µm in diameter. Considering this wide range of cell sizes, why then can’t most organisms be just one giant cell?

Diffusion limits cell size

You know that the plasma membrane allows nutrients to enter the cell and wastes to leave. Within the cell, nutrients and wastes move by diffusion.

Although diffusion is a fast and efficient process over short distances, it becomes slow and inefficient as the distances become larger. Imagine a mitochondrion at the center of a cell with a diameter of 20 cm. It would have to wait months before receiving molecules entering the cell. Because of the slow rate of diffusion, organisms can’t be just one giant-sized cell.

DNA limits cell size

You have learned that the nucleus contains blueprints for the cell’s proteins. Proteins are used throughout the cell by almost all organelles to perform critical cell functions. But there is a limit to how quickly the blueprints for these proteins can be copied in the **nucleus** and made into proteins in the **cytoplasm**. The cell cannot survive unless there is enough DNA to support the protein needs of the cell.

What happens in larger cells where an increased amount of cytoplasm requires increased supplies of enzymes? In many large cells, such as the giant amoeba Pelomyxa shown in Figure 8.8, more than one nucleus is present. Large amounts of DNA in many nuclei ensure that cell activities are carried out quickly and efficiently.

Surface area-to-volume ratio

Another size-limiting factor is the cell’s surface area-to-volume ratio. As a cell’s size increases, its volume increases much faster than its surface area. Picture a cube-shaped cell like those shown in Figure 8.9. The smallest cell has 1 mm sides, a surface area of 6 mm2, and a volume of 1 mm3. If the side of the cell is doubled to 2 mm, the surface area will increase fourfold to 6 × 2 × 2 = 24 mm2. Observe what happens to the volume; it increases eightfold to 8 mm3.

What does this mean for cells? How does the surface area-to-volume ratio affect cell function? If cell size doubled, the cell would require eight times more nutrients and would have eight times more waste to excrete.

The surface area, however, would increase by a factor of only four. Thus, the plasma membrane would not have enough surface area through which oxygen, nutrients, and wastes could diffuse. The cell would either starve to death or be poisoned from the buildup of waste products.

Because cell size can have dramatic and negative effects on a cell, cells must have some method of maintaining optimum size. In fact, cells divide 4 mm before they become too large to function properly. Cell division accomplishes other purposes, too, as you will read next.

Cell Reproduction

Recall that the **cell theory** states that all cells come from preexisting cells. Cell division is the process by which new cells are produced from one cell. Cell division results in two cells that are identical to the original, parent cell. Right now, as you are reading this page, many of the cells in your body are growing, dividing, and dying. Old cells on the soles of your feet and on the palms of your hands are being shed and replaced, cuts and bruises are healing, and your intestines are producing millions of new cells each second. New cells are produced as tadpoles become frogs, and as an ivy vine grows and wraps around a garden trellis. All organisms grow and change; worn-out tissues are repaired or are replaced by newly produced cells.

The discovery of chromosomes

Early biologists observed that just before cell division, several short, stringy structures suddenly appeared in the nucleus. Scientists also noticed that these structures seemed to vanish soon after division of a cell. These structures, which contain DNA and become darkly colored when stained, are called chromosomes.

Eventually, scientists learned that chromosomes are the carriers of the genetic material that is copied and passed from generation to generation of cells. This genetic material is crucial to the identity of the cell. Accurate transmission of chromosomes during cell division is critical.

The structure of eukaryotic chromosomes

For most of a cell’s lifetime, chromosomes exist as chromatin, long strands of DNA wrapped around proteins called histones. Under an **electron microscope**, chromatin looks like beads on a string. Each bead is a group of histones called a nucleosome. Before a cell can divide, the long strands of chromatin must be reorganized, just as you would coil a long strand of rope before storing it. As the nucleus begins to divide, chromosomes take on a different structure in which the chromatin becomes tightly packed. Look at Figure 8.10 for more information on chromosome structure.

The Cell Cycle

Fall follows summer, night follows day, and low tide follows high tide. Many events in nature follow a recurring, cyclical pattern. Living organisms are no exception. One cycle common spent in the growth period known as **interphase**. During **interphase**, a cell grows in size and carries on metabolism. Also during this period, chromosomes are duplicated in preparation for the period of division.

Following interphase, a cell enters its period of nuclear division called mitosis. Mitosis is the process by which two daughter cells are formed, each containing a complete set of chromosomes. Interphase and mitosis make up the bulk of the **cell cycle**. Following mitosis, the cytoplasm divides, separating the two daughter cells.

Interphase:

A Busy Time

Interphase, the busiest phase of the **cell cycle**, is divided into three parts as shown in Figure 8.11. During the first part, the cell grows and protein production is high. In the next part of interphase, the cell copies its chromosomes. DNA synthesis does not occur all through **interphase** but is confined to this specific time. After the chromosomes have been duplicated, the cell enters another shorter growth period in which mitochondria and other organelles are manufactured and cell parts needed for cell division are assembled. Following this activity, interphase ends and mitosis begins.

The Phases of Mitosis

Cells undergo mitosis as they approach the maximum cell size at which the nucleus can provide blueprints for proteins, and the plasma membrane can efficiently transport nutrients and wastes into and out of the cell.

Although cell division is a continuous process, biologists recognize four merging into the next. The four phases of mitosis are **prophase, metaphase, anaphase, and telophase**. Refer to Figure 8.13 to help you understand the process as you read about mitosis.

Prophase: The first phase of mitosis

During **prophase**, the first and longest phase of mitosis, the long, stringy chromatin coils up into visible chromosomes. As you can see in Figure 8.12, each duplicated chromosome is made up of two halves. The two halves of the doubled structure are called sister chromatids.

Sister chromatids and the DNA they contain are exact copies of each other and are formed when DNA is copied during **interphase**. Sister chromatids are held together by a structure called a centromere, which plays a role in chromosome movement during mitosis. By their characteristic location, centromeres also help scientists identify and study chromosomes.

As prophase continues, the nucleus begins to disappear as the nuclear envelope and the nucleolus disintegrate.

By late **prophase**, these structures are completely absent. In **animal cells**, two important pairs of structures, the centrioles, begin to migrate to opposite ends of the cell. Centrioles are small, dark, cylindrical structures that are made of microtubules and are located just outside the **nucleus**, as shown in Figure 8.14. Centrioles play a role in chromatid separation.

As the pairs of centrioles move to opposite ends of the cell, another important structure, called the spindle, begins to form between them. The spindle is a football-shaped, cagelike structure consisting of thin fibers made of microtubules. In **plant cells**, the spindle forms without centrioles. The spindle fibers play a vital role in the separation of sister chromatids during mitosis.

Metaphase: The second stage of mitosis

During **metaphase**, the short second phase of mitosis, the doubled chromosomes become attached to the spindle fibers by their centromeres. The chromosomes are pulled by the spindle fibers and begin to line up on the midline, or equator, of the spindle. Each sister chromatid is attached to its own spindle fiber. One sister chromatid’s spindle fiber extends to one pole, and the other extends to the opposite pole. This arrangement is important because it ensures that each new cell receives an identical and complete set of chromosomes.

Anaphase: The third phase of mitosis

The separation of sister chromatids marks the beginning of anaphase, the third phase of mitosis. During **anaphase**, the centromeres split apart and chromatid pairs from each chromosome separate from each other. The chromatids are pulled apart by the shortening of the microtubules in the spindle fibers.

Telophase: The fourth phase of mitosis

The final phase of mitosis is telophase. **Telophase** begins as the chromatids reach the opposite poles of the cell. During **telophase**, many of the changes that occurred during prophase are reversed as the new cells prepare for their own independent existence. The chromosomes, which had been tightly coiled since the end of **prophase**, now unwind so they can begin to direct the metabolic activities of the new cells. The spindle begins to break down, the nucleolus reappears, and a new nuclear envelope forms around each set of chromosomes. Finally, a new double membrane begins to form between the two new nuclei.

Cytokinesis

Following telophase, the cell’s cytoplasm divides in a process called cytokinesis. Cytokinesis differs between plants and animals. Toward the end of telophase in animal cells, the plasma membrane pinches in along the equator as shown in Figure 8.15. As the **cell cycle** proceeds, the two new cells are separated.

Plant cells have a rigid cell wall, so the plasma membrane does not pinch in. Rather, a structure known as the cell plate is laid down across the cell’s equator. A cell membrane forms around each cell, and new cell walls form on each side of the cell plate until separation is complete.

Results of Mitosis

Mitosis is a process that guarantees genetic continuity, resulting in the production of two new cells with chromosome sets that are identical to those of the parent cell. These new daughter cells will carry out the same cellular processes and functions as those of the parent cell and will grow and divide just as the parent cell did.

When mitosis is complete, unicellular organisms remain as single cells— the organism simply multiplied. In multicellular organisms, cell growth and reproduction result in groups of cells that work together as tissue to perform a specific function. Tissues organize in various combinations to form organs that perform more complex roles within the organism. For example, cells make up muscle tissue, then muscle tissue works with other tissues in the organ called the stomach to mix up food. Multiple organs that work together form an organ system. The stomach is one organ in the digestive system, which functions to break up and digest food.

All organ systems work together for the survival of the organism, whether the organism is a fly or a human. Figure 8.16 shows an example of cell specialization and organization for a complex organism. In addition to its digestive system, the panther has a number of other organ systems that have developed through cell specialization. It is important to remember that no matter how complex the organ system or organism becomes, the cell is still the most basic unit of that organization.

Control of the Cell Cycle

Normal Control of the Cell Cycle

Why do some types of cells divide rapidly, while others divide slowly? What tells a cell when it is time to leave one part of the cell cycle and begin the next?

Proteins and enzymes control the cell cycle

The **cell cycle** is controlled by proteins called cyclins and a set of enzymes that attach to the cyclin and become activated. The interaction of these molecules, based on conditions both in the cell’s environment and inside the cell, control the **cell cycle**. Occasionally, cells lose control of the **cell cycle**. This uncontrolled dividing of cells can result from the failure to produce certain enzymes, the overproduction of enzymes, or the production of other enzymes at the wrong time. Cancer is a malignant growth resulting from uncontrolled cell division. This loss of control may be caused by environmental factors or by changes in enzyme production.

Enzyme production is directed by genes located on the chromosomes. A gene is a segment of DNA that controls the production of a protein.

Many studies point to the portion of interphase just before DNA replication as being a key control period in the **cell cycle**. Scientists have identified several enzymes that trigger DNA replication.

Cancer: A Mistake in the Cell Cycle

Currently, scientists consider cancer to be a result of changes in one or more of the genes that produce substances that are involved in controlling the cell cycle. These changes are expressed as cancer when something prompts the damaged genes into action. Cancerous cells form masses of tissue called tumors that deprive normal cells of nutrients. In later stages, cancer cells enter the circulatory system and spread throughout the body, a process called metastasis, forming new tumors that disrupt the function of organs, organ systems, and ultimately, the organism.

Cancer is the second leading cause of death in the United States, exceeded only by heart disease. Cancer can affect any tissue in the body. In the United States, lung, colon, breast, and prostate cancers are the most prevalent types.

The causes of cancer

The causes of cancer are difficult to pinpoint because both genetic and environmental factors are involved. The environmental influences of cancer become obvious when you consider that people in different countries develop different types of cancers at different rates. For example, the rate of breast cancer is relatively high in the United States, but relatively low in Japan. Similarly, stomach cancer is common in Japan, but rare in the United States.

Other environmental factors, such as cigarette smoke, air and water pollution, and exposure to ultraviolet radiation from the sun, are all known to damage the genes that control the **cell cycle**. Cancer may also be caused by viral infections that damage the genes.

Cancer prevention

From recent and ongoing investigations, scientists have established a clear link between a healthy lifestyle and the incidence of cancer.

Physicians and dietary experts agree that diets low in fat and high in fiber content can reduce the risk of many kinds of cancer. For example, diets high in fat have been linked to increased risk for colon, breast, and prostate cancers, among others. People who consume only a minimal amount of fat reduce the potential risk for these and other cancers and may also maintain a healthy body weight more easily. In addition, recent studies suggest that diets high in fiber are associated with reduced risk for cancer, especially colon cancer. Fruits, vegetables, and grain products are excellent dietary options because of their fiber content and because they are naturally low in fat. The foods displayed in Figure 8.17 illustrate some of the choices that are associated with cancer prevention.

Vitamins and minerals may also help prevent cancer. Key in this category are carotenoids, vitamins A, C, and E, and calcium. Carotenoids are found in foods such as yellow and orange vegetables and green leafy vegetables. Citrus fruits are a great source of vitamin C, and many dairy products are rich in calcium.

In addition to diet, other healthy choices such as daily exercise and not using tobacco also are known to reduce the risk of cancer.

Energy in a Cell

The Need for Energy

Cell Energy

Energy is essential to life. All living organisms must be able to obtain energy from the environment in which they live. Plants and other green organisms are able to trap the light energy in sunlight and store it in the bonds of certain molecules for later use. Other organisms, such as the panda shown in Figure 9.1, cannot use sunlight directly. Instead, they eat green plants. In that way, they obtain the energy stored in plants.

Work and the need for energy

You’ve learned about several cell processes that require energy. Active transport, cell division, movement of flagella or cilia, and the production, transport, and storage of proteins are some examples. You can probably come up with other examples of biological work, such as muscles contracting during exercise, your heart pumping and your brain controlling your entire body. This work cannot be done without energy.

This energy is stored in the chemical bonds of the molecule and can be used quickly and easily by the cell.

The name of this energy molecule is adenosine triphosphate, or ATP for short. ATP is composed of an adenosine molecule with three phosphate groups attached. Recall that phosphate groups are charged particles, and remember that particles with the same charge do not like being too close to each other.

Forming and Breaking Down ATP

The charged phosphate groups act like the positive poles of two magnets. If like poles of a magnet are placed next to each other, it is difficult to force the magnets together. Likewise, bonding three phosphate groups to form adenosine triphosphate requires considerable energy. When only one phosphate group bonds, a small amount of energy is required and the chemical bond does not store much energy. This molecule is called adenosine monophosphate (AMP). When a second phosphate group is added, more energy is required to force the two groups together. This molecule is called adenosine diphosphate, or ADP. An even greater amount of energy is required to force a third charged phosphate group close enough to the other two to form a bond. When this bond is broken, energy is released.

When you finish strenuous physical exercise, such as running cross country, your body needs a quick source of energy, so you may eat a granola bar. On a cellular level, there is a molecule in your cells that is a quick source of energy for any organelle in the cell that needs it.

The energy of ATP becomes available to a cell when the molecule is broken down. In other words, when the chemical bond between the second and third phosphate groups in ATP is broken, energy is released and the resulting molecule is ADP. At this point, ADP can form ATP again by bonding with another phosphate group. This process creates a renewable cycle of ATP formation and breakdown. Figure 9.2A illustrates the chemical reactions that are involved in the cycle.

The formation/breakdown recycling activity is important because it relieves the cell of having to store all of the ATP it needs. As long as phosphate groups are available, the cell can make more ATP. Another benefit of the formation/breakdown cycle is that ADP also can be used as an energy source. Although most cell functions require the amount of energy in ATP, some cell functions do not require as much energy and can use the energy stored in ADP.

How cells tap into the energy stored in ATP

When ATP is broken down and the energy is released, as shown in Figure 9.2B, the energy must be captured and used efficiently by cells. Otherwise, it is wasted. ATP is a small molecule. Many proteins have a specific site where ATP can bind. Then, when the phosphate bond is broken and the energy released, the cell can use the energy for activities such as making a protein or transporting molecules through the plasma membrane. This cellular process is similar to the way energy in batteries is used by a radio. Batteries sitting on a table are of little use if the energy stored within the batteries cannot be accessed. When the batteries are snapped into the holder in the radio, the radio then has access to the stored energy and can use it. When the energy in the batteries has been used, the batteries can be taken out, recharged, and replaced in the holder. In a similar fashion in a cell, when ATP has been broken down to ADP, the ADP is released from the binding site in the protein and the binding site may then be filled by another ATP molecule.

Uses of Cell Energy

You can probably think of hundreds of physical activities that require energy, but energy is equally important at the cellular level.

Making new molecules is one way that cells use energy. Some of these molecules are enzymes. Other molecules build membranes and cell organelles. Cells use energy to maintain homeostasis. Kidneys use energy to move molecules and ions in order to eliminate waste substances while keeping needed substances in the bloodstream. Figure 9.3 shows several ways that cells use energy.

Photosynthesis: Trapping the Sun’s Energy

Trapping Energy from Sunlight

To use the energy in sunlight, the cells of green organisms must trap light energy and store it in a manner that is readily usable by cell organelles—in the chemical bonds of ATP. However, light energy is not available 24 hours a day, so the cell must also store some of the energy for use during the dark hours. The process that uses the sun’s energy to make simple sugars is called **photosynthesis**. These simple sugars are then converted into complex carbohydrates, such as starches, which store energy.

**Photosynthesis** happens in two phases. The light-dependent reactions convert light energy into chemical energy. The molecules of ATP produced in the light-dependent reactions are then used to fuel the light-independent reactions that produce simple sugars. The general equation for photosynthesis is written as follows:

6CO2 + 6H2O  C6H12O6 + 6O2

The BioLab at the end of this chapter can be performed to study what factors influence the rate of photosynthesis.

The chloroplast and pigments

Recall that the chloroplast is the cell organelle where **photosynthesis** occurs. It is in the **membranes of the thylakoid discs** in chloroplasts that the light-dependent reactions take place.

To trap the energy in the sun’s light, the thylakoid membranes contain pigments, molecules that absorb specific wavelengths of sunlight. Pigments are arranged within the thylakoid membranes in clusters known as photosystems. Although a photosystem contains several kinds of pigments, the most common is chlorophyll. Chlorophyll absorbs most wavelengths of light except green. Because chlorophyll cannot absorb this wavelength, it is reflected, giving leaves a green appearance. In the fall, trees stop producing chlorophyll in their leaves. Other pigments become visible, giving leaves like those in Figure 9.4 a wide variety of colors. The MiniLab on this page will allow you to separate the pigments in a leaf. Read the Connection to Chemistry at the end of this chapter to find out more about biological pigments.

Light-Dependent Reactions

The first phase of photosynthesis requires sunlight. As sunlight strikes the chlorophyll molecules in a photosystem of the thylakoid membrane, the energy in the light is transferred to electrons. These highly energized, or excited, electrons are passed from chlorophyll to an electron transport chain, a series of proteins embedded in the **thylakoid membrane**. Figure 9.5 summarizes this process.

Each protein in the chain passes energized electrons along to the next protein, similar to a bucket brigade in which a line of people pass a bucket of water from person to person to fight a fire. At each step along the transport chain, the electrons lose energy, just as some of the water might be spilled from buckets in the fire-fighting chain. This “lost” energy can be used to form ATP from ADP, or to pump hydrogen ions into the center of the thylakoid disc.

After the electrons have traveled down the electron transport chain, they are re-energized in a second photosystem and passed down a second electron transport chain. At the bottom of this chain, the electrons are still very energized. So that this energy is not wasted, the electrons are transferred to the stroma of the chloroplast. To do this, an electron carrier molecule called NADP+ (nicotinamide adenine dinucleotide phosphate) is used. NADP+ can combine with two excited electrons and a hydrogen ion (H+) to become NADPH. NADPH does not use the energy present in the energized electrons; it simply stores the energy until it can transfer it to the stroma. There, NADPH will play an important role in the light-independent reactions.

Restoring electrons

Recall that at the beginning of photosynthesis, electrons are lost from chlorophyll molecules when light is absorbed. If these electrons are not replaced, the chlorophyll will be unable to absorb additional light and the light-dependent reactions will stop, as will the production of ATP. To replace the lost electrons, molecules of water are split in the first photosystem. This reaction is called photolysis. For every water molecule that is split, one half molecule of oxygen, two electrons, and two hydrogen ions are formed, as shown in Figure 9.6. The oxygen produced by photolysis is released into the air and supplies the oxygen we breathe. The electrons are returned to chlorophyll. The hydrogen ions are pumped into the thylakoid, where they accumulate in high concentration. Because this difference in concentration forms a concentration gradient across the membrane, H+ ions diffuse out of the thylakoid and provide energy for the production of ATP. This coupling of the movement of H+ ions to ATP production is called chemiosmosis. The MiniLab on this page shows how the steps of photosynthesis were traced.

Light-Independent Reactions

The second phase of photosynthesis does not require light. It is called the **Calvin cycle**, which is a series of reactions that use carbon dioxide to form sugars. The **Calvin cycle** takes place in the stroma of the chloroplast, as shown in Figure 9.7.

The Calvin cycle

The Calvin cycle, named after Melvin Calvin, who worked out the details of the reactions, is called a cycle because one of the last molecules formed in the series of chemical reactions is also one of the molecules needed for the first reaction of the cycle. Therefore, one of the products can be used again to continue the cycle.

You have learned that in the electron transport chain, an energized electron is passed from protein to protein, and the energy is released slowly. You can imagine that making a complex carbohydrate from a molecule of CO2 would be a large task for a cell, so the light-independent reactions in the **stroma of the chloroplast** break down the complicated process into small steps.

At the beginning of the **Calvin cycle**, one molecule of carbon dioxide is added to one molecule of RuBP to form a six-carbon sugar. This step is called carbon fixation because carbon is “fixed” into a six-carbon sugar. In a series of reactions, the sugar breaks down and is eventually converted to two three-carbon sugars called phosphoglyceraldehyde, or PGAL. After three rounds of the cycle, with each round fixing one molecule of CO2, six molecules of PGAL are produced. Five of these molecules are rearranged to form three molecules of RuBP, the starting material. The sixth molecule of PGAL is available to the organism for making sugars, complex carbohydrates, and other organic compounds. As you will see in the next section, PGAL is important to all organisms because it plays a role in **cellular respiration**.

Getting Energy to Make ATP

Cellular Respiration

The process by which mitochondria break down food molecules to produce ATP is called **cellular respiration**. There are three stages of **cellular respiration**: glycolysis, the **citric acid cycle**, and the electron transport chain. The first stage, glycolysis, is anaerobic—no oxygen is required. The last two stages are aerobic and require oxygen to be completed.

Glycolysis

Glycolysis is a series of chemical reactions in the **cytoplasm of a cell** that break down glucose, a six-carbon compound, into two molecules of pyruvic acid, a three-carbon compound. Because two molecules of ATP are used to start glycolysis, and only four ATP molecules are produced, glycolysis is not very effective, producing only two ATP molecules for each glucose molecule broken down.

In the electron transport chain of **photosynthesis**, an electron carrier called NADP+ was described as carrying energized electrons to another location in the cell for further chemical reactions. Glycolysis also uses an electron carrier, called NAD+ (nicotinamide adenine dinucleotide). NAD+ forms NADH when it accepts two electrons.

Notice in Figure 9.8 that two molecules of PGAL are formed during glycolysis. Recall that PGAL also forms in the Calvin cycle. The PGAL made during **photosynthesis** can enter the glycolysis pathway and lead to the formation of ATP and organic molecules.

Following glycolysis, the pyruvic acid molecules move into the mitochondria, the organelles that transform energy for the cell. In the presence of oxygen, two more stages complete **cellular respiration**: the **citric acid cycle** and the electron transport chain of the mitochondrion. Before these two stages can begin, however, pyruvic acid undergoes a series of reactions in which it gives off a molecule of CO2 and combines with a molecule called coenzyme A to form acetyl-CoA. The reaction with coenzyme A produces a molecule of NADH and H+. These reactions are shown in Figure 9.9.

The citric acid cycle

The **citric acid cycle**, also called the Krebs cycle, is a series of chemical reactions similar to the Calvin cycle in that the molecule used in the first reaction is also one of the end products. Read Figure 9.10 to study the citric acid cycle.

For every turn of the cycle, one molecule of ATP and two molecules of carbon dioxide are produced. Two electron carriers are used, NAD+ and FAD (flavin adenine dinucleotide). A total of three NADH, three H+ ions, and one FADH2 are formed. The electron carriers each pass two energized electrons along to the electron transport chain in the **inner membrane of the mitochondrion**.

The electron transport chain

The electron transport chain in the **inner membrane of the mitochondrion** is very similar to the electron transport chains of the thylakoid membrane in the **chloroplasts of plant cells** during **photosynthesis**. NADH and FADH2 deliver energized electrons at the top of the chain. The electrons are passed from protein to protein within the membrane, slowly releasing their energy in steps. Some of that energy is used directly to form ATP; some is used by an enzyme to pump H+ ions into the center of the mitochondrion. Consequently, the mitochondrion inner membrane becomes positively charged because of the high concentration of positively charged hydrogen ions. At the same time, the exterior of the membrane is negatively charged, which further attracts hydrogen ions. The gradient of H+ ions that results across the **inner membrane of the mitochondrion** provides the energy for ATP production, just as it does in the chemiosmotic process that takes place at the **thylakoid membranes in the chloroplasts** . Figure 9.11 summarizes the electron transport chain and the formation of ATP.

The final electron acceptor at the bottom of the chain is oxygen, which reacts with four hydrogen ions (4H+) and four electrons to form two molecules of water (H2O). This is why oxygen is so important to our bodies. Without oxygen, the proteins in the electron transport chain cannot pass along the electrons. If a protein cannot pass along an electron to oxygen, it cannot accept another electron. Very quickly, the entire chain becomes blocked and ATP production stops.

Overall, the electron transport chain adds 32 ATP molecules to the four already produced. Obviously, the aerobic process of ATP production is very effective. In the absence of oxygen, however, an anaerobic process can produce small amounts of ATP to keep the cell from dying.

Fermentation

There are times, such as during heavy exercise, when your cells are without oxygen for a short period of time. When this happens, an anaerobic process called fermentation follows glycolysis and provides a means to continue producing ATP until oxygen is available again. There are two major types of fermentation: lactic acid fermentation and alcoholic fermentation. The table in Figure 9.12 compares the two processes with respiration.

Lactic acid fermentation

You know that under anaerobic conditions, the electron transport chain backs up because oxygen is not present as the final electron acceptor. As NADH and FADH2 arrive at the chain from the **citric acid cycle** and glycolysis, they cannot release their energized electrons. The **citric acid cycle** and glycolysis cannot continue without a steady supply of NAD+ and FAD.

The cell does not have a method to replace FAD during anaerobic conditions; however, NAD+ can be replaced through lactic acid fermentation. Lactic acid fermentation is one of the processes that supplies energy when oxygen is scarce. In this process, the reactions that produced pyruvic acid are reversed. Two molecules of pyruvic acid use NADH to form two molecules of lactic acid. This releases NAD+ to be used in glycolysis, allowing two ATP molecules to be formed for each glucose molecule. The lactic acid is transferred from muscle cells, where it is produced during strenuous exercise, to the liver that converts it back to pyruvic acid. The lactic acid that builds up in muscle cells results in muscle fatigue.

Alcoholic fermentation

Another type of fermentation, alcoholic fermentation, is used by yeast cells and some bacteria to produce CO2 and ethyl alcohol. When making bread, like that shown in Figure 9.13, yeast cells produce CO2 that forms bubbles in the dough. Eventually the heat of the oven kills the yeast and the bubble pockets are left to lighten the bread. You can do the MiniLab on this page to study alcoholic fermentation in apple juice.

Comparing Photosynthesis and Cellular Respiration

The production and breakdown of food molecules are accomplished by distinct processes that bear certain similarities. Both photosynthesis and cellular respiration use electron carriers and a cycle of chemical reactions to form ATP. Both use electron transport chains to form ATP and to create a chemical and a concentration gradient of H+ within a cell. This hydrogen ion gradient can be used to form ATP by chemiosmosis.

However, despite using such similar tools, the two cellular processes accomplish quite different tasks. **Photosynthesis** produces high-energy carbohydrates and oxygen from the sun’s energy, whereas **cellular respiration** uses oxygen to break down carbohydrates to form ATP and compounds that provide less energy. Also, one of the end products of **cellular respiration** is CO2, which is one of the beginning products for **photosynthesis**. The oxygen produced during **photosynthesis** is a critical molecule necessary for **cellular respiration**. Table 9.1 compares these complementary processes.

**Textbook 4: Dynamics of Life Vol. 2**

Atoms, Elements, and Compounds

Atoms

Chemistry is the study of matter—its composition and properties. Matter is anything that has mass and takes up space. All of the organisms you study in biology are made up of matter. Atoms are the building blocks of matter.

In the fifth century B.C., the Greek philosophers Leucippus and Democritus first proposed the idea that all matter is made up of tiny, indivisible particles. It wasn’t until the 1800s that scientists began to collect experimental evidence to support the existence of atoms. As technology improved over the next two centuries, scientists proved not only that atoms exist but also that they are made up of even smaller particles.

The structure of atoms

An atom is so small that billions of them fit on the head of a pin. Yet, atoms are made up of even smaller particles called neutrons, protons, and electrons, as illustrated in Figure 6.1. Neutrons and protons are located at the center of the atom, which is called the nucleus. Protons are positively charged particles (p+), and neutrons are particles that have no charge (n0). Electrons are negatively charged particles that are located outside the nucleus (e—). Electrons constantly move around an atom’s nucleus in energy levels. The basic structure of an atom is the result of the attraction between protons and electrons. Atoms contain an equal number of protons and electrons, so the overall charge of an atom is zero.

Elements

An element is a pure substance that cannot be broken down into other substances by physical or chemical means. Elements are made of only one type of atom.

There are over 100 known elements, 92 of which occur naturally. Scientists have collected a large amount of information about the elements, such as the number of protons and electrons each element has and the atomic mass of each element. Also, each element has a unique name and symbol. All of these data, and more, are collected in an organized table called the periodic table of elements.

The periodic table of elements

As shown in Figure 6.2, the periodic table is organized into horizontal rows, called periods, and vertical columns, called groups. Each individual block in the grid represents an element. The table is called periodic because elements in the same group have similar chemical and physical properties. This organization even allows scientists to predict elements that have not yet been discovered or isolated. As shown in Figure 6.3, elements found in living organisms also are found in Earth’s crust.

Isotopes Although atoms of the same element have the same number of protons and electrons, atoms of an element can have different numbers of neutrons, as shown in Figure 6.4. Atoms of the same element that have different numbers of neutrons are called isotopes. Isotopes of an element are identified by adding the number of protons and neutrons in the nucleus. For example, the most abundant form of carbon, carbon-12, has six protons and six neutrons in its nucleus. One carbon isotope—carbon-14—has six protons and eight neutrons. Isotopes of elements have the same chemical characteristics.

Radioactive isotopes

Previously, you read that neutrons have no charge. Changing the number of neutrons in an atom does not change the overall charge of the atom (it still has no charge). However, changing the number of neutrons can affect the stability of the nucleus, in some cases causing the nucleus to decay, or break apart. When a nucleus breaks apart, it gives off radiation that can be detected and used for many applications. Isotopes that give off radiation are called radioactive isotopes.

Carbon-14 is a radioactive isotope that is found in all living things. Scientists know the half-life, or the amount of time it takes for half of carbon-14 to decay, so they can calculate the age of an object by finding how much carbon-14 remains in the sample. Other radioactive isotopes have medical uses, such as in radiation therapy to treat cancers, as shown in Figure 6.5.

Compounds

Elements can combine to form more complex substances. A compound is a pure substance formed when two or more different elements combine. There are millions of known compounds and thousands more discovered each year. Figure 6.6 shows you a few. Each compound has a chemical formula made up of the chemical symbols from the periodic table. You might know that water is the compound H2O. Sodium chloride (NaCl) is the compound commonly called table salt. The fuel people use in cars is a mixture of hydrocarbon compounds. Hydrocarbons only have hydrogen and carbon atoms. Methane (CH4) is the simplest hydrocarbon. Bacteria in areas such as the wetlands shown in Figure 6.6 release 76 percent of global methane from natural sources by decomposing plants and other organisms. They are made of compounds, too.

Compounds have several unique characteristics. First, compounds are always formed from a specific combination of elements in a fixed ratio. Water always is formed in a ratio of two hydrogen atoms and one oxygen atom, and each water molecule has the same structure. Second, compounds are chemically and physically different than the elements that comprise them. For example, water has different properties than hydrogen and oxygen.

Another characteristic of compounds is that they cannot be broken down into simpler compounds or elements by physical means, such as tearing or crushing. Compounds, however, can be broken down by chemical means into simpler compounds or into their original elements. Consider again the example of water. You cannot pass water through a filter and separate the hydrogen from the oxygen, but a process called electrolysis, illustrated in Figure 6.7, can break water down into hydrogen gas and oxygen gas.

Chemical Bonds

Compounds such as water, salt, and methane are formed when two or more substances combine. The force that holds the substances together is called a chemical bond. Think back to the protons, neutrons, and electrons that make up an atom. The nucleus determines the chemical identity of an atom, and the electrons are involved directly in forming chemical bonds. Electrons travel around the nucleus of an atom in areas called energy levels, as illustrated in Figure 6.8. Each energy level has a specific number of electrons that it can hold at any time. The first energy level, which is the level closest to the nucleus, can hold up to two electrons. The second can hold up to eight electrons.

A partially-filled energy level is not as stable as an energy level that is empty or completely filled. Atoms become more stable by losing electrons or attracting electrons from other atoms. This results in the formation of chemical bonds between atoms. It is the forming of chemical bonds that stores energy and the breaking of chemical bonds that provides energy for processes of growth, development, adaptation, and reproduction in living things. There are two main types of chemical bonds—covalent bonds and ionic bonds.

Covalent bonds

When you were younger, you probably learned to share. If you had a book that your friend wanted to read as well, you could enjoy the story together. In this way, you both benefited from the book. Similarly, one type of chemical bond happens when atoms share electrons in their outer energy levels.

The chemical bond that forms when electrons are shared is called a covalent bond. Figure 6.9 illustrates the covalent bonds between oxygen and hydrogen to form water. Each hydrogen (H) atom has one electron in its outermost energy level and oxygen (O) has six. Because the outermost energy level of oxygen is the second level, which can hold up to eight electrons, oxygen has a strong tendency to fill the energy level by sharing the electrons from the two nearby hydrogen atoms.

Hydrogen does not completely give up the electrons, but also has a strong tendency to share electrons with oxygen to fill its outermost energy level. Two covalent bonds form, which creates water.

Most compounds in living organisms have covalent bonds holding them together. Water and other substances with covalent bonds are called molecules. A molecule is a compound in which the atoms are held together by covalent bonds. Depending on the number of pairs of electrons that are shared, covalent bonds can be single, double, or triple, as shown in Figure 6.10.

Ionic bonds

Recall that atoms are neutral—they do not have an electric charge. Also recall that for an atom to be most stable, the outer-most energy level should be either empty or completely filled. Some atoms tend to give up (donate) or obtain (accept) electrons to empty or fill the outer energy level in order to be stable. An atom that has lost or gained one or more electrons becomes an ion and carries an electric charge. For example, sodium has one electron in its outermost energy level. Sodium can become more stable if it gives up this one electron, leaving its outer energy level empty. When it gives away this one negative charge, the neutral sodium atom becomes a positively charged sodium ion (Na+). Similarly, chlorine has seven electrons in its outer energy level and needs just one electron to fill it. When chlorine accepts an electron from a donor atom, such as sodium, chlorine becomes a negatively charged ion (Cl—).

An ionic bond is an electrical attraction between two oppositely charged atoms or groups of atoms called ions. Figure 6.11 shows how an ionic bond forms as a result of the electrical attraction between Na+ and Cl— to produce NaCl (sodium chloride). Substances formed by ionic bonds are called ionic compounds.

Ions in living things include sodium, potassium, calcium, chloride, and carbonate ions. They help maintain homeostasis as they travel in and out of cells. In addition, ions help transmit signals among cells that allow you to see, taste, hear, feel, and smell.

Some atoms tend to donate or accept electrons more easily than other atoms. Look at the periodic table of elements inside the back cover of this textbook. The elements identified as metals tend to donate electrons, and the elements identified as nonmetals tend to accept electrons. The resulting ionic compounds have some unique characteristics. For example, most dissolve in water. When dissolved in solution, ionic compounds break down into ions and these ions can carry an electric current. Most ionic compounds, such as sodium chloride (table salt), are crystalline at room temperature. Ionic compounds generally have higher melting points than molecular compounds formed by covalent bonds.

Although most ionic compounds are solid at room temperature, other ionic compounds are liquid at room temperature. Like their solid counterparts, ionic liquids are made up of positively and negatively charged ions. Ionic liquids have important potential in real-world applications as safe and environmentally friendly solvents that can possibly replace other harmful solvents. The key characteristic of ionic liquid solvents is that they typically do not evaporate and release chemicals into the atmosphere. Most ionic liquids are safe to handle and store, and they can be recycled after use. For these reasons, ionic liquids are attractive to industries that are dedicated to environmental responsibility.

van der Waals Forces

You have learned that positive ions and negative ions form based on the ability of an atom to attract electrons. If the nucleus of the atom has a weak attraction for the electron, it will donate the electron to an atom with a stronger attraction. Similarly, elements in a covalent bond do not always attract electrons equally. Recall also that the electrons in a molecule are in random motion around the nuclei. This movement of electrons can cause an unequal distribution of the electron cloud around the molecule, creating temporary areas of slightly positive and negative charges.

When molecules come close together, the attractive forces between these positive and negative regions pull on the molecules and hold them together. These attractions between the molecules are called van der Waals forces, named for the Dutch physicist Johannes van der Waals who first described the phenomenon. The strength of the attraction depends on the size of the molecule, its shape, and its ability to attract electrons. Van der Waals forces are not as strong as covalent and ionic bonds, but they play a key role in biological processes.

Scientists have determined that geckos, such as the one shown in Figure 6.12, can climb smooth surfaces due to van der Waals forces between the atoms in the hairlike structures on their toes and the atoms on the surface they are climbing.

van der Waals forces in water

Let’s consider how van der Waals forces work in a common substance—water. The areas of slight positive and negative charge around the water molecule are attracted to the opposite charge of other nearby water molecules. These forces hold the water molecules together. Without van der Waals forces, water molecules would not form droplets, and droplets would not form a surface of water. It is important to understand that van der Waals forces are the attractive forces between the water molecules, not the forces between the atoms that make up water.

Chemical Reactions

Reactants and Products

A new car with its shining chrome and clean appearance is appealing to many drivers. Over time, however, the car might get rusty and lose some of its appeal. Rust is a result of a chemical change called a chemical reaction. A chemical reaction is the process by which atoms or groups of atoms in substances are reorganized into different substances. Chemical bonds are broken and/or formed during chemical reactions. The rust on the chain in Figure 6.13 is a compound called iron oxide (Fe2O3), and it was formed when oxygen (O2) in the air reacted with iron (Fe).

It is important to know that substances can undergo changes that do not involve chemical reactions. For example, consider the water in Figure 6.13. The water is undergoing a physical change. A physical change alters the substance’s appearance but not its composition. It is water before and after the change.

How do you know when a chemical reaction has taken place? Although you might not be aware of all the reactions taking place inside your body, you know the surface of the chain in Figure 6.13 has changed. What was once silver and shiny is now dull and orange-brown. Other clues that a chemical reaction has taken place include the production of heat or light, and formation of a gas, liquid, or solid.

Chemical equations

When scientists write chemical reactions, they express each component of the reaction in a chemical equation.

When writing chemical equations, chemical formulas describe the substances in the reaction and arrows indicate the process of change.

Reactants and products A chemical equation shows the reactants, the starting substances, on the left side of the arrow and the products, the substances formed during the reaction, on the right side of the arrow. The arrow can be read as “yields” or “react to form.”

Reactants → Products

The following chemical equation can be written to describe the reaction that provides energy in Figure 6.14.

C6H12O6 + O2 → CO2 + H2O

Glucose and oxygen react to form carbon dioxide and water.

Balanced equations

In chemical reactions, matter cannot be created or destroyed. This principle is called conservation of mass. Accordingly, all chemical equations must show this balance of mass. This means that the number of atoms of each element on the reactant side must equal the number of atoms of the same element on the product side. Use coefficients to make the number of atoms on each side of the arrow equal.

C6H12O6 + 6O2 → 6CO2 + 6H2O

Multiply the coefficient by the subscript for each element. You can see in this example that there are six carbon atoms, twelve hydrogen atoms, and eighteen oxygen atoms on each side of the arrow. The equation confirms that the number of atoms on each side is equal, and therefore the equation is balanced. You will study this important reaction further in Chapter 8.

Energy of Reactions

A sugar cookie is made with flour, sugar, and other ingredients mixed together, but it is not a cookie until you bake it. Something must start the change from cookie dough to cookies. The key to starting a chemical reaction is energy. For the chemical reactions that transform the dough to cookies to happen, energy in the form of heat is needed. Similarly, most compounds in living things cannot undergo chemical reactions without energy.

Activation energy

The minimum amount of energy needed for reactants to form products in a chemical reaction is called the activation energy. For example, you know a candle will not burn until you light its wick. The flame provides the activation energy for the reaction of the substances in the candle wick with oxygen. In this case, once the reaction begins, no further input of energy is needed and the candle continues to burn on its own. Figure 6.15 shows that for the reactants X and Y to form product XY, energy is required to start the reaction. The peak in the graph represents the amount of energy that must be added to the system to make the reaction go. Some reactions do not happen because they have a very high activation energy.

Energy change in chemical reactions Compare the progress of the reaction in Figure 6.15 to the progress of the reaction in Figure 6.16. Both reactions require activation energy to get started. However, notice from the graph in Figure 6.15 that the energy of the product is lower than the energy of the reactants. This reaction is exothermic—it released energy in the form of heat. The reaction in Figure 6.16 is endothermic—it absorbed heat energy. The energy of the products is higher than the energy of the reactant. In every chemical reaction, there is a change in energy due to the making and breaking of chemical bonds as reactants for products. Your body temperature of about 37°C is evidence that chemical reactions are happening inside your body.

Enzymes

All living things are chemical factories driven by chemical reactions. However, these chemical reactions proceed very slowly when carried out in the laboratory because the activation energy is high. To be useful to living organisms, additional substances must be present where the chemical reactions occur to reduce the activation energy and allow the reaction to proceed quickly.

A catalyst is a substance that lowers the activation energy needed to start a chemical reaction. Although a catalyst is important in speeding up a chemical reaction, it does not increase how much product is made and it does not get used up in the reaction. Scientists use many types of catalysts to make reactions go thousands of times faster than the reaction would be able to go without the catalyst.

Special proteins called enzymes are the biological catalysts that speed up the rate of chemical reactions in biological processes. Enzymes are essential to life. Compare the progress of the reaction described in Figure 6.17 to see the effect of an enzyme on a chemical reaction. Like all catalysts, the enzyme is not used up by the chemical reaction. Once it has participated in a chemical reaction, it can be used again.

An enzyme’s name describes what it does. For example, amylase is an important enzyme found in saliva. Digestion of food begins in your mouth when amylase speeds the breakdown of amylose, one of the two components of starch. Like amylase, most enzymes are specific to one reaction.

Follow Figure 6.18 to learn how an enzyme works. The reactants that bind to the enzyme are called substrates. The specific location where a substrate binds on an enzyme is called the active site. The active site and the substrate have complementary shapes. This enables them to interact in a precise manner, similar to the way in which puzzle pieces fit together. As shown in Figure 6.18, only substrates with the same size and shape as the active site will bind to the enzyme.

Once the substrates bind to the active site, the active site changes shape and forms the enzyme-substrate complex. The enzyme-substrate complex helps chemical bonds in the reactants to be broken and new bonds to form—the substrates react to form products. The enzyme then releases the products.

Factors such as pH, temperature, and other substances affect enzyme activity. For example, most enzymes in human cells are most active at an optimal temperature close to 37°C. However, enzymes in other organisms, such as bacteria, can be active at other temperatures.

Enzymes affect many biological processes. When a person is bitten by a poisonous snake, enzymes in the venom break down the membranes of that person’s red blood cells. Hard green apples ripen due to the action of enzymes. Photosynthesis and cellular respiration, which you will learn more about in Chapter 8, provide energy for the cell with the help of enzymes. Just as worker bees are important for the survival of a beehive, enzymes are the chemical workers in cells.

Water and Solutions

Water’s Polarity

Earlier in this chapter, you discovered that water molecules are formed by covalent bonds that link two hydrogen (H) atoms to one oxygen (O) atom. Because electrons are more strongly attracted to oxygen’s nucleus, the electrons in the covalent bond with hydrogen are not shared equally. In water, the electrons spend more time near the oxygen nucleus than they do near the hydrogen nuclei. Figure 6.19 shows that there is an unequal distribution of electrons in a water molecule. This, along with the bent shape of water, results in the oxygen end of the molecule having a slightly negative charge and the hydrogen ends of the molecule a slightly positive charge. Molecules that have an unequal distribution of charges are called polar molecules, meaning that they have oppositely charged regions.

Polarity is the property of having two opposite poles, or ends. A magnet has polarity—there is a north pole and a south pole. When the two ends are brought close to each other, they attract each other. Similarly, when a charged region of a polar molecule comes close to the oppositely charged region of another polar molecule, a weak electrostatic attraction results. In water, the electrostatic attraction is called a hydrogen bond. A hydrogen bond is a weak interaction involving a hydrogen atom and a fluorine, oxygen, or nitrogen atom. Hydrogen bonding is a strong type of van der Waals interaction. Figure 6.20 describes polarity and the other unique properties of water that make it important to living things.

Mixtures with water

Most students are familiar with powdered drink products that dissolve in water to form a flavored beverage. When you add a powdered substance to water, it does not react with water to form a new product. You create a mixture. A mixture is a combination of two or more substances in which each substance retains its individual characteristics and properties.

Homogenous mixtures When a mixture has a uniform composition throughout, it is called a homogeneous mixture. A solution is another name for a homogeneous mixture. For example, in the powdered tea drink solution shown in Figure 6.21, tea is on top, tea is in the middle, and tea is at the bottom of the container. The water retains its properties and the drink mix retains its properties.

In a solution, there are two components: a solvent and a solute. A solvent is a substance in which another substance is dissolved. A solute is the substance that is dissolved in the solvent. In the case of the drink mix, water is the solvent and the powdered substance is the solute. A mixture of salt and water is another example of a solution because the solute (salt) dissolves completely in the solvent (water). Saliva moistens your mouth and begins the digestion of some of your food. Saliva is a solution that contains water, proteins, and salts. In addition, the air you breathe is a solution of gases.

Heterogenous mixtures Think about the last time you ate a salad. Perhaps it contained lettuce and other vegetables, croutons, and salad dressing. Your salad was a heterogeneous mixture. In a heterogeneous mixture, the components remain distinct, that is, you can tell what they are individually. Compare the mixture of sand and water to the solution of salt and water next to it in Figure 6.22. Sand and water form a type of heterogeneous mixture called a suspension. Over time, the particles in a suspension settle to the bottom.

A colloid is a heterogeneous mixture in which the particles do not settle out like the sand settled from the water. You are probably familiar with many colloids, including fog, smoke, butter, mayonnaise, milk, paint, and ink. Blood is a colloid made up of plasma, cells, and other substances.

Acids and bases

Many solutes readily dissolve in water due to water’s polarity. This means that an organism, which might be as much as 70 percent water, can be a container for a variety of solutions. When a substance that contains hydrogen is dissolved in water, the substance might release a hydrogen ion (H+) because it is attracted to the negatively charged oxygen atoms in water, as shown in Figure 6.23. Substances that release hydrogen ions when dissolved in water are called acids. The more hydrogen ions a substance releases, the more acidic the solution becomes.

Similarly, substances that release hydroxide ions (OH–) when dissolved in water are called bases. Sodium hydroxide (NaOH) is a common base that breaks apart in water to release sodium ions (Na+) and hydroxide ions (OH–). The more hydroxide ions a substance releases, the more basic the solution becomes.

Acids and bases are key substances in biology. Many of the foods and beverages we eat and drink are acidic, and the substances in the stomach that break down the food, called gastric juices, are highly acidic.

pH and buffers

The amount of hydrogen ions or hydroxide ions in a solution determines the strength of an acid or base. Scientists have devised a convenient way to measure how acidic or basic a solution is. The measure of concentration of H+ in a solution is called pH. As shown in Figure 6.24, pure water is neutral and has a pH value of 7.0.

Acidic solutions have an abundance of H+ and have pH values lower than 7. Basic solutions have more OH– than H+ and have pH values higher than 7.

The majority of biological processes carried out by cells occur between pH 6.5 and 7.5. In order to maintain homeostasis, it is important to control H+ levels. If you’ve ever had an upset stomach, you might have taken an antacid to feel better. The antacid tablet is a buffer to help neutralize the stomach acid. Buffers are mixtures that can react with acids or bases to keep the pH within a particular range. In cells, buffers keep the pH in a cell within the 6.5 to 7.5 pH range. Your blood, for example, contains buffers that keep the pH about 7.4.

The Building Blocks of Life

Organic Chemistry

The element carbon is a component of almost all biological molecules. For this reason, life on Earth often is considered carbon-based. Because carbon is an essential element, scientists have devoted an entire branch of chemistry, called organic chemistry, to the study of organic compounds— those compounds containing carbon.

As shown in Figure 6.25, carbon has four electrons in its outermost energy level. Recall that the second energy level can hold eight electrons, so one carbon atom can form four covalent bonds with other atoms. These covalent bonds enable the carbon atoms to bond to each other, which results in a variety of important organic compounds.

These compounds can be in the shape of straight chains, branched chains, and rings, such as those illustrated in Figure 6.25. Together, carbon compounds lead to the diversity of life on Earth.

Macromolecules

Carbon atoms can be joined to form carbon molecules. Similarly, most cells store small carbon compounds that serve as building blocks for large molecules. Macromolecules are large molecules that are formed by joining smaller organic molecules together. These large molecules are also called polymers. Polymers are molecules made from repeating units of identical or nearly identical compounds called monomers that are linked together by a series of covalent bonds. As shown in Table 6.1, biological macromolecules are organized into four major categories: carbohydrates, lipids, proteins, and nucleic acids.

Carbohydrates Compounds composed of carbon, hydrogen, and oxygen in a ratio of one oxygen and two hydrogen atoms for each carbon atom are called carbohydrates. A general formula for carbohydrates is written as (CH2O)n. Here the subscript n indicates the number of CH2O units in a chain. Biologically important carbohydrates that have values of n ranging from three to seven are called simple sugars, or monosaccharides. The monosaccharide glucose, shown in Figure 6.26, plays a central role as an energy source for organisms.

Monosaccharides can be linked to form larger molecules. Two monosaccharides joined together form a disaccharide. Like glucose, disaccharides serve as energy sources. Sucrose, also shown in Figure 6.26, which is table sugar, and lactose, which is a component of milk, are both disaccharides. Longer carbohydrate molecules are called polysaccharides. One important polysaccharide is glycogen, which is shown in Figure 6.26. Glycogen is an energy storage form of glucose that is found in the liver and skeletal muscle. When the body needs energy between meals or during physical activity, glycogen is broken down into glucose.

In addition to their roles as energy sources, carbohydrates have other important functions in biology. In plants, a carbohydrate called cellulose provides structural support in cell walls. As shown in Figure 6.27, cellulose is made of chains of glucose linked together into tough fibers that are well-suited for their structural role. Chitin is a nitrogen-containing polysaccharide that is the main component in the hard outer shell of shrimp, lobsters, and some insects, as well as the cell wall of some fungi.

Lipids

Another important group of biological macromolecules is the lipid group. Lipids are molecules made mostly of carbon and hydrogen that make up the fats, oils, and waxes. Lipids are composed of fatty acids, glycerol, and other components. The primary function of lipids is to store energy. A lipid called a triglyceride is a fat if it is solid at room temperature and an oil if it is liquid at room temperature. In addition, triglycerides are stored in the fat cells of your body. Plant leaves are coated with lipids called waxes to prevent water loss, and the honeycomb in a beehive is made of beeswax.

Saturated and unsaturated fats Organisms need lipids in order to function properly. The basic structure of a lipid includes fatty acid tails as shown in Figure 6.28. Each tail is a chain of carbon atoms bonded to hydrogen and other carbon atoms by single or double bonds. Lipids that have tail chains with only single bonds between the carbon atoms are called saturated fats because no more hydrogens can bond to the tail. Lipids that have at least one double bond between carbon atoms in the tail chain can accommodate at least one more hydrogen and are called unsaturated fats. Fats with more than one double bond in the tail are called polyunsaturated fats.

Phospholipids A special lipid shown in

Figure 6.28, called a phospholipid, is responsible for the structure and function of the cell membrane. Lipids are hydrophobic, which means they do not dissolve in water. This characteristic is important because it allows lipids to serve as barriers in biological membranes.

Steroids

Another important category of lipids is the steroid group. Steroids include substances such as cholesterol and hormones. Despite its reputation as a “bad” lipid, cholesterol provides the starting point for other necessary lipids such as vitamin D and the hormones estrogen and testosterone.

Proteins

Another primary building block of living things is protein.

A protein is a compound made of small carbon compounds called amino acids. Amino acids are small compounds that are made of carbon, nitrogen, oxygen, hydrogen, and sometimes sulfur. All amino acids share the same general structure.

Amino acid structure

Amino acids have a central carbon atom like the one shown in Figure 6.29. Recall that carbon can form four covalent bonds. One of those bonds is with hydrogen. The other three bonds are with an amino group (–NH2), a carboxyl group (–COOH), and a variable group (–R). The variable group makes each amino acid different. There are 20 different variable groups, and proteins are made of different combinations of all 20 different amino acids. Several covalent bonds called peptide bonds join amino acids together to form proteins, which is also shown in Figure 6.29. A peptide forms between the amino group of one amino acid and the carboxyl group of another.

Three-dimensional protein structure

Based on the variable groups contained in the different amino acids, proteins can have up to four levels of structure. The number of amino acids in a chain and the order in which the amino acids are joined define the protein’s primary structure. After an amino acid chain is formed, it folds into a unique three-dimensional shape, which is the protein’s secondary structure. Figure 6.30 shows two basic secondary structures—the helix and the pleat. A protein might contain many helices, pleats, and folds. The tertiary structure of many proteins is globular, such as the hemoglobin protein shown in Table 6.1, but some proteins form long fibers. Some proteins form a fourth level of structure by combining with other proteins.

Protein function Proteins make up about 15 percent of your total body mass and are involved in nearly every function of your body. For example, your muscles, skin, and hair all are made of proteins. Your cells contain about 10,000 different proteins that provide structural support, transport substances inside the cell and between cells, communicate signals within the cell and between cells, speed up chemical reactions, and control cell growth.

Nucleic acids

The fourth group of biological macromolecules are nucleic acids. Nucleic acids are complex macromolecules that store and transmit genetic information. Nucleic acids are made of smaller repeating subunits called nucleotides. Nucleotides are composed of carbon, nitrogen, oxygen, phosphorus, and hydrogen atoms arranged as shown in Figure 6.31. There are six major nucleotides, all of which have three units—a phosphate, a nitrogenous base, and a ribose sugar.

There are two types of nucleic acids found in living organisms: deoxyribonucleic acid (DNA) and ribonucleic acid (RNA). In nucleic acids such as DNA and RNA, the sugar of one nucleotide bonds to the phosphate of another nucleotide. The nitrogenous base that sticks out from the chain is available for hydrogen bonding with other bases in other nucleic acids. You will learn more about the structure and function of DNA and RNA in Chapter 12.

A nucleotide with three phosphate groups is adenosine triphosphate (ATP). ATP is a storehouse of chemical energy that can be used by cells in a variety of reactions. It releases energy when the bond between the second and third phosphate group is broken.

Cell Discovery and Theory

History of the Cell theory

For centuries, scientists had no idea that the human body consists of trillions of cells. Cells are so small that their existence was unknown before the invention of the microscope. In 1665, as indicated in Figure 7.1, an English scientist named Robert Hooke made a simple microscope and looked at a piece of cork, the dead cells of oak bark. Hooke observed small, box-shaped structures, such as those shown in Figure 7.2. He called them cellulae because the boxlike cells of cork reminded him of the cells in which monks live at a monastery. It is from Hooke’s work that we have the term cell. A cell is the basic structural and functional unit of all living organisms.

During the late 1600s, Dutch scientist Anton van Leeuwenhoek—inspired by a book written by Hooke—designed his own microscope. To his surprise, he saw living organisms in pond water, milk, and various other substances. The work of these scientists and others led to new branches of science and many new and exciting discoveries.

The cell theory Naturalists and scientists continued observing the living microscopic world using glass lenses. In 1838, German scientist Matthias Schleiden carefully studied plant tissues and concluded that all plants are composed of cells. A year later, another German scientist, Theodor Schwann, reported that animal tissues also consisted of individual cells. Prussian physician Rudolph Virchow proposed in 1855 that all cells are produced from the division of existing cells. The observations and conclusions of these scientists and others are summarized as the **cell theory**. The **cell theory** is one of the fundamental ideas of modern biology and includes the following three principles:

1. All living organisms are composed of one or more cells.

2. Cells are the basic unit of structure and organization of all living organisms.

3. Cells arise only from previously existing cells, with cells passing copies of their genetic material on to their daughter cells.

Microscope Technology

The discovery of cells and the development of the **cell theory** would not have been possible without microscopes. Improvements made to microscopes have enabled scientists to study cells in detail, as described in Figure 7.1.

Turn back to the opening pages of this chapter and compare the magnifications of the skin shown there. Note that the detail increases as the magnification and resolution—the ability of the microscope to make individual components visible—increase. Hooke and van Leewenhoek would not have been able to see the individual structures within human skin cells with their microscopes. Developments in microscope technology have given scientists the ability to study cells in greater detail than early scientists ever thought possible.

Compound light microscopes

The modern **compound light microscope** consists of a series of glass lenses and uses visible light to produce a magnified image. Each lens in the series magnifies the image of the previous lens. For example, when two lenses each individually magnify 10 times, the total magnification would be 100 times (10 × 10). Scientists often stain cells with dyes to see them better when using a **light microscope** because cells are so tiny, thin, and translucent. Over the years, scientists have developed various techniques and modifications for **light microscopes**, but the properties of visible light will always limit resolution with these microscopes. Objects cause light to scatter, which blurs images. The maximum magnification without blurring is around 1000×.

Electron microscopes

As they began to study cells, scientists needed greater magnification to see the details of tiny parts of the cell. During the Second World War, in the 1940s, they developed the electron microscope. Instead of lenses, the **electron microscope** uses magnets to aim a beam of electrons at thin slices of cells. This type of electron microscope is called a **transmission electron microscope** (TEM) because electrons are passed, or transmitted, through a specimen to a fluorescent screen. Thick parts of the specimen absorb more electrons than thin parts, forming a black-and- white shaded image of the specimen. **Transmission electron microscope**s can magnify up to 500,000×, but the specimen must be dead, sliced very thin, and stained with heavy metals.

Over the past 65 years, many modifications have been made to the original electron microscopes. For example, the **scanning electron microscope (SEM)** is one modification that directs electrons over the surface of the specimen, producing a three-dimensional image. One disadvantage of using a TEM and an SEM is that only nonliving cells and tissues can be observed.

Another type of microscope, the **scanning tunneling electron microscope (STM)**, involves bringing the charged tip of a probe extremely close to the specimen so that the electrons “tunnel” through the small gap between the specimen and the tip. This instrument has enabled scientists to create three-dimensional computer images of objects as small as atoms. Unlike TEM and SEM, STM can be used with live specimens. Figure 7.3 shows DNA, the cell’s genetic material, magnified with a **scanning tunneling electron microscope**.

The **atomic force microscope (AFM)** measures various forces between the tip of a probe and the cell surface. To learn more about AFM, read the Cutting Edge Biology feature at the end of this chapter.

Basic Cell Types

You have learned, according to the **cell theory**, that cells are the basic units of all living organisms. By observing your own body and the living things around you, you might infer that cells must exist in various shapes and sizes. You also might infer that cells differ based on the function they perform for the organism. If so, you are correct! However, all cells have at least one physical trait in common: they all have a structure called a plasma membrane. A plasma membrane, labeled in Figure 7.4, is a special boundary that helps control what enters and leaves the cell. Each of your skin cells has a plasma membrane, as do the cells of a rattlesnake. This critical structure is described in detail in the next section.

Cells generally have a number of functions in common. For example, most cells have genetic material in some form that provides instructions for making substances that the cell needs. Cells also break down molecules to generate energy for metabolism. Scientists have grouped cells into two broad categories. These categories are prokaryotic cells and eukaryotic cells. Figure 7.4 shows TEM photomicrographs of these two cell types. The images of the prokayotic cell and eukaryotic cell have been enlarged so you can compare the cell structures. Eukaryotic cells generally are one to one hundred times larger than prokaryotic cells.

Look again at Figure 7.4 and compare the types of cells. You can see why scientists place them into two broad categories that are based on internal structures. Both have a plasma membrane, but one cell contains many distinct internal structures called organelles—specialized structures that carry out specific cell functions.

Eukaryotic cells contain a nucleus and other organelles that are bound by membranes, also referred to as membrane-bound organelles. The nucleus is a distinct central organelle that contains the cell’s genetic material in the form of DNA. Organelles enable cell functions to take place in different parts of the cell at the same time. Most organisms are made up of eukaryotic cells and are called eukaryotes. However, some unicellular organisms, such as some algae and yeast, are also eukaryotes.

Prokaryotic cells are defined as cells without a nucleus or other membrane-bound organelles. Most unicellular organisms, such as bacteria, are prokaryotic cells. Thus they are called prokaryotes. Many scientists think that prokaryotes are similar to the first organisms on Earth.

Origin of cell diversity If you have ever wondered why a company makes two products that are similar, you can imagine that scientists have asked why there are two basic types of cells. The answer might be that eukaryotic cells evolved from prokaryotic cells millions of years ago. According to the **endosymbiont theory**, a symbiotic mutual relationship involved one prokaryotic cell living inside of another.

Imagine how organisms would be different if the eukaryotic form had not evolved. Because eukaryotic cells are larger and have distinct organelles, these cells have developed specific functions. Having specific functions has led to cell diversity, and thus more diverse organisms that can adapt better to their environments. Life-forms more complex than bacteria might not have evolved without eukaryotic cells.

The Plasma Membrane

Function of the Plasma Membrane

Recall from Chapter 1 that the process of maintaining balance in an organism’s internal environment is called homeostasis. Homeostasis is essential to the survival of a cell. One of the structures that is primarily responsible for homeostasis is the plasma membrane. The plasma membrane is a thin, flexible boundary between a cell and its environment that allows nutrients into the cell and allows waste and other products to leave the cell. All prokaryotic cells and eukaryotic cells have a plasma membrane to separate them from the watery environments in which they exist.

A key property of the plasma membrane is selective permeability, by which a membrane allows some substances to pass through while keeping others out. Consider a fish net as an analogy of selective permeability. The net shown in Figure 7.5 has holes that allow water and other substances in the water to pass through but not the fish. Depending on the size of the holes in the net, some kinds of fish might pass through, while others are caught. The diagram in Figure 7.5 illustrates selective permeability of the plasma membrane. The arrows show that substances enter and leave the cell through the plasma membrane. Control of how, when, and how much of these substances enter and leave a cell relies on the structure of the plasma membrane.

Structure of the Plasma Membrane

Most of the molecules in the plasma membrane are lipids. Recall from Chapter 6 that lipids are large molecules that are composed of glycerol and three fatty acids. If a phosphate group replaces a fatty acid, a phospholipid forms. A phospholipid is a molecule that has a glycerol backbone, two fatty acid chains, and a phosphate-containing group. The plasma membrane is composed of a phospholipid bilayer, in which two layers of phospholipids are arranged tail-to-tail, as shown in Figure 7.6. In the plasma membrane, phospholipids arrange themselves in a way that allows the plasma membrane to exist in the watery environment.

The phospholipid bilayer Notice in Figure 7.6 that each phospholipid is diagrammed as a head with two tails. The phosphate group in each phospholipid makes the head polar. The polar head is attracted to water because water also is polar. The two fatty acid tails are nonpolar and are repelled by water.

The two layers of phospholipid molecules make a sandwich, with the fatty acid tails forming the interior of the plasma membrane and the phospholipid heads facing the watery environments found inside and outside the cell, as shown in Figure 7.6. This bilayer structure is critical for the formation and function of the plasma membrane. The phospholipids are arranged in such a way that the polar heads can be closest to the water molecules and the nonpolar tails can be farthest away from the water molecules.

When many phospholipid molecules come together in this manner, a barrier is created that is polar at its surfaces and nonpolar in the middle. Water-soluble substances will not move easily through the plasma membrane because they are stopped by the nonpolar middle. Therefore, the plasma membrane can separate the environment inside the cell from the environment outside the cell.

Other components of the plasma membrane

Moving with and among the phospholipids in the **plasma membrane** are cholesterol, proteins, and carbohydrates. When found on the outer surface of the plasma membrane, proteins called receptors transmit signals to the inside of the cell. Proteins at the inner surface anchor the plasma membrane to the cell’s internal support structure, giving the cell its shape. Other proteins span the entire membrane and create tunnels through which certain substances enter and leave the cell. These transport proteins move needed substances or waste materials through the plasma membrane, and therefore contribute to the selective permeability of the plasma membrane.

Locate the cholesterol molecules in Figure 7.6. Nonpolar cholesterol is repelled by water and is positioned among the phospholipids. Cholesterol helps to prevent the fatty-acid tails of the phospholipid bilayer from sticking together, which contributes to the fluidity of the plasma membrane. Although avoiding a high-cholesterol diet is recommended, cholesterol plays a critical role in plasma membrane structure and it is an important substance for maintaining homeostasis in a cell.

Other substances in the membrane, such as carbohydrates attatched to proteins, stick out from the plasma membrane to define the cell’s characteristics and help cells identify chemical signals. For example, carbohydrates in the membrane might help disease-fighting cells recognize and attack a potentially harmful cell.

Together, the phospholipids in the bilayer create a “sea” in which other molecules can float, like apples floating in a barrel of water. This “sea” concept is the basis for the **fluid mosaic model** of the plasma membrane. The phospholipids can move sideways within the membrane just as apples move around in water. At the same time, other components in the membrane, such as proteins, also move among the phospholipids. Because there are different substances in the **plasma membrane**, a pattern, or mosaic, is created on the surface. You can see this pattern in Figure 7.7. The components of the plasma membrane are in constant motion, sliding past one another.

Structures and Organelles

Cytoplasm and Cytoskeleton

You just have investigated the part of a cell that functions as the boundary between the inside and outside environments. The environment inside the plasma membrane is a semifluid material called cytoplasm. In a **prokaryotic cell**, all of the chemical processes of the cell, such as breaking down sugar to generate the energy used for other functions, take place directly in the cytoplasm. Eukaryotic cells perform these processes within organelles in their cytoplasm. At one time, scientists thought that cell organelles floated in a sea of cytoplasm.

More recently, cell biologists have discovered that organelles do not float freely in a cell, but are supported by a structure within the cytoplasm similar to the structure shown in Figure 7.8. The cytoskeleton is a supporting network of long, thin protein fibers that form a framework for the cell and provide an anchor for the organelles inside the **cells**. The cytoskeleton also has a function in cell movement and other cellular activities.

The cytoskeleton is made of substructures called microtubules and microfilaments. Microtubules are long, hollow protein cylinders that form a rigid skeleton for the cell and assist in moving substances within the cell. Microfilaments are thin protein threads that help give the cell shape and enable the entire cell or parts of the cell to move. Microtubules and microfilaments rapidly assemble and disassemble and slide past one another. This allows cells and organelles to move.

Cell Structures

In a factory, there are separate areas set up for performing different tasks. Eukaryotic cells also have separate areas for tasks. Membrane-bound organelles make it possible for different chemical processes to take place at the same time in different parts of the cytoplasm.

Organelles carry out essential cell processes, such as protein synthesis, energy transformation, digestion of food, excretion of wastes, and cell division. Each organelle has a unique structure and function. You can compare organelles to a factory’s offices, assembly lines, and other important areas that keep the factory running. As you read about the different organelles, refer to the diagrams of plant and animal cells in Figure 7.9 to see the organelles of each type.

The nucleus

Just as a factory needs a manager, a cell needs an organelle to direct the cell processes. The nucleus, shown in Figure 7.10, is the cell’s managing structure. It contains most of the cell’s DNA, which stores information used to make proteins for cell growth, function, and reproduction.

The nucleus is surrounded by a double membrane called the **nuclear envelope**. The nuclear envelope is similar to the plasma membrane, except the nuclear membrane has nuclear pores that allow larger-sized substances to move in and out of the nucleus. Chromatin, which is a complex DNA attached to protein, is spread throughout the **nucleus**.

Reading Check Describe the role of the nucleus.

Ribosomes

One of the functions of a cell is to produce proteins. The organelles that help manufacture proteins are called ribosomes. Ribosomes are made of two components—RNA and protein—and are not bound by a membrane like other organelles. Within the nucleus is the site of ribosome production called the **nucleolus**, shown in Figure 7.10.

Cells have many ribosomes that produce a variety of proteins that are used by the cell or are moved out and used by other cells. Some ribosomes float freely in the **cytoplasm**, while others are bound to another organelle called the endoplasmic reticulum. Free-floating ribosomes produce proteins for use within the cytoplasm of the cell. Bound ribosomes produce proteins that will be bound within membranes or used by other cells.

Endoplasmic reticulum

The endoplasmic reticulum, also called ER, is a membrane system of folded sacs and interconnected channels that serves as the site for protein and lipid synthesis. The pleats and folds of the ER provide a large amount of surface area where cellular functions can take place. The area of ER where ribosomes are attached is called rough endoplasmic reticulum. Notice in Figure 7.11 that the rough ER appears to have bumps on it. These bumps are the attached ribosomes that will produce proteins for export to other cells.

Figure 7.11 also shows that there are areas of the ER that do not have ribosomes attached. The area of ER where no ribosomes are attached is called smooth endoplasmic reticulum. Although the smooth ER has no ribosomes, it does perform important functions for the cell. For example, the smooth ER provides a membrane surface where a variety of complex carbohydrates and lipids, including phospholipids, are synthesized. Smooth ER in the liver detoxifies harmful substances.

Golgi apparatus

After the hiking boots are made in the factory, they must be organized into pairs, boxed, and shipped. Similarly, after proteins are made in the endoplasmic reticulum, some might be transferred to the Golgi apparatus, illustrated in Figure 7.12. The Golgi apparatus is a flattened stack of membranes that modifies, sorts, and packages proteins into sacs called vesicles. Vesicles then can fuse with the cell’s plasma membrane to release proteins to the environment outside the cell. Observe the vesicle in Figure 7.12.

Vacuoles

A factory needs a place to store materials and waste products. Similarly, cells have membrane-bound vesicles called vacuoles for temporary storage of materials within the cytoplasm. A vacuole, such as the plant vacuole shown in Figure 7.13, is a sac used to store food, enzymes, and other materials needed by a cell. Some vacuoles store waste products. Interestingly, animal cells usually do not contain vacuoles. If animal cells do have vacuoles, they are much smaller than those in plant cells.

Lysosomes Factories and cells also need clean-up crews. In the cell, lysosomes, shown in Figure 7.14, are vesicles that contain substances that digest excess or worn-out organelles and food particles. Lysosomes also digest bacteria and viruses that have entered the cell. The membrane surrounding a lysosome prevents the digestive enzymes inside from destroying the cell. Lysosomes can fuse with vacuoles and dispense their enzymes into the vacuole, digesting the wastes inside.

Centrioles Previously in this section you read about microtubules and the cytoskeleton. Groups of microtubules form another structure called a centriole. Centrioles, shown in Figure 7.15, are organelles made of microtubules that function during cell division.

Centrioles are located in the **cytoplasm** of animal cells and most protists and usually are near the **nucleus**. You will learn about cell division and the role of centrioles in Chapter 9.

Mitochondria

Imagine now that the boot factory has its own generator that produces the electricity it needs. Cells also have energy generators called mitochondria that convert fuel particles (mainly sugars), into usable energy. Figure 7.16 shows that a mitochondrion has an outer membrane and a highly folded inner membrane that provides a large surface area for breaking the bonds in sugar molecules. The energy produced from that breakage is stored in the bonds of other molecules and later used by the cell. For this reason, mitochondria often are referred to as the “powerhouses” of cells.

Chloroplasts Factory machines need electricity that is generated by burning fossil fuels or by collecting energy from alternative sources, such as the Sun. Plant cells have their own way of using solar energy. In addition to mitochondria, plants and some other eukaryotic cells contain chloroplasts, which are organelles that capture light energy and convert it to chemical energy through a process called **photosynthesis**. Examine Figure 7.17 and notice that inside the **inner membrane** are many small, disk-shaped compartments called thylakoids. It is here that the energy from sunlight is trapped by a pigment called chlorophyll. Chlorophyll gives leaves and stems their green color.

Chloroplasts belong to a group of plant organelles called plastids, some of which are used for storage. Some plastids store starches or lipids. Others, such as chromoplasts, contain red, orange, or yellow pigments that trap light energy and give color to plant structures such as flowers or leaves.

Cell wall

Another structure associated with plant cells is the cell wall, shown in Figure 7.18. The cell wall is a thick, rigid, mesh of fibers that surrounds the outside of the **plasma membrane**, protecting the cell and giving it support. Rigid cell walls allow plants to stand at various heights—from blades of grass to California redwoods. Plant cell walls are made of a carbohydrate called cellulose, which gives the wall its inflexible characteristics. Table 7.1 lists cell walls and various other cell structures.

Cilia and flagella

Some eukaryotic cell surfaces have structures called cilia and flagella that project outside the **plasma membrane**. As shown in Figure 7.19, cilia are short, numerous projections that look like hairs. The motion of cilia is similar to the motion of oars in a rowboat. Flagella are longer and less numerous than cilia. These projections move with a whiplike motion. Cilia and flagella are composed of microtubules arranged in a 9 + 2 configuration, in which nine pairs of microtubules surround two single microtubules. Typically, a cell has one or two flagella.

Prokaryotic cilia and flagella contain cytoplasm and are enclosed by the plasma membrane. They consist of protein building blocks.

While both structures are used for cell movement, cilia are also found on stationary cells.

Comparing Cells

Table 7.1 summarizes the structures of eukaryotic plant cells and animal cells. Notice that plant cells contain chlorophyll—they can capture and transform energy from the Sun into a usable form of chemical energy. This is one of the main characteristics that distinguishes plants from animals. In addition, remember that animal cells usually do not contain vacuoles. If they do, vacuoles in **animal cells** are much smaller than vacuoles in **plant cells**. Also, animal cells do not have cell walls.

Cell walls give plant cells protection and support.

Organelles at Work

With a basic understanding of the structures found within a cell, it becomes easier to envision how those structures work together to perform cell functions. Take, for example, the synthesis of proteins.

Protein synthesis begins in the **nucleus** with the information contained in the **DNA**. Genetic information is copied and transferred to another genetic molecule called RNA. Then RNA and ribosomes, which have been manufactured in the **nucleolus**, leave the nucleus through the **pores** of the nuclear membrane. Together, RNA and ribosomes manufacture proteins. Each protein made on the rough ER has a particular function; it might become a protein that forms a part of the plasma membrane, a protein that is released from the cell, or a protein transported to other organelles. Other ribosomes will float freely in the **cytoplasm** and make proteins as well.

Most of the proteins made on the surface of the ER are sent to the Golgi apparatus. The Golgi apparatus packages the proteins in **vesicles** and transports them to other organelles or out of the cell. Other organelles use the proteins to carry out cell processes. For example, lysosomes use proteins, enzymes in particular, to digest food and waste. Mitochondria use enzymes to produce a usable form of energy for the cell.

After reading about the organelles in a cell, it becomes clearer why people equate the cell to a factory. Each organelle has its job to do, and the health of the cell depends on all of the components working together.

Cellular Transport

Diffusion

As the aroma of baking cookies makes its way to you, the particles are moving and colliding with each other in the air. This happens because the particles in gases, liquids, and solids are in random motion. Similarly, substances dissolved in water move constantly in random motion called Brownian motion. This random motion causes diffusion, which is the net movement of particles from an area where there are many particles of the substance to an area where there are fewer particles of the substance. The amount of a substance in a particular area is called concentration. Therefore, substances diffuse from areas of high concentration to low concentration. Figure 7.20 illustrates the process of diffusion. Additional energy input is not required for diffusion because the particles already are in motion.

For example, if you drop red and blue ink into a container of water at opposite ends the container, which is similar to the watery environment of a cell, the process of diffusion begins, as shown in Figure 7.20(A). In a short period of time, the ink particles have mixed as a result of diffusion to the point where a purple color blend area is visible. Figure 7.20(B) shows the initial result of this diffusion.

Given more time, the ink particles continue to mix and, in this case, continue to form the uniform purple mixture shown in Figure 7.20(C). Mixing continues until the concentrations of red ink and blue ink are the same in all areas. The final result is the purple solution. After this point, the particles continue to move randomly, but no further change in concentration will occur. This condition, in which there is continuous movement but no overall change, is called dynamic equilibrium.

One of the key characteristics of diffusion is the rate at which diffusion takes place. Three main factors affect the rate of diffusion: concentration, temperature, and pressure. When concentration is high, diffusion occurs more quickly because there are more particles that collide. Similarly, when the temperature or pressure increases, the number of collisions increases, thus increasing the rate of diffusion.

Recall that at higher temperatures particles move faster, and at higher pressure the particles are closer together. In both cases, more collisions occur and diffusion is faster. The size and charge of a substance also affects the rate of diffusion.

Diffusion across the plasma membrane In addition to water, cells need certain ions and small molecules, such as chloride ions and sugars, to perform cellular functions. Water can diffuse across the plasma membrane, as shown in Figure 7.21(A), but most other substances cannot. Another form of transport, called facilitated diffusion, uses transport proteins to move other ions and small molecules across the plasma membrane. By this method, substances move into the cell through a water-filled transport protein called a channel protein that opens and closes to allow the substance to diffuse through the plasma membrane, as shown in Figure 7.21(B). Another type of transport protein called a carrier protein also can help substances diffuse across the plasma membrane. Carrier proteins change shape as the diffusion process continues to help move the particle through the membrane, as illustrated in Figure 7.21(C).

Diffusion of water and facilitated diffusion of other substances require no additional input of energy because the particles are moving from an area of high concentration to an area of lower concentration. This is also known as passive transport. You will learn later in this section about a form of cellular transport that does require energy input.

Osmosis: Diffusion of Water

Water is a substance that passes freely into and out of the cell through the **plasma membrane**. The diffusion of water across a selectively permeable membrane is called osmosis.

Regulating the movement of water across the plasma membrane is an important factor in maintaining homeostasis within the cell.

How osmosis works

Recall that in a solution, a substance called the solute is dissolved in a solvent. Water is the solvent in a cell and its environment.

Concentration is a measure of the amount of solute dissolved in a solvent. The concentration of a solution decreases when the amount of solvent increases.

Examine Figure 7.22 showing a U-shaped tube containing solutions with different sugar concentrations separated by a selectively permeable membrane. What will happen if the solvent (water) can pass through the membrane but the solute (sugar) cannot?

Water molecules diffuse toward the side with the greater sugar concentration—the right side. As water moves to the right, the concentration of the sugar solution decreases. The water continues to diffuse until dynamic equilibrium occurs—the concentration of the solutions is the same on both sides. Notice in Figure 7.22 that the result is an increase in solution level on the right side. During dynamic equilibrium, water molecules continue to diffuse back and forth across the membrane. But, the concentrations on each side no longer change.

Cells in an isotonic solution When a cell is in a solution that has the same concentration of water and solutes—ions, sugars, proteins, and other substances—as its cytoplasm, the cell is said to be in an isotonic solution. Isocomes from the Greek word meaning equal.

Water still moves through the plasma membrane, but water enters and leaves the cell at the same rate. The cell is at equilibrium with the solution, and there is no net movement of water. The cells retain their normal shape, as shown in Figure 7.23. Most cells in organisms are in isotonic solutions, such as blood.

Cells in a hypotonic solution

If a cell is in a solution that has a lower concentration of solute, the cell is said to be in a hypotonic solution. Hypo- comes from the Greek word meaning under. There is more water outside of the cell than inside. Due to osmosis, the net movement of water through the plasma membrane is into the cell, as illustrated in Figure 7.24. Pressure generated as water flows through the plasma membrane is called osmotic pressure. In an animal cell, as water moves into the cell, the pressure increases and the plasma membrane swells. If the solution is extremely hypotonic, the plasma membrane might be unable to withstand this pressure and the cell might burst.

Cells in a hypertonic solution

When a cell is placed in a **hypertonic solution**, the concentration of the solute outside of the cell is higher than inside. Hyper comes from the Greek word meaning above. During osmosis, the net movement of water is out of the cell, as illustrated in Figure 7.25. Animal cells in a hypertonic solution shrivel because of decreased pressure in the cells. Plant cells in a **hypertonic solution** lose water, mainly from the central vacuole. The plasma membrane shrinks away from the cell wall. Loss of water in a plant cell causes wilting.

Active Transport

Sometimes substances must move from a region of lower concentration to a region of higher concentration against the passive movement from higher to lower concentration. This movement of substances across the plasma membrane against a concentration gradient requires energy, therefore, it is called active transport. Figure 7.26 illustrates how active transport occurs with the aid of carrier proteins, commonly called pumps. Some pumps move one type of substance in only one direction, while others move two substances either across the membrane in the same direction or in opposite directions. Due to active transport, the cell maintains the proper balance of substances it needs. Active transport helps maintain homeostasis.

Na+/K+ ATPase pump

One common active transport pump is called the sodium-potassium ATPase pump. This pump is found in the **plasma membrane** of animal cells. The pump maintains the level of sodium ions (Na+) and potassium ions (K+) inside and outside the cell. This protein pump is an enzyme that catalyzes the breakdown of an energy-storing molecule. The pump uses the energy in order to transport three sodium ions out of the cell while moving two potassium ions into the cell. The high level of sodium on the outside of the cell creates a concentration gradient. Follow the steps in Figure 7.27 to see the action of the Na+/K+ ATPase pump.

The activity of the Na+/K+ ATPase pump can result in yet another form of cellular transport. Substances, such as sugar molecules, must come into the cell from the outside, where the concentration of the substance is lower than inside. This requires energy. Recall, however, that the Na+/K+ ATPase pump moves Na+ out of the cell, which creates a low concentration of Na+ inside the cell. In a process called coupled transport, the Na+ ions that have been pumped out of the cell can couple with sugar molecules and be transported into the cell through a membrane protein called a **coupled channel**. The sugar molecule, coupled to a Na+ ion, enters the cell by facilitated diffusion of the sodium, as shown in Figure 7.28. As a result, sugar enters the cell without spending any additional cellular energy.

Transport of Large Particles

Some substances are too large to move through the plasma membrane by diffusion or transport proteins and get inside the cell by a different process. Endocytosis is the process by which a cell surrounds a substance in the outside environment, enclosing the substance in a portion of the plasma membrane. The membrane then pinches off and leaves the substance inside the **cell**. The substance shown on the left in Figure 7.29 is engulfed and enclosed by a portion of the cell’s plasma membrane. The membrane then pinches off inside of the cell and the resulting vacuole, with its contents, moves to the inside of the cell.

Exocytosis is the secretion of materials at the plasma membrane. The illustration on the right in Figure 7.29 shows that exocytosis is the reverse of endocytosis. Cells use exocytosis to expel wastes and to secrete substances, such as hormones, produced by the cell. Both endocytosis and exocytosis require the input of energy. Cells maintain homeostasis by moving substances into and out of the cell. Some transport processes require additional energy input, while others do not. Together, the different types of transport allow a cell to interact with its environment while maintaining homeostasis.

How Organisms Obtain Energy

Transformation of Energy

Many chemical reactions and processes in your cells are ongoing, even when you might not think you are using any energy. Macromolecules are assembled and broken down, substances are transported across cell membranes, and genetic instructions are transmitted. All of these cellular activities require energy—the ability to do work. Thermodynamics is the study of the flow and transformation of energy in the universe.

Laws of thermodynamics

There are two laws of thermodynamics. The first law of thermodynamics is the law of conservation of energy, which states that energy can be converted from one form to another, but it cannot be created nor destroyed. For example, the stored energy in food is converted to chemical energy when you eat and to mechanical energy when you run or kick a ball.

The second law of thermodynamics states that energy cannot be converted without the loss of usable energy. The energy that is “lost” is generally converted to thermal energy. Entropy is the measure of disorder, or unusable energy, in a system. Therefore, the second law of thermodynamics can also be stated “entropy increases.” One example of the second law of thermodynamics is evident in food chains. Recall from Chapter 2 that in a food chain the amount of usable energy that is available to the next trophic level decreases.

Autotrophs and heterotrophs

All organisms need energy to live. Directly or indirectly nearly all the energy for life comes from the Sun.

Recall from Chapter 2 that some organisms make their own food, while others must obtain it from other organisms. Autotrophs are organisms that make their own food. Some autotrophs, called chemoautotrophs, use inorganic substances such as hydrogen sulfide as a source of energy. Other autotrophs, such as the plant in Figure 8.2, convert light energy from the Sun into chemical energy. Autotrophs that convert energy from the Sun are called photoautotrophs. Heterotrophs, such as the aphid and the ladybug in Figure 8.2, are organisms that need to ingest food to obtain energy.

Metabolism

All of the chemical reactions in a cell are referred to as the cell’s metabolism. A series of chemical reactions in which the product of one reaction is the substrate for the next reaction is called a metabolic pathway. Metabolic pathways include two broad types: catabolic pathways and anabolic pathways. Catabolic pathways release energy by breaking down larger molecules into smaller molecules.

Anabolic pathways use the energy released by catabolic pathways to build larger molecules from smaller molecules. The relationship of anabolic and catabolic pathways results in the continual flow of energy within an organism.

Energy continually flows between the metabolic reactions of organisms in an ecosystem. **Photosynthesis** is the anabolic pathway in which light energy from the Sun is converted to chemical energy for use by the cell. In this reaction, autotrophs use light energy, carbon dioxide, and water to form glucose and oxygen. As shown in Figure 8.3, the energy stored in the **glucose** produced by **photosynthesis** can be transferred to other organisms when the molecules are consumed as food.

**Cellular respiration** is the catabolic pathway in which organic molecules are broken down to release energy for use by the cell. In **cellular respiration,** oxygen is used to break down organic molecules, resulting in the production of carbon dioxide and water. Notice the cyclical nature of these processes in Figure 8.3, where the products of one reaction are the reactants for the other reaction.

ATP: The Unit of Cellular Energy

Energy exists in many forms including light energy, mechanical energy, thermal energy, and chemical energy. In living organisms, chemical energy is stored in biological molecules and can be converted to other forms of energy when needed. For example, the chemical energy in biological molecules is converted to mechanical energy when muscles contract. Adenosine triphosphate—ATP—is the most important biological molecule that provides chemical energy.

ATP structure Recall from Chapter 6 that ATP is a multipurpose storehouse of chemical energy that can be used by cells in a variety of reactions. Although other carrier molecules transport energy within cells, ATP is the most abundant energy-carrier molecule in cells and is found in all types of organisms. As shown in Figure 8.4, ATP is made of an adenine base, a ribose sugar, and three phosphate groups.

ATP function ATP releases energy when the bond between the second and third phosphate groups is broken, forming a molecule called adenosine diphosphate (ADP) and a free phosphate group, as shown in Figure 8.4. Energy is stored in the phosphate bond formed when ADP receives a phosphate group and becomes ATP. As shown in Figure 8.4, ATP and ADP can be interchanged by the addition or removal of a phosphate group. Sometimes ADP becomes adenosine monophosphate (AMP) by losing an additional phosphate group.

There is less energy released in this reaction, so most of the energy reactions in the cell involve ATP and ADP.

Photosynthesis

Overview of Photosynthesis

Most autotrophs—including plants—make organic compounds, such as sugars, by a process called **photosynthesis**. Recall that photosynthesis is a process in which light energy is converted into chemical energy. The overall chemical equation for photosynthesis is shown below.

**Photosynthesis** occurs in two phases. The locations of these phases are shown in Figure 8.5. In phase one, the light-dependent reactions, light energy is absorbed and then converted into chemical energy in the form of ATP and NADPH. In phase two, the light-independent reactions, the ATP and NADPH that were formed in phase one are used to make glucose. Once glucose is produced, it can be joined to other simple sugars to form larger molecules. These larger molecules are complex carbohydrates, such as starch. Recall from Chapter 6 that carbohydrates are composed of repeating units of small organic molecules. The end products of **photosynthesis** also can be used to make other organic molecules, such as proteins, lipids, and nucleic acids.

Phase One: Light Reactions

The absorption of light is the first step in **photosynthesis**. Plants have special organelles to capture light energy. Once the energy is captured, two energy storage molecules—NADPH and ATP—are produced to be used in the light-independent reactions.

Chloroplasts Large organelles, called chloroplasts, capture light energy in photosynthetic organisms. In plants, chloroplasts are found mainly in the cells of leaves. As shown in Figure 8.5, chloroplasts are disc-shaped organelles that contain two main compartments essential to **photosynthesis**. The first compartment is called the thylakoid. Thylakoids are flattened saclike membranes that are arranged in stacks. Light-dependent reactions take place within the **thylakoids**. The thylakoids form stacks called grana. The second important compartment is called the stroma—the fluid-filled space that is outside the grana. This is the location of the light-independent reactions in phase two of **photosynthesis**.

Pigments Light-absorbing colored molecules called pigments are found in the thylakoid membranes of chloroplasts. Pigments differ in their ability to absorb specific wavelengths of light, as illustrated in Figure 8.6.

The major light-absorbing pigments in plants are chlorophylls. There are several types of chlorophylls, but the most common two are chlorophyll a and chlorophyll b. The structure of chlorophyll can differ from one molecule to another, enabling distinct chorophyll molecules to absorb light at unique areas of the visible spectrum. In general, chlorophylls absorb most strongly in the violet-blue region of the visible light spectrum and reflect light in the green region of the spectrum. This is why plant parts that contain chlorophyll appear green to the human eye.

In addition to chlorophylls, most photosynthetic organisms contain accessory pigments that allow plants to trap additional light energy from other areas of the visible spectrum. One such group of accessory pigments is the carotenoids. Carotenoids, such as ß-carotene (beta-carotene), absorb light mainly in the blue and green regions of the spectrum, while reflecting most light in the yellow, orange, and red regions. Carotenoids produce the colors of carrots and sweet potatoes.

Chlorophylls are more abundant than other pigments in leaves, and thus hide the colors of the other pigments. However, autumn in certain parts of the United States can bring out shades of yellow, red, and orange as the leaves turn colors, as shown in Figure 8.7. As trees prepare to lose their leaves before winter, the chlorophyll molecules break down, revealing the colors of the other pigments.

Electron transport

The structure of the thylakoid membrane is the key to the efficient energy transfer during electron transport. Thylakoid membranes have a large surface area, which provides the space needed to hold large numbers of electron-transporting molecules and two types of protein complexes called photosystems. Photosystem I and photosystem II contain light-absorbing pigments and proteins that play important roles in the light reactions. Follow along in Figure 8.8 as you continue to read about electron transport.

•First, the light energy excites electrons in photosystem II. The light energy also causes a water molecule to split, releasing an electron into the electron transport system, a hydrogen ion (H+)—also called a proton—into the thylakoid space, and oxygen (O2) as a waste product. This breakdown of water is essential for **photosynthesis** to occur.

•The excited electrons move from photosystem II to an electron-acceptor molecule in the thylakoid membrane.

•Next, the electron-acceptor molecule transfers the electrons along a series of electron-carriers to photosystem I.

•In the presence of light, photosystem I transfers the electrons to a protein called ferrodoxin. The electrons lost by photosystem I are replaced by electrons shuttled from photosystem II.

•Finally, ferrodoxin transfers the electrons to the electron carrier

NADP+, forming the energy-storage molecule NADPH.

Chemiosmosis ATP is produced in conjunction with electron transport by the process of chemiosmosis—the mechanism by which ATP is produced as a result of the flow of electrons down a concentration gradient. The breakdown of water is not only essential for providing the electrons that initiate the electron transport chain, but also for providing the protons (H+) necessary to drive ATP synthesis during chemiosmosis. The H+ released during electron transport accumulate in the interior of the thylakoid. As a result of a high concentration of H+ in the thylakoid interior and a low concentration of H+ in the stroma, H+ diffuse down their concentration gradient out of the thylakoid interior into the stroma through ion channels spanning the membrane, as shown in Figure 8.8. These channels are enzymes called ATP synthases. As H+ moves through ATP synthase, ATP is formed in the **stroma**.

Phase Two: The Calvin Cycle

Although NADPH and ATP provide cells with large amounts of energy, these molecules are not stable enough to store chemical energy for long periods of time. Thus, there is a second phase of **photosynthesis** called the **Calvin cycle** in which energy is stored in organic molecules such as glucose. The reactions of the Calvin cycle are also referred to as the light-independent reactions. Follow along in Figure 8.9 as you learn the steps of the Calvin cycle.

•In the first step of the **Calvin cycle** called carbon fixation, six carbon dioxide (CO2) molecules combine with six 5-carbon compounds to form twelve 3-carbon molecules called 3-phosphoglycerate (3-PGA). The joining of carbon dioxide with other organic molecules is called carbon fixation.

•In the second step, the chemical energy stored in ATP and NADPH is transferred to the 3-PGA molecules to form high-energy molecules called glyceraldehyde 3-phosphates (G3P). ATP supplies the phosphate groups for forming G3P molecules, while NADPH supplies hydrogen ions and electrons.

•In the third step, two G3P molecules leave the **cycle** to be used for the production of glucose and other organic compounds.

•In the final step of the **Calvin cycle**, an enzyme called rubisco converts the remaining ten G3P molecules into 5-carbon molecules called ribulose 1, 5-bisphosphates (RuBP). These molecules combine with new carbon dioxide molecules to continue the cycle.

Because rubisco converts inorganic carbon dioxide molecules into organic molecules that can be used by the cell, it is considered one of the most important biological enzymes. Plants use the sugars formed during the **Calvin cycle** both as a source of energy and as building blocks for complex carbohydrates, including cellulose, which provides structural support for the plant.

Alternative Pathways

The environment in which an organism lives can impact the organism’s ability to carry out **photosynthesis**. Environments in which the amount of water or carbon dioxide available is insufficient can decrease the ability of a photosynthetic organism to convert light energy into chemical energy. For example, plants in hot, dry environments are subject to excessive water loss that can lead to decreased **photosynthesis**. Many plants in extreme climates have alternative photosynthesis pathways to maximize energy conversion.

C4 plants

One adaptive pathway that helps plants maintain **photosynthesis** while minimizing water loss is called the C4 pathway. The C4 pathway occurs in plants such as sugar cane and corn. These plants are called C4 plants because they fix carbon dioxide into four-carbon compounds instead of three-carbon molecules during the **Calvin cycle**. C4 plants also have significant structural modifications in the arrangement of cells in the leaves. In general, C4 plants keep their stomata (plant cell pores) closed during hot days, while the four carbon compounds are transferred to special cells where CO2 enters the **Calvin cycle**. This allows for sufficient carbon dioxide uptake, while simultaneously minimizing water loss.

CAM plants

Another adaptive pathway used by some plants to maximize photosynthetic activity is called crassulacean acid metabolism (**CAM photosynthesis**). The CAM pathway occurs in water-conserving plants that live in deserts, salt marshes, and other environments where access to water is limited. CAM plants, such as cacti, orchids, and the pineapple in Figure 8.10, allow carbon dioxide to enter the leaves only at night, when the atmosphere is cooler and more humid. At night, these plants fix carbon dioxide into organic compounds. During the day, carbon dioxide is released from these compounds and enters the **Calvin cycle**. This pathway also allows for sufficient carbon dioxide uptake, while minimizing water loss.

Cellular Respiration

Overview of Cellular Respiration

Recall that organisms obtain energy in a process called cellular respiration. The function of **cellular respiration** is to harvest electrons from carbon compounds, such as glucose, and use that energy to make ATP. ATP is used to provide energy for cells to do work. The overall chemical equation for cellular respiration is shown below. Notice the equation for cellular respiration is the opposite of the equation for photosynthesis.

C6H12O6 + 6O2 → 6CO2 + 6H2O + Energy

**Cellular respiration** occurs in two main parts: glycolysis and aerobic respiration. The first stage, glycolysis, is an anaerobic process. Anaerobic metabolic processes do not require oxygen. **Aerobic respiration** includes the **Krebs cycle** and electron transport and is an aerobic process. Aerobic metabolic processes require oxygen. Cellular respiration with aerobic respiration is summarized in Figure 8.11.

Glycolysis

Glucose is broken down in the cytoplasm through the process of glycolysis. Two molecules of ATP and two molecules of NADH are formed for each molecule of glucose that is broken down. Follow along with Figure 8.12 as you read about the steps of glycolysis.

First, two phosphate groups, derived from two molecules of ATP, are joined to glucose. Notice that some energy, two ATP, is required to start the reactions that will produce energy for the cell. The 6-carbon molecule is then broken down into two 3-carbon compounds. Next, two phosphates are added and electrons and hydrogen ions (H+) combine with two NAD+ molecules to form two NADH molecules. NAD+ is similar to NADP, an electron carrier used during **photosynthesis**.

Last, the two 3-carbon compounds are converted into two molecules of pyruvate. At the same time, four molecules of ATP are produced.

Krebs Cycle

Glycolysis has a net result of two ATP and two pyruvate. Most of the energy from the glucose is still contained in the pyruvate. In the presence of oxygen, pyruvate is transported into the **mitochondrial matrix**, where it is eventually converted to carbon dioxide. The series of reactions in which pyruvate is broken down into carbon dioxide is called the Krebs cycle or tricarboxylic acid (TCA) cycle. This cycle also is referred to as the citric acid cycle.

Steps of the Krebs cycle

Prior to the Krebs cycle, pyruvate first reacts with coenzyme A (CoA) to form a 2-carbon intermediate called acetyl CoA. At the same time, carbon dioxide is released and NAD+ is converted to NADH. Acetyl CoA then moves to the **mitochondrial matrix**. The reaction results in the production of two carbon dioxide molecules and two NADH. Follow along in Figure 8.13 as you continue reading about the steps of the Krebs cycle.

•The **Krebs cycle** begins with acetyl CoA combining with a 4-carbon compound to form a 6-carbon compound known as citric acid.

•Citric acid is then broken down in the next series of steps, releasing two molecules of carbon dioxide and generating one ATP, three NADH, and one FADH2. FAD is another electron carrier similar to NAD+ and NADP+.

•Finally, acetyl CoA and citric acid are generated and the cycle continues.

Recall that two molecules of pyruvate are formed during glycolysis, resulting in two “turns” of the Krebs cycle for each glucose molecule. The net yield from the **Krebs cycle** is six carbon dioxide molecules, two ATP, eight NADH, and two FADH2. NADH and FADH2 move on to play a significant role in the next stage of aerobic respiration.

Electron Transport

In aerobic respiration, electron transport is the final step in the breakdown of glucose. It also is the point at which most of the ATP is produced. High-energy electrons and hydrogen ions from NADH and FADH2 produced in the **Krebs cycle** are used to convert ADP to ATP.

As shown in Figure 8.14, electrons move along the **mitochondrial membrane** from one protein to another. As NADH and FADH2 release electrons, the energy carriers are converted to NAD+ and FAD, and H+ ions are released into the **mitochondrial matrix**. The H+ ions are pumped into the **mitochondrial matrix** across the inner mitochondrial membrane. H+ ions then diffuse down their concentration gradient back across the membrane and into the matrix through **ATP synthase molecules** in chemiosmosis. Electron transport and chemiosmosis in cellular respiration are similar to these processes in **photosynthesis**. Oxygen is the final electron acceptor in the electron transport system in **cellular respiration**. Protons and electrons are transferred to oxygen to form water.

Overall, electron transport produces 24 ATP. Each NADH molecule produces three ATP and each group of three FADH2 produces two ATP. In eukaryotes, one molecule of glucose yields 36 ATP.

Prokaryotic cellular respiration

Some prokaryotes also undergo aerobic respiration. Because prokaryotes do not have mitochondria, there are a few differences in the process. The main difference involves the use of the prokaryotic cellular membrane as the location of electron transport. In **eukaryotic cells**, pyruvate is transported to the mitochondria. In **prokaryotes**, this movement is unnecessary, saving the prokaryotic cell two ATP, and increasing the net total of ATP produced to 38.

Anaerobic Respiration

Some cells can function for a short time when oxygen levels are low. Some prokaryotes are anaerobic organisms—they grow and reproduce without oxygen. In some cases these cells continue to produce ATP through glycolysis. However, there are problems with solely relying on glycolysis for energy. Glycolysis only provides two net ATP for each molecule of glucose, and a cell has a limited amount of NAD+. Glycolysis will stop when all the NAD+ is used up if there is not a process to replenish NAD+. The anaerobic pathway that follows glycolysis is anaerobic respiration, or fermentation.

Fermentation occurs in the cytoplasm and regenerates the cell’s supply of NAD+ while producing a small amount of ATP. The two main types of fermentation are lactic acid fermentation and alcohol fermentation.

Lactic acid fermentation

In lactic acid fermentation, enzymes convert the pyruvate made during glycolysis to lactic acid, as shown in Figure 8.15. This involves the transfer of high-energy electrons and protons from NADH. Skeletal muscle produces lactic acid when the body cannot supply enough oxygen, such as during periods of strenuous exercise. When lactic acid builds up in muscle cells, muscles become fatigued and might feel sore. Lactic acid also is produced by several microorganisms that often are used to produce many foods, including cheese, yogurt, and sour cream.

Alcohol fermentation Alcohol fermentation occurs in yeast and some bacteria. Figure 8.15 shows the chemical reaction that occurs during alcohol fermentation when pyruvate is converted to ethyl alcohol and carbon dioxide. Similar to lactic acid fermentation, NADH donates electrons during this reaction and NAD+ is regenerated.

Photosynthesis and Cellular Respiration

As you have learned, photosynthesis and cellular respiration are two important processes that cells use to obtain energy. They are metabolic pathways that produce and break down simple carbohydrates.

Figure 8.16 shows how these two processes are related. Recall that the products of photosynthesis are oxygen and glucose—the reactants needed for cellular respiration. The products of **cellular respiration**— carbon dioxide and water—are the reactants for **photosynthesis**.

Cellular Growth

Cell Size Limitations

Most cells are less than 100 µm (100 × 10–6 m) in diameter, which is smaller than the period at the end of this sentence. Why are most cells so small? This section investigates several factors that influence cell size.

Ratio of surface area to volume

The key factor that limits the size of a cell is the ratio of its surface area to its volume. The surface area of the cell refers to the area covered by the plasma membrane.

Recall from Chapter 7 that the plasma membrane is the structure through which all nutrients and waste products must pass. The volume refers to the space taken by the inner contents of the cell, including the organelles in the cytoplasm and the nucleus.

To illustrate the ratio of surface area to volume, consider the small cube in Figure 9.1, which has sides of one micrometer (µm) in length. This is approximately the size of a bacterial cell. To calculate the surface area of the cube, multiply length times width times the number of sides (1 µm × 1 µm × 6 sides), which equals 6 µm2. To calculate the volume of the cell, multiply length times width times height (1 µm × 1 µm × 1 µm), which equals 1 µm3. The ratio of surface area to volume is 6:1.

If the cubic cell grows to 2 µm per side, as represented in Figure 9.1, the surface area becomes 24 µm2 and the volume is 8 µm3. The ratio of surface area to volume is now 3:1, which is less than it was when the cell was smaller. If the cell continues to grow, the ratio of surface area to volume will continue to decrease, as shown by the third cube in Figure 9.1. As the cell grows, its volume increases much more rapidly than the surface area. This means that the cell might have difficulty supplying nutrients and expelling enough waste products. By remaining small, cells have a higher ratio of surface area to volume and can sustain themselves more easily.

Transport of substances

Another task that can be managed more easily in a small cell than in a large cell is the movement of substances. Recall that the plasma membrane controls cellular transport because it is selectively permeable. Once inside the **cell**, substances move by diffusion or by motor proteins pulling them along the cytoskeleton. Diffusion over large distances is slow and inefficient because it relies on random movement of molecules and ions. Similarly, the cytoskeleton transportation network, shown in Figure 9.2, becomes less efficient for a cell if the distance to travel becomes too large. Therefore, cells remain small to maximize the ability of diffusion and motor proteins to transport nutrients and waste products. Small cells maintain more efficient transport systems.

Cellular communications

The need for signaling proteins to move throughout the cell also limits cell size. In other words, cell size affects the ability of the cell to communicate instructions for cellular functions. If the cell becomes too large, it becomes almost impossible for cellular communications, many of which involve movement of substances and signals to various organelles, to take place efficiently. For example, the signals that trigger protein synthesis might not reach the ribosome fast enough for protein synthesis to occur to sustain the cell.

The Cell Cycle

Once a cell reaches its size limit, something must happen—either it will stop growing or it will divide. Most cells will eventually divide.

Cell division not only prevents the cell from becoming too large, but it also is the way the cell reproduces so that you grow and heal certain injuries. Cells reproduce by a cycle of growing and dividing called the **cell cycle**. Each time a cell goes through one complete cycle, it becomes two cells. When the **cell cycle** is repeated continuously, the result is a continuous production of new cells. A general overview of the cell cycle is presented in Figure 9.3.

There are three main stages of the **cell cycle**. **Interphase** is the stage during which the cell grows, carries out cellular functions, and replicates, or makes copies of its DNA in preparation for the next stage of the cycle. Interphase is divided into three substages, as indicated by the segment arrows in Figure 9.3. Mitosis is the stage of the **cell cycle** during which the cell’s nucleus and nuclear material divide. Mitosis is divided into four substages. Near the end of mitosis, a process called cytokinesis begins. Cytokinesis is the method by which a cell’s cytoplasm divides, creating a new cell. You will read more about mitosis and cytokinesis in Section 9.2.

The duration of the cell cycle varies, depending on the cell that is dividing. Some eukaryotic cells might complete the cycle in as few as eight minutes, while other cells might take up to one year. For most normal, actively dividing animal cells, the **cell cycle** takes approximately 12–24 hours. When you consider all that takes place during the **cell cycle**, you might find it amazing that most of your cells complete the cell cycle in about a day.

The stages of interphase

During interphase, the cell grows, develops into a mature, functioning cell, duplicates its DNA, and prepares for division. Interphase is divided into three stages, as shown in Figure 9.3: G1, S, and G2, also called Gap 1, synthesis, and Gap 2.

The first stage of interphase, **G1**, is the period immediately after a cell divides. During G1, a cell is growing, carrying out normal cell functions, and preparing to replicate DNA. Some cells, such as muscle and nerve cells, exit the **cell cycle** at this point and do not divide again.

The second stage of interphase, **S**, is the period when a cell copies its DNA in preparation for cell division. Chromosomes are the structures that contain the genetic material that is passed from generation to generation of cells. Chromatin is the relaxed form of DNA in the cell’s nucleus. As shown in Figure 9.4, when a specific dye is applied to a cell in interphase, the nucleus stains with a speckled appearance. This speckled appearance is due to individual strands of chromatin that are not visible under a **light microscope** without the dye.

The **G2 stage** follows the S stage and is the period when the cell prepares for the division of its nucleus. A protein that makes microtubules for cell division is synthesized at this time. During **G2**, the cell also takes inventory and makes sure it is ready to continue with mitosis. When these activities are completed, the cell begins the next stage of the cell cycle—mitosis.

Mitosis and cytokinesis

The stages of mitosis and cytokinesis follow interphase. In mitosis, the cell’s nuclear material divides and separates into opposite ends of the cell. In cytokinesis, the cell divides into two daughter cells with identical nuclei. These important stages of the cell cycle are described in Section 9.2.

Prokaryotic cell division

The **cell cycle** is the method by which eukaryotic cells reproduce themselves. Prokaryotic cells, which you have learned are simpler cells, reproduce by a method called binary fission. You will learn more about binary fission in Chapter 18.

Mitosis and Cytokinesis

Mitosis

You learned in the last section that cells cycle through **interphase**, mitosis, and cytokinesis. During mitosis, the cell’s replicated genetic material separates and the cell prepares to split into two cells. The key activity of mitosis is the accurate separation of the cell’s replicated DNA.

This enables the cell’s genetic information to pass into the new cells intact, resulting in two daughter cells that are genetically identical. In multicellular organisms, the process of mitosis increases the number of cells as a young organism grows to its adult size. Organisms also use mitosis to replace damaged cells. Recall the last time you accidently got cut. Under the scab, the existing skin cells divided by mitosis and cytokinesis to create new skin cells that filled the gap in the skin caused by the injury.

The Stages of Mitosis

Like interphase, mitosis is divided into stages: **prophase, metaphase, anaphase, and telophase**.

Prophase

The first stage of mitosis—the stage of mitosis during which a dividing cell spends the most time—is called **prophase**. In this stage, the cell’s chromatin tightens, or condenses, into chromosomes.

In **prophase**, the chromosomes are shaped like an X, as shown in Figure 9.5. At this point, each chromosome is a single structure that contains the genetic material that was replicated in **interphase**. Each half of this X is called a sister chromatid. Sister chromatids are structures that contain identical copies of DNA. The structure at the center of the chromosome where the sister chromatids are attached is called the **centromere**. This structure is important because it ensures that a complete copy of the replicated DNA will become part of the daughter cells at the end of the cell cycle. Locate prophase in the cell cycle illustrated in Figure 9.6, and note the position of the sister chromatids.

As **prophase** continues, the nucleolus seems to disappear. Microtubule structures called spindle fibers form in the **cytoplasm**. In **animal cells and most protist cells**, another pair of microtubule structures, called centrioles, migrates to the ends, or poles, of the cell. Coming out of the centrioles are yet another type of microtubule called aster fibers, which have a starlike appearance. The whole structure, including the spindle fibers, centrioles, and aster fibers, is called the spindle apparatus and is shown in Figure 9.7. The spindle apparatus is important in moving and organizing the chromosomes before cell division. Centrioles are not part of the spindle apparatus in plant cells—only spindle fibers are present.

Near the end of **prophase**, the nuclear envelope seems to disappear. The spindle fibers attach to the sister chromatids of each chromosome on both sides of the centromere and then attach to opposite poles of the cell. This arrangement ensures that each new cell receives one complete copy of the DNA.

Metaphase During the second stage of mitosis, **metaphase**, the sister chromatids are pulled by motor proteins along the spindle apparatus toward the center of the cell and line up in the middle, or equator, of the cell, as shown in Figure 9.8. **Metaphase** is one of the shortest stages of mitosis, but when completed successfully, it ensures that the new cells have accurate copies of the chromosomes.

Anaphase

The chromatids are pulled apart during **anaphase**, the third stage of mitosis. In **anaphase**, the microtubules of the spindle apparatus begin to shorten. This shortening pulls at the centromere of each sister chromatid, causing the sister chromatids to separate into two identical chromosomes. All of the sister chromatids separate simultaneously, although the exact mechanism that controls this is unknown. At the end of **anaphase**, the microtubules, with the help of motor proteins, move the chromosomes toward the poles of the cell.

Telophase

The last stage of mitosis is called telophase. **Telophase** is the stage of mitosis during which the chromosomes arrive at the poles of the cell and begin to relax, or decondense. As shown in Figure 9.9, two new nuclear membranes begin to form and the nucleoli reappear.

The spindle apparatus disassembles and some of the microtubules are recycled by the cell to build various parts of the cytoskeleton. Although the four stages of mitosis are now complete and the nuclear material is divided, the process of cell division is not yet complete.

Cytokinesis

Toward the end of mitosis, the cell begins another process called cytokinesis that will divide the cytoplasm. This results in two cells, each with identical nuclei. During the later phases of mitosis, microtubules are formed that will be involved in cytokinesis. In **animal cells**, cytokinesis is accomplished by using microfilaments to constrict, or pinch, the cytoplasm, as shown in Figure 9.10.

Recall from Chapter 7 that plant cells have a rigid cell wall covering their plasma membrane. Instead of pinching in half, a new structure, called a cell plate, forms between the **two daughter nuclei**, as illustrated in Figure 9.10. Cell walls then form on either side of the cell plate. Once this new wall is complete, there are two genetically identical cells.

Prokaryotic cells, which divide by binary fission, finish cell division in a different way. When prokaryotic DNA is duplicated, both copies attach to the plasma membrane. As the plasma membrane grows, the attached DNA molecules are pulled apart. The cell completes fission, producing two new prokaryotic cells.

Cell Cycle Regulation

Normal Cell Cycle

The timing and rate of cell division are important to the health of an organism. The rate of cell division varies depending on the type of cell. A mechanism involving proteins and enzymes controls the **cell cycle**.

The role of cyclins

To start a car, it takes a combination of a key turning in the ignition to signal the engine to start. Similarly, the **cell cycle** in eukaryotic cells is driven by a combination of two substances that signal the cellular reproduction processes. Proteins called cyclins bind to enzymes called cyclin-dependent kinases (CDKs) in the stages of interphase and mitosis to start the various activities that take place in the **cell cycle**. Different cyclin/CDK combinations control different activities at different stages in the **cell cycle**. Figure 9.11 illustrates where some of the important combinations are active.

In the **G1 stage** of interphase, the combination of cyclin with CDK signals the start of the **cell cycle**. Different cyclin/CDK combinations signal other activities, including DNA replication, protein synthesis, and nuclear division throughout the **cell cycle**. The same cyclin/CDK combination also signals the end of the cell cycle.

Quality control checkpoints

Recall the process of starting a car. Many manufacturers use a unique microchip in the key to ensure that only a specific key will start each car. This is a checkpoint against theft. The **cell cycle** also has built-in checkpoints that monitor the cycle and can stop it if something goes wrong. For example, a check-point near the end of the **G1 stage** monitors for DNA damage and can stop the cycle before entering the **S stage** of interphase. There are other quality control checkpoints during the **S stage** and after DNA replication in the **G2 stage**. Spindle checkpoints also have been identified in mitosis. If a failure of the spindle fibers is detected, the **cycle** can be stopped before cytokinesis. Figure 9.11 shows the location of key checkpoints in the cell cycle.

Abnormal Cell Cycle: Cancer

Although the **cell cycle** has a system of quality control checkpoints, it is a complex process that sometimes fails. When cells do not respond to the normal **cell cycle** control mechanisms, a condition called cancer can result. Cancer is the uncontrolled growth and division of cells—a failure in the regulation of the cell cycle. When unchecked, cancer cells can kill an organism by crowding out normal cells, resulting in the loss of tissue function. Cancer cells spend less time in **interphase** than do normal cells, which means cancer cells grow and divide unrestrained as long as they are supplied with essential nutrients. Figure 9.12 shows how cancer cells can intrude on normal cells.

Causes of cancer

Cancer does not just occur in a weak organism. In fact, cancer occurs in many healthy, active, and young organisms. The changes that occur in the regulation of cell growth and division of cancer cells are due to mutations or changes in the **segments of DNA** that control the production of proteins, including proteins that regulate the **cell cycle**. Often, the genetic change or damage that occurs is repaired by various repair systems. But if the repair systems fail, cancer can result. Various environmental factors can affect the occurrence of cancer cells. Substances and agents that are known to cause cancer are called carcinogens.

Although not all cancers can be prevented, avoiding known carcinogens can help reduce the risk of cancer. A governmental agency called the Food and Drug Administration (FDA) works to make sure that the things you eat and drink are safe. The FDA also requires labels and warnings for products that might be carcinogens. Industrial laws help protect people from exposure to cancer-causing chemicals, such as asbestos, in the workplace. For example, asbestos has been removed from many old buildings to protect people living and working inside them. Avoiding tobacco of all kinds, even secondhand smoke and smokeless tobacco, can reduce the risk of cancer.

Some radiation, such as ultraviolet radiation from the Sun, is impossible to avoid completely. There is a connection between the amount of ultraviolet radiation to which a person is exposed and the risk of developing skin cancer. Therefore, sunscreen is recommended for everyone who is exposed to the Sun. Other forms of radiation, such as X rays, are used for medical purposes, such as to look at a broken bone or check for tooth cavities. To protect against exposure, you might have worn a heavy lead apron when an X ray was taken.

Cancer genetics More than one change in DNA is required to change an abnormal cell into a cancer cell. Over time, it is possible that there might be many changes in DNA. This might explain why the risk of cancer increases with age. The fact that multiple changes must occur also might explain why cancer runs in some families. An individual who inherits one or more changes from a parent is at a higher risk for developing cancer than someone who does not inherit these changes.

Apoptosis

Not every cell is destined to survive. When an embryo divides, some cells go through a process called apoptosis, or programmed cell death. Cells going through apoptosis actually shrink and shrivel in a controlled process. All animal cells appear to have a “death program” that can be activated. One example of apoptosis occurs during the development of the human hand and foot.

When the hands and feet begin to develop, cells occupy the spaces between the fingers and toes. Normally, this tissue undergoes apoptosis, with the cells shriveling and dying at the appropriate time so that the webbing is not present in the mature organism. An example of apoptosis in plants is the localized death of cells that results in leaves falling from trees during autumn. Apoptosis also occurs in cells that are damaged beyond repair, including cells with DNA damage that could lead to cancer. Apoptosis can help to protect organisms from developing cancerous growths.

Stem Cells

The majority of cells in a multicellular organism are designed for a specialized function. Some cells might be part of your skin, and other cells might be part of your heart. In 1998, scientists discovered a way to isolate a unique type of cell in humans called the stem cell. Stem cells are unspecialized cells that can develop into specialized cells when under the right conditions, as illustrated in Figure 9.13. Stem cells can remain in an organism for many years while undergoing cell division. There are two basic types of stem cells: embryonic stem cells and adult stem cells.

Embryonic stem cells After a sperm fertilizes an egg, the resulting mass of cells divides repeatedly until there are about 100–150 cells.

These cells have not become specialized and are called embryonic stem cells. If separated, each of these cells has the capability of developing into a wide variety of specialized cells. If the embryo continues to divide, the cells specialize into various tissues, organs, and organ systems. Embryonic stem cell research is controversial because of ethical concerns about the source of the cells.

Adult stem cells

The second type of stem cells, adult stem cells, is found in various tissues in the body and might be used to maintain and repair the same kind of tissue in which they are found. The term “adult stem cells” might be somewhat misleading because even a newborn has adult stem cells. Like embryonic stem cells, certain kinds of adult stem cells also might be able to develop into different kinds of cells, providing new treatments for many diseases and conditions. In 1999, researchers at Harvard Medical School used nervous system stem cells to restore lost brain tissue in mice. In 2000, a team of researchers at the University of Florida used pancreatic stem cells to restore pancreas function in a mouse with diabetes. Research with adult stem cells, like that shown in Figure 9.14, is much less controversial because the adult stem cells can be obtained with the consent of their donor.

Meiosis

This variety of characteristics is a result of two sex cells combining during sexual reproduction.

Chromosomes and Chromosome Number

Each student in your biology class has characteristics passed on to them by their parents. Each characteristic, such as hair color, height, or eye color, is called a trait. The instructions for each trait are located on chromosomes, which are found in the nucleus of cells. The DNA on chromosomes is arranged in segments that control the production of proteins. These DNA segments are called genes. Each chromosome consists of hundreds of genes, each gene playing an important role in determining the characteristics and functions of the cell.

Homologous chromosomes Human body cells have 46 chromosomes. Each parent contributes 23 chromosomes, resulting in 23 pairs of chromosomes. The chromosomes that make up a pair, one chromosome from each parent, are called homologous chromosomes. As shown in Figure 10.1, homologous chromosomes in body cells have the same length and the same centromere position, and they carry genes that control the same inherited traits. For instance, the gene for earlobe type will be located at the same position on both homologous chromosomes. Although these genes each code for earlobe type, they might not code for the exact same type of earlobe.

Haploid and diploid cells In order to maintain the same chromosome number from generation to generation, an organism produces gametes, which are sex cells that have half the number of chromosomes. Although the number of chromosomes varies from one species to another, in humans each gamete contains 23 chromosomes. The symbol n can be used to represent the number of chromosomes in a gamete. A cell with n number of chromosomes is called a haploid cell. Haploid comes from the Greek word haploos, meaning single.

The process by which one haploid gamete combines with another haploid gamete is called fertilization. As a result of fertilization, the cell now will contain a total of 2n chromosomes—n chromosomes from the female parent plus n chromosomes from the male parent. A cell that contains 2n number of chromosomes is called a diploid cell.

Notice that n also describes the number of pairs of chromosomes in an organism. When two human gametes combine, 23 pairs of homologous chromosomes are formed.

Meiosis I

Gametes are formed during a process called meiosis, which is a type of cell division that reduces the number of chromosomes; therefore, it is referred to as a reduction division. Meiosis occurs in the reproductive structures of organisms that reproduce sexually. While mitosis maintains the chromosome number, meiosis reduces the chromosome number by half through the separation of homologous chromosomes. A cell with 2n number of chromosomes will have gametes with n number of chromosomes after meiosis, as illustrated in Figure 10.2. Meiosis involves two consecutive cell divisions called meiosis I and meiosis II.

Interphase Recall that the cell cycle includes interphase prior to mitosis. Cells that undergo meiosis also go through interphase as part of the **cell cycle**. Cells in interphase carry out various metabolic processes, including the replication of DNA and the synthesis of proteins.

Prophase I As a cell enters **prophase I**, the replicated chromosomes become visible. As in mitosis, the replicated chromosomes consist of two sister chromatids. As the homologous chromosomes condense, they begin to form pairs in a process called synapsis. The homologous chromosomes are held tightly together along their lengths, as illustrated in Figure 10.3. Notice that in Figure 10.4 the purple and green chromosomes have exchanged segments. This exchange occurs during synapsis. Crossing over is a process during which chromosomal segments are exchanged between a pair of homologous chromosomes.

As **prophase I** continues, centrioles move to the cell’s opposite poles. Spindle fibers form and bind to the sister chromatids at the **centromere**.

Metaphase I In the next phase of meiosis, the pairs of homologous chromosomes line up at the **equator of the cell**, as illustrated in Figure 10.5.

In meiosis, the spindle fibers attach to the centromere of each homologous chromosome. Recall that during **metaphase** in mitosis, the individual chromosomes, which consist of two sister chromatids, line up at the **cell’s equator**. During **metaphase I** of meiosis, the homologous chromosomes line up as pairs at the **cell’s equator**. This is an important distinction between mitosis and meiosis.

Anaphase I During **anaphase I**, the homologous chromosomes separate, which is also illustrated in Figure 10.5. Each member of the pair is guided by spindle fibers and moves toward opposite poles of the cell. The chromosome number is reduced from 2n to n when the homologous chromosomes separate. Recall that in mitosis, the sister chromatids split during **anaphase**. During **anaphase I** of meiosis, however, each homologous chromosome still consists of two sister chromatids.

Telophase I

The homologous chromosomes, consisting of two sister chromatids, reach the cell’s opposite poles. Each pole contains only one member of the original pair of homologous chromosomes. Notice in Figure 10.5 that each chromosome still consists of two sister chromatids joined at the centromere. The sister chromatids might not be identical because crossing over might have occurred during synapsis in **prophase I**.

During **telophase I**, cytokinesis usually occurs, forming a furrow by pinching in animal cells and by forming a cell plate in plant cells. Following cytokinesis, the cells may go into interphase again before the second set of divisions. However, the DNA is not replicated again during this interphase. In some species, the chromosomes uncoil, the nuclear membrane reappears, and nuclei reform during **telophase I**.

Meiosis II

Meiosis is only halfway completed at the end of meiosis I. During **prophase II**, a second set of phases begins as the spindle apparatus forms and the chromosomes condense. During **metaphase II**, the chromosomes are positioned at the equator by the spindle fibers, as shown in Figure 10.5. During **metaphase** of mitosis, a diploid number of chromosomes line up at the equator. During **metaphase II** of meiosis, however, a haploid number of chromosomes line up at the equator. During **anaphase II**, the sister chromatids are pulled apart at the **centromere**  by the spindle fibers, and the sister chromatids move toward the **opposite poles of the cell**. The chromosomes reach the poles during **telophase II**, and the nuclear membrane and nuclei reform. At the end of meiosis II, cytokinesis occurs, resulting in four haploid cells, each with n number of chromosomes, as illustrated in Figure 10.5.

Meiosis provides variation Recall that pairs of homologous chromosomes line up at the equator during **prophase I**. How the chromosomes line up at the equator is a random process that results in gametes with different combinations of chromosomes, such as the ones in Figure 10.6.

Depending on how the chromosomes line up at the equator, four gametes with four different combinations of chromosomes can result.

Notice that the first possibility shows which chromosomes were on the same side of the equator and therefore traveled together. Different combinations of chromosomes were lined up on the same side of the equator to produce the gametes in the second possibility. Genetic variation also is produced during crossing over and during fertilization, when gametes randomly combine.

Sexual Reproduction v. Asexual Reproduction

Some organisms reproduce by asexual reproduction, while others reproduce by sexual reproduction. The life cycles of still other organisms might involve both asexual and sexual reproduction. During asexual reproduction, the organism inherits all of its chromosomes from a single parent. Therefore, the new individual is genetically identical to its parent. Bacteria reproduce asexually, whereas most protists reproduce both asexually and sexually, depending on environmental conditions. Most plants and many of the more simple animals can reproduce both asexually and sexually, compared to more advanced animals that reproduce only sexually.

Why do some species reproduce sexually while others reproduce asexually? Recent studies with fruit flies have shown that the rate of accumulation of beneficial mutations is faster when species reproduce sexually than when they reproduce asexually. In other words, when reproduction occurs sexually, the beneficial genes multiply faster over time than they do when reproduction is asexual.

**Textbook 5: Focus on Life**

Cells and Life

Cells are the smallest unit of life.

Real-World Reading Connection People once thought Earth was flat because they did not have tools to discover that it is round. People also had many wrong ideas about living things on Earth. They did not have the tools to observe very small living things.

Early Ideas about Cells

Most cells are so small, as shown in Figure 1, that you cannot see them without some type of magnifying device. There even was a time when people did not know that cells existed. People also once believed that an egg contained a miniature version of an adult organism. They thought the organism’s structures just had to increase in size as the organism grew.

Early Microscopes

After the invention of the light microscope, around 1600, ideas about living things changed. A light microscope uses light and has one or more lenses that enlarges an image of something.

Modern Microscopes

Most of the structures within a cell are too small to be observed even with a **light microscope**. The best **light microscopes** can only enlarge images of objects up to about 1,500 times their original size. With the invention of the electron microscope in the 1930s, scientists were able to see most structures inside a cell, like those shown in Figure 2. **An electron microscope** can enlarge images 100,000 times or more. Improved types of electron microscopes include one that can produce images of atoms on or in surfaces of materials.

The Cell theory

Even after the invention of microscopes, scientists were only beginning to understand how cells relate to living things. In the 1830s, a German scientist observed that all plant parts are made of cells. Around the same time, another German scientist made the same observation about animals. Nearly two decades later, a German physician proposed that all cells come from preexisting cells. Together, these ideas became known as the **cell theory**, which is listed below.

All organisms are made of one or more cells.

The cell is the smallest unit of life.

All new cells come from preexisting cells.

Characteristics of Life

Suppose your class took a field trip to a place like the one shown in Figure 3. Your teacher asks you to identify living and nonliving things in the environment. Could you complete this assignment?

From observations and evidence gathered over time, scientists agree that all living things, or organisms, have common characteristics. Whether made of one cell or many cells, organisms are organized, respond, grow and develop, reproduce, maintain certain internal conditions, and use energy.

Organization

The rooms and things in your home are organized in some way.

The oven and refrigerator are in the kitchen, a sink and toilet are in the bathroom, and clothes and shoes are in the closets. Cells and organisms also are organized. Cells contain structures that have specialized functions similar to the way a house has rooms for different activities. Some organisms have a system of nerves controlled by a brain, much like a house has a system of electrical wires controlled by a circuit-breaker box. You will read more about the organization inside individual cells in the next lesson, and about the organization within organisms in Chapter 2.

Responses

If someone throws a ball at you, you might try to catch it. This is because you are able to respond to changes in your environment. Another example of the way living things respond is the way your body responds to an invasion by a disease-causing virus or bacterium. There are cells in your body that can recognize these invaders and respond with different processes to get rid of them.

Growth and Development

Humans grow and develop just as all organisms do. When organisms grow, they increase in size. Growth in multicellular organisms—organisms made of many cells—usually happens as the number of cells increases. An organism that is only one cell also grows because the cell increases in size.

Development includes all the changes that occur in an organism. For example, you might now be able to play a musical instrument or some sport that you could not play ten years ago. Some organisms have extraordinary changes over their lifetime, such as the butterfly shown in Figure 4.

Reproduction

You read earlier in this lesson that all cells come from preexisting cells. The same is true for organisms. In order for organisms to continue to exist, they must reproduce and create offspring similar to themselves. Not every organism must reproduce. However, for a type of organism to continue to exist, reproduction must occur among some organisms of that type. Some organisms must have a mate to reproduce but others can reproduce without a mate.

Homeostasis

Have you ever noticed that if you drink more water than usual, you have to use the restroom more often? That is because your body is maintaining homeostasis or trying to keep its internal conditions within certain limits. All organisms have the ability to maintain homeostasis, but the methods and needs vary depending on the organism and its environment. For example, many freshwater fish would not survive if placed in salt water because they would not be able to control the amount of salt in their bodies. A human maintains a body temperature of about 37°C by sweating, shivering, or changing the flow of blood.

Energy

You use energy when you look at the pages of this book, sit at your desk, and when your heart pumps blood throughout your body. Cells continuously use energy to transport substances, make new cells, and perform chemical reactions. Our cells get energy from the food we eat. This energy originally came to Earth from the Sun, as shown in Figure 5. The Sun provides energy for nearly all the organisms on Earth. In Lesson 3, you will read about how plants use light energy to make food.

Chemistry of a Cell

When you were younger, you might have played with some kind of building blocks. You probably made many things using different sizes and shapes of blocks. In a similar way, a cell can make different things using atoms and molecules as its building blocks. You might recall from another science class that atoms combine to make molecules. Most of the molecules in living things are made from six kinds of atoms: sulfur, nitrogen, potassium, hydrogen, oxygen, and carbon. The molecules in cells can combine in many ways to make different substances that are used for thousands of different functions.

Water—The Main Ingredient

Have you ever wondered what you are made of? About two-thirds of your body’s mass is water. Most of that water—67 per- cent—is inside cells and the rest surrounds cells. The water surrounding cells helps to maintain homeostasis because it helps to insulate your body. Water also can dissolve many different kinds of molecules. This enables your blood, which is mostly water, to transport substances throughout your body.

A unique property of water is that each water molecule has an area that is more negative—called the negative end—than the other area—called the positive end. As shown in Figure 6, the negative end of a water molecule is attracted to the positive end of another water molecule, similar to the way magnets are attracted to each other. This attraction is one reason why water can travel from the ground to leaves at the top of trees.

Basic Substances

Besides water, cells contain substances that can be classified as proteins, nucleic acids, lipids, or carbohydrates. They also are called macromolecules. The prefix macro- means large. Macromolecules are large complex molecules usually made of long chains of smaller molecules.

Proteins The molecules necessary for nearly everything cells and organisms do are proteins, such as the examples listed in Table 1. There are thousands of different proteins. Proteins are folded chains or groups of folded chains of molecules called amino acids. Each protein has a specific sequence of amino acids within its chains. They must be folded correctly for the protein to function properly. Proteins have many functions in organisms and some proteins have the same function in different species of organisms.

Nucleic Acids Proteins are important to all cells but they cannot be made without nucleic acids. Nucleic acids are long chains of molecules called nucleotides. One kind of nucleic acid is deoxyribonucleic acid (DNA). It consists of only four types of nucleotides, but there are billions of them in DNA. The arrangement of nucleotides in a cell’s DNA is a code that contains the cell’s genetic information, or genome. The genetic information in DNA is used to make another kind of nucleic acid—ribonucleic acid (RNA). It is RNA that is used to make proteins.

You will read more about DNA in Lesson 2 of this chapter and in other chapters of this book.

Lipids

Have you ever tried to mix oil and water? You might have noticed that they do not mix with each other. This is because oil is a lipid. A lipid is a large molecule that does not dissolve in water. The main kinds of lipids are fats, phospholipids, steroids, and waxes. Fat molecules store large amounts of chemical energy. Phospholipids, and cholesterol, a steroid, are important parts of cells. Waxes help reduce water loss and can form a barrier to invaders like the wax in our ears does.

Carbohydrates Fruits and candy contain different kinds of sugar. Breads and pasta are mostly starch. Vegetables have large amounts of fiber made of cellulose. The shell of a lobster is mostly made of a substance called chitin.

What do sugar, starch, cellulose, and chitin have in common? They are all carbohydrates. A carbohydrate stores energy and is made of one sugar molecule, a pair of sugar molecules, or a chain of sugar molecules. The energy in sugars and starches can be released quickly through chemical reactions in cells. Most cells cannot release the energy in cellulose and chitin. Carbohydrates make up the structural parts of cells.

What have you learned about cells and life?

The Cell

Cells have structures with specific functions.

Real-World Reading Connection Your body contains different structures that work together to keep you alive and healthy. For example, your skin protects your body, your stomach helps to digest food, and your brain controls your responses. Your cells also have structures that function in each cell.

Cell Shape and Movement

Cells in your body have a variety of shapes and sizes, as shown in Figure 7. Different shapes relate to different functions. For example, a human red blood cell easily passes into the smallest blood vessels. A nerve cell can send signals over long distances within your body. Other organisms also have cells with shapes that relate to their functions. Some plant cells are hollow and make up tubelike structures that carry water and dissolved substances.

Cell Membrane

Regardless of a cell’s shape and function, every cell has a flexible covering that surrounds it called the cell membrane. A membrane can be made of one or more layers of linked molecules. The cell membrane protects the inside of a cell from the environment outside the cell. Because of its specific chemical makeup, the cell membrane is selectively permeable. This means the cell membrane does not allow all types of substances into the cell. As shown in Figure 8, some things cannot enter a cell and others cannot leave a cell.

Cell Wall

Some cells, such as those in plants, fungi, and some bacteria, have a rigid cell wall that surrounds the cell outside its **cell membrane**. Plants and fungi can grow upward against the force of gravity because the rigid cell wall maintains the cell’s shape, supports, and protects the cell. Substances can pass freely through a cell wall, unlike the cell membrane. Cell walls of plants, as shown in Figure 9, are made mostly of cellulose, a carbohydrate. Humans cannot digest cellulose, but cellulose is an important part of our diets. Cellulose stimulates the production of mucus in our **intestines**, which helps food travel smoothly through them.

Cell Appendages

Some animals can run, hop, fly, or swim using appendages such as legs, wings, or fins. Cells can also have appendages. A flagellum is a tail-like appendage. Some single-celled organisms have one or more flagella. Many organisms produce sperm and each sperm moves by using its flagellum.

Cilia (SIH lee uh) (singular, cilium) are short, hairlike appendages. Cilia usually occur in large numbers on a cell, but a cell usually only has one flagellum. Some single-celled organisms move by the coordinated motion of cilia. Cilia also can be on the surface of a cell that does not move, as shown in Figure 10. In this case, the cilia help fluids move across the cell’s surface.

Cytoplasm and the Cytoskeleton

The inside of a cell contains cytoplasm—a thick fluid made mostly of water. The structures and substance that are inside a **cell**, as shown in Figure 9 and Figure 11, are suspended in the **cytoplasm**.

Your body contains a skeleton and muscles that allow you to move and maintain your shape. Individual cells do not have a skeleton of bones. Instead, cells have a network of fibers called the cytoskeleton. The cytoskeleton, shown in Figure 11, is like a thick web and plays a role in muscle contraction, cell division, cell movement, and maintenance of cell shape. Both cilia and flagella are able to move because they contain fibers of the cytoskeleton.

Cell Organelles

What if your school had only one large room? You might be trying to learn about cell organelles, while the teacher standing beside you is conducting the school choir. This would probably make learning nearly impossible. However, your school has classrooms in which different things can occur at the same time without interference. Cells also have many processes going on at the same time. Instead of classrooms, some cells have organelles—structures in the cytoplasm that have specific functions. However, the single-celled organisms—commonly called bacteria—do not have these structures.

Nucleus—The Control Center

A large organelle inside many **cells** is the nucleus, as shown in Figure 9 and Figure 11 on the previous two pages. The nucleus contains the genetic material—a chemical code for making all the molecules of a cell. Because of this, the nucleus often is called the control center of the cell. The nucleus is membrane-bound. That means it has a **membrane** surrounding it. Substances can pass into and out of the nucleus through small holes or pores in the **nucleus’ membrane**. Inside the nucleus is the **nucleolus**. It helps make structures that make proteins.

The genetic material in the **nucleus** is made of long chains of DNA that are coiled into structures called chromosomes.

Proteins in **chromosomes** also help the DNA coil. Cells in the same kind of organisms have the same number of chromosomes. For example, humans have 23 pairs of chromosomes in each cell but mice have 20 pairs in each cell, as shown in Figure 12.

Manufacturing

A cell makes many kinds of molecules in order to perform different functions. You read in Lesson 1 that proteins are important molecules in cells. Proteins are built within small structures called ribosomes. A ribosome is different from other cell organelles because it is not surrounded by a membrane and is found in all cells. For a cell with a nucleus, ribosomes are made in the **nucleolus** and move into the **cytoplasm** through the **nucleus’ membrane**. Ribosomes can be attached to an organelle called the endoplasmic reticulum. The endoplasmic reticulum (ER), as shown in Figure 13, is a highly-folded membrane that is connected to the nucleus’ membrane.

ER with ribosomes on its surface is called rough ER. Rough ER is important for making and modifying proteins. ER without ribosomes is called smooth ER. Smooth ER is important for making lipids and helps rid cells of chemicals and poisons.

Energy Processing

Recall that using energy is a characteristic of life. There are two types of organelles that process the energy used by cells.

Chemical processes that release most of the energy used by a cell occur inside membrane-bound organelles called **mitochondria**.

This energy is used for nearly all of a cell’s and an organism’s functions. A mitochondrion, as shown in Figure 14, transforms the unusable energy in food molecules, into a form of usable energy. Mitochondria sometimes are called the power plants of a cell. Cells that require a lot of energy, such as muscle cells, have more mitochondria than cells that require less energy, such as skin cells.

Some organisms, such as nearly all plants and some single-celled organisms, can make their own food. In plants, this happens in membrane-bound organelles called **chloroplasts**. A chloroplast, as shown in Figure 15, uses light energy to make food—a type of sugar—from water and carbon dioxide. Some bacteria can make their own food, but they do not have chloroplasts.

Processing, Transporting, and Storing

The Golgi apparatus, as shown in Figure 16, is like a processing factory for the cell. It makes, sorts, and ships molecules. The Golgi apparatus also modifies, stores, and directs the movement of molecules made in the ER. Some cells contain large numbers of Golgi apparatuses because the cells secrete substances that are needed by other cells or processes in an organism.

Within the **cytoplasm** are small, ball-like organelles called vesicles. A vesicle is made of membranes and transports or carries molecules throughout the cytoplasm. They carry substances to the cell membrane where they are released from the cell. Some vesicles form from the Golgi apparatus.

The storage organelles of a cell are vacuoles. Small vacuoles can contain food molecules, water, or waste products from the cell.

An animal cell contains a special vacuole called the lysosome that stores digestive enzymes. A plant cell, like the one shown in Figure 17, has a large vacuole called the central vacuole. It stores water and other molecules important for a plant cell. The central vacuole enlarges when water enters a plant cell and shrinks when water leaves.

As you have just read, cells are organized and have specialized structures for different functions. Some cell structures are common to all cells, but other structures are found only in certain cell types, as shown in Table 2 on the next page.

Cell Types

You have read that not all cells have organelles. Scientists use this and other facts about cells to classify cells. A cell without a nucleus and most other organelles is classified as a prokaryotic cell. A cell with a nucleus and other organelles is classified as a eukaryotic cell.

Prokaryotic Cells

The first living things to inhabit Earth probably were prokaryotic cells. Evidence indicates that they were the only forms of life on Earth for billions of years. Like all cells, a prokaryotic cell has a cell membrane and DNA, as shown in Figure 18. Instead of pairs of chromosomes, a prokaryotic cell has a loop of DNA. Prokaryotic cells exist only as single-celled organisms. An organism that is one prokaryotic cell is called a prokaryote.

Many prokaryotes have cell walls and flagella.

Prokaryotes also are known as bacteria. Some bacteria harm humans, such as Salmonella bacteria that cause food poisoning. Other bacteria are beneficial to humans. Many antibiotics are produced using Streptomyces bacteria. Escherichia bacteria live in our intestines and protect us from infections caused by other harmful bacteria. They also help us digest food and absorb some nutrients.

Some bacteria in the environment are essential for decomposing dead organisms and recycling nutrients. Other bacteria can survive in extreme environmental conditions, such as extreme hot or cold temperatures or extreme salty conditions. For example, some bacteria, such as the type shown in Figure 19, can survive in temperatures up to 85°C (185°F).

Eukaryotic Cells

The plant and animal cells on pages 18 and 19 are examples of eukaryotic cells. Besides differences in their structures and components, eukaryotic cells are larger than prokaryotic cells, as shown in Figure 20. Protists, fungi, plants, and animals all are made of one or more eukaryotic cells so they are called eukaryotes. Many scientists suggest that the eukaryotic cell evolved as a result of one prokaryotic cell becoming part of another prokaryotic cell. Because mitochondria and chloroplasts contain their own DNA, scientists suggest that they might have been prokaryotic cells that became part of another prokaryotic cell. Over time, the mitochondrion and chloroplast lost the ability to exist on their own.

What have you learned about the cell?

The parts of a cell have functions that ensure the survival of the cell. The cell membrane controls what enters and exits a cell. The cell wall and cytoskeleton determine the shape of a cell. Most cell organelles are membrane-bound. Chromosomes in a cell’s nucleus contain genetic information.

Two cell types are prokaryotic and eukaryotic. Prokaryotic cells do not contain any membrane-bound organelles. Prokaryotic cells live in a wide range of environments and have various roles.

Eukaryotic cells have a nucleus and other membrane-bound organelles. Eukaryotic cells have different roles in organisms.

Cells and Energy

All cells can release energy from food molecules. Only some cells can make food molecules using light energy.

Real-World Reading Connection When gasoline burns in a car engine, energy is released. It is transformed in many ways, making the car function as it should. Carbon dioxide, water vapor, and other waste gases from the burning gasoline exit through the exhaust pipe. Mitochondria are like engines in cells. Wastes are also produced when mitochondria release energy from food molecules.

Cellular Respiration

Automobiles cannot use the crude oil that comes out of the ground as fuel. It must be processed and refined into gasoline or diesel fuel. As you read in the previous lesson, the energy stored in the food molecules is not in a form that cells can use. **Cellular respiration** is a series of chemical reactions that transforms the energy in food molecules to usable energy. The usable energy is in molecules of ATP—**adenosine triphosphate**.

Reactions in the Cytoplasm

**Cellular respiration** happens in three steps. The first step is glycolysis and it happens in a cell’s **cytoplasm**. Glycolysis breaks down a glucose molecule—a type of sugar— into two smaller molecules, as shown in Figure 21. The chemical reactions of glycolysis require energy and release electrons that are used in the last step of **cellular respiration**.

Reactions in Mitochondria

The second step of **cellular respiration** happens in mitochondria and uses the smaller molecules produced by glycolysis. The smaller molecules are broken down into molecules of carbon dioxide—a waste product—and more electrons are released.

The third and final step of **cellular respiration** requires the presence of oxygen, as shown in Figure 22. This step uses the electrons released during the first two steps. It produces large amounts of ATP—usable energy—and water—a waste product.

Fermentation

Some cells can release energy from food molecules using a chemical process called fermentation. This process begins and ends in the **cytoplasm** and does not involve mitochondria or use oxygen. However, all types of fermentation produce fewer molecules of ATP than cellular respiration.

Lactic Acid Fermentation

When our muscles use oxygen faster than our lungs and blood can deliver it to them for cellular respiration, they can release energy by lactic acid fermentation. This process releases energy from glucose and produces lactic acid and carbon dioxide as wastes. Cheese and yogurt are made using fungi and bacteria that perform lactic acid fermentation.

Alcohol Fermentation

Did you know that bread is made by using yeast? Why is yeast necessary? Yeast are single-celled fungi that can perform alcohol fermentation—another kind of fermentation that releases energy, as shown in Table 3. This kind of fermentation is similar to lactic acid fermentation except it produces ethanol (a kind of alcohol) instead of lactic acid. Like lactic acid fermentation, alcohol fermentation produces carbon dioxide but fewer ATP molecules than **cellular respiration**. Now do you know why yeast is needed to make the bread?

Photosynthesis

You read in Lesson 1 that we get energy from the food that we eat. You also read that some organisms can make their own food using energy from the Sun or other light sources. **Photosynthesis** is a series of chemical reactions that makes food in these organisms.

Light and Pigments

We see things because light reflects off them. Light from the Sun contains all colors: red, orange, yellow, green, blue, indigo, and violet. A rainbow is evidence of this. The color of an object is the result of that object reflecting only that color of light. The object absorbs the other colors of light. For example, a red shirt only reflects red light and absorbs all the other colors of light. The same is true for living things. Plants contain substances called pigments that reflect and absorb light. Chloroplasts contain the pigment chlorophyll that reflects green light. When leaves appear green, it is because they contain more chlorophyll than other pigments. Have you ever seen leaves change colors in the fall? This happens because the chlorophyll in leaves breaks down and is not replaced. So, you see colors reflected by the other pigments in leaves, like the yellow pigments in the grape leaves shown in Figure 23.

Reactions in Chloroplasts

The light energy absorbed by chlorophyll and other pigments powers the chemical reactions of **photosynthesis**. These reactions occur in chloroplasts. During **photosynthesis**, light energy, water, and carbon dioxide are used to make sugars. **Photosynthesis** also produces oxygen that is released into the atmosphere, as shown in Figure 24.

Importance of Photosynthesis

The fruits and vegetables we eat grow because of photosynthesis. The cells of most organisms, even most bacteria, use sugars made by **photosynthesis**. **Photosynthesis** supplies Earth’s atmosphere with oxygen, which we must have for our cells to perform **cellular respiration**. The carbon dioxide produced by organisms from **cellular respiration** would become toxic if it were not used during **photosynthesis**.

What have you learned about cells and energy?

Cells perform **cellular respiration** and transform the unusable energy in large food molecules into usable energy in ATP molecules. Lactic acid fermentation and alcohol fermentation produce ATP molecules without the use of oxygen but fewer ATP molecules than **cellular respiration**. Light energy powers **photosynthesis** that produces sugars. Organisms that perform **photosynthesis** contain pigments that absorb light energy. Almost all organisms are dependent on **photosynthesis**. Cellular respiration and photosynthesis are important to most life on Earth.

The Cell Cycle and Cell division

The life of a cell usually includes periods of growth and reproduction.

Real-World Reading Connection

A multicellular organism like yourself is made of trillions of cells. How are all of these cells made? How long does a cell live?

The Cell Cycle

You probably are still growing, but not as fast as when you were younger. You might have entered the phase of development when your reproductive organs mature. All these phases are part of your life cycle. Similarly, cells have a life cycle called the **cell cycle**. The cell cycle, as shown in Figure 1, usually includes phases of growth and development and reproduction.

Phases of the Cell Cycle

You’ll spend most of your life growing and developing. Only a small portion of a human’s life cycle is spent in the reproductive phase. The same is true for cells. The phase of a cell cycle when a cell is preparing to reproduce is called **interphase**. It usually lasts longer than other phases of the cell cycle. The phase when a eukaryotic cell reproduces is called the **mitotic phase**. During the **mitotic phase**, the nucleus and cytoplasm of a cell divides, producing two new cells.

Length of a Cell Cycle

The length of time for the **cell cycle** is different for different types of organisms and cells. During the earliest stages of animal growth, the **cell cycle** can repeat quickly. For example, a zebra fish grows from a fertilized egg to 256 cells in just 2.5 h, as shown in Figure 2.

Characteristics of Interphase

A cell performs specific functions during **interphase**. For example, a cell in your **stomach** might produce substances that help digest your food. A plant cell, such as the onion root cell shown in Figure 3, might perform **cellular respiration** during **interphase**.

Recall from Chapter 1 that a cell’s nucleus contains chromosomes. During **interphase**, the chromosomes in the nucleus are like a bunch of thin spaghetti noodles. Each chromosome is so thin that it cannot be observed with a **light microscope**.

Also, you read that there are two sets of chromosomes in a **nucleus**. For each chromosome in one set of chromosomes, there is a similar chromosome in the other set of chromosomes.

Scientists call each pair of similar chromosomes a pair of homologous chromosomes. Humans have 23 pairs of homologous chromosomes.

Imagine if you had two sets of instructions for making a pizza.

The instructions are similar because they are both for making pizzas. However, they are not identical because the pizzas can have different toppings and crusts. The same is true for your homologous chromosomes; they have similar but not identical instructions.

Phases of Interphase Scientists have established that interphase consists of three phases—G1, S, and G2. During **G1 phase**, a cell grows and carries out its usual cellular functions. Some cells remain in G1 and do not reproduce. For example, your muscle cells, some nerve cells, and red blood cells never reproduce. Injuries to nerve and muscle cells can result in a permanent loss of function because they are not replaced. Red blood cells are replaced because they are produced by certain cells in the center of some bones.

Growth continues into **S phase**. During S, however, the chromosomes inside a **cell’s nucleus** replicate. This means that they make copies of themselves, as the diagram in Figure 4 shows.

The copies of a chromosome made during **S phase** are called sister chromatids. Sister chromatids are held together at a region near the middle of each chromatid called the **centromere**. The replication of chromosomes during **S phase** ensures that the two new cells formed by cell division are identical.

Organelle Replication

A cell continues to grow and carry out cellular functions during the final phase of interphase, **G2 phase**. It also replicates organelles during this phase of interphase. Some organelles, such as mitochondria and chloroplasts, can duplicate themselves because they contain their own DNA. The major events of interphase and the cell cycle are summarized in Table 1.

Mitosis and Cell division

You read in Chapter 1 that eukaryotic cells have organelles and other structures. You also read that the control center of the cell is the nucleus. When new cells are made, it is important that the contents of the nucleus be copied correctly. The nucleus divides in a process called mitosis. The cytoplasm divides in a process called cytokinesis. These events ensure that each new cell receives all it needs to function normally.

Importance of Mitosis and Cell division

As you read earlier in this lesson, a characteristic of all living things is that they grow and develop. Making more cells is one way multicellular organisms grow. They also grow because some cells increase in size.

Sometimes cells get old, wear out, and die. For example, every- one is constantly shedding old skin cells, as shown in Figure 5.

New cells formed by mitosis and cell division replace these skin cells. Some cells, such as the ones that line your stomach, live only for a few days. Because of mitosis and cell division, new cells constantly replace these short-lived cells.

Some organisms reproduce by mitosis and cell division. These organisms produce offspring that are identical to the parent. You will read about this kind of reproduction in Chapter 3.

Cell division Have you ever fallen and scraped your knee? The scrape heals because new cells are made to replace the cells that were damaged or lost. These new cells are made by mitosis and cell division.

It is important to understand that the processes of mitosis and cell division do not produce all cells. For example, a different kind of cell division produces sperm cells or egg cells from reproductive cells. You will read about this kind of cell division in Chapter 3.

Phases of Mitosis

Mitosis is a continuous process. However, mitosis has four recognizable phases or stages, as shown in Figure 6.

Prophase

The first phase of mitosis is **prophase**. Two major events happen during **prophase**. First, the DNA that makes up a replicated chromosome twists into tight coils. Have you ever twisted a rubber band so tightly that it coiled around itself? This is similar to what happens to chromosomes during **prophase**. Once the replicated chromosomes coil, they can be observed with a **light microscope**. The other major event during **prophase** is that the membrane around the **nucleus** breaks apart. After this happens, chromosomes can move to other areas of a cell.

Metaphase During the second stage of mitosis, **metaphase**, the replicated chromosomes move to the middle of the **cell**. The pairs of sister chromatids line up end-to-end across the center of the **cell**. How does this happen? Hairlike fibers pull and push the chromosomes to the middle of the cell.

Anaphase In the third stage, **anaphase**, the sister chromatids of each replicated chromosome begin to separate. The hairlike fibers extend from each end of a cell and attach to the centromere of the sister chromatids. These fibers pull the centromere apart and chromatids move away from each other, toward opposite ends of the **cell**. Now, the chromatids are called chromosomes.

Telophase

The final phase of mitosis is **telophase**. During **telophase**, a new membrane forms around each set of chromosomes. The chromosomes also become less tightly coiled. These two events are nearly the reverse of what happens in **prophase**. At the end of **telophase**, there are two new nuclei that are identical to each other and the original nucleus. However, the cell has not divided.

Dividing the Cell’s Components

Cytokinesis is the final stage of cell division. During cytokinesis, the cytoplasm and its components divide to form two identical cells called daughter cells. A sign that cytokinesis has begun is when the cell membrane squeezes inward, as shown in Figure 7.

This is similar to squeezing the middle of a balloon.

During cytokinesis, the appearance of a cell with a cell wall, such as a plant cell also in Figure 7, is different from an animal cell that does not have a cell wall. In a cell with a cell wall, a cell plate forms between the two new nuclei. The cell plate eventually becomes the cell membrane. The new cell walls of the plant daughter cells are built from molecules released by the cell membrane.

The process of mitosis divides a cell’s nucleus. However, the nucleus is just one of many organelles inside a cell. How do daughter cells get organelles? Recall that organelles and other structures are suspended in the **cytoplasm** of a cell. During cytokinesis, each daughter cell receives half the cytoplasm with organelles that were replicated during **G2 of interphase**.

Results of Cell division

It is important to realize that after mitosis and cell division, the original cell—called the parent cell—no longer exists. However, daughter cells’ chromosomes are identical to those of the parent cell, both in number and type. In other words, the daughter cells are genetically identical to each other and to the original parent cell. All the cells in your body, except sperm and egg cells, have identical chromosomes because of mitosis and cytokinesis.

Levels of Organization

From single-celled to multicellular organisms, all living things are organized.

Real-World Reading Connection

How do people organize their belongings? They usually place things with similar functions together. For example, things used to cook food are probably in the kitchen. Pots and pans might be in a cabinet and silverware in a drawer. In a similar way, cells and organisms are organized. They have structures that have specialized functions.

Single-Celled Organisms

Why would something that is one cell—a single-celled organism—need to be organized? A single-celled organism carries out all the functions needed for its survival, including obtaining nutrients, waste removal, movement, protection, and reproduction.

Prokaryotes

Recall from Chapter 1 that prokaryotes are single-celled organisms that do not have membrane-bound organelles. For example, bacteria are prokaryotes without nuclei or other organelles. However, bacteria still have structures with specific functions. Some processes that occur in organelles of eukaryotes happen in bacteria along specialized membranes, such as those shown in Figure 8.

Eukaryotes

Single-celled eukaryotes include protists, such as the amoeba shown in Figure 9, and some fungi. They are more complex than bacteria. Each single-celled eukaryote has a nucleus and other membrane-bound organelles. Membranes seperate organelles and their specialized functions from each other. They also enable organelles to transport substances into and out of a cell.

Single-celled eukaryotes are also more complex than cells in multicellular eukaryotes. They usually exist alone and obtain by themselves all the things that they need to survive. Cells in multi-cellular organisms, however, rely on one another to provide their needs, and cannot survive alone.

Some single-celled eukaryotes, however, live and function together in groups or colonies. After cell division, the daughter cells stay together. The protist colony Volvox, as shown in Figure 10, is made of specialized eukaryotic green algal cells. Some scientists propose that colonies of single-celled organisms might have led to the development of multicellular organisms.

Multicellular Organisms

When doing a group project, someone must organize the group and assign tasks. Someone must also make sure everyone does his or her task. The project requires organization to be completed successfully. In a similar way, multicellular organisms require organization. Multicellular organisms have many cells and usually have more than one type of cell. Therefore, they have more complex organization than single-celled organisms.

Cell Differentiation

You started as one fertilized egg. You grew by mitosis and cell division and developed into an organism with many types of cells. How did this happen? It occurred by a process called cell differentiation in which cells become different types of cells.

You read in the previous lesson that nearly all the cells of an organism have identical sets of chromosomes. Also, recall that chromosomes contain the instructions of a cell. How can cells be different if they have the identical sets of instructions? Part of the answer is that different cell types use different parts of the instructions on the chromosomes. It’s like the actors in a play who only need to memorize the lines in the script for their roles, not the entire script of the play.

Differentiated cells often have specialized structures and shapes for specific functions that are different from other cells. For example, liver cells, like those shown in Figure 11, have a greater amount of smooth ER than some other cells. Filtering blood is the main function of liver cells. Recall from Chapter 1 that smooth ER modifies poisons, making them easier to remove from a cell.

Brain cells, also shown in Figure 11, are another type of differentiated cell. Brain cells often are highly branched. Branching enables a brain cell to send and receive signals from many directions and over long distances. There are many other differentiated cells in humans, such as the ones shown in Figure 12.

Stem Cells

Once most human cells differentiate, they cannot become any other type of cell. However, some cells in your body are undifferentiated. These cells, called stem cells, can become different types of cells. For example, there are stem cells in the middle of some of your bones. Under the right conditions, these stem cells can become many different types of cells, as shown in Figure 13.

Plant Cells

Some plant cells can differentiate into another type of cell after they have differentiated into a specific type of cell. For example, the leaf of a begonia plant, as shown in Figure 14, contains differentiated cells with specialized functions. However, if you correctly cut a leaf from a begonia plant, place it on soil, and give it proper growing conditions, it can produce new plants.

Tissues

The cast of a play is made of different actors. Each actor must recite his or her lines to tell the play’s story. Similarly, most multicellular organisms have groups of similar cells that work together and perform a function. A group of cells that work together and perform a function is a tissue. For example, skeletal muscle tissue in your body is made of cells called muscle fibers, as shown in Figure 15. The muscle fibers contract and relax and allow your body to move. Plants also have tissues, as also shown in Figure 15.

Organs

A play’s stage crew might include carpenters and electricians. Both groups have specific functions, but work together to create a setting where the actors can perform. In a similar way, an organ is a group of similar tissues that work together to perform a function. Each tissue must function properly for the organ to function.

Human Organs

Your heart, lungs, brain, stomach, and muscles are some of the organs in your body. A muscle, such as the bicep in your upper arm, is made of different tissues, as shown in Figure 16. Besides muscle tissue, a muscle also includes tissues that surround and protect muscle cells and other tissues that connect muscle tissues.

Plant Organs Plants also have organs that perform functions such as storing nutrients, exchanging gases, transporting water or nutrients, or performing photosynthesis. A leaf, as shown in Figure 17, is an example of a plant organ. Leaves have tissues that transport substances, provide protection, or are specialized for photosynthesis.

Organ Systems

The cast of actors in a play work with makeup and wardrobe specialists, the director, and others in order to perform on stage. In a similar way, some organs in an organism work together. An organ system is one or more organs that work together and perform one or more functions. The muscles in your body work together with the bones of your skeleton to allow your body to move. The muscles and bones in your body make up your musculoskeletal system. Your heart, lungs, and digestive system work together to supply your muscles and bones with what they need to work together. Your brain receives and sends signals that coordinate all the organ systems in your body, as listed in Table 2.

Organism

The most complex unit of living things is a multicellular organism. Multicellular organisms, such as the dog shown in Figure 18, usually have many organ systems. Each organ system has its own function but is dependent on other organ systems. For example, your circulatory system transports nutrients throughout your body. The nutrients come from the breakdown of food in your digestive system. Your organ systems depend on your nervous system. You would not be able to breathe, move, reproduce, or eat without your nervous system. Without all these levels of organization, your body would be like a jumble of food, wastes, gases, and cells.
