# Supplementary material for: A semantic classification of nominal technical terms in secondary school biology textbooks
Source: PLoS One. 2024 Nov 11;19(11):e0312040. doi: 10.1371/journal.pone.0312040 (PMC11554214; doi:10.1371/journal.pone.0312040)
Supplement: S2 File — (DOCX) [file pone.0312040.s004.docx]

**Textbook 1: NSW Oxford Insight Science Year 8&9**

Chapter 1 Ecosystems

1.1 Interactions between organisms in ecosystems

THE BIOSPHERE AND ECOSYSTEMS

The biosphere describes the living component of the Earth. The biosphere is made up of all the living organisms found within the kingdoms of life – bacteria, protozoa, fungi, plants and animals of all shapes and sizes. It extends into the atmosphere, where some birds and insects can be found, as well as deep underground and to the bottom of the ocean where bacteria and other unusual forms of life can be found. The biosphere extends to any place on the Earth where life exists.

The biosphere can be thought of as a link between the atmosphere (air), the hydrosphere (water) and the lithosphere (land). It is large and all encompassing, and its relationships and interactions can be very complex, so we tend to study smaller components of the biosphere, called ecosystems.

Ecosystems

An ecosystem is made up of all the living organisms (**biotic factors**) and the physical, non-living conditions (**abiotic factors**) in a particular area and the interactions within and between the two. So a wetlands ecosystem is not made up of just the plants and animals that live there, but includes which animal eats what, where the different organisms live, the climate, water and nutrient availability, and how the organisms have adapted to the conditions.

Ecosystems are made up of habitats. A habitat is a particular area where a group of different organisms live, and the area has similar abiotic conditions throughout (e.g. temperature, water availability, wind speed). Habitats vary in size depending on the amount of food, water and shelter they provide.

All the individuals of a particular species living in the same habitat are called a population. All the populations of all the different organisms in that habitat are called the community. The biotic community interacts with the abiotic conditions of the habitat to form the ecosystem.

A habitat must supply all the needs of a population, such as food, water, shelter, suitable temperature and pH (acid level), and mineral availability. If that population’s needs are not met, individuals or the entire population may move to a different habitat or die out.

Benefits of ecosystems

Humans are a part of ecosystems too, and we rely on ecosystems as much as any other organism for our survival.

Water, oxygen, carbon dioxide and other chemicals cycle through ecosystems with the help of the biosphere. Plants and animals continuously cycle oxygen and carbon dioxide between themselves and with the soil, water and air. Plants take in carbon dioxide from the air (or from the water if they are aquatic plants) and release oxygen in the process known as photosynthesis. Plants and animals, including humans, use that oxygen in a process called respiration, and release carbon dioxide back into the air.

Plants and animals contribute to the filtering and cleaning of water in ecosystems as well. Root systems act like filters and slow down the flow of water, trapping sediment and some pollution. Some aquatic animals such as clams and anemones filter microscopic particles from the water to feed on, and in doing so they help clean the water they live in.

One example of plants and animals interacting is when animals are searching for food. Some plants attract insects, birds and even bats with brightly coloured or strong-smelling flowers. The animals eat the sweet sugar-rich liquid called nectar produced by the flower and get dusted with a fine powder called pollen. As the animal moves to the next plant, it transfers the pollen to the new flower and the pollen helps fertilise that plant, which is then able to make seeds. Pollination is important not only for wild flowers but for crop plants too. Insects, birds and other animals pollinate over 70% of plant species worldwide, including the fruits and vegetables commonly eaten by people.

FOOD CHAINS AND FOOD WEBS

The characteristics that identify something as a living thing are the abilities to move, to respond to their environment, to grow and to reproduce. These activities all require energy, which means all organisms require a source of energy for their continued survival. All animals, including humans, get their energy from the food they eat. But what is the ultimate source of this energy?

The Sun – the source of energy on the Earth

On the Earth, the primary source of energy for most organisms is sunlight. While sunlight is readily available to almost all organisms, it is a difficult energy source for most organisms to use, and it cannot be stored to use later.

All plants, some bacteria and some protozoa use photosynthesis to convert the energy in sunlight into a sugar that is easily transferred and stored. The Sun’s energy is used to join carbon dioxide and water molecules to form oxygen and a sugar called glucose. The energy is stored in the chemical bonds between the atoms in glucose.

The energy stored in the bonds of glucose can be released slowly through the process of respiration in individual cells. While not all cells can photosynthesise, all living cells respire, which means that glucose is a vital source of energy for the entire ecosystem.

The processes of photosynthesis and respiration are examined in chapter 2.

Glucose – transferable energy

Plants use the glucose they produce during photosynthesis for their own growth, repair, reproduction and other cellular functions, and they store any excess glucose for use in the future. Photosynthesis provides all the energy they require and therefore plants are autotrophic (‘auto’ meaning ‘self’ and ‘troph’ meaning ‘nutrition’).

The glucose in plants, and all the energy the glucose contains, is passed on to any organism that eats the plant. Organisms that cannot make their own glucose and must eat something to obtain their required energy are heterotrophic (‘hetero’ meaning ‘other/different’).

Autotrophs are also called producers because they make (produce) glucose using the chemical reaction of photosynthesis.

Heterotrophs are also called consumers because they must eat (consume) food to gain the glucose and the energy they require.

Energy, in the form of glucose, moves through ecosystems as different organisms eat each other. The paths the energy can take from producers to consumers can be represented by **food chains**. A **food chain** shows a list of organisms in the order of what eats what with an arrow between each organism to indicate the direction of the flow of energy.

Food chains

All **food chains** must start with a producer, because producers make the glucose for all other organisms to consume. The type of producer will vary depending on the ecosystem. The producer could be grass, a massive tree, seaweed or microscopic phytoplankton. In **food chains**, an arrow pointing from one organism to another means that the first organism is eaten by the second.

Consumers are then ordered according to what they eat. The first consumer in the **food chain** is referred to as a first-order consumer. Each step away from the producer is represented in the name: second-order consumer; third-order consumer, and so on.

Consumers are also named according to what they eat. First-order consumers only eat plants or other producers, and are called herbivores. Organisms that only eat other consumers are called carnivores. Consumers that eat both plants and other animals, like most humans do, are called omnivores. The highest carnivore in a **food chain** is referred to as the top predator.

Another vital component of food chains are detritivores and decomposers.

Detritivores are usually small invertebrate animals such as maggots, worms and insects, which eat dead and decaying matter.

Decomposers are organisms such as bacteria and fungi, which break down the dead matter into basic chemicals and return them to the soil, air and water. These chemicals can then be absorbed by plants through their roots and recycled back into the start of a new **food chain**. You will learn more about microorganisms and their impact on ecosystems later in this chapter.

Food webs

Like humans, most organisms will eat more than just one type of food. Eating more than one type of food ensures that if one food source becomes scarce, the organism can eat something else. This means a species may be part of many food chains at the same time. These multiple **food chains** can be linked together in a **food web** to represent the feeding relationships between the different organisms in an ecosystem.

Some consumers will have several labels to describe their trophic level (position) in the food web depending on which **food chain** within the web you are examining.

Figure 1.15 shows a food web with three different producers. The skink could be a second-order or a third-order consumer, depending on the **food chain** that is followed, while the ant is only a first-order consumer.

Sustainable food chains

Ecosystems are sustainable if there is enough energy available to pass through the **food webs** to maintain all the organisms in the community. Sustainable ecosystems have a consistent flow of energy and nutrients through the system so that all life can be supported. Unsustainable ecosystems use up energy or nutrients faster than they can be produced; therefore, some or all of the organisms in it will die.

Not all the energy from one trophic level of a **food chain** is passed to the next level.

Some of the energy is used by the organisms for their own movement, growth, repair and cellular functions. Most of the energy is lost from the **food chain** in the form of heat.

Only around 10% of the original energy at any level is passed up the **food chain**. The decreasing amount of energy available at each trophic level of the **food chain** is what limits the number of organisms at each level and explains why there are usually more herbivores than carnivores. Imagine visiting the African savannah and watching hundreds of lions hunting a herd of only a few zebras. The lions would very quickly run out of food!

Changing food webs

The addition or removal of a species from a food web will affect all other species within that web. Consider the food web in Figure 1.15 on page 11. The cat is an introduced species to the native food web. It competes with the magpies for skinks and is a major predator of the other birds.

Without cats, the populations of skinks, wagtails, honeyeaters and magpies would all increase.

When considering changes in food webs, it is important to consider all trophic levels. How will competitor species on the same level be affected? What about prey species on lower levels? What about predator species on higher levels?

THE EFFECT OF HUMAN ACTIVITIES ON FOOD WEBS

**Food webs** can show the delicate balance between producers and consumers. For example, if more grass grows, then animals that eat grass will have more energy and nutrients and so will have a greater chance of survival and greater chance to reproduce. More herbivores means more food for carnivores, but greater numbers of herbivores will increase the grazing on the grass and control its growth, balancing the ecosystem.

Food webs and the ecosystems they are a part of can be surprisingly fragile.

**Food webs** can become unbalanced when organisms are removed or introduced to the ecosystem. Even the smallest change can have surprisingly large effects later on and can seriously disrupt the flow of energy and food.

Altering habitats

Organisms are constantly changing their environments. They use resources and provide resources for other organisms.

Humans are certainly no exception. The impact of humans on environments is considerable due to our population numbers and our ability to manipulate our surroundings to suit our needs. We are all able to make both positive and negative changes to our environment.

Urbanisation and deforestation

Humans require a lot of space to live. Urban sprawl is the spread of urban areas into rural and coastal areas. Urban sprawl increases the distance between the city centre and its outer edge.

Huge areas of natural ecosystems have been cleared for human cities, roads and farmland as a part of urban sprawl. Today, Australians are clearing land at the massive rate of over half a million hectares a year.

That is the equivalent of about one million cricket grounds each year! Much of that land is used for our homes, to grow our food or to manufacture products. Clearing land of large producers, such as trees, significantly alters the habitat and food webs of the area.

Human changes to the producers in natural ecosystems will affect the number of types of herbivores, which in turn will affect the number and types of carnivores in the ecosystem. In areas that are being used for agriculture, the main types of producers tend to be grasses rather than large trees.

Many different animal species are forced to compete fiercely for the food and shelter the few remaining trees provide. However, organisms that feed on grasses have plenty of food and their populations often significantly increase.

Eastern grey kangaroos are an example of a species to benefit from the clearing of woodlands. Grass is the eastern grey kangaroo’s main source of food – with the increased amount of grassland available their populations have significantly increased. However, eastern grey kangaroos compete with the livestock for which the land was cleared in the first place. Kangaroo culling is now common in New South Wales and many other parts of Australia.

Land degradation

Human activities have led to a degradation (a decrease in health and quality) of the physical environment. Soil erosion is a major problem caused by the clearing of land for agriculture. In ecosystems with many trees, a dense mat of plant roots stabilises the soil. A layer of leaf litter covers the surface, which protects the soil from wind and water erosion. Water from rainfall is quickly absorbed through the top layers of soil.

Plants filter water as it soaks into the soil. The stems and leaves of grasses catch large particles of pollutants, and the plants themselves often remove toxins from the water that is absorbed into their roots. When these plants are cleared for human habitation or agriculture, these pollutants and toxins can get into the groundwater and can contaminate natural waterways.

Soil is compacted by the grazing of animals with hard hooves, such as cattle. This slows the absorption of water into the soil, increasing the water run-off and erosion. Wind erosion is also increased with less vegetation.

Other forms of land degradation include overuse of ecosystems, where humans take more resources from an ecosystem than the system can replenish. Examples of this type of degradation include overcropping (taking too many of a particular type of organism for food or materials), over-stocking of farmland (too many cattle in one area) and overfishing of oceans, rivers and lakes. Pollution of soil and water with chemical wastes, animal dung or excess fertilisers can also cause lasting damage to ecosystems.

Introduced organisms

Humans have introduced a large number of species into natural ecosystems. An introduced species is any organism not native or natural to that ecosystem. Introduced species can upset the balance of an ecosystem because they can alter the existing food web. Introduced species may outcompete native species for a limited resource, or they may not have a predator to keep their numbers in check. Many introduced species are considered to be a pest or, in the case of plants, a weed.

Humans often introduce pest species accidently. It is thought the northern Pacific seastar was carried into Australian waters by ships from its native ecosystem in northern Pacific Ocean areas.

The northern Pacific seastar is a huge pest in Australian waters – it is an efficient predator that feeds on a large variety of marine organisms, including fish eggs, oysters and mussels. It outcompetes the native seastars and has become the dominant predator in some areas. With very few natural predators and a large amount of food available to them, the population of northern Pacific seastars has increased dramatically and is also spreading out.

Some plant and animal species were deliberately introduced into Australia. Cows, sheep, horses, rabbits, foxes, cats and dogs are among the commonly known introduced mammals. Prickly pear, an introduced cactus species from South America, was brought to Australia to be used in the production of dye. However, it flourished in the dry conditions and spread out of control through agricultural areas. None of the Australian native herbivores were able to eat such a large cactus, and even cattle couldn’t eat it quickly enough. By around 1912, more than 10 million acres had been infected in New South Wales and Queensland.

In 1929, after much research and laboratory experiments into the prickly pear problem, a new species, a moth called Cactoblastis cactorum, was introduced to Australia. This cactus moth was also from South America and was the main consumer of the prickly pear in its original habitat.

The C. cactorum moths lay their eggs in the prickly pear, which then hatch caterpillars that eat the plant. Once the caterpillars turn into moths, native Australian insectivores eat them. The caterpillars don’t eat any Australian plants so they did not become a pest themselves. Ecological knowledge of the prickly pear’s native food chain identified

C. cactorum as a natural way to control the spread of the plant in Australia. Using existing food web relationships to control a species is called biological control.

Unfortunately, not all stories of biological control in Australia have such a good ending. Cane toads were introduced into Australia from Hawaii in an attempt to control the cane beetle, a native beetle species that was eating cane sugar and damaging crops in northern Queensland. Over 100 cane toads bred in a laboratory were released in August of 1935 around Cairns and Innisfail. However, there was very little research or experimentation conducted before the introduction of the cane toad.

Adult cane beetles infest the upper leaves of the sugar cane and the toads cannot climb that high. The beetle grubs live in the root system of the sugar cane and the toads are unable to find them. So the toads were not performing the role that they were expected to. Natural food webs involving cane toads were not investigated and so the impact of the toads on Australian food webs could not be accurately predicted. Cane toads are poisonous and very few native Australian animals have immunity to this poison and so almost all die when they eat the toads. This has a devastating effect on the populations of many native species. The endangered status of some species can be linked directly to the introduction of cane toads.

Without predators to control their numbers, the cane toad populations increased significantly. There are well over 200 million toads throughout Queensland and the Northern Territory, and they are now spreading into Western Australia and New South Wales.

While populations of native predators such as the northern quoll have been severely affected by cane toads, some predators are showing signs of behavioural adaptations by either avoiding the toads or handling them appropriately. The Torresian crow has learnt to flip toads onto their backs and only eat their soft underside to avoid the poison on the toads’ backs. Captive populations of northern quolls in Kakadu National Park are being taught to avoid eating the toads. These ‘educated’ animals are then released into the wild to hopefully ‘teach’ other quolls not to eat toads.

Biological control can be a very effective way to manage a pest species, but food webs must be thoroughly researched. Ecologists do just that, and must prepare forecasts and predictions about the introduced species and their impact on natural ecosystems.

Any introduced species must fit into the **food web** and maintain that balance or risk affecting other species.

Loss of biodiversity

Biodiversity is a measure of the number of different species in a particular ecosystem.

A rainforest ecosystem with hundreds of different producers, consumers and decomposers has much higher biodiversity than a wheat field ecosystem.

High biodiversity increases the chances of the ecosystem surviving environmental change. The different species within the community will all have slightly different adaptations and requirements that can be met by the habitat. If those conditions change, for example due to a new disease in the area, not all the species in the community will be able to survive the change. But the more different species there are in that ecosystem, the more likely it is that some will survive the change.

In an ecosystem with low biodiversity, if the conditions change so that the main species is affected, then the ecosystem can collapse because the majority of the individuals move away or die.

Biodiversity of an ecosystem can be reduced by the active removal of species or by introducing a new species that unbalances the food web.

1.2 Microorganisms in ecosystems

MICROORGANISMS AROUND US

Microorganisms were one of the first forms of life on the Earth. Some of the oldest fossils discovered, which date back 3.5 billion years, were found to contain microorganisms that could photosynthesise. These microorganisms called blue–green algae, or cyanobacteria, lived in colonies in the ocean and used photosynthesis to produce usable energy, just as modern plants do today.

The cyanobacteria colonies trapped sediment in the water, which over time create limestone structures called stromatolites. The live cyanobacteria colonies are spread out in a thin layer on the surface of these stromatolites, with the next layer of limestone slowly forming beneath them. It can take up to 100 years for a stromatolite to grow five centimetres!

Types of microorganisms

Microorganisms are too small to be seen by the human eye. Instead, a microscope is needed to study them. Microorganisms vary in size from one-tenth of a millimetre to one-thousandth of a millimetre.

Some microorganisms, such as bacteria and protozoa, are unicellular (single-celled). Others, such as some fungi, are multicellular. Microorganisms exist within and around us all the time.

Bacteria

Bacteria are from the kingdom Monera and are prokaryotic. While they have DNA, prokaryotic cells do not have a true nucleus or membrane-bound organelles. Bacteria come in many different shapes and sizes, and the shape of an individual bacterium can help identify the bacteria species.

Bacteria cells can be identified by the following characteristics:

•very small size

•presence of a cell wall (although it is made from a different chemical from those in plants)

•absence of a membrane-bound nucleus and organelles.

Some bacteria may have cilia (short hair- like structures) and/or flagella (long tail-like structures) on the surface of the cells. Cilia and flagella both help with movement.

Extremophiles are a relatively newly discovered group of microorganisms that can survive in extreme conditions such as the frozen poles of the Earth (cryophiles), the hottest deserts (thermophiles), environments with high acid concentrations (acidophiles), environments with high salt concentrations (halophiles), or environments with no oxygen (anaerobic extremophiles). Once considered part of the bacteria family, extremophiles are now in their own domain called Archaea.

Protozoa

Protozoa are from the kingdom Protista and tend to be much larger than bacteria, up to one millimetre in length. They are eukaryotic organisms and as such have membrane-bound organelles and a true nucleus. Rather than a cell wall, they have an outer membrane called a pellicle that is more rigid than the inner cell membrane, which helps to give them additional structure and support. However, like bacteria, they may have cilia and/or flagella on their cell surface to help with movement.

The shape and internal structure of protozoa vary greatly between species depending on their requirements. Protozoa can appear in food webs as producers, consumers, parasites and decomposers.

Fungi

When we think of organisms from the kingdom Fungi, we often think of mushrooms. But this group of microorganisms is much broader and includes organisms such as yeasts and moulds. Yeast is a unicellular fungi commonly used to make bread. The yeast organisms break down the sugar in the dough and form carbon dioxide gas as a by- product. It is this gas that causes the bread to rise.

Many fungi species, such as food moulds, grow as a mass of small threads called hyphae. These long thin cells are connected end-to-end. We cannot see this jungle of hyphae without a microscope. Instead, we often see a furry mass (the hyphae) with tiny black spots, which are the reproductive cells of the fungi. Fungi are eukaryotic (like protozoa), and so have a membrane-bound nucleus and organelles. However, fungi are unable to photosynthesise so they never contain chloroplasts. Fungi are heterotrophic, like animals. They are surrounded by a cell wall made from a chemical called chitin.

Fungi must grow on a surface, so do not require cilia or flagella for movement.

BENEFICIAL MICROORGANISMS

Microorganisms are present in every ecosystem on the planet and appear in every trophic level of food webs. When we think of microorganisms, we tend to think of them in terms of things that give us disease and make us ill. However, only a small percentage of microorganism species cause disease. Most of the microorganisms that live on our skin or inside us are actually helping to keep us alive and healthy.

Microorganisms as producers

All plants need nitrogen to make important molecules for their cells. Many soils do not contain enough nitrogen in the soil alone to keep a plant growing. Instead, some plants work with the nitrogen-fixing bacteria in the soil. The plants provide protection for the bacteria, which live in their roots, and the bacteria take the nitrogen out of the air and soil and change it to a form the plant can use. When the plant dies, the excess nitrogen and the nitrogen-fixing bacteria return to the soil for other plants to use.

Phytoplankton is a group made up of a huge variety of microorganisms that live in the ocean and photosynthesise. Phytoplankton is a vital food source for many marine organisms and the producer in many marine ecosystems.

Phytoplankton reproduces very quickly, but is eaten almost as fast and so their numbers remain fairly consistent and relatively low compared with the number of consumers they support. However, their rapid reproduction allows them to maintain a balanced food web and to provide around 40% of the Earth’s oxygen through photosynthesis.

Microorganisms as consumers

Yoghurt is a result of bacteria being deliberately being added to milk. The milk is heated, and then a bacteria such as Lactobacillus delbrueckii is added. These microorganisms consume and break down the lactose in the milk to produce lactic acid, which thickens the milk and forms yoghurt.

Other microorganisms are used to make soft cheeses. Yeast is used to help make bread rise and also to form alcohol. All the microorganisms used in food production are simply consuming nutrients and converting them into different chemicals. What are considered as wastes for the microorganisms are vital ingredients for some of our food products.

The microorganisms we use to help produce food were originally found in natural ecosystems. Discovering that different microorganisms could be used to make food for us was often accidental.

Microorganisms also live inside the digestive systems of animals to help them digest the food they eat. Herbivores eat only plants. Although there is a lot of energy in plant tissue, it is difficult to digest. Animals such as cows can digest the cellulose in grass with the help of archaea (ancient group of bacteria) that live in their four-chambered stomachs. Cows repeatedly regurgitate the food in their stomach, which is covered in digestive chemicals and bacteria, back into their mouths to be re-chewed and mixed further. This ‘chewing of cud’ explains why you often see cows that seem to be chewing without eating anything new. Without the bacteria, even this long chewing process would not be enough to obtain the nutrients from the grass.

Biofuels

We are very dependent on fossil fuels for many things, such as energy production and transport. Bacteria discovered in the hot springs of Yellowstone National Park in the United States of America are starting to help us run our cars on plants instead of petrol. The bacteria consume and break down a plant called switchgrass to produce a fuel called ethanol. This ethanol is called a biofuel because living organisms make it. Although the ethanol is currently only being used to fuel the rangers’ trucks within the park, scientists are hopeful that they can develop ways of using the bacteria to produce enough ethanol to run much larger numbers of cars… potentially for the whole country!

Oil spills

When 800 million litres of oil was spilled in the Gulf of Mexico in 2010, scientists were surprised to find that the oil did not spread as far as expected. After taking samples of the sea water in the area, scientists found the numbers of a particular bacteria species were larger than expected. These bacteria live naturally in the ocean, consuming petrochemicals (such as oil) that seep from the ground. The large oil spill caused the bacteria to grow and divide rapidly, ‘eating’ the petrochemicals within a few months and changing them into a less toxic substance. Scientists are now trying to determine what helps these bacteria to grow so we can use them to clean up oil spills in the future.

Microorganisms as decomposers

Imagine if dead things did not go soft and mouldy, and did not eventually decompose back into the soil. We would have to wade through dead plants and animals every time we left the house! Bacteria and fungi are responsible for the breakdown of once-living tissue. This allows the molecules that made up the tissue to be used over and over again. We also use microorganisms in the treatment of our sewage. Once we flush our waste down the toilet, it flows to a sewerage plant, where it is treated. One part of this treatment uses bacteria to digest the chemicals within the sewage into less toxic forms.

Microorganisms inside us

Some of the microorganisms we most depend on are the natural flora that live on our skin and inside our intestines. The microorganisms on our skin protect us from other microorganisms that might be harmful. The bacteria in our intestines also prevent other ‘bad’ bacteria from growing there.

The average adult human has 1 kilogram of microorganisms inside their large intestine alone. This kilogram is made up of thousands of different species of microorganisms. Without the microbes in our gut, we would not be able to digest food properly, get rid of wastes or make essential vitamins. You might be familiar with the bacteria Escherichia coli (E. coli) from reading about cases of food poisoning. However, there are also harmless, beneficial forms of this bacteria that live in our intestines and make vitamin K. We need vitamin K to help keep our body healthy.

HARMFUL MICROORGANISMS

Not all microorganisms are beneficial to us or to the environment. Too many ‘good’ microorganisms can also be bad for us or bad for the ecosystem in which they live.

Too much of a good thing

Eutrophication of an aquatic ecosystem occurs when excess nutrients are available. Eutrophication can happen naturally but is most often caused by fertiliser runoff reaching freshwater lakes or oceans. In eutrophication, protozoan algae and cyanobacteria in the water thrive on the extra nutrients and reproduce rapidly, and form what is known as an algal bloom.

These photosynthetic algae form a layer on the surface of the water, preventing the sunlight from reaching other aquatic plants or photosynthetic organisms below the surface. Without access to sunlight, these organisms die.

The huge numbers of algae also cause massive changes in the amount of oxygen available in the water. During the day, the algae photosynthesise faster than they respire, and therefore produce much more oxygen than they use. However, during the night when they cannot photosynthesise, the algae use up the dissolved oxygen during the process of respiration, which then limits the amount of oxygen available to other organisms. When the plants killed by the lack of light and oxygen decay, the process of decay caused by other bacteria will also use up dissolved oxygen from the water body, further limiting the amount of oxygen to other organisms.

In some cases, anaerobic bacteria (which do not require oxygen to respire) flourish.

These bacteria release toxins that are deadly to birds and mammals, harming anything that may live in or drink the water.

Microorganisms that cause disease

We have all been sick at some stage in our lives. Wild animals and plants in natural ecosystems can also become sick and die, and if many animals or plants are affected, it can affect the overall balance of an ecosystem.

Some diseases are caused by pathogens.

A pathogen is a foreign body that can potentially cause a disease. Not all pathogens are microorganisms. Viruses and prions (rings of faulty proteins) can also cause diseases, but they are not living organisms.

It is important to remember that not all microorganisms are pathogens. In fact, most microorganisms are either harmless or beneficial to our health. Microorganisms that are pathogenic (cause disease) include bacteria, fungi and protists. If these ‘bad’ microorganisms make it through your skin and your body’s other defences, they will make you sick.

Pathogenic fungi

Fungi can be harmful to both plants and animals. Fungi can be passed between people through clothing, towels and face cloths. A fungal infection often shows up as a small or rough patch of irritated red skin. Fungal infections such as tinea and ringworm are often itchy and uncomfortable.

Fungi and mould can grow on damp clothing, leaving small black spots and eventually decomposing the material. These microorganisms can also grow in damp houses. The fruiting bodies of the mould can be released into the air and inhaled by people living there. This can cause severe asthma-like reactions or potentially life-threatening chest infections.

Fungi are the most common pathogens that infect plants. The names of the plant diseases usually relate to the description of the symptoms rather than the pathogen that causes the disease because plant diseases were first noticed in crop plants, many years before the invention of the microscope. Fungal plant diseases include rusts, rots, blights, wilts, cankers and galls.

Pathogenic protozoa

Most pathogenic protozoa are considered to be parasitic because they take nutrients directly from their host, the organism in which they live. For example, Giardia lamblia infects the small intestines of mammals, absorbing digested nutrients and causing weight loss, diarrhoea, vomiting and stomach pain in the host.

Another common group of pathogenic protists are those in the genus Plasmodium.

These pathogens cause malaria, one of the most common diseases in the world.

Plasmodium cells are transferred to humans via the saliva of an infected mosquito. Once inside the body, they travel to the liver and infect red blood cells, absorbing nutrients. The symptoms and signs of malaria are similar to a bad flu; headaches, fever, nausea, vomiting and muscle aches. In some severe cases, malaria can lead to coma or death.

Pathogenic bacteria

Clostridium tetani is a bacteria species that lives in the soil and on rusty objects. When we get a cut or scratch, these bacteria can enter our body. They grow well in the anaerobic (oxygen-free) environment under the skin and start to reproduce. They produce a toxin that causes our muscles to seize, including the jaw muscles. For this reason, the infection is sometimes called lockjaw, as well as tetanus.

Salmonella is another genus of bacteria that produces a toxin. It causes nausea, vomiting, stomach cramps and bloody diarrhoea. It can often be found in raw chicken or fish. Although cooking can kill the bacteria, the toxin it produces is not affected. This is why it is important to refrigerate food, because few bacteria can grow below 4ºC (the temperature of a fridge).

Only around 100 species of bacteria infect plants. These bacteria invade the plant cells and produce chemicals that are toxic, destroy cell walls, or block the plant’s transport system.

1.3 Managing ecosystems

ECOLOGY IN AUSTRALIA

Ecosystems in Australia are affected by human activities, such as deforestation and the use of fossil fuels, as well as natural events such as floods, droughts and bushfires. Such changes can affect individual or multiple species, often reducing biodiversity and potentially causing food webs and ecosystems to collapse.

Around 85% of Australia’s flowering plants, 84% of mammals, 45% of birds and 89% of inland fish species are native only to Australia and nowhere else in the world. If these species become extinct in Australia, they will be lost from the biosphere forever.

Ecology is the study of ecosystems and changes to ecosystems. Ecologists and environmental scientists work to reduce the impact of natural events and human activities on ecosystems to develop sustainable management strategies for those ecosystems.

Lifestyle and biodiversity

Not everyone in the world has the same lifestyle as we do in Australia. Our basic standard of living in most of Australia is far higher than that of most other people in the world.

On average, Australians now own more goods, use more energy, eat more processed food and have larger houses than ever before. All this consumption, on top of a growing population, can create problems for biodiversity. Land is cleared to grow our food, to make room for industries and to build houses, which results in removing habitats for other living things. Clearing land can cause increasing salinity (salt concentrations) of some Australian soils. A severe salinity increase makes the soil unsuitable for many of the plants that usually grow on the land. Without the producers, consumers have nothing to eat and the balance of the ecosystem can fail.

The environmental effect of consumption is not confined to a local area. For example, using fossil fuels such as coal for energy in Australia affects global carbon dioxide levels and most scientists believe this contributes to the **enhanced greenhouse effect**. Many of the products consumed by Australians are made from materials sourced overseas. Australian consumption from a global perspective leaves a very large ‘ecological footprint’ on the environment.

Why conserve biodiversity?

Worldwide, many species are rapidly becoming extinct. It is likely that 20% of all known species will become extinct within the next 30 years, largely due to human activities that result in habitat loss. Orangutans (see Figure 1.55) are currently listed as endangered and are under threat from habitat loss and hunting. But why does it matter if we lose some species?

There are several reasons why we should conserve biodiversity.

The biosphere connects the planet

The biosphere links all the abiotic spheres – the hydrosphere, atmosphere and lithosphere. A decrease in biodiversity in the biosphere will eventually result in large- scale changes to the other spheres. Healthy ecosystems are necessary to maintain our atmosphere, climate, clean water and productive soils.

Biological resources

Biological resources include the whole or part of an organism, the genetic material found in the cells of organisms and the chemicals produced by its cells. These chemicals might be used for food, clothing, medicine or even transport.

Bioprospecting is the search among living things for new biological resources with potential medical or commercial use. Of the world’s 25 top-selling pharmaceuticals, ten were originally sourced from animals, plants or microorganisms.

A quick look in your bathroom cabinet will reveal many medicines that rely on biodiversity. Aspirin, a common painkiller, is based on a chemical from willow trees.

Penicillin, a very common antibiotic, is naturally produced by a particular species of fungi.

Australia has a huge potential for bioprospecting because it is one of the world’s most biodiverse nations.

Social and cultural values

There are many cultural reasons for maintaining biodiversity. Native plants, animals and ecosystems are part of our cultural identity. People value such areas for relaxation and enjoyment, outdoor activities such as bushwalking and bird watching. The Australian natural landscape has featured in many films, literature and photographs. Our natural environment is a major international tourist attraction.

Indigenous Australians are well known for describing themselves as custodians rather than owners of Australian land, placing such immense value on the country.

Conserving biodiversity raises important ethical questions. Do other species have as much right to the Earth as humans do? What responsibilities do we have for the environment?

We are only one of many species on the Earth. Viewing the planet as a shared one is very important to conserving biodiversity.

Conservation in Australia

At one time, conservation was thought to be the responsibility of politicians and scientists. Today, individuals and local communities also work to help conserve biodiversity.

If you have access to a piece of land, no matter how large or small, then you have the power to conserve a part of our biodiversity. This includes land such as your backyard, school grounds, community garden or local reserve. The secret is learning how to share our living space with the plants and animals around us.

Corridors of green

Many cities have set aside a permanent ‘green belt’ as part of their future planning.

Green wedges are the non-urban areas of metropolitan areas such as parks and large gardens. These areas protect a city’s open spaces and natural areas from overdevelopment. In some places, green wedges are important habitats for animals. They are also important for tourism and recreation. Some green wedges include areas with high heritage values for local Indigenous Australians.

Green corridors are consistent stretches of native habitat that allow animals to move from one location to another, through farmland or developed areas. Young animals can use green corridors to move out of an area and form their own territories. Areas of bush with a range of vegetation linked by green corridors provide a safe and suitable area for native birds and animals to live in and travel through. These areas also benefit farmers as they provide windbreaks and shelter. Pest problems are significantly reduced because of the increased number of predatory birds and insects that thrive in these native habitats.

MANAGING THE IMPACTS OF NATURAL EVENTS

Change is inevitable in all ecosystems, whether they are natural changes or as a result of human activities. Natural changes can be long-term, such as climatic changes. These long-term changes take such a long time to happen that they are usually beyond the control of humans.

Natural short-term changes are either regular and cyclical, or they are irregular events that can sometimes be considered as natural disasters. Regular and cyclical changes include the changes in abiotic conditions between day and night, between high tide and low tide, and between the different seasons. Examples of irregular natural events include bushfires, floods and droughts. Irregular changes are unpredictable in terms of when the event occurs, how long it lasts, and the intensity or severity of the event. Research into irregular natural events can help ecologists understand and predict the effect on ecosystems and the species within the ecosystems. If the frequency and intensity of natural events are too great, an ecosystem may experience a significant loss of biodiversity.

In this situation, knowledge from ecological research is critical in the development of sustainable management strategies to help conserve ecosystems and reduce the impact of the change on organisms.

Bushfires

Bushfire is a common natural event that ecosystems all over Australia experience at some stage. Many plant species have adapted to survive irregular burning with particularly thick or heat-reflective bark and special epicormic buds that re-sprout from the trunk after burning, and some species even require fire to germinate their seeds.

Fire intensity is a measure of the damage a bushfire does to an ecosystem. The plant life of the ecosystem provides the fuel for the fire and some plants are better fuel than others. The more or better the fuel, the more intense the fire is likely to be.

Short, sparse or spread-out undergrowth burns quickly and forms fast-moving ‘cool’ fires. These low-intensity fires will only damage the undergrowth, leaving scorch marks on the trunks and lower branches of trees. Tall trees may not even lose any of their leaves. The ash of the burnt plants provides nutrients for the soil, and the spaces that remain after the plants are gone are prime locations for new seedlings to grow.

Tall, dense grasses can provide lots of fuel for an intense bushfire. The fire takes time to burn all the fuel and can result in very hot fires. Slow-moving and hot fires tend to do the most damage. The fire has time to climb trunks and burn the upper branches and tops of the trees, removing all leaves. Without leaves, the trees cannot photosynthesise.

To survive, the trees must quickly regrow new leaves from epicormic buds. Very hot fires can burn through bark and damage the epicormic buds, and can also burn through the soil and kill the seeds, which prevents them from sprouting. Sap within the trees can even evaporate into a highly flammable gas and gives the fire even more fuel.

Natural fires of this intensity are usually started by lightning strikes in long-unburnt areas with lots of dry undergrowth for fuel. It can take years for an ecosystem to recover from the devastating effects of an intense fire. Unfortunately, many bushfires are also started by people. Accidental fires can be started by discarded cigarette butts and poorly managed campfires. Occasionally people deliberately light bushfires.

Intentionally lighting fires to cause bushfires is illegal and extremely dangerous as it can result in massive fires that may destroy homes and cause injury or death.

Effects of bushfires on ecosystems

Long-term ecological research shows that areas that were badly burnt one year are protected from major fires in the following years. The loss of undergrowth from the previous fire means less fuel for any fire that may come through that same area again. Recovering burnt patches act as natural firebreaks and can even stop the progression of a moving fire.

Ecologists who specialise in bushfire management have put fire regimes into practice. Positive fire regimes are intentionally lit fires that are carefully managed, with the controlled burns conducted in rotation through specific areas within an ecosystem to create a mosaic of habitats as the end result. Recently burnt patches of land protect long- unburnt patches from natural bushfires, and also reduce the intensity of natural bushfires. You may have heard of this process referred to as ‘back burning’.

The frequency of burning a particular patch of land can also influence the biodiversity of that patch. If left too long, a dominant species in the ecosystem may overgrow most other species and reduce the overall biodiversity of plant species in the area. Too frequent burning may not give enough time for the plants to recover and produce seeds. If there are no seeds or seedlings left, those species could become extinct in that particular habitat.

With the loss of adult plants as well as the seedlings and seeds of the plants, the open spaces often become vulnerable to introduced plant species. Fast-growing introduced grasses compete with the slower- growing native species and can take up all the available space. Introduced grasses have not adapted to bushfires in the same way native grasses have. They tend to be denser, be taller and make much better fuel for future bushfires.

AGRICULTURAL PRACTICES

Clearing land for agricultural use is one of the main reasons for the deforestation of natural ecosystems. Land for grazing livestock and planting crops is vital to support the growing human population in Australia. The knowledge gained through ecological research can be applied to agriculture, improving the productivity and sustainability of existing cleared land and reducing the need for further land to be cleared.

Soil organic carbon

Producers like plants convert energy from the Sun and chemicals from the soil into a form that animals and other organisms can use. Carbon stored in soil is a vital nutrient for producers and for microorganisms that live in the soil. Carbon stored within soil is called soil organic carbon (SOC) and is made up of decaying plant and animal material. Dead plants and animals on the surface of the ground are not a part of soil organic carbon, and when they decompose, the carbon is often released as carbon dioxide into the atmosphere.

Ecological and agricultural research shows that deliberately increasing SOC raises the health and yield (amount of food produced) of crops and improves pastureland for grazing livestock.

Decomposition of plant and animal material in the soil also releases other nutrients such as nitrogen and phosphates, which are vital for plant growth.

The presence of SOC also changes the overall soil composition. It increases the amount of water that can be absorbed and stored, improves gas exchange, and breaks up the soil, making it easier for root systems to grow and spread. SOC provides an excellent food source for many microorganisms, many of which continue to improve soil quality.

Many Australian farmers are looking for ways to increase the carbon in their soils.

There are a number of different agricultural practices that help improve SOC.

Conservative farming

Traditionally, after a crop has been harvested, the stubble (remaining roots and bases of plants) is removed. This is often done by using ploughs that tear up the soil or by burning the fields. Both of these methods release carbon dioxide into the atmosphere. Removing the stubble means there is less material to decay and turn into SOC.

Conservative farming does not plough the field after harvesting and leaves the stubble behind to decay and generate SOC.

Organic fertilisers

Compost and manure are common organic fertilisers made from partially decomposing plant and animal materials. Organic fertilisers instantly increase SOC when added to soil. Organic fertilisers are more sustainable than chemical fertilisers because their effect is longer lasting and they are less likely to pollute waterways and cause eutrophication.

Dung beetles reduce impact of cattle

Cattle for beef and dairy are large industries in Australia, but cows are not native to Australia. In the early 1950s, entomologist (insect specialist) and ecologist Dr. George Bornemissza noticed that cow pats lay around in paddocks for extended periods of time, suffocating and killing the plant life under them. In Europe, this was not a problem because dung beetles were removing the cow pats quickly. Dung beetles are detritivores that eat dung and other decaying material. They also lay their eggs in dung so their larvae can eat the dung.

Native Australian dung beetles have adapted to dealing with native Australian dung from grazing marsupials like kangaroos and wombats. Dung from these animals is usually small and fairly dry. The introduced cattle produce huge quantities of dung and the native beetles cannot break it all down quickly enough.

Hawaiian dung beetles were released between 1968 and 1984 as an introduced species to help control the level of cow dung. These Hawaiian dung beetles were bred in sterile captivity to ensure no new diseases were accidentally introduced along with the beetles.

Cow dung is now under better control.

Soil quality in areas with beetle activity is greatly improved and excessive fly populations, which also breed in cow pats, are less common.

Ecological research into dung beetle species is ongoing, and European dung beetles were introduced into Western Australia as recently as 2011.

Selective breeding

There are many examples of animals and plants being bred to keep, lose or enhance characteristics by humans carefully choosing their ‘partners’. This is known as selective breeding. For example, a cow that is known to produce lots of milk would be chosen to breed with a bull that is known to produce healthy, strong offspring. This would mean a greater chance of any female offspring being good milk producers and any male offspring being good meat producers.

The same applies to plants. A type of wheat known to survive frost or disease can be cross-pollinated with a type of wheat that produces high-quality grains to hopefully produce a combination of both features.

Selective breeding can also be used to reduce disease. Inbreeding results from animals reproducing with animals to which they are related. When this happens, rare diseases are more likely to occur. Inbreeding has been quite a problem with dog breeds, especially when people do not properly check the animal’s ancestry. Selective breeding and genetic testing can be used to make sure the animals are not closely related and reduce the chances of these rare diseases occurring.

Chapter 3 Understanding and managing ecosystems

3.1 Dynamic ecosystems

CYCLES OF MATTER

In natural ecosystems, matter is neither created nor destroyed. The amount of each chemical element doesn’t increase or decrease, but remains the same overall. Each element takes part in natural cycles, in which it moves through the Earth’s spheres. These cycles of matter from the atmosphere or the Earth’s crust and back again are called **biogeochemical cycles** (bio means living; geo means earth). These biogeochemical cycles break and make chemical bonds, shuffling the elements around without creating new atoms or destroying old ones. Biogeochemical cycles are examples of how living and non-living things interact. The nitrogen cycle and the carbon–oxygen cycle are two important examples covered in detail in this chapter. Other important biogeochemical cycles include the water cycle and phosphorous cycle.

Nitrogen cycle

Nitrogen (N2) is found as a gas in the atmosphere. It is an incredibly stable chemical, and comprises around 70% of our atmosphere. Nitrogen is cycled around the Earth in a process called the **nitrogen cycle**. Very few living organisms can actually use the nitrogen found in the atmosphere, but all living things need nitrogen as it makes up substances such as proteins. Special microorganisms called nitrogen-fixing bacteria change the nitrogen in the atmosphere into a form we can use. Some plants called legumes have nitrogen-fixing bacteria living on their roots. Lightning can also change some of the nitrogen in the atmosphere.

Denitrifying bacteria return nitrogen to the atmosphere as nitrogen gas. Nitrogen is absorbed from the soil by plants, and then into animals that eat the plants.

When living things die, organisms called decomposers break down the tissue of dead organisms and return the nitrogen back into the soil again.

Nitrogen is often the substance that limits the growth of organisms. When we farm, we tend to add extra nitrogen into the soil in a form that plants can use so they grow faster. For this reason, most fertilisers are nitrogen-based. Using too much nitrogen-containing fertiliser on plant crops can cause nitrate to run off from agricultural land into the waterways. This excess of nutrients causes an effect known as **eutrophication**, which results in population explosions of microorganisms known as blue–green algae or cyanobacteria in the water – ‘algal blooms’. These blooms then deplete the ecosystem of nutrients required by different species, and also decrease the oxygen level in the ecosystem as the cyanobacteria decay. This often causes the community to crash (see section 3.2).

**Carbon–oxygen cycle** Carbon is found in the atmosphere as carbon dioxide (CO2). Oxygen (O2) is the gas that we need to breathe to survive.

Carbon dioxide moves into the air during a process in cells called respiration, as well as through the decomposition of organic material. The main natural process that removes carbon dioxide from the atmosphere is the reaction called photosynthesis in plants. Photosynthesis and respiration will be discussed in more detail later in this chapter.

Scientists are now investigating the effect of the increasing levels of carbon dioxide in the atmosphere due to burning of fossil fuels. Burning of forests causes particularly large impacts to the amount of carbon dioxide in the atmosphere: carbon stored within plant tissues is released as carbon dioxide during burning, and the loss of trees means less photosynthesis to remove carbon dioxide from the atmosphere.

Carbon and oxygen are found in places other than the atmosphere. Figure 3.5 shows the cycling of carbon and oxygen. They are changed and used in different ways in the different parts of the Earth.

As with nitrogen in the nitrogen cycle, carbon and oxygen can become part of different substances as they move through the environment. However, the overall net amount of each element does not change.

Living things and the cycles of matter

You have seen the importance of living things in the nitrogen and carbon– oxygen cycles. In the nitrogen cycle, microorganisms ‘fix’ nitrogen into a form our bodies can use. In the **carbon–oxygen cycle**, plants absorb simple substances, such as carbon dioxide and water, and convert them into sugars by photosynthesis. Plants make other compounds from the sugars using minerals, and store them for further use, such as for growth and reproduction. Animals eating the plants have access to the sugars and other compounds.

When plants and animals die, decomposers (such as fungi and bacteria) break down the dead matter to obtain energy. Organisms do not absorb all the matter eaten. For example, cellulose in plant cell walls is not digested by some animals and is passed through the body unused.

Decomposers act on this waste material. Decomposers break down organic chemicals into simple substances, which are released into the atmosphere, surrounding soil and water to be reused by plants, so continuing the cycle.

Matter cycles can be easily disrupted by significant changes in the communities of living organisms that are a part of them. Algal blooms are the result of excess nitrogen in an ecosystem, leading to eutrophication. Although excess nitrogen seems like an advantage for the organisms, the algae grow to such extreme numbers that they strip most of the other nutrients, specifically oxygen, from the water. In the long term, all aquatic organisms are affected, as both the plants and animals that require oxygen for cellular respiration need to absorb it from the water.

The clear-felling of forests and woodland to make way for grazing pastures has a double effect on the carbon–oxygen cycle.

Fewer trees means less photosynthesis to remove carbon dioxide from the atmosphere and produce oxygen. High-density populations of stock like cows or sheep increase the amount of respiration, which increases carbon dioxide production and reduces the amount of available oxygen in the atmosphere.

ENERGY IN ECOSYSTEMS

All ecosystems rely on movement of energy from one part to another. The first source of energy in most ecosystems is the Sun. However, only plants and other photosynthetic organisms can use solar energy directly. These types of organisms are called autotrophs or producers. All animals and some microorganisms cannot use the Sun’s energy and must obtain their energy through food they eat. Organisms that require a source of food for their energy are called heterotrophs or consumers.

Energy is passed through ecosystems via **food chains** and **food webs**. Plants and other photosynthetic organisms originally harness solar energy. This energy is then passed to herbivores when they eat the plants. Carnivores obtain their energy from the herbivores they eat, and so on.

Not all the energy in ecosystems is passed on. Some of the energy is used by living things (to do work), and much of the energy is lost to the atmosphere as heat. Only 10% of the available energy at each food chain level is passed on to the next level. Energy cannot be created or destroyed (we say it is transferred and transformed), but energy in a useable form is often lost between each stage as a non-useable form such as heat. Energy moves and changes in ecosystems, but unlike matter, it is not recycled. Natural ecosystems must continuously take in the Sun’s energy.

Energy for work

Many energy transformations keep a living organism alive, functioning and carrying out chemical reactions that keep cells working. We can describe these processes as the work of living organisms. Some of the types of ‘work’ performed by living organisms are shown in Table 3.1.

Photosynthesis

Plants, some algae and some bacteria are able to gain energy during the process called photosynthesis. In photosynthesis, glucose is synthesised (made) from water and carbon dioxide. This requires light energy and the presence of a chemical called chlorophyll, which is found in cellular organelles called chloroplasts. Chloroplasts are concentrated in the cells of plant leaves. Stomata in the leaves are tiny pores through which the carbon dioxide needed for photosynthesis enters and the oxygen exits (see Figure 3.8). The solar energy is converted into chemical energy in glucose. The overall equation for photosynthesis is:

carbon dioxide + water → glucose + oxygen

6CO2 + 6H2O → C6H12O6 + 6O2

Photosynthesis produces oxygen and removes carbon dioxide from the atmosphere. The glucose product is soluble and readily transported around the plant. Any extra glucose can be converted into the sugar starch for storage.

Experiments can detect starch readily, and the presence of starch in leaves is often an indicator that photosynthesis has been occurring. Another way to detect photosynthesis is to measure the rate of oxygen bubbles released under water by aquatic plants such as Spirogyra.

Cellular respiration

Whenever we burn a fuel, such as wood or oil, we release energy that has been chemically stored in the molecules of the fuel. Burning requires oxygen and is a very rapid process, producing a lot of heat energy, carbon dioxide and water.

In living organisms, chemical energy is also released for cells to use, but in a much slower process called cellular respiration. In respiration, oxygen and glucose (C6H12O6) react together to give the products carbon dioxide, water and energy. This process occurs in all eukaryotic cells within organelles called mitochondria.

The overall equation for cellular respiration is:

glucose + oxygen → carbon dioxide + water + energy C6H12O6 + 6O2 → 6CO2 + 6H2O + energy

Photosynthesis and respiration: ‘opposite’ reactions

In the **carbon-oyxygen cycle**, photosynthesis and respiration are effectively the opposite of each other. Photosynthesis traps energy from the Sun into chemical bonds, such as those of glucose. Respiration moves the energy out of glucose, where it can be accessed directly by cells. However, the chemical equations listed here are only summaries of the processes. Both photosynthesis and respiration are actually a series of smaller reactions that involve many more compounds. These processes are not reversible because respiration and photosynthesis require their own specific enzymes to catalyse the reaction in one direction.

RELATIONSHIPS IN ECOSYSTEMS

A community and the relationships between individuals in the community make up the biotic part of an ecosystem. In every community you will find producers, herbivores, carnivores and decomposers.

Complex interactions happen between these living things. Predators hunt for other animals to eat, herbivores keep an eye out for carnivores to avoid being eaten, and decomposers slowly break down dead plants and animals. Organisms compete within their own species and with other species in the community for food and other resources.

Although some organisms do not have any direct effect on each other in an ecosystem, most organisms have some kind of relationship. These relationships may be beneficial, neutral or detrimental (harmful), and they may be ongoing or short-lived.

Relationships may be between organisms of the same or different species.

Relationships within a species

Intraspecific (‘intra’ meaning within; ‘specific’ meaning species) relationships exist between individuals of the same species, usually of the same population. There are three main types of intraspecific relationships: collaboration, mating and competition.

Collaboration

Collaboration occurs when organisms cooperate with each other in a way that ensures their survival. This usually occurs in species that exist in large populations.

Examples include:

•ants – leaving a scent trail when they search for food so other ants can find the food too

•wolves – hunting in packs

•sea lions – leaving pups in ‘crèches’ to be looked after while they go hunting

•flowering plants – growing close together to increase the chances of cross- pollination

•fish – swimming in large schools to help confuse predators and avoid being eaten (Figure 3.12).

Mating

Mating between same-species partners produces viable offspring, thus ensuring the survival of the species. All sexually reproducing species must come together at some stage in their **life cycle**. Some species, such as antelope, may live in large groups of males and females. In other species, such as orang-utans, the genders live separately until breeding season. The young usually stay with their mother until they are old enough to fend for themselves. Many plants sexually reproduce, but often need the help of the wind or a pollinator to bring the pollen (sperm) to the ovum (egg).

Competition

Competition occurs when organisms use the same limited resource. Organisms will compete for all possible resources if there is not enough for everyone. Individuals that are good competitors will get more of the resource than weaker competitors. Those that do not gain enough of that resource may die. Examples include:

•seedlings from the same plant species competing with each other for light and space as they grow

•males competing for the right to mate with females

•predators like lions competing over food

•birds competing over nest sites.

Relationships between different species

Interspecific (‘inter’ meaning between) relationships exist between individuals or populations of different species.

These relationships can be beneficial or detrimental to one or both species involved.

Beneficial or neutral relationships

Mutualism, symbiosis and commensalism are examples of relationships between different species. Mutualism is a relationship between two organisms in which both organisms benefit. A relationship between two species that are so interdependent that neither can survive without the other is called symbiosis.

Lichen is an example of a symbiotic relationship of a fungus and an alga, whereas the anemone and anemone fish in Figure 3.15 is an example of only mutualism. Mutualistic and symbiotic relationships tend to last a long time, often a lifetime. These relationships have driven adaptation to better suit the other species. For example, flowers have evolved to take on particular colours and shapes, and to produce specific scents and nectars, to better attract specific pollinators.

Commensalism is a relationship in which one organism benefits and the other organism is not affected. Commensalism is relatively rare in the natural world – it is unlikely an organism that has a relationship with another will not be affected in some way.

Detrimental relationships

Predator–prey, parasitism and competition are relationships in which at least one of the species has the potential to be greatly harmed. We call these detrimental relationships.

In a predator–prey relationship, the predator organism eats the prey organism. Therefore the predator benefits and the prey is harmed. Predators and their prey have a balanced relationship with each other. If all the prey is eaten then the predator is disadvantaged. Figure 3.19 shows a typical graph of predator– prey population fluctuations.

Parasitism is a relationship in which one organism (the parasite) lives in or on the body of another (the host). An effective parasite benefits and the host is harmed to varying degrees. A good parasite can survive and reproduce inside or on its host for close to the normal life span of the host. However, if the parasite takes too many nutrients from the host, the host may get sick or even die. If the parasite cannot leave the host in time, then it will die too.

Competition may exist between members of different species that share a resource such as food or nesting sites. Many different animals nest in tree hollows. The removal of dead trees reduces the number of nesting sites and increases the competition for them. Without adequate shelter, many animals cannot reproduce or survive. Inhibition, or allelopathy, is a particular type of competition that occurs when one organism produces a chemical that directly inhibits or hinders the growth and development of another. This is commonly seen in plants and microorganisms.

A dynamic balance

All organisms live in a complex web of interrelationships – relationships with each other and with their environment. Many of these relationships are between living things, as you have seen. Non- living (abiotic) factors play a critical role in determining what type of community survives at a particular place.

**Abiotic factors** include the physical and chemical parts of the environment. Abiotic factors in terrestrial (land-based) ecosystems are different from those factors in aquatic (water-based) ecosystems. Sunlight, temperature, rainfall and soil type are all examples of physical factors. Chemical factors include availability of minerals, oxygen and carbon dioxide, and soil or water pH (acidity levels) and salinity (saltiness). Ecosystems in New South Wales include grasslands, rainforests, rock platforms, mangroves, salt marshes, sand dunes, freshwater lakes, mallees and alpine herbfields. Some people distinguish natural ecosystems from agricultural and urban ecosystems, which are dominated by people.

A group of organisms of the same species living in the same ecosystem is called a population. An ecosystem needs to be able to maintain a balance so all species can exist at their optimum population size. Gains due to reproduction and immigration (moving in) must balance the losses due to death and emigration (moving out). Changes in one species’ population can dramatically affect the population of a different species.

**Food webs** are diagrams showing several different **food chains** connected together. The arrows in a food web indicate one animal eating another. Consider the food web for the ecosystem shown in Figure 3.26. Frog numbers have decreased in parts of Australia for reasons still being researched. If frogs decreased in this particular ecosystem, consequences could include:

•an increase in grasshoppers and thus a loss of grass

•an increase in praying mantises

•a decrease in lizards

•a diversion of birds towards a diet of praying mantises rather than frogs and lizards

•a consequent decrease in praying mantises

•a further increase in grasshoppers and more loss of grass – if this was severe enough the ecosystem would be at risk as it depends on a good supply of grass.

The most likely outcome is that the bird population would decrease so all species would return to balance with reduced population sizes. A positive effect of reduced bird numbers is that it might enable the frog population to recover.

Ecosystem balance is a type of dynamic equilibrium (‘dynamic’ meaning changing; ‘equilibrium’ meaning balance). Changes may upset the equilibrium but another equilibrium becomes established. Often, it is not greatly different from the original.

Gains and losses in ecosystems occur naturally but they may be intensified by factors such as floods and bushfires.

Reproduction, death, migration, natural events (such as seasonal changes), disasters (such as floods, droughts and earthquakes) and human intervention occur regularly and affect population sizes.

3.2 Changing populations

POPULATION DYNAMICS

A common indicator of the health of a natural ecosystem is its biodiversity. Biodiversity is a measure of the number of different species in an ecosystem (‘bio’ meaning life; ‘diversity’ meaning different). A rainforest with thousands of different species has very high biodiversity, but potentially a low population of each species. A field of wheat might have millions of individual plants in the population, but very low biodiversity due to the vast majority of individuals in the community being the same species.

In any one ecosystem you must consider all the different kingdoms represented.

Microorganisms are important to the health and functioning of an ecosystem, as are all the plants and animals. Significant changes to any of the populations within the community can seriously affect the others.

Ecosystems that have high biodiversity tend to be more resilient to environmental change. An individual species might die out in that ecosystem, but due to the different characteristics and adaptations in the different species, other species will survive the change. For example, if a fungus infects a field of wheat, it could potentially kill every plant in the field. However, if the same fungal disease infected a rainforest ecosystem, only some plants may be affected while different species may have a natural resistance and survive.

Population dynamics is the study of the fluctuations (changes) in population numbers within ecosystems. Surveys of an ecosystem may be to monitor the population size of a particular species, or to measure the biodiversity of the area.

Scientists can make predictions and take certain precautions to conserve species if they know approximately how many of each species are in a certain location. Regular sampling provides information about changes in population numbers, and causes of the changes can be identified.

Counting organisms

There are a number of ways to determine the size of a population. Counting every individual organism in a population is the most accurate way, but in practice this is rarely possible and is very time-consuming.

Estimates are more easily achieved by surveying from helicopters or using quadrats, line transects or capture–recapture methods. For human populations, a questionnaire called a census is the usual method. A census actually takes a snapshot of information about every individual in a population.

For plants and stationary animals, quadrats (square frames) are most commonly used for population estimates. The quadrat frames are either randomly placed (tossed over the shoulder) or sequentially marked out in a designated area within the ecosystem. Every individual is counted in each plot and its species noted. An average number of each species is calculated for each quadrat and then used to estimate the total number of organisms in the ecosystem (by using the known total area of the ecosystem).

This method works well if a large number of quadrats are used and the organisms are small and relatively evenly spread or dispersed throughout the ecosystem.

A line transect is an excellent method of examining the diversity of an ecosystem and the distribution of a particular species over a varied ecosystem. A straight line, often a measuring tape, is placed through an ecosystem. Any organism that touches the line (including directly under or over it) is counted and its species noted. This may be done along the whole length of a short transect, or at regular intervals (every metre) along a long transect. A number of parallel transects can be used to calculate an average of each species at each distance along the line. Transects are also only used for plants or stationary animals. They are particularly useful in an ecosystem where the conditions change significantly in predictable bands, like intertidal zones.

For most mobile animals, capture– recapture is a popular method for population estimation. Animals are captured in traps then marked with tags or other marks such as food dye or permanent marker on their tails, feet or other easily seen body parts as shown in Figure 3.33.

The number counted on the first capture is N1. The animals are then released and it is assumed they disperse evenly throughout the population. They are then recaptured one or two days (or nights) later and the total number of animals in the second capture is N2. Not every animal that was captured in the first trapping session will necessarily be caught the second time, and new individuals may be captured in the second trapping session. The number of marked animals in the recapture, those that have been captured twice, is M2.

An estimate of the population is then obtained using the formula:

It is assumed that the probability of capturing animals on both occasions is the same, but this is not always the case. The animals may not like the traps and may avoid them on the second occasion, or they may love the bait (often muesli, honey, peanut butter or oats for small mammals) and deliberately come back for more! Markings can be temporary, like food dye, and wear off within a few days, or it can be more permanent like leg rings, ear tags or GPS inserts. These more permanent tags enable ecologists to follow individual animals over long periods of time and can be used to gather data on distribution, migration and long-term changes to populations. But it is also important to ensure the tagging does not affect the normal lifestyle of the organism so it does not affect its survival. For example, tagging a possum with something brightly coloured would cause it to be more visible to predators and less appealing to potential mates.

Capture–recapture is a very suitable technique for estimating the population size of small Australian mammals such as the marsupial mouse Antechinus. Because most native Australian mammals are nocturnal (active at night), the traps may be set at night and checked the next morning.

NATURAL FACTORS AFFECTING POPULATIONS

What causes the changes to the births, deaths, emigration or immigration that may change a population size? The dynamic equilibrium of a population means there are continual small changes to the population size, but overall the total number of individuals remains roughly the same.

New births and immigration into the area cause the population to increase, while deaths and emigration cause it to decrease. Significant or relatively long-term changes to population size are usually due to specific events or causes. Many of these changes are natural, but others are caused by human intervention.

Limiting resources

All populations are limited in size by their carrying capacity. This is the maximum number of species members that the environment is able to support in terms of resources. As a population increases and gets close to its carrying capacity, some of the environmental resources will be significantly depleted. Competition for the resources will increase, because there simply isn’t enough food, water or shelter to go around if for so many individuals. Some organisms will either die or leave the area. Hence, the population will stabilise (reach its maximum size). Only one resource needs to be limiting to restrict the size of a population. That resource may be nesting sites, a food or water source, physical space or light availability for plants, or many other examples.

Seasonal changes

When the weather becomes colder, many birds and other animals migrate to warmer areas, resulting in their population significantly decreasing in one environment and increasing in another. During the breeding season, usually spring, numbers of animals will increase as the next generation is born. Flowering plants are pollinated and form seeds that disperse (spread out) in the environment and later germinate.

Presence or absence of organisms of other species

Predators such as the pied currawong in Figure 3.37 and competitors may decrease population numbers. Some organisms will be familiar with predators and competitors and have ways to avoid predation or increase their own ability to compete for resources. Introduced predators and competitors can have devastating effects on resident species. Organisms will need to adapt quickly to avoid predation (being preyed upon) and will have to ‘fight’ for resources.

Disease

The introduction of a disease into a population may have a major or minor impact on a species. The impact will be determined by the cause of the disease and the species’ ability to fight it. Like humans, prior exposure to a disease can enable organisms to launch a successful immune response.

Completely new diseases are likely to have more of an effect on a population.

Populations that are small or heavily interbred tend to have similar resistance to disease. One new disease may wipe out the entire population. Cheetahs in Africa are very vulnerable due to their small numbers and low genetic variation. This effect is also seen more in Australia with the Tasmanian devil and their low resistance to facial tumours.

Extreme natural changes

Extreme natural changes have varying population effects. For example, bushfires can produce ash containing minerals suitable for seed germination, and many native Australian plant species need fire to release their seeds from the woody fruits (Figure 3.39). In both these cases, bushfire results in a population increase. If the fires occur too frequently, young plants can be destroyed before they can produce their own seeds. Animal populations can be severely reduced because they may be killed in bushfires or they may flee to other ecosystems.

Drought affects all organisms, as water is a vital resource for life. Plant and animal populations will suffer as the availability of water reduces and competition for it significantly increases.

Floods, earthquakes and all other natural disasters affect population sizes, usually reducing them significantly as individuals are killed outright, or from the resulting loss of resources increasing competition.

While there are plenty of natural impacts on populations, the effect of human activites are felt in many ecosystems to varying degrees. Humans can have a significant short-term or long-term impact on ecosystems and the wellbeing and survival of other species.

Competition for resources

At present, the human population is about seven billion and is predicted to rise to about nine billion by 2050. Humans have developed technologies that efficiently remove resources from the environment.

Demand for food is increasing with the human population, which means more pressure on the natural resources of the land and sea. These resources may be needed by other species. For example, humans use a lot of water from the Murray–Darling Basin for agriculture, which seriously affects other species that depend on the river system such as the Murray cod. The river red gum forests that surround the Murray River have been placed under extreme stress during droughts.

Permanent removal of habitats by humans to use the land for building or agriculture, or the trees for wood, is very likely to result in significant changes to populations within ecosystems. Animals that previously lived in the habitat will migrate or die. Plant removal may reduce pollination of similar species in the area as well as reduce nesting sites for many animals.

Pollution

Human activity has introduced many chemicals into ecosystems. Some chemicals can cause mutation and/or death of certain species and, in some cases, can result in the collapse of entire food webs.

A common pollutant into waterways is fertiliser runoff. The excess nutrients in the water cause blue–green algal blooms (eutrophication). Although the blue– green algae photosynthesise, once the bloom is over they die and decay, which removes dissolved oxygen from the water and starves all other aquatic organisms of oxygen. The microorganisms also grow across the surface of the water, blocking sunlight from reaching organisms lower down in the water, preventing them from photosynthesising and replenishing the oxygen in the water.

Irrigation (the artificial application of water to land or soil) also increases fertiliser runoff. On the east coast of Queensland, this runoff also carries topsoil and silt into the ocean. Once in the ocean, the silt settles on top of the coral reef as ‘marine snow’, suffocating the coral polyps and blocking out the sun for the photosynthetic symbiotic algae that live in the coral. Coral growth is extremely slow, so the death of sections of the reef is devastating to the reef ecosystem.

There are now more restrictive rules about the chemicals industries can release into the environment.

Enhanced greenhouse effect

Increasing numbers of humans, increasing wealth and more sophisticated technology have resulted in large amounts of fossil fuels being used for transport, industry, agriculture and electricity. Burning of these fuels is contributing to the amount of carbon dioxide in the atmosphere. More carbon dioxide means more trapped heat, causing an overall average increase in the global temperature. This is called the **enhanced greenhouse effect**.

While some areas are getting hotter, others are getting colder. In some ecosystems, regular seasonal changes are becoming less predictable, and extreme weather events are occurring more often. Organisms that cannot adapt to these changes in their ecosystems will either emigrate or die.

Introduced species

Humans have introduced many plant and animal species to Australia as a whole, but also to specific ecosystems. Foxes, rabbits, cats, dogs, cattle, crop plants and decorative plants were all introduced for food, sport, familiarity or companionship. All of these new species compete with native species.

New predators have devastated existing native populations that have not adapted to avoid them.

Biological control is the carefully considered and planned introduction of a native or non-native species to an area to control pest species. The pest species may be a native species with uncontrolled population growth, or it may be an introduced species like the prickly pear or cane toad. The biological control species must fulfil strict criteria so that it, in turn, does not become a pest.

The cane toad (Figure 3.43) was introduced into Queensland in an attempt to reduce the numbers of beetles that were eating sugar cane crops. However, the toad instead preyed on other species. The introduced cane toad is still spreading south, outcompeting many other toad and frog species, and poisoning potential native predators like quolls and owls.

The introduction of the cactus moth, Cactoblastis cactorum, was a successful application of biological control for the prickly pear cactus. Huge areas of pastureland were overgrown with the introduced cactus. The prickly pear cactus continued to reproduce and spread without a natural grazer until its natural predator, the cactus moth, was introduced. This moth lays its eggs on the cactus and the moth grubs that hatch eat it. The moth grubs don’t eat any native Australian plants and therefore do not harm any other plants.

When the number of cactus plants reduced, the moths had nowhere to reproduce and so died off, causing no lasting negative impact on the ecosystem.

However, not all effects of introduced species reduce the population of native species. The increased pastureland created by humans for cattle and sheep has enabled native populations of kangaroos and wallabies to increase. Human homes and rubbish tips provide food and shelter for many bird species and possums.

3.3 Managing sustainable ecosystems

INDIGENOUS LAND MANAGEMENT

Populations of Indigenous Australians survived and managed their environments through periods of changing climate. There is no doubt they changed aspects of the environment with the introduction of the dingo and fire regimes, but they managed the environment sustainably and with great respect.

Indigenous Australians demonstrate great diversity in their language groups, cultures and activities, yet retain a common theme of shared ownership and living in harmony with the environment. Sustainability has economic, social and environmental components. In trying to move to a more sustainable way of managing our environment, current Australians are recognising the need to learn and incorporate traditional knowledge.

The following examples demonstrate that traditional Indigenous Australian societies were far more than just nomadic hunters and gatherers.

Kakadu: burning for biodiversity

It is believed the name ‘Kakadu’ is derived from the local Aboriginal language Gagadju. The Gagadju-speaking Indigenous Australians were believed to be that region’s original inhabitants about 50 000 years ago.

Kakadu wetlands faced a problem after the removal of all feral Asian water buffalo in the 1980s. The buffalo kept the native grass mudja (Hymenachne acutigluma) in check, but after the removal of the buffalo it spread, choking wetland plants, restricting the feeding of water birds, reducing the variety of habitats and limiting access for hunting and food gathering by Indigenous Australians. The solution involved a return to traditional fire management practices.

Mudja had not been a problem before the buffalo introduction because regular burning by Indigenous Australians had maintained biodiversity and supported their ability to hunt and gather food throughout the year. They had sustainably managed their environment.

Traditional ecological knowledge has been combined with modern science to develop and monitor the solution. CSIRO and the Bushfire Cooperative Research Centre worked with a family of Indigenous Australians traditional land owners in Kakadu as part of a northern Australian ‘Burning for Biodiversity’ project. The project applies traditional fire management techniques in the Boggy Plain floodplains of the South Alligator River and now also the well-known Yellow Water wetlands.

Woodlands and paperbark forests that surround the grasslands are burnt progressively between May and August, early in the dry season. This ensures there is little fuel beyond the grasslands so later grass fires remain contained. From September to the beginning of the downpours of the wet season in December, the mudja is burnt.

The burning has been a success. Populations of turtles, magpie geese, other wetland birds and water lilies have increased. Plant variety such as wild rice and spike rushes and a greater variety of habitats, including more open water, can now be seen. Care is being taken to ensure the traditional knowledge is recognised, remembered and passed to the next generation.

Uluru and Kata Tjuta: Reducing human impact

The Uluru and Kata Tjuta (the Olgas) area contains one of the most significant arid (dry) ecosystems in the world. It receives less than 250 millimetres of rainfall per year. Despite the harsh climate, this area is biodiverse.

When early European explorers first visited this region in the 1870s they were confronted by a harsh landscape. Their initial aim was to find a route for the overland telegraph line from Adelaide to the Northern Territory, and to set up pastures for sheep and cattle grazing. However, they soon decided the region was unsuitable, and left.

However, the traditional owners of the land, a group of Anangu Indigenous people, had lived on this land for thousands of years and understood it well. They lived a nomadic life, travelling in small family groups and surviving by hunting wildlife and gathering food from the land. Their sustainable hunting and gathering practices meant people could successfully live in a very fragile and harsh ecosystem.

The Anangu knew where to find food to survive and, more importantly, which areas were best for hunting and gathering at different times of the year. The Anangu classified their environment to help them locate food (Table 3.2). The Anangu also distinguished five seasons (Table 3.3).

Introduced species have affected the Uluru–Kata Tjuta communities. Buffel grass was used to reduce erosion in some areas, but has spread and is now having a major effect on the diversity and distribution of native grasses and the availability of seeds used for food. Camels, domestic mice, rabbits, feral cats, dogs and foxes have had a significant detrimental influence on the communities and environment.

SUSTAINABILITY AND ECOSYSTEMS

People in Australia expect a lifestyle that provides essential food, clothing and shelter, but also many additional comforts. Meeting these demands can damage natural ecosystems. Environmental sustainability is about balancing the way we live with its impact on ecosystems. For sustainable practices to begin and to continue, individuals, communities and governments need to work together.

Table 3.4 includes the benefits and problems of some local strategies used to balance human activities and needs with conserving, protecting and maintaining the quality and sustainability of the environment.

Notice how some of the activities can be done as an individual or as a family, and others can be done as a community, organisation or country. Energy suppliers can make major decisions about the installation of renewable power generation plants such as wind turbines. Individuals can make decisions about whether they support ‘green’ energy sources or install solar hot water. More will be achieved when communities and nations work together to achieve common sustainability goals.

**Textbook 2: NSW Biology Year 11**

11.1 Relationships between biotic and abiotic factors in an ecosystem

Communities are made up of different organisms grouped together at a given time and place. On a larger scale, these communities interact with other communities and their physical surroundings, and are classified as ecosystems. For example, Wollemi National Park in the Greater Blue Mountains World Heritage area, New South Wales is a dynamic ecosystem with communities of flora and fauna such as grey box eucalypts and wombats interacting with their physical environment of sandstone gorges, canyons and river valleys.

This section explores the interactions between biotic and abiotic factors in an ecosystem including the ways in which organisms interact with their environment (e.g. physical barriers, migration across landscapes and the use of abiotic resources) and the ways in which organisms interact with one another (e.g. predation, competition and symbiotic relationships).

ABIOTIC FACTORS SHAPE ECOSYSTEMS

Organisms affect, and are affected by, their physical surroundings. For example, earthworms live in soil, where they bury and consume dead plant material. This helps to recycle nutrients and aerate the soil. Like all animals and plants, earthworms take in oxygen, water and nutrients and give out carbon dioxide and other wastes. Plants take in carbon dioxide and produce oxygen during photosynthesis. Without the first photosynthetic organisms that evolved billions of years ago, Earth's atmosphere would have lacked oxygen and the biosphere would be very different today.

The interdependency, or strong connection, between biotic and abiotic factors is key to ecosystem functioning. **Abiotic factors** determine where organisms can and cannot live (their distribution) and if an organism reproduces, thrives or dies. Biologists can use information about abiotic factors to predict an organism's growth, abundance and distribution and understand how changes in abiotic factors might affect species.

The changing Earth

The conditions on Earth have always determined the variety of living organisms that can exist. Earth's atmosphere has changed remarkably over time. The early Earth, which formed 4.6 billion years ago, was volcanically very active and the atmosphere was very different from that of today. Climates (long-term weather conditions) changed over time, and there have been multiple ice ages and hot, dry periods. Ice ages, or glacial episodes, affected Earth's climates by changing sea levels and affecting air and water circulation, leading to species migration, shifts in vegetation zones and species extinction. These historical changes have influenced and shaped the ecosystems we see on Earth today.

The movement of the large tectonic plates that make up Earth's crust has also shaped environmental conditions. As the positions of land masses and oceans has changed throughout Earth's history, so too have ocean circulation patterns, climatic conditions and many abiotic and biotic factors. Oceans are also barriers to the migration of terrestrial (land) species and influence the distribution of marine organisms. Ocean ecosystems are especially affected by plate tectonics as mid­ocean ridges (underwater mountain ranges formed when two tectonic plates meet, causing the ocean floor to lift) are responsible for the spread and depth of oceans, as well as the development of ocean 'climates'.

The movement of tectonic plates continues to shape modern ecosystems. For example, tectonic activity near Japan led to the eruption of a new volcano and emergence of a new land mass known as Nishinoshima in 2013 (Figure 11.1.2). The land mass now covers approximately 2.46km2 and although it is mostly bare rock derived from cooling lava, deposits of faeces from birds flying over Nishinoshima are leading to the development of nutrient-rich soil. Recent studies on the island in 2016 identified the presence of primitive plants and insects.

Climate zones

Differences in climate have created many varied terrestrial and aquatic ecosystems, from the frozen soil of the tundra landscape, home to polar bears and caribou, to the tropical waters of the coral reef with its shallow pools, clownfish and turtle species.

Climate zones, or belts, are regions of distinct climates occurring in an east­to-west direction across Earth (Figure 11.1.6). Solar radiation determines many aspects of Earth's climatic zones; ecosystems at latitudes close to the equator are exposed to more sunlight than ecosystems towards the North and South Poles. Solar radiation also determines the seasons and other factors such as rainfall, which influence the characteristics of climates. There are three main climate zones: tropical, temperate and polar (Figure 11. 1.6). A fourth zone, subtropical, is sometimes used to describe the region between the tropical and temperate zones. Polar zones are the coldest regions on Earth, with the least solar radiation. Temperatures are often below freezing throughout the year and ecosystems in the polar zone have low biodiversity (variety of species). The polar zone is towards the North and South Poles at the highest latitudes on Earth (blue regions in Figure 11.1. 6).

Temperate zones have moderate temperatures and rainfall, with seasonal variation throughout the year. The temperate regions on Earth are located between the polar and tropical zones (green regions in Figure 11.1.6). Many parts of the temperate zone are rich in biodiversity.

Tropical zones are the warmest and wettest regions on Earth. The tropics experience the most solar radiation because they are located close to the equator, where radiation from the Sun is almost perpendicular to Earth throughout the year (red/orange regions in Figure 11.1.6). The high rainfall and high temperatures in the tropics support ecosystems witl1 high biodiversity. Tropical rainforests and most coral reef ecosystems are found in the tropical zone.

Climate plays an important role in ecosystem functioning, not only determining the organisms found in that ecosystem, but also the behaviour and interactions of organisms. Climate affects many life cycle events such as the migration of species, germination and breeding. For example, humpback whales (Megaptera novaeangliae) spend the summer months feeding in the cold waters of Antarctica. By late autumn, the whales commence their annual migration along the east coast of Australia to the warmer tropical waters of the Pacific Ocean to breed and give birth (Figure 11.1. 7).

Changing climates

Every ecosystem has a 'threshold'-a point past which the ecosystem changes irreversibly. Human-induced climate change is one significant contributor to ecosystems passing this threshold. Climate change affects all aspects of ecosystem functioning, from life cycle events such as flowers blossoming, to changes in species' distributions and food webs.

For example, multiple species of whales are being affected by climate change, with rising ocean temperatures causing the populations to migrate earlier and travel further. With increasing ocean acidification, birth rates are dropping and breeding grounds are changing. Krill are an important food source for many species of whales, and responsible for increasing their body fat ahead of migration. Krill populations are drastically declining due to rising ocean temperatures.

The impact of changing climates on ecosystems will be discussed further in Chapter 13.

Biogeography

Biogeography is the study of species' distributions to understand their evolutionary past and the abiotic and biotic factors that might determine species' abundance and distribution now and in the future. For example, the island of Madagascar (Figure 11.1.8) has been studied with interest by biogeographers for a long time. Madagascar is located in the Indian Ocean off Africa's south-east coast and is home to many endemic species (species that are found nowhere else on Earth). Biogeographers have investigated why there are so many unique species on Madagascar and have discovered that large-scale **abiotic factors** such as geology, geography and climate have all influenced the distribution and abundance of species.

Madagascar was landlocked in the supercontinent Gondwana 1 70 million years ago, before breaking apart and remaining isolated for the last 88 million years. It is believed that some species from the supercontinent remained on Madagascar, while others arrived on the island after Madagascar had broken away. After becoming isolated, the species on Madagascar evolved unique adaptations, different to those species on mainland Africa. Madagascar's species had many different abiotic factors to adapt to, with ecosystems on the island including mountain ranges, rainforests (Figure 11.1. 9a), deserts, coral reefs and mangroves. As a result, Madagascar is home to species including baobab trees (Figure 11.1.9b), lemurs (Figure 11.1.9c), tortoises, chameleons and comet moths (Figure 11.1. 9d). Madagascar is very high in biodiversity, that is, the variety of organisms that live in a particular habitat.

Biodiversity

High biodiversity is closely linked to latitude and climate. The abundance of species per unit area (species richness) increases from the poles to the equator, with the highest biodiversity at the tropics near the equator. Over 50% of the species on Earth live in the tropics. **Abiotic factors** in tropical regions strongly contribute to this high species diversity, including a more stable climate, warm temperatures, high levels of rainfall and a higher level of primary productivity (plant growth), which supports more consumers (animals, fungi and bacteria).

BIOTIC FACTORS SHAPE ECOSYSTEMS

The manner in which **biotic factors** interact also affect the growth, abundance and distribution of communities in an ecosystem. In an ecosystem, the organisms interact and depend upon one another for survival. They influence one another by being part of each other's environment. Interactions between organisms can be harmful, neutral or beneficial.

INTERACTIONS BETWEEN SPECIES

Interactions between species are called interspecific interactions and interactions within species are called intraspecific interactions. These interactions are usually classified according to how the interaction affects the survival and reproduction of the organisms involved.

Some interactions involve organisms acting in opposition to one another (competition and predation) and some interactions involve organisms working together (symbiosis). Interactions between species can also be classified as feeding and non-feeding interactions.

Competition

The presence of other organisms may limit the distribution of some species through interspecific competition. Interspecific competition is a struggle between organisms of different species for the same supply of food, water, space, nest sites or any other environmental resource that is in limited supply. Intraspecific competition is competition between individuals of the same species.

Because they use similar resources, green plants mainly compete with other green plants, herbivores with other herbivores, and carnivores with other carnivores. Competition can lead to one species being forced out of a habitat by its competitor. The species that was outcompeted usually continues to survive in adjacent parts of the habitat.

For example, populations reaching a plateau when numbers get too high for the environment

(Figure 11.1.13a, b). However, if these species are grown together, the population of P. caudatwn grows initially, but then its population decreases to extinction (Figure 1 l .1.13c). In other words, P. aurelia outcompetes P. caudatwn. P. aurelia continues to reproduce to reach a population density similar to when the species are cultured separately.

The kind of interaction seen in the Paramecium is called competitive exclusion-where one species is better at obtaining resources, excluding the other from the available resources and sometimes driving them to extinction. Other types of competition result in resource partitioning, where species change their behaviour and resource use, allowing both species to access resources in the same environment.

You will learn more about competition in ecosystems in Sections 11.2 and 11.3.

Predation

Predation occurs when one animal species (the predator) kills and feeds on another animal (the prey). Predators are carnivores. Some predators hunt for their prey (Figure 11.1.14a) and others catch their prey in traps (Figure 11.1.14b). In both cases the predator benefits by eating and killing the prey, which is obviously harmed. The role of predation in ecosystems will be further discussed in 'Feeding interdependencies' on page 506 and Section 11.3.

Symbiosis

Sometimes two quite different organisms live and function together in a close association for the benefit of at least one of them. Close, long-term relationships between different species are a type of symbiosis. Symbiosis can be beneficial for both organisms (mutualism), beneficial for one organism while not affecting the other (commensalism), beneficial for one organism and harmful for the other (parasitism), or harmful to one organism with no benefit to the other (amensalism) (Table 11.1.1). Each organism involved in the interaction is called a symbiont. Symbiotic relationships can be further classified as obligate symbiosis (necessary for survival) or facultative symbiosis (beneficial but not necessary for survival).

Beneficial interactions

Mutualism

Mutualism is a symbiotic partnership that benefits both organisms. Some of the most common partners in mutualism are unicellular algae. The bright colours of corals, the green of some hydra and the brilliant blues of the mantles of giant clams are produced by algae living in the animal's tissues (Figure 11.1.16). The algae photosyntl1esise and produce glucose and oxygen, which are used by the animal. In turn, the animal produces carbon dioxide during respiration, which the algae need for photosynthesis. The algae also benefit by having safe shelter within the animal.

Sometimes organisms evolve adaptations that encourage symbiosis. For example, most flowering plants are adapted to attract pollinators. The plants have colourful flowers, nectar or fruit that attract animals such as bees and bats. The animals benefit by collecting food from the plant and the plant benefits as the animals pollinate their flowers or disperse their seeds. An example of this type of relationship is between the desert quandong or Australian native peach (Santalum acwninatum) and emus. The emu can digest the soft part of the desert quandong's fruit, but not the seed, which it passes out in its droppings. The droppings act like fertiliser and help the seed germinate and grow. The emu and quandong have a mutualistic relationship­the emus benefit from eating tl1e fruit and the quandong benefits by having its seeds dispersed (Figure 11.1.1 7).

Benign interactions

Benign interactions are interactions in which no species is harmed.

Commensalism

Commensalism is an interaction between species in which only one species benefits but the other species is not affected. Animals such as birds or possums nesting in a tree hollow is an example of commensalism. In this case, the bird or possum benefits and the tree is not harmed (Figure 11.1.27).

Trees are also often host to epiphytes: smaller plants such as orchids, ferns, mosses, liverworts and lichens that live on the trunk or in the crown of the tree (Figure 11.1.28). The epiphyte receives sunlight and rainwater. This relationship is usually benign for the tree because it is neither helped nor harmed (unless it becomes overloaded with the weight of the epiphytes on its branches).

Harmful interactions

Harmful interactions are those in which only one species benefits and the other species is harmed as a result of the interaction.

Parasitism

In parasitism, one species (the parasite) benefits and the other species (the host) is harmed. Ectoparasites such as ticks and mistletoes live on or outside the host (Figure 11.1.29). Endoparasites such as parasitic fungi and wood-borers live inside the host (Figure 11.1.30). A parasite obtains its food from tl1e host but does not necessarily kill it. The type of harm the parasite causes the host varies, but may include the following effects:

shortened lifespan

impaired functions such as digestion, photosynthesis or reproduction

less ability to withstand stresses such as drought or cold

greater vulnerability to predators.

Many parasites have more than one host during their **life cycle**. A host that transfers a parasite to another host is called a vector. For example, the malarial mosquito is a vector for the Plasmodiwn parasite. It transfers tl1e parasite when it bites a person to obtain blood. An infected person is also a vector for Plasmodiwn because they transfer the parasite to the mosquito when it bites them.

All organisms have parasites. For example, every species of plant and animal that has been studied has been found to have at least one parasitic species living in it. Tapeworms are a common parasite in animals (Figure 11.1.31). They are ingested and then attach to the small intestine, where they absorb nutrients and can grow quite large. Although they can cause illness, loss of appetite and anaemia, they sometimes produce no symptoms at all.

Amensalism

Amensalism refers to an association between species in which one is inhibited or killed and the other species is unaffected.

A simple model of amensalism is the way in which animals can inadvertently damage vegetation around them but are unaffected by the relationship. For example, animals such as sheep and cattle often trample grass. The grass may be damaged or killed, but the animals receive no benefit from having done so (Figure l .1.32a). Similarly, some waterbirds such as cormorants kill vegetation in places where they roost or nest (Figure 11.1. 3 2b). This is because their droppings are high in nitrogen, phosphate and potassium, which plants cannot tolerate.

FEEDING INTERDEPENDENCIES

One important way that species interact and apply selection pressures on one another in an ecosystem is through feeding relationships. For example, a leaf-eating insect is part of the environment of a grevillea shrub. The insect has a source of food and benefits from the interaction, but the shrub is harmed because its photosynthetic leaf area is reduced. On the other hand, a honeyeater (Figure 11.1.34), which is another part of the environment of the shrub, is not harmful but useful to the tree. As the bird gathers nectar from the grevillea flowers, it transfers pollen from shrub to shrub. The interaction benefits both organisms. The bird pollinates the grevillea and gathers its reward as food. Having a diverse range of species that includes both harmful and helpful organisms for the grevillea means that the ecosystem remains balanced.

Species form a web of feeding interdependencies within an ecosystem. Any change to one species within an ecosystem will have a flow-on effect on other species in the same food web. Feeding interdependencies make species vulnerable to changes in their ecosystem. Ecosystem changes can present new selection pressures that may cause the extinction of species or lead to organisms adapting to the new conditions. The giant panda (Ailuropoda melanoleuca) is an example of a species that is especially vulnerable to environmental change because of its specialist diet (Figure 11.1.35).

All organisms in an ecosystem require energy to survive. Some organisms can create their own food (autotrophs), but others need to consume specific types of food from a particular environment (heterotrophs). Some organisms feed on plants, some feed on insects or other animals, and others feed on dead and decaying material. Almost all organisms are consumed by at least one other organism.

Species in an ecosystem are interdependent; that is, they rely on each other. If one species is removed from an ecosystem, any species that interacted with it is affected. For example, sea otters along the coasts of the northern and eastern North Pacific Ocean feed on sea urchins and keep the sea urchin numbers in balance. If sea otters were suddenly removed from the ecosystem, sea urchin numbers would increase, and the kelp that the urchins feed on would be overgrazed (Figure 11.1.36).

The removal of a species may have a positive effect or a negative effect on other species in the ecosystem. Likewise, a species being added to an ecosystem can also have far-reaching effects. By understanding the relationships within an ecosystem, the impact of introducing or removing species can be predicted. For example, studying the feeding relationships between organisms in an ecosystem can help scientists understand why some species are affected by such changes. This understanding then allows the scientists to predict the effects of ecosystem changes, assisting them in the management and conservation of populations, species and ecosystems.

Food chains

A **food chain** links organisms according to their feeding relationships. The two main groups of organisms in a food chain are producers (also known as autotrophs) and consumers (also known as heterotrophs). The energy that producers (plants) make using photosynthesis may be eaten by a consumer, which may be eaten by another consumer, and so on. In this way, the energy in organic matter is transferred between organisms.

In a food chain diagram (Figure 11.1.38) the arrows show the flow of energy and matter through the chain. In a eucalypt tree, kookaburras feed on skinks that feed on insects that feed on the eucalypt leaves. This is an example of a food chain with three links between four types of organisms. In the pond food chain discussed later (Figure 11.1 .40) there is just one link pictured: the link between the catfish and the parasites that feed on them.

Food chain participants

Producers

Plants and algae in a pond, and a eucalypt tree in a forest, play an important role as the first links in **food chains**. They manufacture their own food by photosynthesis, so they are autotrophs. In **food chains** and **food webs**, autotrophs are called producers. Producers are always the first link in a **food chain** because they produce organic compounds from simple inorganic compounds. There are two types of autotrophs: photosynthetic autotrophs and chemosynthetic autotrophs.

Most producers, including plants and algae, are photosynthetic autotrophs (Figure 11.1.39). They use photosynthesis to make their own food. Some specialist autotrophs are chemosynthetic. This means they obtain their energy for producing organic compounds directly from inorganic molecules such as hydrogen sulfide and methane. Autotrophs are examined in detail in Chapter 5.

Consumers

All organisms that are not producers are consumers. They are heterotrophs; that is, they obtain their energy by consuming other organisms. There are six types of consumers:

herbivores

carnivores

parasites

scavengers

detritivores

decomposers.

Consumers are further classified as primary, secondary, tertiary or quaternary depending on where they fit into a food chain. In the eucalypt tree, insects that eat the leaves are primary or first-order consumers; skinks that eat the insects are secondary or second-order consumers; and the kookaburra that eats the skink is a tertiary or third-order consumer. If there is no other carnivore to consume it, the kookaburra is the top carnivore.

Consumers can be classified according to the type of organisms they feed on and the ways in which they feed on them. The different classifications are shown in Table 11.1.2 (page 510), with examples of each type.

Predator-prey food chains

Many food chains are **predator-prey food chains**. A simple example from a pond is the catfish that feeds on crustaceans (Daphnia) that feed on algae (diatoms). The food chain linking the kookaburra, skink, insect and eucalypt tree (Figure 11.1.38) is also an example of a predator-prey food chain.

Predators usually eat prey smaller than themselves. A hawk preys upon animals the size of a mouse or small rabbit. **Predator-prey food chains** usually proceed from small organisms to large organisms.

Parasite-host food chains

**Parasite-host food chains** look a little different from predator-prey food chains. **Parasite-host food chains** have a large organism (the host) as a source of food for a smaller organism (the parasite). Figures 11.1.40 and 11.1.41 are examples of parasite-host food chains in aquatic ecosystems.

The **life cycle** of many parasites is complex. At different stages of development, from larva to adult, each feeds on a separate host species. Thus one species of parasite may be linked to a number of food chains. In Figure 11.1.41 the tapeworm Ligula intestinalis feeds on three hosts. An egg of the tapeworm hatches in the water. A copepod (first host) eats the larva. The larva grows in the intestinal cavity of the copepod after penetrating the intestinal wall. The copepod is eaten by a fish (second host) such as a bream, and continues to develop and grow in the intestine of the fish. An aquatic bird (third host), such as a grebe, then eats the fish. The adult tapeworm develops within two days of being eaten because of the higher temperature in the bird's intestine. Before they die in the bird, the adult worms produce eggs which pass out into the water, and the cycle continues.

Detritivore-decomposer food chains

In temperate forests only about 10% of the plant material is eaten directly each year by herbivores. Much of the plant material falls as leaf litter. Dead leaves, dead branches, fallen tree trunks, dead roots in the soil, and the remains of dead animals are a major source of food for detritivores such as snails, worms, termites, springtails, millipedes and mites, and decomposers such as fungi and bacteria. **Detritivore and decomposer food chains** are most abundant in forests (Figure 11.1.42). They are also important in aquatic ecosystems where detritus (organic waste material) builds up on the bottom of a pond, lake or bay (Figure 11.1.43).

As a detritivore eats dead leaves, it also eats decomposers (bacteria and fungi) that are on and in the dead leaves. The detritivore also has symbiotic gut bacteria (microflora), distinct from the habitat microflora, which enables it to digest cellulose and other plant matter.

Detritivores are important because they physically break down plant litter into small particles. Parts of the dead leaves that a detritivore chews pass out of the animal as faecal pellets. These smaller particles are then easier for decomposers to consume. Some detritivore animals also eat faecal pellets (either their own or those of other species). Fly and beetle maggots are two examples of animals that consume faeces and dead organisms.

The decomposers, bacteria and fungi, are the last link in this chain of eating, re­eating and breaking down detritus. The digestive enzymes that decomposers secrete break down the organic matter into soluble organic molecules such as sugars and amino acids, and eventually into inorganic nutrients such as carbon dioxide and phosphate. Some of these products are absorbed by decomposers and some remain in water, soil or the air.

Decomposers

Decomposers are the final link in the cycle of matter in ecosystems. They are able to use the materials left by all the other organisms in a food chain. All organisms eventually die, leaving carcasses. They also discard matter such as dead skin, undigested food, leaves and dead branches while they are alive. In the examples shown in Figure 11.1.44, tl1e cabbage sheds its leaves after blooming, and all animals regularly deposit faeces.

Decomposers can use these materials and transform them into simple inorganic compounds such as carbon dioxide, water, hydrogen sulfide and ammonia. These compounds can then be used by autotrophs. In this way, decomposers complete the cycle of matter. The inorganic matter taken up by autotrophs goes through producers and consumers and decomposers and back to inorganic matter.

Simple **food chains** in which the organisms have only one food source are rare in nature. A plant is usually eaten by several herbivore species. Herbivores that feed on just one plant species are rare. Predators, even those that are specialised for catching and feeding on one type of animal, can adapt to a different kind of prey if the animals that they normally hunt are in short supply. An example of a predator that has done this is the wedge-tailed eagle (Aquila audax) shown in Figure 11.1.4 7. As the diets of species become broader, the **food web** becomes more complex.

The complexity of the **food web** gives an ecosystem its stability. In a simple **food chain**, the removal of one species (i.e. one link) is likely to have a disastrous effect on other organisms. The loss of one species from a complex web has less effect, because alternative food sources are usually available. For example, the giant panda's food web is really one simple **food chain** of two species: bamboo and the giant panda. The simplicity of this food chain makes the giant panda vulnerable because it only has one food source.

Trophic levels

Each feeding level in a food chain or web is called a trophic level. All the producers in a typical food chain or web make up the first trophic level. Herbivores (primary consumers) are at the second trophic level because they feed on the producers from the first level. Secondary consumers, which feed on herbivores, are at the third trophic level, and so on. You can see these roles labelled in the food chain in Figure 11.1.48.

An organism may function at more than one trophic level in a **food web**. In the example of the pond food web in Figure 11.1.49, the catfish feeds on small crustaceans and insects, as well as snails and small fish. The catfish is a secondary consumer at the third trophic level because it eats Daphnia (also called water fleas) and a tertiary consumer at the fourth trophic level because it eats water striders that eat Daphnia.

11.2 Ecological niches

The ecological niche of a species includes all its interactions with biotic and abiotic factors in the ecosystem. This includes the resources it needs for food and shelter, as well as its ability to survive and reproduce. It includes both how a population responds to factors (e.g. plants growing when resources are abundant) and how the species affects those factors (e.g. the resources depleting through plant uptake of nutrients).

ECOSYSTEM ROLES

An organism's ecological niche is its role in its environment. This includes how the organism uses its resources and interacts with other species and its environment. It is essentially the organism's 'job description' in the ecosystem. For example, the ecological niche of a eucalypt tree in an open woodland ecosystem (Figure 11.2.1) would be:

photosynthesis

producing oxygen by photosynthesising

absorbing water and nutrients from the soil

providing shelter and food for organisms including plants, animals and fungi

stabilising the soil.

COMPETITIVE EXCLUSION

The **competitive exclusion principle** is an ecological principle which states that two species cannot have the same niche in an ecosystem. This means that two species cannot use the same resource in the same space at the same time. If two competitors try to occupy the same ecological niche, one species will eliminate the other. This may occur through one species becoming extinct, or a species adapting to fill a different ecological niche (this can include moving to a different space, occupying the habitat at a different time, or changing its diet).

However, it is common for different species to use different parts of a resource at the same time, a term called resource partitioning. For example, brown creepers (Certhia ainericana) feed on insects on the trunk of a tree, while nuthatches (Sitta sp.) feed on insects among the branches at the top of a tree (Figure 11.2.2).

Many endangered species become threatened because they are unable to adapt to different ecological niches when competition arises. For example, the Australian koala (Phascolarctos cinereus) will only eat foliage from a few of more than 600 species of Eucalyptus tree that grow in Australia (Figure 11.2.4). Further, koalas are locally specific in their diet; for example, koalas in New South Wales forage on different eucalypts than those in Victoria. An ecosystem can only support a limited number of koalas due to their specific dietary preferences; therefore, when an ecosystem becomes unbalanced, the species comes under threat. This can be seen where eucalypt forests are being cleared by humans for development, leaving ecosystems with fewer eucalypt trees for koalas. The koalas then eat more foliage than the trees can regrow and the tree and/or koalas perish.

11.3 Predicting and measuring population dynamics

Chapter 7 explored the potential for exponential growth in populations (i.e. the birth rate of a population is higher than the death rate, allowing the population to grow), but many factors can affect population size and density and prevent initial or continued exponential population growth. The size of a population is affected by many factors interacting in complex ways. However, the effects of one factor can often amplify (increase) the effects of other factors.

ENVIRONMENTAL RESISTANCE

Environmental resistance is the term used to describe the sum of the factors that limit the growth of populations. Environmental resistance includes **abiotic factors** such as temperature, drought and fire, as well as **biotic factors** such as competition, predation and disease. These factors can reduce the health, reproduction rate and survival of individuals, leading to a reduction in the rate of population growth. Factors that influence population size in an ecosystem are either density-independent factors or density-dependent factors.

DENSITY-INDEPENDENT FACTORS

**Density-independent factors** affect a population's size regardless of the size or density of the population. They include:

the conditions in which the species can survive; that is, its daily and seasonal tolerance range for various abiotic factors

major changes or disturbances to the environment, such as bushfires, droughts or floods.

Tolerance range of a species to abiotic factors

As discussed in Chapter 7, tolerance is an organism's ability to survive within the physical conditions of a location. For any one factor, such as temperature or light, there are limits that the organism can tolerate while maintaining homeostasis (internal balance). These limits are known as the organism's range of tolerance and are determined by the genotype (genes) and phenotype (observable traits), which allow the organism to adapt to its environment. Within these limits, organisms have what is known as an optimum range where they are best-suited to conditions and able to outcompete other species. A suboptimum range for an organism is the range of conditions in which it can survive, but it may be outcompeted by species that are better adapted to these conditions.

When graphed, an organism's tolerance to an abiotic factor is often a bell-shaped curve, as shown in Figure 11.3.1

Major changes to an environment

Other types of density-independent factors affecting population size are those related to sudden or major changes to an ecosystem. These can occur in a number of different ways, including:

natural disasters, such as flood, bushfire (Figure 11.3.2a), drought, volcanic eruption, tsunami, earthquake and cyclone

anthropogenic changes (human-made), such as construction or pollution.

Natural disasters and human-made changes can have wide-ranging effects on plant and animal species. The destruction of habitat (Figure 11.3.2b) can displace many organisms, and some major changes to the ecosystem can kill organisms (Figure 11.3.2c).

DENSITY-DEPENDENT FACTORS

**Density-dependent factors** influence the rate of births and deaths in a population. The effects of these factors increase as the population increases. **Density-dependent factors** include:

competition for resources, such as food, water, shelter and mates predation

crowding

parasitism (a type of symbiosis)

infectious disease.

Competition

All organisms have a set of biological requirements for their survival and reproduction. All organisms need resources such as nutrients and water to sustain themselves, shelter for protection, and mates for reproduction. If two organisms require the same resource and there is limited access to this resource, there will be competition. Competition can be intraspecific (between individuals of the same species) or interspecific (between different species).

Limiting factor

The **limiting factor** of a population's size, density or growth is the scarcest of the resources needed by a population. For example, food, water, shelter, nutrients and light are essential for a population's growth. If all of these resources except water are available in large quantities, water is the limiting factor and organisms will have to compete for it (Figure 11.3.3).

/nterspecific and intraspecific competition

Competition can be interspecific or intraspecific. Interspecific competition occurs when different species compete for the same resource. For example, different species might need tree hollows for nesting and shelter. If trees with hollows are blown over or cut down, hollows might become the **limiting factor** for these species and they will have to compete for available sites. If one species is much more abundant or much stronger than the other, then the population of the weaker species will decline.

Intraspecific competition occurs when individuals of the same species compete for a resource. For example, if the food resource of a species suddenly becomes scarce, individuals of the same species will have to compete for the remaining food (Figure 11.3.6).

Competition can result in reduced growth, inability to reproduce and death or emigration. Either type of competition is more likely to occur when population densities are high and there is greater demand for resources. However, intraspecific competition is far more likely to occur than interspecific competition, because all individuals in a population have the same basic requirements for survival. Different species generally have different basic requirements for survival, although some of their needs might overlap.

Both kinds of competition select for stronger individuals or species. In intraspecific competition only the strongest individuals will be able to acquire the resources needed to survive. In interspecific competition, if one species is much more abundant or much stronger than the other, then the population of the weaker species may decline. An example of this is shown in Figure

Predation

Another way in which a species can affect the population of another species is through a feeding relationship, such as predation. If the density of the prey species increases, predators will have more access to this source of food and their population will increase. This will then reduce the population of the prey species because more predators are eating them. As the number of prey falls, intraspecific competition in the predator population will reduce its population size.

Predator and prey relationships constantly fluctuate m this way. For example, the Canadian lynx (Lynx canadensis) preys almost exclusively on the snowshoe hare (Lepus ainericanus) (Figure 11.3.8). The population of hares varies according to factors such as climate, disease and availability of food.

An increase in the hare population leads to an increase in the lynx population. When there are more predators, the hare population may decline because more are killed by lynxes. This in turn may cause the lynx population to decline again. The graph in Figure 11.3.9 shows this repeating cycle.

Crowding

Crowding affects populations of different species in different ways. For example, when the density of aphids is low few aphids develop wings, because they do not need to move far to obtain the resources they need. However, as the population density increases, more aphids develop wings (Figure 11.3.10). Winged aphids can only produce about half the usual number of offspring, but they can disperse to areas with more resources and form new populations.

Some animals become stressed when their density is high and may produce fewer offspring, or their immune systems become affected and they will be more prone to disease infection. The density at which negative effects occur is different for each species.

Parasitism

Parasitism, a type of symbiotic relationship, occurs when an organism lives in or on its host for an extended period of time or for its entire **life cycle**. When population density increases, individual organisms have more contact with each other. They are also more likely to be weakened; for example, as a result of limited supply of a resource such as food. By these means, parasites are more easily able to invade and spread in a host species of increased density.

Infectious disease

Diseases may spread faster within a denser population, assuming that conditions are favourable for infection. Disease-causing fungi, bacteria and viruses that can kill individuals may then affect the size of the host population. However, organisms in natural environments may be infected with a disease but still be able to survive and reproduce.

A critical factor for any pathogen (disease-causing agent) is the method by which it spreads to other individuals (Figure 11.3.11). The rate at which a pathogen spreads depends on various factors, including environmental conditions such as wind, water flow and temperature. The genetic variation of the host population can limit the spread of a pathogen, because a more genetically diverse host population is more likely to have resistant individuals.

ECOSYSTEM CARRYING CAPACITY

In the absence of a limiting factor, the population growth of a species will always be exponential. However, in a real ecosystem, population growth is affected by **density­dependent factors** such as competition for resources.

When a species' population reaches equilibrium and becomes relatively constant, with the number of births and deaths in the population cancelling each other out, the species has reached the maximum population size that the ecosystem can sustain indefinitely. This is the ecosystem's carrying capacity for that species.

The S-shaped graph in Figure 11.3.13 shows the initial exponential growth of a population, which then flattens out as it begins to be affected by **density-dependent factors**. The population growth rate may decline until birth and death balance each other and the population is limited to the carrying capacity of the environment. This pattern of growth is known as logistic growth.

Consider the following example. A new volcanic island emerged in the middle of an ocean and became populated with plants and insects. A few birds of one species happened to be carried to the island by strong winds. They raised young and fed on the seeds, insects and fruit available. As they were the only bird species, they had no competition for space or food and no natural enemies, so the population grew rapidly. Eventually there was no space left for new nests. Seeds, fruit and insects became scarcer, and then rats arrived on the island by ship and began eating bird eggs. The bird population then levelled out (Figure 11.3.14).

The graph in Figure 11.3.15 shows the changes in the size of the bird species population over time. This pattern of growth is observed in every population in a natural environment.

The dynamic nature of carrying capacity

The carrying capacity of an environment is dynamic; that is, it varies over time. Factors that can affect carrying capacity include:

weather and climate changes

major changes in an ecosystem

fluctuations in populations of food species or competitors.

The factors that can affect carrying capacity can be a biotic or biotic (Table 11. 3 .1).

Water availability is an example of an abiotic factor that can affect carrying capacity. For example, during a drought water availability might become the **limiting factor** for a population of kangaroos, and the number of individuals that the environment can support will be reduced.

MEASURING POPULATIONS IN AN ECOSYSTEM

When studying ecosystems, it is often necessary to determine the type and number of populations and organisms living in an area. In natural environments, it is usually impossible to count all the individuals of a species. Even just counting the living things in your school would take a very long time. Sampling gives us a good idea of the organisms in an ecosystem without needing to count each one. Table 11.3.2 outlines some fieldwork techniques and when they are best used.

When sampling in the field, you should always consider the time and equipment available, the organisms involved and the impact the sampling may have on the environment.

Point sampling

Point sampling involves counting organisms only at selected points (Figure 11.3.17). These points might be selected randomly or regularly, depending on the type of sampling being done. It can be used to determine the range of organisms that live in an area and how common they are. Point sampling is quick, but you might miss rare organisms.

Quadrats

A quadrat is a sampling method that allows you to estimate the number and variety of organisms in a large area by counting in a small area (Figure 11.3.18). A quadrat is usually square, and sometimes rectangular but may be circular. As suggested by its name, a quadrat was traditionally a square (a shape with four equal sides). A rectangle still conforms to the four-sided nature of the name. However, you may find it odd that a quadrat can also be a circle. The following points are helpful when planning to use quadrats.

Quadrats are most useful in sampling immobile organisms such as plants or corals.

Determine the size of the quadrat based on the size and abundance of the organism that you are sampling.

The more quadrats you use, the more accurate your results will be.

Very abundant organisms can be measured as a percentage of the area covered, rather than as a number of organisms.

Photographing the quadrat can be a useful record-keeping method.

Transects

A transect is a straight line along which vegetation is sampled (Figure 11.3.19). Transects are useful for investigating the distribution of a population of plants, animals or insects across areas with different abiotic factors. For example, Figure 11.3.19 shows how a transect was used to sample and describe the change from eucalypt forest to heathland. A transect running from the sea to the land could be used to record the changes in species from rock pool ecosystems to mangroves to inland saltmarsh communities.

Physical aspects of the environment such as soil type and pH, salinity, amount of light, slope angle and height can also be measured along a transect to see if they correlate with changes in biological communities.

Techniques for sampling aquatic habitats

Marine and freshwater habitats can be sampled in many ways. Open water habitats are sampled by pulling a net through the water to collect swimming and floating organisms. Reefs and other underwater habitats are sampled using techniques similar to those used on land, such as quadrats and transects.

You might sample a freshwater habitat during your studies (Figure 11.3.20). Water is collected in a bucket and tipped into a white tray so that free-floating organisms can be easily spotted. Loose rocks are also collected to search for organisms that cling to them, such as stoneflies. Mud might also be collected and sampled to search for organisms such as nematodes and snails. Larger animals such as fish and yabbies can be collected using a net, then quickly identified and returned to the water.

Mark-recapture

In a mark-recapture study, animals are captured, marked and then released. When they are recaptured or observed again, their mark is used to identify them (Figure 11.3.21). Mark-recapture is used to determine the total population of a mobile species such as birds and turtles. It can also be used to track the movements of individual animals. However, it is very time consuming and requires a lot of expertise to be done properly.

11.4 Extinction

EXTINCTION EVENTS

Species that fail to adapt to environmental changes or to compete for limited resources can die out. This loss of a species or groups of species is called extinction.

Background extinctions

The average rate of natural loss of species is called the background extinction. Extinction can occur as a result of changes in the physical environment or changes in the ecological interactions between species, such as the arrival of a new predator or competitor. The average life of a species varies, depending on the type of organism, but is generally a few million years. Based on the fossil record, some marine animals appear to have existed for 5-10 million years, while mammals tend to last only 1 million years. The coelacanth (Latiineria chalurnnae) is considered a living fossil because coelacanths as a group date back to the Devonian period of 400 million years ago (Figure 11.4. 1).

Mass extinctions

Throughout the fossil record, there is evidence of some significant mass extinction events. Mass extinctions are large-scale extinctions following disruptive changes to the global climate, or loss of sea or land due to the shifting of continents (plate tectonics). Whether these extinctions were caused by global warming or cooling, drowning with sea-level rise, asteroid impacts, volcanic eruptions or perhaps even disease, the aftermath of such large extinction events leads to changes in selection pressures that affect the number of surviving species.

Mass extinctions, like the one that wiped out most of the dinosaurs at the end of the **Cretaceous period**, led to many vacant ecological niches. Remaining species may have taken advantage of the sudden availability of resources and reduced competition or predation.

Five mass extinction events are evident from the fossil record, and in each more than 50% of hard-bodied marine species became extinct (Figure 11.4.2). For example, during the **Permian period** (299-251 million years ago), all of the continents came together and shallow continental seas were gradually lost. This large land mass (called Pangaea) caused reduced rainfall, temperature extremes, harsh conditions and the death of many species including the extinction of marine trilobites. The fossil evidence is particularly well documented as hard-bodied organisms fossilise well, and the environmental conditions of shallow seas are ideal for the fossilisation process.

RECENT EXTINCTION

Extinction events are not limited to the age of the dinosaurs. In recent times, humans have caused species extinctions at an accelerating rate. The rate and scale at which extinction is currently occurring has led many scientists to conclude that Earth's biodiversity is undergoing an extinction crisis-the sixth mass extinction event.

Extinctions have occurred for millennia as humans arrived on new islands and continents. A background extinction rate of 1-5 species per year was estimated for most of this time. However, the current background rate is estimated to be 1000-10 000 times that, meaning we are experiencing the greatest rate of species extinction since the dinosaurs were wiped out 65 million years ago. In the last 520 years, human activity has caused the extinction of 820 species; however, there is likely a large number of unknown species that have become extinct without documentation. For example, the San Cristobal vermilion flycatcher (Pyrocephalus dubius) has recently been registered as a newly discovered species on the famous Galapagos Islands. Unfortunately, there is one catch to the discovery-the species is already Extinct (Figure 11.4.3). Researchers studying bird specimens at the California Academy of Sciences recently distinguished the bird as a unique species (rather than a subspecies of other birds); however, it was discovered that the species likely became Extinct in 1987 when the last sighting was documented. The introduction of invasive rats on the island is the likely cause of the flycatcher's extinction, with the rats feeding on bird eggs.

Species are believed to be disappearing at 100 times the natural rate, with 99% of species threatened purely by human activities. The main causes of this human-driven species and biodiversity loss include: habitat alteration (including land clearing and habitat fragmentation from roads and urban areas), invasive species, pollution, hunting and wildlife trade (such as poaching). A fifth factor driving modern-day extinctions has also been added, with global climate change now listed as a major driving force for species loss.

The first species to become Extinct as a direct result of global climate change is the golden toad (Jncilius periglenes) of Central America (Figure 11 .4.4). This unique amphibian had many curious physical and behavioural features such as black and yellow poisonous skin and a lack of eardrums. Also, without the capacity to call to other frogs the golden toad would wave its arms to communicate. The last sighting of the species was in 1989 in the Costa Rican cloud forest of Monteverde, where a team of scientists removed a population of the species to an amphibian conservation centre. It is believed that global climate change affected the golden toad's specific habitat to such an extent that the species became susceptible to a range of other pressures, eventually leading to extinction. Global climate change caused the high­altitude rainforest habitat to become hotter and drier, leaving the toad vulnerable to a fatal skin disease known as chytrid fungus. This, combined with forest clearing and overcollection of species for wildlife trade, left the species vulnerable and unable to recover.

Due to the complexity of ecosystems, any extinction event will have 'knock-on' effects to biotic and abiotic factors in the species' environment. This means that a snowball effect occurs leading to damaged ecosystems, which are less resilient to further change. An example of this is the African teak tree (Pericopsis elata), which grows in the forests of Central and West Africa (Figure 11.4. 5). Excessive logging of this species for furniture, boat and flooring trades have left the African teak tree endangered. Should this species become extinct, it would have devastating effects for the entire ecosystem, including soil stability, livelihoods of the local indigenous communities, as well as the survival of endangered species of chimpanzees, gorillas and elephants that inhabit the forests.

Extinctions in Australia

Australia has one of the highest rates of biodiversity loss in the world. The isolated ecosystems of Australia have seen the evolution of some of the world's most unique species. Many of Australia's species are found nowhere else, with 87% of mammal species, 93% of reptiles, and 45% of bird species endemic to the continent. The evolution of Australia's native species in isolation has left them vulnerable to change. When Europeans colonised Australia, their activities caused rapid alterations to ecosystems. In just over 200 years, dozens of Australian mammal, bird and plant species have become extinct and many more are threatened with extinction. The rapid extinction of species in Australia is due to a combination of factors including the introduction of invasive species, land clearance and alteration, habitat loss and altered fire regimes.

12.1 Ecosystem dynamics: changes and causes

In Chapter 7 you learnt that an ecosystem is a biological system of interacting organisms and their physical environment. Every component of an ecosystem, living (biotic) and non-living (abiotic), plays a role in the processes that occur within the ecosystem. Think of a rock pool, which is home to small fish, crustaceans, molluscs and seaweeds (Figure 12.1.1). These creatures provide shelter or food for each other and are all affected by **abiotic factors** of changing tides, wave action, wind and sunlight. We can examine ecosystems at any size and scale, from a small rock pool on the shore of a Sydney beach to the Middleton Reef ecosystem in the Tasman Sea. We can even study some of the largest ecosystems on Earth, formed by the currents of the Pacific Ocean.

The complexity of ecosystems and the many interactions between the organisms within them make ecosystems dynamic and ever-changing. A shift in one component can have a ripple effect on many components and potentially lead to changes to the whole ecosystem. We can observe these ecosystems as they are today, and technology allows us to understand how these systems have changed over time and how they may have looked in the past.

In this chapter you will learn about past ecosystems and how these complex dynamic systems and the organisms that inhabit them can change over time.

ECOSYSTEM STABILITY AND DISTURBANCE

Ecosystems are dynamic and changes are occurring all the time. Fluctuations in population numbers of a particular species or group of species can cause imbalance in an ecosystem. Ecosystem imbalance can be seen in the destruction and loss of biodiversity caused by invasive species. In a stable ecosystem, small changes are balanced out with no major effect on ecosystem processes or the structure. Stable ecosystems generally have higher biodiversity than unstable ecosystems.

Negative feedback loops

A stable ecosystem is a self-regulating system, where populations are maintained through a negative feedback loop. Feedback loops maintain balance in a system by increasing (positive feedback loop) or decreasing (negative feedback) a component in response to an imbalance in the system. For example, in a productive season with lots of sunshine and rain, plants are likely to thrive. The plants will grow and reproduce successfully leading to a spike in their population. An increase in the plant populations means there will be a lot of food available for herbivores. The number of herbivores is then likely to increase in response to the abundant food supply.

Birth rates may increase, survival rates will improve and animals may migrate to the areas with abundant food resources. If the number of herbivores becomes too high, the plants will be overgrazed and die out. This limits the food supply to the herbivores who will either move on to new habitats or starve. As the number of herbivores declines, the plants grow back and their population increases again. The overgrazing by the herbivores resulted in a negative feedback loop-the decrease in the food supply led to a decrease in the herbivores, which allowed the plant population to recover and the cycle to begin again.

Disturbance

A stable ecosystem has the ability to self-regulate and maintain its average state even in the face of disturbance. A disturbance is an event that causes a temporary change in an ecosystem. Usually, the change is a loss of individual organisms, which may in turn alter the structure and function of an ecosystem. Ecological disturbances are natural events including earthquakes, floods, storms, volcanic activity, disease and fires. Anthropogenic changes or disturbances are human activities that can alter ecosystems, such as oil spills, forest clearing (Figure 12.1.4), pollution, radioactive contamination and introduction of invasive species.

An ecosystem can be described as resistant or resilient. A resistant ecosystem is tolerant to disturbances and remains fundamentally the same over a long period of time with little deviation from its average state. A resilient ecosystem can return to its normal structure and function after a disturbance.

The impact of a disturbance on an ecosystem depends on its intensity and frequency plus the spatial scale and distribution of the disturbance. For example, an extremely hot forest fire that reaches the canopy of a forest and burns for a long time will have a greater impact on an ecosystem than a short-lived, cooler burn through the undergrowth.

Though disturbances are destructive, they also provide opportunities. When some individuals are removed from an ecosystem, space is created for other organisms to reproduce and grow. Disturbances can encourage biodiversity in an ecosystem as different species move into an area or thrive in new conditions. Biodiversity can make an ecosystem more resilient and resistant as different species will respond to future disturbances in different ways.

Intermediate disturbance hypothesis

The **intermediate disturbance hypothesis** (IDH) proposes that high and low levels of disturbance reduce the species diversity of an ecosystem. Species diversity is maximised when disturbances are at an intermediate level.

Low-level disturbances are characterised by lower intensity or lower frequency where disturbances have little effect on an ecosystem and do not occur very often.

High-level disturbances are frequent and intense, occur often and have a great effect on ecosystem structure and function.

At high levels of disturbance, few species will be able to survive and levels of biodiversity will be low. At low levels of disturbance the most competitive species will eventually dominate an ecosystem, outcompeting other species and creating low levels of biodiversity.

Intermediate levels of disturbance allow for an increase in biodiversity over time and some competition until competitive exclusion occurs.

TRENDS AND PAST CHANGES: THE GREAT

BARRIER REEF ECOSYSTEM CASE STUDY

Coral reefs are complex, diverse marine ecosystems that support a wide variety of habitats, processes and species. The reefs are built by colonies of coral polyps, which are small, soft-bodied animals that are related to sea jellies and sea anemones. Coral polyps secrete calcium carbonate to form a hard, protective base. It is these calcium carbonate secretions that form the structure of coral reefs.

The Great Barrier Reef is the largest coral reef system in the world (Figure 12. 1.8). It stretches over 2300 km along the coast of Queensland and covers approximately 344400km2. It is so large that it can be seen from outer space. The Great Barrier Reef is made up of 2900 individual reefs and 900 islands and is home to many threatened species, some of which are only found in the Great Barrier Reef area. The Great Barrier Reef was classified as a World Heritage Site in 1981 and is considered one of the seven natural wonders of the world.

Threats to the Great Barrier Reef

The greatest threat to the Great Barrier Reef is climate change, which is causing sea temperatures to rise, ocean acidification and increased extreme weather events.

Many reef species are sensitive to changes in sea temperature and pH levels. Corals may stop growing or die due to changes in these variables. Reduced coral growth and coral death has flow-on effects for other reef species that rely on corals for habitat.

Rising sea temperatures

Reef-building corals are very sensitive to the effects of increasing sea temperatures. In Chapter 11 you learnt that many corals and anemones have a symbiotic relationship with tiny, photosynthetic algae called zooxanthellae. Zooxanthellae provide the corals and anemones with nutrients as well as their colour (Figure 12.1. 9). Coral bleaching occurs from heat stress when water temperatures rise above normal levels and the zooxanthellae leave or are expelled from coral (Figure 12.1.10). The corals do not die from a bleaching event, but they will eventually starve if the zooxanthellae are not restored. Rising sea temperatures can reduce the stability of coral reef ecosystems if heat stress disturbance events occur often or over long periods of time. Consistently high water temperatures or many consecutive bleaching events can cause coral death and the destruction of reef ecosystems.

Ocean acidification

Reef corals are negatively affected by ocean acidification in two major ways. First, acidic waters inhibit the corals' ability to make the calcium carbonate skeletons that give reefs their structure. The pH of ocean water decreases (becomes more acidic) when atmospheric levels of carbon dioxide (CO2) are high and excess carbon dioxide is absorbed into the sea from the atmosphere. High levels of dissolved carbon dioxide in the ocean result in less free carbonate ions available for corals to create calcium carbonate structures. Acidic waters can also cause existing calcium carbonate reef structures (corals) to break down. In waters with pH levels that are lower than normal, calcium carbonate structures are dissolved faster than they can be replaced by corals leading to a net loss of important reef habitat for other species.

Extreme weather events

Extreme weather events such as cyclones and floods can cause long-term damage and fragmentation of reef systems (Figure 12.1.11). These weather events create strong wave actions that break the underlying reef structures (Figure 12.1.12). Turbulent waters can cause a change in water quality with decreased salinity and increased nutrient and sediment loads, which cause stress to many reef species. Coral reefs can withstand some weather disturbances, but may be unable to recover from frequent and intense weather events.

Two recent major tropical cyclone events, Cyclone Yasi (2011) and Cyclone Debbie (201 7), have caused major structural and coral cover damage to the Great Barrier Reef. Since 2005 more strong cyclones (category 3 or higher) have been recorded than in previous decades.

Coral reefs usually take 10-15 years to recover after a major disturbance event. The back-to-back nature of recent disturbances has not allowed enough time for the Great Barrier Reef to be restored to its pre-disturbance state. Overall decline in coral cover can be attributed to a change in disturbance regime where intense disturbances are occurring frequently.

The Great Barrier Reef since the 1980s

A report released in 2012 stated that the coral cover of the Great Barrier Reef had reduced substantially since 1985 due to cyclones, coral bleaching and outbreaks of crown-of-thorns sea stars, which can destroy coral when in high numbers (Figure 12.1.13).

During this time, sea surface temperatures in Australian waters had increasingly exceeded long-term sea temperature records. Extreme weather and coral bleaching events have continued to occur after this time.

Calcification (the production of calcium carbonate by corals) declined by 14.2% between 1990 and 2009. Ocean acidification had also occurred throughout this time, but is not the only factor contributing to the decline in coral calcification as other environmental changes, such as rising sea temperatures, occurred at the same time.

A study was conducted in the Great Barrier Reef to test the effect of ocean acidification on coral reef calcification. Researchers increased the pH (decreased acidity) of the water over an existing coral reef to mimic the estimated pH levels 200 years ago. Atmospheric carbon dioxide levels were much lower and oceans were less acidic 200 years ago. The study found that coral calcification was 7% higher in the reef in less acidic water (as it was 200 years ago) than in a reef in present-day conditions.

A survey conducted in 2016 found that corals in the north and far north region of the Great Barrier Reef have undergone substantial decline, with losses of up to 67% (Figure 12.1.14). This represents one of the largest coral die-offs ever recorded for the Great Barrier Reef. Although this part of the reef may recover in 10-15 years' time, it is expected that more bleaching events in this time will slow the recovery. Two-thirds of the reef was found to have minor damage with signs of recovery in the southern region. Scientists think that the southern part of the reef may be protected from heat stress by cooler water from the Coral Sea.

Bleaching events

The following is a timeline of coral bleaching events:

1998-one of the hottest summers recorded for the 20th century. Moderate to high levels of bleaching occurred where 50% of coral reefs were bleached, but most recovered fully and 5% were damaged2002-moderate to high levels of bleaching where 60% of reefs were affected and about 5% of reefs suffered high mortality

2005-06-a bleaching event occurred and was isolated to the southern regions of the Great Barrier Reef where mortality was close to 30%

2016-record-breaking temperatures were associated with a mass bleaching event in 2016 where an average 6 7% loss of coral cover was recorded in the northern third of the reef, 6% in the central region and 1 % in the south 2017-further record-breaking temperatures led to a bleaching event that substantially impacted the central section of the Great Barrier Reef.

12.2 Technology and evidence for past ecosystem change

Understanding ecosystems that existed in the past is a difficult task. How do we know what an ecosystem was like if we were not there to record data and make observations? Fortunately, present-day ecosystems contain information that scientists can use to understand what these ecosystems were like in the past. Using technology, scientists can reconstruct ecosystems to understand how they were during different periods of Earth's history. In this section you will learn about evidence for past ecosystem change and the technology that is used to understand it.

ROCK STRUCTURE AND FORMATION

A lot of our understanding of past ecosystems comes from Earth's crust. Discoveries from fossils and information gathered from mineral deposits and rock strata adds to our knowledge of what Earth was like in the past. In Chapter 10 you learnt about the process of fossilisation and how the fossil record provides insight into the evolution of organisms through geological time. The fossil record is a window into ecosystems of the past; it can tell us about the kinds of organisms that once lived, their adaptations, diet and behaviour, how abundant they were through time and the habitats they occupied.

Rocks not only preserve information about the biotic components of past ecosystems but also record the conditions of the environment throughout time. Rocks contain evidence of changes in climate, volcanic activity, atmospheric oxygen levels, sea levels, shifting landmasses and much more.

The structure of rocks gives insight into how they were formed and the conditions they have been exposed to. Igneous rocks are formed when magma or lava cools and crystallises. Some igneous rocks form on Earth's surface after a volcanic eruption but many form underground or underwater, on the ocean floor.

Sedimentary rocks are formed when small particles of weathered rock are compacted and cemented into layers. These layers accumulate over time and are visible as horizontal layers known as strata (Figure 12.2.1). Fossils are preserved in layers of sedimentary rock. Different environments contain different types of sediment. The sediment deposited in a swamp will be very different from the sediment in a desert. Layers of sedimentary rock are an important record of the environmental changes that have taken place throughout Earth's history.

Metamorphic rocks are formed when existing rocks are exposed to extreme heat or pressure, causing physical or chemical changes.

Each of these rock types tells different stories about the environment at the time of their formation.

Environmental conditions can also alter the structure of rocks after their formation. Physical weathering by wind, water and ice and temperature changes can cause rocks to crack or break into smaller pieces. The shape of rocks can give important clues about the environmental changes that have taken place over time. For example, valleys are formed by rivers and glaciers, and wave action wears away coastal cliffs. These environmental processes leave characteristic rock formations for thousands of years after the rivers have stopped flowing and the glaciers have melted. Geological events such as earthquakes and tectonic plate collisions also change the structure of rocks.

Rocks are a rich source of information about the past. An understanding of the structure and formation of rocks is an important step in understanding past ecosystems. Technology can be used to gain further insight into the composition and age of rocks, revealing more information about the history of Earth and the organisms that inhabit it.

RADIOMETRIC DATING

Radioactive or radiometric dating is a technique used to calculate the age of rocks and minerals using radioactive isotopes. Radioactive isotopes are elements that have an unstable nucleus. Over time the isotope decays and energy and matter are released as radiation and the element eventually becomes stable.

Radioactive decay occurs at a regular measurable rate for each isotope. A radioactive parent atom eventually decays into a stable daughter atom. This decay process is measured with a unit of time called a half-life. A half-life is the time it takes for half of the parent atoms in a radioactive material to turn into daughter atoms. The length of a half-life is unique to every radioactive isotope because different elements decay at different rates. For example, the half-life of carbon-14 is 5730 years while uranium-245 has a half-life of 704 million years.

A spectrometer is a scientific instrument that is used to measure the radioactivity of a particular isotope in a sample. By examining the relative amounts of parent and daughter atoms in a sample of rock or mineral, the age of a sample can be identified. For example, if 75% of the atoms in a rock sample are in the stable daughter form and 25% are still in the radioactive parent form, the rock is in its second half-life.

For example, if a sample was thought to be very old, uranium-lead dating could be used. Radioactive uranium-238 eventually becomes the stable daughter isotope lead-206. The half-life of uranium-238 is 4500 million years. A sample with 75% stable lead-206 atoms would leave a remaining 25% of radioactive uranium-238 atoms making the sample 9000 million years old.

The type of sample determines what kind of parent-daughter radio isotope dating should be used. For example, potassium-argon dating is used for molten rock and volcanic ash where potassium-40 decays into argon-30. Uranium-lead dating can be used for rocks that formed a long time ago, from millions of years to over 4.5 billion years. Carbon isotopes are used to date organic matter such as bones, wood and shells.

Carbon dating

Carbon dating is a type of radiometric dating that is used to identify the age of something that was once living (Figure 12.2.4). We can use carbon dating to extract information about the age of organisms in the fossil record. By measuring the amount of carbon-14 in a sample of organic matter, we can determine when that organism was last alive. All living organisms are constantly taking in carbon. Their bodies contain two carbon isotopes: carbon-14 and carbon-12. At any given moment, all living things have approximately the same ratio of carbon-12 to carbon-14 in their bodies as in the atmosphere.

Most carbon atoms in the atmosphere are in the stable carbon-12 form and only a small amount are carbon-14. Cosmic rays from the Sun create energetic neutrons that collide with atoms in the atmosphere. Carbon-14 is created when an atmospheric nitrogen atom gains a neutron, becoming radioactive and unstable. Both carbon isotopes can combine with atmospheric oxygen to create carbon dioxide (CO2).

Plants absorb carbon dioxide through photosynthesis and some of the carbon dioxide they absorb contains radioactive carbon-14. Animals also acquire radioactive carbon by consuming plants and other organisms. The carbon-14 in an organism is constantly decaying and being replaced. When an organism dies, it stops taking in carbon. The carbon-14 already contained in the body of the organism is not replaced and decays at a predictable rate while the amount of carbon-12 stays the same. By measuring the amount of carbon-14 and carbon-12 in a dead organism and comparing that with the amount in a living organism or the atmosphere, we can calculate how long ago the organism died.

Carbon dating cannot be used for organisms older than 50 000 years. The relatively short half-life (5730 years) of carbon-14 means the amount of carbon-14 is too small to detect after this time. Analysis of single samples can only tell us about an individual organism. With a suite of samples and data, we can learn more about changes in communities through space and time. Using this information, scientists can understand more about population booms and extinctions of species, expansions and collapses of ecosystems, and the role that fluctuating environmental conditions played in changing past ecosystems.

ICE CORE DRILLING

An ice core is a cylinder-shaped sample of ice that has been drilled out of a glacier or ice sheet. Examining ice cores can provide us with information about abiotic and biotic factors in past ecosystems.

Glaciers and ice sheets are formed by gradual accumulation of snow. Snowfall builds up in layers over time; more and more snow is added every year and the underlying layers of snow get compressed, eventually turning into ice.

Collecting ice core samples is a difficult process where a long vertical hole is drilled from Earth's surface and a 'core' of ice is removed from the hole (Figure 12.2.5). The ice core is like a timeline; the ice furthest away from the surface is the oldest and the ice on top is the most recent. Seasonal differences in snowfall are visible as annual bands or layers along the ice core, but these bands are more difficult to see the further they go back in time. Numerical flow models have also been developed to date ice cores more accurately. The longest ice core ever sampled is from Antarctica-it is 3 km long and dates back 800 000 years.

Evidence about past air temperatures, precipitation rates and atmospheric gases are trapped within the layers of ice. Small particles such as pollen, microbes, dissolved chemicals and air bubbles that were captured at the time of snow fall, can be extracted and used as if they were samples taken at that time. To find out information about a particular time in the past, scientists isolate the layer or layers of ice corresponding to the time frame that they want to examine.

HUMAN RECORDS

Along with using geological evidence, we can examine ancient human records to understand ecosystems of the past. Ancient humans may not have recorded observations in the same way or for the same scientific purposes as we do today, but the records of our ancestors still hold a wealth of evidence about ecosystem change.

Australia is home to one the oldest lineages of modern humans and culture. By examining the historic artefacts of Indigenous Australians, we can collect information about Australia's natural history and gain insight into the changes that occurred in the ecosystems of Australia's past.

Indigenous Australian art

Australia has some of the oldest artistic images in the world, with a long tradition of rock painting and engravings. Rock paintings created by Indigenous Australian people date from modern time back to 28 000 years ago. Rock painting is a significant part of the history and culture of Indigenous Australian people and is an important record of the people and the places they lived.

Ancient art usually depicts the types of animals that were abundant or important at the time the artwork was created. Examining the subject matter of the ancient rock paintings of Indigenous Australians can tell us about the diversity of species at that time, where they lived in the past and changes in animal communities over time.

For rock paintings to be used in reconstructing past ecosystems we must understand two things: what is depicted in the artwork and the age of the rock art. Using radiometric dating, it is possible to discover the age of the rock painting. Samples of pigments may be taken directly, but this method can be destructive to the artwork. To preserve a rock painting a sample of material overlying the artwork, including sediments or mud, can be taken. In northern Australia mud-wasp nests overlying rock paintings have been used to date rock paintings underneath. Carbon dating was used to estimate the age of the wasp nest as approximately 17 000 years old. This indicates that the rock paintings underneath the wasp nest is at least 17000 years old.

Rock paintings found in Arnhem Land in the Northern Territory depict species that are no longer found in that area and species that are Extinct. Representations of animals that look like the Tasmanian devil (Sarcophilus harrisiz), thylacine ( Thylacinus cynocephalus) and numbat (Myrniecobius fasciatus) provide evidence that these animals existed alongside the communities of Indigenous Australians in Arnhem Land between 1500 and 20000 years ago (Figure 12.2.7). It is thought that the thylacine became Extinct on mainland Australia between 2000 and 3000 years ago and colonial documentation suggests the species was only found in Tasmania by the time Europeans colonised Australia. Rock paintings from across Australia indicate that the thylacine once had a much broader distribution on mainland Australia, providing evidence that has helped scientists reconstruct the historical distribution of this species.

Ubirr is a rock site in Kakadu National Park in the Northern Territory that has a rich collection of Indigenous Australian rock paintings. Most of the paintings at this site have been dated to approximately 2000 years ago, a time that is known as the freshwater period in that region. The rock paintings depict a time of abundant food supply with many images of fish, mussels, waterfowl and goannas. The collection of the rock paintings at Ubirr provides an important record of the local ecosystems 2000 years ago and helps us to understand the changes that have occurred in the region since.

Middens

Studying how humans lived in the past can tell us a lot about the way they interacted with the environment and the populations of organisms that existed at the same time. A midden is a historic site of human occupation where people left debris from their meals (Figure 12.2.8). In Australia, the middens of Aboriginal and Torres Strait Islander people contain animal remains such as sea shells and bones.

Studying the contents of middens can indicate the main food sources that made up the human diet and how it may have changed across different seasons or over a long period of time. Some middens were used by many generations of people and can be metres deep. The size of a midden depends on the length of occupation at that site. Middens are mostly found on the coast or near lakes, rivers and estuaries. Lake Mungo is a dry lake in south-west New South Wales and is a rich archaelogical site providing a window into the lives of the Indigenous Willandra people. The remains of fish, shellfish, yabbies and mammals have been found in middens around Lake Mungo. Evidence of human habitation near Lake Mungo dates back to 50 000 years ago.

Middens are distinct from natural deposits of shells and bone because they contain a high proportion of mature, edible species. Natural shell bed deposits include juveniles and inedible species.

12.3 Living evidence of ecosystem change

Examination of current ecosystems and living populations of organisms can inform us about changes in the recent past and long ago. Comparison of present-day plant and animal species with their ancestors can provide insights into the way Australia's climate and environment has changed and influenced evolution. The extinction of species indicates that these species were not as well adapted to ecosystem change as those that persist today.

GONDWANA: ANCIENT SUPERCONTINENT

In Australia, the evolution of sclerophyll plants and small mammals provides evidence for historic climate change in Australia. Australia was once connected to a tropical supercontinent called Gondwana, which included most of the land masses found in today's Southern Hemisphere.

Over the period of 132-96 million years ago the land mass that now forms Australia separated from Gondwana. Australia completed its separation from Antarctica 30 million years ago and has been isolated from other land masses since. Initially Australia was warm and humid. Fossil evidence shows that most of the country was dominated by tropical rainforest habitat. This is hard to imagine in present-day Australia where desert and semi-arid environments are dominant.

After Australia's separation from other land masses it drifted north and its weather patterns changed; temperatures increased and rain became more seasonal. These changes eventually led to long-term changes in climate and the arid environment that we see in Australia today. Surviving pockets of tropical rainforest still exist in Australia and contain the same kind of species that might have been present on Gondwana 100 million years ago. One of the most extensive surviving pockets of Gondwana rainforest is in the Gondwana Rainforests of Australia World Heritage Area, in New South Wales and Queensland (Figure 12.3.2). Many species that are found in Australia today evolved from these early tropical Gondwanan species.

ADAPTATIONS TO CHANGING ECOSYSTEMS

Adaptations of present-day species indicate the drivers of change that have acted on them in the past. A comparison of living species and their ancestors indicate past selection pressures.

Sclerophyll plants

Sclerophyll plants are well adapted to harsh, dry climates and nutrient-deficient soils (Figure 12.3.3). They have hard, tough leaves and some contain toxic or indigestible chemicals, making them unpalatable to herbivores. Sclerophyll plants include Eucalyptus and Acacia, the dominant shrubs and trees found throughout the forests, savannahs and scrubland of present-day Australia. Today there are more than 800 species of Eucalyptus and 900 species of Acacia found in Australia. Eucalyptus and Acacia originated between 50 million and 35 million years ago, but few species existed during this time and their distribution was limited.

The spatial spread and speciation of Acacia and Eucalyptus coincided with an increase of charcoal deposits in the fossil record about 20 million years ago. Incidences of fire increased as the Australian climate became hotter and drier. Rainforests declined and Gondwanan species were restricted to small areas of the country where there was higher rainfall and sufficient soil nutrients. Sclerophyll plants being well adapted to more arid conditions began to diversify to take advantage of the newly opened ecological niches where rainforest species could no longer survive.

Small mammals and kangaroos

Australia's shift to a drier, hotter climate and the spread of arid-adapted vegetation also affected mammal species. When rainforests dominated Australia, mammal diversity was at its peak.The rainforest was home to the ancestors of modern mammal groups that are now found in drier parts of Australia. Many small, arboreal (tree­dwelling) mammals existed, living in trees. As climate change occurred and the vegetation changed from tropical rainforest to more open sclerophyll forest and grasslands, tree-dwelling mammals became limited to pockets of rainforest and larger ground-dwelling animals began to dominate.

The evolution of the kangaroo species that are found all over Australia today follows this pattern. Around 25 million years ago ancestors of the kangaroo existed in the rainforest. Their ancestors were arboreal and had possum-like features, climbing up trees and running through the forest floor on all fours.

Between 15 million and 20 million years ago kangaroo species diversified and expanded their range, taking over open woodlands and grasslands. As arid areas continued to increase, the kangaroo ancestors adapted to moving on just two legs (bipedal locomotion) and their teeth adapted to grazing on grass and tough sclerophyll plants. The red kangaroo (Figure 12.3.4) appeared about 2 million years ago and became increasingly successful as other large herbivores began to die out.

The success of modern kangaroos might be attributed to their ability to hop. Hopping is a very efficient way to move, allowing kangaroos to cover large distances without expending much energy. This adaptation would have helped the kangaroo reach food and water quickly as these resources became scarce and farther apart with a drying climate. Hopping locomotion would have been much easier and more advantageous in open woodlands and grasslands than in dense rainforest. Kangaroo-like megafauna that existed alongside modern kangaroos include Procoptodon goliah, which was up to 3m tall and weighed up to 240kg (Figure 12.3.5). These large animals disappeared about 15 000 years ago and are thought to have walked upright instead of hopping.

The closest surviving relative of early kangaroos is the musky rat kangaroo which evolved 20 million years ago. The musky rat kangaroo still exists today in the tropical rain forests of north-east Australia. It is a small marsupial that retains some of the ancestral possum-like features, which have been lost in modern kangaroos. The musky rat kangaroo is well adapted to life in the dense forest. Its hind feet have grooves on the pads and a mobile first toe, which help it to climb through the undergrowth and obstacles of the forest floor.

LOCALISED EXTINCTION

Local extinctions are evidence for ecosystem change. Local extinction (also known as extirpation) occurs when a population of a particular species ceases to exist in an area of its former range, but other populations of the species still exist elsewhere. When a species persists in one area but its numbers have declined in another area, we can infer that a selection pressure is occurring in one habitat that is not occurring in other areas. Species with specialised requirements for diet or habitat are more susceptible to selection pressures and ecosystem change.

By examining local extinctions, it is possible to identify potential threats to a species as a whole. Factors leading to extinction are often complex and may be acting independently or together. Local extinctions are more likely to occur when populations become fragmented and migration of individuals in and out of an area is limited.

For example, in Australia koalas (Figure 12.3.6) have a large distribution from north-eastern Queensland through New South Wales and Victoria to a small part of South Australia. The national distribution of koalas has not drastically reduced since the end of the fur trade in the 1927, but individual populations have declined and local extinctions have occurred since this time. Koala populations have become fragmented and increasingly isolated from one another due to land clearing and road construction for urban development (Figure 12.3.7). Land clearing creates habitat fragmentation, which isolates koala populations. Population isolation prevents the migration of individuals and genes between populations, creating pockets of smaller, less-diverse populations that are more vulnerable to selection pressures, such as disease, drought and extreme heat. Urbanisation and the development of roads also increases the mortality of koalas due to vehicle strikes and dog attacks.

Species that have suffered local extinctions may be good candidates for reintroduction to their habitat once the threatening processes have been eliminated. It is important to identify the selection pressure or ecosystem changes that caused the local extinction before reintroducing the species. Once the selection pressure is managed or removed the species can be reintroduced into the same habitat. It is also possible to introduce species to new areas if the habitat is suitable and there are no threatening processes present.

INVASIVE SPECIES CHANGE ECOSYSTEMS

Invasive species are organisms that are able to establish populations outside their natural ranges. These species are not normally part of the ecosystem where they have become established, and their ability to maintain a self-sustaining population can have drastic consequences for native biodiversity and ecosystems (Figure 12.3.10). Most species that are introduced to a new ecosystem or habitat do not establish and invade, but when they do the effects on ecosystems are often difficult to reverse and can have long-term conservation and economic impacts. For example, the introduction of a parasitic insect to an agricultural ecosystem could destroy crops and cost the agricultural industry millions of dollars. For these reasons, many countries have strict biosecurity laws to prevent potentially invasive species crossing their borders.

Invasive species pose a threat to ecosystems when their success in a new environment displaces the native species of that area. Invasive species have the largest impact on island and isolated ecosystems because species in these ecosystems have evolved in isolation, are often highly specialised and have had very few encounters with new species. New species coming into these vulnerable ecosystems often have few natural predators or competitors and niches in the community may still be vacant. These features make islands and other isolated areas very susceptible to invasion when a new species arrives.

13.1 Human-induced changes leading to extinction

Understanding causes of past extinction events is essential in preventing further extinctions and loss of biodiversity. In most cases a combination of factors contribute to the decline or extinction of a species. Species extinctions have been occurring since the beginning of life on Earth. An estimated 30 billion species have existed since early multicellular life forms evolved and only about 0.1 % exist today. Extinction is a normal and natural part of the evolution of life on Earth. A base rate of extinction describes the historically typical rate of loss of species on Earth. There have been five mass extinction events in Earth's past where the rate of extinction markedly exceeded the base rate and was very high through a short period of time. Causes included volcanic activity, asteroid impact, and changes in climate, sea levels and atmospheric and oceanic chemistry. It seems we are currently witnessing another mass extinction event where species are being lost at 1000 to 10 000 times the base rate.

In any ecosystem where one population or species suddenly thrives, others become displaced. This is currently occurring on a global scale as the human population continues to soar and many other species disappear. People's ability to spread out all over the world, utilising and changing the environment, creates anthropogenic pressure on other species. Anthropogenic pressures are caused by human activity and restrict the range of other species whose population numbers and genetic variation may eventually become so low the species becomes extinct. The primary threat to biodiversity and causes of extinction are habitat destruction, invasive species, pollution, overexploitation and climate change.

HABITAT DESTRUCTION

Habitat destruction is a leading cause of the global loss of biodiversity and of species extinction. Habitat destruction occurs when human activities alter or remove a natural habitat and the organisms previously existing in the habitat can no longer survive. More than 50% of Earth's land area has been modified by human land use changes and all aquatic environments have been affected. The main reason humans change natural habitats is to sustain our ever-increasing human population. With advances in technology, the life-span and the survival and reproduction rates of humans are increasing. Natural habitats are changed or destroyed to harvest resources so that the human population has food, water, energy and a place to live.

Reasons for habitat destruction include:

agriculture, through the conversion of complex habitats to sustain only a few species of crops or livestock

mining and oil, gas and geothermal exploration and development

logging, which involves clearing areas of forest for timber

urbanisation and infrastructure development, creating urban areas and road networks

water body restructuring that diverts natural water flow and storage for irrigation and livestock and for drinking water for towns and cities

•trawling and dredging, fishing practices that alter the ocean floor

•waste disposal areas used as landfills or dumping grounds for toxins and nuclear waste

outdoor recreation, with humans accessing more remote areas for activities (e.g. skiing, off-road driving).

Different types of ecosystems have differing values in terms of resources for humans. Not all types of ecosystem have undergone the same amount of habitat destruction. Some habitats have only been slightly modified on a global scale such as deserts, which do not provide a particularly good place to grow crops and pose difficulties for human survival and urbanisation. Rainforests, on the other hand, have been disappearing at a huge rate since the beginning of the 20th century. In Australia 75% of rainforest habitat has been lost. Habitat destruction often occurs in the same areas as high levels of biodiversity.

Biodiversity hotspots

A biodiversity hotspot is a region that hosts a large amount of biodiversity and also experiences a lot of habitat destruction. These areas have high concentrations of endemic species found nowhere else in the world and which are under serious threat from extinction due to loss of habitat.

Ecologist Norman Myers worked on creating a list of biodiversity hotspots that met certain criteria. Myers identified 25 biodiversity hotspots around the world that are undergoing substantial habitat destruction. Today 36 global hot spots have been recognised as meeting Myer's definitions (Figure 13 .1.1). To qualify as a biodiversity hotspot a region must contain at least 0.5% of the world's plant species as endemic and must have lost 70% or more of its original habitat. Historically these hotspots covered 12% of Earth's land but today their undamaged habitat covers less than 2% of the land. This tiny percentage of land area is home to a huge number of species: 50% of the world's plant species and 42% of the land vertebrate species.

Two biodiversity hotspots have been identified in Australia: Kwongan (south­west Australia) and the forests of east Australia.

Habitats can be lost by complete destruction where one habitat is converted to a completely different sort. Take, for example, a forest knocked down to clear land for farming or the development of housing. The forest that contained ecological niches for an array of species is replaced by buildings or simple pastures, which cannot support species that rely on trees. Species are likely to become extinct if they are destroyed in the deforestation process or if they cannot reach a new forest habitat.

Habitat fragmentation

Another way that loss and change of habitat can cause extinction is through habitat fraginentation. Habitat might not be destroyed completely but an area of habitat that was once large and continuous is broken up into smaller patches with dissimilar habitat in between. This changes the amount of habitat available and the spatial distribution of the habitat, which leads to reduced connectivity throughout the habitat.

Habitat fragmentation can cause local extinction for a number of reasons.

Smaller population sizes

Fragmentation of habitat generally isolates a once large population into smaller populations that cannot breed with each other. An isolated population might only contain a subset of the genes of the original population. These genes cannot be replenished as migration and gene flow is restricted by the new habitat that has divided up the old habitat. Inbreeding can occur and the population is likely to become extinct.

Lack of habitat between fragments

Some species are area sensitive, and may need to forage or hunt over very large areas using multiple fragments. Travelling between fragments can cause mortality as fragmented habitat is often divided up by dangerous areas like roads.

The bare-nosed (common) wombat (Figure 13.1.2) has a home range of up to 27 ha. When travelling on highways through eastern New South Wales and Victoria, it is very likely that you will spot a wombat that has become roadkill. The large home range of wombats means they might not have enough continuous habitat to use without having to cross roads.

Edge effects

When habitat is fragmented, an abrupt boundary occurs between the existing and altered habitat. When a large area of habitat is divided up into smaller patches, boundary areas increase and more edges exist. The edges of a habitat have different species compositions and **abiotic factors** than the middle of a habitat. The edges of forests often have higher temperatures and lower soil moisture and humidity than the middle of a forest. If the forest becomes fragmented into patches, the habitat will have more areas with high temperatures and low humidity than before. Plants that need lower temperatures and high soil moisture and humidity will be less likely to survive. The distance between the edge and middle of a patch also decreases with habitat fragmentation. Edges that have been created by human activities are also more abrupt than natural habitat edges, which normally display a more gradual change between habitats.

INVASIVE SPECIES

Invasive species have caused extensive biodiversity loss worldwide. The success of invasive species is often due to their association with humans. Species have been introduced to new areas by humans either accidentally or deliberately, and the movement of humans across the globe has enabled species to establish in areas they would not normally reach. Invasive species include pests, weeds, disease and parasites.

Im pacts of invasive species

Invasive species can cause declines in native populations through a range of direct and indirect interactions.

Predation and herbivory

The greatest cause of native extinction by invasive species is from the introduction of new predators or herbivores. When a species has evolved in the absence of another, it is unlikely to have defence mechanisms to combat predation or herbivory by the new species.

In Australia 30 mammal species have become Extinct since the arrival of European humans and animals. Introduced cats and foxes have contributed to at least 20 of these extinctions and still pose threats to the small- and medium-sized mammals that exist in Australia today such as species of bandicoots, wallabies and native rodents. Foxes hunt animals up to 5.5 kg and cats prefer prey under 2 kg. Australia's mammals are very vulnerable to predation from cats and foxes due to having evolved without the presence of feline or canine mammalian predators­with the exception of the dingo, which has only existed in Australia for around 4000 years. The dingo has not had the same impact as cats and foxes, perhaps because its numbers have always been comparatively low and because it favours different prey and hunting techniques. Feral cat numbers are estimated to be up to 18 million and each cat is capable of catching up to 30 prey each night. Cats and foxes face few predators in Australia and their population numbers have gone uncontrolled for a long time. Foxes and cats are both generalist hunters and vary their diet depending on the availability of prey. When rabbits are plentiful they are favoured for prey.

Introduced herbivores such as camels (Figure 13.1.3) and rabbits can cause significant land degradation and pose problems for Australian wildlife. Control regimes to limit rabbit numbers can cause diet shift in foxes and cats, resulting in higher predation of native mammals. Native mammals are especially at risk in areas where fire regimes are poor and agriculture has degraded their natural habitat. In these areas ground cover is removed and there are fewer places for native mammals to shelter and hide, making predation much easier for cats and foxes.

Poison baiting, shooting and trapping programs have been set up all over Australia to attempt to reduce feral fox and cat numbers but populations are so large and widespread that it is a very difficult and costly task. Research suggests that dingoes might be useful in controlling fox and cat numbers. Dingoes can pose a threat to livestock so populations have mostly been removed or heavily controlled in agricultural areas. In places where dingo populations still exist, cat and fox numbers are lower. It has been shown that the dingo preys on these invasive species and the presence of dingoes also limits the hours that cats spend hunting. Cats come out much later at night when the dingoes are less active. Native mammal species are also less active during this time, decreasing their chance of being discovered and eaten by a cat.

Habitat modification

The presence of an invasive species can cause modification of an entire habitat and threaten the existence of native species that require a particular environment for survival. The behaviour of an invasive species can strongly affect the native organisms in the area it invades, changing species abundance and distribution. An invasion can make a habitat more uniform and remove a complex arrangement of microhabitats. For example, beavers from North America were introduced to Argentina in the 1940s to begin a fur trade (Figure 13 .1.4). The beaver population started witl1 only 50 or so individuals but a lack of predators and competitors and plentiful resources for beavers caused rapid population growth and widespread distribution. Invasive beavers in Argentina are causing modification of forest and stream habitats where tl1ey rapidly chew down trees to create dams. The tree species in Argentina are not able to grow back once chewed down to roots, unlike tl1e species found in North America. Beavers also modify the shape and structure of streams, which has a great impact on the water cycle. Beavers have converted millions of acres of standing forest to logged areas and turned many streams into boggy wetlands. Native species that live in forests and flowing fresh water have been displaced. This habitat change can also facilitate the spread of other introduced species that take advantage of the grassland habitats where native forests are unable to regenerate.

Competition

Invasive species can create new compet1t1ve interactions with native species for food and space. An invasive species might occupy a similar niche to a native species requiring the same habitat and resources. According to the **competitive exclusion principle** two species cannot coexist if they use the same resources in the same way and eventually one species will out-compete the other. Invasive species may be strong competitors against natives and cause native extinction if they are dominating resources. In the UK introduced grey squirrels from North America have a competitive advantage over the native red squirrels of Europe. Both species of squirrel feed on acorns but the grey squirrel has a tolerance to chemicals found in unripe acorns. This means grey squirrels can eat unripe and mature acorns but red squirrels can only eat ripe acorns, as they find the unripe ones unpalatable. Grey squirrels get to the food resource of acorns first, eating them in the unripe stage which limits the number of acorns that can ripen and become available to the red squirrels. Red squirrels may be prevented from reproducing if they cannot gain enough food. Their population numbers are declining while grey squirrels become more abundant (Figure 13 .1. 5).

Characteristics of successful invaders

Many invasive species have established populations and become successful invaders in Australian ecosystems. Most of these invasive species arrived with European colonisation. Some species were brought over intentionally to be used for hunting or agriculture, such as foxes, rabbits, goats and cats (Figure 13.1.6), while others were accidentally introduced, such as rats and house mice. Many of these species have characteristics that allow them to readily adapt and survive in new environments.

Examining a range of ecosystems that have become invaded and the types of species which invade successfully reveals common traits of susceptible ecosystems and successful invaders.

Few enemies

When a species arrives somewhere outside of its natural range it may escape its natural enemies. A predator or competitor that existed in its natural environment is unlikely to exist in its new environment. The introduced species can quickly become invasive because its population numbers are not controlled. For example, a plant species may become invasive in a new environment if no herbivores exist in that ecosystem to limit its population growth.

Rapid growth, maturation and reproduction

Species that grow, mature and reproduce quickly are more likely to be successful invaders. Producing large numbers of offspring allows populations to grow and spread quickly. Covering large areas of habitat in large numbers helps to ensure the species' persistence even if mortality is high within its populations.

Human association

Species that are often associated with humans are likely to reach new environments outside their natural habitats. This can happen intentionally where people deliberately bring organisms into a new ecosystem for hunting, agriculture or aesthetic reasons. It may also occur unintentionally where a species is unknowingly introduced. Animals such as rats and mice have become successful on nearly every continent on Earth. They often stowed away on ships and could survive the journey then thrive once they reached new environments.

Adaptability

Generalist species can tolerate a range of environments and food sources, making them very adaptable to new environmental conditions. Having a varied diet contributes to the success of a species outside its natural range; species with specialised diets (specialists) are not likely to become successful invaders as their specific food source might not be found in a new environment. Koalas are an example of a specialist species, as they only eat Eucalyptus leaves and would therefore not survive anywhere outside the range of particular species of Eucalyptus. Invasive plants are often generalists and can tolerate various temperatures and soil conditions. Generalist plants that do not require specific pollinators or seed dispersers may be able to improve their reproductive success in their new environment by attracting and utilising the local animals as pollinators and seed dispersers.

Feral cats (Pelis catus) are one of the most successful invasive species in Australia. They have many of the characteristics of successful invaders: no natural predators in Australia, rapid growth and reproduction, a close association with humans and have adapted to a range of environments. Feral cats now pose one of the greatest threats to Australia's native wildlife.

Characteristics of vulnerable ecosystems

Some ecosystems are more likely to be susceptible to invasive species than others (Figure 13 .1. 7). Ecosystems that are most readily invaded often share common characteristics. Usually the ecosystem has 'space' for the invader; this may be because a disturbance event has left a niche unoccupied or because the niche was never occupied. An invader is also likely to be successful if the new ecosystem is similar to its native ecosystem.

Disturbed ecosystems

A recently disturbed or degraded ecosystem has a higher likelihood of being invaded than a stable ecosystem. Invasive species may be able to become established in a new ecosystem if disturbance has weakened species interactions and the population numbers of native species. In Chapter 12 you learnt about New South Wales sea urchins invading south-eastern Australian waters as an example of two different disturbances allowing for invasive success. The warming currents have allowed the sea urchin to expand its range. This plus the reduction in numbers of rock lobsters, the sea urchins' natural predator, have contributed to the invasion and establishment of sea urchins in kelp forest ecosystems.

Niche and resource availability

Invasive species are successful in ecosystems with high resource availability and unoccupied niches. If resources are more plentiful in a new ecosystem the introduced species may be able to grow faster and reproduce better than in its natural ecosystem. A new species in an ecosystem might also be able to take advantage of unoccupied niches without being limited by competition.

Moderate climate

Ecosystems in extreme climates are less likely to be invaded than ecosystems in moderate climates. Moderate climates are more likely to be within the range of survival for most species. The extreme environment of Antarctica means few introduced species have managed to survive and establish in the cold climate. The opposite is true for temperate and tropical climates where many invasions occur. However, as climate change warms Earth and more tourists are reaching Antarctica, invasive species may become more of a problem in this region.

OVEREXPLOITATION

Overexploitation has caused many species to become Extinct. Harvesting some species for food or products can remove individuals from a population faster than they can reproduce. European colonisation saw the exploitation of species on most continents. Previously abundant, large or edible species were harvested to extinction. A classic example is the case of the passenger pigeon, which numbered in the billions at the beginning of the 1800s and was the most numerous bird in North America. By 1900 none survived in the wild and they had been hunted to extinction.

An increasing human population, shrinking habitats and advances in technology mean that harvesting most wild populations is no longer sustainable, yet harvesting of these populations still continues. Unfortunately, as a species disappears demand for the species increases because products derived from the species become rarer and more expensive. Many mammalian species have suffered from exploitation where they have been hunted for fur, horns or antlers, or trapped and traded as exotic pets. As exploited animals become rarer, more restrictions have been placed on hunting these populations and trading animal products. This has created a black market and caused illegal poaching in reserves and protected areas, further depleting population numbers.

Overexploitation greatly affects marine plants and animals where large, commercial fishing operations occur on a global scale and exploit over 400 species. An estimated 90% of all fish stocks are overexploited, depleted, fully exploited or in recovery from exploitation. Some fishing techniques also lead to a decline in non­target marine species. Seabirds, turtles and marine mammals can get caught in nets and lines intended to catch fish and edible crustaceans.

POLLUTION

Pollution of the environment is another anthropogenic driver of species extinction and declines in biodiversity. Chemical and physical changes caused by pollutants can affect nutrient cycles and cause changes in environments and ecosystems. Pollutants released by human activities are present in terrestrial and aquatic environments as well as the atmosphere.

With improved technology, chemical production increased 400 times from 1930 to 2000 causing abnormal levels of naturally occurring chemicals and synthesis of new chemicals. Fertilisers have been used to increase crop productions to keep up with a growing human population and more mouths to feed. Fertilisers are applied to agricultural land and contain naturally occurring minerals like nitrogen, phosphorous and potassium. Only some of the fertiliser is taken up by plants; the rest accumulates in soil, leaches into the groundwater or ends up as run-off in surface waters where it can reach streams, rivers, lakes and oceans. Fertiliser run-off brings new nutrients into waterways and can cause eutrophication (Figure 13 .1.10), where excess nutrients in the water allow an overgrowth of plankton and algae. Algal blooms take over where they flourish using high levels of phosphates and nitrates but eventually, when these chemicals run out, the algae dies and decomposers flourish. Large numbers of decomposers mean they use up most of the available oxygen in the water and species that require oxygen-rich waters die. This process can cause dead zones where algae and decomposers persist and all other species in an ecosystem are lost.

Litter and plastics pollute water and cause mortality among many aquatic species. Around 300 million tonnes of plastic is produced each year worldwide, a huge number that is continually increasing; 10-20 million tonnes of that plastic end up in the oceans. Most plastics take hundreds of years to break down and plastic floats so it does not all just sink to the bottom of the sea floor. The Great Pacific Garbage Patch was discovered in 1997 and is a huge accumulation of plastic. The extent of the patch is large, possibly about the size of Australia but estimates are difficult as the plastic is suspended in water. It is mostly composed of small plastic particles in the top 10 m of the water, with some larger plastic objects floating around as well. There is six times more plastic than zooplankton in this area.

Small plastic particles can enter **food webs**, where they are taken up by zooplankton and transferred to higher trophic levels. Many large species mistakenly ingest plastic as well. It is easy to see how a plastic bag in the water could look like a jellyfish or other edible sea creature. Autopsies performed on sea life often reveal a lot of plastic in the stomach contents that can prevent the organism from feeding properly (Figure 13 .1.11).

Pollution of the air and atmosphere also occurs from human activities, affecting species all over the world. Many chemicals that are used in air conditioners, pesticides and aerosols destroy the ozone layer in the upper atmosphere. The ozone forms a protective layer that restricts harmful wavelengths from reaching Earth. When ozone is depleted it lets more UV-B rays reach Earth's surface. B radiation can inhibit reproduction of single-celled organisms like algae and phytoplankton that support **food webs** of many species. Reduction in these producer populations could cause collapses and extinction in a huge amount of consumer species.

Acid rain is caused by pollution in the air and can have negative effects on aquatic and terrestrial ecosystems. When burning fossil fuels sulfur dioxide and oxidised nitrogen are released, which form acids in the atmosphere. When it comes back down to Eartl1 as rain, it sinks into the soil and ends up in lakes, streams and oceans. Acidic water absorbs more aluminium than water with neutral pH levels. Low pH and high levels of aluminium are harmful to many fish and aquatic invertebrates. It can kill them directly or disrupt reproduction (e.g. prevent eggs from hatching).

Low pH levels in soils also kill important microbes, and chemicals needed by plants are leached away from roots. Acidic soils also allow mobilisation of toxins like aluminium, making it difficult for plants to take up water. Acid rain can also cause physical damage to plants, leaves and other structures, impeding photosynthesis and reproduction.

CLIMATE CHANGE

Human activities that cause pollution also drive climate change. Burning fossil fuels and some agricultural practices produce greenhouse gases that are released into the atmosphere. Greenhouse gas emissions cause an overall surface warming effect on the planet which in turn changes water systems and patterns of extreme weather. The cumulative effect of climate change place selection pressures on species all over the world.

Most species are adapted to a particular temperature range, and temperature change in their natural habitat may mean that habitat is no longer suitable. Some species can migrate and move to areas with appropriate temperatures as global averages increase. Other species that require extremely cool temperatures or are found in already warm environments are threatened with extinction.

Global warming can have direct impacts on species reproduction. Many species of reptiles lay eggs whose sex is determined by incubation temperature. For example, the New Zealand tuatara (Figure 13.1.12), the only living reptile of the Sphenodontidae family, is experiencing a change in sex ratios throughout the population where males are outnumbering females. Studies have shown that incubating eggs at higher temperatures (above 22°C) produces males, while lower temperatures (under 21 °C) produce females. As global temperatures increase, the tuatara population could eventually lose its females and the species would no longer be able to reproduce.

Shifts in temperature also cause shifts in water. Warm temperatures are causing sea ice to melt which means there is more water in the ocean and sea levels are rising. Thermal expansion also adds to sea level rise where increased ocean temperatures mean the water expands and takes up more space. Low-lying coastal areas are most affected where sea level rise may destroy those habitats. The first mammalian extinction directly related to global warming and sea level rise occurred in 2016. The Bramble Cay melomys (Meloinys rubicola) (Figure 13.1.13) was a small rodent endemic to the Great Barrier Reef. Sea level rise occurred at 6 mm per year between 1993 and 2010 in the region which is twice the global average. This species of melomys lived on a small coral reef and the cause of extinction seems to have been from ocean inundation and a higher incidence of storms causing habitat loss and possibly direct mortality of individuals.

13.2 Predicting impacts on biodiversity

Reflecting on past extinction events and ecosystem changes helps to inform us about the ways we might expect future changes in the environment to affect biodiversity. Predicting these changes and their effects allows us to take action to prevent population declines and extinctions before they occur. Species will have a much better chance of survival if we understand the way they react to certain environmental changes and if we can mitigate those changes.

MONITORING

To understand how a species is affected by changing environmental conditions, we need to examine the environment and the species itself. Monitoring is the process of researching and gathering information on some sort of variable; for example, population size of an endangered species, forest cover or water quality. Monitoring is used to assess the state of a system or population and record changes over time, allowing us to infer and understand reasons for those changes. Monitoring is useful for extracting information at any range of spatial or time scales. It is possible to monitor global temperatures from many thousands of years ago up until the present day or we can monitor the daily growth and distribution of microbes on an agar plate. Sampling of biotic and abiotic components of an ecosystem allows conclusions to be made about the condition of that ecosystem.

Bioindicators

Bioindicators are useful species for monitoring changes within an ecosystem. They are species that reflect a particular environmental condition. It is often easier to examine theses indicator species than to examine the environmental condition, or set of conditions, which can involve measuring many different parameters. Measuring the dynamics of a single population of indicator species is a relatively cost-effective and reliable way to detect ecosystem change. The presence, absence or abundance of a bioindicator demonstrates a distinctive aspect of the environment. A physical change or change in the behaviour of indicator species can also reflect a change in the environment. The types of organisms that make good indicators are species that are sensitive to change and react consistently to environmental shifts. They must be representative of the other organisms in the ecosystem, by quickly reflecting stress events that cause harm to other species and the ecosystem as a whole. Bioindicator species should also be easy to observe and sample.

Lichens

Lichens are often used as bioindicators because they are very sensitive to air pollution (Figure 13.2.2). Lichens are a group of composite organisms made up of algae or photosynthetic bacteria and fungi in a symbiotic relationship. Lichens occur in many terrestrial environments and can grow on almost any substrate. This is because they do not rely on ground roots for nutrient transfer but instead obtain all nutrients from direct exposure to the atmosphere and rainwater. The do not have a cuticle and have a high surface area to volume ratio. These characteristics mean lichens are unable to avoid accumulation of atmospheric toxins making them useful in assessing air pollution, ozone depletion and metal contamination. The types of lichen/species composition found in an ecosystem indicate levels of air pollution. Presence and abundance of leafy, hairy or branching lichen growth forms indicate clean, non-polluted air. The most tolerant lichens have a crusty growth type and indicate some air pollution. Where no lichens are present, air is likely to be heavily polluted with sulfur dioxide.

Macroinvertebrates

Macroinvertebrates are invertebrates (animals without a backbone) that can be seen with the naked eye (Figure 13.2.3). Aquatic macroinvertebrates include a range of species that spend some or all of their life stages in water. Freshwater macroinvertebrates can be used to determine the water quality of lakes, streams and rivers. Different species of these macroinvertebrates have different tolerances or sensitivities to variables associated with pollution. Some species can survive in high levels of salinity, turbidity or nutrients or in poorly oxygenated waters, while other species cannot. Examining the diversity and abundance of different macroinvertebrates in a system provides an indication of the condition of the water at a particular site. Sampling and comparison across different sites can reflect contamination from anthropogenic processes, such as run-off from agriculture, occurring near waterways (Figure 13.2.4).

MODELLING CLIMATE CHANGE

Normally, when scientists want to understand the effect of a particular variable on a system they conduct an experiment where that variable is manipulated in some way. Experimental climate change research is difficult since we do not have a second Earth to experiment on and we cannot easily manipulate climate processes. In order to understand what might happen to Earth when certain climate variables change, mathematical models are used to simulate climate processes and make projections. Models are also discussed in Chapter 1.

Similar models to those used by weather forecasters are used to predict future climate trends. Weather models are run at a high spatial resolution focusing on small, specific areas and using the most recent sets of satellite and surface data as the starting point to predict weather events over the following days. Climate models are run at a much larger spatial scale and are used to predict average climate conditions over time rather than the exact weather at a given time on a certain day.

In a **global climate model** (GCM), Earth is divided up into a three-dimensional grid of cells of about 15 OOOkm2 (Figure 13.2.5). A grid cell is the smallest unit of a model and holds climate-variable information about the land, ocean and atmosphere in that cell. Climate models can be very complex and include many variables. The Global Climate Observing System (GCOS) recognises 50 Essential Climate Variables (ECVs) for global climate modelling (Table 13.2.5). These variables include air temperature, wind speed, atmospheric carbon dioxide, precipitation, sea-surface temperature and vegetation cover.

Equations are used to simulate change and calculate climate-variable data over a series of time steps. The data given after a single time step are used as the initial state for the next step. Climate models are used to generate simulations of a range of different scenarios and help us to answer questions about what might happen if humans do or do not modify our behaviour about the way we use the Earth.

SPECIES DISTRIBUTION MODELS

To determine the distribution of a species, scientists record the locations where a species is found. Data can be taken at these locations to understand the ecological niche requirements of a species. For example, records of temperature, rainfall, vegetation type, substrate type, elevation, weather events and the presence or absence of other species provide information about why a species occurs in that area. Once we understand the niche requirements of a species we can determine the conditions that allow a species to survive, grow and reproduce as well as the limits to the conditions it can tolerate. This knowledge can be used along with equations to construct species distribution models which predict a species' geographic distribution based on environmental data from locations where it is known to occur.

**Species distribution models** allow us to:

determine locations where a species may be able to live outside its existing geographical range; this can be useful for choosing reserve and restoration sites in species conservation

look for rare species or species whose distribution is poorly understood with more accuracy; models help us know where we should look

predict shifts in species distribution in response to climate change; this is useful for anticipating how climate change factors might affect a species in its current distribution.

In 1990 **species distribution modelling** was used to predict the possible further spread of the invasive cane toad in Australia. The **CLIMEX model** was used to examine the potential spread of the cane toad under conditions in 1990 and projected climate scenarios. This model is used to determine the relative climatic potential of areas for population growth and persistence of amphibians and reptiles. The model produced maps of potential habitat of the cane toad in Australia based on climate and occurrence data from Central and North America where the cane toad is native. The distribution of the cane toad in 1990 is shown in Figure 13.2.8.

Outputs from the **CLIMEX model** predicted habitat suitability under the two scenarios shown in Figure 13.2.9: the persisting average climate of 1990 (a) and a climate-warming scenario (b). In this figure, the Ecoclimatic index shows the suitability of each location for cane toad colonisation.

**Species distribution modelling** is not always perfect. So many variables account for the success of a species within a habitat and a model cannot make exact predictions. As models are used more and become more complex and advanced, accuracy is improved. Though they may never be entirely accurate they are very useful for anyone trying to anticipate habitat suitability for a species.

13.3 Managing and conserving biodiversity

People all over the world depend on different aspects of biodiversity every day. We are part of the biodiversity that currently exists on Earth and we rely on the rest of the planet's biodiversity for our own existence. We need biodiversity to grow food, we use timber for building and we use plants to make an extensive variety of medicines and other products.

Unfortunately, Earth is currently losing biodiversity faster than at any other time in human history. This will have a major impact on all remaining biodiversity including ourselves. While there is a struggle for survival between species in an individual ecosystem, all are dependent on the ecosystem functioning as a whole. Our survival depends on ensuring that species and ecosystems are not lost or damaged, so we have to improve the way we use and manage biodiversity as a resource.

THE VALUE OF BIODIVERSITY

Biodiversity holds different types of value for different reasons among different groups of people. A healthy biodiversity of organisms holds many benefits for humanity.

Direct practical value

Organisms have practical values for humans (Figure 13.3.1). The following are examples of biological resources provided by organisms:

Food-more than 600 species of finfish and shellfish are caught and sold in Australia for human consumption.

Medicines and drugs-about 25% of prescribed medicines contain natural plant compounds; many other prescribed medicines are synthetic versions of natural compounds.

Industrial materials-including wood, fibres, dyes, resins, gums, rubber and oil. Ornamental plants-more than 2500 Australian native plant species are listed as useful in horticulture.

Crop pollinators-39 of the 57 global crops benefit from natural pollinators such as birds and insects.

Future resources-bioprospecting of new food and medicinal drugs.

Engineering and design-biomimicry is a new branch of science that studies the structure and function of organisms and applies this knowledge to solve practical problems.

Plants-for the restoration of waste land after mining.

Diversity in genes, species and ecosystems-for example, diversity in genes and species can help to protect crops from devastating diseases, such as potato blight that caused a famine in Ireland in the 1840s.

Breeding stocks and population reservoirs-maintaining genetically diverse wild or captive populations to ensure the continuity of species that may have practical values.

Ecological value

Plants, animals and microorganisms have ecological value by performing distinct functions within an ecosystem. The functions of ecosystems that also directly or indirectly benefit humans are known as ecosystem services. Plants are producers of food, and during photosynthesis they release the oxygen that we breathe. Rain, snow and the flow of rivers provide drinking water. Plants also provide shelter and nest sites for animals. Animals pollinate flowers, and many also disperse seeds (Figure 13.3.2). Predators keep prey numbers in check. All organisms depend on certain bacteria and cyanobacteria for nitrogen fixation in ecosystems. Fungi and bacteria decompose dead organic matter and recycle nutrients.

Aesthetic value

Many people have an aesthetic appreciation for biodiversity and the beauty of nature. We enjoy the way it looks and makes us feel. This is why people pick colourful flowers, keep exotic fish or enjoy views of trees, mountains, lakes and wildlife (Figure 13.3.3). Biodiversity also inspires art and is often the subject of poems, songs and paintings.

Intrinsic value

Biodiversity has value regardless of its usefulness to humans. All living things are entitled to life; therefore, biodiversity has intrinsic value. The intrinsic value of all species means every living thing has value in its own right, and should be allowed to exist purely because it exists in the first place. This also means one species should not be valued more highly than another; for example, a mosquito and koala both have the same intrinsic value and right to life. These intrinsic values of biodiversity should be respected and humans have an ethical responsibility to prevent harm to other species and protect, conserve and restore ecosystems.

Social and cultural value

Biodiversity can hold value for many social and cultural reasons. In some cultures or religions different species may be considered holy or sacred and elements of biodiversity may be included in cultural identity and traditions. It may be important to some groups of people that a particular species be conserved because of a spiritual connection. In Indigenous Australian cultures totemic species have practical value as well as spiritual significance. Biodiversity also has recreational value. Bushlands are valuable to people who enjoy hiking, and maintaining healthy aquatic ecosystems is important to people who enjoy swimming and recreational fishing.

Option value

All of Earth's biodiversity has the potential to be useful and there may be many more uses of biodiversity that have not been discovered yet. Maintaining Earth's biodiversity is essential because it is a reservoir for bioprospecting new food sources and medicinal drugs. Bioprospecting is the exploration of biodiversity for new resources, such as chemical compounds and genetic material that has social or commercial value. If we allow species and ecosystems to become extinct we may lose the 'option' of making these discoveries.

Bioprospecting

Bioprospecting is the search for new plant and animal substances that have medicinal or other uses. This is not an entirely new thing to do. Indigenous Australians have been bioprospecting for a very long time. For example, the Wurundjeri people in Victoria have many uses for the Muyan or silver wattle tree (Acacia dealbata) (Figure 13.3.4).

The timber of the silver wattle is used for axe handles, and the bark is made into string for baskets and bags. Sap extracted from cuts in the wattle tree trunk can be mixed with ashes from a wood fire to make glue, or mixed with nectar from flowers to make a sweet drink. The wattle's seed pods can be cooked and eaten, or pounded to make flour. Currently there is research from all over the world into finding, developing and testing chemicals produced by a range of plants. The National Cancer Institute in the United States alone has tested 35 000 species of plants for anti-cancer properties. Testing is also being done for properties to fight other diseases such as cardiovascular disease, arthritis and AIDS. Bioprospecting relies on biodiversity conservation to ensure that all plant species can be investigated for potentially useful substances.

Well-managed bioprospecting can be advantageous because it can generate income for developing countries and can provide motivation for conservation and management. But if it is managed poorly it could result in environmental, social and economic problems. Problems can also occur if private organisations or individuals exploit knowledge ofbioresources that they do not own witl10ut sharing the profits with Indigenous peoples. This is known as 'biopiracy'.

It is estimated that over 200 companies are screening plant and animal substances for drugs. About 20% of modern pharmaceutical drugs come from the Amazon rainforest (Figure 13.3.5). Interestingly, 90% of the drugs come from tl1e Southern Hemisphere, but 90% of the people who use them live in the Northern Hemisphere. In 2010, which was the International Year of Biodiversity, the 10th Conference of Parties to the Convention on Biological Diversity adopted the Nagoya Protocol, which relates to access to bioresources and sharing of benefits. It specifically addresses the issue of bioprospecting and the rights of Indigenous people to access forest resources, intellectual property and adequate compensation. The Protocol has been signed by 92 countries, 53 of which have ratified it so far. It came into force in 2014.

Most of the world's food supply depends on about 150 plant species, but only 12 species provide 75% of the world's food. More than half of the world's food energy comes from a limited number of varieties of three 'mega-crops': rice, wheat and maize. Sorghum, millet, potatoes, sweet potatoes, soybean and sugar provide another 25%. People living in poverty (almost half the world's population) depend on plants for as much as 90% of their needs (food, fuel, medicine, shelter, transport). Approximately 1.4 billion people, mostly resource-poor farmers, use and improve their own crop seeds to maintain and enhance the genetic variation of crops. It is vital to ensure continued genetic variation in these major crops to avoid vulnerability to diseases that could affect production worldwide. Plant and food research combines traditional breeding techniques with modern genetic techniques to develop better cultivars faster. Bioprospecting combined with genetic engineering could increase the diversity of food crops available.

DECISION-MAKING

The values placed on biodiversity determine which species or ecosystems receive the most attention in terms of conservation. Because the value of biodiversity can be subjective, different people and groups value biodiversity in different ways. There is no consensus on which species or ecosystems are most important to conserve.

One way to overcome this problem is to focus conservation efforts on the most threatened species and systems.The International Union for Conservation of Nature (IUCN) has created a Red List which can help to guide conservation activities. The IUCN Red List is a global inventory of threatened species and their conservation status. When a species is assessed it is put into a category depending on the level of threat that it faces. The categories are: Extinct, Extinct in the Wild, Critically Endangered, Endangered, Near Threatened, Lower Risk, and Least Concern. Some species may also fall under Data Deficient or Not Evaluated categories. Species that fall into the Critically Endangered category are of highest conservation priority because they are more likely to soon become Extinct.

Conservation triage

Unfortunately, human, funding and time resources are limited when it comes to conservation. Without more resources being allocated to conservation some projects are prioritised while others might have to wait. A systematic approach to deciding where to invest conservation resources is known as conservation triage and can be considered in the same way as triage in a hospital. In emergency medicine doctors constantly determine the priority of patients based on the severity of their condition and the resources available. In triage situations resources are insufficient for all patients to be treated immediately and resources are allocated to maximise the number of survivors. This means those who need critical attention are seen to first but only if the care they receive is likely to have a positive outcome. In terms of conservation the world is in a state of extinction emergency. It is possible that more species require conservation attention than we have time or money to conserve. A triage approach to conservation decision-making is controversial because it would mean abandoning the aim to conserve all species and ecosystems. Resources will be focused on conservation projects that would provide the best overall conservation results but some species could be left to go Extinct. For example, priority might be given to a species whose existence has a great effect on all others in an ecosystem (e.g. a top predator), and another species might not receive any assistance and eventually become Extinct.

SPECIES CONSERVATION

There is no global consensus as to what constitutes an important species. However, species that have a high value may be chosen for conservation because they fall into one of the following categories:

•species that are under threat of extinction

•species of ecological importance

•species that have economic value to humans

•species of cultural or social importance.

There are two main species-based conservation approaches: in situ and ex situ.

In situ conservation

In situ conservation involves keeping the species in its natural environment. This conservation approach involves:

preserving the habitat through private purchase or government action

eliminating invasive or pest species from the area

managing protected areas to sustain native flora and fauna

restoring degraded ecosystems.

Ex situ conservation

Ex situ conservation involves protecting the animal or plant outside its natural habitat so threats to the species' survival can be managed or removed. This approach can involve:

captive breeding with the possibility of reintroducing the organism to its natural habitat

maintaining a captive population (e.g. in a zoo)

conserving genetic variation by storing samples of seeds, pollen, tissues or cell cultures.

Many conservationists prefer the in situ approach because it means the species ECOSYSTEM AND HABITAT CONSERVATION

Some species have to be saved from the brink of extinction by growing them in special nurseries or breeding them in captivity, but saving species is best done by protecting and preserving habitats and ensuring that populations are stable or increasing. Rainforests, woodlands, heathlands, grasslands, wetlands and coral reefs are among the most endangered habitats on Earth.

One obvious reason for preserving areas of habitats is that it ensures the protection of a diverse range of organisms. There are many species in a pond, or in the litter of a eucalypt forest, that we rarely see. Scientists believe there are also a huge number of species of insects, spiders, crustaceans, plants, protists, fungi and bacteria that are yet to be discovered. They are all part of the **food web** and they are all important in maintaining the balance of the ecosystem.

Some types of habitats are especially important to conserve because they support a large range of biodiversity. Areas that contain large numbers of species, especially endemic species (species that are only found in one area of the world) are often referred to as 'biodiversity hotspots' (Figure 13.1.1). Concentrating conservation efforts in these areas can help to maintain global biodiversity.

Conservation of habitat also protects the interrelationships between organisms. For example, the broadleaf ballart (Exocarpos latzfolius) found in northern Australia is the food plant for the larval stages of a butterfly known as the fiery jewel (Hypochrysops ignitus). The broadleaf ballart is also a parasitic tree so it relies on a host tree species for its survival (Figure 13.3.8).

Establishing protected areas

Habitat loss is considered to be the primary cause of biodiversity loss around the world. To counter this, governments establish protected areas. Effective management of protected areas is essential to deal with human settlement, illegal harvesting or poaching, unsustainable tourism and impacts of alien species.

Designing reserves

Throughout the world, governments put aside land for the preservation of animals, plants and ecosystems. Some reserves in the world are very large, covering thousands of square kilometres, such as the swamps of the Florida Everglades. But important reserves can also be quite small. For example, narrow reserves along roadsides and railway lines are important for many small animals and plants. Even home gardens can be important sources of food and nectar for birds. In Australia, Kakadu National Park, the Wet Tropics of Queensland, Lord Howe Island and the Great Barrier Reef are all listed as World Heritage Sites, which recognises their global importance (Figure 13.3.10).

Reserves that are small or fragmented may be unsuitable for animals that need large territories to search for food, build nests or find mates (Figure 13. 3 .11). Populations can become fragmented into small groups with a low genetic variation. Small reserves are also more vulnerable to destruction. For example, a small group of trees can be damaged more severely by wind storms than a more extensive forest, where the trees buffer one another. Events such as fires and floods could also destroy a small reserve or make it uninhabitable for many species, and the animals might not be able to reach other suitable habitat.

If animals cannot find all the resources they need in a small fragment of habitat, especially food, they must try to reach other suitable habitat. Some small animals such as butterflies and birds are especially vulnerable to habitat fragmentation because they will not fly across clearings, even the width of a road.

Organisms in small reserves are more likely to be exposed to edge effects because of the relatively large length of the edges compared to the area of the reserve. **Edge effects** include an increase in sunlight and temperature, exposure to wind, lower humidity and greater chance of invasion by pest plants and animals. The abiotic environment is quite different from the cool, dark, moist forest floor within the rainforest. Many organisms are not adapted to survive in the drier edge microhabitat (Figure 13.3.12).

RESTORATION OF DAMAGED ECOSYSTEMS

To accommodate the growing human population huge areas of Earth's natural environment have been changed or destroyed. Many ecosystems have been disturbed to the point where the functions and structure of ecosystems have been lost. This has occurred on land from logging forest, intensive grazing and building cities and roads, and in aquatic environments by creating dams, using boats and polluting waterways.

Ecosystem restoration aims to return an ecosystem to the state it was in before a disturbance occurred. Restoration projects may focus on reintroducing the species composition of an area, removing invasive or pest species and relieving a disturbance pressure.

Mining site restoration

Mining operations cause considerable environmental impacts of erosion, biodiversity loss and contamination of soil and water. The construction of a mining site and its infrastructure changes the land use of an area resulting in major habitat modification and destruction. Unfortunately, areas with geological formations that are prospectively useful for mining are also often areas of high conservation value supporting rare ecosystems or endemic species (Figure 13.3.14).

Mining is an important part of the Australian economy, accounting for over half of the value of national exports. Australia has significant reserves of minerals and resources including iron ore, nickel, aluminium, copper, gold, silver, uranium, opals, zinc, coal and natural gas. Land used for mining makes up less than 1 % of Australia's total land area but still has damaging effects on the environment. Australian law requires mining companies to minimise the environmental impacts of their operations. One tool that is used is the ecological restoration of mining sites once a mine is closed. Mining is a temporary activity relying on finite resources where operations at a site eventually cease. The land at a mining site is generally severely degraded in terms of ecosystem function. In the industry restoration of mining sites is referred to as 'rehabilitation', with the aim of returning land and water to productive use and recreating sustainable ecosystems that integrate with the surrounding area.

Approaches to mining reclamation depend largely on the physical and geochemical properties of the area, the type of mining operations that occurred and the ecosystem that is to be restored. Mining sites differ greatly in these aspects so each site must be considered case by case. Generally, rehabilitation of all mining sites requires the following:

Clean up of contaminants-contaminants and waste products from the mining operation are contained or removed to prevent the release of toxic chemicals or acid drainage into the environment.

Land form reconstruction-excavation during the mining process alters the initial topography of the land. Landforms must be reconstructed and stabilised to allow for appropriate drainage and to minimise erosion. Deep holes and steep slopes from waste piles are reshaped.

Soil restoration-once land forms are stabilised, soils must be restored to sustain plant growth. During the mining operation, important nutrient-rich topsoil is generally set aside and stored. This soil is then respread over the rehabilitation site and may be supplemented with appropriate fertiliser. Contours are created to assist with drainage and mulch or tree debris is spread to mimic natural conditions and prevent erosion.

Revegetation-restoration of a stable ecosystem relies on successional establishment of the plant community. Some seed reserves may still be present in the topsoil and can re-establish, sowing of seeds or planting individual plants and seedlings may be required, or recolonisation from species in surrounding areas may occur.

Fauna recolonisation-restoration of the plant community can allow animals to recolonise without human intervention, though full habitat restoration is complex and can take a long time. If components of the habitat are missing, additional resources and support for animal species may be required such as constructing nesting boxes or feeding stations and controlling pest species. Rehabilitated mining sites require constant monitoring through the restoration process and once the ecosystem has been re-established.

Restoring degraded agricultural land

Conversion of natural ecosystems and environments to agricultural land has drastic impacts on biodiversity and productivity. In Australia over 60% of the continent's land area is used for agriculture but only a very small part of that land is naturally suitable for growing crops. Agricultural practices such as irrigation and use of fertilisers have allowed crops to be grown in areas that are not usually arable. Agricultural land use also relies on clearing native vegetation to create pastures for grazing and cropping. Unfortunately, these practices are not always sustainable and result in degraded land that cannot support further agriculture or native species and functional ecosystems.

The main causes of land degradation in agricultural areas are:

Overgrazing-high grazing pressure from having too much stock in an area or high numbers of grazing pest species can damage vegetation cover. Intensive grazing over long periods of time does not allow for vegetation to recover, which can cause soil erosion and desertification in arid areas.

Removal of vegetation-clearing native plant species leaves soil susceptible to erosion from wind and water. Soil is protected by vegetation cover and when it is removed wind can blow soil away or rainfall can wash soil down slopes along with important nutrients. Removal of deep-rooted vegetation can also cause changes to water moving through the soil profile. More water is free to move, depositing dissolved salts and increasing soil salinity and making the soil unfavourable for many plant species to grow.

Over-irrigation-irrigation can cause land degradation if more water is applied than plants can use. This can occur in areas with poor drainage or leaky soils. When water in the soil is not able to be dispersed, groundwater rises to the surface. This causes soils to become waterlogged and increases soil salinity as dissolved salts rise with the water.

Unbalanced fertiliser use-using fertilisers is important for growing crops but if fertilisers are applied inappropriately plants do not use up the nutrients and leaching occurs. Use of nitrate-containing fertilisers can cause soil acidification where nitrates leach through the soil and pH decreases. Acidic soils become infertile as most plants can only grow between pH 5 and 8.

Restoration of degraded agricultural land can only occur when the pressures of agriculture are decreased or removed and steps are taken to improve soil quality so vegetation and biodiversity can re-establish.

Peniup restoration project

In 2007 a restoration project was set up on a property called Peniup in Western Australia (Figure 13 .3 .15). Peniup had previously been farmed and significant areas of the property were cleared for running sheep and crops. Remnant woodland vegetation covered 27% of the property area. The aim of the project was to protect existing bushland on the property and restore habitats and native biodiversity in the areas previously used for agriculture. The project covers a large area that would naturally include a diverse landscape and species association.

Through analysis of spatial data, soil testing and biophysical information a detailed map was created describing the different soil and landscape types across 950ha of the property. This map informed ecologists about the different plant communities that would typically be found across different areas of the cleared landscape. Nine vegetation associations were identified and seeds and seedlings of a mix of species specific to soil types and landscape positions were planted in appropriate areas. To maintain local genetics, 120 species were collected from the remnant vegetation in the surrounding area. Weed control and management were used to ensure pest species did not colonise the area.

Monitoring of 42 permanent sampling sites over the landscape of the project has shown patchy establishment of plant density and growth. This is expected in a diverse landscape and important for providing a variety of habitat types. It was also found that many seedlings did not survive the first hot, dry summer. In some areas new seedlings emerged a few years later and replaced those that had died.

Reducing pollution to protect species

Pollution created by humans can poison all forms of life on land and in water, and it is contributing to climate change. Transport, industry, construction, mining, power generation, volcanoes and bushfires all contribute polluting substances to the environment. These substances can directly cause death or disease in organisms, or alter the chemistry of the environment so that it is unfit for some organisms (Figure 13.3.16).

We can protect vulnerable species by reducing pollution. While industry has a major role to play in this, you can try to minimise your impact by:

walking or cycling instead of travelling by car

driving a car that uses less fuel

turning off electrical appliances when they are not being used choosing energy-efficient appliances

using environmentally friendly cleaning products, reducing the amount of phosphates and nitrates going into waterways

reducing the amount of rubbish you generate by recycling and composting, and repairing rather than replacing goods

limiting your use of chemical fertilisers, pesticides and herbicides and using environmentally friendly alternatives.

Combating climate change

Climate change is threatening many species that cannot evolve fast enough to cope with the increase in temperature. Extreme weather events may also pose a threat. Climate change will affect the abundance and distribution of organisms and will influence the crops we grow. If we can halt the increase in carbon dioxide levels we may be able to prevent the devastating effects of climate change on species, habitats and ecosystems. International agreements such as the Kyoto Protocol aim to reduce the emissions of carbon dioxide worldwide, but not all countries are signatories to such agreements.

Regulation to counter overexploitation

Biodiversity is exploited mainly for food (meat, vegetables, fruits) and construction materials (e.g. timber), but also for industrial products, the pet trade, fashion and medicines. Most species can tolerate some exploitation without it affecting the survival of its populations. However, overexploitation can push species towards extinction, upset the normal relationships between species in an ecosystem, and harm other species. For example, hunting a particular bird for food could reduce its numbers to an unsustainable level, reduce the food available to predators or cause predators to switch to another prey, and affect the survival of plants that depend on the bird for pollination or seed dispersal.

Overexploitation usually occurs because there is an economic benefit for those who are exploiting the species. The economic benefit creates an incentive for those people to continue exploiting the species or ecosystem despite the long-term costs of loss of biodiversity and ecosystem services.

Overexploitation can often be managed by making strict laws and enforcing them, but this is often difficult to do in practice. For example, poachers in Southern Africa will risk their lives to kill a rhinoceros for its valuable horn. The horn is sold as an ornament or more commonly ground up for use in traditional Chinese medicines, even though there is no scientific evidence that shows the therapeutic benefit of rhino horn keratin. It is illegal to trade rhinoceros horns; however, they fetch a very high price on the black market. Sometimes rangers at private game reserves remove rhinoceros horns from live rhinoceroses as a deterrent to poaching. Even so, rhinoceroses are still killed by poachers for the remaining horn stub or as an act of vengeance.

**Textbook 3: Dynamics of Life Vol. 1 (Ecology)**

Organisms and Their Environment

Where in the world am I?

Finding the Main Idea When you start to study a new topic in depth, it is sometimes difficult to see the big ideas and make the connections that you need to make. Learning about ecology is more than memorizing the vocabulary. Of all the subjects that you might study in biology, ecology makes you stand back to get the big picture—of how individual organisms interact with each other and with their environment, because it is your environment, too.

Organize Information As you study this chapter, use the red and blue titles throughout the chapter to organize information or outline the main ideas of ecology.

Sharing the World

How much do you know about the environment and the organisms that share your life? As cities and suburbs expand, and humans move into territories previously occupied by fields and wildlife, animals such as raccoons and deer are tipping over garbage cans and meandering through backyards. Every day, you also interact with houseflies, mosquitoes, billions of dust mites, and other organisms that you cannot even see. What affects their environment also affects you. Understanding what affects the environment is important because it is where you live.

Studying nature

People have always shown an interest in their natural surroundings. You may know someone who can identify every animal, plant, and rock they see. Other people keep records of rainfall and temperature. The study of plants and animals, including where they grow and live, what they eat, or what eats them, is called natural history. Collecting data like these is similar to taking the pulse of an individual. These data reflect the status or health of the world in which you live.

What is ecology?

The branch of biology that is developed from natural history is called ecology. Ecology is the study of interactions that take place between organisms and their environment.

Ecological research

Scientific research includes using descriptive and quantitative methods. Ecological research combines information and techniques from many scientific fields, including mathematics, chemistry, physics, geology, and other branches of biology. Most ecologists use both descriptive and quantitative research. They obtain descriptive information by observing organisms. They obtain quantitative data by making measurements and carrying out experiments in the field and in the laboratory. Ecologists may ask what a coyote eats, how day length influences plants or migrating birds, or why tiny shrimp help rid ocean fishes of parasites.

The Biosphere

On Earth, living things are found in the air, on land, and in both fresh- and salt water. The biosphere is the portion of Earth that supports living things. It extends from high in the atmosphere to the bottom of the oceans. This may seem extensive, but if you could shrink Earth to the size of an apple, the biosphere would be thinner than the apple’s peel.

Although it is thin, the biosphere supports a diverse group of organisms in a wide range of climates. The climate, soils, plants, and animals in one part of the world can be very different from those same factors in other parts of the world. Living things are affected by both the physical or nonliving environment and by other living things.

The nonliving environment: Abiotic factors

The nonliving parts of an organism’s environment are the **abiotic factors**. Examples of abiotic factors include air currents, temperature, moisture, light, and soil. Ecology includes the study of features of the environment that are not living because these features are part of an organism’s life. For example, a complete study of the ecology of moles would include an examination of the types of soil in which these animals dig their tunnels. Similarly, a thorough investigation of the life cycle of trout would need to include whether they need to lay their eggs on rocky or sandy stream bottoms.

**Abiotic factors** have obvious effects on living things and often determine which species survive in a particular environment. For example, extended lack of rainfall in the grassland shown in Figure 2.1 can cause drought. What changes in a grassland might result from a drought? Grasses would grow more slowly. They might produce fewer seeds, and the animals that depend on seeds for food would find it harder to survive. Examine other ways that abiotic factors affect living things in the MiniLab and Problem-Solving Lab shown on these pages.

The living environment: Biotic factors

In addition to abiotic factors, a key consideration of ecology is that living organisms affect other living organisms. All the living organisms that inhabit an environment are called **biotic factors**.

Think about a goldfish in a bowl. Now consider its relationships with other organisms. Does the fish live alone or with other fishes? Are there live plants in the bowl? The fish may depend on other living things for food, or it may be food for other life. The goldfish needs members of the same species to reproduce. To meet its needs, the goldfish may compete with organisms of the same or different species that share the bowl.

All organisms depend on others directly or indirectly for food, shelter, reproduction, or protection. If you study an individual organism, such as a male white-tailed deer, you might find out what food it prefers, how often it eats, and how far it roams to search for food. However, studying a single individual won’t tell you all there is to know about white-tailed deer. In fact, white tails are social animals. They live in small groups or a herd in which there is a strong social structure built around visual and vocal communications that keep the herd safe.

So you can see that the study of a single individual provides only part of the story of its life.

Levels of Organization

Ecologists study individual organisms, interactions among organisms of the same species, and interactions among organisms of different species, as well as the effects of abiotic factors on interacting species.

To help them understand the interactions of the biotic and abiotic parts of the world, ecologists have organized the living world into levels—the organism by itself, populations, communities, and ecosystems.

Interactions within populations

A population is a group of organisms, all of the same species, which interbreed and live in the same area at the same time.

How the organisms in a population share the resources of their environment may determine how far apart the organisms live and how large the populations become. Members of the same population may compete with each other for food, water, mates, or other resources. Competition increases when resources are in short supply.

Some species have adaptations that reduce competition within a population. An example is the life cycle of a frog. The juvenile stage of the frog, called the tadpole, looks very different from the adult and has different food requirements. As you can see in Figure 2.2, many species of insects, including butterflies and moths, also produce juveniles that differ from the adult in body form and food requirements.

Interactions within communities

No species lives independently. Just as a population is made up of individuals, several different populations make up a biological community. A biological community is made up of interacting populations in a certain area at a certain time. An example of a community is shown in Figure 2.3.

A change in one population in a community may cause changes in the other populations. Some of these changes can be minor, such as when a small increase in the number of individuals of one population causes a small decrease in the size of another population. For example, if the population of mouse-eating hawks increases slightly, the population of mice will, as a result, decrease slightly. Other changes might be more extreme, as when the size of one population grows so large it begins affecting the food supply for another species in the community. Figure 2.4 on the next page is a visual summary of the ecological levels of organization.

Biotic and abiotic factors

In a healthy forest community, interacting populations might include birds eating insects, squirrels eating nuts from trees, mushrooms growing from decaying leaves or bark, and raccoons fishing in a stream. In addition to how individuals in a population interact with each other, ecologists also study interactions between separate populations and their physical surroundings. An ecosystem is made up of interacting populations in a biological community and the community’s abiotic factors. Because animals and plants in an area can change, and because abiotic factors can change, ecosystems are subject to change.

There are two major kinds of ecosystems—terrestrial ecosystems and aquatic ecosystems. Terrestrial ecosystems are those located on land. Examples include forests, meadows, and rotting logs. Aquatic ecosystems occur in both fresh- and saltwater forms. Freshwater ecosystems include ponds, lakes, and streams. Saltwater ecosystems, also called marine ecosystems, make up approximately 70 percent of Earth’s surface. Figure 2.5 shows a freshwater and a marine ecosystem. Examples of ecosystems are given in Table 2.1.

Organisms in Ecosystems

A prairie dog living in a grassland makes its home in burrows that it digs underground. Some species of birds make their homes in the trees of a beech-maple forest. In these areas, they find food, avoid enemies, and reproduce. A habitat is the place where an organism lives out its life. A lawn, the bottom of a stream, and beech-maple forests are examples of habitats. Other habitats could be a wetland, a specific species of tree, a city lot or park, a pond, or a specific area in the ocean. Habitats can change, and even disappear. Habitats can change due to both natural and human causes. Examples of habitat changes are presented in Biology and Society at the end of this chapter.

Niche

Although several species may share a habitat, the food, shelter, and other essential resources of that habitat are often used in different ways. For example, if you turn over a log like the one shown in Figure 2.6, you will find a community of millipedes, centipedes, insects, slugs, and earthworms. In addition, there are billions of fungi and bacteria at work breaking down the log, the leaves, and wastes produced by these animals. At first, it looks like members of this community are competing for the same food because they all live in the same habitat. But close inspection reveals that each population feeds in different ways, on different materials, and at different times. These differences lead to reduced competition. Each species is unique in satisfying all its needs. Each species occupies a niche.

A niche is all strategies and adaptations a species uses in its environment—how it meets its specific needs for food and shelter, how and where it survives, and where it reproduces. A species’ niche, therefore, includes all its interactions with the biotic and abiotic parts of its habitat.

It is an advantage for a species to occupy a niche different from those of other species in the same habitat, although a species’ niche may change during its life cycle. It is thought that two species can’t exist for long in the same community if their niches are the same. In time, one of the species will gain control of the resources both need. The other will become extinct in that area, move elsewhere, or, over time, become adapted in the way its species uses that particular habitat’s resources.

Symbiosis

People may have once assumed that all organisms living in the same environment are in a continuous battle for survival. However, studies have shown that most species survive because of the relationships they have with other species.

These relationships help maintain survival in many species. The relationship in which there is a close and permanent association between organisms of different species is called symbiosis. Symbiosis means living together. Three kinds of symbiosis are recognized: mutualism, commensalism, and parasitism.

Mutualism

Sometimes, two species of organisms benefit from living in close association. A symbiotic relationship in which both species benefit is called mutualism. Ants and acacia trees living

Commensalism

Commensalism is a symbiotic relationship in which one species benefits and the other species is neither harmed nor benefited.

Commensal relationships occur among animals and in plant species, too. Spanish moss is a kind of flowering plant that drapes itself on the branches of trees, as shown in Figure 2.8. Orchids, ferns, mosses, and other plants sometimes grow on the branches of larger plants. The larger plants are not harmed, but the smaller plants benefit from the habitat.

Parasitism

Some interactions are harmful to one species, yet beneficial to another. Have you ever owned a dog or cat that was attacked by ticks or fleas? Ticks, like the one shown in Figure 2.9, are examples of parasites. A symbiotic relationship in which a member of one species derives benefit at the expense of another species (the host) is called parasitism. Parasites have evolved in such a way that they harm, but usually do not kill the host species. If the host were to die, the parasite also would die unless it can quickly find another host. Some parasites, such as certain bacteria, tapeworms, and roundworms, live in or on other organisms.

Brown-headed cowbirds, in a behavior called brood parasitism, lay their eggs in the nests of songbirds, often at the expense of the host bird’s eggs. The cowbird is about the size of an American Robin. It is not uncommon to see a much smaller bird species, such as a chipping sparrow, in the act of feeding a much larger, but younger, cowbird. Brown-headed cowbirds are known to parasitize about 200 other species of birds in North America.

A predator is a type of consumer. Predators seek out and eat other organisms. Predation is found in all ecosystems and includes organisms that eat plants and animals. Predators may be animals such as lions and insect-eating birds. The animals that predators eat are called prey. Predator-prey relationships such as the one between cats and mice involve a fight for survival. Use the BioLab at the end of this chapter to examine a predator-prey relationship.

Nutrition and Energy Flow

How Organisms Obtain Energy

The mosquito in Figure 2.10 takes in a blood meal. This is one means by which the female mosquito obtains nutrients. Mosquitoes also feed on nectar as a source of energy. An important characteristic of a species’ niche is how it obtains energy. Ecologists trace the flow of energy through communities to discover nutritional relationships between organisms.

The producers: Autotrophs

The ultimate source of the energy for life is the sun. Plants use the sun’s energy to manufacture food in a process called photosynthesis. An organism that uses light energy or energy stored in chemical compounds to make energy-rich compounds is a producer, or autotroph. Grass and trees in Figure 2.11 are autotrophs. Although plants are the most familiar autotrophs, some unicellular organisms such as green algae, also make their own nutrients. Other organisms in the biosphere depend on autotrophs for nutrients and energy. These dependent organisms are called consumers.

The consumers: Heterotrophs

A deer nibbles the leaves of a clover plant; a bison eats grass; an owl swallows a mouse. The deer, bison, and owl are consumers, incapable of producing their own food. They obtain nutrients by eating other organisms. An organism that cannot make its own food and feeds on other organisms is called a heterotroph. Heterotrophs include organisms that feed only on autotrophs, organisms that feed only on other heterotrophs, and organisms that feed on both autotrophs and heterotrophs.

Some heterotrophs, such as grazing, seed-eating, and algae-eating animals, feed directly on autotrophs. Heterotrophs display a variety of feeding relationships.

1. A heterotroph that feeds only on plants is an herbivore. Herbivores include rabbits, grasshoppers, beavers, squirrels, bees, elephants, fruit-eating bats, and some humans.

2. Some heterotrophs eat other heterotrophs. Animals such as lions that kill and eat only other animals are carnivores.

3. Some heterotrophs, called scavengers, do not kill for food. Instead, scavengers eat animals that have already died. Scavengers, such as black vultures, feed on dead animals and garbage and play a beneficial role in the ecosystem. Imagine for a moment what the environment would be like if there were no vultures to devour animals killed on the African plains, no buzzards to clean up dead animals along roads, and no ants and beetles to remove dead insects and small animals from sidewalks and basements.

Humans are an example of a third type of heterotroph. Most people eat a variety of foods that include both animal and plant materials. They are omnivores. Raccoons, opossums, and bears are other examples of omnivores. Some organisms, such as bacteria and fungi, are decomposers. They break down and release nutrients from dead organisms. Decomposers break down the complex compounds of dead and decaying plants and animals into simpler molecules that can be more easily absorbed. Some protozoans, many bacteria, and most fungi carry out this essential process of nutrient recycling.

Flow of Matter and Energy in Ecosystems

When you eat food, such as an apple, you consume matter. Matter, in the form of carbon, nitrogen, and other elements, flows through the levels of an ecosystem from producers to consumers. In doing so, the matter is cycled. The apple also contains energy from sunlight that was trapped in the plant during the process of photosynthesis. As you cycle the matter of the apple, some trapped energy is transferred from one level to the next. At each level, a certain amount of energy is also transferred to the environment as heat.

How do matter and energy flow through ecosystems? You have already learned that feeding relationships and symbiotic relationships describe the ways in which organisms interact. Ecologists’ study these interactions and make models to trace how matter and energy flow through ecosystems. The simplest models are called food chains.

Food chains: Pathways for matter and energy

A **food chain** is a simple model that scientists use to show how matter and energy move through an ecosystem. In a **food chain**, nutrients and energy move from autotrophs to heterotrophs and, eventually, to decomposers.

The forest community pictured in Figure 2.12 illustrates examples of food chains. A food chain is drawn using arrows to indicate the direction in which energy is transferred from one organism to the next. One simple food chain on this page would be shown as:

berries mice black bear

Most **food chains** consist of two, three, or four transfers. The amount of energy remaining in the final transfer is only a portion of what was available at the first transfer. A portion of the energy is given off as heat at each transfer.

Trophic levels represent links in the chain

Each organism in a food chain represents a feeding step, or trophic level, in the passage of energy and materials. A first order heterotroph is an organism that feeds on plants, such as a grasshopper. A second order heterotroph is an organism that feeds on a first order heterotroph. An example of this would be a bird that feeds on a grasshopper. Examine how energy flows through trophic levels in the Problem-Solving Lab shown here.

A **food chain** represents only one possible route for the transfer of matter and energy through an ecosystem. Many other routes may exist. As Figure 2.12 showed, many different species can occupy each trophic level in a forest ecosystem. In addition, many different kinds of organisms eat a variety of foods, so a single species may feed at several trophic levels. For example, the North American black bear may eat the mouse, but it also eats berries. The hawk may feed on the fish, or a mouse.

Food webs

A simple food chain such as grass mouse hawk is easy to study, but it does not indicate the complex relationships that exist for organisms that feed on more than one species. Ecologists interested in energy flow in an ecosystem may set up experiments with as many organisms in the community as they can. The model they create, called a **food web**, shows all the possible feeding relationships at each trophic level in a community. A **food web** is a more realistic model than a food chain because most organisms depend on more than one other species for food. The food web of the desert ecosystem shown in Figure 2.13 represents a network of interconnected **food chains** formed by herbivores and carnivores.

Energy and trophic levels: Ecological pyramids

Ecologists use food chains and food webs to model the distribution of matter and energy within an ecosystem. They also use another kind of model called an **ecological pyramid**. An **ecological pyramid** can show how energy flows through an ecosystem.

The base of the ecological pyramid on the next page represents the autotrophs, or first trophic level. Higher trophic levels are layered on top of one another. Examine each of the three types of ecological pyramids on the following pages.

The **pyramid of energy** illustrates that the amount of available energy decreases at each succeeding trophic level. The total energy transfer from one trophic level to the next is only about ten percent because organisms fail to capture and eat all the food energy available at the trophic level below them. When an organism consumes food, it uses some of the energy in the food for its metabolism—some for building body tissues, and some is given off as heat. When that organism is eaten, the energy that was used to build body tissue is again available as energy to be used by the organism that consumed it. According to the law of conservation of energy, energy is neither lost nor gained. Some of the energy transferred at each successive trophic level enters the environment as heat, but the total amount of energy remains the same.

A **pyramid of numbers** shows that population sizes decrease at each higher trophic level. This is not always true. For example, one tree can be food for thousands of insects. In this case, the pyramid would be inverted.

Biomass is the total weight of living matter at each trophic level. A **pyramid of biomass** represents the total dry weight of living material available at each trophic level.

Cycles in Nature

Food chains, food webs, and ecological pyramids are all models that show how energy moves in only one direction through the trophic levels of an ecosystem. Some of the energy also is transferred to the environment as heat generated by the body processes of organisms. Sunlight is the primary source of all this energy, and is always being replenished by the sun.

Matter, in the form of nutrients, also moves through, or is part of, all organisms at each trophic level. But matter is cycled and is not replenished like the energy from sunlight. There is a finite amount of matter. The atoms of carbon, nitrogen, and other elements that make up the bodies of organisms alive today are the same atoms that have been on Earth since life began. Matter is constantly recycled. It is never lost. Life on Earth depends on water. Even before there was life on Earth, water cycled through stages. Have you ever left a glass of water out and a few days later observed there was less water in the glass? This is the result of evaporation. Just as the water evaporated from the glass, water evaporates from lakes and oceans and becomes water vapor in the air, as shown in Figure 2.17.

Where do the drops of water that form on a cold can of soda come from? The water vapor in the air condenses on the surface of the can because the can is colder than the surrounding air. What takes place in the glass of water and on the cold soda can is similar to the global water cycle on the previous page. Water vapor also condenses on dust in the air and forms clouds. Further condensation makes small drops that build in size until they fall from the clouds as precipitation in the form of rain, ice, or snow. The water falls to Earth and accumulates in oceans and lakes where evaporation continues. Plants and animals need water to live. Natural processes constantly recycle water throughout the environment. Plants pull water from the ground and lose water from their leaves through the process of transpiration. This activity puts water vapor into the air. Animals breathe out water vapor in every breath. When they perspire or urinate, water also is returned to the environment.

The **carbon cycle**

All life on Earth is based on carbon molecules. Atoms of carbon form the framework for proteins, carbohydrates, fats, and other important molecules. More than any other element, carbon is the molecule of life. It is an important part of all living organisms.

The carbon cycle described in Figure 2.18 on the next page starts with an autotroph. During photosynthesis, energy from the sun is used by autotrophic organisms to convert carbon dioxide gas into energy-rich carbon molecules that many organisms use for food and a source of energy. Autotrophs use these molecules for growth and energy. Heterotrophs, which feed either directly or indirectly on the autotrophs, use these carbon molecules for growth and energy. When the autotrophs and heterotrophs use the carbon-containing molecules and release energy, carbon dioxide is released and returned to the atmosphere. As the term implies, carbon cycles again and again through this system. How rapidly it cycles depends upon whether it is in soil, leaves, roots, tied up in a forest, in oil or coal, in animal fossils, or in the world’s vast calcium carbonate reserves. Learn how to detect the presence of carbon dioxide in the MiniLab shown here.

The Carbon Cycle

The nitrogen cycle

If you add nitrogen fertilizer to a lawn, houseplants, or garden, you may see that they become greener, bushier, and taller. Even though the air is 78 percent nitrogen, plants seem to do better when they receive nitrogen fertilizer. This is because most plants cannot use the nitrogen in the air. They use nitrogen in the soil that has been converted into more usable forms.

As Figure 2.19 shows, certain bacteria convert the nitrogen from air into these more usable forms. Chemical fertilizers also give plants nitrogen in a form they can use.

Plants use the nitrogen to make important molecules such as proteins. Herbivores eat plants and convert nitrogen-containing plant proteins into nitrogen-containing animal proteins. After you eat your food, you convert the protein components in food into human proteins in the form of muscle cells, blood cells, enzymes, and urine. Urine, an animal waste, contains nitrogen compounds. When an animal urinates, nitrogen returns to the water or soil. When organisms die and decay, nitrogen returns to the soil and eventually to the atmosphere. Plants reuse this nitrogen. Soil bacteria also act on these molecules and put nitrogen back into the air.

The **phosphorus cycle**

Materials other than water, carbon, and nitrogen cycle through ecosystems. Substances such as sulfur, calcium, and phosphorus, as well as others, must also cycle through an ecosystem. One essential element, phosphorus, cycles in two ways.

All organisms require phosphorus for growth and development. Plants obtain phosphorus from the soil. Animals obtain phosphorus by eating plants. When these animals die, they decompose and the phosphorus is returned to the soil to be used again. This is the **short-term phosphorus cycle** in Figure 2.20. Phosphorus also has a **long-term cycle**, where phosphates washed into water become incorporated into rock as insoluble compounds. Millions of years later, as the environment changes, the rock containing phosphorus is exposed. As the rock erodes, the phosphorus again becomes part of the local ecological system.

Communities and Biomes

Life in a Community

Look closely at a square meter of healthy, green lawn and you will discover that, hidden in the grass population, there are also populations of weeds, beetles and other insects, earthworms, and grubs. There may also be twigs, seeds, and maybe a bird feather, along with soil and moisture. Not so visible are the populations of bacteria and fungi that outnumber all the other organisms. This community is alive, and each population or factor in it contributes something important to the life of the lawn.

How do plants and animals survive where they live? What is there about a climate where green lawns live and die that is different from a climate where polar bears thrive? Various combinations of abiotic and biotic factors interact in different places around the world. The result is that conditions in one part of the world are suitable for supporting certain forms of life, but not others.

Limiting factors

Factors that affect an organism’s ability to survive in its environment, such as the availability of water and food, predators, and temperature, are called **limiting factors**. A **limiting factor** is any biotic or abiotic factor that restricts the existence, numbers, reproduction, or distribution of organisms. The timberline in Figure 3.1 on the next page shows that limiting factors affect the plant life of an ecosystem. High elevations, low temperatures, strong winds, and soil that is too thin to support the growth of anything more than small, shallow-rooted plants, mosses, ferns, and lichens are all limiting factors. Other common limiting factors are listed in Table 3.1.

Factors that limit one population in a community may also have an indirect effect on another population. For example, a lack of water could restrict the growth of grass in a grassland, reducing the number of seeds produced. The population of mice dependent on the seeds for food will also be reduced. What about hawks that feed on mice? Their numbers also may be reduced as a result of a decrease in their food supply.

Ranges of tolerance

Corn plants need two to three months of warm, sunny weather and a regular supply of water to produce a good yield. Corn grown in the shade or during a long dry period may survive, but probably won’t produce a marketable crop. The ability of an organism to withstand fluctuations in biotic and abiotic environmental factors is known as tolerance. Figure 3.2 illustrates that a population will survive according to its tolerance for environmental extremes.

Succession: Changes over Time

If grass were no longer cut on a lawn, what would it look like in one year? Five years? In 90 years? From experience, ecologists can predict the changes that will take place.

1. The grass gets taller; weeds start to grow. The area resembles a meadow.

2. Later, bushes grow, trees appear and different animals enter the area to live.

3. The bushes and trees change the environment; less light reaches the ground. The grass slowly disappears.

4. Thirty years later, the area is a forest. Ecologists refer to the orderly, natural changes and species replacements that take place in the communities of an ecosystem as succession.

Succession occurs in stages. At each stage, different species of plants and animals may be present. The conditions at each stage are suitable for some organisms but not for others. As succession progresses, new organisms move in. Others may die out or move out. Succession often is difficult to observe because it can take decades or even centuries for one community to succeed another. There are two types of succession— primary and secondary.

Primary succession

The colonization of barren land by communities of organisms is called primary succession. Primary succession takes place on land where there are no living organisms. For example, lava flowing from a volcano destroys everything in its path. When it cools, new, but barren, land has formed. The first species to take hold in an area like this are called pioneer species. An example of a pioneer species is a lichen, which is a combination of small organisms. Examine lichens in the MiniLab on this page.

Pioneer species eventually die. Decaying lichens, along with bits of sediment in cracks and crevices of rock, make up the first stage of soil development. In time, new soil makes it possible for small weedy plants, small ferns, fungi, and insects to become established. As these organisms die, more soil builds. Seeds, carried by water, wind or animals, move into these expanding patches of soil and begin to grow.

After some time, primary succession slows down and the community becomes fairly stable, or reaches equilibrium. A stable, mature community that undergoes little or no change in species is a climax community. A climax community may last for hundreds of years.

Stability or equilibrium does not mean that change stops. Change is dynamic as the numbers of species may rise and fall in an area. Over time, however, the changes are balanced, so long as nothing drastic—such as fire— happens to the area. Succession from bare rock to a climax community is illustrated in Figure 3.3.

Secondary succession

What happens when a natural disaster such as a forest fire destroys a community? What happens when a field isn’t replanted or when a building is demolished in a city and nothing is built on the site? Then secondary succession begins. Secondary succession is the sequence of changes that takes place after an existing community is severely disrupted in some way.

During secondary succession, as in primary succession, the community of organisms inhabiting an area gradually changes.

Secondary succession, however, occurs in areas that previously contained life, and on land that still contains soil. Therefore, the species involved in secondary succession are different from those in primary succession. Because soil already exists, secondary succession may take less time than primary succession to reach a climax community. Learn more about the differences between primary and secondary succession in the Problem-Solving Lab.

An example of secondary succession

In 1988, forest fires burned from June to September in Yellowstone National Park. Hundreds of thousands of hectares of trees, shrubs, and grasses were burned. The fire has given biologists an opportunity to study secondary succession in a community. Ecologically, the fire represented change, not total destruction. Annual wildflowers, like those in Figure 3.4, were among the first plants to grow back. Previously, the shade of the trees was a limiting factor for wildflower growth. Within three years of the fire, perennial wild-flowers, grasses, ferns, and thousands of lodgepole-pine seedlings began to replace the annuals. Once the pine seedlings grow above the shade cast by the grasses and perennials, the trees will grow more quickly.

What is a biome?

Ecosystems that reach similar climax communities can be grouped into a broader category called a biome. A biome is a large group of ecosystems that share the same type of climax community. There are terrestrial biomes and aquatic biomes, each with organisms adapted to the conditions characteristic of the biome. Biomes located on land are called terrestrial biomes. Organisms such as the cardon cactus shown here, populate terrestrial desert biomes. Oceans, lakes, streams, ponds, or other bodies of water are aquatic biomes.

Aquatic Biomes

As a human who lives on land, you may think of Earth as a terrestrial planet. But one look at a globe, a world map, or a photograph of Earth taken from space tells you there is an aquatic world, too.

Approximately 75 percent of Earth’s surface is covered with water. Most of that water is salty. Oceans, seas, and even some inland lakes contain salt water. Freshwater is confined to rivers, streams, ponds, and most lakes. Saltwater and freshwater environments have important differences. As a result, aquatic biomes are separated into marine biomes and freshwater biomes.

Marine biomes

Different parts of the ocean differ in abiotic factors (salinity, depth, availability of light, and temperature) and biotic factors found there. The oceans contain a large amount of biomass, or living material. Most of this biomass is made up of extremely small, often microscopic, organisms that humans usually don’t see but that large marine animals, such as baleen whales, depend upon. One of the ways ecologists study marine biomes is to make separate observations in shallow, sunlit zones (photic zones) and deeper, unlighted zones (aphotic zones). The portion of the marine biome that is shallow enough for sunlight to penetrate is called the photic zone. Shallow marine environments exist along the coastlines of most landmasses on Earth. These coastal ecosystems include bays, rocky shores, sandy beaches, mudflats, and estuaries. Coral reefs also are located in shallow water in warmer parts of the ocean. All are part of the photic zone. Deeper water that never receives sunlight makes up the aphotic zone. The aphotic zone includes the deepest, least explored areas of the ocean.

Estuaries—Mixed waters

If you were to follow the course of a river, you would, in most cases, reach a sea or ocean. Wherever rivers join oceans, freshwater mixes with salt water. In many such places, an estuary forms. An estuary is a coastal body of water, partially surrounded by land, in which freshwater and salt water mix.

The salinity, or amount of salt, in an estuary ranges between that of seawater and that of freshwater, and depends on how much fresh-water the river brings into the estuary. Salinity in the estuary also changes with the tide and so a wide range of organisms can live in estuaries. Estuaries, as illustrated in Figure 3.5, may contain salt marsh ecosystems, which are dominated by salt-tolerant smooth cordgrass, salt marsh hay, or eelgrasses. These grasses can grow so thick that their stems and roots form a tangled mat that traps food material and provides a “nursery” habitat for small developing snails, crabs, and shrimp. These organisms feed on decaying, suspended materials. In turn, these small organisms attract a wide range of predators, including birds.

With the help of bacteria, decay of dead organisms proceeds quickly in an estuary and nutrients are released. Nutrients are recycled through the **food web** and as a result, microorganisms help maintain equilibrium.

The effects of tides

Daily, the gravitational pull of the sun and moon causes the rise and fall of ocean tides. The portion of the shoreline that lies between the high and low tide lines is called the intertidal zone. The size of this zone depends upon the slope of the land and the difference between the high and low tides. Intertidal ecosystems have high levels of sunlight, nutrients, and oxygen.

Tide pools, pools of water left when the water is at low tide, can isolate the organisms that live in the intertidal zone until the next high tide. Therefore, these areas can vary in nutrient and oxygen levels from one time of day to another. Compare and contrast oxygen content between tide pools and the ocean in the Problem-Solving Lab on this page.

Intertidal zones differ in rockiness and wave action. Figure 3.6 shows a rocky intertidal zone. If the shore is rocky, waves constantly threaten to wash organisms into deeper water. Many intertidal animals, such as snails and sea stars, have adaptations that act by suction to hold onto wave- beaten rocks. Other animals, such as barnacles, secrete a strong glue that helps them remain anchored. If the shore is sandy, wave action keeps the bottom in constant motion.

Clams, worms, snails, crabs, and other organisms that live along sandy shores survive by burrowing into the sand.

In the light

As you move into deeper water, the ocean bottom is less affected by waves or tides. Thousands of organisms live in this shallow-water region. Nutrients washed from the land by rainfall and runoff contribute to the abundant life and high productivity of this region of the photic zone.

The photic zone of the marine biome also includes the vast expanse of open ocean that covers most of Earth’s surface. Most of the organisms that live in the marine biome are plankton. Plankton are small organisms that drift and float in the waters of the photic zone. They include autotrophs, diatoms, eggs, and the juvenile stages of many marine animals. Plankton are important because they form the base of all **aquatic food chains**. Not all organisms that eat plankton are small. Baleen whales and whale sharks, some of the largest organisms that have ever lived, consume vast amounts of plankton. Examine plankton in the MiniLab shown here.

In the dark

Imagine a darkness blacker than night and pressure so intense it exerts hundreds of pounds of weight on every square centimeter of your body’s surface. These are the conditions deep in the ocean where light does not penetrate. Much of the ocean is more than a kilometer deep. The animals living there are far below the photic zone where plankton abound. Many of them still depend on plankton for food, either directly, or indirectly, by eating organisms that feed on plankton.

Freshwater biomes

Have you ever gone swimming or boating in a lake or pond? If so, you may have noticed different kinds of plants, such as cattails, growing around the shoreline and into the water. The shallow water in which these plants grow serves as home for tadpoles, aquatic insects, turtles that bask on rocks and fallen tree trunks, and worms and crayfishes that burrow into the muddy bottom. Insect larvae, whirligig beetles, dragonflies, and fishes such as minnows, bluegill, and carp also live here and are each part of the local food chain.

Although the spring and summer sun heats the surface of a lake like the one in Figure 3.7, the water a few feet below the surface remains cold. Cold water is more dense than warm water. If you were to dive all the way to the bottom of the lake, you would discover layers of increasingly colder water as you descended. These temperature variations within a lake are an abiotic factor that limits the kinds of organisms that can survive in deep lakes.

Another abiotic factor that limits life in deep lakes is light. Not enough sunlight penetrates to the bottom to support photosynthesis, so few aquatic plants or algae grow. As a result, population density is lower in deeper waters. As dead organisms drift to the bottom, bacteria break them down and recycle the nutrients. Decay takes place more slowly at the bottom of a deep lake.

Other aquatic biomes

Other places where land and water meet are called wetlands, but there are several different kinds of wetlands. Swamps have trees. Marshes do not, but both usually have water flowing through them. Marshes are found inland and in coastal regions. Both are highly productive and are the source of food for many migratory birds and other animals. Other wetland areas, called bogs, get their water supply from rain. Water does not flow through bogs.

Terrestrial Biomes

If you are setting off on an expedition beginning at the north pole and traveling south to the equator, what kinds of environmental changes do you expect to experience and why? The weather gets warmer, and you see a change in the sizes, numbers, and kinds of plants that cover the ground. At the polar cap, temperatures are always freezing and no plants exist. A little farther south, where temperatures sometimes rise above freezing but the soil never thaws completely, you would be attacked by hordes of mosquitoes and black flies. You’d see soggy ground with lichens and low- growing cushion plants.

As you continue on your journey, temperatures rise a little and you enter forests of coniferous trees. Then there are deciduous forests, with moderate rainfall and temperatures. Farther on are grasslands and deserts, with high summertime temperatures and very little rain. Finally, as you approach the equator, you find yourself surrounded by the lush growth of a tropical forest, where it rains almost every day.

Latitude and climate

What caused the changes that you experienced as you moved south from the north pole to the equator? As you traveled, you were changing latitude. Latitude describes your position in degrees north and south of the equator. Look at Figure 3.8. At different latitudes, the sun strikes Earth differently. As a result, the climate—wind, cloud cover, temperature, humidity and precipitation in that area—are different. Latitude and climate are abiotic factors that affect what plants and animals will survive in a given area. The graph in Figure 3.9 shows how two abiotic factors—temperature and precipitation—influence the kind of climax community that develops. Small differences in temperature or precipitation can create different biomes. Look at the distribution of the six most common terrestrial biomes on pages 1062 and 1063 in the Focus On.

Life on the tundra

As you begin traveling south from the north pole, you reach the first of two biomes that circle the north pole. This first area is the tundra, a treeless land with long summer days and short periods of winter sunlight.

Because of its latitude, temperatures in the tundra never rise above freezing for long, and only the top-most layer of soil thaws during the summer. Underneath this top layer is a layer of permanently frozen ground called permafrost.

In most areas of the tundra, the topsoil is so thin that it can support only shallow-rooted grasses and other small plants. The soil is lacking in nutrients. The process of decay is slow due to the cold temperatures and, as a result, nutrients are not recycled quickly. Lack of nutrients limits the types of organisms the tundra can support.

Summer days on the tundra may be long, but the growing season is short. Because all food chains depend on the producers of the community, the short growing season limits the type of plants found in this biome shown in Figure 3.10, to grasses, dwarf shrubs, and cushion plants. These organisms live a long time and are resistant to drought and cold.

Hordes of mosquitoes and black-flies are some of the most common tundra insects during the short summer. The tundra also is home to a variety of small mammals, including ratlike lemmings, weasels, arctic foxes, snowshoe hares, and even birds such as snowy owls and hawks. Musk oxen, caribou, and reindeer are among the few large animals that migrate into the area and graze during the summer months. Figure 3.11 shows two common tundra animals.

Life on the taiga

Just south of the tundra lies another biome that circles the north pole. The taiga also is called the boreal or northern coniferous forest. The taiga, shown in Figure 3.12, forms an almost continuous belt of coniferous trees worldwide. Common trees are larch, fir, hemlock, and spruce trees.

How can you tell when you leave the tundra and enter the taiga? The line between these two biomes can be indistinct, and one can blend into the other. For example, if the soil in the taiga is waterlogged, a peat swamp habitat develops that looks much like tundra. Because of their latitude, taiga communities usually are somewhat warmer and wetter usually are somewhat warmer and wetter than tundra. However, the prevailing climatic conditions are still harsh, with long, severe winters and short, mild summers.

In the taiga, which stretches across much of Canada, Northern Europe, and Asia, permafrost is usually absent. The topsoil, which develops slowly from decaying coniferous needles, is acidic and poor in minerals. When fire or logging disrupt the taiga community, the first trees to recolonize the land may be birch, aspen, or other deciduous species because the new soil conditions are within their ranges of tolerance. The abundance of trees in the taiga provides more food and shelter for animals than the tundra. More large species of animals are found in the taiga as compared with the tundra. Figure 3.13 shows some animals of the taiga. Others include weasels, red squirrels, voles, elk, red deer, and moose, along with a variety of migratory birds.

Life in the desert

The driest biome is the desert biome. A desert is an arid region with sparse to almost nonexistent plant life. Deserts usually get less than 25 cm of precipitation annually. One desert, the Atacama Desert in Chile, is the world’s driest place. This desert receives an annual rainfall of less than 0.004 inches because it is in the rain shadow of the Andes.

With rainfall as the major **limiting factor**, vegetation in deserts varies greatly. Areas that receive more rainfall produce a shrub community that may include drought-resistant trees such as mesquite. Less rainfall results in scattered plant life and produces an environment with large areas of bare ground. The driest deserts are drifting sand dunes. Plants such as the creosote bush shown in Figure 3.14 have various adaptations for living in arid areas. Many desert plants are annuals that germinate from seed and grow to maturity quickly after sporadic rainfall. Cacti have leaves reduced to spines, photosynthetic stems, and thick waxy coatings—all adaptations that conserve water. The leaves of some desert plants curl up, or even drop off altogether, thus reducing water loss during extremely dry spells. Spines, thorns, or poisons also are adaptations thought to discourage herbivores.

Many desert mammals are small herbivores that remain under cover during the heat of the day, emerging at night to forage on plants. The kangaroo rat is a desert herbivore that does not have to drink water. These rodents obtain the water they need from the water content in their food. Coyotes, hawks, owls, and roadrunners are carnivores that feed on the snakes, lizards, and small mammals of the desert. Scorpions are an example of a desert carnivore that uses venom to capture prey. Two of the many reptiles that make the desert their home are shown in Figure 3.15.

Life in the grassland

If an area receives between 25 and 75 cm of precipitation annually, a grassland usually forms. Grasslands are large communities covered with rich soil, grasses, and similar plants. Grasslands, such as the ones shown in Figure 3.16, occur principally in climates that experience a dry season, where insufficient water exists to support forests.

Grasslands contain few trees per hectare, though larger numbers of trees usually are found near streams and other water sources. This biome has a higher biological diversity than deserts, often having more than 50 species per hectare.

The soils of grasslands have considerable humus content because many grasses die off each winter, leaving byproducts to decay and build up in the soil. Grass roots survive through the winter, enlarging every year to form a continuous underground mat called sod.

Some grasslands are ideal for growing cereal grains such as oats, rye, and wheat. Each of these is a different species of grass; therefore, grasslands are known as the breadbaskets of the world. Many other plant species live in this environment, including drought-resistant and late-summer flowering species of wildflowers, such as blazing stars and sunflowers.

At certain times of the year, many grasslands are populated by herds of grazing animals. Bison, a species of mammal shown in Figure 3.16A, once ranged over the American prairie, but are now found only in small pockets of rangeland. Other important prairie animals include jack rabbits, deer, elk, and prairie dogs. Prairie dogs are seed-eating rodents that build underground “towns” that are known to stretch mile after mile under the grassland. Foxes and ferrets prey on prairie dogs. Many species of insects, birds, and reptiles, also make their homes in grasslands.

The term prairie is used in Australia, Canada, and the United States. Similar communities are called steppes in Russia, savannas in Africa, and pampas in Argentina. Grasslands in the United States can be found in the central and south-western states.

Life in the temperate forest

When precipitation ranges from about 70 to 150 cm annually in the temperate zone, temperate deciduous forests, like the one in Figure 3.17, develop. Temperate or deciduous forests are dominated by broad-leaved hardwood trees that lose their foliage annually. Examples of these trees include maple, oak, birch, elm, and ash. European settlers cleared vast tracts of temperate forest for farmland and lumber. Since then, secondary succession has restored much of the original forest, especially in the eastern United States.

The soil of temperate forests usually consists of a top layer that is rich in humus and a deeper layer of clay. If mineral nutrients released by the decay of the humus are not immediately absorbed by the roots of the living trees, they may be washed into the clay and lost from the food web for many years.

The animals that live in the temperate deciduous forest, as shown in Figure 3.18, include squirrels, mice, rabbits, deer, and bears. Many birds, such as bluejays, live in the forest all year long, whereas other birds migrate seasonally.

Life in rain forests

Rain forests are home to more species of organisms than any other biome on Earth. There are two types of rain forests in the world—the temperate rain forest and the more widely known tropical rain forest shown in Figure 3.19. Both are identified by extensive amounts of moisture supplied by rainfall or by coastal clouds and fog. Temperate rain forests are found on the Olympic Peninsula in Washington State and in other places throughout the world, such as South America, New Zealand, and Australia. The huge number of species in rain forests has made their protection an important objective.

As their name implies, tropical rain forests have warm temperatures, wet weather, and lush plant growth. These forests are warm because they are near the equator. The average temperature is about 25°C. They are moist because wind patterns drop a lot of precipitation on them. Rain forests receive at least 200 cm of rain annually; some rain forests receive 600 cm.

Why do tropical rain forests contain so many species? The following hypotheses have been proposed by ecologists:

1. Due to their location near the equator, tropical rain forests were not covered with ice during the last ice age. Thus, the communities of species had more time to evolve and greater biodiversity exists.

2. Unlike the temperate forests— where deciduous trees drop their leaves in autumn—the warm weather near the equator gives tropical rain forest plants year-round growing conditions. This creates a greater food supply in tropical rain forests, which can support larger numbers of organisms.

3. Tropical rain forests provide a multitude of habitats and niches for diverse organisms.

One reason for the large number of niches in rain forests is vertical layering. How are these layers, or stories, arranged? Find out by studying Figure 3.20 on the next page. From top to bottom, the three major stories are the canopy, understory, and ground layers. The layers often blend together, but their differences allow many organisms to find a niche.

Most of the nutrients in a tropical rain forest are tied up in the living material. There are very few nutrients held in the soil and most are quickly recycled through **complex food webs**. The hot humid climate enables ants, termites, fungi, bacteria, and other decomposers to break down dead plants and animals rapidly. Plants must quickly absorb these nutrients before they are carried away from the soil by rain.

Tropical rain forest habitats support a wide variety of plants and animals. This makes them the most species-rich places on Earth.

Biomass, the total weight of organisms living in the area, is high. This is because sunlight, moisture, and nutrients are available in abundance for plants to convert light energy to chemical energy. This energy is used by the plants and passed to consumers, such as those pictured in Figure 3.21.

Some rain forest plants are important sources of medicinal products and hardwood trees and have provided a source of income for people. Agricultural land is not common in rain forests. The soil there does not convert to cropland easily. In temperate deciduous forests, topsoil has taken hundreds or thousands of years to develop as leave decayed and their nutrients became part of the soil. In contrast, soils in rain forests do not have substantial amounts of organic matter because leaf matter, which contains nutrients, disappears so quickly. Without organic matter, once rain forest soil is exposed and farmed, it becomes hard, almost brick-like, and nutrient-poor in a matter of a few years. Research is underway to find out how people can manage these lands so that they will be able to obtain the food and products they need.

Population Biology

Population Dynamics

Principles of Population Growth

A population is a group of organisms, all of the same species, that live in a specific area. There are populations of spruce trees, populations of maple trees, of bluebirds, dandelions, fruit flies, and house cats.

Every organism you can think of is a member of a population. A healthy population will grow and die at a relatively steady rate unless it runs out of water, food, or space, or is attacked in some way by disease or predators.

Scientists study changes in populations in a variety of ways. One method involves introducing organisms into a controlled environment with abundant resources; then watching how the organisms react. That is what is happening in Figure 4.1. Bacterial cells are placed in a dish of sterile, nutrient-rich solution and population growth is observed over a period of time. Through studies such as these, scientists have been able to identify some trends in the growth of bacterial cells.

Information on bacterial cell growth might be helpful in fighting disease. Studies of populations of larger organisms, such as an elk population in a national park, require methods such as the use of radio monitors. Use the MiniLab on this page to learn one method of measuring growth in a fruit fly population.

How fast do populations grow?

The growth of populations is unlike the growth of pay you get from a job. Suppose your job pays $5 per hour. You know if you work for two hours, you will be paid $10; if you work for four hours, you will be paid $20; if you work for eight hours, you will be paid $40; and so on. If you were to plot money earned against your time in hours, the graph would show a steady, straight-line (linear) increase.

Populations of organisms, however, do not experience linear growth. Rather, the graph of a growing population starts out slowly, then begins to resemble a J-shaped curve, as illustrated in a population of houseflies in Figure 4.2. The initial increase in the number of organisms is slow because the number of reproducing individuals is small. Soon, however, the rate of population growth increases because the total number of individuals that are able to reproduce has increased.

Is growth unlimited?

A J-shaped **growth curve** illustrates exponential population growth. Exponential growth means that as a population gets larger, it also grows at a faster rate. Exponential growth results in unchecked growth.

What can limit growth?

Can a population of organisms grow indefinitely? Through observation and population experiments, scientists have found that population growth does have limits. Eventually, **limiting factors**, such as availability of food, disease, predators, or lack 0 of space, will cause population growth to slow. Under these pressures, the population may stabilize in an **S-shaped growth curve**, which you can see in Figure 4.3.

Carrying capacity

The number of organisms of one species that an environment can support indefinitely is its carrying capacity. When a population is developing in an environment with resources, there are more births than deaths and the population increases until the carrying capacity is reached or passed. When a population overshoots the carrying capacity, then limiting factors may come into effect. Deaths begin to exceed births and the population falls below carrying capacity. Thus, the number of organisms in a population is sometimes more than the environment can support and sometimes less than the environment can support. Figure 4.4 on the next page shows a population growth line that moves above and below the carrying capacity. Many different types of organisms can show such growth patterns in nature.

Reproduction Patterns

In nature, animal and plant populations change in size. For example, mosquitoes are more numerous at certain times of the year than others. Why don’t populations reach carrying capacity and remain stable? To answer this question, population biologists study the factor that determines population growth—an organism’s reproductive pattern, also called its life-history pattern.

A variety of population growth patterns are possible in nature. Two extremes of these patterns are demonstrated by the population growth rates of mosquitoes and elephants. Mosquitoes exhibit a rapid life-history pattern. Elephants, like many other large organisms, exhibit characteristics of the slow life-history pattern. Mosquitoes reproduce very rapidly and produce many offspring in a short period of time, whereas elephants have a slow rate of reproduction and produce relatively few young over their lifetime.

Rapid life-history patterns

Rapid life-history patterns are common among organisms from changeable or unpredictable environments. Rapid life-history organisms have a small body size, mature rapidly, reproduce early, and have a short life span. Populations of rapid life-history organisms increase rapidly, then decline when environmental conditions such as temperature suddenly change and become unsuitable for life. The small population that survives will reproduce exponentially when conditions are again favorable. The Problem-Solving Lab on this page explores growth in bacteria, an organism with a rapid life-history pattern.

Slow life-history patterns

Large species that live in more stable environments usually have slow life-history patterns. Elephants, bears, whales, humans, and plants, such as trees, are long lived. The pronghorn antelope shown in Figure 4.5, are slow life-history organisms. Slow life-history organisms reproduce and mature slowly, and are long-lived. They maintain population sizes at or near carrying capacity.

Density factors and population growth

Recall that limiting factors are biotic or abiotic factors that determine whether or not an organism can live in a particular environment. Limited food supply, space, chemicals produced by plants themselves, extreme temperatures, and even storms affect populations.

How organisms are dispersed can also be important. Figure 4.6 shows three patterns of dispersal: random, clumped, and uniform.

Ecologists have identified two kinds of limiting factors that are related to dispersal: density-dependent and density-independent factors. Population density describes the number of individuals in a given area.

**Density-dependent factors** include disease, competition, predators, parasites, and food. These factors have an increasing effect as the population increases. Disease, for example, can spread more quickly in a population with members that live close together. In crops such as corn or soybeans in which large numbers of the same plant are grown together, a disease can spread rapidly throughout the whole crop. In less dense populations, fewer individuals may be affected. Disease is also a factor in human populations. The presence of HIV/AIDS in many of the world’s populations is considered by some scientists to be a limiting factor in the growth of those populations.

**Density-independent factors** can affect populations, regardless of their density. Most density-independent factors are abiotic factors, such as volcanic eruptions, temperature, storms, floods, drought, chemical pesticides, and major habitat disruption, such as that shown in Figure 4.7. Although all populations can be affected by these factors, the most vulnerable appear to be small organisms with large populations, such as insects. No matter how many earthworms live in a field, they will drown if it floods. It doesn’t matter if there are many or few mosquitoes— a severe winter will kill the adults of most species.

Organism Interactions Limit Population Size

Population sizes are limited not only by abiotic factors, but also are controlled by various interactions among organisms that share a community.

Predation affects population size

A barn owl kills and eats a mouse. A swarm of locusts eats and destroys acres of lettuce on a farm. When the brown tree snake was introduced in Guam, an island in the South Pacific, there were no native predators for the snake. Consequently, it freely preyed on the native birds of the island. These examples demonstrate how predation can affect population sizes in both minor and major ways. When a predator consumes prey on a large enough scale (as in the case of the brown tree snake), it can have a drastic effect on the size of the prey population. For this reason, predation can be a limiting factor on population size.

Populations of predators and their prey are known to experience cycles or changes in their numbers over periods of time. Under controlled conditions, such as in a laboratory, predator-prey relationships often show a predictable cycle of population increases and decreases over time. In nature, these cycles have also been observed. One classic example of this has been demonstrated in Figure 4.8 on the next page, which shows a graph of 90 years of data about the populations of the Canadian lynx and the snowshoe hare. A member of the cat family, the lynx stalks, attacks, and eats the snowshoe hare as a primary source of food.

The data in Figure 4.8 show the lynx and hare populations appear to rise and fall fairly closely in a 10-year cycle. When the hare population increases, there is more food for the lynx population, and the lynx population increases. When the lynx population rises, predation increases, and the hare population then declines. With fewer hares available for food, the lynx population then declines. Then, with fewer predators, the hare population increases, and the cycle continues. This example shows how predator populations can affect the size of the prey populations. At the same time, prey populations affect the size of the predator populations. As the snowshoe hare’s food supply of grasses and herbs dwindles during the fall and winter months, the hare population decreases. Because there are now fewer hares to hunt, the lynx population also decreases. With the return of spring, the hare’s food supply and its population recover. This leads to more hares, allowing the lynx population to increase as well.

Usually, in prey populations, the young, old, or injured members are caught. Predation increases the chance that resources will be available for the remaining individuals in a prey population.

Competition within a population

The hare and the lynx belong to different populations. What happens when organisms within the same population compete for resources? When population numbers are low, resources can build up and become plentiful. Then, as these resources are used, the population increases in size and competition for resources such as food, water, and territory again increases significantly. Competition is a **density-dependent factor**. When only a few individuals compete for resources, no problem arises. When a population increases to the point at which demand for resources exceeds the supply, the population size decreases.

The effects of crowding and stress

When populations of certain organisms become crowded, individuals may exhibit symptoms of stress. The factors that create stress are not well understood, but the effects have been documented from experiments and observations of populations of several organisms including fish, deer, rabbits, and rats as shown in Figure 4.9.

As populations increase in size in environments that cannot support increased numbers, individual animals can exhibit a variety of stress symptoms. These include aggression, decrease in parental care, decreased fertility, and decreased resistance to disease. All of these symptoms can have negative effects on a population. They become **limiting factors** for growth and keep populations below carrying capacity.

Human Population

World Population

In the United States, a census is taken every ten years. Among other things, this information provides a picture of how many people there are in the United States, their economic condition, and where they live. Worldwide, the United Nations Population Division tracks similar information on all the countries of the world. One of the most useful pieces of data is the rate at which each country’s population is growing or declining. These figures are the basis for demography, the study of human population size, density and distribution, movement, and its birth and death rates.

What is the history of population growth for humans? Figure 4.10 summarizes how world human population has grown since 1800. The graph indicates that until the 1800s, human population growth remained fairly slow. Since the 1930s, world population has grown rapidly, reaching 6 billion in 1999. In 2002, the human population was growing at a rate of 1.3 million people per year.

Human population growth

What factors affect growth of human population? In Section 1 of this chapter, bacteria and housefly populations were shown to continue to grow so long as they had sufficient resources. Human population growth is different because humans can consciously change their environment. During the past century, humans have eradicated diseases such as smallpox. They have developed methods for producing more food. Infant mortality rate has decreased and technological developments have improved the delivery of clean water. When these factors are accounted for, people live longer and are able to produce offspring that live long enough to produce offspring, hence, a population grows.

Calculating growth rate

There are a number of factors that determine population growth rate. These are births, deaths, immigration and emigration. Birthrate is the number of live births per 1000 population in a given year. Death rate is the number of deaths per 1000 population in a given year. Movement of individuals into a population is immigration. Movement out of a population is emigration. You can calculate a country’s population growth rate with a formula that takes these four factors into account:

(Birthrate + Immigration rate) — (Death rate + Emigration rate) = Population Growth Rate (PGR)

For convenience, and because immigration and emigration rates are not always accurate, this formula is often stated as:

Birthrate — Death rate = Population Growth Rate (PGR)

If the birthrate of a population equals its death rate, then the population growth rate is zero. If the rate is zero, that doesn’t mean that the population isn’t changing. Rather, it means that new individuals enter the population (by birth and immigration) at the same rate that individuals are leaving (by death and emigration) the population. The population is changing, but it is stable. If the PGR is above zero, more new individuals are entering the population than are leaving, so the population is growing.

A PGR can also be less than zero. In 2002, the population growth rate for Europe was negative (—0.1 percent) as fewer individuals are entering the population than are leaving.

The effect of a positive growth rate

If the world population growth rate in the year 1995 were 1.7 percent and had dropped to 1.3 percent in 2001, the population growth rate would have become lower, but world population would have continued to grow, just at a slower rate. In other words, unless the growth rate becomes negative, the population continues to grow, but just not as rapidly as it did before.

Doubling time

Another quantitative factor that demographers look at is the doubling time of a population. Doubling time is the time needed for a population to double in size. The time it takes for a population to double varies depending on the current population and growth rate. A slow or negative growth rate means that it will take a country’s population a long time to double in size, if ever. A rapid growth rate indicates that a country’s population will double in a shorter time. A country that has a slow doubling time is sometimes categorized as a developed country. One with a rapid doubling time may be referred to as a developing country. Doubling time can be calculated for the world, a country, or even a smaller region, such as a city. Learn how to calculate doubling time in the MiniLab on this page.

Age structure

Have you ever filled out a survey? Often, one of the questions is about age. Are you between the ages of 10 and 14? 15 and 19? 20 and 24? The survey is trying to pinpoint where you are in the age structure of the population. Age structure refers to the proportions of the population that are in different age levels. Based on information from population counts, an age structure graph has been constructed for every country in the world. Look at the age structure graphs in Figure 4.11. An age structure graph can tell you approximately how many males and females there are in a population, and how many people there are at each age level. Rapidly growing countries have age structures with a wide base because a large percentage of the population is made up of children and teenagers. If the percentage of people in each age category is fairly equal, the population is stable.

Ecology and growth

The needs of populations differ greatly throughout the world. Some countries are concerned about providing the most basic needs for their growing population. Other, more stable growth populations are concerned about maintaining the healthy conditions that they already have.

What do populations need? Think about the resources that humans depend upon every day. Some of these resources might be uncontaminated water for drinking and agriculture, adequate sewage facilities, and the ability to provide food for a growing population.

Sometimes, a population grows more rapidly than the available resources can handle. Resources that are needed for life, such as food and water, become scarce or contaminated. The amount of waste produced by a population becomes difficult to dispose of properly. These conditions can lead to stress on current resources and contribute to the spread of diseases that affect the stability of human populations both now and to come.

Biological Diversity and Conservation

Vanishing Species

Biological Diversity

A rain forest has a greater amount of biological diversity, or biodiversity, than a cornfield. Biodiversity refers to the variety of species in a specific area. The simplest and most common measure of biodiversity is the number of different species that live in a certain area. For example, a hectare of farmland, like the one in Figure 5.1B, is dominated by one species of plant—corn. In contrast, one hectare of a rain forest may contain 400 species of plants. The cornfield also may contain hundreds of species of insects and several species of birds, but the rain forest may have thousands of species of insects and hundreds of species of birds.

Where is biodiversity found?

Areas around the world differ in biodiversity.

A hectare of tropical rain forest in Amazonian Peru may have 300 tree species, while one hectare of temperate deciduous forest in the United States is more likely to have only 30 tree species. Therefore, the tropical rain forest has more biodiversity. Biodiversity increases as you move toward the equator. Tropical regions contain two-thirds of all land species on Earth.

The richest environments for biodiversity all seem to be warm places: tropical rain forests, coral reefs, and large tropical lakes.

Studying biodiversity

How do ecologists perform experiments related to biodiversity? The study of islands has led to an understanding of factors that influence biodiversity. In the 1960s, an investigation was devised for testing the development of biodiversity on islands. The scientists thought that using a miniaturized situation such as very small islands would help them see clearly what changes take place when organisms move into or out of a defined area. To do this, the scientists selected some small islands of mangrove trees off the coast of Florida like those in Figure 5.2. They counted the number of insect and spider species that were on each island, and then removed all the existing species from the islands except for the trees. Then they observed the following as organisms moved back onto the islands.

1. Insects and spiders returned first.

2. The farther away the island was from the source of the new species (the mainland), the longer it took for the island to be recolonized.

3. Eventually, the islands had about the same number of species that they had originally, but the makeup of the community was now different from the original community.

The scientists also saw that the larger the island, the more habitats and species it seemed to have, implying that the number of species depends on the number of habitats.

Research like this is not simple to do. Today you can read about projects in rain forests that require scientists to work 150 meters up in the canopy while they collect species that live only at that level. Other researchers catalogue the organisms that live in coral reefs, and others attach radio collars to deer. Still others work in laboratories comparing the DNA of members of isolated populations to see how or if these populations might be changing.

Importance of Biodiversity

Compare a parking lot covered with asphalt to your favorite place in nature, perhaps your backyard, a wooded area, or a local lake. You might go to an area like this to relax or to think. Artists get inspiration from these areas for songs, paintings, photographs, and literature. Looking at one of Art Wolfe’s photographs in the Connection to Art on page 128 can help you appreciate the beauty biodiversity gives our world. Beyond beauty, why is biodiversity important?

Importance to nature

Living things are interdependent. Animals could not exist without green plants. Many flowering plants could not exist without animals to pollinate them. Plants are dependent on decomposers that break down dead or decaying material into nutrients they can absorb. In a rain forest, a tree grows from nutrients released by decomposers. A sloth eats the leaves of the tree. Moss grows on the back of the sloth. Thus, living things can be niches for other living things.

Populations are adapted to live together in communities. Although ecologists have studied many complex relationships among organisms, many relationships are yet to be discovered. Scientists do know that if a species is lost from an ecosystem, the loss may have consequences for other living things in the area. An organism suffers when a plant or animal it feeds upon is removed permanently from a food chain or food web. A population may soon exceed the area’s carrying capacity if its predators are removed. If the symbiotic relationships among organisms are broken due to the loss of one species, then the remaining species will also be affected.

Biodiversity brings stability

Biodiversity can bring stability to an ecosystem. A pest could easily destroy all the corn in a farmer’s field, but it would be far more difficult for a single type of insect or disease to destroy all individuals of a plant species in a rain forest. There, instead of being clumped together, the plants exist scattered in many parts of the rain forest, making it more difficult for the disease organism to spread. In summary, ecosystems are stable if their biodiversity is maintained. A change in species can destabilize them.

Importance to people

Humans depend on other organisms for their needs. Oxygen, on which animals depend, is supplied, and carbon dioxide is removed from the air by diverse species of plants and algae living in a variety of ecosystems throughout the world. Beef, chicken, tuna, shrimp, and pork are a few of the meats and seafood humans eat. Think of all the plant products that people eat, from almonds to zucchini. Yet only a few species of plants and animals supply the major portion of the food eaten by the human population. Biodiversity could help breeders produce additional food crops. For example, through crossbreeding with a wild plant, a food crop might be made pest-resistant or drought-tolerant. People also rely on the living world for raw materials used in clothes, furniture, and buildings.

Another important reason for maintaining biodiversity is that it can be used to improve people’s health. Living things supply the world pharmacy. Although drug companies manufacture synthetic drugs, active compounds in these drugs are usually first isolated from living things, such as those in Figure 5.3. The antibiotic penicillin came from the mold Penicillium. The antimalarial drug quinine came from the bark of the cinchona tree. Even the importance of soil microorganisms should not be overlooked. The drug cyclosporine, which prevents rejection of transplanted organs, was discovered in a soil fungus in 1971. Preserving biodiversity ensures there will be a supply of living things, some of which may provide future drugs. Will a cure for cancer or HIV be found in the leaves of an obscure rain forest plant?

Loss of Biodiversity

Have you ever seen a flock of passenger pigeons? How about a blue pike, or a dusky seaside sparrow? Unless you have seen a photograph or a specimen in a museum, your answer will be “No” to each of these questions. These animals are extinct. Extinction is the disappearance of a species when the last of its members dies. Extinction is a natural process and Earth has experienced several mass extinctions during its history. There is also a certain level of natural extinction, called background extinction, that goes on. Scientists estimate that background extinction accounts for the loss of one species per year per million species. However, the current rate of extinction exceeds that by many times. Scientists hypothesize that this rise is due in part to the needs of the expanding human population, habitat loss, and land exploitation. Is there evidence of a link between land use and species extinction? Look at one scientist’s analysis in the Problem-Solving Lab on this page.

A species is considered to be an endangered species when its numbers become so low that extinction is possible. Figure 5.4 shows species listed as endangered in the United States.

When the population of a species is likely to become endangered, it is said to be a threatened species. African elephants, for example, are listed as a threatened species. In 1979, the estimated wild elephant population was about 1.3 million. Twenty years later, the population was estimated to be 70000. In 1998, a survey published by the African Elephant Database estimated a minimum number of elephants at about 300 000. The United States Fish and Wildlife Service maintains a listing of threatened and endangered species for the United States and the world. Figure 5.5 shows the type of information available from the Fish and Wildlife Service on its Threatened and Endangered Species System database.

Threats to Biodiversity

Complex interactions among species make each ecosystem unique. The species there are usually well adapted to their habitats. Changes to habitats can therefore threaten organisms with extinction. What are some of the activities that can bring this about?

Habitat loss

One of the biggest reasons for decline in biodiversity is habitat loss. In the 1970s and 1980s, in the Amazonian rain forest, thousands of hectares of land were cleared in an effort to create farmland and to supply firewood. Much of this land lost its usefulness for agriculture after only a few years because rain forest soil by itself has little or no useful nutrient supply. Clearing the land erased habitats that will not be reestablished easily. Without these habitats, certain plants and animals become vulnerable to extinction.

Other areas affected by habitat loss are coral reefs. Coral reefs, like the one in Figure 5.6, are thought to be similar to tropical rain forests in biodiversity richness. The structure of coral provides habitats for varieties of fish, anemones, sponges, and other marine organisms. Disease and changes in water temperature can damage or kill coral. As a result, habitats are lost and the organisms that depend on the coral also are affected.

Habitat fragmentation

Habitat fragmentation is the separation of wilderness areas from other wilderness areas. Habitat fragmentation has been found to contribute to:

•increased extinction of local species.

•disruption of ecological processes.

•new opportunities for invasions by introduced or exotic species.

•increased risk of fire.

•changes in local climate. Fragmented areas are similar to islands. The smaller the fragment, the less biodiversity the area can support. This is because, as species migrate from an area that has become unsuitable for some reason, other species that depend on the migrating individuals lose their life support. As a result, overall species diversity declines.

Geographic isolation can lead to genetic isolation. When an individual organism’s habitat becomes too small, its population becomes isolated from other populations of its species. The organism doesn’t have the chance to breed with members of its species in other populations.

Habitat fragmentation, as shown in Figure 5.7, presents problems for organisms that need large areas to gather food or find mates. Large predators may not be able to obtain enough food if restricted to too small an area. Habitat fragmentation also makes it difficult for species to reestablish themselves in an area. Imagine a small fragment of forest where a species of salamander lives. A fast-burning fire started by lightning destroys trees and the salamanders living there. In a non-fragmented forest, as the area recovers, new salamanders would eventually move into the area. However, if the burned forest was isolated from another forest where other salamanders live, no route would exist for these salamanders to reestablish populations in the burned area. In the next section of this chapter, read about corridors that connect one piece of fragmented land to another.

Edge Effect

The edge of a habitat or ecosystem is where one habitat or ecosystem meets another. This can be where a forest meets a field, where water meets land, or where a road cuts through a field or wooded area. The different conditions along the boundaries of an ecosystem are called **edge effects**. An edge may have two different sets of abiotic factors. Edges tend to have greater biodiversity because different habitats with different species are brought together. When an edge changes, animals from one area might migrate from the area or move to the new edge, thereby bringing species from different ecosystems in contact with one another. If a piece of land is cleared or divided by a road, new edges are created. This action may expose animals attracted to the edge to more predators than they were previously.

What happens at the edge of a habitat may affect what goes on in the interior of the area. In a developed area, there may be more housecats, and therefore, birds that nested undisturbed in the area before may be preyed upon.

Habitat degradation

Another threat to biodiversity is habitat degradation, the damage to a habitat by pollution. Three types of pollution are air, water, and land pollution. Air pollution can cause breathing problems and irritate membranes in the eyes and nose. Pollutants enter the atmosphere in many ways— including volcanic eruptions and forest fires. Burning fossil fuels is also a major source of air pollutants such as sulfur dioxide.

Acid precipitation—rain, snow, sleet, and fog with low pH values— has been linked to the deterioration of some forests and lakes. Sulfur dioxide from coal-burning factories and nitrogen oxides from automobile exhaust combine with water vapor in the air to form acidic droplets of water vapor. When these droplets fall from the sky, the moisture leaches calcium, potassium, and other nutrients from the soil. This loss of nutrients can lead to the death of trees. Acid precipitation also damages plant tissues and interferes with plant growth. Worldwide, many trees such as those shown in Figure 5.8 are dying and acid rain and fog are thought to be the cause. Acid precipitation also is linked to degrading lake ecosystems. When acid rain falls into a lake, or enters as runoff from streams, the pH of the lake water falls.

Ultraviolet waves emitted by the Sun also can cause damage to living organisms. Ozone, a compound consisting of three oxygen atoms, is found mainly in a region of Earth’s atmosphere between about 15 km and 35 km altitude. The ozone in this region—known as the ozone layer— absorbs some of the ultraviolet waves striking the atmosphere, reducing the ultraviolet radiation reaching Earth’s surface. Over some parts of Antarctica, the amount of ozone overhead is reduced by as much as 60 percent during the Antarctic spring. Ozone amounts then increase during the summer. This seasonal ozone reduction is known as the Antarctic ozone hole, and is caused by the presence in the atmosphere of human-produced chemicals such as chlorofluorocarbons (CFCs). Smaller seasonal reductions also have been observed over the Arctic and there is a small downward trend in global ozone concentrations. However, the causes of this ozone loss and its biological consequences are still uncertain.

Water pollution

Water pollution degrades aquatic habitats in streams, rivers, lakes, and oceans. A variety of pollutants can affect aquatic life. Excess fertilizers and animal wastes, as shown in Figure 5.9, are often carried by rain into streams and lakes. The sudden availability of nutrients causes algal blooms, the excessive growth of algae. As the algae die, they sink and decay, removing needed oxygen from the water. Silt from eroded soils can also enter water and clog the gills of fishes. Detergents, heavy metals, and industrial chemicals in runoff can cause death in aquatic organisms. Abandoned drift nets in oceans have been known to entangle and kill dolphins, whales, and other sea life.

Land pollution

How much garbage does your family produce every day? Trash, or solid waste, is made up of the cans, bottles, paper, plastic, metals, dirt, and spoiled food that people throw away everyday. The average American produces about 1.8 kg of solid waste daily. That’s a total of about 657 kg of waste per person per year. At what rate does it decompose? Although some of it might decompose quickly, most trash becomes part of the billions of tons of solid waste that are buried in landfills. Strict controls on the design, construction, and placement of landfills are meant to reduce contamination of groundwater supplies.

The use of pesticides and other chemicals can also lead to habitat degradation. For many years, DDT was used liberally to control insects and to kill mosquito larvae. Birds that fed on DDT-treated crops, or insects, fish, and other small animals exposed to DDT, were observed to have high levels of DDT in their bodies. The DDT was passed on in food chains to the predators that ate these animals.

Because of the DDT in their bodies, some species of predators, such as the bald eagle and the peregrine falcon, were found to lay eggs with very thin shells that cracked easily, killing the chicks and leading to sharp population declines. These observations contributed to the ban on DDT in the United States in 1972.

Exotic species

People sometimes introduce a new species into an ecosystem, either intentionally or unintentionally. These species can cause problems for the native species. When people brought goats to Santa Catalina Island, located off the coast of California, 48 native species of plants soon disappeared from the local environment. Building the Erie canal in the nineteenth century made it possible for the sea lamprey to swim into the Great Lakes. The sea lamprey, which resembles an eel, clamps onto a fish’s body and, using its sharp teeth and tongue, sucks fluids out of the fish. The lamprey has totally eliminated certain fish species from some of the Great Lakes. Exotic species, such as the goat and the lamprey, are not native to a particular area. Some other examples of exotic species are shown in Figure 5.10. When exotic species are introduced, these species can grow at an exponential rate due to the fact that they are not immediately as vulnerable to local competitors or predators as are the established native species.

Conservation of Biodiversity

Conservation biology is the study and implementation of methods to protect biodiversity. Effective conservation strategies are based on principles of ecology. These strategies include natural resource conservation and species conservation. Even soil has to be conserved. Learn about what can happen to soil in the MiniLab on the next page.

Natural resources are those parts of the environment that are useful or necessary for living organisms. Natural resources include sunlight, water, air, and plant and animal resources. Because species are dependent upon sufficient supplies of natural resources, they must be considered during the planning of any conservation activity.

Legal protections of species

In response to concern about species extinction, the U.S. Endangered Species Act became law in 1973. This law made it illegal to harm any species on the endangered or threatened species lists. Further, the law made it illegal for federal agencies to fund any project that would harm organisms on these lists. Harm includes changing an ecosystem where endangered or threatened species live.

Worldwide, the Convention on International Trade in Endangered Species (CITES) has established lists of species for which international trade is prohibited or controlled. This agreement has been endorsed by more than 120 countries.

Preserving habitats

The importance of preserving habitats has been recognized in the United States and many other countries. A habitat is the physical location where an organism lives and interacts with its environment. One way that habitats have been protected is through the creation of natural preserves and parks. The United States established its first national park—Yellowstone National Park—in 1872. Initially Yellowstone was created to protect the region’s unique geology. However, its ecological importance is recognized as being equally significant. Species of bear, bison, moose, and elk roam the park in much the same way that they roamed the area hundreds of years ago. Other national parks in the United States include Big Cypress National Preserve, Crater Lake National Park, Big Bend National Park, and Sequoia National Park. Each park protects a unique natural environment and provides habitats for many organisms.

Establishing parks and other protected regions has been an effective way to preserve ecosystems and the communities of species that live in them. Although natural preserves make up a relatively small amount of land in some countries, these areas contain a large amount of biodiversity. For example, 3.9 percent of the land in the Democratic Republic of Congo in Africa has been protected. However, this small amount of land is home to almost 90 percent of the nation’s bird species.

Habitat corridors

Is it better to protect one large piece of land or several smaller, disconnected pieces of land? Recall the research describing the number of insect and spider species on islands of different sizes. In general, larger islands had more species than smaller islands had. Therefore, a general strategy for protecting the biodiversity of an area probably is to protect the largest area possible. However, research is showing that keeping wildlife populations completely separate from one another may be resulting in inbreeding within populations. Therefore, another strategy for preserving biodiversity is to connect protected areas with habitat corridors.

Corridors such as the one in Figure 5.11 are being built in Florida to protect the Florida panther. Habitat corridors are protected strips of land that allow the migration of organisms from one wilderness area to another. Research has shown that corridors can help overcome some of the effects of habitat destruction and are beneficial for both plants and animals.

Working with people

Saying an area is protected does not automatically make all the species there safe. Parks and protected areas usually hire people, such as rangers, to manage the parks and ensure the protection of organisms. In some areas, access by people is restricted. In other lands, people can harvest food or obtain materials but this sort of activity is managed. The philosophy of sustainable use strives to enable people to use natural resources in ways that will benefit them and maintain the ecosystem. For example, in Figure 5.12, people harvest Brazil nuts to eat and to sell. This provides the opportunity to earn a living and the ecology of the area is maintained.

Reintroduction and species preservation programs

The year is 1991. A wildlife manager carries a cage containing a captive-bred black-footed ferret, like the one in Figure 5.13. She opens the cage door, and the ferret steps out onto the ground. In the 1970s, the black-footed ferret was almost lost from the wild and was listed as an endangered species. The ferret depends upon prairie dogs for food, and prairie dog habitat had been reduced by rural land use. In 1981, a small population of black-footed ferrets was found by a rancher. Biologists studied the ferrets and established a captive-breeding program at the National Black-footed Ferret Conservation Center in Wyoming. The captive-breeding program has become a success, and black-footed ferrets have been released into the wild in a number of western states. Reintroduction programs, such as this one, release organisms into an area where the species once lived. Today, about 350 black-footed ferrets live in the wild.

The most successful reintroductions occur when organisms are taken from an area in the wild and transported to a new suitable habitat. The brown pelican was once common along the shores of the Gulf of Mexico. DDT caused this bird’s eggs to break, and the brown pelican completely disappeared from these areas. After DDT was banned in the United States in 1972, 50 brown pelicans were taken from Florida and put on Grand Terre Island in Louisiana. The population grew and spread, and today more than 7000 brown pelicans live in the area.

Captivity

Some species no longer exist in the wild, but a small number of individual organisms is maintained by humans. An organism that is held by people is said to be in captivity. The ginkgo tree, as shown in Figure 5.14, is an example of a species surviving extinction because it was kept by people. The ginkgo is an ancient tree; all similar species became extinct long ago. However, Chinese monks planted the ginkgo tree around their temples, thereby preventing the tree from becoming extinct.

Protecting plant species

The ideal way to protect a plant species is to allow it to exist in a natural ecosystem. But seeds can be cooled and stored for long periods of time. By establishing seed banks for threatened and endangered plants, the species can be reintroduced if they become extinct. Reintroductions of captive animals are more difficult than for plants. Keeping animals in captivity, with enough space, adequate care, and proper food, is expensive. Animals kept in captivity may lose the necessary behaviors to survive and reproduce in the wild. Despite the difficulties involved, some species held in captivity, such as the Arabian Oryx and the California condor, have been reintroduced to their native habitats after becoming nearly extinct in the wild.

**Textbook 4: Dynamics of Life Vol. 2**

Ecology

Scientists can gain valuable insight about the interactions between organisms and their environments and between different species of organisms by observing them in their natural environments. Each organism, regardless of where it lives, depends on nonliving factors found in its environment and on other organisms living in the same environment for survival. For example, green plants provide a source of food for many organisms as well as a place to live. The animals that eat the plants provide a source of food for other animals. The interactions and interdependence of organisms with each other and their environments are not unique. The same type of dependency occurs whether the environment is a barren desert, a tropical rain forest, or a grassy meadow. Ecology is the scientific discipline in which the relationships among living organisms and the interaction the organisms have with their environments are studied.

The study of organisms and their environments is not new. The word ecology was first introduced in 1866 by Ernst Haeckel, a German biologist. Since that time, there have been many significant milestones in ecology, as shown in Figure 2.1.

Scientists who study ecology are called ecologists. Ecologists observe, experiment, and model using a variety of tools and methods. For example, ecologists, like the one shown in Figure 2.2, perform tests in organisms’ environments. Results from these tests might give clues as to why organisms are able to survive in the water, why organisms become ill or die from drinking the water, or what organisms could live in or near the water. Ecologists also observe organisms to understand the interactions between them. Some observations and analyses must be made over long periods of time in a process called longitudinal analysis.

A model allows a scientist to represent or simulate a process or system. Studying organisms in the field can be difficult because there often are too many variables to study at one time. Models allow ecologists to control the number of variables present and to slowly introduce new variables in order to fully understand the effect of each variable.

The Biosphere

Because ecologists study organisms and their environments, their studies take place in the biosphere. The biosphere is the portion of Earth that supports life. The photo of Earth taken from space shown in Figure 2.3 shows why the meaning of the term biosphere should be easy to remember. The term bio means “life,” and a sphere is a geometric shape that looks like a ball. When you look at Earth from this vantage point, you can see how it is considered to be “a ball of life.” Although “ball of life” is the literal meaning of the word biosphere, this is somewhat misleading. The biosphere includes only the portion of Earth that includes life. The biosphere forms a thin layer around Earth. It extends several kilometers above the Earth’s surface into the atmosphere and extends several kilometers below the ocean’s surface to the deep-ocean vents. It includes landmasses, bodies of freshwater and saltwater, and all locations below Earth’s surface that support life.

Figure 2.4 shows a satellite image of Earth’s biosphere on the surface of Earth. The photo is color-coded to represent the distribution of chlorophyll. Chlorophyll is a green pigment found in green plants and algae that you will learn about in later chapters. Because most organisms depend on green plants or algae for survival, green plants are a good indicator of the distribution of living organisms in an area. In the oceans, red represents areas with the highest density of chlorophyll followed by yellow, then blue, and then pink, representing the lowest density. On land, dark green represents the area with highest chlorophyll density and pale yellow represents the area with the lowest chlorophyll density.

The biosphere also includes areas such as the frozen polar regions, deserts, oceans, and rain forests. These diverse locations contain organisms that are able to survive in the unique conditions found in their particular environment. Ecologists study these organisms and the factors in their environment. These factors are divided into two large groups—the living factors and the nonliving factors.

Biotic factors

The living factors in an organism’s environment are called the **biotic factors**. Consider the biotic factors in the habitat of salmon shown in Figure 2.5. These biotic factors include all of the organisms that live in the water, such as other fish, algae, frogs, and microscopic organisms. In addition, organisms that live on the land adjacent to the water might be biotic factors for the salmon. Migratory animals, such as birds that pass through the area, also are biotic factors. The interactions among organisms are necessary for the health of all species in the same geographic location. For example, the salmon need other members of their species to reproduce. Salmon also depend on other organisms for food and, in turn, are a food source for other organisms.

Abiotic factors

The nonliving factors in an organism’s environment are called **abiotic factors**. The abiotic factors for different organisms vary across the biosphere, but organisms that live in the same geographic area might share the same abiotic factors. These factors might include temperature, air or water currents, sunlight, soil type, rainfall, or available nutrients. Organisms depend on abiotic factors for survival. For example, the abiotic factors important to a particular plant might be the amount of rainfall, the amount of sunlight, the type of soil, the range of temperature, and the nutrients available in the soil. The abiotic factors for the salmon in Figure 2.5 might be the temperature range of the water, the pH of the water, and the salt concentration of the water.

Organisms are adapted to surviving in the abiotic factors that are present in their natural environments. If an organism moves to another location with a different set of abiotic factors, the organism might die if it cannot adjust quickly to its new surroundings. For example, if a lush green plant that normally grows in a swampy area is transplanted to a dry desert, the plant likely will die because it cannot adjust to abiotic factors present in the desert.

Levels of Organization

The biosphere is too large and complex for most ecological studies. To study relationships within the biosphere, ecologists look at different levels of organization or smaller pieces of the biosphere. The levels increase in complexity as the numbers and interactions between organisms increase. The levels of organization are

•organism;

•population;

•biological community;

•ecosystem;

•biome;

•biosphere.

Organisms, populations, and biological communities

The lowest level of organization is the individual organism itself. In Figure 2.6, the organism is represented by a single fish. Individual organisms of a single species that share the same geographic location at the same time make up a population. The school of fish represents a population of organisms. Individual organisms often compete for the same resources, and if resources are plentiful, the population can grow. However, usually there are factors that prevent populations from becoming extremely large. For example, when the population has grown beyond what the available resources can support, the population size begins to decline until it reaches the number of individuals that the available resources can support.

The next level of organization is the biological community. A biological community is a group of interacting populations that occupy the same geographic area at the same time. Organisms might or might not compete for the same resources in a biological community. The collection of plant and animal populations, including the school of fish, represents a biological community.

Ecosystems, biomes, and the biosphere

The next level of organization after a biological community is an ecosystem. An ecosystem is a biological community and all of the abiotic factors that affect it. As you can see in Figure 2.6, an ecosystem might contain an even larger collection of organisms than a biological community. In addition, it contains the abiotic factors present, such as water temperature and light availability.

Although Figure 2.6 represents an ecosystem as a large area, an ecosystem also can be small, such as an aquarium or tiny puddle. The boundaries of an ecosystem are somewhat flexible and can change, and ecosystems even might overlap.

The next level of organization is called the biome and is one that you will learn more about in Chapter 3. A biome is a large group of ecosystems that share the same climate and have similar types of communities. The biome shown in Figure 2.6 is a marine biome. All of the biomes on Earth combine to form the highest level of organization—the biosphere.

Ecosystem Interactions

The interactions between organisms are important in an ecosystem. A community of organisms increases the chances for survival of any one species by using the available resources in different ways. If you look closely at a tree in the forest, like the one shown in Figure 2.7, you will find a community of different birds using the resources of the tree in different ways. For example, one bird species might eat insects on the leaves while another species of bird eats the ants found on the bark. The chance of survival for the birds increases because they are using different resources.

The trees shown in Figure 2.7 also are habitats. A habitat is an area where an organism lives. A habitat might be a single tree for an organism that spends its life on one tree. If the organism moves from tree to tree, its habitat would be a grove of trees.

Organisms not only have a habitat—they have a niche as well. A niche is the role or position that an organism has in its environment. An organism’s niche is how it meets its needs for food, shelter, and reproduction. The niche might be described in terms of requirements for living space, temperature, moisture, or in terms of appropriate mating or reproduction conditions.

Community Interactions

Organisms that live together in a biological community constantly interact. These interactions, along with the abiotic factors, shape an ecosystem. Interactions include competition for basic needs such as food, shelter, and mates, as well as relationships in which organisms depend on each other for survival.

Competition

Competition occurs when more than one organism uses a resource at the same time. Resources are necessary for life and might include food, water, space, and light. For example, during a drought, as shown in Figure 2.8, water might be scarce for many organisms. The strong organisms directly compete with the weak organisms for survival. Usually the strong survive and the weak die. Some organisms might move to another location where water is available. At times when water is plentiful, all organisms share the resources and competition is not as fierce.

Predation

Many, but not all, species get their food by eating other organisms. The act of one organism consuming another organism for food is predation. The organism that pursues another organism is the predator, and the organism that is pursued is the prey. If you have watched a cat catch a bird or mouse, you have witnessed a predator catch its prey.

Some insects also prey on other insects. Ladybugs and praying mantises are two examples of insects that are predators. Some insect predators also are called beneficial insects because they are used by organic gardeners for insect control. Instead of using insecticides, organic gardeners use beneficial insects to control other insect populations.

Animals are not the only organisms that are predators. The Venus flytrap, a plant native to some regions of North and South Carolina, has modified leaves that form small traps for insects and other small animals. The plant emits a sweet, sticky substance that attracts insects. When the insect lands on the leaf, the leaf trap snaps shut. Then, the plant secretes a substance that digests the insect over several days.

Symbiotic relationships

Some species survive because of relationships they have developed with other species. The close relationship that exists when two or more species live together is symbiosis. There are three different kinds of symbiosis: mutualism, commensalism, and parasitism.

Mutualism The relationship between two or more organisms that live closely together and benefit from each other is mutualism. Lichens, shown in Figure 2.9, display an example of a mutualistic relationship between fungi and algae. The tree merely provides a habitat for lichens, allowing it to receive ample sunlight. The algae provide food for the fungi, and the fungi provide a habitat for the algae. The close association of these two organisms provides two basic needs for the organisms—food and shelter.

Commensalism Look back at Figure 2.9. This time, think about the relationship between the lichens and the tree. The lichens benefit from the relationship by gaining more exposure to sunlight, but they do not harm the tree. This type of relationship is commensalism.

Commensalism is a relationship in which one organism benefits and the other organism is neither helped nor harmed.

The relationship between clownfish and sea anemones is another example of commensalism. Clownfish are small, tropical marine fish. Clownfish swim among the stinging tentacles of sea anemones without harm. The sea anemones protect the fish from predators while the clownfish eat bits of food missed by the sea anemones. This is a commensal relationship because the clownfish receives food and protection while the sea anemones are not harmed, nor do they benefit from this relationship.

Parasitism A symbiotic relationship in which one organism benefits at the expense of another organism is parasitism. Parasites can be external, such as ticks and fleas, or internal, such as bacteria, tapeworms, and roundworms, which are discussed in detail in Chapters 18 and 25. The heartworms in Figure 2.10 show how destructive parasites can be. Pet dogs in many areas of the United States are treated to prevent heartworm infestation. Usually the heartworm, the parasite, does not kill the host, but it might harm or weaken it. In parasitism, if the host dies, the parasite also would die unless it quickly finds another host.

Another type of parasitism is brood parasitism. Brown-headed cowbirds demonstrate brood parasitism because they rely on other bird species to build their nests and incubate their eggs. A brown-headed cowbird lays its eggs in another bird’s nest and abandons the eggs. The host bird incubates and feeds the young cowbirds. Often the baby cowbirds push the host’s eggs or young from the nest, resulting in the survival of only the cowbirds. In some areas, the brown-headed cowbirds have significantly lowered the population of songbirds through this type of parasitism.

Flow of Energy in an Ecosystem

Energy in an Ecosystem

One way to study the interactions of organisms within an ecosystem is to follow the energy that flows through an ecosystem. Organisms differ in how they obtain energy, and they are classified as autotrophs or heterotrophs based on how they obtain their energy in an ecosystem.

Autotrophs

All of the green plants and other organisms that produce their own food in an ecosystem are primary producers called autotrophs. An autotroph is an organism that collects energy from sunlight or inorganic substances to produce food. As you will learn in Chapter 8, organisms that have chlorophyll absorb energy during photosynthesis and use it to convert the inorganic substances carbon dioxide and water to organic molecules. In places where sunlight is unavailable, some bacteria use hydrogen sulfide and carbon dioxide to make organic molecules to use as food. Autotrophs are the foundation of all ecosystems because they make energy available for all other organisms in an ecosystem.

Heterotrophs

A heterotroph is an organism that gets its energy requirements by consuming other organisms.

Therefore, heterotrophs also are called consumers. A heterotroph that eats only plants is an herbivore such as a cow, a rabbit, or grasshopper. Heterotrophs that prey on other heterotrophs, such as wolves, lions, and lynxes, shown in Figure 2.11, are called carnivores.

In addition to herbivores and carnivores, there are organisms that eat both plants and animals, called omnivores. Bears, humans, and mockingbirds are examples of omnivores. The detritivores, which eat fragments of dead matter in an ecosystem, return nutrients to the soil, air, and water where the nutrients can be reused by organisms. Detritivores include worms and many aquatic insects that live on stream bottoms. They feed on small pieces of dead plants and animals. Decomposers, similar to detritivores, break down dead organisms by releasing digestive enzymes. Fungi, such as those in Figure 2.12, and bacteria are decomposers.

All heterotrophs, including detritivores, perform some decomposition when they consume another organism and break down its body into organic compounds. However, it is primarily the decomposers that break down organic compounds and make nutrients available to producers for reuse. Without the detritivores and decomposers, the entire biosphere would be littered with dead organisms. Their bodies would contain nutrients that would no longer be available to other organisms. The detritivores are an important part of the cycle of life because they make nutrients available for all other organisms.

Models of Energy Flow

Ecologists use food chains and food webs to model the energy flow through an ecosystem. Like any model, food chains and food webs are simplified representations of the flow of energy. Each step in a food chain or food web is called a trophic level. Autotrophs make up the first trophic level in all ecosystems. Heterotrophs make up the remaining levels. With the exception of the first trophic level, organisms at each trophic level get their energy from the trophic level before it.

Food chains

A **food chain** is a simple model that shows how energy flows through an ecosystem. Figure 2.13 shows a typical grassland food chain. Arrows represent the one-way energy flow which typically starts with autotrophs and moves to heterotrophs. The flower uses energy from the Sun to make its own food. The grasshopper gets its energy from eating the flower. The mouse gets its energy from eating the grasshopper. Finally, the snake gets its energy from eating the mouse. Each organism uses a portion of the energy it obtains from the organism it eats for cellular processes to build new cells and tissues.

The remaining energy is released into the surrounding environment and no longer is available to these organisms.

Food webs

Feeding relationships usually are more complex than a single food chain because most organisms feed on more than one species. Birds, for instance, eat a variety of seeds, fruits, and insects. The model most often used to represent the feeding relationships in an eco-system is a food web. A **food web** is a model representing the many interconnected **food chains** and pathways in which energy flows through a group of organisms. Figure 2.14 shows a food web illustrating the feeding relationships in a desert community.

Ecological pyramids

Another model that ecologists use to show how energy flows through ecosystems is the ecological pyramid. An **ecological pyramid** is a diagram that can show the relative amounts of energy, bio-mass, or numbers of organisms at each trophic level in an ecosystem.

Notice in Figure 2.15 that in a pyramid of energy, approximately 90 percent of all energy is not transferred to the level above it. This occurs because most of the energy contained in the organisms at each level is consumed by cellular processes or released to the environment as heat. Usually, the amount of biomass—the total mass of living matter at each trophic level—decreases at each trophic level. As shown in the pyramid of numbers, the relative number of organisms at each trophic level also decreases because there is less energy available to support organisms.

Cycling of Matter

Cycles in the Biosphere

Energy is transformed into usable forms to support the functions of an ecosystem. A constant supply of usable energy for the biosphere is needed, but this is not true of matter. The law of conservation of mass states that matter is not created or destroyed. Therefore, natural processes cycle matter through the biosphere. Matter—anything that takes up space and has mass—provides the nutrients needed for organisms to function. A nutrient is a chemical substance that an organism must obtain from its environment to sustain life and to undergo life processes. The bodies of all organisms are built from water and nutrients such as carbon, nitrogen, and phosphorus.

In most ecosystems, plants obtain nutrients, in the form of elements and compounds, from the air, soil, or water. Plants convert some elements and compounds into organic molecules that they use. The nutrients flow through organisms in an ecosystem such as the ecosystem shown in Figure 2.16. The green grass captures substances from the air, soil, and water, and then converts them into usable nutrients. The grass provides nutrients for the cow. If an organism eats the cow, the nutrients found in the cow are passed on to the next consumer. The nutrients are passed from producer—the green grass—to consumers. Decomposers return the nutrients to the cycle at every level.

The cycling of nutrients in the biosphere involves both matter in living organisms and physical processes found in the environment such as weathering. Weathering breaks down large rocks into particles that become part of the soil used by plants and other organisms. The exchange of matter through the biosphere is called the **biogeochemical cycle**. As the name suggests, these cycles involve living organisms (bio), geological processes (geo), and chemical processes (chemical).

The water cycle Living organisms cannot live without water. Hydrologists study water found underground, in the atmosphere, and on the surface of Earth in the form of lakes, streams, rivers, glaciers, ice caps, and oceans. Use Figure 2.17 to trace processes that cycle water through the biosphere.

Water is constantly evaporating into the atmosphere from bodies of water, soil, and organisms. Water in the atmosphere is called water vapor. Water vapor rises and begins to cool in the atmosphere. Clouds form when the cooling water vapor condenses into droplets around dust particles in the atmosphere. Water falls from clouds as precipitation in the form of rain, sleet, or hail, transferring water to the Earth’s surface. As you can see in Figure 2.17, groundwater and runoff from land surfaces flow into streams, rivers, lakes, and oceans, only to evaporate into the atmosphere to continue the **water cycle**. Approximately 90 percent of water vapor evaporates from oceans, lakes, and rivers; about 10 percent evaporates from the surface of plants through a process called transpiration. You will learn more about transpiration in Chapter 22.

All living organisms rely on freshwater. Freshwater constitutes only about 3 percent of all water on Earth. Water available for living organisms is about 31 percent of all freshwater. About 69 percent of all fresh-water is found in ice caps and glaciers, which then is unavailable for use by living organisms. Even ocean-dwelling organisms rely on freshwater flowing to oceans to prevent high saline content and maintain ocean volume.

The carbon and oxygen cycles

As you will learn in Chapter 6, all living things are composed of molecules that contain carbon. Atoms of carbon form the framework for important molecules such as proteins, carbohydrates, and fats. Oxygen is another element that is important to many life processes. Carbon and oxygen often make up molecules essential for life, including carbon dioxide and simple sugar.

Look at the cycles illustrated in Figure 2.18. During a process called photosynthesis, discussed in Chapter 8, green plants and algae convert carbon dioxide and water into carbohydrates and release oxygen back into the air. These carbohydrates are used as a source of energy for all organisms in the food web. Carbon dioxide is recycled when autotrophs and heterotrophs release it back into the air during cellular respiration. Carbon and oxygen recycle relatively quickly through living organisms.

Carbon enters a long-term cycle when organic matter is buried underground and converted to peat, coal, oil, or gas deposits. The carbon might remain as fossil fuel for millions of years. Carbon is released from fossil fuels when they are burned, which adds carbon dioxide to the atmosphere.

In addition to the removal of carbon from the short-term cycle by fossil fuels, carbon and oxygen can enter a long-term cycle in the form of calcium carbonate, as shown in Figure 2.19. Calcium carbonate is found in the shells of plankton and animals such as coral, clams, and oysters. These organisms, such as algae, fall to the bottom of the ocean floor, creating vast deposits of limestone rock. Carbon and oxygen remain trapped in these deposits until weathering and erosion release these elements to become part of the short-term cycle.

The **nitrogen cycle** Nitrogen is an element found in proteins. The largest concentration of nitrogen is found in the atmosphere. Plants and animals cannot use nitrogen directly from the atmosphere. Nitrogen gas is captured from the air by species of bacteria that live in water, the soil, or grow on the roots of some plants. The process of capture and conversion of nitrogen into a form that is useable by plants is called nitrogen fixation. Some nitrogen also is fixed during electrical storms when the energy from lightning bolts changes nitrogen gas to nitrates. Nitrogen also is added to soil when chemical fertilizers are applied to lawns, crops, or other areas.

Nitrogen enters the **food web** when plants absorb nitrogen compounds from the soil and convert them into proteins, as illustrated in Figure 2.20. Consumers get nitrogen by eating plants or animals that contain nitrogen. They reuse the nitrogen and make their own proteins. Because the supply of nitrogen in a **food web** is dependent on the amount of nitrogen that is fixed, nitrogen often is a factor that limits the growth of producers.

Nitrogen is returned to the soil in several ways, also shown in Figure 2.20. When an animal urinates, nitrogen returns to the water or soil and is reused by plants. When organisms die, decomposers transform the nitrogen in proteins and other compounds into ammonia. Organisms in the soil convert ammonia into nitrogen compounds that can be used by plants. Finally, in a process called denitrification, some soil bacteria convert fixed nitrogen compounds back into nitrogen gas, which returns it to the atmosphere.

The **phosphorus cycle** Phosphorus is an element that is essential for the growth and development of organisms. Figure 2.21 illustrates the two cycles of phosphorus—a short-term and long-term cycle. In the short-term cycle, phosphorus as phosphates in solution, is cycled from the soil to producers and then from the producers to consumers. When organisms die or produce waste products, decomposers return the phosphorus to the soil where it can be used again. Phosphorus moves from the short-term cycle to the long-term cycle through precipitation and sedimentation to form rocks. In the long-term cycle, weathering or erosion of rocks that contain phosphorus slowly adds phosphorus to the cycle. Phosphorus, in the form of phosphates, may be present only in small amounts in soil and water. Therefore, phosphorus often is a factor that limits the growth of producers.

Community Ecology

Communities

When you describe your community, you probably include your family, the students in your school, and the people who live nearby. A biological community is a group of interacting populations that occupy the same area at the same time. Therefore, your community also includes plants, other animals, bacteria, and fungi. Not every community includes the same variety of organisms. An urban community is different from a rural community, and a desert community is different from an arctic community.

In Chapter 2, you learned that organisms depend on one another for survival. You also learned about abiotic factors and that abiotic factors affect individual organisms. How, then, might abiotic factors affect communities? Consider soil, which is an abiotic factor. If soil becomes too acidic, some species might die or become extinct. This might affect food sources for other organisms, resulting in a change in the community.

Organisms adapt to the conditions in which they live. For example, a wolf’s heavy fur coat enables it to survive in harsh winter climates, and a cactus’s ability to retain water enables it to tolerate the dry conditions of a desert. Depending on which factors are present, and in what quantities, organisms can survive in some ecosystems but not in others. As an example, the plants in the desert oasis shown in Figure 3.1 decrease in abundance away from the water source.

Limiting factors

Any abiotic factor or biotic factor that restricts the numbers, reproduction, or distribution of organisms is called a **limiting factor**. Abiotic limiting factors include sunlight, climate, temperature, water, nutrients, fire, soil chemistry, and space. Biotic limiting factors include living things, such as other plant and animal species.

Factors that restrict the growth of one population might enable another to thrive. For example, in the oasis shown in Figure 3.1, water is a limiting factor for all of the organisms. Temperature also might be a limiting factor. Desert species must be able to withstand the heat of the Sun and the cold temperatures of desert nights.

Range of tolerance

For any environmental factor, there is an upper limit and lower limit that define the conditions in which an organism can survive. For example, steelhead trout live in cool, clear coastal rivers and streams from California to Alaska. The ideal range of water temperature for steelhead trout is between 13°C and 21°C, as illustrated in Figure 3.2. However, steelhead trout can survive water temperatures from 9°C to 25°C. At these temperatures, steelhead trout experience physiological stress, such as inability to grow or reproduce. They will die if the water temperature goes beyond the upper and lower limits.

Have you ever had to tolerate a hot day or a boring activity? Similarly, the ability of any organism to survive when subjected to abiotic factors or biotic factors is called tolerance. Consider Figure 3.2 again. Steelhead trout tolerate a specific range of temperatures. That is, the range of tolerance of water temperature for steelhead is 9°C to 25°C. Notice the greatest number of steelhead live in the optimum zone in which the temperature is best for survival. Between the optimum zone and the tolerance limits lies the zone of physiological stress. At these temperatures, there are fewer fish. Beyond the upper tolerance limit of 25°C and the lower tolerance limit of 9°C, there are no steelhead trout. Therefore, water temperature is a limiting factor for steelhead when water temperature is outside the range of tolerance.

Ecological Succession

Ecosystems are constantly changing. They might be modified in small ways, such as a tree falling in the forest, or in large ways, such as a forest fire. They also might alter the communities that exist in the ecosystem. Forest fires can be good and even necessary for the forest community. Forest fires return nutrients to the soil. Some plants, such as fireweed, have seeds that will not sprout until they are heated by fire. Some ecosystems depend on fires to get rid of debris. If fires are prevented, debris builds up to the point where the next fire might burn the shrubs and trees completely. A forest fire might change the habitat so drastically that some species no longer can survive, but other species might thrive in the new, charred conditions.

The change in an ecosystem that happens when one community replaces another as a result of changing abiotic and biotic factors is ecological succession. There are two types of ecological succession— primary succession and secondary succession.

Primary succession

On a solidified lava flow or exposed rocks on a cliff, no soil is present. If you took samples of each and looked at them under a microscope, the only biological organisms you would observe would be bacteria and perhaps fungal spores or pollen grains that drifted there on air currents. The establishment of a community in an area of exposed rock that does not have any topsoil is primary succession, as illustrated in Figure 3.3. Primary succession usually occurs very slowly at first.

Most plants require soil for growth. How is soil formed? Usually lichens, a combination of a fungus and algae that you will learn more about in Chapter 20, begin to grow on the rock. Because lichens, along with some mosses, are among the first organisms to appear, they are called pioneer species. Pioneer species help to create soil by secreting acids that help to break down rocks.

As pioneer organisms die, their decaying organic materials, along with bits of sediment from the rocks, make up the first stage of soil development. At this point, small weedy plants, including ferns, and other organisms such as fungi and insects, become established. As these organisms die, additional soil is created. Seeds, brought in by animals, water, or wind, begin to grow in the newly formed soil. Eventually, enough soil is present so that shrubs and trees can grow.

A climax community eventually can develop from bare rock, as illustrated in Figure 3.3. The stable, mature community that results when there is little change in the composition of species is a climax community. Scientists today realize that disturbances, such as climate change, are ongoing in communities, thus a true climax community is unlikely to occur.

Secondary succession Disturbances such as fire, flood, or a windstorm can disrupt a community. After a disturbance, new species of plants and animals might occupy the habitat. Over time, there is a natural tendency for the species belonging to the mature community to return. Secondary succession is the orderly and predictable change that takes place after a community of organisms has been removed but the soil has remained intact. Pioneer species—mainly plants that begin to grow in the disturbed area—are the first species to start secondary succession.

During secondary succession, as in primary succession, the community of organisms changes over a period of time. Figure 3.4 shows how species composition changes after a forest fire. Secondary succession usually occurs faster than primary succession because soil already exists and some species still will be present (although there might be fewer of them). Also, undisturbed areas nearby can be sources of seeds and animals.

Succession’s end point Ecological succession is likely a very complex process that involves many factors. The end point of succession after a disturbance cannot be predicted. Natural communities are constantly changing at different rates, and the process of succession is very slow. Human activities also affect the species that might be present.

Because of these factors, it is difficult to determine if succession has reached a climax community anywhere on Earth.

Terrestrial Biomes

Effects of Latitude and Climate

Regardless of where you live, you are affected by weather and climate. On the news, a meteorologist will make forecasts about the upcoming weather. Weather is the condition of the atmosphere at a specific place and time. What causes the variation in the weather patterns that you experience? What are the effects of these weather patterns on organisms that live in different areas on Earth? One of the keys to understanding these communities is to be aware of latitude and climatic conditions.

Latitude The distance of any point on the surface of Earth north or south from the equator is latitude. Latitudes range from 0° at the equator to 90° at the poles. Light from the Sun strikes Earth more directly at the equator than at the poles, as illustrated in Figure 3.5. As a result, Earth’s surface is heated differently in different areas. Ecologists refer to these areas as polar, temperate, and tropical zones.

Climate

The average weather conditions in an area, including temperature and precipitation, describe the area’s climate. An area’s latitude has a large effect on its climate. If latitude were the only abiotic factor involved in climate, biomes would be spread in equal bands encircling Earth. However, other factors such as elevation, continental landmasses, and ocean currents also affect climate. The graph in Figure 3.6 shows how temperature and precipitation influence the communities that develop in an area. You can investigate the relationship between temperature and latitude in Minilab 3.1.

Recall from Chapter 2 that a biome is a large group of ecosystems that share the same climate and have similar types of communities. It is a group of plant and animal communities that have adapted to a region’s climate. A biome’s ecosystems occur over a large area and have similar plant communities. Even a small difference in temperature or precipitation can affect the location of a biome. Refer to Figure 3.7 to learn how Earth’s ocean currents and prevailing winds affect climate. Also illustrated in Figure 3.7 are two ways humans have affected climate—through the hole in the ozone layer and through global warming. Global warming is in part a result of the **greenhouse effect**.

Major Land Biomes

Biomes are classified primarily according to the characteristics of their plants. Biomes also are characterized by temperature and precipitation. Animal species are an important characteristic of biomes as well. This section describes each of the major land biomes.

Tundra Extending in a band below the polar ice caps across northern North America, Europe, and Siberia in Asia is the tundra. The tundra is a treeless biome with a layer of permanently frozen soil below the surface called permafrost. Although the ground thaws to a depth of a few centimeters in the summer, its constant cycles of freezing and thawing do not allow tree roots to grow. Some animals and shallow-rooted plants that have adapted to tundra conditions are illustrated in Figure 3.8.

Boreal forest

South of the tundra is a broad band of dense evergreen forest extending across North America, Europe, and Asia, called the boreal forest. The boreal forest, illustrated in Figure 3.9, also is called northern coniferous forest, or taiga. Summers in the boreal forest are longer and somewhat warmer than in the tundra, enabling the ground to remain warmer than in the tundra. Boreal forests, therefore, lack a permafrost layer.

Temperate forest

Temperate forests cover much of southeastern Canada, the eastern United States, most of Europe, and parts of Asia and Australia. As shown in Figure 3.10, the temperate forest is composed mostly of broad-leaved, deciduous trees—trees that shed their leaves in autumn.

The falling red, orange, and gold leaves return nutrients to the soil. Winters are cold. In spring, warm temperature and precipitation restart the growth cycles of plants and trees. Summers are hot.

Temperate woodland and shrubland Open woodlands and mixed shrub communities are found in areas with less annual rainfall than in temperate forests. The woodland biome occurs in areas surrounding the Mediterranean Sea, on the western coasts of North and South America, and in South Africa and Australia. Areas that are dominated by shrubs, such as in California, are called the chaparral. Figure 3.11 illustrates woodland and shrub communities.

Temperate grassland

A biome that is characterized by fertile soils that are able to support a thick cover of grasses is called grassland, illustrated in Figure 3.12.

Drought, grazing animals, and fires keep grasslands from becoming forests. Due to their underground stems and buds, perennial grasses and herbs are not eliminated by the fires that destroy most shrubs and trees. Temperate grasslands are found in North America, South America,

Asia, Africa, and Australia. Grasslands are called steppes in Asia; prairies in North America; pampas, llanos, and cerrados in South America; savannahs and velds in Africa; and rangelands in Australia.

Desert Deserts exist on every continent except Europe. A desert is any area in which the annual rate of evaporation exceeds the rate of precipitation. You might imagine a desert as a desolate place full of sand dunes, but many deserts do not match that description. As shown in Figure 3.13, deserts can be home to a wide variety of plants and animals.

Tropical savanna

A tropical savanna is characterized by grasses and scattered trees in climates that receive less precipitation than some other tropical areas. Tropical savanna biomes occur in Africa,

South America, and Australia. The plants and animals shown in Figure 3.14 are common to tropical savannas.

Tropical seasonal forest Figure 3.15 illustrates a tropical seasonal forest. Tropical seasonal forests, also called tropical dry forests, grow in areas of Africa, Asia, Australia, and South and Central America. In one way, the tropical seasonal forest resembles the temperate deciduous forest because during the dry season, almost all of the trees drop their leaves to conserve water.

Tropical rain forest Warm temperatures and large amounts of rainfall throughout the year characterize the tropical rain forest biome illustrated in Figure 3.16. Tropical rain forests are found in much of Central and South America, southern Asia, western Africa, and northeastern Australia. The tropical rain forest is the most diverse of all land biomes. Tall, broad-leaved trees with branches heavy with mosses, ferns, and orchids make up the canopy of the tropical rain forest. Shorter trees, shrubs, and plants, such as ferns and creeping plants, make up another layer, or understory, of tropical rain forests.

Other Terrestrial Areas

You might have noticed that the list of terrestrial biomes does not include some important areas. Many ecologists omit mountains from the list.

Mountains are found throughout the world and do not fit the definition of a biome because their climate characteristics and plant and animal life vary depending on elevation. Polar regions also are not considered true biomes because they are ice masses and not true land areas with soil.

Mountains

If you go up a mountain, you might notice that abiotic conditions, such as temperature and precipitation, change with increasing elevation. These variations allow many communities to exist on a mountain. As Figure 3.17 illustrates, biotic communities also change with increasing altitude, and the tops of tall mountains may support communities that resemble those of the tundra.

Polar regions Polar regions border the tundra at high latitudes. These polar regions are cold all year. In the northern polar region lies the ice-covered Arctic Ocean and Greenland. Antarctica is the continent that lies in the southern polar region. Covered by a thick layer of ice, the polar regions might seem incapable of sustaining life. The coldest temperature, —89˚C, was recorded in the Antarctica. However, as shown in Figure 3.18, colonies of penguins live in Antarctica. Additionally, whales and seals patrol the coasts, preying on penguins, fish, or shrimplike invertebrates called krill. The arctic polar region supports even more species, including polar bears and arctic foxes. Human societies have also inhabited this region throughout history. Although average winter temperature is about —30˚C, the Arctic summer in some areas is warm enough for vegetables to be grown.

Aquatic Ecosystems

The Water on Earth

When you think about water on Earth, you might recall a vacation at the ocean or a geography lesson in which you located Earth’s oceans and seas. You probably have heard about other large bodies of water, such as the Amazon river and the Great Salt Lake. A globe of Earth is mainly blue in color because the planet is largely covered with water.

Ecologists recognize the importance of water because of the biological communities that water supports. In this section, you will read about freshwater, transitional, and marine aquatic ecosystems. You also will read about the abiotic factors that affect these ecosystems.

Freshwater Ecosystems

The major freshwater ecosystems include ponds, lakes, streams, rivers, and wetlands. Plants and animals in these ecosystems are adapted to the low salt content in freshwater and are unable to survive in areas of high salt concentration. Only about 2.5 percent of the water on Earth is freshwater, as illustrated by the circle graph on the left in Figure 3.19.

The graph on the right in Figure 3.19 shows that of that 2.5 percent,

68.9 percent is contained in glaciers, 30.8 percent is groundwater, and only 0.3 percent is found in lakes, ponds, rivers, streams, and wetlands. Interestingly, almost all of the freshwater species live in this 0.3 percent.

Rivers and streams The water in rivers and streams flows in one direction, beginning at a source called a headwater and traveling to the mouth, where the flowing water empties into a larger body of water, as illustrated in Figure 3.20. Rivers and streams also might start from underground springs or from snowmelt. The slope of the landscape determines the direction and speed of the water flow. When the slope is steep, water flows quickly, causing a lot of sediment to be picked up and carried by the water. Sediment is material that is deposited by water, wind, or glaciers. As the slope levels, the speed of the water flow decreases and sediments are deposited in the form of silt, mud, and sand.

The characteristics of rivers and streams change during the journey from the source to the mouth. Interactions between wind and the water stir up the water’s surface, which adds a significant amount of oxygen to the water. Interactions between land and water result in erosion, changes in nutrient availability, and changes to the path of the river or stream.

The currents and turbulence of fast-moving rivers and streams prevent much accumulation of organic materials and sediment. For this reason, there usually are fewer species living in the rapid waters shown in Figure 3.21. An important characteristic of all life in rivers and streams is the ability to withstand the constant water current. Plants that can root themselves into the streambed are common in areas where water is slowed by rocks or sand bars. Young fish hide in these plants and feed on the drifting microscopic organisms and aquatic insects.

In slow-moving water, insect larvae are the primary food source for many fish, including American eel, brown bullhead catfish, and trout. Other organisms, such as crabs and worms, are sometimes present in calm water. Animals that live in slow-moving water include newts, tadpoles, and frogs.

Lakes and ponds

An inland body of standing water is called a lake or a pond. It can be as small as a few square meters or as large as thousands of square meters. Some ponds might be filled with water for only a few weeks or months each year, whereas some lakes have existed for hundreds of years. Figure 3.22 illustrates how in temperate regions the temperature of lakes and ponds varies depending on the season.

During the winter, most of the water in a lake or pond is the same temperature. In the summer, the warmer water on top is less dense than the colder water at the bottom. During the spring and fall, as the water warms or cools, turnover occurs. The top and bottom layers of water mix, often due to winds, and this results in a uniform water temperature. This mixing circulates oxygen and brings nutrients from the bottom to the surface.

Nutrient-poor lakes, called oligotrophic lakes, often are found high in the mountains. Few plant and animal species are present as a result of small amounts of organic matter and nutrients. Nutrient-rich lakes, called eutrophic lakes, usually are found at lower altitudes. Many plant and animal species are present as a result of organic matter and plentiful nutrients, some of which come from agricultural and urban activities.

Lakes and ponds are divided into three zones based on the amount of sunlight that penetrates the water. The area closest to the shore is the littoral zone. The water in this zone is shallow, which allows sunlight to reach the bottom. Many producers, such as aquatic plants and algae, live in these shallow waters. The abundance of light and producers make the littoral zone an area of high photosynthesis. Many consumers also inhabit this zone, including frogs, turtles, worms, crustaceans, insect larvae, and fish.

The limnetic zone is the open water area that is well lit and is dominated by plankton. Plankton are free-floating photosynthetic autotrophs that live in freshwater or marine ecosystems.

Many species of freshwater fish live in the limnetic zone because food, such as plankton, is readily available.

The water of the profundal zone, the deepest areas of a large lake, is much colder and lower in oxygen than the other two zones. Little light is able to penetrate the limnetic zone in order to enter the profundal zone, which limits the number of species that are able to live there. Figure 3.23 illustrates the zones and biodiversity of lakes and ponds.

Transitional Aquatic Ecosystems

In many areas, aquatic ecosystems do not look like a stream or a pond or even an ocean. In fact, many aquatic environments are a combination of two or more different environments. These areas, which ecologists call transitional aquatic ecosystems, can be areas where land and water or saltwater and freshwater intermingle. Wetlands and estuaries are common examples of transitional aquatic ecosystems.

Wetlands Areas of land such as marshes, swamps, and bogs that are saturated with water and that support aquatic plants are called wetlands. Plant species that grow in the moist, humid conditions of wetlands include duckweed, pond lilies, cattails, sedges, mangroves, cypress, and willows. Bogs, like the cedar bog shown in Figure 3.24, are wet and spongy areas of decomposing vegetation that also support many species of organisms. Wetlands have high levels of species diversity. Many amphibians; reptiles; birds, such as ducks and herons; and mammals, such as raccoons and mink; live in wetlands.

Estuaries

Another important transitional ecosystem is an estuary, shown in Figure 3.25. Estuaries are among the most diverse ecosystems, rivaled only by tropical rain forests and coral reefs. An estuary is an ecosystem that is formed where fresh-water from a river or stream merges with salt water from the ocean.

Estuaries are places of transition—from freshwater to salt water and from land to sea—that are inhabited by a wide variety of species. Algae, seaweeds, and marsh grasses are the dominant producers. However, many animals, including a variety of worms, oysters, and crabs, depend on detritus for food. Detritus is comprised of tiny pieces of organic material.

Mangrove trees also can be found in tropical estuaries, such as the Ever-glades National Park in Florida, where they sometimes form swamps. Many species of marine fishes and invertebrates, such as shrimp, use estuaries as nurseries for their young. Waterfowl, such as ducks and geese, depend on estuary ecosystems for nesting, feeding, and migration rest areas.

Salt marshes are transitional ecosystems similar to estuaries.

Salt-tolerant grasses dominate above the low-tide line, and seagrasses grow in submerged areas of salt marshes. Salt marshes support different species of animals, such as shrimp and shellfish.

Marine Ecosystems

Earth is sometimes called “the water planet.” As such, marine ecosystems have a significant impact on the planet. For example, through photosynthesis, marine algae consume carbon dioxide from the atmosphere and produce over 50 percent of the atmosphere’s oxygen. Additionally, the evaporation of water from oceans eventually provides the majority of precipitation—rain and snow. Like ponds and lakes, oceans are separated into distinct zones.

Intertidal zone

The intertidal zone is a narrow band where the ocean meets land. Organisms that live in this zone must be adapted to the constant changes that occur as daily tides and waves alternately submerge and expose the shore. The intertidal zone is further divided into vertical zones, as illustrated in Figure 3.26. The area of the spray zone is dry most of the time. It is only during high tides that this part of the shoreline is sprayed with salt water, and few plants and animals are able to live in this environment. The high-tide zone is under water only during high tides. However, this area receives more water than the spray zone, so more plants and animals are able to live there. The mid-tide zone undergoes severe disruption twice a day as the tides cover and uncover the shoreline with water. Organisms in this area must be adapted to long periods of air and water. The low-tide zone is covered with water unless the tide is unusually low and is the most populated area of the intertidal zone.

Open ocean ecosystems

As illustrated in Figure 3.27, the zones in the open ocean include the pelagic zone, abyssal zone, and benthic zone. The area to a depth of about 200 m of the pelagic zone is the photic zone, also called the euphotic zone. The photic zone is shallow enough that sunlight is able to penetrate. As depth increases, light decreases. Autotrophic organisms in the photic zone include surface seaweeds and plankton. Animals in the photic zone include many species of fish, sea turtles, jellyfish, whales, and dolphins. Many of these animals feed on plankton, but others feed on larger species.

Below the photic zone lies the aphotic zone—an area where sunlight is unable to penetrate. This region of the pelagic zone remains in constant darkness and generally is cold, but there is thermal layering with a mixing of warm and cold ocean currents. Organisms that depend on light energy to survive cannot live in the aphotic zone.

The benthic zone is the area along the ocean floor that consists of sand, silt, and dead organisms. In shallow benthic zones, sunlight can penetrate to the bottom of the ocean floor. As depth increases, less sunlight penetrates the deeper water, and temperature decreases. Species diversity tends to decrease with depth, except in areas with hydrothermal vents, where shrimp, crabs, and many species of tubeworms are found. Many species of fishes, octopuses, and squids live in the benthic zone.

The deepest region of the ocean is called the abyssal zone. Water in this area is very cold. Most organisms in this zone rely on food materials that drift down from the zones above. However, on the seafloor along the boundaries of Earth’s plates, hydrothermal vents spew large amounts of hot water, hydrogen sulfide, and other minerals. Scientists have found bacterial communities existing in these locations that can use the sulfide molecules for energy. These organisms are at the bottom of a food chain that includes invertebrates, such as clams and crabs, and vertebrates, such as fishes.

Coastal ocean and coral reefs

As you read at the beginning of this chapter, one of the world’s largest coral reefs is off the southern coast of Florida. Coral reefs are among the most diverse ecosystems.

They are widely distributed in warm shallow marine waters. Coral reefs form natural barriers along continents that protect shorelines from erosion. The dominant organisms in coral reefs are corals. When you think of coral, you might picture a hard, stony structure, but this is only the framework secreted by tiny animal polyps.

Most coral polyps have a symbiotic relationship with algae called zooxanthellae. These algae provide corals with food, and in turn, the coral provides protection and access to light for the algae. Corals also feed by extending tentacles to obtain plankton from the water. Other coral reef animals include species of microorganisms, sea slugs, octopuses, sea urchins, sea stars, and fishes. Figure 3.28 shows only a small portion of the diversity of Florida’s coral reef.

Like all ecosystems, coral reefs are sensitive to changes in the environment. Changes that are the result of naturally occurring events, such as increased sediment from a tsunami, can cause the death of a reef. Human activities, such as land development and harvesting for calcium carbonate, also can damage or kill a coral reef. Today, ecologists monitor reefs and reef environments to help protect these delicate ecosystems.

Population Dynamics

Population Characteristics

All species occur in groups called populations. There are certain characteristics that all populations have, such as population density, spatial distribution, and growth rate. These characteristics are used to classify all populations of organisms, including bacteria, animals, and plants.

Population density

One characteristic of a population is its population density, which is the number of organisms per unit area. For example, the population density of cattle egrets, shown with the water buffalo in Figure 4.1, is greater near the buffalo than farther away. Near the water buffalo, there might be three birds per square meter. Fifty meters from the water buffalo, the density of birds might be zero.

Spatial distribution

Another characteristic of a population is called dispersion—the pattern of spacing of a population within an area. Figure 4.2 shows the three main types of dispersion—uniform, clumped groups, and random. Black bears are typically dispersed in a uniform arrangement. American bison are dispersed in clumped groups or herds. White-tailed deer are dispersed randomly with unpredictable spacing. One of the primary factors in the pattern of dispersion for all organisms is the availability of resources such as food.

Population ranges No population, not even the human population, occupies all habitats in the biosphere. Some species, such as the iiwi shown in Figure 4.3, have a very limited population range. This songbird is found only on some of the islands of Hawaii.

Other species, such as the peregrine falcon shown in Figure 4.3, have a vast distribution. Peregrine falcons are found on all continents except Antarctica. Note the distribution of the animals in Figure 4.2.

Recall from Chapter 2 that organisms adapt to the biotic and abiotic factors in their environment. A species might not be able to expand its population range because it cannot survive the abiotic conditions found in the expanded region. A change in temperature range, humidity level, annual rainfall, or sunlight might make a new geographic area uninhabitable for the species. In addition, biotic factors, such as predators, competitors, and parasites, present threats that might make the new location difficult for survival.

Population-Limiting Factors

In Chapter 3, you learned that all species have limiting factors. **Limiting factors** keep a population from continuing to increase indefinitely. Decreasing a limiting factor, such as the available food supply, often changes the number of individuals that are able to survive in a given area. In other words, if the food supply increases a larger population might result, and if the food supply decreases a smaller population might result.

Density-independent factors

There are two categories of limiting factors—density-independent factors and density-dependent factors. Any factor in the environment that does not depend on the number of members in a population per unit area is a **density-independent factor**. These factors usually are abiotic and include natural phenomena such as weather events. Weather events that limit populations include drought or flooding, extreme heat or cold, tornadoes, and hurricanes.

Figure 4.4 shows an example of the effects that fire can have on a population. Fire has damaged this ponderosa pine forest community. Sometimes the extreme heat from a crown fire, which is a fire that advances to the tops of the trees, can destroy many mature ponderosa pine trees—a dominant species in forests of the western United States.

In this example, the fire limits the population of ponderosa trees by killing many of the trees. However, smaller but more frequent ground fires have the opposite effect on the population. By thinning lower growing plants that use up nutrients, a healthier population of mature ponderosa pines is produced.

Populations can be limited by the unintended results of human alterations of the landscape. For example, over the last 100 years, human activities on the Colorado River, such as building dams, water diversions, and water barriers, have significantly reduced the amount of water flow and changed the water temperature of the river. In addition, the introduction of nonnative fish species altered the biotic factors in the river. Because of the changes in the river, the number of small fish called humpback chub was reduced. During the 1960s, the number of humpback chub dropped so low that they were in danger of disappearing from the Colorado River altogether.

Air, land, and water pollution are the result of human activities that also can limit populations. Pollution reduces the available resources by making some of the resources toxic.

Density-dependent factors

Any factor in the environment that depends on the number of members in a population per unit area is a **density-dependent factor**. Density-dependent factors are often biotic factors such as predation, disease, parasites, and competition. A study of density-dependent factors was done on the wolf–moose populations in northern Michigan on Isle Royale, located in Lake Superior.

Prior to the winter of 1947–48, apparently there were no wolves on Isle Royale. During that winter, a single pair of wolves crossed the ice on Lake Superior, reaching the island. During the next ten years, the population of wolves reached about twenty individuals. Figure 4.5 shows some of the results from the long-term study conducted by population biologists.

Notice that the rise and fall of the numbers of each group was dependent on the other group. For example, follow the wolves’ line on the graph. As the number of wolves decreased, the number of moose increased.

Disease

Another density-dependent factor is disease. Outbreaks of disease tend to occur when population size has increased and population density is high. When population density is high, disease is transmitted easily from one individual to another because contact between individuals is more frequent. Therefore, the disease spreads easily and quickly through a population. This is just as true for human populations as it is for populations of protists, plants, and other species of animals.

Competition

Competition between organisms also increases when density increases. When the population increases to a size so that resources such as food or space become limited, individuals in the population must compete for the available resources. Competition can occur within a species or between two different species that use the same resources. Competition for insufficient resources might result in a decrease in population density in an area due to starvation or to individuals leaving the area in search of additional resources. As the population size decreases, competition becomes less severe.

The lemmings shown in Figure 4.6 are an example of a population that often undergoes competition for resources. Lemmings are small mammals that live in the tundra biome. When food is plentiful, their population increases exponentially. As food becomes limited, many lemmings begin to starve and their population size decreases significantly.

Parasites Populations also can be limited by parasites, in a way similar to disease, as population density increases. The presence of parasites is a density-dependent factor that can negatively affect population growth at higher densities.

Population growth rate

An important characteristic of any population is its growth rate. The population growth rate (PGR) explains how fast a given population grows. One of the characteristics of the population ecologists must know, or at least estimate, is natality. The natality of a population is the birthrate, or the number of individuals born in a given time period. Ecologists also must know the mortality—the number of deaths that occur in the population during a given time period.

The number of individuals emigrating or immigrating also is important. Emigration is the term ecologists use to describe the number of individuals moving away from a population. Immigration is the term ecologists use to describe the number of individuals moving into a population. In most instances, emigration is about equal to immigration. Therefore, natality and mortality usually are most important in determining the population growth rate.

Some populations tend to remain approximately the same size from year to year. Other populations vary in size depending on conditions within their habitats. To better understand why populations grow in different ways, you should understand two mathematical models for population growth—the **exponential growth model** and the **logistic growth model**.

Exponential growth model Look at Figure 4.7 to see how a population of mice would grow if there were no limits placed on it by the environment. Assume that two adult mice breed and produce a litter of young. Also assume the two offspring are able to reproduce in one month. If all of the offspring survive to breed, the population grows slowly at first. This slow growth period is defined as the **lag phase**. The rate of population growth soon begins to increase rapidly because the total number of organisms that are able to reproduce has increased. After only two years, the experimental mouse population would reach more than three million mice.

Notice in Figure 4.7 that once the mice begin to reproduce rapidly, the graph becomes J-shaped. A J-shaped growth curve illustrates exponential growth.

Exponential growth, also called geometric growth, occurs when the growth rate is proportional to the size of the population. All populations grow exponentially until some limiting factor slows the population’s growth. It is important to recognize that even in the **lag phase**, the use of available resources is exponential. Because of this, the resources soon become limited and population growth slows.

Logistic growth model

Many populations grow like the model shown in Figure 4.8 rather than the model shown in Figure 4.7. Notice that the graphs look exactly the same through some of the time period. However, the second graph curves into an S-shape.

An **S-shaped curve** is typical of logistic growth. Logistic growth occurs when the population’s growth slows or stops following exponential growth, at the population’s carrying capacity. A population stops increasing when the number of births is less than the number of deaths or when emigration exceeds immigration.

Carrying capacity In Figure 4.8 on the previous page, notice that logistic growth levels off at the line on the graph identified as the carrying capacity. The maximum number of individuals in a species that an environment can support for the long term is the carrying capacity.

Carrying capacity is limited by the energy, water, oxygen, and nutrients available. When populations develop in an environment with plentiful resources, there are more births than deaths. The population soon reaches or passes the carrying capacity. As a population nears the carrying capacity, resources become limited. If a population exceeds the carrying capacity, deaths outnumber births because adequate resources are not available to support all of the individuals. The population then falls below the carrying capacity as individuals die. The concept of carrying capacity is used to explain why many populations tend to stabilize.

Reproductive patterns

The graph in Figure 4.8 shows the number of individuals increasing until the carrying capacity is reached. However, there are several additional factors that must be considered for real populations. Species of organisms vary in the number of births per reproduction cycle, in the age that reproduction begins, and in the life span of the organism. Both plants and animals are placed into groups based on their reproductive factors.

Members of one of the groups are called the r-strategists. The rate strategy, or r-strategy, is an adaptation for living in an environment where fluctuation in biotic or abiotic factors occur. Fluctuating factors might be availability of food or changing temperatures. An r-strategist is generally a small organism such as a fruit fly, a mouse, or the locusts shown in Figure 4.9. R-strategists usually have short life spans and produce many offspring.

The reproductive strategy of an r-strategist is to produce as many offspring as possible in a short time period in order to take advantage of some environmental factor. They typically expend little energy or none at all in raising their young to adulthood. Populations of r-strategists usually are controlled by density-independent factors, and they usually do not maintain a population near the carrying capacity.

Just as some environments fluctuate, others are fairly predictable.

Elephants on the savanna, shown in Figure 4.10, experience a carrying capacity that changes little from year to year. The carrying-capacity strategy, or k-strategy, is an adaptation for living in these environments. A k-strategist generally is a larger organism that has a long life span, produces few offspring, and whose population reaches equilibrium at the carrying capacity. The reproductive strategy of a k-strategist is to produce only a few offspring that have a better chance of living to reproductive age because of the energy, resources, and time invested in the care for the young. Populations of k-strategists usually are controlled by density-dependent factors.

Human Population

Human Population Growth

The study of human population size, density, distribution, movement, and birth and death rates is demography. The graph in Figure 4.11 shows demographers’ estimated human population on Earth for several thousand years.

Notice that the graph in Figure 4.11 shows a relatively stable number of individuals over thousands of years, until recently. Notice also the recovery of the human population after the outbreak of the bubonic plague in the 1300s when an estimated one-third of the population of Europe died. Perhaps the most significant feature in this graph is the increase in human population in recent times.

In 1804, the population of Earth was an estimated one billion people. By 1999, the human population had reached six billion people. At this growth rate, about 70 million people are added to the world population annually, and the world’s population is expected to double in about 53 years.

Technological advances

For thousands of years, environmental conditions kept the size of the human population at a relatively constant number below the environment’s carrying capacity. Humans have learned to alter the environment in ways that appear to have changed its carrying capacity.

Agriculture and domestication of animals have increased the human food supply. Technological advances and medicine have improved the chances of human survival by reducing the number of deaths from parasites and disease. In addition, improvements in shelter have made humans less vulnerable to climatic impact.

Human population growth rate

Although the human population is still growing, the rate of its growth has slowed. Figure 4.12 shows the percent increase in human population from the late 1940s through 2003. The graph also includes the projected population increase through 2050.

Notice the sharp dip in human population growth in the 1960s. This was due primarily to a famine in China in which about 60 million people died. The graph also shows that human population growth reached its peak at over 2.2 percent in 1962. By 2003, the percent increase in human population growth had dropped to almost 1.2 percent. Population models predict the overall population growth rate to be below 0.6 percent by 2050. The decline in human population growth is due primarily to diseases such as AIDS and voluntary population control.

Trends in Human Population Growth

The graph in Figure 4.12 is somewhat deceptive. Population trends can be altered by events such as disease and war. Figure 4.13 shows a few historical events that have changed population trends. Figure 4.12 could also easily be misinterpreted because human population growth is not the same in all countries. However, there are population growth trends in countries that have similar economies.

For example, one trend that has developed during the previous century is a change in the population growth rate in industrially developed countries such as the United States. An industrially developed country is a country that is advanced in industrial and technological capabilities and whose population has a high standard of living. In its early history, the United States had a high birthrate and a high death rate. It was not uncommon for people to have large families and for individuals to die by their early forties. Many children also died before reaching adulthood. Presently, the birthrate in the United States has decreased dramatically and the life expectancy is greater than seventy years. A change in a population from high birth and death rates to low birth and death rates is a demographic transition.

How do population growth rates compare in industrially developed countries and developing countries—countries with a relatively low level of capabilities and a low standard of living? In the United States, the birthrate in 2005 was 14.1 births per 1000 people, the death rate was 8.2 deaths per 1000 people, and the migration rate was 3.3 people entering the country per 1000 people. The population growth rate was 0.92 percent in the United States.

In a developing country such as Honduras—location shown in Table 4.1—the situation is different. In the same year, Honduras had a birthrate of 30.4 births per 1000 people, a death rate of 6.9 deaths per 1000 people, and a migration rate of 1.9 people leaving the country per 1000 people. This results in a population growth rate of 2.16 percent. This is among the highest population growth rates of any country in the world.

Developing countries will add 73 million people to the world population compared to only three million people added in the industrially developed countries. For example, between now and 2050, the developing country Niger—also shown in Table 4.1—is expected to be one of the most rapidly growing countries. Its population will expand from 12 to 53 million people. The industrially developed country Bulgaria is expected to have a population decline from eight to five million people in the same time period.

Zero population growth

Another trend that populations can experience is zero population growth. Zero population growth (ZPG) occurs when the birthrate equals the death rate. One estimate is that the world will reach ZPG between 2020 with 6.64 billion people and 2029 with 6.90 billion people. This will mean that the population has stopped growing, because births and deaths occur at the same rate. Once the world population reaches ZPG, the age structure eventually should be more balanced with numbers at pre-reproductive, reproductive, and post-reproductive ages being approximately equal.

Age structure

Another important characteristic of any population is its age structure. A population’s age structure is the number of male and females in each of three age groups: pre-reproductive stage, reproductive stage, and post-reproductive stage. Humans are considered to be pre-reproductive before age 20 even though they are capable of reproduction at an earlier age. The reproductive years are considered to be between 20 and 44, and the post-reproductive years are after age 44.

Analyze the age structure diagrams for three different representative countries in Figure 4.14—their locations are shown in Table 4.1. The age structure diagrams are typical of many countries in the world.

Notice the shape of the overall diagram for a country that is rapidly growing, one that is growing slowly, and one that has reached negative growth. The age structure for the world’s human population looks more like that of a rapidly growing country.

Human carrying capacity Calculating population growth rates is not just a mathematical exercise. Scientists are concerned about the human population reaching or exceeding the carrying capacity. As you learned in Section 4.1, all populations have carrying capacities, and the human population is no exception. Many scientists suggest that human population growth needs to be reduced. In many countries, voluntary population control is occurring through family planning.

Unfortunately, if the human population continues to grow—as most populations do—and areas become overcrowded, disease and starvation will occur. However, technology has allowed humans to increase Earth’s carrying capacity, at least temporarily. It might be possible for technology and planning to keep the human population at or below its carrying capacity.

Another important factor in keeping the human population at or below the carrying capacity is the amount of resources from the biosphere that are used by each person. Currently, individuals in industrially developed countries use far more resources than those individuals in developing countries, as shown in Figure 4.15. This graph shows the estimated amount of land required to support a person through his or her life, including land used for production of food, forest products and housing, and the additional forest land required to absorb the carbon dioxide produced by the burning of fossil fuels. Countries such as India are becoming more developed, and they have a high growth rate. These countries are adding more people and are increasing their use of resources. At some point, the land needed to sustain each person on Earth might exceed the amount of land that is available.

Biodiversity

What is biodiversity?

The loss of an entire species in a food web is not an imaginary situation. Entire species permanently disappear from the biosphere when the last member of the species dies in a process called extinction. As species become extinct, the variety of species in the biosphere decreases, which decreases the health of the biosphere. Biodiversity is the variety of life in an area that is determined by the number of different species in that area. Biodiversity increases the stability of an ecosystem and contributes to the health of the biosphere. There are three types of biodiversity to consider: genetic diversity, species diversity, and ecosystem diversity.

Genetic diversity

The variety of genes or inheritable characteristics that are present in a population comprises its genetic diversity. Figure 5.1 shows several characteristics that are shared by the ladybird beetles, such as general body structure. The variety of colors demonstrates a form of genetic diversity. The beetles have other characteristics that differ, but they are not as apparent as their color. These characteristics might include resistance to a particular disease, the ability to recover from a disease, or the ability to obtain nutrients from a new food source should the old food source disappear. The beetles with these characteristics are more likely to survive and reproduce than beetles without these characteristics.

Genetic diversity within interbreeding populations increases the chances that some species will survive during changing environmental conditions or during an outbreak of disease.

Species diversity

The number of different species and the relative abundance of each species in a biological community is called species diversity. As you look at Figure 5.2, notice how many different species of organisms are in this one area. This habitat represents an area with a high level of species diversity because there are so many species present in one location. However, species diversity is not evenly distributed over the biosphere. As you move geographically from the polar regions to the equator, species diversity increases. For example, Figure 5.3 shows the number of bird species from Alaska to Central America. Use the color key to see how diversity changes as you move toward the equator.

Ecosystem diversity

The variety of ecosystems that are present in the biosphere is called ecosystem diversity. Recall from Chapter 2 that an ecosystem is made up of interacting populations and the abiotic factors that support them. The interactions of organisms affect the development of stable ecosystems. Different locations around the world have different abiotic factors that support different types of life. For example, an ecosystem in Alaska has a set of abiotic factors that supports Dall sheep, which are shown in Figure 5.4. An ecosystem in Central America has a different set of abiotic factors that supports tropical birds, also shown in Figure 5.4. All of the ecosystems on Earth support a diverse collection of organisms.

The Importance of Biodiversity

There are several reasons to preserve biodiversity. Many humans work to preserve and protect the species on Earth for future generations. In addition, there are economic, aesthetic, and scientific reasons for preserving biodiversity.

Direct economic value

Maintaining biodiversity has a direct economic value to humans. Humans depend on plants and animals to provide food, clothing, energy, medicine, and shelter. Preserving species that are used directly is important, but it also is important to preserve the genetic diversity in species that are not used directly. Those species serve as possible sources of desirable genes that might be needed in the future.

The reason there might be a future need for desirable genes is that most of the world’s food crops come from just a few species. These plants have relatively little genetic diversity and share the same problems that all species share when genetic diversity is limited, such as lacking resistance to disease. In many cases, close relatives of crop species still grow wild in their native habitat. These wild species serve as reservoirs of desirable genetic traits that might be needed to improve domestic crop species.

The distant relative of corn shown on the left in Figure 5.5 is resistant to the viral diseases that damage commercial corn crops. Using this wild species, plant pathologists have developed disease-resistant corn varieties. If this wild species had not been available, this genetic diversity would have been lost, and the ability to develop disease-resistant corn varieties would have been lost as well.

In addition, biologists are beginning to learn how to transfer genes that control inherited characteristics from one species to the other. This process, sometimes referred to as genetic engineering, is discussed in Chapter 13. Crops have been produced that are resistant to some insects, that have increased nutritional value, and that are more resistant to spoilage. Most wild species of plants and animals have not been evaluated for useful genetic traits. The opportunity to benefit from these genes is lost forever if wild species of plants and animals become extinct. This increases the importance of species that currently have no perceived economic value because their economic value might increase in the future.

Many of the medicines that are used today are derived from plants or other organisms. You probably know that penicillin, a powerful antibiotic discovered in 1928 by Alexander Fleming, is derived from bread mold. Ancient Greeks, Native Americans, and others extracted salicin, a painkiller, from the willow tree. Today, a version of this drug is synthesized in laboratories and is known as aspirin. Figure 5.6 shows a Madagascar periwinkle flower, which recently was found to yield an extract that is useful in treating leukemia. This extract has been used to develop drugs that have increased the survival rate for leukemia patients from 20 percent to more than 95 percent.

Scientists continue to find new extracts from plants and other organisms that help in the treatment of human diseases. However, many species of organisms are yet to be identified, especially in remote regions of Earth, so their ability to provide extracts or useful genes is unknown.

Indirect economic value

A healthy biosphere provides many services to humans and other organisms that live on Earth. For example, green plants provide oxygen to the atmosphere and remove carbon dioxide. Natural processes provide drinking water that is safe for human use. Substances are cycled through living organisms and nonliving processes, providing nutrients for all living organisms. As you will soon learn, healthy ecosystems provide protection against floods and drought, generate and preserve healthful fertile soils, detoxify and decompose wastes, and regulate local climates.

It is difficult to attach an economic value to the services that a healthy biosphere provides. However, some scientists and economists have attempted to do just that. In the 1990s, New York City was faced with the decision of how to improve the quality of its drinking water. A large percentage of New York City’s drinking water was supplied by watersheds, shown in Figure 5.7. Watersheds are land areas where the water on them or the water underneath them drains to the same place. The Catskill and Delaware watersheds did not meet clean water standards and no longer could supply quality drinking water to the city.

The city was faced with two choices: build a new water filtration system for more than $6 billion or preserve and clean up the watersheds for approximately 1.5 billion dollars. The economic decision was clear in this case. A healthy ecosystem was less expensive to maintain than using technology to perform the same services.

This example shows that nature can provide services, such as water that is safe for human consumption, at less expense than using technology to provide the same service. Some scientists believe the natural way should be the first choice for providing these services. Research indicates that when healthy ecosystems are preserved, the services the ecosystems provide will continue to be less expensive than performing the same services with technology.

Aesthetic and scientific value

Two additional considerations for maintaining biodiversity and healthy ecosystems are the aesthetic and scientific values that they provide. It is difficult to attach a value to something that is beautiful, such as the ecosystem shown in Figure 5.8, or something that is interesting to study. Perhaps it is best to consider how life would be if all that was present on Earth was a barren and desolate landscape. The value of biodiversity and healthy ecosystems would be more obvious to us then.

Threats to Biodiversity

Extinction Rates

Many species have become extinct and paleontologists study fossils of those extinct species today. The gradual process of species becoming extinct is known as background extinction. Stable ecosystems can be changed by the activity of other organisms, climate changes, or natural disasters. This natural process of extinction is not what scientists are worried about. Many worry about a recent increase in the rate of extinction. Some scientists predict that between one-third and two-thirds of all plant and animal species will become extinct during the second half of this century. Most of these extinctions will occur near the equator.

Some scientists estimate the current rate of extinction is about 1000 times the normal background extinction rate. These scientists believe that we are witnessing a period of mass extinction. Mass extinction is an event in which a large percentage of all living species become extinct in a relatively short period of time. The last mass extinction occurred about 65 million years ago, as illustrated in Table 5.1, when the last of the surviving dinosaurs became extinct.

The accelerated loss of species began several centuries ago. Table 5.2 shows the estimated number of extinctions that have occurred by group since 1600. Many of the species’ extinctions in the past have occurred on islands. For example, 60 percent of the mammals that have become extinct in the past 500 years lived on islands, and an 81 percent of bird extinctions occurred on islands.

Species on islands are particularly vulnerable to extinction because of several factors. Many of these species evolved without the presence of natural predators. As a result, when a predator, such as a dog, cat, rat, or human, is introduced to the population, the native animals do not have the ability or skills to escape. When a nonnative species is introduced to a new population, it can be a carrier of a disease to which the native population has no resistance. The native population often dies off as a result. In addition, islands typically have relatively small population sizes and individual animals rarely travel between islands, which increases the vulnerability of island species to extinction.

Factors that Threaten Biodiversity

Scientists point out that today’s high rate of extinction differs from past mass extinctions. The current high rate of extinction is due to the activities of a single species—Homo sapiens. After a mass extinction in the past, new species evolved, and biodiversity recovered after several million years. This time, the recovery might be different. Humans are changing conditions on Earth faster than new traits can evolve to cope with the new conditions. Evolving species might not have the natural resources they need. Natural resources are all materials and organisms found in the biosphere, including minerals, fossil fuels, nuclear fuels, plants, animals, soil, clean water, clean air, and solar energy.

Overexploitation

One of the factors that is increasing the current rate of extinction is the overexploitation, or excessive use, of species that have economic value. For example, the great herds of bison that once roamed the central plains of North America were hunted to the brink of extinction because their meat and hides could be sold commercially and because they were hunted for sport. At one time, it is estimated that there were 50 million bison. By 1889, there were less than 1000 bison left.

Passenger pigeons are another example of a species that has been overexploited. At one time, there were huge flocks of these birds that would darken the skies of North America during their migration.

Unfortunately, they were overhunted and forced from their habitats. By the early 1900s, they had become extinct.

The ocelot, shown in Figure 5.9, is found from Texas to Argentina and is in danger of becoming extinct. The increasing loss of their habitat and the commercial value of their fur are reasons for their declining numbers. The white rhinoceros, also shown in Figure 5.9, is one of five species of rhinos, all of which are in danger of becoming extinct. They are hunted and killed for their horns, which are then sold for medicinal purposes. Historically, overexploitation was the primary cause of species extinction. However, the number one cause of species extinction today is the loss or destruction of habitat.

Habitat loss

There are several ways that species can lose their habitats. If a habitat is destroyed or disrupted, the native species might have to relocate or they will die. For example, humans are clearing areas of tropical rain forests and are replacing the native plants with agricultural crops or grazing land.

Destruction of habitat

The clearing of tropical rain forests, like what is shown in Figure 5.10, has a direct impact on global biodiversity. As mentioned earlier, the tropical latitudes contain much of the world’s biodiversity in their native populations. In fact, estimates show that more than half of all species on Earth live in the tropical rain forests. The removal of so much of the natural forest will cause many species on Earth to become extinct because of habitat loss.

Disruption of habitat Habitats might not be destroyed, but they can be disrupted. For example, off the coast of Alaska, a chain of events occurred in the 1970s that demonstrates how the declining numbers of one member of a food web can affect the other members. As you can see from the chain of events shown in Figure 5.11, the decline of one species can affect an entire ecosystem. When one species plays such a large role in an ecosystem, that species is called a keystone species. A decline in various fish populations, possibly due to overfishing, has led to a decline in sea lion and harbor seal populations. Some scientists hypothesize that global warming also played a role in the decline. This started a chain reaction within the marine ecosystem that affected many species.

Fragmentation of habitat

The separation of an ecosystem into small pieces of land is called habitat fragmentation. Populations often stay within the confines of the small parcel because they are unable or unwilling to cross the human-made barriers. This causes several problems for the survival of various species.

First, the smaller the parcel of land, the fewer species it can support. Second, fragmentation reduces the opportunities for individuals in one area to reproduce with individuals from another area. For this reason, genetic diversity often decreases over time in habitat fragments.

Smaller, separated, and less genetically diverse populations are less able to resist disease or respond to changing environmental conditions.

Third, carving the large ecosystem into small parcels increases the number of edges—creating edge effects, as illustrated in Figure 5.12. **Edge effects** are different environmental conditions that occur along the boundaries of an ecosystem. For example, edges of a forest near a road have different abiotic factors, such as temperature, wind, and humidity, than the interior of a forest. Typically, the temperature and wind will be higher and the humidity lower on the edges in a tropical forest. Species that thrive deep in the dense forest might perish on the edges of the ecosystem. Predators and parasites also thrive on the boundaries of ecosystems, which makes the species in these areas more vulnerable to attack. **Edge effects** do not always create a disadvantage for all species. Some species find these conditions favorable and they thrive.

Pollution

Pollution and atmospheric changes threaten biodiversity and global stability. Pollution changes the composition of air, soil, and water. There are many types of pollution. Substances—including many human-made chemicals that are not found in nature—are released into the environment. Pesticides, such as DDT (dichloro-diphenyl-trichloroethane), and industrial chemicals, such as PCBs (polychlo-rinated biphenyls), are examples of substances that are found in food webs. These substances are ingested by organisms when they drink water or eat other organisms that contain the toxic substance. Some substances are metabolized by the organism and excreted with other waste products. However, other substances, such as DDT and PCBs, accumulate in the tissues of organisms.

Carnivores at the higher trophic levels seem to be most affected by the accumulation because of a process called biological magnification. Biological magnification is the increasing concentration of toxic substances in organisms as trophic levels increase in a food chain or food web, as shown in Figure 5.13. The concentration of the toxic substance is relatively low when it enters the food web. The concentration of toxic substance in individual organisms increases as it spreads to higher trophic levels.

Current research implies that these substances might disrupt normal processes in some organisms. For example, DDT might have played a role in the near extinction of the American bald eagle and the peregrine falcon. DDT is a pesticide that was used from the 1940s to the 1970s to control crop-eating and disease-carrying insects. DDT proved to be a highly effective pesticide, but evidence suggested that it caused the eggshells of fish-eating birds to be fragile and thin, which led to the death of the developing birds. Once these toxic effects were discovered, the use of DDT was banned in some parts of the world.

Acid precipitation

Another pollutant that is affecting biodiversity is acid precipitation. When fossil fuels are burned, sulfur dioxide is released into the atmosphere. In addition, the burning of fossil fuels in automobile engines releases nitrogen oxides into the atmosphere.

These compounds react with water and other substances in the air to form sulfuric acid and nitric acid. These acids eventually fall to the surface of Earth in rain, sleet, snow, or fog. Acid precipitation removes calcium, potassium, and other nutrients from the soil, depriving plants of these nutrients. It damages plant tissues and slows their growth, as shown in Figure 5.14. Sometimes, the acid concentration is so high in lakes, rivers, and streams that fish and other organisms die, also as shown in Figure 5.14.

Eutrophication Another form of water pollution, called eutrophication, destroys underwater habitats for fish and other species. Eutrophication occurs when fertilizers, animal waste, sewage, or other substances rich in nitrogen and phosphorus flow into waterways, causing extensive algae growth. The algae use up the oxygen supply during their rapid growth and after their deaths during the decaying process. Other organisms in the water suffocate. In some cases, algae also give off toxins that poison the water supply for other organisms. Eutrophication is a natural process, but human activities have accelerated the rate at which it occurs.

Introduced species Nonnative species that are either intentionally or unintentionally transported to a new habitat are known as introduced species. These species are not a threat to biodiversity in their native habitats. Predators, parasites, and competition between species keep the native ecosystem in balance. However, when these species are introduced into a new area, these controlling factors are not in place. Introduced species often reproduce in large numbers because of a lack of predators, and become invasive species in their new habitat.

Imported fire ants are a species that is believed to have been introduced to the United States through the port of Mobile, Alabama in the 1920s by ships from South America. The fire ants spread throughout the southern and southwestern United States, as illustrated in Figure 5.15. Fire ants attack and feed on some wildlife, such as newborn deer and hatching or newly-hatched ground-nesting birds.

Introduced species are a worldwide environmental problem. An estimated 40 percent of the extinctions that have occurred since 1750 are due to introduced species, and billions of dollars are spent every year in an effort to clean up or control the damage caused by introduced species.

Conserving Biodiversity

Natural Resources

The biosphere currently supplies the basic needs for over six billion humans in the form of natural resources. The human population continues to grow and the growth is not evenly distributed throughout the world. An increase in human population growth increases the need for natural resources to supply the basic needs of the population.

The consumption rate of natural resources also is not evenly distributed. Figure 5.16 shows the consumption of natural resources per person for selected countries. The natural resource consumption rate is much higher for people living in developed countries than for people in developing countries. As developing countries become more industrialized and the standard of living increases, the rate of natural resource consumption also increases. Because of the rising human population growth and an increased rate of consumption of natural resources, a long-term plan for the use and conservation of natural resources is important.

Renewable resources Plans for long-term use of natural resources must take into consideration the difference between the two groups of natural resources—renewable and nonrenewable resources. Those resources that are replaced by natural processes faster than they are consumed are called renewable resources. Solar energy is considered a renewable resource because the supply appears to be endless. Agricultural plants, animals, clean water, and clean air are considered renewable because normally they are replaced faster than they are consumed. However, the supply of these resources is not unlimited. If the demand exceeds the supply of any resource, the resource might become depleted.

Renewable v. nonrenewable resources

Those resources that are found on Earth in limited amounts or those that are replaced by natural processes over extremely long periods of time are called nonrenewable resources. Fossil fuels and mineral deposits, such as radioactive uranium, are considered nonrenewable resources. Species are considered renewable resources until the last of a species dies. When extinction occurs, a species is nonrenewable because it is lost forever.

The classification of a resource as renewable or nonrenewable depends on the context in which the resource is being discussed. A single tree or a small group of trees in a large forest ecosystem is renewable because replacement trees can be planted or can regrow from seeds present in the soil. Enough of the forest is still intact to serve as a habitat for the organisms that live there. However, when the entire forest is cleared, as shown in Figure 5.17, the forest is not considered a renewable resource. The organisms living in the forest have lost their habitat and they most likely will not survive. In this example, it is possible that more than one natural resource is nonrenewable—the forest and any species that might become extinct. If a species is found only in this forest, this species might become extinct if it loses its only habitat.

Sustainable use

One approach to using natural resources called sustainable use is demonstrated in Figure 5.18. Just as the name implies, sustainable use means using resources at a rate in which they can be replaced or recycled while preserving the long-term environmental health of the biosphere. Conservation of resources includes reducing the amount of resources that are consumed, recycling resources that can be recycled, and preserving ecosystems, as well as using them in a responsible manner.

Protecting Biodiversity

In Section 2, you learned how human activities have affected many ecosystems. Many efforts are underway worldwide to slow the loss of biodiversity and to work toward sustainable use of natural resources.

Protected areas in the United States Conservation biologists recognize the importance of establishing protected areas where biodiversity can flourish. The United States established its first national park—Yellowstone National Park—in 1872 to protect the area’s geological features. Many additional national parks and nature reserves have been established since 1872.

International protected areas The United States is not the only country to establish national parks or nature reserves. Currently, about seven percent of the world’s land is set aside as some type of reserve. Historically, these protected areas have been small islands of habitat surrounded by areas that contain human activity. Because the reserves are small, they are impacted heavily by human activity. The United Nations supports a system of Biosphere Reserves and World Heritage sites. Costa Rica has established megareserves. These reserves contain one or more zones that are protected from human activity by buffer zones—an area in which sustainable use of natural resources is permitted. This approach creates a large managed area for preserving biodiversity while providing natural resources to the local population.

Biodiversity hot spots Conservation biologists have identified locations around the world that are characterized by exceptional levels of endemic species—species that are only found in that specific geographic area—and critical levels of habitat loss. To be called a hot spot, a region must meet two criteria. First, there must be at least 1500 species of vascular plants that are endemic, and the region must have lost at least 70 percent of its original habitat. The 34 internationally recognized hot spots are shown in Figure 5.19.

Approximately half of all plant and animal species are found in hot spots. These hot spots originally covered 15.7 percent of Earth’s surface, however, only about a tenth of that habitat remains.

Biologists in favor of recovery efforts in these areas argue that focusing on a limited area would save the greatest number of species. Other biologists argue that concentrating funding on saving species in these hot spots does not address the serious problems that are occurring elsewhere. For example, saving a wetland area might save fewer species, but the wetland provides greater services by filtering water, regulating floods, and providing a nursery for fish. These biologists think that funding should be spent in areas around the world rather than focused on the biodiversity hotspots.

Corridors between habitat fragments Conservation ecologists also are focusing on improving the survival of biodiversity by providing corridors, or passageways, between habitat fragments. Corridors, such as those shown in Figure 5.20, are used to connect smaller parcels of land. These corridors allow organisms from one area to move safely to the other area. This creates a larger piece of land that can sustain a wider variety of species and a wider variety of genetic variation. However, corridors do not completely solve the problem of habitat destruction. Diseases easily pass from one area to the next as infected animals move from one location to another. This approach also increases edge effect. One large habitat would have fewer edges, but often a large habitat is hard to preserve.

Restoring Ecosystems

Sometimes biodiversity is destroyed in an area such that it no longer provides the abiotic and biotic factors needed for a healthy ecosystem. For example, the soil from cleared tropical rain forests becomes unproductive for farming after a few years. After mining activities are completed, the land might be abandoned in a condition that does not support biodiversity. Accidental oil spills and toxic chemical spills might pollute an area to such a degree that the native species cannot live there.

Given time, biological communities can recover from natural and human-made disasters, as illustrated in Figure 5.21. The length of time for recovery is not related directly to whether the disaster is natural or human-made.

The size of the area affected and the type of disturbance are determining factors for recovery time. In general, the larger the affected area, the longer it takes for the biological community to recover. Ecologists use two methods to speed the recovery process of these damaged ecosystems— bioremediation and biological augmentation.

Bioremediation The use of living organisms, such as prokaryotes, fungi, or plants, to detoxify a polluted area is called bioremediation. In 1975, a leak from a fuel-storage facility in South Carolina released about 80,000 gallons of kerosene-based jet fuel. The fuel soaked into the sandy soil and contaminated the underground water table. Microorganisms that naturally are found in the soil break down these carbon-based fuels into carbon dioxide. Scientists found that by adding additional nutrients to the soil, the rate at which the microorganisms decontaminated the area was increased. In a few years, the contamination in the area had been greatly reduced. These microorganisms can be used in other ecosystems to remove toxins from soils that are contaminated by accidental oil or fuel spills.

Some species of plants are being used to remove toxic substances, such as zinc, lead, nickel, and organic chemicals, from damaged soils, as shown in Figure 5.22. These plants are planted in contaminated soils, where they store the toxic metals in their tissues. The plants then are harvested, and the toxic metals are removed from the ecosystem. Bioremediation is relatively new, but there appears to be great promise in using organisms to detoxify some ecosystems that have been damaged.

Biological augmentation

Adding natural predators to a degraded ecosystem is called biological augmentation.

For example, aphids—very small insects—eat vegetables and other plants, which can result in the destruction of farm crops. Aphids also can transmit plant diseases. Some farmers rely on ladybugs to control pests that eat their crops. Certain species of ladybugs eat aphids, as shown in Figure 5.23, and can be used to control aphid infestation.

The ladybugs do not harm the crops, and the fields are kept free of aphids.

Legally Protecting Biodiversity

During the 1970s, a great deal of attention was focused on the destruction to the environment and maintaining biodiversity. Laws were enacted in countries around the world and many treaties between countries were signed in an effort to preserve the environment. In the United States, the Endangered Species Act was enacted in 1973. It was designed to protect legally the species that were becoming extinct or in danger of becoming extinct. An international treaty, the Convention on International Trade in Endangered Species of Wild Fauna and Flora (CITES), was signed in 1975. It outlawed the trade of endangered species and animal parts, such as ivory elephant tusks and rhinoceros horns. Since the 1970s, many more laws and treaties have been enacted and signed with the purpose of preserving biodiversity for future generations.
