# Supplementary material for: A semantic classification of nominal technical terms in secondary school biology textbooks
Source: PLoS One. 2024 Nov 11;19(11):e0312040. doi: 10.1371/journal.pone.0312040 (PMC11554214; doi:10.1371/journal.pone.0312040)
Supplement: S3 File — (DOCX) [file pone.0312040.s005.docx]

**Textbook 1: NSW Oxford Insight Science**

Chapter 1 Genetics and biotechnology

1.1 DNA and genetic code

THE STRUCTURE OF DNA

Deoxyribonucleic acid (DNA) exists within all living cells and is like a blueprint for every structure and function in an organism. It contains a code unique to the individual that can be passed to offspring, generation after generation. The DNA molecules of all living things on the Earth have the same general structure, but each species has its own unique DNA that defines the species. There are also slight differences in the DNA between individuals within a species, which is why we are all unique. Understanding the structure of DNA enables us to explain not only the similarities that exist between and within species, but also the differences.

Watson and Crick

James Watson and Francis Crick (Figure 1.1) worked together at the University of Cambridge in the United Kingdom during the early 1950s. Watson was a young chemist from the United States and Crick was a physicist from the United Kingdom. As a team they unraveled the secret of the DNA structure; however, they performed no experiments themselves. Instead, their talent lay in interpreting secondary data – the experimental results of others.

There was a lot of information available about DNA when Watson and Crick began their investigation. This information included the following:

•DNA was known to be a large, long, thin molecule composed of units called nucleotides containing the nitrogenous bases adenine (A), guanine (G), cytosine (C) and thymine (T).

•Russian biochemist Phoebus Levene thought the four bases were arranged in a fixed, repeating pattern.

•American biochemist Linus Pauling’s work on proteins led him to believe that DNA had a helical or spiral structure.

•X-ray crystallography pictures of DNA (Figure 1.2b) from the laboratories of Maurice Wilkins and Rosalind Franklin of King’s College, London, showed markings that almost certainly indicated the turns of a giant helix.

•American biochemist Erwin Chargaff’s data indicated that the amount of adenine equalled the amount of thymine, and that the amount of guanine equalled the amount of cytosine.

Watson and Crick tried to put all this information together and build a model of the structure of DNA. In February 1953 they completed their model, which established the now familiar structure of DNA – the double helix.

The double helix

DNA is a type of nucleic acid. Nucleic acids are specific types of chemical, like proteins, lipids or carbohydrates. There are two main types of nucleic acids: DNA and RNA (ribonucleic acid). Nucleic acids are polymers, which are chemical structures made up of repeating units (nucleotides in this case). The Watson–Crick model of DNA as a double-helix structure can be likened to a ladder twisted into a spiral.

NUCLEOTIDES

A nucleotide is the basic unit of nucleic acids. Nucleotides are complex molecules made up of three components: •a nitrogenous base •a five-carbon sugar •a phosphate group.

DNA contains the sugar deoxyribose and the four bases adenine (A), guanine (G), cytosine (C) and thymine (T).

Nucleotides are joined together by their sugar and phosphate groups. This forms a sugar–phosphate backbone. The bases are attached to the sugars and are at right angles to the sugar–phosphate backbone, like the rungs in a ladder. Any number of nucleotides can join together, in any order, to form the nucleic acid polymer, or polynucleotide chain.

One of the greatest achievements of Watson and Crick was their realisation that bases on one polynucleotide strand are bonded to bases on the other strand, with A always bonding with T, and G always bonding with C. The implications of the results of Chargaff’s research, with

DNA containing similar percentages of A and T, and C and G, were suddenly obvious. These base pairs are called complementary bases or complementary base pairs.

Two polynucleotide chains, or strands, are attracted to each other due to the chemical nature of the complementary bases. This is important for the easy ‘unzipping’ of the ladder during replication. A large base (adenine or guanine) is always bonded to a small base (thymine or cytosine) because this gives a consistent amount of space between the strands.

The two chains then wind into a double helix.

RNA is a single-stranded polynucleotide with a similar sugar–phosphate backbone to that of DNA. However, RNA contains the sugar ribose and the four bases adenine (A), guanine (G), cytosine (C) and uracil (U). RNA plays a key role in protein synthesis.

Genes and chromosomes

DNA is located in the nucleus of organisms, where it looks a little like a tangled pile of cotton threads. Theoretically, the polynucleotide strands can be infinitely long, but they actually form specific lengths called chromosomes. The number of chromosomes per cell and the length of each chromosome vary between species. However, the number of chromosomes is not necessarily an indication of organism complexity. Table 1.1 shows the number of chromosomes of a variety of different organisms.

The number of chromosomes per species is always an even number because chromosomes exist in homologous pairs. During sexual reproduction, each parent provides one set of chromosomes. The chromosomes are paired according to their length, which is determined by the genes they carry. A gene is a length of DNA that has a specific sequence of base pairs and codes for a particular characteristic. For example, a gene may have the information for making the melanin (a pigment that gives colour to your skin), for making insulin, or even for determining shape of your hairline. A single chromosome may have hundreds or even thousands of genes.

Chromosomes can be extracted from a cell, stained and photographed through a digital microscope. The image is called a karyotype and is used to identify chromosomal abnormalities.

REPLICATION AND CELL DIVISION

The genes that make up chromosomes hold the code for every characteristic of an organism. A full set of these blueprints is present in almost every cell of a multicellular organism. The few exceptions include red blood cells, which do not have a nucleus, and gametes (sperm and ova), which only have one set of chromosomes rather than pairs.

Two forms of cell division create new cells in a multicellular organism: mitosis and meiosis. The process and purpose of these two types of division are different, but they both begin by copying the cell’s DNA so each new cell contains the correct number of chromosomes.

Replication of DNA

Watson and Crick determined that, because of the double-stranded structure of DNA and complementary base pairing, DNA could make copies of itself with a process called replication.

With the aid of several specific enzymes, the hydrogen bonds between bases are broken in sections so that the strands separate and expose the bases. Spare nucleotides that are floating around in the nucleus are added in a complementary sequence to the exposed strands until two full new strands have been completed.

Replication is the first stage of both mitosis and meiosis, and every chromosome is replicated. After replication, the doubled chromosomes are still attached at a point called the centromere and the chromosomes often look like an ‘X’. Chromosomes are commonly drawn like this because it is the stage at which they are most easily visible under a microscope. Each strand of the doubled chromosome is called a chromatid.

Key things to remember about replication:

•Before replication: single chromosome = 1 molecule of DNA (double-stranded double helix) = 1 copy of every chromosome

•After replication: doubled chromosome = 2 molecules of DNA = 2 chromatids joined at the centromere = 2 copies of every chromosome.

Mitosis

The nuclei of somatic (body) human cells contain 46 chromosomes arranged into 22 homologous pairs and one pair of sex chromosomes, which determine the gender of the individual. We can also say that the somatic cells are diploid (two sets of chromosomes in homologous pairs). One member of each pair has come from the female parent and the other from the male parent via the gametes.

Mitosis is the process of replicating exact copies of somatic cells. Mitosis was first introduced on page 118 of Oxford Insight Science 7 and is discussed further on page 62 of Oxford Insight Science 8. You may recall that mitosis is the cell division used for growth and repair in multicellular organisms, and is a form of asexual reproduction (binary fission) in unicellular organisms. In mitosis, the original parent cell divides once into two identical daughter cells. Every cell in your body, except gametes (the sex cells), is produced by mitosis.

Meiosis

A gamete is a sex cell. In animals, the male gamete is a sperm and the female gamete is an ovum. In flowering plants, the male gamete is contained in a pollen grain and the female ovum is located in the flower’s ovary. The male and female gametes of the same species join to form the first cell of the offspring, the zygote.

Gametes differ from all other body cells because they contain half the number of chromosomes of body cells – they are haploid (one set). Human gametes have 23 individual chromosomes that are not in pairs.

Meiosis is the process by which diploid cells are converted into haploid gametes and it occurs in the gonads of multicellular, sexually reproducing organisms. In humans, it occurs in the testes in males and ovaries in females.

Meiosis is sometimes called reduction division. It involves a growth and preparation phase called **interphase**, and then two separate division events known as meiosis I and meiosis II. Essentially, meiosis I separates the homologous pairs of chromosomes and meiosis II separates the chromatids.

The only purpose of a gamete is to fuse or join with another gamete to make a new cell called a zygote. This fusion of gametes is called fertilisation. The zygote will be diploid because it contains two sets of chromosomes, one from each gamete. Therefore, the original number of chromosomes is restored again in the body cells of the new organism (offspring).

Sperm production

In human males, meiosis and the formation of gametes occurs in the testes (singular: testis), specifically inside the seminiferous tubules.

In adult males, these very fine tubes may have a combined length of 300–500 metres per testis.

Spermatagonia are the starting cells of the process of sperm production, and are found in the lining of the seminiferous tubules.

They divide once by mitosis to produce two diploid cells; one daughter cell remains a spermatagonium to repeat the process, and the other becomes a primary spermatocyte.

The primary spermatocyte then undergoes meiosis to produce four haploid spermatids.

These cells grow tails, develop into sperm cells and are released into the seminiferous tubule. The immature sperm move to the epididymis where they are stored until they mature. By the time they leave the testis and move into the vas deferens, the sperm are fully motile (able to swim by themselves) and capable of fertilising an ovum.

The production of sperm is a continuous process. Mitosis of spermatagonia ensures there are always cells that can produce new sperm.

Ova production

Female gametes begin their development as primordial follicles (gamete starting cells) in the ovaries (singular: ovary). Unlike human males, who can produce sperm continuously, human females possess all their potential ova at birth (around 1–2 million). The primordial follicle is made up of an oocyte (the undeveloped ovum) and a protective layer of granulosa cells. Most primordial follicles never develop or mature. Because the oocyte is unable to repair itself, most die before puberty, leaving only 300 000–400 000 potential ova with around another 1000 or so dying every month after that.

If hormones trigger the primordial follicle to develop, it increases in size and grows protective layers of cells through mitosis. When the follicle is matured, the first division of meiosis takes place. However, cytokinesis divides the cells so that the vast majority of the cytoplasm ends up in the oocyte. The smaller cell dies.

During ovulation (the release of an ovum from the ovary), the mature follicle ruptures and releases the oocyte and its protective layer of cells into the fallopian tube. The second division of meiosis does not take place unless a sperm cell penetrates the protective layer and combines with the oocyte. The second division separates the chromatids of the doubled chromosomes and cytokinesis forms a tiny cell and a large ovum. Again, the smaller cell dies. The haploid ovum accepts the DNA from the sperm, fertilisation is achieved and a diploid zygote is formed.

On average, females release around 400 oocytes in their lifetime. Perhaps only one or two will be fertilised and develop into a foetus.

MUTATION

When cells divide, exact copies of every chromosome are usually produced because of the double-stranded structure of DNA and the complementary pairing of bases. The chromosome copies are generally distributed evenly between daughter cells during the process of mitosis. However, nature is not perfect all the time. Mistakes are made during both replication and cell division. These mistakes are called mutations. Mutations can involve individual genes or entire chromosomes.

The genetic code

A gene mutation is a change to the sequence of bases within a gene and usually happens during replication. Most of these mutations are detected and fixed by enzymes (specialised ‘helper’ proteins that act as proofreaders). The impact of these mutations varies depending on the nature of the change to the genetic code.

The genetic code is the sequence of bases in a gene and provides the specific instructions for the synthesis of proteins. Some proteins are the building material for major structures and organelles within the cell, whereas others are functional proteins such as enzymes.

Proteins are polymers made up of polypeptides, which are chains of amino acids. There are only 20 amino acids that occur naturally in human proteins, but they can be used in millions of different combinations. Each amino acid requires a specific code, which is made up of the DNA bases A, T, C and G.

Protein synthesis

Protein synthesis is a multi-step process where the relevant section (the gene) of DNA is copied into RNA. The short strands of RNA leave the nucleus and go to a ribosome where they form a template for an amino acid chain.

Individual amino acids are coded for by a triplet of bases called a codon. The four bases can be arranged as triplets in 4 × 4 × 4 = 64 different ways. This is more than enough for 20 amino acids. Hence, there is usually more than one codon for each amino acid. The sequence of amino acids in a protein is coded for by the sequence of codons along the DNA molecule.

The codons for the amino acids are typically listed using RNA rather than DNA. All proteins start with the amino acid methionine, which is coded for by the ‘start’ codon AUG.

Mutagens

The base sequence (order of bases) in DNA is critical. A tiny change in the sequence may alter the amino acids being coded for, which can change the protein produced and may affect the normal functioning of the organism. Although the aim of replication is to preserve the base sequence, occasional errors, or mutations, can occur. On most occasions these mutations can be corrected. Sometimes a mutation will result in a new codon that still codes for the same amino acid, or the change is not in an important part of the DNA. But on other occasions, mutations can change the protein produced, which can cause problems and can even be fatal.

Natural, spontaneous mutations occur continuously at a low rate. However, environmental factors called mutagens can increase the frequency of mutations. Mutagens include chemicals, radiation and ultraviolet (UV) light.

Somatic mutations occur during mitosis of body cells. The effect is localised to that individual and may lead to an illness such as cancer. Germ-line mutations occur during meiosis and the formation of gametes. These mutations do not affect the individual, but are passed on to their children if that gamete is fertilised. Germ-line mutations are said to heritable because they can be passed on or ‘inherited’ by offspring.

Genetic mutations

Genetic mutations only affect individual genes. There are three possible outcomes of genetic mutations:

•Sequence still codes for the same amino acids (some amino acids have more than one code) so there is no change to the polypeptide.

•Sequence codes for at least one different amino acid, which alters the structure and function of the polypeptide to varying degrees.

•Sequence is changed to include an earlier stop codon, shortening the polypeptide and often significantly altering its structure and function.

Chromosomal mutations

Chromosomal mutations are classified according to whether they change the structure of chromosomes or alter the number of chromosomes in the cell. Chromosomal mutations are often identified by their karyotype.

Mutations involving chromosome number

Mutations that alter chromosome numbers are usually the result of a homologous pair of chromosomes failing to separate during meiosis. In such cases, one of the daughter cells (gametes) will have too many chromosomes and the other will have too few. If an abnormal gamete is fertilised, the offspring will have either too many or too few chromosomes.

Down syndrome is the result of a person having three copies of chromosome 21 (trisomy 21).

1.2 Genetic inheritance

THE FATHER OF GENETICS

Gregor Mendel was an Austrian monk whose hobby was performing scientific experiments on the pea plants in the monastery garden. Most of his experimental work was done in the 1850s, a few decades before the discovery of DNA and 100 years before the structure and genetic code of DNA had been identified. Mendel’s success in obtaining reliable results to analyse and make predictions was because he:

•studied a large number of characteristics in the plants

•carried out a large number of crosses

•used pure breeding lines.

Most of Mendel’s conclusions based on his results still hold true today, even with all the additional information we now know about genetics. Without the current technology, Mendel may not have been able to explain exactly what was going on inside the cells, but he determined the ways in which parents pass on their characteristics to their offspring. Mendel is often referred to as the father of modern genetics.

Mendel’s experiments

Mendel noticed that although the pea plants in his garden were of the same species and successfully bred together to produce healthy, fertile offspring, they were not all the same. Some plants had white flowers, whereas others had purple flowers; some plants produced round peas, whereas others produced wrinkled peas; and so on. These differences between individuals of the same species are called variations. Mendel’s experiments were largely to determine how these variations were passed from one generation to the next.

Mendel began by establishing pure or true- breeding lines for seven different pea plant characteristics that typically only appeared in two different forms. True-breeding organisms are genetically identical to their parents and will always produce genetically identical offspring when interbred.

Mendel would then take two true-breeding plants with different forms of the same characteristic and breed them together to determine which form the characteristic would take in the next few generations of offspring.

For example, Mendel studied the characteristic of pea seed shape: either round (spherical) or wrinkled.

1 Mendel bred two groups of true-breeding pea plants: one with round seeds and the other with wrinkled seeds. These were called the P or parental generation.

2 Mendel crossed the true-breeding plants for round seeds with the true-breeding plants for wrinkled seeds. He did this by manually transferring the pollen grains from one flower to another. The offspring were all round seeded pea plants. They were known as the F1 or first generation.

3 Mendel then allowed the round F1 plants to interbreed to produce an F2 generation. In the F2 generation, Mendel’s crosses resulted in about 296 round to 103 wrinkled seeds, which is roughly ¾ round and ¼ wrinkled seeds, also expressed as a ratio of 3:1.

Basic principles of genetics

Mendel’s explanations about the results of his experiments were amazingly accurate. With no microscope work and very few colleagues with whom to discuss the results, he made the following conclusions.

•There must be ‘factors’ inside cells that control characteristics. Mendel’s ‘factors’ were later renamed genes.

•Two copies of each ‘factor’ are present in every cell and control each characteristic: one ‘factor’ is from the male parent and the other from the female parent.

•Each ‘factor’ separates from the other before fertilisation (meiosis and gamete formation) and recombines at fertilisation, but the two, factors, do not blend.

•The ‘factors’ that control different characteristics are passed on to the next generation independently of each other.

Without knowledge of genes, chromosomes or DNA, Mendel managed to accurately explain how such variation between individuals of the same species was possible. As knowledge and understanding of Mendel’s ‘factors’ improved, his basic conclusions were expanded to become two of the fundamental laws of genetics.

**The law of segregation**

There are two copies of every gene in all sexually reproducing organisms that control each characteristic, and the same genes are grouped together on homologous pairs of chromosomes. During meiosis these homologous chromosomes segregate (separate), with one copy of each chromosome and all the genes it holds appearing in every gamete (for example, in the ovum/egg and in the sperm/pollen).

These chromosomes recombine at fertilisation. They do not blend, but instead match together to form homologous pairs again.

**The law of independent assortment**

When the homologous pairs of chromosomes segregate, they do so independently of other pairs of chromosomes. In other words, when the chromosomes line up in their homologous pairs, the side each chromosome takes is completely random. The mother’s chromosome may be on the right for one pair but on the left for a different pair.

We now know this law applies in all cases except when the genes are linked (situated on the same chromosome). Mendel, by chance, did not study any linked genes and so he did not know that genes are situated on chromosomes. We also know now it is the chromosomes that segregate rather than the individual genes as Mendel thought.

Genotypes and phenotypes

The law of segregation does not completely explain why Mendel often found that one form of the characteristic he was examining would disappear from the offspring and then reappear in subsequent generations during his crosses.

Some genes come in different forms. These different forms of the same gene are called alleles. In the previous example, the gene is seed shape, and the alleles are round seeds or wrinkled seeds. When a gene has more than one allele, an individual may have different combinations of those alleles. The specific combination of alleles is called the genotype. The genotype helps to determine the appearance of the individual, known as the phenotype.

A true-breeding individual has two copies of the same allele for each gene. They can only pass one form of the characteristic on to their offspring. True-breeding individuals are also described as being as homozygous for that trait or characteristic.

In Mendel’s experiments, he crossed two different true-breeding individuals together, guaranteeing that the F2 generation would have one copy of each allele, or would be heterozygous for that trait. Even though all the offspring had the two different alleles, they all showed the same phenotype. One allele was being expressed rather the other. The allele expressed in the phenotype of a heterozygous individual is said to be dominant, and the allele that is hidden is said to be recessive.

Individuals showing the dominant trait only need to have one copy of the dominant allele for it to show in the phenotype. This means individuals with the dominant trait could be heterozygous or homozygous dominant for that gene. Dominant alleles are represented by a capital letter that represents the gene.

The recessive trait is only expressed if there are no dominant alleles present. Therefore, all individuals with the recessive phenotype must be homozygous recessive for that gene. Recessive alleles are always shown by a lowercase version of the letter used for the dominant allele (Table 1.2).

AUTOSOMAL INHERITANCE

Chromosomes exist in homologous pairs. They are homologous because they carry the same genes. Because many genes have more than one form, homologous chromosomes may not carry the same allele for those genes. Figure 1.26 shows a homologous pair of chromosomes that carry the same alleles for skin pigmentation and cheek shape, but different alleles for eye colour.

However, one pair of chromosomes is not always homologous. Sometimes the chromosomes carry completely different genes and are totally different lengths. This ‘pair’ of chromosomes are called sex chromosomes. Although they carry genes that code for normal body cells, they also determine the gender of the individual (see more about these chromosomes on page 33). All other chromosomes that have no influence on gender differentiation are called autosomal chromosomes.

Chromosomes can carry thousands of individual genes, but it is easiest to study them one at a time as Mendel did.

Monohybrid crosses

A monohybrid cross is the genetic cross between two individuals that are heterozygous (hybrid) for one (mono) particular gene.

When Mendel crossed F1 individuals with other F1 individuals to produce the F2 generation, this was a monohybrid cross.

In the example used on page 25, Mendel found when he crossed plants that were heterozygous for seed shape, the resulting offspring were in the ratio 3 round to 1 wrinkled. To work this out, you can use a Punnett square.

Coat colour in some dogs is controlled by one gene with two alleles. The black pigment allele is dominant over the recessive brown pigment allele.

Punnett squares

Punnett squares can be used to determine the possible genotypes of the offspring and to calculate the ratios in which the offspring’s genotypes and phenotypes will occur. The parents’ genotypes are used to work out the possible gametes that can be produced, and these are written around the outside of the Punnett square. Within the grid, all possible fertilisation combinations can be modelled.

Mendel repeated his experiments with so many plants (around 29 000 plants over seven years) that his actual data reflected the estimated mathematical ratios and percentages for the experiments. The huge numbers of plants that were cross-bred in every experiment, combined with Mendel’s method of repeating all his experiments, made his data extremely reliable. This is one of the many reasons why his findings are the foundations of modern genetics and why Mendel is such a well- respected scientist.

Test crosses

When looking at a single gene, it is easy to determine the genotype of an individual who expresses the recessive trait. There is only one possibility: they must be homozygous recessive.

A dominant trait only requires the presence of one dominant allele for it to be expressed. This means an individual with a dominant phenotype could be either homozygous dominant or heterozygous for the characteristic.

To determine the genotype of an unknown individual, a DNA test could be carried out or a number of crosses could be performed. Mendel worked hard to ensure his parental generation were true-breeding plants. He would have carried out several test crosses to determine which dominant trait plants were homozygous and which were heterozygous.

A test cross is where an individual with the dominant phenotype but unknown genotype is crossed with a recessive individual. The recessive parent can only supply the recessive allele to the offspring, so the phenotypes of the offspring can be used to determine the genotype of the unknown parent. Figure 1.30 demonstrates the two possible outcomes of a test cross.

Environmental influence on gene expression

While our genes determine what our physical characteristics will be, they do not act alone on the final phenotype. Environmental factors also influence phenotypes. Sunlight can bleach your hair, tan your skin, and cause freckles and moles to appear. The quality of nutrition during growth and development stages of life can influence height, muscle tone and proper cellular functioning. A car accident may result in a loss of a limb. All of these environmental factors can change an individual’s phenotype. Identical twins, who have identical DNA, are never exactly the same. There are always some tiny differences between them. But changes to a phenotype cannot alter the genotype.

SEX-LINKED INHERITANCE

As previously mentioned, humans have 23 pairs of chromosomes – 22 pairs of autosomal chromosomes and one pair of sex chromosomes. The two sex chromosomes in mammals are called X and Y. The genotype for females is XX and the genotype for males is XY. The specific genes on the chromosomes contain the information required for sexual characteristics.

The X chromosome is significantly longer than the Y chromosome. The X chromosome is one of the longest human chromosomes, whereas the Y chromosome is one of the shortest. In addition to carrying the genes responsible for sexual characteristics, the X chromosome also carries many non-sexual genes, such as for blood clotting and red–green colour vision. Traits or phenotypes (and the alleles that code for them) that are carried on a sex chromosome are said to be sex-linked. Many more genes and the specific traits they control are linked to the X chromosome because it is so much larger than the Y chromosome (see Figure 1.34), therefore these genes are common called X-linked genes. Males are much more likely than females to show X-linked traits because they only have one X chromosome that cannot be masked by the Y chromosome.

In general, when investigating the pattern of inheritance for a particular trait, it is useful to consider each trait as one of the following: •autosomal dominant •autosomal recessive •X-linked dominant •X-linked recessive.

Traits show different trends within populations, which may help identify whether they are autosomal or sex-linked, dominant or recessive. These trends are summarised in Table 1.5.

Sex-linked conditions

Two well-known conditions caused by defective sex-linked genes are red–green colour blindness and haemophilia.

Red–green colour blindness

Red–green colour blindness is an X-linked recessive trait. A gene on the X chromosome controls the colour receptors in the retina of the eye. When the gene is defective, the receptors do not function properly and the person cannot distinguish red from green.

Approximately 8% of males and less than 1% of females have red–green colour blindness.

The difference in incidence is because the defective allele is recessive and can be masked by a dominant normal allele in females. It is very rare for a female to have two defective alleles, but not so rare for her to be a carrier.

A carrier is someone who has an allele for a genetic disorder, but does not show the disorder in their phenotype – in other words, they are heterozygous for a recessive disorder.

Haemophilia

Haemophilia is an X-linked recessive disease that prevents blood from clotting. Even a small injury to a person with haemophilia can result in prolonged bleeding and excessive blood loss, or bruising (internal bleeding). It is possible to treat this disease today because the clotting factors can be produced from blood donations or made in the laboratory.

PEDIGREES

Although each of your parents contributes to your genotype, the genotypes of other family members (such as grandparents, aunts and uncles) can all be important in explaining who you are. Inheritance of characteristics is often traced through families using family tree diagrams or pedigrees.

While a Punnett square shows the possible genotypes of the offspring and the percentage chance for each combination, a pedigree indicates what actually happened. Specific symbols are used when constructing pedigrees.

•Males are represented by squares.

•Females are represented by circles.

•If the sex is unknown, they are represented by triangles.

•A marriage or breeding is indicated by a horizontal line between a male and female.

•A vertical line between the parents indicates offspring; if there are multiple offspring, they are listed in order of birth and joined together by a horizontal line above them.

•The characteristic being investigated is shown by shading. Note that this is not always the recessive trait!

•Generations are represented by Roman numerals and individuals by Arabic numerals.

Pedigrees can be used to analyse the inheritance pattern of a particular characteristic. When analysing a pedigree to determine whether an allele is dominant or recessive, the following rules can be used:

•If neither parent has a characteristic and some of their offspring have it, then it must be recessive.

•If both parents have a characteristic and some of their children have it, then it must be dominant.

•If both parents have a characteristic and none of their children has it, then it must be dominant.

For the pedigree in Figure 1.430b, red hair is recessive because individual II2 and his partner do not have red hair but some of their children have it. The parents are both carrying the allele for red hair, but not expressing it. They both contribute their allele for red hair to some of their offspring.

1.3 Gene technology

GENETICS IN MEDICINE

A key step to using genetics in medicine was the completion of the Human Genome Project in the early 21st century. A genome is the full set of genes and alleles found in a species. The Human Genome Project determined the number of all human genes, their locations, and many of their key base sequences. The genomes of other species have also been determined and are stored in huge databases. Geneticists are continually adding to this database as new information and sequences are identified.

Knowledge of the positions of genes and their specific sequences allows geneticists to identify mutations, and investigate the interaction between different genes and the proteins that they code for.

The analysis of genomes from humans and other species, and the base sequencing of the genes, has been made possible by the development of fast computers. Scientists and geneticists can also share their information all around the world in a matter of moments, saving time by not having to repeat work that has already been done.

Advances in technology and scientific understanding

Scientists such as Walter Sutton, Theodor Boveri, Reginald Punnett, and Thomas Hunt Morgan in the 1920s made progress in understanding the location of genes on chromosomes. Where Mendel used the pea plant, Morgan used the fruit fly (Drosophila melanogaster), and his studies resulted in the discovery of sex linkage. Morgan and his team spent numerous hours anaesthetising the flies, and checking their eye colour and other characteristics. From his results, Morgan established the relative position of many genes on the chromosomes. This is called gene mapping and is used to determine the genome of a species.

The fruit fly was a very convenient organism to use to study genomes and inheritance because it only has four pairs of chromosomes: three autosomal pairs and one pair of sex chromosomes. Fruit flies also breed prolifically, with each pair producing dozens of offspring approximately every 2 weeks. This enabled Morgan to generate lots of data from repetitive trials and to ensure his results were reliable.

Knowledge of the genome and gene sequences of a species as well as understanding protein synthesis enabled geneticists to identify sequences that cause diseases or other characteristics of interest. The techniques and technology used for sequencing fruit- fly DNA were used to sequence genes and discover genomes of many other species, and also helped lead to the discovery of restriction enzymes and DNA ligases.

Restriction enzymes were first extracted from bacteria in the 1960s. Restriction enzymes are proteins that can cut the DNA molecule at specific base sequences. Their natural role is to protect bacteria from foreign DNA (which they cut up and destroy). They are called restriction enzymes because they restrict the growth of other organisms by destroying their DNA. The hunt for more restriction enzymes continues because each is specific for one sequence. The more restriction enzymes we can use, the more ways we can manipulate DNA.

Scientists and geneticists use restriction enzymes with another group of enzymes called DNA ligases, which were also discovered in the 1960s. DNA ligases are found in all types of living things, not just in bacteria. The ligases attach or ‘link’ pieces of DNA together. They tend to be less specific than restriction enzymes and so can be used on a number of different sequences. DNA ligases are vital for normal DNA replication and are one of the main enzyme groups responsible for repairing damage to DNA and mutations.

Geneticists use restriction enzymes as scissors and DNA ligases as glue. With these genetic tools, genes can be cut and pasted into different positions, chromosomes, organisms and even into different species. These different forms of DNA manipulation are collectively known as genetic engineering.

Genetic engineering and bioinformatics

Genetic engineering is the manipulation of the genome of an organism, usually through altering the base sequence of specific genes or by transferring genes from one organism to another. On a very basic level, genetic engineering may be simply altering phenotypes of organisms through artificial selection (bred by humans to possess specific characteristics).

Genetic sequencing of genes can be useful to identify the differences in base sequences between alleles, especially those that may cause genetic disorders. Sanger sequencing is a technique that was developed in 1977, but is still used today.

Sanger sequencing involves artificially replicating DNA with bases that have radioactive or fluorescent tags attached. The ‘tagged’ DNA is analysed by a computer that detects the different tags. The sequence can then be ‘read’, recorded, analysed, and stored in large databases and accessed by scientists all over the world.

Using fast computers is critical for the analysis of DNA sequences. It enables the comparison of DNA between individuals of the same or different species, and can help to determine changes in DNA over time and to establish evolutionary relationships. The use of computers in genetic analysis is termed ‘bioinformatics’.

Once the sequence is determined for a chosen gene, it can be cut out of a chromosome using restriction enzymes and inserted into a different organism using DNA ligase. The second organism does not even need to be the same species as the donor organism. The organisms with new, inserted genes are called transgenic organisms. Transgenic organisms have artificially created genotypes.

The scope of this work is enormous. Genes from other organisms can be cloned (copied) inside rapidly multiplying bacteria and then cut out of the bacterial chromosomes. The genes can then be purified and inserted into faulty cells to treat diseases. This process is called gene therapy.

Transgenic organisms can also be used to produce human hormones or tissues for transplants. For example, the human gene responsible for producing insulin can be inserted and expressed (activated) in bacteria. With the bacteria now containing recombinant DNA (DNA with the new gene), it produces human insulin that can be used to treat diabetes.

This form of biotechnology avoids the rejection issues faced by using similar products from animals such as pigs or sheep.

Gene technologies in medicine

Genetic technologies use the cutting power of restriction enzymes and the sticking power of ligases to remove genes from one organism and insert them into another. New transgenic organisms can be generated, such as pigs that produce human proteins, or bacteria that produce human hormones like insulin, that can be extracted and used to treat illnesses.

Genetic sequencing and fast computers are often used to diagnose and treat genetic diseases because the location of defective genes can be found quickly.

Gene therapy has been quite successful in the treatment of cystic fibrosis (CF). Patients with CF have a deficiency in a gene that controls the production of a protein that regulates the movement of ions across cell membranes. CF sufferers have an accumulation of thick mucus that can damage lung tissue, which reduces their life span significantly.

Medical scientists have been able to clone the healthy form of the cell membrane protein gene in bacteria. The purified gene is then attached to a carrier molecule called a vector. The vector is usually a harmless virus and it is administered as a drip inserted in the nose of a patient. The viruses enter many of the lung cells and insert themselves into the DNA in the nucleus, just like a normal virus. However, the DNA they inject is not harmful at all and contains the healthy gene. When the lung cells divide, the new cells contain the healthy gene.

In the cells where the gene is successfully inserted, the change is permanent. All new cells generated from these ‘treated’ cells will produce the protein required to regulate ion movement, and so reduce the build-up of mucus in the lungs.

A controversial type of gene therapy involves the insertion of a healthy gene into an embryo. Parents who are known carriers of a defective gene can undergo in vitro fertilisation (IVF) and the defective gene of the embryo can be replaced shortly after fertilisation – even at the zygote stage. As the embryo develops, the cells will have the unaffected gene. The controversy arises because some people believe that any alteration of an embryo is unethical.

Transgenic and gene therapy research is ongoing. While the immediate results look promising, there are still some concerns about possible long-term effects of genetic engineering on individuals and populations. However, with more research comes more data, which improves the reliability of the results and conclusions that can be drawn.

OTHER APPLICATIONS OF GENE TECHNOLOGIES

Gene technology has wide applications. In addition to medicine, gene technology has uses in law, agriculture, ecology and conservation, world hunger problems and the economies of many countries. For example, the technique of DNA profiling has become established as a major tool in solving criminal cases and in determining paternity (identifying the father of a child).

Environmental applications

Some of the devastating effects of environmental oil spills and waste products from mines have been reduced by the development of bacteria containing genes that produce proteins to break down oil deposits and mining wastes. Other microbes are genetically modified to be able to extract heavy metals, such as copper and lead, from deposits. This is particularly useful when the amounts of metals in the earth become too low for large-scale mining.

Agricultural applications

Agriculture has been significantly affected by the introduction of transgenic animals as well as genetically modified (GM) crops and foods, including plants that are resistant to certain chemicals and pests.

Crops that have been engineered to resist disease mean that poorer farmers can grow them without the extra expense of spraying herbicides and pesticides. Because lower amounts of sprays are necessary when growing these crops, production costs and environmental pollution are reduced. It is also possible to produce plants that are resistant to herbicides, meaning that when herbicides are sprayed on crops and weeds, only the weeds will be killed.

Plants have even been developed with genes that control the production of vitamins and minerals. Golden rice has had genes inserted from daffodils that help produce vitamin A, making Golden rice much richer in vitamin A than non-transgenic rice. Without adequate amounts of vitamin A, people’s eyesight can be severely impaired, even leading to blindness. Other GM rice strains are being developed to increase iron content. There are also ‘pharm’ plants and animals that produce pharmaceutical proteins required by humans.

Increasing the nutrient value of cheap crops, such as rice and wheat, may prevent starvation and reduce malnutrition in areas where people may not have enough money to produce a wide variety of food for the population.

Examples of plants that have been genetically engineered include those shown in Figures 1.58–1.60.

THE ETHICS DEBATE

Ever since humans began to domesticate plants and animals approximately 10 000 years ago, we have been influencing nature. The artificial selection of plants and animals for breeding became common about 200 years ago. Some of the results include cattle with good muscle mass for beef or high milk production for dairy products, high-yielding rice and wheat, and disease-resistant crops.

The discovery of DNA and the development of various gene technologies have enabled us to manipulate organisms even further. But along with all the benefits there are ethical issues. It is no longer a question of ‘can we?’ but rather ‘should we?’ How much is human interference affecting human welfare and the welfare of other species? Do the advantages outweigh the disadvantages?

Genetic screening and testing

Genetic testing is carried out on people who are known to be at risk of a particular genetic disease or condition. This is usually evident from an individual’s family history or pedigree. Genetic screening refers to testing for a variety of conditions regardless of a previous family history of genetic disease.

Genetic screening and testing services available in Australia include:

•maternal serum screening (MSS) – offered to all pregnant women for the detection of Down syndrome and spina bifida

•newborn screening – the screening of all newborn babies for genetic diseases, including phenylketonuria (PKU), hypothyroidism and cystic fibrosis

•adult screening to diagnose an existing disease, determine a predisposition to disease, or identify carriers with a reproductive genetic risk.

Genetic screening helps with early diagnosis of genetic diseases and subsequent intervention. This will potentially minimise the frequency of such diseases in subsequent generations; however, it sometimes involves some very difficult decisions. For example, should parents who are carriers of genetic mutations have children? What are the risks of the tests? Who should be screened, and for what? What is the impact of false positives? What options are available if the result is positive?

Genetic counsellors can help clarify the situation, but they cannot make the decision for the people involved.

Privacy is another big issue. Insurance companies in the future may make a DNA analysis part of an application for insurance. Such information could lead to discrimination against affected individuals or denial of insurance.

The collection, storage and potential uses of genetic information raise many ethical questions, including access to and the possible misuse of such information.

Gene therapy

Gene therapy involves the insertion of a healthy gene into the chromosomes of an individual with a defective gene. Gene therapy that involves the body cells (somatic cells) can be therapeutic only. This means that the treatment of the mutation cannot be passed on to the next generation. At present, gene therapy targeting germ-line cells (cells destined to become gametes) is not legal in Australia.

Apart from the success with cystic fibrosis and the great potential of gene therapy, limited progress has been made since the first clinical trials of gene therapy in 1990. Human trials with gene therapy suffered setbacks with the deaths of several people as a consequence of the technique. The ethics of treating patients with a technique that involves significant risks to life have to be considered carefully.

Stem cells

Stem cells are undifferentiated cells that can differentiate (change in structure and function) into many different types of specialised cell types, such as muscle, nerve, liver and blood cells. There are two types of stem cells. Embryonic stem cells are able to give rise to most cell types, whereas adult stem cells can only give rise to certain cell types. The use of adult stem cells is relatively common and does not raise the ethical concerns associated with embryonic stem cell use.

Embryonic ethical concerns

There are many ethical issues associated with the use of embryonic stem cells. Fertilising ova in a laboratory can artificially produce embryos. At present, such procedures are illegal in Australia. The only embryos used for research are those classed as ‘excess embryos’, having been originally produced for use in IVF. The use of these excess embryos is considered unethical by some people because the collection of stem cells destroys the embryo. In their opinion, the embryos are potential life and their use in research represents the deprivation of life to these embryos. However, embryonic stem cells have the potential to treat a variety of diseases, including cancer, multiple sclerosis (MS), Parkinson’s disease, motor neurone disease and spinal cord injuries.

Some potential parents may also want to select certain embryos over others. For example, they may want a male child or a female child. They may want a child with particular eye colour or hair colour. They may choose a healthy embryo over one with, say, cystic fibrosis.

Should parents be able to pick and choose the characteristics of their child? What if the embryo that has been produced does not have the desired characteristics? What happens to undesirable embryos? Should embryonic screening be mandatory to help eradicate genetic disorders?

Cloning

A clone is an exact copy of something else. Individual genes are often cloned using bacteria (see Figure 1.68). It is also possible to clone an entire organism by the technique of nuclear transfer. This production of a new organism is called reproductive cloning.

Therapeutic cloning is a type of reproductive cloning that uses the embryo as a source of embryonic stem cells. Because the embryo is effectively ‘killed’ for this to occur, some people are ethically opposed to this technology, which raises the question ‘when does life actually begin?’

Some simple animals are able to clone themselves, such as some worms and seastars. Many plants are easily cloned from cuttings. Large mammals including sheep and cows have also been cloned by nuclear transfer.

However the process is not perfect, as the clone organisms tend to have a much shorter life span than normal.

The prospect of cloning organs and tissues for transplants would have enormous medical potential; there would be no risk of rejection because the transplanted tissue would be genetically identical to that of the patient.

But what if a whole human were cloned? What ethical concerns would need to be considered?

Genetically modified organisms

One of the most controversial developments in modern food production is the apparent rise in genetically modified organisms, or GMOs. As mentioned earlier, GM plants have been modified to enhance desired traits, such as increased resistance to herbicides or improved nutritional content.

GM crops pose a threat to biodiversity because they replace a number of natural varieties of plants with one variety: the genetically engineered plant. The number of GM plant varieties has significantly increased in the last decade or so.

The organic food movement is completely against the principle of GM foods, and public debate into the benefits and dangers of such foods is likely to continue well into the future. Some people believe that GM foods pose health risks, although there is no clear evidence for or against this.

A criticism of GM foods is the potential for accidental gene transfer to other species. GM plants may also contaminate non-GM plants of the same species through natural cross- pollination. Another concern is that increased pesticide and herbicide resistance may develop in insects and other pests. The GM plants that have the pesticide and herbicide resistance may then become vulnerable to the resistant pests.

Chapter 2 Evolution

2.1 Explaining biodiversity

EARLY EVOLUTIONARY IDEAS

Evolutionary ideas were all first proposed without any knowledge of DNA and genetic inheritance. As scientific knowledge has increased, some ideas have been rejected, while some have been supported by new findings in genetics, developmental biology and palaeontology. There is now considerable unbiased, reliable and valid scientific evidence that supports evolution, which is why it is now considered a scientific theory.

Lamarckian theory

One of the first documented explanations for changes in species over time was by Jean-Baptiste de Lamarck, a French naturalist, who believed in evolutionary change – that organisms change over time due to changing environmental conditions. He is best known for his theory of inheritance of acquired characteristics, which was first presented in 1801. In this theory, Lamarck proposed that an organism can develop characteristics during its lifetime in order to adapt to its environment, and that those changes are passed on to its offspring. In other words, the change is made by what the organism wants or needs. Lamarck also stated that body parts not being used, such as the human appendix and little toes, are gradually disappearing and eventually people will be born without these parts. So, the more a limb or aspect of the body was used, the bigger or stronger it became. The less a limb was used, the smaller and weaker it became until it disappeared entirely.

Although most of Lamarck’s ideas were supported by what he saw around him, they could not be tested or replicated and were attacked by the French scientific establishment as being unreliable.

Darwin sets sail

For Charles Darwin, an English naturalist and geologist, explaining biodiversity was of great importance. Darwin was well educated and had been exposed to the sciences through his father and grandfather, who were both physicians.

Darwin’s grandfather had sought to explain life in evolutionary terms in a book he published in 1794: ‘Would it be too bold to imagine that all warm-blooded animals have arisen from one living filament?’ This question was too bold for its time and lacked substantial evidence.

Darwin had also read the works of Lamarck. With this background of scientific thought and process, the young naturalist set sail on a 5-year world cruise as the unpaid naturalist aboard HMS Beagle. The year was 1831 and Darwin was just 22 years of age.

A diversity of life

Over the 5 years of the HMS Beagle’s voyage, Darwin investigated the geology of the places he visited and collected all manner of wildlife and fossils – the remains of living organisms. Darwin carefully recorded the details of each specimen before sending the collection back to England by ship. During this period of intense record keeping and observation of natural systems, Darwin questioned traditional views and developed his hypothesis for the origin of species. Darwin made his most significant observations during the final stages of the voyage, when the HMS Beagle headed westward into the Pacific to the Galapagos Islands.

Galapagos Islands

The Galapagos Islands are a chain of volcanic islands about 1000 km west from mainland Ecuador. The eastern-most islands are the oldest, with substantial plant growth and weathering, whereas the western-most islands are the newest and are still volcanically active. Darwin and his helpers collected specimens from the Galapagos, seeking to obtain at least one of each species. Among the specimens collected were 13 finches, all of which resembled one another in terms of the general form of their bodies and plumage. Yet each specimen had slight differences in beak size and shape and represented a new species. Most had also been found on different islands. In his journal, Darwin noted that these creatures held a striking similarity to those found on the mainland. He wondered, if new and different beings had been created for each island, why did they look so much like those from the mainland?

The dry, volcanic Galapagos Islands looked desolate and the only plants present struck Darwin as ‘wretched-looking weeds’. As he walked across Chatham (San Cristobal) Island’s rugged lava surface, Darwin came across two huge tortoises ambling along a well-beaten path.

A few days before Darwin left the Galapagos Islands, the Islands’ Vice-Governor remarked that he could tell which island a tortoise came from by the shape of its shell. This provided Darwin with the inspiration he needed. Indeed, this is the main way in which the various types of tortoise on the Galapagos Islands differ: the shape of the tortoise’s shell depends on its environment, which varies significantly depending on the island. Tortoises that live on dry islands, such as Española Island, have shells that are raised at the front so the tortoises can reach up for vegetation.

In contrast, tortoises that live on large islands with dense vegetation have domed shells to help them push through the shrubbery.

DEVELOPING THE THEORY OF EVOLUTION

On 2 October 1836, the HMS Beagle reached the shores of England. A year later, Darwin was arranging his Galapagos collections and finishing work on his journal, which became known as The Voyage of the Beagle. Again, it struck Darwin that if each species had been created independently, why should some details have been repeated in the different tortoises and birds, whereas other features were distinctly different?

The idea of species haunted Darwin and he thought that if he was ever to make sense of it, he needed to collect as many facts about variations in plants and animals as possible. This search led him to investigate the breeding of domestic species such as pigeons.

Artificial and natural selection

Selective breeding, or artificial selection, has been a human pursuit for well over 10000 years, when many human populations moved from the hunter–gatherer lifestyle to more permanent settled communities. Selective breeding is essentially humans choosing breeding partners for plants and animals in an effort to ‘select’ certain traits for their offspring. Over many generations, the ‘wild’ traits are often lost and the species is considered ‘domesticated’. Darwin, and many other pigeon breeders, selected all manner of different traits to create new breeding lines of pigeons.

Darwin then wondered how ‘selection’ occurred in nature. The answer Darwin was seeking came from the work of Thomas Malthus, whose paper An Essay on the Principle of Population gave Darwin the insight he needed. Malthus argued in his paper that the human race would completely overrun the Earth if it were not held in check by war, famine and disease. Darwin extrapolated from this that, under changing circumstances, favourable variations would tend to be preserved and unfavourable ones would be destroyed.

At last Darwin had a hypothesis to test, although it would take another 20 years of painstaking hard work before he was convinced that his hypothesis had enough support to be developed into a theory. Alfred Russell Wallace, a naturalist who had worked in the East Indies, sent Darwin a copy of his manuscript in which he had independently arrived at the same concept of natural selection. In 1858, Darwin and Wallace jointly published a paper, and in 1859 Darwin published his book On the Origin of Species by Means of Natural Selection.

One mechanism Darwin proposed that enabled natural selection to occur was **sexual selection**. All organisms that reproduce sexually have some measure of choice over which individual they reproduce with, although some much more than others. This is most easily seen in animal species where one gender picks or selects the other based on particular characteristics. For example, female peafowl (peahens) will mate with the male peafowl (peacock) with the most and brightest eyespot feathers. This is probably because the quality of feathers is an indication of health.

Evolution by natural selection occurs as a result of competition between individuals in a population with different traits. This competition may be for food, shelter or mates. Selection for traits that provide an advantage with regard to mating is called sexual selection. Sexual dimorphism is the term that describes the male and female of the same species being different in appearance (di means two; morph means shape).

On the origin of species

Darwin’s book On the Origin of Species by Means of Natural Selection was largely one long argument for the theory of evolution by natural selection. Darwin made the following observations.

•Individuals in a species vary.

•Much of the variation is heritable (traits are passed from parents to offspring).

•Reproductive capacity is greater than needed (many species produce more offspring than are required to maintain population size).

•Resources are limited.

Based on these observations, Darwin inferred:

•there is a struggle for existence

•individuals best suited to the environment (the ‘fittest’ individuals) survive and reproduce

•over time, this results in populations adapting to the environment and can lead to new species.

Darwin recognised the role of geographic isolation in the formation of new species and said that the small differences within a species could lead to more distinct differences between species over time. Although these observations may seem relatively obvious to us, they were nevertheless a major conceptual challenge for many people, including many scientists, of Darwin’s time.

The two basic ideas that flowed from Darwin’s book are the concept of the ‘tree of life’ and the theory of evolution by means of natural selection.

In his tree-of-life metaphor, Darwin depicted living organisms as being organised like the limbs of a great tree, with more general groups branching into more specific ones.

He noted that classification systems already reflected this branching in their hierarchical arrangement of species. Darwin suggested it was the conditions of the environment in which a species lived that determined which traits and characteristics would make them ‘fit’, and the differences in environmental conditions across the world explained the differences between species.

In his **theory of evolution** by means of natural selection, Darwin concluded that:

•species change over time •some species become extinct •some species keep diverging, splitting eventually into multiple descendent species– ‘common descent’.

Biogeography

At the beginning of the 17th century, the English philosopher Francis Bacon noted that the east coast of South America and the west coast of Africa looked as though they could fit together like pieces of a jigsaw. Since this time, geologists have developed our knowledge of the structure of the Earth and the movement of continents. The theory of plate tectonics is well supported by observations across the planet. At one time all the continents were connected in a single landmass called Pangaea. This supercontinent then broke in two to form Gondwana in the south and Laurasia north of the Equator.

The theory of plate tectonics has had a major impact on evolutionary theory because living organisms were carried on the landmasses when they moved and separated.

Biogeography, the distribution of the fossils of extinct plants and animals as well as modern- day species, supports the theory of plate tectonics. Some continents share very similar organisms even though they are separated by large stretches of ocean because they were once joined together. The distribution for animals that are able to fly or swim is less predictable, but for the rest, continental movement is the only convincing explanation.

Plate tectonics provide a well-supported explanation for the geographical isolation of species that eventually results in speciation – the evolution of a new species. The biogeography of groups of similar species, such as the ratites (flightless birds), and the existence of marsupials on several continents, can be explained by movement of continents. ‘Coincidence’ is simply not a scientific explanation. Wallace’s line identifies this variation and similarity in species according to geography, although he did not have the knowledge and understanding of plate tectonics to explain why this variation existed.

2.2 Evolution of a species

NATURAL SELECTION

To date, the theory that best explains the diversity of life forms and the evidence of change over time is Darwin’s **theory of evolution by natural selection**. Darwin argued that it was entirely possible for one species to evolve gradually into a separate species, with its own unique traits, over many generations.

As Charles Darwin and Alfred Russell Wallace pointed out, an individual doesn’t evolve; rather, populations do. A population is a group of interacting individuals of a species living in a particular area. A species is defined as organisms that can reproduce to produce offspring that are both viable (able to survive) and fertile (able to reproduce).

Variations in populations

Natural selection depends on the variation of traits within a population, but where does this variation come from? All members of a species share a set of common traits that help to define them as a species. For example, Homo sapiens (humans) are identified for the purpose of classification as:

•an animal (kingdom Animalia) •with a notochord (phylum Chordata)

•with a segmented spinal cord (subphylum Vertebrata) •that suckles its young (class Mammalia)

•that gestates its young with the aid of a placenta (subclass Eutheria) •that is equipped with five-digit limbs, a collarbone and a single pair of mammary glands on the chest (order Primates)

•that has eyes at the front of the head, stereoscopic vision and a proportionately large brain (suborder Anthropoidea).

Our species belongs to the family Hominidae and the genus Homo (larger- brained hominids that appeared approximately 2 million years ago) and is characterised by a higher and more vertical forehead, a round skull, small face and teeth, a prominent chin and a longer, more slender skeleton. Despite these unifying traits, no two people (except for identical twins) look the same. Most traits differ from one individual to another, especially in sexually reproducing species.

Much of the variation between individuals is due to genetic differences that can be inherited – something that Darwin and his contemporaries observed but did not understand. Individuals of the same population generally have the same number and types of genes, but different alleles (variations of the genes, as discussed in chapter 1). All the genes in the entire population can be thought of as a gene pool– a collection of genetic information. The gene pool includes all the alleles for all the genes in the population: all the variations possible without new mutations.

Mutations

You have about 20 500 genes, each of which may have several alleles. It has been estimated that there may be more than 70 trillion different allele combinations! Consequently, it is extremely unlikely that another person with your exact genetic make-up has ever lived or ever will (unless you have an identical twin). Only alleles that already exist in parent DNA can be inherited, unless a mutation creates new alleles in the DNA of gametes.

As you read in chapter 1, mutations can occur at the gene level or at the chromosome level. Some mutations may be lethal, having a drastic effect that results in death. Others may be neutral, neither helping nor harming an individual. Natural selection does not increase or decrease the frequency of neutral mutations in a population because they do not influence an individual’s chances of surviving or reproducing. One example of this is syndactyly – webbing between the fingers or toes. It is common because a developing foetus has webbing that undergoes apoptosis, or ‘programmed cell death’, to remove it before the baby is born (see Figure 2.34).

A mutation may also give an individual a survival advantage. Even if the advantage is small, it increases the chance of that individual surviving to reproduce and pass on those ‘favourable’ alleles. Chance events or natural selection may preserve the mutated gene and ensure its representation in the next generation.

Beneficial and neutral mutations have been accumulating in different lineages for billions of years. Through all that time mutations have been the raw material for evolutionary change–the basis for the staggering range of biological diversity, past and present, as environmental conditions change and favour them.

Allele frequencies

In the real world, populations are always evolving. How common an allele is within a population is considered to be its frequency. The allele frequency changes when environmental conditions change to make that allele favourable or detrimental.

SPECIATION

When a variation is favoured by the environmental conditions, it is referred to as an adaptation. Variations within a species provide ‘options’ for the species in the face of changing environmental conditions. Although individual organisms may be wiped out, some members of the population will continue the species gene pool.

Along the way, some entire species will become extinct and new species will emerge. Under normal conditions, genes in a given population are exchanged via breeding, which is known as gene flow. Even if some variation occurs, it is limited by gene flow. Gene flow is interrupted if the population becomes divided into two groups so that the groups experience some sort of **isolating mechanism**.

Temporal isolation occurs when individuals of different populations reproduce at different times. For example, cicadas mature underground (Figure 2.37). One species emerges and reproduces every 17 years. A different species, which is almost the same in terms of appearance, emerges every 13 years to reproduce. At this rate, the two species would release gametes at the same time only once every 221 years!

Mechanical isolation occurs when there is a physical incompatibility between the body parts of potential mates or pollinators. For example, the flowers of some plants vary in size and shape, or they may have spiny barriers, thus excluding certain pollinators (Figure 2.39).

If there is no exchange of genes between the two isolated groups, then they may begin to look and behave differently from each other. Given enough time for evolution to occur, the two populations may become so different that they are incapable of interbreeding should they ever come together again: a new species has been created (speciation). Speciation can occur in a variety of ways.

Allopatric speciation

Allopatric speciation occurs when one species divides into two or more through some form of geographic isolation like the development of a mountain range, widening of a river or even continental drift. Each population begins to experience very different environmental selective pressures. In their separate habitats, the groups go their own evolutionary ways, accumulating different gene mutations and being subjected to different selective pressures, which favour different adaptations.

The finch species on the Galapagos Islands (refer back to Figure 2.7 on page 62) would have most likely evolved from a population of an ancestral finch species that was blown off mainland

South America by a storm. The distance between the islands and the mainland is too far for the little birds to fly and so gene flow between the mainland and the island species stopped. The conditions on the Galapagos Islands were, and still are, different from those on the mainland. Thus, the finches evolved from the ancestral mainland species by allopatric speciation.

Extreme events can also result in geographical isolation (Figure 2.41). In the summer of 1995, Hurricane Luis and Hurricane Marilyn ripped through the northern Lesser Antilles in the Caribbean. Fifteen green iguanas survived on a raft of uprooted trees for nearly a month, floating over 300 km before reaching the northern- most island of Anguilla. These few individuals were the first recorded of their species, Iguana, to reach the island. The iguanas have since established themselves as an independent breeding colony on the island and, in time, natural selection may operate on this isolated group until they become a new species through allopatric speciation.

Allopatric speciation is thought to have occurred among rock wallabies in Australia (Figure 2.40). Rock wallabies live in rocky outcrops, which are often separated by long distances, resulting in their geographic isolation from each other. There are approximately 20 different genetically and chromosomally different forms of rock wallabies in Australia, of which 16 are classified as different species.

Sympatric speciation

In sympatric speciation, new species arise within an existing species that share the same geographical location. This form of speciation is much more common in plants than in animals. It may occur as a result of the failure of chromosome separation during meiosis or it may be the result of a cross between two (plant) species. The resulting new species cannot breed with the parent species, but, in the case of plants, may be able to reproduce asexually.

Sympatric speciation is most common in animals when some individuals take advantage of a different niche (small area within the habitat with highly specific and limited conditions) within the same environment.

A new niche may arise because of an environmental change, such as a new food source. Sympatric speciation is less common than allopatric speciation because gene flow is not disrupted within the original population.

The Galapagos finches have also shown sympatric speciation. It is likely that the ancestral finches were of a single species, but due to the fierce competition for food on the newly established volcanic islands, the finches became highly specialised foragers. Large, thick beaks are better at cracking the hard seeds of the Tribulus plant, whereas long slender beaks are better at reaching the nectar in cactus flowers, and small beaks are better at dealing with little seeds. The natural variation within the population of beak shape and size meant that different individuals were better equipped to eat certain foods.

Slowly, over time, the different feeding groups became different species, occupying different niches within the same habitat.

2.3 Evidence for evolution

RELIABLE EVIDENCE

Support for any theory requires valid and reliable evidence from a range of sources. Evolution is no different. Research continues to discover more detail about the mechanisms and the relationships between selective pressure and the nature of adaptation, but the data gathered from this kind of research consistently supports the overall theory.

Early evidence

Early evidence for the theory of evolution came from the study of fossils. Fossils are the remains or traces of organisms from a past geological age embedded in rocks or other substances by natural processes. In the late 1600s, Danish anatomist and geologist Nicholas Steno realised that fossils were not just interesting things to collect and catalogue, but the remains of past life. He observed the landscape around him and came up with the law of superposition – that older layers of rock are deeper and younger layers lie on top of them. He argued that these layers form slowly and that each set of fossils is a snapshot of life at that time. Evidence of large-scale extinctions reinforced that life forms change with changing environmental pressures, even if that simply means that many die and only few survive.

In the 1790s, British naturalist William Smith studied mines and proposed that rock layers with the same type of fossils must have been laid down at the same time. This allowed scientists to estimate the age of a rock layer relative to others. Geologists realised that the Earth must be millions of years old, but an accurate estimate was not made until the 1900s, after the discovery of radioactive decay and the development of radiometric dating.

The fact that the Earth is more than 4 billion years old provided more evidence for evolution, as it allowed time for significant changes to occur.

Another step forward in support of evolutionary theory came from the extensive records that were created, and continually added to, as curious observers travelled the globe and documented the different species they saw.

Anatomists studied the wealth of new organisms and noticed underlying similarities in many groups, sometimes finding similarities to fossils.

Human influence on evolution

Humans play a role in evolution, both intentionally and unintentionally. New species of domestic animals and plants are created by artificial selection. These species make it possible to feed and clothe the rapidly growing human population. However, our use of pesticides and antibiotics has had unintended consequences.

The pesticide DDT (dichlorodiphenyl- trichloroethane) was introduced in the 1940s to kill insects that spread human disease, such as mosquitoes, which transmit malaria. This was a great success, reducing malaria cases in Sri Lanka from 3 million per year before the program to just 18 in 1963. Five or six years after the start of spraying for both mosquito control and agricultural use, insects resistant to DDT began to increase in numbers as resistant insects survived and bred. DDT is now virtually useless for mosquito control in Sri Lanka and many other countries.

The evolution of antibiotic-resistant bacteria has been even faster due to the short generation times of bacteria. Penicillin was first introduced in the 1940s, and resistant bacteria appeared in that same decade.

Widespread use of penicillin in the 1950s and 1960s, to treat diseases caused by bacterial infections and to promote growth in livestock, accelerated the evolution of resistant bacteria. Hospitals are home to many strains of multidrug-resistant bacteria, commonly called ‘superbugs’. Use of antibiotics is now strictly regulated in an attempt to control the problem and preserve the effectiveness of existing antibiotics.

Many people get ‘flu’ vaccinations each year to reduce the likelihood of catching the influenza disease. However, the need to have a new vaccination each year is another indication of evolution. The high rate of mutation in the influenza virus results in the evolution of dozens of new strains each year.

ANALYSING FOSSILS

Fossils include the remains of dead organisms, parts of a dead organism or even the evidence that an organism existed. Fossils are extremely important for understanding the evolutionary history of life on the Earth because they provide direct evidence of evolution and detailed information on the ancestry of organisms. Palaeontologists study fossil records and determine their relationships with different geological time periods.

Fossilisation requires the organism, or its traces, to be buried quickly so that weathering and total decomposition do not occur. Skeletal structures, as well as other hard parts of the organisms that resist weathering and are slower to decompose, are the most commonly occurring form of fossilised remains. Trace fossils are moulds, casts or imprints of the activity of previous organisms. An example of a trace fossil is a dinosaur footprint preserved in rock.

Dating fossils

It is possible to find out how a particular group of organisms evolved by arranging its fossil record in a chronological sequence. Relative dating can provide approximate dates for most fossils because fossils are found mainly in sedimentary rock. As you learned in year 8, layers of silt or mud on top of each other form sedimentary rock. The resulting rock contains a series of horizontal layers, or strata. Each layer contains fossils that are typical for a specific time period during which they were made. The lowest strata contain the oldest rock and the earliest fossils, whereas the highest strata contain the youngest rock and more recent fossils.

Geological time is divided into eras and periods on the basis of different sets of fossils. Major changes (extinction events) mark the larger divisions.

Advances in our understanding of matter have led to technologies that can provide more accurate timeframes for fossils. Absolute dating (also known as radiometric dating) relies on the level of radioactivity detected in rocks containing radioisotopes. The best rocks for radiometric dating are volcanic rocks that contain uranium isotopes, but these rocks do not contain fossils. To obtain a more accurate idea of the age of a fossil, scientists examine volcanic rock layers above or below the fossil. These provide a range of dates for that rock stratum. Scientists can calculate an absolute date range for a set of fossils by combining data from several sites. In rare cases, a layer of volcanic ash in sedimentary rocks will provide an absolute date.

Transitional fossils

It is thought that life originated in the sea, crawled onto land and then took to the skies. But what evidence links these stages? Transitional fossils show the intermediate states between the ‘before’ and ‘after’ stages.

They are sometimes referred to as ‘missing links’, but knowledge of geology and evolution allows palaeontologists to predict the location of transitional fossils and find them.

When Darwin first published his theory of evolution, he stressed that the lack of transitional fossils was the most formidable obstacle to his theory because, at that time, very little was known about the fossil record. Since then, numerous examples have been found, starting with the discovery of Archaeopteryx in the Solnhofen area of Germany just 2 years after Darwin’s work was published.

Archaeopteryx is considered by some scientists to be the earliest and most primitive bird known, displaying a number of features common to both birds and reptiles. Other scientists think that Archaeopteryx should be considered a feathered dinosaur. All agree that it is an important transitional species.

Living fossils

According to fossil records, some modern species of plants and animals are almost identical to species that lived in ancient geological ages. Living fossils are existing species of ancient lineages that have remained unchanged in structure and form for a very long time.

Examples of living fossils include the coelacanth fish (Figure 2.55), horseshoe crabs, the Ginkgo trees and Metasequoia conifers of China, and the Wollemi pine, which was discovered in New South Wales in 1994.

ANALYSING THE LIVING

Your family shares similar traits. There are more similarities shared between members of the immediate family than with extended family. The same applies to all living organisms. Such observations of the living are considered important evidence for evolution.

**Evolutionary theory** is supported by the analysis of similarities between organisms – both living and dead. These similarities can be in body parts, cell structure, biochemistry, embryo development or even vestigial structures (structures that once performed a function in an ancestor but are now functionless). The evidence studied to date suggests that the greater the level of similarity, the more closely related two organisms are.

Comparative anatomy

Comparative anatomy is a method of studying evolutionary relationships between different species, and involves comparing the similarities and differences in anatomy (physical body structures). Structures that are found across different species and have a similar pattern but different function are known as homologous structures. The basic pattern comes from common ancestry, whereas differences come from adaptations to specific environmental conditions in a form of evolution called adaptive radiation or divergent evolution.

The most commonly discussed homologous structure is the pentadactyl limb – the pattern of limb bones in all groups of tetrapods (four-legged vertebrates) that ends in five digits (Figure 2.61). This structure can be traced back to the fins of certain fossil fishes from which the first amphibians are thought to have evolved. In all tetrapods, the fundamental structures of the pentadactyl limbs are the same, indicating that they originated from a common ancestor. During the course of evolution, these structures have been modified to serve different functions as a result of adaptations to different environments and modes of life.

Structures in organisms that perform the same function but are structurally different are described as analogous structures. An example of this is the wing of a butterfly and the wing of a bird (Figure 2.62). They have a similar shape because they are both used to fly, but the fundamental structure is very different. This absence of similarity in structure suggests there is no common ancestor in the recent past. However, the similarity in function suggests that both species have evolved under similar environmental pressures and arrived at similar adaptations.

Analysing embryos

Scientists have noticed that, although adult vertebrates have clear differences, many embryos demonstrate huge similarities during the early stages of development. For example, a chicken and a human are very different when fully formed, but chicken embryos are very similar to human embryos. Even reptile embryos are similar to human embryos.

Embryos may also show many interesting features that are not seen in the fully developed animal. As the embryo develops, it goes through a variety of stages. Many of these stages show homologous structures with different species.

If the various life forms developed independently, it would be logical that their embryonic development would be distinct and reflect what the organism would look like when it was fully developed. Why should a bird’s three-digit wing develop from a five- digit limb? This makes no sense if organisms developed independently. The embryological similarities are explained by inferring that these organisms all had a common ancestry. Birds develop their three-digit limbs as embryos from five-digit limbs because they evolved from ancestors with five-digit limbs.

The more similar the early stages of embryonic development, the more closely related the species are or the more recently divergent evolution has caused the species to separate.

Comparing molecules

Advances in the understanding of the biochemical processes of life have provided a wealth of evidence in support of evolution. Biochemical homologies (similarities) provide some of the strongest evidence for evolution because of the detailed level of information they give. Most biochemical evidence for evolution comes from comparative examination of genes or proteins.

Comparing DNA

The best evidence in support of evolutionary theory comes from a study of the base sequences of genes. Comparing DNA sequences examines the relationship between different species, where the more similarities indicate a closer relationship. Darwin’s original hypothesis of common descent (all life evolved from a single life form) is reinforced by this evidence.

If the hypothesis of common descent is true, then species that share a common ancestor will have inherited that ancestor’s DNA sequence. In addition, they will have inherited mutations unique to that ancestor. These specifics can be used to produce timelines that indicate roughly when speciation would have taken place and therefore can be used to create a ‘family tree’ similar to Darwin’s tree of life.

The most detailed DNA sequence reconstructions have been performed using the genomes of mitochondria, which are shared by all eukaryotic organisms and are shorter and easier to sequence than nucleic DNA.

Early DNA sequencing work on the genome of humans and great apes has shown that humans share an ancestor with gorillas and chimpanzees (Figure 2.67). The chimpanzee is our closest living relative, with a 98% similar genome. DNA sequencing of the ß-haemoglobin gene has also confirmed this common ancestry.

Comparing amino acids in proteins

Proteins are coded for by genes and are made up of a string of amino acids (see chapter 1). Proteins range in size from approximately 50 to thousands of amino acids. The characteristics of a protein are determined by the sequence of amino acids from which it is constructed.

All livings things use the same 20 amino acids to make proteins, even though approximately 250 amino acids occur naturally. If life evolved from a common ancestor with only these 20 amino acids, we may expect that these same 20 amino acids are always used.

Protein sequences also show remarkable similarities. A number of different genetic codons can code for the same amino acid, and small changes in some of the amino acids that make up a protein may have little effect on the functioning of the protein. So, we can have a set of proteins that do essentially the same thing but are not identical.

Haemoglobin is an example of variation in amino acid sequences. Several types of haemoglobin molecules are found among different vertebrates and invertebrates. These haemoglobin molecules are all very similar in structure and all serve the function of binding oxygen in the blood, yet they differ in their amino acid sequences.

Insulin is another example. All mammals produce insulin, which helps cells to absorb sugar, but there are slightly different versions among different species. People with insulin- dependent diabetes used to use insulin from animals such as pigs and cows. This substitute insulin worked in the human body, but not as well as human insulin. Since the 1980s, human insulin is produced by genetically engineered bacteria, leading to fewer side effects.

**Textbook 2: NSW Pearson Biology Year 11 & 12**

7.1 Selection pressures: abiotic factors

Organisms do not live in isolation, but interact with biotic and abiotic factors in their environment. In other words, they interact both with each other and with their physical surroundings (Figure 7 .1.1).

To live in a particular habitat, an organism must have access to the basic requirements necessary for growth and reproduction. These requirements are usually met by the organism's environment, which consists of:

abiotic factors-the non-living components of an environment, such as temperature, light and chemical components (e.g. pH, water, gases, soil minerals)

biotic factors-the living components of an environment, such as bacteria, fungi, plants and animals.

The adaptations of organisms enable them to overcome challenges in their environment, such as cold temperatures, excess salinity, lack of water and the threat of predators. All organisms face pressures from biotic and a biotic factors in their environment. These are known as selection pressures.

SELECTION PRESSURES DRIVE EVOLUTION

In every environment, certain organisms will have characteristics that are better suited to specific environmental conditions and give them a survival benefit over other individuals. These organisms are more likely to successfully reproduce under the environmental conditions, passing on their beneficial traits to their offspring. Those organisms that are less suited to the specific conditions are less likely to survive or successfully reproduce, and their genes will not be passed onto the next generation. Therefore, environmental selection pressures drive evolutionary change. Individuals that are well suited to the environment will have higher rates of reproduction and survival, passing on their well-adapted traits to their offspring. This process is called evolution by natural selection and is examined in Chapters 8, 9 and 10.

SELECTION PRESSURES OF THE ABIOTIC ENVIRONMENT

Abiotic pressures are created by non-living components in the environment (e.g. snow and light). Some of these factors are favourable to an organism's survival, while some are detrimental and others neutral. For example, the Antarctic environment, with temperatures ranging between -20°C and -50°C, favours the survival of the emperor penguin (Aptenodytes forsterz), a species adapted to below-freezing temperatures (Figure 7 .1.2). Wind gusts in Antarctica can reach up to 200 km/h, an abiotic factor that emperor penguins can tolerate neutrally, being neither advantaged nor disadvantaged. However, as emperor penguins mostly colonise 'fast ice' (frozen seawater located between larger islands), rising global temperatures due to climate change and expected melting of ice are detrimental abiotic factors.

Abiotic factors belong to the atmosphere, lithosphere and hydrosphere, and create unique selection pressures for organisms as these three spheres interact. The atmosphere is the layer of gases that surround Earth. The lithosphere is the outside layers of Earth, including the crust and upper mantle. The hydrosphere is all the liquid on Earth, including the oceans, rivers, lakes and ice.

Light

Radiation that reaches Earth from the Sun is known as solar energy. Due to the shape of Earth, solar energy is spread unevenly across the planet's surface. The amount of solar energy an environment receives changes with latitude, season and time of day. For example, environments that lie between 30°N and 30°S of the equator receive the greatest amount of solar radiation, and as a result generally have very dry climates.

Plants, as well as some bacteria and protists, use solar energy for photosynthesis. This process produces oxygen used by aerobic organisms, and plays a critical role in food chains and food webs.

Light also affects many plant and animal behaviours and characteristics. Light levels can affect a plant's root growth, promote leaf expansion, and determine pigment systems, such as chlorophyll and phytochrome. In animals, light can affect a species' growth, colouration, migration, reproduction, metabolism and circadian rhythms. For example, humans and other diurnal animals are most active during daylight hours, whereas nocturnal species such as possums are most active at night (Figure 7 .1. 3). This behaviour in response to light conditions is due to circadian rhythms. In mammals, these rhythms are controlled by the brain's hypothalamus.

Temperature

As a result of solar radiation distribution across Earth's surface, as well as variation in climate, microclimate and weather, temperatures differ between environments.

The effect of environmental temperature on an organism is clearly demonstrated in the case of dormancy, a state in which an organism's growth, development and activity temporarily slows, or stops, to conserve energy. Dormancy generally occurs when environmental conditions are not favourable for the organism to use energy. Animals can exhibit short-term dormancy, known as daily torpor, or sustained dormancy, known as hibernation. For example, birds exhibit daily torpor by allowing their body temperatures to drop at night when temperatures are cooler, while species such as the black bear can drop their metabolic rate for several months during the coldest part of the year.

Arctic ground squirrels (Spernwphilus parryiz) respond to below-freezing temperatures in the Arctic tundra by entering a state of hibernation for the 7-8 months of winter (Figure 7.1.4). While most hibernating individuals lower their body temperature significantly, the Arctic ground squirrel takes this to an extreme by lowering its body temperature to sub-zero. The Arctic ground squirrel is the only warm-blooded mammal known to have this ability. The squirrel is thought to dehydrate its body, removing up to 70% of all fluid before hibernation. Without this additional body fluid, ice cannot form internally and the squirrel can safely exit hibernation when the temperature begins to rise again.

In plants, the hormone abscisic acid is responsible for the establishment and maintenance of dormancy. For example, many boreal plant species (plants adapted to subarctic regions in the Northern Hemisphere) exhibit bud dormancy during the winter months when growtl1 is not favourable. The environmental temperature also affects the germination of plant seeds. For example, the Australian banksia species, Banksia elegans, requires at least two minutes in 500°C temperatures to melt the resin on its pods and open the seed follicles to release its seeds for germination (Figure 7 .1. 5).

Weather

Short-term variation in atmospheric conditions, such as temperature, solar radiation, wind, moisture and atmospheric pressure, change the physical environment and can affect the growth, behaviour and reproduction of organisms. This can be seen in a garden, when plant leaves wilt after several days of low rainfall and high temperatures. In the animal kingdom, researchers have discovered that birds can predict changes in weather using their 'internal barometer', which senses subtle changes in air pressure. This affects the birds' behaviour, such as flight patterns and feeding (Figure 7 .1. 7).

Water

While a great amount of water exists on Earth, not all water is readily accessible for an organism's needs. The availability of water in an organism's physical environment will depend on factors such as rainfall, the presence of fresh or salt water, whether water is 'locked' in glaciers, and the amount of water vapour in the air (humidity). Plants and animals have a wide range of structural, physiological and behavioural adaptations to overcome changes in water availability in their environment. These adaptations are examined in Chapter 8.

Aquatic environments are some of the most complex and dynamic ecosystems on Earth. These environments are shaped by many factors, including:

•lithospheric elements, such as the shape of the surrounding landscape, processes of erosion, transportation and deposition, as well as sediments and suspended particles in the water

•energy, in the form of waves and tidal movements

•water chemistry, including fluctuations in oxygen levels, and freshwater versus saltwater environments

•water physics, including factors of specific gravity, heat capacity, turbidity, light penetration and upwelling.

Estuaries are an example of a complex aquatic environment, where marine saltwater is diluted by freshwater where the river meets the sea (Figure 7 .1.8). This environment has high salt levels and waterlogged land. Highly specialised organisms, such as mangroves, have evolved adaptations to tolerate the fluctuating conditions of estuaries.

Shelter

Shelter provides both plants and animals with protection from weather and predators, as well as providing space for growth, development and social activity. Shelter can be formed by biotic elements in the environment, such as a tree providing shade for a lion, or can be abiotic, such as a wombat sheltering in a burrow (Figure 7 .1. 9).

Topography

Variation in topography (the shape of the land) affects water runoff and soil type, and can create microclimates within an environment. For example, aspect (the direction a slope faces) causes variation in the amount of sunlight received by an area of land. In the Southern Hemisphere, slopes with a southerly aspect generally have colder conditions and often receive harsher winds.

As well as aspect, organisms can also be affected by altitude, which is the height of a land mass above sea level. As altitude increases, air pressure and temperature decrease, as well as the amount of readily available oxygen. The purple copper butterfly (Paralucia spinifera) inhabits the central tablelands of New South Wales and will only tolerate altitudes greater than 900 m. It feeds exclusively on the blackthorn tree (Bursaria spinosa), which occurs in this environment and elsewhere in south­eastern Australia (Figure 7 .1.10).

A critical component of terrestrial landscapes is soil. Soil is essential for the exchange of gases, nutrients and water, and for structural stability in terrestrial plants. Soil formation, type and quality are determined by topography, climate, aspect, and the geological or organic material the soil was formed from.

Chemical components

The abiotic environment of an organism includes chemicals essential for ecosystem functioning. Chemical components such as trace elements, heavy metals and pH vary within and between environments. For example, the human stomach is a naturally highly acidic (low pH) environment. Low pH is required to help activate digestion, break down food and kill bacteria and parasites. The bacteria Helicobacter pylori can withstand the highly acidic environment of the human stomach, penetrate the stomach lining and cause ulcers.

The physical environment also involves processes such as nutrient cycles, where essential elements are used, moved and recycled through an ecosystem. Naturally occurring elements, such as carbon, oxygen, hydrogen, phosphorus and nitrogen, are crucial for individual and species survival. For example, nitrogen is harnessed from the atmosphere, lithosphere and oceans, and converted to accessible forms to be used for protein synthesis in living organisms. These nutrient cycles involve biological, geological and chemical components, and are known as biogeochemical cycles.

ORGANISM TOLERANCE

Tolerance is an organism's ability to survive within the physical conditions of an environment. Every organism has a particular range of conditions in which it can survive. This is known as their tolerance range. For each abiotic factor, there is an ideal range that is favourable for the growth, development, reproduction and survival of the organism. Outside its ideal range for a particular factor, an organism will experience stress. Development may be delayed, and health, reproduction and lifespan may be negatively affected. At a certain point outside the ideal range, the organism will die. For example, a particular species may survive between temperatures of 10-40°C, but its ideal temperature for optimum health and reproduction may be 25-30°C. At temperatures below 10°C or above 40°C, death is likely.

Bacteria are particularly susceptible to temperature ranges. For example, while the Salmonella genus of bacteria can survive between 5.2-46.2°C, its ideal temperature for growth and reproduction is 35-43°C (Figure 7.1.11). Meats such as chicken, which often carry the Salmonella bacteria, must be cooked to temperatures over 46.2°C and stored at temperatures below 5.2°C to kill the bacteria and avoid infections that lead to gastroenteritis (food poisoning).

Organisms with a wide range of tolerance are generally widely distributed in an environment, whereas those with narrow tolerance ranges have a restricted distribution. When an organism moves outside its tolerance limits, it can no longer survive. An example of an organism with a very narrow tolerance range is the photosynthetic algae called zooxanthellae, which inhabits coral tissues (Figure 7 .1.12). Zooxanthellae are not widely distributed, being limited to shallow tropical and subtropical waters between latitudes of 30°N and 30°S. They have an optimum temperature range of 16-18°C. The waters become uninhabitable for zooxanthellae when temperatures drop below 16°C for more than a few weeks at a time, or when temperatures rise by more than 1-2°C for more than a few days or weeks.

HUMAN IMPACTS ON THE ABIOTIC ENVIRONMENT

Human activities, such as industry and urban development, affect the atmosphere, hydrosphere and lithosphere, and in turn place new or altered selection pressures on organisms. For example, the atmosphere has been heavily polluted, leading to climate change, thinning of the ozone layer, and decreased sunlight penetration. Similarly, the lithosphere has been gradually polluted and altered, which has increased runoff, salinity and erosion. The hydrosphere also faces new challenges, such as changes in water pH, reduced dissolved gases, rising sea levels, and pollution with toxic chemicals and litter.

These human-induced effects alter the abiotic selection pressures already placed on organisms, as well as introducing new selection pressures. For example, increased levels of carbon dioxide in the atmosphere causing a rise in global temperatures places exaggerated selection pressures on the corals of the Great Barrier Reef.

As mentioned above, corals have a close partnership with photosynthetic zooxanthellae, which give the coral their brilliant colours and provide nutrients. In return, the coral provides shelter for the zooxanthellae. The human-induced rise in global temperatures is placing stress on corals and zooxanthellae, causing the corals to eject the zooxanthellae. Without the zooxanthellae, the coral lose their colour, causing coral bleaching (Figure 7 .1.13).

The relationship between coral and zooxanthellae is explored further in Chapter 11.

7.2 Selection pressures: biotic factors

While organisms are under selection pressures from abiotic factors in their environment, they also experience selection pressures from biotic factors. In an ecosystem, organisms interact and depend upon one another for survival. They influence one another by being part of each other's environment. In this section, you will learn about the biotic factors in an ecosystem and how these factors place selection pressures on organisms in their environment.

ORGANISING THE ENVIRONMENT

Environments can be studied at different levels. The biological levels of organisation listed below follow a structured hierarchy of living things.

Individuals

An individual is a single organism, such as one animal, plant, fungus or unicellular organism. A single Queensland umbrella tree (Scheffiera actinophylla) is an example of an individual (Figure 7 .2.1). This tree usually has many flowers, which produce large amounts of nectar: an important food source for many animals.

Populations

A population is a group of organisms of the same species, living together in a defined geographic area. The spectacled flying fox (Pteropus conspicillatus), also known as a fruit bat, lives in colonies that roost together and interact (Figure 7 .2.2). The fruit bats in a colony could be described as a population.

Communities

A community is an ecological grouping of different species that live together and interact. Many species rely on the Queensland umbrella tree for the nectar it produces, including the spectacled flying fox, various species of bird and the Bennett's tree kangaroo (Dendrolagus bennettianus) (Figure 7.2.3). The diverse species living in and feeding in a population of umbrella trees could be considered a community.

Ecosystems

An ecosystem is a system formed by communities of organisms interacting with one another and their physical surroundings. To be defined as an ecosystem, a system must be self-sustaining. This means that it can be maintained long term, largely without inputs from outside the system.

The Queensland umbrella tree, spectacled flying fox and Bennett's tree kangaroo can all be found in the Daintree Rainforest in Queensland. The Daintree Rainforest is regarded as the most complex ecosystem in Australia. It is a distinct area with many different species (high biodiversity) that interact and thrive together (Figure 7.2.4).

Ecosystems can be almost any size. An ecosystem can be as small as a dead tree trunk, or it can be large, like the Victorian Mallee (Figure 7.2.5).

Ecosystems also vary in complexity. A tropical forest is the most complex land ecosystem and contains the greatest number of species. A desert ecosystem is one of the simplest, because it has fewer different species. Cities and towns are urban ecosystems.

Ecosystems can also be found within ecosystems. For example, rock pools along the seashore are part of larger marine ecosystems, but they can also be studied as small ecosystems (Figure 7 .2.6).

Biomes

A biome is a group of communities that have similar structures and habitats extending over a large area; for example, a rainforest biome, a grassland biome or coral reef biome. The Daintree Rainforest is a part of the tropical rainforest biome. The tropical rainforest biome is close to the equator and experiences high temperatures and rainfall (Figure 7.2.7).

Biosphere

The largest and most complex ecosystem of all is the biosphere, which is the sum of all ecosystems on Earth (Figure 7.2.8). The biosphere includes all those parts of Earth that are inhabited by living organisms, including oceans, rivers, lakes (hydrosphere), soil and rocks (lithosphere) and air (atmosphere). The biosphere occupies a thin layer of atmosphere, the hydrosphere, and a thin layer of lithosphere (Figure 7.2.9).

HABITAT

To study an organism, you first have to know where to find it. The type of place where an organism lives is its habitat. For example, the habitat of rock orchids, as the names suggests, is rocky outcrops in forests. The habitat of a water lily is lakes and ponds. The bush rat lives in forests, while the swamp rat lives mainly in grasslands and heathlands close to water. The European house sparrow, introduced into Australia, makes its home in towns and cities. Different habitats support different species (Figure 7.2.10).

Some organisms live in only one type of habitat. In Victoria, a species of snow daisy is found only in the mountains of the Snowy Range and near Mount Buller. The Sydney rock oyster, although found at many locations along the east coast of Australia, lives along the shore in a narrow band between high and low tide.

Other organisms live in a greater range of habitats. Australian flying foxes inhabit forests, paperbark swamps and mangroves from Queensland to Victoria (Figure 7.2.11). Some organisms move from one habitat to another according to seasonal changes. For example, pelicans move inland from their coastal habitat to take advantage of wetlands that form during times of rain and flood.

Microhabitat

Within a habitat are smaller areas known as microhabitats. A microhabitat could be a burrow, a tree canopy or even the inside of other organisms.

In a microhabitat, an organism experiences a slightly different environment than the overall habitat, such as a lower temperature, more moisture, less sunlight or more humidity. This variation in environmental conditions can be essential for the organism to survive.

The moist trunk of the tree fern (Dicksonia antarctica) in a wet forest is the microhabitat of many mosses, liverworts, ferns, fungi, spiders and insects. The soft trunk of the tree fern is shaded by its umbrella of large fern leaves, and can absorb and hold a lot of water (Figure 7.2.12). The tree fern trunk is a different microhabitat from the trunk of trees such as silver wattles (Acacia dealbata) in the same forest habitat (Figure 7.2.13).

CHANGES TO A SPECIES WITHIN AN ECOSYSTEM

Species are interconnected in many ways within an ecosystem. When a change occurs to one species, other species and sometimes even the entire ecosystem are affected.

Food webs are one way in which species are interconnected. The complexity of a food web provides stability in an ecosystem. In a simple food web, the loss of one species would have a disastrous effect on the other organisms. In a complex food web, the loss of one species has less effect, since alternative food sources are often available. Food chains and food webs are examined further in Chapter 11.

Changes to a keystone species

Some species in an ecosystem can be identified as keystone species. A keystone species plays a critical role in maintaining the structure of an ecosystem (Figure 7 .2.15). When a keystone species is removed, the ecosystem becomes much less stable, and its structure changes. At times like this, the selection pressures in an ecosystem are likely to be significant, with consequences for both the diversity and the abundance of species.

The term keystone species was first applied during a study of food webs in rock pools. The carnivorous sea star Pisaster ochraceus (Figure 7.2.16) was identified as the top predator in the rock pools. As an experiment, all the Pisaster sea stars were removed from one rock pool, with a second rock pool nearby left undisturbed as a control. In the rock pool that the sea stars were removed from, the remaining species competed with each other to occupy the extra space and to use the additional resources made available. Two types of barnacles and a mussel species began to dominate. The barnacles and mussels consumed so much of the limpets' food source (algae) that the limpet population decreased. Within a year, the number of limpet species decreased from 15 to 8. In the control rock pool, there was no change in species number or distribution. Because these significant changes in the ecosystem resulted from the removal of one species-the sea star-it was called a keystone species.

This experiment showed the significant impact of removing a keystone species from an ecosystem. Many other species have since been identified as keystone species. Because of their important role in the structure and functioning of ecosystems, keystone species are frequently targeted for conservation efforts.

The number of the great white sharks (Carcharodon carcharias) (Figure 7. 2 .1 7) has been declining, mostly because they are caught in fishing nets or hunted. This has had far-reaching effects on marine ecosystems. The great white shark is a predator at the top of the food chain, keeping the populations of fish, seal and sea lion species they consume in check, as well as the animals that those species consume. The great white shark is a keystone species that helps maintain the stability of marine food chains.

Another well-known keystone species is the northern quoll (Dasyurus hallucatus), also known as the native cat (Figure 7.2.18). This species has become endangered for many reasons, including bushfires and feeding on poisonous cane toads. The quoll feeds on a large variety of foods, including fruit, insects, birds, mammals and reptiles. Through feeding, the quoll helps control the numbers of its prey species. With the quoll's decline, the delicate balance of those populations is being disrupted.

Keystone species and habitat

Some species are keystone species because they maintain important habitats within an ecosystem. For example, elephants preserve the grasslands of African savannas by eating any young trees that grow (Figure 7.2.19).Without the elephants, the savannas would be invaded by trees and shrubs and eventually become forests or shrublands. The many smaller grazing herbivores, such as wildebeests and zebras, would starve.

HUMAN IMPACTS ON THE BIOTIC ENVIRONMENT

The impact of human activities on the biotic environment also adds to selection pressures, both directly and indirectly. For example, trawling certain fish species can reduce their abundance, which decreases food availability for higher predators, such as sharks, indirectly affecting shark populations (Figure 7.2.20). Humans can directly place selection pressures on organisms through processes such as artificial selection. Artificial selection involves selectively breeding organisms with specific traits to increase the number of offspring with these desired traits. Examples of artificial selection are selecting and breeding hens that lay large eggs, or developing drought-resistant crops. You will learn more about artificial selection in Chapter 9.

By studying biological diversity and understanding how organisms function and interact, scientists can predict how species, populations, communities and ecosystems may be affected by human activities. This knowledge can be applied to help conserve environments and minimise the damage of human activities in the future.

Human impacts on keystone species

Human activities can harm ecosystems, particularly where these activities affect a keystone species. One example is the culling of grey wolves from Yellowstone National Park in the United States (Figure 7.2.21). The wolves were originally seen as a pest, but their eradication allowed a rapid increase in the elk population, which massively overgrazed the aspen and willow plants. This led to a loss of habitat and food for many smaller species, such as beavers and songbirds, as well as stream bank erosion and water sedimentation. In 1995, grey wolves were reintroduced. The ecosystem is slowly recovering.

7.3 Population changes

In theory, populations should continually increase in size as a species produces more individuals. However, this is rarely the case in an ecosystem. Instead, population density and size are determined by a variety of factors that influence rates of birth, immigration (coming into a population), emigration (leaving a population) and death. Population sizes also vary considerably between species (Figure 7. 3 .1). In this section, you will explore the selection pressures that affect the distribution, density and size of populations within an ecosystem. In particular, the introduction of the cane toad and prickly pear to Australia will be examined.

POPULATION DISTRIBUTION

Geographic distribution (or range) is all the places where a species is found. For example, emus are found only in Australia, and kiwis are found only in New Zealand. The rock orchid (Dendrobium kingianum) has a restricted range, because it is only found in parts of eastern Australia (Figure 7.3.2).

To investigate changes in population, an accurate way of measuring their distribution and abundance is required. The method used will depend on the size, mobility and location of the organism. Some populations will be easier to measure than others; organisms that are abundant, less mobile and in easily accessible locations are more likely to be accurately counted than those that are rare, mobile and difficult to access. For example, a population of Acacia bushes is easier to count directly than a flock of galahs in flight. The distribution pattern will also influence the method used to estimate a population's distribution and abundance.

A uniform distribution means individuals are equally spaced apart: for example, penguins on a continental ice shelf (Figure 7. 3. 4). A randomly distributed population arises where individuals are spaced unpredictable distances from each other. This may occur in wildflowers that have been wind dispersed as seeds (Figure 7.3.5). Clumped populations arise where individuals are clustered together: for example, lions surrounding a carcass to feed (Figure 7.3.6).

Factors affecting population distributions

The distribution pattern of a population may be a reflection of the physical environment, the characteristics of the species, or the behaviour of the organism. Each distribution has advantages and disadvantages. For example, clumped populations may have to compete with other individuals for local resources; however, their social setting allows for higher probability of finding a mate.

Geographic distributions may change over time. For example, humans have aided the spread of weeds and animal pests. Humans have also reduced the distribution range of species by clearing forests and interfering with other natural ecosystems.

Prickly pear introduction to Australia

An example of human-induced distribution change is the prickly pear cactus (Opuntia species) (Figure 7.3.7). The term prickly pear includes around 10 members of the Cactaceae family native to the Americas. It is a mostly leafless plant, characterised by fleshy, spine-covered growth and large (often yellow) flowers. The prickly pear's original geographic distribution expanded when it was taken to Europe by the Spanish, and subsequently introduced to Australia by early settlers in approximately 1788. Its distribution rapidly expanded across Australia and it soon became an invasive species.

The prickly pear was introduced to Australia as part of the textile dye industry. A scarlet dye was produced from cochineal insects, which feed on certain species of cactus-one of which was tl1e prickly pear. The insects were harvested on cacti and squashed to obtain the cochineal pigment (Figure 7.3.8). During the 18th century, Spain was the largest contributor to the cochineal dye industry. The scarlet dye was incredibly valuable during this time, with the colour representing wealth, power and royalty. As such, tl1e British government wanted its own supply of this dye, especially for the colouring of its red military coats (Figure 7. 3. 9).

The First Fleet brought the first collection of cochineal-infested prickly pear specimens to Australia in 1788. It was not until the mid-19th century that the cochineal industry ended when synthetic dyes were introduced.

STUDYING POPULATION DENSITIES AND DISTRIBUTION

The density of a population is the number of individuals per unit of area or volume. For example, this might be the number of prickly pear plants in a given area of land, or the number of fish in a particular volume of water.

If it is difficult to count individuals, the size of a population can be measured in biomass. For example, counting blades of grass is a tedious process, so a grass population is often measured in kg per unit area (Figure 7. 3 .10). A small area of grass can be cut and weighed, and then this value can be used to calculate the total biomass of the population.

The area used to measure density might sometimes be represented by a less conventional unit. For example, the population density of plant lice can be directly counted and expressed as the number of individuals on one leaf (Figure 7 .3.11).

POPULATION GROWTH

The size of a population can be affected by four processes:

•births or germination (also called natality) •deaths (also called mortality) •immigration (organisms moving into a population from another population) •emigration (organisms moving out of a population).

Birth and immigration introduce new individuals and thus increase the population size. Death and emigration decrease the population size. Immigration and emigration are collectively known as migration. These four processes determine the rate of change in a population over time (Figure 7.3.12).

Exponential population growth

When populations are not limited by resources, predators or disease, they can experience continual, unlimited growth known as exponential growth. Because individuals continue to reproduce regardless of population size, the rate of population growth increases each generation. In nature, population growtl1 is eventually limited by the carrying capacity of the environment.You will learn more about ecosystem carrying capacity and its effects on populations in Chapter 11.

Theoretical exponential growth

Ecologists use mathematical formulae to model the theoretical growth of a population over time. The graph in Figure 7 .3.13 shows a theoretical growth curve for a population in an ideal environment, which includes all the resources that an organism needs (e.g. food, water, shelter and mates for reproduction).

The graph in Figure 7.3.13 assumes that the number of immigrants equals the number of emigrants over time, meaning that the rate of overall migration is zero. Change in this population is therefore a function of births and deaths only. This type of growth is known as exponential growth. The J-shaped curve of the graph is characteristic of exponential population growth.

As long as the birth rate is higher than the death rate, a population will grow. If the birth rate remains consistently higher, then the population may grow exponentially.

Exponential growth in real populations

Species that tend to experience exponential population growth are those that have a short generation time and give rise to large numbers of offspring. Examples of these species are bacteria, many weed species and some types of insects. In most instances, exponential population growth occurs only for relatively short periods.

Some species may experience exponential growth during certain periods of their lifecycle. Organisms that reproduce during a particular period of the year often have massive increases in their population size during this time, and decreases in population size throughout the rest of the year.

An example of this is a sea turtle. Sea turtles come ashore once a year and bury large numbers of eggs in the sand. When these hatch, the turtle population is very large. However, very few baby turtles make it safely to the water or survive predation in the ocean. Other species reproduce even less frequently.

Another example is that of periodical cicadas (genus Magicicada). They live underground for up to 1 7 years before emerging to reproduce, when the females lay hundreds of eggs in 3 or 4 weeks. The population growth at this time is explosive, but only occurs for a short time (Figure 7.3.14).

POPULATION EXPLOSIONS

Exponential growth is normal for some plants and animals when environmental conditions are favourable and resources are abundant. Because these conditions generally last only a short time, exponential growth is usually short-lived. But if favourable conditions continue, then a population explosion may occur.

Salvinia fern is a free-floating aquatic weed that often has population explosions (Figure 7.3.19). It can survive for up to 20months in dry conditions. But under favourable environmental conditions, such as high nutrient levels, it can double its population every 2-5 days. If these conditions continue, Salvinia will form a dense mat on top of a waterway, preventing other aquatic plant life from receiving sunlight. Due to its growth rate and damage to aquatic habitats, this introduced species is declared as a prohibited weed throughout Australia.

Prickly pear population explosion

The expansion of the prickly pear (Opuntia species) is an example of a population explosion over a long period of time. The species invaded the Australian landscape during its introduction in 1 788 and continued spreading until its control in the 1920s. The species is prone to exponential population growth, and the Australian environment provided favourable conditions for a population explosion.

Bioclimates found in Australia are similar to the prickly pear's natural habitat in the Americas. This provided suitable conditions for population growth and survival, with no species naturally limiting its growth. Reproduction and spread was well facilitated, with new plants quickly establishing from seeds or pads (pieces of plant tissue). The seeds can germinate years after they have been planted, and pads that break off the plant have such large water and nutrient stores that they can propagate after months of being separated from the original plant. Birds feed heavily on the prickly pear fruit, spreading seeds in their droppings. Early settlers also facilitated their population explosion, with plants and seeds distributed throughout Australia through:

deliberate introduction of plants for the cochineal industry

use as food for livestock during droughts (livestock manure provided a nutrient­rich environment for germination)

use as decorative plants in home gardens.

The population explosion of prickly pears was so great that by 1920, more than 60 million acres of land had been infested, and the species was spreading at rates of over 1 million acres a year (Figure 7.3.20).

Early settlers depended on the land for agriculture. Desperate farmers tried everything to remove the prickly pear from their land, including poisoning, burning and crushing the plant. However, the expense of these methods bankrupted many farmers, who were already under pressure during wartime.

From 1880 to 1926, the Australian Government conducted research and provided monetary rewards to anyone who could assist in the control of the prickly pear. In 1926, the cactus moth (Cactoblastis cactorwn) was introduced to Australia from South America. The moth larvae feed on the prickly pear flesh and destroy the plant. It took six years to control the prickly pear population explosion. However, the species still exists in Australia-mostly in cooler areas, where the bioclimate is less favourable for the survival of the cactus moth.

Cane toad population explosion

The cane toad (Rhinella marina) is a large amphibian native to South and Central America (Figure 7.3.22). They are robust, ground-dwelling predators that feed largely on insects. Cane toads have leathery, dry skin and can be grey, yellow, reddish-brown or olive-green in colour. Most adult cane toads reach 15 cm in length; however, the largest female measured was 24 cm and weighed a huge 1.3 kg.

The cane toad was introduced to Australia from Hawaii in 1935 as a biological control method for the control of the cane beetle that was destroying Queensland sugarcane crops. Unfortunately, the cane toad had little effect on the cane beetle populations, because the toads can't jump very high. The beetle simply stayed on the upper limbs of the sugarcane, out of reach of the toads. The cane toads soon spread through the local environment, breeding rapidly and exploding in numbers.

The cane toad has many features that have facilitated its population explosion. Rapid dispersal-The cane toad expanded its range through northern Australia, and is now found as far south as Port Macquarie in New South Wales. It is moving westward at a rate of 40-60 km per year.

Rapid reproduction-Cane toads have a rapid rate of reproduction and breed all year round. They lay eggs in still or slow-moving water and produce more eggs than most amphibians. One female can lay 8000-30 000 eggs at one time, with the eggs hatching in just 2-3 days.

Highly adaptable-Cane toads are adapted to a wide range of environments, which has helped facilitate its exponential growth in Australia. Cane toads are well suited to varied climates and habitat, being found in sand dunes, rainforest, mangroves, grassland and even urban areas. While insects are its staple diet, cane toads feed on many alternative food sources, including household scraps and pet food.

Lack of predators-The cane toad has no current predators in Australia, or diseases to which it is susceptible. Its highly toxic skin secretions are often fatal to any organism that tries to eat it-even freshwater crocodiles.

Environmental impacts

Cane toads impose a range of environmental impacts.

• Cane toads have caused declines in native predators, such as kookaburras (Figure 7. 3 .23), northern quolls and goannas, which die after ingesting the toad. The cane toad also competes with native species for shelter, space and food resources.

• Cane toads pose a risk to human and domestic pet health, due to their highly poisonous skin secretions.

Indigenous Australians using traditional food sources have also been affected, because native species numbers have declined.

Population management and control

Local habitats can be protected by humanely disposing of the cane toads and their eggs. But due to the population explosion of the cane toad and its ability to adapt to its surroundings, a broadscale method of control is unlikely to be effective. Conservationists are currently putting their efforts into protecting the native species affected by the cane toad. This appears promising. Some species, such as the red-bellied black snake, have already demonstrated rapid evolutionary adaptations in response to the introduction of the cane toad.

Population explosions of the cane toad are expected to continue, with researchers finding that the species has developed the ability to reproduce earlier in its life cycle. Toads on the 'invasion front' (those colonising new environments) have faster growth rates and reach breeding size earlier than other cane toad populations. This is helping to drive the cane toad population explosion and increase its distribution across the Australian landscape (Figures 7.3.24 and 7.3.25). You will learn more about the evolution of cane toads in Chapter 10.

8.1 Structural adaptations

Organisms have different features that enable them to survive and reproduce in different environments. These features have evolved in response to various environmental factors, and are known as adaptations. Adaptations enable animals and plants to live in extreme environments, access resources and mates, defend themselves and their territory, and communicate and interact with their own and other species. Adaptations are the result of the evolutionary process of natural selection, in which those organisms that are best suited to their environment survive and reproduce, passing on their advantageous adaptations to their offspring.

Structural adaptations are anatomical or morphological features that improve an organism's ability to cope with abiotic and biotic factors in their environment, increasing their chances of survival and reproduction. These are physical characteristics relating to body size and shape.

STRUCTURAL ADAPTATIONS OF PLANTS

Water is essential for photosynthesis. Therefore, many of the structures found in plants are adaptations to reduce water loss caused by salinity, heat and wind in their environment. Some of these adaptations include:

•reduced leaf surface area

•fewer stomata

•stomatal hairs that create a humid microclimate

•sunken or protected stomata

•thick, waxy cuticle

•extensive root systems

•rolled leaves

•leaves orientated away from sunlight

•leaf abscission (shedding).

Some adaptations to hot, dry environments are very similar to adaptations to cold environments. This is because in very cold climates, water freezes and becomes inaccessible to the plant. For this reason, cold climates are also often dry environments. When the air is cold and dry, plants lose water through transpiration, just as they do in hot, dry environments. Plants that grow in hot, dry environments are known as xerophytes (from Greek 'xeros', meaning 'dry', and 'phyton', meaning 'plant structure').

Structural adaptations to hot, dry environments

Cacti are well-known examples of xerophytes. Xerophytes have adaptations that conserve moisture and prevent the leaf temperature from rising too much. They also have an increased tolerance for desiccation (drying). Some of the adaptations of xerophytes are shown in Figures 8.1.1 and 8.1.2.

Rolled leaves

Marram grasses (Ammophila species) are xerophytes that grow well in the salty, sandy soils of coastlines (Figure 8.1.3a). The leaves of marram grasses are lined with bubble-shaped (bulliform) cells. When conditions are hot and dry, the bulliform cells partially collapse. This causes the leaves to roll inwards so that the two sides of the blade almost touch. Hairs on the inside of the rolled-up leaf trap moisture, creating a humid microclimate (Figure 8.1.3b). The humidity reduces the concentration gradient between the outside and inside of the leaf, which in turn reduces transpiration. Because of this and other adaptations, these grasses have been used to stabilise sand dunes that are prone to erosion.

Leaf orientation

Eucalypt trees have structural features that enable them to survive in hot, dry environments. They have hard leaves with waxy cuticles on both sides to reduce water loss. In many species, the leaves also hang vertically, which reduces the amount of direct sunlight they receive (Figure 8.1.4). This reduces transpiration and water loss.

Structural adaptations to cold, dry environments

Plants in cold environments can protect themselves from water loss through transpiration by reducing the surface area of the leaf. Conifers such as pines (Pinus species) achieve this by growing their leaves as needles (Figure 8.1.6a). Some cold­adapted plants shed their leaves entirely during winter, a process known as leaf abscission. Trees that do this are called deciduous (Figure 8.1.6b). Alternatively, the leaves of cold-adapted plants may have a waxy cuticle to prevent water loss, just like heat-adapted plants.

Structural adaptations to warm, wet environments

Not all plant adaptations are related to conserving water. Some structures are adaptations to help plants survive in environments with excess water. Plants that live in tropical rainforests have to cope with high rainfall and high humidity. Some structural adaptations of plants in tropical rainforests are:

thin bark-plants in tropical rainforests do not need thick bark to prevent water loss

thick, waxy leaves (Figure 8.1.8)-water runs off these leaves quickly to prevent fungal growth in warm, wet environments

a 'drip tip' on leaves (Figure 8.1.8)-a pointed end that funnels water off the leaves and prevents fungal growth

buttressing, stilt roots and prop roots

buttresses are the large ridges at the base of some rainforest trees, while stilt and prop roots are rapidly growing above-ground root systems

buttress and above-ground roots allow large trees to maintain stability in shallow soils

epiphytes ('epi', meaning 'on' and 'phytes', meaning 'plant')-plants that grow on other plants, such as ivy and creepers

- epiphytes can grow in rainforest environments because they climb above the shady undergrowth that would otherwise prevent them getting enough light to grow.

STRUCTURAL ADAPTATIONS OF ANIMALS

All animals have evolved structures that enable them to survive in their environment. These adaptations allow animals to cope with abiotic factors, such as temperature and water availability, and biotic factors, such as predators, prey and competitors.

Some examples of the structural adaptations of animals include: thick fur and blubber (fat) to insulate against cold.

•bright feathers to help attract mates large ears to increase heat loss

•small ears to reduce heat loss

webbed feet and flippers for swimming

spines for protection against predators

overall body shape and size (surface-area-to-volume ratio) to conserve body heat or water

patterned body coverings for camouflage.

Surface-area-to-volume ratio and structural adaptations

If two objects have the same shape but are different sizes, the smaller object will have a larger surface area relative to its volume. You learnt in Chapter 3 how the relationship between surface area and volume applies to cells. This relationship also applies to larger structures and whole organisms.

A larger surface-area-to-volume ratio means that a small animal can cool down and heat up much more quickly; this strategy is well suited to hot, dry climates. A large animal of roughly the same shape will have a greater volume relative to its surface area, meaning that it can more effectively conserve body heat. This strategy is well suited to cold, icy environments.

Cat species are an excellent example of this: compare a tiny desert sand cat (Pelis margarita) (2-4kg) (Figure 8.1.9) with an alpine snow leopard (Panthera uncia) (35-55 kg) (Figure 8.1.10). The sand cat's small size allows it to lose and gain heat quickly in its hot, dry environment, while the snow leopard's large size allows it to conserve heat in its cold environment.

There are variations to this rule-for example, lions are large cats that are found in hot, dry environments. But lions, unlike sand cats, experience advantages due to their larger size, such as the ability to capture larger prey. These benefits outweigh the advantage of being small, so lions must also use behavioural strategies to cool their larger bodies.

The relationship between surface area and volume can also be seen in animals that are the same size but of different shapes. This example is best illustrated by comparing the desert bird, the Kori bustard (Ardeotis korz) (Figure 8.1.11) with the emperor penguin (Aptenodytesforsterz) (Figure 8.1.12).

Both birds are roughly the same size: the Kori bustard grows up to 18kg, while the emperor penguin ranges from 22 kg after the breeding season to 45 kg before breeding. But the Kori bustard has long legs and a long neck, while the emperor penguin has extremely short legs and a short neck. The Kori bustard's long legs and neck give this bird a larger surface area relative to its volume than the emperor penguin. Just like the two different-sized cat species, this means that the Kori bustard will be better able to regulate temperature in the variable environments of its native southern Africa, while the emperor penguin will be better able to conserve body heat in the Antarctic.

Body coverings

The emperor penguin has many structural adaptations to cope with life in the harsh Antarctic climate. Penguins have four layers of thick, scale-like feathers, creating a windproof coat (Figure 8.1.12). They also have thick blubber to keep them warm while swimming in the icy ocean. Juvenile penguins have soft down for insulation, which is a more effective insulator on land than the adult feathers, but of little use in the sea. Juvenile penguins must moult before they can swim.

Vascular body parts

Animals in hot, dry climates may have large ears, long tails or a long body. These extremities are often highly vascular, which means they contain many blood vessels. This enables the animals to release body heat to the external environment, keeping their bodies cool. The fennec fox (Vulpes zerda) of the Negev Desert is an example of a desert dweller with highly vascularised ears (see Figure 8.1.13).

The cardiovascular system plays an important role in regulating the body temperature of animals. When the body is overheated, blood vessels expand, allowing blood to flow closer to the surface (i.e. the skin) and cool. When the body is cold, blood vessels constrict, and the blood flows away from the surface area to conserve heat. This method of thermoregulation is discussed further in Section 8.2.

Tearers and crushers: dental adaptations

Not all structural adaptations are related to temperature. Many structures are adaptations to meet dietary requirements: the arrangement and structure of teeth (dentition) is one of the most obvious of these. Compare the skull of the koala (Phascolarctos cinereus, Figure 8. l .14a) and the Tasmanian devil (Sarcophilus harrisii, Figure 8.1.14b).

The koala is adapted to eat the leaves of eucalypts. Eucalypt leaves are tough because of their high cellulose content, which makes them difficult for mammals to digest. The koala must chew the leaf thoroughly, mechanically breaking down as much of the leaf's fibre as possible before eating it. This is why koalas' molars are flat and wide (Figure 8.1.14a). When the jaw is closed, the molars sit directly on top of one another, making ideal structures for grinding and crushing leaves.

In contrast, the Tasmanian devil is a carnivore (Figure 8.1.14b). The molars are sharper and arranged so that one set sits inside the other when the jaw is closed, creating a shearing, scissor-like effect for tearing meat. You can often draw conclusions about the diet of an animal by examining its teeth and jaws.

8.2 Physiological adaptations

Physiological adaptations affect functioning at different levels of organisation. They can range from the biochemical reactions that take place in organelles and cells to the physiological functions at the tissue, organ, system, or even whole organism level.

PHYSIOLOGICAL ADAPTATIONS IN PL ANTS

Plants live in an incredible range of environments, from hot deserts and high mountain peaks to fast-flowing rivers and coastal zones. They need an equally impressive range of adaptations to cope in what are often stressful conditions. Physiological adaptations play an important role in enabling plants to cope with environmental challenges.

Crassulacean acid metabolism (CAM)

Crassulacean acid metabolism, also known as CAM photosynthesis, is an example of a physiological adaptation that reduces water loss in plants. It is most commonly found in plants living in dry environments, such as succulent plants in deserts. Some xerophytes and some plants adapted to saline conditions can minimise water loss during the heat of the day by using the CAM pathway.

In CAM plants, the stomata open only at night to collect carbon dioxide. Rather than using the carbon dioxide immediately, as non-CAM photosynthesising plants do, the plant stores the carbon dioxide in cell vacuoles as an organic compound called malic acid.

During the day, the malic acid is transported to the chloroplasts, where it is used to produce the carbon dioxide needed for photosynthesis (Figure 8. 2 .1). By storing the carbon dioxide required for photosynthesis at night, the plant can close its stomata during the heat of the day to reduce water loss. This physiological adaptation allows plants to survive in environments of extreme heat and aridity (dryness).

Frost tolerance

Extreme cold can be very damaging-even lethal-to plants that are not adapted to cope with such conditions. Ice crystal formation inside cells bursts the cell membranes, killing the cells. Cold temperatures can also decrease enzyme activity and change the fluidity of cell membranes, both of which affect a wide range of physiological processes in the plant. To overcome these problems, plants living in cold climates have evolved strategies that enable them to tolerate freezing temperatures (Figure 8.2.2).

A high concentration of solutes, such as sugars and salts, lowers the freezing point of water. Plants that can accumulate high concentrations of these solutes in their leaves are therefore less W,ely to be damaged by freezing temperatures.

Some plants produce proteins that reduce the risk of cell damage from freezing. Antifreeze proteins inhibit the growth and recrystallisation of ice crystals by binding to them. Dehydrin proteins bind to water molecules inside the cell, changing the structure of the water and stabilising the cell membrane.

Plants can also change the lipid composition of their cell membranes to improve function in cold temperatures.

Regulation of salinity

High salinity (salt content) is a major problem for many agricultural crops. In many areas, over-irrigation of agricultural land has resulted in highly saline soils, which most food crops cannot tolerate. Saline soils disrupt water and nutrient uptake by the roots by altering the concentration gradient, ultimately suppressing plant growth and starving the plant. When salt enters the plant's cells, it causes ion imbalance, inhibits metabolic processes and eventually leads to cell death.

Plants living in saline environments, such as coastal dunes, salt marshes or salt lakes, have evolved physiological mechanisms to cope with the salinity (Figure 8.2.3). Plant species that can survive high salinity are known as halophytes (from Greek 'halos', meaning 'salt' and 'phyton', meaning 'plant structure'). These plants use a variety of mechanisms to exclude or regulate the concentration of salt in their tissues.

Some physiological adaptations that plants have evolved to cope with salinity include transporting excess salt to vacuoles and old tissue, which avoids the toxic accumulation of salt ions in the cytoplasm, and excluding salt from the roots and leaves. Plants can exclude salt by:

• shedding leaves that are overloaded with salt

• excreting salt from salt glands

• pumping salt out of the roots

• controlling transpiration to avoid excess salt being delivered from the soil to the shoots

• balancing the rate of growth with the uptake of soluble ions to maintain a constant salt concentration in tissues

• increasing water uptake to dilute salt concentrations in tissues.

PHYSIOLOGICAL ADAPTATIONS IN ANIMALS

Animals display an astounding diversity of physiological adaptations. With these adaptations, some species can overcome extreme conditions and exploit seemingly uninhabitable environments.

Examples of physiological adaptations in animals include:

• producing concentrated urine to conserve water in desert animals, such as the spinifex hopping mouse

• producing venom for prey capture or defence in most snakes, wasps, spiders, many marine animals and even some mammals, such as the platypus

• changing colour in response to sunlight to aid in thermoregulation in animals such as chameleons

• shivering to maintain body temperature when cold in endothermic animals, including humans.

Camouflage

Camouflage enables many organisms to blend in with their environment. This adaptation has many advantages, but it is particularly useful for avoiding predators or for capturing prey. One of the most amazing examples of camouflage is seen in the common octopus, Octopus vulgaris (Figure 8.2.9). It can change colour and texture to match its underwater environment, blending in with corals, sand or kelp to hide itself from predators and prey.

The common octopus has specialised colour-changing cells called chromatophores, which enable it to change colour to match its surroundings. Physiological mechanisms move pigment to and from the cells and change their reflective characteristics to produce the camouflage. In addition, tissues under the octopus's skin can create textures to match its environment.

Evaporative cooling

Humans are one of the few animals that produce sweat to cool down. Adults can sweat up to 4 L/hr during vigorous exercise (Figure 8.2.10). Even when not exercising, this physiological adaptation plays an important role in thermoregulation through evaporative cooling. When warm sweat comes into contact with cooler air, it evaporates, carrying heat away and lowering body temperature. It does this through a process of energy (and therefore heat) transfer.

While sweating itself is not a common strategy, evaporative cooling occurs widely in the animal kingdom. Rather than sweating, many mammals employ evaporative cooling in the nasal passages, using secreted moisture to cool warm inhaled air. Other forms of evaporative cooling are behavioural in nature. You will learn about behavioural adaptations in Section 8.3.

Heat exchange for cooling

Heat exchange works in different ways in different animals. Desert ungulates (hoofed animals, from the Latin 'ungula', meaning 'hoof') such as the gemsbok oryx (Oryx gazella) use a heat exchanger to keep the brain cool (Figure 8. 2 .11 a). If the oryx is dehydrated and can no longer afford to lose water, it stops sweating. This causes its body temperature to rise, sometimes as high as 43°C. If blood at such high temperatures entered the brain, the animal would die. To avoid this, the hot arterial blood travels through a smaller network of arteries before it enters the brain. This network of arteries is intertwined with another network of veins and smaller arteries. Because the arteries and veins are so close to one another, they can exchange heat. This network of veins and smaller arteries is called the carotid rete system (Figure 8.2.llb).

The venous blood in the carotid rete system has travelled through the nasal sinuses and has been cooled using evaporative cooling in the nostrils. As this cooler blood from the nostrils passes in the opposite direction to the warmer blood from the body, the heat flows from the hotter blood to the cooler blood in the neighbouring network of blood vessels. This process is known as countercurrent heat exchange. This cools the blood entering the brain by several degrees, enabling the animal to survive in extreme heat and drought.

Heat exchange for heating

Countercurrent heat exchange also occurs in animals living in extremely cold climates, to reduce heat loss and maintain body temperature.

Penguins have heat exchangers in their flippers, feet and tails. These extremities have a relatively large surface area and are exposed to the cold, so they lose heat quickly. Blood from the penguin's feet flows back to the heart through veins close to the arteries. The warm blood in the arteries transfers heat to the veins to warm the blood moving back towards the heart, maintaining the penguin's body temperature (Figure 8.2.12). The blood travelling to the feet is cooled, minimising heat loss.

The diameter of the arteries flowing through the penguin's feet is also reduced to decrease the flow of blood to the extremities and further reduce heat loss. This is referred to as vasoconstriction. In this way, the cells in the feet receive oxygen and nutrients and remain warm enough to function, but less heat is lost to the environment.

Antifreeze proteins

Some fish that inhabit very cold water, such as the Antarctic cod (Notothenia coriz"ceps), manufacture a type of protein that prevents tissue from freezing. The antifreeze proteins, similar to those produced by plants, circulate in the blood of the fish and prevent the growth of ice crystals, keeping their blood liquid (Figure 8. 2 .13).

Deep diving

Some diving mammals, such as the crab-eater seal (Lobodon carcinophagus), can stay submerged at depths of 430m for more than lOminutes (Figure 8.2.14). Diving mammals can store oxygen much more efficiently than other mammals. For instance, some seals can store 70% of their oxygen in their blood, while humans can store only 51 %. The larger oxygen stores in diving mammals are made possible by their larger blood volumes, as well as increased levels of haemoglobin in the blood and myoglobin in muscles. Haemoglobin and myoglobin are proteins that bind to oxygen.

Diving mammals can also carry out anaerobic respiration (i.e., respiration in the absence of oxygen). They have a high tolerance for lactic acid build up, so their muscles can still function efficiently when oxygen stores have been depleted. These animals also have excellent control over their organs, reducing blood flow to those that are not needed for immediate survival, such as the digestive organs, while conserving precious oxygen for vital organs such as the heart and brain. This also reduces the work of the heart, slowing the heart rate dramatically and further conserving oxygen.

Torpor

Torpor is a physiological state in which the metabolic rate is lowered to save energy. This enables an organism to cope with environmental stresses, such as extreme cold or heat or decreased food or nutrient availability.

Torpor can occur over short or long periods. It involves both behavioural adaptations (retiring to a cave or seeking shelter and going to sleep) and physiological adaptations (slowing of the heart, breathing and metabolic rates).

A long period of torpor is often called dormancy, and can be triggered by many different stimuli, including day length (photoperiod), reduced food availability or a change in air temperature. Hibernation, brumation and aestivation are different forms of prolonged torpor. These often involve different metabolic processes and may have different triggers, but many triggers are shared by the three forms of torpor.

Hibernation

Hibernation is prolonged torpor during winter. Over summer and autumn, the animal builds up a thick layer of body fat that will provide it with energy during the hibernation period in winter. During hibernation, the animal can decrease its body temperature and heart rate to conserve energy. Hibernation occurs mostly in mammals, but some species of birds also hibernate. Bears, bats and squirrels are examples of animals that hibernate (Figure 8.2.15).

Brumation

Brumation is similar to hibernation, but involves different metabolic processes. Reptiles such as snakes and lizards undergo brumation. It begins just before winter and can last between one and eight months. How long a reptile remains in brumation depends on the air temperature and the size and age of the animal. Once brumation begins, the reptile eats less or not at all, but wakes regularly to drink.

Aestivation

Aestivation is prolonged torpor in hot and dry conditions. Examples of aestivating animals are snails, frogs, crocodiles, tortoises, lungfish, some insects and some birds. This form of dormancy can be rapidly reversed when conditions change, but it can still continue for prolonged periods. Many aestivating animals move into shady and sheltered locations for the duration.

Green-striped burrowing frogs (Cyclorana alboguttata) are one example of a species that aestivates (Figure 8.2.16). These frogs inhabit semi-arid to arid regions of eastern Queensland and northern New South Wales. They spend up to nine months of the year in aestivation. During this time they live underground in small burrows and do not eat. They can reduce their metabolic rate by up to 80% during this time, allowing them to survive these underground periods just on their store of body fat.

Bioluminescence

Bioluminescence is a physiological adaptation in which light is produced by an organism to attract attention, frighten enemies or lure prey. Bioluminescence is a form of chemiluminescence, which involves the release of light energy following a chemical reaction.

Fireflies, deep-sea fish and sea jellies are examples of bioluminescent organisms (Figure 8.2.17). They produce chemicals called luciferin (a pigment) and luciferase (an enzyme). The luciferin reacts with oxygen to create light. The energy system for bioluminescence is highly efficient, with no excess heat being produced. While most bioluminescent organisms produce light directly, some merely play host to bioluminescent bacteria, which live on the organism and produce light on their behalf.

8.3 Movement and behavioural adaptations

Movement and behavioural adaptations are actions that an organism takes to improve survival or reproduction. Plants have movement adaptations that allow them to move toward favourable conditions and away from unfavourable conditions. In animals, behaviours may be learnt, such as the use of tools in chimpanzees and crows, or instinctive, such as a spider spinning a web. The behaviour of animals can be incredibly complex, but even the simplest behaviours can be critical for the survival of individuals and populations.

ADAPTATIONS FOR MOVEMENT IN PLANTS

Although plants do not have muscles or a nervous system like animals do, they can still move in response to their environment. In most cases, the mechanisms for plant movement are controlled by hormones or turgor pressure, both of which are physiological processes. Therefore, although the end results can share some similarities with the behavioural adaptations of animals, we do not usually refer to plant movements as 'behavioural'.

Plants that are capable of rapid movement rely on internal changes in turgor. Changes in turgor are usually initiated by contact with objects outside the plant. The cells involved are in the parenchyma tissue of the cortex, or specialised swellings (pulvini) at the base of leaves or leaflets. Some movements may be very fast, occurring in less than a second.

Plants can undergo two types of movement in response to environmental stimuli. One is called tropism and the other is called nastic movement.

Tropism

Tropism is plant growth in response to an environmental factor, such as gravity, light or water (Figure 8.3.1).The response depends on the direction of the stimulus. The plant will either grow towards the stimulus (positive tropism) or away from the stimulus (negative tropism). Tropisms are controlled by plant hormones, such as auxin, gibberellin, ethylene and cytokinin.

Types of tropisms include: phototropism-growth in response to light and geotropism or gravitropism-growth in response to gravity chemotropism-growth in response to chemicals thigmotropism-growth in response to touch hydrotropism-growth in response to water concentration.

Phototropism

Phototropism in seedlings is an elegant example of movement in plants, and it owes its effectiveness to a group of hormones called auxins.

Auxins are produced at the tip of the plant, and function by encouraging elongation in plant cells. In darkness, they are spread evenly down both sides of the stem, but the presence of light interrupts their flow. When the light is on one side of the seedling, the auxins become concentrated on the dark side-the side facing away from the light-so that the cells on that side of the plant become elongated. However, the cells on the side facing the light contain less auxin, and do not elongate. Therefore, the dark side of the seedling becomes longer than the light side, causing the seedling to bend towards the light (Figure 8.3.2).

Nastic movement

Nastic movement is a movement of plant tissue in response to an environmental stimulus (but not in the direction of the stimulus). This allows a plant to adapt to changes in its environment by changing its orientation. Some nastic movements in plants are:

• thigmonasty-movement in response to touch

• photonasty-movement in response to a change in light intensity

• thermonasty-movement in response to a change in temperature.

Thigmonasty

Thigmonastic movements include the rapid opening and closing of plant parts in response to touch, such as those observed in the Venus fly trap, Dionaea 111uscipula (Figure 8.3.3).

The Venus fly trap is a carnivorous plant that is adapted to low levels of nitrogen in the soil. It obtains nitrogen by trapping prey such as flies, which it attracts by secreting a sweet sap. When a fly touches the tiny hairs (mechanosensors) on the leaves, an electrical signal is sent to the centre of the trap. This signal opens pores in the trap's lower layer of cells, allowing water to rush in from the cells in the upper layer of the trap. The rapid change in pressure (turgor) causes the cells on the lower side of the trap to expand, forcing the trap to snap shut, trapping the fly inside. Enzymes released by the plant then digest the insect. About one­third of the ATP in the cells is used in each movement. This is why after repeated touches, a leaf will not respond until its energy reserves have been replenished.

Photonasty

The flowers and leaves of many plants respond to changes in light intensity, opening during the day and closing at night or on cloudy days (Figure 8.3.4). This is an example of photonasty.

Thermonasty

An example of thermonastic behaviour is the opening and closing of tulips in response to air temperature. The petals open as the air temperature rises and close when the temperature falls. This behaviour allows the pollen to be exposed only in warmer weather, when pollinators are more likely to visit the flower, and protects it during cooler weather. As in the thigmonastic movement of the Venus fly trap, this movement is a result of turgor pressure.

BEHAVIOURAL ADAPTATIONS OF ANIMALS

Behavioural adaptations in animals that help them to survive m extreme environmental conditions include:

•seeking or leaving shade or shelter •evaporative cooling to lower temperature

•huddling to maintain body temperature •migration.

Seeking or leaving shade or shelter

Many desert animals regulate the rate of heat exchange with their environment by seeking shade when the heat is too great and leaving it when temperatures fall. The central netted dragon (Ctenophorus nuchalis) is a good example of such a behavioural adaptation. To raise its body temperature, the lizard emerges from under a rock and basks in the sunshine, spreading itself out at right angles to the Sun's rays. To lower its body temperature or reduce the rate of increase in body temperature, the lizard orientates its body parallel to the Sun's rays, minimising the exposed surface area, or simply retreats beneath a rock or into a burrow (Figure 8.3.6).

Some animals, such as desert snakes and tortoises, adopt nocturnal behaviour during summer to prevent overheating. They move only in the cooler evening, avoiding the extreme heat of the day. Animals may also seek shelter to increase their body temperature when it is cold or windy.

Evaporative cooling

Many land animals use evaporative cooling to lower their body temperature by releasing heat into the environment. Although this is a physiological adaptation (as explained in Section 8.2), it is often achieved by behavioural adaptations, such as:

•panting or licking limbs •spraying water on the body •mouth gaping •wallowing in mud or water

•gular fluttering •urohydrosis.

Panting or licking

Panting or licking limbs enable animals to release heat effectively using evaporative cooling. For example, kangaroos lick their paws, and animals such as dogs, gazelles and foxes pant. The fennec fox (Fenecus zerda) has been observed panting at a rate of 690 times per minute after chasing prey.

The rate of panting is proportional to the amount of air flowing over the tongue.

If animals can flatten their tongue to increase its surface area while increasing their panting rate, then the cooling effect is greater. Sometimes even penguins have to pant. In warmer weather, they also hold their flippers out of the water so that both surfaces are exposed and can release heat via evaporative cooling.

Spraying water

Elephants commonly spray water on their body to cool off via evaporative cooling (Figure 8.3.7). Mud remaining on the elephant's skin provides protection against solar radiation. Water spraying behaviour is also used by many other animals.

Mouth gaping

Mouth gaping is seen m many animals, such as crocodiles and alligators (Figure 8.3.8). This behaviour allows air to move across the moist surface of an open mouth. Evaporative cooling from the membranes inside the mouth reduces the temperature of blood being supplied to the brain.

Wallowing in mud or water

Wallowing in mud or water is a very common behaviour. Animals such as pigs, elephants, rhinoceroses and deer wallow in wet mud to cool the skin, while animals such as hippopotamuses, tapirs, bison, horses and cattle wallow in water.

Wallowing in mud has many advantages for animals, including skin maintenance, camouflage, parasite control, protection from solar radiation and social play. One of the more common reasons is thermoregulation. Like sweating, the evaporation of the water in the mud cools the animal's skin by carrying heat away from the body. It can cool the animal's body by up to 2°C, making it more efficient than sweating. Wallowing in mud has an advantage over water too; the water in the mud evaporates more slowly than water alone, keeping the animals cooler for longer (Figure 8.3.9).

Gular fluttering

Gular fluttering is a cooling behaviour in which birds flap membranes in their throat to increase evaporation from the moist buccal (mouth) region. As the air temperature increases, birds increase the amount of gular fluttering.

Urohydrosis

Urohydrosis is a cooling behaviour exhibited by some birds, including vultures and storks. They urinate on their legs, creating an evaporative cooling effect.

Huddling

Many animals, such as penguins, huddle to cope with cold temperatures (Figure 8.3.10). Thousands of emperor penguin chicks may huddle together for warmth in the spring, when they begin to develop their adult plumage. By huddling, penguins decrease the surface area of the group exposed to the harsh environment. They continually rotate the animals on the outside, each taking a turn in the freezing cold winds.

Migration

Some animals move extremely long distances each year to inhabit a different area. This type of seasonal pattern of relocation is known as migration.

The purpose of migration is usually to seek better food availability, to move to a better site for breeding, or to find suitable climatic conditions. Birds navigate their migratory paths using the position of the Sun and Moon, as well as topographical details and cues from Earth's magnetic field. Migration is an innate behaviour prompted by cues from the environment, such as the length of daylight. These cues are closely coordinated with an animal's biological clock and trigger biological responses, such as increased feeding before migration.

Migration can occur on a number of different scales. For example, humpback whales of the Southern Hemisphere migrate vast distances on an annual basis. They spend the warmer southern months in Antarctic waters, feeding on krill blooms. In autumn, they migrate to warmer waters in the tropical Pacific for calving and breeding. African elephants (Loxodonta aji-icana) also migrate over long distances, travelling more than 80 km annually in search of food resources (Figure 8.3.11).

By contrast, zooplankton in all the oceans of the world exhibit what is called diurnal vertical migration. This means that they migrate daily from the bottom of the ocean to the surface. At sunset, zooplankton rise to the surface to feed on the phytoplankton that live in the surface waters. However, during the day, the zooplankton would be visible to predators, so they return to the depths at sunrise. In terms of biomass (the total mass of all the organisms involved), this is the largest mass migration in the world.

8.4 Forming a theory: Charles Darwin and natural selection

We now accept the theory of evolution by natural selection as the best explanation for the origin of species and how they adapt to their environment. However, people once believed that all organisms were created in their current forms. By the 18th and 19th centuries, many people were no longer satisfied with this explanation. The question of how so many different species came to exist, and how they were so well adapted to their particular environment and lifestyles, puzzled many great thinkers of the time.

Several different scientists tackled the question and contributed to the model of evolution we now accept (see Additional box on p. 372). But it was Charles Darwin (1809-1882) who most famously publicised the principles of evolution by natural selection. The story of Darwin's discoveries and the formation of his theory is one of the most important stories in the history of scientific thinking.

CHARLES DARWIN AND THE VOYAGE OF THE HMS BEAGLE

Charles Darwin (Figure 8.4.1) was an English naturalist who sailed on the HMS Beagle. He made numerous biological and geological observations on the voyage. He also collected specimens from every location that the ship visited. Many of these specimens can still be seen in museums around the world (Figure 8.4.2).

The second voyage of the HMS Beagle (Figure 8.4.3a) was nearly five years in length. Departing England in December 1831 and returning in October 1836, the voyage surveyed South Africa, large portions of southern America, Tahiti, Australia, New Zealand and, most famously, the Galapagos Archipelago (Figure 8.4.3b).

During the voyage, Darwin-a young man at the time-collected his most crucial initial observations. These observations would later influence the formation of his theory, which he would neither publish nor publicise for many years. Like any good scientist, Darwin took extensive notes on his observations. By reading these notes, we can follow the progress of his ideas. These ideas eventually came together to form his theory of evolution by natural selection, which Darwin published in his book, entitled On the Origin of Species by Means of Natural Selection (1859).

Finches of the Galapagos Islands

When Darwin first examined tl1e variety of birds found across the Galapagos Islands, he had no idea that most of them were finches and belonged to the same family (Thraupidae). It was only when he returned to England that John Gould, the famous ornithologist (bird specialist), demonstrated that these species were slight variations of each other. Some, such as the warbler finch, had narrow pointed beaks; others, such as the medium ground finch, had strong wide beaks (Figure 8.4.4).

Darwin used John Gould's observations of beak size and length, along with his own records of the types of food available on each island, to develop his theory of evolution by natural selection. The differing forms of the beaks were the traits (inherited physical characteristics) altered by natural selection.

Each island of the Galapagos Archipelago has a different environment, with different foods available: from cacti to large seeds. These environments provided a variety of selection pressures for birds that migrated between the islands. For example, if a warbler finch, which has a slender beak, migrated to an island with only large seeds and few insects, it would struggle to find enough food to eat, resulting in selection pressure and adaptation to the local environment over time. Table 8.4.1 shows some of the Galapagos finches that diverged from a common ancestor and adapted to their particular environment and ecological niche.

The famous example of the Galapagos finches is significant on several levels. First, it is a prime example of adaptive radiation, when a single species evolves into several species by adapting to the requirements of different environmental niches (Table 8.4.1). Second, Darwin did not realise how important his observations would be at the time, but his collection of data would turn out to support his theories later on. This is often the case in science. Finally, without Gould's input on tl1e relatedness of the birds, he would never have understood their significance. Scientists often rely upon one another's expertise to place data in context and draw reliable inferences.

Australian flora and fauna

Darwin visited Australia in January 1836, in the latter part of his journey. He was struck by the apparent strangeness of the Australian landscape.

"A little time before this," he wrote in his diary, "I had been lying on a sunny bank and was reflecting on the strange character of the Animals of this country as compared to the rest of the World."

By this time, Darwin had already observed that animals that occurred in neighbouring environments bore a strong resemblance to one another, regardless of whether or not those environments were similar. This puzzled him. In On the Origin of Species, Darwin wrote:

"Why should the species which are supposed to have been created in the Galapagos Archipelago, and nowhere else, bear so plain a stamp of affinity to those created in America? There is nothing in the conditions of life, in the geological nature of the islands, in their height or climate, or in the proportions in which the several classes are associated together, which resembles closely the conditions of the South American coast: in fact there is a considerable dissimilarity in all these respects."

In other words, the species of the Galapagos Islands and South America appeared to be similar in spite of facing very different environmental challenges. We now know that this is because they all shared a recent common ancestor before evolving their different adaptations.

During his visit to Australia, Darwin observed several platypuses playing in a river (Figure 8.4.6a). He noted that they occupied a similar environmental niche to the English water rat (also known as the European water vole; Figure 8.4.6b). He was struck by an observation: animals that existed in very similar environments might bear almost no resemblance to one another at all.

We now know that this is because the water rat and the platypus do not share a recent common ancestor. The finches of the Galapagos Islands are closely related to finches of South America, and so they look similar. The water rat and the platypus do share some similarities: they are both mammals, both furred, both excellent divers and swimmers, and both consume plants that grow in or near the water. However, unlike the finches of the Galapagos Islands and South America, the platypus and the water are very different in many ways. For example, the platypus is a monotreme (an egg-laying mammal), while the water rat is a placental (a mammal that carries its young in utero). This important biological difference indicates that the platypus and water rat have been separated by millions of years of evolution.

Looking over his notes, Darwin would later consider these two observations: animals from the same region could closely resemble one another, even if they existed in very different environments animals in distant regions could look very different, even if they existed in very similar environments.

These two observations are key to understanding the theory of evolution by natural selection.

THE MODERN MODEL OF NATURAL SELECTION

The model of natural selection that we use now has been refined and altered a great deal since Darwin's initial publication. While he proposed a mechanism of natural selection that drove species to adapt to their environment and separate from one another over time, he could not explain how characteristics were inherited across generations. Genetics did not yet exist as a field of study, and Mendel's influential work on peas would not be discovered until nearly 20 years after both Darwin and Mendel had died. We have now added our understanding of genetics and heredity into Darwin's original model, and we therefore have a much more detailed understanding of how natural selection works. In this context, natural selection and its role in the evolution of life will be examined in more detail in Chapter 9.

Selection pressures and genetic variation

There is always variation between individuals within a population. Genes come in different forms. We use the term allele (gene variants) to describe these different forms. For example, a gene that affects hair could exist as an allele that produces curly hair, or an allele that produces straight hair. The various combinations of alleles in an individual make up their genotype (also known as an individual's genetics or genome).

The genotype, together with the environment, determine an individual's observable traits. These are also known as their phenotype (an individual's physical characteristics). For example, curly hair is a phenotype, while the combination of alleles that determine curly hair is a genotype. Another example is coat colour in dogs. Different combinations of alleles result in different coat colours (i.e. phenotypes) (Figure 8.4.10).

The total genetic variation in a population-that is, all the alleles that exist in that population-is referred to as the gene pool. The variation in the gene pool can also arise from random mate selection, recombination during gamete formation, the independent assortment of alleles during cell division, and random mutations. These factors, and the individual differences in gene expression and environmental factors, can lead to differences in phenotypes.

Should a particular phenotype give an individual a survival advantage, that phenotype, and the genes and alleles that control it, is more likely to survive in the population. Successful organisms are more likely to breed, and those phenotypes will be inherited by their offspring, which in turn will be more successful than those with less useful phenotypes. For example, thicker-furred animals might be more successful at surviving and breeding in cold environments. Animals with thinner fur might be more tired and weak from cold, and therefore will be less able to survive, mate and rear young. Their offspring are also likely to inherit their less successful phenotypes and face many of the same problems.

The conditions or factors that influence which phenotypes are most successful in a population-and therefore, influence allele frequency in that population are known as selection pressures. Selection pressures, together with mutation, are the driving force of evolution. Selection pressures can be natural environmental pressures or artificial pressures brought about by humans through selective breeding. You will learn more about artificial selection in Chapter 9.

Natural selection

Natural selection is the influence of environmental pressures on allele frequency in a population. Environmental selection pressures affect the survival and reproduction of an organism. Individuals with the most advantageous phenotypes have an increased chance of producing viable offspring. Viable offspring are offspring that are fertile and able to survive and breed the next generation.

Examples of environmental selection pressures include:

climatic conditions, such as extreme temperature changes and drought (Figure 8.4.11)

competition for resources, such as food, water, shelter.

mate availability

predator abundance.

The allele frequencies of a gene pool are heavily influenced by environmental pressures. This is because of the following factors.

Variation-there are genetic differences between individuals of a population.

Reproduction-organisms can reproduce and alleles are heritable. The offspring are genetically similar to parents (if sexually reproducing) or genetically identical (if asexually reproducing).

Survival-not all individuals survive long enough to reproduce and produce offspring.

Environmental selection pressures-some phenotypes are better suited to the environmental conditions and give the individual a survival and reproductive advantage over those of a different phenotype.

When it comes to survival, some phenotypes (traits) have a high adaptive value and give the individual an advantage over individuals with phenotypes of lower adaptive value. This concept is often referred to as 'the survival of the fittest'. Having an advantageous trait means the individual is more likely to survive to reproduce and pass their alleles on to the next generation.

Alleles for the advantageous trait tend to increase in frequency in the gene pool, while alleles of the less advantageous trait tend to decrease. Advantageous traits of high adaptive value may persist in the population until all individuals have the alleles for this trait (100% allele frequency). Over time, the population evolves and adapts to its environment.

Thorny devils (Figure 8.4.12), for example, have physical adaptations that enable them to thrive in the very arid ecosystems of central Australia. Their mottled camouflage colouring and hard spikes have high adaptive value, because these features reduce the likelihood of predation. Thorny devils also have highly textured skin, which allows capillary action to collect any moisture in their environment and channel it directly into their mouths.

9.1 Evolution and biodiversity

Evolution is the change in the genetic composition of populations over time. This can be observed as changes in allele frequencies (gene variants) and phenotypes (physical traits) in a population. New species can evolve in response to changes in environmental conditions or after populations become isolated and accumulate genetic differences. Biodiversity (the diversity of life) increases as genetic changes result in new genetic variation and the divergence of populations and species. In this sense, evolution promotes biodiversity. However, evolution can also lead to the loss of biodiversity, through the extinction of alleles, populations and species.

In Chapter 8 you learnt about the important contributions of Charles Darwin (Figure 9 .1.1) and Alfred Russel Wallace (Figure 9 .1.2) to our current understanding of evolution. In this section you will learn how their theory of evolution by natural selection accounts for different evolutionary processes and the diversity of life on Earth.

A MECHANISM FOR EVOLUTION: NATURAL SELECTION

In July 1858 two celebrated naturalists, Charles Darwin (Figure 9.1.1) and Alfred Russel Wallace (Figure 9.1.2), jointly presented a theory to the Linnean Society of London, which proposed a mechanism for species change. The two men had not worked together and had independently arrived at the same theory. The following year, Darwin published the theory in his book, On the Origin of Species by Means of Natural Selection (Figure 9.1.3).

**The theory of evolution** by natural selection proposed that species were not created in their present forms but had evolved from ancestral species. The work also proposed a mechanism for evolution, termed natural selection, based on two key observations. 1 Members of a population often vary in their inherited traits (Figure 9 .1.4).

Based on these two key observations, Darwin and Wallace drew two inferences.

1 Individuals whose inherited traits give them a higher probability of surviving and reproducing in a given environment tend to leave more offspring than other individuals.

2 This unequal ability of individuals to survive and reproduce will lead to the accumulation of favourable traits in the population over generations.

The theory of evolution by natural selection, also called Darwinian theory or Darwinism, is relatively simple to express, yet the processes it seeks to explain are complex.

At the time of the theory's publication, both Darwin and Wallace were unaware of the genetic basis of heritable traits and the mechanisms of heredity. Now we know what they did not: that the physical form (phenotype) of an organism is an expression of its underlying genetic information (genotype).

Individuals with the most advantageous phenotypes (traits) have an increased chance of producing fertile offspring. When it comes to survival, some phenotypes have a high adaptive value and give the individual an advantage over individuals with phenotypes of lower adaptive value. This concept is often referred to as 'the survival of the fittest'. Fitness refers to an organism's suitability to its environment. Having an advantageous phenotype means the individual is more likely to survive to reproduce and pass their alleles on to the next generation.

Every species that exists today has experienced evolution by natural selection. This can be seen in the specialised adaptations that enable organisms to survive in their environment. The addax (Addax nasomaculatus) is an example of an animal that is extremely well-adapted to its desert environment. The addax that are best­suited to hot, dry conditions will survive and reproduce; that is, they will be selected (Figure 9 .1.6). The environmental conditions select the phenotypes that are well­suited to those conditions, enabling organisms with those phenotypes to live and reproduce in that environment. Individuals with traits that are not suited to their environment are less likely to survive or reproduce, and so are removed from the breeding population. What remains are the individuals that are suited to that environment.

Over successive generations, a greater proportion of the population expresses the well-suited traits (adaptive phenotypes) and a small evolutionary step has taken place.

Natural selection in action

Looking at changes in biological diversity, there are many examples that demonstrate the mechanisms of Darwin and Wallace's theory of evolution by natural selection. Further evidence and examples will be covered in Chapter 10.

Insecticide resistance

Often when farmers start using a chemical insecticide to protect their crops, most of the insects have no defence and die. Yet a few are naturally resistant; the chemical does not kill them. The resistant individuals breed and pass on their resistant traits to some individuals in the next generation. Sexual reproduction results in parental genes being recombined in the offspring in new sequences; therefore, two surviving, resistant parents can still produce non-resistant offspring. The resistant individuals in the next generation again survive the insecticide, and go on to breed as well. In each subsequent generation vulnerable individuals die while those fit for the environment survive. With each generation the proportion of the insect population carrying the resistant trait increases, eventually approaching 100%.

The development of genetic resistance over many generations has been documented in numerous species. For example, DDT insecticide resistance in malaria-carrying mosquitoes, antibiotic resistance in many disease-causing bacteria and resistance to the disease myxomatosis in Australian rabbits. While not always about insecticides, these are similar examples of natural selection at work.

Lactose tolerance

Several thousand years ago, humans began domesticating cattle. Previously, the hunted wild animals had been a source of meat only, but domestication also made milk readily available. At first, a very high proportion of the human population was lactose intolerant, and unable to digest that component of the nutritious new food. Yet the fortunate few who could prospered, reproducing more successfully than the lactose-intolerant proportion. The frequencies of the alleles for lactose tolerance increased in the population. Today, lactose intolerance is rare in populations that have dairy in their diet, whereas populations that have little to no dairy in their diet have high levels of lactose intolerance. Populations in East Asia, Central Asia, Africa and southern India are examples of populations in which over 70% of people are lactose intolerant.

In all cases alleles of the advantageous trait tend to be more frequent in the gene pool, while alleles of the less advantageous trait tend to decrease. Advantageous traits of high adaptive value may persist in the population until all individuals possess them. Over time, the population evolves in response to environmental changes. This point is a key difference from Lamarckism (the theory of Jean Baptiste Lamarck), which proposed that the individual evolves.

Darwin noted the similarity of natural selection to artificial selection. The difference is that in artificial selection, human breeders take natural species and select traits they want retained in the next generation. These may include above­average milk or egg production, tameness in animals or sweetness in fruits. This can produce a new population substantially different from the original over a short period of time.

In contrast, natural selection does not operate by human design, and species usually change according to ecological factors. Such factors may include climate, competition for resources, predation or many others. Yet the potential for physical and other change in species due to natural selection is immense. This process has been responsible for nearly all change to life on Earth.

Summary of natural selection

**Natural selection** is one of the mechanisms of evolution. The concept of natural selection is quite simple-individuals with traits that are well-suited to their environment survive and reproduce-but the way it functions in nature can be complex. The following points summarise the process of natural selection:

Natural selection does not involve intent. Organisms never plan their eventual outcome, not even the most sophisticated camouflage or mimicry. Natural selection merely rewards whatever has already survived with ongoing survival: it does not estimate the likely chance of survival in the future.

•Consequently, selection does not work for the survival of the species. The mechanism favours individual survival and reproduction.

•Natural selection does not always lead to greater complexity or sophistication. Sometimes selection can lead to simplification, as in the loss of useless eyes in cave-dwelling or deep sea animals.

Darwinian fitness does not mean athletic fitness. Fitness in an evolutionary context means suitability for a particular environment.

Genetic variation not affecting the phenotype is selectively invisible and may accumulate over time.

Not all traits serve a survival function. For example, human earlobes seem to have no purpose. Some traits are simply effects of developmental processes or other characteristics.

•Natural selection does not produce perfection. Most often, selection favours 'good enough' solutions that may be inelegant or inefficient.

•The evolution of complex structures such as eyes always proceeds in stages, each providing some advantage (Figure 9.1.8).

PUNCTUATED EQUILIBRIUM

Natural selection is commonly misunderstood to mean constant, directional change.

Darwin was also guilty of this mistake; he predicted that the evolution of species occurs gradually and that the fossil record would reflect this, with transitional forms of species as they underwent evolution from one form to another. Although such forms do exist in the fossil record, more than 70% of fossil sequences show relatively rapid change, rather than gradual change. Evidence of gradual evolution in the fossil record is comparatively rare, implying that most evolution must be rapid.

A modification to Darwinism, called punctuated equilibrium, explains rapid evolutionary change.

**The theory of punctuated equilibrium** predicts that over geological time, the main selection pressure on a species will be for stability. Once an organism is well-adapted to its environment, selection acts to maintain the well-adapted traits. The fossil record for that species would then show long periods of no change (the equilibrium).

Yet such periods are punctuated with short bursts of very rapid change to a new stable form. When evolution does occur, it is still gradual, but occurs so rapidly that the transitional forms are seldom preserved in the fossil record. Hence the fossil record mostly shows sudden jumps; these sudden changes punctuate the equilibrium.

Sudden changes in the environment cause rapid evolutionary change. A species well-adapted to the previous environment is vulnerable during environmental change and so is under intense selection pressure to evolve and adapt as rapidly as possible. This rapid evolutionary change has been seen in the cane toad since its arrival in Australia just over 80 years ago.

TYPES OF EVOLUTION

Coevolution

Species that interact closely exert selection pressures on each other. Both species also experience similar environmental conditions. In such situations coevolution can be seen, with the two species evolving together in a reciprocal response to selection pressures.

Coevolution is often seen in flowers and their pollinators. New variations of flowers appear through mutation, and these may be more likely to survive and produce seeds. As a result, some pollinators will be more suited to these flowers, and will therefore evolve alongside the flowers (Figure 9 .1.12). Coevolution can also be observed in predator-prey relationships. When predators pick off the weaker prey, stronger individuals are left to reproduce. The next generation of predators will need to be stronger and faster to keep up with the stronger prey.

Parallel evolution

Parallel evolution is the evolution of similar features in related species that have experienced similar environments and selection pressures. For example, the similarities in the colouration of different bird species that live in similar environments.

Convergent evolution

Convergent evolution is the evolution through natural selection of similar features in unrelated groups of organisms (Figure 9.1.13). Unrelated species that have adapted to a particular environment in similar ways are said to have converged, or become more alike.

The Australian marsupial sugar glider (Petaurus breviceps) and the American placental flying squirrel (Glaucornys sp.) have both developed large membranes between their fore and hind limbs that enable them to glide successfully (Figure 9 .1.14). This is an example of two unrelated species that have converged due to similar environments and lifestyles despite different origins.

In general, similar selection pressures tend to produce a strong resemblance among unrelated species.

The process of convergent evolution can also produce similar-looking features from entirely different ancestral structures. For example, cephalopod (the order containing octopus and squid) and vertebrate eyes outwardly look similar (9 .1.15). Yet the similarity is superficial; examination of the differences shows that natural selection has produced the same result using different tissues and in a different arrangement. Relative to the lens, vertebrate eyes have the nerve fibres in front of (i.e. over the top of) the light-sensitive retina, while the nerve fibres in the cephalopod eye are behind the retina (Figure 9 .1.15). In vertebrate eyes, the nerve has to pass through the retina on the way to the brain, resulting in an inefficient blind spot. Cephalopod eyes lack this limitation.

9.2 Speciation and microevolutionary change

The ability to interbreed defines species. Interrupting breeding and the exchange of alleles through isolation can lead to the evolution of new species.

Gene pools can change when new individuals join the population from a different gene pool or when some individuals leave a population. Such migration of individuals can result in gene flow.

When gene flow exists between two different populations, the gene pools may remain fairly similar. When gene flow is absent between populations, the gene pools are said to be isolated.

Different selection pressures and different mutations in the separated populations cause them to become genetically different. Eventually, the separated populations may accumulate different characteristics, become reproductively isolated and be recognised as· two new species. The new species may be subject to different selection pressures and so the gap between them will widen; they will diverge.

Genetic isolation of one species from another can be a result of one or more mechanisms. In this section you will learn that these mechanisms can act before reproduction (prezygotic) or after reproduction (postzygotic).

SPECIES

A species is the largest group of individuals that can breed with one another. In order to be considered members of the same species, individuals must be genetically similar enough to produce fertile viable offspring (Figure 9.2.1). A species can also be thought of as a gene pool that is isolated from the gene pools of other species.

While this definition of species fits most groups of organisms, there are exceptions. For instance, organisms that reproduce asexually, particularly single­celled organisms, can be difficult to categorise into discrete species. Also, species covering a wide geographic range may vary subtly over that range. Adjacent populations may freely interbreed, but the two extreme ends of the range may be genetically incompatible.

The current definition of species is also problematic because of another important fact: species change.

Fossil evidence shows that many species existed in the past that do not now, and that the species we see today did not exist in the past. Thus, species come and go. Fossil evidence also shows that while species exist, they can take on different forms over time.

Around the end of the 18th century, it was accepted that species change (evolve). However, the scientific community did not know the mechanisms by which the changes occurred.

PREZYGOTIC ISOLATING MECHANISMS

**Prezygotic isolating mechanisms** are those that prevent individuals from different species from interbreeding (producing fertile offspring). Prezygotic isolating mechanisms may prevent individuals coming into contact, prevent mating when they do come into contact or prevent fertilisation if mating occurs. There are a variety of prezygotic isolating mechanisms that work to prevent interbreeding at different stages.

Geographical (spatial) isolation

Populations may be separated by physical and geographical barriers, such as oceans, deserts, mountain ranges and glaciers. For example, the southern boobook-(Ninox boobook) is an Australian owl that is genetically distinct from the New Zealand owl, morepork (Ninox novaeseelandiae) (Figure 9.2.3). One reason that they are genetically isolated is that the Tasman Sea separates them.

Ecological isolation

Populations occupy different ecological niches within the same ecosystem. For example, brown stringybark (Eucalyptus baxterz) and Mt Abrupt stringybark (Eucalyptus verrucata) are closely related species that grow side-by-side in the Grampians, in Victoria. Mt Abrupt stringybark grows on upper slopes on rocky sites and brown stringybark occurs on lower slopes on deeper soils. The two species are usually reproductively isolated but sometimes their flowering times overlap and neighbouring trees will interbreed. Hybrids (the offspring of two different species) are fertile, but are generally found only along the border between the two species. The obvious boundary between species can be seen in Figure 9.2.4.

Temporal isolation

The breeding cycles or active times of populations do not overlap. For example, a nocturnal animal is unlikely to breed with a diurnal one. Likewise, many similar plant species will flower at slightly different times of the year, preventing cross-pollination.

Behavioural isolation

This occurs when behaviours such as mating calls and courtship rituals are different. This isolating mechanism is only possible in animals. An example is mate attraction to different types of vocal signals, such as bird songs or frog calls, which are unique to species. Behavioural isolation is often the result of sexual selection (see page 397).

Structural or morphological isolation

The reproductive organs of different species are physically incompatible and individuals are unable to mate. For example, a sparrow could not breed with an albatross. For more similar species, even slight differences can prevent mating, such as the different breeding pheromones produced by different moth species.

Gamete mortality

This occurs after mating has taken place. Egg and sperm (gametes) fail to fuse in fertilisation and a zygote does not form. For example, the sperm of one species may not be able to recognise the egg of another without the appropriate signalling molecules, or the conditions of the female reproductive tract of one species may not sustain the sperm of another species. Pollen may not germinate on the style of the flower of another species due to a chemical barrier, preventing the plant's sperm from reaching an ovum.

POSTZYGOTIC ISOLATING MECHANISMS

Postzygotic isolating mechanisms are those that typically prevent a zygote of two different species from developing into a fertile adult. The offspring resulting from interbreeding between individuals from different species are called hybrids.

**Hybrid inviability** is a mechanism of reproductive isolation in which the sperm from one species successfully fertilises the egg of another species to form a hybrid zygote, but the hybrid zygote has unmatched chromosomes. As a result, normal embryonic development cannot proceed because of the lack of homologous chromosome pairs in the zygote. The zygote does not usually survive long.

Sometimes the zygote survives and undergoes cell division but the offspring does not develop fully and will not reach adulthood. This is known as reduced hybrid viability. Most hybrids that do develop into adulthood are sterile; that is, they are incapable of producing offspring themselves (Figure 9.2.5). Hybrid sterility usually results from problems during gamete formation. One of the best-known hybrids is the mule (Figure 9.2.22 on page 409). As the offspring of a female horse (2n = 64) and a male donkey (2n = 62), the mule has 63 chromosomes in total. Hybrids such as the mule do not have homologous pairs of chromosomes because their genetic material came from different species. Without homologous pairs, meiosis cannot proceed normally and the gametes, if any are formed at all, cannot interact correctly in order for fertilisation to occur. Mules, like all hybrid offspring, cannot reliably reproduce. Mules are still valued and used for some purposes today. Each breeding of a mule requires crossing a male horse and female donkey.

In some situations the first generation of hybrids is semifertile and can occasionally produce offspring when reproducing with another hybrid or with one of the parental species. However, the second generation is typically sterile. This form of postzygotic isolating mechanism is called hybrid breakdown.

SEXUAL SELECTION

**Sexual selection** is a selection pressure that also functions as a prezygotic isolating mechanism.

Most animals exhibit some level of sexual selection in which at least one sex selects their mate based on specific traits. Although it may appear that mates are chosen on the basis of an irrelevant characteristic, the chosen traits are often indicators of good health, strength and fitness or high adaptive value. The alleles of these mates will then be inherited by offspring. Sexual selection is particularly common in birds. For example, barn swallows (Hirundo rustica) select mates based on the length of tail streamers (elongated tips of tail feathers), which indicate health and fitness.

Another example is the bowerbird, which selects a mate based on the showiness, structure and colour of the bower it builds from collected objects (Figure 9.2.7). This again indicates the bird's health and fitness.

Animals may compete with members of their own sex for mates of the opposite sex. Animals such as sea lions, antelope and kangaroos come into direct physical conflict over mates, usually resulting in a single male winning the right to mate with a large number of females. These conflicts ensure that the individuals with the fittest phenotypes are most likely to produce more and healthier offspring after mating. In this way the fitter alleles are more likely to be inherited by the next generation and increase the frequency of those favourable alleles in the gene pool over time.

Sexual selection both produces and explains many striking features in animals and plants.

SPECIATION

Speciation is the evolution of new species from an ancestral species. There are different evolutionary mechanisms that can lead to speciation; however, each of these mechanisms essentially results in an accumulation of genetic changes that leads to the reproductive isolation of populations. When populations become reproductively isolated, they can no longer interbreed and are considered distinct biological species.

Allopatric speciation

The most common form of speciation is allopatric speciation which occurs when a population becomes divided by a geographical barrier (Figure 9.2.9). The spatial isolation prevents individuals of the separated subpopulations from interbreeding.

Over time, different environmental selection pressures and genetic drift drive change in the allele frequencies of the two subpopulations. Eventually, the two subpopulations may diverge genetically and physically, to the point where they can no longer interbreed if they come into contact again. When populations can no longer interbreed, they are considered distinct species (speciation has occurred).

For example, as Australia moved northward approximately 56-23 million years ago, the centre became arid, which partitioned what had previously been a single continuous southern habitat into eastern and western zones. The species of each zone then continued evolving according to separate selection pressures, leading to the evolution of new local species. That is why the south-west corner of Western Australia has a very high number of endemic species, both plant and animal. Tasmania also has many unique species as a result of its separation (Figure 9.2.10). Tasmanian species diversified after Tasmania became separated from the Australian mainland following the rise in sea levels at the end of the most recent ice age.

Adaptive radiation

Adaptive radiation is the rapid divergent evolution of a large number of related species from a single common ancestor. Adaptive radiation results from accelerated speciation after organisms evolve different adaptations in response to new conditions and opportunities. This can occur following changes to the environment (e.g. extinction of competitors) or colonisation of a new environment where vacant ecological niches are available. Adaptive radiation can result in a wide diversity of species, each with unique adaptations to their environment.

The finches of the Galapagos Islands are an example of adaptive radiation. The beaks of these birds are adapted to different food types. Darwin collected specimens of finches from the different Galapagos Islands when he sailed on the voyage of HMS Beagle (1831-36) (Figure 9.2.13). When Darwin returned to England, the ornithologist John Gould examined the specimens and recognised them as related but distinct species. Darwin had collected 13 different species of finch, with every island in the archipelago home to a number of species. Each species has a particular beak, body size and feeding behaviour that is advantageous for the conditions on the island on which they are found. The various finch species had diversified from a single common ancestor; they had undergone adaptive radiation. The finches of the Galapagos Islands are also examined in Chapter 8.

Australia also provides examples of adaptive radiations. The family Macropodidae includes 10 genera and 65 species (with some recent extinctions) including kangaroos, wallabies, wallaroos, quokkas, pademelons and tree-kangaroos (Figure 9. 2.14). Macropods diverged from a common marsupial ancestor approximately 53 million years ago, with modern kangaroos radiating about 25 million years ago. For each genus today there are multiple species. The modern species have adapted to a browsing or grazing lifestyle with teeth and digestive systems specialised for feeding on plant material. This example of adaptive radiation, like most, occurred as multiple divergences over millions of years.

MICROEVOLUTIONARY CHANGES

Microevolution involves changes in alleles, populations or species over short periods of evolutionary time. Mutation, migration, genetic drift and natural selection are the main processes that drive microevolutionary change. An accumulation of these changes over time can lead to speciation.

Evolution of the platypus

Australia's platypus (Ornithorhynchus anatinus) has been delighting and confusing biologists for centuries. Discovered in 1797, the European scientific establishment first thought the animal was a hoax.

Platypuses are mammals that have several pnmltlve reptile-like features. Platypuses, plus the four species of echidna, are all that remains of an ancient group of egg-laying mammals called monotremes. Monotremes diverged from the main mammal lineage around 165-180 million years ago, during the **Jurassic period**. Platypuses and echidnas diverged between 48 and 19 million years ago.

Like its echidna relatives, platypuses lay soft, leathery eggs which they incubate next to their skin. Both groups also produce milk from pores in the skin, like sweat, in contrast to the lactation (milk production) through specialised nipples of other mammals. Uniquely, platypuses have a venomous spur on their hind legs, and have an extremely sophisticated bill capable of detecting the electrical impulse of prey animals.

The platypus fossil record is patchy. The oldest confirmed fossil of a platypus ancestor was found in New South Wales in 1985 and dates to around 110 million years ago, during the **Cretaceous period**. The next oldest fossil was a 62 million-year-old tooth found in Argentina. A further three fossils are all Australian, and date to 25-15 million years ago. The oldest fossil of the modern species is about 100 000 years old.

Such evidence suggests platypuses evolved in Australia when it was still connected to South America and Antarctica. A population of platypus relatives must have survived in South America for a time, but died out. Since then, platypuses have been confined to Australia. Recent genetic studies, comparing the modern platypus to other vertebrate groups, support the fossil evidence.

The new information confirms that many platypus features are truly primitive. For example, certain genes from egg-yolk proteins are shared only with reptiles and fish. Thus, egg-laying was retained from reptilian ancestors. Platypuses have a reptile-like cloaca, a single orifice serving both reproductive and excretory functions. Lactation also seems a very old feature, dating from the origin of the monotreme group during the Jurassic. Giving birth to eggless young ( called live-bearing) was a much more recent feature developed by placental mammals.

The genetic analysis also shows that the platypus has put primitive reptilian genes to innovative uses. For instance, platypus venom appears to result from modification of genes that once served other purposes. While platypus and snake venom are similar, platypuses seem to have evolved the same feature but in a completely independent way via convergent evolution.

Some changes are recent accumulations and thus are described as advanced features. Today's platypuses are smaller than their ancestors, and this may be an ongoing speciation trend. Platypuses are substantially larger in Tasmania and southern Australia, becoming smaller towards the northern part of their range. Their populations may be diverging into northern and southern varieties.

The modern species also seems to have become more specialised for an aquatic lifestyle, plus its distribution has become restricted to Australian rivers (Figure 9.2.16). As part of this trend, the original teeth of the platypus have been replaced with horny pads, and there has been some anatomical simplification. Some recently evolved features also allow platypuses to smell while underwater, supplementing their electro-detection sense.

Evolution of the horse

Unlike the patchy fossil record for the platypus, the case of horse evolution is well documented. In fact it is one of the most complete transitional series in palaeontology.

A transitional series is a group of fossils showing microevolutionary change from one form to another. Each individual fossil is subtly different from the previous, like still images from a movie. Dramatic long-term transitions can result from a series of microevolutionary changes.

The ancestral horse genus Eohippus (containing only one species, Eohippus angustidens arose around 52million years ago (Figure 9.2.17). It was a wombat­sized rainforest animal, living much as tapirs (horse relatives) do now. Fossil teeth show that Eohippus was an unselective herbivore, eating mainly soft leaves and fruit. Its legs were much shorter relative to its body than modern horses, while its wrist and leg-joints were flexible. The animal had five splayed-out toes on each foot; a primitive feature retained from the common ancestor of mammals and still possessed by humans today who have five digits on each hand and foot. Four of the five front toes touched the ground, while at the rear Eohippus walked on the three middle toes. All toes had soft, pad-like hooves. Eohippus' teeth were generalised and fairly flat. Eohippus remained relatively unchanged for about 20 million years.

Towards the end of the **Oligocene epoch**, Earth started cooling and drying. Rainforest habitat slowly gave way to open forest, woodland and then grassland. The tougher vegetation favoured animals with sturdier grinding teeth (molars). At the same time, the increasingly open spaces would have selected for larger and faster animals able to evade predators.

The changing environment selected for various new traits through a series of slightly different species. The general trends included greater size, proportionally larger brains, improved running ability via loss of outer toes and weight being borne on the middle toe, stronger and longer leg and toe bones, more developed hooves, plus tougher teeth. Not all changes were gradual; for instance, high-crowned teeth suited to grazing appeared relatively suddenly. The extreme ends of the transition are shown in Figure 9.2.18.

However, this conventional image of evolutionary change as a linear series iepresents an incorrect understanding. Such transitions are extremely rare, and old-fashioned sequences such as this simplify the true complexity. Instead, adaptive radiation is the norm (Figure 9.2.20). Each new species would have been subject to new environmental and selection pressures, and rapid change according to punctuated equilibrium.

Today, seven species remain from a family that was until recently even more diverse: three species of zebra, three asses (including donkeys), and the 'true horse' in both wild and domesticated forms (Figure 9.2.21). The latter group diverged from its nearest relatives as little as 43 000 years ago.

The fossil record of the horse family shows typical prezygotic isolation, speciation and adaptive radiation.

Instant speciation

Modern horses are also examples of postzygotic isolation. At least some of the incompatibility between species results from chromosomal differences (Table 9 .2.2). In the recent past these may have appeared via instant speciation. Sometimes chromosomes fail to separate properly during meiosis (this is known as a nondisjunction), resulting in offspring witl1 an extra set of chromosomes (polyploid). Such individuals are chromosomally incompatible with other members of the herd. In most cases such individuals would be destined to never reproduce. However, if an affected individual found and mated with another member of the herd having the same number of chromosomes, the offspring may be viable. They would constitute a new species, phenotypically similar to the original but no longer reproductively compatible.

9.3 Macroevolution and biodiversity over time

Today, Earth is very different from when it formed over 4600 million (4.6 billion) years ago. Starting as a totally molten and lifeless world, it has passed through many different phases driven by a mix of biological and geophysical forces. Significant events included a long period of intense volcanism that helped create complex molecules and life, a toxic gas added to the original atmosphere, numerous ice ages including a completely frozen world, ocean acidification, continental pile-ups, a planet-wide desert and vast tropical forests. Since the origin of biomolecules, the first cellular life in the oceans and the formation of a solid surface on Earth (Figure 9.3.1), living organisms have been constantly responding to environmental change. This change drives the evolution of life on Earth; constant conditions would mean little or no evolution. Environmental change can lead to the extinction of some species but bring new opportunities for others.

Macroevolution means change above the species level. It is separate from microevolution (changing allele frequencies within a species). The processes are not fundamentally different: macroevolution is essentially the cumulative results of microevolution over very long periods. As such, life has become more complex since it first appeared on Earth.

In this section you will learn about the significant changes in life forms over Earth's geological history, including the evolution and diversification of multicellular organisms, land animals, the first flowering plants, and mammals.

GEOLOGICAL TIME SCALE OF EARTH

The history of Earth and evolving life can be traced using the geological time scale, which covers events that occurred on Earth from its formation to now.

Cliff faces often show layered sequences of different-looking rocks, each with specific collections of fossilised remains of ancient organisms within the rock strata (singular stratum) (Figure 9.3.2). The layering of the various strata is consistent across sites around the world. These features help us reconstruct Earth's geological history.

The first such histories relied on relative dating. Sedimentary rocks are deposited in a top-down direction, like layers of snow on a ski slope. Therefore, those at the top must be youngest while the oldest are at the bottom. For much of the history of geology, the layers could only be compared as older or younger than those adjacent, and the actual ages were unknown. Since the advent of radiometric dating, the exact ages of the rocks can be measured.

The geological time scale is divided into many subdivisions, the largest being eon. Eons are subdivided into smaller and smaller divisions: eras, periods and epochs (Table 9.3.1).

PRECAMBRIAN TIME

Although the Cambrian did not begin until the most recent fifth of Earth's history, when Earth was already over 4000 million years old, the period has special significance for geologists and biologists. For many years, it seemed that rocks from this period contained the oldest fossils of complex creatures. According to pre-20th century science, fossils were seemingly absent from earlier periods and some scientists had wondered whether life spontaneously appeared during the Cambrian. We now know that macroscopic life appeared much earlier than the Cambrian, and that instead of being the origin of life, the Cambrian merely marked the origin of hard body parts that easily fossilise.

**Precambrian** time is not a true geological eon, era, period or epoch. The **Precambrian** is divided into three parts: the Hadean, the Archaean eon and the Proterozoic eon. Life on Earth first appeared during the **Archaean eon**.

The Hadean (~4600-4000 mya)

Earth had formed by 4600 million years ago. Planets form by attracting planetesimals (space rocks) via gravity; the high-speed collisions impart enough energy to melt the entire mass. Earth was molten at first, and remained so for a further 600 million years. This was the **Hadean** time, named after the mythological Hades underworld, of 4600-4000 million years ago.

The molten rock released into the atmosphere much of the water that had been part of its source material. As the planet gradually cooled, molten material solidified, creating a rocky terrain (Figure 9. 3. 3). During this cooling period, the atmospheric water condensed, falling as a colossal rainstorm lasting millions of years. The water helped cool the surface, eventually enough for the water to pool permanently as oceans.

The **Hadean** is technically not a geological period as no terrestrial rocks survive from this time. Estimation of the time of Earth's formation is based on the age of lunar rocks from the Apollo 16 mission in 1972, dated to approximately 4500 million years. The Moon is of similar age to Earth, but slightly younger. The Moon formed from a collision between Earth and a Mars-size proto-planet, very early in Earth's history. Meteorite samples also provide a consistent age for the rocky material of the inner solar system, collectively pointing to Earth's age being about 4600 million years.

Earth's oldest rocks are roughly 3800 million years old. Although solid rock formed earlier, erosion and plate tectonics have since destroyed all of Earth's oldest rocks. Yet grains from such rocks survive, and have since been incorporated into younger rocks. Called zircons, these grains have been dated to 4400 million years (Figure 9. 3 .4).

The advent of a rock record marks both the start of Earth's geological history and the beginning of the **Archaean eon**.

Archaean eon (4000-2500 mya)

The **Archaean eon** spanned about 1500 million years. At the time the atmosphere was mainly carbon dioxide, methane and ammonia. At the start of this eon, the atmosphere had neither free oxygen nor nitrogen, because both gases are by-products of life. What little land existed consisted of many small island proto­continents called cratons.

Life appeared early in the **Archaean**. The earliest free-living cellular life forms not bound to geophysical structures were bacteria, soon followed by a related but different kind of prokaryote called archaea.

The oldest fossils are microfossils of bacteria that are roughly 3800 million years old. Stromatolites came after these earliest life forms but since they fossilise easily, they became common in the fossil record around 3500 million years ago. Stromatolites are mats of sticky photosynthesising bacteria, which grow on the outer surface and collect sandy material. Because stromatolites collect layers of sediment, they form characteristic layered fossils. Stromatolite fossils in cross­section contain many thin mineral layers (Figure 9.3.5a). They have been found in early Archaean rocks of South Africa and Western Australia (Figure 9.3.5b) and increased in abundance throughout the **Archaean**, but began to decline during the **Proterozoic eon**. Stromatolites still exist today, most famously in Shark Bay, Western Australia (Figure 9.3.5c).

The two main kinds of early prokaryotes (archaea and bacteria) were Earth's sole inhabitants for more than 1500 million years.

THEORIES ON HOW LIFE CAME ABOUT

At some point, organic molecules accumulated to form the first self-replicating life form. We know that life depends on elements including carbon, hydrogen, nitrogen, oxygen, phosphorus and sulfur, and that water is vital. Several theories about how life first began are currently being investigated.

Often in science, new theories disprove and replace old theories, but in this case two older theories-the primordial soup theory and the RNA world theory-are both partially correct and are now part of a more complete theory: the hydrothermal vent theory. The hydrothermal vent theory is our best current understanding of the origins of life on Earth.

The primordial soup theory

**The primordial soup theory** suggested that amino acids (the building blocks of proteins) were the result of gases from early Earth's atmosphere and molecules in the vast oceans being energised and changed by lightning strikes and ultraviolet light. This theory was proposed independently by Russian scientist Aleksandr Oparin and English geneticist John Haldane, and is often referred to as the Oparin­Haldane theory.

Many scientists have attempted experiments to replicate the conditions of early Earth and produce amino acids, most famously Stanley Miller and Harold Urey in the USA in 1953. The Miller-Urey experiment produced organic compounds, including amino acids, using a closed system of laboratory equipment to model the primordial environment (Figure 9. 3. 6).

As our understanding of the ancient atmosphere and weather has developed, it has become clear that the significance of the Miller-Urey experiment lies m demonstrating that organic molecules can be made from inorganic molecules.

**The RNA world theory**

All living cells on Earth contain the following three biomolecules, each serving a different critical function:

•proteins-made of amino acids; they have structural and catalytic functions

•DNA-double-stranded nucleic acid that carries information from one generation to the next

•RNA-single-stranded nucleic acid that carries information and has metabolic functions within a cell.

In the early 1980s Sidney Altman and Thomas Cech found that some RNAs can act as catalysts for chemical reactions. These catalytic RNAs are known as ribozymes, and earned them the 1989 Nobel Prize in Chemistry.

The discovery of ribozymes supported a theory that RN As were the first molecules able to store information, replicate and catalyse reactions. Due to the instability of RNA, DNA may have eventually evolved to become the dominant genetic material.

**Hydrothermal vents theory**

Convection currents within Earth push the crustal plates around the planet's surface. The plates move past or under each other in various ways.

Some pairs of plates also move apart in opposite directions, driven by upwelling lava. Today, such sites are nearly always in the deep ocean, so the plates are saturated with water. Around the sites of lava upwelling, the highly mineralised water is very hot. The water rises, and following contact with cold seawater, the minerals precipitate out to form towers up to 60 m tall. This process resembles smoke rising, so two of the three kinds of towers are called smokers (black smokers or white smokers) (Figure 9.3.7). Today, the smoker towers are very rich in life, although life did not originate there.

A third kind of tower, discovered in 2000, occurs up to 10 km from lava fields. The rising water is cooler-150-200°C compared to 450°C for the smoker towers. The water deposits carbonate mineral structures, and from their appearance they are known as Lost City Hydrothermal Vent Fields (Figure 9.3.8). These have a spongy, cell-like texture. The mineral walls are porous to water, but not to large molecules.

The combination of heat and pressure builds many complex organic molecules, inside the mineral cells, from water and carbon dioxide. Also, the Lost City structures are highly alkaline, and seawater is less alkaline, setting up a steep ion gradient that is effectively a powerful electric current. With this power source, and given that water continually circulates through the Lost City towers, the biomolecules become increasingly complex. This process combines both the Miller-Urey experiment principles and those of the RNA world theory. The current evidence from the Lost City towers suggests that geophysical forces alone produced metabolic processes.

Metabolic processes preceded life. Life merely incorporated the existing chemical pathways. The metabolism at first occurred only within the Lost City structures. At some point, the metabolism became complex enough to be fully self-replicating while also consuming material from the environment. So the simplest kind of life was born, although completely tied to the Lost City structures.

In the steps leading to the evolution of the biological cell, replicating organic molecules became enclosed in vesicles, surrounded by a biological membrane. The membrane provided an internal environment that was different from the external environment and in which metabolic processes could develop.

After developing membranes, eventually free-living cells moved away from the Lost City towers. Yet they kept the chemistry of the original structures, including chemically reproducing the necessary electric current.

The **hydrothermal vent theory** is also the only one compatible with the fact that Earth completely froze over during the **Cryogenian period**. Life in the deep ocean volcanic vents had no need of sunlight and was unaffected by the frigid surface conditions.

Evolution of cellular organisms

The two earliest forms of cellular life were bacteria and archaea, both prokaryotes. The two forms look very similar, and neither has any visible organelles; yet chemically they are quite different, with archaea closely resembling eukaryotic metabolism and membrane biochemistry. As stated by the **endosymbiotic theory**, it is now clear that bacteria entered archaean cells, but were not consumed, and continued to live. The combination of both types of prokaryotes was effectively the birth of eukaryotic cells that were more metabolically efficient. The endosymbiotic theory is discussed in more detail in Chapter 2.

The mitochondria and chloroplast organelles of eukaryotic cells are effectively simplified bacteria in a symbiotic relationship with the cell. Both types of organelle maintain their own DNA, independent of the cell's nuclear DNA. Cellular nuclear membranes probably developed from infolding of the bacterial cell membrane, enclosing the bacterial DNA.

Eventually, eukaryotic cells started joining together in colonies of a single type of cell, working as one. That was the origin of multicellular organisms. Such organisms further developed specialised cells for various functions.

PROTEROZOIC EON (2500-541 MYA)

During the **Proterozoic eon**, large continental landmasses were formed by the convergence of smaller cratons. The stromatolite-forming bacteria, plus other species similar to modern cyanobacteria (formerly called blue-green algae) had been photosynthesising since the **Archaean**. Photosynthesis uses light energy to make sugars from carbon dioxide and water, with oxygen being a by-product. At first oxygen gas made no difference to the atmosphere. Iron in the sea reacted with oxygen, removing oxygen from the air and forming iron-oxide sedimentary layers in the deep ocean. After many millions of years, the iron in the sea was used up and oxygen then started building up in the atmosphere.

By about 2000 million years ago, the atmosphere was rich in oxygen. This caused the extinction of many early anaerobic life forms because oxygen was toxic to them. However, oxygen in the atmosphere allowed for the evolution of aerobic and complex multicellular life forms. The earliest eukaryotes are known from fossils 1400 million years old. Multicellular algae (red algae and green algae) and the first animals evolved towards the middle of the **Proterozoic eon**.

One reason it took complex life so long to appear was that the supporting structural molecules that eukaryotes and multicellular organisms needed to grow larger can only exist in an oxygen environment. Evolution was limited until the atmosphere became oxygenated.

The Cryogenian period (720-635 mya): Snowball Earth

Geological evidence from the **Cryogenian period** of the middle-Proterozoic shows severe glaciation across the whole planet, even on continents near the equator at the time. This is currently interpreted as two separate phases of total ice coverage during the Cryogenian, both of which also froze the oceans to a considerable depth (Figure 9.3.9).

However, life at the bottom of the oceans, powered by volcanic energy, was hardly affected. This period saw the emergence of Amoeba, red and green algae, and sea sponges.

The Ediacaran period (635-541 mya): multicellular life diversifies

The **Ediacaran period**, at the end of the **Proterozoic eon**, reveals the earliest evidence of diverse multicellular animals. These animal fossils are collectively called the Ediacaran fauna after the Ediacara Hills in the Flinders Ranges of South Australia where they were first found in 1946 (Figure 9.3.10). The fauna has since been recorded from all continents. The fossils are of small, soft-bodied sea creatures that resemble modern jellyfish, segmented worms, and animals with plant-like branching forms (Figure 9.3.11). They include representatives of all the major groups of invertebrate animals, plus some unique to the period. The fauna was entirely aquatic, and mostly benthic (bottom living) but also fixed in place rather than free swimming. The atmosphere of the time had low oxygen but very high carbon dioxide compared to today.

THE PALAEOZOIC ERA

The **Palaeozoic** (meaning 'ancient life') was a time of great change for Earth. By the early **Palaeozoic**, Earth's landmasses had combined to form a single supercontinent: Pangaea. These movements of land masses greatly affected the climate as well as land and sea environments, and hence the evolution of organisms.

Cambrian period (541-485.4 mya): the Cambrian explosion

Fossil evidence from 542million years ago shows a dramatic increase in the number and complexity of life forms in the oceans. This is known as the Cambrian explosion. Fossils include worms, sea jellies, brachiopods and arthropods, the most common being trilobites (Figure 9.3.12). The number and diversity of Cambrian fossils is significantly greater than those from the **Ediacaran period** due to the emergence of organisms with hard exoskeletons, which are more readily preserved.

Ordovician period (485.4-443.8 mya): the first vertebrates

The **Ordovician** was a time of very warm water (45°C), high sea levels and extensive shallow seas rich in algae. Simple, non-vascular land plants may have started colonising the shoreline.

The fauna diversified. Major groups included straight-shelled nautili’s, and various types of arthropods including trilobites (Figure 9.3.14). Reef-forming corals appeared during this time. Other newcomers included the first vertebrates: jawless armoured fishes (ostracoderms) (Figure 9.3.15).

Volcanic activity at the end of the period deposited silicate rocks that absorbed atmospheric carbon dioxide. The lowered temperatures introduced a brief but extreme ice age, which dropped sea levels, thereby dramatically impacting coastal habitats. The end of the Ordovician was Earth's second-largest mass extinction.

Silurian period (443.8-419.2 mya): the first life on land

After the Ordovician ice age, the **Silurian** rebounded to a long and relatively stable warm period. The relative oxygen levels were low compared to today, but the carbon dioxide was high.

The earliest evidence of life on land is from terrestrial rocks of the **Silurian period**. The first known air-breathing animals were arthropods. Millipedes, centipedes and the earliest arachnids also first appeared during the Silurian. The oldest known land plants date from the late Silurian. These were small, spore-bearing, vascular plants such as Cooksonia (Figure 9.3.16). Cooksonia had an aerial stem but lacked roots and leaves. It had xylem and phloem tissue to transport water and nutrients. It may be that non-vascular plants such as liverworts and mosses evolved on land earlier, but there is no definitive evidence of them until much later. Terrestrial fungi are also recorded as hyphae and spores from the Silurian.

Devonian period (419.2-358.9 mya): the first land vertebrates

The emergence of land-based plants and animals resulted in organic matter being deposited into the barren soils, promoting further colonisation of the land.

The vegetation of the **Devonian period** would have been only a few centimetres tall and spread by spores. The **Devonian period** is often referred to as the 'age of fishes' due to the diversification of this group. Jawed fishes evolved in the sea, along with armoured placoderms, ray-finned and lobe-finned fishes and early sharks (Figure 9.3.17).

One group of fleshy-finned Devonian fishes developed such sturdy fins that they were able to support their weight at the edge of the water. These animals would give rise to the first terrestrial vertebrates, the tetrapods (meaning four-footed) (Figure 9.3.18). The earliest tetrapods included amphibians.

Carboniferous period (358.9-298.9 mya): abundant forests

The **Carboniferous period** (the coal age) is characterised by abundant terrestrial plant life and huge arthropods. Spore-bearing plants developed tree-like forms, having woody stems, roots and leaves. Most of the land was covered in vast forests. New forest habitats influenced the diversification of animal species (Figure 9.3.19).

Since plants produce oxygen as a by-product of photosynthesis, the extent of forest coverage meant levels of atmospheric oxygen that have never been equalled (35% compared to today's 21 %).

Arthropods' diffusion-based circulatory system can only oxygenate tissues over a certain distance, which is dependent on atmospheric oxygen concentration. At today's levels, this distance is only a few centimetres. The very high Carboniferous levels allowed a much greater distance, meaning that arthropods grew to enormous sizes up to 3 m.

A second effect of the forests was the build-up of dead woody material on the ground. Fungi of the Carboniferous had not yet evolved the ability to decompose lignin, the main structural component of wood. Thus, undecomposed wood accumulated in very deep layers, with the forest constantly growing over the top. Over geological time the buried carbon-rich material was compressed to form coal, now mined as a fossil fuel.

During the Carboniferous, the tetrapods were losing their amphibian-like bodies in favour of the long snouts and more agile limbs of early reptiles. The tetrapods were spending more time out of the water adapting to the terrestrial environment. An amniotic egg evolved and supported reproduction on land; scaly skin evolved and protected animals from dehydration.

Abundant plant growth also removes carbon dioxide from the atmosphere, so towards the end of the Carboniferous carbon dioxide levels fell and the period ended with another major ice age.

Permian period (298.9-252.2 mya): the greatest mass extinction

The **Permian** began in the grips of the severe ice age that started in the late Carboniferous. Also early in the **Permian**, the continents joined to form the supercontinent known as Pangaea (Figure 9.3.20).

Such joining is widely misunderstood to have been a unique event, but it was just the latest in a cycle of at least six earlier continental conjunctions and breakups. However, the formation and breakup of Pangaea affected the evolution of complex life far more than any other continental conjunction.

The formation of Pangaea had several major effects, the first being a tremendous reduction of coastal and continent-margin habitats. Second, coastal winds could only transport moisture a short distance inland from the coast. So the vast bulk of the inland region was a desert, far dryer than any on Earth today.

The rest of Earth's surface was a single huge ocean, which would have had a circular current around the supercontinent. Such conditions may have caused stagnation and de-oxygenation of the deep ocean, releasing large quantities of toxic hydrogen sulfide gas into the atmosphere.

Due to these and other factors, the Permian saw the greatest of all mass extinctions. Around 70% of land species and 90% of ocean species were wiped out. The Permian was the only mass extinction of arthropods, including the trilobites.

However, the dry conditions favoured the rise of reptiles. The Permian was also the time of mammal-like reptiles, including sailback species such as Diinetrodon (Figure 9.3.21).

THE MESOZOIC ERA: THE AGE OF THE DINOSAURS

During the **Mesozoic era**, Earth gradually rebounded from the toll of the Permian. Pangaea started breaking apart during the **Triassic period** in the early **Mesozoic**. By the **Jurassic period** of the mid-Mesozoic, the breakup had led to two large continents, Laurasia and Gondwana. During the final period of the **Mesozoic**, the **Cretaceous**, each of these further broke up into somewhat familiar continents. During the **Mesozoic**, the world also started greening again, and the climate became less arid. Such changes provided new opportunities for animals and plants. Life diversified rapidly and some organisms grew enormous.

Triassic period (252.2-201.3 mya): the first mammals

The early **Triassic** was still quite dry. As Pangaea started breaking up during the mid-Triassic, warmer and more humid conditions returned. The change favoured seed plants, such as cycads and gingko trees, and also ferns. These groups are generally tough and difficult to eat so herbivores grew not only in number, but in size as well (Figure 9.3.22).

The period saw the proliferation of reptiles and their relatives. Mammal-like therapsids gave rise to the true mammals in the late Triassic. The early mammals were small, insectivorous, nocturnal, hairy and warm-blooded (endothermic) (Figure 9.3.23). The Triassic also saw the emergence of ichthyosaurs and other marine reptiles, plus the archosaurs: a huge and very important family that included pterosaurs, crocodilians and dinosaurs. Dinosaurs remained small during the Triassic.

Before, during and for a while after Pangaea's breakup, the land remained heavily de-vegetated. This affected the atmospheric oxygen content, which was 16% compared to today's 21 %. In response the early dinosaurs evolved an efficient respiration system involving air-sacs and one-way lungs (Figure 9.3.24). This allowed them to maintain an endothermic, high-energy metabolism in a relatively low-oxygen environment such as birds have today.

The end of the Triassic saw another mass extinction, possibly related to the volcanism that broke up Pangaea. The extinction event affected the oceans more than the land, yet also cleared out major families of archosaurs, certain true reptiles, large amphibians and the last of the mammal-like reptiles. Dinosaurs were poised to take over.

Jurassic period (201.3-145 mya): the age of the dinosaurs

The early **Jurassic** saw the completion of the Pangaea breakup that had started during the Triassic. Pangaea broke up into two landmasses: Gondwana in the south and Laurasia in the north, separating northern and southern flora and fauna. That meant a greater amount of coastline, milder and more humid conditions and a re-vegetation of the land. Widespread return of forests raised atmospheric oxygen levels while carbon dioxide levels remained high, making the Jurassic mostly tropical or subtropical.

The period saw the arrival of the non-egg-laying mammals, and the first modern lizards.

The Jurassic is most famous for being the heyday of the dinosaurs. Although initially bipedal sauropods appeared late in the **Triassic**, the family reached its most impressive sizes during the Jurassic (Figure 9.3.25). The sauropods remain the most successful vertebrate group of all time.

Birds also first appeared during the **Jurassic**. While it is often implied that birds were separate from dinosaurs, they were then (and still are) a subgroup of reptiles. The oldest bird fossil from this period, Archaeopteryx (-150mya), was simply a feathered dinosaur and otherwise indistinguishable from other therapods of the time (Figure 9.3.26). Feathers probably evolved first for insulation, and were later used for flight. Birds today retain all characteristic dinosaur features. Today we refer to dinosaurs as non-avian dinosaurs.

Cretaceous period (145-66 mya): the first flowering plants

Atmospheric carbon dioxide began lower in the **Cretaceous** han in the Jurassic. That meant a cooler climate, with frequent snow in high latitudes. However, carbon dioxide levels rose mid-period due to prolonged volcanic eruption, and soon the climate warmed up again. Laurasia had fully broken up during the Jurassic, and during the **Cretaceous period** Gondwana was still breaking up. High sea levels compared to today meant that about one-third of present-day lands were submerged, so shallow inland seas were common.

Dinosaurs reached their peak diversity. The first true placental mammals and marsupials appeared, as did mosasaurs: a terrifying family of predatory marine reptiles. The first grasses also arose.

At the end of the **Cretaceous period**, about 65 million years ago, another mass extinction occurred. This time an asteroid crashed into Earth, causing tremendous ecological disruption. Most forms of dinosaurs-except birds-became extinct, as did 76% of all land and marine species.

The dawn of the flowering plants

The early Cretaceous environments were still dominated by ferns, seed ferns, cycads and conifers; but angiosperms (flowering plants) also developed about 135 million years ago. Angiosperms diversified rapidly, and by the end of the Cretaceous were by far the most diverse group of land-based plants. The first angiosperms co-evolved with thriving insect populations, which pollinated the flowers (Figure 9.3.27).

Angiosperms may have arisen in direct response to dinosaurs. The largest dinosaurs were herbivores that also travelled in large herds that caused great forest disturbance, such as the clearance of vegetation. Fast-reproducing angiosperms had a clear advantage in colonising the bare soil compared to other plant groups of the time. Angiosperms have since gone on to overwhelmingly dominate Earth's flora, now comprising around 90% of plant species.

THE CENOZOIC ERA: THE RISE OF BIRDS AND MAMMALS

The **Cenozoic era** marked the shift towards the life forms we know today. Extinction of the Cretaceous giants (dinosaurs, pterosaurs and marine reptiles) enabled smaller species to quickly diversify and become larger. The era experienced an overall cooling and drying trend, culminating in a succession of severe ice ages caused in part by cyclic wobbles in the angle of Earth's axis of rotation.

Palaeogene period (66-23.03 mya): mammals diversify

The **Palaeogene** climate began with cooling and drying, followed by some exceptional warming periods. Both the carbon dioxide and oxygen concentrations were higher than today, but down from Mesozoic levels.

Mammals and birds took advantage of the niches left by the extinction of the dinosaurs. Birds became abundant and diverse, as new species of plant life evolved that served as shelter and food. Mammals thrived, diversifying to include most of the modern families. Mammals also took to the oceans for the first time. Some mammals became giants almost as large as dinosaurs. Early primates (prosimians) were common, but were eventually replaced by true monkeys and apes.

The continents continued moving towards their present positions. In particular India began its collision with Asia, which is still ongoing. The resulting changed ocean and atmospheric currents affected climates. In what is now Australia, rainforest vegetation was more widespread than it is today.

Neogene period (23.03-2.58 mya): the first hominins

The **Neogene** continued the cooling trend, which suited grasses over forests. Savannahs and steppes arose for the first time, favouring herds of grazing mammals.

Further major continental movement saw Australia fully separated from Antarctica, while South America and North America came together (Figure 9. 3. 30). Atmospheric gas concentrations were around their present levels, as were sea levels, and a series of ice ages started.

Flowering plants, birds and mammals had become recognisably modern. Animals across the globe included giant forms: the megafauna. In the Northern Hemisphere these included mammoths and other giant members of the elephant family, sabre­toothed tigers and ground sloths (Figure 9.3.31). Southern Hemisphere megafauna included huge lizards and marsupials.

The first hominin (human-like) fossils are 6-7 million years old, and the first species of the genus Homo, Homo habilis, dates to around 2.5 million years ago (Figure 9.3.32).

Quaternary period (2.58 mya-today): the expansion of modern humans

The **Quaternary period** is the time we are living in now and is the latest period of the **Cenozoic era**. It includes two epochs: the **Pleistocene epoch** (2.58-0.01 mya) and the **Holocene epoch** (0.01 mya-present). During the **Quaternary period** climates fluctuated from cold, dry glacial periods with low sea levels to warmer interglacial periods and higher sea levels. The **Holocene epoch** (up to today) is an interglacial period of unusually warm conditions relative to the era overall.

The climate started to become drier, which caused the rainforests to shrink in size. Plants and animals better suited to dry conditions started to spread. In Australia giant marsupials roamed, such as Diprotodon, Procoptodon and Thylacoleo. There were enormous goannas and large flightless birds.

Modern humans moved out of Africa, and the Neanderthals, Homo neanderthalensis, became extinct.

During glacial periods, when water was frozen in ice sheets, sea levels dropped and exposed land bridges for species to cross. Modern humans migrated via land bridges as well as using rafts or boats.

The **Holocene epoch** is by far the shortest geological time, only about 11 000 years. It did not include significant changes in species (until the period's end), but did include significant movement of species across continents. Among these species walked the first modern humans.

10.1 Evidence for evolution by natural selection

Evolution is a process of change. The modern theory of evolution states that all living organisms share a common origin that dates back to around 3800 million years ago. In Chapter 9 you learnt that the environment and its inhabitants have changed dramatically since life began on Earth. The earliest organisms were bacteria and, over a long period of time, very different groups of organisms diverged from these early forms of life. Some groups became extinct, while others changed over time to become the types of organisms that we see today.

In this section, you will learn about the evidence for biological change over time, including evidence from the fossil record, biogeography, comparative anatomy, comparative embryology and biochemical evidence such as DNA sequences.

FOSSILS AND PALAEONTOLOGY

Palaeontology involves the study of ancient life represented by fossils. Fossils are the preserved remains, impressions or traces of organisms found in ice (Figure 10.1.1), rocks, amber (fossilised tree sap) (Figure 10.1.2), coal deposits or soil. Preserved remains are usually hard structures that are not easily destroyed or are slow to decompose, such as bone, shell, wood, leaves, pollen and spores. The fossil record refers to the total number of fossils that have been discovered, providing evidence of the evolution of living organisms through geological time. Fossils tell palaeontologists about the kinds of organisms that lived in the past, what they looked like, and where and when they lived. This allows scientists to put a time scale on evolution.

Fossilisation process

Fossilisation is the preservation of the hardened remains or traces of organisms in rock formations.

The chances of an organism becoming fossilised after death are small. Soft­bodied organisms are unlikely to be preserved, because soft body parts decay readily or are subject to predation and scavenging. Fossilised parts of plants are commonly wood and leaves made up of cellulose and lignin, which does not decay readily, and spores and pollen, which are even more resistant to decay.

Fossilisation has a chance of occurring when an organism is buried by sediments. This reduces the chance of decay, due to lack of oxygen for decomposer microorganisms, and hides the organism from scavengers. When sediments of sand, silt or mud in a sea, lake or slow-flowing stream accumulate over the organism, the organism is preserved. The weight of many layers of sediments squeezes out the water between the particles of sand, silt or mud. As the deposit deepens, the temperature increases and soft sediments become solid rock, known as sedimentary rock. Examples of sedimentary rock are sandstone, siltstone, mudstone and shale (a mixture of clay and silt).

Sediments accumulate in bodies of water such as seas, estuaries and lakes; hence, a large proportion of fossils are found where ancient bodies of water existed. Fossil shells formed in this way in Tasmania. In the **Carboniferous and early Permian periods** (about 280 million years ago) a marine gulf formed the Tasmanian Basin. The basin filled with mud and silt washed down from glaciated uplands. The sediments formed layers of mudstone, siltstone, sandstone and some limestone. The basin later became a larger plain, with lakes and freshwater streams that deposited other sediment layers. Fossil shells, fishes (including lungfish) and amphibians have been found in siltstone and sandstone at Mount La Perouse in Tasmania (Figure 10 .1.4).

Organisms on land are less likely to be preserved than those that live in aquatic environments. For example, plants that grow along river banks or on the edge of swamps, where sediments can trap leaves, fruits and seeds, are more likely to be fossilised than plants that grow only on rocky outcrops. Delicate plant parts such as flowers are rarely fossilised, although some are preserved by being buried rapidly (e.g. by ash from an erupting volcano). For these reasons the fossil record is biased towards certain sorts and parts of organisms and certain environmental conditions, which then limits the available evidence of past life and our understanding of it.

Types of fossils

The four main types of fossils are impression fossils, mineralised fossils, trace fossils and mummified organisms.

Impression fossils

Impression fossils are left when the entire organism decays but the shape or impression of the external or internal surface remains (Figure 10 .1. 5). In some rocks such as limestone the fossils keep their three-dimensional shape, but in other rocks (e.g. shales) or in coal deposits that are physically compressed, fossils are flattened. Impression fossils include the internal surface of a shell, tree trunks and plant leaves. If the vacant space of the mould is later filled with foreign material, a three-dimensional 'sculpture' of the organism is formed; this is called a cast fossil.

Mineralised fossils

Mineralised fossils occur when minerals replace the spaces in structures of organisms such as bones. Minerals may eventually replace the entire organism, leaving a replica of the original fossil (e.g. petrified wood). This process is known as mineralisation or petrification. Minerals can include opal, pyrite and silica (Figure 10 .1. 6).

Trace fossils

Trace fossils (also called ichnofossils) are the preserved evidence of an animal's activity or behaviour, without containing parts of the organism. Impressions such as footprints are trace fossils, as are casts of burrows or even coprolites (fossilised faeces) (Figure 10 .1. 7).

Trace fossils of footprints are formed when an organism steps into soft mud. The impression is then covered with loose sand so that the footprint is filled. The sand in the footprint is eventually compacted into sandstone. Finally, when the rock is split open along the bedding surface, the original footprint is revealed (Figure 10.1.8).

Mummified organisms

Mummified organisms are those that have been trapped in a substance under conditions that reduce decay and so undergo little change. Examples include insects trapped in amber, leaves that still contain carbon dioxide (Figure 10.1. 9) and animals frozen in ice or trapped in a peat bog (known as a 'bog body'). Mummified animals, including humans, can have hair and skin preserved in a dehydrated state, while limbs and occasionally entire bodies are preserved in peat bogs and tar pits (Figure 10.1.10).

Dating fossils

The age of a fossil is almost as important as its physical details because it gives a time scale of evolution. The age of a fossil can be determined by relative dating or by absolute dating methods.

Relative dating

Relative dating is based on stratigraphy. Stratigraphy is the study of the relative positions of the rock strata (singular stratum), or layers, some of which contain fossils. The lowest stratum is the oldest and the upper strata are progressively younger. The age of a fossil is estimated relative to the known age of the layers of rock above and below the layer in which the fossil is found (Figure 10.1.12). For example, if a layer containing fossils lies below rock that is dated at 200 million years old, then the fossils must be at least that age or older. Relative dating can be difficult in areas where rock layers have been eroded, or where rocks have been buckled, moved or reburied, altering the original sequence of strata.

An index fossil (sometimes known as an indicator fossil) is a fossil used to define and identify geologic periods. Sometimes the only way to age a fossil bed is by using index fossils and stratigraphy. Index fossils are commonly found fossils from similar sites for which an absolute age has been determined. For example, in Europe the same type of ammonite (extinct mollusc) is found in different regions. A species of ammonite fossil is called an index fossil because it indicates that the rocks at each locality are of similar age (Figure 10 .1.13).

Absolute dating

Absolute dating provides a more precise estimate of age, although it does not mean that it provides an exact date. Radiometric dating, thermoluminescence and electron spin resonance are all methods of absolute dating that are used to determine the age of fossils.

Radiometric dating is a quantitative technique used to determine the proportion of particular radioactive elements (isotopes) within rocks around fossils or sometimes within a fossil. Radioactive elements decay into different forms (e.g. uranium to lead, carbon to nitrogen) at rates that are constant for a particular element. The rate of decay of the element is independent of the nature of the rocks or the environmental conditions to which they are exposed, so they act as accurate clocks. The half-life of a radioactive element is the time taken for half the atoms of the element to decay, and can be used to calculate the age of the rock in which it is contained (Figure 10 .1.16).

Carbon dating is a commonly used method of radiometric dating. It can only be used to determine the age of artefacts and fossils that were once living (i.e. contain carbon) and are younger than 50 000 years old. This is because the amount of carbon is too small to detect after this time. You will learn more about radiometric dating in Chapter 12.

Thermoluminescence is a technique able to date objects such as pottery, cooking hearths and fire-treated tools up to 500 000 years old. Thermoluminescence is the emission of light from a mineral when it is heated. The amount of light emitted is proportional to the amount of radiation an object has absorbed: the older the object the more light it emits. The intensity of the light can be calibrated to reveal how much time has passed since the object was last heated or burnt in a fire. This technique is used to date artefacts related to human evolution.

Electron spin resonance (ESR) is used to date calcium carbonate in limestone, coral, fossil teeth, molluscs and egg shells. Palaeoanthropologists have used ESR mostly to date samples from the last 300 000 years. Unlike thermoluminescence dating, the sample is not destroyed with ESR, allowing samples to be dated more than once.

Information from fossils

Although fossils can indicate the appearance and structure of an organism, other information can be gained or inferred from examining fossils. For example, animal fossils have been found with young in the womb or inside eggs or guarding eggs (Figure 10.1.17). If young are fossilised next to adults, it is likely that the animal parented the young for a period (Figure 10.1.18). If large numbers of organisms are fossilised together, it could be assumed that they lived in herds. The contents of the animal's last meal may even be preserved in the fossil's stomach area.

BIOGEOGRAPHY

Biogeography is the study of the geographic distribution of organisms and the factors that influence these distributions. Biogeography helps us to understand how the evolution and distribution of species has been shaped by geological processes, such as shifting continents, changing climates and fluctuating sea levels. This understanding can provide insight into the past as well as help us predict the distribution and success of species in the future.

Early biogeographers were interested in understanding how similar animals and plants in different parts of the world, separated by vast distances and huge oceans, came about. For example, they sought to understand why there are marsupial possums in Australia and South America, and whether or not the ostrich from Africa, the rhea from South America and the emu from Australia are related.

Patterns of evidence

When distributions of different groups of organisms in the world are mapped, patterns become evident that give clues to the evolutionary histories of the groups and of Earth itself. Observations by naturalists such as Alfred Russel Wallace led to the division of the world into six biogeographic regions: Palaearctic, Nearctic, Ethiopian, Indo-Malay, Neotropical and Australasian. There are now eight recognised biogeographic regions with the addition of the Oceanic region and the Antarctic region (Figure 10 .1. 21). Marine biogeographic regions include the tropical, temperate and Arctic zones, which differ in climate (Figure 10.1.21). Each region is recognised as having a unique set of related organisms, suggesting that the patterns are a result of evolution.

Continental drift

Scientists noticed that the shape of continents appeared to fit together as if they had been cut from a single larger continent, known today as Pangaea (Figure 10.1.22). The west coast of Africa, for example, seemed to fit well against the east coast of South America. The implications of this were to become important evidence supporting the theory of evolution. If landmasses had at some point been joined, then it follows that organisms on continents separated today might share a common ancestry. Although the theory of continental drift had been proposed in the early 20th century, it was only confirmed much later by geological evidence and the study of plate tectonics. Australia, for example, once part of the southern supercontinent Gondwana, drifted from Antarctica and continues today to move northwards at a rate of approximately 3.7 cm/year for the western side and 5.6 cm/year for the eastern side. Geologists have been able to calculate the rate at which plates are moving and work backwards to determine the point at which the landmasses were joined. This information can help us understand how and when groups of organisms evolved in different parts of the world, and how they may be related to one another.

COMPARATIVE ANATOMY

If you compare the human body to that of a chimpanzee, you can see a striking resemblance in structure. The same applies to many other species, and this is more than a coincidence. Studying the morphology of species, or their body structures, gives an insight into the relationships between species. The field of comparing the structure of organisms is referred to as comparative morphology or comparative anatomy.

Homologous features

Features of organisms that have a fundamental similarity based on common ancestry are called homologous features. Often homologous features evolve different functions, but their similar structures provide evidence that the organisms shared a common ancestor from which they diverged over time. This is known as divergent evolution and is covered in Chapter 9.

Mutations in the DNA sequences regulating the length of the bones in a limb can result in the limb being used in different ways. Close examination of tetrapod forelimbs, for example, shows that the same series of bones is present in each, but the genetic sequence has been modified, resulting in different structures with different functions.

For example, the forelimbs of all mammals, including humans, cats, whales and bats, show the same arrangement (with different lengths) of bones from the shoulder to the tips of the digits, even though these appendages have very different functions: lifting, walking, swimming and flying (Figure 10.1.27).

Homologous features are evident in all groups of organisms. For example, the seeds of cycads, Ginkgo, conifer trees and flowering plants (angiosperms) show a variety of shapes and sizes but they have the same basic structure. The seeds of most conifers are winged and blown about by the wind, whereas the seeds of an Acacia lack a wing but have a tough outer coat and a coloured nutritious appendage to attract ants that disperse the seeds. Despite the variation, these plants all reproduce by seeds, and it can be argued that they have evolved from a common ancestral group (Figure 10 .1. 28).

Vestigial structures

Some organisms possess structures that seem to have little or no function. These structures are often remnants of organs that had a function in an ancestral species but have become reduced in size over time and have ceased to be used. Such structures are referred to as vestigial organs or vestigial structures and they provide further evidence of divergent evolution from a common ancestor.

Examples of vestigial structures include pelvic bones in whales and pythons (Figure 10.1.29); the coccyx, ear muscles, wisdom teeth and inner eyelid in humans; and the reduced eyes of certain blind cavefish and salamanders. Structures such as the wings of flightless birds can be considered vestigial for flight, but in many cases, such as that of the ostrich, the reduced wings provide a new function of temperature regulation.

Analogous features

Organisms can also show similarity that is not due to common ancestry. Flying animals such as butterflies and birds have wings (Figure 10.1.32). Fish and dolphins have swimming appendages and a streamlined body for moving through water. Many burrowing animals have powerful feet. Each of these features can be seen as an adaptation to a particular lifestyle.

Anatomical structures that are found in different groups of organisms, such as wings in birds and butterflies, are described as analogous features; that is, they serve the same function but have evolved independently. Analogous features may evolve because unrelated organisms have experienced similar selection pressures. Thus, when biologists attempt to work out evolutionary relationships, they must distinguish between homologous and analogous features. Identifying features as analogous provides evidence of a convergent pattern of evolution but not evidence of descent from a common ancestor. Convergent evolution occurs when similar features evolve independently in unrelated groups of organisms. Convergent evolution is examined in Chapter 9.

COMPARATIVE EMBRYOLOGY

The comparison of organisms is complicated by the way organisms change substantially between life stages. The metamorphosis of a tadpole to a frog or a caterpillar to a butterfly are examples of this (Figure 10 .1. 3 3). The field of biology that studies the development of a zygote to the form of an adult is called developmental biology. To study evolutionary relationships the embryo development of different species is compared. This field of study is known as comparative embryology.

Homologous structures can sometimes be seen in the embryo of species but not in the adult form. When two gametes (egg and sperm) fuse, a zygote and eventually an embryo forms. The development of an embryo is controlled by a series of master genes that organise the position and rate of growth of cells. Organisms that shared a common ancestor often have similar master genes. This means that the embryos will pass through similar stages of development. A human embryo, for example, passes through a stage in which it has gill slits like those of a fish (Figure 10.1.34). Comparative embryology is therefore an important source of evidence of evolutionary relationships.

BIOCHEMICAL EVIDENCE

Before biochemical or molecular techniques were available, structural (morphological) and functional similarities were the main evidence used to determine relatedness. Humans, chimpanzees, gorillas and orangutans each have forelimbs with hands and five digits (fingers). These morphological similarities can be used as evidence to support the theory that humans are related to the great apes and are descended from a common ancestor. Biochemical evidence, such as DNA sequences, reflects the similarity in the morphology of humans and great apes, indicating that these groups have indeed diverged from a common ancestor.

However, anatomical similarities can also be due to convergent evolution of organisms that did not share a recent common ancestor. A shark and a dolphin, for example, have a similar shape suited to living and swimming in water (Figure 10.1.35). Despite an overall similarity of appearance due to living in similar environments and experiencing similar selection pressures, sharks and dolphins have very different DNA sequences, indicating that they are not closely related. Similar morphological features can result from shared ancestry (homologous features) or from independent evolutionary paths (analogous features) so biochemical evidence plays an important role in clarifying uncertain evolutionary relationships.

Determining relatedness between species using molecules

If species have a very similar set of proteins, chromosomes or DNA sequences, it is evidence that they shared a recent common ancestor. Recent, in evolutionary terms, may be hundreds of thousands, even millions, of years.

All living organisms on Earth once shared a common ancestor. If two populations become isolated from each other, they will accumulate different mutations in their DNA. As time passes, the sequence of nucleotides in their DNA becomes more different and what was once similar DNA gradually diverges (Figure 10.1.36). The more mutations that accumulate in the DNA sequences between two species, the more time will have passed since the two species diverged from their common ancestor. For example, there are more differences between the DNA sequence of a frog and a dog, than between a frog and a toad. This is because a dog and a frog have a more distant common ancestor than a frog and a toad and have therefore had more time to accumulate genetic changes.

Changes in nucleotide sequences are caused by mutations. When a cell copies its DNA, it may make errors. Usually these errors are repaired before mitosis (cell division) occurs. Occasionally these errors are not repaired and become a permanent part of the genome. This is a mutation. If these mutations occur within the germ line cells (cells involved in the production of eggs and sperm), then they can be passed on to the next generation.

DNA and amino acid sequences

As two species diverge from a common ancestor, they accumulate different mutations in their DNA and amino acids. DNA carries the code for amino acids and amino acids are the building blocks of proteins. Changes in the DNA sequence may lead to changes in amino acids and the proteins synthesised. In this way, DNA mutations can lead to changes in an organism's phenotype (features). Sometimes point mutations in the DNA sequence, such as nucleotide substitutions, insertions or deletions, may not cause a difference in the amino acid sequence. This is because the genetic code is degenerate, which means more than one codon (triplet DNA sequence) codes for the same amino acid; for example, GUU, GUC, GUA and GUG all code for the amino acid valine. Consequently, differences in amino acids accumulate more slowly than differences in DNA.

Amino acid changes that do not cause a change in the protein are called conservative substitutions. Semi-conservative substitutions result in an amino acid being replaced by one that is similar in structure but has different biochemical properties. This may lead to a change in the protein. Non-conservative substitutions result in an amino acid being replaced by one that is very different and this usually leads to major changes in the protein and its function.

Even when a point mutation leads to a change in an amino acid, the mutation may not lead to a change in phenotype. All mammals, for example, produce milk containing the protein casein, suggesting that all mammal species have the same gene for this protein. However, if the DNA sequence that makes up this gene is compared, slight differences might be observed. For this reason, and because it is now technically easier and cheaper to analyse nucleic acids than proteins, DNA comparisons are the preferred type of data.

Modern sequencing techniques provide an accurate measure of genetic differentiation between organisms. The exact number of nucleotide differences between organisms can be determined by comparing their nucleotide or amino acid sequences. Figure 10.1.39 shows an alignment of five organisms' amino acid sequences. The amino acid sequence shown is from the mammalian histone protein family, Hl. Histones play important roles in DNA packaging and gene regulation. The evolutionary relationships between the organisms can be estimated by comparing the number of differences in their amino acid sequences.

Table 10.1.1 shows the number of amino acid differences in the mammalian histone protein family, Hl, between humans, mice, rats, cows and chimpanzees. From this data, the evolutionary relationships of these organisms can be inferred. For example, there are six amino acid differences between humans and mice in Hl but zero between humans and chimpanzees. This indicates that humans have a closer evolutionary relationship with chimpanzees than with mice. The amino acid sequence data from Hl reflects what is understood about the evolutionary relationships of these organisms from comparative anatomy, comparative embryology and other biochemical evidence.

Molecular clocks

A molecular clock is a technique that uses the rate of accumulation of mutations in DNA to calculate how long ago organisms diverged from one another. The **molecular clock hypothesis** is the basis of this technique and was first proposed in the 1960s by Emile Zuckerkandl and Linus Pauling. This **hypothesis** states that changes in DNA and proteins are constant over evolutionary time and across different lineages.

The change in DNA over time is also known as the mutation rate and can be expressed as the number of nucleotide changes that occur every million years. The molecular clock hypothesis can be applied by calculating the rate of mutation of a region of DNA, along with the number of differences between the DNA of two organisms, and using this information to estimate how long ago they diverged (Figure 10 .1.42). The more unique mutations each species accumulates, the more time has passed since they shared a common ancestor.

In order to estimate how long ago two lineages diverged, the molecular clock is calibrated using evidence from the fossil record. Techniques such as radiometric dating and stratigraphy (the study of rock layers) are used to date fossils. The molecular clock for a particular gene can then be calibrated by comparing the number of differences in DNA sequences with the dates of evolutionary branch points known from the fossil record of similar organisms.

Limitations of molecular clocks

The molecular clock is a useful phylogenetic tool but it does have some limitations. One of its major limitations is the assumption that the rate of genetic change is constant and therefore accurately represents evolutionary time (Figure 10.1.43). Although we know that genetic difference represents evolutionary distance and that both these factors are positively correlated with time (i.e. the greater the difference between organisms, the more time has passed since they last shared an ancestor), the rate of genetic change over time is not always constant. This means that genetic change is not an accurate measure of time from which we can determine exact dates of lineage divergence.

In order for genetic changes to occur at a constant rate, those changes (mutations) need to be neutral or not affected by natural selection. This needs to be considered when applying a molecular clock to genetic data. Any DNA regions that code for the phenotype of the organism (i.e. its structure or function) are under natural selection and will change according to outside selection pressures. Therefore, the mutation rate of proteins and protein-coding DNA (genes) will not be constant.

Some sections of DNA mutate more frequently than others: that is, at a faster rate. This means there are different molecular clocks within an organism, ticking at different speeds in different regions of DNA. Genes that are essential to an organism's survival, such as those that code for cytochrome c, very rarely accumulate mutations and the gene sequence is therefore highly conserved (mostly unchanged) throughout evolution (Figure 10.1.44). Any variations to the sequence of these essential genes may result in these proteins losing function and the organism dying. For example, changes in cytochrome c may cause the electron transport chain to fail, preventing the formation of ATP. As a result, the organism will die. Sections of DNA that are not essential to the survival of an organism accumulate mutations at a faster rate. As a consequence, there are many molecular clocks within each organism that run at different rates.

The molecular clock is also limited when looking at very recent or ancient timescales. When looking at recent timescales, it is less likely that enough time has passed to generate evolutionarily meaningful fixed differences in the sequences of different populations. Instead, alternative alleles that may be present in both populations will lead to an overestimation of evolutionary distance. Over ancient timescales, single sites in the sequence will have changed multiple times and this is known as saturation. Because we can only know of those changes that can be observed today, a molecular clock will underestimate the divergence that has occurred.

Mitochondrial DNA as a molecular clock

Genetic material is not just found in the nucleus of a cell. Mitochondria found in eukaryotic cells have their own genome (mitochondrial DNA or mtDNA). In humans, mtDNA contains 37 genes tl1at code for 2 ribosomal RNAs, 22 transfer RNAs and 13 proteins.

MtDNA is unique in that it is passed through the maternal line of sexually reproducing organisms; tl1at is, from mothers to their offspring (Figure 10.1 .45). A father's mtDNA is not passed on to his offspring.

Mutations in mtDNA accumulate over time just as they do in nuclear DNA. However, because mtDNA does not have the same repair mechanisms as nuclear DNA, the rate of mutation in mtDNA is usually higher than in nuclear DNA (there are also some highly conserved regions). For this reason, mtDNA can be used as a molecular clock in relatively closely related species, while nuclear DNA is used to compare older lineages. An added advantage of using mtDNA is that it is easier to obtain high yields of DNA because most cells contain many mitochondria.

Phylogenetic trees

**Phylogenetic trees** (or phylogenies, also known as evolutionary trees) are branching diagrams that depict the evolutionary relationships between different groups of organisms. They are constructed using homologous features, both morphological and molecular, to reveal the branching history of common ancestry between groups of organisms. As information regarding the true evolutionary history of an organism is mostly unknown, scientists use evidence from the morphology and DNA or RNA sequences of living species to reconstruct their evolutionary past. A **phylogenetic tree** represents an evolutionary hypothesis.

Today, most phylogenetic trees are built using DNA or RNA sequence data, and organisms are grouped on the basis of the similarity of their nucleotide sequences. Using this technology, we have gained remarkable insight into the evolutionary history of life on Earth.

Building phylogenetic trees

A phylogenetic tree is built by placing organisms in a branching sequence, according to their shared biological characteristics (morphological or molecular). A simple example using morphological characters is seen in Figure 10 .1.4 7. By assessing the characters that different organisms share, the evolutionary relationships between organisms can be hypothesised. Starting with the most shared character, which is assumed to be the most ancestral, organisms are added to the tree sequentially, ending with the least shared character at the top of the tree (Figure 10.1.4 7). In Figure 10.1.47, the most-shared (ancestral) character is the vertebral column and the least-shared character is hair. Each branch in the tree represents a change in character state from the last common ancestor.

**Phylogenetic trees** based on molecular characters (DNA or RN A nucleotides) can be used to compare any organism, even if they seem to have very few characteristics in common. The greater the number of nucleotide differences between DNA or RNA sequences, the greater the distance between them in the tree, reflecting their evolutionary relationships (Figures 10.1.48 and 10.1.49). Most phylogenetic trees are now built using computational methods to generate complex trees from large datasets of thousands of nucleotides.

Figure 10.1.49 shows the evolutionary relationships between four groups of plants: three flowering plants and a pine tree. The pine tree (Pinus sp.) has the longest branch in the phylogenetic tree, indicating it is the most ancient lineage and the most closely related to the common ancestor. This evidence supports what is understood about the evolution of flowering plants from non-flowering plants. The eucalypt ( Corymbia sp.) is the most ancient flowering plant represented in the phylogenetic tree (Figure 10.1.49b) and the bean plants ( Vigna sp. and Glycine sp.) are the most recently diverged group with the most similar DNA sequences (Figure 10.1.49a).

10.2 Recent evolutionary change

Charles Darwin believed that evolution is usually a gradual process. While it is sometimes, recent evidence points to this being unusual. Evolution normally proceeds in rapid bursts, potentially occurring within just a few years. This rapid evolutionary process is known as punctuated equilibrium and is covered in more detail in Chapter 9.

In this section, you will learn about two case studies of recent, rapid evolution. These are cane toads and antibiotic resistance in bacteria.

CANE TOADS

In Chapter 7 you learnt about the highly adaptable cane toad (Rhinella marina) (Figure 10.2.2) and its invasion and population explosion in Australia since its introduction from Hawaii in 1935. In this section you will learn about the rapid evolution of the cane toad in Australia and the evolutionary responses of the native species it interacts with.

The impact of cane toads in Australia

The cane toad continues to reproduce and spread at a rapid rate across Australia. Since their release, they have colonised a wide zone stretching from northern New South Wales to west of Darwin. The spread continues at an accelerating rate. Toads are projected to take over most of the northern half of Australia.

The cane toad was introduced to Australia as a biological control measure to save the sugar cane industry from cane beetles that were destroying crops. Cane toads showed little interest in cane beetles, but had a devastating effect on native wildlife. Toads consume many other water species, including insects, fish and reptiles. This also greatly reduced the amount of food available to native predators. Most importantly, toads have large poison glands on their backs. Often predators that eat toads die as a result. Native predator numbers have sharply declined everywhere toads have migrated. The toads have particularly affected Australia's reptiles.

Toads also devour dung beetles in large numbers. Dung beetles bury cattle dung. When dung beetles are unavailable, the dung piles up and affects cattle health. Thus cane toads also impact Australia's cattle industry.

Cane toad evolution

In less than a century, toad behaviour and phenotype has changed markedly. Cane toads are an example of modern-day rapid evolution.

The toads first introduced to Queensland were not especially active. Toads at the expansion frontier are different. Compared to their recent ancestors, frontier toads are larger (since large animals are faster), plus their legs are proportionally about 10% longer and stronger. The frontier toads' behaviour is different as well. Whereas ancestral toad populations move about 10 m/day, in no particular direction, frontier toads move 10 times that distance and in straight lines. Immediately after introduction, the area of toad distribution initially expanded by around 10 km/year. Now, the increase is around 60 km/year.

Over millions of years in their Amazon homeland, toads developed an ability to chemically detect the presence of their own species' eggs. If laying females sense the eggs of other individuals, they eat the eggs, clearing the water body for their own. Thus toads compete reproductively. Individuals able to escape the competition have an advantage. The new environmental context in Australia meant that speedy toads reproduced more successfully.

In what some researchers termed the 'Olympic Village effect', fast toads breed with other fast toads; in each generation the fastest offspring reproduce. This is an extreme example of selection pressure. It means that frontier toads are physically diverging from the core population at an unusually rapid rate.

The selection for speed only applies on the edge of toad territory. Toads deep within the species' range experience a different pressure. No matter how quickly they move, they cannot escape other toads, and speed offers no advantage. Those toads are thus relatively sedentary.

Once the main front catches up, the super-toads are disadvantaged. Many of the most robust individuals suffer from severe spinal arthritis in older age that restricts movement to short, slow hops. They also reproduce less. In that case, the selection pressure reverts to normal: the extreme phenotype is selected out, exaggerating the sharp difference between frontier and main-population toads.

While the divergence phenomenon lasts, it provides an unusual insight into evolutionary processes.

ANTIBIOTIC RESISTANCE

Bacteria is literally all around us (Figure 10.2. 7). We co-exist with some species. Gut bacteria, for example, are essential for food digestion and we could not live without them. Yet those or other species cause harm if they enter other parts of the body, whether via wounds, unhygienic surgery, inhalation or other means. The infection causes an immune response. That is when the body's defences, particularly white blood cells, attack the foreign cells.

Normally our immune systems cope with minor infections. However, in cases of weakened immunity or extensive infection, our immune systems cannot cope. Throughout human history, severe infections usually proved fatal. The bacteria's exponential growth (increasing rapidly at a nonlinear rate) overwhelms our immunity.

This changed from the 1940s, with the development of antibiotics. These are drugs that disrupt bacterial reproduction without effect on human cells. With the exponential reproduction halted, our immune systems can easily deal with the infection. The drugs have saved at least 200 million lives that otherwise would have been lost to infection.

The active ingredients of antibiotics are naturally occurring compounds, evolved by various kinds of organisms as defence against bacterial infection (Figure 10.2.8). Countless such compounds exist in nature. Yet so far only around 100 antibiotic drugs have been developed, falling into seven main classes.

However, just a few years after first being introduced, antibiotics had stopped working in certain cases. The target bacteria evolved resistance. Antibiotic resistance is now a serious international health concern.

Evolved resistance

The genetics of bacterial variation is both simpler and more complex than for more advanced organisms. It is simpler because bacteria reproduce asexually. Bacteria do not shuffle two sets of parental genes during meiosis (cell division). Each bacterium has only one parent, of which subsequent generations are nearly perfect clones. Mutation is one of few sources of genetic variation. Yet the situation is also complex because bacteria (and most other microbes) are capable of horizontal gene-transfer. This means that any microbe can transfer genes to any other. In this way, bacteria can rapidly acquire tremendous genetic variation and new traits from unrelated species. The greater the variation, the more natural selection has to work with, and the more adaptable a species will be. Because bacteria can reproduce so rapidly, one bacterium quickly becomes two, two become four, four become eight, and so on. Bacterial populations can double in 4 to 20 minutes, giving them the ability to form millions of cells in as little as a few hours. This rapid rate of reproduction means that bacteria can also rapidly evolve.

Natural selection works the same way for all organisms. Individuals most suited to their environment survive to reproduce, whereas the less fit reproduce less or not at all. Selection for certain traits means preservation of the underlying alleles for that trait. The proportion of the population carrying alleles for advantageous traits increases over successive generations.

In practice, each antibiotic prevented reproduction in most bacteria but not all. By chance, some were naturally resistant. The unaffected few passed on their alleles for resistance to subsequent generations. Survivors may also have acquired alleles for resistant traits from other species. Through many generations, the most effective combination of alleles for resistance increased in the population.

In other words, bacteria evolve, and at tremendous speed. Drugs that once reliably inhibited bacteria's exponential reproduction rapidly become ineffective. New bacterial strains continue to emerge, evolving resistance to new treatments as they are discovered and introduced to the human population.

The current crisis

Although many antibiotics still work, many others do not. Because of evolved resistance, some forms of infection now require multiple antibiotics in combination. Many infections are becoming less treatable or untreatable in some cases. People are again dying of infection, and the problem will continue to worsen.

The situation arose from widespread misuse of the drugs. A major cause was use of low-dose antibiotics in livestock feed. Modern factory-farming methods put animals into unnaturally close contact in unhygienic conditions where disease is common (Figure 10.2.9). Using antibiotics in food prevents low-level infections that would slow animals' growth and lower financial returns. In the USA, livestock feed accounts for 80% of antibiotic usage. Continual weak doses are a perfect way to cultivate resistant bacterial strains.

A second major cause has been physicians inappropriately prescribing antibiotics for human patients (Figure 10.2.10). Many antibiotic prescriptions are unwarranted. A common situation is patients requesting or being given antibiotics for cold symptoms, although antibiotics have no effect on viral infections. In other cases, patients often either do not finish the full course of antibiotics, or continue taking the drugs for too long. Short, high-dose applications are most effective.

Many of the world's leading medical authorities agree that a crisis is imminent. Solutions may involve the development of new drugs plus tighter guidelines on usage.

Misuse has undoubtedly accelerated antibiotic resistance. However, the problem would have eventually arisen anyway. Bacteria and other life forms have been locked in an evolutionary arms race for billions of years. Every time one side develops a new weapon, the other adapts.

Therefore, there can never be any single, permanent solution. We must understand that evolution is a constant fact of life. Thus each drug has a limited useful lifespan; every new introduction must include plans for its replacement.

Bacteria make it easy to appreciate evolution: in them we can literally watch it happening.

Chapter 2 Reproduction

2.1 Asexual reproduction

Individual organisms do not live forever. The continuity of life from generation to generation is the result of reproduction. Reproduction is one of the distinctive characteristics of living organisms.

The simplest way that organisms can reproduce is asexually. Asexual reproduction is the production of identical offspring from just one parent. It produces new individuals or offspring by mitosis, in which each daughter cell receives a copy of every chromosome of the parent cell.1'he offspring are therefore clones, that is, they are genetically identical to the parent unless genetic mutations occur.

Asexual reproduction occurs in every major group of life—Archaea, Bacteria, Protista and Fungi, as well as in many plants (Plantae) and some animals (Animalia). Some species are capable of both asexual and sexual reproduction, either at different stages of their life cycle or under different environmental conditions. In this section you will learn about the different methods of asexual reproduction, the types of organisms that use these methods to reproduce and evaluate the advantages and disadvantages of asexual reproduction for the continuity of species.

ADVANTAGES AND DISADVANTAGES OF ASEXUAL

REPRODUCTION

Asexual reproduction is an efficient and simple way to reproduce because individuals do not need to find a mate and can quickly reproduce large numbers of offspring in isolation. It uses the cell division process of mitosis, which is less demanding on an organism. The asexual reproductive strategy works well when environmental conditions are ideal and relatively static. For a species already well suited to a stable environment, there is no disadvantage in producing offspring with the same inherited traits. Asexually reproducing organisms are therefore commonly found in relatively stable and uniform environments to which they are well suited.

However, when environmental conditions are variable, asexually reproducing populations are at a disadvantage. Because asexual organisms are genetically the same, they will all respond to the environment in the same way. Lack of genetic variation in a population means that there will be no unusual individuals that may be able to tolerate new selection pressures in changed environmental conditions. As a group, they will either survive or die.

The advantages and disadvantages of asexual reproduction are summarised at the end of this section.

METHODS OF ASEXUAL REPRODUCTION

Asexual reproduction is the most common method of reproduction for unicellular organisms. This is because there is no cell specialisation in unicellular organisms, so there are no reproductive organs or germ cells to produce gametes.

Many multicellular organisms also have the capacity to reproduce asexually. In multicellular organisms, the new individual arises from body cells, known as somatic cells. Asexual reproduction takes place in a variety of organisms and can be by:

fission-splitting of one cell into two (binary fission) or many (multiple fission), of equal size

budding-outgrowths from a parent cell, each smaller than the parent

fragmentation of body parts

spore formation

vegetative reproduction in plants

parthenogenesis-in some female animals.

BACTERIA

Bacteria are unicellular, microscopic prokaryotes that reproduce asexually. You learnt about bacteria in Year 11. Fossil evidence has confirmed that bacteria were the first type of living organism on Earth, evolving at least 3. 7 billion years ago. Bacteria have very diverse cell chemistry (metabolism) and can survive in a wide range of environments. They play an important role in ecosystems because they decompose many substances, including plant and animal remains and wastes. There are specialist bacteria that fix atmospheric nitrogen into a form that plants can use. Bacteria cause many diseases for plants and animals, such as tomato wilt, citrus canker, tetanus, cholera and toxic shock syndrome. Humans use specific bacteria in safe ways to manufacture foods such as cheese and yoghurt and medicines such as antibiotics, enzymes and prescribed drugs including human insulin for diabetics.

Reproduction by binary fission in bacteria

Due to the lack of organelles and smaller amount of DNA, cell replication in prokaryotes (Archaea and Bacteria) occurs more quickly than in eukaryotes. It is not the same process as mitosis because there is no nucleus in a prokaryotic cell. 1'his asexual process is called binary fission because a single parent cell splits into two approximately equal daughter cells. Binary fission is a relatively rapid form of reproduction that produces new cells genetically identical to the parent.

Like mitotic division, binary fission is an exponential process because the population doubles after every cycle of division. In ideal conditions, some bacteria can undergo binary fission every 20 minutes, meaning that the number of cells can double every 20 minutes. This means that in six hours up to 18 cycles of binary fission could occur and in this time one bacterium could have produced a population of 262144 individuals.

PROTISTS

All protists are eukaryotes that live in aquatic or moist environments. They can be unicellular or multicellular, but if multicellular they are not multi-tissued (i.e. their cells are not organised into functional tissues). Protists can reproduce both asexually and sexually. This extensive diversity within Protista makes it difficult to classify them as one group. Currently, protists are classified as any eukaryotic organism that is not a fungus, plant or animal. Because protists do not fit into any of the other kingdoms, they have been temporarily grouped together until there is more taxonomic knowledge available.

Protists include protozoans such as Euglena, water moulds, slime moulds, dinoflagellates, diatoms and some algae. Most protists are free-living but some are parasites (e.g. plasmodia that cause malaria and Giardia that causes intestinal disease with abdominal pain, diarrhoea and nausea when a person is infected from contaminated water.

Reproduction by binary fission in protists

The typical mode of reproduction for most of the protists is asexual binary fission. The binary fission of protists is different to that of bacteria because protists have a membrane-bound nucleus that needs to be replicated. For protists without a cell wall, the body of an individual is simply pinched into two parts or halves; the parent cell disappears and is replaced by a pair of daughter nuclei in two new cells, although these may need to mature to be recognisable as members of the parental species. The length of time needed for binary fission varies among groups of organisms and with environmental conditions, ranging from a few hours in an optimal situation to many days. In protists the binary fission can be along a transverse or longitudinal axis or across a diameter, dividing the cytoplasm in half.

Multiple fission is common for some parasitic protist species. The nucleus divides repeatedly to produce a number of daughter nuclei before division of the rest of the cell occurs. Eventually these daughter nuclei become the nuclei of multiple progeny after repeated cellular divisions.

It is interesting to consider that after fission occurs, the parent cell no longer exists but neither has it died. It has redistributed itself to the daughter cells. In other forms of unicellular asexual reproduction (e.g. budding and spores) the parent cell continues with its life and death cycle.

Reproduction by budding in protists

Although fission is the most common form of asexual reproduction among protists, there are also other methods such as budding. Budding occurs when a new identical organism grows from the body of the parent. This usually occurs on the outside of the cell from which it detaches to live independently or sometimes remains in contact to form a colony. In some species a bud forms internally. The new nucleus is formed in a similar way to fission but, unlike fission, the division of the cytoplasm is unequal. At first the new organism is much smaller than the parent.

FUNGI

The kingdom Fungi includes species that are commonly known, like mould, mildew, mushrooms, yeasts, lichens (a symbiotic organism of fungi and algae) and truffles. Fungi are composed of eukaryotic cells that secrete enzymes over the surface of their food and absorb the breakdown products directly. Some fungi reproduce asexually by spores released from fruiting bodies. Mould, mushrooms and puffballs are examples of fungi that reproduce by spores. Other fungi, such as yeasts, reproduce by budding.

Reproduction by budding in fungi

Most yeasts reproduce asexually by the asymmetric division process called budding. First the parent yeast cell produces a small outgrowth that grows larger and forms a bud. The nucleus of the parent cell splits off a smaller daughter nucleus, which migrates into the daughter cell. The bud detaches from the parent by pinching inwards at the base, although it may remain in contact with the parent cell. At this stage the bud is much smaller than the parent but is genetically identical. Repeated budding forms a chain of connected but independent cells.

The clouds of blue-green powder that come off the surface of a mouldy lemon are a type of asexually reproduced spore of blue mould (Penicillium expansum). These spores, produced by budding, are called conidia. In Penicillium expansum these spores are formed in long chains.

Reproduction by spores

Spore formation is the method of reproduction most commonly associated with fungi. Spore formation can be either asexual or sexual. Spores that are produced asexually are called mitospores and are produced by mitosis. Spores produced sexually by meiosis are called meiospores. You will learn more about meiospores in Section 2.2.

Mitospores are haploid (one set of single chromosomes) reproductive cells capable of developing into an adult without fusion with a second cell. The daughter cells are genetically identical to the parent fungus and are usually produced in large numbers. These spores are cells that are encased in a protective coating that enables them to survive in unfavourable environments.

Some fungi produce a cluster of spores inside a structure called a sporangium (plural sporangia). The spores are released when the sporangium wall disintegrates and are dispersed by wind or water. When a spore lands in a suitable environment it germinates, forming a new fungus. Spore formation and dispersal can rapidly increase identical fungal cells, expanding and spreading the population of a fungal species.

PLANTS

The kingdom Plantae is the most conspicuous group of producer organisms on land. As with some protists and all the cyanobacteria, plants are autotrophs (self-feeders). Plants are multicellular and include small and structurally simple forms, such as mosses and liverworts, and large, more complex forms such as ferns and seed plants. Living seed plants include cycads, ginkgo, conifers and flowering plants, but in the current era flowering plants dominate the landscape. Plants use both asexual and sexual modes of reproduction. Flowering plants produce seeds by sexual reproduction. Other plants reproduce by a variety of asexual means and some species are capable of both.

Natural vegetative reproduction

Many members of the kingdom Plantae, including flowering plants, can reproduce asexually by vegetative reproduction. This does not involve the formation of seeds or spores. Instead it is the growth of specialised plant tissues that can grow into a new plant if it becomes separated from the parent plant. Naturally occurring vegetative reproduction may arise from many parts of the plant, such as the leaves and stems. An advantage of vegetative reproduction is that it can produce a rapid increase in the number of plants growing ill a favourable area so that they outcompete, or displace, neighbouring species. In contrast, seeds produced by sexual reproduction may land in unfavourable conditions and fail to germinate. Potential disadvantages of vegetative reproduction are competition from sister and parent plants for resources, and Jack of genetic variation to protect the population against disease or changing environmental conditions. Some types of vegetative reproduction ill plants are described ill Figure 2.1.15.

Artificial asexual propagation of plants

In the agricultural and horticultural fields propagation from fragments of plants, by grafting, cuttings or tissue culture, is advantageous for maintaining varieties that are genetically identical. It guarantees that the features of a desirable plant are preserved from generation to generation and can be produced in commercial quantities. Such techniques are also being used in research and recovery programs for endangered plant species, such as the round-leaved sundew (Drosera rotundifolia) and the Wollemi pine (Wollena nobilis).

ANIMALS

The kingdom Animalia includes an extremely diverse group of organisms, living in the sea, in freshwater and on land. All animals are multicellular. Most animals can move around to find food, nesting sites and mates. But many marine animals cannot, such as coral polyps, sponges and barnacles.

Asexual reproduction is less common in animals but it is not absent. The ability to move about means that animals are more likely to be able to find a mate and a suitable environment for sexual reproduction. When mates are not available or rapid identical reproduction is an advantage, asexual methods are used by some animals. Asexual methods of reproduction in animals include regeneration, fission, budding, fragmentation and parthenogenesis. Sometimes a species alternates asexual and sexual modes of reproduction, gaining the advantages of both, while avoiding the disadvantages.

Regeneration

Regeneration occurs when a detached part of an individual grows into another individual. The echinoderms, which include sea stars, sea cucumbers and sea urchins, undergo tissue regeneration to grow replacement body parts. If the replacement is a large enough section with enough essential body tissues, the part could be regarded as reproduction of a new individual. However, the growing of a replacement arm for an otherwise intact sea star is not reproduction, but regeneration of a body part of the same adult. Likewise, the growing of a replacement tail for a lizard, a new leg for an axolotl, or replacement teeth for sharks, are not forms of reproduction.

Fission

As already described for unicellular organisms, longitudinal fission occurs when a cell splits along its longest axis and transverse fission occurs when the cell splits across its shortest axis. Fission can also occur in multicellular animals. A process similar to transverse fission is called strobilation in animals. A segment on the parent organism forms and when it matures it detaches to become a new individual.

Flat-worms like planarians have been studied extensively for their ability to split and grow into new individuals. They can do this by longitudinal fission or fragmentation.

Budding

In a similar way to the fungi and unicellular protists, there are some animals that produce genetically identical but smaller buds as outgrowths from the parent body. The difference to fission is that there is an unequal division of cytoplasm with the buds being much smaller than the parent. Budding has been extensively documented in a small, multicellular animal called Hydra that is common in fresh water.

Sponges are aquatic animals that display asexual reproduction. In sponges this occurs by budding, which happens when a part of a sponge is broken off or one of its branch tips is constricted, and then this small piece grows into a new sponge. They may also reproduce asexually by producing multiple buds as packets of internal cells called gemmules, sometimes known as survival pods. If the parent sponge experiences damaging environmental conditions, such as drying out, the gemmules can remain dormant until conditions suit them to revive. Fragmentation and regeneration also occur in sponges.

Like other simple organisms with long evolutionary histories, sponges exhibit sexual reproduction as well as asexual reproduction.

Fragmentation

Fragmentation is similar to fission, but it happens in multicellular organisms. The body of the organism breaks into two or more parts, each of which regenerates the missing pieces to form a new, complete individual.

Fragmentation is common in some flatworms, marine worms and echinoderms. These organisms retain a population of stem cells throughout their life. The stem cells can develop into any cell type in the body, giving these organisms the remarkable ability to regenerate a body part that is completely lost through injury or even to reproduce new individuals.

Many species of annelids (segmented worms) reproduce via fragmentation. For example, California black worms have 150-250 body segments and live in muddy sediment beside marshes and ponds, hence their other common name of mudworm. They are widely used for bait and aquarium fish food. Like earthworms, these worms are hermaphrodites, meaning they have both male and female reproductive parts in one body and can reproduce sexually. However, it is more common for them to reproduce using fragmentation. In this case, blackworms break apart and each segment can become a new worm. Hermaphrodites are covered in more detail in Section 2.2.

Parthenogenesis

The development of an egg in the absence of fertilisation is an unusual form of asexual reproduction known as parthenogenesis, a Greek term meaning 'virgin birth'. Because it involves the development of an egg, parthenogenesis can only occur in females. Parthenogenesis is a normal part of the life cycle of some reptiles, birds, insects (bees, wasps and ants), rotifers and nematodes. There are at least 2000 species that are known to use parthenogenesis, even if it is not their usual means of reproduction.

SUMMARY OF THE ADVANTAGES AND DISADVANTAGES OF ASEXUAL REPRODUCTION

Given the abundance of organisms that reproduce by asexual means to ensure the continuity of their species, there must be significant advantages to asexual reproductive strategies. At the same time, because asexual reproduction almost always results in genetically identical offspring, there are some disadvantages. The key advantages and disadvantages of asexual reproduction are summarised in Table 2 .1. 2.

FEATURES OF SEXUAL REPRODUCTION

Life cycles of sexually reproducing organisms follow a pattern called alternation of generations. They alternate between haploid (n) and diploid (2n) stages. In most animals, including humans, the **diploid stage** is what we see as everyday body structure and function, and the **haploid stage** is the unseen internal production of sperm in males and eggs in females. For plants, there is a true alternation between a **gametophyte stage** (all cells haploid) and a **sporophyte stage** (all cells diploid) with the latter being the plant structure that we usually observe. The time spent in each stage varies greatly between different species.

Multicellular organisms are composed of two main types of cells: somatic cells and germ cells. Somatic cells are all the cells in the body of an organism apart from the sex cells (gametes). Examples of somatic cells in animals include skin cells, muscle cells and nerve cells. Germ cells are the cells that give rise to gametes, which are the specialised sex cells that combine in sexual reproduction.

Male gametes (sperm) and female gametes (eggs or ova) are often different in appearance. The formation of gametes occurs by meiosis in specialised reproductive organs, called gonads. In animals the gonads are the testis (plural testes) or the ovary. Although the male and female gonads look very different, they have the same function of producing haploid cells for reproduction. The sperm or eggs formed as a result of meiotic cell division are haploid, which means the number of chromosomes in the gametes is halved. A normal eukaryotic organism is composed of diploid cells (represented as 2n) that carry one set of chromosomes (n) inherited from each parent. When the parental chromosomes pair up at fertilisation, they complete the diploid set for the new sexually reproduced offspring.

Female gametes (eggs or ova) are large, immobile cells. They contain the food stores needed for the development of the embryo. The male gametes (spermatozoa or sperm) contain limited food reserves and usually have a tail (flagellum) for motility, which enables them to move towards an egg.

After fertilisation the two haploid cells fuse to form a diploid zygote. The zygote then divides by mitosis to produce a large number of cells forming an embryo. The embryonic cells then differentiate to form the specialised tissues that make up a fetus. After birth for animals, or germination for plants, the organism continues to develop by mitotic divisions and becomes an adult. The reproductive cycle may then begin again. Fetal development in mammals will be covered in more detail in Section 2.3.

INTRODUCING VARIATION-SEXUAL REPRODUCTION

Sexual reproduction requires the formation of gametes by me process of cell division called meiosis. Unlike mitosis it produces cells that are not genetically identical to each other or to the parent cell. There will be similarities in genetic content between parents and offspring, but the offspring are always genetically different from the parents and from each oilier (except for identical twins). Here lies much of the advantage and disadvantage for any species using this form of reproduction. However, the widespread occurrence of sexual reproduction in almost all eukaryotic organisms shows that the long-term benefits to me species far outweigh any costs to the individuals.

Advantages of sexual reproduction

The main advantage of sexual reproduction is the introduction of genetic variation that enables a species to survive and reproduce in varied and changing environments. In the long term, increased genetic variation allows greater adaptability and evolutionary potential in changing conditions. The pool of genetic variation in a population also facilitates the selection of beneficial traits and elimination of unfavourable traits, according to the survival and reproductive success of individuals. This process ultimately benefits the population, as those individuals that are most successful will reproduce, increasing beneficial genetic variants in the population.

Disadvantages of sexual reproduction

While there are advantages to sexual reproduction, there are also many disadvantages. In both plants and animals, energy must be used to produce gametes and ensure that mature gametes are brought together at the right time of year. In other words, not all the food the parent makes or eats is used to maintain its own body systems. For plants, relying on pollination by wind or an animal pollinator poses a risk, as does relying on environmental factors or another species for seed dispersal.

Finding and competing for a mate can be time consuming, energetically costly and risky. Some reproductive behaviours, such as calling or displays to attract mates, might also attract the attention of predators. In some animals, mating leads to considerable, and potentially harmful, competition between males. Providing parental care and protection for offspring is a substantial investment of time and resources, often shortening the lifespans of parents due to the excess expenditure of energy. Although individual adult animals are disadvantaged by me stress of parental care, the careful rearing of young has evolutionary advantages for the species.

FUNGI

The mycelium of a fungus is a mass of branching, threadlike hyphae (singular hypha), often growing underground, sometimes over a surface. The hyphae are the vegetative feeding state of me fungus and absorb the food digested by secreted enzymes. The nuclei of me mycelium cells are haploid (n).

In the reproductive state, a fungus produces a more conspicuous fruiting body or stalk with spores that result from either asexual or sexual reproduction. You learnt about asexual reproduction in fungi in Section 2.1.

Although less common, many fungi can produce spores sexually with alternation of generations, as well as asexually. The mating cell types, male and female, are formed for sexual reproduction.

There are many variations in fungal sexual reproduction but all include me following three stages: **plasmogamy** (cytoplasm union), **karyogamy** (nuclear union) and **gametangia** (haploid spores formed by meiosis).

Sexual reproduction introduces genetic variation into a population of fungi by combining genetic material from two fungal strains.

PROTISTA

Some unicellular organisms can reproduce sexually as well as asexually. This includes a group of protists known as ciliates. Ciliates have two types of nuclei: a micronucleus, which contains a normal diploid (2n) set of chromosomes; and one or more macronuclei (singular, macronucleus), which contain many sets of chromosomes (polyploid). You learnt about asexual reproduction in protists in Section 2.1.

Ciliates (known as paramecia) of the genus Paramecium usually reproduce asexually by mitosis of the micronucleus. The macronuclei simply pinch into two roughly equal pieces then fission occurs across the middle of the cell.

However, under stressful environmental conditions Paramecium can also reproduce sexually, by a method known as conjugation.

The benefit for a Paramecium species of switching to sexual reproduction and alternation of generations is that it introduces genetic variation, improving their ability to adapt to changing environments.

PLANTS

All groups in the kingdom Plantae reproduce sexually. Some also have asexual means of reproduction as detailed in Section 2.1. The two structures most commonly observed for sexual reproduction in seed-producing plants are seed cones of the gymnosperm plants and flowers of the angiosperm plants. The other plant types, grouped here as mosses and ferns, reproduce sexually with spores formed using less prominent structures.

Plants have a life cycle that alternates generations between haploid and diploid stages.

Ferns and mosses are the plant groups for which alternation of generations is most clearly displayed during their life cycles because both the sporophyte and gametophyte stages are free-living independent plants. In the seed-producing plants, gymnosperms and angiosperms, the diploid sporophyte stage is the dominant plant structure with the gametophyte stage present only as a small extension from the main plant, either a cone or a flower. The seeds are formed in or on the sporophyte after pollination. Seeds have great economic and survival value for humans as they are an important source of nutrition in the human diet.

Mosses (Bryophyta)

Mosses and liverworts are the only plant groups without vascular systems, a state that restricts their size to low growing and their habitat to moist, shady places. Mosses reproduce sexually with spores and are characterised by small, flat, green leaf-like structures above ground and root-like structures called rhizoids below ground. Mosses do not have true leaves or roots because they lack vascular tissue (xylem and phloem).

TI1e moss life cycle follows a sexual pattern of reproduction with alternation of generations and production of haploid meiospores by meiosis from a diploid plant (sporophyte). The meiospores grow into gametophytes that form male and female gametophores, which in turn fertilise to form a sporophyte again. Spores are released from capsules on the sporophyte to produce new gametophytes after meiosis and so on. Each spore capsule has a ring of tissue around the opening, made of triangular, close-fitting flaps that open and close to release spores when the moisture level in the surrounding environment is ideal. The haploid life stage of moss is dominant so we observe the gametophyte generation as the main plant.

Ferns (Polypodiopsida)

Unlike mosses ferns have vascular tissues that transport water and soluble nutrients. They are a diverse group of plants characterised by the absence of flowers and fruit, the production of tiny spores instead of seeds, and by alternating generations of free living , spore producing plants (sporophytes, diploid) and gamete-producing plants (gametophyte, haploid). Ferns are different from other land plants in that both the gametophyte and the sporophyte phases are free-living. The sporophyte is the dominant stage in the life cycle of a fern.

Gymnosperms-cone-producing plants

Gymnosperms (meaning naked seed) are the vascular, non-flowering seed plants. They include the conifers (pine, spruce, fir, cedar and redwood trees), the cycads and ginkgo. The world's tallest, widest, heaviest and oldest living trees are all conifers of various types. The seeds of gymnosperms are produced by cones instead of flowers and then mature they are exposed rather than surrounded by a fruit. Pollination is always by wind because conifers do not have flowers to attract pollinators. Conifer seeds start their development as a **haploid stage** inside a protective cone that is woody for many species or, less commonly, fleshy in conifers like the Podocarpus. Cones take from four months to three years to reach maturity, and vary in size from 2 mm to 60 cm long depending on the species.

Generally a conifer has a **haploid stage** with separate female and male cones, called the seed cone and pollen cone respectively. Small pollen grains (called microspores) develop in the male cones and when released they are transported by wind to the female cone which contains the megaspores. A pollen tube grows towards the ovule enclosed inside the female cone. In some species it may take months for the growing pollen tube to make its way to the megaspore inside the ovule where the mature haploid sperm can fertilise the haploid egg to form a new diploid cell. A conifer sperm has no tail and is not motile. It is carried on the tip of the growing pollen tube. Like the flowering plants (angiosperms), each pollen grain divides into two sperm (n) and only one will fertilise the egg cell and contribute its genetic information to the new seed (2n) that develops.

An unusual feature of conifer reproduction is that it can take two or more years from the **haploid stage** of pollination to the **diploid stage** of fertilisation and the release of the seed. Pollination is seasonal, once a year in spring, a fact noticed by the many hay fever sufferers. The female megaspore that has been formed earlier by meiosis remains in a dormant state until the pollen tube starts to grow, which may take months. Fertilisation is often a year or more after the pollen grains are deposited on the female cone. Subsequently the seed can take another year to mature and be released.

Angiosperms-flower-producing plants

Angiosperms are the flowering plants. Sexual reproduction for them involves meiosis, which produces haploid cells that then undergo several mitotic divisions and develop into haploid male or female gametophytes that are genetically different. The male gametophyte is the pollen grain, which contains sperm cells, held on the anther at the top of the stamen. The female gametophyte stage is the embryo sac, which contains the egg held inside an ovule in the plant's ovary at the base of the flower.

Flower structure

The reproductive organs of flowering plants are contained in the flowers. On the outside of a flower are sepals and petals. The sepals enclose and protect the other parts of the flower during the bud stage. Sepals are usually small and green, but in a few species they are large and brightly coloured.

The petals are arranged in a circle or cylinder around the reproductive organs. Inside the ring of petals are the stamens, which are the male reproductive organs. Each stamen usually has a long stalk called the filament with a small yellow pollen sac on the end, called the anther.

In the centre of the flower is the female reproductive organ, called a pistil. The term carpel can also be used to describe the female reproductive organ of a flower. The pistil can consist of one carpel or many, which may be fused or separate, combining to form the pistil in a flower. Each pistil consists of an ovary, which is a central swelling at the base, and a slender stalk called the style bearing the stigma at the top, which is the receptive surface for pollen. Inside the ovary are one or several ovules that contain the female gametophytes. The ovary is usually seen as a swelling and may be positioned above the petals and sepals or below.

Most flowers contain both male and female reproductive organs and are known as bisexual (e.g. rose, tea tree, mango). In some species, the flowers are unisexual, with the male and female organs in separate flowers on the same plant (e.g. maize, zucchini).

Pollination and fertilisation

Wind, insects or birds carry out pollination in most plants. Less commonly, the agents of pollination are bats, other animals or water.

Pollination occurs when a haploid male pollen grain lands on a receptive female stigma and begins to grow a tube. One of the cells in the pollen grain produces a tube that penetrates the surface of the stigma. The pollen tube carries two sperm cells and grows down through the style inside specialised nutritive tissues, towards the ovary, until it reaches an ovule. Fertilisation takes place in the ovule when the egg fuses with one of the two sperm cells, forming a diploid cell that grows into a seed. Other cells in the ovule combine with the second sperm cell and then divide rapidly to provide tissue called endosperm that nourishes the developing embryo inside the seed.

Although most flowers are bisexual, most of them do not self-pollinate because this would reduce the genetic variation in the offspring. Flowering plants have efficient mechanisms for preventing self-pollination and promoting cross­pollination. One mechanism includes the maturation of the anthers (male) and the stigma (female) at different times. For example, the stigma at the top of the pistil is sticky (for pollen grains to adhere) at different times to when pollen of the same flower is mature. Plants may also reject their own pollen, preventing the pollen tube from growing and therefore avoiding self-pollination.

Successful fertilisation can only occur following acceptance of the pollen grain by the stigma and of the pollen tube by the style. Unlike animals, a plant cannot move around to select its partner, so plants have evolved mechanisms to choose gametes from appropriate partners and reject those that are inappropriate. Pollen of each species has a characteristic shape that allows recognition by the plant.

Seeds and fruit

After fertilisation the ovule develops into a seed protected by a tough outer seed coat. This process involves the ovule (in which the zygote develops) expanding, the endospenn forming, and the zygote undergoing a series of mitotic divisions to produce a multicellular embryo. All the cells are diploid. The embryo develops seed leaves (cotyledons) and a root tip, and epidermal and vascular tissues begin to form.

As the ovule changes into a seed, the ovary containing the ovule becomes a mature fruit. Nutrition for seed development and fruit growth is obtained through the phloem and xylem of the parent plant. Fruits are specialised structures that protect the seeds and may enhance seed dispersal. Some fruits contain a large store of nutrition to feed the seed after it germinates. Humans have taken advantage of this for their own food supply, and in some cases, have selected and bred fruit­bearing plants to increase or improve the flesh of the fruit. The many examples include apples, citrus, mango, watermelon and passionfruit, all of which carry seed inside fleshy nutritious fruit.

Germination and development

The embryos in seeds lie dormant until conditions are appropriate. Water, oxygen, temperature and day length are major environmental factors that influence seed germination. Many seeds can remain dormant and only germinate when conditions are favourable. Seed dormancy allows plants to disperse their progeny into the future, something that animals generally cannot do. Dormant seeds can wait months, years and even decades to continue propagation of their species. The oldest known germinating seed was almost 2000 years old.

Dormant seeds have a water content of around 10% compared to regular plant cells at 85% or more. When mature, a seed already contains within it a multicellular diploid embryo and one or two cotyledons (seed leaves) surrounded by the nutritious endosperm tissue and protected by a tough seed coat.

ANIMALS

Members of the kingdom Animalia have an amazing diversity of sexual reproductive strategies, often involving complex behavioural, physiological and structural adaptations for attracting mates, mating, and protecting and nurturing developing offspring.

As animals moved from protective aquatic environments to exposed terrestrial environments, there was a need to shift from external fertilisation to internal fertilisation to prevent dehydration of gametes. This evolution of reproductive strategies is evident in animals today, including the land-based mammals. It is understood that aquatic mammals like seals, dolphins and whales are species that returned to life in the water after an ancestral phase on land. These mammals still reproduce by internal fertilisation.

Most other aquatic animals reproduce by external fertilisation. The amphibians return to aquatic environments to lay and externally fertilise their eggs. The male frog appears to be mating internally with the female but he is actually depositing sperm onto her eggs as she lays them into the water. The fertilised eggs clump together until they hatch into tadpoles.

Most terrestrial animals use internal fertilisation. Reptiles and birds reproduce by internal fertilisation and then protect their developing offspring outside the body by laying shelled eggs. The eggs may have a hard outer shell (birds) or a tough membrane (reptiles). Mammals use internal fertilisation and most protect their developing offspring within the female's body until the fetus is fully developed. These are called the placental mammals because the fetus is nourished in utero through a placenta until it is born.

Many native Australian animals are marsupials, unique amongst maminals in that they do not hold the developing offspring within the female's body for the full period of fetal development. The young are born at a very early stage and continue their development, nourished and protected, inside an external pouch. Even more unusual and iconic are the only two representatives of monotremes, the platypus (endemic to Australia) and the echidna (native to Australia and New Guinea). The platypus and the echidna are classed as monotreme manm1als, they practise internal fertilisation, then Jay and protect eggs enclosed in tough flexible membranes.

External fertilisation

External fertilisation is when a male’s sperm fertilises a female’s egg outside of the female’s body. Most aquatic animals have external fertilisation, indicating that the advantages outweigh the disadvantages. Some examples of external fertilisation in animals are described below.

In amphibians and bony fish the female usually lays her unfertilised eggs in water and the male waits nearby to deposit sperm onto the eggs. The mass release of eggs for fertilisation is called spawning and it can be spectacular to see. A notable example is the long distance, mass migration of Atlantic and Pacific salmon species from the ocean, upriver to the fresh water gravel beds, where they spawn in the same location that they hatched. After the massive effort of annual spawning most adult salmon die but their species lives on.

On tropical reefs once a year, coral colonies have a synchronised mass spawning event across the whole reef system. The invertebrate coral polyps in one area release eggs and sperm simultaneously forming a great floating cloud in the ocean, resembling a shaken snow dome with white, red, yellow and orange colours. Triggers for a coral spawning event and the way it is coordinated are still being researched. Most corals are hermaphrodites as they produce both male and female gametes inside one body. This means they could self-fertilise, but cross-fertilisation has the evolutionary advantage of creating genetic variation. By expelling the eggs and sperm at the same time, the coral increases the likelihood that cross-fertilisation will take place. When an egg is fertilised by a sperm it develops into a coral larva called a planula that floats around in the water for several days to weeks before settling on the ocean floor. After the planula has settled in a particular area it starts to bud (asexual reproduction) and the new coral colony develops.

Internal fertilisation

Internal fertilisation is when the male transfers his gametes directly into the female’s body through a tube in his penis. This copulation process usually places his semen, containing the sperm, directly into the female's reproductive tract which greatly increases the chance of successful fertilisation with her egg. Internal fertilisation also overcomes the need for an environment with water because the reproductive tract is always moist. If a mature egg has been released from one of the female’s ovaries and the swimming sperm meet it, only one sperm will be able to penetrate the protective layer (zona pellucida) surrounding the egg and fuse with the egg nucleus. Each sperm has a rounded head and a long tail, which it uses to both swim and burrow into the egg.

Fertilisation occurs when the chromosomes in the sperm’s head pair up with those in the egg, forming the zygote. The egg then forms a barrier to other sperm. A diploid zygote cell is created with an equal mix of genetic information from male and female parents. After fertilisation, development of the zygote continues internally using mitotic division, either inside the female (mammals) or externally inside a shelled egg fed by the yolk (birds, reptiles and monotremes). Section 2.3 covers these reproductive features in more detail for mammals.

2.3 Pregnancy and birth in mammals

For all types of mammals, sexual reproduction produces genetically variable offspring, promoting the continuity of their species. The reproductive structures of female mammals are essential for creating a protective, watery environment for internal fertilisation. While all mammals reproduce using internal fertilisation, the physiology of pregnancy and birth varies widely. There are three types of mammals that are classified based on their reproductive strategies. These are placentals, marsupials and monotremes.

The differences between placental, marsupial and monotreme mammals are based on the extent of fetal development before birth and how the fetus is nourished during its growth period.

In this section you will learn about the stages of sexual reproduction and fetal development in mammals, particularly the placental mammals, of which humans are one. These stages include formation of gametes, fertilisation of gametes, formation and implantation of a zygote, the development of a zygote to embryo, the development of an embryo to fetus, and finally the birth of offspring. You will also gain an understanding of how these events are controlled by hormones.

MAMMALIAN REPRODUCTIVE SYSTEMS

In the placental mammals, a uterus provides nourishment and protection, via a placenta and umbilical cord, for the developing embryo and fetus until birth. After birth, placental babies are nourished with milk and develop a covering of fur. Some examples of placental mammals are humans, horses, dogs, mice, seals, elephants and whales.

For marsupial mammals, the under-developed joey is protected and nourished in the external pouch after an early birth, allowing another fertilisation to occur internally. Some examples of marsupials are kangaroo, brushtail, possum, wombat and koala. Both placentals and marsupials are viviparous, that is they give birth to developed, live young.

With the two monotreme mammals, the female Jays eggs and each puggle (baby monotreme) develops inside a leathery eggshell, then hatches to be protected and fed milk by the mother. There are only two monotremes: the platypus and echidna. Monotremes are oviparous, that is they lay eggs in which their young develop.

For all the mammals, before fertilisation can occur, haploid gametes must be produced by specialised reproductive organs in each parent. And for internal fertilisation, there must be a way for the male to introduce sperm into the female’s reproductive tract.

The male reproductive system in mammals

The male reproductive system consists of the following.

Paired testes (testicles; singular, testis), held inside the scrotum, which produce and store mature sperm continuously during mating periods; the main structures are the seminiferous tubules, where sperm cells are formed, and the epididymis that stores the sperm cells.

Accessory glands that produce secretions which make up about 95% of the volume of semen; these include prostate, seminal vesicles and Cowper’s glands.

A paired system of ducts, called vas deferens (also known as ductus deferens and sperm duct), leading from the testes to the urethra.

Luteinising hormone (LH) from the pituitary gland (in the brain) to stimulate the secretion of the male steroid hormone testosterone in the testes.

A penis, the male organ that grows to full size during puberty and has both sexual and excretory functions. The urethra tube passes through the penis, delivering urine or semen out of the body but not at the same time.

In a male the penis becomes erect when ready for copulation. Erection results from increased blood flow into columns of spongy tissue until the organ is rigid.

Mitotic divisions of precursor germ cells in the testes produce diploid spermatocytes, each of which divides by meiosis to produce four haploid sperm cells. During mating, contractions of the vas deferens move sperm towards the urethra. Secretions of the accessory glands are added, forming the seminal fluid, which has two main functions: it causes the sperm to become motile, and it provides an alkaline nutritious medium that is rich in protein, ions, vitamins and fructose sugar. Mammalian sperm each have a single flagellum that is used to propel them through the female reproductive tract towards the egg after copulation. They literally swim through the liquid internal environment in a race to be the first to reach and fertilise an egg.

1'he head of a sperm contains the nucleus with a haploid set of chromosomes, and a cap called the acrosome that contains enzymes used for penetrating the outer layers of the female egg. Mitochondria in the midpiece produce adenosine triphosphate (ATP) for energy during the journey through the female reproductive tract. The tail makes lashing movements that propel the sperm on this journey.

The female reproductive system in mammals

The human female reproductive system consists of the following.

A single uterus where, if an egg is fertilised it implants in the uterine wall, a placenta forms and the fetus develops until the time of birth. The uterus undergoes changes that are controlled by hormones.

Paired ovaries which hold the oocytes (immature egg cells) until puberty when monthly ovulation starts under hormonal control.

Paired fallopian tubes (also called oviducts or uterine tubes) connecting each ovary to the uterus.1'he open end of each tube has fringe-like structures called fimbriae (singular fimbria) that surround the ovary to catch the eggs when released. Fertilisation usually takes place high in an oviduct.

A cervix, a narrow muscular canal 2-3cm length lined with mucous, that connects the uterus and vagina. The cervix dilates (stretches open) to at least 10cm width for childbirth. During monthly menstruation it is controlled by the hormone oestrogen to become softer and more open.

A vagina, a muscle-lined canal from the cervix to the genitals, which receives the male penis during sexual intercourse. Monthly menstrual blood flow from the uterus exits the body through the vagina. The vagina is also the birth canal for the baby to enter the outside world. Unlike in males, the opening for the excretion of urine (the urethra) is separate to the female reproductive tract.

Most female placental mammals have a single cervix. Humans and chimpanzees also have a single uterus, limiting the number of young the female can bear at one time. Some mammals such as cats, horses, deer, dogs and whales have two uteri, sometimes with the lower uteri joined into one. Rodents, rabbits and hares have a pair of uteri and cervices joined to a single vagina, allowing for reproduction of large litters. Marsupials also have paired reproductive tracts. An early birth at 4-5 weeks of age removes a developing marsupial from its mother's body much sooner than in placental mammals, thus marsupials have not developed a complex placenta. In monotremes, the uterus only functions to form a leathery eggshell around the embryo.

Ovulation

Unlike human males who continually produce sperm after puberty, a female is born with all the immature egg cells already in her ovaries. After reaching maturity (puberty), ovarian cycles commence. Later in life, at the time called menopause, females cease to ovulate. Thus before a female is born, meiosis has begun in all oocytes (immature egg cells) but is arrested at an early stage. Once she reaches puberty, pituitary hormones control the continuation and completion of meiosis I. Meiosis II begins, but again is paused until actual fertilisation occurs. Meiosis II only completes after fertilisation.

Under the influence of follicle stimulating hormone (FSH), one or more of the oocytes will resume its meiotic division up to **metaphase** II and matures within a group of nutritive cells called a follicle. Only one egg forms from each oocyte during meiosis. When the oocyte is maturing it grows much larger by adding nutrients and extra cell organelles. These are stored in its cytoplasm for use after fertilisation when cleavage begins and rapid mitotic division forms many new smaller cells.

Follicles containing a maturing egg release the hormone oestrogen, which causes changes to the lining of the uterus (endometrium) and also acts on the anterior pituitary gland. The uterine lining becomes thicker, softer and spongy, and richly supplied with blood vessels in readiness to receive a fertilised egg.

Ovulation is the release of a mature egg and is triggered by a surge of luteinising hormone (LH) released from the anterior pituitary gland in the brain. The ovum (ripe egg) bursts out of the follicle and is drawn by fluid currents into the oviduct. Eggs, unlike sperm, cannot move by themselves. The fimbriae move to create a current that sweeps the egg into the start of the oviduct. Contractions of the oviduct and synchronised movement of cilia on its internal walls then help to propel the egg along towards the uterus. A human oviduct is about 10 cm long and 1 cm in diameter.

Left behind in the ovary, the burst follicle, now without its egg, is called the corpus luteum. The corpus luteum, stimulated by LH, secretes large amounts of both oestrogen and progesterone. These hormones cause a further thickening of the lining of the uterus during the latter part of the cycle. The actions on the endometrium are to prepare the uterus to receive an embryo, should fertilisation occur.

If it is not fertilised, the egg simply passes out of the reproductive tract. The corpus luteum slowly disintegrates and stops releasing its hormones. As a result, the thickened uterine lining breaks down and menstruation (monthly bleeding) occurs.

FERTILISATION

Fertilisation is the fusion of two haploid gametes (egg and sperm) to form a single diploid zygote cell. The zygote cell contains the genetic material of both the egg and the sperm. There are equal genetic contributions from the male and the female parents to the zygote and subsequent offspring. For humans, fertilisation is often called conception.

The fertilisation event

In mammals, and most terrestrial animals, fertilisation occurs internally following mating and most often takes place in the upper part of the oviduct. The male inserts his penis into the female’s vagina and a muscular contraction (ejaculation) pushes sermon from his urethra into her vagina. From the vagina the sperm swim, using movement of their flagella, through the cervix into the uterus and into an oviduct until one sperm reaches and penetrates the egg. The other factor is timing. Ovulation to release a mature egg must have occurred and fertilisation of this egg has to take place. Sperm can survive for up to five days within the female's reproductive tract but three days is more typical.

Fertilisation occurs in four steps that are similar for all types of mammals.

The sperm uses enzymes from the acrosome to dissolve and penetrate the protective layer (zona pellucida) surrounding the egg to reach the cell membrane. Molecules on the sperm surface bind to receptors (specialised proteins) on the egg’s cell membrane to ensure that a sperm of the same species fertilises the egg, then the nucleus of the sperm enters the cytoplasm in the egg cell.

3 Changes at the surface of the egg occur to prevent the entry of multiple sperm nuclei into the egg.

4 Fusion of the haploid egg and sperm nuclei results in a diploid zygote cell (the fertilised eggs).

IMPLANTATION

After fertilisation, the zygote continues to travel down the oviduct until it reaches the uterus. Already the process of embryonic development has begun with a stage called **cleavage**. The development process continues as it passes down the oviduct. When the embryo reaches the uterus, ready for implantation, it is known as a blastocyst and looks like a ball of cells.

Cleavage

The first stage of development of the new zygote is cleavage, which commences following activation of the egg by sperm penetration. Cleavage is a period of rapid cell proliferation during which the single-celled zygote is divided into many hundreds of smaller cells by mitosis. You will learn more about mitosis in Chapter 3.

Morula

The early embryo continues to divide until, three to four days later, it consists of 16 cells and then enters the uterus. At this stage, the embryo resembles a mulberry and is known as a morula. The morula is a ball of unspecialised embryonic stem cells.

Blastocyst

In the uterus, mitotic divisions continue, and the morula becomes a blastocyst as its cells begin to differentiate. By day eight to nine, the blastocyst is ready to attach to the wall of the uterus. The multicellular blastocyst consists of a single layer of surface cells and an inner cell mass that will later give rise to the embryo. The outer layer of cells sends out finger-like projections into a part of the wall of the uterus (endometrium) and this area develops into the placenta (Figure 2.3.10).

Gastrula

After the blastocyst is implanted, gastrulation occurs over approximately five days, and the blastocyst becomes a gastrula, which has three different layers of cells. Eventually the gastrula becomes an embryo then a fetus when it has formed all the basic adult features.

The placenta and umbilical cord

The blastocyst adheres to the lining of the uterus (endometrium) and becomes implanted there. The outer layer of cells in the blastocyst initiates the formation of a placenta. Later an umbilical cord develops by the fifth week of the **embryo stage** from the remnants of the egg's yolk sac. It replaces the yolk sac as the source of nutrients for the embryo, acting as a conduit for embryonic blood vessels to reach the placenta. The umbilical cord remains attached to the fetus until after birth.

The placenta is an exchange organ bringing blood vessels of the fetus into close contact with maternal blood. There is no direct exchange of blood, rather nutrients and oxygen from the mother diffuse across into the blood of the umbilical vein and move to the fetus. The reverse happens for removal of waste products and circulation of depleted blood through the umbilical arteries from the fetus back to the placenta. After birth, the umbilical cord is cut (for humans) or in other animals the mother often severs it by biting. A person's navel (belly button) is the scar where their umbilical cord dried up and dropped off after birth.

The placenta is also an important source of hormones to maintain the pregnancy.

DEVELOPMENT OF THE EMBRYO

During the embryonic period of development, the major organs of the body are formed from the three primary layers of the gastrula. In humans this is completed about eight weeks after fertilisation (or 10 weeks after the last menstrual period). At the end of the **embryonic stage**, the developing organism has distinct features and is known as a fetus for the remainder of its development

Embryonic germ layers and cell specialisation

After implantation in the uterus, the blastocyst undergoes gastrulation, folding in on itself to form a gastrula with three primary layers of cells: ectoderm, mesoderm and endoderm. These primary layers are known as germ layers and are also supported by two membranes.

The yolk sac which surrounds the egg yolk. It has a well-developed vascular system that transports nutrients from the egg yolk to the developing embryo.

The amniotic cavity, which surrounds the developing embryo, is filled with fluid. Its main role is as a shock absorber to protect the embryo against any impacts or movements.

The three embryonic germ layers that form will eventually give rise to the different types of specialised cells that make up the tissues and organs in humans:

ectoderm (outermost layer of the embryo) forms epidermis, hair, peripheral nervous system, brain and spinal cord cells

mesoderm (middle layer of the embryo) forms muscle, cartilage, kidney and gonad cells

endoderm (innermost layer of the embryo) forms the lungs, bladder and lining of the digestive system, including the stomach, colon, liver and pancreas.

DEVELOPMENT OF THE FETUS

As already mentioned, at the end of the embryonic stage the developing organism is known as a fetus for the remainder of its development, until birth at around

38 weeks after fertilisation. However, since it is almost impossible to determine exactly when fertilisation or implantation occurred, the period of development for a human baby is calculated from the first day of the mother's last menstruation.

The time between the last menstrual period (LMP) and ovulation is approximately two weeks, so full term human pregnancy is usually 40 weeks (280 days but often estimated as nine months). The fetus grows in size and organs continue to develop for the rest of the pregnancy (this process is also known as prenatal development).

Cells and tissues become specialised to carry out their particular functions. The fetus is protected in the amniotic cavity, which provides a fluid-filled environment in which it can move about.

Gestation periods in mammals

In mammals, pregnancy is the period of reproduction during which a female carries one or more live offspring from implantation in the uterus through to birth. Pregnancy is also known as gestation. It begins when a zygote implants in the female's uterus and ends when the fetus leaves the uterus. Smaller species of mammals normally have a shorter gestation period than larger mammals. For example, gestation for the house mouse is 20 days, for the domestic cat it is 58-65 days, and 21 months for an elephant. Figure 2.3.16 shows a range of mammals and their gestation periods.

Human pregnancy can be divided roughly into three trimesters, each approximately three months long. The first trimester is from the last menstruation to week 13 of pregnancy, the second trimester is from week 13 to week 27, and the third trimester is from the week 28 to the week 42 (38 to 40 weeks growth plus two weeks from the start of the last menstruation). In humans, birth normally occurs at a gestational age of about 40 weeks (280 days) but births can occur between 37 and 42 weeks and still be considered a full term pregnancy.

The development of the fetus is monitored using ultrasound technology that is safe for both the fetus and mother. It uses very high frequency sound waves reflected back from structures inside a body to produce an image. The images produced in fetal ultrasound can be good enough to see the heart with all four chambers and valves, at 20 weeks when the organ is only 15 mm long, less than the size of a five cent coin.

HORMONAL CONTROL OF PREGNANCY AND BIRTH

Hormones are signalling molecules that are responsible for the communication between organs and tissues to regulate physiological and behavioural processes. In animals, hormones are synthesised by specialised cells (either in the endocrine glands or other tissues) and are transported to where they are needed via the circulatory and lymphatic systems or by diffusion through the extracellular fluid.

Hormones transmit signals to their target cells by altering specific biochemical reactions in these cells. The target cells have a matching surface receptor for a particular hormone. Hormones exert their effects either directly by passing through the cell membrane into the cell, or indirectly by interacting with a receptor on the surface of the cell. You will learn more about hormones in Chapter 14, but their role in pregnancy and birth is examined here.

The pituitary gland secretes hormones involved in the regulation of lactation and reproduction even though it is located in the brain. This gland lies immediately below, and is connected to, the hypothalamus, a region of the brain that acts as a master control centre. The hypothalamus receives information from all parts of the body and produces releasing hormones, which control the release of oilier hormones from the pituitary gland.

Labour and birth in humans

A correct balance of hormones is essential to maintain the pregnancy, starting with hCG (human chorionic gonadotropin) from the placenta when the embryo has implanted. The hCG stimulates increased blood flow to the pelvic area and helps regulate the ovarian hormones.

Progesterone is required at high levels throughout pregnancy with levels steadily rising until the birth of the baby. Initially the progesterone comes from the corpus luteum then after six weeks the placenta produces it. Progesterone stimulates early preparation of the uterus for pregnancy and later it prevents lactation and uterine contraction until it is time for the birth.

Oestrogen levels also rise throughout the pregnancy to work in partnership with progesterone. Some organ development in the fetus, including liver, kidneys and lungs, requires oestrogen. This hormone promotes growth of breast tissue in preparation for maternal milk production.

There are several other human pregnancy hormones, not all of which have functions that are well understood. Some of the hormones cause side effects and emotional changes, including nausea (commonly known as morning sickness) and mood swings. These effects are mostly in the first trimester.

In the period just before a human birth the balance of two hormones, oestrogen and progesterone, changes. The natural level of prostaglandins increases which in turn increases the sensitivity of the cervix and uterus to oxytocin. Oxytocin is the hormone that causes uterine contractions. The hormonal changes create irregular uterine tightening or contraction. The fetus has usually moved with its head low in the pelvis, putting pressure on the cervix. This pressure stimulates further release of oxytocin and so labour begins. When the cervix reaches full dilation (10cm or more in width), oxytocin and adrenaline hormones work together to start the final series of muscular contractions. After the baby is delivered the uterine contractions are maintained by oxytocin until the placenta is pushed out and the uterus starts shrinking back to normal size. Oxytocin also promotes tee protective mothering instinct and works with prolactin to stimulate lactation for feeding the newborn baby.

Although the section above has specifically described hormonal controls for a human pregnancy, the hormones and features of pregnancy and birth are much the same in other mammals.

2.4 Manipulation of plant and animal reproduction in agriculture

Humans have used selective breeding to produce animals and plants with more useful or more attractive characteristics for tens of thousands of years. This was done without using a high level of scientific or technological knowledge. They chose those animals or plants that expressed the characteristics they wanted to conserve and selectively bred them together, hoping that their offspring would show even more of these characteristics. In the past, selective breeding could only use characteristics that already existed in the genetic pool of a species. We now have the knowledge and skills to use cloning, recombinant DNA techniques, gene editing and transgenic technologies to transfer genes from one species to another and produce organisms with DNA combinations never seen before. This offers many benefits, but we also need to evaluate if the impacts of reproductive manipulation are environmentally, scientifically, socially and ethically acceptable.

In this section, you will learn about some examples of reproductive manipulation in agriculture and start to consider the advantages and disadvantages of such manipulations. Agricultural ecosystems are, by their very nature, low diversity systems dominated by only a few species that are protected by humans. You will gain an understanding of the importance of conserving species and genetic variation for the future.

SELECTIVE BREEDING

Evolution by natural selection is an ongoing and, as the name implies, natural process. In addition, humans have been manipulating the gene pools of populations for thousands of years through deliberate selection of particular individuals for breeding. The process by which humans decide which individuals may breed and leave offspring to the next generation is called selective breeding or artificial selection.

All modern crops and livestock were developed by genetic manipulation of plant and animal species through the process of selective breeding. However, new molecular technologies are being used to alter the characteristics of organisms in a more targeted and specific way, and more quickly than by traditional breeding. These new methods, called genetic engineering, can also allow the exchange of genes bermeen organisms that are sexually incompatible and normally cannot interbreed. New forms of plants and animals developed in this way are referred to as genetically modified organisms (GMOs).

There are four basic steps that apply to all forms of selective breeding, whether it be with a plant or an animal.

1 Determine the desired trait. 2 Interbreed parents who show the desired trait. 3 Select the offspring with the best form of the trait and breed these offspring. 4 Continue this process until the population reliably reproduces the desired trait.

Gene linkage

A common problem with selective breeding is gene linkage, meaning that it is not only the desired trait that is selected for. Other traits may be inadvertently selected because genes are carried on chromosomes with many other genes. Those genes that are located close together tend to travel linked together through cell division and into the offspring. For example, wheat that has been selected for the trait of high grain production may also carry a gene for tall stems that are not strong enough to support the ripe heads for harvesting. The success of selective breeding of both plants and animals may be limited by the presence of undesirable linked genes.

Selective breeding in plants

A1ost selective breeding of plants is done to produce higher-quality food. Typically, seeds are collected from the individuals with the largest, most attractive or most numerous grains, fruits, nuts or other part of the plant that will be eaten. Those seeds are planted and the new generation of plants is cross-pollinated in a controlled way with other individuals having similar traits. The resulting plants produce larger, more nutritious or more aesthetically pleasing food products. Many food crops, such as cereals, tomatoes, potatoes and bread wheats have been modified by selective breeding to have higher yields, greater resistance to common diseases, to be more palatable or for improved nutritional value.

Once a desirable plant has been bred, artificial pollination or cloning methods, such as cuttings, grafts or tissue culture, may be used to mass produce identical plants by asexual means.

Examples of selective breeding in plants

Maize or corn, (Zea mays)-maize is one of the most widely grown crops in the world. It is thought that maize was selectively bred from the wild grass, teosinte. Modern maize has significantly larger cobs with many more rows of much larger kernels compared to the ancestral teosinte. The higher-yielding modern maize provides more food for people than the ancestral form.

Wheat (Triticum aestivum)--during 10 000 years of cultivation, numerous forms of wheat, many of them hybrids, have developed under a combination of artificial and natural selection. Modern wheat has become polyploid with strains that are tetraploid (4n, two sets of chromosomes) and Hexaploid (6n, three sets). It has been selected for traits like high yield, high gluten content and heads that do not shed their seeds easily. me 4n variety called durum wheat is used for making semolina flour for pasta. The 6n, called bread wheat, has several variants that are used for pastry, cakes and bread making. William Farrer (1845-1906) pioneered Australian wheat research when he systematically used cross-breeding (hybridisation) on his own property to improve bread wheats. Farrer was known to have cross-pollinated the plants using his wife's hairpins to transfer the fine grains of pollen until he acquired a pair of forceps.

Orange-fleshed sweet potatoes (OFSP)—the 2016 World Food Prize was awarded to a group of scientists who worked on breeding and introducing biofortified, orange-fleshed sweet potatoes to Africa. Their project recognised the importance of provitamin A, also known as beta-carotene, which is converted in the body to vitamin A. Deficiency of vitamin A is considered to be one of the most harmful forms of malnutrition in the developing world. It can cause blindness, limit growth, and weaken the body's immune system, especially in young children. An orange colour in vegetables indicates the presence of provitamin A. While orange-fleshed sweet potatoes are naturally occurring in South America, they were not grown in African countries where white-fleshed varieties are common and preferred. The biofortified varieties used for the OFSP project were specifically bred by plant breeders to be rich in provitamin A. A successful grassroots-education program has Jed to the introduction and acceptance of the orange variety in some African countries.

Polyploidy

During the process of selective breeding of plants, it is quite common for polyploidy to emerge. This is the condition where the cell nucleus has more than two sets of chromosomes (e.g. 3n, 4n or 6n rather than 2n). Polyploidy can come about naturally through errors in meiosis where gametes may end up being diploid rather than haploid. In this case a fertilised egg becomes 3n or 4n. In plants, a polyploid zygote often continues to develop into an adult plant that is sterile. Unlike vertebrate animals, many polyploid plant species can survive using asexual reproduction and continue to breed the polyploid variety into the future. Some banana varieties are triploid (3n); cultivated cotton and potatoes are examples of tetraploid (4n) organisms; bread wheats are hexaploid (6n); and strawberries are octoploid (8n). In humans, polyploid zygotes do not survive. The condition is rare in animals but it is known to occur in some insects, earthworms and tree frogs.

Humans have selected for polyploidy in some crop plants because it can result in larger and more vigorous plants. It was discovered that polyploidy can be induced with a chemical called colchicine. When exposed to colchicine, the paired chromosomes are prevented from pulling apart during cell division, resulting in 2n gametes from meiosis or 4n cells from mitosis. As well as the examples listed above, polyploids are now found in a large number of agricultural crops such as turnips, spinach, apples, radishes, grapes and watermelons.

A drawback of inducing polyploidy in plants is that the seed crop produced by many polyploids is sterile or has lower fertility rates than their diploid types. In some situations, polyploid crops are preferred because they are sterile. For example, many seedless fruit varieties are seedless as a result of polyploidy, such as bananas, strawberries and seedless watermelon. To preserve the characteristics of the variety, such crops are propagated using asexual techniques, such as grafting, suckers, runners or tissue culture.

Hybridisation

The crossing of different varieties within one species to produce new varieties with different combinations of characteristics is one kind of hybridisation. In general, hybrid plants are more vigorous, higher yielding and may be more disease-resistant but the outcome of hybridisation is not always an improvement. It is also important to note that when hybrid offspring are produced artificially, they are designed to be cultivated or reared under controlled conditions of intensive agriculture, horticulture or farming and may not be suited to conditions in the wild.

When a hybrid has been deliberately bred, the induction of polyploidy is a common technique to overcome the sterility of a hybrid species. For example, triticale is the hybrid of a wheat and rye. It combines sought-after characteristics of both parent plants, but the initial hybrids are sterile. After polyploidisation using the chemical colchicine, the triticale hybrid becomes fertile and can continue to sexually reproduce itself, usually by self-fertilisation. At present, several types of triticale are grown for stock feed, either grain or forage while research continues to try and improve it for human consumption.

Heirloom plants

An heirloom plant, also known as a heritage variety, is a traditional cultivated plant that is maintained by small-scale gardeners and farmers. These may have been commonly grown during earlier periods in human history but are not used in modern large-scale agriculture. In modern agriculture most food crops are now growing using limited varieties in large, monoculture plots to keep the product of a consistent standard. These varieties have often been selectively bred for high productivity, ability to withstand mechanical picking and storage, and tolerance to drought, frost or pesticides. Fruit varieties such as apples have been propagated over the centuries through grafts and cuttings to maintain consistent traits such as size, colour and flavour. Many crop plants that are grown annually are from hybrid, or even genetically modified, seed purchased from a commercial supplier. The crop will be sterile as far as further reproduction goes and may have an intellectual property-right on it to prevent replanting. For example, in the USA (where it is legal), a grower of the patented GMO, Roundup Ready® soybean is prohibited by contract from saving and replanting the seed. This variety has been genetically modified to be resistant to herbicide spray used to kill weeds in the crop.

Heirloom gardening is a reaction against the limited varieties used in conventional agriculture and aims to preserve both species and genetic variation. The crops produced vary in output and quality, so are not acceptable to all consumers or economically viable for producers, especially in developed countries. However, maintaining species and genetic variation with heirloom varieties is a precaution against monopoly by a restricted number of companies. It also provides food security for the future in the face of climate change, new pests and diseases, salinity and other issues that may make current monoculture varieties no longer viable.

Selective breeding in animals

Just as crops have been selectively bred for desired traits, so too have many animal species. In agriculture, sheep have been selected for the quality and quantity of the wool they grow dairy cows have been selected for the milk they produce, beef cattle for their muscle mass and poultry for body weight and egg-laying. Aquaculture may involve selective breeding and is Australia’s fastest growing primary industry producing fish, prawns, and shellfish including oysters and pearls. There are niche industries for sale of pedigree pets that involve intensive breeding programs.

When a selected species has a variety of traits, different traits may be useful in different situations. A single wild species can be the original source of a great variety of different breeds with an obvious example being domestic dogs bred from a single wolf species.

GENETIC MODIFICATION AND CLONING OF PLANTS AND ANIMALS

Over the last few decades, scientists have developed techniques that allow for the alteration of an organism's genome and for the transfer of genes from one organism to another. Genetic technologies are used in plant agriculture to increase crop productivity, provide crop resistance to insects and prevent disease. Genetic modification (GM) of animals has also been used to improve fertility, and the quality and yield of meat, milk, eggs and wool.

Cloning plants to retain desirable traits has been used for centuries in horticulture and agriculture. However, the direct manipulation and cloning of cells and embryos is a more recent development in agriculture. Cloning is the production of new individuals that contain the same genetic information as the parent organism. Natural clones are produced by asexual reproduction when a single parent cell divides to produce two new identical daughter cells. You learnt about asexual reproduction in Section 2.1. The term cloning is also used to refer to artificial methods of producing genetically identical organisms. The cloning techniques used in agriculture are artificial methods carried out by humans and include cuttings and grafts, tissue culture, embryo splitting (or artificial embryo twinning) and somatic cell nuclear transfer (SCNT).

IMPACT OF MANIPU LATING PLANT AND ANIMAL REPRODUCTION IN AGRICULTURE

By looking at the examples outlined in this section, it is clear that selective breeding and genetic modification has already brought considerable benefits to humankind. The quality and quantity of our food has been improved, sometimes costs have decreased, and plants and animals have become more suitable for human purposes. The improvements have not come without costs and controversy, especially in relation to animal welfare and long-term food security. Given the rapid development of genetic technology and its many potential applications in agriculture, the impacts of this technology must be evaluated. Some of the potential problems that have been raised concerning the manipulation of plants and animals in agriculture are outlined in Table 2.4.1.

Chapter 3 Cell replication

3.1 Mitosis and meiosis

**Cell theory** states that all cells arise from pre-existing cells. In order for this to occur, cells must be able to replicate. This process is essential to the life of all organisms. Once you were a single cell-a fertilised egg known as a zygote. Now, your body is made up of about 37 trillion cells with many different specialisations. In order to start producing the millions of cells that make you, that first single-celled zygote had to replicate itself. Since then, as the cells in your body wear out and die or are damaged, more cells are replicated to replace them throughout your lifetime.

In this section, you will learn about the process of identical cell replication called mitosis and the cell division process to form gametes called meiosis. You will compare the two processes and come to appreciate the complexity and importance of both in living things.

WHY DO CELLS REPLICATE?

Cell replication is the form of cell division in which a parent cell divides to produce two daughter cells. In eukaryotes (protists, fungi, plants and animals), cells replicate by mitosis. For a multicellular organism, cells replicate for the following reasons.

Restoring the nucleus-to-cytoplasm ratio-egg cells have an unusually large volume of cytoplasm, too much for the nucleus to control. After fertilisation of an egg, the early rounds of mitosis proceed without pausing for cell growth, a process called cleavage that decreases the volume of cytoplasm per cell.

Growth and development-multicellular organisms grow in size by increasing the number of their cells through repeated cell replications. The new cells then grow in size, increasing the size of the organism. As the new individual continues to develop, new cells become specialised for different purposes, such as muscle cells in animals and phloem cells in plants. More replications follow and the specialised cells become organised into tissues, which form the body of the organism.

Maintenance and repair-cells become damaged or die as a result of normal functioning, and also as a result of injury, such as sunburn in the case of human skin cells. Maintaining and repairing tissues requires the production of identical new cells to replace those that die. The new cells are produced by the cell replication process of mitosis.

Unicellular organisms do not need to replicate for these purposes because they remain a single cell throughout their entire life cycle. Instead, cell replication in unicellular organisms (whether prokaryotes or eukaryotes) is a simple form of reproduction and creates new, genetically identical individuals.

THE CELL CYCLE

The eukaryotic cell cycle is the life cycle of a cell, involving growth, replication of DNA (deoxyribonucleic acid) and division to produce two identical daughter cells. The cell cycle has three main phases:

interphase mitosis cytokinesis.

These phases always occur in this order, with a cell spending most of its time in **interphase**. During **interphase** the cell doubles its mass and duplicates its entire components. During mitosis the nucleus divides, and during cytokinesis the cytoplasm divides. The cell cycle is the period between one cytokinesis and the next. The time for a cell cycle is called generation time (T) and varies considerably between different cell types.

Interphase

A cell spends most of its time in **interphase**, carrying out cellular functions and preparing for cell division. There are three stages in **interphase**:

G1 (Gap I)-during G1 the cell produces more organelles and the cytoplasm increases in volume, doubling the size of the cell. If a cell is not going to divide again it will enter the G0 phase. Human red blood cells, nerve cells and surface skin cells enter this phase from early in G1, meaning that they cannot replicate again. Other cells enter the G0 phase temporarily as a resting phase when they carry out cell functions but do not grow or replicate. These cells re-enter G1 and continue with the cell cycle. That some cells spend temporary periods in G0 explains the wide variations in interphase length. For example, adult human liver cells have generation times of 300-500 days, most of which will be spent in G0. S (Synthesis)-DNA replication occurs during the S phase of interphase. Prior to the division of the cell’s nuclei (mitosis), the DNA content must be replicated so that each new daughter cell receives a full set of the DNA-carrying chromosomes. It is essential that the genetic information is passed on accurately, because the activities of cells are ultimately controlled by the genetic information in the nucleus (in eukaryotes) or nucleoid (in prokaryotes). You will learn more about DNA replication in Section 3.2.

G2 (Gap 2)-during G2 the cell undergoes a secondary stage of growth, metabolism and energy acquisition. It prepares for mitosis by synthesising the materials, such as proteins, needed for division.

Mitosis

The term mitosis refers to the division of the nucleus into two genetically identical daughter nuclei. Mitosis is a continuous process but has four sub-phases: prophase metaphase anaphase telophase.

Each sub-phase can be distinguished by the appearance and the position of the chromosomes in the cell. During **interphase** S in the cell cycle, each chromosome is duplicated forming a pair of sister chromatids. However, they are not visible under a microscope because they have not condensed. The structure and function of chromosomes and DNA will be explained in more detail in Section 3.2. For now it is sufficient to understand that DNA molecules carry coded genetic information and they are coiled into chromosome structures that reside in the nucleus of a cell.

Prophase

Early in **prophase**, chromosomes begin to condense (shorten and thicken) and become increasingly visible under the microscope. As they condense further, each chromosome can be seen as two chromatids held together at the centromere. At the same time the centrioles, which were replicated during **interphase**, move to opposite ends of the cell to form the poles.

Later in **prophase** the nuclear membrane breaks d0\'1'11. The centrioles begin to form a network of fibres, called the spindle, which extends between the t\\'O poles of the cell. The centromere of each individual chromosome attaches to spindle fibres extending from each of the poles (Figure 3. l.11). Plant cells do not usually have centrioles; they use a different mechanism to produce the mitotic spindle.

Meta phase

During **metaphase** the centromeres continue to be drawn by the spindle fibres so that the chromosomes are aligned along the equator in the middle of the cell. Chromosomes are most easily observed at this stage because they are highly condensed.

Anaphase

In **anaphase** the spindle fibres contract, pulling the centromere in two directions. The centromere splits, separating the two chromatids. Contraction of the spindle fibres continues and the separated chromatids are pulled to opposite poles. Thus, daughter cells receive the same genetic information---one copy of every chromosome that was in the original nucleus at **interphase**.

Telophase

The final stage of mitosis is called **telophase**. It is rather like **prophase** in reverse. A nuclear membrane reforms around the chromosomes at each pole. The spindle is dismantled and disappears. The chromosomes become longer and thinner, and therefore less visible under the microscope.

Cytokinesis

At the end of mitosis the cytoplasm divides, separating the two nuclei and other organelles into two complete and identical daughter cells. The division of the cytoplasm is called cytokinesis and it finalises the cell division stage. Cytokinesis in animal cells occurs in a different way to cytokinesis in plant and fungi cells. In animal cells the cell membrane moves inwards, pinching the two daughter cells apart.

Plant and fungi cells lay down a new cell membrane and cell wall between the two daughter nuclei to separate the daughter cells. Components of the new cell wall, called the cell plate, are initially deposited in the centre of the cell. The growth of the cell plate extends outwards until the two daughter cells are completely separated.

MEIOSIS

The other form of cell division in eukaryotic cells is meiosis. Meiosis is not identical cell replication because the nature of the process produces daughter cells that are different from each other and also from the parent cell. Meiosis is an important cell division process that is required for sexual reproduction and creating genetic variation; it produces four daughter cells (gametes) that are genetically unique. Meiosis occurs only in eukaryotes and only to form the gametes. The formation of gametes from germ cells occurs by meiosis in the specialised reproductive organs of sexually reproducing animals and plants.

Meiosis is called a reduction division because, unlike mitosis, it reduces the number of chromosomes in gametes (daughter cells) to half (m or n) of that in somatic cells (2n). Cells with n chromosomes are called haploid cells and cells with 2n chromosomes are diploid cells. Gametes receive only one copy of each pair of homologous chromosomes (n = 23 chromosomes in human gametes). Compare this to mitosis, where each daughter cell receives a copy of every chromosome and they are genetically identical with 2n chromosomes.

Like mitosis, meiosis is a form of cell division that involves prophase, metaphase, anaphase, telophase and cytokinesis. Unlike mitosis, there are two sequential rounds of division in meiosis, called meiosis I and meiosis II each with these sub-phases.

The first division of meiosis: meiosis I

The sub-phases of meiosis I occur in the following order: prophase I, metaphase I, anaphase I, telophase I.

During meiosis I, homologous chromosomes are separated, reducing the chromosome number by half (reduction division) and producing two haploid daughter cells. The sister chromatids remain joined together at the centromere so each chromosome is still double-stranded.

Each chromosome pairs up precisely along its length with its matching (homologous) chromosome. This pairing is called synapsis. Because each chromosome has already replicated, each chromosome consists of two copies, called sister chromatids. So a pair of homologous chromosomes has a total of four chromatids.

Crossing over may occur between homologous chromosomes.

Crossing over and recombination

A key event now occurs-chromatids of homologous chromosomes may exchange portions of their genetic information in a process called crossing over. Crossing over is a natural genetic process that occurs between homologous chromosomes and leads to the switching of genetic material between the chromosomes. DNA strands from the chromatids of two homologous chromosomes are cut at the equivalent point, a segment is exchanged, and the strands are reconnected.

The point where crossing over occurs is called a chiasma (plural chiasmata). It consists of a temporary molecular scaffold that disappears later. A long chromosome may have several chiasmata.

The significance of crossing over is that it produces chromosomes with new combinations of genetic information. This process is called recombination and is essential to the production of genetic variation.

When crossing over is finished, the homologous chromosome pairs align along the midline of the cell; they do this randomly, meaning the maternal and paternal chromosomes do not line up on the same side of the midline.

The homologous chromosomes then separate and move to opposite poles. These two steps result in the random assortment of maternal and paternal chromosomes and their alleles (gene variants) in the gametes. The centromeres do not split. It is the chromosomes of a pair that separate, not the chromatids.

The spindle breaks down and the nuclear membrane reforms.

At the end of this first division of meiosis, there are two daughter cells with the chromosome number halved-they contain only one set (n) of chromosomes (i.e. they are haploid). Each chromosome is still made up of two chromatids.

The second division of meiosis: meiosis II

The sub-phases of meiosis II occur in the following order: prophase II, metaphase II, anaphase II, telophase II. The second division of meiosis does not involve chromosome duplication. Meiosis II is similar to mitosis in that sister chromatids are separated by splitting the centromere. Each of the two haploid cells from meiosis I divide into two, producing four haploid daughter cells. This occurs in the following sequence:

Chromosomes align on the spindle equator, the centromeres split and the chromatids separate.

A chromatid from each chromosome moves to each pole.

The final nuclei from the two divisions are each haploid (n).

The cytoplasm divides by cytokinesis, and four daughter cells (non-identical) are formed from one original parent cell.

In a male, meiosis II results in four viable, haploid sperm. However, in a female only one haploid ovum results and the other three haploid cells degenerate. This occurs because of the uneven distribution of cytoplasm in cytokinesis. One daughter cell is very large, containing most of the cytoplasm and organelles, ready for the cleavage process if fertilised. The other three are called polar bodies and they usually enter apoptosis (programmed cell death). The polar bodies of human oocytes apoptose by 24 hours after formation and the resulting fragments remain within the zona pellucida of the large oocyte. That explains why the three polar bodies disappear from diagrams of meiosis II.

COMPARISON OF MITOSIS AND MEIOSIS

The common feature between mitosis and meiosis is that both are processes of cell division to form new, additional cells. However, they have very different purposes, there are some different steps within the processes and different outcomes, which are outlined in Table 3.1.3. The distinction between somatic cells and germ cells is important to understanding mitosis and meiosis. Somatic cells are all the body cells except for the germ cells that produce gametes (sperm and egg in animals; pollen and egg in plants). Mitosis produces new, somatic cells (e.g. skin cells) and meiosis produces sex cells (gametes, e.g. sperm and egg).

3.2 DNA structure and replication

Throughout history, people have observed that children resemble their parents more than they resemble unrelated individuals. Today we know that many characteristics are inherited, such as the colour of our hair, eyes and skin. However, children are not identical to their mother or father, and they are not identical to their sisters or brothers (except in the case of identical twins).

We also know that the hereditary information is carried in coded form (genes) on complex molecules called deoxynbonucleic acid (DNA). During the formation of gametes by meiosis, the DNA is contained in structures called chromosomes in the nuclei of cells. Chromosomes are passed from parent to child after a sperm cell fertilises an egg cell. The DNA in the resultant zygote is a unique and equal mix of chromosomes from the mother and father. After fertilisation, the cell cycle at the S stage of DNA replication and the mitosis stage of cell division is responsible for accurately replicating cells including copying the inherited genetic code.

In this section you will learn about the structure of DNA and how this structure supports its function of carrying genetic information from one generation to the next. You will also come to understand the packaging of lengthy DNA molecules into compact chromosomes in a cell nucleus and how exact copies of the DNA are replicated prior to each cell division.

DNA STRUCTURE

DNA is one of the two types of nucleic acids: DNA (deoxyribonucleic acid) and RNA (ribonucleic acid). Both DNA and RNA are made up of a repeating series of nucleotide units formed from nitrogenous bases and a sugar-phosphate backbone. RNA is single-stranded and DNA has a double-stranded helix (spiral) structure with complementary base pairing across the helix for its nitrogenous bases (Figure 3.2.3). DNA carries the genes (genetic information) needed to assemble functional protein molecules from amino acid sub-units. Identical copies of the original parental DNA are passed from a parent cell to each daughter cell during every mitotic cell division.

RNA works with DNA to play a role in the synthesis of the proteins within cells. Proteins are many and varied with a wide range of important structural and biochemical functions in all organisms. For example, haemoglobin protein carries oxygen, enzymes are proteins that control all the chemical reactions of an organism, antibodies and most hormones are protein molecules. Sections 4.2 and 4.3 cover more information about proteins and how they are made inside cells to the DNA specifications.

The DNA double helix has become a familiar and iconic symbol since James Watson and Francis Crick announced the discovery of its structure in 1953. Like much of scientific discovery, the final understanding of DNA structure may seem like a eureka moment, but it was built on many years of painstaking earlier investigations and technological developments by other scientists. Some of the important discoveries that led to our understandi.J1g of DNA structure are listed below.

1866-Mendel and others had shown that information is inherited from parents to offspring. At first it was thought to be carried by protein molecules because of their complexity.

1869-lvtiescher isolated an unknown chemical from the nuclei of white blood cells and called it nuclei. The name was later changed to nucleic acid, then to deoxyribonucleic acid (DNA).

Early 1900s-Sutton using grasshoppers, Bovery with sea urchms and Morgan with fruit fly all proved that chromosomes carry the inherited information bermeen generations.

1905-39-Levene identified sugar, phosphate and base components of DNA, gave nucleotides their name and distinguished RNA from DNA.

1943-Avery showed that DNA carries the genetic code, after much dispute if it was carried by protein (with its complex structure) or nucleic acid (with a simpler structure).

1940s-Chargaff expanded on Levene's research and made three important discoveries that laid the foundation for fully understanding DNA structure: different species have the same nucleotides but arranged in different orders the amounts of nitrogenous bases A and T are always similar; amounts G and C are singular, but A-T and G-C may be present in different amounts—now, known as Chargaff's rule

-A + G always equals T + C.

Early 1950s-Rosalmd Franklin and Maurice Wilkins used X-ray crystallography to produce photos of DNA structure, after working out how to get DNA into crystal form. This technology began with the Bragg father and son team m 1913-14. Franklin was skilful at improving it. Sadly, working with X-rays may have contributed to her early death in 1958 when she was 38 years old from cancer, making her ineligible for the 1965 Nobel Prize shared by Watson, Crick and Wilkins.

1953-James Watson and Francis Crick, using Chargaffs rule plus Franklin and Wilkins' information, made adjustments to their 3D modelling of DNA and announced the successful discovery of DNA structure amidst great excitement at their local pub. Positioning the bases inside the helix was the key to finally determining the structure.

Genetics research has advanced rapidly since the 1950s discoveries, including understanding the universal genetic code in all species-in essence, there is a set of 64 codons (sets of three bases) corresponding to the 20 amino acids used for protein synthesis and as the signals for starting and stopping protein synthesis. Section 4.2 will explain the universal code of DNA and its all-important role in protein synthesis.

Nucleotides building blocks of DNA and RNA

Nucleotides are the chemical building blocks of DNA and RNA. Each nucleotide consists of:

a phosphate group

a five-carbon sugar (deoxyribose). The five carbon atoms are numbered 1'-5'. In an individual nucleotide, a phosphate is attached to the 5' carbon, and a base is attached to the 1' carbon.

one of four nitrogen-containing bases: adenine (A), guanine (G), thymine (1') and cytosine (C) (Figure 3.2.6c). There are now types: purines, with a double ring molecular structure, and pyrimidines, with a single ring. The purine bases are adenine and guanine, and the pyrimidine bases are thymine and cytosine (and uracil (U) in RNA where it replaces T).

DNA

DNA is a large (macro) molecule, which is made up of a series of nucleotides.

Figure 3.2.7 shows a single polynucleotide chain (strand) of DNA in which individual nucleotides are joined in a line by phosphodiester bonds (a type of strong covalent bond). Note that in this diagram, the DNA has not yet formed into the typical double-stranded helix.

The nitrogen-containing base distinguishes the nucleotides from one another. The covalent bonds holding adjacent nucleotides together are between carbon, phosphorus and oxygen atoms (Figure 3.2.9). When many nucleotides are joined together, a single polynucleotide chain, which runs from 5' to 3', is formed. Different nucleotides can occur in any order within a strand-if a particular base is A, the next base in the sequence could be A, G, T or C.

RNA

RNA is the other nucleic acid molecule and its role in the cell for polypeptide synthesis to produce functional proteins is explained in Chapter 4.

The differences between RNA and DNA are:

RNA only exists as a single strand

RNA polynucleotide strands are usually much shorter than DNA

the sugar-phosphate backbone has ribose sugar not deoxyribose

the nitrogenous bases are G, C,A and U ( uracil) which in RNA replaces T (thymine)

there are three main forms of RNA: messenger RNA (mRNA), ribosomal RNA (rRNA), transfer RNA (tRNA).

Bonding and pairing of nucleotides

The way that free nucleotides bond together to form long strands is through a condensation polymerisation reaction. This reaction occurs initially between two nucleotides, enabling them to join to form a dinucleotide, and releasing a water molecule as described below.

The hydroxyl group (OH) on the 3' carbon atom of the sugar of one nucleotide joins with the phosphate (P04) on the 5' carbon of the sugar of the other nucleotide to form water (H20), which is released.

Free nucleotides can then be continuously added to the 3' carbons in this way, forming a long sugar- phosphate-sugar-phosphate backbone strand known as a polynucleotide.

The nucleotides in the sugar-phosphate chain are joined by phosphodiester bonds.

In polynucleotide strands, one end has a free phosphate group on the S' carbon; this is called the S' end (five prime). The other end of the strand has a free hydroxyl on the 3' carbon; this is called the 3' end (three prime). The S' and 3' ends are significant when DNA copies itself for cell division.

Both DNA and RNA are polynucleotides, formed through condensation polymerisation reactions.

DNA is a double-stranded helix

A full double-stranded DNA molecule is made up of two polynucleotide chains. The two polynucleotide chains of DNA are held together by hydrogen bonds between complementary base pairs, rather like steps on a ladder. The sides of the ladder are now sugar-phosphate backbones. The rungs of the ladder are the paired nitrogenous bases of each nucleotide. There is always direct pairing between A and T and between G and C in the DNA molecule. This complementary base pairing results in the now polynucleotide strands joining together to form the double-stranded DNA molecule. Given the base sequence of one strand you can determine the sequence of the other by the complementary base pairing rule.

In complementary base pairing, the: purine adenine (A) always pairs with the pyrimidine thymine (T), held together with two weak hydrogen bonds.

purine guanine (G) always pairs with the pyrimidine cytosine (C), held together with three weak hydrogen bonds.

For the bases to face each other and form hydrogen bonds, one polynucleotide strand must run antiparallel to the other, meaning that one strand runs from 5' to 3' and the other runs from 3' to 5'. The two polynucleotide strands will then spiral around an imaginary axis in a right-hand turn, forming a double helix. It is like a rope ladder that is held at one end and twisted in a right-hand turn, or a spiral staircase. The 'steps' are only about 2 nm (nanometers) wide. The DNA double helix is a right-hand helix, meaning it curls in the direction of the right-hand fingers when holding the right thumb up. There are ten base pairs for each complete turn.

CHROMOSOMES

In a eukaryotic cell, chromosomes can have various shapes and sizes, and their appearance changes during the life of the cell may remain inside the nucleus except during some stages of the cell division processes. Chromosomes carry genetic information as a unique set of genes inherited from parents. Eukaryotic organisms have sets of linear chromosomes and the number of chromosomes is constant in each species (e.g. 46 (23 pairs) in humans). These chromosomes are passed on to daughter cells during mitosis and to germ cells during meiosis.

Prokaryotes have a single circular chromosome. Prokaryotes may also contain smaller circular DNA molecules called plasmids, which can move between cells.

Structure

Common wisdom says that chromosomes are made from DNA but this is only partly correct. Eukaryotic chromosomes also contain many small structural proteins called histones that are roughly shaped like spheres. The long thin DNA molecules are wound around a series of these histones forming structures called nucleosomes.

This complex structure is made into a supercoil that contracts tightly together to become the chromosome during condensation before the cell divides. At this stage in **prophase**, the chromosomes first become large enough to be seen under a light microscope if a stain for DNA is used.

Nucleosomes give the DNA strand the appearance of a string of beads. This arrangement of DNA wrapped around histones serves to package the long lengths of DNA efficiently and to protect it from enzymatic degradation. The end result is a fibre known as chromatin. The chromatin is looped and coiled again to produce a super-coiled thread-like structure called a chromosome.

Function

Chromosomes carry genes encoded in the base sequence of the DNA. The process by which the information in the gene is decoded to make proteins is called gene expression. By definition, the information carried in each gene can be expressed to make one polypeptide chain of amino acids, the precursor of protein molecules.

Genomes

The term genome is often mentioned in connection with chromosomes, DNA and genes. A genome is the complete set of genetic information in an organism and is measured in the number of base pairs. It provides all the information the organism requires to function and is distinctive for each organism and species. In eukaryotes, each cell’s genome is contained within the membrane-bound structure called the nucleus. Prokaryotes (bacteria), which contain no inner membranes, store their genome in a region of the cytoplasm called the nucleoid. The full range of RNA molecules expressed by a genome is known as its transcriptome, and the full assortment of proteins produced by the genome is called its proteome. The study and analysis of genomes is called genomics.

Features of chromosomes

When a eukaryotic cell is preparing to divide, chromosomes become condensed and are visible when the cell is stained and viewed at high magnification under a light microscope. The nucleosomes fold in a regular manner, producing supercoils.

In comparison, the DNA in prokaryotic cells is usually present in a single circular chromosome that is located in the nucleoid region of the cytoplasm. Prokaryotic chromosomes are less condensed than their eukaryotic counterparts. The internal features of prokaryotic cells are not easily identifiable when viewed under a light microscope so more powerful electron microscopes are required. The differences between the DNA of prokaryotic and eukaryotic cells are covered more comprehensively in Chapter 4.

The longest human chromosome (designated as chromosome number 1) has about 2000 genes. Each gene has a particular position, called a locus (plural loci), on a specific chromosome. Regions called spacer DNA separate the genes of each DNA molecule. Genes are highlighted in red and the spacer regions of DNA, separating the genes, are shown in blue. Spacer regions include DNA that does not encode a protein product. However, they may function in spacing genes far enough apart to enable enzymes or other molecules to interact easily with them. Chromosomes differ in length because of differences in the number of genes and the amount of spacer DNA between the genes.

Humans have 46 chromosomes, comprising 23 inherited from each parent. Forty-four of these chromosomes form 22 matching pairs. The same genes are found at the same locations (loci) on the two chromosomes in a matching pair. They are referred to as homologous chromosomes or homologues.

1'he sex chromosomes for all females are homologues because they have a matching pair of X chromosomes. The sex chromosomes for males are not homologous because they have an X and a Y chromosome, which contain different gene sets and are different lengths. Nevertheless, in most mammals the X and Y chromosomes behave as a homologous pair during meiosis because some small sections of these chromosomes match (i.e. are homologous).

Genes and alleles

Before continuing, it is worth revisiting some essential information from earlier in this section. DNA is the molecule of life that encodes the information from which organisms are built. The code carried by DNA consists of many genes, and these genes (genotype) determine the characteristics of an organism (phenotype). At a molecular level, a gene is a unique sequence of bases in the DNA. Each gene carries a particular instruction for a cell that is usually the production of a polypeptide chain. This process, by which the information in the gene is decoded to assemble the polypeptide, is called the gene expression. One or more polypeptides form proteins, which perform essential functions in an organism's cells.

Alleles

You can see, simply by looking at people around you, that there is variation in the human population. Observable physical characteristics or traits such as skin colour, eye colour and hair colour all vary within populations and even within families. Although an individual gene may be responsible for a specific trait, that gene can exist in different forms known as alleles. For example, there are genes that code for eye colour. Alternative forms of the gene (alleles) exist, including one for blue eye colour and another for brown eye colour. This means that the DNA sequence of bases for these new alleles is slightly different. Both alleles still code for eye colour and are found in the same place (locus) on the chromosome (DNA strand).

Somatic cells of a diploid organism contain two alleles for every gene, with one allele of every gene in an organism inherited from each parent. In Chapter 5, the gene expression of alleles will be investigated in more detail.

DNA REPLICATION

The DNA molecule is passed on from one cell to another when cells divide. In order to transmit an exact copy of the DNA molecule without losing any instructions, cells have a mechanism for synthesising and accurately copying new DNA in a process called self-replication. In the cell cycle before mitosis occurs, self-replication of DNA occurs in the S phase of interphase.

During self-replication, each strand of a parental DNA molecule acts as a template strand on which a new strand is synthesised. This involves the double­stranded DNA unwinding or 'unzipping' under control of enzymes (helicase and topoisomerase) at the replication fork. There is a bank of free nucleotide molecules stored in the nucleus. An enzyme called primase initiates the first stage of synthesis. The free nucleotides start to pair up with each template strand, using the rules of complementary base pairing (A pairs with T, G pairs with C), to make new strands. The replication process proceeds from the 5' towards the 3' end of DNA. As each new complementary strand forms, the replication fork moves along and the completed pairs of DNA strands begin to rewind into doubles helices under control of yet more enzymes, DNA polymerases. Each double helix coils around histone proteins and reforms as a chromosome with twice as much DNA as a non-replicated chromosome. The structure now consists of sister chromatids joined at a centromere and ready to undergo cell division. The mitotic cell division process will separate the sister chromatids into single chromosomes again, each with a full set of identical genes.

In summary

DNA replication is described as semi-conservative replication because each daughter DNA molecule consists of one old and one newly synthesised strand. It is a very accurate process with three distinct phases that can be summarised as:

parent DNA helix starts to separate its strands from one end as enzymes break the weak hydrogen bonds joining base pairs

free nucleotides attach to complementary partners on each strand, from the open end towards the replication fork

two identical sets of DNA are formed that each coil back into a double helix.

The full story

The full story of the DNA replication process is complex and it requires many enzymes to work in a coordinated fashion. For example, DNA polymerase is actually a multi-protein complex, with as many as 10 different proteins, acting like a sliding clamp that circles the DNA. After primase initiates synthesis using a RNA primer piece, the polymerase complex slides along adding more complementary daughter bases in sequence. It also checks the bases already added, editing and replacing incorrect ones, before rewinding the double helix that now consists of one parent and one daughter strand. The precision of the initial assembly mechanism, plus the checking process, means that DNA replication has a very low number of errors even though it proceeds at an amazingly rapid rate. Up to 4000 free nucleotides can be added every second. The estimated error rate under normal conditions is only 1 in 109 (one in a billion).

As already mentioned, the daughter strands must be built from their 5' to their 3' end. This is because DNA polymerase can only add nucleotides to the 3'-0H of an existing nucleotide. The job of primase includes synthesis of a short RNA primer that is used as a starter nucleotide section for the DNA replication. This primer is removed before completion of the DNA replication process. Knowledge of primers from the natural process of replication has been usefully applied for the technology of PCR (polymerase chain reaction) now widely used to copy DNA in the laboratory.

From one of the parent strands, the synthesis of a daughter strand, called the leading strand, can proceed continuously from S' to 3'. Because the two parent strands ran anti-parallel, the second parent strand is running in the wrong direction. Synthesis of this daughter strand, called the lagging strand, is achieved by forming short lengths of DNA that are later joined together by the DNA ligase enzyme. These sections are named Ozaki fragments in honour of the scientist who discovered them.

TI1us, DNA replication is continuous on the leading strand and discontinuous on the lagging strand. On the lagging strand, DNA polymerase has to operate by moving away from the replication fork, which is why the copying must be done as discontinuous fragments that are joined later. Having a discontinuous process makes it even more remarkable that accuracy is so high.

MUTATION AND OTHER DAMAGE TO DNA

Cells cannot function properly if the genetic material is changed, a situation usually classed as mutation. Mutations of DNA may affect a single gene or multiple genes. Despite the very high rate of accuracy, mistakes or damage to a cell’s DNA can occur during DNA replication. It can also be caused by environmental factors. Checkpoints throughout the cell cycle monitor for damage to the DNA. If the damage cannot be repaired, the cell will undergo apoptosis (progranm1ed cell death).

Somatic mutations occur in body cells and only affect that individual. Germline mutations are heritable because they affect gametes and can therefore be passed on to offspring. A germline mutation may bring a new allele into a gene pool, potentially influencing the allele frequencies. It is important to remember that mutation means change, not the introduction of a fault or disease. Mutations can have a beneficial or harmful effect, or no effect at all, on the organism.

Repair

It has already been mentioned that cells have enzymes that can detect and repair damage to DNA. These enzymes run along strands of DNA like a zip, checking that the DNA is intact and has been replicated properly. If there is minor damage, such as DNA breakages, these will be corrected by enzymes before the cell continues its progress through the cell cycle. If the bases are not complementary matches, they can be replaced if detected before DNA replication is completed in the S phase of the cell cycle. Without repairs to any faulty DNA, the cell may not be able to make the correct proteins or it could become a cancerous cell that replicates itself without going through the complete cell cycle.

DNA is the extraordinary molecule that carries a genetic code universal to all living things on our planet, even the prokaryotic bacterial cells. The code is faithfully inherited from one generation to the next, no matter what the species or which method of reproduction they use. DNA’s complex, but predictable and tightly-regulated structure was only elucidated fully in the 1950s. Since then, the developments and achievements of genomics have accumulated at an extraordinary pace.

To achieve its natural role, DNA self-replicates so that each time a cell divides there is a fresh set of identical DNA copied and ready to be inherited by a new cell. In both mitosis and meiosis of eukaryotic cells, the DNA has replicated before the start of nuclear and cell division.

During most of the cell cycle, DNA is reliably carrying out its routine function of producing polypeptides to be made into the many proteins needed by even the simplest organism.

3.3 Cell replication and the continuity of species

It seems contradictory that, on the one hand, cell division can produce cells that are the exact replicas of the parent cell and, on the other hand, can also introduce enough genetic variation for the evolution of new species. Even a quick glance at Figure 3.3.1 shows the huge variety and complexity of species on Earth that have evolved by diverging from simple, unicellular bacteria. The answer to this puzzle lies in the different outcomes of mitosis and meiosis that you learnt about in Section 3.1.

According to the **cell theory**, all cells arise from pre-existing cells. You have learnt about the processes at the cellular and molecular level that allow cells to replicate themselves. It should be apparent by now that the DNA inside cells is the key to understanding cell replication and inheritance from one generation to the next. DNA is universal (found in all organisms) but also characteristic of each species. This ensures genetic continuity is maintained within species but also allows genetic change for adaptation and evolution.

You can think about genetic continuity in different ways. It refers to the identical replication of genetic information from a parent cell to two daughter cells. Another aspect is the continuance of parental traits in their offspring. At a broader level, you can look at the effects of evolution on the gene pool within a population or species. All of these perspectives depend on an understanding of DNA.

In this section you will learn about the different outcomes for cell replication processes and the effect this has on the continuity of species. You will also consider the meaning of the continuity of species in the context of evolution and come to understand how the boundaries between species are defined.

GENETIC CONTINUITY IN A SPECIES

Section 3.1 emphasised how the process of cell replication produces daughter cells that are genetically identical to the parent cell. Exact replicas of the DNA are made in the S phase of the cell cycle and distributed to the new cells. 1'he cells need to make copies of themselves in order to maintain the continuity of life. This is the case for both unicellular and multicellular organisms.

In eukaryotes (protists, fungi, plants and animals), cells replicate by mitosis. Mitosis is part of the eukaryotic cell cycle described in Section 3.1. Cells replicate for a number of reasons, all of which must produce reliable copies. For a multicellular organism, cells replicate for:

restoring the nucleus-to-cytoplasm ratio during the cleavage stage of earliest embryo development

growth and development of the fetus ready for birth, then of the infant to adult size

maintenance and repair during an individual's lifetime.

Unicellular organisms do not need to replicate for these purposes because they remain a single cell throughout their entire life cycle. Instead, cell replication in unicellular organisms whether prokaryotes or eukaryotes) is a simple form of reproduction and creates a new, genetically identical individual.

Cell replication therefore maintains the species continuity by processes that copy the same genetic make-up of cells for any one individual in its lifetime from fertilisation to death. Barring mutations and rare diseases, an individual organism has the same number of chromosomes with the same genes as the parent, even though they have a different set of alleles for those genes. For sexual reproduction, they are going to be compatible with other members of their species because their chromosomes will form homologous pairs at fertilisation. The species can therefore continue from one generation to the next.

Healthy cells replicate in a highly regulated way with a cell cycle that uses in­built checkpoints in a cell cycle control system. However, there are times when cells divide uncontrollably. If uncontrolled cell division occurs during embryo development, the embryo will be abnormal and, in most circumstances will abort. If uncontrolled cell division occurs in a mature organism, a neoplasm may form.

A neoplasm is an abnormal growth of tissue that usually, but not always, forms a mass of abnormal cells. Neoplasms are more commonly referred to as tumours, but not all are cancerous. If a neoplasm develops, the genetic continuity has been lost because normal cell cycles of replication cannot continue.

GENETIC CHANGE IN SPECIES

Genetic continuity should not be confused with a lack of variation. A sexually reproduced individual is unique, resembling both parents but identical to neither.1bis is largely due to the variation introduced by meiosis during the production of gametes.

Variation within a species

Over the course of two cell cycles (meiosis I and II), germ cells undergo meiosis and form gametes containing only one set of chromosomes (haploid, n). The change from diploid to haploid chromosomal number is why meiosis is referred to as a reduction cell division. The mixed set (n) of chromosomes in a gamete contains a single copy of each chromosome randomly supplied from either of the partner chromosomes in the diploid (2n) parental set. For example, human somatic cells contain 46 chromosomes (23 pairs) but the sperm and egg gametes have 23 single chromosomes drawn at random from the homologous partners, giving each gamete a different combination of alleles for the same genes.

During meiosis I even more variability is added by crossing over between some homologous chromosomes. Crossing over is a process found only in meiosis. It exchanges sections of DNA carrying some alleles, and creates new recombinant chromosomes with a set of alleles not found in either parent. At fertilisation, the union of egg and sperm (conception) restores the full diploid number of chromosomes (2n) to the zygote. Fertilisation itself is a random event, when only one sperm contributes its mixed set of chromosomes to form homologous pairs with the female chromosomes in the egg. The randomness of which specific sperm fertilises the egg introduces yet more variation to the offspring from one parental pair and helps explain why siblings are different.

The genetic continuity here is that a successful fertilisation event, leading to viable fertile offspring, can only occur with matching (homologous) chromosomes carrying the same genes at the same loci. Variation in individuals is present in the unique combinations of alleles at gene loci but the genes themselves must have continuity from generation to generation to maintain a species. The species continues generation after generation but with individuals in that species having some genetic variation, such as tall or short height, yellow or green pea pods, black or white fur, or blood group type. Such variation in traits does not prevent interbreeding for the next generation within the same species. In fact, populations of organisms that reproduce sexually have considerable genetic flexibility, which enables species to survive and reproduce in varied and changing environments.

When meiosis goes wrong

Meiosis is usually an exact process, but sometimes errors occur. Missing, extra or malformed chromosomes can result from defective gametes, which may have serious consequences for offspring. One of the errors that can occur is if members of a pair of homologous chromosomes do not separate properly, resulting in abnormal development of the offspring. There are also changes in chromosome structure such as deletions, duplications, translocations and inversions of chromosome segments resulting in missing, duplicated or misplaced alleles. Deletions result in the embryo having a number of essential genes missing and are usually lethal. Duplications, translocations and inversions can result in changes in physical characteristics because a gene's expression can be influenced by its location among neighbouring genes. Another situation, much more common in plants, is polyploidy (3n, 4n, and so on) that can arise through errors in meiosis. For example, gametes may end up being diploid rather than haploid.

Variation between species

1'here is a wide variation in the diploid number of chromosomes (2n) among species, ranging from 2 (1 chromosome pair) in some Australian ants to 78 (39 homologous pairs) for the dingo, and even higher for some plants. Difference in the number of chromosomes is the main distinction between species and the barrier to successful interbreeding between different species.

Hybrids between species were discussed in the Year 11 course. Even if their differing numbers of chromosomes did combine into a zygote at fertilisation, the resultant embryo is likely to be malformed and not develop into a fetus. The few species that do interbreed successfully usually have offspring that are not fertile and cannot continue to keep breeding. For example, the hybrid offspring of a female horse (2n = 64) and a male donkey (2n = 62) is a mule with a total of 63 chromosomes making it infertile (unable to produce viable offspring). The chromosomes of different species are incompatible because they have different genes at the specific loci on chromosomes, meaning that their chromosomes are not homologous and the alleles are not alternatives for the same genes. Without a full set of homologous pairs of chromosomes, meiosis cannot proceed normally to produce future generations. There is a discontinuity rather than continuity because the break down in the meiosis process blocks further cell replication.

Chapter 4 DNA and polypeptide synthesis

4.1 DNA in eukaryotes and prokaryotes

There are two fundamentally different types of cells: prokaryotic and eukaryotic. Organisms are classified according to the type of cell that forms their structure. You learnt about the structure of typical prokaryotic and eukaryotic cells in Year 11.

Prokaryotes are usually unicellular, composed of a single prokaryotic cell, and include all species of Bacteria and Archaea. Prokaryotic cells are generally smaller and less complex than eukaryotic cells. The main distinction is that prokaryotic cells have chromosomal DNA (deoxyribonucleic acid) but do not hold it in a defined membrane-bound nucleus inside the cell.

Eukaryotes are composed of eukaryotic cells and include plants, animals, fungi and protists. They may be unicellular (protists, some fungi) or multicellular (plants, animals and some fungi). Eukaryotic cells contain membrane-bound organelles including the nucleus that contains the DNA inside a double nuclear membrane.

DNA IN PROKARYOTIC CELLS

Prokaryotic cells are small and lack most membrane-bound organelles, including a distinct nucleus. The term prokaryote actually means 'before nucleus' their cytoplasm does contain scattered ribosomes, built of two subunits which are not bound by membranes and are involved in the synthesis of proteins. The gene-tic material of prokaryotic cells is usually one double-stranded, circular DNA chromosome, which is contained in an irregularly shaped region called the nucleoid. Unlike the nucleus of eukaryotes, the nucleoid is a region of the cytoplasm, not an organelle, and it does not have a nuclear membrane surrounding it.

This circular chromosomal DNA is attached to the inside of the prokaryote's outer cell membrane by a region of the chromosome called the origin. Because the DNA is joined in a circle, unlike the linear chromosomes of eukaryotic cells, it does not have the protective end regions called telomeres that have a role in the ageing process of a eukaryotic cell.

In addition to the chromosomal DNA, many prokaryotic cells also contain small rings of double-stranded DNA called plasmids, which can move between cells Replication of plasmids is independent of chromosomal replication during binary fission. Plasmids often provide bacteria with genetic advantages, such as antibiotic resistance. From the 1970s, seeing the possibilities in working with a complete small ring of self-replicating DNA, scientists began to use bacterial plasmids in genetic engineering as vectors to clone, transfer and manipulate genes.

Prokaryotic chromosomes are less condensed than their eukaryotic counterparts. Unlike eukaryotic chromosomes, the prokaryotic chromosomes do not have features that are easily identifiable when viewed under a light microscope. Usually a stained slide will just show the dark area of the nucleoid.

Most of what we know about the chromosomes of prokaryotes has been obtained from studies of the bacteria, Escherichia coli. This species is used as the model organism for much of the research on prokaryotes. It is known that E. coli and other bacteria do not have the histone proteins used to condense DNA in eukaryotes. However, some Archaea (the other domain of prokaryotes) do have histones, which is of significance in evolutionary history.

Many prokaryotes also have small hair-like projections called pill, which are involved in the transfer of plasmid DNA between organisms by conjugation and can also help generate movement. You learnt about asexual reproduction by conjugation in Chapter 2.

Gene expression in prokaryotes

In prokaryotic cells, gene expression consists only of polypeptide synthesis and occurs completely within the cytoplasm. Here the two processes of synthesis, transcription and translation, are closely coupled and occur at almost the same time. The action is between the DNA, messenger RNA (mRNA) and ribosomes in the cytoplasm of prokaryotic cells. The two stages involved in synthesis of polypeptide chains will be covered in detail in Section 4.2. Gene expression in prokaryotes is regulated during transcription, the first stage. Unlike eukaryotes, in prokaryotes there is very little processing of RNA, which is the level at which regulation of gene expression takes place for the eukaryotic cells.

DNA IN EUKARYOTIC CELLS

Eukaryotic cells are more complex than prokaryotes. Eukaryotic cells not only have a cell membrane around the cytoplasm, but also have internal membranes that form specialised membrane-bound compartments within the cell. This is known as cell compartmentalisation. The membrane-bound structures are called organelles.

Packaging of DNA in eukaryotic cells

In eukaryotic cells, the double-stranded DNA is found in the nucleus where, at times, it is tightly coiled up (condensed) to form me linear structures called chromosomes. At other stages of the cell cycle, the uncondensed DNA strands form a tightly woven mass. Because eukaryotic cells are more complex than prokaryotes, the eukaryotes have an average of 25 times more DNA man prokaryotes. For it to fit into the nucleus, this amount of DNA has to be much more tightly packaged.

TI1e processes involved in DNA replication are significantly faster in prokaryotic cells. Some bacterial cells take just 40 minutes to replicate their DNA, while in some animal cells this can take up to 10 hours before cell division.

The structure of DNA and its packaging into nucleosomes, men into linear pairs of homologous chromosomes for eukaryotic cells, has been discussed in Chapter 3. Figure 4.1. 7 summarises this information in a diagram.

Gene expression in eukaryotes

Gene regulation is tightly controlled in both eukaryotes and prokaryotes. However, as the process of gene expression in eukaryotes is more complex, gene regulation checks occur at a greater number of stages.

In Section 4.2 you will learn that gene expression in eukaryotes comprises the processes of transcription, RNA processing and translation. Gene expression can be regulated at any of these stages. In eukaryotic cells, transcription and RNA processing occur within the nucleus, and translation occurs in the cytoplasm.

COMPARISON OF PROKARYOTIC AND EUKARYOTIC DNA

Both types of cells contain double helix DNA as their inherited genetic material, making DNA universal in structure and function to all living things on our planet. An essential difference between prokaryotes and eukaryotes is the way me DNA molecule is packaged inside their cells.

Similarities

Although there are many differences between the gene structures and DNA packaging of prokaryotes and eukaryotes, shared evolutionary history means that there are also many fundamental similarities.

Both prokaryotes and eukaryotes have double-stranded DNA, twisted into a double helix and built from the same type of nucleotides with the same nitrogenous base molecules-adenine (A), thymine (T), cytosine (C) and guanine (G).

Both have mRNA, which acts as an intermediate code to building proteins with the base uracil (U) replacing thymine (T) in the mRNA.

Because the genetic information of prokaryotes and eukaryotes is composed of the same code, the way mRNA is translated into amino acids and proteins is also much the same. The universality of the DNA and mRNA coding has made the use of prokaryotic plasmids possible as vectors for genetic engineering across all species. Examples of the use of plasmid vectors can be found in Chapter 9. Section 4.2 will cover polypeptide synthesis in more detail.

Differences

Prokaryotic and eukaryotic genes are structurally different in several ways. These differences affect the way in which genetic information is transcribed, translated and expressed.

4.2 Polypeptide synthesis

The genetic code represents the inherited genetic information stored in DNA as triplet bases \\rithin sections called genes. This information is transcribed to RNA, then used to synthesise the amino acid sequences that form polypeptides through a process called gene expression. Chains of polypeptides combine to form proteins (Figure 4.2. l). Proteins are biological molecules that carry out most of the functions that are essential to life. Antibodies for immune response, collagen for tissue repair, enzymes that catalyse biochemical reactions, hormones for cellular signalling and haemoglobin for transport are all types of proteins.

THE GENETIC CODE

The genetic code is a set of rules that defines how the information in DNA and RNA is translated into polypeptides, which join to form proteins. The genetic code is universal-the rules are the same for all organisms on Earth.

As you learnt in Chapter 3, the information in DNA and RNA is stored as a three-letter code of nucleotides. RNA works in partnership with DNA to synthesise polypeptides as required by a eukaryotic cell.

Role of DNA in polypeptide synthesis

DNA provides the instructions, which are translated by RNA into polypeptides and proteins. DNA stores and transmits hereditary information as a sequence of nucleotides. The order of the nucleotides in DNA determines which polypeptides are synthesised. Groups of three nucleotides are called triplets. When a DNA triplet is transcribed into mature mRNA, the triplet is then called a codon. For polypeptide synthesis to occur, DNA and RNA must function together.

Role of RNA in polypeptide synthesis

RNA plays an important role in expressing the information contained in the DNA sequence to synthesise polypeptides. There are three main forms of RNA and each has a different role in polypeptide synthesis:

Messenger RNA (mRNA) is formed in the nucleus by the process of .transcription. The mRNA carries a complementary copy of the nucleotide sequence of DNA that specifies the amino acid sequence for a particular polypeptide. During transcription, the primary transcript (pre­mRNA) is first formed by the enzyme RNA polymerase. Pre-mRNA is then processed (post-transcriptional modification) to form mature mRNA, which is a single-stranded complementary copy of the coding DNA (gene or exon). The mature mRNA travels from the nucleus to the cytosol where it binds to ribosomes ready for translation.

Ribosomal RNA (rRNA) is synthesised in the nucleolus of the cell nucleus and is based on the nucleotide sequence of the DNA. Together with proteins, rRNA forms a small organelle called a ribosome. Ribosomes are the sites where the information in the mRNA is translated into a chain of amino acids.

Transfer RNA (tRNA) molecules transfer amino acids from the cytoplasm to the ribosomes, where they are joined to form a polypeptide chain based on the sequence of nucleotides in the mRNA.

There are 61 different tRNA molecules, each of 1which combines with only one particular amino acid at one end of its molecule. (There are 64 codons that each represent an amino acid, three of which are stop codons. There are no tRNA molecules that recognise these stop codons and so translation is terminated.) There are three places for tRNA to bind to the ribosome: the exit site (E), the pep, tidyl site (P) and the aminoacyl (A) as shown in Figure 4.2.6b. At the other end of the tRNA molecule, there is a sequence of nucleotides known as the anticodon. The anticodon recognises a particular sequence of nucleotides in the mRNA. This enables an amino acid to be positioned in the correct place on a polypeptide chain.

Degeneracy of the genetic code

The genetic code is said to be degenerate because more than one RNA codon can code for the same amino acid. Differences in codons encoding the same amino acid usually occur at the second or third base. As the genetic code uses four nucleotides and three nucleotides code for an amino acid, the combinations of these nucleotides make a total of 64 possible codons (43 = 64), to code for the total 20 amino acids. The degeneracy of the code acts as a buffer against mutations in that a single change in one base may not necessarily lead to a change in the amino acid produced. Therefore, it may not necessarily change the structure of the protein produced.

THE STRUCTURE OF GENES

While the genetic code is universal, the structure of genes and chromosomes differs between prokaryotes and eukaryotes. Prokaryotes contain fewer introns than eukaryotes, which simplifies the process of polypeptide synthesis in prokaryotic cells.

Eukaryotic genes all have structural features in common, including:

Stop and start triplet sequences-regions where encoding DNA begins and ends for a specific gene

Promoter--sections of a gene that are found on the DNA before the start triplet (start codon), at the 5' end of the site where transcription will begin. The promoter is the location where the RNA polymerase (the enzyme that initiates transcription) attaches to the gene. In many eukaryotic genes, the promoter region is coded for by the sequence of bases TATAAA, which is sometimes called the TATA box

Exons-DNA regions that are the coding segments

Introns (or spacer DNA)-DNA regions that are non-coding segments.

Introns and exons

In eukaryotes, not all DNA regions are translated.

Exons are DNA regions that are usually 'expressed' as proteins or RNA. Exons come together to make up mRNA, which is then translated into proteins.

Introns are non-coding, or intervening, regions of DNA. Introns are spliced out of the mRNA during RNA processing.

GENE EXPRESSION

Gene expression is the process by which the information stored in a gene synthesises a functional gene product (protein or RNA). This process is highly regulated so that proteins or RNA molecules are only produced if they are required by a cell. Multicellular organisms in particular have specialised cells that require a specific set of proteins. For example, in humans, the cells in connective tissue and bone require the protein fibrillin to form elastic fibres, and skin cells require the enzyme tyrosinase to produce melanin and other pigments. The ability to regulate gene expression conserves energy and materials in the cell.

Gene expression leading to polypeptide synthesis in eukaryotic cells occurs in three stages:transcription

RNA processing translation.

In eukaryotic cells, transcription and RNA processing occur within the nucleus and translation occurs in the cytoplasm.

Transcription

Producing single-stranded mRNA from DNA is called transcription and occurs within the nucleus of eukaryotic cells. The DNA segment that undergoes transcription is known as the transcription unit.

Transcription occurs in three steps: initiation elongation termination

Many RNA polymerase molecules may attach to the gene being transcribed, producing many of the same mRNA molecules. The strand of DNA that is transcribed to the mRNA is known as the non-coding strand (or template strand) and the other complementary strand is known as the coding strand (or non­template strand). The mRNA carries the same base sequence as the coding strand, (except it contains uracil in place of thymine) because it has been copied from the non-coding strand into complementary bases.

RNA processing

After transcription, the primary RNA transcript is processed before being translated. RNA processing forms mature rnRNA from pre-rnRNA (the primary transcript of rnRNA), after removing non-coding sequences (introns) so that only the coding sequences (exons) are carried to the ribosome for translation (Figure 4.2.11). RNA processing is present in both eukaryotic and prokaryotic cells but is much more complex in eukaryotes, as prokaryotes carry fewer introns that need to be spliced out after transcription. The RNA processing stage of gene expression in eukaryotes includes:the addition of a 5' cap, the addition of a poly(A) tail (The 5' cap and poly(A) tail make the mRNA more stable and prevent it from degrading.), splicing (removal) of the introns (mRNA maturation).

For prokaryotes, without a nuclear membrane, the transcription and translation processes are closely coupled in the cytoplasm, with the mRNA moving off the DNA and becoming attached to ribosomes even before transcription is complete.

Translation

Translation is the process in which the codons on mRNA are translated into a sequence of amino acids resulting in a polypeptide. 1'his process occurs on ribosomes in the cytoplasm. Ribosomes bind to an mRNA molecule and act as docking stations for the tRNAs to deposit their specific amino acids. A part of the tRNA, called an anticodon, recognises and binds to the codon on the mRNA by complementary base pairing. Each tRNA carries a specific amino acid related to the codon to which it binds.

Like transcription, translation also occurs in a series of three steps: 1 initiation 2 elongation 3 termination

Many ribosomes can translate the same, single strand of mRNA, enabling many polypeptide chains to be produced at the same time. Once the polypeptides are fully functional, they either remain in the cell for use, or are exported from the cell by vesicles (via a process called exocytosis) for use elsewhere in the organism.

Protein synthesis in prokaryotes

Prokaryotes do not have membrane-bound organelles, so all cellular processes occur within the cytosol. This allows transcription and translation to be a continuous process rather than two separate stages. Ribosomes can attach to the mRNA while it is being transcribed, so translation can occur at the same time. Prokaryotes mostly contain exons, so splicing rarely occurs before translation.

There are many differences between protein synthesis in prokaryotic and eukaryotic cells. Table 4.2.1 summarises the major differences, many of which have been used to develop drugs that target protein synthesis in prokaryotes only. For example, some antibiotics disrupt or inhibit the production of proteins in disease-causing bacteria.

AMINO ACIDS, POLYPEPTIDES AND PROTEINS

Protein molecules are more complex than other biomolecules like carbohydrates or lipids. For example, the haemoglobin that carries oxygen in human blood has a chemical formula of C2952H46640g32N812S8Fe4. Proteins make up more than 50% of the dry weight of cells. There are thousands of different kinds of proteins, their functions vary widely and they are essential to the wellbeing of any organism. Although carbohydrates and lipids are similar in all plants and animals, organisms can have a variety of unique proteins that are specific to a species. This section explains the way proteins are synthesised inside cells from the inherited DNA code.

Proteins are biomacromolecules made of chains of subunits called amino acids. Amino acids are linked by a chemical bond called a peptide bond and form polypeptides or polypeptide chains. Polypeptide means 'many peptide bonds'. A peptide is a linear sequence of fewer than 50 amino acids. A dipeptide is a molecule consisting of two amino acids joined with a peptide bond. A polypeptide is a molecule consisting of many (more than 50) amino acids joined together by peptide bonds. A protein is formed by one or more polypeptides arranged in a biologically functional way. In other words, 'protein' is the term used for a fully functioning molecule while 'polypeptide' refers to a non-functioning component.

There are 20 different amino acids commonly found in proteins. Nine of these are known as essential amino acids because they cannot be produced by humans. Humans must obtain these from their food. The properties of many proteins are determined by their shape, which is determined in turn by their amino acid sequence.

There are many steps involved in producing a functional protein. Although protein structure, size and function are quite diverse, all proteins are made up of amino acids. These smaller subunits (or monomers) are joined together in a particular order to form polypeptide chains. The polypeptide chains are then folded and coiled into proteins.

The function and importance of polypeptide synthesis

The genome consists of many thousands of genes. A cell can express a selection of these genes at a given time. The genes that are expressed determine which proteins are produced, giving the cell its functionality and characteristics. Gene expression is the process through which information from a gene is used to synthesise a specific functional gene product-a polypeptide or length of RNA. Gene expression in eukaryotes is tightly regulated by multiple mechanisms at different points, usually in the transcription phase. The regulation determines that the correct polypeptides and proteins are produced when and where they are required. For example:

growth hormone proteins are produced in infants, children and teenagers but switched off at other times

plasma B cells of the immune system are regulated to only produce antibody proteins when they are required to fight an infection

enzyme proteins are recycled but new ones ,viii be synthesised to replace those that have reached the end of their useful life

continual production of haemoglobin in tile bone marrow of vertebrates.

GENOTYPE, PHENOTYPE AND THE ENVIRONMENT

The proteome is the complete set of proteins expressed by the genome (the complete set of genes) of an individual cell or organism at a given time. The proteome varies between cell type, developmental stage and environmental conditions. Although a cell may contain the entire genome, only specific genes will be expressed, or 'switched on', at any given time. This ensures a cell produces only the proteins required for the specific functions it carries out.

The phenotype is the set of characteristics expressed from the genetic information of a cell. That is, the part of the genotype that has been switched on to allow transcription and translation to produce polypeptides, then proteins.

An organism's genotype and environment determine its phenotype.

If an individual with a given genotype develops in one environment, its phenotype may be different than if it had developed in a different environment.

For example, the average height of humans has gradually increased in the last few hundred years because of improved nutrition.

On the other hand, a human cannot grow to the full height potential that is coded in their genotype if they are severely undernourished as a child or if a bone disease affects their growth.

A tree will be stunted in height if it does not receive the water and minerals needed to reach its genetic potential or if it grows with continual strong wind, in constant shade or is grown in a small pot where it becomes root-bound.

A human's skin colour depends partly on the amount of sunlight exposure.

In the short term, it can be altered by air temperature or exercise, such as being red-faced after strenuous exercise or pale-skinned on a very cold day.

Other examples of environmental influence on phenotype are:

the development and severity of inherited disorders such as phenylketonuria (PKU)

flower colour variation with soil pH

seasonal variation in the fur colour of Arctic foxes

feather colour variation in flamingos with different levels of carotenoids (organic pigments produced by algae, bacteria and fungi) in their diet

4.3 Structure and function of proteins

Nearly every function of a living organism depends on proteins. Proteins speed up chemical reactions, and play a role in cell-to-cell recognition and cellular communication, movement, storage, cell membrane functions and even structural support. A human has tens of thousands of different proteins, and each protein has a specific sequence of amino acids, giving it a unique shape that enables it to carry out a particular function. Proteins have a large variety of functions, so they vary extensively in structure with each type of protein having its own complex three-dimensional shape. Many proteins function with other chemical groups attached to them, such as the glycoproteins that are a combination of polypeptides and sugar molecules.

In this section, you will learn about the diversity in the functions of proteins and the nature of the proteome. First, the importance of the four hierarchal levels of protein structure will be investigated as molecular structure plays an essential role in effective functioning for each type of protein.

PROTEIN STRUCTURE

Proteins are large biomolecules that vary in size, from less than 10 amino acids to thousands of amino acids, and may be synthesised as one or several polypeptide chains. These chains are folded and organised into specific shapes that are vital to the correct functioning of the protein. Most proteins are required to bind to other molecules to achieve functionality. A single change to one amino acid within the sequence can alter the shape, and consequently the function, of a protein. Likewise, if an essential partner atom or molecule is missing, the protein may lose or reduce its ability to function. For example, the haemoglobin protein that carries oxygen in the human bloodstream has iron as part of its quaternary structure to bind oxygen.

Synthesis of proteins

There are many steps involved in producing a functional protein. Although protein structure, size and function are quite diverse, all proteins are made up of amino acids. These 20 types of smaller subunits (or monomers) are joined together in a specific order to form polypeptide chains. The polypeptide chains are then folded and coiled into proteins. With 64 different triplet codes for gene expression, 20 different amino acids, more than one polypeptide per protein molecule and a variety of extra partner molecules, there are almost endless possibilities for different forms of proteins.

Amino acid structure

All amino acids have the same basic structure

an amine group (NH2) a carboxyl group (COOH) an R group (or side chain)

In the synthesis of proteins in organisms, there are 20 different standard amino acids, and each has a different R group. The variable properties of the R group (e.g. charged or uncharged, polar or non-polar, hydrophobic or hydrophilic) determine the type of protein that the amino acid will form. R groups can be as simple as a hydrogen atom (as in the amino acid glycine) or more complex (e.g. -CH(CH3)2) as in the amino acid valine.

Condensation polymerisation of amino acids

Amino acids are joined by peptide bonds in a condensation polymerisation reaction, which involves the removal of water (dehydration). A hydrogen atom and an oxygen atom from the carboxyl group of one amino acid join with a hydrogen atom from the amine group of another amino acid to produce water. The water is released and a dipeptide is synthesised, with a peptide bond holding the two amino acids together. A chain of amino acids joined by peptide bonds is known as a polypeptide chain. The backbone of the polypeptide chain is formed by the repeats of the carboxyl and amine groups, with the R groups forming the side chains of the polypeptide chain.

A polypeptide chain forms the primary structure of a protein. With further folding and modification, a fully functional protein can be formed.

Levels of protein structure

There are four different levels of organisation when describing protein structure:

primary secondary tertiary quaternary

Primary structure

The linear sequence of amino acids in the polypeptide chain is referred to as the primary structure of a protein. It is unique to each protein. The primary structure of one polypeptide may consist of only 50 amino acids, whereas another may consist of 103 amino acids. Linear sequences shorter than 50 amino acids are known as peptides.

The linear sequence:

provides information on how proteins will fold

can be compared to identify differences between functional and non-functional proteins and determine the changes that render proteins non-functional

can be compared between proteins to determine the evolutionary history of a protein.

Secondary structure

The next step in forming a functional protein is folding or coiling the polypeptide chain-its secondary structure. Folding or coiling occurs because of the formation of hydrogen bonds between the amine and carboxyl groups of amino acids within a polypeptide chain that have come in close proximity to each other. This results in the formation of secondary structures. There are three types of secondary structures.

Alpha helix-Hydrogen bonds form between adjacent amine and carboxyl groups within the polypeptide chain and the chain coils to form a helical shape.

Beta-pleated sheets-Hydrogen bonds form between amine and carboxyl groups in different parts of adjacent polypeptide chains, causing the chains to fold back on each other.

Random coil-Although parts of the polypeptide chain appear to have a random structure, the folding is not random and the same folding occurs in all molecules of the same protein. For example, all insulin molecules will have the same random coils within their structure.

Tertiary structure

Polypeptides also fold further, forming more compact and stable globular or fibrous three-dimensional shapes. This is known as the tertiary structure, and is usually the result of a combination of alpha helices and beta-pleated sheets along with other folded areas. The structure occurs because of different types of bonds, such as the disulfide bridge and the hydrogen bridge, between the R groups (side chains) of the amino acids.

The three-dimensional structure of a protein is critical to its function. In some smaller polypeptides, this folding process occurs spontaneously due to its chemical environment. However, larger, more complex proteins require specialised proteins (e.g. chaperonins) to help them fold correctly and, in some cases, to refold if they unravel and lose their natural shape (i.e. denature).

Quaternary structure

A quaternary structure is formed when two or more polypeptide chains or prosthetic groups (an inorganic compound that is involved in protein structure or function) join to create a single functional protein. The polypeptides may be identical or different. Some proteins will not become active until they achieve this structure. A protein with a prosthetic group is known as a conjugated protein. Haemoglobin is an example of a conjugated protein with a quaternary structure. It has two haem units containing iron (Fe) atoms that are essential to its oxygen transport function.

PROTEIN CLASSIFICATION

Proteins can be classed as one of two types depending on their shapes.

Fibrous proteins are typically elongated and insoluble with little or no tertiary folding. Many fibrous proteins have structural roles (e.g. collagen found in connective tissue and keratin found in hair and nails).

Globular proteins are compactly folded and coiled into spherical tertiary and quaternary structures. They are generally soluble, having a core with hydrophobic properties and an outer hydrophilic region. Most enzymes and hormones are globular proteins.

FUNCTIONAL DIVERSITY OF PROTEINS

There are many different types of proteins in organisms. Each protein has a different function, and each plays a vital role in the regulation, functioning and maintenance of both individual cells and entire organisms. In fact, almost every function of living organisms depends on proteins. The specific structure of each protein enables it to carry out its function. The main types of proteins can be grouped according to their function.

THE NATURE OF THE PROTEOME

The proteome varies between cell type, developmental stage and environmental conditions. Although a cell may contain the entire genome, only specific genes will be expressed, or switched on', at any given time.1'his ensures a cell produces only the proteins required for the specific functions it carries out.

Interestingly, there are many similarities between human and other proteomes, reflecting their common evolutionary origins and the universality of DNA. The human proteome contains proteins related by evolutionary descent with 61% of the fruit fly proteome, 43% of the worm proteome and 46% of the proteome of baker's yeast.

Proteomics

Proteomics is the large-scale study of the structure, function and interactions of proteins. Proteomics is a vital area of biology because it is proteins that carry out most of the activities of the cell, not the genes that encode them. By knowing when and where proteins are produced in an organism, as well as how proteins interact, we can better understand how cells and organisms function.

One of the ways to determine changes in the proteome is by comparing the proteomes of cells under different conditions. For example, by comparing the protein expression of a diseased cell and a healthy cell, the proteins affected by the disease can be determined.

Proteomic research can lead to the creation of protein biomarkers that can be used for screening individuals and populations for early detection of disease. The study of proteomics is also important in producing drugs that interact with proteins involved in disease and alter their function. Proteomics has been revolutionised by creation of the Protein Data Bank (PDB), a free international database that stores information about all known proteins and nucleic acids.

FACTORS THAT AFFECT THE STRUCTURE AND FUNCTION OF A PROTEIN

The environment surrounding proteins plays an important role in maintaining its structure and function. Usually the protein's loss of function is due to denaturation of the protein. The factors in the environment that affect protein structure and function include:

temperature

pH

concentration of ions or molecules that act as cofactors.

Denaturation and renaturation of proteins

A protein is said to have denatured when the hydrogen bonds, disulfide bridges, hydrophobic interactions and van der Waals forces that create the tertiary structure of the protein are broken and the shape of the protein is altered. As a result, the misshapen protein is biologically inactive and non-functional. If a protein becomes fully denatured, the reaction is non-reversible and the protein remains non-functional. In this case, a protease will usually destroy the defective molecule. However, a protein that is partially denatured may be able to fold again when the appropriate conditions are present.

The effect of temperature on protein function

Proteins can be denatured at high temperatures due to the breaking of bonds. For example, hydrogen bonds break at temperatures above 40°C. However, at temperature below 35°C, the bonds are not flexible enough to allow the necessary conformational changes to break apart.

The optimal temperature for proteins varies with the organism and its environment. In humans, the optimum temperature for proteins is 37°C, but proteins found in organisms living in extreme environments, such as hot springs or icy environments, tend to have different optimal temperatures. Prokaryotes of the domain Archaea include cells that function at up to l 20°C and in sub-zero temperatures where enzymes of other organisms would fail.

The effect of pH on protein function

Most proteins have a specific pH range in which their function is optimal, but this range can be quite different for each protein. The pH effect is unlike temperature, where the range of optimal temperatures is similar for most proteins within an organism. In humans the optimum pH for some enzymes of the digestive system are pH= 7 for amylase (starts digestion of starch in the mouth), pH= 2 for pepsin (digestion of protein food in the stomach), and pH= 8 for trypsin (digestive enzyme in small intestines).

If the pH increases too far above or falls too far below the optimal pH, then the tertiary structure of a protein is affected. The interactions between the R groups of different amino acids are altered and the bonds between them are broken. As a result, the protein may be denatured, and in the case of enzymes the enzyme activity will decrease or halt.

The effect of cofactors on protein function

Some proteins require non-protein chemical compounds known as cofactors for their biological function. The presence and concentration of cofactors such as salts, specific elements such as iron, magnesium and calcium ions, or organic molecules such as vitamins can play a significant role in the folding and function of proteins.

Examples of proteins that require cofactors include:

haemoglobin, which requires iron other respiratory proteins called haemocyanins use copper to attach and transport oxygen in a variety of arthropods and molluscs

carboxypeptidase, an enzyme that requires zinc for splitting peptide bonds

fibrillin, an important structural protein that uses calcium.

some molecular pump proteins in humans use copper and when these are defective there can be a toxic build-up of copper causing liver scarring or a shortage of copper that causes babies to have hair resembling steel wool (Menkes disease). The fleece of sheep grazing in copper-deficient areas show similar symptoms to humans with Menkes disease.

the cytochrome group of proteins perform oxidation and reduction reactions in mitochondria of all living organ isms, thus powering all the cellular processes that require energy. They require cofactors that include copper, iron and sulphur without which the cell's metabolic rate reduces or stops.

Chapter 5 Genetic Variation

5.1 Formation of genetic variation

You inherited many of your physical features or traits from your parents. You share certain traits \\rith your mother and others with your father. You might even appear to have a totally different version of a trait.

In this section you \viii look at what determines the traits and characteristics that humans have and how they are passed to children from parents. You will learn the difference between genotype and phenotype, the use of symbols for alleles, and the distinction between dominant phenotypes and recessive phenotypes. You will also explore how polygenic inheritance contributes to continuous variation in a population, through examples such as human skin colour and variation in height.

GENOTYPES AND PHENOTYPES

A genotype is the set of alleles present in the DNA (deoxyribonucleic acid) of an individual organism. It is the result of inheritance. An allele is an alternative form of a gene. Each individual usually only has two alleles for each trait: one inherited from their mother and one inherited from their father. But one gene may have many alleles, and this is what leads to variation in a population.

An organism's phenotype is all of its observable characteristics. It is the result of inheritance and the effects of the organism's environment. An example of a phenotype is skin colour. Your skin colour depends on how much skin pigment (melanin) is produced. But skin colour also depends on environmental factors such as exposure to sunlight, especially in pale-skinned people. The greater the exposure, the more melanin is produced.

Genotype

You will recall from Chapter 3 that a diploid cell has two sets of chromosomes (one set from each parent). Humans are described as diploid because we have two copies of every chromosome in all cells except gametes (which only have one copy and are therefore haploid). Chromosomes therefore exist in pairs. When both members of a pair of chromosomes are identical, that is, they carry the same genes, they are referred to as homologous chromosomes. In the human genome, only the XY pairing of sex chromosomes is non-homologous. All other chromosome pairs are homologous.

Consider a gene, which might be called gene A, that has two alleles. One allele can be represented by an upper case A, and the other by a lower case a. The names of genes and alleles are always italicised.

If gene A is in a homologous chromosome, there will be two copies of the gene. If you inherited the allele A from both parents, your genotype for gene A will be AA. On the other hand, if you inherited the A allele from one parent and the a allele from the other parent, you will have the genotype Aa. If you inherited the allele a from both parents, you will have the genotype aa.

Therefore, there are three different combinations or genotypes of gene A: AA, Aa or aa. Genotypes AA and aa contain only one type of allele, so the individual is said to be homozygous for that gene and is called a homozygote. Genotype Aa contains two different alleles (i.e. one copy of each allele), so the individual is said to be heterozygous for that gene and is called a heterozygote.

A single gene locus (position on a chromosome) may have more than two alleles. The human ABO blood group system is based on such alleles. In this case there are three alleles, represented as Ia, Ib, and i. Each person carries two copies of these three possible alleles. There are therefore six possible genotypes and four phenotypes, as shown in Figure 5.1.3.

Phenotype

When studying inheritance it is important to know the genotypes of parents and offspring. However, it is equally important to know the characteristics that can result from a given genotype, known as the phenotype. The phenotype includes any distinct property of an organism: physical, chemical, physiological or behavioural.

Importantly, environmental conditions can affect the phenotype of certain genotypes. In these cases, the genotype determines the possible range of phenotypes for a particular characteristic, and the environment influences where in that range the actual phenotype will be.

For example, Arctic foxes (Vulpes lagopus) have two genotypes that affect fur colour: white morph' and 'blue morph'. The fur of the blue morph remains dark blue-grey throughout the year, but the fur of the white morph varies from dark brown or grey to pure white. In summer the fur is dark, but as winter approaches the fur gradually changes to white in response to the increasing cold and shorter day length. At the end of winter the fur gradually returns to its summer colour. You will learn more about environmental influences on phenotypes on page 207.

Although the phenotype is relevant to the functioning of an individual organism, the genotype is what is passed on to the next generation.

DOMINANT AND RECESSIVE PHENOTYPES

The relationship between genotype and phenotype gives an insight into an important property of phenotypes known as dominance. For a given gene, you can determine whether a phenotype is completely dominant, co-dominant or recessive by examining the phenotype of the heterozygote compared with that of the homozygote. Dominant phenotypes are expressed if the individual carries at least one allele for the dominant trait. Recessive phenotypes are expressed only if the individual carries two alleles for the recessive trait. It is important to understand that dominance (and recessiveness) are properties of alleles, not genes. They are expressed as dominant or recessive phenotypes. Genes themselves are neither dominant nor recessive.

Complete dominance

To understand complete dominance, it is useful to consider the white eye gene in blowflies. There are two alleles for the white eye gene, Wand w. Individuals with genotype WW have red eyes, while individuals with genotype ww have white eyes. Individuals with genotype Ww do not show an in-between trait such as pink eyes but instead have red eyes, making them indistinguishable from those of the WW genotype.

Blowflies with genotype Ww display the red eye because the W allele makes enough membrane transporter protein to give the eye normal red pigment levels. The red eye colour phenotype is referred to as the dominant phenotype, because it only needs one W allele for that phenotype to be displayed. The white phenotype is referred to as the recessive phenotype because it is not observed in the heterozygote. It needs two copies of the w allele for it to be observed in the phenotype. This example shows that scientists can determine which phenotype is dominant by examining the heterozygote (assuming that a dominant phenotype exists, which is not always true).

By convention, the allele associated with the dominant phenotype is represented by an upper-case symbol (e.g. W).The allele associated with the recessive phenotype is represented by a lower-case symbol (e.g. w).The blowfly’s white eye is an example of complete dominance. Ww individuals have the same eye colour as WW flies.

POLYGENIC INHERITANCE

So far, we have been discussing phenotypes that are monogenic; that is, they are controlled by a single gene. For s0me traits, such as skin colour, eye colour and height in humans, more than one gene contributes to the phenotype of an individual. This is known as polygenic inheritance and results in a much greater range of phenotypes. Polygenic traits in non-human animals include wing shape and bristle count in Drosophila; birth weight, temperament and milking ability in cattle; and plumage and beak size in birds. When the phenotypes of a polygenic trait are shown on a graph, the result is often a bell-shaped curve (typical of continuous variation), which is referred to as a normal distribution.

Height in humans

It has been found that height in humans is controlled by about 50 genes or regions of the genome. Some of these individual genes control secretion of thyroid gland hormones and human growth hormones. A deficiency in the production of these hormones during childhood and puberty can result in stunted growth. On the other hand, too much production can cause excessive growth resulting in exceptional height. The greater the number of genes that control a characteristic, the more possible gene combinations exist and as a result, the wider the range of possible phenotypes.

Human skin colour

The colour of human skin is determined by the amount of melanin (dark pigment) it contains, and there are three types of melanin: eumelanin, pheomelanin and neuromelanin. At least four genes are involved in melanin production. For each gene, one allele codes for melanin production while the other does not. For example, the melanocortin 1 receptor gene (1vf C 1 R) is primarily responsible for determining whether pheomelanin and eumelanin is produced in the human body. The agouti signalling peptide gene (ASIP) inhibits eumelanin production. The tyrosinase gene (TYR) is responsible for making an enzyme called tyrosinase. Tyrosinase converts tyrosine to dopaquinone. A series of additional chemical reactions convert dopaquinone to melanin in the skin. The presence or absence of various alleles of these genes affects the level of melanin production and hence dark skin colour.

Other genetic factors affect skin colour, including the formation of melanocytes (melanin producing cells). The KIT ligand gene (Kh'LG) is involved in the permanent survival, proliferation and migration of melanocytes to the skin surface.

The combination of melanin production (affected by 1vfCJR, ASIT' and TYR) and formation of melanocytes (affected by KITLG) together determines the degree of pigmentation. This results in a wide variation of observable skin tones.

ENVIRONMENTAL INFLUENCES ON PHENOTYPE

The examples discussed so far show that the phenotype is determined by the genotype but may also be affected by the environment. If an individual with a given genotype develops in one environment, its phenotype may be different than if it had developed in some other environment. For example, the average height of humans has gradually increased in the last few hundred years because improvements in nutrition have had a positive overall effect on growth.

Phenylketonuria

The inherited disorder phenylketonuria (PKU) is a consequence of the build-up of an amino acid called phenylalanine in the blood. This is toxic to developing neurons, leading to abnormal development of the nervous system and intellectual disability. PKU is caused by a mutation in the PAH gene, which codes for an enzyme that converts phenylalanine into another amino acid, tyrosine. If an individual inherits two copies of the mutant allele (that is, they are homozygous for the gene), they will develop PKU. Fortunately, development of the symptoms can be prevented by modifying the diet (environment) of babies that test positive for PKU shortly after birth. If homozygous individuals reduce their intake of dietary phenylalanine, particularly during childhood, they show normal brain development. Newborns in Australia are routinely tested for genetic disorders like PKU.

Fur colour in Himalayan rabbits

Fur colour of Himalayan rabbits provides an example of the effect of environmental temperature on phenotype. The Himalayan rabbit is homozygous for a mutant allele that encodes a heat-sensitive tyrosinase. Tyrosinase is produced but it is inactivated at normal body temperature, meaning that no melanin is produced and the rabbit develops a white coat. At low temperatures, tyrosinase is activated and results in the formation of melanin, causing black fur to form. When a small section of fur is shaved from a white region on the back, the fur grows back black if the animal is kept at low temperatures, but white if the animal is kept at high temperatures.

Flower colour in hydrangeas

Hydrangeas are a commonly seen example of environmental effects on phenotype. If cuttings of a single hydrangea plant are grown in very acidic soil (pH 5.5 or less), the flowers produced are blue; if the cuttings are grown in weakly acidic or alkaline soil (pH 6.5 or more), the flowers are pink. The cuttings are of identical genotype, so it must be the environment (the pH of the soil) that affects the phenotype of the hydrangea.

This effect is caused by the relationship between soil pH, a pigment called anthocyanin and the availability of aluminium in the soil for uptake by the plant. At a soil pH of 5.5 or less, aluminium is free to be taken into the plant. Anthocyanin is normally red, but it binds to aluminium in the plant to form a blue pigment called metalloanthocyanin, resulting in blue flowers. At a soil pH of 6 or more the aluminium binds to soil particles and is less available to the plants. This leaves most of the anthocyanin in the plant in its red form, resulting in pink flowers.

Epigenetics

Phenotypes can sometimes be affected by the interaction of DNA with other molecules. For example, the queen and worker honeybees (Apis mellifera) are genetically identical, but their behaviour, physiology and appearance are different. The phenotype differences are due to the differences in the diet of the bees. Queen bees are fed royal jelly while worker bees are fed nectar. Royal jelly contains ingredients that inhibit an enzyme called cytosine methyltransferase.

This enzyme adds a methyl group (-CH3) onto cytosine bases in honeybee DNA, preventing certain genes from being expressed. When the enzyme is inhibited, methylation does not occur and these genes are expressed. When scientists mimicked the effects of royal jelly on worker bees, worker bees exhibited characteristics of queen bees.

This effect of royal jelly in the honeybee is an example of epigenetics. Epigenetics is a term used to describe molecular events, such as adding methyl groups, which occur on DNA without altering the DNA sequence. Such modifications are called 'epigenetic marks' and result in changes in gene expression and variations in phenotype. Other forms of epigenetic modification include addition of methyl or phosphate groups to histones, which affect how DNA is coiled and whether particular genes are expressed. Another example of epigenetics is X-inactivation, where one of the two X chromosomes in female mammals is inactivated during embryonic development. m each cell, X-inactivation randomly switches off one X chromosome to ensure the correct number of genes are expressed and development proceeds as normal.

5.2 Inheritance of genetic variation

Much of what is now understood about natural variation and patterns of inheritance in sexually reproducing organisms was originally gained through the work of Gregor Mendel in the 1860s. Mendel accurately deduced the basic principles of inheritance by studying several inheritable traits in pea plants (Pisum species), using precise experimentation and careful observations over many years.

MENDEL'S STUDY OF PATTERNS OF INHERITANCE

Mendel demonstrated that traits are passed from parents to offspring, and that these traits form specific patterns over generations of crossbreeding.

Mendel made several observations in his pea experiments: purple flowers were dominant over white flowers, a round seed shape was dominant over a wrinkled seed shape, and a green pod colour was dominant over a yellow pod colour. These different phenotypes occur because of variations in genes (alleles).

A cross of the traits being studied can be carried out to determine which trait is dominant. A monohybrid cross is a cross between two individuals with different alleles at a single locus. In the standard monohybrid cross, homozygous parents (P) with different phenotypes of the same trait (e.g. white fur and black fur) are crossed first to produce heterozygous offspring (the first filial generation, or Fl generation). These heterozygous offspring are then crossed with each other to produce the second filial generation (F2 generation). The phenotypic ratios in the offspring of Fl and F2 generations indicate which phenotypes are dominant or recessive. Mendel used monohybrid crosses to discover the dominance relationships of traits in pea plants.

Parent generation

Phomozygous dominant x homozygous recessive

first filial generation heterozygous offspring

second filial generation

PUNNETT SQUARES

In 1905, geneticist Reginald Punnett devised a simple method for showing the random combination of gametes and the genotypes of the resulting offspring. In a Purmett square the alleles of each parent are first written in the top and side cells. Then by going down each column and across each row the alleles are combined and written into the remaining cells.

Punnett squares make it easy to establish all the possible combinations of alleles carried by the gametes and, therefore, all the possible genotypes of the offspring. This is useful in fields such as animal husbandry and horticulture because it allows breeders to select individuals to cross according to the desired traits of the offspring.

Genotypic and phenotypic ratios

Genotypic and phenotypic ratios are used to express the expected frequency of genotypes and phenotypes in the offspring from a genetic cross. Punnett squares are used to calculate the expected outcomes of a cross and the possible genotypes and phenotypes generated in the offspring.

The ratio of genotypes in the offspring is written in the following order: homozygous dominant: heterozygous : homozygous recessive.

The ratio of phenotypes observed in the offspring is written as: dominant phenotype: recessive phenotype.

TI1e genotypic and phenotypic ratios sometimes differ because the dominant or recessive nature of traits means that different genotypes can result in the same phenotype (e.g. both WW and Ww result in red eyes in flies).

The genotypic ratio I : 2 : I of the F2 generation resulting from a monohybrid cross occurs because of the following reasons.

In meiosis, heterozygous (Ww) individuals (both male and female) produce two gametes (a W gamete and a w gamete).This is because of the separation of pairs of alleles during the formation of reproductive cells.

Fertilisation occurs at random. For example, a W sperm has equal chance of fertilising a W egg or a w egg, because these eggs are produced in equal frequency. A w sperm also has an equal chance of fertilising a W egg or a w egg. So the four equally possible genotypic outcomes are WW, U1/w, wW, ww.

The Punnett square accounts for both of these factors in demonstrating the possible outcomes of the cross.

AUTOSOMAL DOMINANT INHERITANCE

Autosomal dominant inheritance (complete dominance) refers to a dominant trait that is passed on to offspring via an autosomal gene. An autosome is any chromosome that is not a sex chromosome. Only one copy of an autosomal allele (i.e. from one parent) is needed to express a dominant phenotype.

Parent (P) generation

The inheritance of eye colour in the Australian sheep blowfly is an example of a single gene with two alleles (found on an autosomal chromosome) coding for the trait. In the blowy, red eye colour is dominant over white eye colour. The homozygous genotypes are WW and ww. Homozygous genotypes produce only one type of gamete. WW individuals produce only W gametes and ww individuals produce only w gametes.

In a cross between two red-eye homozygous (WW) individuals, all the offspring would be homozygous WW (red eye). As long as WW individuals were crossed together, it would be a true-breeding strain. True-breeding (also called pure­breeding) crosses produce offspring with the same trait as the parents. Similarly, as you can see on the right side of Figure 5.2.3, crosses between two homozygous white-eye (ww) individuals would yield a true-breeding white eye (ww) strain.

The Fl generation

To test the principle of dominance, two true-breeding parents with two different traits can be crossed.

In the blowfly example, two true-breeding strains (one with red eyes, WW, and one with white eyes, ww) can be crossed to produce an Fl generation. The results of the cross can be shown in a Punnett square, as shown in Figure 5.2.4.

Each of the offspring in the F1generation has the heterozygous genotype Ww. The phenotype resulting from this genotype is red eyes. From this, it can be deduced that the red-eye phenotype is dominant over the white-eye phenotype.

The F2 generation

The F2 generation is the result of crossing the individuals from the F1 generation. In this example, half of the gametes produced by an F1 individual (Ww), will be W and half will be w. So three different combinations of alleles are possible in the F2 generation: WW, Ww and ww, as shown by the Punnett square in Figure 5.2.5.

In the F2 generation the dominant phenotype is likely to occur in three out of four crossings, and the recessive phenotype only once.

The Punnett square shows that the F2 genotypic ratio is: WW: Ww : Ww : ww or i WW: 2 Ww : 1 ww. Because red eye colour is dominant over white eye colour, the F2 phenotypic ratio is:

3 red-eyed flies (WW: Wr: Wr) : l white-eyed fly (ww)

From this information it can be determined that the wild type is red eyed. The wild type is the typical or most common form of a trait in a natural population.

TEST CROSSES

It is not immediately obvious whether an individual with a dominant phenotype is homozygous, because it might be either AA or Aa. Apart from sequencing the gene involved, the only way to determine this is to do a test cross. A test cross involves crossing an individual with the dominant phenotype (heterozygous or homozygous) with an individual with the recessive phenotype (homozygous). Homozygous individuals produce gametes with one type of allele, whereas heterozygous individuals can produce gametes with two types of alleles.

If the offspring from the test cross all have the dominant phenotype, then both the parents are likely to be homozygous (although it is not possible to be certain because of the random nature of fertilisation). If the offspring have both dominant and recessive phenotypes, then the parent with the dominant phenotype must also carry a recessive allele and is therefore heterozygous.

Coat colour in guinea pigs

The coat colour of guinea pigs is determined by the alleles of one gene, and black fur is the dominant phenotype. If a true-breeding white guinea pig (bb) is crossed with a true-breeding black guinea pig (BB), the resulting F1 has black fur (Bb). But if the genetic history of a black guinea pig is unkn0\\'11, its genotype can be determined by crossing the black guinea pig with a white-coated guinea pig, which must be bb (homozygous recessive).

Figure 5.2.6 illustrates the test cross that would be carried out. Of the resulting offspring in this example, half are white and half are black. This ratio of about 1 : 1 is consistent with the results of a heterozygote crossed with a homozygote if the trait is determined by the alleles of one gene and one trait is dominant. So the black guinea pig is likely to be heterozygous. If all the offspring of the test cross were black-coated (all Bb), the Fq guinea pig in question would have been shown to be homozygous.

The predicted outcome for a cross between heterozygote black guinea pigs is 1 black (Bb): white (bb). However, as the diagram shows, the resulting ratio of the test crosses was 27 black (Bb): 23 white (bb) rather than, for example, 23 : 23 which is equal to a 1: l ratio). The difference between predicted and observed ratios is due to chance.

AUTOSOMAL CO-DOMINANT INHERITANCE

Some traits do not show simple dominance or recessiveness. TI1ese are instances in which both alleles are expressed to varying degrees in the phenotype of heterozygous individuals. This is called co-dominance. Sometimes neither phenotype is completely dominant, so that intermediate phenotypes occur. This is known as incomplete dominance.

Autosomal co-dominance and incomplete dominance

Flower colour in snapdragons is a trait showing incomplete dominance. For example, snapdragons can have a red flower phenotype (R1R1) or a white flower phenotype (R2R2). In this case upper-case letters and subscripts are used to distinguish the alleles because neither phenotype is completely dominant. Plants of the R1R2 genotype have pink flowers. In R1R1 flowers, both copies of the R1 allele produce an enzyme required to produce red pigment. In R2R2 flowers, no pigment is produced because the R2 allele produces no enzyme or a defective enzyme.

Since the R1R2 flower has one R1 allele (which produces the active enzyme) and one R2 allele (which does not), it will produce half the amount of pigment as the R R1 flower, and will be pink in colour.

Crossing red-flowering snapdragons (R1 genotype) with white-flowering snapdragons (R2R2 genotype) will yield an Fl generation in which all individuals have the genotype R1R2 and are pink-flowering.

The heterozygote pink-flowering snapdragon (R1R2) can be distinguished from the two homozygotes, red R1R1 and white R2R2, due to the co-dominance of both the red and white alleles resulting in a pink-flowering phenotype.

Genotype ratio: 1 R1R1 : 2 R1R2: 1 R2R2 Phenotype ratio: 1 white : 2 pink : 1 red

This phenotypic ratio of 1 : 2 : 1 (Figure 5.2.8) is different to the 3 : 1 ratio of two phenotypes observed in complete dominance.

ABO blood grouping

As described in Section 5.1 human blood type is an example of autosomal co­dominant inheritance. In th.is case, depending on the alleles inherited, the expression of the genotype differs. There are three alleles for blood type at the same locus, and individuals can have A, B, AB or O phenotypes. This is illustrated in Figure 5 .1.3. Those with the less common AB blood group are heterozygotes carrying one allele that produces an A antigen and one allele that produces a B antigen.

Because both the A and B antigens are present on the surface of red blood cells, which can be detected using antibodies, neither phenotype is fully dominant. This is another example of co-dominance. So A and B phenotypes are co-dominant, while the O phenotype is recessive.

Multiple alleles at a single locus

Blood group systems, provide a demonstration of the effects of multiple alleles at the same locus. In this case, the three alleles are represented as IA, 18 and i. JA codes for the A antigen, 18 codes for the B antigen while i does not produce either antigen. Therefore the effects of IA and [8 dominate over i. Each person carries copies of one or two of these three possible alleles.

The possible genotypes and phenotypes of the offspring of a parent with blood type O and a parent with blood type AB can be determined using a Punnett square.

The F1 generation in this example would be either blood type A or B, but all would be heterozygous.

If a heterozygous individual for blood type A and a heterozygous individual for blood type B were to have children, all four combinations of blood type are possible: AB, A, B and 0.

The important principle illustrated by this example is that phenotypes are not always dominant or recessive. The dominance of a phenotype is always in relation to another phenotype. Thus, phenotype A is co-dominant with B, but dominant to O.

SEX-LINKED INHERITANCE

So far you have examined the inheritance of genes located on autosomes. However, the patterns of inheritance are not the same for genes located on either of the two sex chromosomes. Phenotypes inherited through genes on sex chromosomes are said to be 'sex-linked' and they show sex-linked inheritance. It is important to remember that sex chromosomes also carry other genes which are not related to sex determination.

Figure 5.2.9 shows how sex chromosomes are transferred to the offspring, with an equal probability of the offspring being female or male. The XY system determines sex in humans, most other mammals, some insects and some plants. In this system, females are homogametic (XX) and males are heterogametic (XY). The female passes one X chromosome on to her offspring, while the male can pass on either an X or Y chromosome; an X chromosome produces female offspring and a Y chromosome produces male offspring. It is therefore the father's genetic contribution that determines the sex of the offspring. In birds, some fish, some insects and some reptiles, sex is determined by ZW chromosomes. In this system females are the heterogametic sex (ZW) and males are the homogametic sex (ZZ).

X-linked recessive inheritance

In humans, X-linked recessive traits are predominantly expressed in males, because males carry only one X chromosome. Females carrying an X-linked recessive allele might not express the trait, or show only mild expression. This is because the second X chromosome that females carry could mask the recessive trait. The probability in humans of a female carrying two X-linked recessive alleles is very low.

The pattern of sex-linked inheritance is evident when a reciprocal cross is performed. This is a cross used to investigate the role of parental sex on the inheritance of genotypes. Two crosses are performed: one crossing a male with the trait of interest with a female not expressing the trait (usually homozygous wild type), and another crossing a female with the trait of interest (homozygous) with a male that does not express the trait (usually wild type). If the trait is sex-linked (carried on the X chromosome), the phenotypic ratios of the male and female offspring will be different.

Paralysis in Drosophila

The temperature-sensitive paralytic gene, named after the mutant phenotype, is on the X chromosome of the fruit fly (D. melanogaster). A mutant phenotype is one that is different to the normal wild type and arises from a genetic mutation. Individuals with the mutant allele are paralysed when incubated to a temperature of 29°C, whereas wild type flies show normal behaviour at this temperature. The paralytic phenotype is recessive to wild type. The wild type (for this trait) is defined as normal movement at an ambient temperature of 29°C, that is, a lack of paralysis.

The alleles are defined differently for sex-linked traits. An X is used to indicate that the trait is carried on me X chromosome, and the allele is written in superscript next to the X. In me example of the fruit flies, the alleles can be written as:

XP = wild type

XP = paralysis

As the paralytic phenotype is recessive, females that are homozygous for the mutant allele (xt' A,p) express me mutant paralysis phenotype; females that are homozygous dominant (Xpx") and females that are heterozygous (x"'xt') are both wild type phenotype.

As males have only one X chromosome there are only two male genotypes: xt'Y males are paralytic and x'y males are wild type.

If paralytic females (xt' xt') are crossed with wild type males (x'Y), all of the F1 male offspring will be paralytic (XP'y) and all of the F1 female offspring will be wild type phenotype (Xpxt'). This pattern of transmission of the mutant phenotype from the female parent to male offspring is characteristic of X-linked recessive inheritance.

The reciprocal (reverse) cross, shown in Figure 5.2, produces a different outcome. If a wild type homozygous female (x' X') is crossed with a paralytic male (XPY) all of the offspring (male and female) are wild type phenotype (X i and .xPY).

These different outcomes of the reciprocal crosses are characteristic of X-linked recessive inheritance. In contrast, reciprocal crosses give the same outcome for autosomally inherited genes.

Haemophilia in the British royal family

Figure 5.2.11 shows part of the family tree of the British royal family, including Queen Victoria, whose eighth child Leopold was born with haemophilia. Haemophilia is a blood disorder in which blood clotting is slow, resulting in excessive bleeding.

It results from a mutation in a gene on the X chromosome that is involved in the production of a blood-clotting protein that controls bleeding.

The incidence of haemophilia in the descendants of Queen Victoria shows the hallmarks of X-linked recessive inheritance. All of the haemophiliacs shown in the tree are male. The female carriers of the disease are heterozygous, carrying one haemophiliac allele and one normal allele. Given that the haemophilia phenotype is recessive, carrier females are phenotypically normal. However, because females produce eggs carrying the normal and haemophiliac alleles with equal frequency, and males receive their single X chromosome from the egg, there is a 50% chance that the son of a carrier will have haemophilia.

Through marriage, some of Victoria's phenotypically unaffected daughters who carried this mutation spread haemophilia to other royal families in Europe. For example, Irene of Hesse transmitted the haemophilia allele to her eldest and youngest sons Alexandra and Henry, and the normal allele to her other son, Sigismund. This form of haemophilia occurs at a frequency of one in 10000 males and one in 100 million females.

In general, X-linked recessive disorders occur at much higher frequencies in males than females because females need to inherit a copy of the allele from both parents to be affected (that is, the mother must be a carrier and the father must be affected by the disorder). Males, however, need only inherit one copy of the X-linked allele from their carrier mother.

X-linked dominant inheritance

X-linked disorders may also display a dominant phenotype. Consider the inheritance of vitamin D-resistant rickets disorder, which causes bone deformities. This is shown in the pedigree chart in Figure 5.2.14. The mother of the first generation is heterozygous and affected by the condition. Her children had a 50% chance of having vitamin D-resistant rickets, regardless of whether they were male or female.

When a father is affected and a mother is normal (as in the second generation), all female offspring will show the condition, and all male offspring will be normal. This is because female children all receive an X chromosome, which carries the allele for the disease, from their father.

The alleles for this trait could be shown as: x° = vitamin D-resistant (rickets) allele x1 = normal allele.

TI1e following Punnett square shows the pattern of inheritance of offspring of a heterozygous female affected by the vitamin D-resistant rickets allele and a male with the normal allele.

The possible genotypes of the offspring are:

xDx1: x1x1: xDY: x1Y<1: 1: 1: 1)

The possible phenotypes are therefore:

female with vitamin D-resistant rickets : normal female : male with vitamin D-resistant rickets : normal male ( 1 : 1 : 1 : 1).

The following Punnett square shows the pattern of inheritance in the offspring of a homozygous unaffected female and a male with vitamin D-resistant rickets.

The possible genotypes of the offspring are:

XDJ<1: J<1y (1 : 1)

The possible phenotypes are therefore:

All females have vitamin D-resistant rickets: all males are unaffected ( 1 : 1).

Y-linked inheritance

Compared to the X chromosome, the Y chromosome has few genes: it has only about 72 protein coding genes, compared to 800-900 on the X chromosome. Most of these genes are involved in male sex determination and fertility. Therefore there are far fewer Y-linked traits than X-linked traits.

If a trait is passed from father to son and never observed in fe111ales, it is likely to be Y-linked, meaning the gene for that trait is on the Y chromosome. Until recently it was thought that hairy ears were controlled by a Y-linked gene, but recent studies suggest there are also autosomal genes involved in the trait.

Sex-limited inheritance

The Y-linked pattern of inheritance is sometimes confused with sex-limited inheritance. Sex-limited traits can only occur in one sex because the feature affected is unique to that sex. Therefore, males and females have different phenotypes. For example, complete androgen insensitivity syndrome, in which the fetus is unresponsive to male hormones, can only occur in males, because only males carry the Y chromosome. This means that even if females have the genotype for this syndrome, they cannot express the condition.

PEDIGREE ANALYSIS

Studying the patterns of inheritance in humans has its challenges, but patterns of inheritance of alleles across generations of families can be analysed using pedigree charts.

Pedigree analysis can be used to follow the inheritance of traits through a family over a number of generations. Given sufficient data, the likely mode of inheritance can be determined (e.g. dominance patterns and whether inheritance is autosomal or sex-linked). When it comes to studying the genetics of humans, pedigree analysis is often the only method available for the following reasons:

Experimental crosses cannot be set up as required.

The environment in which humans live cannot be controlled experimentally.

There are strict legal and ethical laws concerning human experimentation.

Humans tend not to have large families, so there are rarely large numbers of offspring to score.

Each generation of humans takes many years to reach sexual maturity and produce offspring.

Some of the unique problems of human genetic analysis are being overcome as a result of the Hu man Genome Project (HGP). Knowledge of the mechanisms of inheritance in humans and other organisms continues to advance through research in model genetic organisms, such as the bacterium Esiherichia colz􀀅 fruit fly D. ntelanogaster, yeast Saccharonzyces cerevisiae, mouse Mus inusculus, nematode Caenorhabditis elegans and the plant Arabidopsis thalia.na.

In the meantime, studying existing families can assist in tracing inheritance of traits. Pedigree analysis is a technique involving studying a family tree for the occurrence of a particular character or trait in one family over a number of generations. In practice it may be necessary to combine information gained from the pedigree data of several families to determine the most likely mode of inheritance of a particular character.

Pedigrees can be used to determine the pattern of inheritance of particular alleles, as well as the presence of particular alleles within a family and the chances of the allele occurring in offspring.

Pedigree charts

Pedigree analysis makes use of pedigree charts to track and organise data. When analysing a pedigree chart, key features in the pattern of inheritance can be used to distinguish between one type of inheritance and another.

Symbols and conventions used for pedigrees

Pedigree charts use a number of standard symbols and conventions. The main ones you need to know are described below.

Circles represent females and squares represent males.

Shapes are shaded or unshaded to represent the presence of a phenotype for a particular trait.

A horizontal line represents a cross between the individuals.

A vertical line represents a link from parents to offspring.

Individuals are numbered from left to right (if required).

Generations are represented with Roman numerals (if required), with the first generation in the pedigree being generation I.

A carrier of an autosomal trait is shown with a half-filled symbol. A carrier of an X-linked recessive trait is shown with a dot in the centre of the symbol.

RECOGNISING INHERITANCE PATTERNS IN PEDIGREES

Pedigree analysis can be used to understand the way in which particular genetic traits are inherited, but as you will have learnt by now, there are many possible modes of inheritance. Fortunately, these can be recognised if you are aware of a few simple methods. Once you know these methods, you should be able to examine a comprehensive pedigree and then be able to work out the inheritance pattern for a trait.

Autosomal inheritance

When the inheritance of a trait is just as likely in males as in females, it is an autosomal trait-that is, the gene(s) associated with the trait are not located on the sex chromosomes, but on the autosomes. This is not always immediately obvious, particularly if a mating produces only male or only female offspring.

Autosomal recessive inheritance

Autosomal recessive inheritance of a trait is likely if two parents do not have a particular phenotype but one or more of their offspring does. Figure 5.2.18 shows the inheritance pattern of a particular trait in a population of unisexual plants (so plants are either male or female). Shaded individuals are affected (that is, they express the trait), and unshaded individuals are not affected.

For this exercise it may be assumed that the inheritance of the trait is not sex-linked. Figure 5.2.18 shows that: the cross between individuals II-3 and II-4 resulted in three offspring individuals II-3 and II-4 are unaffected offspring III-1 is female and unaffected, III-2 is male and unaffected, and III-3 is female and affected.

'CT1e parents both contribute one allele each for the trait to ill-3, so both parents must carry the allele responsible for the trait. Since the parents are both unaffected, both must be heterozygous. The trait is therefore autosomal recessive.

Another indicator of autosomal recessive inheritance is that the trait skips generations (that is, it does not appear in every generation). However, not skipping a generation does not rule out autosomal recessive inheritance.

Once the form of inheritance is determined, it is possible to work out the genotypes of the individuals in the pedigree. First, a symbol should be allocated for the two alleles. In this particular example:

P can represent the allele for purple flower (dominant trait)

P can represent the allele for white flower (recessive trait).

So III-3 (white flowers) must be pp, both parents must be heterozygous (Pp) and individuals III-1 and III-2 must be heterozygous (Pp) or homozygous (PP).

Autosomal dominant inheritance

Autosomal dominant inheritance of a trait is likely if both parents show the trait but one or more of their offspring do not show the trait. The pedigree in Figure 5.2.20 shows the inheritance pattern of round (smooth) and wrinkled pea shape in pea plants. The shaded individuals represent round peas and the unshaded individuals represent wrinkled peas.

Again, it may be assumed for this example that the trait is not sex-linked. Consider the section of the chart highlighted by the square. This shows that: II-3 and II-4 produce three offspring both Il-3 and Il-4 are affected offspring III-1 is female and unaffected, and III-2 and 111-3 are male and affected. Because both Il-3 and ll-4 contribute one allele each to Ill-1, each must carry the allele for the unaffected phenotype. Since each parent carries the wrinkled allele, they must both be heterozygous. As they are heterozygous and show the round phenotype, their phenotype (round) must be dominant.

Other indicators of autosomal dominant inheritance are that the trait may be seen in all generations, and individuals showing the trait must have at least one parent showing the trait. Examples of autosomal dominant inheritance in humans include Huntington's disease and neurofibromatosis type 1. Examples in other animals include the hornless (polled) trait in cattle.

Sex-linked inheritance

When an inheritance pattern is 'sex-linked', that means the trait in question affects males and females differently. We therefore conclude that one or more of the gene(s) responsible for the trait is located on a sex chromosome. If sufficient male and female offspring are produced in each generation, sex-linked inheritance should be obvious when looking at the pedigree.

Distinguishing between autosomal and sex-linked inheritance

m the previous examples of autosomal inheritance it was assumed that inheritance 1was autosomal. However, when investigating inheritance it is important to check whether the inheritance might be sex-linked, because similar patterns can occur in both types.

The inheritance pattern of colour blindness in humans (Figure 5.2.23) can be examined by studying the pedigree of a family in which some individuals are colour blind. By convention, if a mating partner is not shown in the pedigree, he or she is not affected by the phenotype (in this case, not colourblind).

Differences in the incidence of the trait between males and females, and the frequency of the trait across generations, are two inheritance patterns that help determine whether a trait is sex-linked or autosomal.

In the pedigree shown in Figure 5.2.24 only the males are colour blind. 1'his demonstrates that the colour blind trait is not dominant and is potentially sex-linked (X-linked). Y-linked alleles affect only males and are very rare, and the phenotype will be present in every male offspring. Because of these factors, colour blindness cannot be a Y-linked trait.

X-linked recessive inheritance

The pedigree in Figure 5 .2.24 also indicates that the inheritance of colour blindness is more likely to be X-linked recessive than autosomal recessive, because X-linked recessive traits affect more males than females. This is because males only inherit one X chromosome, while females inherit two. This second X chromosome has a masking effect on recessive alleles, resulting in females carrying the affected allele but not expressing the phenotype. In order for females to be affected by X-linked recessive traits, they must carry two copies of the allele, one on each X chromosome.

Given the low number of offspring, it is possible that all the colourblind individuals are males purely by chance. However, in reality, many such pedigrees have been studied and show similar patterns, confirming that the inheritance of colour blindness is X-linked recessive.

X-linked dominant inheritance

Traits that are X-linked dominant are rare and affect more females than males. This is because females inherit two X chromosomes and so have twice the chance of inheriting an affected X chromosome compared to males, who inherit only one.

Evidence of X-linked dominant inheritance is seen in a pedigree in which affected males have daughters who are all affected and sons who are not affected. This is because daughters inherit their father's only X chromosome, while sons inherit their father's Y chromosome. If the X chromosome carries an allele for a dominant trait, then the daughter will express its phenotype. X-linked dominant inheritance can be seen in Figure 5.2.25.

Males I-1, III-7 and ill-14 have daughters who are all affected and sons who are unaffected.

Because females pass on one of their two X chromosomes to both their daughters and sons, there is a 50% chance that they will pass on an X-linked dominant trait to their offspring. This pattern of inheritance is seen in female individuals II-2, II-4 and II-8, who have both affected and unaffected daughters and sons. An example of X-linked dominant inheritance in humans is Rett syndrome (RT T), a rare genetic neurological disorder of the grey matter of the brain. Another example is vitamin D-resistant rickets.

Y-linked inheritance

Male offspring inherit their father's Y chromosome, so any alleles carried on this chromosome will be passed on from father to son. The phenotype of Y-linked disorders is therefore only seen in fathers and all their sons. Females are never affected by Y-linked traits because they do not inherit a Y chromosome. As there is only one Y chromosome (hemizygous), and thus only one allele present, the general principles of dominant and recessive inheritance do not apply.

A Y-linked trait is likely if:

•only males are affected

•all male offspring are affected

•the trait is observed in every generation in which males are born.

This pattern of inheritance can be seen in Figure 5.2.27. Because the Y chromosome has far fewer genes that encode for proteins compared to the X chromosome, Y-linked inheritance of disorders is relatively rare.

Ruling out sex-linked inheritance

Consider the following pedigree for a trait in humans. It is possible to rule out sex­linked inheritance in this pedigree (Figure 5.2.28) by looking for a pattern that will rule out each type of sex-linked inheritance in turn. (If no such pattern can be found, then that type of inheritance cannot be ruled out.)

Ruling out X-linked recessive inheritance

In order for a trait to be X-linked recessive, affected mothers must have affected sons. In Figures 5.2.28 and 5.2.29, individual II-8 has two sons, one affected by the trait and the other unaffected. For the trait to be X-linked recessive, the mother would have to carry the allele on both her X chromosomes (x'1x'1) to be affected. The mother contributes one of these chromosomes to her offspring, so both sons would have to receive a chromosome with .the affected allele. But one son does not show the trait (and therefore did not receive the affected allele), so X-linked recessive inheritance cannot be involved.

Ruling out X-linked dominant inheritance

For X-linked dominant inheritance to be involved, every daughter of an affected male must be affected. This is because the daughters must receive an X chromosome £rom their father, and the father has only one X chromosome. In the pedigree shown in Figure 5.2.30, male I-1 has the trait but one of his daughters (II-6) does not. X-linked dominant inheritance therefore cannot be involved in this pedigree.

Ruling out Y-linked inheritance

If a trait is Y-linked, only males are affected (because females lack a Y chromosome) and affected fathers pass the trait on to all their sons. In the pedigree shown in Figure 5.2.31, some females have the trait, and not all fathers with the trait passed it on to their sons (e.g. male I-1 and son II-3; also II-1 and III-2).Y-linked inheritance is therefore not involved in this pedigree.

After ruling out X-linked recessive, X-linked dominant and Y-linked inheritance, it can be concluded that the trait shown in the previous figures must be the result of autosomal inheritance. Although the trait is observed in every generation, which often indicates dominant inheritance, in this case the trait could be autosomal dominant or autosomal recessive inheritance. Further information would be needed to determine which is involved in this pedigree.

STEPS IN PEDIGREE ANALYSIS

A pedigree analysis should be carried out in a methodical series of steps to determine the pattern of inheritance. These steps are outlined below and illustrated in Figure 5.2.32.

Step 1: Determine whether the condition is sex-linked.

a. Are mostly males affected? If only males are affected, and the trait passes from father to son in every generation, inheritance is most likely Y-linked.

b. Do affected daughters have an affected father?

c. Do affected mothers have affected sons but not affected daughters? If conditions in (b) or (c) are met, inheritance is most likely X-linked recessive.

d. If you answered 'no' to either question in (b) and (c), go to step 2.

Step 2: Look for two affected/unaffected parents that have a child with a different phenotype.

a. If two unaffected parents have an affected offspring, inheritance is autosomal recessive.

b. If two affected parents have an unaffected offspring, inheritance is autosomal dominant.

INDEPENDENT ASSORTMENT

Mendel’s second law of inheritance, the **Law of Independent Assortment** <*Semiotic: idea: theory, linguistically defined: trained gaze*>, states that the alleles of a gene controlling one trait assort independently of alleles of another gene controlling a different trait. This can be illustrated by considering crosses involving two genes that affect two distinct traits.

You can cross true-breeding strains of fruit flies (D. melanogaster) that differ for two traits: eye colour and body colour and then conduct a dihybrid cross ('di' meaning two). The two traits in this example are eye colour and body colour in D. melanogaster.

The eye-colour gene in this example is the yellow eye gene. (This is a different gene from the white eye-colour gene discussed earlier, and is located on a different chromosome). The 'yellow eye' gene is autosomal; the alleles are Y and y.

The wild type 'red eye' phenotype (genotypes YY and Yy) is dominant and the 'yellow eye' phenotype (genotype yy) is recessive.

The second trait is body colour and the gene in this example is called 'brown body'. It is an autosomal gene with (two alleles B and b. The wild type 'green body' phenotype (genotypes BB and Bb) is dominant and the 'brown body' phenotype (genotype bb) is recessive.

The eye-colour and body-colour genes are on different chromosomes.

LINKED GENES

Mendel's Law of Segregation and Law of Independent Assortment are the cornerstones upon which our current understanding of inheritance is built. Scientists have continued to refine and extend these Laws or principles to explain new and unexpected aspects of heredity, and more complex patterns of inheritance.

Although many traits are inherited in accordance with Mendel’s Law, this is not always the case. The exceptions occur when two or more genes are located on a single chromosome and are inherited together. This is known as linkage, and is another key principle of inheritance. The closer the genes are, the more likely they are to be inherited together, or 'linked'. But linkage is never complete because of crossing over, which occurs during meiosis.

Linkage and recombination

Crossing over is a normal event that results in genetic exchange between non­sister chromatids. It will occur in most germline cells going through meiosis. The probability of at least one cross-over event occurring somewhere on the chromosome is high because there is usually at least one chiasma (point of crossing over between chromosomes; plural: chiasmata) for each homologous pair in meiosis.

If the A and B loci are very close together, the probability of a random cross-over event occurring between them (outcome 2) is very low.

If genes are close together, there will be fewer recombinant gametes and more parental gametes produced (outcome l). The closer the two genes are together, the rarer the recombinant gametes will be.

If genes are so far apart (on the same chromosome) that close to 50% of the gametes are recombinants, then independent assortment is observed. If the percentage of recombinant gametes is less than 50%, the two genes are considered to be linked.

Recombination and distance between linked genes

By measuring the percentage of recombinant gametes produced by an F1 heterozygote when genes are linked, it is possible to estimate the distance between the two genes. The farther apart two genes are on the chromosome, the more frequently crossing over will occur and the higher the observed percentage of recombination. By repeating such measurements for different pairs of genes, the position of any identifiable gene on a particular chromosome can be found. This process is called gene mapping.

Making use of gene linkage

An important consequence of linkage is that very different traits can be inherited together. This can be extremely important in terms of understanding human health, and in plant and animal breeding.

Information about one locus provides us with the likely genotype at the other locus. When discussing gene mapping, the term genetic marker refers to a sequence of DNA with a known location on a chromosome (it is important to note that in other contexts, such as population genetics, the location of a genetic marker might be unknown). If a gene of interest is closely linked to a genetic marker, then this can be used to determine if someone carries a mutation (a change in DNA sequence). The closer two loci are, the greater the linkage and the more precisely a genotype can be used at one locus to predict the other.

Molecular techniques and genetic markers play an important role in this area. Linkage relationships are becoming increasingly important in providing information about the likelihood that a child will have a particular disorder. A marker locus closely linked with the gene that causes a disorder can be extremely useful in determining the genotype of a parent who has the disorder. If a child has the same marker as the parent with the disease, the child is also likely to have the disorder.

5.3 Genetic variation in populations

Now that you have learnt how genetic variation is inherited in related groups, you can begin to predict the patterns of genetic variation in large groups of unrelated individuals. In this section, you will learn how to model the behaviour of alleles in populations.

ALLELE FREQUENCIES IN A POPULATION

When a gene has different alleles in a population, it is referred to as polymorphic. These different alleles are more common in some populations than others. The rate at which alleles occur in a population is known as the allele frequency. An allele that occurs in 30% of people in a population would have therefore an allele frequency of 0.3. An allele that has a frequency of 1.0 in a population (that is, it occurs in every single individual in that population) is referred to as a fixed allele. In that population, the allele is considered monomorphic. All allele frequencies in a population must add up to 1.0.

The allele frequencies in a population can be estimated by examining phenotypes. When discussing genes with only two alleles, the alleles are represented asp and q.

Differences between populations

When studying the genetics of a population, it is important to understand what is meant by population. The term 'population' often refers to the inhabitants of a particular place, but in biology, it is used to refer to any group of interbreeding organisms. Genetics can be used to estimate where one population ends and another begins by determining if there is gene flow between the populations. Gene flow is the exchange of genes between populations (e.g. via migration and interbreeding).

One way to do this is by comparing allele frequencies of different populations. Imagine two populations of snapdragons. If the two populations are exchanging genes/interbreeding then their allele frequencies should be very similar (i.e. in equilibrium). Note that this does not mean that their phenotypic ratios will be the same. If the two populations are not exchanging genes/interbreeding the allele frequencies will be different (i.e. not in equilibrium).

If there is a high level of gene flow between populations they may be considered a single interbreeding population.

If there is not much gene flow between populations, they may be considered separate gene pools or populations.

In some cases, where multiple genes are examined, there may be enough evidence to conclude that the populations are even different species.

Of course, examining a single gene does not provide much information. The results could be due to random chance, as fertilisation success is subject to chance. To get a statistically significant result, you need samples with many individuals and many different genes. Then you can run a more robust statistical analysis, to see whether the allele frequencies in these populations are significantly different from one another. If they are, this would indicate that something is restricting the flow of genes between these populations. Tins could be distance, a physical barrier such as a mountain range, or even that the populations have overlapping distributions but can no longer interbreed.

GENETIC MARKERS

A genetic marker is any piece of DNA that can be reliably analysed using sequencing or genotyping. Genetic markers can be in coding regions (exons) or non­coding regions (introns). The location of a genetic marker in the genome is usually known but sometimes it is not. When the location of a genetic marker is not known, it is referred to as anonymous DNA. Although the location and fw1ction of anonymous DNA is unknown, sequences of this DNA can provide valuable information about the genetic variation within and between populations. Such genetic markers provide records of change and insight into evolutionary relationships.

Although you do not need to know where a genetic marker comes from in order for it to be useful, you cannot just use any genetic marker for population genetic research. An ideal genetic marker has the following properties.

1 It is polymorphic within the population of interest. That is, it must show variation. If a genetic marker is the same for all individuals, it will not provide any information.

2 It is usually a neutral marker, meaning that it is not currently under selection.

This is so that probability-based models such as HWE can be applied. Probability assumes a level of random chance, whereas selection is non-random. There are situations where a marker that is under selection can be very useful in distinguishing populations, but they are difficult to analyse because of that selection pressure.

3 It is easy and affordable to work with (practical consideration). Some genetic markers are very expensive to isolate and analyse from the organism; some are more reliable while others are difficult to work with. Many population genetics projects have a focus on conservation and there is often a limited budget.

Sequence data

The most direct way to examine genetic variation is to examine the DNA sequence. It is common in population genetics to look at mitochondrial DNA (mtDNA), rather than DNA from the nuclear genome with its multiple chromosomes. Mitochondrial DNA is only inherited maternally (from the mother) in most species, and therefore each sequence will only have one allele. In a haploid genome such as the mitochondrial genome, an allele is referred to as a haplotype. MtDNA changes more rapidly than most nuclear DNA, meaning that differences in populations will be easier to see. A single DNA sequence from a useful genetic marker will have multiple points of difference in a population, rather than just one. We can then use these differences to work out which individuals are more closely related to one another, and how these populations have changed over time.

Single nucleotide polymorphisms (SNPs)

Single nucleotide polymorphisms (SNPs), pronounced 'snips', are another way of examining genetic variation. These are single base changes, but unlike the examination of a single DNA sequence, these are scattered throughout the genome across multiple chromosomes. This means that many points of variation can be examined simultaneously, sometimes thousands at a time, providing a snapshot of genetic variation. Development of SNPs-that is, finding out what parts of the genome vary and ho¼' they can best be isolated-can be technically challenging and expensive (Figure 5.3.3). SNJ>s usually have two alleles per marker.

Short tandem repeats (STRs)

Another way of looking at genetic variation is to use anonymous markers called short tandem repeats (STRs) (also known as microsatellites). Instead of directly sequencing the DNA, each STR marker is examined for size differences, which is much cheaper and easier than sequencing a large number of individuals.

STRs are usually non-coding pieces of DNA that contain a string of repeating nucleotides (e.g. 'AGAGAG' or 'TGAl􀀦fGATfGAT'). STRs usually have repeats of two nucleotides (dinucleotide, e.g. AG), three nucleotides (trinucleotide, e.g. GC1') or four nucleotides (tetra nucleotide, e.g. TGA1'). An STR with three repeats of'TGAT' will be shorter in length (have fewer nucleotides) than an STR with seven repeats of 'TGAT'. The number of repeats in an STR varies between individuals in a population. For example, one individual may have 10 repeats of ATG and another individual may have 14 repeats of ATG for the same STR.

The number of repeats in an STR can also vary within an individual. Individuals have two copies of every STR (one inherited from each parent) because the same DNA sequence occurs on each member of a homologous pair of chromosomes. If the number of repeats for the STR is the same on each chromosome, the individual is homozygous for that STR. If the number of repeats for the STR is different on each chromosome, the individual is heterozygous for that STR.

It is this variation within and between individuals that make STRs useful for population genetic studies and identifying individuals in paternity tests or crime investigations. Unlike SNPs, which usually only have two alleles, STRs are usually highly variable and have many different repeat variations (alleles) within a population. Therefore fewer STRs are needed (compared to SNPs) to get the same amount of information.

Chapter 6 Inheritance patterns in a population

6.1 DNA sequencing and profiling

Most cells contain the entire complement of an organism's DNA (deoxyribonucleic acid), known as its genome, which in humans includes thousands of genes. Today biologists can isolate fragments of DNA and study a single gene or determine the base sequence of an entire genome. DNA sequencing and profiling are powerful technologies that use DNA manipulation techniques. In this section you will learn about these techniques and how they are used to determine inheritance patterns in a population.

GENETIC TESTING

Genetic testing is a medical test used to detect specific alleles, mutations, genotypes or karyotypes that are associated with heritable traits, diseases or predispositions to diseases. It may also be used to determine parentage or ancestry and has become an important part of forensic analysis.

There are many kinds of tests, but the methodologies fall into three main categories-molecular genetic testing, cytogenetic testing and biochemical genetic testing. Examples of methods used in genetic testing are listed in Table 6.1.1.

Molecular genetic testing

Molecular genetic testing is used to identify single genes or short lengths of DNA. Through molecular testing, scientists can identify variations or mutations that lead to a genetic disorder. It is the most effective method if the gene sequence of interest is known and the function of the protein is unknown.

Molecular genetic testing can be performed on any tissue sample and requires only a small sample. There are a few different kinds of procedures used to analyse the sample. Two of the most common are the polymerase chain reaction (PCR) and restriction fragment length polymorphism (RFLP).

Polymerase chain reaction (PCR)

PCR is a technique used to make many copies of a piece of DNA, a process referred to as DNA amplification. A DNA sample is mixed with primers (short lengths of DNA that target specific regions for PCR) and other chemicals in a small tube, and the mixture is heated and cooled in cycles. At each cycle of synthesis, the number of copies of the DNA fragment doubles-the DNA has been amplified. In this way a large amount of DNA can be produced in less than an hour. The DNA primers used in PCR target the genes associated with the disorder. The amplified DNA product is then sequenced using DNA sequencing to reveal the presence or absence of the disease-causing mutation.

PCR is a key tool for generating a DNA profile (also known as a DNA fingerprint) using DNA regions called short tandem repeats (STRs) (also known as microsatellites). STRs are short sequences of DNA that frequently appear throughout the genome, characterised by short motifs (e.g. AT) that repeat multiple times (e.g. ATATAT). These are not usually located in the coding regions (exons), but in the non­coding spacer DNA between the exons. The STRs wary in length-that is, the number of times that the motif repeats-in different people. These differences usually have no impact on the phenotype or the health of a person, but they are very useful for DNA comparisons. In forensic analysis, PCR is used to amplify 13 different STR regions (using different primers that target different STRs) to gain a DNA profile unique to each individual. STRs are covered in more detail in Chapter 5.

In molecular genetic testing, the DNA primers used in PCR target the genes associated with the disorder. For example, specific pruners can target the gene associated with cystic fibrosis (CFTR gene) when screening parents to identify carriers of the disease. Different primers target the PAH gene when screening newborns carrying an allele (gene variant) that causes phenylketonuria. The amplified DNA product is then sequenced using DNA sequencing to reveal the presence or absence of the disease-causing mutation. Alternatively, the amplified DNA is treated \\rith special enzymes, known as restriction enzymes (also known as restriction endonucleases) to identify specific alleles.

Sequencing DNA

The products of PCR-copies of a particular sequence of interest-can be used for STR analysis, but they can also be sequenced directly in a DNA sequencer. This allows individual sequences to be directly compared.

The first stage of DNA sequencing involves replicating a piece of purified DNA in a test-tube, just like the usual PCR process. However, in this case, the mixture contains an additional ingredient-terminating nucleotides that are tagged with coloured fluorescent dyes. Each type of terminating nucleotide (A, T, G or C) is tagged with a different coloured dye.

During PCR tile DNA strand is split and one strand (called the template strand) is used to build the section of DNA required. When a primer binds to a DNA strand, tile enzyme DNA polymerase begins to replicate the sequence by adding nucleotides. However, when it uses a terminating nucleotide, the replication stops. This can happen at any point during tile replication, so tile process results in many different lengths of DNA fragments.

After tile PCR is completed, tile DNA mixture is cleaned up to remove any unused nucleotides and oilier substances, so that it contains only fragments of tile DNA sequence required.

In the next stage, the DNA fragments are injected into very fine capillaries filled with a polymer. A large voltage is then applied, causing the negatively charged DNA fragments to be drawn towards tile positive electrode. This technique is called capillary electrophoresis. Smaller fragments move faster than larger ones. Just before reaching the positive electrode, the fragments pass through a laser beam that causes the terminating nucleotide to fluoresce. The colour and strength of the fluorescence is recorded and the data is assembled into a data file.

This file is then analysed by a computer program that arranges the detected bases in the correct sequence. The output from the software is the sequence of bases and a matching chromatogram showing the strength of the fluorescence and the base that produced it. Because the data from the fluorescence detector is sometimes misread (especially if the quality or volume of DNA is low), the sequence must be checked manually by the researcher. Sequences are usually obtained in both directions (5' to 3' and 3' to 5'), and the two sequences are matched up by the program.

The order of bases in the original sample of DNA can be determined from the order of the peaks in the chromatogram.

Restriction fragment length polymorphism (RFLP)

Restriction fragment length polymorphism (RFLP) is a technique that identifies individuals based on differences (polymorphisms) in their DNA sequences at restriction enzyme cut sites. RFLP analysis rapidly provides information about DNA sequences without having to sequence the DNA itself. There are two parts to this procedure-producing DNA fragments using restriction enzyme digestion (cutting) of a DNA molecule, then separation of the fragments using gel electrophoresis.

DNA can be cut into fragments by a set of enzymes called restriction enzymes. These enzymes recognise short sequences of bases (a recognition site) in a DNA molecule. The different sequences of bases in individuals result in restriction enzymes cutting the target DNA at different sites. Therefore, DNA samples from two individuals that have been mixed with the same restriction enzymes will form different DNA fragments, giving each person a unique DNA profile. This type of analysis is used in forensics and paternity testing.

Restriction enzymes are frequently used in combination with PCR to identify specific alleles, such as in screening for carriers of inherited disorders.

Comparing DNA using gel electrophoresis

When the mixture of DNA fragments has been produced, gel electrophoresis is used for separating fragments of DNA. DNA molecules are separated in a slab of jelly-like substance called agarose.

The gel is immersed in a buffered salt solution to keep the pH suitable for DNA molecules; salts are needed to conduct the electric current through the gel. The DNA is mixed with a fluorescing dye that attaches to the DNA fragments. A small amount of this mixture is transferred into wells along the top of the gel, together with a standard mixture of DNA fragments of different known molecular weights (also known as a molecular weight standard or DNA ladder due to its appearance). The standard is crucial to be able to compare the different sizes of the DNA fragments.

The phosphate groups in DNA fragments are negatively charged, so the DNA fragments move through the gel towards the positive terminal when an electric current is applied. Hea,rier (longer) fragments move more slowly than lighter (shorter) fragments, so the fragments become more and more separated with time. After a set time the current is turned off. The gel is removed from the buffer solution and placed on an ultraviolet (UV) light box. When the light is switched on, the dye attached to the DNA fragments fluoresces so the DNA fragments can be seen and their sizes estimated by comparing them to the fragments in the standard (DNA ladder).

This technique can be used to compare the DNA of individuals and identify relationships. Because individuals have different DNA sequences, restriction enzymes cut DNA at different sites, resulting in a unique banding pattern on a gel. This unique banding pattern is known as a DNA profile.

Cytogenetic testing

There are two types of cytogenetic testing--conventional cytogenetic testing (also known as karyotyping) and molecular cytogenetic testing by fluorescence in situ hybridisation (FISH).

Conventional cytogenetic testing (karyotyping)

Conventional cytogenetic testing (or karyotyping) is used to detect abnormalities in the number or structure of chromosomes in **metaphase** cells. This method is useful for detecting large changes in chromosomes using light microscopic methods at approximately x1000 magnification. An image of an individual's set of chromosomes is known as a karyotype.

Molecular cytogenetic testing by FISH

Fluorescence in situ hybridisation (FISH) is a technique for detecting the presence or absence of specific DNA sequences. Molecular cytogenetic testing by FISH is a method in which fluorescently labelled DNA fragments are allowed to hybridise, or attach, to whole chromosomes. This allows scientists to assess the chromosomes for changes in their structure, such as the deletion or duplication of certain sections. For example, a chromosome that normally has sections in the order of A-B-C-D might have sections A-B-B-C-D (duplication of B), A-C-D (deletion of B) or A-B-C-P (replacement of D with section P from a different chromosome). FISH can detect these chromosomal alterations. Chromosomes can also be assessed for the presence of certain DNA sequences of interest.

Biochemical genetic testing

Biochemical genetic testing studies the amount or activity level of certain proteins. Abnormalities in protein activity levels can indicate mutations in the DNA sequence that are disrupting normal polypeptide synthesis. Scientists may use biochemical tests when the specific gene defect has not been identified, or when there are several possible causes of the disease, such as different mutations in one or more genes. For example, a genetic disease called mucopolysaccharidosis I (MPS I) is caused by an enzyme deficiency that results in an accumulation of molecules called glycosaminoglycans (GAGs) within the lysosomes of cells. If GAGs are not broken down by the enzyme when entering the cells, they accumulate and cause permanent cell damage that progressively hinders physical development. MPS I leads to skeletal deformities, coarse facial features, enlarged liver and spleen, and mental retardation. There is no known cure and individuals rarely live past the age of 10 years. If a person is suspected of having MPS I, biochemical testing can determine if there is evidence of the enzyme deficiency associated with the disorder.

DNA PROFILING

In 1984, an English geneticist named Alec Jeffreys developed a technique he called DNA fingerprinting to identify individuals using variable regions of their DNA. Today, the more conm1only used method is called DNA profiling. This can be used to distinguish one individual from another. It is often used in forensics to identify the perpetrator of a crime, identify bodies after disasters or to confirm if a child is genetically related to a parent.

DNA profiling relies on an individual's unique DNA. The non­coding sections of the DNA, those that do not code for proteins, can vary widely between individuals. DNA profiling uses multiple polymorphic (variable) DNA regions (called genetic markers) to identify individuals.

STRs are examples of commonly used genetic markers. They are short sections of DNA with repeating nucleotides. Variation in the number of repeating units within an STR is common between individuals, making them valuable for identification purposes. There are thousands of STRs throughout the human genome-using multiple STRs increases the variation available for analysis, which in turn improves the likelihood of identifying individuals. The inheritance pattern, structure and usage of STRs is described in more detail in Chapter 5.

DNA profiling is also used for genealogy, biogeographical population comparisons, historical population migration patterns and evolutionary relationships. For these purposes, the DNA sequences used for comparisons include STRs (different markers from those used for crime scene analysis), mitochondrial DNA, Y chromosome genes and single nucleotide polymorphisms (SNPs).

Most recently, DNA profiles have been used to identify key features of an individual's appearance. The identification of alleles for eye, skin and hair colour allows investigators to narrow down the list of suspects based on these characteristics. Occasionally the suspect's ancestry can also be determined. This is a developing field of science and the analysis may not yet be reliable.

Techniques involved in DNA profiling

If a small amount of blood (white blood cells contain DNA, but red blood cells do not), semen or another sample containing DNA is fow1d at a crilne scene, the following steps will be carried out to determine the DNA profile and match it to a suspect.

•DNA is extracted from the sample.

•A small amow1t of this DNA is added to a PCR, which contains specific pruners for each STR.

•The STRs are amplified using PCR, producing a much larger sample for testing, even from a very small amount of DNA.

•Differences in the size of the STRs can be detected by standard gel electrophoresis or by capillary electrophoresis, a rapid, automa ted method. In capillary electrophoresis, the DNA fragments move ill a thin tube under the influence of an electric field. The smaller the size of the fragment, the faster it moves through the capillary tube. As each fragment moves through the tube a laser detector registers a peak on a graph.

•The STR analysis of DNA from the crime scene is compared with the STR analysis of DNA from a suspect.

•If the repeats for all the STRs analysed are the same for the crime scene DNA and the suspect's DNA, then it is a match. The chance that two individuals have the same STR repeats at multiple markers is hundreds of billions to one. Therefore, a match means that it is highly probable that the DNA from the crime scene belongs to the suspect.

Issues related to DNA profiling

While it is unlikely that a person will be incorrectly identified by DNA profiling if the procedure is carried out accurately, it is possible. For example, foreign DNA may contaminate a sample at a crime scene or at the laboratory where the sample is tested. Failing to properly clean equipment could cause a sample from one suspect to contaminate a sample for another test.

When it comes to DNA profiling, privacy is a contentious issue. In Victoria, DNA samples cannot be obtained from a person unless they give permission. However, they can be ordered to provide a sample if there is strong evidence that they may have committed the crime and if the DNA profile could help to confirm or deny their guilt. These DNA samples must be destroyed if the person is not guilty or is not charged. However, in some countries, the DNA may be kept for up to 10 years. This has enabled the identification of criminals who have committed crimes in unsolved cases that occurred before DNA profiling technology was developed. It has also resulted in the exoneration of wrongly accused people.

Storing DNA after a person has served their sentence for a crime may be seen as unethical. Others are in favour of the creation of a 'bank' of DNA samples, provided by everyone in the community, which could be used to solve crimes and perhaps trace the remains of unidentified missing persons. Opponents of a DNA bank argue the potential for these samples to be stolen or used unethically.

6.2 Population genetics and bioinformatics-identifying trends, patterns and relationships

In this section you will learn about the complex field of genetic data analysis, or bioinformatics. This usually involves analysing large amounts of genetic information. It uses powerful computers to detect patterns and build models that will allow researchers to understand more about the structure and function of genes and the genome. You will also learn about the Human Genome Project and the role of genomic research in sequencing the genes of many organisms, exploring the relatedness between species and determining gene function. You will also explore how population genetic techniques and our understanding of inheritance patterns in populations can be used in conservation management, in broad scale studies of disease inheritance and to highlight key aspects of human evolution.

THE HUMAN GENOME PROJECT

In the human genome there are 23 pairs of chromosomes. Between 1990 and 2003, all 23 chromosome pairs were fully sequenced through a massive international research effort known as the Human Genome Project (HGP) led by an American Institute. It was the world's largest collaborative biological project ever undertaken, involving biologists from at least 20 universities, one private company and thousands of people from around the world working towards one common goal-sequencing the human genome. The goal of HGP was to determine the sequence of the complete human genome-the precise order of nucleotides within a DNA molecule and the number of genes in one human individual. Through the Human Genome Project it was found that each haploid set of human chromosomes consists of approximately 3.2 billion DNA base pairs. Diploid cells contain twice as many base pairs-approximately 6.4 billion. The sequence of bases tells scientists where particular genes are located on the chromosomes. The position is known as the locus (plural loci). Genomics technology has improved rapidly since the HGP, private individuals can now pay to have their own genome analysed and many other species have been sequenced. Today's large-scale sequencing projects would be impossible without computer power for the data and automatic sequencing machines, which became commercially available in the late 1980s and have made DNA sequencing much quicker and more reliable. Previously it took up to a year for a scientist to complete a sequence of 20 000 to 50 000 bases, which is just a single chromosome of the shortest length. Today's automated machines can produce a rough draft of a DNA sequence of 50 000 bases in a few hours, ready for assembly and final checks.

THE ROLE OF GENOMIC RESEARCH

When the Human Genome Project began in 1990, a few laboratories helped sequence approximately 100 000 bases. Since then, technological improvements and automation have increased the speed of DNA sequencing enormously. New methods could reportedly sequence a human genome in 10 days, compared to more than 10 years for the first full human genome sequence. These methods are now being used to sequence many other organisms.

Determining gene function

Finding the sequence of the entire human genome does not tell us about the functions of the genes. The protein encoded by the gene must be identified in order to study its location and function. A gene sequence can be analysed and compared to other species where its function is already understood.

Molecular techniques based on gene sequences allow scientists to identify alleles and mutant forms of a gene that may cause a malfunction. The gene sequence can be modified and inserted into model organisms, and studying the effects of the modification helps scientists to understand the normal function. Alternatively, genes can be disabled in model organisms to provide direct evidence for the function of the gene and the protein it encodes. The complete human genome sequence provides the raw data for scientists to search for genes already identified in other organisms that may be useful for therapeutic purposes in humans.

Early detection and diagnosis of human diseases

By analysing DNA sequences, scientists can identify base differences in a gene suspected to be the cause of a genetic disorder. This is done by comparing the candidate gene in a DNA sequence from a healthy person with a DNA sequence from those with the disorder. For example, an inherited form of breast cancer is caused by mutations in the BRCA1gene on chromosome 17. The BRCA1 gene is responsible for the production of enzymes that repair damaged DNA. When normal function is disrupted by mutations in this gene, the cells are more likely to develop additional genetic mutations that can lead to cancer. In such cases, determining exactly how a gene differs in affected and healthy individuals of a family is important for predicting who may be at risk of developing cancer, and in developing a cure.

A person with a family history of an inherited disease can have their DNA sequenced to determine if they carry the allele that caused the disease in other family members. In some cases, they can then take measures to delay the onset or reduce the severity of the disease.

Sequencing genes of many organisms

Besides sequencing the human genome, genome sequences have now been generated for some key model organisms (organisms commonly used in scientific research). Examples include the house mouse (Mus musculus), brown rat (Rattus norvegicus), fruit fly (Drosophila melanogaster), rice (Oryza sativa) and wild mustard cress (Arabidopsis thaliana). More and more species are being sequenced as the technology becomes more affordable and accessible. In particular, the relatively small genomes of prokaryotes and viruses are being added to collective genomic databases, increasing our knowledge of how these important organisms function genetically. At the time of writing, one of the most recent animals to have an entire genome sequenced was the rare and endangered golden bat (Myotis rufoniger) in South Korea.

Scientists have used gene sequencing to understand the function of many genes used in agriculture. For example, researchers have found that fragrant varieties of rice, such as jasmine and basmati rice, have a change in the allele of a gene on chromosome 8. This different allele causes a fragrant protein to be produced. The difference does not affect any other part of the development of the plant.

POPULATION GENETICS IN CONSERVATION

AND MANAGEMENT

Population genetics is a diverse field, and has uses outside of animal and plant breeding. Examining the variation within and between populations can give us insight into the processes of evolutionary change--after all, evolution itself is merely the result of changes in allele frequencies in populations over time.

Genetic variation and conservation

For populations of species to remain viable in changing environments, there must be genetic variation (also known as genetic diversity).

One reason for this is that genetic variation is what allows populations to adapt to environmental change-genetic variation creates a greater variety of individuals that will respond to selection pressures differently. For example, populations with high genetic variation are more resistant to disease. Individuals with alleles that provide resistance to a disease are more likely to survive and reproduce than individuals that do not have these alleles. In populations with low genetic variation, alleles that provide disease resistance are less likely to occur, leaving the entire population vulnerable in the event of a disease outbreak.

Another reason that genetic variation is necessary for the long-term survival of a population or species is that, over time, inbreeding allows unhealthy mutations to accumulate. While one mutation in one gene in one individual might not be harmful (because that individual most likely has another healthy copy of that gene from the other parent), two copies of that mutation may be lethal. The more closely related individuals are, the more likely it is that they share copies of potentially lethal mutations. This is not a concern if they do not breed, but inbreeding concentrates those alleles, increasing the chance that offspring will suffer from genetic diseases.

Population genetics researchers can measure the genetic variation of a population by sampling multiple individuals and analysing multiple genetic markers. Population genetic data has many applications in conservation and the preservation of populations and species, such as captive breeding programs, taxonomy (identifying and classifying species) and prioritising areas for habitat protection.

Another way in which population genetics can assist in conservation is by identifying invasive species. When these appear in a new environment and begin to cause damage to the local ecosystem, population genetics can be used to find out where they came from. For example, the Northern Pacific sea star (Asterias aniurensis), was introduced to Tasmania in ballast water and has since spread to coastal areas in Victoria and in southern NSW. This highly successful invasive species has caused significant damage to local ecosystems. Using population genetics, researchers were able to pinpoint Japan as the most likely region of origin.

Defining species

It is not unusual for distinct populations of a species to be quite genetically different from one another, simply because they are separated by geographic distance or other barriers that prevent gene flow. However, population genetic studies sometimes reveal that two populations that are thought to belong to one species are in fact two distinct species. These are called cryptic species. Such research is very important as it allows scientists to properly understand the species that make up an ecosystem-if a species is not described, it cannot be adequately monitored or protected. For example, the common octopus (Octopus vulgaris) was once thought to be a single species with a global distribution. However, genetic studies have shown that the common octopus could be at least six species, if not more. When there are many species together that are difficult to tell apart, this is called a species complex.

To monitor the populations of species, it is also important to be able to tell them apart without sequencing their DNA. Compared to simply looking at an animal, genetic analysis is time consuming and expensive, and conservation work often has limited funding. For this reason, finding a visible difference between the species in a complex is very important.

POPULATION GENETICS TO DETERMINE DISEASE INHERITANCE

While pedigree analyses can tell us a great deal about the way in which certain genetic diseases are inherited, they are often only useful in fairly straightforward situations where historical family information is readily available. You learnt about pedigrees in Chapter 5. In diseases where multiple genes are involved or are only partially expressed, inheritance patterns are more complicated and more data is required. Where time is of the essence and family history data is not available, population genetics can shed some light on the inheritance of diseases.

Isolated populations

Isolated populations are a particularly rich source of data when it comes to genetic disorders. This is because isolation often results in inbreeding and the genetic characteristics of the population become concentrated over multiple generations. The overall genetic variation in isolated populations is reduced, and traits that are rare in the wider population are more likely to occur. When these traits are more common, they are easier to study.

A famous isolated population is that of the Pitcairn Islanders. Pitcairn Island is a volcanic island in the South Pacific Ocean. The population on the island descends from the survivors of the famous mutiny on the HMS Bounty in 1789, including a number of British sailors and Polynesians kidnapped from another island in Tahiti. When the founders of a population are very few in number, and there is no subsequent immigration, the population will experience a phenomenon known as the **founder effect** <*Semiotic: fact: effect, linguistically defined: trained gaze*>. Even if the founding population is genetically variable (in this case, a mix of Polynesian men and women, and the surviving mutineers from the British navy ship), inbreeding will rapidly concentrate alleles in subsequent generations. In 1856, the Pitcairn Islanders (having discovered that Pitcairn Island was too small to feed their growing population) migrated to Norfolk Island. However, 18 months later, 17 of the Pitcairn Islanders returned to Pitcairn Island and another 27 followed five years later. In 20 14, only 57 individuals remained on Pitcairn Island.

Today, over half of the population of Norfolk Island can still be genetically traced to the survivors of the Bounty, which is a strong indicator of inbreeding. Studies of this population have discovered a much higher proportion of cardiovascular disease indicators in the Norfolk Island population than in the wider population. The incidence of cardiovascular disease is correlated with the proportion of Polynesian ancestry present in an individual. The **founder effect** <*Semiotic: fact: effect, linguistically defined: trained gaze*> has in this case resulted in these traits being concentrated in the population. Studying the genetic profiles of individuals descended from the Bounty survivors may shed light on the multiple genes associated with heart disease.

Twin studies

The study of genetically identical (or monozygotic) twins in a population is another way to monitor disease im1eritance. One of the key questions facing medical researchers is how to distinguish between the effects of genetic and environmental influences on a disorder. Sometimes a disorder will not necessarily be expressed to the same degree in different individuals, and it is not clear whether this is due to differences in their DNA or in their lifestyle or environment. Identical twins, however, offer an opportunity to separate those factors due to their identical DNA. If a condition occurs in both identical twins less than 100% of the time, it has an environmental component. By comparison, fraternal (or dizygotic) twins are only related to the extent that most siblings are. Comparing the occurrence of a disorder between identical twins and fraternal twins can shed light on the degree to which a condition is genetic (or heritable).

Twin studies are particularly common in psychological research, because the presentation of mental illness is often highly variable and subject to environmental influence. For example, if one identical t\vin has a diagnosis of schizophrenia, the other twin will also have a diagnosis in 50% of cases. By contrast, if a fraternal twin has schizophrenia, the other twin will only have a diagnosis in 10 to 15% of cases. This difference suggests that genetics plays a very strong role in the expression of the disease. Similar numbers have also been found for bipolar disorder, anorexia, and other mental illnesses, suggesting that these also have a strong genetic component as well as being subject to environmental influences.

Genome-wide association studies

DNA sequence variation is being studied on a large scale in populations. Genome-1,vide association studies (GWAS) search for gene variants, often single base changes called single nucleotide polymorphisms (SNPs) that are associated with various traits. This sort of study requires very large samples, comparing the SNP arrays of a large group of people with a particular disorder, and another large group without the disorder (the control group). A G\X1AS will screen for thousands of SNPs at once. Because there is so much natural variation in the genome, it is often very difficult to find a true association between an SNP and a disorder.

The demographics of the sample also make these studies difficult. Biological sex and age need to be controlled for in the sample. In addition, genetic mutations leading to disorders are known to be much more common in the populations where they first occurred-therefore, samples need to be controlled for the geographical, historical and ethnic background of the individuals. This is called population stratification, and is the main reason that sample sizes for G\'<1AS need to be very large. However, there have been some notable successes. SNPs associated with age-related macular degeneration were the first to be identified in a study in 2005. These SNPs can then be used for screening individuals to identify those at risk of developing the disorder.

POPULATION GENETICS AND THE EVOLUTION OF MODERN HUMANS

The quest for answers about human origins and the evolutionary paths that led modern humans to where we are today is more exciting than ever, with rapidly advancing DNA technology and the discovery of new fossils and archaeological artefacts. Our evolutionary story is a dynamic one, changing as new information comes to light or new ideas are found to have greater explanatory power.

Modern humans and evolutionary relationships

Homo sapiens is the Latin term for our species. The term translates as 'wise man' and refers to anatomically and behaviourally modern humans. How sapiens is one of the most widespread, adaptable and influential species to have ever existed. Under the Linnaean system of biological classification, Homo sapiens is a eukaryote and a member of the animal kingdom.

Among animals, humans are mammals, with the characteristics of body hair and the ability to suckle young. Humans are also classified as primates, having a grasping hand, bicuspid teeth, a short nose and well-developed eyes and brain. Primates are also characterised by their mobile hip and shoulder joints. Within the Primate order, humans belong to the same family (Hominidae) as the great apes, which include orangutans, gorillas, chimpanzees and bonobos. All members of the family Hominidae (hominids) lack a tail and have similar skeletal and skull features. In addition, hominids are bipedal (meaning they walk on two legs) and have very large brains. As well as sharing many anatomical and behavioural features with all the great apes, modem humans share 98.8% of their DNA with chimpanzees and bonobos, our closest living relatives. Although modern humans are very similar to chimpanzees and bonobos, humans did not directly evolve from chimpanzees or any other living primate. DNA and fossil evidence reveals that humans last shared a common ancestor with chimpanzees and bonobos approximately six to eight million years ago.

There are various fossilised human-like species that have been discovered, all characterised by walking upright. Sahelanthropus is the oldest known ancestor of modern humans, with skulls found in central Africa that date to approximately seven million years ago. Some scientists think that Sahelanthropus may represent a common ancestor of humans and chimpanzees, but this is still debated in the scientific community. Species of the extinct genus Australopithecus are the closest relatives to our genus, Homo Australopithecus had a forward-jutting face, a small brown and a pronounced ridge above the eyes, but hands and teeth that were similar to ours. Homo diverged from Australapithecus approximately 2.8 million years ago. The genus Homo is characterised by a larger brain and includes nine to 15 extinct species known to date (the identities of some fossil specimens are uncertain). Our species, Homo sapiens, is the only living member of the genus Homo. Of the extinct species, Homo habilis, found in Africa, is the oldest. A later species, Homo erectus, which had a larger brain than Homo habilis, spread into Southeast Asia about 1.6 million years ago, possibly surviving to about 300 000 years ago.

Fossil and DNA evidence is continuing to uncover new information and helping us to tell our story, but many questions remain concerning the origins of modern humans and fossil species.

DNA evidence for early human origins

Fossil evidence is a rich source of information when it comes to the evolution of humans and other hominids, but in the last few decades, DNA evidence has shed light on more recent events. Fossil evidence indicates that humans almost certainly originated in Africa approximately 200000 years ago, and that the first appearance of modern humans outside of Africa dates to around 92 000 years ago, but fossil evidence could not reliably tell us what happened afterwards. DNA evidence can help us answer a number of questions. When and how did humans emerge from Africa and spread across the globe? Did modem humans replace the other hominids, like the Neanderthals (Homo neanderthalensis) that lived at the same time, or did they interbreed with them?

One of the first ways that researchers used DNA to look at human origins was by using mitochondrial DNA (mtDNA). Since mtDNA does not recombine and is only maternally inherited (i.e. from mother to offspring), it is much simpler to analyse and offers a much more straightforward story than nuclear DNA. A wide sampling of the mitochondrial genome from humans currently living provided an important insight into human evolution. In 1987, researchers learnt that the most recent common ancestor-that is, the woman from, whom all current human mitochondrial genomes were inherited---could be traced to Africa around 200 000 years ago. In the science media of the time, this woman was referred to as 'Eve', but it is important to remember that she is only the mitochondrial Eve--different genes have different histories, and this tells us only about the mitochondrial genome of lineages that have survived to the present day.

Other analyses of the mitochondrial genome can tell us about the migration pattern of early humans out of Africa approximately 100 000 years ago. By examining the differences in gene sequences between people from different regions with differing ancestries, we can reconstruct this path.

After the discovery of mitochondrial Eve, other research focused on the inheritance of the Y chromosome and other nuclear genes that undergo very limited recombination. Like mitochondrial DNA, the Y chromosome can be used to trace the most recent common ancestor from whom all current human Y chromosomes were inherited. Because the Y chromosome is paternally inherited (i.e. from father to son) this ancestor is a male who lived in Africa approximately 90 000 years ago. In some ways, the migratory patterns show by the analyses of nuclear genes are very similar to those of the mitochondrial analyses, but there are distinct differences. This does not mean that either pattern is incorrect, but that different genes experience different changes due to recombination, independent assortment, mutation and genetic drift (random changes in allele frequencies). The more genes included in a study, the more closely the results will represent the true history of the organism.

Ancient DNA evidence, Neanderthals and the Denisovans

Fossil evidence has also shown that early Homo species co-existed with two other species of the genus Homo the Neanderthals and the Denisovans (Homo denisovans). While the Neanderthals have been well-studied, the Denisovans are known only from a single finger bone and two teeth. DNA has, however, been successfully recovered from both species. Isolation of DNA from very old material (such as the bones of a Neanderthal) is a difficult and complex process. The DNA is heavily degraded (broken into very small pieces) and requires special care. Techniques used to handle such old genetic material are known as 'ancient DNA techniques'. Fragmentary DNA, such as ancient DNA, must be sequenced in very small overlapping pieces. The process is also vulnerable to contamination (from other samples or even from researcher DNA), so this work is only done in specialised clean laboratories.

A small segment of mitochondrial DNA from a Neanderthal bone was first analysed in 1997, and showed that the Neanderthals were genetically distinct from modern humans. It was a further 11 years before an entire mitochondrial genome of Neanderthals was sequenced in 2008, and finally in 2014 an entire nuclear genome was sequenced. The genetic evidence from multiple Neanderthal samples no1,v shows indisputable evidence of interbreeding between early modern humans and Neanderthals, but the presence of Neanderthal DNA in human genomes only occurs in non-African genomes, suggesting that this occurred after modern humans left Africa. This is not surprising, however, as Neanderthals were restricted to Europe and Asia.

It appears that while Neanderthals have contributed 1-4% of the genomes of non-African modern humans, this contribution is restricted to the nuclear genome. There is no evidence of Neanderthal contribution to the human mitochondrial genome. There are a number of possible explanations for this.

1 It is possible that human lineages existed that carried Neanderthal mitochondrial DNA, but they have since died out.

2 If only Neanderthal males were interbreeding successfully with female humans, the resulting offspring would have the mother's (human) mitochondrial DNA. Given that humans and Neanderthals were widely divergent genetically, they may have only been capable of fertilisation between a male Neanderthal and a female human, and not between a female Neanderthal and a male human.

3 There may have been social or cultural issues affecting the survival or production of any hybrid offspring.

Genetic research has also found that Denisovans were distinct from humans and Neanderthals (though this is based only on a single sample). There is also evidence that the Denisovans may have interbred with Neanderthals, with a 17% similarity in their genomes. Nuclear DNA studies have also shown that 3-5% of the DNA of present-day humans is shared with Denisovans, indicating that the Denisovans interacted closely with both early humans and Neanderthals.

Module 6 Genetic Change

Chapter 7 Mutation

7.1 Types of mutations

All mutations are changes in DNA, but this can take many forms and occur on many scales, from a large-scale change in chromosomal arrangement to a single change in one nucleotide. In this section, you will learn about the three major types of mutations and how these different processes affect the organism.

MUTATION

All genetic variation between species and between individuals of the same species is a result of mutation. Mutations are changes in DNA (deoxyribonucleic acid). It is important to remember that mutation means change, not the introduction of a fault or disease. Mutations can have a beneficial or harmful effect or no effect at all on an organism.

Mutations can occur randomly as errors during cell replication and can affect a single gene, multiple genes or may involve entire chromosomes. They occur spontaneously or as a result of mutagens-factors that induce mutation. Most mutations are detected and repaired by enzymes. Those that cannot be repaired fall into one of three categories-neutral, beneficial or harmful:

•neutral mutations have no effect on survival

•beneficial mutations increase the likelihood of survival

•harmful mutations decrease the likelihood of survival.

POINT MUTATIONS

Genetic sequences are read in sets of three nucleotides. These nucleotide 'triplets' are more formally known as codons. Codons code for specific amino acids. As you learnt in Chapter 4, amino acids begin as DNA triplets, which are then transcribed into mRNA codons. The mRNA codons (which now have the nucleotide U, uracil in place of T, thymine) directly code for specific amino acids. Using the four nucleotides, A, C, G and U, there are 64 possible codons but only 20 amino acids. This means that most of the ammo acids are coded for by more than one codon. This is why the DNA code is referred to as degenerate.

A mutation that alters, adds or removes only one or very few nucleotides from a sequence of DNA or RNA is called a point mutation. Point mutations typically only affect a single gene. They include substitution and frameshift mutations.

Substitution mutations

A substitution mutation is a point mutation in which one nucleotide is replaced by another type of nucleotide. There are different types of substitution mutations including:

•silent mutations

•missense mutations

•nonsense mutations.

Silent mutations

A silent mutation occurs when a substitution results in a new codon that still codes for the same amino acid. For example, the DNA triplet CCA is transcribed into the messenger RNA (mRNA) codon GGU, which codes for the amino acid glycine (Gly). If the last nucleotide in the sequence is substituted for any other, the codon will still code for glycine and will not have any effect on the final polypeptide. The normal and mutated sequences of this silent mutation are shown below as an example (the mutation is indicated in red).

Missense mutations

Substitution mutations that result in an amino ac id replacement are said to be missense mutations. Missense mutations still produce a protein. Whether this altered protein can function properly or not depends on the importance of the amino acid that was replaced.

Normal and sickle cell haemoglobins differ due to a missense mutation in which the amino acid glutamic acid (Glu) is replaced by valine (Val) in the beta chain of the haemoglobin protein. The distorted shape of the sickle-cell haemoglobin, called 'haemoglobin S', affects the overall shape of red blood cells, causing them to become sickle- or crescent-shaped.

Below are the normal and the mutated sickle-cell nucleotide sequences (the mutation is indicated in red).

Nonsense mutations

When a substitution mutation results in the creation of a stop codon (UAA, UAG or UGA) it is classified as a nonsense mutation. Stop codons (also known as termination codons) signal translation to stop, resulting in the termination of protein synthesis. Stop codons are an important part of normal protein synthesis but if it appears too early in an amino acid sequence, it can have severe effects. Mutations mat result in premature stop codons are called nonsense mutations because may result in incomplete and usually non-functional protein products.

Thalassaemia is an example of a nonsense mutation in which codon 17, of a 14 7-codon sequence, codes for 'stop' instead of lysine (Lys) (AAG is changed to UAG).

Below are the normal and the mutated thalassaemia nucleotide sequences (me mutation is indicated in red).

Frameshift mutations

Frameshift mutations involve one or two nucleotides being either added or removed from a nucleotide sequence, altering every codon in mat sequence from that point onwards. These mutations can have significant effects on the polypeptide because as every codon is altered, so too is every amino acid they code for after the point of mutation. This results in the Joss of functional protein, as it is likely that me resulting polypeptide would be completely different. In cases where the frameshift mutation creates a stop codon earlier in the sequence, the resulting polypeptide will be shorter.

There are two types of frameshift mutation:

•a nucleotide insertion adds one or two new nucleotides into the sequence and pushes the rest of the nucleotides back one or two places

•a nucleotide deletion removes one or two nucleotides and pulls all the following nucleotides forwards by one or two places.

CHROMOSOMAL MUTATIONS

Mutations that affect large sections of a chromosome, typically multiple genes, are called chromosomal mutations (or block mutations). These types of mutations usually occur during meiosis in eukaryotic cells. 'They can also be caused by mutagens such as radiation. When a gene is disrupted by the mutation, the effects are serious, even lethal. There are five main forms of block mutations:

Duplication mutations

Duplication mutations involve the replication of a section of a chromosome that results in multiple copies of the same genes on that chromosome (Figure 7.1.4a). There can be thousands of repeats. This often increases gene expression, which can be harmful or beneficial depending on the gene involved.

Inversion mutations

An inversion mutation involves a section of the sequence breaking off the chromosome, rotating 180° and reattaching to the same chromosome. Inversions may involve as few as two bases or they may involve several genes.

Deletion mutations

Deletion mutations remove sections of a chromosome. Deletions lead to disrupted or missing genes, which can have serious effects on growth and development. Chromosomal deletions are often fatal.

Insertion mutations

An insertion mutation occurs when a section of one chromosome breaks off and attaches to a different chromosome. In eukaryotes, the effects of this type of mutation depend on whether the cell retains two copies of every gene. During meiosis, random assortment may separate the chromosome with the insertion from the chromosome that originally contained the inserted section. This can result in some gametes with two copies of the genes in the inserted section, while other gametes may be missing the genes entirely.

Translocation mutations

In translocation mutations, a whole chromosome or a segment of a chromosome becomes attached to or exchanged with another chromosome or segment. For example, sections from two non-homologous chromosomes (chromosomes that are not a pair) may break off at the same time. They may reattach to the other chromosome, swapping genetic material. Translocations typically interrupt normal gene regulation and are the cause of some forms of cancer.

CHROMOSOMAL ABNORMALITIES

When a mutation involves whole chromosomes, or the number of chromosomes, it is termed a chromosomal abnormality. This type of mutation is easily detected with a karyotype, which is a technique of staining and photographing chromosomes to help classify them and detect chromosomal mutations. There are two main forms of chromosomal abnormalities: aneuploidy and polyploidy.

Aneuploidy

Aneuploidy is the presence of an abnormal number of a particular chromosome, either an extra chromosome (called a trisomy when there are three copies of one chromosome) or a missing chromosome. Examples are Down syndrome (trisomy 21) and Patau syndrome (trisomy 13). Aneuploidy is usually caused by non­disjunction during meiosis. This is when two homologous chromosomes do not separate during the first division of meiosis. Aneuploidy results in gametes with an incorrect haploid number, and so results in an abnormal diploid number, which often leads to miscarriage of the embryo in humans. Aneuploidy can also occur in plants, such as maize, resulting in sterility. You will learn more about non­disjunction of chromosomes in Section 7.4.

Aneuploidy of sex chromosomes

Aneuploidy of the human sex chromosomes, X and Y, can result in a number of different conditions, some of which are summarised in Table 7.1.1.

Polyploidy

Most eukaryotic cells are diploid, that is, they have two complete sets of chromosomes. For sexually reproducing organisms, the ovum provides one set of chromosomes and the sperm provides the other set. The gametes themselves are typically haploid, to ensure that fertilisation restores the correct number of chromosomes to the zygote.

However, errors during meiosis can result in diploid gametes. If one of these gametes is fertilised, it will result in a zygote with more than the usual two sets of chromosomes. Polyploidy is the condition that results from cells and organisms that contain more than two full sets of chromosomes; that is, more than two of every chromosome in the set.

In humans, polyploid zygotes do not survive. However, polyploidy can also arise from errors during mitosis and produce groups of polyploid somatic cells. Having a few cells with an abnormal chromosome number may not affect health. A liver, for example, may function normally but have patches of polyploid cells.

Polyploid animals include some insects, earthworms and tree frogs. However, polyploidy is more common in plants than animals because many plants can survive by asexual or vegetative reproduction. A triploid (3n) organism, resulting from the fusion of one haploid gamete and one diploid gamete, is typically sterile or has low fertility because of problems with chromosome pairing during meiosis and gamete formation. However, a triploid plant could survive by vegetative reproduction or by spontaneous duplication of all chromosomes to 6n. Tetraploid organisms can result from two diploid gametes fusing. A tetraploid organism can produce viable offspring if it breeds with another tetraploid, but not with a diploid individual.

7.2 Mutagens and their impacts

Mutagens are substances or processes that can dramatically increase the rate of mutations, often beyond what an organism can tolerate. While we often think of mutagens as artificial, many occur naturally. In this section, you will learn about a wide range of mutagens and how they function. Mutagens can also function as carcinogens, or cancer-causing agents; you will also learn about how this process takes place.

HOW CARCINOGENS FUNCTION

You will be familiar with the process of cell division and the steps of the cell cycle from Chapter 3, and the processes of DNA transcription and translation from Chapter 4. While the cell cycle is highly regulated, the regulatory mechanism can be disrupted by a number of factors, such as damage to DNA caused by exposure to mutagens. If uncontrolled cell division occurs during embryonic development, the embryo will be abnormal and, in most circumstances, will abort. If this occurs in a mature organism, uncontrolled cell division may lead to neoplasms, which can eventually transform into cancer.

A neoplasm is an abnormal growth of tissue that usually, but not always, forms a mass. Neoplasms are more commonly referred to as tumours, but not all are cancerous. There are three types of neoplasm:

•benign-these form localised masses but do not transform into cancer

•potentially malignant-these form localised masses that will eventually invade other tissues and transform into cancer

•malignant-these form masses that invade other tissues and transform into cancer.

Comparing benign and malignant neoplasms

Benign and malignant neoplasms can appear similar.

Disruptions to the control of cell division

While the cell cycle is highly regulated, the regulatory mechanisms can be disrupted by a number of factors. These interruptions can result in uncontrolled cell divisions; this is the process by which neoplasms are produced, which can eventually develop into cancer.

Genetic factors

Genes code for the enzymes that regulate cell division or regulate apoptosis (programmed cell death). When mutations to these genes occur, regulation of cell division or apoptosis can cease.

Proto-oncogenes are a group of normal genes involved in the regulation of cell division. One of their functions is to stimulate cell growth. They are required for the normal growth and development of cells. However, mutations of these genes can change them into oncogenes, which induce uncontrolled cell division leading to the development of neoplasms.

One example is an important regulator of cellular proliferation called platelet derived growth factor (PDGF). Mutations of the PDGF gene are connected with the development of brain cancer. Mutations of the PDGP receptor gene are associated with thyroid cancer.

Genes that play a role in the development of cancer include:

•Tumour-suppressor genes-the group of genes that code for proteins involved in the slowing down of cell division, the repair of DNA or apoptosis are called tumour-suppressor genes. If these genes are damaged, cell division may go unchecked.

•Inherited oncogenes or mutations-some people also have a genetic predisposition to certain forms of cancer. An individual could receive an oncogene or mutated tumour-suppressor gene from one or both parents. This does not necessarily mean that all family members with that gene will develop cancer as sometimes more than one mutation needs to occur before cancer develops. Environmental factors also play a significant role in cancer development. A family history of breast cancer, for example, is associated with mutations in the BRCAJ or BRCA2 genes, which are located on chromosome 17. A protein coded by these genes takes part in the repair of damaged DNA, but changes in its structure lead to uncontrolled growth. The growth occurs mainly in tissues whose functioning depends on oestrogen (e.g. tissues in the mammary glands (Figure 7.2.3) and ovaries).

Loss of immunity

The immune system is usually able to detect and destroy abnormal cells, including those that are replicating in an uncontrolled manner. If an individual's immune system is weakened, cells that are dividing in an uncontrolled manner may not be detected and may continue to divide to form tumours.

TYPES OF MUTAGENS

While there are internal genetic factors that are linked to naturally occurring mutations in an organism, the external environment also contains numerous mutagens that can damage DNA and, in some cases, disrupt cell division. This disruption will occur if the damage occurs to proto-oncogenes or tumour-suppressor genes. Mutagens can be divided into three categories: chemical, physical, and biological.

Chemical mutagens

Chemical mutagens can be divided into three categories in terms of function:

1 Intercalating agents are chemicals that insert themselves into the bonds between base pairs and alter the shape of the DNA, leading to subsequent errors in replication. A common laboratory stain used to visualise DNA on agarose gels, ethidium bromide, falls into this category.

2 Base analogues are chemicals that are structurally similar enough to that of the nitrogenous bases in DNA that they are incorporated into the DNA sequence during replication instead of the usual bases, meaning that the DNA no longer functions.

3 DNA reactive chemicals are chemicals that react directly with DNA, such as reactive oxygen species (ROS) (highly reactive molecules containing oxygen), which can cause breakages and cross-links in DNA strands. While ROS are produced during normal cellular function, excessive amounts are thought to contribute to the development of cancer. There are numerous other chemicals that fall under this heading. Many of these are not mutagenic on their own, but they do interact with metabolic processes and produce mutagenic compounds.

In many cases, we do not know why some chemicals are mutagenic and what category they fall into. The first step in identifying a mutagenic or carcinogenic chemical is noting a clear correlation between exposure and obvious signs of cancer or mutation. A dose­dependent effect must be shown; that is, the higher the exposure, the greater the risk of a mutagenic effect. In studies of human health, this is very difficult, as human lifestyles are enormously complex and variable and only observational studies are possible. Fortunately, experimental work on model organisms such as fruit flies (Drosaphila melanogaster) and mice have allowed us to draw some more solid conclusions.

Numerous chemical carcinogens occur in the world. The first chemical mutagen to be demonstrated was mustard gas, which was shown to cause mutations in fruit flies in the 1940s. Mustard gas was already known to be toxic and deployed as a chemical weapon in World War I, but its long-term mutagenic effects were unknown at the time. This is not uncommon when it comes to chemical mutagens, as often long-term cell) Sure is required to cause obvious mutagenic effects (such as cancer).

Tobacco contains mutagenic compounds, some of \\,Juch are carcinogenic (Figure 7.2.4). Prior to the widespread smoking of tobacco, lung cancer was an uncommon form of cancer. Now we know that smokers are much more likely to develop malignant neoplasms of the respiratory system, pharynx, larynx and lungs than non-smokers are. In Australia, smoking is responsible for 84% of new lung cancers in men and 77% in women.

Outdoor air pollution is another significant cause of cancer. Air pollution contains a number of known or suspected carcinogens, such as diesel engine exhaust and solvents.

Not all chemical mutagens are artificial. Some processed foods have been linked to an increased risk of cancer, such as refined sugar and processed meat. 1'hese substances are generally present in such small quantities that they are extremely unlikely to do you any harm. To experience an increased risk of neoplasm (or other form of damage to your DNA), exposure would have to be significantly higher than that of the average diet. This is not the case with all naturally occurring mutagens. Alcohol, for example, is a Group 1 carcinogen, meaning that the evidence linking it to cancer is extremely strong. It is worth noting that tobacco itself is a plant containing mutagenic compounds and therefore is a naturally occurring mutagen.

Other naturally occurring chemical mutagens include metals such as cadmium and arsenic. While the mechanism by which mutagenic metals damage DNA is not known in all cases, it is thought that some affect the processes of DNA repair, that is, prevent DNA from fixing random errors in replication that would normally be corrected. Other metals cause the production of reactive oxygen species (ROS).

Physical mutagens

Physical mutagens include particle radiation and electromagnetic radiation (EMR). Radiation is simply the emission or movement of energy through space or materials. In some cases, this energy can interrupt cellular processes and ionise molecules, damaging DNA; however, not all radiation is strong enough to do this. For this reason, when considering biological effect, it is more useful to split radiation into two types: non-ionising radiation and ionising radiation.

Particle radiation is the energy that is emitted by fast-moving subatomic particles. This is the radiation produced by nuclear fission and fusion, such as that which occurs in nuclear power plants and nuclear weaponry, as ,veil as in solar flares. Particle radiation is an extremely powerful mutagen.

Electromagnetic radiation is the radiant energy emitted by the electromagnetic field, and it exists on a spectrum. It includes radio waves, microwaves, infrared, (visible) light, ultraviolet (UV), X-ray and gamma radiation. Unlike particle radiation, EMR does not have mass. EMR is emitted by electrical devices of all kinds and is usually encountered at relatively low levels. 1'he electromagnetic spectrum is split into IO\\' frequency and high frequency radiation.

Ionising radiation

Ionising radiation includes both particle radiation and high-frequency electromagnetic radiation (X-rays, gamma rays and the high-frequency end of the UV light spectrum). On an atomic and chemical level, such radiation contains enough energy to push an electron out of its orbit, thus creating an ion. This alters electric charges, thus it can break chemical bonds causing damage to DNA and basic cellular function. Repeated Jong-term exposure to ionising radiation can cause cancer. The effect is dependent on dosage and frequency; a few X-rays over a lifespan is unlikely to result in neoplasm, while continuous exposure to UV light is responsible for the high levels of skin cancer in the Australian population. It is estimated that around two in three Australians will be diagnosed with some form of skin cancer by the time they are 70, although this figure includes non-malignant neoplasm.

In the short term, high levels of exposure can result in acute radiation syndrome (ARS), commonly known as radiation sickness or radiation poisoning. While this is a very serious condition (and often fatal), it is extremely rare. Historically, ARS is most commonly associated with large-scale nuclear incidents such as the bombing of Hiroshima and Nagasaki, and the containment failure of nuclear power plants such as Chernobyl in Russia in 1986. In the present day, ARS is largely caused by industrial workplace accidents at nuclear power plants and other worksites related to nuclear energy and waste containment.

Non-ionising radiation

Non-ionising radiation is that which exists at the low frequency end of the electromagnetic spectrum: infrared light, microwaves, radio waves, visible light and the near and middle portions of the ultraviolet spectrum. Non-ionising radiation is not associated with damage to DNA or with acute radiation syndrome. The level of energy emitted is simply too low to have an effect on the chemical bonds of DNA or to cause cellular damage.1rus is why energy emitted by mobile phone usage and by wireless Internet signal is not dangerous or carcinogenic. However, non-ionising radiation can cause susceptible materials, particularly those containing water (such as food, fluids, and living tissue) to heat up. This is why you can use microwaves to heat food as they cause the food materials to warm, without altering the chemical structure of the food.

Biological mutagens

Biological mutagens are living molecules that can cause mutations and cancers by interfering with the functions of oncogenes and tumour-suppressor genes. These fall into two categories: certain viruses (termed oncogenic viruses) and a category of DNA known as a transposable element (TE) (also called a transposon).

Viruses

Retroviruses, such as HTLV-1, insert their genetic information into the chromosomes of the host cell (Figure 7.2.11). HTLV-1 is responsible for certain types of leukaemia or blood neoplasms and is considered to be an oncogenic virus. Oncogenic viruses can either be direct or indirect. Direct oncogenic viruses either carry oncogenes themselves or enhance the host cell's proto-oncogenes, while indirect oncogenic viruses cause cancer through decades of chronic inflammation. ID'LV-1 is a direct oncogenic virus, while hepatitis C is an indirect oncogenic virus. Another well-known oncogenic virus includes human papillomavirus (HPV), also known as genital warts, which is responsible for the majority of cervical cancers.

Transposable elements

Transposable elements (also known as 1'E or transposons) are short DNA sequences that move around the genome, giving them the name 'jumping genes'. These are extremely common in eukaryotic genomes and are thought to comprise over 45% of the human genome. These can also cause errors in replication and interfere in the functioning of certain genes. Multiple copies of the same sequence can change chromosome crossover points and cause errors in chromosome duplication. TEs may also insert themselves into the middle of a functional gene, interrupting its function. If these errors are not corrected, this can result in mutations that are passed to the next generation. The original mutation for one form of haemophilia was caused by the insertion of a TE into a gene necessary for effective blood clotting. When this error occurred during meiosis, it was passed on to subsequent generations, resulting in a heritable genetic condition. TEs have also been linked to certain cancers. For example, a virus-like sequence called LINE-1 (or L-1) has been strongly linked with certain forms of colon cancer and also oesophageal cancer. There is a great deal of research yet to be done on the role of transposable elements in the progression and development of cancers.

7 .3 Mutations in non-coding regions

So far you have learnt about mutations mostly as they affect the coding regions of genes, but most of the DNA in a genome is non-coding. Non-coding DNA does not code for amino acids and polypeptides. As you have already learnt in Chapter 4, this does not mean that this DNA is non-functional. Introns (non-coding DNA), non-coding RNAs (genes that form ribosomal RNA and transfer RNA), promoter and ternlinator regions are essential to the functioning of the cell. In this section, you will learn about the effects of mutations in non-coding DNA and how they can be just as significant as alterations to coding sequences. A large proportion of known human genetic disorders can be traced to non-coding regions of DNA.

INTRON MUTATIONS

Introns are the non-coding sequences of DNA separating one exon (coding region) of a gene from another. Often, a protein-coding gene is several exons long. During the transcription process, the introns (which do not code for proteins) must be removed to create functional mRNA. The removal is known as splicing and requires a protein complex known as a spliceosome. The spliceosome is made up of small nuclear ribonucleoprotein particles (snRNPs). This spliceosome binds to three recognised sequences along the intron: one at the 5' end (known as the 5' junction) one at the 3' end (the 3' junction) and one near the 3' end (the branch point) that allows the intron to fold into a loop before it is removed. After the intron is removed, the exons can join together to create an mRNA molecule with a neat, continuous coding sequence.

Because introns are non-coding, they can often tolerate mutations without having any effect on an organism. However, a mutation at any one of these spliceosome binding sites (either the 3' or 5' junctions, or the branch point) may affect the splicing of the intron. Mutations can even have significant effects if they are more than 100 nucleotides from the binding sites if the sequence starts to resemble a splice site. These are known as deep intronic mutations.

If the mutations occur at the binding site, splicing may fail to occur and the intron will be retained in the mRNA. This means that the intron sequence will be read as a coding sequence when the mRNA codes for a polypeptide, which could cause any of the mutations detailed in Section 7.1, including nonsense mutations and unexpected stop codons. This results in what is known as a pseudo-exon. Pseudo-exons are the cause of most of the well-known genetic disorders resulting from intron mutations, such as beta thalassaemia, some of the mutations that cause cystic fibrosis, Duchenne muscular dystrophy, Becker muscular dystrophy, retinitis pigmentosa (a condition causing tunnel vision and ultimately blindness) and Pompe disease (a severe disorder of glucose metabolism).

If the mutations within the intron resemble a splice site, splicing may sometimes occur at the wrong point. This means that the expression of the gene will be reduced, as the correct protein product will be produced only when the correct splicing site is recognised. Therefore, intron mutations that create new splicing sites can reduce gene expression.

Introns may also contain sequences that regulate transcription and translation. Mutations in these sequences can either reduce or increase the amount of gene product produced.

PROMOTER AND TERMINATOR MUTATIONS

Promoter and terminator sequences are neither introns nor exons. Mutations in these regions will mostly result in either over- or under-expression of the gene, leading to either an excess or shortage of the gene product. There are many genetic diseases that could be the result of changes to promoter or terminator sequences, but these are rarely monogenic diseases (diseases caused by a single gene). Some forms of beta thalassaemia are caused by mutations to promoter regions in the globin B gene (HBB).

NON-CODING RNA MUTATIONS

Genes that code for RNA proteins such as ribosomal RNAs (rRNAs) or transfer RNAs (tRNAs) are known as non-coding RNAs, because these genes are not based on a sequence of codons. The product of a non-coding ribosomal RNA gene is not a chain of amino acids, but a highly structured piece of RNA that binds to ribosomal proteins to form a ribosome, while transfer RNA genes produce a structure necessary for the attachment of amino acids to mRNA molecules. These are therefore known as structural genes. The correct structure and binding of ribosomal and transfer RN As are crucial to the functioning of the cell, and therefore mutations are not well tolerated. Minor changes to certain parts of the sequence will not have an effect, but changes in other sites will be lethal, resulting in a failure of the cell to function. The effect of the mutation will depend exactly where in the sequence it occurs and how it affects the folding of the resulting RNA molecule. For this reason, there are currently no known human genetic diseases based on functional mutations to non-coding RNA in the nuclear genome. Mutations in the genes that form ribosomal proteins can cause disease, but these are changes to coding genes, rather than non-coding RNA.

However, there is some evidence of disruptive mutations in non-coding RNA sequences in the mitochondrial genome. There are many different forms of mitochondrial disease, many of which affect protein coding genes, but two of the more common mutations occur in a tRNA gene. This means that the particular amino acid that binds to this tRNA (leucine) cannot be effectively added to mitochondrial proteins. Overall, this has a negative effect on cellular respiration. One of these mutations is associated with a cluster of symptoms known as Melas syndrome (myopathy [muscle weakness] encephalopathy [brain disease] lactic acidosis [a build-up of lactic acid in the body and stroke-like symptoms]) and the other is associated with myopathy alone. The effects of Melas syndrome on the brain are shown in Figure 7.3.3. There are also other mitochondrial diseases associated with mutations to ribosomal RNA genes.

7.4 Mutation and genetic variation

Somatic mutations occur in body cells and only affect the individual. Germline mutations occur in germ cells (cells that differentiate into gametes). They are heritable because they affect gametes and can therefore be passed on to offspring. Mutations in germ1ine cells are not just a matter of concern for individual offspring: these mutations are the source of new alleles in the population. While some of these mutations can cause problems, others can be advantageous. For natural selection to take place, there must be variation in a population. You can think of mutation as the raw material of evolution. In this section, you will learn about both somatic and germ1ine mutations and how they affect an individual organism, the next generation, populations and even species.

SOMATIC MUTATIONS

Somatic mutations are genetic changes in somatic cells (body cells other than gametes). Any mutation that occurs during the normal process of mitotic cell division and growth in non-reproductive tissue is a somatic mutation. Somatic mutations can also be referred to as postzygotic mutations, since they are mutations that occur after fertilisation.

Mutation and the ageing process

Like all mutations, somatic mutations may occur as a result of exposure to mutagens, but they also occur naturally as a result of errors in DNA transcription. The rate of natural mutation in human cell division is much higher in somatic cells than in germline cells. While somatic mutations may result in neoplasm and possible cancer if the mutation occurs in certain genes, this is not the most common result. We are all experiencing a low level of somatic mutation as we grow, develop and age. In fact, the ageing process is largely due to an accumulation of errors during DNA replication over time. To put it another way, we get old because we gather somatic mutations. This is another reason that humans become more prone to develop certain cancers as we age; the more errors we accumulate in our DNA, the more likely it is that those errors will trigger the formation of a neoplasm.

The more that cells divide, the more opportunity there is for replication error (and therefore mutation). Therefore, we would expect a higher rate of cancers in children who are growing rapidly-but we do not see that. In fact, for most cancers, the rate of occurrence in children is very low. This is because evolution has selected for the increased activity of DNA repair enzymes in children and young people, but this protective effect does not last.

Mosaicism

Most somatic mutations do not survive to produce daughter cells, either because of destruction by the immune system, or because of regulatory mechanisms that cause the cell to self-destruct. If the mutations do survive, the altered cells will continue to divide as normal, populating the relevant tissues. This means that an individual may carry different genomes: the normal, inherited genome, which occurs throughout most of the body; and the mutated genome, which only occurs in a select few cells. This condition is called mosaicism. Somatic mutation is common enough that most likely all multicellular organisms experience a certain amount of mosaicism. Most of the time, the effects of genetic mosaicism are not immediately obvious but there are some situations where it is easy to see. The port wine birthmark is an example of this. This birthmark is caused by a single somatic mutation that occurs early in development, before birth. The mutated cell divides and proliferates, causing expanded blood vessels just beneath the skin in the affected tissues.

GERMLINE MUTATIONS

Germline mutations are mutations in reproductive tissue (germ cells) that give rise to gametes. Germline mutations are significantly rarer than mutations in somatic cells. This is because of the elevated levels of DNA repair enzymes in these tissues.

If a germline cell (gamete) undergoes mutation and survives to form a zygote, the offspring will be heterozygous for that mutation in all cells; however, the parent organism will not experience any changes to their own phenotype. These mutations will only affect the next generation.

Like all mutations, germline mutations may occur due to exposure to mutagens that damage DNA, or occur naturally due to errors in meiosis.

Non-disjunction in meiosis

Meiosis is usually an exact process but sometimes errors occur. Missing, extra or malformed chromosomes can result from defective gametes, which may have serious consequences for offspring. Most of these have been discussed in Section 7.1, but there is one error that is specific to meiosis-non-disjunction. This is the usual cause of aneuploidy.

Sometimes during meiosis a chromosome does not separate. This is known as a non-disjunction. Non-disjunction can occur during meiosis I if members of a pair of homologous chromosomes do not move apart properly, or during meiosis II if sister chromatids fail to separate. As a result, one gamete receives two of the same type of chromosome and another gamete receives no copy. If either of the gametes unites with a normal gamete at fertilisation, the zygote will also have an abnormal number of a particular chromosome. An example is non-familial Down syndrome. In this disorder, an individual has three copies of chromosome 21. Therefore, non­familial Down syndrome is often referred to as trisomy 21.

In people with non-familial Down syndrome, the extra copy of chromosome 21 usually results from an error in meiosis in one of the parents. Chromosome 21 undergoes non-disjunction so that cell division produces gametes with either an extra or missing chromosome 21. If a gamete with (n+ 1) chromosomes unites with a normal gamete (n) the zygote will be trisonlic (2n + 1) (Figure 7.4.5).

The additional chromosome 21 results in slower and limited physical development, so people with non-familial Down syndrome tend to be shorter and have less muscle development than people without the syndrome. Intellectual development is also slowed, but well-designed support programs for families, communities and educators can help individuals with Down syndrome to lead healthy, productive lives. The physical and intellectual expressions of Down syndrome also vary greatly; some individuals are more severely affected than others.

MUTATION AS A SOURCE OF NEW ALLELES

When you look at the people around you, you will notice that they all look quite different. They have different heights, different shapes, different skin tones and different facial features. They have different eye colours and hair textures. These phenotypic variations are mostly genetic in origin (although environmental factors will play a role). Most of these variations have their origin in the distant past as mutations in germline cells.1ranscription and translation are precisely regulated processes and any variation in those processes can cause problems. This is why mutations are usually harmful, or--depending on when and how they occur­neutral. However, if that there always the case then all humans would be identical. Mutation is ultimately the source of genetic variation and necessary for evolution to occur.

Variation in one population can be a source of new alleles for another population. The movement of alleles between populations is known as gene flow. When gene flow between populations is high, their gene pools (total genetic variation) will be similar. When gene flow is low or absent between populations, their gene pools will become increasingly different. If two populations are isolated for long enough, they may eventually become different species. Allele frequencies in a gene pool may also change randomly over time as a result of chance events (e.g. birth and death). This is called genetic drift. In small, isolated populations, the random loss of alleles due to genetic drift can have a significant effect, resulting in loss of genetic variation.

Chapter 8 Biotechnology

8.1 Biotechnology-past, present and future

At its simplest, biotechnology is the merging of 'biology' and 'technology', and at its broadest, it is an interdisciplinary technology which encompasses many fields of science and technologies along with biology including chemistry, physics, mathematics and engineering, to help improve the future of humans and the planet. Biotechnology uses biological processes and organisms to create new products and develop new technologies. These new, products and technologies are used in many fields including medicine, agriculture, industry and the environment.

HISTORIC BIOTECHNOLOGY

Humans have been using biological processes to improve their lifestyles long before genes and DNA (deoxyribonucleic acid) were understood or the term 'biotechnology' was first used. Most historic biotechnology was discovered by accident or through simple observation of nature, and was then used to improve everyday life. For example, agriculture developed in ancient times when humans needed to grow their food closer to home to avoid travelling Jong distances for supplies. Plant seeds for crops were tended to by experimenting with different amounts of water, light and nutrients, and crop improvement soon developed where the most successful plants were used to obtain seeds for future crops. The manipulation of biological processes for human use in early agriculture was the basis of biotechnology.

The deliberate selection and breeding of individuals with favourable characteristics is known as selective breeding or artificial selection. Selective breeding of plants and animals is carried out with the intention of retaining favourable characteristics in future generations. For example, corn existed in 5000BC with small cobs and very few kernels. By 1500 AD, selective breeding had produced corn cobs that were almost five times the size of ancestral corn cobs and full of large, juicy kernels. Cross-breeding of individuals with different favourable characteristics led to a vast array of local varieties of common crops such as rice, corn and wheat that had higher productivity than their wild ancestors.

Early agriculture also involved the domestication of wild animals by using selective breeding to produce animals suitable for food and transport plus fur and hides for clothing. A classic example of selective breeding is the mule, which is bred from a female horse and a male donkey. Mules were commonly used in ancient times for the transport of heavy loads. Dogs have also been selectively bred for a variety of purposes including rounding-up livestock, hunting small animals and companionship. As a result of this selective breeding there are now hundreds of dog breeds, all with different appearances and behaviours.

One of the most popular dog breeds in the world, the Labrador Retriever was originally bred to retrieve fishing nets and catch from the freezing Newfoundland waters of Canada. Known in the 1700s as the St John's dog, or Newfoundland dog, the breed ,vent on to become a hunting companion, retrieving game from land and water. The breed became extremely popular in England and America with black coats preferred over the recessive yellow and chocolate coloured coats.

Fermentation has been dated back to 6000 BC when Sumerians and Babylonians used fermentation to make beer. Fermentation occurs when microorganisms (such as yeast and bacteria) breakdown sugars during anaerobic respiration (when there is no oxygen present). This biological process was commonly used in other ancient civilisations to make bread, wine, cheese, yoghurt, tofu and sake. Vinegar was another product of historic biotechnology, produced for consumption with food and used to prevent food spoilage. We now know that it is the acidity of vinegar which prevents the growth of microorganisms, however in ancient times it ,vas simply used like drying, salting and freezing to help food last longer.

Many traditional medicines also used biological processes and organisms. For example, ancient Egyptians used honey to treat wounds and infections, Ukrainians used mouldy cheese to heal infections, and the Chinese used mouldy soybean curds to treat boils. These industrious uses of biological products to improve living conditions and human health demonstrate the important role biotechnology has played in past societies, even before people understood the biological processes they were using or manipulating.

MODERN BIOTECHNOLOGY

While Gregor Mendel identified genes and their role in inheritance in 1865, it was the discovery of DNA in biological processes during the 1940s that marked the beginning of modern biotechnology. It has Jed to more sophisticated production methods including pasteurisation to remove pathogens (disease-causing bacteria), as well as using purified cultures of bacteria. Agriculture has also advanced by using modern techniques to improve crop yields, decrease the use of chemicals and water as well as develop healthier food alternatives.

Modern biotechnology, while continuing to use classic biological processes such as fermentation, also uses a range of molecular techniques such as polymerase chain reactions (PCR) and DNA sequencing. Some common techniques and applications of biotechnology include DNA profiling, gene cloning, genetically modifying organisms, genetic screening, gene therapy and recombinant DNA technologies. You learnt about some of these technologies in Chapter 6 and others are examined in more detail in Chapter 9.

Molecular techniques are used in modern medicine where genetic technology is being used to decrease rates of infectious and chronic diseases, provide improved methods of disease detection and personalise patient treatment. Modern biotechnology is also used in modern industries, for streamlining manufacturing processes, developing clean energy sources, increasing the use of recyclables, providing more accurate forensic data and decreasing water waste.

FUTURE OF BIOTECHNOLOGY

The interdisciplinary science of biotechnology is driving progress across many fields, from medicine and industry, to agriculture and environmental sustainability. Biotechnology research and innovation has the potential to bring about advances on a global scale, such as sustainable agriculture methods to meet the food demands of future world populations, as well as improvements to the lives of individuals (e.g. improved disease detection). While biotechnology may raise social, ethical and environmental concerns, the field continues to bring about new scientific advances worldwide.

8.2 Biotechnology and society

Scientific knowledge can bring great benefits to society; but it can also bring great challenges, as societies consider if or how new technologies should be implemented, and the possible biological, social, economic and ethical implications of their use.

SOCIAL IMPLICATIONS OF BIOTECHNOLOGY

Technology has the power to change people's lives and social structures. Biotechnology is no exception to this; it has the potential to influence the values of a society and change its demographic and economic conditions. In turn, the financial position, lifestyle and social profile of an individual or community can determine whether they have access to ne\v products and services created by biotechnology.

Social equality, accessibility and cost

Biotechnology has allowed people greater access to goods, services and opportunities. For example, recombinant DNA technology can be applied to the development of safer vaccines. One of the current techniques is to remove selected genes from a virus so that it cannot replicate. It can then be safely injected iI1to humans to trigger an immune response.

Biotechnology also has the potential to improve the nutrition and yield of crops grown by farmers. This could lead to improved health and result in greater availability of food to some of the poorest people in the world. Golden Rice is an example of a genetically modified organism (GMO) developed to improve health and nutrition in some of the poorest communities. Rice is a staple food for millions of people around the world; traditional white rice varieties grown in countries such as Vietnam, Bangladesh and the Philippines are lo\v in vitamin A. Vitamin A is a coenzyme that is essential for healthy eyes and the inm1une system. Diets poor in vitamin A contribute to the deaths of many children in developing countries each year. Vitamin A deficiency can lead to preventable blindness in children and increases the risk of common infections such as measles and diarrhoea.

Golden Rice is a transgenic rice crop that contains pro-vitamin A. Golden Rice is produced when two plant genes and one bacterial gene are inserted into the white rice genome. These genes switch on a biochemical pathway in the rice plant that sends vitamin A to the rice grains rather than the leaves, as would occur m non-modified rice. Australian researchers are exploring other genetic modifications to improve the ability of rice plants to use nitrogen for improved growth and reduce the need for nitrogen fertiliser. They also aim to make rice grams richer m elements such as iron, zmc and phosphorus through a process called biofortification. While the success of some GMOs such as Golden Rice is documented, some suggest that the best way to end hunger and malnourishment is a political solution to mequitable global food distribution rather than reliance on any agricultural technology, including GlviOs.

Patenting

Biotechnology companies often control access to ne\v technologies due to patenting of products. Patents are available for any invention and allow the patent-holder to be protected by Jaw so that no one else can make, use or sell the invention for a certain period of time. This raises an issue in biotechnology as new biological material is generally classed as a 'discovery' and non-patentable. Some argue that biotechnology products are human-made 'inventions' and therefore should be patentable.

Patenting of biotechnology has raised concerns regarding the affordability of the technology. If companies control the rights to the genome of genetically modified (Glv1) crops, they also control the prices of the seeds. Farmers that plant GM crops need to spend money each year to buy GM seeds. For example, Bt cotton is a transgenic crop that has been modified to contain genes from the soil bacterium, Bacillus thuringiensis. Expression of these genes produces resistance to conm1on crop pests such as certain caterpillars. Bt cotton seeds lose their efficacy after one generation and the farmer is therefore forced to purchase new seeds each year, limiting the farmer’s income.

Patenting of GM crops also raises legal concerns for farmers. Cross-pollination between GM and non-G1\ti crops occurs naturally as pollen and seeds are carried by the wind or animals. If a farmer's G1\ti crop is 'contaminated' with a non-GM crop, the company that own the patent for the GM crop has legal grounds to sue the farmer for breaching commercial agreements. The opposite can also happen when organic crops are contaminated with Gl'v1 crops and can no longer be certified as organic. For example, a Western Australian farmer lost his organic certification after GM canola crops \Vere planted at a neighbouring farm and cross-pollinated with the organic GM canola. As a result the organic farmer sued the G1\ti canola farmer for losses of over $85 000.

Ne\v technologies are not equally accessible to all communities because countries vary in terms of needs, priorities, legislation and economic and social stability. This raises the question of whether biotechnology creates social inequality. Many of these technologies are expensive but also have the potential to bring about great benefits. For example, GM crops that require less insecticides allow money to be channelled into other needs such as health and education. This has caused debate around who should be able to benefit from these technologies and if limiting access through patents and pricing brings about greater social inequality. These concerns do not appear to have limited the uptake of Glv1 technologies, and many developing countries have embraced GM food and fibre crops while some developed countries oppose their introduction.

Privacy

The ability to choose which personal information is made available to others, and when, is becoming increasingly difficult in today's society. Privacy is a contentious issue in biotechnology with applications such as DNA profiling requiring the storage of people's genetic information in databases. In NSW, DNA samples cannot be obtained from a person unless they give their permission. They can, however, be ordered to do so if there is evidence that they may have committed a crime currently under investigation. These DNA samples must be destroyed if the person is not guilty or is not charged. However, in some countries, the DNA may be kept for up to 10 years. This has enabled the identification of criminals who have committed crimes in unsolved cases that occurred before DNA profiling technology was developed. It has also resulted in the exoneration of people who have been wrongly accused.

Collecting blood samples from newborn babies for genetic screening for abnormalities is another common reason for storing people's genetic information. In NSW, genetic information is retained for 15 years after the individual reaches 18 years old. However, this varies from state to state, with Victoria retaining the information indefinitely. Access to this information is also a contentious issue; parents may choose to give permission for the samples to be used in research projects for which individual identification is removed. Access to the samples may be legally protected by legislation in some states because they contain personal, identifiable, genetic information. However, the genetic information in the samples is the property of the child who cannot, at the time of testing, give consent for it to be used.

Many people argue that there is a potential for misuse of this stored genetic information in the future. For example, unless there is legislation to prevent discrimination, an insurance company may refuse life insurance to a person carrying disease-associated alleles detected by genetic screening. The legislation relating to DNA data varies across states and countries. Laws may change in response to new understanding of the technology and improved techniques, experience in particular legal cases, or changes in political or social attitudes.

Health

While the products and services of biotechnology become more accessible, some concerns have been raised about the safety of these new technologies for humans. GM foods have been introduced to modern diets and are readily available in grocery stores however, some groups have raised concerns about the safety of these foods for human consumption. While some claim that GM foods cause toxic effects on organs and have the potential to change consumers' DNA, most studies have found no evidence of this. Research from independent groups worldwide has concluded that GM foods are safe for human consumption, with no adverse effects on health, reproduction or DNA evident. However, consumer groups are lobbying to have all GM foods labelled so people can choose whether or not they consume them. In Australia, manufacturers are required by law to label foods that contain GM ingredients; however, these laws do not apply to foods prepared by restaurants and other food vendors.

Concerns for human health have also been raised in response to gene therapy. The death of a participant in an early clinical trial prevented further gene therapy trials for some time while scientists investigated the cause. It was found that the participant had experienced an unexpected adverse immune reaction to the viral vector that was used to transport the therapeutic gene. In another gene therapy trial, the target gene was inserted into a vital gene, disrupting its function and causing additional health problems in the patient. There have also been cases of contamination with an infectious virus when viral vectors have been used. As a result of the rise in concerns over the safety of biotechnology, risk assessments and evaluations are conducted to determine any potential threats to the health and safety of the community. The risks of all known and future biotechnologies are evaluated with regard to their durability, exact purpose, social relevance, safety and security. In Australia, biotechnology is regulated by the following governing bodies:

•Therapeutic Goods Administration (TGA)-medicines, medical devices, blood and tissues

•Food Standards Australia New Zealand-foods

•Office of the Gene Technology Regulator (OGTR)-gene technologies

•Australian Pesticides and Veterinary Medicines Authority-chemicals used in agriculture and veterinary medicine

•National Chemicals Notification and Assessment Scheme-industrial chemicals

•Department of Agriculture (Biosecurity)-imported GM products.

Society's view of biotechnology

The rapid advancement of biotechnology represents one of the great technological revolutions of modern times. It has introduced unprecedented changes in how we deal with the challenges faced in human health, agriculture and the environment. The public's perception of biotechnology is complex, and there is no clear correlation between people’s knowledge of biotechnology and their acceptance of new technologies. In general, society is more accepting of biotechnology's application to the human health sector, and more opposed to its application in agriculture and industry. For example, the development of genetic testing for the BRCAJ and BRCA2 genes that can be used to assess a woman's genetic predisposition to breast and ovarian cancers has been embraced by society, while the consumption of produce from GM crops has been strongly opposed. Biotechnology is now also impacting society's expectations and values. For example, gene therapy and genetic screening have changed people's expectations of medical intervention. However, like many other procedures, genetic testing can have errors. Parents may sue individual doctors or companies for the cost of raising their child because the doctors failed to identify that the child had a genetic defect in utero.

ETHICAL USE OF BIOTECHNOLOGY

Ne\v technologies have had a profound impact on society, and biotechnology can be celebrated for the many benefits it has brought to humanity. However, biotechnology tends to produce products and technologies that lead to polarised views. As a result, ethical, philosophical and religious issues are also part of the biotechnology debate.

Philosophical, cultural and religious views

The philosophy, culture and religion of many in society shape what they know of the world. Biology, religion and philosophy all attempt to explain the world, and life on Earth, each in different ways. While biotechnology has the potential to bring about benefit, many people believe biotechnology practices and products overstep ethical boundaries. Many biotechnological developments, such as cloning and gene therapy, are raising issues and generating questions that traditionally fall within the domain of religion and philosophy, such as the definition of human life, DNA as central to the individual identity and the control of nature. Many cultures and religions have opposed biotechnologies that are seen to intervene with nature, with the aim of maintaining respect for all life and protecting what many people view as central to the human species and identity.

The religious, cultural and philosophical views of society, and an individual, become particularly relevant when dealing with the areas of genetic screening and reproductive decisions. For example, if a fetus is found to have a genetic abnormality, the parent(s) must decide if they want to continue the pregnancy or terminate it. The difficult decision requires balancing the loss of the pregnancy with the knowledge or prediction of the suffering and challenges for the parents and child if it \Vere born. The decision will be informed by the severity of the genetic disorder and the capacity of the family to accommodate any special needs of the child. Genetic counsellors (Figure 8.2. 7) may assist by providing information on the disorder, discuss issues related to passing on the genetic defect to other potential children and help the parents come to a decision consistent with their values. Genetic abnormalities can also be identified via preimplantation genetic diagnosis, which identifies genetic abnormalities before the embryo is implanted. At this stage, the embryo is a collection of undifferentiated cells. 1vtany people feel that choosing not to implant an affected embryo is a choice that reduces potential suffering for both the potential child and its parents. Others believe that at fertilisation the embryo is uniquely human and has the same right as any other child. Some argue that removing embryos that are less than perfect will bring about a society that lacks compassion and acceptance of difference.

Religious, philosophical and cultural views also influence a person's view of transgenic animals and genetically modified foods. For example, some religious and cultural traditions have dietary practices that restrict the use of transgenic animals for food. This is because while a transgenic animal may appear to be of one species, it may contain some genetic elements of a species that members of the religion choose not to eat. Many cultures also place high value on all life, which raises animal rights issues in regard to biotechnology.

Medical intervention and consent

With technologies such as cloning and genetic screening, it is easy to see how relevant biotechnology becomes to the field of medicine. However, with the use of this new technology come ethical issues such as the values of dignity, bodily integrity and independent choice. Regulations are in place in Australia to ensure that patients must give informed consent for all forms of medical and genetic testing, screening and therapeutic treatment. Informed consent for prenatal genetic testing of a fetus, for example, would include medical staff informing the pregnant woman whether the disorder being tested for can be prevented or treated, whether she will be faced with a decision to terminate the pregnancy and any potential risk of testing to her or the fetus. In Australia, strict regulations also govern medical research. Guidelines are in place to instruct researchers in the selection of clinical trial participants, the handling and storage of biological material, and the storage, analysis and reporting of data.

Legal implications

Legislation in Australia tightly regulates biotechnology, with laws to prohibit cloning humans, as well as regulating research conducted on human embryos. However, legal 'grey areas' can arise such as tile legal impacts of storing genetic information. For example, if a four-year-old child tests positive for a genetic disorder that will affect them as an adult, issues regarding who should have legal rights to access this information arises (e.g. future employers, insurers and financial institutions). Another issue is whether medical staff have tile legal right to inform tile child's blood relatives that they tested positive for a genetic disorder if tile condition is likely to be heritable.

DNA profiling raises significant legal implications as it is regularly used in forensics to help personnel in tile legal system determine a person's guilt or innocence of a crime. These days it is unlikely mat a person \viii be incorrectly identified by DNA profiling if tile procedure is carried out accurately. However, mere is tile possibility of incorrectly identifying an individual through DNA profiling. For example, foreign DNA may contaminate a sample at a crime scene or at tile laboratory where tile sample is tested. Failure to properly clean equipment could cause a sample from one suspect to contaminate a sample for another test. Legal implications from these potential errors include tile wrongful conviction of suspects of crimes. Further, storing DNA after a person has served their sentence for a crime may be seen as unethical. Others are in favour of the creation of a 'bank' of DNA samples, provided by everyone in the community, which could be used to solve crimes and perhaps erase tile remains of unidentified missing persons. Opponents of a DNA bank argue that there would be potential for these samples to be stolen or used unethically.

Animal welfare

Biotechnology has improved animal welfare in many ways. This includes being able to use bacteria for therapeutic recombinant proteins rather than animal products, the production of synthetic enzymes, which were once taken from livestock as well as selective breeding to remove characteristics which can cause harm to animals (e.g. cattle bred without horns to avoid fight wounds).

Many applications of biotechnology are also harmful to animal welfare. Genetically modified animals may have characteristics that are beneficial to humans and agricultural productivity but negatively impact the animal's health. For example, transgenic pigs often grow very quickly, detrimentally affecting their heart and joints and causing pain when they are overactive.

While animals remain a source of products for human use many believe the production of transgenic animals violates the fundamental rights of an animal, manipulating them for human purpose, rather than respecting their inherent value.

THE POTENTIAL BENEFITS OF BIOTECHNOLOGY

RESEARCH TO SOCIETY

While biotechnology prompts many ethical, moral and legal discussions, it has great potential to benefit society. Biotechnology research can be applied to fields including medicine and agriculture, and may include studies on gene expression, natural genetic variation and other biological processes (Figure 8.2.13). In Australia, biotechnology research is undertaken by scientific institutions like the CSIRO and universities. Research institutions can also be affiliated with hospitals, such as the Westmead Institute for Medical Research. Research is supported by funding from various sources including government agencies, no t-for-profit organisations and private donors.

Pioneering medicine

Potential advancements in the medical field as a result of biotechnology research include improved diagnosis of disease and predisposition to medical conditions, designer drugs individualised to match a patient's specific genetic profile, new vaccines and new treatments for chronic diseases. For example, current research is looking to develop a technique to switch off a gene in muscle cells that causes a certain form of muscular dystrophy. These medical advancements have benefits for the individual patient and the wider community. Preventative medicine and early intervention help alleviate pressures on the medical system and improve the economy as people are better able to maintain health and remain employed.

Pharmacogenomics is the study of how genes affect a person's response to drugs. Traditional pharmacology has used a 'one size fits all' approach to medicine, leaving patients at risk of adverse side effects ranging from minor to severe to fatal. The mapping of the human genome allowed researchers to identify how a person's genes can affect their response to certain drugs. This has the potential to develop effective and safe medications tailored to an individual's specific needs. Pharmacogenomics holds the potential to create safe and more effective treatments for cancer, HIV/AIDS, asthma, epilepsy, depression, cardiovascular disease and Alzheirner's disease.

Gene therapy continues to be a field transforming into the future. The success of new technologies such as vector engineering, CRISPR (Clustered Regularly Interspaced Short Palindromic Repeats) technology, and the discovery of new structures such as microRNA hold incredible promise for new clinical trials. This sophisticated gene technology has the potential to correct inheritable (germline) genetic disorders, meaning families with genetic defects could have access to targeted treatment. These advances in gene technology may also provide improved treatments for diseases caused by somatic mutations. For example, viral vectors that directly deliver dopamine-synthesising DNA are being trialled for the treatment of Parkinson's disease, a condition in which the brain cannot produce enough dopamine for normal function.

Nanomedicine is another emerging field of medical biotechnology that involves the construction of technologies on a nanoscale (1 nanometre = one-billionth of a metre). The use of nanoparticles allow new technologies to be relevant in size to biological structures such as antibodies, which are approximately 10 nanoparticles in size. Nanomedicine will allow targeted interaction and manipulation of biological structures to improve health care. For example, nanoparticles are being trialled to deliver anti-cancer drugs directly to cancer cells without damaging healthy cells. Other applications of nanomedicine include the use of nanoparticles for biological devices, biosensors and biological machines.

Food production

Biotechnology research also holds potential benefits for society through developments in agriculture and environment. Research is continually developing GM crops, making them more disease and pest resistant and tolerant to a range of environmental conditions. For farmers, this means increased productivity and lower input of labour and costs. This technology has the potential to benefit society by producing more nutritious food, grown with fewer chemicals.

Feeding the hungry

In 2017 the global human population reached 7.6 billion and this number is expected to climb to 9.5 billion by 2050. With this steep rise in the population the demand for food also continues to rise. Along with the escalating demand for food is the ongoing trend of food distribution inequality, with world hunger and food insecurity a recurring problem. Malnutrition and poor diet is the leading driver of disease in the world, with malnutrition most prevalent in Asia and Africa. To simply feed the projected global population by 2050 will require a 70% increase in food production.

Biotechnologists are hoping to meet the demand for increased food sources, as well as improve food accessibility for the world's poorest people. However, biotechnology in agriculture has been a debated topic in society. GM crops may have adverse effects on non-target species and soil ecosystems, reduce biodiversity and potentially transfer genes to wild species creating 'superweeds'. Further, they may exacerbate social inequality and financial loss. However many biotechnologists believe that these issues can be mitigated, and that the future of genetic research and technology to improve the global food supply remains optimistic.

GM crop technology is focused on producing more yield on less land, with benefits for society and the environment. Further, new crops with enhanced vitamin and nutrient levels are aiming to lower malnutrition levels globally.

8.3 Biotechnology and biodiversity

LOSS OF BIODIVERSITY

The biotechnology feared to be posing one of the most significant threats to global biodiversity is the use of GM crops in agriculture. It is believed that as more farmers use GM crops, there will be fewer crop varieties grown, reducing the gene pool of some species. If disease or environmental change occurs, there could be widespread and catastrophic effects on food production. This is already an issue in modern agriculture where monocultures and commercial practices have reduced genetic variation. Genetic variation is not only essential for evolution, it is also important for biotechnology-if it is lost, so is the potential for new cultivars of plants and breeds of animals. There is a modern movement to revive heritage varieties of crops and breeds of livestock such as Berkshire pigs (Figure 8.3. la) and heirloom tomatoes. Maintaining the biodiversity of organisms used in agricultural practices ensures the health of future generations of crops and livestock and improves their ability to adapt to changing environmental conditions.

Cross-pollination between GM crops and non-GM crops can further exacerbate the risk to genetic variation; pollen distributed by wind and insects can transfer genes between neighbouring plots of the same species. Cross-pollination may happen in both directions, potentially impacting both types of crop. Uncontrollable pest plant species can then be a threat, with crops that have been modified for herbicide or insect resistance having the potential to breed with wild plants, producing a hybridised pest species that farmers may not be able to control. Plants do not usually pollinate different species, so this is only a potential concern for cross-pollination with wild relatives, such as between crop canola and wild canola. Uncontrollable growth would be a concern if there was a selective advantage to having the genes in a wild environment. A Joss of biodiversity can also occur due to non-target effects of GM plants on beneficial and native organisms such as bees, beetles and moths. Some GM crops have also been known to affect soil ecosystems including microbial populations, decomposition rates and carbon and nitrogen levels.

Another potential threat to biodiversity is the risk of GM animals escaping and interbreeding or competing with natural populations with unforeseen consequences. This may be a potential concern if there are wild relatives of the GM animals and there is a selective advantage to the presence of the transgene (the gene transferred to the organism) .in a wild population (e.g. if GM salmon escaped from aquaculture enclosures adjacent to the ocean and bred with wild salmon). Biotechnology also poses an indirect threat to biodiversity by increasing the value of certain species. For example, if a successful biotechnology that uses a wild species of plant is developed, the value of this plant may increase and put the species at risk of overexploitation.

CONSERVATION OF BIODIVERSITY

While there is concern that some biotechnologies are leading to a loss of biodiversity, many molecular methods and gene technologies are being used to conserve biodiversity. Today's ecosystems are faced with several key threats including habitat loss, climate change and invasive species. Molecular tools are now more easily accessible due to improved ease-of-use and cost-efficiency, and this, combined with a new generation of scientists who are more comfortable using biotechnology, has led to a range of promising new conservation methods.

Traditional conservation methods focus on in situ practices, that is, strategies focusing on maintaining organisms in their natural environment. In situ methods are favoured for their ability to retain the wild, natural state of organisms, along with genetic variation. However, conservationists are facing increasing challenges of habitat loss and fragmentation in ecosystems. As a result, biologists are looking toward ex situ conservation methods that conserve organisms outside of their natural habitat (e.g. in zoos or seed banks). Ex situ conservation methods are being revolutionised by biotechnology and the new molecular techniques being offered.

Plant propagation

As discussed earlier in the chapter, with the human population continuing to grow, GM crops are being increasingly utilised to ensure future food security. Increasing crop productivity, especially in less developed countries, often leads to massive habitat destruction when land is cleared for agriculture. However, biotechnology offers avenues to increase the yields of crops, allowing more productivity on smaller parcels of land. This is particularly promising for biodiverse tropical regions, where land clearing for agriculture poses a significant threat to global biodiversity. GM crops can also be used to reintroduce characteristics from wild crops that have been lost through modern agricultural practices. Ex situ conservation methods are being used to help preserve genetic variation and endangered native plant species, with molecular methods being used to establish seed and gene banks, as well as plant micro-propagation and cloning.

Monitoring genetic variation

With global biodiversity declining at unprecedented rates, conservation of wild animal species requires a targeted approach. Biotechnology provides tools which allow conservationists a wealth of genetic information to better utilise both ex situ and in situ conservation programs. For example, next-generation sequencing (NGS) is being used to determine the pedigree of individual organisms, allowing more informed choices of which individuals to use in breeding programs. The technology also allows biologists to monitor the genetic variation of captive populations, ensuring it is reflecting (or improving) the variation in wild populations. This technique is being used in the captive breeding of endangered northern quolls (Dasyurus hallucatus) to provide greater options for reintroducing quolls to the wild, and to assist setting up a more robust captive population as an insurance policy in case the species becomes extinct in the wild.

DNA sequencing techniques and software are also allowing conservationists to study both functional and neutral genetic variation. Functional genetic variation can provide insight into the adaptive potential of organisms to new conditions such as the ability of captive-bred organisms to survive in the wild, the adaptability of wild organisms to environmental change or the susceptibility of populations to disease. For example, scientists are closely monitoring genes associated with immune response in the Tasmanian devil ( Sarcophilus harrisit) to determine if there are individuals with natural immunity to devil facial tumour disease (DFTD).This knowledge could then help researchers to develop a vaccine.

Heterozygosity

Genetic variation in populations is measured in terms of heterozygosity. This is a measure of the variation (number of alleles) at a locus (region in a DNA sequence). Scientists use computer software to analyse data from a population and provide estimates of genetic variation.

High heterozygosity indicates that the population contains many different alleles (i.e. has high genetic variation), while low heterozygosity indicates that the population contains very few different alleles (i.e. has low genetic variation).

Low heterozygosity is a concern for the evolutionary potential and long-term viability of populations and may signal issues such as genetic bottlenecks and inbreeding. High heterozygosity is generally a positive sign for the evolutionary potential and stability of populations but it may also indicate a sub-division in the population. Sub-divided populations may require taxonomic assessment to determine if the populations are in fact separate sub-species or species.

A SUSTAINABLE FUTURE

Biotechnology is helping to develop more efficient biofuels to decrease the carbon emissions produced from traditional fossil fuel-derived petroleum. Biofuels are renewable alternatives to the fossil fuels used for petroleum and fuel used in transport and industry. Australia has embraced the firs t-generation biofuels made from waste starch, sorghum, cooking oil and other plant and animal waste products. Biofuels have proven to reduce carbon emissions by 85%. Their sustainable nature provides fuel as well as economic security, and as it is waste products that are being used, it does not require more land and does not impact food security.

Current studies are looking at third generation biofuels, which use microalgae to produce an oil which can be refined to gasoline and even diesel fuel. The microalgae have the added benefit of being carbon dioxide consumers, meaning they decrease atmospheric carbon through the production process. Fourth generation biofuels go one step further, looking to develop an engineered biological process to create oil directly from carbon dioxide through the use of an embryonic cell culture. The future of biofuels is also looking to diversify its use and scale, with studies being conducted to produce biofuel jet fuel for military and commercial aviation.

Biotechnology is also providing options to help clean up environmental pollution, through processes of phytoremediation and bioremediation. Phytoremediation is the use of transgenic plants to remove heavy metal pollution from contaminated soils. For example, mercury pollution, which is an ongoing problem has been found to be reducible by producing plants that accumulate the heavy metal in their tissues and are then harvested, allowing the waste to be extracted and disposed of safely. Bioremediation on the other hand uses microorganisms to destroy hazardous contaminants, or convert them to a safer form. Research is looking at new methods to achieve better contact between microorganisms and contaminants and better understand microbial processes to use bioremediation for previously untreatable contaminants.

Chapter 9 Genetic technologies

9.1 Reproductive technologies

Reproductive technologies are used to artificially intervene in processes of sexual and asexual reproduction. Imitating asexual reproduction may include the genetic cloning of a cell or propagating a plant through cuttings. While reproductive technologies such as artificial insemination and artificial pollination are used to improve the chances of pregnancy they are also used to deliberately select the phenotype of offspring. Reproductive technologies can also assist in the screening of disease and genetic conditions that can be transmitted to offspring.

ARTIFICIAL INSEMINATION

Artificial insemination is a reproductive technology that involves the deliberate introduction of male sperm into the female reproductive tract by a method other than sexual intercourse. This reproductive technology is used in animal breeding as well as to help humans conceive a child.

Whether in wildlife conservation or animal husbandry, artificial insemination allows for the expansion of the genetic pool, allowing inbreeding to be avoided and increasing resilience through genetic variation. It also allows for semen samples to be screened for disease and quality, improving reproductive health of the population. However, there are some disadvantages, such as breeders having to detect the best time for female conception, as well as selecting the most valuable male sire for the gene pool.

Animal husbandry

Artificial insemination is a beneficial reproductive technique in animal husbandry (breeding and caring for farm animals) as it allows desirable characteristics from a specific male animal to be passed on to future generations. The process involves the collection of semen from the male and the insertion of fresh or stored sperm into the female reproductive tract under hygienic conditions. The sperm can also be frozen using a technique known as cryopreservation, where the biological samples are cooled to very JO\\' temperatures (usually -135°C). This allows for the long­term storage and transportation of semen without losing viability. Artificial insemination also allows for the impregnation of multiple female animals in different geographic locations, meaning that more offspring can be produced with desirable characteristics than would be possible with normal mating. For example, one semen specimen from a sire (male parent of an animal) can produce thousands of offspring. Further, artificial insemination is a viable breeding option for farmers who often cannot afford or do not want to maintain a large male animal, such as a bull, on their property.

Wildlife conservation

Artificial insemination is also widely used in wildlife conservation to improve the reproductive success of endangered species. Semen samples that have been cryopreserved are often transported between zoos and wildlife parks to introduce new genetic variation to captive breeding programs. This is advantageous as animals do not have to be transported long distances between wildlife centres, which can place a strain on their health and requires teams of specialist animal handlers. Introduction of wild animals to a new potential mate can also result in a lack of natural mating behaviour or even aggression, leading to injury.

Human reproduction

Artificial insemination in humans is referred to as intrauterine insemination (IUI), or assisted reproductive technology. This reproductive technology is often used when a male is unable to engage in sexual intercourse, has low sperm count or when donor sperm is used. However, female fertility issues such as endometriosis can also require the use of this reproductive technology. The semen may be fresh or from a cryopreserved sample. The semen is inserted directly into the woman's vagina, cervix or uterus close to the ovulation phase of her menstrual cycle. The treatment may require medication to stimulate ovulation prior to treatment. Artificial insemination is a less invasive form of fertility treatment than other reproductive technologies and requires little to no recovery time for the female patient and it has very few side effects. Cryopreservation also allows semen to be stored and used in the future; this often allows a couple to conceive a child if the male partner becomes sterile from treatment such as chemotherapy or radiotherapy. Artificial insemination differs from in vitro fertilisation (IVF) which is a reproductive technology which takes the egg and sperm from patients and fertilises them in a specialised laboratory, before transferring the fertilised egg back into the female uterus.

GENETIC SCREENING

Genetic screening is used to detect abnormalities or changes to genes. Screening is used to confirm or rule out suspected genetic conditions, to determine if a person carries a faulty gene that can be passed on to their offspring or whether a person is at risk of developing a disease. Genetic screening can be undertaken from the embryo stage, right up to adulthood.

ARTIFICIAL POLLINATION

Artificial pollination occurs when humans manually facilitate the natural plant pollination process. The pollen is taken from the stamen (male part) of one flower and dusted over the stigma (female part) of another flower resulting in fertilisation and the developments of seeds. These seeds may develop into fruits or new plants. Artificial pollination allows humans to choose pollen from plants with desired characteristics and to spread these genetic traits quickly through a population.

Traditionally, artificial pollination was used for small-scale plant breeding and study. For example, Gregor Mendel used artificial cross-pollination of pea plants to study heredity and genetics in the 1800s. Plant breeders used the reproductive technique to develop new varieties of plants and select for specific characteristics (e.g. pollinating from a specific plant population to ensure the genetic traits for larger fruit are passed on to a new crop). Farmers may use artificial pollination to speed-up the fertilisation process and improve crop yields. This is common in orchard farms where fruit trees such as apples and pears need to be pollinated by a different tree of the same species. This would naturally require the pollen to be carried by the wind or a pollinating animal; however, artificial pollination can quickly achieve the same result. The need for artificial pollination has also risen with the decline in natural pollinators such as honeybees. Some countries such as China have so few natural pollinators that some crops can only continue to be produced through artificial pollination.

Mechanical pollination

Artificial pollination can occur mechanically or by hand. Mechanical pollination involves the mass-dusting of pollen onto plants from small aircraft or large blowers. Although this can be a quick process the accuracy of the pollen reaching plants is limited. Scientists have discovered that natural pollination (bees and other pollinators distributing the pollen directly to the female organs of the flower) is far more accurate than mechanical pollination.

Hand pollination

Hand pollination generally involves the gardener or farmer using a small brush to transfer the pollen from one plant to the stigma of the other plant. This method is more accurate than mechanical pollination, but does take more time and labour. Passionfruit (Passifiora edulis) is a commonly grown home-garden fruit. However, many gardeners complain that the vine never produces any fruit. The reason for this is that the male and female parts of this plant are very far apart and require a pollinator to carry pollen to the pistil (female organs). Home-gardeners will often use a small paint brush to take some pollen from the anthers (male) and dust it onto the female stigma. This fertilises the plant and artificially induces the development of fruit. However, in Brazil, farmers hand­pollinate passionfruit crops due to the decline of the natural bee population from pesticide use in the region. This expensive labour is pushing up the price of fruits and vegetables and causing Brazilians to look to more affordable, but unhealthy, options such as fast-food and fatty meats.

Conservation of plant biodiversity

Artificial pollination is also being used in conservation efforts to save endangered species of plants. Global plant biodiversity is at-risk globally from human-induced threats of habitat destruction and degradation, climate change and invasive species. However, the key ecological interaction of plants and pollinators (Figure 9.1.10) is also under threat, with pollinator species such as bees globally in decline. The extinction of a pollinator species may cause the coextinction of a plant, and the extinction of a plant may lead to the coextinction of a pollinator species. This is known as mutualism disruption, where the mutualistic relationship between two species is disturbed, which can have far-reaching consequences to ecosystem functioning. Studies show that Africa, Asia and the Caribbean are at particular risk of mutualism disruptions. As a result, scientists are turning to artificial pollination to help fertilise endangered plants and increase their populations both in the wild and in botanic gardens.

9.2 Cloning

A clone is a genetically identical copy of a gene, cell or organism. It may occur naturally, as in the case of bacteria and plants which reproduce asexually, or identical twins, but clones can also be created artificially. There are three types of cloning: whole organism cloning, gene cloning and therapeutic cloning.

WHOLE ORGANISM CLONING

Whole organism cloning (also known as reproductive cloning) has many applications in the areas of medicine and agriculture. Whole organism cloning is used in many areas of scientific research where genetically identical test subjects are desired for reliable research outcomes, in animal husbandry and in agriculture where cloning enables desirable traits to be passed onto many offspring in a method faster than traditional breeding. The cloning of plants through techniques such as cutting and grafting has been used both commercially and in home-gardens for centuries to create identical plants. Tissue culture techniques have also developed to clone individual plants.

Whole organism cloning of animals has been a more challenging process. To date, it has been limited to mammals such as cattle, chickens, sheep and dogs. While whole organism cloning has many potential uses, the disadvantages of this reproductive technology include the high cost of breeding, consumer resistance to cloned foods and the higher rate of mortality and health problems in cloned animals. Cloning also raises social, moral and ethical concerns. Human cloning is illegal in most countries, including Australia.

The two methods for whole organism cloning are artificial embryo twinning and somatic cell nuclear transfer (SCNT).

Artificial embryo twinning

Artificial embryo twinning is a technique that m1rmcs the natural process which leads to identical twins where the embryo splits in two very early in the development process and both halves of the embryo continue to divide leading to two separate individuals. As both individuals developed from the same fertilised egg they are genetically identical. Artificial embryo twinning is carried out in a laboratory petri dish. The early embryo is separated into individual cells and allowed to continue dividing. The embryos are then transferred into a surrogate mother.

Somatic cell nuclear transfer (SCNT)

Somatic cell nuclear transfer removes the single set of chromosomes from an egg cell and replaces them with the nucleus of a somatic cell which has two sets of chromosomes) taken from the organism being cloned. The egg cell is then induced to divide as though under the natural fertilisation process. Once the embryo has developed, it is transferred to the uterus of a surrogate female organism. The individual that is born following gestation will be an identical genetic copy of the animal from which the original somatic cell was taken.

Cloning in agriculture

Somatic cell nuclear transfer was the technique used to successfully breed the first 'large cloned animal in 1997. The cloned sheep, named Dolly (Figure 9.2.3a), was bred in Scotland by researchers at the Roslin Institute in Edinburgh. Cells from the udder of an adult sheep were collected and transferred to an egg cell whose DNA (deoxyribonucleic acid) had been removed. The egg was then implanted into a female sheep and, following gestation, a live sheep was born. The lamb was genetically identical to the sheep whose tissues were sampled. Dolly was euthanised in 2003 when a large tumour, due to a viral infection, was found in her lungs. Australia bred its first cloned animals in 2000-Suzi, a Holstein cow, and Matilda, a merino sheep. While Australia has some 100 clones of beef and dairy cattle, as well as sheep clones, products from cloned animals have not been approved as safe for consumption.

Cloning for conservation

Cloning has also been suggested for use in conservation practices to save Critically Endangered species and as a method of de-extinction of species. For example, the northern white rhinoceros (Ceratotheriuni simum cottoni) stands on the brink of extinction with only two females remaining. The last male northern white rhinoceros died in March 2018. The heavily-poached species remain under armed guard in Kenya's 01 Pejeta Conservancy. Scientists have harvested the last eggs from the two remaining females with the plan to use stem cell technology and IVF to create embryos to be surrogated by another rhinoceros sub-species.

GENE CLONING

Gene cloning allows scientists to produce exact copies of a gene of interest. Gene cloning is very different from the cloning of whole organisms. In gene cloning the end product is many copies of a specific gene.1'vvo techniques can be used for gene cloning: in vivo and in vitro methods.

The in vivo technique involves the use of restriction enzymes, ligases and vectors to incorporate the desired gene into the DNA of a living organism, where this gene will replicate. Because the genetic code is universal-the same nucleotides, adenine (A), guanine (G), cytosine (C) and thymine (T), are found in all species-the original gene taken from one organism will express the same protein in the host organism.

In the in vitro gene cloning technique, the polymerase chain reaction (PCR) is used to produce multiple copies of the specific gene. You learnt about PCR in Chapter 6.

Gene cloning has many potential benefits for individuals and society and contributes to important areas in biological research, medicine, food production and industry. Applications of gene cloning include whole genome sequencing, characterising genes (e.g. where they are located, how they are expressed and their biological function), the production of proteins, which are important in medicine and research, the development of transgenic organisms and gene therapy.

DNA and genome sequencing

Gene cloning techniques allow scientists to obtain DNA sequences of different species. The DNA sequences can then be compared to understand evolutionary relationships and the changes in DNA sequences that have led to functional changes over evolutionary time. Using software, DNA sequences of an organism can be pieced together and genes mapped to their location within the genome. DNA sequencing is covered in more detail in Chapter 6.

Characterising genes

Gene cloning and sequencing allows scientists to examine how gene expression may be regulated by other nearby genes or the environment and how mutations can disrupt gene expression and lead to disease. Researchers can also manipulate the expression of cloned genes to understand their functional roles in organisms. For example, researchers have found that mutations in the tumour suppressor gene BRCAJ disrupt normal tumour suppressive activity increasing a person's risk of developing breast cancer. Information about specific genes can be used to develop methods for earlier disease detection and treatments.

Gene therapy

Gene therapy is an application of cloning technology that is used in medicine. Gene therapy refers to the insertion of a gene into an individual's cells to correct or replace defective gene function that leads to disease. Gene therapy is a promising area of research and gives hope to many people living with disease. Gene therapy for disease will be examined in detail in Chapter 17.

THERAPEUTIC CLONING

The somatic cell nuclear transfer (SCNT) cloning technique used to create Dolly the sheep can be used to produce human embryos for therapeutic purposes (i.e. the treatment of disease). Cloning embryonic stem cells for the research and treatment of disease is known as therapeutic cloning (sometimes simply called somatic cell nuclear transfer). Much scientific and ethical debate has surrounded the use of somatic cell nuclear transfer on human cells, both for reproductive purposes and also as a means to extract embryonic stem cells for treatment of certain diseases. Currently, the reproductive cloning of humans is banned due to ethical and scientific objections. Stem cells are unspecialised cells and have the potential to develop into specialised cells such as nerve and muscle cells as well as being used for a variety of functions including growth and repair. Stem cells found in the embryo stage are the most plentiful and versatile. Therapeutic cloning has the potential to overcome problems with immunological rejection of cells and tissues if a patient's own stem cells are used to create the therapeutic cells. Research is currently underway to reprogram cloned adult stem cells. These reprogrammed cells are called induced pluripotent stem (iPS) cells. This would allow patient­specific stem cells to be produced without the need to use human eggs or embryos.

9.3 Recombinant DNA technologies

When DNA from two different sources is joined together, the resulting molecule is called recombinant DNA. Scientists create recombinant DNA to clone a particular gene. Following this step, they may also produce large quantities of the protein expressed by the cloned gene.

USING PLASMIDS AS VECTORS

To introduce foreign genetic material into a cell, scientists often use plasmids as the vector. They insert target DNA into a double-stranded circular plasmid, producing a recombinant plasmid. The plasmid is inserted in a cell, where the self-replicating system of the plasmid and cell replicates the plasmid genes. Each cell containing the plasmid will produce the protein products of the genes in the plasmid, including those of the target DNA. For example, if a recombinant plasmid containing the insulin-coding gene is placed in a bacterial cell, .the cell will produce large amounts of the protein insulin which can be used in the treatment of diabetes.

Plasmids are used as vectors as they are self-replicating, stable and easy to manipulate in a laboratory. Plasmids vary in size and functionality but contain several common features:

•origin of replication (also called ori)-so that the plasmid can continue to replicate independently within the host cell

•at least one restriction enzyme recognition site-so the target DNA can be inserted into the plasmid

•a selectable marker or a reporter gene--most commonly confers antibiotic­resistance or produces a coloured or fluorescent protein so that cells containing the plasmid are selected.

A plasmid containing the lacZ gene used in bacteria is shown in Figure 9.3.2.

Restriction enzyme sites for inserting the gene of interest are located within the lacZ gene. If the gene insertion is successful it will disrupt the lacZ gene and therefore protein expression of this gene. You will learn more about how plasmids are used in a bacterial system later in this section. The plasmid also contains the antibiotic resistance gene amp, which encodes resistance to ampicillin.

Creating recombinant plasmids

The process of creating a recombinant plasmid is outlined below, and in Figure 9.3.3. 1 DNA is isolated from the cell and the target DNA is cut out using a restriction enzyme that will create either sticky or blunt ends. In tile example in Figure 9.3.3 sticky ends are produced.

2 The bacterial plasmid is cut by the same restriction enzyme used to cut out the target DNA. The plasmid and the target DNA now have the same sticky ends with exposed bases that are complementary to each other.

3 The target DNA and plasmids are placed together. Some plasmids will simply close back up (known as non-recombinant plasmids), while other plasmids will incorporate the target DNA by complementary base pairing (known as recombinant plasmids).

4 The enzyme DNA ligase is added to rejoin the sugar-phosphate backbone of the DNA.

Transforming bacterial cells

Bacterial cells that have had foreign DNA incorporated into them are said to be transformed because the cells can express a new gene and therefore has a new characteristic.

Two methods of artificial bacterial transformation are used: heat shock and electroporation.

•Heat shock involves placing bacterial cells and a mixture of recombinant and non­recombinant plasmids in an ice-cold solution containing calcium ions and then rapidly increasing the temperature to disrupt the cell membranes of the bacteria. The plasmids can then penetrate the membrane and enter the bacteria.

•In electroporation, the bacterial cells and a mixture of recombinant and non­recombinant plasmids are subjected to an electrical current that alters the cell membrane. Again, the plasmids are then able to enter the bacteria.

Very few of the bacterial cells will be transformed with recombinant plasmids.

Some will take up the non-recombinant plasmids (plasmids without the target DNA) and others will not be transformed at all or will die from the heat shock or electroporation treatment.

Selection and screening of transformed bacteria

When determining which bacterial cells have been transformed with recombinant plasmids containing target DNA, the characteristics of the plasmid vectors become important. Recall from the earlier example that the plasmid vector contains other genes, including a gene for antibiotic resistance (this example has ampicillin resistance) and a gene that displays a particular phenotype, such as a coloured product.

To determine which of the bacterial cells have been transformed, the cells are incubated at 37°C on agar plates that contain the antibiotic ampicillin. The only bacteria to survive will be those that have taken up the plasmid, whether it is a recombinant or non-recombinant plasmid. These bacteria have the ampicillin resistance gene. Bacteria that have not taken up the plasmid will not have the ampicillin resistance gene and will be killed.

The next step is to determine which bacterial colonies contain the recombinant plasmid. In our example, the plasmid carries the lacZ gene, which codes for an enzyme that breaks down an indicator called X-gal, resulting in a blue product. Bacteria carrying the non-recombinant plasmid with an intact and functioning lacZ gene produce blue colonies on agar plates. If the target DNA has been successfully inserted within the lacZ gene, expression is disrupted and the enzyme coded by this gene is not produced. Therefore, bacteria, transformed with recombinant plasmids appear as white colonies.

Bacteria transformed with the recombinant plasmids are then cultured with nutrients in order for them to replicate and produce the protein encoded by the target DNA.

GENETICALLY MODIFIED AND TRANSGENIC ORGANISMS

As discussed in Chapter 8, humans have used selective breeding to produce animals and plants with more useful or more attractive characteristics since ancient times. They chose those animals or plants that expressed the characteristics they wanted to conserve and selectively bred them together, so that their offspring inherited those characteristics. In the past, selective breeding could only use characteristics that already existed in the genetic pool of a species. We now have the knowledge and skills to use recombinant DNA techniques and techniques to transfer genes from one species to another to produce organisms with DNA combinations never seen before.

Genetically modified organisms (GMOs)

Over the last few decades techniques have been developed that allowed for the alteration of an organism's genome and for the transfer of genes from one organism to another. Because the DNA code is universal, almost any gene transferred from one organism to another will express the protein that it expressed in the original organism. 1'his means that a desirable characteristic seen in one animal or plant could be transferred to another organism lacking this characteristic.

An organism that has had its genome altered in this \\'ay is considered to be a genetically modified organism (GMO). An organism that has had genes from another species inserted is called a transgenic organism. 111e gene that came from another organism is called a transgene. Organisms may have their genome modified by directed mutation (mutagenesis) or by newer technologies called gene editing. These methods may change the genes of an individual without introducing a new gene, so it would be genetically modified but not a transgenic organism.

Transgenic crops

Transgenic crops are used in agriculture to increase crop productivity, provide resistance to insect herbivory and prevent disease. Several genetically modified crops have been developed or are grown in Australia. For example, insect-resistant GM cotton has been grown since 1996, and herbicide-tolerant GM canola was approved for commercial production in Victoria in 2008. In Australia, the Office of the Gene Technology Regulator (OGTR) assesses all GM animals and plants before research, agricultural and commercial use.

Techniques for producing transgenic plants

Transferring a gene into plant cells can be limited by the presence of the cell wall. The introduction of foreign genes into plants is usually done using a biological vector. One method uses Agrobacteriuni tuniefaciens, a soil bacterium that is able to naturally transfer a plasmid into plant cells. A. tuniefaciens normally causes crown gall disease because it carries a plasmid with genes that cause the growth of a tumour. A recombinant plasmid (the vector) carrying a desired gene from a different species, but lacking the tumour-inducing genes, is introduced into A. tuniefaciens cells. When the transformed A. tutnefaciens is cultured with plant cells, the recombinant plasmid can move from the bacterium into plant cells and be directed into the chromosomes of plants. These transformed plant cells are then grown in tissue culture into ne\v plants for transplanting into the field as a transgenic crop.

Salt-tolerant wheat

Soil salinity is a major problem for Australian agriculture. A high level of sodium salts in the soil leads to osmotic water loss from roots and other tissues in which salt accumulates. Cells are stressed due to the altered ratios of sodium and potassium ions in cells. Salt-tolerant plants protect themselves from the effects of salinity by preventing sodium entry into cells, storing the salt in the vacuole or pumping the sodium out of the cells. Scientists have found the genes that control these features of salt-tolerant plants.

To increase crop productivity, Australian scientists from the University of Adelaide introduced a gene from a salt-tolerant Australian native plant into wheat plants. This greatly improved the grain yield of wheat grown on salty soils without affecting grain yield in normal soil. The salt-tolerant gene codes for a protein that removes sodium from the leaves, allowing water to move normally from the roots to the leaves. This increases the geographical range that can be used for wheat production in Australia and other countries facing salinity problems. This is becoming increasingly important as the global population grows.

Bt cotton

Cotton is a plant that attracts many insect pests. To protect the cotton crops, they are sprayed with insecticides up to four times before the crop is harvested. This high use of insecticides impacts the populations of both harmful and beneficial insects as well as the animals that feed on them. Insecticides may also have an impact on human health. In addition, insecticides are expensive.

Bt cotton is a transgenic crop that has been modified to contain two genes from the soil bacterium, Bacillus thuringiensis. Expression of these genes produces proteins in the cotton plant that kill the main caterpillar pest of cotton by disrupting its digestive system. In Australia, almost all cotton grown is Bt cotton and this has reduced the use of pesticides dramatically. This decreases the environmental impacts of pesticides and saves farmers money. Australian regulators have reported no adverse effects over 15 years of Bt cotton use in Australia. Cotton seed oil extracted from Bt cotton can be sold without GM labelling as the extraction processes separate the oil from the plant's proteins and nucleic acids, therefore the oil does not have any GM components.

Transgenic animals

Transgenic animals have been used in scientific research (Figure 9 .3 .13), medicine, pharmaceutical production and agriculture. In research, mice, rats and rabbits are used as living models of biological processes in healthy and diseased states. Scientists can use transgenic animals to study disease progression and potential treatments. For example, scientists studying motor neuron disease have identified a mutation in the gene for the enzyme superoxide dismutase 1 (SODl) that is associated with some inherited cases of this disease. To study the disease in an animal model, scientists produced a transgenic mouse expressing the SOD1 enzyme mutation to establish links between the mutation and disease symptoms to investigate treatments.

Scientists may also produce knock-out mouse models in which gene technology is used to disable or knock-out expression of a particular gene. The phenotype (appearance, behaviour and biological function) of the animal gives the first indications of the function of the protein encoded by the gene. Structural and regulatory genes are studied in this way.

In agriculture, transgenic sheep and cows are used for improved fertility, meat production, milk quality and yield, and wool quality and yield. The use of genetically modified farm animals has not expanded to the extent it has for GM plants, perhaps because of detrimental effects of some modifications in animals. For example, genes that promote growth may also cause altered skeletal growth, arthritis, heart and kidney problems.

To date, GM animals are not approved for human consumption in Australia. Transgenic fish have been approved in the USA, but they are unlikely to be approved for sale in Australia in the near future.

Spider silk protein is an example of a potentially useful product made in transgenic goats. The gene for spider 'dragline' silk has been put into the genome of goats, along with regulatory genes so that it is expressed in the milk. Spider silk is of great interest for its extraordinary strength and flexibility. Potential applications include a biopolymer for artificial ligaments and tendons, bandages, biodegradable bottles and tough bulletproof clothing.

Some animals are being used for the production of therapeutic proteins such as antibodies that are difficult to make in bacteria and cultured cells. This process has been referred to as 'pharming', combining the words farming and pharmaceutical. The products are released into blood or milk from where they can be readily extracted.

Medical researchers are also exploring ways to modify genes in insect vectors of disease and modify pig embryos to make their organs suitable for transplantation into humans, a procedure called xenotransplantation. You will learn more about xenotransplantation in Section 9.4.

9.4 Benefits of genetic technologies

Reproductive technologies, cloning and recombinant DNA technology have a range of applications across fields including 1,vildlife conservation, agriculture and medicine, many of which have the potential to benefit society in the future.

Medicine

Artificial insemination, which is mostly used via intrauterine insemination, allows humans to conceive a child despite challenges including fertility problems or me absence of a sexual partner. It is a relatively inexpensive and non-invasive procedure, allowing many to access reproductive technology.

Cloning holds many potential applications for medicine. One biomedical application is xenotransplantation, which involves me transplantation of cells, tissues or organs from one donor species to humans. Scientists are currently working on me genetic modification of animal sample grafts, allowing mem to be disguised to reduce me likelihood of rejection by me human body. This would be a medical breakthrough in me need for organ donors.

Cloning can produce genetically altered cells mat can be used in me treatment of diseases, such as Parkinson's and Alzheimer's, in which brain cells degenerate. Researchers aim to replace these cells with cloned and modified cells from oilier animals such as pigs. Cloning can also be used to develop genetically modified animals, which more closely mimic the human genome to better test new drugs. For example, many viruses such as HIV do not affect animals the same way as humans, a genetically modified animal would allow scientists to better develop drugs if they are able to monitor human-like responses.

A range of proteins for therapeutic purposes are produced using recombinant DNA technology. Examples include epidermal growth factor used in the treatment of bums to improve the survival of skin grafts, interleukin-2 used in cancer treatment, antibodies for immunotherapy and vaccines against a number of viruses.

Recombinant protein production

Recombinant proteins are produced by introducing recombinant DNA into bacteria or eukaryotic cells and allowing them to synthesise the protein. The main types of proteins produced by this technology are hormones, cytokines, enzymes and vaccines for human therapeutic purposes. This is much safer and more effective than using proteins purified from other organisms, such as the purification of insulin from pigs and growth hormone from human pituitary glands, as was done in the past. Examples of human proteins that are now produced in bacteria, yeast or animal cell culture are viral coat proteins for hepatitis B vaccines, viral capsid protein for human papilloma virus vaccines, cytokines (e.g. interleukin-2) for the treatment of cancer and insulin for the treatment of diabetes.

Agriculture

Artificial insemination is the method for breeding the majority of food production animals in developed countries; it can be used to improve reproductive efficiency and breed more productive livestock. Artificial insemination can also help limit the spread of infectious diseases between animals as samples are screened, and no physical contact between animals occurs. This technique is an inexpensive reproductive technique which allows breeding to occur despite geographic barriers, as well behavioural, physical and physiological challenges. However, researchers have documented a decline in fertility in dairy cattle and horses since the mainstream use of this reproductive technology began.

With the global decline of natural pollinators, such as bees, artificial pollination will play a large role in the future of agriculture, with over 90% of foods dependent on pollination in some form. While hand-pollination is still a popular option in many sectors, mechanical pollination is taking a leap forward with the help of pollinator drones.

Cloning for food production has already been approved in many parts of the world, and some countries have started mass-producing cloned animals. For example, in China, upwards of 500 pigs are being cloned annually at facilities to meet rising food demands. However, many countries including Australia, believe there is not enough research to ensure cloned meat is safe for human consumption.

Industry

Recombinant DNA technology is widely used in biotechnology from its use in the food industry with the production of GM foods and synthetic enzymes such as amylase and lipase, to the development of pharmaceutical products, textile treatments and detergent additives. There is also continued advances in the fields of bioremediation and biofuels. You learnt about these technologies in Chapter 8.

EFFECTS ON BIODIVERSITY

As discussed in Chapter 8, the use of biotechnology can have a significant impact on the genetic variation of individuals, populations and species. A reduction in genetic variation lessens the species' resilience to changes in the environment and disease, as well as reducing the ability of the environment to meet the needs of humans in the future. Reproductive technologies such as artificial insemination and pollination can lead to a reduction in genetic variation by focusing on, and selecting for, fewer varieties and breeds. For example, artificial insemination has allowed for the breeding of Fresian dairy cows in Africa, resulting in greater milk output and economic gain. However, with the introduction of this new population, the native Ankole cattle are becoming threatened. This is a concern as Ankole cattle are well-adapted to the semi-arid conditions of countries such as Uganda, and do not require great amounts of water, feed or veterinary attention. During droughts farmers walked their Ankole cows long distances to water sources, whereas those farmers with Fresian breeds lost their whole herd.

Whole organism cloning poses a risk to biodiversity, with the potential for multiple identical genetic copies becoming widespread in a population resulting in a loss of natural vigour. As discussed in Chapter 8, transgenic and genetically modified species pose a significant threat to genetic variation, with threats to agriculture and the natural environment as a result of:

•selection of a narrow range of cultivars over wild species

•risk of pesticide- and insect-resistant species leading to invasive pests in the natural environment

•reduction of non-target species such as honeybees as a result of GM. However, the use of whole organism and genetic cloning does hold some advantages for biodiversity. Artificial insemination and pollination allow for the introduction of new genes to a population, which can increase the genetic variation in the gene pool. De-extinction methods hold the potential to re-introduce lost genetic variation into a population, restore ecosystem functioning as well as genetic resilience. The use of gene cloning can also be used in GMOs to re-introduce wild varieties of crops.

**Textbook 3: Focus on life**

What is sexual reproduction?

Many young organisms resemble their parents, such as the guinea pig pups shown in Figure 1. Like most animals, each guinea pig pup has two parents—a mother and a father. The genetic material that an organism receives from its parents determines what it looks like and how it functions.

Reproduction in organisms produces new offspring. Sexual reproduction is the production of an offspring that results when the genetic materials from two different cells combine. Half of the genetic material is contained in an egg cell. The other half is contained in a sperm cell. A process called fertilization is the fusing together of a sperm cell and an egg cell. The new cell that forms is called a zygote. A zygote develops into a new organism.

Advantages of Sexual Reproduction

Sexual reproduction is the most common form of reproduction in eukaryotes. As you just read, offspring from sexual reproduction receive genetic material from two cells. Each guinea pig pup in Figure 1 inherited genetic material from the same two parents. Each pup inherited a different mix of genetic material. In a population of guinea pigs, or any other species that reproduces sexually, no two individuals, except identical twins, have the same mix of genetic material. This means that each individual has a different set of traits. The variety of genetic traits in a population of the same species is known as genetic variation.

Genetic Variation When environmental conditions change, genetic variation can help a species survive. Imagine a population of California pop- pies, like those shown in Figure 2. During a drought, poppy plants with traits that enable them to grow in dry conditions are more likely to survive and produce seeds. Plants without these traits might not survive. Because their genetic makeups vary, the population of poppies would include a few plants that could survive a drought.

A lack of genetic variation can cause problems for a species. Suppose every plant in the poppy population had the same genetic traits. If none of those traits enabled the plants to survive hot, dry weather, the entire population might die out.

Selective Breeding Plant breeders and animal breeders often select a male organism and a female organism with certain preferred traits to be the parents of offspring. This method of sexual reproduction is called selective breeding. It produces groups of organisms with similar traits, such as the dogs shown in Figure 3, but reduces genetic variation. Offspring produced by selective breeding can also inherit health problems.

Disadvantages of Sexual Reproduction

Because males produce sperm and females produce eggs, one of the disadvantages of sexual reproduction is getting egg and sperm together for fertilization. Some species, such as the sea urchin shown in Figure 4, produce millions of egg cells or sperm cells and release them into water. This increases the chances that a sperm will find an egg. Other organisms have specific methods that ensure fertilization.

Another disadvantage of sexual reproduction is the time needed for organisms to grow and develop until they can reproduce. For example, male elephant seals, as shown in Figure 4, do not mate until they are about 9–10 years of age. Humans are even older.

Later in this chapter, you will read about types of reproduction that can happen before organisms grow and develop enough to reproduce sexually and do not require fertilization.

Why is meiosis important?

You read in Chapter 1 that genetic material is part of the chromosomes in the nucleus of each eukaryotic cell. Sexual reproduction includes fertilization, which is the combining of the genetic material in a sperm cell and the genetic material in an egg cell.

Meiosis is cell division that produces sperm or eggs from certain reproductive cells in an organism. Meiosis takes place only in eukaryotes that reproduce sexually.

What kinds of cells does meiosis produce?

Meiosis is important because it ensures that a species’ offspring inherit the correct chromosome number. For example, tomatoes inherit 24 chromosomes, humans inherit 46 chromosomes, and chickens inherit 78 chromosomes. Without meiosis, the chromo- some number would double with each generation. Instead of inheriting 46 chromosomes, children would inherit 92 chromosomes, their children would inherit 184, and so on.

Maintaining Diploid Cells

Recall from Chapter 2 that homologous chromosomes are similar in size, shape, and genetic material. A diploid cell in an organism contains pairs of chromosomes that equal the chromosome number of that organism’s species. A dip- loid human cell has 23 pairs of homologous chromosomes. The chromosome number for humans is 46.

Homologous chromosomes are similar, but not identical. For example, each human chromosome in a homologous pair might contain genetic material for eye color. However, one chromosome might have blue eye-color information and the other brown eye- color information.

Creating Haploid Cells

Sperm and egg cells have half the chromosome number of the species. For example, a human sperm cell contains 23 chromosomes—one chromosome from each homologous pair of chromosomes. Similarly, a human egg cell contains 23 chromosomes. A cell that contains one chromosome from each homologous pair is called a haploid cell.

It’s important for offspring to inherit the correct number of chromosomes for their species. Because an egg and a sperm are haploid, the chromosome number of offspring does not double, as described on the previous page. Meiosis ensures that fertilization creates a zygote with the correct number of chromosomes, as shown in Figure 5.

What are the phases of meiosis?

In Chapter 2, you read about the stages of mitosis—the division of a cell nucleus. Meiosis happens in a similar way. For example, before meiosis begins, a cell’s chromosomes replicate, just as they do before mitosis. Recall that each replicated chromosome consists of two identical sister chromatids that are held together near their middles.

In mitosis, division of the nucleus and cytokinesis happen once. In meiosis, division of the nucleus and cytokinesis happen twice. These two processes are called meiosis I and meiosis II. Like mitosis, the phases of each process are called prophase, metaphase, anaphase, and telophase. Diagrams of the phases of meiosis I and meiosis II are shown in Figure 6.

Phases of Meiosis I

**Prophase** I The membrane surrounding the nucleus breaks apart. The replicated chromosomes condense and thicken. Then, unlike mitosis, homologous chromosomes line up close to each other.

**Metaphase I** The pairs of replicated homologous chromosomes form a line along the middle of the cell. Fibers of the cytoskeleton attach to each pair of sister chromatids.

**Anaphase** I Cytoskeleton fibers pull each pair of sister chromatids to opposite ends of the cell. Notice that the pairs of replicated homologous chromosomes separate, but not the sister chromatids. Sister chromatids stay together.

**Telophase I** A membrane forms around each group of replicated chromosomes. Then, the cytoplasm divides to form two daughter cells. Sister chromatids still are together.

Phases of Meiosis II

During meiosis II, the nucleus of each daughter cell formed during meiosis I divides. Then, each daughter cell divides.

**Prophase** II Chromosomes do not replicate before **prophase** II. They remain condensed and thickened.

**Metaphase** II The cytoskeleton fibers move the replicated chromosomes to the middle of the cell.

**Anaphase** II The sister chromatids of each replicated chromosome separate and move toward opposite ends of the cell.

**Telophase** II A membrane forms around each set of chromatids, which now are called chromosomes. The cytoplasm divides, and meiosis II is complete.

What are the results of meiosis?

As you read earlier, meiosis is similar to mitosis, but there are important differences. Some of the similarities and differences are listed in Table 1.

Both meiosis and mitosis begin with one diploid parent cell.

Both meiosis and mitosis take place only in eukaryotic cells. Recall that eukaryotic cells are cells that contain a nucleus sur- rounded by a membrane.

In meiosis, the nucleus of the parent cell divides, then the two newly formed nuclei divide. Mitosis has only one division of a nucleus. Also, mitosis is usually followed by cytokinesis that results in two daughter cells of equal size. Sometimes when meiosis occurs for egg production, the cytoplasm divides unequally during meiosis I. This results in a small cell that breaks down and a large cell that undergoes meiosis II. In meiosis II, the cytoplasm divides unequally again. The larger cell becomes the egg and the smaller cell breaks down.

Plant Reproduction

A plant’s life cycle includes a diploid generation that produces spores and a haploid generation that produces eggs and sperm.

Real-World Reading Connection In early spring, you might see cars or sidewalks covered with something that looks like yellow dust. Where did it come from? It came from some plants that are reproducing. As in all living things, reproduction is part of the life cycles of plants.

What is alternation of generations?

The human body is made primarily of diploid cells. The only human haploid cells are sperm or eggs. As a result, you will live your entire life as a diploid organism. To put it another way, your life cycle includes only a **diploid stage**.

That isn’t true for all organisms. Some organisms, including plants, have two life stages called generations. One generation has primarily diploid cells. The other generation has only haploid cells. Organisms that alternate between diploid and haploid generations have an alternation of generations as shown in Figure 7.

The Diploid Generation

When you look at a tree or a flower, you’re seeing part of the diploid generation of a plant. Meiosis occurs in certain cells in the reproductive structures of a diploid plant. The daughter cells produced from haploid structures are called spores. Spores grow by mitosis and cell division to form the haploid generation of a plant.

The Haploid Generation

In most plants, the haploid generation is tiny and lives sur- rounded by specific tissues of the diploid plant. In other plants, the haploid generation lives on its own. Certain reproductive cells of the haploid generation produce haploid sperm and/or eggs by mitosis and cell division. Fertilization takes place when a sperm and an egg fuse to form a diploid zygote. Through mitosis and cell division, the zygote grows into the diploid generation of the plant.

How are the sperm and eggs produced by the hap- loid generation of a plant formed?

How do seedless plants reproduce?

You’ve probably planted seeds and watched them grow into new plants. But, not all plants grow from seeds. The first land plants to inhabit Earth probably were seedless plants—plants that grow from haploid spores, not from seeds. The mosses and ferns in

Figure 8 are examples of seedless plants found on Earth today.

The life cycle of a moss is typical for some seedless plants. It begins with haploid spores that grow by mitosis and cell division into haploid plants. The tiny, green moss plants that carpet rocks, bark, and soil in moist areas are haploid plants. They have male structures that produce sperm and female structures that produce eggs. Fertilization results in a diploid zygote that grows by mitosis and cell division into the diploid generation. The diploid generation of mosses is tiny and not easily seen. It produces haploid spores by meiosis, and the cycle repeats.

How do seed plants reproduce?

Most of the land plants that cover Earth grew from seeds. Plants that grow from seeds are called seed plants. There are two groups of seed plants—flowerless seed plants and flowering seed plants.

Unlike seedless plants, the haploid generation of a seed plant is within diploid tissue. Separate diploid male and diploid female reproductive structures produce haploid sperm and haploid eggs that join during fertilization.

The Role of Pollen Grains

A pollen grain forms from tissue in a male reproductive structure of a seed plant. Each pollen grain contains nutrients and has a hard, protective outer covering, as shown in Figure 9.

Sperm cells form inside pollen grains. Wind, animals, gravity, or water currents can carry pollen grains to female reproductive structures. Plants cannot move to find a mate like most animals can.

Recall reading about the yellow dust at the beginning of this chapter. That dust was pollen grains. Male reproductive structures produce a vast number of pollen grains. When pollen grains land on a female reproductive structure of a plant that is the same species as the pollen grains, pollination occurs.

The Role of Ovules and Seeds

The female reproductive structure of a seed plant contains one or more ovules. A haploid egg develops inside each ovule. Following pollination, sperm enter the ovule and fertilization occurs.

A seed, as shown in Figure 10, develops from an ovule after fertilization. It consists of an embryo, a food supply, and a protective covering. The embryo is an immature diploid plant that developed from the zygote. A seed’s food supply provides the embryo with nourishment for its early growth.

How do flowerless seed plants reproduce?

Flowerless seed plants are also known as gymnosperms. The word gymnosperm means “naked seed” and gymnosperm seeds are not surrounded by a fruit. The most common gymnosperms are conifers. Conifers, such as pines, firs, cypresses, redwoods, and yews, are trees and shrubs with needle-like or scale-like leaves. Most conifers are evergreen and can live for many years. Bristlecone pines, such as the one shown in Figure 11, are among the oldest living trees on Earth.

Cones are the male and female reproductive structures of conifers. They contain the haploid generation. Male cones are papery and produce pollen grains. Female cones are woody, berry- like, or soft and produce eggs. Seeds form as part of the female cone.

How do flowering seed plants reproduce?

Do you enjoy the fragrance of roses or the bold colors of lilies?

These are examples of flowering seed plants, also known as angiosperms. Most of the plants you see around you are angiosperms. Fruits and vegetables come from angiosperms. Many animals depend on angiosperms for food.

Reproduction and the Flower

Reproduction of an angiosperm begins in a flower. A typical flower has male and female reproductive organs surrounded by petals, as shown in Figure 12. Most flowers have several male reproductive organs but only one female reproductive organ. Some flowers have only male or only female organs.

The male reproductive organ of a flower is the stamen. Pollen grains form at the tip of the stamen, in a structure called the anther. The filament is a long stalk that supports the anther and connects it to the base of the flower.

The female reproductive organ of a flower is the pistil. At the tip of the pistil is the stigma, where pollen can land. The stigma is at the top of a long tube called the style. At the base of the style is the ovary. Inside the ovary is usually one or more ovules. As you read earlier in this lesson, each ovule eventually will contain a haploid egg.

What is an angiosperm’s life cycle?

A typical life cycle for an angiosperm is shown in Figure 13.

Pollen grains travel by wind, gravity, water, or an animal from the anther to the stigma, where pollination occurs. A pollen tube grows from the pollen grain into the stigma, down the style, to the ovary at the base of the pistil. Sperm develop from a haploid cell in the pollen tube. When the pollen tube enters an ovule, the sperm are released and fertilization takes place.

Do sperm develop before or after pollination?

As you read earlier, the zygote that results from fertilization develops into an embryo. Each ovule and its embryo will become a seed. The ovary, and sometimes other parts of the flower, will develop into a fruit that contains one or more seeds. The seeds can grow into new, genetically related plants that produce flowers, and the cycle repeats.

Fruit and Seed Dispersal

Fruits and seeds, including the peas, corn, and strawberries shown in Table 2, are important sources of food for people and animals. In most cases, seeds of flowering plants are contained inside fruits. Green pods are the fruits of a pea plant. The peas nestled inside a peapod are the seeds. A cob is the fruit of a corn plant. The kernels on a cob are the seeds. Strawberries have tiny seeds on the outside of the fruit. We usually think of fruits as juicy and edible, like an orange or a watermelon. However, some fruits are hard and dry, and not particularly edible. Each parachutelike structure of a dandelion is a dry fruit.

Fruits help protect seeds and help scatter or disperse them. For example, some fruits, like that of a dandelion, are light enough to float on air currents. When an animal eats a fruit, the fruit’s seeds can pass through the animal’s digestive system with little or no damage. Imagine what happens when an animal, such as the mouse shown in Figure 14, eats blackberries. The animal digests the juicy fruit, but deposits the seeds on the soil with its wastes. By the time that this happens, the animal might have traveled some distance away from the blackberry bush. This means that the animal helped to disperse the seed away from the blackberry bush.

Plant Reproduction Summary

In this lesson, you learned that plants reproduce sexually. All plant life cycles include an alternation of generations—they alternate between a haploid generation and a diploid generation. Most of the plants you see around you, including trees, flowers, and grasses, are the diploid generation.

Familiar mosses are haploid plants that grew from a haploid spore. The diploid stage is small and often overlooked. Ferns are diploid plants. The haploid stage is small and rarely seen. Conifers and flowering plants are diploid seed plants. The haploid stage of seed plants is surrounded by diploid tissue. Seed plants reproduce by forming seeds. In conifers, seeds form as part of a female cone. In flowering plants, seeds form as part of a flower.

Animal Reproduction

Animals have specialized structures for sexual reproduction.

Real-World Reading Connection Have you ever found a cluster of tiny, beadlike things on the underside of a leaf? What could they be? They might be eggs laid by a butterfly, a ladybug, or some other insect. Eggs are an important part of the life cycle of all animals.

What are animal reproductive organs called?

Animals often have external physical characteristics that distinguish males from females. For example, it’s fairly easy to tell the difference between the male and female animals shown in Figure 15. In mammals and birds, males are often larger or more colorful than females.

Male Reproductive Organs

The reproductive systems of animals include specialized reproductive organs called gonads that produce sperm or eggs. Figure 16 shows the reproductive systems of male and female chickens. Male animals have gonads called testes that produce sperm. Testes contain a network of coiled tubes in which sperm cells form. Sperm have tails that enable them to swim through fluid to reach an egg cell. Most male animals have two testes located inside the body cavity.

What type of cell forms in the testes?

Vertebrate animals—animals with backbones—have an additional adaptation that contributes to successful sexual reproduction. Glands near the testes produce fluid that nourishes sperm and helps them travel from the testes to the eggs of a female.

Female Reproductive Organs

Female animals have gonads called ovaries. Most female animals have two ovaries except female birds, which have only one ovary. Ovaries produce egg cells. Eggs are larger than sperm and cannot move on their own. Many female mammals are born with all the eggs they will ever have. You will read more about the testes and ovaries of humans in Chapter 13.

How does animal fertilization occur?

As you read earlier in this chapter, sexual reproduction requires fertilization—the fusion of a haploid egg cell and a haploid sperm cell that forms a diploid zygote. The way in which a sperm reaches the egg differs among animal species.

Internal Fertilization

When fertilization happens inside the body of an organism, it is called internal fertilization. For many animals, the male has a specialized structure that can deposit sperm in or near a female’s reproductive system. The sperm swim to the egg or eggs. Earth- worms, spiders, insects, reptiles, birds, and mammals have internal fertilization.

Internal fertilization ensures that an embryo, which develops from a fertilized egg, is protected and nourished until it leaves the female’s body. This protection increases the chance that the embryo will survive, develop into an adult, and reproduce.

External Fertilization

A female toad, like the one shown in Figure 17, deposits unfertilized eggs under water. A male toad releases his sperm above the

eggs as she lays them. Fertilization that occurs in the environment, outside of an animal’s body, is called external fertilization. In most cases, the female animal releases eggs into water at about the same time a nearby male animal releases sperm into the water. When a sperm reaches an egg, fertilization takes place. Animals that repro- duce using external fertilization include jellyfishes, clams, sea urchins, sea stars, many species of fish, and amphibians.

Most animals that reproduce using external fertilization do not care for the fertilized eggs or for the newly hatched young. As a result, eggs and young are exposed to predators and other dangers in the environment, reducing their chances of surviving. Successful reproduction of animals with external fertilization requires that a large numbers of eggs be produced. This helps to ensure that at least a few offspring will survive to become adults that repro- duce. For example, a female California red abalone, a species of sea snail, produces about 2.5 million eggs in one breeding season.

How do animal embryos develop?

The zygote produced by fertilization is only the beginning of an animal’s life. The zygote grows by mitosis and cell divisions and becomes an embryo—the next stage in an animal’s life. A growing embryo needs nourishment and protection from predators and other dangers in the environment. Different animals have different ways of supplying the needs of an embryo. In some animals, the embryo develops outside the body of the mother. In others, the embryo develops inside the mother.

External Development

The duck-billed platypus, as shown in Figure 18, is an example of an animal whose embryos develop outside the mother. Animals that develop outside the mother usually are protected inside an egg. In most instances, one embryo develops inside each egg. Most eggs contain a yolk that provides food for the developing embryo.

Some kind of covering surrounds the egg. The covering protects the embryo, helps keep it moist, and discourages predators. Eggs laid by lizards, snakes, and other reptiles have a tough, leathery covering. A tough, jellylike substance usually surrounds eggs laid under water. As shown in Figure 19, bird eggs, like those of a robin, have a hard covering called a shell.

Metamorphosis Some animals—including amphibians and many animals without backbones—go through more than one phase of development. Metamorphosis is a developmental process in which the form of the body changes as an animal grows from the egg to an adult.

The metamorphosis of a ladybug beetle and the metamorphosis of a frog are shown in Figure 20. A ladybug beetle goes through four stages during its development—egg, larva, pupa, and adult.

The tadpole is the larval stage of a frog. Larva and adult forms often have different lifestyles. The larva of the frog lives only in the water. The adult frog can live on land or water.

Internal Development

The embryos of some animals, including most mammals, develop inside the mother. These embryos get nourishment from the mother. An organ or tissue transfers nourishment from the mother to the embryo. Other embryos, such as those of some snakes, insects, and fishes, develop in an egg with a yolk while it is inside the mother. For these animals, the yolk, not the mother, provides nourishment for the developing young. The young hatch from the eggs while they are inside the mother and then leave the mother’s body.

Gestation

The length of time between fertilization and birth of an animal is called gestation. Gestation varies from species to species and usually relates to the size of the animal at birth—the smaller the animal, the shorter its gestation. For example, gestation for a mouse is about 21 days; a dog, about 60 days; humans, about 266 days; and an elephant, about 600 days. A kangaroo is an exception. Gestation for a kangaroo is 35 days. A kangaroo is only about

2.5 cm long at birth, as shown in Figure 21.

Animal Reproduction Summary

In this lesson, you learned how animals reproduce sexually.

Males produce sperm in organs called testes. Females produce eggs in organs called ovaries. Internal fertilization takes place inside the female’s reproductive system. External fertilization takes place out- side the female, in the environment. Embryos that develop inside the body of the female are nourished and protected until they leave the female’s body at birth. Embryos that develop outside the body of the female most often develop inside an egg. An egg has a protective covering and a yolk that provides nourishment to the developing embryo.

Asexual Reproduction

Asexual reproduction produces offspring that are identical to the parent.

Real-World Reading Connection Have you ever joked about wanting to clone yourself? That way, you could enjoy time with your friends while your clone does chores and homework. Do any organisms reproduce by making exact copies of themselves?

What is asexual reproduction?

When we think of reproduction, we usually think of sexual reproduction. Recall that sexual reproduction occurs when a sperm and egg fuse, and it usually requires two parents. However, many organisms have only one parent.

Asexual reproduction is the production of offspring by one parent without a sperm and an egg joining. Asexual reproduction results in offspring that are genetically identical to the parent organism. The poinsettias shown in Figure 22 were produced by asexual reproduction.

Advantages of Asexual Reproduction

Unlike sexual reproduction, asexual reproduction does not require a mate. Therefore, an asexually reproducing organism does not have to spend time and energy finding a mate. Also, to reproduce a number of offspring asexually takes less time than to reproduce the same number of offspring sexually. Since parent and offspring are genetically identical, both are equally well adapted to the same environmental conditions.

Disadvantages of Asexual Reproduction

The major disadvantage of asexual reproduction is the lack of genetic variation. Recall that genetic variation in a population increases the chances that a few individuals will survive a change in the environment. Imagine a population of poinsettia plants growing in a greenhouse, like those shown in Figure 22. Suppose a greenhouse worker unknowingly plants the poinsettias in soil contaminated with disease-causing bacteria. Because the poinsettias are genetically identical, the disease might affect all of them. The entire poinsettia population might be destroyed.

Another disadvantage of asexual reproduction involves genetic changes, or mutations, that can occur. A harmful mutation in cells of an organism will be passed to asexually reproduced offspring.

This could affect the offspring’s ability to survive.

What are the types of asexual reproduction?

There are many types of asexual reproduction. However, each type involves cell division. Prokaryotes, or bacteria, reproduce

asexually by cell division that does not involve mitosis. Eukaryotes reproduce asexually by mitosis and cell division.

Fission

A bacterium has a small, circular DNA chromosome but no nucleus. Bacteria reproduce asexually by a process called fission, as shown in Figure 23. Fission produces two genetically identical cells. Asexual reproduction by fission can occur rapidly. E. coli, a species of bacteria found in human intestines, can reproduce asexually every twenty minutes.

Mitotic Cell Division

Some single-celled eukaryotes, such as the diatom in Figure 24, reproduce asexually by mitotic cell division—mitosis followed by cell division. Recall from Chapter 2 that mitosis and cell division produce two genetically identical cells from one cell. Asexual reproduction in a single-celled eukaryote also produces two identical cells, except that each cell is an individual organism.

Budding

Yeasts are single-celled eukaryotes that are related to mush- rooms and other fungi. As shown in Figure 25, yeast cells repro- duce by budding—a type of asexual reproduction in which a new organism forms on the parent organism. The new organism is called a bud and forms by mitosis and cell division. It is genetically identical to the parent and eventually separates from the parent to live on its own.

Some multicellular animals, such as freshwater hydra also shown in Figure 25, can reproduce by budding. Notice how the bud grows from the stalk of the parent hydra. Like many multi- cellular eukaryotes, including plants, hydras can reproduce both sexually and asexually.

Plant Cuttings

In Lesson 3, you read how plants reproduce sexually. Many plants can also reproduce asexually. If you cut a green stem from a houseplant and place it in water, roots and leaves can grow, producing a new plant. A new plant grown from a stem cutting is genetically identical to the parent plant. Poinsettias, like those in Figure 22, can be grown from stem cuttings. Depending on the type of plant, leaf cuttings or root cuttings can also be used to grow a new plant.

Some plants propagate themselves asexually. The kalanchoe plant shown in Figure 26 has produced tiny plantlets at the edges of its leaves. These plantlets can fall to the ground, take root, and produce new plants. A strawberry plant, as shown in Figure 26, produces new plants along horizontal stems that grow on the sur- face of the ground.

Animal Regeneration

Recall from Chapter 2 that the process in which cells in an embryo become different types of cells is called cell differentiation.

But as you just read, some plant cells can dedifferentiate—change from a differentiated cell type and grow into other cell types. You also read in Chapter 2 that differentiated human cells cannot change and grow into other cell types. However, some animals have cells that can change into other cell types.

Producing New Animals Asexual reproduction that produces new animals from pieces of an animal’s body is called regeneration. The sea star shown in Figure 27 can asexually reproduce by regeneration. If it is broken into pieces, each piece can grow the missing parts. The new organisms are genetically identical.

Producing Body Parts Sometimes, the term regeneration is used to describe growth that replaces a missing part of an animal. For example, if a sea star just loses part of an arm, it can regrow that lost part. The regrowth of a body part is not an example of asexual reproduction, because a new individual is not created.

What is cloning?

Fission, budding, and regeneration are all types of asexual reproduction that produce genetically identical offspring in nature. In the past, the term cloning described any process that produced genetically identical offspring. Today, however, cloning usually refers to a method of asexual reproduction developed by scientists and performed in laboratories. Cloning produces identical individuals from a cell or from a cluster of cells taken from a multicellular organism.

Plant Cloning

Plant tissue culture is a cloning technique that enables scientists to produce genetically identical plants from a few plant cells grown in a test tube, as shown in Figure 28. Plant tissue culture can be used to produce thousands of identical plants from a plant that has desirable genetic traits, such as high nutritional value or rapid growth.

Animal Cloning

The first animal to be successfully cloned from an adult cell was a sheep. In 1996, scientists in Scotland cloned a female sheep that they named Dolly. Figure 29 illustrates the reproductive methods used to make Dolly. Since then, scientists have cloned other animals, including mice, cows, and a horse.

Although several cloned animals have been produced success- fully, they often are not as healthy as animals produced by sexual reproduction. Some animal clones, including Dolly, have had a much shorter life span than animals produced by sexual reproduction. Animal cloning raises ethical issues that people are concerned about. For example, many people think the cloning of humans should never be allowed. You might be asked to consider issues like this during your lifetime.

Asexual Reproduction Summary

In this lesson, you read that many organisms reproduce asexually, which means they have only one parent. Offspring from asexual reproduction have genetic material that is identical to the genetic material of the parent. Bacteria reproduce asexually by fission, which is a type of cell division. Some eukaryotes, including yeast, reproduce asexually by budding. Other eukaryotes repro- duce asexually by mitosis and cell division. Parts of plants, such as stem cuttings or root cuttings, can grow into new plants. In some animals, a body part can regenerate and form a new individual.

Cloning is a scientific process that produces offspring genetically identical to one parent organism.

Foundations of Genetics

Gregor Mendel discovered the basic principles of genetics.

Real-World Reading Connection Why are all dogs of the same breed similar? Why do some disorders run in families? Why do grapevines produce only one type of grape? People have asked questions like these for many years, but scientists have found the answers only recently.

Early Ideas About Heredity

In Chapter 3, you read that a sperm and an egg contain genetic material that combines at fertilization. The combined genetic material determines the traits or features of an offspring. The passing of traits from parents to offspring is called heredity (huh REH duh tee).

Have you ever mixed two paint colors to get a new paint color? People used to believe that the genetic material from a sperm cell and an egg cell mixed like colors of paint. They believed that because offspring resembled both parents, the genetic material mixed or blended. Blending inheritance is the idea that offspring are a blend of genetic material from both parents. Supporters of this idea proclaimed that, over many generations, populations would eventually look alike because of blending inheritance. This does not happen. Blending inheritance also cannot explain why some traits appear to skip generations, such as eye color, as shown in Figure 1. Because of the work of Gregor Mendel, these questions have new answers.

Gregor Mendel and His Experiments

The first person known to record evidence that traits of organ- isms are determined by factors passed from parents to offspring was Gregor Mendel. He was born in 1822 in a part of Europe that is now the Czech Republic. Mendel made his discoveries during the 1850s at a monastery, as shown in Figure 2. The monks at the monastery were dedicated to teaching science and scientific research.

Mendel experimented with garden pea plants in the monastery’s gardens. After carefully analyzing the results of his experiments, Mendel established the basic laws of heredity. Because of his scientific research, Mendel is known as the father of genetics. Genetics is the study of how traits of organisms are passed from parents to offspring.

Mendel’s Experimental Methods

Mendel studied genetics by conducting breeding experiments with pea plants. He chose pea plants because they are easy to grow, they flower and reproduce quickly, they come in many varieties, and the peas are edible. Mendel studied seven different traits of pea plants, as shown in Table 1. Each trait had only two variations. For example, flower color was either purple or white and seeds were either round or wrinkled.

Controlled Exeriments Recall from Chapter 3 that a flower contains male reproductive organs (stamens) and/or female reproductive organs (pistils). The flowers of pea plants have both stamens and pistils. Mendel controlled fertilization in the experimental pea plants. He allowed some of the flowers to self-fertilize as they usually do in nature. Mendel also performed cross-fertilization by transferring pollen from the stamen of one pea flower to the stigma of a pistil in another pea flower, as shown in Figure 3.

This allowed him to record the parents of the offspring produced from the cross, and observe how traits pass from one generation to the next.

Mendel’s Unique Methods Mendel was not the first person to breed plants. So, what made his experimental methods unique? First, Mendel used true-breeding plants for each trait. When true- breeding plants for a trait self-pollinate, they always produce off- spring with that trait. For example, when pea plants that are true-breeding for wrinkled seeds self-pollinate, they only produce offspring with wrinkled seeds. Second, Mendel recorded the inheritance of traits for several generations. Last, and most importantly, Mendel used a mathematical approach. He was careful to breed large numbers of plants and count the number of each kind of off- spring, generation after generation. He collected and recorded large amounts of numerical data.

Mendel’s Experimental Results

After analyzing the results of his experiments, Mendel concluded that two factors control each inherited trait. He also pro- posed that when organisms reproduce, each gamete—sperm or egg—contributes one factor for each trait.

Dominant Factors Mendel often crossed true-breeding plants to create hybrids. A hybrid inherits a different form of a specific trait from each parent. For example, when Mendel crossed a true- breeding, purple-flowered plant with a true-breeding, white-flowered plant, the hybrid offspring had purple flowers, as shown in Figure 4. Why were there no white flowers? Mendel hypothesized that the offspring had one genetic factor for purple flowers and one genetic factor for white flowers, but only the purple factor is observed because it blocks the white factor. A genetic factor that blocks another genetic factor is called dominant. A dominant trait, such as purple flower color in pea plants, is observed when offspring have one or two dominant factors.

Recessive Factors A genetic factor that is blocked by the presence of a dominant factor is called recessive. A recessive trait, such as white flower color in pea plants, can be observed only when two recessive genetic factors are present in offspring.

Mendel’s Laws of Heredity

Because Mendel did many experiments and carefully recorded the results, he was able to form two important hypotheses that allowed him to predict how traits are inherited. His hypotheses have been supported by other scientists and are called Mendel’s laws of heredity—the law of segregation and the law of independent assortment.

According to the **law of segregation**, the two factors for each trait segregate or separate from each other during meiosis when gametes form. You can review meiosis in Chapter 3.

Mendel’s **law of independent assortment** states that the factors for one trait separate independently of how factors for other traits separate, and gametes have all possible combinations of traits. For example, the separation of the two factors for seed color does not affect how the two factors for seed shape separate. The independent separation of the factors for these two traits makes four possible combinations of traits, as shown in Figure 5.

Modern Definitions of Mendel’s Ideas

Mendel did not know about DNA or how cells reproduce, yet his ideas about inheritance remain true today. However, the terms used to describe his ideas have changed over time.

Genes and Alleles

Recall that a chromosome is made up of DNA and proteins. A section of DNA that has information about a specific trait of an organism is called a gene (JEEN). However, the gene’s information about a trait can vary among the same kind of organisms.

Recall that Mendel used pea plants with purple or white flowers. Each pea plant had a gene for flower color, but each plant’s gene for flower color had either purple or white information. In a similar way, we all have genes for eye color, but we all do not have the same information about the color of our eyes. Each form of a gene with different information is called an allele (uh LEEL). Mendel called these factors instead of alleles. As shown in Figure 6, scientists now know that the alleles of a gene are at the same locations on a pair of homologous chromosomes.

Phenotype and Genotype

How would you describe the dog in Figure 7? You might say the dog has a lot of hair, short legs, and a wrinkled face. These observable traits and all characteristics of an organism make up the organism’s phenotype. You read in Chapter 2 that an organism can have different levels of organization—organ system, organ, tissue, and cell. Each level of organization has a phenotype. A dog’s phenotype includes not only its physical appearance, but also how its organs function, how it reproduces, and many other characteristics.

An organism’s phenotype results from the interactions among its alleles and genes. In the next lesson, you will read that the environment can influence phenotype, too. The alleles of all the genes on an organism’s chromosomes make up the organism’s genotype. An organism’s genotype can refer to one or more genes. The alleles of a particular gene are that gene’s genotype.

Homozygous and Heterozygous Genotypes Because eukaryotic organisms have pairs of chromosomes, a genotype for a gene has two alleles. If the two alleles have the same information, their genotype is called homozygous, as shown in Figure 8. If the two alleles for a gene have different information, their genotype is called heterozygous.

Representing Genotypes Also shown in Figure 8 are the possible genotypes for the smooth-pea phenotype—SS and Ss. Uppercase letters represent dominant alleles, and lowercase letters represent recessive alleles. Both of these genotypes result in a smooth phenotype because the S allele is dominant over the s allele. The wrinkled phenotype is possible only if the two recessive alleles—ss—are present.

Law of Segregation Explained

The movement of chromosomes during meiosis explains Mendel’s law of segregation. Recall that in meiosis I, replicated homologous-chromosome pairs separate from each other. Then, each set of sister chromatids separates into different gametes during meiosis II, as shown in Figure 9. Each gamete only receives one allele.

Law of Independent Assortment Explained

In Mendel’s law of independent assortment, he hypothesized that the separation and movement of two factors for a trait is independent of the separation and movement of the factors for other traits. This can also be explained by the movement of chromosomes during meiosis. The daughter cells produced by meiosis receive only one chromosome from each pair of homologous chromosomes. As shown in Figure 10, a daughter cell might receive a chromosome with an A allele from one pair and a chromosome with a B allele from the other pair. On the other hand, a daughter cell might receive a chromosome with an a allele from one pair and a chromosome with a b allele from the other pair. This results in four possible allele combinations for two homologous pairs of chromosomes.

Importance of Mendel’s Genetic Studies

You might be surprised to learn that Mendel’s discoveries were unnoticed for about 35 years. In the 1860s, no one knew about the existence of chromosomes or about the process of meiosis. There- fore, it might have been hard for others to understand Mendel’s discoveries. However, scientists doing background research for their genetic studies rediscovered Mendel’s work early in the 1900s. All the research of modern genetics is based on Mendel’s conclusions from his work with pea plants.

Mendel’s Studies: A Summary

Mendel’s discoveries about how traits are inherited paved the way for future genetic scientists. Below are **Mendel’s principles** about genetics that are true today for many characteristics.

An individual has two sets of factors (alleles) for each trait—one from each parent.

A factor (allele) might not be observed in one generation if it is recessive and masked by a dominant allele.

The two factors (alleles) for each trait can be the same or be different (heterozygous).

The two factors (alleles) for each trait segregate or separate from each other during meiosis—the law of segregation.

The separation and movement of the two factors (alleles) for a trait during meiosis is independent of the separation of the factors for other traits—the law of independent assortment.

Understanding Inheritance

The interactions among alleles, genes, and the environment determine an organism’s traits.

Real-World Reading Connection Have you ever wondered why your nose or lips are shaped the way they are? How can scientists determine if you are at risk for carrying alleles for any genetic disorders, such as sickle cell disease? Scientists now have tools to answer these questions and study patterns of human inheritance over many generations.

Modeling Inheritance

Plant breeders and animal breeders need a way to predict how traits will appear in offspring. Two tools—a Punnett square and a pedigree—can be used to identify and predict traits among genetically related individuals, such as the family shown in Figure 11.

Punnett Squares

If the genotypes of the parents are known, then the different genotypes and phenotypes of the offspring can be predicted. **A Punnett square** is a model used to predict possible genotypes and phenotypes of offspring.

One-Trait Model Figure 12 shows a Punnett square of the possible offspring of two parents—a true-breeding pea plant for yellow seeds and a true-breeding pea plant for green seeds. Y represents the dominant allele for yellow seeds, and y represents the recessive allele for green seeds. The Punnett square shows that the only possible genotype for hybrid offspring is heterozygous—Yy. The true-breeding pea plant for yellow seeds only can contribute gametes that have a Y allele. The true-breeding pea plant for green seeds can only contribute gametes that have a y allele. All, or 100 per- cent, of the offspring will have the genotype Yy. The phenotype of the genotype Yy is yellow seeds because Y is dominant to y.

**Hybrid-Cross Model** What would a Punnett square look like if two of these hybrid offspring were crossed? The second Punnett square, also shown in Figure 12, is the cross between two organisms with heterozygous genotypes—Yy and Yy. The possible offspring from this cross includes three different genotypes but only two phenotypes. We expect that three-fourths, or 75 percent, of the offspring from this cross will have yellow seeds and one-fourth, or 25 percent, will have green seeds. In other words, the probability is that for every four seeds, three should be yellow, and one should be green. This can be stated as a 3:1 ratio. However, you cannot expect that every group of four seeds will consist of three yellow seeds and one green. When studying genetics, a large number of offspring need to be counted in order to get accurate results, as Mendel determined during his experiments.

The more individuals counted, the closer the actual numbers will be to the predictions.

Pedigrees

All genetically related members of a family are part of a family tree. A pedigree shows genetic traits that were inherited by members of a family tree, as illustrated in Figure 13. A pedigree usually only indicates the phenotype of individuals. The genotypes of the individuals in a pedigree might not be known but can often be determined. In a pedigree, circles represent females, and squares represent males. Connecting lines indicate relationships among members of the family tree. A line connects a set of parents.

Branching lines below a set of parents indicate their offspring. The inheritance of attached and free earlobes in two generations of a family is shown in Figure 13. Besides tracking common inherited traits, pedigrees are important tools to track complex patterns of inheritance and genetic disorders in families.

Complex Patterns of Inheritance

By chance, Mendel studied traits only influenced by one gene with two alleles. However, we know now that some inherited traits have complex patterns of inheritance.

Types of Dominance

You read in Lesson 1 about dominant alleles and recessive alleles. Recall that for pea plants, the presence of one dominant allele results in a dominant phenotype. Not all allele pairs, however, have a dominant-recessive interaction.

Complex Patterns of Inheritance

By chance, Mendel studied traits only influenced by one gene with two alleles. However, we know now that some inherited traits have complex patterns of inheritance.

Types of Dominance

You read in Lesson 1 about dominant alleles and recessive alleles. Recall that for pea plants, the presence of one dominant allele results in a dominant phenotype. Not all allele pairs, however, have a dominant-recessive interaction.

Incomplete Dominance Sometimes traits appear to be blends of alleles. Alleles show incomplete dominance when they produce a phenotype that is a blend of the parents’ phenotypes. For example, a pink camellia, as shown in Figure 14, results from incomplete dominance. A cross between a white camellia flower and a red camellia flower only produces camellia plants with pink flowers.

Codominance The human blood type AB is an example of another type of interaction between two alleles. When both alleles can be observed in a phenotype, this type of interaction is called codominance. If an individual inherits the B allele from one parent and an A allele from the other parent, he or she will have type AB blood, not type A blood or type B blood.

Multiple Alleles

Some genes only have two alleles. However, there are genes that have more than two alleles, or multiple alleles. Besides codominance, the human ABO blood group also is an example of a trait that is determined by multiple alleles. There are three different alleles for the ABO blood type—IA, IB, and i. The IA and IB alleles are codominant to each other, but both are dominant to the

i allele. Even though there are multiple alleles, a person can only inherit two of these alleles—one from each parent, as shown in Table 2.

Sex-Linked Inheritance

Recall that we have 23 pairs of homologous chromosomes in the cells of our bodies, except for sperm and egg cells that have only one chromosome from each chromosome pair. Most pairs of homologous chromosomes are of equal size with one exception— the long X and short Y pair, as shown in Figure 15. Chromosomes X and Y are the sex chromosomes because they contain the genes that determine a person’s gender or sex. With the exception of sperm and eggs, each cell in a male has an X chromosome and a

Y chromosome, and each cell in a female has two X chromosomes. Because the Y chromosome is shorter than the X chromosome, many genes on the X chromosome are not on the Y chromosome.

Therefore, each of those genes has only one allele, not two. It is the allele on the X chromosome. Recall that a recessive phenotype usually is observed only if the genotype is homozygous. However, a recessive phenotype is observed in a male when a one-allele gene on his X chromosome has a recessive allele. That’s why males are more likely than females to have X-linked recessive conditions, as shown in Figure 16.

Polygenic Inheritance

Some traits are determined by only one gene, but one gene can affect more than one trait in an organism. In fact, many traits result from the interactions of more than one gene. Polygenic inheritance is when multiple genes determine the phenotype of a trait. When several genes determine a trait, many alleles affect the phenotype even though each gene has only two alleles. There- fore, many phenotypes are possible when polygenic inheritance determines a trait, such as the color of horses, as shown in

Figure 17. Height, weight, and skin color in humans are examples of characteristics that are determined by polygenic inheritance.

Maternal Inheritance

When discussing human heredity, we usually only talk about the DNA that makes up the chromosomes in the nucleus in each of our cells. But, you read in Chapter 1 that mitochondria contain DNA too. Mitochondria are scattered throughout the cytoplasm of cells, including an egg cell, but a sperm cell only has mitochondria in its tail. A sperm’s tail does not enter the egg during fertilization. As a result, humans inherit mitochondrial genes, such as the genes involved in making ATP during cellular respiration, only from their mothers. This means that the inheritance of traits related to mitochondria can be traced from a grandmother to her children and then to her grandchildren.

Human Genetic Disorders

Imagine that you are putting a bicycle together. You have all the right parts, but the directions say to put the handlebars on the seat post. If you did this, your bicycle could not function the way it should. A similar thing can happen if a mutation, or a change to a gene, occurs. The organism with the mutation cannot function as it should.

An inherited mutation can result in a phenotype called a genetic disorder. A genetic disorder can result in minor or major health problems and sometimes shorten a person’s life. A common genetic disorder among Caucasians is a recessive disorder called cystic fibrosis. People with cystic fibrosis have tissues that produce abnormally thick mucus. This thick mucus can affect the functions of the respiratory, digestive, and reproductive systems. Other human genetic disorders are listed in Table 3.

Genes and the Environment

You read earlier in the chapter that the genotype determines the phenotype. Scientists have determined that genes are not the only factors that can affect phenotypes. An organism’s environment can also affect its phenotype.

Many genes affect a person’s chances of having heart disease.

However, what a person eats and the amount of exercise he or she gets can influence whether heart disease will develop. Also, your genotype for skin color determines the amount of pigment in your skin. But, long exposures to sunlight can temporarily change your skin color. Other examples of how environmental factors can affect phenotype are shown in Figure 18.

Natural Selection

Charles Darwin developed a theory of how organisms with the same ancestors can look and behave differently over time.

Real-World Reading Connection Did you know that there are more species of insects on Earth than any other type of organism? Did you ever wonder why there are so many different species? Charles Darwin thought about this very question as he explored some of life’s most remarkable diversity around the world. It was Darwin who would uncover an important process that accounts for the great variety of species seen today.

Charles Darwin

Evolution is change over time. Modern scientists refer to evolution as genetic change in a population over time. Charles Dar- win was not the first person to talk about evolution, but he was the first person to write the most thorough collection of evidence supporting evolution.

Charles Darwin was a naturalist, a person who studies the natural world, including plants, rock formations, and animals. During his extensive traveling and research on the HMS Beagle, shown in Figure 1, he developed an important theory about how organisms evolve. His revolutionary theory, which is described in more detail later in this lesson, helps explain the unity and diversity of life. Darwin’s theory also transformed the natural sciences and serves as the basis of much biological research today.

Voyage of the Beagle

In December 1831, Charles Darwin was 22 when he accepted	an invitation to serve as a naturalist on the British naval ship	called the HMS Beagle. The Beagle sailed from England to South

America and other parts of the world to make navigational maps. The voyage of the Beagle would last for almost five years. Examine Figure 2 and follow the route the Beagle traveled.

Darwin spent time exploring South America and its remote islands, such as the Galápagos Islands. The Galápagos Islands are located about 1000 km off the coast of South America, as shown in Figure 2. Darwin spent most of his time observing nature, collecting samples of new plants and animals, and writing about places and organisms few had ever seen.

Darwin’s Observations

Darwin made extensive observations and detailed notes of the biology and geology of the locations he visited. Collecting numerous samples, some of his most interesting findings were of the diversity and uniqueness of the organisms he saw, especially on the Galápagos Islands. He noticed many new animals and plants that had not been recorded prior to this trip. He became particularly fascinated in comparing the similarities and differences among the animals and fossils of animals from the different islands.

When he compared them, many of the turtles, birds, and lizards on the Galápagos were similar, but not the same as organisms that he found in the South American mainland. Darwin reasoned from these observations that perhaps some of the animals and plants on the Galápagos originally came from South America, and over time, they evolved to be different.

Tortoises During his travels, Charles Darwin made interesting observations of the giant Galápagos tortoises, or land turtles. He found tortoises on all of the Galápagos Islands and nowhere else that he visited. The tortoises were enormous in size. What was most interesting to Darwin was that the tortoises varied from island to island in the Galápagos. Darwin wondered why all the tortoises were different from each other even though they lived on islands only 80 km apart.

Look at Figure 3 and notice the unique shape of the shells found in the two different tortoises. Darwin found that tortoises had dome-shaped shells and short necks in places where they primarily ate low-growing plants. But, tortoises that fed on high-growing cacti had saddle-shaped shells that seemed to allow their longer necks to reach the cactus pads. Darwin reasoned that perhaps the different tortoises “descended with modification” from an ancestral population in South America. Each generation of the population would change genetically until a trait for long necks evolved.

Finches Darwin also made important observations of birds called finches on the Galápagos Islands. These birds were not the same as the birds he observed in other parts of the world. He described 13 finch species, although at the time he thought they were all the same species.

Like the tortoises, Darwin was impressed by the diversity of finches on the different islands, as shown in Figure 4. He was most fascinated by the diversity of beak size and shape in the finches, as shown in Figure 4 and Figure 5. Beaks ranged from small to large and each beak type was well-suited for eating a particular food.

For example, the large ground finch shown in Figure 4 has a large beak that was well-suited for cracking open large seeds on the ground. The small tree finch in Figure 5 has a long and narrow beak that was compatible for catching insects in the trees. Darwin would later explain that if individuals from an ancestral species in South America were separated for a long enough period of time, the future generations or descendants on the different islands might look and behave differently. Thus, in a similar way to the tortoises, the finches became different because genetically, they fit the different habitats on the islands. Figure 5 indicates the differences in beak size and shape in all of Darwin’s finches. Notice how the beak size and shape is related to the kind of food the finch eats and where it must go to get the food. Ground finches need a large beak to break and eat seeds on or near the ground. Small tree finches have smaller beaks to catch insects in the air.

Selective Breeding

For thousands of years humans have been breeding plants and animals. In plants the goal might be to get the largest fruit, the best taste, the tallest plant, or the prettiest flower. In animals farmers want the cow with the most milk, the largest hog, or the fastest horse. You can probably think of other traits you might want in dogs or cats. When a plant or an animal is bred to get these characteristics, it is known as selective breeding. Selective breeding does not lead to a new species. The organism is still able to breed with other members of its species. Dogs have been bred for many special features, such as size and fur length. Even so, crosses between breeds still produce animals with doglike characteristics. Figure 6 shows some pigeons that have been selectively bred. The original species of these pigeons is the rock dove.

Darwin’s Theory of Natural Selection

Darwin and other scientists realized that organisms with a trait that allowed them to survive under particular environmental conditions produced more offspring than those organisms without this trait. Eventually, these traits would become very common in a species.

Genetic Variation

Recall from Chapters 3 and 4 that traits are passed on to the offspring from father and mother. Traits are sorted during the process of gamete formation. Sometimes changes occur in the genes and new traits are created. Figure 7 shows the genetic variation of some common flowers. If a trait is harmful, the organism might die, but if it is beneficial, the organism is more likely to survive and reproduce. That trait will then be passed on to the next generation. With time, it will become common in many populations of the species. Genetic variation is necessary for evolution to occur. But, survival depends on other factors as well.

The Struggle to Survive

One influence on Darwin’s ideas was an essay by Thomas Mal- thus, which presented the argument that if the human population were to continue to grow unchecked, eventually humans would run out of food and space. There would be a “struggle to survive.”

Population Growth Darwin too had noticed that animals often produced more offspring than could survive. Limited resources, such as food, water, and habitat, allowed for only some individuals to survive. Darwin decided that this was a natural process that selected which organisms could survive. Darwin’s next step was to find out how the organism was selected. He reasoned that an organism that was better prepared to get food or protect its space would be better able to survive. You have read about niches and the competition within those niches. Figure 8 shows competition between two animals. The animal that is best suited to the environment will ultimately win the competition.

Environmental Factors Different habitats can put pressure on animals to survive as well. Darwin suggested that those organisms best prepared for living in specific habitats would survive and be most able to reproduce. Their offspring would also be able to survive in the specific habitat.

Natural Selection

Darwin defined natural selection as a process by which individuals with traits that better suit the environment are more likely to survive longer and reproduce more successfully than those individuals without these traits. Inherited traits that increase an organism’s chance of surviving and reproducing in a particular environment are called adaptations. Adaptations spread through a population in future generations if natural selection favors them.

Darwin spent a great amount of time working out how natural selection can lead to adaptation within a species and to the evolution of a new species. He proposed that if groups of organisms with common ancestors are isolated from other organisms of the same species, natural selection can cause them to become different over generations. The isolated groups adapt to different environ- mental conditions and this process can develop a new species.

With enough time, this could explain the large number of species on Earth today.

Darwin’s **theory of evolution** by natural selection can be best explained by the following four steps or requirements.

1	Overproduction Organisms produce more offspring than can be supported by the available food, water, and shelter in an environment. Many will die due to natural events such as predation, competition, and starvation.

2	Variation Offspring vary in traits such as color and size, as shown in a hypothetical fish population in Figure 9.

3	Inherited Variation Recall from Chapter 4 that some of the variation in populations is genetic and such traits can be passed from parents to offspring. Sources of genetic variation include mutations, formation of the gametes, and sexual reproduction. Genetic variation is necessary for evolution by natural selection to occur.

4	Natural Selection Individuals with inherited variations that are better able to survive and reproduce in a particular environment will have more offspring, and thus pass on these favorable traits, than individuals without those features.

Populations will change over time in particular environments.

They will evolve, and come to look different and behave differently from their ancestors as favorable traits spread through a population. If the environment changes again, different heritable traits may be favored and the population can evolve again.

Evolution and Diversity

Charles Darwin spent years developing his ideas on natural selection. Darwin finally published his revolutionary and controversial book called The Origin of Species by Natural Selection. In his book, he built a strong case for evolution by natural selection. He also developed the idea that all organisms have “descended with modification” from common ancestors over a long period of time. Thus, all species have changed through time and are related by descent from a common ancestor.

Considering the geological evidence that Earth is millions of years old, Darwin proposed there had been enough time for organisms to change and for new species to develop from ancestral species.

Adaptation and Extinction

Adaptations are traits that help an organism sur- vive and reproduce in a particular environment, and the inability to adapt can lead to extinction.

Real-World Reading Connection Have you ever thought about why giraffes have such long necks? It makes sense if you consider that giraffes are the only plant eaters in Africa that can reach the top of the tallest trees. Such adaptive traits can be explained by Darwin’s theory of natural selection.

Adaptations

Humans are very skilled at changing their environment. We have buildings, heaters, and air conditioners to make our environment more comfortable. Other organisms are not able to change their environment to meet their needs. Plants cannot walk into a greenhouse when the weather gets cold. Organisms that do have unique characteristics, or adaptations, to live in their specific environments have evolved them over time by the process of natural selection.

Understanding Adaptations

Recall that adaptations are inherited traits that increase an organism’s chance of surviving and reproducing in a particular environment. The night-dwelling tarsier shown in Figure 10 has large eyes that are adapted for seeing in dim light.

Natural Selection in Action

Sometimes things change. A prey species might be devastated by disease. A natural disaster like a hurricane or a tsunami might affect a local area, or a volcano spewing ash into the atmosphere might affect the whole Earth. These changes may kill off some individuals, but the survivors can repopulate the species. But what about longer lasting changes in climate, or physical features? These will require organisms to adapt to match the changes. Can we see natural selection happening? No, but we can see the results. Natural selection leads to adaptations in organisms.

How do organisms adapt? Many people misunderstand Darwin’s theory of natural selection because they misinterpret the word adaptation. Adaptations are the final products of the long process of natural selection. Adaptations occur in groups of organisms when a trait is passed from one generation to another. The organ- isms receiving the trait are better suited to survive and reproduce.

Not all traits are adaptations. Recall from Chapter 4 that a chromosome has many genes that code for traits. In this way, some traits can be passed down because they are linked with other traits, whether they are beneficial to the organism or not. So, an undesirable trait might stay in the population because it is somehow linked to another more beneficial trait. Some traits might help an organ- ism survive, while other traits might cause it to die off. Remember, adaptations are naturally selected, not intentionally chosen.

One Example of Adaptation There is an abundance of examples of adaptation in the natural world. For example, the desert rabbits in Figure 11 have blotchy, brown coats that help them blend in with their environment. Desert rabbits also have long ears that help them stay cool as heat is released through the extensive system of blood vessels in the ears.

How might natural selection have shaped the ears of a rabbit? The population may have initially been made of rabbits with different-sized ears. Those rabbits with long ears would be better able to survive in higher temperatures than rabbits with small ears. So, rabbits with big ears would be more likely to live long enough to have offspring than those with smaller ears. Their offspring would probably have big ears, too. After many generations of rabbits, if it remains hot, it will be hard to find rabbits with small ears. This is because the small-eared rabbits will not have thrived and thus left fewer offspring. When the population shifts to mostly large-eared rabbits, the rabbit population has adapted to the environment, and thus evolved by natural selection.

Types of Adaptations

There are many kinds of adaptations. Scientists divide adaptations into two main categories—structural and behavioral.

Structural Adaptations

Structural Adaptations are inherited physical traits of a species that make its members better suited to their environment. You

have already read about the desert rabbit’s ears. The ears have lots of blood vessels and the blood carries body heat to the ears. The heat is given off to the air and helps the rabbit stay cool. An added advantage of large ears is that they help the rabbit hear any predator that might be sneaking up on it.

Other structural adaptations of the desert rabbit are its long back legs for running and jumping fast, its high-placed eyes that allow it to see almost 360° around, and its mottled appearance. The mottled fur color helps it blend in with its surroundings.

Camouflage Another type of structural adaptation is camouflage, or blending in with the surrounding environment. Some lizards have a coloration that matches their primary surrounding area, such as the lava lizards on the Galápagos Islands shown in Figure 12. Darwin probably observed many lava lizards. Those living on islands with black lava are difficult to see because they are of the same blackish color as the volcanic rocks on which they sunbathe. Other lava lizards match the color of the sandy beaches.

Darwin probably also saw Sally Lightfoot crabs on the Galápagos Islands. The adult crabs are not well camouflaged. They are very bright and colorful. However, their young are a dull-grey color and are well camouflaged among the volcanic rocks. This coloration helps protect vulnerable young crabs from being captured by predators.

Mimicry Another structural adaptation is mimicry, in which one species (the mimic) looks like another species (the model) so that a third species is deceived or fooled. There are several types of mimicry.

Batesian Mimicry In one type of mimicry, Batesian mimicry, the model organism is dangerous or venemous, and the mimic is not dangerous. For example, the harmless snake eel looks like the venomous black and white banded sea snake. Because the snake eel looks like the banded sea snake, predators stay away from both organisms. Notice the similarities between the two in Figure 13.

Mullerian Mimicry In a second type of mimicry, called Mullerian mimicry, two different species that are either venomous or distasteful have evolved to look like one another. Unlike Batesian mimicry, in which only the mimic species benefits from the mimicry, Mullerian mimicry is beneficial to both the mimic species and the model species. An example of a Mullerian mimic and model pair is the mon- arch and viceroy butterflies. Both butterflies have an orange and black color pattern, and both species are distasteful to birds. However, a bird needs to eat only one member of either species to learn to avoid orange and black butterflies. More members of both species will ultimately survive.

Self Mimicry In another type of mimicry, self mimicry, a species fools its prey by looking inviting or familiar. As shown in Figure 13, frogfish display this type of mimicry by using a small body part like a lure. This attracts other smaller fish looking for food. Unfortunately for the deceived animals, when they get close, the frogfish eats them.

Behavioral Adaptations

In addition to natural selection of physical traits, natural selection of inherited behaviors can also occur. Behavioral adaptations are inherited behaviors of a species that make its members better suited to their environment.

Scientists sometimes call them instinctive or inborn behaviors.

These adaptations enhance survivorship and reproduction. For example, as shown in Figure 14, an endangered bird called the clapper rail lives in muddy, brackish, salt marshes. Twice a day, high tides cover this habitat with water and the low tides leave it exposed. As an adaptive trait, the clapper rail builds its nest in tall, grassy marsh plants. Thus, when the water rises, the nests float up, but the plants keep the nest from drifting away. When the tide is low, the nests slide back onto the wetland mud.

Many behavioral adaptations are designed to attract mates. The satin bower bird in Figure 14 uses berries, saliva, and blue scraps to decorate its nest. This technique attracts females for mating, which makes it possible for him to pass along his genes to the next generation.

Herding Instinct You might have gone for a ride in the country and driven by a farm where you saw a herd of cows. If you go to a national park you will likely see a herd of bison or elk. Many grass-eating mammals live in large groups or herds. They instinctively know that a group is less likely to be attacked by a predator than one animal alone. The herding instinct is a behavioral adaptation. Prey animals can band together to frighten or chase away a group of predators.

Extinction

When all the individuals of a particular species die off, that species becomes extinct. Natural selection can lead to extinction if the conditions of the environment change in a way that none of the organisms of a species can survive. Climate change, volcanoes, and earthquakes are some of the environmental factors associated with extinction.

There have been several major extinction events in the history of Earth. For example, some scientists have proposed that a meteorite collided with Earth about 65 million years ago causing the extinction of the dinosaurs. It has been suggested that when this large meteorite hit Earth, it caused so much dust in the atmosphere that the plants did not receive enough sunlight to grow.

Thus, the dinosaurs all starved. You will read more about extinctions in a later chapter.

Causes of Extinction

There are many causes for extinction. They include habitat destruction, loss of genetic diversity, and the introduction of exotic species. Most species are not able to withstand these factors as well as other severe environmental changes.

Loss of Habitat Extinction rates for individual species are on the rise. One important reason is that habitats for plants and animals are shrinking in size as humans develop and occupy more space and use more resources. Figure 15 shows some effects human activities may have on natural habitats. With diminishing natural habitat, fewer organisms can survive.

Loss of Genetic Diversity

The fewer the number of individuals left of a species, the less genetic variability there is in the population. Species need genetic variability to increase the likelihood that some individuals will have the right gene combinations to survive different environmental conditions and for evolution to occur. If there are only a few individuals or if the individuals have limited genetic variability, it is much easier for a change in environmental conditions to lead to extinction.

We designate some species as threatened if they are likely to become endangered in the near future within much of its range. Cheetahs are threatened because they have little genetic diversity. In fact, related individuals share 99 percent of the same genes.

This has led to low survivorship, low fertility, and disease in the remaining cheetah populations.

Competition with Exotic Species Sometimes competition between organisms is caused by the introduction of a new species to the habitat, known as an exotic species. Humans are constantly introducing new species of plants to their environment because of their uniqueness or beauty. If there is no known consumer of a species in the new environment, it may be able to outcompete the native species and push them toward extinction. Introduction of natural consumers or disease-causing agents may reduce the numbers of the invading species.

The kudzu plant was introduced in the southern United States in the 1930s to help prevent erosion. The vine grows so well there that it can grow up to 30 cm per day. However, it shades out forests and kills the trees that are its climbing base. The plant grows naturally in Japan where cool weather, diseases, and consumers help keep it under control. Figure 16 shows some kudzu taking over a tree line.

Inability to Adapt

Another possible reason that species become extinct is their inability to adapt. If a climate changes suddenly, a species might not have any individuals with genetic traits that will allow them to adapt. Over time the population becomes smaller and the individuals become less able to reproduce. For example, there are so few cheetahs on Earth that inbreeding is resulting in the appearance of recessive traits that are harmful to the species.

Inbreeding is mating between closely related individuals and occurs in small populations. Should a rapid environmental change occur, the species could become extinct. The potential causes of slow extinction are summarized in Table 1.

Darwin’s Conclusions Today

Just as extinction does not always happen at the same rate or in the same way, new species formation occurs at different rates, too.

Because the environment is always changing natural selection is always acting on organisms. Most of the environmental changes are small and localized, leading to very slow changes among populations over many generations. Think about a large environmental change that would affect many species at the same time. Whether the species lives or becomes extinct depends on whether it has adapted to the change. In the next chapter you will read about evidence that has helped to reinforce Darwin’s ideas.

Fossils and Evolution

Fossils provide evidence of changes to species and the environment over time.

Real-World Reading Connection Do you watch television shows about forensic science? A show usually includes one or more forensic scientists who search for and analyze evidence to solve a mystery. Studying evolution is similar to solving a mystery. What evidence do scientists look for to determine the types of organisms that lived on Earth in the past?

What are fossils?

Organisms leave evidence of their existence on Earth. Much of this evidence lasts for only a short time, but occasionally it is preserved for a long time. Fossils are the naturally preserved remains, imprints, or traces of organisms that lived long ago.

Fossils, as shown in Figure 1, can include bones, shells, and footprints of ancient life.

A paleontologist is a scientist who studies fossils. Paleontologists study fossils to determine the relationships among organisms, the approximate times when life first appeared, when different organisms first lived on Earth, and when organisms became extinct. Some paleontologists work outside to discover and then carefully uncover fossils. Other paleontologists work in laboratories analyzing and learning about fossils.

When do fossils form?

Have you ever forgotten about a fruit in the refrigerator? When you discover it, the fruit might be soft and have mold growing on it. The fruit is decomposing—breaking down into substances that can be used by other organisms. The process of decomposition, as shown in Figure 2, is part of an organism’s life cycle. In nature, organisms, such as insects, worms, bacteria, fungi, and others, consume and help break down dead organisms. These organisms are decomposers and help the decomposition process. Scavengers might eat part of a dead organism and then scatter the parts they cannot eat. What keeps decomposers and scavengers from destroying the remains of an organism before it is preserved as a fossil?

What conditions provide for fossil formation?

For a dead organism to become a fossil, it must be well protected from decomposers, scavengers, and environmental factors, such as heavy rains or acidic soils. Also, if a dead organism has hard structures such as teeth, bones, or a shell, it has a better chance of becoming a fossil than if it does not have hard structures. Hard structures decay more slowly than softer structures do, also shown in Figure 2, and scavengers are less likely to eat hard structures.

How are fossils formed?

When the word fossil was first used, no one knew that fossils were from ancient organisms. In fact, some scholars argued that fossils could not be from ancient animals. They thought that the only way for fossils to have originated from once-living animals was for the bones and teeth to change magically to stone, which was impossible. It was not until the seventeenth century that scientists began to figure out how fossils form.

Fossils only form under certain conditions. This means that fossils are not commonly found in most rocks or in most locations.

Usually, when we find a fossil, it is only part of a once-living organism. The most frequently found fossils are preserved hard structures, but occasionally soft structures are preserved. In rare instances, entire organisms are preserved. There are several different methods of preservation.

Permineralization

A living organism’s hard structures usually contain tiny spaces filled with air, blood, or other substances. After the organism dies, the substances inside the tiny spaces decompose leaving the spaces empty. If the hard structure is buried, layers of sediment slowly begin to compact and cement to form rock. During this process, permineralization can occur if water in the ground seeps into the tiny empty spaces and deposits minerals. Usually silica, calcite, or a similar mineral is left in the tiny spaces. This forms a strong, rocklike fossil. The details of the organism’s original hard structure are often preserved. Most bones and trees become fossilized through permineralization, as shown in Figure 3. They have hard structures with small spaces where minerals can be deposited. When trees are fossilized through this process, they are often referred to as petrified wood.

Replacement

There are similarities between permineralization and replacement. Minerals such as silica, iron, and pyrite are critical for fossilization in both processes. However, in replacement, the hard structures of the organism dissolve and are replaced with minerals. During replacement, the original microscopic details, such as the inside of a bone, are partially or totally destroyed. Only the shape of the original organism remains. For example, a solution of water and dissolved silica might flow into and through the shell of a dead organism. If the water is acidic, it might dissolve the shell.

Simultaneously, the dissolved silica in the water crystallizes and fills in the places where the shell had been and replaces the original shell. Figure 4 shows an example of replacement.

Carbonization

If a dead organism is quickly buried under conditions without oxygen, elements that are normally found in the living tissue of the organism, such as hydrogen, oxygen, and nitrogen, are removed. A thin film of carbon is all that is left when these elements leave the organism’s remains. As pressure from built-up sediment compresses the buried organism, a carbon film forms that preserves an image or shape of the original organism on a rock. Also, soft materials of animals, such as skin, fur, and feathers, can be preserved as carbon films, as shown in Figure 4.

Countless plant fossils are preserved as carbon films and are the bases of present-day coalfields.

Molds and Casts

Preservation of an impression or indentation of an organism is a mold or cast, as shown in Figure 5. In these fossils, there are no remaining parts of the original organism. For example, molds can be the imprints from a shell or the skin of an animal. There are molds of bite marks, footprints, and eggs in a nest. If the mold is filled in with sediment that hardens into rock, a cast fossil forms.

Original Material

Did you see the movie in which an entire organism was preserved in amber—the fossilized sap of an ancient gymnosperm? A pre- served organism in amber is a type of fossil called original material. Another example of original material is the head from a frozen woolly mammoth shown in Figure 6. An original material fossil is unique because none of the hard and soft structures of the original organ- ism have been replaced or altered. The organism is preserved in its original form. It is rare to find original material, but when it happens it provides much information to paleontologists. Most original-material fossils are more recent than other fossil types.

What do fossils tell us?

Much of the evidence for the pattern of evolution and evolutionary relationships comes from fossils. Scientists also study fossils in order to understand some processes and rates of evolution. Recall from Chapter 5 that natural selection is the survival and reproduction of species with traits that enable them to survive under particular conditions. Over generations, species change due to the loss of organisms that did not have those traits that enabled them to survive. Fossils provide a record of different species that lived in the past.

Relative Fossil Ages

Generally, the older the rock layer, the deeper it is in Earth. If a paleontologist finds fossils in a fairly shallow sedimentary rock layer and then finds others in a deeper sedimentary rock layer, the older fossils are usually those in the deeper sedimentary rock layer, as illustrated in Figure 7. This is true unless there has been an unusual disturbance to the area that has rearranged the rock layers. In this way, ages of the fossils can be compared by the relative ages of the rocks.

Species and Environmental Changes

The fossil record is made of all known fossils and their placements in the formation of rocks and positions in time. It is evidence that supports the evolution of organisms. However, the fossil record has gaps, much like a story in a book that has missing pages. The gaps exist because most organisms decay before fossilization happens, geological processes destroy fossils, or fossils are undiscovered. Even with the gaps, the fossil record is evidence that most of the species that ever lived on Earth are now extinct.

Fossils provide evidence of how life and environmental conditions have changed throughout time. By studying fossils, scientists have determined that during Earth’s early history, life was not as complex as it is now. More complex organisms appeared later in Earth’s history.

Scientists can use fossils to make models that show what an organism might have looked like, as shown in Figure 8. From fossils, scientists can sometimes determine whether an organism lived in family groups or alone, what types of food it ate, what kind of environment it lived in, and many other things about it.

How do fossils provide evidence of the past?

Most organisms decompose and leave no direct evidence of their existence. However, the parts of organisms that are preserved through permineralization, replacement, carbonization, or other methods can tell us much about when organisms lived, how they changed, and when they became extinct. Fossils provide clues that paleontologists can use to reconstruct extinct organisms.

The locations of fossils in sedimentary rock layers can indicate the relative ages of fossils. The pattern of when organisms appear in the fossil record and the structure of the organisms is best explained by the theory of evolution by natural selection.

Biological Evidence

Evidence from comparative anatomy and molecular biology are best explained by the theory of evolution by natural selection.

Real-World Reading Connection At the zoo, there might be animals that appear similar. For example, different bears might have different colors of fur but you can easily recognize that they are bears. Are these similar animals related? Is it coincidence that they are similar? Scientists have wondered about the same things.

Comparative Anatomy

Another form of evidence that supports the theory of evolution by natural selection is comparative anatomy. Comparative anatomy is the study of similarities and differences in the structures of organisms. By using comparative anatomy, scientists have made important scientific discoveries.

Scientists who study insects have known for years that two groups of insects—true flies and scorpionflies—are similar. A true fly, shown in Figure 9, has a pair of large, thin wings and a pair of small, knoblike appendages behind them. A scorpionfly has two pairs of large, thin wings. Scientists predicted that if these groups of insects had a common ancestor, a fossil existed of an ancestor of true flies with two pairs of wings. In 1976, scientists found fossils of four-winged true flies that confirmed their prediction of a common ancestor. The anatomy of the tiny insect fossils provided much evidence to support evolution. The combination of studying and comparing the structures of fossils and living organisms has supported the pattern of evolution.

Structures in Living Organisms

Humans, frogs, bats, birds, and cats all have a common set of three bones in their front or upper appendages, as shown in

Figure 10. The sizes of the bones are different but their forms are similar. At some point in our past, we all shared a common ancestor, but some share more recent ancestors than others.

Homologous Structures In plants, many angiosperms have similar fruits. Recall from Chapter 3 that fruits develop mostly from flower ovaries and they protect the seeds. Angiosperms have a more recent common ancestor than gymnosperms—seed plants without fruits. Parts of organisms that are similar in origin and structure are called homologous structures.

Homologous structures are the result of evolution and can indicate how closely two or more species share common ancestors.

Analogous Structures

The wings of birds and insects are examples of analogous structures. They appear similar but have different ancestral origins. They resulted from similar environmental conditions that produced similar natural selection outcomes over time, but on distantly related organisms.

Scientists do not observe two organisms and decide that because they look alike, they share an ancestor. Instead, scientists consider geography, environmental conditions, fossil records, structures and functions of the body parts, and genetics when possible, in order to understand evolutionary relationships. The accumulation of evidence from these different sources supports evolutionary lines.

Vestigial Structures

Another source of evidence for evolution are vestigial structures. These structures have no function in their present-day form. However, scientists hypothesize that vestigial structures once functioned in an ancestor. For example, most mammals have pelvic bones that support a pair of legs.

Present-day whales have pelvic bones but do not have leg bones like their ancestors, as shown in Figure 11. Some people question why vestigial structures would remain through natural selection if they have no function. It is because vestigial structures are genetically related to an advantageous trait of the species or because their absence would have harmful effects for the survival of the species.

Embryology

The science of the development of embryos from fertilization to birth is embryology. Scientists who study embryos of different animals often compare and contrast their patterns of development. Similar patterns can provide clues to the evolutionary relationships among organisms.

Embryos of different vertebrate animal species have similar early developmental stages during which they show common features. As the embryos grow and develop, those features become unique structures in different species. For example, embryos of vertebrate animals have bulges in the region of the neck called pharyngeal pouches, as shown in Figure 12. They become facial and neck structures. In fish, they develop into structures called gill arches; in humans, one of the pharyngeal pouches develops into part of the ear.

It is important to remember that the more closely related species are, the more features they share during development. These shared similarities are best explained by the theory of common ancestors and evolution through natural selection. However, while developmental patterns indicate evolutionary history, they are not an historical journey through evolution.

Molecular Biology

What is known today about genetics, biochemistry, and molecular biology probably would have amazed Darwin. New technology provides the tools to continue making rapid discoveries in these areas. These molecular data also support the theory of evolution through natural selection.

The proteins in all organisms consist of countless arrangements of just 20 different molecules called amino acids. Recall from

Chapter 1 that cellular respiration requires oxygen and releases energy. Organisms that use cellular respiration must have a protein called cytochrome c for this process to occur. Scientists have dis- covered slight differences in the cytochrome c molecules found in different organisms that use cellular respiration. It is unlikely that these differences developed independently from different ancestral lines. For example, this protein is more similar among mammals, such as a pig, a monkey, and a human, as shown in Figure 13, than are the forms of the protein found in other organisms. This supports the theory that different mammals have a more recent ancestor in common than do mammals and yeast.

Scientists also look at other proteins and at the sequences of DNA. Recent molecular data suggests that closest living relative of whales are hippopotamuses. This molecular evidence supports prior evidence from comparative anatomy about whales’ relation- ships to even-toed hoofed animals, but offers specific information about the relationship than previously known.

How does biological evidence support the theory of evolution?

Evolution is also supported through different biological evidence. Scientists use homologous and analogous structures to determine possible relationships among modern organisms and their ancestors. Examining vestigial structures provides further understanding of the evolution of present-day organisms from ancient ancestors. Studying the embryology of different organisms provides supporting evidence for evolution from a common ancestor, but does not provide evidence of how evolution occurred. Recent technologies have enabled scientists to use molecular biology to search for common ancestors. The data from different branches of science are compared and used to build a better understanding of evolution.

Evolution and Plate Tectonics

Over time, the movement of lithospheric plates has changed environments that led to changes in species.

Real-World Reading Connection If you quickly glance at a clock, you probably notice the movement of the second hand but not the movement of the hour hand. Like the hour hand, the ground below you is slowly moving. Over time, the slow movement of Earth’s plates has led to changes in species.

Continental Drift

Earth’s surface slowly changed over time, and it is hard to recognize the changes. However, earthquakes are evidence that changes to Earth’s lithospheric plates are still occurring.

Recall that Alfred Wegener proposed the continental drift hypothesis in 1912. The most obvious evidence for his hypothesis included the fit of shorelines of Africa and South America, as shown in Figure 14. As lithospheric plates move, environmental changes result for the species that live on and near them. Natural selection acts upon species when there are environmental changes. Only individuals that are well-suited to the new conditions will be able to survive and pass their genes to offspring.

Environmental changes can also lead to species extinctions.

Geographic Isolation

Landforms are created when lithospheric plates move. Mountains and volcanoes form when lithospheric plates move together. Valleys form where lithospheric plates move apart. Sometimes, landforms become barriers between populations of species, and breeding between these populations is prohibited. When this hap- pens, geographic isolation can occur. Once separated, the populations in different environments might follow different evolutionary paths over time. Other geographic features, such as rivers and large bodies of water, can lead to geographic isolation of species, also.

Figure 15 illustrates geographic isolation in California. The geography of a location plays an important role in how organisms evolve.

Darwin’s Observations of Geographic Isolation

Geographic isolation was responsible for many of the organisms described in Darwin’s journals. The results of geographic isolation puzzled Darwin and prompted much of his research. Imagine if you have two islands with very similar environmental conditions— island A and island B—but on different sides of Earth. Imagine that island A is near the mainland, but the environmental conditions on island A and those on the mainland are quite different. Obviously, you would expect the organisms on island A and island B to be more similar than those on island A and the mainland. In the Galápagos Islands and Ecuador, Darwin found just the opposite. Species on the Galápagos Islands were more similar to those on the Ecuador mainland even though the environments of the islands and the mainland were different. Yet, the similar organ- isms were clearly unique species. These observations led to the idea of evolution by natural selection.

Geographic Isolation v. Convergent Evolution

Sometimes distant locations with similar environmental conditions have species with similar traits. These species have evolved independently but under similar conditions. This type of evolution is known as convergent evolution and results in structural and functional similarities. New research in genetics has supported the finding that although two species in similar environments can appear similar, they did not evolve from common ancestors.

Figure 16 shows two plant species that have undergone convergent evolution. They appear similar, but they are from different branches of the evolutionary tree. Geographic isolation leads to closely related species that appear different while convergent evolution results in very distantly related species that appear similar.

How are plate tectonics and evolution related?

The moving lithospheric plates result in changes to Earth’s surface, such as the formations of mountains, valleys, and bodies of water. These geographic barriers can result in the evolution of new species when populations become separated. Some related species are now separated because of plate tectonics. Widely separated species can evolve similar traits that ensure survival in similar environments.

Changes in climate, whether from plate tectonics or for other reasons, resulted in the evolution of new species by natural selection.

Recall from Chapter 5 that environmental changes can cause species adaptations or extinctions. Only members of the species with traits that enable them to survive the new environmental conditions will survive, reproduce, and pass their genetic material to offspring.

Classifying Organisms

Scientists use traits and evolutionary history to classify species.

Real-World Reading Connection How do you organize your clothes? Do you hang your T-shirts with your other shirts in your closet or put them in a drawer with other folded clothes?

Do your friends organize their clothes in the same way? Scientists organize Earth’s organisms into groups, but they do not always agree on the best system.

Historic Classification Systems

The Greek philosopher Aristotle was one of the first people to classify organisms. He categorized things as animals, plants, or minerals. Aristotle also grouped living things by where they lived—in the air, on the land, or in the sea. Scholars used this system for hundreds of years. When Europeans began exploring new lands, they discovered many new plants and animals that could not be classified using Aristotle’s system. A new classification system was needed.

In the mid-eighteenth century, Swedish botanist and explorer Carolus Linnaeus developed a classification system that grouped organisms based on similar physical structures. The Linnaeus classification system has many related levels. The largest group of organisms is a kingdom and the smallest group is a species. A species includes organisms that have the greatest number of traits in common and can breed and produce fertile offspring. Only species are subject to natural selection and evolution.

Naming and Grouping Species

Linnaeus also developed a system for naming species that is still used. The two-word scientific name of an organism is its species name. For example, the species name for the California black oak is Quercus kelloggii.

Groups of similar living species belong to a genus (plural, genera). The first word of a species name identifies the genus to which the species belongs. All oaks have Quercus as the first word of their species name. Similar genera belong to a family, similar families belong to an order, similar orders belong to a class, similar classes belong to a phylum (plural, phyla), and similar phyla belong to a kingdom. Table 1 shows these classifications levels for the western spotted skunk.

Determining Kingdom

Cell type, the presence of a cell wall, or whether organisms are single-celled or multicellular, are used to define a kingdom. Scientists classify living organisms into one of six kingdoms—Kingdom Eubacteria, Kingdom Archeabacteria, Kingdom Protists, Kingdom Fungi, Kingdom Plantae, and Kingdom Animalia.

Modern Methods of Classification

The modern study of classification is called systematics. In systematics, mostly DNA and molecular biology are used to identify relationships between organisms.

Scientists can now determine the order or sequence of the molecules in an organism’s DNA. The more DNA sequences two species have in common, the more likely it is that they share a recent ancestor. Such DNA evidence has confirmed many existing classifications.

However, scientists have found that species established as close relatives, do not share as many DNA sequences as expected. When this occurs, scientists review existing evidence of evolution such as fossils and comparative anatomy data, and often reclassify organisms.

It is too expensive to find the complete DNA sequence for all organisms. However, scientists can determine the sequence of a sample of 1,000 molecule pairs in DNA called haplotype. Scientists compare these small sequences of DNA between organisms to search for similarities. The more similar two haplotypes are, the more closely the organisms are related.

DNA hybridization is another tool used in systematics. Scientists do not learn the actual sequences of DNA that two organisms share using DNA hybridization, but they can determine the percentage of DNA that is the same.

A new level of classification that developed because of molecular biology is domain.

Domain is now the highest level instead of kingdom. Based on differences in a particular DNA sequence, organisms are separated into three domains—Bacteria, Archaea, or Eukarya. The Eukarya includes organisms that have cells with a nucleus. As other molecular biology techniques are developed, classification systems might change.

How are classification of organisms and evolution related?

Classification in based on the idea of common ancestors, from the theory of natural selection. Aristotle developed the first classification system. Linnaeus used similar physical structures to place organisms into groups and developed the species naming system. The first word of a species name identifies the genus of the species. Molecular systematics is part of the classification process now. The new hierarchy of classification is domain, kingdom, phylum, class, order, family, genus, and species. Classification is very useful for understanding and communicating evolutionary relationships.

Relative Ages of Rocks

Geologists can determine the relative ages of rocks by studying the order of rock layers, fossils, and geologic processes that are occurring today.

Real-World Reading Connection When detectives go to the scene of a crime, they look for clues. After recording their observations, they try to determine what happened. Geologists search for clues in the rocks that can provide information about events that occurred millions of years ago.

The Beginning of Modern Geology

Before the late 1700’s most scientists believed that rocks formed by evaporation of minerals dissolved in sea water. By observing rock features and the movement of sediments in streams, a Scotsman named James Hutton first realized there were two processes at work on Earth. One process formed rock and the other tore it down again. Figure 1 shows one of these destructive processes.

The Principle of Uniformitarianism

Geologists, like all scientists, search for ideas to explain how Earth’s processes work. Hutton had an idea that “the present is the key to the past.” His **principle of uniformitarianism** states that the processes that are at work today are the same processes that have been at work in Earth’s past. Another way to say this is that the same Earth processes have been at work for a very long time.

Therefore, scientists can observe the processes that are active on Earth today, and use them to interpret what happened in the past.

So when scientists observe soil layers being put down during annual flooding, they can compare that to how layering in rocks might have occurred. Figure 2 shows what might have happened to a sand beach.

Change is Slow

No one has ever directly observed a mountain forming or a steep river valley becoming wider and less steep. Yet we know these things happen. The process is so slow that direct observation is not possible. James Hutton realized this. He reasoned that if erosion occurred for a long time, it could greatly change Earth’s surface. In fact, with enough time, erosion could wear down mountain ranges. Figure 3 shows a young, rugged mountain range and an older, smoother range. How might time and erosion change the rugged mountains?

The Rock Cycle

James Hutton had begun to recognize what geologists now call the rock cycle, shown in Figure 4. The rock cycle is the series of processes that make and change rocks through heating, melting, cooling, uplift, weathering, burial, and increasing pressure. You can trace the formation of the three major types of rocks—igneous, sedimentary, and metamorphic—on the figure.

Igneous, Metamorphic, and Sedimentary

Geologists divide rocks into three groups determined by how a rock is formed. Igneous rocks are produced when magma solidifies. Different kinds of igneous rock are identified by the size of the crystals that they contain. Large crystals form when magma cools very slowly underground. Smaller crystals form in igneous rock that cools rapidly at the surface. Some igneous rocks have no crystals because they cool very rapidly, perhaps in the ocean water.

Metamorphic rock is any rock that is put under extreme pressure or heat short of melting. The rocks that are most affected are those that come in contact with hot fluids and magma moving through fractures toward the surface. Pressure may cause the mineral spaces to collapse and become more closely packed. Temperature may cause the minerals to recrystallize and form a new rock mineral.

Sedimentary rocks are those that form from sediments. Sediments are the tiny particles that come from the erosion of various types of rocks. The particles eventually become compacted and cemented into rocks.

Estimating the age of each type of rock is a challenge geologists encounter as they observe these rock formation processes. The most important ideas in determining the ages of rocks are related to sedimentary rock formation.

Sediment Formation and Layering

Sedimentary rocks form from pieces of preexisting rocks. There are four steps in the process that forms sedimentary rock: weathering, transportation, deposition, and lithification. These four steps occur simultaneously everywhere on Earth’s surface.

Weathering

The first step in the formation of sedimentary rocks is weathering. Weathering is the physical or chemical breakdown of rocks into smaller pieces. Physical weathering breaks down rocks without changing the mineral composition. Frost wedging, the weathering process that occurs when water freezes and expands in cracks, can force rocks to break apart. This process is illustrated in the top photo of Figure 5.

Chemical weathering changes the mineral composition of rocks.

When minerals that are holding a rock together change to new minerals, the rock often falls apart. This crumbling increases the surface area of the rock. As surface area increases, the rate of chemical weathering increases. Chemical weathering usually involves water. Rainwater can combine with carbon dioxide in the atmosphere to form carbonic acid. Acidic rainwater can dissolve limestone. The cave shown in Figure 5 was formed when acidic rainwater dissolved limestone under Earth’s surface.

Transportation The next step in sedimentary rock formation is transportation. It occurs when sediments move downhill to lower areas where they come to rest. Sediments are moved by running water, wind, moving ice, and gravity.

Sediments vary in size from large boulders to microscopic bits of rock. These different-sized pieces of rock are called clasts. Different amounts of force are needed to move them. The force is applied by gravity as the slope of the landscape changes. When the slope of the landscape is steep, more force can be applied to the clast. The more force that is applied to the clast, the larger the clast that can be moved.

Gravity pulling on water, air, and ice can also move the clasts.

Fast-moving water can move larger clasts than slowly moving water. You can understand how wind might move large clasts during large storms while sand and smaller clasts are commonly blown across the landscape. Clasts of all sizes can be moved by glaciers because of the glacier’s mass and size, but they move very slowly. During the process of being transported, clasts may change size and shape. They become rounded as they knock into each other and chips are broken off. This reduces their size at the same time.

Deposition The third step in the formation of sedimentary rocks is deposition. It occurs when sediment being trans- ported by water, wind, or a glacier slows down or stops. This usually happens in low areas on the landscape called depositional environments. As the sediments are dropped, gravity causes them to form parallel, horizontal layers. Distinct layering is a common feature in sedimentary rocks, as shown in Figure 6. Another characteristic of deposition is sorting. As the carrier of the sediment slows down, heavier objects are dropped first. Lighter and lighter objects are carried farther and deposited later. Figure 7 shows how different materials may be sorted.

Lithification The final step in the formation of sedimentary rock is lithification. As older sediment layers become buried beneath layers of younger sediments, the weight of the younger sediments squeezes the older sediments together. This is called compaction. Mineral-rich liquids are still able to seep into the pore spaces between the sediment grains. Evaporation of the water and the weight of the layers cause the minerals in the pore spaces to turn to cement. This compaction and cementation changes the sediments into rock. The sediments have become lithified.

Superposition and the Fossil Record

Layers of rocks are called strata (singular, stratum). In 1669, Nicolas Steno, a Danish physician, presented four principles that helped geologists study strata and interpret the rocks’ history. They are the principles of superposition, original horizontality, original lateral continuity, and cross-cutting relationships.

Principle of Superposition

The **principle of superposition** states that in a stack of undisturbed sedimentary rock layers, the layers on the bottom were deposited before the layers on the top. This means that the rocks on the bottom are older than the rocks on the top. Figure 8 shows this relationship. The exact ages of the rocks are not known, but their relative age is known. Relative age tells you how old something is when compared to something else. For example you know that the middle strata are younger than the bottom stratum, but are older than the top stratum.

The principle of superposition is perhaps the most important of Steno’s four principles. Once the relationship between strata and age was understood, geologists could study the rock layers and determine the geologic events that resulted in their deposition.

Fossils and Relative Age

You have read that the fossil record provides information on changes in life throughout Earth’s history.

By keeping track of which fossils came from which

strata, and by applying the principle of superposition, geologists use the fossil occurrences in layers to con- firm or assign relative ages to rock strata. Figure 9 show how the superposition has helped date the layers.

Using Rocks to Determine Relative Age

Estimating when a rock layer formed or the age of certain fossils in those layers is one of the tasks of a geologist. James Hutton explained the process of uniformitarianism. Scientists can use geologic processes observed today to interpret geologic events of the past. Nicolas Steno stated four principles that help scientists deter- mine the order, or the relative ages, of these geologic events. The rock cycle outlines the formation processes of the three main types of rock. Following the four steps in the formation of sedimentary rocks helps scientists understand how Earth formed. Layering and fossils are common in sedimentary rocks. Fossils can also be used to determine or confirm the relative ages of rock strata.

Absolute Ages of Rocks

Unstable parent isotopes change to stable daughter isotopes at a constant rate. Geologists measure the amounts of these isotopes in minerals to determine how long ago the minerals formed.

Real-World Reading Connection Bog bodies are well-pre- served human remains found in peat bogs. They range in age from hundreds to thousands of years old. Their clothes and hair- styles have allowed researchers to infer about when these people lived. What dating methods allow researchers to confirm their inferences and more closely pinpoint when these people lived?

How would you go about finding out how old Earth is? In the past, scientists have tried measuring rates of erosion to see how long it would take mountains to erode. They tried calculating the time it would take Earth to cool from a molten mass to its present temperature. Each attempt was unsuccessful at predicting Earth’s age. Finally, scientists discovered a natural “clock” that ticked away with great accuracy. This new clock allowed geologists to date the age of Earth, meteorites, and the Moon. In this lesson, you will read about natural clocks and how they work. Figure 10 shows a bog body. Since bog bodies are well preserved by the acid conditions of the bog, scientists used the natural clock to determine their age.

Atoms and Isotopes

Atoms are the building blocks of all matter. Atoms are made of protons, neutrons, and electrons. Protons and neutrons are located in the center of an atom called the nucleus. Electrons are located outside the nucleus. Figure 11 shows a representation of a carbon atom.

Carbon is one of more than 100 known chemical elements that exist on Earth. An element is defined by the number of protons in its atoms. For example, a carbon atom contains six protons while an oxygen atom contains eight protons. Although the number of protons in an atom of any given element is always the same, the number of neutrons in that element can vary. Isotope is the term for atoms of a given element that have the same number of protons, but a differing number of neutrons. Isotopes of carbon atoms can have six, seven, or eight neutrons. The isotopes are written as carbon-12, carbon-13 and carbon-14. The carbon isotope numbers are found by adding the number of protons and neutrons. Carbon- 12 has six protons and six neutrons. Carbon-13 has six protons and seven neutrons. Carbon-14 has six protons and eight neutrons.

Radioactive Decay

Isotopes of an element may be stable or unstable. Carbon has two stable isotopes and one unstable, or radioactive, isotope. An isotope that has an unstable nucleus emits particles and energy. A more stable nucleus is formed through a process called radioactive decay. Radioactive decay occurs when an unstable atomic nucleus changes into another nucleus by emitting particles and energy. A nucleus that is unstable and undergoes radioactive decay is called radioactive. This decay is the natural clock that scientists use to find the ages of Earth’s rocks. Figure 12 illustrates the radioactive decay process for the element polonium. Radioactive decay is useful for finding the age of objects that are very old. Figure 13 shows a method for dating things only a few thousand years old.

Parent and Daughter Isotopes Scientists call the isotope that undergoes radioactive decay the parent isotope. The stable form of the element that forms is called the daughter isotope. Recall that carbon has two stable isotopes and one radioactive isotope. Uranium-238 goes through fourteen steps as it decays to a stable isotope of lead. Uranium decay is the process behind nuclear fission and nuclear power.

Half-Life

In 1902, Ernest Rutherford and Frederick Soddy discovered that parent isotopes decay into daughter isotopes at a constant rate. This rate is the decay rate. Table 2 lists the decay rates of some common radioactive isotopes. To measure decay rates, scientists must compare the amount of parent and daughter isotopes in a material.

They then measure the rate at which the radioactive element gives off energy and particles. The time it takes for a sample of a radioactive isotope to decay to half its original mass is called half- life. Geologists can determine the absolute age of a piece of the Earth’s crust by calculating the absolute age of the minerals that compose it.

They do this by measuring the parent-to-daughter ratio of an isotope in the minerals. The method for calculating half-lives and age is illustrated in Figure 14.

Determining Earth’s Age

When scientists discovered that radioactive isotopes decay at a constant rate, they knew they had found a “natural clock.” Then they turned their attention to the age of Earth. If they could find a way to use this “clock,” would they be able determine the age of Earth’s oldest rocks?

Radiometric Dating

The procedure that scientists use to calculate absolute ages of rocks and minerals is called radiometric dating. By measuring the amount of parent material and comparing it to the daughter material in a rock, the number of half-lives the material has been through can be counted. Igneous rocks are the most common rocks used for radiometric dating. They usually contain only parent isotopes when they formed. Dates calculated from minerals in igneous rocks indicate when the mineral crystallized from magma.

Determining the age of metamorphic rocks can be difficult. The increase in temperature and pressure during metamorphism can cause a rock to partially melt. When rocks melt, they become igneous and their radiometric clock gets reset. Decay begins again.

Geologists rarely use radiometric dating for sedimentary rocks. This is because the dates from sedimentary rocks indicate when the minerals in the rock formed, not when the sedimentary rock itself formed. The lab shown in Figure 15 is one that does radio- metric dating of rocks.

The Absolute Age of Earth

As you have read, Earth’s material constantly moves through the rock cycle. Geologists feared this recycling would have destroyed Earth’s oldest rocks.

In 1989, scientists made an important discovery. Figure 16 shows that the oldest rocks on Earth are part of areas called continental shields. Continental shields are areas where exposed rock dates back to **Precambrian** time—billions of years ago. Grains from sedimentary rock can be dated as if they are an igneous rock sample. The ages reported by different scientists using different isotope pairs ranged between 3.62 and 3.65 billion years. Until 1989, geologists doubted they would find any older rocks from Earth. Then researchers reported a 3.96-billion-year-old age for a zircon grain from a metamorphic rock in Canada.

New technology allowed scientists to date other zircon crystals from rocks in Canada in 1998 and Australia in 2001. The ages they reported were 4.0 and 4.4 billion years.

Meteorites and the Moon Scientists use radiometric dating to determine the age of meteorites and the Moon. The ages of stony meteorites collected in Antarctica have been calculated to be between 4.48 and 4.56 billion years old. As shown in Figure 17, the Moon’s surface is covered with loose rock material. Rocks brought back from the Moon have been radiometrically dated to be approximately 4.6 billion years old. This closeness of the calculated ages of Earth, the Moon, and meteorites helps to confirm the idea that the entire solar system formed at the same time.

Isotopes and Earth’s Age

Scientists use radioactive isotopes to measure the percentage of parent to daughter material in a substance. This allows scientists to provide absolute, or numerical ages to the geologic layers and events. Unstable parent isotopes decay into stable daughter isotopes. Measuring the rate at which this occurs provides useful data that can be transformed into a unit called a half-life. Using half- life values of certain isotopes allows scientists to calculate the absolute ages of igneous rocks and some metamorphosed rocks.

Earth’s age has been calculated to be about 4.55 billion years, the age of its oldest rocks.

In the next chapter you will read how relative and absolute age dating have been used to help explore the history of Earth’s life.

Geologic Time and Mass Extinctions

Fossils provide evidence of how life and environ- mental conditions have changed over time. Geologists used fossils to create the geologic time scale.

Real-World Reading Connection The storage media for home computers has changed over the last 25 years. During that time people have used 5.25-inch floppy disks, 3.5-inch disks, and now read-writable CDs and portable flash drives. In a similar way, life on Earth has changed throughout Earth’s history.

Development of the Geologic Time Scale

The changes to life and Earth that have occurred throughout Earth’s history are recorded on a time line called the geologic time scale. Because extinctions, growth rates, and environmental changes happen at different rates, the time scale’s units are uneven. To organize geologic time into units, paleontologists study the types and ages of fossils in rock layers and decide where to mark the units.

Index Fossils

Units of time on the geologic time scale are described by the presence and the absence of certain types of fossils called index fossils. An index fossil is the remains of a species that existed over vast regions of Earth for a short period of time. Table 1 explains how a species is selected to be an index fossil.

Divisions of the Geologic Time Scale

Figure 1 shows the geologic time scale used today. Notice that no two divisions of time have the same number of years. There are several major divisions of the geologic time scale.

The largest divisions are called eons and are the longest units of time. Eons have been bro- ken into smaller units called eras. Eras in turn are divided into periods and epochs.

Eras Paleontologists have defined three eras within the Phanerozoic eon. The **Paleozoic era**, which means “ancient life,” was dominated by invertebrate marine organisms. Although they might have a shell, invertebrate marine animals are those without a backbone. During the **Mesozoic era**, which means “middle life,” dinosaurs and mammals lived on land. The **Cenozoic era**, which means “recent life,” continues today.

Modern mammals and humans evolved during this recent time.

Periods and Epochs Divisions of eras are called periods. The **Cenozoic era** was once divided into two periods, the Tertiary period and the Quaternary period. As scientists learn more and do more research, they sometimes change the way they describe and name things. Today we no longer use the term Tertiary to describe the first division of the Cenozoic.

Instead paleontologists divide the Tertiary into two parts called the Paleogene period and

Neogene period. You can find these listed in the chart in Figure 1.

The Quaternary period began about 1.8 mil- lion years ago. The periods have been divided into epochs. The **Pleistocene epoch** ended about 8,000 years ago.

The **Holocene** is the epoch we live in.

What are mass extinctions?

How do paleontologists determine when one unit on the geologic time scale ends and another begins? Scientists look for layers of rock that include more diversity and greater numbers of fossils than rock layers above them. This indicates that many organisms might have died over a short period of time. Several units on the geo- logic time scale begin and end with mass extinction, as shown in Figure 2. Mass extinction is the dying off of many different species of organ- isms over a short period in geologic time.

The extinction of a single species of organisms is common. In fact, 99 percent of all species that have lived on Earth are now extinct. However, the extinction of several species over a short period of time is rare. It is important to note that mass extinctions do not occur instantaneously.

They can happen over as great a span of time as a few million years.

Possible Causes of Mass Extinction

Once paleontologists recognized that mass extinctions occurred throughout geologic time, they began to search for catastrophic events that might cause them. A catastrophic event is an event that causes a drastic change in the numbers of organisms of one or more species over a short period in geologic time. It may seem that all catastrophic events cause mass extinction, but that is not always true. Scientists still do not fully understand the specific catastrophic events that have caused each of the mass extinctions.

They have identified several types of events, however, that have the ability to cause mass extinction. These events include changes in cli- mate, volcanic eruptions, and asteroid impacts.

Climate Change

There is evidence that some mass extinctions were caused by relatively sudden changes in climate. Figure 3 shows a diver collecting climatic evidence. Recall that climate is an average weather pattern over a long period of time. Species that are unable to sur- vive a change in climate become extinct. Climate change can be caused by several events, including some volcanic eruptions and asteroid impacts. Global warming and global cooling are two types of climate change that might have caused some mass extinctions.

Global Warming Burning of fossil fuels, such as coal and oil, adds greenhouse gases, like carbon dioxide, to the atmosphere. Many scientists think greenhouse gases in the atmosphere contribute to global warming, a global increase in atmospheric temperature. If the overall temperature of the atmosphere increases, the temperature of the oceans also increases. Because warm water holds less oxygen than cold water, global warming could cause a decrease in oxygen levels in the water. If the oxygen levels drop in shallow waters, where most organisms live, mass extinctions could occur.

Global warming could also raise sea levels. Water from melting glaciers would flow into the oceans, causing sea levels to rise.

Geologists have found evidence of warm temperatures, a rising sea level, and oxygen-poor waters flooding shallow-water areas during the Devonian mass extinction. Some scientists think global warming might have been the cause.

Global Cooling Mass extinctions could also be caused by global cooling, an overall decrease in atmospheric temperatures. Global cooling could lower sea levels as large amounts of water are frozen in glacial ice. Less water in the oceans would mean fewer warm, shallow-water environments. With fewer warm-water environments, there would be less space to support marine ecosystems.

Global cooling appears to have initiated the Ordovician mass extinction event. Figure 4 shows how at that time, large glaciers were active near the south pole.

Volcanoes

When you think of a volcanic eruption, you might think of a large explosion. However, volcanoes also produce nonexplosive eruptions. The dust, ash, lava, and gas emitted from volcanic eruptions can affect climate and organisms.

Explosive Eruptions Mt. Vesuvius, in Italy, erupted in A.D. 79. The ash and lava buried the cities of Pompeii and Herculaneum, killing thousands of people. The 1815 eruption of Tambora produced a flaming cloud of dust and gas that killed more than 30,000 people. The year after this eruption is referred to as the year without summer as the air was blanketed with ash, reducing the amount of sunlight that reached Earth. Figure 5 shows part of the results of the violent, but relatively small, explosion of Mount St. Helens in Washington State. These eruptions are recent events in geologic time. Although catastrophic, these eruptions have not led to mass extinction. However, a volcanic eruption is one of several hypotheses proposed to explain the mass extinction event at the end of the **Cretaceous period**.

Basalt Flows Along divergent plate boundaries and in isolated hot spots, such as under the Hawaiian Islands, lava flows smoothly onto Earth’s surface. These nonexplosive floods of molten basalt emit the largest volume of matter of any eruptions. This matter comes from Earth’s mantle. Geologic evidence shows that large basalt flows occurred in Siberia during the Permian mass extinction. The flows are called the Siberian Traps. During the Cretaceous mass extinction, another large basalt flow, called the Deccan Traps, was released in India. Although small by comparison, Figure 6 shows a basalt flow around a volcano in Hawaii.

Volcanic Haze

The gases produced by basalt flows cause a complex sequence of effects. First, sulfur dioxide gas is released into the atmosphere, resulting in the formation of acid clouds. These clouds block some of the sunlight from reaching Earth’s surface.

Over the course of months to tens of years, global cooling occurs. This is called the volcanic haze effect. Over periods of tens to hundreds of thousands of years, global temperatures may increase because heat becomes trapped in Earth’s atmosphere by the sulfuric acid clouds. It is uncertain whether basaltic flows and the gases they emit were enough to cause the Permian and Cretaceous mass extinctions.

Asteroid Impacts

Figure 7 shows the location of a possible asteroid impact site on the Yucatan Peninsula in Mexico.

Scientists believe this impact may have contributed to the Cretaceous mass extinction.

Effects of the Impact

Some geologists propose that this impact sent enough dust and other material into the atmosphere to block sunlight. Figure 8 shows how a cold and dark Earth killed plants and other primary producers. Without primary producers, there was no source of food. Mass extinctions followed. Global cooling, created by the initial impact, turned into global warming as carbon dioxide was released from burning plants ignited by the impact. These greenhouse conditions lasted thousands of years.

The Debate Most scientists accept the hypothesis that a large asteroid, or possibly several asteroids, hit Earth at the end of the **Cretaceous period**.

Evidence supports the hypothesis that many species became extinct around the time of the impact.

However, paleontologists propose that the impact did not cause all the extinctions. The fossil record shows that many species were decreasing in number and diversity long before the impact. In fact, many became extinct at least one million years before the impact. The fossil record also shows that the extinction rate increased tens of thousands of years before the impact. It appears that while the impact did cause some extinctions, it clearly did not cause all of them.

Is there an extinction pattern?

Although mass extinctions occur over a short geologic time span, they are not instantaneous. Fossil evidence indicates that mass extinctions can occur over a few million years or more. What caused the mass extinctions in geologic time? Investigations have revealed no clear patterns, although there are several types of catastrophic events that can cause mass extinction. It appears that each extinction event might have been caused by a unique series of events. Species that survive mass extinction inherit an environment with few competitors. In time, their descendants might repopulate Earth. In the next lesson, you will read about the organ- isms that populated Earth during major segments of geologic time.

Early Earth History

Bacteria, the simplest organisms, were the first organisms to evolve on Earth. Increasingly complex organisms followed them.

Real-World Reading Connection Imagine that you go into the kitchen and there on the table you find a paper plate with icing and candle wax on it. You might ask yourself what happened. Your hypothesis might be that in the recent past there was a birthday party in the room. Scientists have a similar task when they find the fossilized remains of ancient organisms.

They ask themselves this question: What was life on Earth like in the past?

Life on Earth Changes

Fossils record the history of life on Earth. Paleontologists, who study fossils, discovered that the system used to classify modern organisms could also be used to classify fossils. They observed that fossils from rock layers that are touching are more similar to each other than fossils from widely separated layers. Geologists also recognized that the more recently a fossil was formed, the more it resembles a living organism. Perhaps you have been to the ocean and collected a sand dollar, or have seen a sand dollar skeleton. It likely looked most like the sand dollar in the first row of Table 2. In this lesson, you will read about these changes in life during the **Precambrian time**.

Precambrian Time

Figure 9 shows the portion of the geologic time scale that represents **Precambrian** time. **Precambrian** time is divided into three eons—Hadean, Archean, and Proterozoic. The **Precambrian** age represents 88 percent of Earth’s history. Precambrian rocks are difficult for scientists to study because most of the rock has been changed through metamorphism. Recall that it is difficult for scientists to determine the absolute age of metamorphosed rock using radiometric dating. Also, much of the Precambrian rock has been destroyed at subduction zones. As the plates move together, the more dense plate sinks into the mantle. There the rock in the plate may melt or become metamorphic. Any fossil remains will be destroyed.

Fossils are not as abundant in Precambrian-aged rocks as they are in younger rocks. But, the fossils that have been dis- covered provide a great deal of information about Earth’s early atmosphere and environment during this time.

The Precambrian Atmosphere

Rocks of Archean age reveal that Earth’s early atmosphere was very different from today’s atmosphere. For example,

Archean sediments contain large amounts of the minerals pyrite and uraninite. Today’s atmosphere contains oxygen that quickly destroys these minerals through the chemical process of oxidation. We see oxidation today when water and oxygen react with iron to make rust. Pyrite contains lots of iron. It makes sense that the presence of pyrite and uranite in very old rocks tells us that Earth’s early atmosphere had very little oxygen.

Ozone

Because we know oxygen is required to make ozone, the absence of oxygen also suggests that there was no ozone layer during **Precambrian** time. Scientists are concerned that the ozone layer today has been partially destroyed by chemicals made by humans. Without atmospheric ozone to protect life on Earth from harmful ultraviolet rays, exposure of life- forms to these rays could result in death or mutation. Recall how mutations occur in cells. Changes in one gene in an organism could result in new life-forms many, many generations later.

The First Organisms

Stromatolites are mounds of alternating thin-layered sediments and photosynthetic cyanobacteria. They contain the fossils of one of the earliest known, ancient organisms.

Figure 10 shows how stromatolites form. Cyanobacteria are single-celled, blue-green algae thought to be one of the earliest organisms. Like other photosynthetic organisms, cyanobacteria take in carbon dioxide and release oxygen. Stromatolites were common during the **Archaen eon** until the late **Proterozoic eon**, when bacteria-eating animals evolved. Today, stromatolites exist only in water that is too salty and warm for other organisms to survive.

A Changing Environment

Recall that Earth’s early atmosphere had no oxygen. Because cyanobacteria are photosynthetic, they released oxygen into the atmosphere. Over the next hundreds of millions of years, oxygen levels rose slowly as cyanobacteria and other early life-forms released oxygen. The earliest organisms did not consume oxygen. In fact, oxygen could kill them. During this period, natural selection favored organisms that could tolerate or even use oxygen.

The amount of ozone in the atmosphere also increased during this time, shielding life on Earth from ultraviolet rays. These gradual changes to the atmosphere resulted in major changes in life on Earth.

Soft-Bodied Organisms

Organisms increased in complexity in the Proterozoic eon.

During this eon, the first invertebrate organisms appeared.

Invertebrates are animals without backbones. Unusual fossils

f soft-bodied organisms were discovered in the Ediacara Hills of Australia. These organisms, called the Ediacaran fauna, had shapes similar to present-day jellies, worms, and corals. Some paleontologists believe that some present-day organisms are descended from the Ediacaran fauna. Others think these organisms became extinct at the end of the **Proterozoic eon**.

The Paleozoic Era

The first appearance of fossils of organisms made from hard parts marks the end of the **Proterozoic eon**. Because organisms made from hard parts fossilize easier than those made of soft parts, fossils are easier to find in Paleozoic rocks than Precambrian rocks. Figure 11 shows the portion of the geologic time scale that contains the **Paleozoic era**.

The Cambrian Explosion

About five million years after the start of the **Cambrian period**, an event known as the Cambrian explosion occurred. The fossil record shows that during a relatively short period of time, the number of animals with shells greatly increased. Small-shelled organisms that looked like cones, plates, and tubes evolved along with larger-shelled organisms. Invertebrates, including sponges, jellies, and corals, also evolved during the Cambrian explosion. Trilobites, animals with hard outer skeletons, were the most commonly fossilized organisms of the Cambrian period. Some species of trilobites are shown in Figure 12.

Invertebrates of the Paleozoic Era

Throughout the **Paleozoic era**, the oceans contained a wide variety of invertebrate organisms including corals, brachiopods, crinoids, and bryozoans. Figure 13 shows what the ocean floor may have looked like during the **Ordovician period**.

Imagine a cockroach 10 cm long or a dragonfly with a wingspan of 74 cm. These insects would have lived during the Silurian period.

Vertebrates of the Paleozoic Era

Vertebrates, animals with backbones, evolved during the early **Paleozoic Era**. The first of these lived in the oceans.

Bony Fish Early vertebrates included two groups of bony fish. One group had thin, bony rays which supported their fins.

Another group of bony fish had thick fins supported by large bones and muscles. Scientists believe that the first group evolved into modern-day fish, while the second group evolved into amphibians that can live both in water and on land. Amphibians later evolved into reptiles and mammals.

A New Egg In order to reproduce, amphibians had to return to the water to lay their eggs. The water carries food and wastes into and out of the eggs. Amphibian eggs are not watertight and they would dry out and die on land. During the **Pennsylvanian period** a significant development occurred in the process of egg-laying.

Early in the **Pennsylvanian period** an organism evolved that could lay its eggs on land. The organism is called an amniote and it lays water-tight, amniotic eggs. This means the eggs do not dry out on land. Since plants had moved on land during the **Ordovician** and provided a food source, this allowed the amniotes to spend all their time on land. Mammals, dinosaurs, and reptiles all evolved from amniotes.

Plants of the Paleozoic Era

The first plants developed in the ocean from green algae. During the **Ordovician period**, plants spread onto land. Because these early plants could not move water and nutrients to all their parts, the plants remained small and lived in low, moist areas. Vascular plants then evolved with vascular systems that could move water and nutrients between their roots and leaves. Vascular club mosses were small, but by the Late **Devonian** they became the trees shown in Figure 14. Ferns and groups that include the present-day conifers appeared during the **Mississippian period**. Large swamps containing abundant trees and ferns developed during the **Mississippian and Pennsylvanian periods**. These plants decayed, and became large coal deposits.

Paleozoic Era Extinctions

The fossil record indicates that mass extinction events occurred during the **Ordovician period** and the **Devonian period**. The **Paleozoic era** ended with the late Permian extinction—the extinction of more than 90 percent of all marine species and more than 70 percent of all land species. Figure 15 shows how the ocean might have looked before and after an extinction event. Several hypotheses have been proposed to explain the Permian extinction. One proposes that the uplifting formation of Pangaea left little room for shallow-water life-forms as marine terraces became dry land. Another proposes that the Siberian Traps released ash and sulfur into the atmosphere, causing global cooling and the formation of glaciers on land.

Mass Extinction Ends the **Paleozoic Era** The early **Cambrian period** saw the expansion on Earth of many new species of life and the development of hard-shelled organisms that would leave fossil evidence of their existence. Before the **Paleozoic era** ended, there would be the evolution of the first fish, land plants, amphibians, and reptiles, all of which had hard parts that left fossil evidence. However, about 250 million years ago, the late Permian extinction resulted in the extinction of most marine and land species.

Changes in species diversity usually follow an extinction event. Niches and habitats once inhabited by some organisms became empty and other species moved into these spaces. Extinction of predators allowed other organisms to increase in number and expand in distribution.

Middle and Recent Earth History

Life continues to evolve into many of the forms we see on Earth today.

Real-World Reading Connection After it rains, you may see a puddle in the street or in your yard. In a day or two it is gone—it evaporated. You didn’t see it happen, but it was evaporating the whole time. Imagine how long it would take for a lake to evaporate. Now imagine the ocean evaporating into nothing. How long would it take?

The Mesozoic Era

Figure 17 on the facing page shows the three periods of the geologic time scale that make up the **Mesozoic era**—the **Triassic period, the Jurassic period, and the Cretaceous period**. Most organisms that survived the Permian mass extinction event diversified widely during the next 50 million years.

A Changing Landscape

About 180 million years ago, the Pangaea land mass began to split into two land masses, Laurasia and Gondwanaland, as shown in Figure 16. The split started a new weather pattern that would create a warm, wet climate for a large area of these land masses.

Invertebrates of the Mesozoic Era

After the extinction event at the end of the **Paleozoic era**, many of the predators of stromatolites had become extinct.

This allowed stromatolites to increase in numbers. Algae and large clams, like the rudistids, were the new life forms to evolve in the **Cretaceous period**. By the Triassic, how- ever, the rudistids had been replaced by reef-building corals. We can still find the descendants of crinoids and brachiopods that populated the Mesozoic oceans. They live in polar seas and in deep oceans. Although most groups of organisms became more widely diversified during the **Mesozoic era**, the insects that had become so abundant during the late **Paleozoic era** declined in number and diversity during the **Mesozoic era**.

Vertebrates of the Mesozoic Era

In addition to the fish that evolved during the **Paleozoic era**, the oceans of the Mesozoic era contained predatory reptiles such as plesiosaurs and mosasaurs. Amphibians, reptiles, and mammals that lived on land continued to evolve during the **Mesozoic era**. Frogs, turtles, crocodiles, pterosaurs, and dinosaurs evolved during this time.

Pterosaurs were flying reptiles. Many were the size of today’s predatory birds. The pterosaur shown in Figure 18 had a wingspan of 12 m and might have weighed 200 kg. Although pterosaurs shared many characteristics with dinosaurs, they were not dinosaurs. Pterosaurs differed from dinosaurs in that they had hollow bones, thin and translucent wings, extra bones in their hands or wings, and were probably covered with a hair-like material. Although they could fly like birds and bats, they are not a related species to either.

Dinosaurs

Dinosaurs evolved during the **Triassic period**. The first dinosaurs were small, but as new species evolved, they grew bigger. Apatosaurus and Diplodocus were some of the largest animals to walk on Earth. The first scientists to study dinosaur fossils thought their skulls, feet, and tails suggested that they were just very large reptiles. Because they imagined them to be reptiles, they believed that they lived and behaved like modern reptiles do today. That would mean that dinosaurs were ectotherms, relying on their surroundings and behavior to help them regulate their body temperatures. As such they would have to find sunlight to warm up and move into the shade to cool off. Recent studies of dinosaur bones show that their structure is more similar to endothermic species. Endotherms generate internal body heat to maintain a constant body temperature. Figure 19 shows a comparison of dinosaur and mammal bones. Rings in bones indicate the organism’s growth rate much like tree rings tell the age and rate of growth of a tree. Similarities in growth rate have led scientists to think that dinosaurs were endothermic and may have lived more like today’s mammals and birds than like reptiles. Other comparisons include speed of travel, carrying the tail and not dragging it, and having an upright stance.

Birds

By the 1970s scientists had proposed that birds are the descendants of dinosaurs. Read about the extinct reptiles in Figure 20 and think about the debate. It has been many millions of years since the time of dinosaurs. Birds have had many opportunities to evolve to fit into new and different environments. Did they become endothermic or have they always been endotherms?

Archaeopteryx

Once scientists discovered a dinosaur-like skeleton with fossilized feathers in Jurassic-aged rocks, they knew they had the support they needed for their theory that birds evolved from dinosaurs. Archaeopteryx is the name that has been given to one ancestral bird species. The fossil and a reconstruction are shown in

Figure 21.

Mammals evolved during the **Triassic period**, alongside the dinosaurs. Early mammals, shown in Figure 22, were small in size and not very numerous or diverse for most of the **Mesozoic era**. In the late **Cretaceous period**, they increased in both number and diversity. As dinosaur species became extinct, mammals were able to move into the niches that dinosaurs once occupied. You will read more about mammals later in this lesson.

Plants of the Mesozoic Era

Plants called gymnosperms dominated the plant population of the Mesozoic era. Gymnosperms produce seeds but no flowers. Some gymnosperms, such as the pine and ginkgo trees, still exist today. Angiosperms evolved near the end of the **Mesozoic era**.

Angiosperms are flowering plants that bear seeds with hard outer coverings. This gave them the ability to live in many different environments and to survive harsh climatic conditions. Figure 23 shows what a plant landscape might have looked like during the **Mesozoic era**.

Mesozoic Era Extinction Events

Scientists have observed evidence of extinction events in rocks of Mesozoic era age. The **Triassic period** ended with a mass extinction event that killed off approximately 20 percent of all marine families. Another mass extinction event occurred at the end of the **Jurassic period**. Several dinosaur groups including some of the largest dinosaurs, such as Diplodocus, became extinct during this event. One of the most well-known extinction events occurred at the end of the **Cretaceous period**. Almost 85 percent of all species in the oceans and all of the remaining dinosaurs became extinct, yet many mammal, bird, and reptile species survived. This event marked the end of the **Mesozoic era**.

The Cenozoic Era

Figure 24 shows the portion of the geologic time scale that includes the **Cenozoic era**. The **Cenozoic era** is divided into seven epochs. During the **Cenozoic era**, marine life began to recover from the Cretaceous mass extinction event. The off- spring of organisms that survived the Cretaceous extinction event make up the present marine ecosystems. A reconstruction of life in the early Cenozoic oceans is shown in Figure 25. How similar do these animals look to today’s marine populations?

Animals on Land

By the beginning of the **Cenozoic era**, the modern mammals had evolved. Some mammals returned to the ocean. During the

**Eocene epoch**, whales evolved from carnivorous, meat-eating, land mammals to the modern marine mammals they are today. Primates, animals with opposable thumbs and two eyes that look directly forward, evolved during the **Eocene epoch**. They diversified rapidly and lived in both the trees and on the ground. About 4.4 million years ago, during the **Pliocene epoch**, the hominids evolved. Hominids are descendants of these early primates. Hominids walk upright on two legs. The human species, Homo sapiens, belongs to the hominid group.

Plants of the Cenozoic Era

Recall that flowering plants evolved near the end of the **Mesozoic era**. They have continued to evolve ever since. Today, there are more than 250,000 species of flowering plants. Fruits, vegetables, and nuts are all produced by flowering plants.

Grasses are flowering plants and during the **Eocene epoch** supported a large diversity of mammals, which enabled the mammals to multiply and diversify.

Cenozoic-Era Extinctions

Cenozoic mass extinction events have not occurred on the scale of those previously discussed in this chapter. Some extinctions are the result of natural selection while others have been induced by humans.

Complexity Increases over Time

Fossil sequences from the **Archean eon** to the **Cenozoic era** show that complexity increases over time. Life began as simple bacteria. Then more complex organisms with shells evolved. Organisms became more diversified and continued to evolve from marine invertebrates to marine and land vertebrates and plants. The fossil record reveals this change.

**Textbook 4: Dynamics of Life Vol.1**

Mendel’s Laws of Heredity

Why Mendel Succeeded

People have noticed for thousands of years that family resemblances are inherited from generation to generation. However, it was not until the mid-nineteenth century that Gregor Mendel, an Austrian monk, carried out important studies of heredity—the passing on of characteristics from parents to offspring. Characteristics that are inherited are called traits. Mendel was the first person to succeed in predicting how traits are transferred from one generation to the next. A complete explanation requires the careful study of genetics—the branch of biology that studies heredity.

Mendel chose his subject carefully

Mendel chose to use the garden pea in his experiments for several reasons. Garden pea plants reproduce sexually, which means that they produce male and female sex cells, called gametes. The male gamete forms in the pollen grain, which is produced in the male reproductive organ. The female gamete forms in the female reproductive organ. In a process called fertilization, the male gamete unites with the female gamete. The resulting fertilized cell, called a zygote, then develops into a seed.

The transfer of pollen grains from a male reproductive organ to a female reproductive organ in a plant is called pollination. In peas, both organs are located in the same flower and are tightly enclosed by petals. This prevents pollen from other flowers from entering the pea flower. As a result, peas normally reproduce by self-pollination; that is, the male and female gametes come from the same plant. In many of Mendel’s experiments, this is exactly what he wanted. When he wanted to breed, or cross, one plant with another, Mendel opened the petals of a flower and removed the male organs, as shown in Figure 10.1A. He then dusted the female organ with pollen from the plant he wished to cross it with, as shown in Figure 10.1B. This process is called cross-pollination. By using this technique, Mendel could be sure of the parents in his cross.

Mendel was a careful researcher

Mendel carefully controlled his experiments and the peas he used. He studied only one trait at a time to control variables, and he analyzed his data mathematically. The tall pea plants he worked with were from populations of plants that had been tall for many generations and had always produced tall offspring. Such plants are said to be true breeding for tallness. Likewise, the short plants he worked with were true breeding for shortness.

Mendel’s Monohybrid Crosses

What did Mendel do with the tall and short pea plants he selected? He crossed them to produce new plants. Mendel referred to the offspring of this cross as hybrids. A hybrid is the offspring of parents that have different forms of a trait, such as tall and short height. Mendel’s first experiments are called monohybrid crosses because mono means “one” and the two parent plants differed from each other by a single trait—height.

The first generation

Mendel selected a six-foot-tall pea plant that came from a population of pea plants, all of which were over six feet tall. He cross-pollinated this tall pea plant with pollen from a short pea plant that was less than two feet tall and which came from a population of pea plants that were all short. When he planted the seeds from this cross, he found that all of the offspring grew to be as tall as the taller parent. In this first generation, it was as if the shorter parent had never existed.

The second generation

Next, Mendel allowed the tall plants in this first generation to self-pollinate. After the seeds formed, he planted them and counted more than 1000 plants in this second generation. Mendel found that three-fourths of the plants were as tall as the tall plants in the parent and first generations. He also found that one-fourth of the offspring were as short as the short plants in the parent generation. In other words, in the second generation, tall and short plants occurred in a ratio of about three tall plants to one short plant, as shown in Figure 10.2. The short trait had reappeared as if from nowhere.

The original parents, the true-breeding plants, are known as the P1 generation. The P stands for “parent.” The offspring of the parent plants are known as the F1 generation. The F stands for “filial”—son or daughter. When you cross two F1 plants with each other, their offspring are the F2 generation—the second filial generation. You might find it easier to understand these terms if you look at your own family. Your parents are the P1 generation. You are the F1 generation, and any children you might have in the future would be the F2 generation.

Mendel did similar monohybrid crosses with a total of seven pairs of traits, studying one pair of traits at a time. These pairs of traits are shown in Figure 10.3. In every case, he found that one trait of a pair seemed to disappear in the F1 generation, only to reappear unchanged in one-fourth of the F2 plants.

The rule of unit factors

Mendel concluded that each organism has two factors that control each of its traits. We now know that these factors are genes and that they are located on chromosomes. Genes exist in alternative forms. We call these different gene forms alleles. For example, each of Mendel’s pea plants had two alleles of the gene that determined its height. A plant could have two alleles for tallness, two alleles for shortness, or one allele for tallness and one for shortness. An organism’s two alleles are located on different copies of a chromosome—one inherited from the female parent and one from the male parent.

The rule of dominance

Remember what happened when Mendel crossed a tall P1 plant with a short P1 plant? The F1 offspring were all tall. In other words, only one trait was observed. In such crosses, Mendel called the observed trait dominant and the trait that disappeared recessive. Mendel concluded that the allele for tall plants is dominant to the allele for short plants. Thus, plants that had one allele for tallness and one for shortness were tall.

Expressed another way, the allele for short plants is recessive to the allele for tall plants. Pea plants with two alleles for tallness were tall, and those with two alleles for shortness were short. You can see in Figure 10.4 how the rule of dominance explained the resulting F1 generation. When recording the results of crosses, it is customary to use the same letter for different alleles of the same gene. An uppercase letter is used for the dominant allele and a lowercase letter for the recessive allele. The dominant allele is always written first. Thus, the allele for tallness is written as T and the allele for shortness as t, as it is in Figure 10.4.

The law of segregation

Now recall the results of Mendel’s cross between F1 tall plants, when the trait of shortness reappeared. To explain this result, Mendel formulated the first of his two laws of heredity. He concluded that each tall plant in the F1 generation carried one dominant allele for tallness and one unexpressed recessive allele for shortness. Each plant received the allele for tallness from its tall parent and the allele for shortness from its short parent in the P1 generation. Because each F1 plant has two different alleles, it can produce two types of gametes—“tall” gametes and “short” gametes. This conclusion, illustrated in Figure 10.5 on the next page, is called the law of segregation. **The law of segregation** states that every individual has two alleles of each gene and when gametes are produced, each gamete receives one of these alleles. During fertilization, these gametes randomly pair to produce four combinations of alleles.

Phenotypes and Genotypes

Mendel showed that tall plants are not all the same. Some tall plants, when crossed with each other, yielded only tall offspring. These were Mendel’s original P1 true-breeding tall plants. Other tall plants, when crossed with each other, yielded both tall and short offspring. These were the F1 tall plants in Figure 10.5 that came from a cross between a tall plant and a short plant.

Two organisms, therefore, can look alike but have different underlying allele combinations. The way an organism looks and behaves is called its phenotype. The phenotype of a tall plant is tall, whether it is TT or Tt. The allele combination an organism contains is known as its genotype. The genotype of a tall plant that has two alleles for tallness is TT. The genotype of a tall plant that has one allele for tallness and one allele for shortness is Tt. You can see that an organism’s genotype can’t always be known by its phenotype.

An organism is homozygous for a trait if its two alleles for the trait are the same. The true-breeding tall plant that had two alleles for tallness (TT) would be homozygous for the trait of height. Because tallness is dominant, a TT individual is homozygous dominant for that trait. A short plant would always have two alleles for shortness (tt). It would, therefore, always be homozygous recessive for the trait of height.

An organism is heterozygous for a trait if its two alleles for the trait differ from each other. Therefore, the tall plant that had one allele for tallness and one allele for shortness (Tt) is heterozygous for the trait of height.

Now look at Figure 10.5 again. Can you identify the phenotype and genotype of each plant? Is each plant homozygous or heterozygous? You can practice determining genotypes and phenotypes in the BioLab at the end of this chapter.

Mendel’s Dihybrid Crosses

Mendel performed another set of crosses in which he used peas that differed from each other in two traits rather than only one. Such a cross involving two different traits is called a dihybrid cross because di means “two.” In a dihybrid cross, will the two traits stay together in the next generation or will they be inherited independently of each other?

The first generation

Mendel	took true-breeding pea plants that had round yellow seeds (RRYY) and crossed them with true-breeding pea plants that had wrinkled green seeds (rryy). He already knew that when he crossed plants that produced round seeds with plants that produced	wrinkled	seeds, all the plants in the F1 generation produced seeds that were round. In other words, just as tall plants were dominant to short plants, the round-seeded	trait	was	dominant	to the wrinkled-seeded trait. Similarly, when he crossed plants that produced yellow seeds with plants that produced green seeds, all the plants in the F1 generation produced yellow seeds— yellow was dominant. Therefore, Mendel was not surprised when he found that the F1 plants of his dihybrid cross all had the two dominant traits of round and yellow seeds, as Figure 10.6 shows.

The second generation

Mendel then let the F1 plants pollinate themselves. As you might expect, he found some plants that produced round yellow seeds and others that produced wrinkled green seeds. But that’s not all. He also found some plants with round green seeds and others with wrinkled yellow seeds. When Mendel sorted and counted the plants of the F2 generation, he found they appeared in a definite ratio of phenotypes—9 round yellow:

3 round green: 3 wrinkled yellow: 1 wrinkled green. To explain the results of this dihybrid cross, Mendel formulated his second law.

The law of independent assortment

Mendel’s second law states that genes for different traits—for example, seed shape and seed color—are inherited independently of each other. This conclusion is known as the law of independent assortment. When a pea plant with the genotype RrYy produces gametes, the alleles R and r will separate from each other (the law of segregation) as well as from the alleles Y and y (the law of independent assortment), and vice versa. These alleles can then recombine in four different ways. If the alleles for seed shape and color were inherited together, only two kinds of pea seeds would have been produced: round yellow and wrinkled green.

Punnett Squares

In 1905, Reginald Punnett, an English biologist, devised a shorthand way of finding the expected proportions of possible genotypes in the offspring of a cross. This method is called a Punnett square. It takes account of the fact that fertilization occurs at random, as Mendel’s law of segregation states. If you know the genotypes of the parents, you can use a Punnett square to predict the possible genotypes of their offspring.

Monohybrid crosses

Consider the cross between two F1 tall pea plants, each of which has the genotype Tt. Half the gametes of each parent would contain the T allele, and the other half would contain the t allele. A Punnett square for this cross is two boxes tall and two boxes wide because each parent can produce two kinds of gametes for this trait. The two kinds of gametes from one parent are listed on top of the square, and the two kinds of gametes from the other parent are listed on the left side, as Figure 10.7A shows. It doesn’t matter which set of gametes is on top and which is on the side, that is, which parent contributes the T allele and which contributes the t allele. Refer to the Punnett square in Figure 10.7B to determine the possible genotypes of the offspring. Each box is filled in with the gametes above and to the left side of that box. You can see that each box then contains two alleles—one possible genotype.

After the genotypes have been determined, you can determine the phenotypes. Looking again at the Punnett square in Figure 10.7B, you can see that three-fourths of the offspring are expected to be tall because they have at least one dominant allele. One-fourth are expected to be short because they lack a dominant allele. Of the tall offspring, one-third will be homozygous dominant (TT) and two-thirds will be heterozygous (Tt). Note that whereas the genotype ratio is 1TT: 2Tt: 1tt, the phenotype ratio is 3 tall: 1 short. You can practice doing calculations such as Mendel did in the Connection to Math at the end of this chapter.

Dihybrid crosses

What happens in a Punnett square when two traits are considered? Think again about Mendel’s cross between pea plants producing round yellow seeds and plants producing wrinkled green seeds. All the F1 plants produced seeds that were round and yellow and were heterozygous for each trait (RrYy). What kind of gametes will these F1 plants form? Mendel explained that the traits for seed shape and seed color would be inherited independently of each other. This means that each F1 plant will produce gametes containing the following combinations of genes with equal frequency: round yellow (RY), round green (Ry), wrinkled yellow (rY), and wrinkled green (ry). A Punnett square for a dihybrid cross will then need to be four boxes on each side for a total of 16 boxes, as Figure 10.8 shows.

Probability

Punnett squares are good for showing all the possible combinations of gametes and the likelihood that each will occur. In reality, however, you don’t get the exact ratio of results shown in the square. That’s because, in some ways, genetics is like flipping a coin—it follows the rules of chance. When you toss a coin, it lands either heads up or tails up. The probability or chance that an event will occur can be determined by dividing the number of desired outcomes by the total number of possible outcomes. Therefore, the probability of getting heads when you toss a coin would be one in two chances, written as 1:2 or 1⁄2. A Punnett square can be used to determine the probability of getting a pea plant that produces round seeds when two plants that are heterozygous (Rr) are crossed.

The Punnett square in Figure 10.9 shows three plants with round seeds out of four total plants, so the probability is 3⁄4. Yet, if you calculate the probability of round-seeded plants from Mendel’s actual data in the Problem-Solving Lab on this page, you will see that slightly less than three-fourths of the plants were round-seeded. It is important to remember that the results predicted by probability are more likely to be seen when there is a large number of offspring.

Meiosis

Genes, Chromosomes, and Numbers

Organisms have tens of thousands of genes that determine individual traits. Genes do not exist free in the nucleus of a cell; they are lined up on chromosomes. Typically, a chromosome can contain a thousand or more genes along its length.

Diploid and haploid cells

If you examined the nucleus in a cell of one of Mendel’s pea plants, you would find it had 14 chromosomes—seven pairs. In the body cells of animals and most plants, chromosomes occur in pairs. One chromosome in each pair came from the male parent, and the other came from the female parent. A cell with two of each kind of chromosome is called a diploid cell and is said to contain a diploid, or 2n, number of chromosomes. This pairing supports Mendel’s conclusion that organisms have two factors—alleles—for each trait. One allele is located on each of the paired chromosomes.

Organisms produce gametes that contain one of each kind of chromosome. A cell containing one of each kind of chromosome is called a haploid cell and is said to contain a haploid, or n, number of chromosomes.

This fact supports Mendel’s conclusion that parent organisms give one factor, or allele, for each trait to each of their offspring.

Each species of organism contains a characteristic number of chromosomes. Table 10.1 shows the diploid and haploid numbers of chromosomes of some species. Note the large range of chromosome numbers. Note also that the chromosome number of a species is not related to the complexity of the organism.

Homologous chromosomes

The two chromosomes of each pair in a diploid cell are called homologous chromosomes. Each of a pair of homologous chromosomes has genes for the same traits, such as plant height. On homologous chromosomes, these genes are arranged in the same order, but because there are different possible alleles for the same gene, the two chromosomes in a homologous pair are not always identical to each other.

Let’s look at the seven pairs of homologous chromosomes in the cells of Mendel’s peas. These chromosome pairs are numbered 1 through 7. Each pair contains certain genes located at specific places on the chromosome. Chromosome 4 contains the genes for three of the traits that Mendel studied. Many other genes can be found on this chromosome as well.

Every pea plant has two copies of chromosome 4. It received one from each of its parents and will give one at random to each of its offspring. Remember, however, that the two copies of chromosome 4 in a pea plant may not necessarily have identical alleles. Each chromosome can have one of the different alleles possible for each gene. The homologous chromosomes diagrammed in Figure 10.10 show both alleles for each of three traits. Thus, the plant represented by these chromosomes is heterozygous for each of the traits.

Why meiosis?

When cells divide by mitosis, the new cells have exactly the same number and kind of chromosomes as the original cells. Imagine if mitosis were the only means of cell division. Each pea plant parent, which has 14 chromosomes, would produce gametes that contained a complete set of 14 chromosomes. That means that each offspring formed by fertilization of gametes would have twice the number of chromosomes as each of its parents. The F1 pea plants would have cell nuclei with 28 chromosomes, and the F2 plants would have cell nuclei with 56 chromosomes.

Clearly, there must be another form of cell division that allows offspring to have the same number of chromosomes as their parents. This kind of cell division, which produces gametes containing half the number of chromosomes as a parent’s body cell, is called meiosis. Meiosis occurs in the specialized body cells of each parent that produce gametes.

Meiosis consists of two separate divisions, known as meiosis I and meiosis II. Meiosis I begins with one diploid (2n) cell. By the end of meiosis II, there are four haploid (n) cells. These haploid cells are called sex cells— gametes. Male gametes are called sperm. Female gametes are called eggs. When a sperm fertilizes an egg, the resulting zygote once again has the diploid number of chromosomes.

The zygote then develops by mitosis into a multicellular organism. This pattern of reproduction, involving the production and subsequent fusion of haploid sex cells, is called sexual reproduction. It is illustrated in Figure 10.11.

The Phases of Meiosis

During meiosis, a spindle forms and the cytoplasm divides in the same ways they do during mitosis. However, what happens to the chromosomes in meiosis is very different. Figure 10.12 illustrates interphase and the phases of meiosis. Examine the diagram and photo of each phase as you read about it.

Interphase

Recall from Chapter 8 that, during **interphase**, the cell replicates its chromosomes. The chromosomes are replicated during **interphase** that precedes meiosis I, also. After replication, each chromosome consists of two identical sister chromatids, held together by a centromere.

Prophase I

A cell entering **prophase** I behaves in a similar way to one entering **prophase** of mitosis. The DNA of the chromosomes coils up and a spindle forms. As the DNA coils, homologous chromosomes line up with each other, gene by gene along their length, to form a four-part structure called a tetrad. A tetrad consists of two homologous chromosomes, each made up of two sister chromatids. The chromatids in a tetrad pair tightly. In fact, they pair so tightly that non-sister chromatids from homologous chromosomes can actually break and exchange genetic material in a process known as crossing over. Crossing over can occur at any location on a chromosome, and it can occur at several locations at the same time.

It is estimated that during **prophase** I of meiosis in humans, there is an everage of two to three crossovers for each pair of homologous chromosomes.

This exchange of genetic material is diagrammed in Figure 10.13B. Crossing over results in new combinations of alleles on a chromosome, as you can see in Figure 10.13C. You can practice modeling crossing over in the MiniLab at the left.

Metaphase I

During **metaphase** I, the centromere of each chromosome becomes attached to a spindle fiber. The spindle fibers pull the tetrads into the middle, or equator, of the spindle. This is an important step unique to meiosis. Note that homologous chromosomes are lined up side by side as tetrads. In mitosis, on the other hand, they line up on the spindle’s equator independently of each other.

Anaphase I

**Anaphase** I begins as homologous chromosomes, each with its two chromatids, separate and move to opposite ends of the cell. This separation occurs because the centromeres holding the sister chromatids together do not split as they do during **anaphase** in mitosis. This critical step ensures that each new cell will receive only one chromosome from each homologous pair.

Telophase I

Events occur in the reverse order from the events of **prophase** I. The spindle is broken down, the chromosomes uncoil, and the cytoplasm divides to yield two new cells. Each cell has half the genetic information of the original cell because it has only one chromosome from each homologous pair. However, another cell division is needed because each chromosome is still doubled.

The phases of meiosis II

The newly formed cells in some organisms undergo a short resting stage. In other organisms, however, the cells go from late **anaphase** of meiosis I directly to **metaphase** of meiosis II.

The second division in meiosis is simply a mitotic division of the products of meiosis I. Meiosis II consists of prophase II, metaphase II, anaphase II, and telophase II. During **prophase** II, a spindle forms in each of the two new cells and the spindle fibers attach to the chromosomes. The chromosomes, still made up of sister chromatids, are pulled to the center of the cell and line up randomly at the equator during **metaphase** II. **Anaphase** II begins as the centromere of each chromosome splits, allowing the sister chromatids to separate and move to opposite poles. Finally, nuclei reform, the spindles break down, and the cytoplasm divides during **telophase** II. The events of meiosis II are identical to those you studied for mitosis except that the chromosomes do not replicate before they divide at the centromeres.

At the end of meiosis II, four haploid cells have been formed from one diploid cell. Each haploid cell contains one chromosome from each homologous pair. These haploid cells will become gametes, transmitting the genes they contain to offspring.

Meiosis Provides for Genetic Variation

Cells that are formed by mitosis are identical to each other and to the parent cell. Crossing over during meiosis, however, provides a way to rearrange allele combinations. Rather than the alleles from each parent staying together, new combinations of alleles can form. Thus, variability is increased.

Genetic recombination

How many different kinds of sperm can a pea plant produce? Each cell undergoing meiosis has seven pairs of chromosomes. Because each of the seven pairs of chromosomes can line up at the cell’s equator in two different ways, 128 different kinds of sperm are possible (2n = 27 = 128).

In the same way, any pea plant can form 128 different eggs. Because any egg can be fertilized by any sperm, the number of different possible offspring is 16 384 (128 × 128). A simple example of how genetic recombination occurs is shown in Figure 10.14A. You can see that the gene combinations in the gametes vary depending on how each pair of homologous chromosomes lines up during **metaphase** I, a random process.

These numbers increase greatly as the number of chromosomes in the species increases. In humans, n = 23, so the number of different kinds of eggs or sperm a person can produce is more than 8 million (223). When fertilization occurs, 223 × 223, or 70 trillion, different zygotes are possible! It’s no wonder that each individual is unique.

In addition, crossing over can occur almost anywhere at random on a chromosome. This means that an almost endless number of different possible chromosomes can be produced by crossing over, providing additional variation to the variation already produced by the random assortment of chromosomes. This reassortment of chromosomes and the genetic information they carry, either by crossing over or by independent segregation of homologous chromosomes, is called genetic recombination. It is a major source of variation among organisms. Variation is important to a species because it is the raw material that forms the basis for evolution.

Meiosis explains Mendel’s results

The behavior of the chromosomes in meiosis provides the physical basis for explaining Mendel’s results. The segregation of chromosomes in **anaphase** I of meiosis explains Mendel’s observation that each parent gives one allele for each trait at random to each offspring, regardless of whether the allele is expressed. The segregation of chromosomes at random during **anaphase** I also explains how factors, or genes, for different traits are inherited independently of each other. Today, Mendel’s laws and the events of meiosis together form the foundation of the chromosome theory of heredity.

Nondisjunction

Although the events of meiosis usually proceed accurately, sometimes chromosomes fail to separate correctly. The failure of homologous chromosomes to separate properly during meiosis is called nondisjunction. Recall that during meiosis I, one chromosome from each homologous pair moves to each pole of the cell. In nondisjunction, both chromosomes of a homologous pair move to the same pole of the cell.

In one form of nondisjunction, two kinds of gametes result. One has an extra chromosome, and the other is missing a chromosome. The effects of nondisjunction are often seen after gametes fuse. For example, when a gamete with an extra chromosome is fertilized by a normal gamete, the zygote will have an extra chromosome. This condition is called trisomy. In humans, if a gamete with an extra chromosome number 21 is fertilized by a normal gamete, the resulting zygote has 47 chromosomes instead of 46. This zygote will develop into a baby with Down syndrome.

Although organisms with extra chromosomes often survive, organisms lacking one or more chromosomes usually do not. When a gamete with a missing chromosome fuses with a normal gamete during fertilization, the resulting zygote lacks a chromosome. This condition is called monosomy. In humans, most zygotes with monosomy do not survive. If a zygote with monosomy does survive, the resulting organism usually does not. An example of monosomy that is not lethal is Turner syndrome, in which human females have only a single X chromosome instead of two. Another form of nondisjunction involves a total lack of separation of homologous chromosomes. When this happens, a gamete inherits a complete diploid set of chromosomes, like those shown in Figure 10.15. When a gamete with an extra set of chromosomes is fertilized by a normal haploid gamete, the offspring has three sets of chromosomes and is triploid. The fusion of two gametes, each with an extra set of chromosomes, produces offspring with four sets of chromosomes—a tetraploid.

Polyploidy

Organisms with more than the usual number of chromosome sets are called polyploids. Polyploidy is rare in animals and almost always causes death of the zygote. However, polyploidy frequently occurs in plants. Often, the flowers and fruits of these plants are larger than normal, and the plants are healthier. Many polyploid plants, such as the sterile banana plant shown in Figure 10.17, are of great commercial value.

Meiosis is a complex process, and the results of an error occurring are sometimes unfortunate. However, the resulting changes can be beneficial, such as those that have occurred in agriculture. Hexaploid (6n) wheat, triploid (3n) apples, and polyploid chrysanthemums all are available commercially. You can see that a thorough understanding of meiosis and genetics would be very helpful to plant breeders. In fact, plant breeders have learned to produce polyploid plants artificially by using chemicals that cause nondisjunction.

Gene Linkage and Maps

Genes sometimes appear to be inherited together instead of independently. If genes are close together on the same chromosome, they usually are inherited together. These genes are said to be linked. In fact, all the genes on a chromosome usually are linked and inherited together. It is the chromosomes, rather than the individual genes, that follow Mendel’s **law of independent assortment**.

Linked genes may become separated on different homologous chromosomes as a result of crossing over. When crossing over produces new gene combinations, geneticists can use the frequencies of these new gene combinations to make a chromosome map showing the relative locations of the genes. Figure 10.16 illustrates this process.

DNA and Genes

DNA: The Molecule of Heredity

What is DNA?

Although the environment influences how an organism develops, the genetic information that is held in the molecules of DNA ultimately determines an organism’s traits. DNA achieves its control by determining the structure of proteins. Living things contain proteins. Your skin contains protein, your muscles contain protein, and your bones contain protein mixed with minerals. All actions, such as eating, running, and even thinking, depend on proteins called enzymes. Enzymes are critical for an organism’s function because they control the chemical reactions needed for life. Within the structure of DNA is the information for life—the complete instructions for manufacturing all the proteins for an organism.

DNA as the genetic material

In the early 1950s, many scientists believed that protein was the genetic material, mainly because the structure of these large molecules was so varied. In 1952, however, Alfred Hershey and Martha Chase performed experiments using radioactively labeled viruses that infect bacteria. These viruses were made of only protein and DNA. Hershey and Chase created two different types of viruses. One type had radioactive DNA and the other type had radioactive protein. Each type of virus infected a separate bacteria culture. Only the DNA entered the bacteria and produced new viruses. These results were convincing evidence that DNA is the genetic material.

The structure of nucleotides

DNA is capable of holding all its information because it is a very long molecule. Recall that DNA is a polymer made of repeating subunits called nucleotides. Nucleotides have three parts: a simple sugar, a phosphate group, and a nitrogenous base. The simple sugar in DNA, called deoxyribose, gives DNA its name—deoxyribonucleic acid. The phosphate group is composed of one atom of phosphorus surrounded by four oxygen atoms. A nitrogenous base is a carbon ring structure that contains one or more atoms of nitrogen. In DNA, there are four possible nitrogenous bases: adenine (A), guanine (G), cytosine (C), and thymine (T). Thus, in DNA there are four possible nucleotides, each containing one of these four bases, as shown in Figure 11.1.

Nucleotides join together to form long chains, with the phosphate group of one nucleotide bonding to the deoxyribose sugar of an adjacent nucleotide. The phosphate groups and deoxyribose molecules form the backbone of the chain, and the nitrogenous bases stick out like the teeth of a zipper. In DNA, the amount of adenine is always equal to the amount of thymine, and the amount of guanine is always equal to the amount of cytosine.

The structure of DNA

In 1953, James Watson and Francis Crick published a letter in a journal that was only one page in length, yet monumental in importance. Watson and Crick proposed that DNA is made of two chains of nucleotides held together by nitrogenous bases. Just as the teeth of a zipper hold the two sides of the zipper together, the nitrogenous bases of the nucleotides hold the two strands of DNA together with weak hydrogen bonds. Hydrogen bonds can form only between certain bases, so the bases on one strand determine the bases on the other strand. Specifically, adenine on one strand pairs only with thymine on the other strand, and guanine on one strand pairs only with cytosine on the other strand. These paired bases, called complementary base pairs, explain why adenine and thymine are always present in equal amounts. Likewise, the guanine-cytosine base pairs result in equal amounts of these nucleotides in DNA. Watson and Crick also proposed that DNA is shaped like a long zipper that is twisted into a coil like a spring. When something is twisted like a spring, the shape is called a helix. Because DNA is composed of two strands twisted together, its shape is called a double helix. This shape is shown in Figure 11.2.

The importance of nucleotide sequences

A cattail, a cat, and a catfish are all different organisms composed of different proteins. If you compare the chromosomes of these organisms, you will find that they all contain DNA made up of the same four nucleotides with adenine, thymine, guanine, and cytosine as their nitrogenous bases. How can organisms be so different from each other if their genetic material is made of the same four nucleotides?

Their differences result from the sequence of the four different nucleotides along the DNA strands, as shown in Figure 11.3.

The sequence of nucleotides forms the unique genetic information of an organism. For example, a nucleotide sequence of A-T-T-G-A-C carries different information from a sequence of T-C-C-A-A-A. In a similar way, two six-letter words made of the same letters but arranged in different order have different meanings. The closer the relationship is between two organisms, the more similar their DNA nucleotide sequences will be.

The DNA sequences of a chimpanzee are similar to those of a gorilla, but different from those of a rosebush. Scientists use nucleotide sequences to determine evolutionary relationships among organisms, to determine whether two people are related, and to identify bodies of crime victims.

Replication of DNA

A sperm cell and an egg cell of two fruit flies, both produced through meiosis, unite to form a fertilized egg. From one fertilized egg, a fruit fly with millions of cells is produced by the process of mitosis. Each cell has a copy of the DNA that was in the original fertilized egg. As you have learned, before a cell can divide by mitosis or meiosis, it must first make a copy of its chromosomes. The DNA in the chromosomes is copied in a process called DNA replication. Without DNA replication, new cells would have only half the DNA of their parents. Species could not survive, and individuals could not grow or reproduce successfully. All organisms undergo DNA replication. Figure 11.4B shows bacterial DNA replicating.

How DNA replicates

You have learned that a DNA molecule is composed of two strands, each containing a sequence of nucleotides. As you know, an adenine on one strand pairs with a thymine on the other strand. Similarly, guanine pairs with cytosine. Therefore, if you know the order of bases on one strand, you can predict the sequence of bases on the other, complementary strand. In fact, part of the process of DNA replication is done in just the same way. During replication, each strand serves as a pattern, or template, to make a new DNA molecule. How can a molecule serve as a template? Examine Figure 11.5 on the next page to find out.

Replication begins as an enzyme breaks the hydrogen bonds between bases that hold the two strands together, thus unzipping the DNA. As the DNA continues to unzip, nucleotides that are floating free in the surrounding medium are attached to their base pair by hydrogen bonding. Another enzyme bonds these nucleotides into a chain.

This process continues until the entire molecule has been unzipped and replicated. Each new strand formed is a complement of one of the original, or parent, strands. The result is the formation of two DNA molecules, each of which is identical to the original DNA molecule.

When all the DNA in all the chromosomes of the cell has been copied by replication, there are two copies of the organism’s genetic information. In this way, the genetic makeup of an organism can be passed on to new cells during mitosis or to new generations through meiosis followed by sexual reproduction.

From DNA to Protein

Genes and Proteins

The sequence of nucleotides in DNA contain information. This information is put to work through the production of proteins. Proteins fold into complex, three-dimensional shapes to become key structures and regulators of cell functions. Some proteins become important structures, such as the filaments in muscle tissue. Other proteins, such as enzymes, control chemical reactions that perform key life functions—breaking down glucose molecules in cellular respiration, digesting food, or making spindle fibers during mitosis. In fact, enzymes control all the chemical reactions of an organism. Thus, by encoding the instructions for making proteins, DNA controls cells.

You learned earlier that proteins are polymers of amino acids. The sequence of nucleotides in each gene contains information for assembling the string of amino acids that make up a single protein.

RNA

RNA, like DNA, is a nucleic acid. However, RNA structure differs from DNA structure in three ways, shown in Figure 11.6. First, RNA is single stranded—it looks like one-half of a zipper—whereas DNA is double stranded. The sugar in RNA is ribose; DNA’s sugar is deoxyribose.

Finally, both DNA and RNA contain four nitrogenous bases, but rather than thymine, RNA contains a similar base called uracil (U). Uracil forms a base pair with adenine in RNA, just as thymine does in DNA. What is the role of RNA in a cell?

Perhaps you have seen a car being built on an automobile assembly line. Complex automobiles are built in many simple steps. Engineers tell workers how to make the cars, and workers follow directions to build the cars on the assembly line. Suppliers bring parts to the assembly line so they can be installed in the car. Protein production is similar to car production. DNA provides workers with the instructions for making the proteins, and workers build the proteins. Other workers bring parts, the amino acids, over to the assembly line. The workers for protein synthesis are RNA molecules. They take from DNA the instructions on how the protein should be assembled, then—amino acid by amino acid—they assemble the protein.

There are three types of RNA that help build proteins. Extending the car-making analogy, you can consider all three of these RNA molecules to be workers in the protein assembly line. One type of RNA, messenger RNA (mRNA), brings instructions from DNA in the nucleus to the cell’s factory floor, the cytoplasm. On the factory floor, mRNA moves to the assembly line, a ribosome.

The ribosome, made of ribosomal RNA (rRNA), binds to the mRNA and uses the instructions to assemble the amino acids in the correct order. The third type of RNA, transfer RNA (tRNA) is the supplier. Transfer RNA delivers amino acids to the ribosome to be assembled into a protein.

Transcription

How does the information in DNA, which is found in the nucleus, move to the ribosomes in the cytoplasm? Messenger RNA carries this information through the nuclear envelope to the ribosomes for manufacturing proteins, just as a worker carries information from the engineers to the assembly line for manufacturing a car. In the nucleus, enzymes make an RNA copy of a portion of a DNA strand in a process called transcription.

The main difference between transcription and DNA replication is that transcription results in the formation of one single-stranded RNA molecule rather than a double-stranded DNA molecule.

Modeling transcription in the BioLab on pages 302–303 will help you to understand this process. You can find out how scientists use new microscopes to “watch” transcription take place by reading the Biotechnology at the end of the chapter.

RNA Processing

Not all the nucleotides in the DNA of eukaryotic cells carry instructions—or code—for making proteins. Genes usually contain many long noncoding nucleotide sequences, called introns (for intervening regions), that are scattered among the coding sequences.

Regions that contain information are called exons because they are expressed. When mRNA is transcribed from DNA, both introns and exons are copied. The introns must be removed from the mRNA before it can function to make a protein. Enzymes in the nucleus cut out the intron segments and paste the mRNA back together. The mRNA then leaves the nucleus and travels to the ribosome.

The Genetic Code

The nucleotide sequence transcribed from DNA to a strand of messenger RNA acts as a genetic message, the complete information for the building of a protein. Think of this message as being written in a language that uses nitrogenous bases as its alphabet. As you know, proteins contain chains of amino acids. You could say that the language of proteins uses an alphabet of amino acids. A code is needed to convert the language of mRNA into the language of proteins. There are 20 common amino acids, but mRNA contains only four types of bases. How can four bases form a code for all possible proteins?

Biochemists began to crack the genetic code when they discovered that a group of three nitrogenous bases in mRNA code for one amino acid. Each group is known as a codon. For example, the codon UUU results in the amino acid phenylalanine being placed in a protein.

Sixty-four combinations are possible when a sequence of three bases is used; thus, 64 different mRNA codons are in the genetic code, shown in Table 11.1. Some codons do not code for amino acids; they provide instructions for making the protein. For example, UAA is a stop codon indicating that the protein chain ends at that point. AUG is a start codon as well as the codon for the amino acid methionine. As you can see, more than one codon can code for the same amino acid. However, for any one codon, there can be only one amino acid.

All organisms use the same genetic code. For this reason, it is said to be universal, and this provides evidence that all life on Earth evolved from a common origin. From the chlorophyll of a birch tree to the digestive enzymes of a bison, a large number of proteins are produced from DNA. It may be hard to imagine that only four nucleotides can produce so many diverse proteins; yet, think about computer programming. You may have seen computer code, such as 00010101110000110. Through a binary language with only two options—zeros and ones—many types of software are created. From computer games to World Wide Web browsers, complex software is built by stringing together the zeros and ones of computer code into long chains. Likewise, complex proteins are built from the long chains of DNA carrying the genetic code.

Translation: From mRNA to Protein

How is the language of the nucleic acid mRNA translated into the language of proteins? The process of converting the information in a sequence of nitrogenous bases in mRNA into a sequence of amino acids in protein is known as translation. You can summarize transcription and translation by completing the MiniLab.

Translation takes place at the ribosomes in the cytoplasm. In prokaryotic cells, which have no nucleus, the mRNA is made in the cytoplasm. In eukaryotic cells, mRNA is made in the nucleus and travels to the cytoplasm. In the cytoplasm, a ribosome attaches to the strand of mRNA like a clothespin clamped onto a clothesline.

The role of transfer RNA

For proteins to be built, the 20 different amino acids dissolved in the cytoplasm must be brought to the ribosomes. This is the role of transfer RNA, modeled in Figure 11.8. Each tRNA molecule attaches to only one type of amino acid.

Correct translation of the mRNA message depends upon the joining of each mRNA codon with the correct tRNA molecule. How does a tRNA molecule carrying its amino acid recognize which codon to attach to? The answer involves base pairing. There is a sequence of three nucleotides on the opposite side of the transfer-RNA molecule from the amino-acid attachment site, that is the complement of the nucleotides in the codon. These three nucleotides are called an anticodon because they bind to the codon of the mRNA. The tRNA carries only the amino acid that the anticodon specifies. For example, one tRNA molecule for the amino acid cysteine has an anticodon of ACA. This anticodon binds to the mRNA codon UGU.

Translating the mRNA code

Follow the steps in Figure 11.9 as you read how translation occurs. As translation begins, a tRNA molecule brings the first amino acid to the mRNA strand that is attached to the ribosome, Figure 11.9A. The anticodon forms a base pair with the codon of the mRNA strand, Figure 11.9B. This places the amino acid in the correct position for forming a peptide bond with the next amino acid. The ribosome slides down the mRNA chain to the next codon, and a new tRNA molecule brings another amino acid, Figure 11.9C. The amino acids bond; the first tRNA releases its amino acid and detaches from the mRNA, Figure 11.9D. The tRNA molecule is now free to pick up and deliver another molecule of its specific amino acid to a ribosome. Again, the ribosome slides down to the next codon; a new tRNA molecule arrives, and its amino acid bonds to the previous one. A chain of amino acids begins to form. When a stop codon is reached, translation ends, and the amino acid strand is released from the ribosome, Figure 11.9E.

Amino acid chains become proteins when they are freed from the ribosome and twist and curl into complex three-dimensional shapes. Each protein chain forms the same shape every time it is produced. These proteins become enzymes and cell structures.

Now that you have followed the process of protein synthesis, you have seen that the pathway of information flows from DNA to mRNA to protein. This scheme is called the central dogma of biology and is found in all organisms from the simplest bacterium to the most complex plant and animal. The formation of protein, originating from the DNA code, produces the diverse and magnificent living world.

Genetic Changes

Mutations

If the DNA in all the cells of an adult human body were lined up end to end, it would stretch nearly 100 billion kilometers—60 times the distance from Earth to Jupiter. This DNA is the end result of thousands of replications, beginning with the DNA in a fertilized egg. Organisms have evolved many ways to protect their DNA from changes. In spite of these mechanisms, however, changes in the DNA occasionally do occur. Any change in the DNA sequence is called a mutation. Mutations can be caused by errors in replication, transcription, cell division, or by external agents. One such cause is discussed in Figure 11.10.

Mutations in reproductive cells

Mutations can affect the reproductive cells of an organism by changing the sequence of nucleotides within a gene in a sperm or an egg cell. If this cell takes part in fertilization, the altered gene would become part of the genetic makeup of the offspring. The mutation may produce a new trait or it may result in a protein that does not work correctly, resulting in structural or functional problems in cells and in the organism. Sometimes, the mutation results in a protein that is nonfunctional, and the embryo may not survive.

In some rare cases, a gene mutation may have positive effects. An organism may receive a mutation that makes it faster or stronger; such a mutation may help an organism—and its offspring—better survive in its environment.

You will learn later that mutations that benefit a species play an important role in the evolution of that species.

Mutations in body cells

What happens if powerful radiation, such as gamma radiation, hits the DNA of a nonreproductive cell, a cell of the body such as in skin, muscle, or bone? If the cell’s DNA is changed, this mutation would not be passed on to offspring. However, the mutation may cause problems for the individual. Damage to a gene may impair the function of the cell; for example, it may cause a cell in the stomach to lose its ability to make acid needed for digestion, or a skin cell may lose its elasticity. When that cell divides, the new cells also will have the same mutation. Many scientists suggest that the buildup of cells with less than optimal functioning is an important cause of aging.

Some mutations of DNA in body cells affect genes that control cell division. This can result in the cells growing and dividing rapidly, producing cancer. As you learned earlier, cancer is the uncontrolled dividing of cells.

Cancer results from gene mutations. For example, ultraviolet radiation in sunlight can change the DNA in skin cells. The cells reproduce rapidly, causing skin cancer.

The effects of point mutations

Consider what might happen if an incorrect amino acid were inserted into a growing protein chain during the process of translation. The mistake might affect the structure of the entire molecule. Such a problem can occur if a point mutation arises. A point mutation is a change in a single base pair in DNA.

A simple analogy can illustrate point mutations. Read the following two sentences. Notice what happens when a single letter in the first sentence is changed.

THE DOG BIT THE CAT. THE DOG BIT THE CAR.

As you can see, changing a single letter changes the meaning of the above sentence. Similarly, a change in a single nitrogenous base can change the entire structure of a protein because a change in a single amino acid can affect the shape of the protein.

Frameshift mutations

When the ribosome moves along the mRNA strand, a new amino acid is added to the protein for every codon on the mRNA strand. What would happen if a single base were lost from a DNA strand?

This new sequence with the deleted base would be transcribed into mRNA. But then, the mRNA would be out of position by one base. As a result, every codon after the deleted base would be different, as shown in Figure 11.11B. This mutation would cause nearly every amino acid in the protein after the deletion to be changed. In the sentence THE DOG BIT THE CAT, deleting a G would produce the sentence THE DOB ITT HEC AT. The same effect would result from the addition of a single base. A mutation in which a single base is added to or deleted from DNA is called a frameshift mutation because it shifts the reading of codons by one base. In general, point mutations are less harmful to an organism because they disrupt only a single codon. The MiniLab on the next page will help you distinguish point mutations from frameshift mutations, and the Problem-Solving Lab on this page will show you an example of a common mutation in humans.

Chromosomal Alterations

Changes may occur in chromosomes as well as in genes. Alterations to chromosomes may occur in a variety of ways. For example, sometimes parts of chromosomes are broken off and lost during mitosis or meiosis. Often, chromosomes break and then rejoin incorrectly. Sometimes, the parts join backwards or even join to the wrong chromosome. These structural changes in chromosomes are called chromosomal mutations.

Chromosomal mutations occur in all living organisms, but they are especially common in plants. Such mutations affect the distribution of genes to the gametes during meiosis. Homologous chromosomes do not pair correctly when one chromosome has extra or missing parts, so separation of the homologous chromosomes does not occur normally. Gametes that should have a complete set of genes may end up with extra copies or a complete lack of some genes.

Few chromosomal mutations are passed on to the next generation because the zygote usually dies. In cases where the zygote lives and develops, the mature organism is often sterile and thus incapable of producing offspring. The most important of these mutations—deletions, insertions, inversions, and translocations—are illustrated in Figure 11.12.

Causes of Mutations

Some mutations seem to just happen, perhaps as a mistake in base pairing during DNA replication. These mutations are said to be spontaneous. However, many mutations are caused by factors in the environment. Any agent that can cause a change in DNA is called a mutagen. Mutagens include radiation, chemicals, and even high temperatures.

Forms of radiation, such as X rays, cosmic rays, ultraviolet light, and nuclear radiation, are dangerous mutagens because the energy they contain can damage or break apart DNA. The breaking and reforming of a double-stranded DNA molecule can result in deletions. Some kinds of radiation can convert a base into a different, incorrect base or fuse two bases together.

Chemical mutagens include dioxins, asbestos, benzene, and formaldehyde, substances that are commonly found in buildings and in the environment, Figure 11.13. These mutagens are highly reactive substances that interact with the DNA molecule and cause changes. Chemical mutagens usually cause substitution mutations.

Repairing DNA

The cell processes that copy genetic material and pass it from one generation to the next are usually accurate. This accuracy is important to ensure the genetic continuity of both new cells and offspring. Yet, mistakes sometimes do occur. There are many sources of mutagens in an organism’s environment. Although many of these are due to human activities, others—such as cosmic rays from outer space—have affected living things since the beginning of life. Repair mechanisms that fix mutations in cells have evolved.

Much like a book editor, enzymes proofread the DNA and replace incorrect nucleotides with correct nucleotides. These repair mechanisms work extremely well, but they are not perfect. The greater the exposure to a mutagen such as UV light, the more likely is the chance that a mistake will not be corrected. Thus, it is wise for people to limit their exposure to mutagens.

RNA Transcription

Patterns of Heredity and Human Genetics

Mendelian Inheritance of Human Traits

Making a Pedigree

At some point, you have probably seen a family tree, either for your family or for someone else’s. A family tree traces a family name and various family members through successive generations. Through a family tree, you can identify the relationships among your cousins, aunts, uncles, grandparents, and great-grandparents.

Pedigrees illustrate inheritance

Geneticists often need to map the inheritance of genetic traits from generation to generation. A pedigree is a graphic representation of genetic inheritance. It is a diagram made up of a set of symbols that identify males and females, individuals affected by the trait being studied, and family relationships. At a glance, it looks very similar to any family tree. Some commonly used pedigree symbols are shown in Figure 12.1.

In a pedigree, a circle represents a female; a square represents a male. Shaded circles and squares represent individuals showing the trait being studied. Unshaded circles and squares designate individuals that do not show the trait. A half-shaded circle or square represents a carrier, a heterozygous individual. A horizontal line connecting a circle and a square indicates that the individuals are parents, and a vertical line connects parents with their offspring. Each horizontal row of circles and squares in a pedigree designates a generation, with the most recent generation shown at the bottom. The generations are identified in sequence by Roman numerals, and each individual is given an Arabic number.

Analyzing a pedigree

An example of a pedigree for a fictitious rare, recessive disorder in humans is shown in Figure 12.2. This genetic disorder could be any of several recessive disorders which shows up only if the affected person carries two recessive alleles for the trait. Follow this pedigree as you read how to analyze a pedigree.

Suppose individual III-1 in the pedigree wants to know the likelihood of passing on this allele to her children. By studying the pedigree, the individual will be able to determine the likelihood that she carries the allele. Notice that information can also be gained about other members of the family by studying the pedigree. For example, you know that I-1 and I-2 are both carriers of the recessive allele for the trait because they have produced II-3, who shows the recessive phenotype. If you drew a Punnett square for the mating of individuals I-1 and I-2, you would find, according to Mendelian segregation, that the ratio of homozygous dominant to heterozygous to homozygous recessive genotypes among their children would be 1:2:1. Of those genotypes possible for the members of generation II, only the homozygous recessive genotype will express the trait, which is the case for II-3.

You can’t tell the genotypes of II-4 and II-5, but they have a normal phenotype. If you look at the Punnett square you made, you can see that the probability that II-4 and II-5 are carriers is two in three for each because they can have only two possible genotypes—homozygous normal and heterozygous. The homozygous recessive genotype is not a possibility in these individuals because neither of the shows the affected phenotype.

Because none of the children in generation III are affected and because the recessive allele is rare in the general population, it is reasonably safe to assume that II-1 is not a carrier. You know that II-2 must be a carrier like her parents because she has passed on the recessive allele to subsequent generation IV. Because III-1 has one parent who is heterozygous and the other parent who is assumed to be homozygous normal, III-1 most likely has a one-in-two chance of being a carrier. If her parent II-1 had been heterozygous instead of homozygous normal, III-1’s chances of being a carrier are increased to two in three.

Simple Recessive Heredity

Most genetic disorders are caused by recessive alleles. You can practice calculating the chance that offspring will be born with some of these genetic traits in the Problem-Solving Lab on this page.

Cystic fibrosis

Cystic fibrosis (CF) is a fairly common genetic disorder among white Americans. Approximately one in 28 white Americans carries the recessive allele, and one in 2500 children born to white Americans inherits the disorder. Due to a defective protein in the plasma membrane, cystic fibrosis results in the formation and accumulation of thick mucus in the lungs and digestive tract. Physical therapy, special diets, and new drugs have continued to raise the average life expectancy of people that have CF.

Tay-Sachs disease

Tay-Sachs disease is a recessive disorder of the central nervous system. In this disorder, a recessive allele results in the absence of an enzyme that normally breaks down a lipid produced and stored in tissues of the central nervous system. Because this lipid fails to break down properly, it accumulates in the cells. The allele for Tay-Sachs is especially common in the United States among Ashkenazic Jews, whose ancestors came from eastern Europe. Figure 12.3 shows a typical pedigree for Tay-Sachs disease.

Phenylketonuria

Phenylketonuria, also called PKU, is a recessive disorder that results from the absence of an enzyme that converts one amino acid, phenylalanine, to a different amino acid, tyrosine. Because phenylalanine cannot be broken down, it and its by-products accumulate in the body and result in severe damage to the central nervous system. The PKU allele is most common in the United States among people whose ancestors came from Norway, Sweden, or Ireland.

A homozygous PKU newborn appears healthy at first because its mother’s normal enzyme level prevented phenylalanine accumulation during development. However, once the infant begins drinking milk, which is rich in phenylalanine, the amino acid accumulates and mental retardation occurs. Today, a PKU test is normally performed on all infants a few days after birth. Infants affected by PKU are given a diet that is low in phenylalanine until their brains are fully developed. With this special diet, the toxic effects of the disorder can be avoided.

Ironically, the success of treating phenylketonuria infants has resulted in a new problem. If a female who is homozygous recessive for PKU becomes pregnant, the high phenylalanine levels in her blood can damage her fetus—the developing baby. This problem occurs even if the fetus is heterozygous and would be phenotypically normal.

You may have noticed PKU warnings on cans of diet soft drinks. Because most diet foods are sweetened with an artificial sweetener that contains phenylalanine, a pregnant woman who is homozygous recessive must limit her intake of diet foods.

Simple Dominant Heredity

Unlike the inheritance of recessive traits in which a recessive allele must be inherited from both parents for a person to show the recessive phenotype, many traits are inherited just as the rule of dominance predicts. Remember that in Mendelian inheritance, a single dominant allele inherited from one parent is all that is needed for a person to show the dominant trait.

Simple dominant traits

A cleft chin is one example of a simple dominant trait. If you have a cleft chin, you’ve inherited the dominant allele from at least one of your parents. A widow’s peak hairline and earlobe types, shown in Figure 12.4, are other dominant traits that are determined by simple Mendelian inheritance. Having earlobes that are attached to the head is a recessive trait (ff), whereas heterozygous (Ff) and homozygous dominant (FF) individuals have earlobes that hang freely.

There are many other human traits that are inherited by simple dominant inheritance. Figure 12.5 shows one of these traits—hitchhiker’s thumb, the ability to bend your thumb tip backward more than 30 degrees. A straight thumb is recessive. Other dominant traits in humans include almond- shaped eyes (round eyes are recessive), thick lips (thin lips are recessive), and the presence of hair on the middle section of your fingers.

Huntington’s disease

Huntington’s disease is a lethal genetic disorder caused by a rare dominant allele. It results in a breakdown of certain areas of the brain. No effective treatment exists.

Ordinarily, a dominant allele with such severe effects would result in death before the affected individual could have children and pass the allele on to the next generation. But because the onset of Huntington’s disease usually occurs between the ages of 30 and 50, an individual may already have had children before knowing whether he or she is affected. A genetic test has been developed that detects the presence of this allele. Although this test allows individuals with the allele to decide whether they want to have children and risk passing the trait on to future generations, it also means that they know they will develop the disease. For this reason, some people may choose not to be tested. The pedigree in Figure 12.6 shows a typical pattern for the occurrence of Huntington’s disease in a family.

When Heredity Follows Different Rules

Complex Patterns of Inheritance

Patterns of inheritance that are explained by Mendel’s experiments are often referred to as simple Mendelian inheritance—inheritance controlled by dominant and recessive paired alleles. However, many inheritance patterns are more complex than those studied by Mendel. As you will learn, most traits are not simply dominant or recessive. The BioLab at the end of this chapter investigates a type of inheritance that doesn’t even involve chromosomes.

Incomplete dominance: Appearance of a third phenotype

When inheritance follows a pattern of dominance, heterozygous and homozygous dominant individuals both have the same phenotype. When traits are inherited in an incomplete dominance pattern, however, the phenotype of heterozygous individuals is intermediate between those of the two homozygotes. For example, if a homozygous red-flowered snap-dragon plant (RR) is crossed with a homozygous white-flowered snap-dragon plant (R'R'), all of the F1offspring will have pink flowers.

Figure 12.7 shows that the intermediate pink form of the trait occurs because neither allele of the pair is completely dominant. Note that the letters R and R', rather than R and r, are used to show incomplete dominance.

The new phenotype occurs because the flowers contain enzymes that control pigment production. The R allele codes for an enzyme that produces a red pigment. The R' allele codes for a defective enzyme that makes no pigment. Because the heterozygote has only one copy of the R allele, its flowers appear pink because they produce only half the amount of red pigment that red homozygote flowers produce. The R'R' homozygote has no normal enzyme, produces no red pigment, and appears white.

Note that the segregation of alleles is the same as in simple Mendelian inheritance. However, because neither allele is dominant, the plants of the F1 generation all have pink flowers. When pink-flowered F1 plants are crossed with each other, the offspring in the F2 generation appear in a 1:2:1 phenotypic ratio of red to pink to white flowers. This result supports Mendel’s law of segregation.

Explain why snap-dragon heterozygotes have flowers with an intermediate color.

Codominance: Expression of both alleles

In chickens, black-feathered and white-feathered birds are homozygotes for the B and W alleles, respectively. Two different uppercase letters are used to represent the alleles in codominant inheritance.

One of the resulting heterozygous offspring in a breeding experiment between a black rooster and a white hen is shown in Figure 12.8. You might expect that heterozygous chickens, BW, would be black if the pattern of inheritance followed Mendel’s law of dominance, or gray if the trait were incompletely dominant. Notice, however, that the heterozygote is neither black nor gray. Instead, all of the offspring are checkered; some feathers are black and other feathers are white. In such situations, the inheritance pattern is said to be codominant. Codominant alleles cause the phenotypes of both homozygotes to be produced in heterozygous individuals. In codominance, both alleles are expressed equally.

Multiple phenotypes from multiple alleles

Although each trait has only two alleles in the patterns of heredity you have studied thus far, it is common for more than two alleles to control a trait in a population. This is understandable when you recall that a new allele can be formed any time a mutation occurs in a nitrogenous base somewhere within a gene. Although only two alleles of a gene can exist within an individual diploid cell, multiple alleles for a single gene can be studied in a population of organisms.

Traits controlled by more than two alleles have multiple alleles. The pigeons pictured in Figure 12.9 show the effects of multiple alleles for feather color. Three alleles of one gene govern their feather color, although each pigeon can have only two of these alleles. The number of alleles for any particular trait is not limited to three, and there are instances in which more than 100 alleles are known to exist for a single trait!

Sex determination

Recall that in humans the diploid number of chromosomes is 46, or 23 pairs. There are 22 pairs of homologous chromosomes called autosomes. Homologous autosomes look alike. The 23rd pair of chromosomes differs in males and females. These two chromosomes, which determine the sex of an individual, are called sex chromosomes and are indicated by the letters X and Y. If you are female, your 23rd pair of chromosomes are homologous, XX, as in Figure 12.10A. However, if you are male, your 23rd pair of chromosomes, XY, look different. Males usually have one X and one Y chromosome and produce two kinds of gametes, X and Y. Females usually have two X chromosomes and produce only X gametes. It is the male gamete that determines the sex of the offspring. Figure 12.10B shows that after fertilization, a 1:1 ratio of males to females is expected. Because fertilization is governed by the laws of probability, the ratio usually is not exactly 1:1 in a small population.

Sex-linked inheritance

Drosophila, commonly known as fruit flies, inherit sex chromosomes in the same way as humans. Traits controlled by genes located on sex chromosomes are called sex-linked traits. The alleles for sex-linked traits are written as superscripts of the X or Y chromosome. Because the X and Y chromosomes are not homologous, the Y chromosome has no corresponding allele to one on the X chromosome and no superscript is used. Also remember that any recessive allele on the X chromosome of a male will not be masked by a corresponding dominant allele on the Y chromosome.

In 1910, Thomas Hunt Morgan discovered traits linked to sex chromosomes. Morgan noticed one day that one male fly had white eyes rather than the usual red eyes. He crossed the white-eyed male with a homozygous red-eyed female. All of the F1 offspring had red eyes, indicating that the white-eyed trait is recessive. Then Morgan allowed the F1 flies	to	mate	among	themselves.

According to simple Mendelian inheritance, if the trait were recessive, the offspring in the F2 generation would show a 3:1 ratio of red-eyed to white-eyed flies. As you can see in Figure 12.11, this is what Morgan observed. However, he also noticed that the trait of white eyes appeared only in male flies.

Morgan hypothesized that the red-eye allele was dominant and the white-eye allele was recessive. He also reasoned that the gene for eye color was located on the X chromosome and was not present on the Y chromosome. In heterozygous females, the dominant allele for red eyes masks the recessive allele for white eyes. In males, however, a single recessive allele is expressed as a white-eyed phenotype. When Morgan crossed a heterozygous red-eyed female with a white-eyed male, half of all the males and half of all the females inherited white eyes. The only explanation of these results is **Morgan’s hypothesis**:

The allele for eye color is carried on the X chromosome and the Y chromosome has no corresponding allele for eye color.

The genes that govern sex-linked traits follow the inheritance pattern of the sex chromosome on which they are found. Eye color in fruit flies is an example of an X-linked trait. Y-linked traits are passed only from a male to male offspring because the genes for these traits are on the Y chromosome.

Polygenic inheritance

Some traits, such as skin color and height in humans, and cob length in corn, vary over a wide range. Such ranges occur because these traits are governed by many genes. Polygenic inheritance is the inheritance pattern of a trait that is controlled by two or more genes. The genes may be on the same chromosome or on different chromosomes, and each gene may have two or more alleles. For simplicity, uppercase and lowercase letters are used to represent the alleles, as they are in Mendelian inheritance. Keep in mind, however, that the allele represented by an uppercase letter is not dominant. All heterozygotes are intermediate in phenotype.

In polygenic inheritance, each allele represented by an uppercase letter contributes a small, but equal, portion to the trait being expressed. The result is that the phenotypes usually show a continuous range of variability from the minimum value of the trait to the maximum value.

Suppose, for example, that stem length in a plant is controlled by three different genes: A, B, and C. Each gene is on a different chromosome and has two alleles, which can be represented by uppercase or lowercase letters. Thus, each diploid plant has a total of six alleles for stem length. A plant that is homozygous for short alleles at all three gene locations (aabbcc) might grow to be only 4 cm tall, the base height. A plant that is homozygous for tall alleles at all three gene locations (AABBCC) might be 16 cm tall. The difference between the tallest possible plant and the shortest possible plant is 12 cm, or 2 cm per each tall allele.

Suppose a 16-cm-tall plant were crossed with a 4-cm-tall plant. In the F1 generation, all offspring would be AaBbCc. If each tall gene A, B, and C contributed 2 cm of height to the base height of Stem Length Variation in a Plant Polygenic for the Trait 4 cm, the expected height of these plants would be 10 cm (4 cm + 6 cm)—an intermediate height. If they are allowed to interbreed, the F2 offspring will show a range of heights. A Punnett square of this trihybrid cross would show that 10-cm-tall plants are most often expected, and the tallest and shortest plants are seldom expected. Notice in Figure 12.12 that when these results are graphed, the shape of the graph confirms the prediction of the Punnett square.

Environmental Influences

Even when you understand dominance and recessiveness, and you have solved the puzzles of the other patterns of heredity, the inheritance picture is not complete. The genetic makeup of an organism at fertilization determines only the organism’s potential to develop and function. As the organism develops, many factors can influence how the gene is expressed, or even whether the gene is expressed at all. Two such influences are the organism’s external and internal environments.

Influence of external environment

Sometimes, individuals	known to have a particular gene fail to express the phenotype specified by that gene. Temperature, nutrition, light, chemicals, and infectious agents all can influence gene expression. In Siamese cats and arctic foxes, as shown in Figure 12.13, temperature has an effect on the expression of coat color. External influences can also be seen in leaves. Leaves can have different sizes, thicknesses, and shapes depending on the amount of light they receive.

Influence of internal environment

The internal environments of males and females are different because of hormones and structural differences. For example, horn size in mountain sheep is expressed differently in males and females, as shown in Figure 12.14.

Male-pattern baldness in humans, and feather color in peacocks also are expressed differently in the sexes, as shown in Figure 12.15. These differences are controlled by different hormones, which are determined by different sets of genes.

An organism’s age can also affect gene function. The nature of such a pattern is not well understood, but it is known that the internal environment of an organism changes with age.

Understanding how genes interact with each other and with the environment gives a more complete picture of inheritance. Mendel’s idea that heredity is a composite of many individual traits still holds. Later researchers have filled in more details of Mendel’s great contributions.

Complex Inheritance of Human Traits

Codominance in Humans

Remember that in codominance, the phenotypes of both homozygotes are produced in the heterozygote. One example of this in humans is a group of inherited red blood cell disorders called sickle-cell anemia.

Sickle-cell anemia

Sickle-cell anemia is a major health problem in the United States and in Africa. In the United States, it is most common in black Americans whose families originated in Africa and in white Americans whose families originated in the countries surrounding the Mediterranean Sea. About one in 12 African Americans, a much larger proportion than in most populations, is heterozygous for the disorder.

In an individual who is homozygous for the sickle-cell allele, the oxygen-carrying protein hemoglobin differs by one amino acid from normal hemoglobin. This defective hemoglobin forms crystal-like structures that change the shape of the red blood cells. Normal red blood cells are disc- shaped, but abnormal red blood cells are shaped like a sickle, or half-moon.

The change in shape occurs in the body’s narrow capillaries after the hemoglobin delivers oxygen to the cells. Abnormally shaped blood cells, like the one shown in Figure 12.16A, slow blood flow, block small vessels, and result in tissue damage and pain. Because sickled cells block blood flow and have a shorter life span than normal red blood cells, the person can have several related disorders.

Individuals who are heterozygous for the allele produce both normal and sickled hemoglobin, an example of codominance. They produce enough normal hemoglobin that they do not have the serious health problems of those homozygous for the allele and can lead relatively normal lives. Individuals who are heterozygous are said to have the sickle-cell trait because they can show some signs of sickle-cell-related disorders if the availability of oxygen is reduced.

Multiple Alleles Govern Blood Type

You have learned that more than two alleles of a gene are possible for certain traits. Mendel’s laws of heredity also can be applied to traits that have more than two alleles. The ABO blood group is a classic example of a single gene that has multiple alleles in humans.

Human blood types, listed in the table in Figure 12.17, are determined by the presence or absence of certain molecules on the surfaces of red blood cells. As the determinant of blood types A, B, AB, and O, the gene I has three alleles: IA, IB, and i. Study the table to see the genotypes of the different blood types.

The importance of blood typing

Determining blood type is necessary before a person can receive a blood transfusion because the red blood cells of incompatible blood types could clump together, causing death. Blood typing also can be helpful in solving cases of disputed parentage. For example, if a child has type AB blood and his or her mother has type A, a man with type O blood could not possibly be the father. But blood tests cannot prove that a certain man definitely is the father; they indicate only that he could be. DNA tests are necessary to determine actual parenthood.

Sex-Linked Traits in Humans

Many human traits are determined by genes that are carried on the sex chromosomes; most of these genes are located on the X chromosome. The pattern of sex-linked inheritance is explained by the fact that males, who are XY, pass an X chromosome to each daughter and a Y chromosome to each son. Females, who are XX, pass one of their X chromosomes to each child, as illustrated in Figure 12.18. If a son receives an X chromosome with a recessive allele, the recessive phenotype will be expressed because he does not inherit on the Y chromosome from his father a dominant allele that would mask the expression of the recessive allele.

Two traits that are governed by X-linked recessive inheritance in humans are red-green color blindness and hemophilia. X-linked dominant and Y-linked human disorders are rare. Determine whether Duchenne’s muscular dystrophy is sex-linked by completing the Problem-Solving Lab on this page.

Red-green color blindness

People who have red-green color blindness can’t differentiate these two colors. Color blindness is caused by the inheritance of a recessive allele at either of two gene sites on the X chromosome. Both genes affect red and green receptors in the cells of the eyes. A serious problem for people with this disorder is the inability to identify red and green traffic lights by color.

Hemophilia: An X-linked disorder

Did you ever notice that most cuts stop bleeding quickly? This human adaptation is essential. If your blood didn’t have the ability to clot, any cut could take a long time to stop bleeding. Of greater concern would be internal bleeding resulting from a bruise, which a person may not immediately notice.

Hemophilia A is an X-linked disorder that causes such a problem with blood clotting. About one male in every 10 000 has hemophilia, but only about one in 100 million females inherits the same disorder. Why? Males inherit the allele for hemophilia on the X chromosome from their carrier mothers. One recessive allele for hemophilia will cause the disorder in males. Females would need two recessive alleles to inherit hemophilia. The family of Queen Victoria, pictured in the Connection to Social Studies at the end of this chapter, is the best-known study of hemophilia A, also called royal hemophilia. Hemophilia A can be treated with blood transfusions and injections of Factor VIII, the blood-clotting enzyme that is absent in people affected by the condition. However, both treatments are expensive. New methods of DNA technology are being used to develop a cheaper source of the clotting factor.

Polygenic Inheritance in Humans

Think of all the traits you inherited from your parents. Although many of your traits were inherited through simple Mendelian patterns or through multiple alleles, many other human traits are determined by polygenic inheritance. These kinds of traits usually represent a range of variation that is measurable.

Skin color: A polygenic trait

In the early 1900s, the idea that polygenic inheritance occurs in humans was first tested using data collected on skin color. Scientists found that when light-skinned people mate with dark-skinned people, their offspring have intermediate skin colors. When these children produce the F2 generation, the resulting skin colors range from the light-skin color to the dark-skin color of the grandparents (the P1 generation), with most children having an intermediate skin color. As shown in Figure 12.19, the variation in skin color indicates that between three and four genes are involved.

Changes in Chromosome Numbers

You have been reading about traits that are caused by one or several genes on chromosomes. What would happen if an entire chromosome or part of a chromosome were missing from the complete set? What if a cell’s nucleus contained an extra chromosome? As you have learned, abnormal numbers of chromosomes in offspring usually, but not always, result from accidents of meiosis. Many abnormal phenotypic effects result from such mistakes.

Abnormal numbers of autosomes

You know that a human usually has 23 pairs of chromosomes, or

46 chromosomes altogether. Of these 23 pairs of chromosomes, 22 pairs are autosomes. Humans who have an extra whole or partial autosome are trisomic—that is, they have three of a particular autosomal chromosome instead of just two. In other words, they have 47 chromosomes. Recall that trisomy usually results from nondisjunction, which occurs when paired homologous chromosomes fail to separate properly during meiosis.

To identify an abnormal number of chromosomes, a sample of cells is obtained from an individual or from a fetus. Metaphase chromosomes are photographed; the chromosome pictures are then enlarged and arranged in pairs by a computer according to length and location of the centromere, as shown in Figure 12.20. This chart of chromosome pairs is called a karyotype, and it is valuable in identifying unusual chromosome numbers in cells.

Down syndrome: Trisomy 21

Most human abnormal chromosome numbers result in embryo death often before a woman even realizes she is pregnant. Fortunately, these rarely occur. Down syndrome is the only autosomal trisomy in which affected individuals survive to adulthood. It occurs in about one in 700 live births.

Down syndrome is a group of symptoms that results from trisomy of chromosome 21. Individuals who have Down syndrome have at least some degree of mental retardation. The incidence of Down syndrome births is higher in older mothers, especially those over 40.

Abnormal numbers of sex chromosomes

Many abnormalities in the number of sex chromosomes are known to exist. An X chromosome may be missing (designated as XO) or there may be an extra one (XXX or XXY). There may also be an extra Y chromosome (XYY), as you can see by examining Figure 12.20. Any individual with at least one Y chromosome is a male, and any individual without a Y chromosome is a female. Most of these individuals lead normal lives, but they cannot have children and some have varying degrees of mental retardation.

Genetic Technology

Applied Genetics

Selective Breeding

For thousands of years, humans have selected plants and animals with certain qualities, and selectively bred them so that the qualities were common and of more use to humans. The same principle of selective breeding is still used today, in the food we eat and the animals we raise.

From ancient times, breeders have chosen plants and animals with the most desired traits to serve as parents of the next generation. Farmers select seeds from the largest heads of grain, the juiciest berries, and the most disease-resistant clover. They raise the calves of the best milk producer and save the eggs of the best egg-laying hen for hatching. Breeders of plants and animals want to be sure that their populations breed consistently so that each member shows the desired trait. You can read about the selective breeding of domesticated cats on pages 1066–1067 in the Focus On.

The process of selective breeding requires time, patience, and several generations of offspring before the desired trait becomes common in a population. Although our ancestors did not realize it, their efforts at selective breeding increased the frequency of a desired allele within a population. Increasing the frequency of desired alleles in a population is the essence of genetic technology.

One example of the effectiveness of selective breeding is seen in a comparison of milk production in cattle in 1947 and 1997. In 1947, an average milk cow produced 4997 pounds of milk per year. In 1997, 50 years later, an average milk cow produced 16 915 pounds of milk in a year, more than three times more milk per cow. Fewer than half the number of cows are now needed to produce the same amount of milk, resulting in savings for dairy farmers.

Inbreeding develops pure lines

To make sure that breeds consistently exhibit a trait and to eliminate any undesired traits from their breeding lines, breeders often use the method of inbreeding. Inbreeding is mating between closely related individuals. It results in offspring that are homozygous for most traits. However, inbreeding can bring out harmful, recessive traits because there is a greater chance that two closely related individuals both may carry a harmful recessive allele for the trait.

Horses and dogs are two examples of animals that breeders have developed as pure breeds. A breed (called a cultivar in plants) is a selected group of organisms within a species that has been bred for particular characteristics. For example, the pure breed German shepherd dog in Figure 13.1 has long hair, is black with a buff-colored base, has a black muzzle, and resembles a wolf.

Hybrids are usually bigger and better

Selective breeding of plants can increase productivity of food for humans. For example, plants that are disease resistant can be crossed with others that produce larger and more numerous fruit. The result is a plant that will produce a lot of fruit and be more disease resistant. Recall that a hybrid is the offspring of parents that have different forms of a trait. When two cultivars or closely related species are crossed, their offspring will be hybrids. Hybrids produced by crossing two purebred plants are often larger and stronger than their parents. Many crop plants such as wheat, corn, and rice, and garden flowers such as roses and dahlias have been developed by hybridization. Figure 13.2 shows some examples.

Determining Genotypes

A good breeder must be careful to determine which plants or animals will have the greatest chances of transmitting a desired trait to the next generation. Choosing the best parents may be difficult. The genotype of an organism that is homozygous recessive for a trait is obvious to an observer because the recessive trait is expressed. However, organisms that are either homozygous dominant or heterozygous for a trait controlled by Mendelian inheritance have the same phenotype. How can a breeder learn which genotype should be used for breeding?

Test crosses can determine genotypes

One way to determine the genotype of an organism is to perform a test cross. A test cross is a cross of an individual of unknown genotype with an individual of known genotype. The pattern of observed phenotypes in the offspring can help determine the unknown genotype of the parent. Usually, the parent with the known genotype is homozygous recessive for the trait in question.

Many traits, such as disease vulnerability in roses and progressive blindness in German shepherd dogs, are inherited as recessive alleles. These traits are maintained in a population by carriers of the trait. A carrier, or heterozygous individual, has the same phenotype as an individual that is homozygous dominant.

What are the possible results of a test cross? If a known parent is homozygous recessive and an unknown parent is homozygous dominant for a trait, all of the offspring will be heterozygous and show the dominant trait (be phenotypically dominant), as shown in Figure 13.3B on the next page. However, if the organism being tested is heterozygous, the expected 1:1 phenotypic ratio will be observed, Figure 13.3C. If any of the offspring have the undesired trait, the parent in question must be heterozygous.

Recombinant DNA Technology

Genetic Engineering

You learned that selective breeding increases the frequency of an allele in a population. You also learned that it may take many generations of breeding for a trait to become homozygous and consistently expressed in the population. Genetic engineering is a faster and more reliable method for increasing the frequency of a specific allele in a population. This method involves cutting—or cleaving—DNA from one organism into small fragments and inserting the fragments into a host organism of the same or a different species. You also may hear genetic engineering referred to as recombinant DNA technology. Recombinant DNA is made by connecting, or recombining, fragments of DNA from different sources.

Transgenic organisms contain recombinant DNA

Recombinant DNA can be inserted into a host organism’s chromosomes and that organism will use this foreign DNA as if it were its own. Plants and animals that contain functional recombinant DNA from an organism of a different genus are known as transgenic organisms because they contain foreign DNA. The glowing tobacco plant shown above contains foreign DNA and is the result of a three-step process that produces a transgenic organism.

The first step of the process is to isolate the foreign DNA fragment that will be inserted. The second step is to attach the DNA fragment to a carrier. The third step is the transfer into the host organism. Each of these three steps now will be discussed in greater detail.

Restriction enzymes cleave DNA

To isolate a DNA fragment, small pieces of DNA must be cut from a chromosome. In the example of the glowing tobacco plant, the fragment is a section of firefly DNA that codes for a light-producing enzyme. The discovery in the early 1970s of DNA-cleaving enzymes called restriction enzymes made it possible to cut DNA.

Restriction enzymes are bacterial proteins that have the ability to cut both strands of the DNA molecule at a specific nucleotide sequence. There are hundreds of restriction enzymes; each can cut DNA at a specific point in a specific nucleotide sequence. The resulting DNA fragments are different lengths. Cutting DNA with restriction enzymes is similar to cutting a zipper into pieces by cutting only between certain teeth of the zipper. Note in Figure 13.4 that the same sequence of bases is found on both DNA strands, but in opposite orders. This arrangement is called a palindrome. Palindromes are words or sentences that read the same forward and backward. The words mom and dad are two examples of palindromes.

Some enzymes produce fragments in which the DNA is cut straight across both strands. These are called blunt ends. Other enzymes, such as the enzyme called EcoRI, cut palindromic sequences of DNA by unzipping them for a few nucleotides, as shown in Figure 13.4. When this DNA is cut, double-stranded fragments with single-stranded ends are formed. The single-stranded ends have a tendency to join with other single-stranded ends to become double stranded, so they attract DNA they can join with. For this reason, these ends are called sticky ends. This is the key to recombinant DNA because if the same enzyme is used to cleave DNA from two organisms, such as firefly DNA and bacterial DNA, the two pieces of DNA will have matching sticky ends and will join together at these ends. When the firefly DNA joins with bacterial DNA, recombinant DNA is formed. The MiniLab on the opposite page models the way restriction enzymes work.

Vectors transfer DNA

Loose fragments of DNA do not readily become part of a host organism’s chromosomes, so the fragments are first attached to a carrier that will transport them into the host organism’s cells. A vector is the means by which DNA from another species can be carried into the host cell. In the case of the transgenic tobacco plant, the light-producing firefly DNA had to be inserted into bacterial DNA before it could be placed inside the plant. The bacterial DNA is the vector.

Vectors may be biological or mechanical. Biological vectors include viruses and plasmids. A plasmid, shown in Figure 13.5, is a small ring of DNA found in a bacterial cell. The genes it carries are different from those on the larger bacterial chromosome.

Two mechanical vectors carry foreign DNA into a cell’s nucleus. One, a micropipette, is inserted into a cell; the other is a microscopic metal bullet coated with DNA that is shot into the cell from a gene gun.

Insertion into a vector

As you have learned, if a plasmid and foreign DNA have been cleaved with the same restriction enzyme, the ends of each will match and they will join together, reconnecting the plasmid ring. The foreign DNA is recombined into a plasmid or viral DNA with the help of a second enzyme.

Gene cloning

After the foreign DNA has been inserted into the plasmid, the recombined DNA is transferred into a bacterial cell. The plasmid is capable of replicating separately from the bacterial host and can produce up to 500 copies per bacterial cell. An advantage to using bacterial cells to clone DNA is that they reproduce quickly; therefore, millions of bacteria are produced and each bacterium contains hundreds of recombinant DNA molecules. Clones are genetically identical copies. Each identical recombinant DNA molecule is called a gene clone. Plasmids also can be used to deliver genes to animal or plant cells, which incorporate the recombinant DNA. Each time the host cell divides it copies the recombinant DNA along with its own. The host cell can produce the protein encoded on the recombinant DNA. Scientists can study the function of this protein in cells that don’t normally produce such proteins. Scientists also can produce mutant forms of a protein and determine how that mutation alters the protein’s function within the cell.

Using other vectors, recombinant DNA can be inserted into yeast, plant, and animal cells. Figure 13.6 summarizes the formation and cloning of recombinant DNA in a bacterial host cell.

Cloning of animals

So far, you have read about cloning one gene. For decades, scientists attempted to expand the technique from a gene to an entire animal. The most famous cloned animal is Dolly, the sheep, first cloned in 1997. Since then, various mammals including goats, mice, cattle, and pigs have been cloned. Although their techniques are inefficient, scientists are coming closer to perfecting the process of cloning animals. One of the benefits for humans in cloning animals is that ranchers and dairy farmers could clone particularly productive, healthy animals to increase yields.

Polymerase chain reaction

In order to replicate DNA outside living organisms, a method called polymerase chain reaction (PCR) has been developed. This method uses heat to separate DNA strands from each other. An enzyme isolated from a heat-loving bacterium is used to replicate the DNA when the appropriate nucleotides are added in a PCR machine. The machine repeatedly replicates the DNA, making millions of copies in less than a day. Because the machine uses heat to separate the DNA strands and cycles over and over to replicate the DNA, it is called a thermocycler.

PCR has become one of the most powerful tools for molecular biologists. The technique is essential to the analysis of bacterial, plant, and animal DNA, including human DNA. PCR has helped bring molecular genetics into crime investigations and the diagnosis of infectious diseases such as AIDS. PCR can help doctors identify extremely small amounts of HIV in blood or the lymphatic system.

Sequencing DNA

Another application of genetic engineering is to provide pure DNA for use in determining the sequence or correct order of the DNA bases. This information can allow scientists to identify mutations.

In DNA sequencing, millions of copies of a double-stranded DNA fragment are cloned using PCR. Then, the strands are separated from each other. The single-stranded fragments are placed in four different test tubes, one for each DNA base. Each tube contains four normal nucleotides (A,C,G,T) and an enzyme that can catalyze the synthesis of a complementary strand. One nucleotide in each tube is tagged with a different fluorescent color. The reactions produce complementary strands of varying lengths. These strands are separated according to size by gel electrophoresis, producing a pattern of fluorescent bands in the gel. The bands are visualized using a laser scanner or UV light. How do the DNA fragments separate from each other in the gel? See Figure 13.8 on the next page to find out.

Applications of DNA Technology

Once it became possible to transfer genes from one organism to another, large quantities of hormones and other products could be produced. How is this technology of use to humans? The main areas proposed for recombinant bacteria are in industry, medicine, and agriculture.

Recombinant DNA in industry

Many species of bacteria have been engineered to produce chemical compounds used by humans. Scientists have modified the bacterium E. coli to produce the expensive indigo dye that is used to color denim blue jeans, like those shown in Figure 13.7.

Gel Electrophoresis

The production of cheese, laundry detergents, pulp and paper production, and sewage treatment have all been enhanced by the use of recombinant DNA techniques that increase enzyme activity, stability, and specificity. Research is currently going on to develop high-protein corn with protein levels comparable to beef and to develop a process for making automobile fuel from discarded cornstalks.

Recombinant DNA in medicine

Pharmaceutical companies already are producing molecules made by recombinant DNA to treat human diseases. Recombinant bacteria are used in the production of human growth hormone to treat pituitary dwarfism. Also, the human gene for insulin is inserted into a bacterial plasmid by genetic engineering techniques. Recombinant bacteria produce large quantities of insulin. Human antibodies, hormones, vaccines, enzymes, and various compounds needed for diagnosis and treatment have been made using recombinant DNA. Read about engineered vaccines in Biotechnology at the end of this chapter.

Transgenic animals

Scientists can study diseases and the role specific genes play in an organism by using transgenic animals. Because mice reproduce quickly, they often are used for transgenic studies. Mouse chromosomes also are similar to human chromosomes. In addition, scientists know the locations of many genes on mouse chromosomes. The roundworm Caenorhabditis elegans is another organism with well-understood genetics that is used for transgenic studies. A third animal commonly used for transgenic studies is the fruit fly, Drosophila melanogaster.

On the same farm in Scotland that produced the cloned sheep Dolly, a transgenic sheep was produced that contained the corrected human gene for hemophilia A. Recall from Chapter 12 that people with hemophilia are missing a protein-clotting factor in their blood. This human gene inserted into the sheep chromosomes allows the production of the clotting protein in the sheep’s milk. The protein then can be separated for use by patients with hemophilia. This farm also has produced transgenic sheep which produce a protein that helps lungs inflate and function properly. The protein is given to people with emphysema, a lung disease associated mainly with cigarette smoking.

Recombinant DNA in agriculture

Recombinant DNA technology has been highly utilized in the agricultural and food industries. Crops have been developed that are better tasting, stay fresh longer, and are protected from disease and insect infestations. Corn, broccoli, cotton, and potatoes have been developed to produce Bt toxin from a bacterial gene, which makes them resistant to certain insect pests. Various plants have been made resistant to a herbicide used to rid the fields of unwanted weeds. You can learn more about this herbicide in the Problem-Solving Lab on the previous page. Canola plants have been modified so they make a higher yield of oil. Currently, research is increasing the amounts of various vitamins in certain crops. These plants could be grown and used in developing countries to supplement local diets that are vitamin deficient. Other research includes the development of peanuts and soybeans that do not cause allergic reactions, a problem for a significant number of people. Figure 13.9 shows a graph of production acreage for the most common genetically modified crops.

The Human Genome

Mapping and Sequencing the Human Genome

In 1990, scientists in the United States organized the Human Genome Project (HGP). It is an international effort to completely map and sequence the human genome, the approximately 35 000– 40 000 genes on the 46 human chromosomes. In February of 2001, the HGP published its working draft of the 3 billion base pairs of DNA in most human cells. The sequence of chromosomes 21 and 22 was finished by May 2000. The MiniLab on the next page gives you an idea of the size of the human genome.

Linkage maps

The locations of thousands of the total number of genes have been mapped to particular chromosomes, and half of the genome has been completely sequenced. However, scientists still don’t know the exact locations of all the genes on the chromosomes.

The genetic map that shows the relative locations of genes on a chromosome is called a linkage map. The historical method used to assign genes to a particular human chromosome was to study linkage data from human pedigrees. Recall from your study of meiosis that crossing over occurs during **prophase** I. As a result of crossing over, gametes and, thus, offspring can have a combination of alleles not found in either parent.

The frequency with which these alleles occur together is a measure of the distance between the genes. Genes that cross over frequently must be farther apart than genes that rarely cross over. Recall from Chapter 10 that the percentage of crossed-over traits appearing in offspring can be used to determine the relative position of genes on the chromosome, and thus, to create a linkage map.

Because humans have only a few offspring compared with the larger numbers of offspring in some other species, and because a human generation time is so long, mapping by linkage data is extremely inefficient. Biotechnology now has provided scientists with new methods of mapping genes. Using polymerase chain reaction (PCR), millions of copies of DNA fragments are cloned in a matter of a few hours. These fragments contain genetic markers that are spread throughout the genome. A genetic marker is a segment of DNA with an identifiable physical location on a chromosome and whose inheritance can be followed. A marker can be a gene, or it can be some section of DNA with no known function. Because DNA segments that are near each other on a chromosome tend to be inherited together, markers are often used as indirect ways of tracking the inheritance pattern of a gene that has not yet been identified, but whose approximate location is known.

Sequencing the human genome

The difficult job of sequencing the human genome is begun by cleaving samples of DNA into fragments using restriction enzymes, as described earlier in this chapter. Then, each individual fragment is cloned and sequenced. The cloned fragments are aligned in the proper order by overlapping matching sequences, thus determining the sequence of a longer fragment. Automated machines can perform this work, greatly increasing the speed of map development.

Applications of the Human Genome Project

As chromosome maps are made, how can they be used? Improved techniques for prenatal diagnosis of human disorders, use of gene therapy, and development of new methods of crime detection are areas currently being researched.

Diagnosis of genetic disorders

One of the most important benefits of the HGP has been the diagnosis of genetic disorders. The DNA of people with and without a genetic disorder is compared to find differences that are associated with the disorder. Once it is clearly understood where a gene is located (see Figure 13.10) and that a mutation in the gene causes the disorder, a diagnosis can be made for an individual, even before birth. Cells are obtained from the fluid surrounding the fetus. DNA is isolated and PCR is used to analyze the area where the mutation is found. If the gene is normal, the PCR product will be a standard size—a deviation means a mutation is present. For some diagnostic tests, the DNA must be analyzed using gel electrophoresis only. This is usually when the disease-causing mutation alters a restriction enzyme-cutting site, producing DNA fragments of different sizes than normal. Thus, when DNA from fetal cells is examined and found to have the mutation associated with the disorder, the fetus will develop the disorder.

Gene therapy

Individuals who inherit a serious genetic disorder may now have hope— gene therapy. Gene therapy is the insertion of normal genes into human cells to correct genetic disorders. This technology is still experimental, but ongoing trials involving over 3000 patients are attempts to treat genetic and acquired diseases. Trials that treat SCID (severe combined immunodeficiency syndrome) have been the most successful. In this disorder, a person’s immune system is shut down and even slight colds can be life-threatening. In gene therapy for this disorder, the cells of the immune system are removed from the patient’s bone marrow, and the functional gene is added to them. The modified cells are then injected back into the patient. Figure 13.11 shows this process.

Other trials involve gene therapy for cystic fibrosis, sickle-cell anemia, hemophilia, and other genetic disorders. Research is also going on to use gene therapy to treat cancer, heart disease, and AIDS. It is hoped that in the next decade DNA technology that uses gene therapy will be developed to treat many different disorders.

DNA fingerprinting

Law-enforcement workers use unique fingerprint patterns to determine whether suspects have been at a crime scene. In the past ten years, biotechnologists have developed a method that determines DNA fingerprints. DNA fingerprinting can be used to convict or acquit individuals of criminal offenses because every person is genetically unique.

Chromosomes consist of genes that are separated by segments of noncoding DNA, DNA that doesn’t code for proteins. The genes follow fairly standard patterns from person to person, but the noncoding segments produce distinct combinations of patterns unique to each individual. In fact, DNA patterns can be used like fingerprints to identify the person (or other organism) from whom they came. DNA fingerprinting works because no two individuals (except identical twins) have the same DNA sequences, and because all cells (except gametes) of an individual have the same DNA.

In a forensic application of DNA fingerprinting, a small DNA sample is obtained from a suspect and from blood, hair, skin, or semen found at the crime scene. The DNA, which includes the unique noncoding segments, is cut into fragments with restriction enzymes. The fragments are separated by gel electrophoresis, then further analyzed. If the samples match, the suspect most likely is guilty.

DNA technology has been used to clone DNA from many sources. Geneticists are using PCR to clone DNA from mummies and analyze it in order to better understand ancient life. Abraham Lincoln’s DNA has been taken from the tips of a lock of his hair and studied for evidence of a possible genetic disorder. The DNA from fossils has been analyzed and used to compare extinct species with living species, or even two extinct species with each other. The uses of DNA technology are unlimited.

Genetics

Genetics is the study of inheritance. The physical traits, or phenotype, of an individual are encoded in small segments of chromosomes called genes. Not all genes are expressed as a phenotype. Therefore, the genotype, the traits encoded in the genes, may be different from the expressed phenotype.

Simple Mendelian Inheritance

A trait is dominant if only one allele of a gene is needed for that trait to be expressed. If two alleles are needed for expression, the trait is said to be recessive. In pea plants, the allele for purple flowers is dominant, and the allele for white flowers is recessive. Any plant with PP or Pp alleles will have purple flowers. Any plant with pp alleles will have white flowers.

Meiosis

Meiosis produces gametes that contain only one copy of each chromosome instead of two. Some stages of meiosis are similar to those of mitosis, but in meiosis, homologous chromosomes come together as tetrads to exchange genes during a process called crossing over. Meiosis also provides a mechanism for re-sorting the genetic information carried by cells. Both crossing over and the re-sorting of genes during meiosis produce genetic variability, which can give offspring a survival advantage if the environment changes.

Producing Physical Traits

Deoxyribonucleic acid (DNA) is a double-stranded molecule made up of a sequence of paired nucleotides that encode each gene on a chromosome. There are four nitrogenous bases in DNA: A, T, C, and G. Because of their molecular shape, A can pair only with T, and C can pair only with G. This precise pairing allows the DNA molecule to copy itself in a process called DNA replication.

Complex Inheritance Patterns

An incomplete dominance pattern of inheritance produces an intermediate phenotype in the heterozygote. In codominant inheritance, the heterozygote expresses both alleles. Some traits, such as human blood types, are governed by multiple alleles, although any individual can carry only two of those alleles.

The X chromosome, one of two sex chromosomes, carries many genes, including the genes for hemophilia and color blindness. Most X-linked disorders appear in males because they inherit only one X chromosome. In females, a normal allele on one X chromosome can mask the expression of a recessive allele on the other X chromosome.

Finally, some traits, such as skin color, are polygenic—governed by several genes.

Recombinant DNA Technology

To make recombinant DNA, a small segment of DNA containing a desired gene is inserted into a bacterial plasmid, a small ring of DNA. The plasmid acts as a vector to carry the DNA segment into a host bacterial cell. Every time the bacterium reproduces, the plasmid containing the inserted DNA is duplicated, producing copies of the recombinant DNA along with the host chromosome.

Because these new DNA segments are identical to the original, they are called clones. The host cell produces large quantities of the protein encoded by the recombinant DNA it contains.

Change through Time

The History of Life

The Record of Life

Early History of Earth

What was early Earth like? Some scientists suggest that it was probably very hot. The energy from colliding meteorites could have heated its surface, while both the compression of minerals and the decay of radioactive materials heated its interior. Volcanoes might have frequently spewed lava and gases, relieving some of the pressure in Earth’s hot interior. These gases helped form Earth’s early atmosphere. Although it probably contained no free oxygen, water vapor and other gases, such as carbon dioxide and nitrogen, most likely were present. If ancient Earth’s atmosphere was like this, you would not have survived in it.

About 4.4 billion years ago, Earth might have cooled enough for the water in its atmosphere to condense. This might have led to millions of years of rainstorms with lightning—enough rain to fill depressions that became Earth’s oceans. Some scientists propose that life originated in Earth’s oceans between 3.9 and 3.4 billion years ago.

History in Rocks

Can scientists be sure that Earth formed in this way? No, they cannot. There is no direct evidence of the earliest years of Earth’s history. The physical processes of Earth constantly destroy and form rocks. The oldest rocks that have been found on Earth formed about 3.9 billion years ago. Although rocks cannot provide information about Earth’s infancy, they are an important source of information about the diversity of life that has existed on the planet.

Fossils—Clues to the past

If you’ve ever visited a zoo or toured a botanical garden, you’ve seen evidence of the diversity of life. But the millions of species living today are probably only a small fraction of all the species that ever existed. About 95 percent of the species that have existed are extinct—they no longer live on Earth. Among other techniques, scientists study fossils to learn about ancient species. A fossil is evidence of an organism that lived long ago.

Because fossils can form in many different ways, there are many types of fossils, as you can see in Table 14.1. Use the MiniLab on the next page to observe some marine fossils under your microscope.

Paleontologists—Detectives to the past

The study of fossils is a lot like solving a mystery. Paleontologists, scientists who study ancient life, are like detectives who use fossils to understand events that happened long ago. They use fossils to determine the kinds of organisms that lived during the past and sometimes to learn about their behavior. For example, fossil bones and teeth can indicate the size of animals, how they moved, and what they ate.

Paleontologists also study fossils to gain knowledge about ancient climate and geography. For example, when scientists find a fossil like the one in Figure 14.1, which resembles a present-day plant that lives in a mild climate, they may reason that the ancient environment was also mild.

By studying the condition, position, and location of rocks and fossils, geologists and paleontologists can make deductions about the geography of past environments.

Fossil formation

For fossils to form, organisms usually have to be buried in mud, sand, or clay soon after they die. These particles are compressed over time and harden into a type of rock called sedimentary rock. Today, fossils still form at the bottoms of lakes, streams, and oceans.

Most fossils are found in sedimentary rocks. These rocks form at relatively low temperatures and pressures that may prevent damage to the organism. How do these fossils become visible millions of years later?

To answer the question, look at Figure 14.3. Fossils are not usually found in other types of rock because of the ways those rocks form. For example, metamorphic rocks form when heat, pressure, and chemical reactions change other rocks. The conditions under which metamorphic rocks form often destroy any fossils that were in the original sedimentary rock.

Relative dating

Scientists use a variety of methods to determine the age of fossils. One method is a technique called relative dating. To understand relative dating, imagine yourself stacking newspapers at home. As each day’s newspaper is added to the stack, the stack becomes taller. If the stack is left undisturbed, the newspapers at the bottom are older than ones at the top.

The relative dating of rock layers uses the same principle. In Figure 14.2, you see fossils in different layers of rock. If the rock layers have not been disturbed, the layers at the surface must be younger than the deeper layers. The fossils in the top layer must also be younger than those in deeper layers. Using this principle, scientists can determine relative age and the order of appearance of the species that are preserved as fossils in the layers.

Radiometric dating

You cannot determine the actual age in years of a fossil or rock by using relative dating techniques. To find the specific ages of rocks, scientists use radiometric dating techniques utilizing the radioactive isotopes in rocks. Most fossils and sedimentary rocks cannot be directly radiometrically dated. Most dates are for volcanic or other igneous rocks, or metamorphic rocks that are closely associated with the sedimentary rocks.

Recall that radioactive isotopes are atoms with unstable nuclei that break down, or decay, over time, giving off radiation. A radioactive isotope forms a new isotope after it decays. The rate at which a radioactive isotope decays is related to the half-life of the isotope. The half-life is the length of time needed for half of the atoms of the isotope to decay.

Scientists try to determine the approximate ages of rocks by comparing the amount of a radioactive iso- tope and the new isotope into which it decays. For example, suppose that when a rock forms it contains a radioactive isotope that decays to half its original amount in one million years. Today, if the rock contains equal amounts of the original radioactive isotope and the new isotope into which it decays, then the rock must be about 1 million years old.

Scientists use potassium-40, a radio-active isotope that decays to argon-40, to date rocks containing potassium-bearing minerals. Based on chemical analysis, chemists have determined that potassium-40 decays to half its original amount in 1.3 billion years. Scientists use carbon-14 to date fossils less than 50 000 years old. Again, based on chemical analysis, they know that carbon-14 decays to half its original amount in 5730 years.

A Trip through Geologic Time

By examining sequences containing sedimentary rock and fossils and dating some of the igneous or metamorphic rocks that are found in the sequences, scientists have put together a chronology, or calendar, of Earth’s history. This chronology, called the geologic time scale, is based on evidence from Earth’s rocks and fossils.

The geologic time scale

Rather than being based on months or even years, the geologic time scale is divided into four large sections that you see in Figure 14.4 —the Precambrian, the Paleozoic Era, the Mesozoic Era, and the Cenozoic Era. An era is a large division in the scale and represents a very long period of time. Each era is subdivided into periods.

The divisions in the geologic time scale are distinguished by the organisms that lived during that time interval. The fossil record indicates that there were several episodes of mass extinction that fall between time divisions. A mass extinction is an event that occurs when many organisms disappear from the fossil record almost at once.

The geologic time scale begins with the formation of Earth about 4.6 billion years ago. To understand the large size of this number, try the MiniLab on the next page, and also try scaling down the history of Earth into a familiar, but hypothetical, calendar year.

Life during the Precambrian

In your hypothetical calendar year, the first day of January becomes the date on which Earth formed. The oldest fossils are found in Precambrian rocks that are about 3.4 billion years old— near the end of March on the hypothetical calendar. Scientists found these fossils, which are shown in Figure 14.5, in rocks found in the deserts of western Australia. They have found more examples of similar types of fossils on other continents. The fossils resemble the forms of modern species of photosynthetic cyanobacteria. You will read more about cyanobacteria in a later chapter.

Scientists have also found dome-shaped structures called stromatolites in Australia and on other continents. Stromatolites still form today in Australia from mats of cyanobacteria, Figure 14.6. Thus, the stromatolites are evidence of the existence of photo- synthetic organisms on Earth during the **Precambrian**.

The **Precambrian** accounts for about 87 percent of Earth’s history— until about the middle of October in the hypothetical calendar year. At the beginning of the **Precambrian**, unicellular prokaryotes—cells that do not have a membrane-bound nucleus— appear to have been the only life forms on Earth. About 2.1 billion years ago, the fossil record shows that more complex eukaryotic organisms, living things with membrane-bound nuclei in their cells, appeared. By the end of the **Precambrian**, about 543 million years ago, multicellular eukaryotes, such as sponges and jelly- fishes, diversified and filled the oceans.

Diversity during the Paleozoic

In the **Paleozoic Era**, which lasted until 248 million years ago, many more types of animals and plants were present on Earth, and some were preserved in the fossil record. The earliest part of the **Paleozoic Era** is called the **Cambrian Period**. Paleontologists often refer to a “Cambrian explosion” of life because the fossil record shows an enormous increase in the diversity of life forms during this time. During the **Cambrian Period**, the oceans teemed with many types of animals, including worms, sea stars, and unusual arthropods, such as the one shown in Figure 14.7.

During the first half of the **Paleozoic**, fishes, the oldest animals with backbones, appeared in Earth’s waters. There is also fossil evidence of ferns and early seed plants existing on land about 400 million years ago. Around the middle of the **Paleozoic**, four-legged animals such as amphibians appeared on Earth. During the last half of the era, the fossil record shows that reptiles appeared and began to flourish on land.

The largest mass extinction recorded in the fossil record marked the end of the **Paleozoic**. About 90 percent of Earth’s marine species and 70 per- cent of the land species disappeared at this time.

Life in the Mesozoic

The **Mesozoic Era** began about 248 million years ago, which would be about December 10 on the hypothetical one-year calendar. Many changes, in both Earth’s organisms and its geology, occurred over the span of this era.

The **Mesozoic Era** is divided into three periods. Fossils from the **Triassic Period**, the oldest period, show that mammals appeared on Earth at this time. These fossils of mammals indicate that early mammals were small and mouselike. They probably scurried around in the shadows of huge fern forests, trying to avoid dinosaurs, reptiles that also appeared during this time.

The middle of the Mesozoic, called the **Jurassic Period**, began about 206 million years ago, or mid- December on the hypothetical calendar.

Recent fossil discoveries support the idea that modern birds evolved from one of the groups of dinosaurs toward the end of this period.

For example, in Figure 14.8A, you see the fossil of Archaeopteryx, a small bird discovered in Germany. The fossil reveals that Archaeopteryx had feathers, a birdlike feature. You also see a present-day bird, the hoatzin, in Figure 14.8B. This bird has a reptilian feature, claws on its wings, for its first few weeks of life. It also flies poorly, as the earliest birds probably did. Scientists suggest that such evidence supports the idea that modern birds evolved from dinosaurs.

A mass extinction

The last period in the Mesozoic, the **Cretaceous**, began about 144 million years ago. During this period, many new types of mammals appeared and flowering plants flourished on Earth. The mass extinction of the dinosaurs marked the end of the **Cretaceous Period** about 65 million years ago. Scientists estimate that not only dinosaurs, but more than two-thirds of all living species at the time became extinct. Some scientists propose that a large meteorite collision caused this mass extinction. Such a collision could have filled the atmosphere with thick, possibly toxic dust that, in turn, changed the climate to one in which many species could no longer survive. Based on geological evidence of a large crater of Cretaceous age in the waters off eastern Mexico, scientists theorize that this was the impact site.

Changes during the Mesozoic

Geological events during the **Mesozoic** changed the places in which species lived and affected their distribution on Earth. **The theory of continental drift**, which is illustrated in Figure 14.9, suggests that Earth’s continents have moved during Earth’s history and are still moving today at a rate of about six centimeters per year. This is about the same rate at which your hair grows. Early in the **Mesozoic**, the continents were merged into one large landmass. During the era, this supercontinent broke up and the pieces drifted apart.

The theory that explains how the continents move is called **plate tectonics**. According to this idea, Earth’s surface consists of several rigid plates that drift on top of a plastic (capable of flow), partially molten layer. These plates are continually moving—spreading apart, sliding by, or pushing against each other. The movements affect organisms. For example, after a long time, the descendants of organisms living on plates that are moving apart may be living in areas with very different climates.

The Cenozoic Era

The **Cenozoic** began about 65 million years ago—around December 26 on the hypothetical calendar of Earth’s history. It is the era in which you now live. Mammals began to flourish during the early part of this era. Among the mammals that appeared was a group of animals to which you belong, the primates. Primates first appeared approximately more than 65 million years ago and have diversified greatly. The modern human species appeared perhaps as recently as 200 000 years ago. On the hypothetical calendar of Earth’s history, 200 000 years ago is late in the evening of December 31.

The Origin of Life

Origins: The Early Ideas

In the past, the ideas that decaying meat produced maggots, mud produced fishes, and grain produced mice were reasonable explanations for what people observed occurring in their environment. After all, they saw maggots appear on meat and young mice appear in sacks of grain. Such observations led people to believe in spontaneous generation—the idea that nonliving material can produce life.

Spontaneous generation is disproved

In 1668, an Italian physician, Francesco Redi, disproved a commonly held belief at the time—the idea that decaying meat produced maggots, which are immature flies. You can follow the steps of Redi’s experiment in Figure 14.10. Redi’s well-designed, controlled experiment successfully convinced many scientists that maggots, and probably most large organisms, did not arise by spontaneous generation.

However, during Redi’s time, scientists began to use the latest tool in biology—the microscope. With the microscope, they saw that microorganisms live everywhere. Although Redi had disproved the spontaneous generation of large organisms, many scientists thought that microorganisms were so numerous and widespread that they must arise spontaneously—probably from a vital force in the air.

Pasteur’s experiments

Disproving the existence of a vital force in air proved difficult. Finally, in the mid-1800s, Louis Pasteur designed an experiment that disproved the spontaneous generation of microorganisms. Pasteur set up an experiment in which air, but no microorganisms, was allowed to contact a broth that contained nutrients. You can see how Pasteur carried out his experiment in Figure 14.11.

Pasteur’s experiment showed that microorganisms do not simply arise in broth, even in the presence of air. From that time on, biogenesis, the idea that living organisms come only from other living organisms, became a cornerstone of biology.

Origins:

The Modern Ideas

Biologists have accepted the concept of biogenesis for more than 100 years. However, biogenesis does not answer the question: How did life begin on Earth? No one has yet proven scientifically how life on Earth began. However, scientists have developed theories about the origin of life on Earth from testing scientific hypotheses about conditions on early Earth. The Biology and Society at the end of this chapter summarizes some important viewpoints about the origin of life on Earth.

Simple organic molecules formed

Scientists hypothesize that two developments must have preceded the appearance of life on Earth. First, simple organic molecules, or molecules that contain carbon, must have formed. Then these molecules must have become organized into complex organic molecules such as proteins, carbohydrates, and nucleic acids that are essential to life.

Remember that Earth’s early atmosphere probably contained no free oxygen. Instead, the atmosphere was probably composed of water vapor, carbon dioxide, nitrogen, and perhaps methane and ammonia. Many scientists have tried to explain how these substances could have joined together and formed the simple organic molecules that are found in all organisms today.

In the 1930s, a Russian scientist, Alexander Oparin, hypothesized that life began in the oceans that formed on early Earth. He suggested that energy from the sun, lightning, and Earth’s heat triggered chemical reactions to produce small organic molecules from the substances present in the atmosphere. Then, rain probably washed the molecules into the oceans to form what is often called a primordial soup.

In 1953, two American scientists, Stanley Miller and Harold Urey, tested Oparin’s hypothesis by simulating the conditions of early Earth in the laboratory. In an experiment similar to the one shown in Figure 14.12, Miller and Urey mixed water vapor (steam) with ammonia, methane, and hydrogen gases. They then sent an electric current that simulated lightning through the mixture. Then, they cooled the mixture of gases, produced a liquid that simulated rain, and collected the liquid in a flask. After a week, they analyzed the chemicals in the flask and found several kinds of amino acids, sugars, and other small organic molecules, providing evidence that supported Oparin’s hypothesis.

The formation of protocells

The next step in the origin of life, as proposed by some scientists, was the formation of complex organic com- pounds. In the 1950s, various experiments were performed and showed that if the amino acids are heated without oxygen, they link and form complex molecules called proteins. A similar process produces ATP and nucleic acids from small molecules. These experiments convinced many scientists that complex organic molecules might have originated in pools of water where small molecules had concentrated and been warmed.

How did these complex chemicals combine to form the first cells? The work of American biochemist Sidney Fox in 1992 showed how the first cells may have occurred. As you can see in Figure 14.13, Fox produced protocells by heating solutions of amino acids. A protocell is a large, ordered structure, enclosed by a membrane, that carries out some life activities, such as growth and division.

The Evolution of Cells

Fossils indicate that by about

3.4 billion years ago, photosynthetic prokaryotic cells existed on Earth. But these were probably not the earliest cells. What were the earliest cells like, and how did they evolve?

The first true cells

The first forms of life may have been prokaryotic forms that evolved from a protocell. Because Earth’s atmosphere lacked oxygen, scientists have proposed that these organisms were most likely anaerobic. For food, the first prokaryotes probably used some of the organic molecules that were abundant in Earth’s early oceans. Because they obtained food rather than making it themselves, they would have been heterotrophs.

Over time, these heterotrophs would have used up the food supply. However, organisms that could make food had probably evolved by the time the food was gone. These first autotrophs were probably similar to present-day archaebacteria.

Archaebacteria are prokaryotic and live in harsh environments, such as deep-sea vents and hot springs like the one shown in Figure 14.14. Some early autotrophs may have made glucose by chemosynthesis rather than by photosynthesis, which requires light-trapping pigments. These autotrophs released the energy of inorganic compounds, such as sulfur compounds, in their environment to make their food.

Photosynthesizing prokaryotes

Eventually, photosynthesizing prokaryotes capable of releasing oxygen from water evolve. Recall that the process of photosynthesis produces oxygen. As the first photosynthetic organisms increased in number, the concentration of oxygen in Earth’s atmosphere began to increase. Organisms that could respire aerobically would have evolved and thrived. In fact, the fossil record indicates that there was a large increase in the diversity of prokaryotic life about

2.8 billion years ago.

The presence of oxygen in Earth’s atmosphere probably affected life on Earth in another important way. The sun’s rays would have converted much of the oxygen into ozone molecules that would then have formed a layer that contained more ozone than the rest of the atmosphere. The ozone layer, that now exists 10 to 15 miles (16–24 km) above Earth’s surface, probably shielded organisms from the harmful effects of ultraviolet radiation and enabled the evolution of more complex organisms, the eukaryotes.

The endosymbiont theory

Complex eukaryotic cells probably evolved from prokaryotic cells. Use the Problem-Solving Lab on this page to determine how long the event might have taken. The **endosymbiont theory**, proposed by American biologist Lynn Margulis in the 1960s, explains how eukaryotic cells may have arisen.

The **endosymbiont theory** as shown in Figure 14.15, proposes that eukaryotes evolved through a symbiotic relationship between ancient prokaryotes. Margulis based her hypothesis on observations and experimental evidence of present-day unicellular organisms. For example, some bacteria that are similar to cyanobacteria and chloroplasts resemble each other in size and in the ability to photosynthesize. Likewise, mitochondria and some bacteria look similar. Experimental evidence revealed that both chloroplasts and mitochondria contain DNA that is similar to the DNA in prokaryotes and unlike the DNA in eukaryotic nuclei.

New evidence from scientific research supports this theory and has shown that chloroplasts and mitochondria have their own ribosomes that are similar to the ribosomes in prokaryotes. In addition, both chloroplasts and mitochondria reproduce independently of the cells that contain them. The fact that some modern prokaryotes live in close association with eukaryotes also supports the theory.

Determining a Rock’s Age

The Theory of Evolution

Natural Selection and the Evidence for Evolution

Charles Darwin and Natural Selection

The modern theory of evolution is the fundamental concept in biology. Recall that evolution is the change in populations over time. Learning the principles of evolution makes it easier to understand modern biology. One place to start is by learning about the ideas of English scientist Charles Darwin (1809–1882)—ideas supported by fossil evidence.

Fossils shape ideas about evolution

Biologists have used fossils in their work since the eighteenth century. In fact, fossil evidence formed the basis of early evolutionary concepts. Scientists wondered how fossils formed, why many fossil species were extinct, and what kinds of relationships might exist between the extinct and the modern species.

Before geologists provided evidence indicating that Earth was much older than many people had originally thought, biologists suspected that species change over time, or evolve. Many explanations about how species evolve have been proposed, but the ideas first published by Charles Darwin are the basis of modern evolutionary theory.

Darwin on HMS Beagle

It took Darwin years to develop his theory of evolution. He began in 1831 at age 22 when he took a job as a naturalist on the English ship HMS Beagle, which sailed around the world on a five-year scientific journey.

As the ship’s naturalist, Darwin studied and collected biological and fossil specimens at every port along the route. As you might imagine, these specimens were quite diverse. Studying the specimens made Darwin curious about possible relationships among species. His studies provided the foundation for his theory of evolution by natural selection.

Darwin in the Galápagos

The Galápagos Islands are a group of small islands near the equator, about 1000 km off the west coast of South America. The observations that Darwin made and the specimens that he collected there were especially important to him.

On the Galápagos Islands, Darwin studied many species of animals and plants, Figure 15.1, that are unique to the islands but similar to species elsewhere. These observations led Darwin to consider the possibility that species can change over time. However, after returning to England, he could not at first explain how such changes occur.

Darwin continues his studies

For the next two decades, Darwin worked to refine his explanation for how species change over time. He read, studied, collected specimens, and conducted experiments.

English economist Thomas Malthus had proposed an idea that Darwin modified and used in his explanation. Malthus’s idea was that the human population grows faster than Earth’s food supply. How did this help Darwin? He knew that many species produce large numbers of offspring. He also knew that such species had not overrun Earth. He realized that individuals struggle to compete in changing environmental conditions. There are many kinds of competition, such as competing for food and space, escaping from predators, finding mates, and locating shelter. Only some individuals survive the competition and produce offspring. Which individuals survive?

Darwin gained insight into the mechanism that determined which organisms survive in nature from his pigeon-breeding experiments. Darwin observed that the traits of individuals vary in populations. Variations are then inherited. By breeding pigeons with desirable variations, Darwin produced offspring with these variations. Breeding organisms with specific traits in order to produce offspring with identical traits is called artificial selection. Darwin hypothesized that there was a force in nature that worked like artificial selection.

Darwin explains natural selection

Using his collections and observations, Darwin identified the process of natural selection, the steps of which you can see summarized in Figure 15.2. Natural selection is a mechanism for change in populations. It occurs when organisms with favorable variations survive, reproduce, and pass their variations to the next generation. Organisms without these variations are less likely to survive and reproduce. As a result, each generation consists largely of offspring from parents with these variations that aid survival.

Darwin was not the only one to recognize the significance of natural selection for populations. As a result of his studies on islands near Indonesia in the Pacific Ocean, Alfred Russell Wallace, another British naturalist, reached a similar conclusion. After Wallace wrote Darwin to share his ideas about natural selection, Darwin and Wallace had their similar ideas jointly presented to the scientific community. Soon thereafter, Darwin published the first book about evolution called On the Origin of Species by Means of Natural Selection in 1859. The ideas detailed in Darwin’s book are a basic unifying theme of biology today.

Interpreting evidence after Darwin

Volumes of scientific data have been gathered as evidence for evolution since Darwin’s time. Much of this evidence is subject to interpretation by different scientists. One of the issues is that evolutionary processes are difficult for humans to observe directly. The short scale of human life spans makes it difficult to comprehend evolutionary processes that occur over millions of years. Despite this, much data about the biological world has been gathered from many sources. These data are best explained by evolution. Almost all of today’s biologists accept the theory of evolution by natural selection. The advent of genetics has added yet more data to our understanding of evolution. This means that the change in the gene pool of a population over time can be added to our modern definition of evolution.

Adaptations: Evidence for Evolution

Have you noticed that some plants have thorns and some plants don’t? Have you noticed that some animals have distinctive coloring but others don’t? Have you ever wondered how such variations arose? Recall that an adaptation is any variation that aids an organism’s chances of survival in its environment. Thorns are an adaptation of some plants and distinctive colorings are an adaptation of some animals. Darwin’s theory of evolution explains how adaptations may develop in species.

Structural adaptations arise over time

According to Darwin’s theory, adaptations in species develop over many generations. Learning about adaptations in mole-rats can help you understand how natural selection has affected them. Mole-rats that live underground in darkness are blind. These blind mole-rats have many adaptations that enable them to live successfully underground. Look at Figure 15.3 to see how these modern mole-rat adaptations might have evolved over millions of years from characteristics of their ancestors.

The structural adaptations of common mole-rats include large teeth and claws. These are body parts that help mole-rats survive in their environment by, for example, enabling them to dig better tunnels. Structural adaptations such as the teeth and claws of mole-rats are often used to defend against predators. Some adaptations of other organisms that keep predators from approaching include a rose’s thorns or a porcupine’s quills.

Some other structural adaptations are subtle. Mimicry is a structural adaptation that enables one species to resemble another species. In one form of mimicry, a harmless species has adaptations that result in a physical resemblance to a harmful species. Predators that avoid the harmful species also avoid the similar-looking, harmless species. See if you can tell the difference between a harmless fly and the wasp it mimics when you look at Figures 15.4A and B.

In another form of mimicry, two or more harmful species resemble each other. For example, yellow jacket hornets, honeybees, and many other species of wasps all have harmful stings and similar coloration and behavior. Predators may learn quickly to avoid any organism with their general appearance.

Another subtle adaptation is camouflage, an adaptation that enables species to blend with their surroundings, as shown in Figure 15.4C. Because well-camouflaged organisms are not easily found by predators, they survive to reproduce.

Physiological adaptations can develop rapidly

In general, most structural adaptations develop over millions of years. However, there are some adaptations that evolve much more rapidly. For example, do you know that some of the medicines developed during the twentieth century to fight bacterial diseases are no longer effective? When the antibiotic drug penicillin was discovered about 50 years ago, it was called a wonder drug because it killed many types of disease-causing bacteria and saved many lives. Today, penicillin no longer affects as many species of bacteria because some species have evolved physiological adaptations to prevent being killed by penicillin. Look at Figure 15.5 to see how resistance develops in bacteria.

Physiological adaptations are changes in an organism’s metabolic processes. In addition to species of bacteria, scientists have observed these adaptations in species of insects and weeds that are pests. After years of exposure to specific pesticides, many species of insects and weeds have become resistant to these chemicals that used to kill them.

Other Evidence for Evolution

The development of physiological resistance in species of bacteria, insects, and plants is direct evidence of evolution. However, most of the evidence for evolution is indirect, coming from sources such as fossils and studies of anatomy, embryology, and biochemistry.

Fossils

Fossils are an important source of evolutionary evidence because they provide a record of early life and evolutionary history. For example, paleontologists conclude from fossils that the ancestors of whales were probably land-dwelling, doglike animals.

Although the fossil record provides evidence that evolution occurred, the record is incomplete. Working with an incomplete fossil record is something like trying to put together a jigsaw puzzle with missing pieces. But, after the puzzle is together, even with missing pieces, you will probably still understand the overall picture. It’s the same with fossils. Although paleontologists do not have fossils for all the changes that have occurred, they can still understand the overall picture of how most groups evolved.

Fossils are found throughout the world. As the fossil record becomes more complete, the sequences of evolution become clearer. For example, in Table 15.1 you can see how paleontologists have charted the evolutionary path that led to today’s camel after piecing together fossil skulls, teeth, and limb bones.

Anatomy

Look at the forelimb bones of the animals shown in Figure 15.6. Although the bones of each forelimb are modified for their function, the basic arrangement of the bones in each limb is similar. Evolutionary biologists view such structural similarities as evidence that organisms evolved from a common ancestor. It would be unlikely for so many animals to have similar structures if each species arose separately. Structural features with a common evolutionary origin are called homologous structures. Homologous structures can be similar in arrangement, in function, or in both.

The structural or functional similarity of a body feature doesn’t always mean that two species are closely related. In Figure 15.7, you can compare the wing of a butterfly with the wing of a bird. Bird and butterfly wings are not similar in structure, but they are similar in function. The wings of birds and insects evolved independently of each other in two distantly related groups of ancestors. The body parts of organisms that do not have a common evolutionary origin but are similar in function are called analogous structures.

Although analogous structures don’t shed light on evolutionary relationships, they do provide evidence of evolution. For example, insect and bird wings probably evolved separately when their different ancestors adapted independently to similar ways of life.

Another type of body feature that suggests an evolutionary relationship is a vestigial structure—a body structure in a present-day organism that no longer serves its original purpose, but was probably useful to an ancestor. A structure becomes vestigial when the species no longer needs the feature for its original function, yet it is still inherited as part of the body plan for the species.

Many organisms, including the whale in Figure 15.8, have vestigial structures. The eyes of blind mole-rats and cave fish are vestigial structures because they are no longer used for sight. Two flightless birds— an extinct elephant bird and an African ostrich—have extremely reduced forelimbs. Their ancestors probably foraged on land for food and nested on the ground. As a result, over time, the ancestral birds became quite large and unable to fly, features evident in fossils of the elephant bird and present in the African ostrich.

Embryology

It’s very easy to see the difference between an adult bird and an adult mammal, but can you distinguish between them by looking at their embryos? An embryo is the earliest stage of growth and development of both plants and animals. The embryos of a fish, a reptile, a bird, and a mammal are shown in Figure 15.9. At this stage of development, all the embryos have a tail and pharyngeal pouches. In fish, these pouches develop into the supports for the gills, while in mammals, reptiles, and birds, they develop into parts of ears, jaws, and throat. It is the shared features in the young embryos that suggest evolution from a distant, common ancestor.

Biochemistry

Biochemistry also provides strong evidence for evolution. Nearly all organisms share DNA, ATP, and many enzymes among their biochemical molecules. One enzyme, cytochrome c, occurs in organisms as diverse as bacteria and bison. Biologists compared the differences that exist among species in the amino acid sequence of cytochrome c. Data from these biochemical studies are shown in Table 15.2. The data show the number of amino acid substitutions in the amino acid sequences for the different organisms. Organisms that are biochemically similar have fewer differences in their amino acid sequences. Groups that share more similarities are interpreted as being more closely related or as sharing a closer ancestor. The evolutionary relationships of some of the groups in the table are shown in Figure 15.10. Since Darwin’s time, scientists have constructed evolutionary diagrams that show levels of relationships among species. In the 1970s, some biologists began to use RNA and DNA nucleotide sequences to construct evolutionary diagrams. Today, scientists combine data from fossils, comparative anatomy, embryology, and biochemistry in order to interpret the evolutionary relationships among species.

Mechanisms of Evolution

Population Genetics and Evolution

When Charles Darwin developed his theory of natural selection in the 1800s, he did so without knowing about genes. Since Darwin’s time, scientists have learned a great deal about genes and modified Darwin’s ideas accordingly. At first, genetic information was used to explain the variation among individuals of a population. Then, studies of the complex behavior of genes in populations of plants and animals developed into the field of study called population genetics. The principles of today’s modern theory of evolution are rooted in population genetics and other related fields of study and are expressed in genetic terms.

Populations, not individuals, evolve

Can individuals evolve? That is, can an organism respond to natural selection by acquiring or losing characteristics? Recall that genes determine most of an individual’s features, such as tooth shape or flower color. If an organism has a feature—called a phenotype in genetic terms—that is poorly adapted to its environment, the organism may be unable to survive and reproduce. However, within its lifetime, it cannot evolve a new phenotype by natural selection in response to its environment.

Rather, natural selection acts on the range of phenotypes in a population. Recall that a population consists of all the members of a species that live in an area. Each member has the genes that characterize the traits of the species, and these genes exist as pairs of alleles. Just as all of the individuals make up the population, all of the genes of the population’s individuals make up the population’s genes. Evolution occurs as a population’s genes and their frequencies change over time.

How can a population’s genes change over time? Picture all of the alleles of the population’s genes as being together in a large pool called a gene pool. The percentage of any specific allele in the gene pool is called the allelic frequency. Scientists calculate the allelic frequency of an allele in the same way that a baseball player calculates a batting average. They refer to a population in which the frequency of alleles remains the same over generations as being in genetic equilibrium. In the Connection to Math at the end of the chapter, you can read about the mathematical description of genetic equilibrium. You can study the effect of natural selection on allelic frequencies in the BioLab at the end of the chapter. Look at the population of snapdragons shown in Figure 15.11. A pattern of heredity called incomplete dominance, which you learned about earlier, governs flower color in snapdragons. If you know the flower-color genotypes of the snapdragons in a population, you can calculate the allelic frequency for the flower-color alleles. The population of snapdragons is in genetic equilibrium when the frequency of its alleles for flower color is the same in all its generations.

Changes in genetic equilibrium

A population that is in genetic equilibrium is not evolving. Because allelic frequencies remain the same, phenotypes remain the same, too. Any factor that affects the genes in the gene pool can change allelic frequencies, disrupting a population’s genetic equilibrium, which results in the process of evolution.

You have learned that one mechanism for genetic change is mutation. Environmental factors, such as radiation or chemicals, cause many mutations, but other mutations occur by chance. Of the mutations that affect organisms, many are lethal, and the organisms do not survive. Thus, lethal mutations are quickly eliminated. However, occasionally, a mutation results in a useful variation, and the new gene becomes part of the population’s gene pool by the process of natural selection.

Another mechanism that disrupts a population’s genetic equilibrium is genetic drift—the alteration of allelic frequencies by chance events.

Genetic drift can greatly affect small populations that include the descendants of a small number of organisms. This is because the genes of the original ancestors represent only a small fraction of the gene pool of the entire species and are the only genes available to pass on to offspring. The distinctive forms of life that Darwin found in the Galápagos Islands may have resulted from genetic drift.

Genetic drift has been observed in some small human populations that have become isolated due to reasons such as religious practices and belief systems. For example, in Lancaster County, Pennsylvania, there is an Amish population of about 12 000 people who have a unique lifestyle and marry other members of their community. By chance, at least one of the original 30 Amish settlers in this community carried a recessive allele that results in short arms and legs and extra fingers and toes in offspring, Figure 15.12. Because of the small gene pool, many individuals inherited the recessive allele over time. Today, the frequency of this allele among the Amish is high—1 in 14 rather than 1 in 1000 in the larger population of the United States.

Genetic equilibrium is also disrupted by the movement of individuals in and out of a population. The transport of genes by migrating individuals is called gene flow. When an individual leaves a population, its genes are lost from the gene pool. When individuals enter a population, their genes are added to the pool.

Mutation, genetic drift, and gene flow may significantly affect the evolution of small and isolated gene pools, such as those on islands. However, their effect is often insignificant in larger, less isolated gene pools. Natural selection is usually the most significant factor that causes changes in established gene pools—small or large.

Natural selection acts on variations

As you’ve learned, traits have variation, as shown in the butterflies pictured in Figure 15.13. Try measuring variations in MiniLab 15.2.

Recall that some variations increase or decrease an organism’s chance of survival in an environment. These variations can be inherited and are controlled by alleles. Thus, the allelic frequencies in a population’s gene pool will change over generations due to the natural selection of variations. There are three different types of natural selection that act on variation: stabilizing, directional, and disruptive.

Stabilizing selection is natural selection that favors average individuals in a population, as shown in Figure 15.14. Consider a population of spiders in which average size is a survival advantage. Predators in the area might easily see and capture spiders that are larger than average. However, small spiders may find it difficult to find food. Therefore, in this environment, average-sized spiders are more likely to survive—they have a selective advantage, or are “selected for.”

Directional selection occurs when natural selection favors one of the extreme variations of a trait. For example, imagine a population of woodpeckers pecking holes in trees to feed on the insects living under the bark. Suppose that a species of insect that lives deep in tree tissues invades the trees in a wood-pecker population’s territory. Only woodpeckers with long beaks could feed on that insect. Therefore, the long-beaked woodpeckers in the population would have a selective advantage over woodpeckers with very short or average-sized beaks.

Finally, in disruptive selection, individuals with either extreme of a trait’s variation are selected for. Consider, for example, a population of marine organisms called limpets. The shell color of limpets ranges from white, to tan, to dark brown. As adults, limpets live attached to rocks. On light-colored rocks, white-shelled limpets have an advantage because their bird predators cannot easily see them. On dark-colored rocks, dark-colored limpets have the advantage because they are camouflaged. On the other hand, birds easily see tan-colored limpets on either the light or dark backgrounds. Disruptive selection tends to eliminate the intermediate phenotypes.

Natural selection can significantly alter the genetic equilibrium of a population’s gene pool over time. Significant changes in the gene pool could lead to the evolution of a new species over time.

The Evolution of Species

You’ve just read about how natural processes such as mutation, genetic drift, gene flow, and natural selection can change a population’s gene pool over time. But how do the changes in the makeup of a gene pool result in the evolution of new species? Recall that a species is defined as a group of organisms that look alike and can interbreed to produce fertile offspring in nature. The evolution of new species, a process called speciation, occurs when members of similar populations no longer interbreed to produce fertile offspring within their natural environment.

Physical barriers can prevent interbreeding

In nature, physical barriers can break large populations into smaller ones. Lava from volcanic eruptions can isolate populations. Sea-level changes along continental shelves can create islands. The water that surrounds an island isolates its populations. Geographic isolation occurs whenever a physical barrier divides a population.

A new species can evolve when a population has been geographically isolated. For example, imagine a population of tree frogs living in a rain forest, Figure 15.15. If small populations of tree frogs were geographically isolated, they would no longer be able to interbreed and exchange genes. Over time, each small population might adapt to its environment through natural selection and develop its own gene pool. Eventually, the gene pools of each population might become so different that they could no longer interbreed with the other populations. In this way, natural selection results in new species.

Reproductive isolation can result in speciation

As populations become increasingly distinct, reproductive isolation can arise. Reproductive isolation occurs when formerly interbreeding organisms can no longer mate and produce fertile offspring.

There are different types of reproductive isolation. Two examples are given here. One type occurs when the genetic material of the populations becomes so different that fertilization cannot occur. Some geographically separated populations of salamanders in California have this type of reproductive isolation. Another type of reproductive isolation is behavioral. For example, if one population of tree frogs mates in the fall, and another mates in the summer, these two populations will not mate with each other and are reproductively isolated.

A change in chromosome numbers and speciation

Chromosomes can also play a role in speciation. Many new species of plants and some species of animals have evolved in the same geographic area as a result of polyploidy, illustrated in Figure 15.16. Any individual or species with a multiple of the normal set of chromosomes is known as a polyploid.

Mistakes during mitosis or meiosis can result in polyploid individuals. For example, if chromosomes do not separate properly during the first meiotic division, diploid (2n) gametes can be produced instead of the normal haploid (n) gametes. Polyploidy may result in immediate reproductive isolation. When a polyploid mates with an individual of the normal species, the resulting zygotes may not develop normally because of the difference in chromosome numbers. In other cases, the zygotes develop into adults that probably cannot reproduce. However, polyploids within a population may interbreed and form a separate species. Polyploids can arise from within a species or from hybridization between species. Many flowering plant species and many important crop plants, such as wheat, cotton, and apples, originated by polyploidy.

Speciation rates

Although polyploid speciation takes only one generation, most other mechanisms of speciation do not occur as quickly. What is the usual rate of speciation?

Scientists once argued that evolution occurs at a slow, steady rate, with small, adaptive changes gradually accumulating over time in populations. Gradualism is the idea that species originate through a gradual change of adaptations. Some evidence from the fossil record supports gradualism. For example, fossil evidence shows that sea lilies evolved slowly and steadily over time.

In 1972, Niles Eldredge and Stephen J. Gould proposed a different hypothesis known as **punctuated equilibrium**. This **hypothesis** argues that speciation occurs relatively quickly, in rapid bursts, with long periods of genetic equilibrium in between. According to this **hypothesis**, environmental changes, such as higher temperatures or the introduction of a competitive species, lead to rapid changes in a small population’s gene pool that is reproductively isolated from the main population. Speciation happens quickly—in about 10 000 years or less. Like gradualism, punctuated equilibrium is supported by fossil evidence as shown in Figure 15.17.

Biologists generally agree that both gradualism and punctuated equilibrium can result in speciation, depending on the circumstances. It shouldn’t surprise you to see scientists offer alternative hypotheses to explain observations. The nature of science is such that new evidence or new ideas can modify theories.

Patterns of Evolution

Biologists have observed different patterns of evolution that occur throughout the world in different natural environments. These patterns support the idea that natural selection is an important agent for evolution.

Diversity in new environments

An extraordinary diversity of unique plants and animals live or have lived on the Hawaiian Islands, among them a group of birds called Hawaiian honeycreepers. This group of birds is interesting because, although similar in body size and shape, they differ sharply in color and beak shape.

Different species of honeycreepers evolved to occupy their own niches.

Despite their differences, scientists hypothesize that honeycreepers, as shown in Figure 15.18, evolved from a single ancestral species that lived on the Hawaiian Islands long ago. When an ancestral species evolves into an array of species to fit a number of diverse habitats, the result is called adaptive radiation.

Adaptive radiation in both plants and animals has occurred and continues to occur throughout the world and is common on islands. For example, the many species of finches that Darwin observed on the Galápagos Islands are a typical example of adaptive radiation.

Adaptive radiation is a type of divergent evolution, the pattern of evolution in which species that once were similar to an ancestral species diverge, or become increasingly distinct. Divergent evolution occurs when populations change as they adapt to different environmental conditions, eventually resulting in new species.

Different species can look alike

A pattern of evolution in which distantly related organisms evolve similar traits is called convergent evolution. Convergent evolution occurs when unrelated species occupy similar environments in different parts of the world. Because they share similar environmental pressures, they share similar pressures of natural selection.

For example, in Figure 15.19 you see an organ pipe cactus (family Cactaceae) that grows in the deserts of North and South America and a plant of the family Euphorbiaceae that looks similar and lives in African deserts. Although these plants are unrelated species, their environments are similar. You can see that they both have fleshy bodies and no leaves. That convergent evolution has apparently occurred in unrelated species, is further evidence for natural selection.

Natural Selection and Allelic Frequency

Primate Evolution

Primate Adaptation and Evolution

What is a primate?

Have you ever gone to a zoo and seen monkeys, chimpanzees, gorillas, or baboons? If you have, then you’ve observed some different types of primates. The primates are a group of mammals that includes lemurs, monkeys, apes, and humans. Primates come in a variety of shapes and sizes, but, despite their diversity, they share common traits. Learn more about primates on pages 1068–1069 in the Focus On.

What characteristics account for the complex behaviors of primates? Find out by reading Figure 16.1 on the next page. Primates have rounded heads with flattened faces, unlike most other mammals. Fitting snugly inside the rounded head is a brain that, relative to body size, is the largest brain of any terrestrial mammal. Primate brains are also more complex than those of other animals. The diverse behaviors and social interactions of primates reflect the complexity of their brains.

The majority of primates are arboreal, meaning they live in trees, and have several adaptations that help them survive there. All primates have relatively flexible shoulder and hip joints. These flexible joints are important to some primates for climbing and swinging among branches.

Primate hands and feet are unique among mammals. Their digits, fingers and toes, have nails rather than claws and their joints are flexible. In addition, primates have an opposable thumb— a thumb that can cross the palm to meet the other fingertips. Opposable thumbs enable primates to grasp and cling to objects, such as the branches of trees. Primates can also hold and manipulate tools, as shown in Figure 16.2.

Primates have a highly developed type of vision, called binocular vision. Primate eyes face forward so that they see an object simultaneously from two viewpoints, or through both eyes. This positioning of the eyes enables primates to perceive depth and thus gauge distances. As you might imagine, this type of vision is helpful for an animal jumping from tree to tree. Primates also have color vision that aids depth perception, enhances their ability to detect predators, and helps them find ripe fruits.

Primate Origins

The similarities among the many primates is evidence that primates share an evolutionary history. Scientists use fossil evidence and comparative anatomical, genetic, and biochemical studies of modern primates to propose ideas about how primates are related and how they evolved. Biologists classify primates into two major groups: strepsirrhines and haplorhines, as shown in Figure 16.3.

Primates

Present-day strepsirrhines are small primates that include, among others, the lemurs and aye-ayes. Most strepsirrhines have large eyes and are nocturnal. They live in the tropical forests of Africa and Southeast Asia. The earliest fossils of strepsirrhines are about 50 to 55 million years old.

Some scientists consider fossils of an organism called Purgatorius to be the earliest of primate fossils. Purgatorius, which probably resembled a squirrel, was a strepsirrhinelike animal that lived about 66 million years ago. Although there are no living species of Purgatorius, present-day strepsirrhines, Figure 16.4, are quite similar.

Humanlike primates evolve

The remaining living primates are members of a group called haplorhines. This group consists of tarsiers and the anthropoids, the humanlike primates. Anthropoids include hominoids and Old and New World monkeys, as shown in Figure 16.5. In turn, hominoids include apes and humans.

Many features characterize anthropoids. Anthropoids have more complex brains than strepsirrhines. Anthropoids are also larger and have different skeletal features, such as a more or less upright posture, than strepsirrhines.

What are commonly called “monkeys” are classified as either New World monkeys or Old World monkeys. New World monkeys, which live in the rain forests of South America and Central America, are all arboreal. A long, muscular prehensile tail characterizes many of these primates. They use the tail as a fifth limb, grasping and wrapping it around branches as they move from tree to tree. Among the New World monkeys are tiny marmosets and larger spider monkeys.

Old World monkeys are generally larger than New World monkeys. They include the arboreal monkeys, such as the colobus monkeys and guenons, the terrestrial monkeys, such as baboons, and monkeys, such as macaques, which are equally at home in trees or on the ground. Old World monkeys do not have prehensile tails. They are adapted to many environments that range from the hot, dry savannas of Africa to the cold mountain forests of Japan.

Hominoids are classified as apes or humans. Apes include orangutans, gibbons, chimpanzees, bonobos, and gorillas. Apes lack tails and have different adaptations for arboreal life from those of the strepsirrhines and monkeys. For example, apes have long, muscled forelimbs for climbing in trees, swinging from branches, and knuckle walking, or walking on two legs with support from their hands. Try the MiniLab shown here to investigate an adaptation for tree climbing. Although many apes are arboreal, most also spend time on the ground. Gorillas, the largest of the apes, live in social groups on the ground. Among the apes, social interactions indicate a large brain capacity.

Humans have an even larger brain capacity and walk upright. You will read more about human primates in the next section. Anthropologists have suggested that monkeys, apes, and humans share a common anthropoid ancestor based on their structural and social similarities. Use the Problem- Solving Lab on the next page to explore this idea. The oldest anthropoid fossils are from Africa and Asia and date to about 37 to 40 million years ago.

Anthropoids evolved worldwide

The oldest monkey fossils are of New World monkeys and are 30 to 35 million years old. Although New World monkeys probably share a common anthropoidlike ancestor with the Old World monkeys, they evolved independently of the Old World monkeys because of geographic isolation. In Figure 16.6, you can see the worldwide geographic distribution of monkeys and apes.

Old World monkeys evolved more recently than New World monkeys. Scientists suspect this is true because the oldest fossils of Old World monkeys are only about 20 to 22 million years old. The fossils indicate that the earliest Old World monkeys were arboreal like today’s New World monkeys.

Hominoids evolved in Asia and Africa

According to the fossil record, there was a global cooling when the hominoids evolved in Asia and Africa. Important changes in vegetation, such as the evolution of grass, also occurred. At about the same time, the Old World monkeys became adapted to this climatic cooling. Fossils indicate how the apes adapted and diversified. You can see two examples of the present-day diversity of apes in Figure 16.7.

Remember that hominoids include the apes and humans. By examining the DNA of each of the modern hominoids, scientists have evaluated the probable order in which the different apes and humans evolved. From this type of evaluation, it appears that gibbons were probably the first apes that evolved, followed by the orangutans that are found in southeast Asia. Finally, the African apes, gorillas and chimpanzees, evolved. Morphological and molecular data suggest that chimpanzees share the closest common ancestor with modern humans.

Human Ancestry

Hominids

Some scientists propose that between 5 and 8 million years ago in Africa, a population that was ancestral to chimpanzees and humans diverged into two lines. According to this hypothesis, one line evolved into chimpanzees, and the other line eventually evolved into modern humans. These two lines are collectively called the hominoids—primates that can walk upright on two legs and include gorillas, chimpanzees, bonobos, and humans. There are relatively few fossils to support this hypothesis, but DNA studies of the modern hominoids provide data that support the idea. You can work with some of these data in MiniLab 16.2 on the next page.

Some scientists suggest that the divergence of the population of ancestral hominoids might have occurred in response to environmental changes that forced some ancestral hominoids to leave their treetop environments and move onto the ground to find food. In order to move efficiently on the ground while avoiding predators, it was helpful for the hominoids to be bipedal, meaning able to walk on two legs. Hominids are bipedal primates that include modern humans and their direct ancestors. In addition to increased speed, walking on two legs leaves the arms and hands free for other activities, such as feeding, protecting young, and using tools. Therefore, hominoids with the ability to walk upright probably survived more successfully on the ground.

These individuals then lived to reproduce and pass the characteristics to their offspring. According to this reasoning, the bipedal organisms that evolved might have been the earliest hominids.

Although the fossil record is incomplete, more hominid fossils are found every year. The many fossils that scientists have found reveal much about the anatomy and behavior of early hominids. Fossils of skulls provide scientists with information about the appearance and brain capacity of the early hominid types. Complete the BioLab at the end of the chapter to learn more about the kinds of information scientists gather from skulls of hominids.

Early hominids walked upright

In Figure 16.8, you see a South African anatomist, Raymond Dart, who, in 1924, discovered a skull of a young hominoid with a braincase and facial structure similar to those of an ape.

However, the skull also had an unusual feature for an ape skull—the position of the foramen magnum, the opening in the skull through which the spinal cord passes as it leaves the brain.

In the fossil, the opening was located on the bottom of the skull, as it is in humans but not in apes. Because of this feature, Dart proposed that the organism had walked upright. He classified the organism as a new primate species, Australopithecus africanus, which means “southern ape from Africa.” The skull that Dart found has been dated at between 2.5 and 2.8 million years old.

Since Dart’s discovery, paleoanthropologists, scientists who study human fossils, have recovered many more australopithecine specimens. They describe an australopithecine as an early hominid that lived in Africa and possessed both apelike and humanlike characteristics.

Early hominids:

Apelike and humanlike

Later, in East Africa in 1974, an American paleoanthropologist, Donald Johanson, discovered one of the most complete australopithecine skeletons that he called “Lucy.” Radiometric dating shows that Lucy probably lived about 3.2 million years ago. Johanson proposed that the Lucy skeleton was a new species, Australopithecus afarensis. Other fossils of A. afarensis indicate that this species probably existed between 3 and 4 million years ago.

Although the fossils show that

A. afarensis individuals had apelike shoulders and forelimbs, the structure of the pelvis, as shown in Figure 16.9, indicates that these individuals were bipedal, like humans. On the other hand, the size of the braincase suggests that their brains had a small, apelike volume and not a larger human volume.

You may be wondering what life was like for hominids like Lucy. Because of the combination of apelike and humanlike features, one idea is that A. afarensis and other species of australopithecines might have lived in small family groups, sleeping and eating in trees. But, to travel, they walked upright on the ground. The fossil record indicates that an

A. afarensis individual rarely survived longer than 25 years.

In addition to fossils of A. afarensis and A. africanus, fossils of three or perhaps four other species of australopithecines have been found. These other species, discovered in East Africa and South Africa, are dated from about 2.5 to 4.3 million years old. Three other species of hominids have been found that are similar to australopithecines. These earlier hominids are grouped into the genus Paranthropus because their fossils suggest that they had larger teeth and jaws and sturdier bodies than australopithecines.

The relationships among australopithecines are not entirely clear from the fossil record. However, the genus disappears from the record between 2.0 and 2.5 million years ago. Although australopithecines became extinct, some paleoanthropologists propose that an early population of these hominids might have been ancestral to modern humans.

Identify the differences in fossil hominid species using anatomical evidence.

The Emergence of Modern Humans

Any ideas about the evolution of modern hominids must include how bipedalism and a large brain evolved. Australopithecine fossils provide support for the idea that bipedalism evolved first. But when did a large brain evolve in a hominid species? When did hominids begin to use tools and develop culture?

Early members of the genus

Homo made stone tools

In 1964, anthropologists Louis and Mary Leakey, Figure 16.10, described skull portions belonging to another type of hominid in Tanzania, Africa. This skull was more humanlike than those of australopithecines. In particular, the braincase was larger and the teeth and jaws were smaller, more like those of modern humans. Because of the skull’s human similarities, the Leakeys classified the hominid with modern humans in the genus Homo. Because stone tools were found near the fossil skull, they named the species Homo habilis, which means “handy human.”

Radiometric dating indicates that H. habilis lived between about 1.5 and 2.5 million years ago. It is the earliest known hominid to make and use stone tools. These tools suggest that H. habilis might have been a scavenger who used the stone tools to cut meat from carcasses of animals that had been killed by other animals. You can see a H. habilis skull in Figure 16.11 on the next page.

Hunting and using fire

Some anthropologists propose that a H. habilis population or another species, Homo ergaster, gave rise to a new species about 1.5–1.8 million years ago. This new hominid species was called Homo erectus, which means “upright human.” H. erectus had a larger brain and a more humanlike face than H. habilis. However, it had prominent browridges and a lower jaw without a chin, as shown in Figure 16.12, which are apelike characteristics.

Some scientists interpret the stone tools called hand axes that they find at some H. erectus excavation sites as an indication that H. erectus hunted. In caves at these sites, they have also found hearths with charred bones. This evidence suggests that these hominids used fire and lived in caves. The distribution of fossils indicates that H. erectus migrated from Africa about 1 million years ago. Then this hominid spread through Africa and Asia, and possibly migrated into Europe, before becoming extinct between 130 000 and 300 000 years ago. However, some scientists propose that more human-looking hominids might have arisen from H. erectus before it disappeared.

Culture developed in modern humans

Many hypotheses have been suggested to explain how modern humans, Homo sapiens, might have emerged. These hypotheses were formed after studying evidence from fossil bones and teeth, and from studies of certain types of DNA. A description of the most popular hypothesis follows.

The fossil record indicates that the species H. sapiens appeared in Europe, Africa, the Middle East, and Asia about 100 000 to 500 000 years ago. The forms that are thought to precede H. sapiens are placed by most scientists into one of two groups—H. antecessor or H. heidelbergensis \. It is not yet clear which of these species represents the direct ancestor to Homo sapiens.

More fossil evidence and additional research into fossil DNA sequences still are needed. These early forms have skulls that resemble H. erectus or H. ergaster but have less prominent browridges, more bulging foreheads, and smaller teeth. Also, the braincases are larger than H. erectus, with brain volumes of 1000 to 1650 cm3, which is within the modern human range. A well-known Homo species was the Neandertals, illustrated in Figure 16.13.

The Neandertals lived from about

35 000 to 100 000 years ago in Europe, Asia, and the Middle East. Fossils reveal that Neandertals had thick bones and large faces with prominent noses. The brains of Neandertals were at least as large as those of modern humans.

The fossil records also indicate that Neandertals lived in caves during the ice ages of their time. In addition, the tools, figurines, flowers, pollen, and other evidence from excavation sites, such as burial grounds, suggest that Neandertals may have had religious views and communicated through spoken language.

What happened to Neandertals?

Could Neandertals have evolved into modern humans? No, the fossil record shows that a more modern type of H. sapiens spread throughout Europe between 35 000 to 40 000 years ago. This type of H. sapiens is called Cro-Magnon. Cro-Magnons were identical to modern humans in height, skull structure, tooth structure, and brain size. Paleoanthropologists suggest that Cro-Magnons were toolmakers and artists, as shown in Figure 16.14. Cro-Magnons probably also used language, as their skulls contain a bulge that corresponds to the area of the brain that is involved in speech in modern humans.

Did Neandertals evolve into Cro-Magnons? Current genetic and archaeological evidence indicates that this is not the case. Current dates for hominid fossils suggest that modern H. sapiens appeared in both South Africa and the Middle East about 100 000 years ago, which was about the same time the Neandertals appeared. In addition, genetic evidence supports the idea of an African origin of modern H. sapiens, perhaps as early as 200 000 years ago. This idea suggests that the African H. sapiens migrated to Europe and Asia.

Most fossil evidence supports the idea that Neandertals were most likely a sister species of H. sapiens, and not an ancestral branch of modern humans. Look at Figure 16.15 to see one possible evolutionary path to modern humans.

Fossil evidence shows that humans have not changed much anatomically over the last 200 000 years. Humans probably first established themselves in Africa, Europe, and Asia. Then evidence shows that they crossed either by sea or using a land bridge into North America. You can read more about the land bridge theory in the Connection to Earth Science at the end of the chapter. By about 10 000 to 8000 years ago, Native Americans had built permanent settlements and were domesticating animals and farming.

Organizing Life’s Diversity

Classification

How Classification Began

Organizing items can help you understand them better and find them more easily. For example, you probably order your clothes drawers and your CD collection. Biologists want to better understand organisms so they organize them. One tool that they use to do this is classification— the grouping of objects or information based on similarities. Taxonomy is the branch of biology that groups and names organisms based on studies of their different characteristics. Biologists who study taxonomy are called taxonomists.

Aristotle’s system

The Greek philosopher Aristotle (384 –322 B.C.) developed the first widely accepted system of biological classification. He classified all the organisms he knew into two groups: plants and animals. He subdivided plants into the three groups, herbs, shrubs, and trees, depending on the size and structure of a plant. He grouped animals according to various characteristics, including their habitat and physical differences.

If you analyze the basis for Aristotle’s groups, you can see it was useful but did not group organisms according to their evolutionary history. According to his system, birds, bats, and flying insects are classified together even though they have little in common besides the ability to fly. As time passed, more organisms were discovered and some did not fit easily into Aristotle’s groups, but many centuries passed before Aristotle’s system was replaced.

Linnaeus’s system of binomial nomenclature

In the late eighteenth century, a Swedish botanist, Carolus Linnaeus (1707–1778), developed a method of grouping organisms that is still used by scientists today. Linnaeus’s system was based on physical and structural similarities of organisms. For example, he might use the similarities in flower parts as a basis for classifying flowering plants, Figure 17.1. As a result, the groupings revealed the relationships of the organisms.

Eventually, some biologists proposed that structural similarities reflect the evolutionary relationships of species. For example, although bats fly like birds, they also have hair and produce milk for their young. Therefore, bats are classified as mammals rather than as birds, reflecting the evolutionary history that bats share with other mammals. This way of organizing organisms is the basis of modern classification systems.

Modern classification systems use a two-word naming system called binomial nomenclature that Linnaeus developed to identify species. In this system, the first word identifies the genus of the organism. A genus consists of a group of similar species. The second word, which sometimes describes a characteristic of the organism, is called the specific epithet. Thus, the scientific name for each species, referred to as the species name, is a combination of the genus name and specific epithet.

For example, the species name of modern humans is Homo sapiens. Modern humans are in the genus Homo, for example. One of their characteristics is intelligence. The Latin word sapiens means “wise.”

Scientific and common names

Latin is the language of scientific names. Taxonomists are required to use Latin because the language is no longer used in conversation and, therefore, does not change. Scientific names should be italicized in print and underlined when handwritten. The first letter of the genus name is uppercase, but the first letter of the specific epithet is lowercase.

Although a scientific name gives information about the relationships of an organism and how it is classified, many organisms have common names just like you and your friends might have nicknames. However, a common name can be misleading. For example, a sea horse is a fish, not a horse. In addition, it is confusing when a species has more than one common name. The bird in Figure 17.2 lives not only in the United States but also in several countries in Europe. It has a different common name in each country. Therefore, if an English scientist uses the bird’s English common name in an article, a Spanish scientist looking for information might not recognize the bird as the same species.

Modern Classification

Expanding on Linnaeus’s work, today’s taxonomists try to identify the underlying evolutionary relationships of organisms and use the information gathered as a basis for classification.

They compare the external and internal structures of organisms, as well as their geographical distribution and genetic makeup to reveal their probable evolutionary relationships. Grouping organisms on the basis of their evolutionary relationships makes it easier to understand biological diversity.

Taxonomy: A framework

Just as similar food items in a supermarket are stacked together, taxonomists group similar organisms, both living and extinct. Classification provides a framework in which to study the relationships among living and extinct species.

For example, biologists study the relationship between birds and dinosaurs within the framework of classification. Are dinosaurs more closely related to birds or to reptiles? The bones of some dinosaurs have large internal spaces like those in birds. In addition, dinosaur skeletons share many other remarkable similarities with birds. Because of such evidence, they suggest that dinosaurs are more closely related to ostriches, which are birds, than to lizards, which are reptiles.

Taxonomy: A useful tool

Classifying organisms can be a useful tool for scientists who work in agriculture, forestry, and medicine.

For example, suppose a child eats berries from a plant in the backyard. The child’s parents would probably rush the child and some of the plant and its berries to the nearest hospital. A scientist working at a poison control center could identify the plant, and the physicians would then know how to treat the child.

Anyone can learn to identify many organisms, even similar ones such as shown in Figure 17.3. The MiniLab on this page will guide you through a way of identifying some organisms using a dichotomous key. Then try the BioLab at the end of this chapter.

Taxonomy and the economy

It often happens that the discovery of new sources of lumber, medicines, and energy results from the work of taxonomists. The characteristics of a familiar species are frequently similar to those found in a new, related species. For example, if a taxonomist knows that a certain species of pine tree contains chemicals that make good disinfectants, it’s possible that another pine species could also contain these useful substances.

How Living Things Are Classified

In any classification system, items are categorized, making them easier to find and discuss. For example, in a newspaper’s classified advertisements, you’ll find a section listing autos for sale. This section frequently subdivides the many ads into two smaller groups—domestic autos and imported autos. In turn, these two groups are subdivided by more specific criteria, such as different car manufacturers and the year and model of the auto. Although biologists group organisms, not cars, they subdivide the groups on the basis of more specific criteria. A group of organisms is called a taxon.

Taxonomic rankings

Organisms are ranked in taxa that range from having very broad characteristics to very specific ones. The broader a taxon, the more general its characteristics, and the more species it contains. You can think of the taxa as fitting together like nested boxes of increasing sizes. You already know about two taxa. The smallest taxon is species. Organisms that look alike and successfully interbreed belong to the same species. The next largest taxon is a genus—a group of similar species that have similar features and are closely related.

It is not always easy to determine the species of an organism. For example, over many years, taxonomists have debated how to classify the red wolf, the coyote, and the gray wolf. Some biologists wanted to classify them as separate species, and others wanted to classify them as a single species. Use the Problem-Solving Lab on this page to explore the evidence for and against classifying these three organisms as separate species.

In Figure 17.4, you can compare the appearance of a lynx, Lynx canadensis, a bobcat, Lynx rufus, and a mountain lion, Felis concolor. The scientific names of the lynx and bobcat tell you that they belong to the same genus, Lynx. All species in the genus Lynx share the characteristic of having a jaw that contains 28 teeth. Mountain lions and other lions, which are similar to bobcats and lynxes, are not classified in the Lynx genus because their jaws contain 30 teeth.

Bobcats, lynxes, lions, and mountain lions belong to the same family called Felidae. Family, the next larger taxon in the biological classification system, consists of a group of similar genera. In addition to domesticated cats, bobcats, lynxes, and lions belong to the family Felidae. All members of the cat family share certain characteristics. They have short faces, small ears, forelimbs with five toes, and hindlimbs with four toes. Most can retract their claws.

The larger taxa

There are five larger taxa following family. An order is a taxon of similar families. A class is a taxon of similar orders. A phylum is a taxon of similar classes. Plant taxonomists use the taxon division instead of phylum. A kingdom is a taxon of similar phyla or divisions. A domain contains one or more kingdoms. The six kingdoms are described in the next section.

The bobcat and lynx that were compared earlier are both classified completely in Figure 17.5. Although the six-kingdom classification system primarily is used in this textbook, several other classification systems exist. Recently, many taxonomists began including a fifth taxa called domains, which include all six kingdoms. You can learn more about domains and other classification systems on pages 1070–1073 in the Focus On.

The Six Kingdoms

How are evolutionary relationships determined?

Evolutionary relationships are determined on the basis of similarities in structure, breeding behavior, geographical distribution, chromosomes, and biochemistry. Because these characteristics provide the clues about how species evolved, they also reveal the probable evolutionary relationships of species.

Structural similarities

Structural similarities among species reveal relationships. For example, the presence of many shared physical structures implies that species are closely related and may have evolved from a common ancestor. For example, because lynxes and bobcats have structures more similar to each other than to members of any other groups, taxonomists suggest that they share a common ancestor. Likewise, plant taxonomists use structural evidence to classify dandelions and sunflowers in the same family, Asteraceae, because they have similar flower and fruit structures.

If you observe an unidentified animal that can retract its claws, you can infer that it belongs to the cat family. You can then assume that the animal has other characteristics in common with cats. Taxonomists observe and compare features among members of different taxa and use this information to infer their evolutionary history.

Breeding behavior

Sometimes, breeding behavior provides important clues to relationships among species. For example, two species of frogs, Hyla versicolor and Hyla chrysoscelis, live in the same area and look similar. During the breeding season, however, there is an obvious difference in their mating behavior. The males of each species make different sounds to attract females, and therefore attract and mate only with members of their own group. Scientists concluded that the frogs were two separate species.

Geographical distribution

The location of species on Earth helps biologists determine their relationships with other species. For example, many different species of finches live on the Galápagos Islands off the coast of South America. Biologists propose that in the past some members of a finchlike bird species that lived in South America reached the Galápagos Islands, where they became isolated. These finches probably spread into different niches on the volcanic islands and evolved over time into many distinct species. The fact that they share a common ancestry is supported by their geographical distribution in addition to their genetic similarities.

Chromosome comparisons

Both the number and structure of chromosomes, as seen during mitosis and meiosis, provide evidence about relationships among species. For example, cauliflower, cabbage, kale, and broccoli look different but have chromosomes that are almost identical in structure. Therefore, biologists propose that these plants are related. Likewise, the similar appearance of chromosomes among chimpanzees, gorillas, and humans suggests a common ancestry.

Biochemistry

Powerful evidence about relationships among species comes from biochemical analyses of organisms. Closely related species have similar DNA sequences and, therefore, similar proteins. In general, the more inherited nucleotide sequences that two species share, the more closely related they are. For example, the DNA sequences in giant pandas and red pandas differ. They differ so much that many scientists suggest that giant pandas are more closely related to bears than to red pandas, such as the one shown in Figure 17.6. Read the Biotechnology feature at the end of this chapter to learn more about how chemical similarities can reveal evolutionary relationships.

Phylogenetic Classification: Models

Species that share a common ancestor also share an evolutionary history. The evolutionary history of a species is called its phylogeny. A classification system that shows the evolutionary history of species is a phylogenetic classification and reveals the evolutionary relationships of species.

Early classification systems did not reflect the phylogenetic relationships among organisms. As scientists learned more about evolutionary relationships, they modified the early classification schemes to reflect the phylogeny of species.

Cladistics

One biological system of classification that is based on phylogeny is cladistics. Scientists who use cladistics assume that as groups of organisms diverge and evolve from a common ancestral group, they retain some unique inherited characteristics that taxonomists call derived traits. Biologists identify a group’s derived traits and use them to make a branching diagram called a cladogram. A cladogram is a model of the phylogeny of a species, and models are important tools for understanding scientific concepts.

Cladograms are similar to the pedigrees, or family trees, you studied in an earlier chapter. Branches on both pedigrees and cladograms show proposed ancestry. In a cladogram, two groups on diverging branches probably share a more recent ancestor than those groups farther away. If two organisms are near each other on a pedigree’s branch, they also share an ancestor. However, an important difference between cladograms and pedigrees is that, whereas pedigrees show the direct ancestry of an organism from two parents, cladograms show a probable evolution of a group of organisms from ancestral groups.

In Figure 17.7, you see the cladogram for modern birds, such as robins. How was the cladogram developed? First, taxonomists identified the derived traits of modern birds—flight feathers, light bones, a wishbone, down feathers, and feathers with shafts. Next, they identified ancestral species that have at least some of these traits. Most biologists agree that the ancestors of birds are a group of dinosaurs called theropods. Some of these theropods are Allosaurus, Archaeopteryx, Velociraptor, Sinornis, and Protarchaeopteryx. Each of these ancestors has a different number of derived traits. Some groups share more derived traits than others.

Finally, taxonomists constructed the robin’s cladogram from this information. They assume that if groups share many derived traits, they share common ancestry. Thus, Archaeopteryx and the robin, which share four derived traits, are on adjacent branches, indicating a recent common ancestor. Use the MiniLab on this page to construct a cladogram for another species.

Another type of model

In this book, you will see cladograms and other types of models that provide information about the phylogenetic relationships among species. One type of model resembles a fan. Unlike a cladogram, a fan-like model may communicate the time organisms became extinct or the relative number of species in a group. A fanlike diagram incorporates fossil information and the knowledge gained from anatomical, embryological, genetic, and cladistic studies.

In Figure 17.8 on the next page, you can see a fanlike model of the six-kingdom classification system. This model includes both Earth’s geologic time scale and the probable evolution of organisms during that time span. In addition, this fanlike diagram helps you to find relationships between modern and extinct species.

Groups of organisms that are closer in the same colored ray share more inherited characteristics.

The Six Kingdoms of Organisms

As you saw in Figure 17.8, the six kingdoms of organisms are archaebacteria, eubacteria, protists, fungi, plants, and animals. In general, differences in cellular structures and methods of obtaining energy are the two main characteristics that distinguish among the members of the six kingdoms. Learn more about the number of species in each kingdom in the Problem-Solving Lab on this page.

Prokaryotes

The prokaryotes, organisms with cells that lack distinct nuclei bounded by a membrane, are microscopic and unicellular. Some are heterotrophs and some are autotrophs. In turn, some prokaryotic autotrophs are chemosynthetic, whereas others are photosynthetic. There are two kingdoms of prokaryotic organisms: Archaebacteria and Eubacteria. The oldest prokaryotic fossils are about3.5 billion years old.

There are several hundred species of known archaebacteria and most of them live in extreme environments such as swamps, deep-ocean hydrothermal vents, and seawater evaporating ponds, like the one in Figure 17.9.

Most of these environments are oxygen-free. The lipids in the cell membranes of archaebacteria, the composition of their cell walls, and the sequence of nucleic acids in their ribosomal RNA differ considerably from those of other prokaryotes. In addition, their genes have a similar structure to those in eukaryotes.

All of the other prokaryotes, about 5000 species of bacteria, are classified in Kingdom Eubacteria. Eubacteria, such as those shown in Figure 17.10, have very strong cell walls and a less complex genetic makeup than found in archaebacteria or eukaryotes. They live in most habitats except the extreme ones inhabited by the archaebacteria. Although some eubacteria cause diseases, such as strep throat and pneumonia, most bacteria are harmless and many are actually helpful.

Protists: A diverse group

Kingdom Protista contains diverse species, as shown in Figure 17.11, that share some characteristics.

A protist is a eukaryote that lacks complex organ systems and lives in moist environments. Fossils of plant-like protists show that protists existed on Earth up to two billion years ago. Although some protists are unicellular, others are multicellular. Some are plantlike autotrophs, some are animal-like heterotrophs, and others are funguslike heterotrophs that produce reproductive structures like those of fungi.

Fungi: Earth’s decomposers

Organisms in Kingdom Fungi are heterotrophs that do not move from place to place. A fungus is either a unicellular or multicellular eukaryote that absorbs nutrients from organic materials in the environment. Fungi first appeared in the fossil record over

400 million years ago. There are more than 50 000 known species of fungi, including the one you see in Figure 17.12.

Plants: Multicellular oxygen producers

All of the organisms in Kingdom Plantae are multicellular, photosynthetic eukaryotes. None moves from place to place. A plant’s cells usually contain chloroplasts and have cell walls composed of cellulose. Plant cells are organized into tissues that, in turn, are organized into organs and organ systems. You can see two of the many diverse types of plants in Figure 17.13.

The oldest plant fossils are more than 400 million years old.

However, some scientists propose that plants existed on Earth’s landmasses much earlier than these fossils indicate. Plants do not fossilize as often as organisms that contain hard structures, such as bones, which more readily fossilize than soft tissues.

There are more than 250 000 known species of plants. Although you may be most familiar with flowering plants, there are many other types of plants, including mosses, ferns, and evergreens.

Animals: Multicellular consumers

Animals are multicellular heterotrophs. Nearly all are able to move from place to place. Animal cells do not have cell walls. Their cells are organized into tissues that, in turn, are organized into organs and complex organ systems. Some organ systems in animals are the nervous, circulatory, and muscular systems, Figure 17.14. Animals first appeared in the fossil record about 600 million years ago.

**Textbook 5: dynamics of life Vol.2**

Chromosomes and Chromosome Number

Each student in your biology class has characteristics passed on to them by their parents. Each characteristic, such as hair color, height, or eye color, is called a trait. The instructions for each trait are located on chromosomes, which are found in the nucleus of cells. The DNA on chromosomes is arranged in segments that control the production of proteins. These DNA segments are called genes. Each chromosome consists of hundreds of genes, each gene playing an important role in determining the characteristics and functions of the cell.

Homologous chromosomes Human body cells have 46 chromosomes. Each parent contributes 23 chromosomes, resulting in 23 pairs of chromosomes. The chromosomes that make up a pair, one chromosome from each parent, are called homologous chromosomes. As shown in Figure 10.1, homologous chromosomes in body cells have the same length and the same centromere position, and they carry genes that control the same inherited traits. For instance, the gene for earlobe type will be located at the same position on both homologous chromosomes. Although these genes each code for earlobe type, they might not code for the exact same type of earlobe.

Haploid and diploid cells In order to maintain the same chromosome number from generation to generation, an organism produces gametes, which are sex cells that have half the number of chromosomes. Although the number of chromosomes varies from one species to another, in humans each gamete contains 23 chromosomes. The symbol n can be used to represent the number of chromosomes in a gamete. A cell with n number of chromosomes is called a haploid cell. Haploid comes from the Greek word haploos, meaning single.

The process by which one haploid gamete combines with another haploid gamete is called fertilization. As a result of fertilization, the cell now will contain a total of 2n chromosomes—n chromosomes from the female parent plus n chromosomes from the male parent. A cell that contains 2n number of chromosomes is called a diploid cell.

Notice that n also describes the number of pairs of chromosomes in an organism. When two human gametes combine, 23 pairs of homologous chromosomes are formed.

Meiosis I

Gametes are formed during a process called meiosis, which is a type of cell division that reduces the number of chromosomes; therefore, it is referred to as a reduction division. Meiosis occurs in the reproductive structures of organisms that reproduce sexually. While mitosis maintains the chromosome number, meiosis reduces the chromosome number by half through the separation of homologous chromosomes. A cell with 2n number of chromosomes will have gametes with n number of chromosomes after meiosis, as illustrated in Figure 10.2. Meiosis involves two consecutive cell divisions called meiosis I and meiosis II.

Interphase Recall that the cell cycle includes **interphase** prior to mitosis. Cells that undergo meiosis also go through **interphase** as part of the cell cycle. Cells in **interphase** carry out various metabolic processes, including the replication of DNA and the synthesis of proteins.

Prophase I As a cell enters **prophase** I, the replicated chromosomes become visible. As in mitosis, the replicated chromosomes consist of two sister chromatids. As the homologous chromosomes condense, they begin to form pairs in a process called synapsis. The homologous chromosomes are held tightly together along their lengths, as illustrated in Figure 10.3. Notice that in Figure 10.4 the purple and green chromosomes have exchanged segments. This exchange occurs during synapsis. Crossing over is a process during which chromosomal segments are exchanged between a pair of homologous chromosomes.

As **prophase** I continues, centrioles move to the cell’s opposite poles. Spindle fibers form and bind to the sister chromatids at the centromere.

Metaphase I In the next phase of meiosis, the pairs of homologous chromosomes line up at the equator of the cell, as illustrated in Figure 10.5.

In meiosis, the spindle fibers attach to the centromere of each homologous chromosome. Recall that during **metaphase** in mitosis, the individual chromosomes, which consist of two sister chromatids, line up at the cell’s equator. During **metaphase** I of meiosis, the homologous chromosomes line up as pairs at the cell’s equator. This is an important distinction between mitosis and meiosis.

Anaphase I During **anaphase** I, the homologous chromosomes separate, which is also illustrated in Figure 10.5. Each member of the pair is guided by spindle fibers and moves toward opposite poles of the cell. The chromosome number is reduced from 2n to n when the homologous chromosomes separate. Recall that in mitosis, the sister chromatids split during **anaphase**. During **anaphase** I of meiosis, however, each homologous chromosome still consists of two sister chromatids.

Telophase I

The homologous chromosomes, consisting of two sister chromatids, reach the cell’s opposite poles. Each pole contains only one member of the original pair of homologous chromosomes. Notice in Figure 10.5 that each chromosome still consists of two sister chromatids joined at the centromere. The sister chromatids might not be identical because crossing over might have occurred during synapsis in **prophase** I.

During **telophase** I, cytokinesis usually occurs, forming a furrow by pinching in animal cells and by forming a cell plate in plant cells. Following cytokinesis, the cells may go into **interphase** again before the second set of divisions. However, the DNA is not replicated again during this **interphase**. In some species, the chromosomes uncoil, the nuclear membrane reappears, and nuclei reform during **telophase** I.

Meiosis II

Meiosis is only halfway completed at the end of meiosis I. During **prophase** II, a second set of phases begins as the spindle apparatus forms and the chromosomes condense. During **metaphase** II, the chromosomes are positioned at the equator by the spindle fibers, as shown in Figure 10.5. During **metaphase** of mitosis, a diploid number of chromosomes line up at the equator. During **metaphase** II of meiosis, however, a haploid number of chromosomes line up at the equator. During **anaphase** II, the sister chromatids are pulled apart at the centromere by the spindle fibers, and the sister chromatids move toward the opposite poles of the cell. The chromosomes reach the poles during **telophase** II, and the nuclear membrane and nuclei reform. At the end of meiosis II, cytokinesis occurs, resulting in four haploid cells, each with n number of chromosomes, as illustrated in Figure 10.5.

Meiosis provides variation Recall that pairs of homologous chromosomes line up at the equator during **prophase** I. How the chromosomes line up at the equator is a random process that results in gametes with different combinations of chromosomes, such as the ones in Figure 10.6.

Depending on how the chromosomes line up at the equator, four gametes with four different combinations of chromosomes can result.

Notice that the first possibility shows which chromosomes were on the same side of the equator and therefore traveled together. Different combinations of chromosomes were lined up on the same side of the equator to produce the gametes in the second possibility. Genetic variation also is produced during crossing over and during fertilization, when gametes randomly combine.

Sexual Reproduction v. Asexual Reproduction

Some organisms reproduce by asexual reproduction, while others reproduce by sexual reproduction. The life cycles of still other organisms might involve both asexual and sexual reproduction. During asexual reproduction, the organism inherits all of its chromosomes from a single parent. Therefore, the new individual is genetically identical to its parent. Bacteria reproduce asexually, whereas most protists reproduce both asexually and sexually, depending on environmental conditions. Most plants and many of the more simple animals can reproduce both asexually and sexually, compared to more advanced animals that reproduce only sexually.

Why do some species reproduce sexually while others reproduce asexually? Recent studies with fruit flies have shown that the rate of accumulation of beneficial mutations is faster when species reproduce sexually than when they reproduce asexually. In other words, when reproduction occurs sexually, the beneficial genes multiply faster over time than they do when reproduction is asexual.

Mendelian Genetics

How Genetics Began

In 1866, Gregor Mendel, an Austrian monk and a plant breeder, published his findings on the method and the mathematics of inheritance in garden pea plants. The passing of traits to the next generation is called inheritance, or heredity. Mendel, shown in Figure 10.7, was successful in sorting out the mystery of inheritance because of the organism he chose for his study—the pea plant. Pea plants are easy to grow and many are true-breeding, meaning that they consistently produce offspring with only one form of a trait.

Pea plants usually reproduce by self-fertilization. A common occurrence in many flowering plants, self-fertilization occurs when a male gamete within a flower combines with a female gamete in the same flower. Mendel also discovered that pea plants could easily be cross- pollinated by hand. Mendel performed cross-pollination by transferring a male gamete from the flower of one pea plant to the female reproductive organ in a flower of another pea plant.

Mendel rigorously followed various traits in the pea plants he bred. He analyzed the results of his experiments and formed hypotheses concerning how the traits were inherited. The study of genetics, which is the science of heredity, began with Mendel, who is regarded as the father of genetics.

The Inheritance of Trait

Mendel noticed that certain varieties of garden pea plants produced specific forms of a trait, generation after generation. For instance, he noticed that some varieties always produced green seeds and others always produced yellow seeds. In order to understand how these traits are inherited, Mendel performed cross pollination by transferring male gametes from the flower of a true-breeding green-seed plant to the female organ of a flower from a true-breeding yellow-seed plant. To prevent self-fertilization, Mendel removed the male organs from the flower of the yellow-seed plant. Mendel called the green-seed plant and the yellow-seed plant the parent generation—also known as the P generation.

F1 and F2 generations When Mendel grew the seeds from the cross between the green-seed and yellow-seed plants, all of the resulting offspring had yellow seeds. The offspring of this P cross are called the first filial (F1) generation. The green-seed trait seemed to have disappeared in the F1 generation, and Mendel decided to investigate whether the trait was no longer present or whether it was hidden, or masked.

Mendel planted the F1 generation of yellow seeds, allowed the plants to grow and self-fertilize, and then examined the seeds from this cross. The results of the second filial (F2) generation—the offspring from the F1 cross—are shown in Figure 10.8. Of the seeds Mendel collected, 6022 were yellow and 2001 were green, which almost is a perfect 3:1 ratio of yellow to green seeds.

Mendel studied seven different traits—seed or pea color, flower color, seed pod color, seed shape or texture, seed pod shape, stem length, and flower position—and found that the F1 generation plants from these crosses also showed a 3:1 ratio.

Genes in pairs Mendel concluded that there must be two forms of the seed trait in the pea plants—yellow-seed and green-seed—and that each was controlled by a factor, which now is called an allele. An allele is defined as an alternative form of a single gene passed from generation to generation. Therefore, the gene for yellow seeds and the gene for green seeds are each different forms of a single gene.

Mendel concluded that the 3:1 ratio observed during his experiments could be explained if the alleles were paired in each of the plants. He called the form of the trait that appeared in the F1 generation dominant and the form of the trait that was masked in the F1 generation recessive. In the cross between yellow-seed plants and green-seed plants, the yellow seed was the dominant form of the trait and the green seed was the recessive form of the trait.

Dominance When he allowed the F1 generation to self-fertilize, Mendel showed that the recessive allele for green seeds had not disappeared but was masked. Mendel concluded that the green-seed form of the trait did not show up in the F1 generation because the yellow-seed form of the trait is dominant and masks the allele for the green-seed form of the trait.

Because the yellow-seed form of the trait is dominant, the allele for the yellow-seed form of the trait is represented by a capital Y. The allele for the green-seed form of the trait is represented by a lowercase y because it is recessive. An organism with two of the same alleles for a particular trait is homozygous for that trait. Homozygous, yellow-seed plants are YY and green-seed plants are yy. An organism with two different alleles for a particular trait is heterozygous for that trait, in this case Yy. When alleles are present in the heterozygous state, the dominant trait will be observed.

Genotype and phenotype

A yellow-seed plant could be homozygous or heterozygous for the trait form. The outward appearance of an organism does not always indicate which pair of alleles is present. The organism’s allele pairs are called its genotype. In the case of plants with yellow seeds, their genotypes could be YY or Yy. The observable characteristic or outward expression of an allele pair is called the phenotype. The phenotype of pea plants with the genotype yy will be green seeds.

Mendel’s law of segregation

Mendel used homozygous yellow-seed and green-seed plants in his P cross. In Figure 10.9, the first drawing shows that each gamete from the yellow-seed plant contains one Y. Recall that the chromosome number is divided in half during meiosis. The resulting gametes contain only one of the pair of seed-color alleles.

The second drawing in Figure 10.9 shows that each gamete from the green-seed plant contains one y allele. **Mendel’s law of segregation** states that the two alleles for each trait separate during meiosis. During fertilization, two alleles for that trait unite.

The third drawing in Figure 10.9 shows the alleles uniting to produce the genotype Yy during fertilization. All resulting F1 generation plants will have the genotype Yy and will have yellow seeds because yellow is dominant to green. These heterozygous organisms are called hybrids.

Monohybrid cross The diagram in Figure 10.10 shows how Mendel continued his experiments by allowing the Yy plants to self-fertilize. A cross such as this one that involves hybrids for a single trait is called a monohybrid cross. The Yy plants produce two types of gametes—male and female—each with either the Y or y allele. The combining of these gametes is a random event. This random fertilization of male and female gametes results in the following genotypes—YY, Yy, Yy, or yy, as shown in Figure 10.10. Notice that the dominant Y allele is written first, whether it came from the male or female gamete. In Mendel’s F1 cross, there are three possible genotypes: YY, Yy, and yy; and the genotypic ratio is 1:2:1. The phenotypic ratio is 3:1—yellow seeds to green seeds.

Dihybrid cross Once Mendel established inheritance patterns of a single trait, he began to examine simultaneous inheritance of two or more traits in the same plant. In garden peas, round seeds (R) are dominant to wrinkled seeds (r), and yellow seeds (Y) are dominant to green seeds (y). If Mendel crossed homozygous yellow, round-seed pea plants with homozygous green, wrinkle-seed pea plants, the P cross could be represented by YYRR × yyrr. The F1 generation genotype would be YyRr—yellow, round-seed plants. These F1-generation plants are called dihybrids because they are heterozygous for both traits.

Law of independent assortment Mendel allowed F1 pea plants with the genotype YyRr to self-fertilize in a dihybrid cross. Mendel calculated the genotypic and phenotypic ratios of the offspring in both the F1 and F2 generations. From these results, he developed the **law of independent assortment**, which states that a random distribution of alleles occurs during gamete formation. Genes on separate chromosomes sort independently during meiosis.

As shown in Figure 10.11, the random assortment of alleles results in four possible gametes: YR, Yr, yR or yr, each of which is equally likely to occur. When a plant self-fertilizes, any of the four allele combinations could be present in the male gamete, and any of the four combinations could be present in the female gamete. The results of Mendel’s dihybrid cross included nine different genotypes: YYRR, YYRr, YYrr, YyRR, YyRr, Yyrr, yyRR, yyRr, and yyrr. He counted and recorded four different phenotypes: 315 yellow round, 108 green round, 101 yellow wrinkled, and 32 green wrinkled. These results represent a phenotypic ratio of approximately 9:3:3:1.

Punnett Squares

In the early 1900s, Dr. Reginald Punnett developed what is known as a Punnett square to predict the possible offspring of a cross between two known genotypes. Punnett squares make it easier to keep track of the possible genotypes involved in a cross.

Punnett square—monohybrid cross Can you roll your tongue like the person pictured in Figure 10.12? Tongue-rolling ability is a dominant trait, which can be represented by T. Suppose both parents can roll their tongues and are heterozygous (Tt) for the trait. What possible phenotypes could their children have?

Examine the Punnett square in Figure 10.12. The number of squares is determined by the number of different types of alleles—T or t—produced by each parent. In this case, the square is 2 squares × 2 squares because each parent produces two different types of gametes.

Notice that the male gametes are written across the horizontal side and the female gametes are written on the vertical side of the Punnett square. The possible combinations of each male and female gamete are written on the inside of each corresponding square.

How many different genotypes are found in the Punnett square? One square has TT, two squares have Tt, and one square has tt. Therefore, the genotypic ratio of the possible offspring is 1:2:1. The phenotypic ratio of tongue rollers to non-tongue rollers is 3:1.

Punnett square—dihybrid cross Now examine the Punnett square in Figure 10.13. Notice that in the P cross, only two types of alleles are produced. However, in the dihybrid cross— when the F1 generation is crossed—four types of alleles from the male gametes and four types of alleles from the female gametes can be produced. The resulting phenotypic ratio is 9:3:3:1, yellow round to green round to yellow wrinkled to green wrinkled. Mendel’s data closely matched the out- come predicted by the Punnett square.

Probability

The inheritance of genes can be compared to the probability of flipping a coin. The probability of the coin landing on heads is 1 out of 2, or 1/2. If the same coin is flipped twice, the probability of it landing on heads is 1/2 each time or 1/2 × 1/2, or 1/4 both times.

Actual data might not perfectly match the predicted ratios. You know that if you flip a coin you might not get heads 1 out of 2 times. Mendel’s results were not exactly a 9:3:3:1 ratio. However, the larger the number of offspring involved in a cross, the more likely it will match the results predicted by the Punnett square.

Gene Linkage and Polyploidy

Genetic Recombination

The new combination of genes produced by crossing over and independent assortment is called genetic recombination. The possible combinations of genes due to independent assortment can be calculated using the formula 2n, where n is the number of chromosome pairs. For example, pea plants have seven pairs of chromosomes. For seven pairs of chromosomes, the possible combinations are 27, or 128 combinations. Because any possible male gamete can fertilize any possible female gamete, the number of possible combinations after fertilization is 16,384 (128 × 128). In humans, the possible number of combinations after fertilization would be 223 × 223, or more than 70 trillion. This number does not include the amount of genetic recombination produced by crossing over.

Gene Linkage

Recall that chromosomes contain multiple genes that code for proteins. Genes that are located close to each other on the same chromosome are said to be linked and usually travel together during gamete formation. Study Figure 10.14 and observe that genes A and B are located close to each other on the same chromosome and travel together during meiosis. The linkage of genes on a chromosome results in an exception to Mendel’s law of independent assortment because linked genes usually do not segregate independently.

Chromosome maps Crossing over occurs more frequently between genes that are far apart than those that are close together. A drawing called a chromosome map shows the sequence of genes on a chromosome and can be created by using crossover data. The very first chromosome maps were published in 1913 using data from thousands of fruit fly crosses. Chromosome map percentages are not actual chromosome distances, but they represent relative positions of the genes. Figure 10.15 shows the first chromosome map created using fruit fly data. Notice that the higher the crossover frequency, the farther apart the two genes are.

In a cross, the exchange of genes is directly related to the crossover frequency between them. This frequency correlates with the relative distance between the two genes. One map unit between two genes is equivalent to 1 percent of the crossing over occurring between them. Genes that are further apart would have a greater frequency of crossing over.

Polyploidy

Most species have diploid cells, but some have polyploid cells. Polyploidy is the occurrence of one or more extra sets of all chromosomes in an organism. A triploid organism, for instance, would be designated 3n, which means that it has three complete sets of chromosomes. Polyploidy rarely occurs in animals but sometimes occurs in earthworms and goldfish. In humans, polyploidy is always lethal.

Basic Patterns of Human Inheritance

Recessive Genetic Disorders

Mendel’s work was ignored for more than 30 years. During the early 1900s, scientists began to take an interest in heredity, and Mendel’s work was rediscovered. About this time, Dr. Archibald Garrod, an English physician, became interested in a disorder linked to an enzyme deficiency called alkaptonuria, which results in black urine. It is caused by acid excretion into the urine. Dr. Garrod observed that the condition appeared at birth and continued throughout the patient’s life, ultimately affecting bones and joints. He also noted that alkaptonuria ran in families. With the help of another scientist, he determined that alkaptonuria was a recessive genetic disorder.

Today, progress continues to help us understand genetic disorders. Review Table 11.1, and recall that a recessive trait is expressed when the individual is homozygous recessive for that trait. Therefore, those with at least one dominant allele will not express the recessive trait. An individual who is heterozygous for a recessive disorder is called a carrier. Review Table 11.2 as you read about several recessive genetic disorders.

Cystic fibrosis One of the most common recessive genetic disorders among Caucasians is cystic fibrosis, which affects the mucus-producing glands, digestive enzymes, and sweat glands. Chloride ions are not absorbed into the cells of a person with cystic fibrosis but are excreted in the sweat. Without sufficient chloride ions in cells, water does not diffuse from cells. This causes a secretion of thick mucus that affects many areas of the body. The thick mucus clogs the ducts in the pancreas, interrupts digestion, and blocks the tiny respiratory pathways in the lungs. Patients with cystic fibrosis are at a higher risk of infection because of excess mucus in their lungs.

Treatment for cystic fibrosis currently includes physical therapy, medication, special diets, and the use of replacement digestive enzymes. Genetic tests are available to determine whether a person is a carrier, indicating they are carrying the recessive gene.

Albinism In humans, albinism is caused by altered genes, resulting in the absence of the skin pigment melanin in hair and eyes. You will learn more about melanin in Chapter 32. Albinism is found in other animals as well. A person with albinism has white hair, very pale skin, and pink pupils. The absence of pigment in eyes can cause problems with vision. Although we all must protect our skin from the Sun’s ultraviolet radiation, those with albinism need to be especially careful.

Tay-Sachs disease

Tay-Sachs disease is a recessive genetic disorder. Its gene is found on chromosome 15. Often identified by a cherry-red spot on the back of the eye, Tay-Sachs disease (TSD) seems to be predominant among Jews of eastern European descent.

TSD is caused by the absence of the enzymes responsible for breaking down fatty acids called gangliosides. Normally, gangliosides are made and then dissolved as the brain develops. However, in a person affected by Tay-Sachs disease, the gangliosides accumulate in the brain, inflating brain nerve cells and causing mental deterioration.

Galactosemia Galactosemia is a recessive genetic disorder characterized by the inability of the body to digest galactose. During digestion, lactose from milk breaks down into galactose and glucose. Glucose is the sugar used by the body for energy and circulates in the blood. Galactose must be broken down into glucose by an enzyme named GALT. Persons who lack or have defective GALT cannot digest galactose. Persons with galactosemia should avoid milk products.

Dominant Genetic Disorders

Not all genetic disorders are caused by recessive inheritance. As described in Table 11.3, some disorders, such as the rare disorder Huntington’s disease, are caused by dominant alleles. That means those who do not have the disorder are homozygous recessive for the trait.

Huntington’s disease

The dominant genetic disorder Huntington’s disease affects the nervous system and occurs in one out of 10,000 people in the U.S. The symptoms of this disorder first appear in affected individuals between the ages of 30 and 50 years old. The symptoms include a gradual loss of brain function, uncontrollable movements, and emotional disturbances. Genetic tests are available to detect this dominant allele.

Testing positive poses a dilemma, however, because no preventive treatment or cure for this disease currently exists.

Achondroplasia

An individual with the dominant genetic condition known as achondroplasia has a small body size and limbs that are comparatively short. Achondroplasia is the most common form of dwarfism. A person with achondroplasia will have an adult height of about four feet and will have a normal life expectancy.

Interestingly, 75 percent of individuals with achondroplasia are born to parents of average size. In a dominant genetic condition, the genotype will be seen in the phenotype. Therefore, when children with achondroplasia are born to parents of average size, the conclusion is that the condition occurred because of a new mutation or a genetic change.

Pedigrees

In organisms such as peas and fruit flies, scientists can perform crosses to study genetic relationships. In the case of humans, a scientist studies a family history using a pedigree, a diagram that traces the inheritance of a particular trait through several generations. A pedigree uses symbols to illustrate inheritance of the trait. Males are represented by squares, and females are represented by circles, as shown in Figure 11.1. One who expresses the trait being studied is represented by a dark, or filled, square or circle, depending on their gender. One who does not express the trait is represented by an unfilled square or circle.

A horizontal line between two symbols shows that these individuals are the parents of the offspring listed below them. Offspring are listed in descending birth order from left to right and are connected to each other and their parents.

A pedigree uses a numbering system in which Roman numerals represent generations, and individuals are numbered by birth order using Arabic numbers. For example, in Figure 11.1, individual II1 is a female who is the firstborn in generation II.

Analyzing Pedigrees

A pedigree illustrating Tay-Sachs disease is shown in Figure 11.2. Recall from Table 11.2 that Tay-Sachs disease is a recessive genetic disorder caused by the lack of an enzyme involved in lipid metabolism. The missing enzyme causes lipids to build up in the central nervous system, which can lead to death.

Examine the pedigree in Figure 11.2. Note that two unaffected parents, I1 and I2, have an affected child—II3, indicating that each parent has one recessive allele—they both are heterozygous and carriers for the trait. The half-filled square and circle show that both parents are carriers.

The pedigree in Figure 11.3 shows the inheritance of the dominant genetic disorder polydactyly. People with this disorder have extra fingers and toes. Recall that with dominant inheritance the trait is expressed when at least one dominant allele is present. An individual with an unaffected parent and a parent with polydactyly could be either heterozygous or homozygous recessive for the trait.

Each unaffected person would be homozygous recessive for the trait. For example, in Figure 11.3, individual I2 has polydactyly, indicated by the dark circle. Because she shows the trait, she is either homozygous dominant or heterozygous. It can be inferred that she is heterozygous—having one dominant gene and one recessive gene— because offspring II3 and II4 do not have the disorder. Notice that II6 and II7, two unaffected parents, have an unaffected offspring—III2. What can be inferred about II2, based on the phenotype of her parents and her offspring?

Infering genotypes Pedigrees are used to infer genotypes from the observation of phenotypes. By knowing physical traits, genealogists can determine what genes an individual is most likely to have. Pheno-types of entire families are analyzed in order to determine family geno-types, as symbolized in Figure 11.3.

Pedigrees help genetic counselors determine whether inheritance patterns are dominant or recessive. Once the inheritance pattern is determined, the genotypes of the individuals can largely be resolved through pedigree analysis. To analyze pedigrees, one particular trait is studied, and a determination is made as to whether that trait is dominant or recessive. Dominant traits are easier to recognize than recessive traits are, because dominant traits are exhibited in the phenotype.

A recessive trait will not be expressed unless the person is homozygous recessive for the trait. That means that a recessive allele is passed on by each parent. When recessive traits are expressed, the ancestry of the person expressing the trait is followed for several generations to determine which parents and grandparents were carriers of the recessive allele.

Predicting disorders

If good records have been kept within families, disorders in future offspring can be predicted. However, more accuracy can be expected if several individuals within the family can be evaluated. The study of human genetics is difficult, because scientists are limited by time, ethics, and circumstances. For example, it takes decades for each generation to mature and then to have offspring when the study involves humans. Therefore, good record keeping, where it exists, helps scientists use pedigree analysis to study inheritance patterns, to determine phenotypes, and to ascertain genotypes within a family.

Complex Patterns of Inheritance

Incomplete Dominance

Recall that when an organism is heterozygous for a trait, its phenotype will be that of the dominant trait. For example, if the genotype of a pea plant is Tt and T is the genotype for the dominant trait tall, then its phenotype will be tall. Examine Figure 11.4. However, when red-flowered snapdragons (RR) are crossed with white-flowered snapdragons (rr), the heterozygous offspring have pink flowers (Rr). This is an example of incomplete dominance, in which the heterozygous phenotype is an intermediate phenotype between the two homozygous phenotypes. When the heterozygous F1 generation snapdragon plants are allowed to self-fertilize, as in Figure 11.4, the flowers are red, pink, and white in a 1: 2: 1 ratio, respectively.

Codominance

Recall that when an organism is heterozygous for a particular trait the dominant phenotype is expressed. In a complex inheritance pattern called codominance, both alleles are expressed in the heterozygous condition. For example, sickle-cell disease follows codominant inheritance.

Sickle-cell disease

The allele responsible for sickle-cell disease is particularly common in people of African descent, with about nine percent of African Americans having one form of the trait. Sickle-cell disease affects red blood cells and their ability to transport oxygen. The photograph in Figure 11.5 shows the blood cells of an individual who is heterozygous for the sickle-cell trait. Changes in hemoglobin—the protein in red blood cells—cause those blood cells to change to a sickle, or “C”, shape. Sickle-shaped cells do not effectively transport oxygen because they block circulation in small blood vessels. Those who are heterozygous for the trait have both normal and sickle-shaped cells.

These individuals can lead relatively normal lives, as the normal blood cells compensate for the sickle-shaped cells.

Sickle-cell disease and malaria Note in Figure 11.5 the distribution of both sickle-cell disease and malaria in Africa. Some areas with sickle-cell disease overlap areas of widespread malaria. Why might such high levels of the sickle-cell allele exist in central Africa? Scientists have discovered that those who are heterozygous for the sickle-cell trait also have a higher resistance to malaria. The death rate due to malaria is lower where the sickle-cell trait is higher. Because less malaria exists in those areas, more people live to pass on the sickle-cell trait to offspring. Consequently, sickle-cell disease continues to increase in Africa.

Multiple Alleles

So far, you have learned about inheritance involving two forms of alleles for a trait. Some forms of inheritance, such as blood groups in humans, are determined by multiple alleles.

Blood groups in humans ABO blood groups have three forms of alleles, sometimes called AB markers: IA is blood type A; IB is blood type B; and i is blood type O. Type O is the absence of AB markers. Note that allele i is recessive to IA and IB. However, IA and IB are codominant; blood type AB results from both IA and IB alleles. Therefore, ABO blood groups are examples of both multiple alleles and codominance, as shown in Figure 11.6.

Blood also has Rh factors, inherited from each parent. Rh factors are either positive or negative (Rh+ or Rh–); Rh+ is dominant. The Rh factor is a blood protein named after the rhesus monkey, because studies of the rhesus monkey led to discovery of that blood protein.

Coat color of rabbits Multiple alleles can demonstrate a hierarchy of dominance. In rabbits, four alleles code for coat color: C, cch, ch, and c. Allele C is dominant to the other alleles and results in a full color coat.

Allele c is recessive and results in an albino phenotype when the genotype is homozygous recessive. Allele cch is dominant to ch, and allele ch is dominant to c and the hierarchy of dominance can be written as C > cch > ch > c. Figure 11.7 shows the genotypes and phenotypes possible for rabbit-coat color. Full color is dominant over chinchilla, which is dominant over Himalayan, which is dominant over albino.

The presence of multiple alleles increases the possible number of genotypes and phenotypes. Without multiple-allele dominance, two alleles, such as T and t, produce only three possible genotypes—in this example TT, Tt, and tt—and two possible phenotypes. However, the four alleles for rabbit-coat color produce ten possible genotypes and four phenotypes, as shown in Figure 11.7. More variation in rabbit coat color comes from the interaction of the color gene with other genes such as the agouti gene or the broken gene.

Epistasis

Coat color in Labrador retrievers can vary from yellow to black. This variety is the result of one allele hiding the effects of another allele, an interaction called epistasis. A Labrador’s coat color is controlled by two sets of alleles. The dominant allele E determines whether the fur will have dark pigment. The fur of a dog with genotype ee will not have any pigment. The dominant B allele determines how dark the pigment will be. Study Figure 11.8. If the dog’s genotype is EEbb or Eebb, the dog’s fur will be chocolate brown. Genotypes eebb, eeBb, and eeBB will produce a yellow coat, because the e allele masks the effects of the dominant B allele.

Sex Determination

Each cell in your body, except for gametes, contains 46 chromosomes, or 23 pairs of chromosomes. One pair of these chromosomes, the sex chromosomes, determines an individual’s gender. There are two types of sex chromosomes: X and Y. Individuals with two X chromosomes are female, and individuals with an X and a Y chromosome are male. The other 22 pairs of chromosomes are called autosomes. The offspring’s gender is determined by the combination of sex chromosomes in the egg and sperm cell, as shown in Figure 11.9.

Human females have 22 pairs of autosomes and one pair of X chromosomes. Males have 22 pairs of autosomes along with one X and one Y chromosome. If you examine the X and Y chromosomes in Figure 11.9, you will notice that the X chromosome is larger than the Y chromosome. The X chromosome carries a variety of genes that are necessary for the development of both females and males. The Y chromosome mainly has genes that relate to the development of male characteristics.

Because females have two X chromosomes, it seems as though females get two doses of the X chromosome and males get only one dose. To balance the difference in the dose of X-related genes, one of the X chromosomes stops working in each of the female’s body cells. This often is called dosage compensation or X-inactivation. Which X chromosome stops working in each body cell is a completely random event. Dosage compensation occurs in all mammals.

As a result of the Human Genome Project, the National Institutes of Health (NIH) has released new information on the sequence of the human X chromosome. Researchers now believe that some genes on the inactivated X chromosome are more active than previously thought.

Chromosome inactivation

The coat colors of the calico cat shown in Figure 11.10 are caused by the random inactivation of a particular X chromosome. The resulting colors depend on the X chromosome that is activated. The orange patches are formed by the inactivation of the X chromosome carrying the allele for black coat color. Similarly, the black patches are a result of the activation of the X chromosome carrying the allele for orange coat color.

Barr bodies

The inactivated X chromosomes can be observed in cells. In 1949, Canadian scientist Murray Barr observed inactivated X chromosomes in female calico cats. He noticed a condensed, darkly stained structure in the nucleus. The darkly stained, inactivated X chromosomes, such as the one shown in Figure 11.11, are called Barr bodies. It was discovered later that only females, including human females, have Barr bodies in their cell nuclei.

Sex-Linked Traits

Recall that females have two X chromosomes and that males have one X and one Y chromosome. Traits controlled by genes located on the X chromosome are called sex-linked traits—also called X-linked traits. Since males have only one X chromosome, they are affected by recessive X-linked traits more often than are females. Females likely would not express a recessive X-linked trait because the other X chromosome will likely mask the effect of the recessive trait.

Some traits that are located on autosomes may appear to be sex-linked even though they are not. This occurs when an allele appears to be dominant in one gender but recessive in the other. For example, the allele for baldness is recessive in females but dominant in males, causing hair loss that follows a typical pattern called male-pattern baldness. A male would be bald if he were heterozygous for the trait, while the female would be bald only if she were homozygous recessive.

Red-green color blindness

The trait for red-green color blind- ness is a recessive X-linked trait. About 8 percent of males in the United States have red-green color blindness. The photo in Figure 11.12 shows how a person with red-green color blindness might view colors compared to a person who does not have red-green color blindness.

Study the Punnett square shown in Figure 11.12. The mother is a carrier for color blindness, because she has the recessive allele for color blindness on one of her X chromosomes. The father is not color blind, because he does not have the recessive allele. The sex-linked trait is represented by writing the allele on the X chromosome. Notice that the only child that can possibly have red-green color blindness is a male offspring. As a result of it being an X-linked trait, red-green color blindness is very rare in females.

Hemophilia Hemophilia, another recessive sex-linked disorder, is characterized by delayed clotting of the blood. Like red-green color blindness, this disorder is more common in males than in females.

A famous pedigree of hemophilia is one that arose in the family of Queen Victoria of England (1819-1901). Her son Leopold died of hemophilia, and her daughters Alice and Beatrice, illustrated in the pedigree in Figure 11.13, were carriers for the disease. Alice and Beatrice passed on the hemophilia trait to the Russian, German, and Spanish royal families. Follow the generations in this pedigree to see how this trait was passed through Queen Victoria’s family. Queen Victoria’s granddaughter Alexandra, who was a carrier for this trait, married Tsar Nicholas II of Russia. Irene, another granddaughter, passed the trait on to the German royal family. Hemophilia was passed to the Spanish royal family through a third granddaughter, whose name also was Victoria.

Men with hemophilia usually died at an early age until the twentieth century when clotting factors were discovered and given to hemophiliacs. However, blood-borne viruses such as Hepatitis C and HIV were often contracted by hemophiliacs until the 1990s, when safer methods of blood transfusion were discovered.

Polygenic Traits

So far, you have examined traits determined by a pair of genes. Many phenotypic traits, however, arise from the interaction of multiple pairs of genes. Such traits are called polygenic traits. Traits such as skin color, height, eye color, and fingerprint pattern are polygenic traits.

One characteristic of polygenic traits is that, when the frequency of the number of dominant alleles is graphed, as shown in Figure 11.14, the result is a bell-shaped curve. This shows that more of the intermediate phenotypes exist than do the extreme phenotypes.

Environmental Influences

The environment also has an effect on phenotype. For example, the tendency to develop heart disease can be inherited. However, environmental factors such as diet and exercise also can contribute to the occurrence and seriousness of the disease. Other ways in which environment influences phenotype are very familiar to you. You may not have thought of them in terms of phenotype, however. For example, sunlight, water, and temperature are environmental influences that affect an organism’s phenotype.

Sunlight and water

Without enough sunlight, most flowering plants do not bear flowers. Many plants lose their leaves in response to water deficiency. Most organisms experience phenotypic changes from extreme temperature changes. In extreme heat, for example, many plants suffer. Their leaves droop, flower buds shrivel, chlorophyll disappears, and roots stop growing. These are examples that probably do not surprise you, though you might never have thought of them as phenotypic changes. What other environmental factors affect the phenotypes of organisms?

Temperature

Temperature also influences the expression of genes. Notice the fur of the Siamese cat shown in Figure 11.15. The cat’s tail, feet, ears, and nose are dark. These areas of the cat’s body are cooler than the rest. The gene that codes for production of the color pigment in the Siamese cat’s body functions only under cooler conditions. Therefore, the cooler regions are darker; and the warmer regions, where pigment production is inhibited by temperature, are lighter.

Twin Studies

Another way to study inheritance patterns is to focus on identical twins, which helps scientists separate genetic contributions from environmental contributions. Identical twins are genetically the same. If a trait is inherited, both identical twins will have the trait. Scientists conclude that traits that appear frequently in identical twins are at least partially controlled by heredity. Also, scientists presume that traits expressed differently in identical twins are strongly influenced by environment. The percentage of twins who both express a given trait is called a concordance rate. Examine Figure 11.16 for some traits and their concordance rates. A large difference between fraternal twins and identical twins shows a strong genetic influence.

Chromosomes and Human Heredity

Karyotype Studies

The study of genetic material does not involve the study of genes alone. Scientists also study whole chromosomes by using images of chromosomes stained during **metaphase**. The staining bands identify or mark identical places on homologous chromosomes. Recall from Chapter 9 that during **metaphase** of mitosis, each chromosome has condensed greatly and consists of two sister chromatids. The pairs of homologous chromosomes are arranged in decreasing size to produce a micrograph called a karyotype. Karyotypes of a human male and a human female, each with 23 pairs of chromosomes, are shown in Figure 11.17. Notice that the 22 autosomes are matched together with one pair of nonmatching sex chromosomes.

Telomeres

Scientists have found that chromosomes end in protective caps called telomeres. Telomere caps consist of DNA associated with proteins. The cap serves a protective function for the structure of the chromosome. Scientists have discovered that telomeres also might be involved in both aging and cancer.

Nondisjunction

During cell division, the chromosomes separate, with one of each of the sister chromatids going to opposite poles of the cell. Therefore, each new cell has the correct number of chromosomes. Cell division during which sister chromatids fail to separate properly, which does happen occasionally, is called nondisjunction.

If nondisjunction occurs during meiosis I or meiosis II, as shown in Figure 11.18, the resulting gametes will not have the correct number of chromosomes. When one of these gametes fertilizes another gamete, the resulting offspring will not have the correct number of chromosomes. Notice that nondisjunction can result in extra copies of a certain chromosome or only one copy of a particular chromosome in the offspring. Having a set of three chromosomes of one kind is called trisomy. Having only one of a particular type of chromosome is called monosomy. Nondisjunction can occur in any organism in which gametes are produced through meiosis. In humans, alterations of chromosome number are associated with serious human disorders, which often are fatal.

Down syndrome

One of the earliest known human chromosomal disorders is Down syndrome. It usually is the result of an extra chromosome 21. Therefore, Down syndrome often is called trisomy 21.

Examine the karyotype of a child with Down syndrome, shown in Figure 11.19. Notice that she has three copies of chromosome 21. The characteristics of Down syndrome include distinctive facial features as shown in Figure 11.19, short stature, heart defects, and mental disability. The frequency of children born with Down syndrome in the United States is approximately one out of 800. The frequency of Down syndrome increases with the age of the mother. Studies have shown that the risk of having a child with Down syndrome is about 6 percent in mothers who are 45 and older.

Sex chromosomes Nondisjunction occurs in both autosomes and sex chromosomes. Some of the results of nondisjunction in human sex chromosomes are listed in Table 11.4. Note that an individual with Turner’s syndrome has only one sex chromosome. This condition results from fertilization with a gamete that had no sex chromosome.

Fetal Testing

Couples who suspect they might be carriers for certain genetic disorders might want to have a fetal test performed. Older couples also might wish to know the chromosomal status of their developing baby, known as the fetus. Various types of tests for observing both the mother and the baby are available.

Many fetal tests can provide important information to the parents and the physician. Table 11.5 describes the risks and benefits of some of the fetal tests that are available. Physicians must consider many factors when advising such examinations. At least a small degree of risk usually is possible in any test or procedure. The physician would not want to advise tests that would endanger the mother or the fetus; therefore, when considering whether to recommend fetal testing, the physician would need to consider previous health problems of the mother and the health of the fetus as well. If the physician and parents determine that any fetal test is needed, the health of both the mother and the fetus need to be closely monitored throughout the testing.

DNA: The Genetic Material

Discovery of the Genetic Material

Once Mendel’s work was rediscovered in the 1900s, scientists began to search for the molecule involved in inheritance. Scientists knew that genetic information was carried on the chromosomes in eukaryotic cells, and that the two main components of chromosomes are DNA and protein. For many years, scientists tried to determine which of these macromolecules—nucleic acid (DNA) or proteins—was the source of genetic information.

Griffith The first major experiment that led to the discovery of DNA as the genetic material was performed by Fredrick Griffith in 1928.

Griffith studied two strains of the bacteria Streptococcus pneumoniae, which causes pneumonia. He found that one strain could be transformed, or changed, into the other form.

Of the two strains he studied, one had a sugar coat and one did not. Both strains are shown in Figure 12.1. The coated strain causes pneumonia and is called the smooth (S) strain. The noncoated strain does not cause pneumonia and is called the rough (R) strain because, without the coat, the bacteria colonies have rough edges.

Avery In 1944, Oswald Avery and his colleagues identified the molecule that transformed the R strain of bacteria into the S strain. Avery isolated different macromolecules, such as DNA, protein, and lipids, from killed S cells. Then he exposed live R cells to the macromolecules separately. When the live R cells were exposed to the S strain DNA, they were transformed into S cells. Avery concluded that when the S cells in Griffith’s experiments were killed, DNA was released. Some of the R bacteria incorporated this DNA into their cells, and this changed the bacteria into S cells. Avery’s conclusions were not widely accepted by the scientific community, and many biologists continued to question and experiment to determine whether proteins or DNA were responsible for the transfer of genetic material.

Hershey and Chase In 1952, Alfred Hershey and Martha Chase published results of experiments that provided definitive evidence that DNA is the transforming factor. These experiments involved a bacteriophage, a type of virus that attacks bacteria.

Two components made the experiment ideal for confirming that DNA is the genetic material. First, the bacteriophage used in the experiment was made of DNA and protein. Second, viruses cannot replicate themselves. They must inject their genetic material into a living cell to reproduce. Hershey and Chase labeled both parts of the virus to determine which part was injected into the bacteria and, thus, which part was the genetic material.

Radioactive labeling Hershey and Chase used a technique called radioactive labeling to trace the fate of the DNA and protein as the bacteriophages infected bacteria and reproduced. Follow along in Figure 12.3 as you continue learning about the Hershey-Chase experiment. They labeled one set of bacteriophages with radioactive phosphorus (32P). Proteins do not contain phosphorous, so DNA and not protein in these viruses would be radioactive. Hershey and Chase labeled another set of bacteriophages with radioactive sulfur (35S). Because proteins contain sulfur and DNA does not, proteins and not DNA would be radioactive.

Hershey and Chase infected bacteria with viruses from the two groups. When viruses infect bacteria, they attach to the outside of the bacteria and inject their genetic material. The infected bacteria then were separated from the viruses.

Tracking DNA Hershey and Chase examined Group 1 labeled with 32P and found that the labeled viral DNA had been injected into the bacteria. Viruses later released from the infected bacteria contained 32P, further indicating that DNA was the carrier of genetic information.

When examining Group 2 labeled with 35S, Hershey and Chase observed that the labeled proteins were found outside of the bacterial cells. Viral replication had occurred in the bacterial cells, indicating that the viruses’ genetic material had entered the bacteria, but no label (35S) was found. Table 12.1 summarizes the results of the Hershey-Chase experiment.

Based on their results, Hershey and Chase concluded that the viral DNA was injected into the cell and provided the genetic information needed to produce new viruses. This experiment provided powerful evidence that DNA, not protein, was the genetic material that could be passed from generation to generation in viruses.

DNA Structure

After the Hershey-Chase experiment, scientists were more confident that DNA was the genetic material. The clues had led to the identification of the genetic material, but the questions of how nucleotides came together to form DNA and how DNA could communicate information remained.

Nucleotides

In the 1920s, the biochemist P. A. Levene determined the basic structure of nucleotides that make up DNA. Nucleotides are the subunits of nucleic acids and consist of a five-carbon sugar, a phosphate group, and a nitrogenous base. The two nucleic acids found in living cells are DNA and RNA, which you learned about in Chapter 6. DNA nucleotides contain the sugar deoxyribose, a phosphate, and one of four nitrogenous bases: adenine, guanine, cytosine, or thymine. RNA nucleotides contain the sugar ribose, a phosphate, and one of four nitrogenous bases: adenine, guanine, cytosine, or uracil.

Notice in Figure 12.4 that guanine (G) and adenine (A) are double-ringed bases. This type of base is called a purine base. Thymine (T), cytosine (C), and uracil (U) are single-ringed bases called pyrimidine bases.

Chargaff Erwin Chargaff analyzed the amount of adenine, guanine, thymine, and cytosine in the DNA of various species. A portion of Chargaff’s data, published in 1950, is shown in Figure 12.5. Chargaff found that the amount of guanine nearly equals the amount of cytosine, and the amount of adenine nearly equals the amount of thymine within a species. This finding is known as Chargaff’s rule: C = G and T = A.

The structure question

When four scientists joined the search for the DNA structure, the meaning and importance of Chargaff’s data became clear. Rosalind Franklin, a British chemist; Maurice Wilkins, a British physicist; Francis Crick, a British physicist; and James Watson, an American biologist, provided information that was pivotal in answering the DNA structure question.

X-ray diffraction

Wilkins was working at King’s College in London, England, with a technique called X-ray diffraction, a technique that involved aiming X rays at the DNA molecule. In 1951, Franklin joined the staff at King’s College. There she took the now famous Photo 51 and collected data eventually used by Watson and Crick. Photo 51, shown in Figure 12.6, indicated that DNA was a double helix, or twisted ladder shape, formed by two strands of nucleotides twisted around each other. The specific structure of the DNA double helix was determined later by Watson and Crick when they used Franklin’s data and other mathematical data. DNA is the genetic material of all organisms, composed of two complementary, precisely paired strands of nucleotides wound in a double helix.

Watson and Crick Watson and Crick were working at Cambridge University in Cambridge, England, when they saw Franklin’s X-ray diffraction picture. Using Chargaff’s data and Franklin’s data, Watson and Crick measured the width of the helix and the spacing of the bases.

Together, they built a model of the double helix that conformed to the others’ research. The model they built is shown in Figure 12.7. Some important features of their proposed molecule include the following:

1.two outside strands consist of alternating deoxyribose and phosphate

2.cytosine and guanine bases pair to each other by three hydrogen bonds

3.thymine and adenine bases pair to each other by two hydrogen bonds

DNA structure DNA often is compared to a twisted ladder, with the rails of the ladder represented by the alternating deoxyribose and phosphate. The pairs of bases (cytosine–guanine or thymine–adenine) form the steps, or rungs, of the ladder. A purine base always binds to a pyrimidine base, ensuring a consistent distance between the two rails of the ladder. This proposed bonding of the bases also explains Chargaff’s data, which suggested that the number of purine bases equaled the number of pyrimidine bases in a sample of DNA. Remember, cytosine and thymine are pyrimidine bases, adenine and guanine are purines, and C = G and

A = T. Therefore, C + T = G + A, or purine bases equal pyrimidine bases. Complementary base pairing is used to describe the precise pairing of purine and pyrimidine bases between strands of nucleic acids. It is the characteristic of DNA replication through which the parent strand can determine the sequence of a new strand.

Orientation Another unique feature of the DNA molecule is the direction, or orientation, of the two strands. Carbon molecules can be numbered in organic molecules. Figure 12.8 shows the orientation of the numbered carbons in the sugar molecules on each strand of DNA. On the top rail, the orientation of the sugar has the 5' (read “five-prime”) carbon on the left, and on the end of that rail, the 3' (read “three-prime”) carbon is on the right of the sugar-phosphate chain. The strand is said to be oriented 5' to 3'. The strand on the bottom runs in the opposite direction and is oriented 3' to 5'. This orientation of the two strands is called antiparallel. Another way to visualize antiparallel orientation is to take two pencils and position them so that the point of one pencil is next to the eraser of the other and vice versa.

The announcement

In 1953, Watson and Crick surprised the scientific community by publishing a one-page letter in the journal Nature that suggested a structure for DNA and hypothesized a method of replication for the molecule deduced from the structure. In articles individually published in the same issue, Wilkins and Franklin presented evidence that supported the structure proposed by Watson and Crick. Still, the mysteries of how to prove DNA’s replication and how it worked as a genetic code remained.

Chromosome Structure

In prokaryotes, the DNA molecule is contained in the cytoplasm and consists mainly of a ring of DNA and associated proteins. Eukaryotic DNA is organized into individual chromosomes. The length of a human chromosome ranges from 51 million to 245 million base pairs. If a DNA strand 140 million nucleotides long was laid out in a straight line, it would be about five centimeters long. How does all of this DNA fit into a microscopic cell? In order to fit into the nucleus of a eukaryotic cell, the DNA tightly coils around a group of beadlike proteins called histones, as shown in Figure 12.9. The phosphate groups in DNA create a negative charge, which attracts the DNA to the positively charged histone proteins and forms a nucleosome. The nucleosomes then group together into chromatin fibers, which supercoil to make up the DNA structure recognized as a chromosome.

Replication of DNA

Semiconservative Replication

When Watson and Crick presented their model of DNA to the science community, they also suggested a possible method of replication—semiconservative replication. During semiconservative replication, parental strands of DNA separate, serve as templates, and produce DNA molecules that have one strand of parental DNA and one strand of new DNA. Recall from Chapters 9 and 10 that DNA replication occurs during **interphase** of mitosis and meiosis. An overview of semi-conservative replication is in Figure 12.10. The process of semiconservative replication occurs in three main stages: unwinding, base pairing, and joining.

Unwinding DNA helicase, an enzyme, is responsible for unwinding and unzipping the double helix. When the double helix is unzipped, the hydrogen bonds between the bases are broken, leaving single strands of DNA. Then, proteins called single-stranded binding proteins associate with the DNA to keep the strands separate during replication. As the helix unwinds, another enzyme, RNA primase, adds a short segment of RNA, called an RNA primer, on each DNA strand.

Base pairing

The enzyme DNA polymerase catalyzes the addition of appropriate nucleotides to the new DNA strand. The nucleotides are added to the 3' end of the new strand, as illustrated in Figure 12.11. DNA polymerase continues adding new DNA nucleotides to the chain by adding to the 3' end of the new DNA strand. Recall that each base binds only to its complement—A binds to T and C binds to G. In this way, the templates allow identical copies of the original double-stranded DNA to be produced.

Notice in Figure 12.11 that the two strands are made in a slightly different manner. One strand is called the leading strand and is elongated as the DNA unwinds. This strand is built continuously by the addition of nucleotides to the 3' end.

The other strand of DNA, called the lagging strand, elongates away from the replication fork. It is synthesized discontinuously into small segments, called Okazaki fragments, by the DNA polymerase in the 3' to 5' direction. These fragments are later connected by the enzyme DNA ligase. Each Okazaki fragment is about 100–200 nucleotides long in eukaryotes. Because one strand is synthesized continuously and the other is synthesized discontinuously, DNA replication is said to be semidiscontinuous as well as semiconservative.

Joining Even though the leading strand is synthesized continuously, in eukaryotic DNA replication there often are many areas along the chromosome where replication begins. When the DNA polymerase comes to an RNA primer on the DNA, it removes the primer and fills in the place with DNA nucleotides. When the RNA primer has been replaced, DNA ligase links the two sections.

Comparing DNA Replication in Eukaryotes and Prokaryotes

Eukaryotic DNA unwinds in multiple areas as DNA is replicated. Each individual area of a chromosome replicates as a section, which can vary in length from 10,000 to one million base pairs. As a result, multiple areas of replication are occurring along the large eukaryotic chromosome at the same time. Multiple replication origins look like bubbles in the DNA strand, as shown in Figure 12.12.

In prokaryotes, the circular DNA strand is opened at one origin of replication, as shown in Figure 12.12. Notice in the figure that DNA replication occurs in two directions, just as it does in eukaryotes. Recall from Chapter 7 that prokaryotic DNA typically is shorter than eukaryotic DNA and remains in the cytoplasm—not packaged in a nucleus.

DNA, RNA, and Protein

Central Dogma

One of the important features of DNA that remained unresolved beyond the work of Watson and Crick was how DNA served as a genetic code for the synthesis of proteins. Recall from Chapter 6 that proteins function as structural building blocks for the cells and as enzymes.

Geneticists now accept that the basic mechanism of reading and expressing genes is from DNA to RNA to protein. This chain of events occurs in all living things—from bacteria to humans. Scientists refer to this mechanism as the central dogma of biology: DNA codes for RNA, which guides the synthesis of proteins.

RNA RNA is a nucleic acid that is similar to DNA. However, RNA contains the sugar ribose, the base uracil replaces thymine, and usually is single stranded. Three major types of RNA are found in living cells. Messenger RNA (mRNA) molecules are long strands of RNA nucleotides that are formed complementary to one strand of DNA. They travel from the nucleus to the ribosome to direct the synthesis of a specific protein. Ribosomal RNA (rRNA) is the type of RNA that associates with proteins to form ribosomes in the cytoplasm. The third type of RNA, transfer RNA (tRNA) are smaller segments of RNA nucleotides that transport amino acids to the ribosome. Table 12.2 compares the structure and function of the three types of RNA.

Transcription

The first step of the central dogma involves the synthesis of mRNA from DNA in a process called transcription. Through transcription, the DNA code is transferred to mRNA in the nucleus. The mRNA then can take the code into the cytoplasm for protein synthesis. Follow along with the process of transcription in Figure 12.13. The DNA is unzipped in the nucleus and RNA polymerase, an enzyme that regulates RNA synthesis, binds to a specific section where an mRNA will be synthesized. As the DNA strand unwinds, the RNA polymerase initiates mRNA synthesis and moves along one of the DNA strands in the 3' to 5' direction. The strand of DNA that is read by RNA polymerase is called the template strand, and mRNA is synthesized as a complement to the DNA nucleotides. The DNA strand not used as the template strand is called the nontemplate strand. The mRNA transcript is manufactured in a 5' to 3' direction, adding each new RNA nucleotide to the 3' end. Uracil is incorporated instead of thymine as the mRNA molecule is made. Eventually, the mRNA is released, and the RNA polymerase detaches from the DNA. The new mRNA then moves out of the nucleus through nuclear pores into the cytoplasm.

RNA processing

When scientists compared the coding region of the DNA with mRNA that ultimately coded for a protein, they found that the mRNA code is significantly shorter than the DNA code. Upon closer examination, they discovered that the code on the DNA is interrupted periodically by sequences that are not in the final mRNA. These sequences are called intervening sequences, or introns. The coding sequences that remain in the final mRNA are called exons. In eukaryotes, the original mRNA made in the nucleus is sometimes called pre-mRNA and contains all of the DNA code. Before the pre-mRNA leaves the nucleus, the introns are removed from it. Other processing of the pre-mRNA includes adding a protective cap on the 5' end and adding a tail of many adenine nucleotides, called the poly-A tail, to the 3' end of the mRNA. Research shows that the cap aids in ribosome recognition, though the significance of the poly-A tail remains unknown. The mRNA that reaches the ribosome has been processed.

The Code

Biologists began to hypothesize that the instructions for protein synthesis are encoded in the DNA. They recognized that the only way the DNA varied among organisms was in the sequence of the bases. Scientists knew that 20 amino acids were used to make proteins, so they knew that the DNA must provide at least 20 different codes.

The hypothesis for how the bases formed the code is based on math and logic. If each base coded for one amino acid, then the four bases could code for four amino acids. If each pair of bases coded for one amino acid, then the four bases could only code for 16 (4 × 4 or 42) amino acids. However, if a group of three bases coded for one amino acid, there would be 64 (43) possible codes. This provides more than the 20 codes needed for the 20 amino acids, but is the smallest possible combination of bases to provide enough codes for the amino acids.

This reasoning meant that the code was not contained in the base pairs themselves, but must run along a single strand of the DNA. Experiments during the 1960s demonstrated that the DNA code was indeed a three-base code. The three-base code in DNA or mRNA is called a codon. Each of the three bases of a codon in the DNA is transcribed into the mRNA code. Figure 12.14 shows a “dictionary” of the genetic code. Notice that all but three codons are specific for an amino acid— they are stop codons. Codon AUG codes for the amino acid methionine and also functions as the start codon.

Translation Once the mRNA is synthesized and processed, it moves to the ribosome. In eukaryotes, this means the mRNA must leave the nucleus and enter the cytoplasm. Once in the cytoplasm, the 5' end of the mRNA connects to the ribosome. This is where the code is read and translated to make a protein through a process called translation.

In translation, tRNA molecules act as the interpreters of the mRNA codon sequence. The tRNA is folded into a cloverleaf shape and is activated by an enzyme that attaches a specific amino acid to the 3' end. At the middle of the folded strand, there is a three-base coding sequence called the anticodon.

Each anticodon is complementary to a codon on the mRNA. Though the code in DNA and RNA is read 5' to 3', the anticodon is read 3' to 5'.

The role of the ribosome

The ribosome consists of two subunits, as shown in Figure 12.15. These subunits are not associated when they are not involved in protein translation. When the mRNA leaves the nucleus, the two parts of the ribosome come together and attach to the mRNA to complete the ribosome. Once the mRNA is associated with the ribosome, a tRNA with the anticodon CAU carrying a methionine will move in and bind to the mRNA start codon—AUG—on the 5' end of the mRNA. The ribosome structure has a groove, called the P site, where the tRNA that is complementary to the mRNA moves in.

A second tRNA moves into a second groove in the ribosome, called the A site, and corresponds to the next codon of the mRNA. The next codon is UUU, so a tRNA with the anticodon AAA moves in, carrying the amino acid phenylalanine.

Part of the rRNA in the ribosome now acts as an enzyme catalyzing the formation of a bond between the new amino acid in the A site and the amino acid in the P site. As the two amino acids join, the tRNA in the

P site is released to the third site, called the E site, where it exits the ribosome. The ribosome then moves so the tRNA found in Groove A is shifted to Site P, as shown in Figure 12.15. Now a new tRNA will enter the A site, complementing the next codon on the mRNA. This process will continue adding and linking amino acids in the sequence determined by the mRNA.

The ribosome continues to move along until the A site contains a stop codon. The stop codon signals the end of protein synthesis and does not complement any tRNA. Proteins called release factors cause the mRNA to be released from the last tRNA and the ribosome subunits to disassemble, ending protein synthesis.

One Gene—One Enzyme

Once scientists learned how DNA works as a code, they needed to learn the relationships between the genes and the proteins for which they coded. Experiments on the mold Neurospora were the first to demonstrate the relationship between genes and enzymes. In the 1940s, George Beadle and Edward Tatum provided evidence that a gene can code for an enzyme. They studied mold spores that were mutated by exposure to X rays. Examine Figure 12.16 to follow along with their experiment.

Normally, Neurospora can grow on an artificial medium that provides no amino acids. This type of medium is called minimal medium. Complete medium provides all the amino acids that Neurospora needs to function. In Beadle and Tatum’s experiment, the spores were exposed to X rays and grown on a complete medium. To test for a mutated spore, the scientists grew spores on a minimal medium. When a spore was unable to grow on the minimal medium, the mutant was tested to see what amino acid it lacked.

When the mold-spore type grew on a minimal medium with a supplement such as arginine, Beadle and Tatum hypothesized that the mutant was missing the enzyme needed to synthesize arginine.

Beadle and Tatum came up with what is known as the “**one gene—one enzyme” hypothesis**. Today, because we know that polypeptides make up enzymes, their hypothesis has been modified slightly to refer to the fact that one gene codes for one polypeptide.

Gene Regulation and Mutation

Prokaryote Gene Regulation

How do prokaryotic cells regulate which genes will be transcribed at particular times in the lifetime of an organism? Gene regulation is the ability of an organism to control which genes are transcribed in response to the environment. In prokaryotes, an operon often controls the transcription of genes in response to changes in the environment. An operon is a section of DNA that contains the genes for the proteins needed for a specific metabolic pathway. The parts of an operon include an operator, promoter, regulatory gene, and the genes coding for proteins. The operator is a segment of DNA that acts as an on/off switch for transcription. A second segment of DNA, called the promoter, is where the RNA polymerase first binds to the DNA. The bacteria Escherichia coli (E. coli) respond to tryptophan, an amino acid, and lactose, a sugar, through two operons.

The trp operon in bacteria, tryptophan synthesis occurs in a series of five steps, and each step is catalyzed by a specific enzyme. The five genes coding for these enzymes are clustered together on the bacterial chromosome with a group of DNA that controls whether or not they are transcribed. This cluster of DNA is called the tryptophan (trp) operon and is illustrated in Figure 12.17.

The trp operon is referred to as a repressible operon because transcription of the five enzyme genes normally is repressed, or turned off. When tryptophan is present in the cell’s environment, the cell has no need to synthesize it and the trp repressor gene turns off, or represses, the transcription process by making a repressor protein. Tryptophan in E. coli combines with an inactive repressor protein to activate it, and the complex binds to the operator in the promoter sequence. If the repressor is bound to the operator, RNA polymerase cannot bind to it, which prevents the transcription of the enzyme genes. This prohibits the synthesis of tryptophan by the cell.

When tryptophan levels are low, the repressor is not bound to tryptophan and is inactive—it does not bind to the operator. The RNA polymerase is able to bind to the operator, turning on transcription of the five enzyme genes. This transcription enables the synthesis of tryptophan by the cell. Notice the location of the repressor protein in Figure 12.17 when the operon is turned both off and on.

The lac operon

When lactose is present in the cell, E. coli makes enzymes that enable it to use lactose as an energy source. The lactose (lac) operon, illustrated in Figure 12.18, contains a promoter, an operator, a regulatory gene, and three enzyme genes that control lac digestion. In the lac operon, the regulatory gene makes a repressor protein that binds to the operator in the promoter sequence and prevents the transcription of the enzyme genes.

When a molecule called an inducer is present, the inducer binds to the repressor and inactivates it. In the lac operon, the inducer is allolactose, a molecule that is present in food that contains lactose. Thus, when lactose is present, the allolactose binds to the repressor and inactivates it. With the repressor inactivated, RNA polymerase then can bind to the promoter and begin transcription. The lac operon is called an inducible operon because transcription is turned on by an inducer.

Eukaryote Gene Regulation

Eukaryotic cells also must control what genes are expressed at different times in the organism’s lifetime. In eukaryotic cells, many genes interact with one another, requiring more elements than a single promoter and operator for a set of genes. The organization and structure of eukaryotic cells is more complex than in prokaryotic cells, increasing the complexity of the control system.

Controlling transcription

One way that eukaryotes control gene expression is through proteins called transcription factors. Transcription factors ensure that a gene is used at the right time and that proteins are made in the right amounts. There are two main sets of transcription factors. One set of transcription factors forms complexes that guide and stabilize the binding of the RNA polymerase to a promoter. The other set includes regulatory proteins that help control the rate of transcription. For instance, proteins called activators fold DNA so that enhancer sites are close to the complex and increase the rate of gene transcription. Repressor proteins also bind to specific sites on the DNA and prevent the binding of activators.

The complex structure of eukaryotic DNA also regulates transcription. Recall that eukaryotic DNA is wrapped around histones to form nucleosomes. This structure provides some inhibition of transcription, although regulatory proteins and RNA polymerase still can activate specific genes even when they are packaged in the nucleosome.

Hox genes Gene regulation is crucial during development. Recall that multicellular eukaryotes develop from a single cell called a zygote. The zygote undergoes mitosis, producing all the different kinds of cells needed by the organism. Differentiation is the process through which the cells become specialized in structure and function. One group of genes that controls differentiation has been discovered. These genes are called homeobox (Hox) genes.

Hox genes are important for determining the body plan of an organism. They code for transcription factors and are active in zones of the embryo that are in the same order as the genes on the chromosome. For example, the colored regions of the fly and fly embryo in Figure 12.19 correspond to the colored genes on the piece of DNA in the figure. These genes, transcribed at specific times, and located in specific places on the genome, control what body part will develop in a given location. One mutation in the Hox genes of fruit flies has yielded flies with legs growing where their antennae should be. Studying these flies has helped scientists understand more about how genes control the body plan of an organism. Similar clusters of Hox genes that control body plans have been found in all animals.

RNA interference

Another method of eukaryotic gene regulation is RNA interference (RNAi). Small pieces of double-stranded RNA in the cytoplasm of the cell are cut by an enzyme called dicer. The resulting double-stranded segments are called small interfering RNA. They bind to a protein complex that degrades one strand of the RNA. The resulting single-stranded small interfering RNA and protein complex bind to sequence-specific sections of mRNA in the cytoplasm, causing the mRNA in this region to be cut and thus preventing its translation.

Figure 12.20 shows the single-stranded small interfering RNA and protein complex binding to the mRNA. Research and clinical trials are being conducted to investigate the possibility of using RNAi to treat cancer, diabetes, and other diseases.

Mutations

Do you ever make mistakes when you are typing an assignment? When you type, sometimes you might strike the wrong key. Just as you might make a mistake when typing, cells sometimes make mistakes during replication. However, these mistakes are rare, and the cell has repair mechanisms that can repair some damage. Sometimes a permanent change occurs in a cell’s DNA and this is called a mutation. Recall that one inheritance pattern that Mendel studied was round and wrinkled pea seeds. It is now known that the wrinkled phenotype is associated with the absence of an enzyme that influences the shape of starch molecules in the seeds. Because the mutation in the gene causes a change in the protein that is made, the enzyme is nonfunctional.

Types of mutations

Mutations can range from changes in a single base pair in the coding sequence of DNA to the deletions of large pieces of chromosomes. Point mutations involve a chemical change in just one base pair and can be enough to cause a genetic disorder. A point mutation in which one base is exchanged for another is called a substitution.

Most substitutions are missense mutations, where the DNA code is altered so that it codes for the wrong amino acid. Other substitutions, called nonsense mutations, change the codon for an amino acid to a stop codon. Nonsense mutations cause translation to terminate early. Nearly all nonsense mutations lead to proteins that cannot function normally.

Another type of mutation that can occur involves the gain or loss of a nucleotide in the DNA sequence. Insertions are additions of a nucleotide to the DNA sequence, and the loss of a nucleotide is called a deletion. Both of these mutations change the multiples of three codons, from the point of the insertion or deletion, and they are called frame-shift mutations because they change the “frame” of the amino acid sequence. Table 12.3 illustrates various types of mutations and their effect on the DNA sequence.

Sometimes mutations are associated with diseases and disorders. One example is Garrod’s alkaptonuria, which was described in Chapter 11.

Patients with this disorder have a mutation in their DNA coding for an enzyme involved in digesting the amino acid phenylalanine. This mutation results in the black-colored homogentistic acid that discolors the urine. Studies have shown that patients with alkaptonuria have a high occurrence of frameshift and missense mutations in a specific region of their DNA. Table 12.3 lists some more examples of diseases associated with types of mutation.

Large portions of DNA also can be involved in a mutation. A piece of an individual chromosome containing one or more genes can be deleted or moved to a different location on the chromosome, or even to a different chromosome. Such rearrangements of the chromosome often have drastic effects on the expression of these genes.

In 1991, a new kind of mutation was discovered that involves an increase in the number of copies of repeated codons, called tandem repeats. The increase in repeated sequences seems to be involved in a number of inherited disorders. The first known example was fragile X syndrome—a syndrome that results in a number of mental and behavioral impairments. Near the end of a normal X chromosome, there is a section of CGG codons that repeat about 30 times. Individuals with fragile X have CGG codons that repeat hundreds of times. The syndrome received its name because the repeated area on the tip of the X chromosomes appears as a fragile piece hanging off the X chromosome, as illustrated in Figure 12.21. Currently, the mechanism by which the repeats expand from generation to generation is not known.

Protein folding and stability

You might expect that large changes in the DNA code, such as frameshift mutations or changes in position, lead to genetic disorders. However, small changes like substitutions also can lead to genetic disorders. The change of one amino acid for another can change the sequence of amino acids in a protein enough to change both the folding and stability of the protein, as illustrated in Figure 12.22.

In Chapter 11, you learned about a genetic disorder caused by a single point mutation called sickle-cell disease. In the case of sickle-cell disease, the codon for a glutamic acid (GAA) has been changed to a valine (GUA) in the protein. This change in composition changes the structure of hemoglobin and is the cause of this disorder.

Hemoglobin is made of four polypeptide chains—two sets of two identical chains. The molecule also contains a large carbon-ring structure that binds iron called the heme group. The substituted glutamic acid is located near the start of one set of chains, as shown in Figure 12.22. Glutamic acid is a polar amino acid, but the valine that substitutes for it in sickle-cell disease is nonpolar. Because of the charge difference, the sickle-cell hemoglobin folds differently than normal hemoglobin. The abnormal folding of the protein caused by the mutation results in a change to the sickle shape of the red blood cell. Numerous other diseases involve problems with protein folding, including Alzheimer’s disease, cystic fibrosis, diabetes, and cancer.

Causes of mutation

Some mutations, especially point mutations, can occur spontaneously. During replication, DNA polymerase sometimes adds the wrong nucleotides. Because the DNA polymerase has a proofreading function, the wrong nucleotide gets added only for one in one hundred thousand bases; it goes unfixed in less than one in one billion.

Certain chemicals and radiation also can damage DNA. Substances which cause mutations are called mutagens. Many different chemicals have been classified as mutagens. Some of these chemicals affect DNA by changing the chemical structure of the bases. Often these changes cause bases to mispair, or bond, with the wrong base. Other chemical mutagens have chemical structures that resemble nucleotides so closely that they can substitute for them. Once these imposter bases are incorporated into the DNA, it can not replicate properly. This type of chemical has become useful medically, especially in the treatment of HIV—the virus that causes AIDS. Many drugs used to treat HIV and other viral infections mimic various nucleotides. Once the drug is incorporated in the viral DNA, the DNA cannot copy itself properly.

High-energy forms of radiation, such as X rays and gamma rays, are highly mutagenic. When the radiation reaches the DNA, electrons absorb the energy. The electrons can escape their atom, leaving behind a free radical. Free radicals are charged atoms with unpaired electrons that react violently with other molecules, including DNA. Ultraviolet (UV) radiation from the Sun contains less energy than X-ray radiation and does not cause electrons to be ejected from the atoms. However, UV radiation can cause adjacent thymine bases to bind to each other, disrupting the structure of DNA, as shown in Figure 12.23. DNA with this structure disruption, or kink, are unable to replicate properly unless repaired.

Body-cell v. sex-cell mutation When a mutation in a body cell, also called a somatic cell, escapes the repair mechanism, it becomes part of the genetic sequence in that cell and in future daughter cells.

Somatic cell mutations are not passed on to the next generation. In some cases, the mutations do not cause problems for the cell. They could be sequences not used by the adult cell when the mutation occurred, the mutation might have occurred in an exon, or the mutation might not have changed the amino acid coded for. These mutations are called neutral mutations. When the mutation results in the production of an abnormal protein, the cell might not be able to perform its normal function, and cell death might occur. In Chapter 9, you learned that mutations in body cells that cause the cell cycle to be unregulated can lead to cancer. All of these effects are contained within the cells of the organism as long as only body cells are affected.

When mutations occur in sex cells, also called germ-line cells, the mutations are passed on to the organism’s offspring and will be present in every cell of the offspring. In many cases, these mutations do not affect the function of cells in the organism, though they might affect the offspring drastically. When the mutations result in an abnormal protein, the results often are more far reaching than when an abnormal protein is produced in an isolated body cell.

Applied Genetics

Selective Breeding

You might be familiar with different breeds of dogs, such as Saint Bernards, huskies, and German shepherds. Observe some of the phenotypic traits of each breed in Figure 13.1. All have strong, muscular bodies. In addition, Saint Bernards have traits such as a keen sense of smell that make them good rescue dogs. Huskies are endurance runners and pull sleds long distances. German shepherds are highly trainable for special services.

Since ancient times, humans have bred animals with certain traits to obtain offspring that have desired traits. As a result, these traits become more common. Breeding for certain desired traits is not restricted to animals alone. Plants also are bred to produce desired traits, such as larger fruits and shorter growing times. The process by which desired traits of certain plants and animals are selected and passed on to their future generations is called selective breeding. Through the processes of hybridization and inbreeding, desired traits can be passed on to future generations.

Hybridization Recall from Chapter 10 that crossing parent organisms with different forms of a trait to produce offspring with specific traits results in hybrids. Farmers, animal breeders, scientists, and gardeners widely use the production of hybrids, also known as hybridization. They select traits that will give hybrid organisms a competitive edge.

These hybrid organisms can be bred to be more disease-resistant, to produce more offspring, or to grow faster. For example, plant breeders might choose to cross two different varieties of tomato plants in order to produce a hybrid that has both the disease resistance of one parent and the fast growth rate of the other parent.

Care must be taken to identify organisms with desired traits and successfully cross them to yield the right combination of traits from both parents.

A disadvantage of hybridization is that it is time consuming and expensive. For example, it took rice breeders three decades to produce hybrid rice varieties that can produce higher yields than non-hybrid varieties. Because hybrids can be bred to be more nutritious, to have the ability to adapt to a wide range of changes in the environment, and to produce greater numbers of offspring, the advantages of hybridization sometimes outweigh the disadvantages.

Inbreeding

Once a breeder observes a desired trait in an organism, a process is needed to ensure that the trait is passed on to future generations.

This process, in which two closely related organisms are bred to have the desired traits and to eliminate the undesired ones in future generations, is called inbreeding.

Pure breeds are maintained by inbreeding. Clydesdale horses, Angus cattle, and German shepherd dogs are all examples of organisms produced by inbreeding. You might have seen Clydesdale horses at parades and petting zoos. Horse breeders first bred the Clydesdale horse in Scotland hundreds of years ago for use as a farm horse. Because of their strong build, agility, and obedient nature, Clydesdales originally were inbred and used extensively for pulling heavy loads.

A disadvantage of inbreeding is that harmful recessive traits also can be passed on to future generations. Inbreeding increases the chance of homozygous recessive offspring. If both parents carry the recessive allele, the harmful trait likely will not be eliminated.

Test Cross

An important thing a breeder has to determine when producing a hybrid is the genotype of the hybrid. Once a breeder observes the desired trait, if the trait is dominant, then the genotype of the organism could be homozygous dominant or heterozygous. The exact genotype is determined by performing a test cross. A test cross involves breedingan organism that has the unknown genotype with one that is homozygous recessive for the desired trait. If the parent’s genotype is homozygous dominant, all of the offspring will have the dominant phenotype; if it is heterozygous, the offspring will show a 1:1 phenotypic ratio.

Performing a test cross

Suppose a breeder wants to produce hybrid white grapefruits. In grapefruit trees, white color is the dominant trait while red is recessive. Therefore, the red grapefruit trees in the orchard must be homozygous recessive (ww). The genotype of the hybrid white grapefruit tree obtained by the breeder can be homozygous dominant (WW) or heterozygous (Ww) for the white color. Therefore, the breeder has to perform a test cross to determine the genotype of the white grapefruit tree. Recall from Chapter 10 that when performing a cross, pollen from the flower of one plant is transferred to the female organ in a flower of another plant.

Results As shown in the top Punnett square in Figure 13.2, if the white grapefruit tree is homozygous dominant (WW) and is crossed with a red grapefruit tree (ww), then all of the offspring will be heterozygous (Ww) and white in color. In this case, all of the offspring will have the dominant phenotype. However, as shown in the second Punnett square in Figure 13.2, if the white grapefruit tree is heterozygous (Ww), then half the number of offspring will be white and half will be red, and the phenotypic ratio will be 1:1. Review the results in the Punnett squares in Figure 13.2. If the white grapefruit tree is homozygous, all offspring will be heterozygous—white in color. If the tree is heterozygous, half of the test-cross offspring will be white and half will be red.

DNA Technology

Genetic Engineering

By about 1970, researchers had discovered the structure of DNA and had determined the central dogma that information flowed from DNA to RNA and from RNA to proteins. However, scientists did not know much about the function of individual genes. Suppose your friend told you the final score of a high school football game but did not tell you how each player contributed to the game. Your curiosity about the details of the game is similar to the curiosity scientists experienced because they did not know how each gene contributed to a cell’s function.

The situation changed when scientists began using genetic engineering, technology that involves manipulating the DNA of one organism in order to insert exogenous DNA, that is, the DNA of another organism. For example, researchers have inserted a gene for a bioluminescent protein called green fluorescent protein (GFP) into various organisms. GFP, which is a substance naturally found in jellyfishes that live in the north Pacific Ocean, emits a green light when it is exposed to ultraviolet light. Organisms that have been genetically engineered to synthesize the DNA for GFP, such as the mosquito larvae shown in Figure 13.3, can be easily identified in the presence of ultraviolet light. The GFP DNA is attached to exogenous DNA to verify that the DNA has been inserted into the organism. These genetically engineered organisms are used in various processes, such as studying the expression of a particular gene, investigating cellular processes, studying the development of a certain disease, and selecting traits that might be beneficial to humans.

DNA Tools

You have learned that selective breeding is used to produce plants and animals with desired traits. Genetic engineering can be used to increase or decrease the expression of specific genes in selected organisms. It has many applications from human health to agriculture. To understand the impact of genetic engineering, it is helpful to understand the basic tools involved in genetic engineering.

An organism’s genome is the total DNA present in the nucleus of each cell. As you will learn in the next section, genomes, such as the human genome, can contain millions and millions of nucleotides in the DNA within genes. In order to study a specific gene, DNA tools can be used to manipulate DNA and to isolate genes from the rest of the genome.

Restriction enzymes

Some types of bacteria contain powerful defenses against viruses. These cells contain proteins called restriction enzymes that recognize and bind to specific DNA sequences and cleave the DNA within that sequence. A restriction enzyme, also called an endonuclease, cuts the viral DNA into fragments after it enters the bacteria. Since their discovery in the late 1960s, scientists have identified and isolated hundreds of restriction enzymes. They use restriction enzymes as powerful tools for isolating specific genes or regions of the genome. When the restriction enzyme cleaves genomic DNA, it creates fragments of different sizes that are unique to every individual.

EcoRI One restriction enzyme that is used widely by scientists is known as EcoRI. As illustrated in Figure 13.4, EcoRI specifically cuts DNA containing the sequence GAATTC. The ends of the DNA fragments created by EcoRI are called sticky ends because they contain single-stranded DNA that is complementary. The ability of some restriction enzymes to create fragments with sticky ends is important, because these sticky ends can be joined together with other DNA fragments that have complementary sticky ends.

However, not all restriction enzymes create sticky ends. Some enzymes produce fragments containing blunt ends—created when the restriction enzyme cuts straight across both strands. Blunt ends do not have regions of single-stranded DNA and can join to any other DNA fragment with blunt ends.

Gel electrophoresis

An electric current is used to separate the DNA fragments according to the size of the fragments in a process called gel electrophoresis. Figure 13.5 shows how the DNA fragments are loaded on the negatively charged end of a gel. When an electric current is applied, the DNA fragments move toward the positive end of the gel. The smaller fragments move farther faster than the larger ones. The unique pattern created based on the size of the DNA fragment can be compared to known DNA fragments for identification. Also, portions of the gel containing each band can be removed for further study.

Recombinant DNA Technology

When DNA fragments have been separated by gel electrophoresis, fragments of a specific size can be removed from the gel and combined with DNA fragments from another source. This newly generated DNA molecule with DNA from different sources is called recombinant DNA. Recombinant DNA technology has revolutionized the way scientists study DNA, because it enables individual genes to be studied.

Large quantities of recombinant DNA molecules are needed in order to study them. First, a carrier, called a vector, transfers the recombinant DNA into a bacterial cell called the host cell. Plasmids and viruses are commonly used vectors. Plasmids—small, circular, double-stranded DNA molecules that occur naturally in bacteria and yeast cells—can be used as vectors because they can be cut with restriction enzymes. If a plasmid and a DNA fragment obtained from another genome have been cleaved by the same restriction enzyme, the ends of each DNA fragment will be complementary and can be combined, as shown in Figure 13.6. An enzyme normally used by cells in DNA repair and replication, called DNA ligase, joins the two DNA fragments chemically. Ligase joins DNA fragments that have sticky ends as well as those that have blunt ends.

Examine Figure 13.6 again. Notice that the resulting circular DNA molecule contains the plasmid DNA and the DNA fragment isolated from another genome. This recombinant plasmid DNA molecule now can be inserted into a host cell so that large quantities of this type of recombinant DNA can be made.

Gene cloning

To make a large quantity of recombinant plasmid DNA, bacterial cells are mixed with recombinant plasmid DNA. Some of the bacterial cells take up the recombinant plasmid DNA through a process called transformation, as shown in Figure 13.7. Bacterial cells can be transformed using electric pulsation or heat. Recall that all cells, including bacterial cells, have plasma membranes. A short electric pulse or a brief rise in temperature temporarily creates openings in the plasma membrane of the bacteria. These temporary openings allow small molecules, such as the recombinant plasmid DNA, to enter the bacterial cell. The bacterial cells make copies of the recombinant plasmid DNA during cell replication. Large numbers of identical bacteria, each containing the inserted DNA molecules, can be produced through this process called cloning.

Recombinant plasmid DNA contains a gene that codes for resistance to an antibiotic such as ampicillin (AMP). Researchers use this gene to distinguish between bacterial cells that have taken up the recombinant plasmid DNA and those that have not. Notice in Figure 13.7 that when the transformed bacterial cells are exposed to the specific antibiotic, only the bacterial cells that have the plasmid survive.

DNA sequencing

The sequence of the DNA nucleotides of most organisms is unknown. Knowing the sequence of an organism’s DNA or of a cloned DNA fragment provides scientists with valuable information for further study. The sequence of a gene can be used to predict the function of the gene, to compare genes with similar sequences from other organisms, and to identify mutations or errors in the DNA sequence. Because the genomes of most organisms are made up of millions of nucleotides, the DNA molecules used for sequencing reactions first must be cut into smaller fragments using restriction enzymes.

Follow Figure 13.8 to understand how DNA is sequenced. Scientists mix an unknown DNA fragment, DNA polymerase, and the four nucleotides—A, C, G, T in a tube. A small amount of each nucleotide is tagged with a different color of fluorescent dye, which also modifies the structure of the nucleotide. Every time a modified fluorescent-tagged nucleotide is incorporated into the newly synthesized strand, the reaction stops. This produces DNA strands of different lengths. The sequencing reaction is complete when the tagged DNA fragments are separated by gel electrophoresis. The gel is then analyzed in an automated DNA sequencing machine that detects the color of each tagged nucleotide. The sequence of the original DNA template is determined from the order of the tagged fragments.

Polymerase chain reaction

Once the sequence of a DNA fragment is known, a technique called the polymerase chain reaction (PCR) can be used to make millions of copies of a specific region of a DNA fragment. PCR is extremely sensitive and can detect a single DNA molecule in a sample. PCR is useful because this single DNA molecule then can be copied, or amplified, numerous times to be used for DNA analysis. Follow Figure 13.9 as you read about the steps of PCR.

Step 1 PCR is performed by placing the DNA fragment to be copied, DNA polymerase, the four DNA nucleotides, and two short single-stranded pieces of DNA called primers in a tube. The primers are complementary to the ends of the DNA fragment that will be copied and used as starting points for DNA synthesis. PCR begins when the tube is heated.

Step 2 The heat separates the two strands of the template DNA fragment. When the tube is cooled, the primers can bind to each strand of the template DNA. An automated machine called a thermocycler is used to cycle the tube containing all of the components involved in PCR through various hot and cool temperatures.

Step 3 As shown in Figure 13.9, each primer is made to bind to one strand of the DNA fragment. Once the primers are bound, DNA polymerase incorporates the correct nucleotides between the two primers as in DNA replication. This process of heating, cooling, and nucleotide incorporation is repeated 20 to 40 times, resulting in millions of copies of the original fragment. Because the separation of DNA strands requires heat, the DNA polymerase used in PCR has to be able to withstand high temperatures. This special DNA polymerase was isolated from a thermophilic, or heat-loving, bacterium such as those found living in the hot springs of Yellowstone National Park.

Because PCR can detect a single DNA molecule in a sample, it has become one of the most powerful tools used by scientists. PCR is not used only by researchers in laboratories, but also by forensic scientists to identify suspects and victims in crime investigations, and by doctors to detect infectious diseases, such as AIDS.

Genetic engineering uses powerful tools, summarized in Table 13.1, to study and manipulate DNA. Although researchers investigate many different problems, their experimental procedures often include cleavage by a restriction enzyme, isolation of fragments, combination with exogenous DNA, cloning or PCR, and identification of sequences.

Biotechnology

Biotechnology—the use of genetic engineering to find solutions to problems—makes it possible to produce organisms that contain individual genes from another organism. Recall that organisms such as the mosquito larvae shown in Figure 13.3 have a gene from another organism. Such organisms, genetically engineered by inserting a gene from another organism, are called transgenic organisms. Transgenic animals, plants, and bacteria are used not only for research, but also for medical and agricultural purposes.

Transgenic animals

Currently, scientists produce most transgenic animals in laboratories for biological research. Mice, fruit flies, and the roundworm Caenorhabditis elegans, also called C. elegans, are widely used in research laboratories around the world to study diseases and develop ways to treat them. Some transgenic organisms, such as transgenic live-stock, have been produced to improve the food supply and human health. Transgenic goats have been engineered to secrete a protein called anti-thrombin III, which is used to prevent human blood from forming clots during surgery. Researchers are working to produce transgenic chickens and turkeys that are resistant to diseases. Several species of fishes also have been genetically engineered to grow faster. In the future, transgenic organisms might be used as a source of organs for organ transplants.

Transgenic plants Many species of plants have been genetically engineered to be more resistant to insect or viral pests. In 2003, about

67.7 million hectares grown by 7 million farmers in 18 countries were planted with transgenic crops. These crops included herbicide- and insecticide-resistant soybeans, corn, cotton, and canola. Scientists now are producing genetically engineered cotton, as shown in Figure 13.10, that resists insect infestation of the bolls. Researchers also are developing peanuts and soybeans that do not cause allergic reactions.

Other crops are being grown commercially and being field-tested. These crops include sweetpotato plants that are resistant to a virus that could kill most of the African harvest, rice plants with increased iron and vitamins that could decrease malnutrition in Asian countries, and a variety of plants able to survive extreme weather conditions. Prospective crops include bananas that produce vaccines for infectious diseases, such as hepatitis B, and plants that produce biodegradable plastics.

Transgenic bacteria Insulin, growth hormones, and substances that dissolve blood clots are made by transgenic bacteria. Transgenic bacteria also slow the formation of ice crystals on crops to protect them from frost damage, clean up oil spills more efficiently, and decompose garbage.

The Human Genome

The Human Genome Project

The Human Genome Project (HGP) was an international project that was completed in 2003. A genome is the complete genetic information in a cell. The goal of the HGP was to determine the sequence of the approximately three billion nucleotides that make up human DNA and to identify all of the approximately 20,000–25,000 human genes. If all of the nucleotides in the human genome were the size of the type on this page and fused together in one continuous line, the line would extend from Los Angeles, California, to Panama, as illustrated in Figure 13.11.

Though the HGP is finished, analysis of the data generated from this project will continue for many decades. To complete this huge task, researchers also have studied the genomes of several other organisms, including the fruit fly, the mouse, and Escherichia coli—the bacterium present in the human gut. Studies in nonhuman organisms help to develop the technology required to handle the large amounts of data produced by the Human Genome Project. These technologies help to interpret the function of newly identified human genes.

Sequencing the genome Recall from Chapter 10 that human DNA is organized into 46 chromosomes. In order to determine one continuous human genome sequence, each of the 46 human chromosomes was cleaved. Several different restriction enzymes were used in order to produce fragments with overlapping sequences. These fragments were combined with vectors to create recombinant DNA, cloned to make many copies, and sequenced using automated sequencing machines. Computers analyzed the overlapping regions to generate one continuous sequence.

Decoding the sequence of the human genome can be compared to reading a book that was printed in code. Imagine the genome as words in a book written without capitalization, punctuation, or breaks between words, sentences, or paragraphs. Suppose there are strings of letters scattered between and within sentences. Figure 13.12 illustrates how a page from such a book might look. In order to understand what is written, you have to decode the jumbled text. Similarly, scientists had to decode the genetic code in the human genome.

After sequencing the entire human genome, scientists observed that less than two percent of all of the nucleotides in the human genome code for all the proteins in the body. That is, the genome is filled with long stretches of repeated sequences that have no direct function. These regions are called noncoding sequences.

DNA fingerprinting

Unlike the protein-coding regions of

DNA that are almost identical among individuals, the long stretches of noncoding regions of DNA are unique to each individual. When these regions are cut by restriction enzymes, as described earlier in this chapter, the set of DNA fragments produced is unique to every individual. DNA fingerprinting involves separating these DNA fragments using gel electrophoresis in order to observe the distinct banding patterns that are unique to every individual. Forensic scientists use DNA fingerprinting to identify suspects and victims in criminal cases, to determine paternity, and to identify soldiers killed in war.

Figure 13.13 shows a sample, obtained from blood, hair, semen, or skin, that forensic scientists can use for DNA fingerprinting. PCR is used to copy this small amount of DNA to create a larger sample for analysis. The amplified DNA then is cut using different combinations of restriction enzymes. The fragments are separated by gel electrophoresis and compared to DNA fragments from known sources, such as victims and suspects in a criminal case, to locate similar fragmentation patterns.

There is a high probability that the two DNA samples came from the same person if two fragmentation patterns match. Since its development in England in 1985, DNA fingerprinting has been used not only to convict criminals, but also to free innocent people who had been wrongfully imprisoned. Figure 13.14 provides a closer look at the history of genetic technology.

Identifying Genes

Once the genome has been sequenced, the next step in the process is to identify the genes and determine their functions. The functions of many of the estimated 20,000–25,000 genes are still unknown.

Researchers are using techniques that integrate computer analysis and recombinant DNA technology to determine the function of these genes.

For organisms such as bacteria and yeast, whose genomes do not have large regions of noncoding DNA, researchers have identified genes by scanning the sequence for open reading frames. ORFs are stretches of DNA containing at least 100 codons that begin with a start codon and end with a stop codon. While these sequences might indicate a gene, testing needs to be done to determine if these sequences produce functioning proteins.

Recall from Chapter 12 that a codon is a group of three nucleotides that code for an amino acid. Researchers look for the start codon AUG and a stop codon such as UAA, UGA, or UAG. ORF analysis has been used to identify correctly over 90 percent of genes in yeast and bacteria. However, the identification of genes in more complex organisms such as humans requires more sophisticated computer programs called algorithms. These algorithms use information, such as the sequence of the genomes of other organisms, to identify human genes.

Bioinformatics

The completion of the HGP and the sequencing of the genomes of other organisms have resulted in large amounts of data. Not only has this enormous amount of data required careful storage, organization, and indexing of sequence information, but it also has created a new field of study. This field of study, called bioinformatics, involves creating and maintaining databases of biological information. The analysis of sequence information involves finding genes in DNA sequences of various organisms and developing methods to predict the structure and function of newly discovered proteins. Scientists also study the evolution of genes by grouping protein sequences into families of related sequences and comparing similar proteins from different organisms.

DNA Microarrays

Analyzing all the expressed genes from a given organism or a specific cell type can be useful. This analysis can be done using DNA microarrays, which are tiny microscope slides or silicon chips that are spotted with DNA fragments. DNA microarrays can contain a few genes, such as the genes that control the cell cycle, or all of the genes of the human genome. Therefore, a large amount of information can be stored in one small slide or chip. DNA microarrays help researchers determine whether the expression of certain genes is caused by genetic factors or environmental factors.

Follow the steps involved in doing the DNA microarray experiment shown in Figure 13.15. mRNA from two different populations of cells is isolated and converted into complementary DNA (cDNA) strands using an enzyme called reverse transcriptase. The complementary DNA from each cell population is labeled with a specific fluorescent dye—for example, red for cancer cells and green for normal cells. Both pools of complementary DNA are combined on the microarray slide and incubated.

Figure 13.15 shows the fluorescent signals that are produced when the microarray slide is analyzed. When the expression of a gene is the same in both the normal and cancer cells, a yellow spot is produced on the chip. If the expression of a gene is higher in cancer cells, then the spot formed is red. However, if the expression is higher in normal cells, then the spot formed is green.

Because one DNA microarray slide can contain thousands of genes, researchers can examine changes in the expression patterns of multiple genes at the same time. Scientists also are using DNA microarrays to identify new genes and to study changes in the expression of proteins under different growth conditions.

The Genome and Genetic Disorders

Although more than 99 percent of all nucleotide base sequences are exactly the same in all people, sometimes there are variations that are linked to human diseases. These variations in the DNA sequence that occur when a single nucleotide in the genome is altered are called single nucleotide polymorphisms or SNPs (SNIHPS). For a variation to be considered an SNP, it must occur in at least one percent of the population. Many SNPs have no effect on cell function, but scientists hypothesize that SNP maps will help identify many genes associated with many different types of genetic disorders.

The HapMap project

A group of international scientists is creating a catalog of common genetic variations that occur in humans. Recall from Chapter 10 that linked genes are inherited together. Similarly, genetic variations located close together also tend to be inherited together. Therefore, regions of linked variations in the human genome, known as haplotypes, can be located. The project to create this catalog is called the haplotype map, or HapMap project. Assembling the Hap-Map involves identifying groups of SNPs in a specific region of DNA.

Figure 13.16 shows how the genome is divided into haplotypes.

Once completed, the HapMap will describe what these variations are, where they occur in our DNA, and how they are distributed among people within populations and among populations in different parts of the world. The HapMap will enable geneticists to take advantage of how SNPs and other genetic variations are organized on chromosomes. This information will help researchers find genes that cause disease and affect an individual’s response to drugs.

Pharmacogenomics Sequencing the human genome plays a central role in many areas of science because it combines the knowledge of genes, proteins, and SNPs with other areas of science. The study of how genetic inheritance affects the body’s response to drugs is called pharmacogenomics. The benefits of pharmacogenomics include more accurate dosing of drugs that are safer and more specific. Researchers hope that pharmacogenomics will allow for drugs to be custom-made for a specific individual based on his or her genetic makeup. Prescribing drugs based on an individual’s genetic makeup will increase safety, speed recovery, and reduce side effects. Perhaps one day when you are sick, your doctor will read your genetic code and prescribe medicine tailor-made for you.

Gene therapy

A technique aimed at correcting mutated genes that cause human diseases is called gene therapy. Scientists insert a normal gene into a chromosome to replace a dysfunctional gene. In most gene therapy studies, fusing a normal gene to a viral vector, as shown in Figure 13.17, produces recombinant DNA. Target cells in the patient are infected with the virus and the recombinant DNA material is released into the affected cells. Once deposited into cells, the normal gene inserts itself into the genome and begins functioning.

In the 1990s, many hospitals around the United States conducted clinical trials using gene therapy. However, in 2003, the Food and Drug Administration halted all gene therapy trials in the United States. The FDA made this decision after the death of a patient undergoing gene therapy. The death was caused by a reaction to the viral vector. Before gene therapy becomes an effective treatment for genetic disorders, viral vectors that are nontoxic and do not activate a body’s defense reaction need to be engineered.

Genomics and Proteomics

Sequencing the human genome began what researchers call “the genomic era.” Genomics is the study of an organism’s genome. Genomics has become one of the most powerful strategies for identifying human genes and interpreting their functions. In addition to the mass of data obtained from sequencing the genomes of humans, rice, mice, fruit flies, and corn, scientists also are investigating the proteins produced by these genes.

Genes are the primary information storage units, whereas proteins are the machines of a cell. Recall that when a gene is expressed, a protein is produced, as illustrated in Figure 13.18. Therefore, an understanding of how proteins function also is important. For instance, if the genome represents the words in a dictionary, the proteome, which represents all the proteins found in a cell, provides the definition of these words and how to use these words in a sentence. The large-scale study and cataloging of the structure and function of proteins in the human body is called proteomics. Proteomics allows researchers to look at hundreds or thousands of proteins at the same time. This type of broad analysis will better define both normal and disease states.

Scientists anticipate that proteomics will revolutionize the development of new drugs to treat diseases such as Type II diabetes, obesity, and atherosclerosis.

Fossil Evidence of Change

Earth’s Early History

What were the conditions on Earth as it formed, and how did life arise on a lifeless planet? Because there were no people to witness

Earth’s earliest history, it might seem that this is a mystery. Like any good mystery, however, it left clues behind. Each clue to Earth’s history and life’s origin is open to investigation by the scientists who study the history of the Earth.

Land environments

By studying other planets in the solar system and rocks on Earth, scientists conclude that Earth was a molten body when it formed about 4.6 billion years ago. Gravity pulled the densest elements to the center of the planet. After about 500 million years, a solid crust formed on the surface, much like the crust that forms on the top of molten iron, as shown in Figure 14.1. The surface was rich in lighter elements, such as silicon. From the oldest rocks remaining today, scientists infer that Earth’s young surface included a number of volcanic features. In addition, the cooling interior radiated much more heat to the surface than it does today. Meteorites would have caused additional heating as they crashed into Earth’s surface. If there had been any life on Earth, it most likely would have been consumed by the intense heat.

Atmosphere Because of its gravitational field, Earth is a planet that is able to maintain an atmosphere. However, no one can be certain about the exact composition of Earth’s early atmosphere. The gases that likely made up the atmosphere are those that were expelled by volcanoes. Volcanic gases today include water vapor (H2O), carbon dioxide (CO2), sulfur dioxide (SO2), carbon monoxide (CO), hydrogen sulfide (H2S), hydrogen cyanide (HCN), nitrogen (N2), and hydrogen (H2). Scientists infer that the same gases would have been present in Earth’s early atmosphere. The minerals in the oldest known rocks suggest that the early atmosphere, unlike today’s atmosphere, had little or no free oxygen.

Clues in Rocks

Earth eventually cooled to the point where liquid water formed on its surface, which became the first oceans. It was a very short time after this—maybe as little as 500 million years—that life first appeared. The earliest clues about life on Earth date to about 3.5 billion years ago.

The fossil record

A fossil is any preserved evidence of an organism. Six categories of fossils are shown in Table 14.1. Plants, animals, and even bacteria can form fossils. Although there is a rich diversity of fossils, the fossil record is like a book with many missing pages. Perhaps more than 99 percent of the species that ever have lived are now extinct, but only a tiny percentage of these organisms are preserved as fossils.

Most organisms decompose before they have a chance to become fossilized. Only those organisms that are buried rapidly in sediment are readily preserved. This occurs more frequently with organisms living in water because the sediment in aquatic environments is constantly settling, covering, and preserving the remains of organisms.

Fossil formation Fossils do not form in igneous or metamorphic rocks. Igneous rocks form when magma from Earth’s interior cools. Metamorphic rocks form when rocks are exposed to extreme heat and pressure. Fossils usually do not survive the heat or pressure involved in the formation of either of these kinds of rocks.

Nearly all fossils are formed in sedimentary rock through the process described in Figure 14.2. The organism dies and is buried in sediments. The sediments build up until they cover the organism’s remains. In some cases, minerals replace the organic matter or fill the empty pore spaces of the organism. In other cases, the organism decays, leaving behind an impression of its body. The sediments eventually harden into rock.

A paleontologist is a scientist who studies fossils. He or she attempts to read the record of life left in rocks.

From fossil evidence, paleontologists infer the diet of an organism and the environment in which it lived. In fact, paleontologists often can create images of extinct communities.

When geologists began to study rock layers, or strata, in different areas, they noticed that layers of the same age tended to have the same kinds of fossils no matter where the rocks were found. The geologists inferred that all strata of the same age contained similar collections of fossils. This led to the establishment of a relative age scale for rocks all over the world.

Dating fossils Relative dating is a method used to determine the age of rocks by comparing them with those in other layers. Relative dating is based on the law of superposition, illustrated in Figure 14.3, which states that younger layers of rock are deposited on top of older layers. The process is similar to stacking newspapers in a pile as you read them each day.

Unless you disturb the newspapers, the oldest ones will be on the bottom.

Radiometric dating uses the decay of radioactive isotopes to measure the age of a rock. Recall from Chapter 6 that an isotope is a form of an element that has the same atomic number but a different mass number. The method requires that the half-life of the isotope, which is the amount of time it takes for half of the original isotope to decay, is known. The relative amounts of the radioactive isotope and its decay product must also be known.

One radioactive isotope that is commonly used to determine the age of rocks is Uranium 238. Uranium 238 (U238) decays to Lead 206 (Pb206) with a half-life of 4510 million years. When testing a rock sample, scientists calculate the ratio of the parent isotope to the daughter isotope to determine the age of the sample.

Radioactive isotopes that can be used for radiometric dating are found only in igneous or metamorphic rocks, not in sedimentary rocks, so isotopes cannot be used to date rocks that contain fossils. Igneous rocks that are found in layers closely associated with fossil-bearing sedimentary rocks often can be used for assigning relative dates to fossils.

Unaltered materials, such as mummies, can be dated directly using carbon-14. Carbon-14 is used to date organic substances, such as bones and tissues. Given the relatively short half-life of carbon-14, as shown in Figure 14.4, only materials less than 60,000 years old can be dated accurately with this isotope. Older materials don’t have enough radioisotope left to date accurately.

The Geologic Time Scale

Think of geologic time as a ribbon that is 4.6 m long. If each meter represents one billion years, each millimeter represents one million years. Earth was formed at one end of the ribbon, and humans appear at the very tip of the other end. This analogy of time as a ribbon will help you understand the long sequences of time represented by the geologic time scale.

The **geologic time scale**, shown in Figure 14.5, is a model that expresses the major geological and biological events in Earth’s history. The geologic time scale is divided into the **Precambrian** time and the **Phanerozoic eon**. An era is the next largest division of the geologic time scale. Notice in Figure 14.5 that the eras of the Phanerozoic eon include the Paleozoic, Mesozoic, and Cenozoic eras. Each era is divided into one or more periods.

In 2004, geologists worldwide agreed on a revision of the names and dates in the geologic time scale based on a project coordinated by the International Commission on Stratigraphy. As in all fields of science, continuing research and discoveries might result in future revisions.

Precambrian

The first 4 m of the geologic time ribbon in Figure 14.5 make up the **Precambrian**. This is nearly 90 percent of Earth’s entire history, stretching from the formation of Earth to the beginning of the **Paleozoic era** about 542 million years ago. The **Precambrian** was an important time. Earth formed and life first appeared. Eventually, autotrophic prokaryotes, much like the cyanobacteria that made the stromatolites in Figure 14.6, enriched the atmosphere with oxygen. Eukaryotic cells also emerged, and by the end of the **Precambrian**, life was flourishing, and the first animals had appeared.

Extensive glaciation marked the second half of the **Precambrian**. This might have delayed the further evolution of life until the ice receded at the beginning of the **Ediacaran period**. The **Ediacaran period** was added to the time scale in 2004. It is the first new period added to the time scale since 1891 and reflects new knowledge of Earth’s history. The **Ediacaran period** lasted from about 630 million years ago to about 542 million years ago, representing about three quarters of a meter on the time ribbon at the end of the **Precambrian**. Simple organisms, such as the fossil in Figure 14.7, inhabited Ediacaran marine ecosystems. Food chains probably were short, and likely were dominated by animals that consumed tiny particles suspended in the water and by animals that ate debris on the bottom of the sea.

The Paleozoic era

A drastic change in the history of animal life on Earth marked the start of the **Paleozoic era**. In the space of just a few million years, the ancestors of most major animal groups diversified in what scientists call the Cambrian explosion. Not all major groups of organisms evolved rapidly at this time, and paleontologists still do not know when the rapid changes started or ended.

Although major changes in ocean life occurred during the **Paleozoic**, there is no evidence of life on land at the start of the Paleozoic. Life in the oceans continued to evolve at the end of the **Cambrian period**. Fish, land plants, and insects appeared during the **Ordovician and Silurian periods**. Organisms of many kinds, including huge insects, soon flourished in the swampy forests that dominated the land, as shown in Figure 14.8. The first tetrapods emerged in the **Devonian period**. Tetrapods were the first land vertebrates—animals with backbones. By the end of the **Carboniferous period**, the first reptiles were roaming the forests.

A mass extinction ended the **Paleozoic era** at the end of the **Permian period**. Recall from Chapter 5 that a mass extinction is an event in which many species become extinct in a short time. Mass extinctions have occurred every 26 to 30 million years on average, which is about 26 to 30 mm on your geologic time scale ribbon. Between 60 and 75 percent of the species alive went extinct in each of these events. Other mass extinctions have not been as severe as the one that ended the **Permian period**, during which 90 percent of marine organisms disappeared. Geologists disagree about the cause of the Permian extinction, but most agree that geological forces, including increased volcanic activity, would have disrupted ecosystems or changed the climate.

The Mesozoic era Biological change continued through the **Mesozoic era**. At the beginning of the **Triassic period**, the ancestors of early mammals were the dominant land animals. Mammals and dinosaurs first appeared late in the **Triassic period**, and flowering plants evolved from nonflowering plants. Birds evolved from a group of predatory dinosaurs in the middle **Jurassic period**. For the rest of the **Mesozoic**, reptiles, particularly the dinosaurs, were the dominant organisms on the planet, as illustrated in Figure 14.9.

Then, about 65 million years ago, a meteorite struck Earth.

The primary evidence for this meteorite impact is found in a layer of material between the rocks of the **Cretaceous period** and the rocks of the **Paleogene period**, the first period of the **Cenozoic era**. Paleontologists call this layer the K-T boundary. Within this layer, scientists find unusually high levels of an element called iridium. Iridium is rare on Earth, but relatively common in meteorites. Therefore, the presence of iridium on Earth indicates a meteorite impact.

Many scientists think that this impact is related to the mass extinction at the end of the **Mesozoic era**, which eliminated all dinosaurs except birds, most marine reptiles, many marine invertebrates, and numerous plant species. The meteorite itself did not wipe out all of these species, but the debris from the impact probably stayed in the atmosphere for months or even years, affecting global climate. Those species that could not adjust to the changing climate disappeared.

Scientists also believe that the course of evolution in the Cenozoic era was shaped by the massive geological changes that characterized the **Mesozoic era**, as shown in Figure 14.10. While it might appear to us that continents are immobile, they actually have been moving since they formed. Alfred Wegener, a German scientist, presented the first evidence for continental drift in the 1920s. Continental drift has since become part of the theory of plate tectonics. **Plate tectonics** describes the movement of several large plates that make up the surface of Earth. These plates, some of which contain continents, move atop a partially molten layer of rock underneath them.

The Cenozoic era

The most recent era is the one in which mammals became the dominant land animals. At the beginning of the Cenozoic era, which means “recent life,” most mammals were small and resembled shrews. After the mass extinction at the end of the **Mesozoic era**, mammals began to diversify into distinct groups, including primates—the group to which you belong. Humans appeared very recently, near the end of the geologic time scale, in the current **Neogene period**. Humans survived the last ice age, but many species of mammals did not. To get an idea of how recently modern humans have appeared, you need to remove about two threads at the end of your geologic time ribbon. These threads represent the time that humans have existed on Earth.

The Origin of Life

Origins: Early Ideas

Perhaps one of the oldest ideas about the origin of life is spontaneous generation. Spontaneous generation is the idea that life arises from nonlife. For example, at one time people thought that mice could be created by placing damp hay and corn in a dark corner, or that mud could give rise to worms, insects, and fish. These ideas might seem humorous to us today, but before much was known about reproduction, it is easy to see how someone might form these conclusions.

One of the first recorded investigations of spontaneous generation came in 1668. Francesco Redi, an Italian scientist, tested the idea that flies arose spontaneously from rotting meat. He hypothesized that flies—not meat—produced other flies. In his experiment, illustrated using present-day equipment in Figure 14.11, Redi observed that maggots, the larvae of flies, appeared only in flasks that were open to flies. Closed flasks had no flies and no maggots. The results of his experiments failed to convince everyone, however. Although people were beginning to use the microscope during Redi’s time and knew that organisms invisible to the naked eye could be found almost everywhere, some thought that these tiny organisms must arise spontaneously, even if flies did not.

The idea of spontaneous generation was not completely rejected until the mid-1800s. It was replaced by the theory of biogenesis, which states that only living organisms can produce other living organisms. Louis Pasteur designed an experiment to show that biogenesis was true even for microorganisms. Pasteur’s experiment is illustrated in Figure 14.12. In one flask, only air was allowed to contact a sterile nutrient broth. Nutrient broth supports the growth of microorganisms. In another flask, both air and microorganisms were allowed to contact the broth. No microorganisms grew in the first container. They did, however, grow in the second container.

Origins: Modern Ideas

If life can arise only from pre-existing life, then how did the first life-form appear? Most biologists agree that life originated through a series of chemical events early in Earth’s history. During these events, complex organic molecules were generated from simpler ones. Eventually, simple metabolic pathways developed. Such pathways allowed molecules to be synthesized or broken down more efficiently. These pathways might have led to the emergence of life as we know it. How this happened is a topic of ongoing research among scientists today.

Simple organic molecule formation

The **primordial soup hypothesis** was an early hypothesis about the origin of life. Scientists Alexander Oparin and John Haldane suggested this hypothesis in the 1920s. They thought that if Earth’s early atmosphere had a mix of certain gases, organic molecules could have been synthesized from simple reactions involving those gases in the early oceans. UV light from the Sun and electric discharge in lightning might have been the primary energy sources. They thought that these organic molecules would have eventually supplied the precursors to life.

In 1953, American scientists Stanley Miller and Harold Urey were the first to show that simple organic molecules could be made from inorganic compounds, as proposed by Oparin and Haldane. Miller and Urey built a glass apparatus, illustrated in Figure 14.13, to simulate the early Earth conditions hypothesized by Oparin. They filled the apparatus with water and the gases that they thought had made up the early atmosphere. The water was boiled and electric discharges were used to simulate lightning as an energy source. Upon examination, the resulting mixture contained a variety of organic compounds including amino acids. Because amino acids are the building blocks of proteins, this discovery supported the primordial soup hypothesis.

Later, other scientists found that hydrogen cyanide could be formed from even simpler molecules in simulated early Earth environments.

Hydrogen cyanide can react with itself to eventually form adenine, one of the nucleotide bases in the genetic code. Many other experiments have since been carried out under conditions that probably reflect the atmosphere of early Earth more accurately. The final reaction products in these experiments were amino acids and sugars as well as nucleotides.

Deep-sea vents

Some scientists suggest that the organic reactions that preceded life’s emergence began in the hydrothermal volcanic vents of the deep sea, where sulfur forms the base of a unique food chain. Still others believe that meteorites might have brought the first organic molecules to Earth.

Making proteins

Wherever the first organic molecules originated, it is clear that the next critical step was the formation of proteins.

Amino acids alone are not sufficient for life. Life requires proteins, which, as you might recall from Chapter 6, are chains of amino acids. In the Miller-Urey experiment, amino acids could bond to one another, but they could separate just as quickly, as illustrated in Figure 14.14.

One possible mechanism for the formation of proteins would be if amino acids were bound to a clay particle. Clay would have been a common sediment in early oceans, and it could have provided a framework for protein assembly.

Genetic code

Another requirement for life is a coding system for protein production. All modern life has such a system, based on either RNA or DNA. Because all DNA-based life-forms also contain RNA, and because some RNA sequences appear to have changed very little through time, many biologists consider RNA to have been life’s first coding system. Researchers have been able to demonstrate that RNA systems are capable of evolution by natural selection. Some RNAs also can behave like enzymes. These RNA molecules, called ribozymes, could have carried out some early life processes. Other researchers have proposed that clay crystals could have provided an initial template for RNA replication, and that eventually the resulting molecules developed their own replication mechanism.

Molecules to cells

Another important step in the evolution of life was the formation of membranes. Researchers have tested ways of enclosing molecules in membranes, allowing early metabolic and replication pathways to develop. In this work, as in other origin-of-life research, the connection between the various chemical events and the overall path from molecules to cells remains unresolved.

Cellular Evolution

What were the earliest cells like? Scientists don’t know because the first life left no fossils. The earliest fossils are 3.5 billion years old. Chemical markings in rocks as old as 3.8 billion years suggest that life was present at that time even though no fossils remain. In 2004, scientists announced the discovery of what appeared to be fossilized microbes in volcanic rock that is 3.5 billion years old. This suggests that cellular activity had become established very early in Earth’s history. It also suggests that early life might have been linked to volcanic environments.

The first cells Scientists hypothesize that the first cells were prokaryotes. Recall from Chapter 7 that prokaryotic cells are much smaller than eukaryotic cells, and they lack a defined nucleus and most other organelles. Many scientists think that modern prokaryotes called archaea are the closest relatives of Earth’s first cells. These organisms often live in extreme environments, such as the hot springs of Yellowstone Park or the volcanic vents in the deep sea, such as the one shown in Figure 14.15. These are environments similar to the environment that might have existed on early Earth.

Photosynthesizing prokaryotes

Although archaea are autotrophic, they do not obtain their energy from the Sun. Instead, they extract energy from inorganic compounds such as sulfur. Archaea also do not need or produce oxygen.

Scientists think that oxygen was absent from Earth’s earliest atmosphere until about 1.8 billion years ago. Any oxygen that appeared earlier than 1.8 billion years ago likely bonded with free ions of iron as oxygen does today. Evidence that iron oxide was formed by oxygen generated by early life is found in unique sedimentary rock formations, such as those shown in Figure 14.16, that are between about 1.8 billion and 2.5 billion years old. Scientists hypothesize that after 1.8 billion years ago, the early Earth’s free iron was saturated with oxygen, and oxygen instead began accumulating in the atmosphere.

Many scientists think that photosynthesizing prokaryotes evolved not long after the archaea—very early in life’s history. Fossil evidence of these primitive prokaryotes, called cyanobacteria, has been found in rocks as old as 3.5 billion years. Cyanobacteria eventually produced enough oxygen to support the formation of an ozone layer. Once an ozone shield was established, conditions would be right for the appearance of eukaryotic cells.

The endosymbiont theory Eukaryotic cells appeared in the fossil record about 1.8 billion years ago, around two billion years after life first formed. Eukaryotic cells have complex internal membranes, which enclose various organelles, including mitochondria and, in plant cells, chloroplasts. Mitochondria metabolize food through cellular respiration, and chloroplasts are the site of photosynthesis. Both mitochondria and chloroplasts are about the size of prokaryotic cells and contain similar prokaryote features. This leads some scientists to speculate that prokaryotic cells were involved in the evolution of eukaryotic cells.

In 1966, biologist Lynn Margulis proposed the endosymbiont theory. According to the **endosymbiont theory**, the ancestors of eukaryotic cells lived in association with prokaryotic cells. In some cases, prokaryotes even might have lived inside eukaryotes. Prokaryotes could have entered a host cell as undigested prey, or they could have been internal parasites. Eventually, the relationship between the cells became mutually beneficial, and the prokaryotic symbionts became organelles in eukaryotic cells. This theory explains the origin of chloroplasts and mitochondria, as illustrated in

Figure 14.17.

Evidence for the endosymbiont theory When Margulis first proposed the endosymbiont theory, many scientists were hesitant to accept it. There is evidence, however, that at least mitochondria and chloroplasts formed by endosymbiosis. For example, mitochondria and chloroplasts contain their own DNA. It is arranged in a circular pattern, just as it is in prokaryotic cells. Mitochondria and chloroplasts also have ribosomes that more closely resemble those in prokaryotic cells than those in eukaryotic cells. Finally, like prokaryotic cells, mitochondria and chloroplasts reproduce by fission, independent from the rest of the cell.

Though the endosymbiont theory is widely endorsed, it is important to understand that scientists do not know the early steps that led to the emergence of life or to its early evolution. It is unlikely that any traces of the first life will ever be found. What scientists do know is that the conditions on Earth shortly after it took shape allowed the precursors of life to form.

The evolution of life is better understood than how the first life appeared. Fossil, geologic, and biochemical evidence supports many of the proposed steps in life’s subsequent evolution. However, future discoveries might alter any or all of these steps. Scientists will continue to evaluate new evidence and test new theories in years to come.

Darwin’s Theory of Natural Selection

Developing the Theory of Natural Selection

When Charles Darwin, shown in Figure 15.1, boarded the HMS Beagle in 1831, the average person believed that the world was about 6,000 years old. Almost everyone, including the young Darwin, believed that animals and plants were unchanging. The concept of gradual change over time—and Darwin’s role in this concept— was still years away.

Darwin on the HMS Beagle The primary mission of the Beagle was to survey the coast of South America. In 1831, the Beagle set sail from England for Maderia and then proceeded to South America, as shown on the map in Figure 15.2. Darwin’s role on the ship was as naturalist and companion to the captain. His job was to collect biological and geological specimens during the ship’s travels. Darwin had a degree in theology from Christ’s College, Cambridge, though he previously had studied medicine and the sciences.

Over the course of the ship’s five-year voyage, Darwin made extensive collections of rocks, fossils, plants, and animals. He also read a copy of Charles Lyell’s Principles of Geology—a book proposing that Earth was millions of years old. This book influenced his thinking as he observed fossils of marine life at high elevation in the Andes, unearthed giant fossil versions of smaller living mammals, and saw how earthquakes could lift rocks great distances very quickly.

The Galápagos Islands In 1835, the Beagle arrived in the Galápagos Islands off the coast of South America.

Darwin was initially disappointed by the stark barrenness of these volcanic islands. But as he began to collect mockingbirds, finches, and other animals on the four islands he visited, he noticed that the different islands seemed to have their own, slightly different varieties of animals. These differences, however, only sparked a mere curiosity. He took little notice of the comment from the colony’s vice governor that the island origins of the giant tortoises could be identified solely by the appearance of the tortoises’ shells.

A few years after Darwin returned to England, he began reconsidering his observations. He took note of the work of John Gould, an ornithologist who was classifying the birds Darwin brought back from the Galápagos. Gould discovered that the Galápagos mockingbirds were separate species and determined that the finches of the Galápagos did not live anywhere else in South America. In fact, almost every specimen that Darwin had collected on the islands was new to European scientists.

These new species most closely resembled species from mainland South America, although the Galápagos and the mainland had different environments. Island and mainland species should not have resembled one another so closely unless, as Darwin began to suspect, populations from the mainland changed after reaching the Galápagos.

Darwin continued his studies Darwin hypothesized that new species could appear gradually through small changes in ancestral species, but he could not see how such a process would work. To understand it better, he turned to animal breeders—pigeon breeders in particular.

Different breeds of pigeons have certain distinctive traits that also are present in that breed’s offspring. A breeder can promote these traits by selecting and breeding pigeons that have the most exaggerated expressions of those traits. For example, to produce pigeons with fan-shaped tails, the breeder will breed pigeons with the most fan-shaped tails. Their offspring will tend to have the fantail of their parents.

Recall from Chapter 13 that this process is called selective breeding. Darwin called it artificial selection.

Artificial selection also occurs when developing new breeds of dogs or new strains of crop plants. Darwin inferred that if humans could change species by artificial selection, then perhaps the same process could work in nature. Further, Darwin thought that, given enough time, perhaps this process could produce new species.

Natural selection

While thinking about artificial selection, Darwin read an essay by the economist Thomas Malthus. The essay suggested that the human population, if unchecked, eventually would outgrow its food supply, leading to a competitive struggle for existence. Darwin realized that Malthus’s ideas could be applied to the natural world. He reasoned that some competitors in the struggle for existence would be better equipped for survival than others. Those less equipped would die. Here, finally, was the framework for a new theory about the origin of species.

Darwin’s theory has four basic principles that explain how traits of a population can change over time. First, individuals in a population show differences, or variations. Second, variations can be inherited, meaning that they are passed down from parent to offspring. Third, organisms have more offspring than can survive on available resources. The average cardinal, for example, lays nine eggs each summer. If each baby cardinal survived and reproduced just once, it would take only seven years for the first pair to have produced one million birds. Finally, variations that increase reproductive success will have a greater chance of being passed on than those that do not increase reproductive success. If having a fantail helps a pigeon reproduce successfully, future generations would include more pigeons with fan-shaped tails.

Darwin called his theory natural selection. He reasoned that, given enough time, natural selection could modify a population enough to produce a new species. Figure 15.3 shows how natural selection might modify a population of sunflowers.

The Origin of Species

Darwin had likely formulated his theory of natural selection by about 1840. Soon after, he began writing a multi-volume book compiling evidence for evolution and explaining how natural selection might provide a mechanism for the origin of species. Table 15.1 summarizes the principles of natural selection. He continued to compile evidence in support of his theory for many years. For example, he spent eight years studying relationships among barnacles. In 1858, Alfred Russel Wallace, another English naturalist, proposed a theory that was almost identical to Darwin’s theory of natural selection. Both men’s ideas were presented to the Linnean Society of London. One year later, Darwin published On the Origin of Species by Means of Natural Selection—a condensed version of the book he had started many years before.

In his book, Darwin used the term evolution only on the last page. Today, biologists use the term evolution to define cumulative changes in groups of organisms through time. Darwin’s theory of natural selection is not synonymous with evolution; it is a means of explaining how evolution works.

Evidence of Evolution

Support for Evolution

Darwin’s book On the Origin of Species demonstrated how natural selection might operate. The book also provided evidence that evolution has occurred on our planet. These are two different, though related, things. Darwin’s theory of natural selection is part of the larger theory of evolution. In science, a theory provides an explanation for how some aspect of the natural world operates. Theories explain available data and suggest further areas for experimentation. The **theory of evolution** states that all organisms on Earth have descended from a common ancestor.

The fossil record Fossils offer some of the most significant evidence of evolutionary change. Fossils provide a record of species that lived long ago. They show that ancient species share similarities with species that now live on Earth, as illustrated in Figure 15.4. Not all extinct fossils have modern counterparts and some ancient species—such as the horse-shoe crab—have remained virtually unchanged for millions of years. The fossil record is an important source of information for determining the ancestry of organisms and the patterns of evolution.

Though Darwin recognized the limitations of the fossil record, he predicted the existence of fossils intermediate in form between species. Today, scientists studying evolutionary relationships have found hundreds of thousands of transitional fossils that contain features shared by different species. For example, certain dinosaur fossils show feathers of modern birds and the teeth and bony tails of reptiles. Figure 15.5 shows an artist’s rendering of Archaeopteryx, one of the first birds. Archaeopteryx fossils provide evidence of characteristics that classify it as a bird, and also show that the bird retained several distinct dinosaur features.

Researchers consider two major classes of traits when studying transitional fossils: derived traits and ancestral traits. Derived traits are newly evolved features, such as feathers, that do not appear in the fossils of common ancestors. Ancestral traits, on the other hand, are more primitive features, such as teeth and tails, that do appear in ancestral forms. Transitional fossils provide detailed patterns of evolutionary change for the ancestors of many modern animals, including mollusks, horses, whales, and humans.

Comparative anatomy

Why do the vertebrate forelimbs shown in Figure 15.6 have different functions but appear to be constructed of similar bones in similar ways? Evolutionary theory suggests that the answer lies in shared ancestry.

Homologous structures

Anatomically similar structures inherited from a common ancestor are called homologous structures. Evolution predicts that an organism’s body parts are more likely to be modifications of ancestral body parts than they are to be entirely new features.

The limbs illustrated in Figure 15.6 move animals in different ways, yet they share similar construction. Bird wings and reptile limbs are another example. Though birds use their wings to fly and reptiles use their limbs to walk, bird wings and reptile forelimbs are similar in shape and construction, which indicates that they were inherited from a common ancestor. While homologous structures alone are not evidence of evolution, they are an example for which evolution is the best available explanation for the biological data.

Vestigial structures

In some cases, a functioning structure in one species is smaller or less functional in a closely related species. For example, most birds have wings developed for flight. Kiwis, however, have very small wings that cannot be used for flying. The kiwi wing is a kind of homologous structure called a vestigial structure. Vestigial structures are structures that are the reduced forms of functional structures in other organisms. Table 15.2 illustrates some vestigial structures in different species. **Evolutionary theory** predicts that features of ancestors that no longer have a function for that species will become smaller over time until they are lost.

Not all anatomically similar features are evidence of common ancestry. Analogous structures can be used for the same purpose and can be superficially similar in construction, but are not inherited from a common ancestor. As shown in Figure 15.7, the wing of an eagle and the wing of a beetle have the same function—they both enable the organism to fly—but they are constructed in different ways from different materials. While analogous structures do not indicate close evolutionary relationships, they do show that functionally similar features can evolve independently in similar environments.

Comparative embryology Vertebrate embryos provide more glimpses into evolutionary relationships. An embryo is an early, prebirth stage of an organism’s development. Scientists have found that vertebrate embryos exhibit homologous structures during certain phases of development but become totally different structures in the adult forms. The embryos shown in Figure 15.8, like all vertebrate embryos, have a tail and paired structures called pharyngeal pouches. In fish the pouches develop into gills. In reptiles, birds, and mammals, these structures become parts of the ears, jaws, and throats. Though the adult forms differ, the shared features in the embryos suggest that vertebrates evolved from a shared ancestor.

Comparative biochemistry

Scientific data also shows that common ancestry can be seen in the complex metabolic molecules that	many different organisms share. Cytochrome c is an enzyme that is essential for respiration and is highly conserved in animals. This means that despite slight variations in its amino acid sequence, the molecule	has changed very little over time.

**Evolutionary theory** predicts that molecules in species with a recent common ancestor should share certain ancient amino acid sequences. The more closely related the species are, the greater number of sequences will be shared. This predicted pattern is what scientists find to be true in cytochrome c. For example, as illustrated in Figure 15.9, the cytochrome c in the pig and in the monkey share more amino acid sequences with humans than the cytochrome c in birds shares with humans.

Scientists have found similar biochemical patterns in other proteins, as well as in DNA and RNA. DNA and RNA form the molecular basis of heredity in all living organisms. The fact that many organisms have the same complex molecules suggests that these molecules evolved early in the history of life and were passed on through the life-forms that have lived on Earth. Comparisons of the similarities in these molecules across species reflect evolutionary patterns seen in comparative anatomy and in the fossil record. Organisms with closely related morphological features have more closely related molecular features.

Geographic distribution

The distribution of plants and animals that Darwin saw during his South American travels first suggested evolution to Darwin. He observed that animals on the South American mainland were more similar to other South American animals than they were to animals living in similar environments in Europe. The South American mara, for example, inhabited a niche that was occupied by the rabbit in Europe. You can compare a mara and a rabbit in Figure 15.10. Darwin realized that the mara was more similar to other South American species than it was to the rabbit because it shared a closer ancestor with the South American animals.

Patterns of migration were critical to Darwin when he was developing his theory. Migration patterns explained why, for example, islands often have more plant diversity than animal diversity: the plants are more able to migrate from the closest mainland as seeds, either by wind or on the backs of birds. Since Darwin’s time, scientists have confirmed and expanded Darwin’s study of the distribution of plants and animals around the world in a field of study now called biogeography. Evolution is intimately linked with climate and geological forces, especially plate tectonics, which helps explain many ancestral relationships and geographic distributions seen in fossils and living organisms today.

Adaptation

The five categories discussed in the previous section—the fossil record, comparative anatomy, comparative embryology, comparative biochemistry, and geographic distribution—offer evidence for evolution.

Darwin drew on all of these except biochemistry—which was not well developed in his time—to develop his own theory of evolution by natural selection. At the heart of his theory lies the concept of adaptation.

Types of adaptation

An adaptation is a trait shaped by natural selection that increases an organism’s reproductive success. One way to determine how effectively a trait contributes to reproductive success is to measure fitness. Fitness is a measure of the relative contribution an individual trait makes to the next generation. It often is measured as the number of reproductively viable offspring that an organism produces in the next generation.

The better an organism is adapted to its environment, the greater its chances of survival and reproductive success. This concept explains the variations Darwin observed in the animals on the Galápagos Islands.

Because the finches were each adapted to their individual islands, they had variations in their beaks.

Camouflage

Some species have evolved morphological adaptations that allow them to blend in with their environments. This is called camouflage. Camouflage allows organisms to become almost invisible to predators, as shown in Figure 15.11. As a result, more of the camouflaged individuals survive and reproduce.

Mimicry Another type of morphological adaptation is mimicry. In mimicry, one species evolves to resemble another species. You might expect that mimicry would make it difficult for individuals in one species to find and breed with other members of their species, thus decreasing reproductive success. However, mimicry often increases an organism’s fitness. Mimicry can occur in a harmless species that has evolved to resemble a harmful species, such as the example shown in Figure 15.12. Sometimes mimicry benefits two harmful species. In both cases, the mimics are protected because predators can’t always tell the mimic from the animal it is mimicking, so they learn to avoid them both.

Antimicrobial resistance Species of bacteria that originally were killed by penicillin and other antibiotics have developed drug resistance. For almost every antibiotic, at least one species of resistant bacteria exists. One unintended consequence of the continued development of antibiotics is that some diseases, which were once thought to be contained, such as tuberculosis, have reemerged in more harmful forms.

Consequences of adaptations

Not all features of an organism are necessarily adaptive. Some features might be consequences of other evolved characteristics. Biologists Stephen Jay Gould and Richard Lewontin made this point in 1979 in a paper claiming that biologists tended to overemphasize the importance of adaptations in evolution.

Spandrel example

To illustrate this concept, they used an example from architecture. Building a set of four arches in a square to support a dome means that spaces called spandrels will appear between the arches, as illustrated in Figure 15.13. Because spandrels are often decorative, one might think that spandrels exist for decoration. In reality, they are an unavoidable consequence of arch construction. Gould and Lewontin argued that some features in organisms are like spandrels because even though they are prominent, they do not increase reproductive success. Instead, they likely arose as an unavoidable consequence of prior evolutionary change.

Human example

A biological example is the helplessness of human babies. Humans give birth at a much earlier stage than other primates, leading to the need for increased care. While scientists thought at one time that early birth might serve an adaptive purpose—for instance, it might increase parental attention and encourage more learning, which might lead to increased fitness—many scientists now think that the helplessness of human babies is merely a consequence of the evolution of big brains and upright posture. To walk upright, humans need narrow pelvises, which means that babies’ heads must be small enough to fit through the pelvic opening.

Shaping Evolutionary Theory

Mechanisms of Evolution

Darwin’s theory of natural selection remains a central theme in evolution. It explains how organisms adapt to their environments and how variations can give rise to adaptations within species. Scientists now know, however, that natural selection is not the only mechanism of evolution. Studies from population genetics and molecular biology have led to the development of evolutionary theory. At the center of this is the understanding that evolution occurs at the population level, with genes as the raw material.

Population genetics

At the turn of the twentieth century, genes had not been discovered. However, the allele was understood to be one form of an inherited character trait, such as eye color, that gets passed down from parent to offspring. Scientists didn’t understand why dominant alleles wouldn’t simply swamp recessive alleles in a population.

In 1908, English mathematician Godfrey Hardy and German physician Wilhelm Weinberg independently came up with the same solution to this problem. They showed mathematically that evolution will not occur in a population unless allelic frequencies are acted upon by forces that cause change. In the absence of these forces, the allelic frequency remains the same and evolution doesn’t occur. According to this idea, which is now known as the **Hardy-Weinberg principle**, when allelic frequencies remain constant, a population is in genetic equilibrium. This concept is illustrated in Figure 15.14.

To illustrate the Hardy-Weinberg principle, consider a population of 100 humans. Forty of these people are homozygous dominant for earlobe attachment (EE). Another 40 people are heterozygous for earlobe attachment (Ee). Twenty people are homozygous recessive (ee). In the 40 homozygous dominant people, there are 80 E alleles (2 E alleles × 40), and in the 20 homozygous recessive people there are 40 e alleles (2 e alleles × 20). The heterozygous people have 40 E alleles and 40 e alleles. Summing the alleles, we have 120 E alleles and 80 e alleles for a total of 200 alleles. The E allele frequency is 120/200, or 0.6. The e allele frequency is 80/200, or 0.4.

The **Hardy-Weinberg principle** states that the allele frequencies in populations should be constant. This often is expressed as p + q = 1. For our example, p can represent the E allele frequency and q can represent the e allele frequency.

Squaring both sides of the equation yields the new equation

p2 + 2pq + q2 = 1. This equation allows us to determine the equilibrium frequency of each genotype in the population: homozygous dominant (p2), heterozygous (2pq), and homozygous recessive (q2). From the above example, p = 0.6, and q = 0.4, so (0.6)(0.6) + 2(0.6)(0.4) + (0.4)(0.4) = 1.

In the example population, the equilibrium frequency for homozygous dominant will be 0.36, the equilibrium frequency of heterozygous will be 0.48, and the equilibrium frequency of homozygous recessive will be 0.16. Note that the sum of these frequencies equals one.

Conditions According to the **Hardy-Weinberg principle**, a population in genetic equilibrium must meet five conditions—there must be no genetic drift, no gene flow, no mutation, mating must be random, and there must be no natural selection. These are listed in Table 15.3. Populations in nature might meet some of these requirements, but hardly any population meets all five conditions for long periods of time. If a population is not in genetic equilibrium, at least one of the five conditions has been violated. These five conditions are known mechanisms of evolutionary change. Of these, only natural selection is thought to provide adaptive advantages to a population, and only natural selection acts on an organism’s phenotype.

Genetic drift

Any change in the allelic frequencies in a population that is due to chance is called genetic drift. Recall from Chapter 10 that for simple traits only one of a parent’s two alleles passes to the offspring, and that this allele is selected randomly through independent assortment. In large populations, enough alleles “drift” to ensure that the allelic frequency of the entire population remains relatively constant from one generation to the next. In smaller populations, however, the effects of genetic drift become more pronounced, and the chance of losing an allele becomes greater.

Founder effect

The **founder effect** is an extreme example of genetic drift. The **founder effect** can occur when a small sample of a population settles in a location separated from the rest of the population.

Because this sample is a random subset of the original population, the sample population carries a random subset of the population’s genes. Alleles that were uncommon in the original population might be common in the new population, and the offspring in the new population will carry those alleles. Such an event can result in large genetic variations in the separated populations.

The **founder effect** is evident in the Amish and Mennonite communities in the United States in which the people rarely marry outside their own communities. The Old Order Amish have a high frequency of six-finger dwarfism. All affected individuals can trace their ancestry back to one of the founders of the Order.

Bottleneck Another extreme example of genetic drift is a bottleneck, which occurs when a population declines to a very low number and then rebounds. The gene pool of the rebound population often is genetically similar to that of the population at its lowest level, that is, it has reduced diversity. Researchers think that cheetahs in Africa experienced a bottleneck 10,000 years ago, and then another one about 100 years ago. Throughout their current range, shown in Figure 15.15, cheetahs are so genetically similar that they appear inbred. Inbreeding decreases fertility, and might be a factor in the potential extinction of this endangered species.

Gene flow

A population in genetic equilibrium experiences no gene flow. It is a closed system, with no new genes entering the population and no genes leaving the population. In reality, few populations are isolated. The random movement of individuals between populations, or migration, increases genetic variation within a population and reduces differences between populations.

Nonrandom mating

Rarely is mating completely random in a population. Usually, organisms mate with individuals in close proximity. This promotes inbreeding and could lead to a change in allelic proportions favoring individuals that are homozygous for particular traits.

Mutation Recall from Chapter 12 that a mutation is a random change in genetic material. The cumulative effect of mutations in a population might cause a change in allelic frequencies, and thus violate genetic equilibrium. Though many mutations cause harm or are lethal, occasionally a mutation provides an advantage to an organism. This mutation will then be selected for and become more common in subsequent generations. In this way, mutations provide the raw material upon which natural selection works.

Natural selection The **Hardy-Weinberg principle** requires that all individuals in a population be equally adapted to their environment and thus contribute equally to the next generation. As you have learned, this rarely happens. Natural selection acts to select the individuals that are best adapted for survival and reproduction. Natural selection acts on an organism’s phenotype and changes allelic frequencies. Figure 15.16 shows three main ways natural selection alters phenotypes: through stabilizing selection, directional selection, and disruptive selection. A fourth type of selection, sexual selection, also is considered a type of natural selection.

Stabilizing selection

The most common form of natural selection is stabilizing selection. It operates to eliminate extreme expressions of a trait when the average expression leads to higher fitness. For example, human babies born with below-normal and above-normal birth weights have lower chances of survival than babies born with average weights.

Therefore, birth weight varies little in human populations.

Directional selection

If an extreme version of a trait makes an organism more fit, directional selection might occur. This form of selection increases the expression of the extreme versions of a trait in a population. One example is the evolution of moths in industrial England. The peppered moth has two color forms, or morphs, as shown in Figure 15.17. Until the mid 1850s, nearly all peppered moths in England had light-colored wings. Beginning around 1850, however, dark moths began appearing. By the early 1900s, nearly all peppered moths were dark. Why? Industrial pollution favored the dark-colored moths at the expense of the light-colored moths. The darker the moth, the more it matched the sooty background of its tree habitat, and the harder it was for predators to see. Thus, more dark moths survived, adding more genes for dark color to the population. This conclusion was reinforced in the mid-1900s when the passage of air pollution laws led to the resurgence of light-colored moths. This phenomenon is called industrial melanism.

Directional selection also can be seen in Galápagos finches. For three decades in the latter part of the twentieth century, Peter and Rosemary Grant studied populations of these finches. The Grants found that during drought years, food supplies dwindled and the birds had to eat the hard seeds they normally ignored. Birds with the largest beaks were more successful in cracking the tough seed coating than were birds with smaller beaks. As a result, over the duration of the drought, birds with larger beaks came to dominate the population.

In rainy years, however, the directional trend was reversed, and the population’s average beak size decreased.

Disruptive selection

Another type of natural selection, disruptive selection, is a process that splits a population into two groups. It tends to remove individuals with average traits but retain individuals expressing extreme traits at both ends of a continuum. Northern water snakes, illustrated in Figure 15.18, are an example.

Snakes living on the mainland shores inhabit grasslands and have mottled brown skin. Snakes inhabiting rocky island shores have gray skin. Each is adapted to its particular environment. A snake with intermediate coloring would be disadvantaged because it would be more visible to predators.

Sexual selection Charles Darwin recognized another type of natural selection that he called sexual selection. This type of selection often operates in populations where males and females differ significantly in appearance. Usually in these populations, males are the largest and most colorful of the group. The bigger the tail of a male peacock, as shown in Figure 15.19, the more attractive the bird is to females. Males also evolve threatening characteristics that intimidate other males; this is common in species, such as elk or deer, where the male keeps a harem of females.

Darwin was intrigued by sexual selection. He wondered why some qualities of sexual attractiveness appeared to be the opposite of qualities that might enhance survival. For example, the peacock’s tail, while attracting females, is large and cumbersome, and it might make the peacock a more likely target for predators. Though some modern scientists think that sexual selection is not a form of natural selection, others think that sexual selection follows the same general principle: brighter colors and bigger bodies enhance reproductive success, whatever the chances are for long-term survival.

Reproductive Isolation

Mechanisms of evolution—genetic drift, gene flow, nonrandom mating, mutation, and natural selection—violate the Hardy-Weinberg principle. To what extent each mechanism contributes to the origin of new species is a major topic of debate in evolutionary science today.

Most scientists define speciation as the process whereby some members of a sexually reproducing population change so much that they can no longer produce fertile offspring with members of the original population. Two types of reproductive isolating mechanisms prevent gene flow among populations. **Prezygotic isolating mechanisms** operate before fertilization occurs. Postzygotic isolating mechanisms operate after fertilization has occurred to ensure that the resulting hybrid remains infertile.

Prezygotic isolation **Prezygotic isolating mechanisms** prevent reproduction by making fertilization unlikely. These mechanisms prevent genotypes from entering a population’s gene pool through geographic, ecological, behavioral, or other differences. For example, the eastern meadowlark and the western meadowlark, pictured in Figure 15.20, have overlapping ranges and are similar in appearance. These two species, however, use different mating songs and do not interbreed. Time is another factor in maintaining a reproductive barrier. Closely related species of fireflies mate at different times of night, just as different species of trout live in the same stream but breed at different times of the year.

Postzygotic isolation

When fertilization has occurred but a hybrid offspring cannot develop or reproduce, postzygotic isolation has occurred. Postzygotic isolating mechanisms prevent offspring survival or reproduction. A lion and a tiger are considered separate species because even though they can mate, the offspring—a liger, shown in Figure 15.21—is sterile.

Speciation

For speciation to occur, a population must diverge and then be reproductively isolated. Biologists usually recognize two types of speciation: allopatric and sympatric.

Allopatric speciation

In allopatric speciation, a physical barrier divides one population into two or more populations. The separate populations eventually will contain organisms that, if enough time has passed, will no longer be able to breed successfully with one another. Most scientists think that allopatric speciation is the most common form of speciation. Small subpopulations isolated from the main population have a better chance of diverging than those living within it. This was the conclusion of the biologist Ernst Mayr, who argued as early as the 1940s that geographic isolation was not only important but required for speciation.

Geographic barriers can include mountain ranges, channels between islands, wide rivers, and lava flows. The Grand Canyon, pictured in Figure 15.22, is an example of a geographic barrier. The Kaibab squirrel is found on the canyon’s north rim, while the Abert squirrel lives on the south rim. Scientists think that the two types of squirrels diverged from an ancestral species and today are reproductively isolated by the width of the canyon. While these animals officially belong to the same species, they demonstrate distinct differences and, in time, they might diverge enough to be classified as separate species.

Sympatric speciation

In sympatric speciation, a species evolves into a new species without a physical barrier. The ancestor species and the new species live side by side during the speciation process. Evidence of sympatric evolution can be seen in several insect species, including apple maggot flies, which appear to be diverging based on the type of fruit they eat. Scientists think that sympatric speciation happens fairly frequently in plants, especially through polyploidy. Recall from Chapter 10 that polyploidy is a mutation that increases a plant’s chromosome number. As a result, the plant is no longer able to interbreed with the main population.

Patterns of evolution

Many details of the speciation process remain unresolved. Relative to the human life span, speciation is a long process, and first-hand accounts of speciation are expected to be rare. However, evidence of speciation is visible in patterns of evolution.

Adaptive radiation More than 300 species of cichlid fish, six of which are illustrated in Figure 15.23, once lived in Africa’s Lake Victoria. Data shows that these species diverged from a single ancestor within the last 14,000 years. This is a dramatic example of a type of speciation called adaptive radiation. Adaptive radiation, also called divergent evolution, can occur in a relatively short time when one species gives rise to many species in response to the creation of new habitat or another ecological opportunity. Likely, a combination of factors caused the explosive radiation of the cichlids, including the appearance of a unique double jaw, which allowed these fish to exploit various food sources. Adaptive radiation often follows large-scale extinctions. Adaptive radiation of mammals at the beginning of the Cenozoic following the extinction of dinosaurs likely produced the diversity of mammals visible today.

Coevolution

Many species evolve in close relationship with other species. The relationship might be so close that the evolution of one species affects the evolution of other species. This is called coevolution. Mutualism is one form of coevolution. Recall from Chapter 2 that mutualism occurs when two species benefit each other. For example, comet orchids and the moths that pollinate them have coevolved an intimate dependency: the foot-long flowers of this plant perfectly match the foot-long tongue of the moth, shown in Figure 15.24.

In another form of coevolution, one species can evolve a parasitic dependency on another species. This type of relationship is often called a coevolutionary arms race. The classic example is a plant and an insect pathogen that is dependent on the plant for food. The plant population evolves a chemical defense against the insect population. The insects, in turn, evolve the biochemistry to resist the defense. The plant then steps up the race by evolving new defenses, the insect escalates its response, and the race goes on. Complex coevolutionary relationships like these might reflect thousands of years of evolutionary interaction.

Convergent evolution

Sometimes unrelated species evolve similar traits even though they live in different parts of the world. This is called convergent evolution. Convergent evolution occurs in environments that are geographically far apart but that have similar ecology and climate. The mara and rabbit discussed in Section 15.2 provide an example of convergent evolution. The mara and the rabbit are unrelated, but because they inhabit similar niches, they have evolved similarities in morphology, physiology, and behavior. Table 15.4 shows examples of convergent evolution between Australian marsupials and the placental mammals on other continents.

Rate of speciation Evolution is a dynamic process. In some cases, as in a coevolutionary arms race, traits might change rapidly. In other cases, traits might remain unchanged for millions of years. Most scientists think that evolution proceeds in small, gradual steps.

This is a theory called gradualism. A great deal of evidence favors this theory. However, the fossil record contains instances of abrupt transitions. For example, certain species of fossil snails looked the same for millions of years, then the shell shape changed dramatically in only a few thousand years. The **theory of punctuated equilibrium** attempts to explain such abrupt transitions in the fossil record.

According to this theory, rapid spurts of genetic change cause species to diverge quickly; these periods punctuate much longer periods when the species exhibit little change.

The two theories for the tempo of evolution are illustrated in Figure 15.25. The tempo of evolution is an active area of research in evolutionary theory today. Does most evolution occur gradually or in short bursts? Fossils can show only morphological structures. Changes in internal anatomy and function go unnoticed. How, then, does one examine the past for evidence?

The question of the tempo of evolution is an excellent illustration of how science works. Solving this puzzle requires insights from a variety of disciplines using a variety of methods. Like many areas of scientific endeavor, evolution offers a complex collection of evidence, and it does not yield easily to simple analysis.

Primates

Characteristics of Primates

Humans, apes, monkeys, and lemurs belong to a group of mammals called primates. Though primates are highly diverse, they share some general features. Some primates have a high level of manual dexterity, which is the ability to manipulate or grasp objects with their hands. They usually also have keen eyesight and long, highly movable arms. Compared to other animals, they have large brains. The primates with the largest brains, which includes humans, have the capacity to reason.

Manual dexterity Primates are distinguished by their flexible hands and feet. All primates typically have five digits on each hand and foot; as you know, humans have fingers and toes. Most have flat nails and sensitive areas on the ends of their digits. The first digits on most primates’ hands are opposable, and the first digit on many primates’ feet are opposable. An opposable first digit, either a thumb or a great toe, is set apart from the other digits. This digit can be brought across the palm or foot so that it touches or nearly touches the other digits. This action allows the primate to grasp an object in a powerful grip. Some primates also have lengthened first digits that provide added dexterity. Figure 16.1 shows a monkey using its opposable thumbs to grasp its food.

Senses

Though there are exceptions, primates rely more on vision and less on their sense of smell than other animals do. Their eyes, protected by a bony eye socket, are on the front of their face. This creates overlapping fields of vision, often called binocular vision. Forward-looking eyes allow for a greater field of depth perception, and enable primates to judge relative distance and movement of an object.

Most primates are diurnal, which means they are active during the day. Because these primates are active in daylight, most also have color vision. Primates that are nocturnal are active at night. They only see in black and white. An increased sense of vision is generally accompanied by a decreased sense of smell. Their snouts are smaller and their faces tend to be flattened, which increases the degree of binocular vision. Their teeth are reduced in size and usually are unspecialized, meaning that they are suitable for many different types of diets.

Locomotion Another characteristic of primates is their flexible bodies. Primates have limber shoulders and hips, and primarily rely on hind limbs for locomotion. Most primates live in trees and have developed an extraordinary ability to move easily from branch to branch. When on the ground, all primates except humans walk on all four limbs. Many primates can walk upright for short distances and many have a more upright posture compared to four-legged animals.

Complex brain and behaviors Primates tend to have large brains in relation to their body size. Their brains have fewer areas devoted to smell and more areas devoted to vision. They also tend to have larger areas devoted to memory and coordinating arm and leg movement.

Along with larger brains, many primates have problem-solving abilities and well-developed social behaviors, such as grooming and communicating. Most diurnal primates spend a great deal of time socializing by spending time grooming each other. In addition, many primates have complex ways of communicating to each other, which include a wide range of facial expressions.

Reproductive rate Most primates have fewer offspring than other animals. Usually, primates give birth to one offspring at a time. Compared to other mammals, pregnancy is long, and newborns are dependent on their mothers for an extended period of time. For many primates, this time period allows for the increased learning of complex social interactions. A low reproductive rate, the loss of tropical habitats, and human predation has threatened some primate populations. Many are endangered. Figure 16.2 illustrates the tropical areas of the world, such as Africa and Southeast Asia, where primates live.

Primate Groups

Primates are a large, diverse group of more than 200 living species. Examine Figure 16.3 as you read about this diverse group. Most primates are arboreal, or tree-dwelling. Arboreal primates live in the world’s tropical and subtropical forests. Primates that lived on the ground are considered terrestrial.

Scientists generally classify primates into two subgroups based on characteristics of their noses, eyes, and teeth. The strepsirrhines, or “wet-nosed” primates, are considered the earliest, most basic primates. The lemurs are the most numerous of this group. The second group consists of the haplorhines, or “drynosed” primates. The haplorhines include the anthropoids, or humanlike primates, as well as a unique primate called the tarsier.

Strepsirrhines

Table 16.1 lists characteristics of some strepsirrhine groups. Strepsirrhines have large eyes and ears. They are the only primates that rely predominantly on smell for hunting and social interaction. They only live in tropical Africa and Asia. Most strepsirrhines, such as lemurs, sifakas, indris, and aye-ayes (I iz) are only found in Madagascar and nearby islands. Madagascar drifted from the African mainland as these animals evolved, and scientists hypothesize that the ancestors of lemurs migrated to the island on rafts made of leaves. There they were reproductively isolated, which resulted in their diversification.

Most small lemurs are nocturnal and solitary. Only a few large species, such as the sifaka shown in Figure 16.4, are diurnal and social.

The indri is unique because it does not have a tail, unlike most lemurs that use their bushy tails for balance as they jump from branch to branch. Lorises are similar to lemurs but are found primarily in India and Southeast Asia. Galagos, also called bushbabies, are found only in Africa.

Haplorhines

The second group of primates is a much larger group. The haplorhines include tarsiers, monkeys, and apes. The apes, in turn, include gibbons, orangutans, gorillas, chimpanzees, and humans.

The tarsier is found only on Borneo and the Philippines. It is a small, nocturnal creature with large eyes. It has the ability to rotate its head 180 degrees like an owl. It lives in trees, where it climbs and leaps among the branches. The tarsier shares characteristics with both lemurs and monkeys. Scientists once classified it with the lemurs, but new evidence suggests that it is more closely related to anthropoids, which makes it part of the haplorhine group.

Anthropoids are generally larger than strepsirrhines, and they have large brains relative to their body size. They are more likely to be diurnal, with eyes adapted to daylight and sometimes to color. Anthropoids also have more complex social interactions. They tend to live longer than lemurs and other strepsirrhines. The anthropoids are split into the New World monkeys and the Old World monkeys. “New World” refers to the Americas; “Old World” refers to Africa, Asia, and Europe. New World monkeys are the only monkeys that live in the Americas.

New World monkeys The New World monkeys are a group of about 60 species of arboreal monkeys that inhabit the tropical forests of Mexico, Central America, and South America. New World monkeys include the marmosets and tamarins. These are among the smallest and most unique primates. Neither species has fingernails or opposable digits.

The New World monkeys also include the squirrel monkeys, spider monkeys, and capuchin monkeys. Some of these monkeys have opposable digits and most are diurnal and live together in social bands. Most are also distinguished by their prehensile tails. A prehensile tail functions like a fifth limb. It can grasp tree branches or other objects and support a monkey’s weight, like that shown in Figure 16.5.

Old World monkeys Old World monkeys live in a wide variety of habitats throughout Asia and Africa, from snow-covered mountains in Japan to arid grasslands in Africa. Some Old World monkeys live in Gibraltar, which is located at the southern tip of Spain. There are about 80 species in this group, including macaques and baboons in one subgroup, and colobus and proboscis monkeys in another. Old World monkeys are similar to New World monkeys in many ways.

They are diurnal and live in social groups. However, their noses tend to be narrower and their bodies are usually larger. They also spend more time on the ground. None have prehensile tails, and some have no tails. Most Old World monkeys have opposable digits.

Apes

Only a handful of ape species exist today. Apes generally have larger brains in proportion to their body size than monkeys. They also have longer arms than legs, barrel-shaped chests, no tails, and flexible wrists. They are often highly social and have complex vocalizations.

They are classified into two subcategories: the lesser apes, which include the gibbons and siamangs, and the great apes, which include orangutans, gorillas, chimpanzees, and humans.

Lesser apes The Asian gibbons and their close relatives, the larger siamangs, are the arboreal gymnasts of the ape family. Though they have the ability to walk on either two or four legs like all great apes, they generally move from branch to branch using a hand-over-hand swinging motion called brachiation. This motion, as shown in Figure 16.6, enables an adult gibbon to move almost 3 m in one swing.

Great apes Orangutans are the largest arboreal primates and the only great ape species that lives exclusively in Asia. Orangutans are large enough that the males are often more comfortable on the ground, though they are not efficient walkers. Female orangutans give birth once every eight years and nurse their young for up to six years. A male orangutan with prominent cheek pads and female orangutan with her offspring are shown in Figure 16.7.

The gorillas are the largest of the primates. Like all great apes, they are predominantly terrestrial animals. They walk on all four limbs, supporting themselves by their front knuckles. Also, like other great apes, they use sticks as simple tools in the wild, and some living in captivity have been taught to recognize characters and numbers.

Chimpanzees and their close relatives, the bonobos, are also knuckle-walkers. They have well-developed communication systems, such as body positions and gestures, and social behavior, and they live in a wide variety of habitats. They are more like humans in their physical structure and behavior than any other primate. The bonobo, shown in Figure 16.8, is slightly smaller than the chimpanzee. It was once called the “pygmy chimpanzee,” but it now is considered a separate species.

Humans are included in the great ape family. They are then classified in a separate subcategory of hominids called hominins. Hominins are humanlike primates that appear to be more closely related to present-day humans than they are to present-day chimpanzees and bonobos. Though many species of hominins have existed on Earth, only one species—the group to which you belong—survives today. The diagram in Figure 16.9 illustrates evolutionary relationships among primates.

Primate Evolution

Most primates today are arboreal. Prehensile tails, long limbs, binocular vision, brachiation, and opposable digits are traits that help them take full advantage of their forest environments.

Arboreal adaptation

Some scientists suggest that primates evolved from ground-dwelling animals that searched for food in the top branches of forest shrubbery. They then evolved into additional food-gathering niches in trees. For example, the flexible hand with its opposable digits evolved not to grasp tree branches but to catch insects. Other scientists suggest that the rise of flowering plants provided new niche opportunities, and that arboreal adaptations allowed primates to take advantage of the fruits and flowers of trees.

Primate ancestors

Genetic data suggests that the first primates probably lived about 85 mya, when dinosaurs still roamed Earth. However, the earliest primate fossils do not appear in the fossil record until the beginning of the Eocene, about 60 mya. One of the earliest fossil primates, called Altiatlasius, was a small, nocturnal animal that ate insects and fruits using its hands and feet for grasping. It might have resembled the tiny tree shrew in Figure 16.10, but it had some features similar to those of lemurs today. Learn more about the early evolution of primates in Data Analysis Lab 16.1.

Diverging primates Lemurlike primates were widespread by about 50 mya, and many species existed on all continents except Australia and Antarctica. Sometime around 50 mya, and possibly earlier, the anthropoids diverged from the tarsiers; this might have occurred in Asia, where the tarsiers are found today. The earliest anthropoids leaped less and walked more than the strepsirrhines and tarsiers, but they were still tiny and their brains were still small. By the end of the Eocene, 30–35 mya, the anthropoids had diverged and spread widely.

Displacement

Many early strepsirrhines appear to have become extinct by the end of the Eocene. This might have been caused by a change in climate. Many major geological events took place at the end of the Eocene and temperatures became cooler. Or it could have been caused by the divergence of the anthropoids. The anthropoids of this time generally were larger and had bigger brains than the strepsirrhines. Thus, the anthropoids might have outcompeted some of the strepsirrhines for resources. This idea is supported by the observation that today the nocturnal strepsirrhines do not interact with the diurnal anthropoids when the habitats of these two groups overlap.

Monkeys

The end of the Eocene also saw the appearance of the monkeys. Early monkeys had larger brains than their anthropoid ancestors, and their eyes were more forward-looking. Their snouts were less pointed and they relied less on smell. Scientists hypothesize that the New World monkeys diverged from the line that gave rise to the Old World monkeys sometime between 35 and 25 mya in Africa. While the Old World monkeys continued to evolve in Africa, the New World monkeys developed distinct characteristics in South America. By this time, Africa and South America had separated into two continents. How, then, did the New World monkeys arrive in South America?

Journey to South America Many scientists hypothesize that the New World monkeys evolved from an isolated group of ancestral anthropoids that somehow drifted to South America from Africa, perhaps on rafts of vegetation and soil, much like how the ancestors of lemurs drifted to Madagascar from the African mainland. Because two thousand miles is a long distance for a colony of monkeys to drift, some scientists suggest that the New World monkeys might have diverged from the anthropoid lineage and made their journey millions of years earlier when sea levels were lower and the continents were closer.

Aegyptopithecus

In Africa and Asia, the anthropoids continued to evolve. Many anthropoid fossils have been found at a site in present-day Egypt called the Fayum basin. Now a desert, the Fayum was predominantly tropical when dozens of anthropoid species lived there 36–31 mya. The largest among them was Aegyptopithecus, often called the dawn ape. Some scientists hypothesize that this arboreal animal, which was about the size of a domestic cat, was ancestral to the apes. It might have been part of the anthropoid line that split from the Old World monkeys and might have given rise to orangutans, gorillas, chimpanzees, and humans.

Hominoids to Hominins

Hominoids

Hominoids include all nonmonkey anthropoids— the living and extinct gibbons, orangutans, chimpanzees, gorillas, and humans. The fossil transition from early anthropoid to ape is not clear; very few fossils from the late Oligocene exist. The earliest hominoid fossils appear in the fossil record only about 25 mya at the beginning of the Miocene. These hominoids retained some ancestral primate features. For example, most had bodies adapted for brachiation. There is evidence that they had relatively large brains and had shoulders and hips that moved freely, and some might even have had the ability to stand on two legs.

Scientists use fossils to help them determine when ancestral hominoids diverged into the hominoids that exist today. But because the fossil record for hominoids is so sparse, scientists also turn to biochemical data to help them in this task. By comparing the DNA of living hominoid species, researchers conclude that gibbons likely diverged first from an ancestral anthropoid, followed by orangutans, gorillas, chimpanzees and bonobos, and, finally, humans. Figure 16.11 shows the potential divergence of these species. Chimpanzees and bonobos are the closest living relatives to humans. All three share at least 96 percent of their DNA sequences.

Hominoid characteristics Hominoids are the largest of the primates, and they have the largest brain size in relation to their body size. They tend to have broad pelvises, long fingers, no tail, and flexible arm and shoulder joints. They also have semi-upright or upright posture, and, except for hominins, their arms are longer than their legs. Their teeth are less specialized than those of other animals, and their molars have a distinctive pattern that scientists use to distinguish hominoid fossils from other primate fossils.

Hominoid biogeography During the Miocene (24–5 mya), the world’s climate became warmer and drier. As a result, tropical rain forests in Africa began to shrink. Many new animals, including new hominoids, evolved as they adapted to the changing environments. Between about 23 and 14 mya, perhaps as many as 100 hominoid species existed. Early hominoids were more diverse than the modern apes, and they migrated from Africa to Europe and Asia.

Proconsul

The best-known hominoid fossils, and some of the oldest, are those from the genus Proconsul. Figure 16.12 shows a fossil skull of one Proconsul species discovered by Mary Leakey in Kenya in 1948. This Proconsul species generally had the smallest brains of the hominoids. Most had freely moving arms and legs, and while they lived predominantly in trees, some might have had the ability to walk upright. Some scientists think that this Proconsul species is a human ancestor, but others suggest that one of the European hominoids—whose fossils are in some ways more humanlike than Proconsul—might have returned to Africa at the end of the Miocene and given rise to the human line.

Hominins

The lineage that most likely led to humans split off from the other African apes sometime between 8 and 5 mya. The hominins include humans and all their extinct relatives. These extinct relatives are more closely related to humans than to chimpanzees. The time line in Figure 16.13 highlights some important hominin discoveries.

Hominin characteristics Hominins have bigger brains than other hominoids, with more complexity in parts of the brain where high-level thought occurs. The hominin face is thinner and flatter than that of other hominoids. Hominin teeth are also smaller. With lengthened thumbs and more flexible wrists, hominins have high manual dexterity. Hominins also are bipedal, which means that they can walk upright on two legs.

Examine Figure 16.14, which illustrates anatomical differences in a quadruped and a biped. When becoming bipedal, hominins developed a fully upright stance, shortened arms, restructured pelvic bones and foot bones, and a change in the position of the head on the spinal cord. In quadrupedal animals, or those that walk on all four limbs, the foramen magnum—the hole in the skull where the spine extends from the brain—is located at the back of the skull. In hominins, it is positioned at the base of the skull.

Disadvantages of bipedalism Bipedalism is not necessarily more efficient than quadrupedalism. Bipedal individuals are easier for predators to see, they might not run as fast, and bipedalism puts greater strain on the hips and back. Also, an upright posture defies gravity and therefore appears to require more energy. Why, then, did hominins become bipedal when their ancestors were so well adapted to life in the trees?

Advantages of bipedalism The African landscape was changing during the period when hominins evolved, and many scientists suggest that bipedalism was an adaptation to the new savanna environment. In this scenario, food resources were sparse and far apart. By walking upright, individuals could travel long distances more efficiently than those walking on four limbs. They could also see food sources more easily. Walking upright for long distances might also have reduced the total area of the body exposed to sunlight and increased the area exposed to cooling winds.

Another hypothesis suggests that bipedal hominins were better able to carry objects or use their hands for other purposes. Still another hypothesis suggests that an upright posture would have helped hominins reach fruit on low branches as they traveled from tree to tree.

Why bipedalism? There is no single answer to the question of bipedalism. A changing environment might have played only a minor role. While grasslands were becoming more prevalent, it is likely that pockets of forest still existed, and there is evidence that some of the early bipedal hominins lived in woodlands. Some scientists have suggested that the most successful hominins might have been those that evolved on the edge of the forest and savanna. The earliest hominins could have slept in trees and ventured out to the savanna in search of food. In this view, adaptability to a range of environments—seen so clearly in humans today—might have its origin in the hominin use of forest and grassland.

Hominin fossils Bipedalism evolved before many other hominin traits, and it is often used to identify hominin fossils. The earliest fossils of species that show some degree of bipedalism are 6–7 million years old. The first hominins that were truly bipedal, however, were the australopithecines.

Australopithecines lived in the east-central and southern part of Africa between 4.2 and 1 mya. They were small—the males were only about 1.5 m tall—and they had apelike brains and jaws. However, their teeth and limb joints were humanlike.

Taung baby

The anthropologist Raymond Dart (1893-1988) discovered the first australopithecine fossil, the “Taung baby,” in Africa in 1926.

He called the species Australopithecus africanus, meaning “southern ape from Africa.” A. africanus likely lived between 3.3 and 2.3 mya. The placement of the foramen magnum in the skull of the Taung baby, shown in Figure 16.15, convinced Dart that A. africanus was bipedal. Not everyone agreed because A. africanus had a small brain. Some scientists thought that larger brains evolved before bipedalism. The question continued to be debated for many years, even after the discovery of other

African australopithecine fossils such as A. bosei and A. robustus that indicated bipedalism and small brains.

Lucy In 1974 in Kenya, the anthropologist Donald Johanson discovered an australopithecine skeleton that helped resolve the debate. Lucy is one of the most complete australopithecine fossils ever found. She was a member of the species A. afarensis, which lived between 4 and 2.9 mya.

Lucy was about the size of a chimpanzee. She had the typical australopithecine skull and small brain, and her arms were still somewhat long in proportion to her legs. She also had finger bones that were more curved than those of modern humans, which indicates that she was capable of arboreal activity. But her hip and knee joints were humanlike, and it was clear that she walked upright. A few years later, Mary Leakey uncovered further evidence that australopithecines were bipedal when she discovered fossilized australopithecine footprints. Lucy’s skeleton and the footprints of her relatives are illustrated in Figure 16.16.

Mosaic pattern

Like other hominin fossils, Lucy and her relatives show a patchwork of human and apelike traits. In this way, they follow a mosaic pattern of evolution. Mosaic evolution occurs when different body parts or behaviors evolve at different rates. For example, hominins developed the ability to walk upright nearly two million years before they developed modern flat faces and larger brains.

Hominin evolution

Within the last 30 years, scientists have discovered many more early hominin fossils. Some defy characterization and have led to new genus designations. Scientists have estimated that Kenyanthropus platyops, for example, lived between 3.5 and 3.2 mya. Some scientists thought that K. platyops, which means “flat-faced man,” represents a completely new hominin genus.

Paranthropus There is also confusion about where A. bosei and A. robustus fit in the classification of hominins. Traditionally, these two species have been classified as robust forms of australopithecines, distinguished from the smaller, more slender forms by their size and muscular jaws. Today, many scientists prefer to put these primates in a separate genus called Paranthropus. Paranthropoids, which thrived between 2 and 1.2 mya, were an offshoot of the human line that lived alongside human ancestors but were not directly related.

Overlapping hominins

However they are classified, these robust hominins appear to have lived alongside some of the slender australopithecines. They might have overlapped, for example, with A. garhi, an African australopithecine that was discovered in 1999. Is A. garhi our ancestor? No one knows. The illustration of the evolution of hominins is more like a bush than a tree. Many species lived successfully for years, often overlapping with earlier species and then—for unknown reasons—became extinct. By 1 mya, all australopithecines had disappeared from the fossil record. The only hominin fossils found after that time belong to the genus Homo.

Human Ancestry

The Genus Homo

The African environment became considerably cooler between 3 and 2.5 mya. Forests became smaller in size, and the range of grasslands was extended. The genus Homo, which includes living and extinct humans, first appeared during these years and although the fossil record is lacking fossils, many scientists infer that they evolved from an ancestor of the australopithecines.

Homo species had bigger brains, lighter skeletons, flatter faces, and smaller teeth than their australopithecine ancestors. They also are the first species known to control fire and to modify stones for tool use. As they evolved, they developed language and culture.

Homo habilis used stone tools

The first undisputed member of the genus Homo for which fossils exist is Homo habilis, called “handy man” because of its association with primitive stone tools. This species lived in Africa between about 2.4 and 1.4 mya. Figure 16.17 shows a scientific illustrator’s idea of what H. habilis might have looked like.

H. habilis possessed a brain averaging 650 cm3, about 20 percent larger than that of the australopithecines. It also had other traits of the Homo species, including a smaller brow, reduced jaw, a flatter face, and more humanlike teeth. Like australopithecines, it was small, long-armed, and it seems to have retained the ability to climb trees. Other Homo species might have coexisted with H. habilis, among them a species called Homo rudolfensis. Because few fossils of H. rudolfensis have been found, its exact relationship to the rest of the Homo line is uncertain.

Homo ergaster migrated

Within about 500,000 years of the appearance of H. habilis, another Homo species, Homo ergaster, emerged with an even larger brain. H. ergaster, illustrated in Figure 16.18, appeared only briefly in the fossil record, from about 1.8 to 1.3 mya. H. ergaster was taller and lighter than H. habilis, and had longer legs and shorter arms. Its brain averaged 1000 cm3, and it had a rounded skull, reduced teeth, and what many scientists believe to be the first human nose (with the nostrils facing downward).

Tools

Carefully made hand axes and other tools associated with H. ergaster fossils suggest to some scientists that H. ergaster was a hunter, but others think that H. ergaster was primarily a scavenger and used the tools to scrape the meat off of scavenged bones.

Migration Both scavenging and hunting are associated with a migratory lifestyle, and H. ergaster appears to have been the first African Homo species to migrate in large numbers to Asia and possibly Europe, perhaps following the trail of migrating animals. The Eurasian forms of H. ergaster are called Homo erectus. H. ergaster shares features with modern humans. Because of these shared features, scientists hypothesize that H. ergaster is an ancestor of modern humans.

Homo erectus used fire H. erectus, illustrated in Figure 16.19, lived between 1.8 million and 400,000 years ago and appears to have evolved from H. ergaster as it migrated out of Africa. While some scientists consider H. ergaster and H. erectus a single species, H. erectus appears to have evolved traits that the early African H. ergaster species did not have. Members of this species seem to have been more versatile than their predecessors, and they adapted successfully to a variety of environments. H. erectus includes “Java Man,” discovered in Indonesia in the 1890s, and “Peking Man,” discovered in China in the 1920s.

In general, H. erectus was larger than H. habilis and had a bigger brain. It also had teeth that were more humanlike. Brain capacity ranged from about 900 cm3 in early specimens to about 1100 cm3 in later ones. It was as tall as H. sapiens but it had a longer skull, lower forehead, and thicker facial bones than either H. ergaster or H. sapiens. It also had a more prominent browridge. Evidence indicates that H. erectus made sophisticated tools, used fire, and sometimes lived in caves.

Homo floresiensis—”The Hobbit” Most scientists believe that

H. erectus went extinct about 400,000 years ago. However, a curious set of fossils were discovered in 2004 on the Indonesian island of Flores that suggest that descendants of H. erectus—or of some other ancient hominin species—remained on Earth until 12,000 years ago. These fossils, which are about 18,000 years old, represent a species called Homo floresiensis. H. floresiensis, nicknamed “The Hobbit,” was only about 1 m tall when full grown. While it had brain and body proportions like all the australopithecines, primitive stone tools were found with its fossils. You can compare H. floresiensis and H. sapiens skulls in Figure 16.20.

Homo heidelbergensis—“mosaic” traits

The transition from H. ergaster to modern humans appears to have occurred gradually. Numerous transitional fossils have been found that display a mixture, or mosaic, of H. ergaster and H. sapiens traits. These fossils are often categorized as Homo heidelbergensis, but others put them in a more generic category called Homo sapiens. These humans generally had larger brains and thinner bones than H. ergaster, but they still had browridges and receding chins.

Homo neanderthalensis built shelter

A distinct human species called Homo neanderthalensis, or the Neanderthals, evolved exclusively in Europe and Asia about 200,000 years ago, likely fromH. erectus or a Homo intermediary. Neanderthals were shorter but had more muscle mass than most modern humans. Their brains were sometimes even larger than the brains of modern humans, though the brains might have been organized in different ways. Neanderthals had thick skulls, bony browridges, and large noses. They also had a heavily muscled, robust stature, as illustrated in Figure 16.21. Evidence of heavy musculature appears in the extremely large muscle attachments and the bowing of the long bones.

Neanderthals lived near the end of the Pleistocene ice age, a time of bitter cold, and their skeletons reflect lives of hardship; bone fractures and arthritis seem to have been common. There is evidence that they used fire and constructed complex shelters. They hunted and skinned animals, and it is possible that they had basic language. There is also some evidence that they cared for their sick and buried their dead.

Are Neanderthals our ancestors? In some areas of their range, particularly in the Middle East and southern Europe, Neanderthals and modern humans overlapped for as long as 10,000 years. Some scientists suggest that the two species interbred. A skeleton found in Portugal, for example, has features of both Neanderthals and modern humans.

However, DNA tests on fossil bones suggest that Neanderthals were a distinct species that did not contribute to the modern human gene pool. Neanderthals went extinct about 30,000 years ago. The reasons why they went extinct are not known.

Emergence of Modern Humans

The species that displaced the Neanderthals, Homo sapiens, is characterized by a more slender appearance than all other Homo species. They have thinner skeletons, rounder skulls, and smaller faces with prominent chins. Their brain capacity averages 1350 cm3. H. sapiens first appeared in the fossil record, in what is now Ethiopia, about 195,000 years ago. These early H. sapiens made chipped hand axes and other sophisticated stone tools. They appear to have had the ability to use a range of resources and environments, and at some point they began migrating out of Africa. Table 16.2 compares modern humans with other Homo species.

Out-of-Africa hypothesis

The world’s population 200,000 years ago looked significantly different than it does today. It was inhabited by a morphologically diverse genus of hominins, including primitive humans, Neanderthals, and modern humans, as illustrated in Figure 16.22. By 30,000 years ago, however, only modern humans remained. Some scientists propose that these modern humans evolved from several dispersed populations of early Homo species at the same time in different areas of the world. According to this multiregional evolution model, modern races of humans arose in isolated populations by convergent evolution.

Most scientists explain the global dominance of modern humans with the African Replacement model or, more commonly, the **Out-of-Africa hypothesis**. According to this **hypothesis**, which was first proposed by Christopher Stringer and Peter Andrews of the British Museum of Natural History in 1988, modern humans evolved only once, in Africa, and then migrated to all parts of the world, eventually displacing other hominins.

“Mitochondrial Eve” The Out-of-Africa hypothesis was supported by mitochondrial DNA analysis of contemporary humans in the early 1990s. Mitochondrial DNA changes very little over time, and humans living today have nearly identical mitochondrial DNA. Researchers

Allan Wilson and Rebecca Cann of the University of California, Berkeley, reasoned that the population with the most variation should be the population that has had the longest time to accumulate diversity. This was exactly what they found in the mitochondrial DNA of Africans.

Because mitochondrial DNA is inherited only from the mother, this analysis suggested that H. sapiens emerged in Africa about 200,000 years ago from a hypothetical “Mitochondrial Eve.”

Later, work by other scientists studying DNA sequences in the male Y chromosome yielded similar results. While some scientists think that a single movement of only a few hundred modern humans ultimately gave rise to the world’s current population, others think the process occurred in phases, with some interbreeding among the species that humans displaced.

The beginning of culture

The first evidence of complex human culture appeared in Europe only about 40,000 years ago, shortly before the Neanderthals disappeared. Unlike the Neanderthals, early modern humans expressed themselves symbolically and artistically in decorative artifacts and cave drawings, as illustrated in Figure 16.23. They developed sophisticated tools and weapons, including spears and bows and arrows. They were the first to fish, the first to tailor clothing, and the first to domesticate animals. These and many other cultural expressions marked the appearance of fully modern humans, or Homo sapiens. Some people call them Cro-Magnons, and they represent the beginning of historic hunter-gatherer societies.

Humans continued their migration throughout Europe and Asia. They probably reached Australia by boat and traveled to North America via a land bridge from Asia. From North America, they spread to South America. They adapted to new challenges along the way, leaving behind a trail of artifacts that we study today.

The History of Classification

Early Systems of Classification

Has anyone ever told you to get organized? Your parents usually want your room kept in order. Your teachers might have asked you to organize your notes or homework. Keeping items or information in order makes them easier to find and understand. Biologists find it easier to communicate and retain information about organisms when the organisms are organized into groups. One of the principal tools for this is biological classification. Classification is the grouping of objects or organisms based on a set of criteria.

Aristotle’s system More than two thousand years ago, the Greek philosopher Aristotle (394–322 B.C.) developed the first widely accepted system of biological classification. Aristotle classified organisms as either animals or plants. Animals were classified according to the presence or absence of “red blood.” Aristotle’s “bloodless” and “red-blooded” animals nearly match the modern distinction of invertebrates and vertebrates. Animals were further grouped according to their habitats and morphology. Plants were classified by average size and structure as trees, shrubs, or herbs.

Aristotle’s system was useful for organizing, but it had many limitations. Aristotle’s system was based on his view that species are distinct, separate, and unchanging. The idea that species are unchanging was common until Darwin presented his theory of evolution. Because of his understanding of species, Aristotle’s classification did not account for evolutionary relationships. Additionally, many organisms do not fit easily into Aristotle’s system, such as birds that don’t fly or frogs that live both on land and in water. Nevertheless, many centuries passed before Aristotle’s system was replaced by a new system that was better suited to the increased knowledge of the natural world.

Linnaeus’s system

In the eighteenth century, Swedish naturalist Carolus Linnaeus (1707–1778) broadened Aristotle’s classification method and formalized it into a scientific system. Like Aristotle, he based his system on observational studies of the morphology and the behavior of organisms. For example, he organized birds into three major groups depending on their behavior and habitat. The birds in Figure 17.1 illustrate these categories. The eagle is classified as a bird of prey, the heron as a wading bird, and the cedar waxwing is grouped with the perching birds.

Linnaeus’s system of classification was the first formal system of taxonomic organization. Taxonomy is a discipline of biology primarily concerned with identifying, naming, and classifying species based on natural relationships. Taxonomy is part of the larger branch of biology called systematics. Systematics is the study of biological diversity with an emphasis on evolutionary history.

Binomial nomenclature Linnaeus’s method of naming organisms, called binomial nomenclature, set his system apart from Aristotle’s system and remains valid today. Binomial nomenclature gives each species a scientific name that has two parts. The first part is the genus name, and the second part is the specific epithet, or specific name, that identifies the species. Latin is the basis for binomial nomenclature because Latin is an unchanging language, and, historically, it has been the language of science and education.

Biologists use scientific names for species because common names vary in their use. Many times the bird shown in Figure 17.2 is called a redbird, sometimes it is called a cardinal, and other times it is called a Northern cardinal. In 1758, Linnaeus gave this bird its scientific name, Cardinalis cardinalis. Biologists are never confused or mistaken about what is being referred to by this name. Binomial nomenclature also is useful because common names can be misleading. If you were doing a scientific study on fish, you would not include starfish in your studies. Starfish are not fish. In the same way, great horned owls do not have horns and sea cucumbers are not plants.

When writing a scientific name, scientists follow certain rules.

•The first letter of the genus name always is capitalized, but the rest of the genus name and all letters of the specific epithet are lowercase.

•If a scientific name is written in a printed book or magazine, it should be italicized.

•When a scientific name is written by hand, both parts of the name should be underlined.

•After the scientific name has been written completely, the genus name often will be abbreviated to the first letter in later appearances. For example, the scientific name of Cardinalis cardinalis can be written C. cardinalis.

Modern classification systems

The study of evolution in the 1800s added a new dimension to Linnaeus’s classification system. Many scientists at that time, including Charles Darwin, Jean-Baptiste Lamarck, and Ernst Haekel, began to classify organisms not only on the basis of morphological and behavioral characteristics. They also included evolutionary relationships in their classification systems. Today, while modern classification systems remain rooted in the Linnaeus tradition, they have been modified to reflect new knowledge about evolutionary ancestry.

Taxonomic Categories

Think about how things are grouped in your favorite video store. How are the DVDs arranged on the shelves? They might be arranged according to genre—action, drama, or comedy—and then by title and year.

Although taxonomists group organisms instead of DVDs, they also subdivide groups based on more specific criteria. The taxonomic categories used by scientists are part of a nested-hierarchal system—each category is contained within another, and they are arranged from broadest to most specific.

Species and genus

A named group of organisms is called a taxon. Taxa range from having broad diagnostic characteristics to having specific characteristics. The broader the characteristics, the more species the taxon contains. One way to think of taxa is to imagine nesting boxes—one fitting inside the other. You already have learned about two taxa used by Linnaeus—genus and species. Today, a genus is defined as a group of species that are closely related and share a common ancestor.

Note the similarities and differences among the three species of bears in Figure 17.3. The scientific names of the American black bear (Ursus americanus) and Asiatic black bear indicate that they belong to the same genus, Ursus. All species in the genus Ursus have massive skulls and similar tooth structures. Sloth bears, despite their similarity to members of the genus Ursus, usually are classified in a different genus, Melursus, because they are smaller, have a different skull shape and size, and have two fewer incisor teeth than bears of the genus Ursus.

Family

All bears, both living and extinct species, belong to the same family, Ursidae. A family is the next higher taxon, consisting of similar, related genera. In addition to the three species shown in Figure 17.3, the Ursidae family contains six other species: brown bears, polar bears, giant pandas, Sun bears, and Andean bears. All members of the bear family share certain characteristics. For example, they all walk flatfooted and have forearms that can rotate to grasp prey closely.

Higher taxa

An order contains related families. A class contains related orders. The bears in Figure 17.3 belong to the order Carnivora and class Mammalia. A phylum or division contains related classes. The term division is used instead of phylum for the classification of bacteria and plants. Sometimes scientists break the commonly used taxa into subcatagories, such as subspecies, subfamilies, infraorders, and subphyla.

The taxon composed of related phyla or divisions is a kingdom. Bears are classified in phylum Chordata, Kingdom Animalia, and Domain Eukarya. The domain is the broadest of all the taxa and contains one or more kingdoms. The basic characteristics of the three domains and six kingdoms are described later in this chapter.

Figure 17.4 shows how the taxa are organized into a hierarchical system. The figure also shows the complete classification from domain to species for the American black bear and the Asiatic black bear. Notice that although these bears are classified as different species, the rest of their classification is the same.

Systematics Applications

Scientists who study classification provide detailed guides that help people identify organisms. Many times a field guide will contain a dichotomous key, which is a key based on a series of choices between alternate characteristics. You can find out whether a plant or animal is poisonous by using a dichotomous key to identify it.

Careers in Biology Systematists, like the one shown in Figure 17.5 also work to identify new species and relationships among known species. They incorporate information from taxonomy, paleontology, molecular biology, and comparative anatomy in their studies. While the discovery of new species is exciting and important, learning a new connection between species also impacts science and society. For example, if a biologist knows that a certain plant, such as the Madagascar periwinkle Catharanthus roseus, produces a chemical that can be used to treat cancer, he or she knows that it is possible related plants also might produce the same or similar chemicals.

Modern Classification

Determining Species

It isn’t always easy to define a species. Organisms that are different species by one definition might be the same species by a different definition. As knowledge increases, definitions change. The concept of a species today is much different than it was 100 years ago.

Typological species concept Aristotle and Linnaeus thought of each species as a distinctly different group of organisms based on physical similarities. This definition of species is called the typological species concept. It is based on the idea that species are unchanging, distinct, and natural types, as defined earlier by Aristotle. The type specimen was an individual of the species that best displayed the characteristics of that species. When another specimen was found that varied significantly from the type specimen, it was classified as a different species. For example, in Figure 17.6 the color patterns on the butterflies’ wings are all slightly different. At one time, they would have been classified as three different species because of these differences, but now they are classified as the same species.

Because we now know that species change over time, and because we know that members of some species exhibit tremendous variation, the typological species concept has been replaced. However, some of its traditions, such as reference to type specimens, remain.

Biological species concept Theodosius Dobzhansky and Ernst Mayr, two evolutionary biologists, redefined the term species in the 1930s and 1940s. They defined a species as a group of organisms that is able to interbreed and produce fertile offspring in a natural setting. This is called the biological species concept, and it is the definition for species used throughout this textbook. Though the butterflies in Figure 17.6 have variable color patterns, they can interbreed to produce fertile offspring and therefore are classified as the same species.

There are limitations to the biological species concept. For example, wolves and dogs, as well as many plant species, are known to interbreed and produce fertile offspring even though they are classified as different species. The biological species concept also does not account for extinct species or species that reproduce asexually. However, because the biological species concept works in most everyday experiences of classification, it is used often.

Phylogenetic species concept In the 1940s, the evolutionary species concept was proposed as a companion to the biological species concept. The evolutionary species concept defines species in terms of populations and ancestry. According to this concept, two or more groups that evolve independently from an ancestral population are classified as different species. More recently, this concept has developed into the phylogenetic species concept. Phylogeny is the evolutionary history of a species. The phylogenetic species concept defines a species as a cluster of organisms that is distinct from other clusters and shows evidence of a pattern of ancestry and descent. When a phylogenetic species branches, it becomes two different phylogenetic species. For example, recall from Chapter 15 that when organisms become isolated—geographically or otherwise—they often evolve different adaptations. Eventually they might become different enough to be classified as a new species.

This definition of a species solves some of the problems of earlier concepts because it applies to extinct species and species that reproduce asexually. It also incorporates molecular data. Table 17.2 summarizes the three main species concepts.

Characters

To classify a species, scientists often construct patterns of descent, or phylogenies, by using characters—inherited features that vary among species. Characters can be morphological or biochemical. Shared morphological characters suggest that species are related closely and evolved from a recent common ancestor. For example, because hawks and eagles share many morphological characters that they do not share with other bird species, such as keen eyesight, hooked beaks, and taloned feet, they should share a more recent common ancestor with each other than with other bird groups.

Morphological characters

When comparing morphological characters, it is important to remember that analogous characters do not indicate a close evolutionary relationship. Recall from Chapter 15 that analogous structures are those that have the same function but different underlying construction. Homologous characters, however, might perform different functions, but show an anatomical similarity inherited from a common ancestor.

Birds and dinosaurs

Consider the oviraptor and the sparrow shown in Figure 17.7. At first you might think that dinosaurs and birds do not have much in common and do not share a close evolutionary relationship. A closer look at dinosaur fossils shows that they share many features with birds. Some fossil dinosaur bones, like those of the large, carnivorous theropod dinosaurs, show that their bones had large hollow spaces. Birds have bones with hollow spaces. In this respect, they are more like birds than most living reptiles, such as alligators, lizards, and turtles, which have dense bones. Also, theropods have hip, leg, wrist, and shoulder structures that are more similar to birds than to other reptiles. Recently, scientists have discovered some fossil dinosaur bones that suggest some theropods had feathers. The evidence provided by these morphological characters indicates that modern birds are related more closely to theropod dinosaurs than they are to other reptiles.

Biochemical characters Scientists use biochemical characters, such as amino acids and nucleotides, to help them determine evolutionary relationships among species. Chromosome structure and number is also a powerful clue for determining species similarities. For example, members of the mustard family (Cruciferae)—including broccoli, cauliflower, and kale—all look different in the garden, but these plants have almost identical chromosome structures. This is strong evidence that they share a recent common ancestor. Likewise, the similar appearance of chromosomes among chimpanzees, gorillas, and orangutans suggests a shared ancestry. Figure 17.8 shows the similar appearance of a chromosome-banding pattern in these three primates.

DNA and RNA analyses are powerful tools for reconstructing phylogenies. Remember that DNA and RNA are made up of four nucleotides.

The nucleotide sequences in DNA define the genes that direct RNA to make proteins. The greater the number of shared DNA sequences between species, the greater the number of shared genes—and the greater the evidence that the species share a recent common ancestor.

Scientists use a variety of techniques to compare DNA sequences when assessing evolutionary relationships. They can sequence and compare whole genomes of different organisms. They can compare genome maps made by using restriction enzymes, like those you learned about in Chapter 13. They also use a technique called DNA-DNA hybridization, during which single strands of DNA from different species are melted together. The success of the hybridization depends on the similarity of the sequences—complementary sequences will bind to each other, while dissimilar sequences will not bind. Comparing the DNA sequences of different species is an objective, quantitative way to measure evolutionary relationships.

A species example

The classification of elephants is one example of how molecular data has changed traditional taxonomic organization. Figure 17.9 shows pictures of elephants that live in the world today. Taxonomists have classified the Asiatic elephant (Elephas maximus) as one species and the African elephant (Loxodonta africana) as another for over 100 years. However, they have classified the two types of African elephant as the same species, even though the two populations look different. The forest-dwelling elephants are much smaller and have longer tusks and smaller ears than the savanna-dwelling elephants. Even so, scientists thought that the elephants interbred freely at the margins of their ranges. Recent DNA studies, however, show that the African elephants diverged from a common ancestor about 2.5 million years ago. Scientists have proposed renaming the forest-dwelling elephant Loxodonta cyclotis. Use Data Analysis Lab 17.1 to explore molecular evidence for renaming the forest-dwelling elephant.

Molecular clocks

You know that mutations occur randomly in DNA. As time passes, mutations accumulate, or build up, in the chromosomes. Some of these mutations are neutral mutations that do not affect the way cells function, and they are passed down from parent to offspring. Systematists can use these mutations to help them determine the degree of relationship among species. A **molecular clock** is a model that is used to compare DNA sequences from two different species to estimate how long the species have been evolving since they diverged from a common ancestor. Figure 17.10 illustrates how a molecular clock works.

Scientists use molecular clocks to compare the DNA sequences or amino acid sequences of genes that are shared by different species. The differences between the genes indicate the presence of mutations. The more mutations that have accumulated, the more time that has passed since divergence. When the molecular clock technique was first introduced in the 1960s, scientists thought the rate of mutation within specific genes was constant. Hence, they used the clock as an analogy. But while clocks measure time in predictable intervals, scientists now know that the speed by which mutations occur is not always the same in a single gene or amino acid sequence.

The rate of mutation is affected by many factors, including the type of mutation, where it is in the genome, the type of protein that the mutation affects, and the population in which the mutation occurs. In a single organism, different genes might mutate, or “tick,” at different speeds.

This inconsistency makes molecular clocks difficult to read. Researchers try to compare genes that accumulate mutations at a relatively constant rate in a wide range of organisms. One such gene is the gene for cytochrome c oxidase, which is found in the mitochondrial DNA of most organisms.

Despite their limitations, molecular clocks can be valuable tools for determining a relative time of divergence of a species. They are especially useful when used in conjunction with other data, such as the fossil record.

Phylogenetic Reconstruction

The most common systems of classification today are based on a method of analysis called cladistics. Cladistics is a method that classifies organisms according to the order that they diverged from a common ancestor. It reconstructs phylogenies based on shared characters.

Character types Scientists consider two main types of characters when doing cladistic analyses. An ancestral character is found within the entire line of descent of a group of organisms. Derived characters are present members of one group of the line but not in the common ancestor. For example, when considering the relationship between birds and mammals, a backbone is an ancestral character because both birds and mammals have a backbone and so did their shared ancestor. However, birds have feathers and mammals have hair. Therefore, having hair is a derived character for mammals because only mammals have an ancestor with hair. Likewise, having feathers is a derived character for birds.

Cladograms Sytematists use shared derived characters to make a cladogram. A cladogram is a branching diagram that represents the proposed phylogeny or evolutionary history of a species or group. A cladogram is a model similar to the pedigrees you studied in Chapter 11. Just as a pedigree’s branches show direct ancestry, a cladogram’s branches indicate phylogeny. The groups used in cladograms are called clades. A clade is one branch of the cladogram.

Constructing a cladogram Figure 17.11 is a simplified cladogram for some major plant groups. This cladogram was constructed in the following way. First, two species were identified, conifers and ferns, to compare with the lily species. Then, another species was identified that is ancestral to conifers and ferns. This species is called the outgroup.

The outgroup is the species or group of species on a cladogram that has more ancestral characters with respect to the other organisms being compared. In the diagram below, the outgroup is moss. Mosses are more distantly related to ferns, conifers, and lilies.

The cladogram is then constructed by sequencing the order in which derived characters evolved with respect to the outgroup. The closeness of clades in the cladogram indicate the number of characters shared. The group that is closest to the lily shares the most derived characters with lilies and thus shares a more recent common ancestor with lilies than with the groups farther away. The nodes where the branches originate represent a common ancestor. This common ancestor generally is not a known organism, species, or fossil. Scientists hypothesize its characters based on the traits of its descendants.

The primary assumption

The primary assumption that systematists make when constructing cladograms is that the greater the number of derived characters shared by groups, the more recently the groups share a common ancestor. Thus, as shown in Figure 17.11, lilies and conifers have three derived characters in common and are presumed to share a more recent common ancestor than lilies and ferns, which share only two characters.

A cladogram also is called a **phylogenetic tree**. Detailed phylogenetic trees show relationships among many species and groups of organisms. Figure 17.12 illustrates a phylogenetic tree that shows the relationships among the domains and kingdoms of the most commonly used classification system today.

The tree of life

In his book On the Origin of Species, Charles Darwin used the analogy of a tree to suggest that all of the species developed from one or a few species. He imagined the tree’s trunk to represent ancestral groups and each of the branches to have similar species. From each branch, smaller and smaller branches grew. Finally, at the tips of the twigs of these branches were the leaves, consisting of individual living species. This concept was developed further, and the term tree of life was coined by German biologist Ernst Haeckel (1834– 1919). Figure 17.13 shows Haeckel’s Genealogical Tree of Humanity. Haeckel was the first to represent phylogenies in the form of a tree, and while his phylogenies are no longer completely accurate, they represent the first step in the reconstruction of phylogenies.

The tree of life diagram in Figure 17.13 is a representation of the diversity of living organisms. A tree of life that incorporates all known organisms is almost unimaginably large. Scientists have discovered and described nearly 1.75 million species, and they estimate that millions more remain unclassified. Assembling a comprehensive tree of life requires a convergence of data from phylogenetic and molecular analysis. It also requires collaboration among many scientists representing many disciplines, from molecular biology to Earth science to computer science. Many scientists believe that the construction of a comprehensive tree of life, though an enormous task, is an important goal.
