# Supplementary material for: A semantic classification of nominal technical terms in secondary school biology textbooks
Source: PLoS One. 2024 Nov 11;19(11):e0312040. doi: 10.1371/journal.pone.0312040 (PMC11554214; doi:10.1371/journal.pone.0312040)
Supplement: S4 File — (DOCX) [file pone.0312040.s006.docx]

**Textbook 1: NSW Oxford Insights Science Year 8 & 9**

Chapter 2 Functioning organisms

2.1 Flowering plants as functioning organisms

OBTAINING WATER AND NUTRITION

Flowering plants have two organ systems in addition to their flowers: the shoot system and the root system. These systems contain a range of cell types and tissues that have structures well suited to their various functions. The shoot system consists of the stem, leaves, buds, flowers and fruits. The root system consists of either a branching or a fibrous system of roots.

Figure 2.1 shows the root and shoot system of a typical flowering plant, but in reality there is great variation in the size and shape of plants. For example, trees and shrubs contain woody tissues, whereas herbs and grasses do not. Plants also grow in many different ways. Some plants grow vertically, some have horizontal branches that droop downwards, while others spread out across the ground. Some plants grow over many years (perennial), whereas others complete their life cycle within a year (annual) or even a single season. Some flowering plants are evergreen while others shed all their leaves in one season to regrow them in another (deciduous).

The root system

The function of the root system is to anchor the plant to a surface and to absorb and transport water and minerals. The root system may also store substances or allow a special type of reproduction called vegetative propagation that results in new plants the same as the parent plant. Vegetative propagation will be explained in more detail later in the chapter.

The shoot system

The stem supports the leaves, flowers and fruit in positions where they can function effectively. Stems contain tube-like vascular bundles through which water and mineral nutrients travel up from the soil to the leaves and glucose solutions travel to the cells throughout the plant. Some stems also can carry out photosynthesis, the food- making process in plants.

The leaves are the main site of photosynthesis, water evaporation and gaseous exchange. Gaseous exchange involves both carbon dioxide and oxygen. Carbon dioxide is absorbed for photosynthesis and the oxygen released. All cells in plants carry out respiration.

During respiration, oxygen is absorbed and carbon dioxide is released through pores in leaves and stems.

The flowers and fruit are responsible for sexual reproduction in plants. Sexual reproduction is important as it produces offspring with different characteristics from the parent plants, which gives a species a better chance to survive a changing environment. In order for sexual reproduction to occur, flowers must be pollinated and, when mature, form the fruit that releases seeds or attracts animals that will disperse the seeds for the plant.

The organ systems and their functions relate back to the needs of individual plant cells. Key features of plant cells (see Figure 2.2) are the possession of a rigid cell wall external to the cell membrane and membrane-bound organelles, which include chloroplasts in the shoot system. The cell wall provides support so there is no need for a skeleton, while the chloroplasts give plants the ability to carry out photosynthesis so there is no need to capture and eat food. Vacuoles may make up 90% of the volume of plant cells and store the cell sap.

How do flowering plants obtain water and minerals?

When you learnt about the states of matter in chapter 4 of Oxford Insight Science 7, you learnt about a special process that occurs in gases and liquids called diffusion. Remember that diffusion is a mixing process in which a substance moves from a place where it is highly concentrated until it is evenly spread out.

In nature, the cell membrane acts as a selective barrier for diffusion. Some substances, such as water, can easily diffuse through the cell membrane.

Other particles, such as dissolved salts and sugars, cannot pass directly through the cell membrane. Osmosis is the movement of water through a semipermeable membrane, which includes the cell membrane.

During osmosis, water will always move from an area with higher water concentration (less dissolved substances) through the cell membrane to an area of lower water concentration (greater dissolved substances). The cytoplasm in root hair cells contains lots of dissolved substances, so the water in the soil moves into the plant by osmosis. Root hairs are elongated so that they have a high surface area to increase the rate of osmosis.

Water and some dissolved minerals move through a part of the vascular bundle called xylem tissue, which is made up of long tube-like cells that are not living. The main force that moves water from the roots up to the leaves is pressure resulting from transpiration (see Figure 2.4).

The transpiration stream

Less than 1% of the water that moves in the transpiration stream of plants is used for photosynthesis or growth. Most of the water is lost through transpiration, the process by which water evaporates from the surface of the leaves. Transpiration is useful for cooling plant leaves, and also for helping to pull the transpiration stream up to the uppermost leaves. Other forces that help to draw water up through plants include the pressure from osmosis as water enters the cytoplasm of the root hairs.

Photosynthesis

Photosynthesis is the reaction used by plants to transform light energy into chemical energy. Photosynthesis needs the help of a chemical called chlorophyll, the green pigment in the chloroplasts of plant cells. Chloroplasts are found in cells in the shoot system, particularly in tissues near the surface of the leaves. The shape and arrangement of the leaves in many flowering plants maximises their ability to absorb sunlight and carry out photosynthesis.

Photosynthesis can be summarised by the following word and symbol equations:

carbon dioxide + light energy + water → glucose + oxygen

light

6CO2 + 6H2O → C6H12O6 + 6O2

Although photosynthesis is summarised as a single equation, it is actually a complex series of reactions. First, the chlorophyll traps the light energy, and then the sugars are made.

How does the plant obtain its requirements for photosynthesis?

Photosynthesis requires carbon dioxide, light energy and water. The light energy is obtained from the sun. Water enters the plant through the roots and is transported to the leaves by the xylem tissue. Carbon dioxide moves into the leaf through microscopic pores called stomata (singular: stoma, Figure 2.7).

The stomata are also involved in releasing the oxygen produced by photosynthesis back into the atmosphere. Guard cells open and close the stomata to regulate the exchange of gases and water loss.

Sometimes plants experience conflicting demands, especially in periods of water shortage. The stomata may be forced to close to prevent damage from wilting and dehydration. This will restrict the availability of carbon dioxide, so photosynthesis may be interrupted.

What happens to the glucose that the plant produces?

Green plants make millions of glucose molecules during daylight hours. Plants require a constant supply of glucose for respiration.

Respiration can be summarised in the following word equation:

glucose + oxygen → carbon dioxide + water + energy

In many ways, respiration is the opposite process to photosynthesis. During the day, the oxygen released into the leaf tissues through photosynthesis will be available for respiration and the carbon dioxide released from respiration will be available for photosynthesis.

Respiration releases energy to run all the metabolic processes within a plant.

These processes include transporting substances around the plant and making leaves, flowers, fruit and seeds. This means the glucose has to be moved from the leaves to all the cells in the plant. Phloem cells in the vascular bundles are responsible for the absorption of excess glucose and its transport in solution around the plant. Some glucose is converted into cellulose, the material used in plant cell walls. Other glucose molecules are converted into components of the plant’s membranes.

Photosynthesis produces more glucose than can be used directly by plants. Excess glucose is stored in the form of a carbohydrate called starch and other carbohydrates in the roots, stems or the leaves. Starch is stored in underground storage organs such as roots and tubers. Potatoes, carrots and parsnips all store starch in this way.

Systems working together

The plant root and shoot systems work together to ensure plant survival, growth and reproduction. These functions are described further in Table 2.1.

GROWTH, REPAIR AND RESPONDING

Most flowering plants are very resilient. Leaves are grazed and trampled upon, termites eat their wood, sap is sucked and trunks are burnt by bushfires. Flowering plants also suffer excesses and shortages of water. Despite these conditions, flowering plants remain the dominant plants on the Earth. Their ability to regenerate, grow and reproduce is dependent on the process of cell division.

Cell division

Cell division is the process by which a cell divides into two or more cells. Cells that have the ability to divide and become specialised cells are called stem cells. But not all cells can divide. Some cells develop specialised structures so they can carry out particular functions but, in the process, they lose the ability to divide.

Mitosis

In chapter 3 of Oxford Insight Science 7 you learnt about mitosis. Mitosis is a type of cell division where one parent cell divides to produce two daughter cells with the same identical genetic instructions carried in their DNA (see Figure 2.11). DNA is the genetic material that includes the instructions to make every part of a cell. DNA is made up of enormous molecules found in the nucleus of cells. These molecules have the incredible ability to replicate or copy themselves. When the cell divides, these molecules condense into structures called chromosomes, which are visible under the light microscope (see Figure 2.12).

Asexual reproduction

Asexual reproduction results from mitosis, and the offspring are essentially the same as the parents. Asexual reproduction occurs in many unicellular organisms as well as many plants and a few simple animals. In plants, asexual reproduction is generally referred to as vegetative reproduction.

Vegetative reproduction is the process where new plants arise without the production of seeds. It occurs in the specialised cells and tissues of roots, stems and leaves of some flowering plants.

Vegetative reproduction often involves part of the plant breaking off and surviving as a new organism that is genetically the same as the original ‘parent’ plant. Vegetative reproduction can occur naturally or be induced by humans (such as during plant cloning: see Deeper Understanding on page 65). Often we carefully manage vegetative reproduction to maximise the production of desirable plants.

Role of cell division

Cell division is essential for growth. Cell division is also a response by plants to changes in the environment. For example, increasing day length (such as in spring) may stimulate cell division and result in the production of new leaves or flower buds.

Cell division is stimulated by the production of plant hormones.

Cell division for growth occurs at the tips of roots and stems, and in special buds called axils in flowering plants. The cluster of actively dividing cells at the tips of roots and shoots, are called the apical meristems. Some plants, such as woody plants, produce new vascular bundles by cell division in the outer layers beneath the bark. This can be seen as growth rings.

Sometimes, plant cells develop abnormal growths. Parasites, fungi, bacteria or insects may cause these growths. For example, wasps lay their eggs in plant tissues, which can stimulate the surrounding plant cells to divide. This cell division results in the production of structures such as galls, which serve to isolate the infected area.

THE ROLE OF FLOWERS

Asexual or vegetative reproduction is more common in plants than in animals, yet sexual reproduction in plants remains very important. Sexual reproduction involves the production of special sex cells that combine in the process of fertilisation to result in a new organism.

In the case of flowering plants, pollination needs to occur before fertilisation. Pollination is the transfer of pollen (the male sex cell) to the female reproductive organ.

Diversity of flower structure, arrangement and mechanisms to achieve pollination has contributed to the success of flowering plants.

Meiosis

Sexual reproduction results in increased genetic diversity because it allows the genetic material from two different parents to combine. Sexual reproduction is dependent on the production of sex cells or gametes in a special type of cell division called meiosis. In this division, the daughter cells contain only one member from each of the pairs of chromosomes of the original parent cell.

Meiosis (see Figure 2.17) has two divisions to enable this halving of genetic material. The first division separates the pairs of chromosomes; the second division is similar to mitosis. The final product is four daughter cells that have only half the number of chromosomes of the original cell.

A new organism is the result of the union or fertilisation of two gametes, usually one male gamete and one female gamete, which restores the full complement of chromosomes to the cells of the first cell of the new organism, called the zygote.

Flowers: sexual reproduction

Sexual reproduction in plants results in increased genetic diversity because it allows new combinations of genetic material or DNA from two different parents. Sexual reproduction helps plants spread over large areas by releasing seeds or fruit that are spread by agents. These agents include wind, running water and animals.

Flowers come in all shapes and sizes. Not all of them are attractive and some smell terrible! However, the purpose of a flower is not necessarily to be sweet smelling and beautiful, but to contain the sexual reproductive organs of the plant and to help fertilisation to occur.

Meiosis occurs in the anther (male) and ovary (female) organs, resulting in the production of pollen and ova (singular: ovum).

The anther is supported by a stalk-like filament, and together these structures form the stamen. The ovary sits below a stalk-like style that is topped by the sticky stigma, and together they form the carpel. For fertilisation to occur, the pollen needs to find the ovum.

This requires pollination – the pollen is transferred to the stigma where it grows a pollen tube down the style to the ovary.

Many flowers need assistance from agents for pollination to occur. Self-pollination involves pollen from a flower landing on its own stigma or that of another flower on the same plant (see Figure 2.19). Cross- pollination occurs when pollen from one flower lands on the stigma of a flower on a different plant, producing greater variation (see Figure 2.20). Just like animals, the pollen from one flower can only fertilise flowers from the same or very similar species.

After fertilisation, the ovary takes on a role similar to a bird’s egg. It swells to become a fruit, which provides nutrition and protection for the zygotes to grow into embryos in the enclosed seeds. The ovary structure is reflected in the structure of the seed-bearing area of the fruit (see Figure 2.21).

2.2 Humans as functioning organisms

THE DIGESTIVE SYSTEM

Digestion is the process by which foods (and drinks) are broken down and absorbed into your blood for transport to your cells. Food provides us with the materials for energy, growth and repair. The food we eat is broken down into smaller chemicals, called nutrients, by our digestive system.

Nutrients provide nutrition to the body. They are mostly soluble substances that are absorbed by the blood and circulated around the body. Different types of foods provide us with different nutrients. The function of the digestive system is to break down food into substances that can be transported around the body.

The digestive tract (also known as the gastrointestinal tract) is made up of a group of organs that form a tube travelling from the mouth to the anus. Along the way, food is broken down and absorbed into the blood. The internal walls of the intestines are highly folded into projections called villi to increase their surface area for absorption into the blood (see Figure 2.25). Food that is not digested by the body remains in the digestive tract where it is broken down further by bacteria and forms solid faeces. When it reaches the end of the digestive tract, the faeces is released into the toilet. Figure 2.27 on page 74 shows what happens as food moves down through the digestive tract.

The mouth

Breakdown of food begins in the mouth. The human mouth has three main types of teeth: incisors (the front ones), canines (the pointy teeth next to the incisors) and molars (the flatter ones toward the back).

The tongue is a large muscular organ in the mouth that can push upwards, sideways and backwards to help move food onto the teeth to be chewed. Saliva is the liquid found in the mouth. It is mostly water, but also contains enzymes. Enzymes are special chemical molecules that help chemical reactions happen.

The stomach

The human stomach is J-shaped and spends its time churning its contents round and round, with muscular walls pulling in all directions. The stomach is filled with gastric juices made of hydrochloric acid and enzymes. The cells lining the inside of the stomach produce mucus to stop the acid burning the stomach walls.

The intestines

In the small intestine, digestion via enzyme action and the absorption of nutrients continues. Although the stomach absorbs water and some other simple substances, including alcohol and some medications (which is why they have a rapid effect), most nutrients are absorbed in the small intestine. Figure 2.29 shows the villi that allow absorption of nutrients.

The junction of the small intestine and large intestine contains the appendix, which is not believed to be functional in humans. Infection and swelling of the appendix is known as appendicitis. The large intestine contains lots of bacteria to help with the absorption of some vitamins, minerals and water from undigested fibre. Wastes (faeces) collect and are stored in the rectum before being passed out through the anus. Passing faeces is not considered part of excretion since it is not a waste product from within the body’s cells.

THE CIRCULATORY SYSTEM

The circulatory system is the body’s transport system and is responsible for moving blood around your body. The heart pumps the blood through the system of blood vessels including the main arteries and veins (Figure 2.30). Many different substances, including nutrients and wastes, are transported in the blood, picked up from and dropped off at different locations.

Blood

Blood is a combination of cells, cell fragments, liquid and dissolved substances.

•Oxygen is carried by a chemical called haemoglobin in red blood cells from the lungs to all the cells of the body. You will learn more about red blood cells later in this chapter. Carbon dioxide is also carried in the red blood cells. However, it generally moves from the cells of the body to the lungs.

•Nutrients and wastes are dissolved in the plasma for transport to and from cells.

•White blood cells are involved in fighting foreign cells, including disease-causing pathogens, and travel in the blood to places where they are needed.

•Platelets are cell fragments that travel in the blood to places where cuts need to be blocked – they fill the hole.

The heart

The heart is a large pump about the size of your fist. It is made of four chambers: two atria (singular: atrium) at the top and two ventricles at the bottom. The right side of the heart pumps blood to the lungs to ‘drop off’ carbon dioxide and ‘pick up’ oxygen, whereas the left side pumps blood around the rest of your body. Valves keep the blood moving in the right direction. The aorta extends from the top of the heart and is the largest blood vessel in the body.

Blood vessels

Blood travels through tubes called blood vessels. Blood vessels have different sizes and structures depending on the amount of blood they need to carry as well as the speed of the blood and whether it is picking up or dropping off substances.

Arteries have thick, muscular walls to cope with high pressure and to squeeze the blood along. Arteries travel away from the heart – the blood is at a higher pressure here because it has just been pumped. Arteries branch into arterioles (smaller arteries).

Veins carry blood back to the heart to be pumped elsewhere. These vessels are similar in size to the arteries, but they only have a small amount of muscle in their walls. To avoid any blood going backwards due to a lack of pressure, veins have one-way valves in them.

Capillaries (see Figure 2.33) are the thinnest of the blood vessels: their walls are only one cell thick to allow substances to easily pass in and out of the blood.

Capillaries are the vessels connecting the arteries and veins.

THE RESPIRATORY SYSTEM

The respiratory system (see Figure 2.35) is responsible for gas exchange through breathing. The key processes involved in gaseous exchange occur in the lungs. Oxygen enters the blood from the air we inhale and carbon dioxide leaves the blood and is exhaled.

We breathe air in through our nose and mouth, cleaning it with hairs and wet surfaces as it travels to our throat or pharynx. At the bottom of the pharynx is a trapdoor called the epiglottis, which controls the path of food and air. Food goes down the oesophagus to the stomach while air goes down the trachea to the lungs.

For gas exchange to occur, the circulatory system is involved. The blood vessels need to be able to get really close to the air in the lungs. We will now look at how the structure of the lungs allows this to happen.

The lungs

There are two lungs in our chest, which change in size every time we take a breath and they fill with air. The trachea branches into two to carry air into each lung. These branches are called bronchi. The lungs feel spongy to touch because they are home to millions of tiny air sacs called alveoli. If these air sacs were unravelled and flattened, they would have a surface area of approximately half the size of a tennis court. Each tiny alveolus is covered by a mesh of capillaries. The lungs are structured to have as many air sacs as close to as many blood vessels as possible to aid gas exchange.

The diaphragm

The diaphragm is a dome-shaped muscle that is attached to your ribs and moves up and down beneath your lungs. The muscle contracts down and relaxes up. The diaphragm also separates the heart and lungs from the stomach and digestive system. The lungs have no muscle tissue, so they can’t move on their own. Muscles between the ribs lift the rib cage up and out to increase the ‘suction’ of air into the lungs.

Comparing respiration with breathing

The reaction that occurs in human body cells to produce energy is the same reaction we saw in plant cells: respiration. Oxygen taken in by the lungs reacts with glucose, which the digestive system releases from the food we eat.

glucose + oxygen → carbon dioxide + water + energy C6H12O6 + 6O2 → 6CO2 + 6H2O + energy

The energy produced by respiration is then used for all the jobs the cell needs to perform, from making and breaking down substances to making new cells. You can see why people get confused about the difference between breathing and respiration – respiration is the actual process that happens in cells and breathing is the inhalation of oxygen and exhalation of carbon dioxide by your lungs and other organs in the respiratory system.

THE EXCRETORY SYSTEM

Our cells and our bodies create a number of waste products. To keep functioning correctly, these wastes need to be removed. The process of removing wastes is called excretion.

The organs of excretion are the kidneys, liver, lungs and skin. These organs make up the excretory system.

The kidneys

You have two kidneys, one on each side of your lower back. They are approximately 10 centimetres long. Blood carrying waste products enters your kidneys to be filtered by tiny structures in the kidney called nephrons. At the end of this filtering process there are two main outputs: clean blood in the renal vein and urine in the ureter.

The kidneys are also responsible for regulating the levels of water and salts in the blood. This can affect blood volume and pressure. If you drink large quantities of water, you will produce a larger volume of pale-coloured urine than if you do not drink much water. Urine is stored in the bladder before it passes down the urethra to be expelled from the body.

The skin

In chapter 3 of Oxford Insight Science 7 you looked at the structure of the skin. The skin plays a very important role in releasing waste heat by evaporation of sweat from wet skin. If you have ever licked your upper lip after exercise, you will know that your sweat is also very salty. This is because some salt is also excreted through the skin.

The liver

All our food has to be metabolised, or processed. Metabolism is the name given to the chemical reactions that occur in the body. These reactions can break down substances or build new substances. The liver is responsible for the metabolism of many substances, especially waste substances.

Waste substances that can be dangerous to the body are often changed into less dangerous forms by the liver before their removal from the body.

What is waste?

Our bodies produce a number of substances that need to be removed to avoid damage to our bodies. The human body, like all organisms, relies on a careful balance of inputs and outputs to work properly. Some substances are just taking up precious space, whereas others can actually harm us. Water is important in controlling wastes because it can dilute harmful substances, reducing the damage they can cause at the same time. Water also helps move substances quickly and is essential for keeping our body temperature just right.

When your body digests proteins, it breaks them down into smaller molecules called amino acids. Your liver converts these amino acids into glucose for energy. During this process, a very toxic substance called ammonia is produced. The liver then uses energy to change the ammonia into a safer substance called urea, which is then filtered by the kidneys for removal. When you have not drunk much water, you will have noticed that your urine is darker and smells more strongly. When you have drunk enough water your urine is lighter and has no smell. The amount of water in your urine can dilute the colour and smell of urea.

Storage of the faeces in the rectum and their expulsion through the anus is not considered part of the excretory processes. The contents of the gastrointestinal tract are not really considered to be within the body until the digested food is absorbed into the blood in the intestines.

THE SKELETAL/MUSCULAR SYSTEM

The skeletal/muscular system allows for voluntary movement in humans. Muscle cells have the special ability of being able to contract. Skeletal muscles occur in pairs, each one attached to a different side of a bone. Bones are the levers that bring about the movement of the body. Contraction of one member of a muscle pair, such as the biceps, allows movement of the arm in one direction, or the arm flexes. When it relaxes and the opposite member (the triceps) of the pair contracts, the arm will return to the original straight position.

The skeletal system

The skeletal system or skeleton in the adult human consists of 206 bones of varying shapes and sizes. It includes the bones that make up the skull and spine and the bones of the limbs, shoulders and pelvis. It also consists of the three tiny bones that transfer vibrations in the ear to allow us to hear. The skeletal system includes the long bones found in our limbs that are levers for movement.

The vertebrae provide support and flexibility, and the skull and ribs protect vital organs.

Bones are living tissues, which means they can repair themselves when damaged. They contain cells surrounded by calcium phosphate, the substance that makes the bone hard, and collagen, which provides elasticity. Blood vessels supply the bone with the nutrients it needs. The marrow inside bone is also very important because it is the site of production of blood cells.

Bones meet other bones at joints, which are the pivot points of the bone levers. Some joints are highly moveable because they are capsules containing membranes that secrete a type of lubricating liquid. Many bones are tipped by a tissue called cartilage, which cushions impacts with the joining bone and reduces friction.

Ligaments hold adjoining bones together in the joint. The shoulder and hip joints are ball-and-socket joints, which allow movement in many directions. The elbow and knee are hinge joints, and they only move in one direction. Other types of freely moveable joints include pivot joints, which allow the head to turn on the spine, and gliding and saddle joints that allow movement in the wrist and ankle.

The muscular system

The muscular system consists of about 640 skeletal muscles that are responsible for movement and posture, smooth muscles and cardiac muscle.

A muscle is made up of muscle fibres containing protein molecules that slide over one another to bring about muscle shortening or contraction. Muscle fibres are bundled together and the end of the muscle, the connective tissue from the bundles, combines to form tendons that attach to the skeleton. Skeletal muscles attach to bones of the skeleton to bring about voluntary movement.

In addition to skeletal muscles (also called voluntary muscles), the muscular system contains smooth muscles that contract automatically in internal organs such as the digestive tract and the uterus (womb) in females. A third muscle type, cardiac muscle in the heart, contracts in a highly coordinated way to bring about a heartbeat.

THE ROLE OF MITOSIS IN GROWTH AND REPAIR

In section 2.1, you learnt about the role of two types of cell division in flowering plants: mitosis and meiosis. Vegetative reproduction is not an option in humans, but mitotic cell divisions are essential to the functioning of humans to allow growth, cell replacement and repair of body tissues.

When the first cell (zygote) of a new human forms, all the subsequent cell divisions are by mitosis. These mitotic cell divisions result in the growth and development of the embryo into a foetus, an infant, a teenager and eventually an adult. Eventually, humans end up with about 10 trillion cells of over 200 different specialised types.

To achieve the growth of various tissues, organs and organ systems, the cells develop specialised structures through a process called differentiation. The first cell or zygote has the ability to differentiate into all types of cells. As humans mature, some of the cells become so specialised that they can

no longer carry out mitosis. In these cases, specific cell groups called stem cells retain the ability to divide and differentiate. Stem cells are important in the repair, growth and normal functioning of humans.

Repair and maintenance of the human body are complex processes. Mitotic cell divisions are essential for the production of new cells but cell differentiation must also occur so that cells can carry out their specialised roles. Some organs consist of a variety of cells and tissues, so it is not a simple process to repair them when something goes wrong.

Mitosis and red blood cells

Red blood cells are tiny but they are present in our bodies in huge numbers, making up one quarter of our total number of cells.

Red blood cells are highly specialised to carry oxygen on haemoglobin molecules throughout the body.

Red blood cells have no nucleus or organelles, which gives them their distinctive biconcave shape. This shape is beneficial as it allows more room for oxygen and provides the flexibility for a red blood cell to squeeze through even the smallest capillary. The disadvantage of not having a nucleus is that red blood cells only have a life span of 100–120 days and cannot divide by mitosis. Our bodies are constantly making red blood cells, at the rate of about 2 million per second. This occurs in the bone marrow of the long bones in adults and in the liver of embryos.

People who lose a lot of blood may not be able to make enough red blood cells to survive. Blood transfusions can be given, but the donated blood must be a compatible blood type to that of the recipient or a fatal rejection may occur.

All living things reproduce, leaving new organisms to carry on when others die. We are constantly seeking ways to live longer, to monitor the health and development of our babies even before they are born, and to help those who are unable to have babies naturally.

Sexual reproduction

Sexual reproduction produces variations in a population. The offspring (babies) are all different from their parents, having new combinations of features. This variation is important for the survival of the entire species. Imagine what life would be like if all humans looked and behaved exactly the same!

The structure and function of the human reproduction system enables people all around the world to have babies– approximately four births occur every second! As of 2014, the world population was just over 7 billion people.

The vast majority of animals reproduce sexually. Many are also sexually dimorphic, which means that the males look physically different from the females. This is true for humans, who have external and internal differences between the genders. Just like in flowering plants, meiosis is a key process in the formation of male and female gametes in humans.

Female reproductive system

The reproductive system in females is mostly internal. The external opening of the vagina leads up to the uterus, which has two fallopian tubes branching off that connect to the ovaries. The female gametes or ova (singular: ovum) are produced in the ovaries through meiosis. Once released during ovulation, the ova move slowly down the fallopian tube towards the uterus. Fertilisation with the male’s sperm usually happens in the fallopian tube.

Male reproductive system

The male reproductive system is much more obvious from the outside of the body. Both the penis and testes are external.

The testes are where sperm are produced through meiosis. The testes are contained in the scrotum, which is able to regulate the temperature 1–2° below normal body temperature of 37°C. To get outside of the body, the sperm move up through the vas deferens, has seminal fluid added from the seminal vesicles, and then moves into the urethra and out through the glans penis.

Puberty

While the specific reproductive systems are already in place before birth, both the male and female reproductive systems do not mature until **puberty**. **Puberty** is the phase of physical development when sexual maturity takes place. It is a time of significant change and is experienced by everyone differently.

**Puberty** is triggered and controlled by hormones that affect both the internal system and the external secondary sexual characteristics, such as facial hair in males and breasts in females.

Before **puberty**, boys and girls have a very similar basic body shape. After **puberty** the differences are very obvious.

In females, **puberty** triggers the onset of the menstrual cycle. The menstrual cycle continues until menopause, with the exception of during pregnancy. The menstrual cycle is controlled by chemicals called hormones and is essentially the preparation of the female body for a potential pregnancy. During the menstrual cycle, the lining of the uterus grows thicker and develops an increased blood supply.

If the ovum released during the menstrual cycle is not fertilised, menstruation will occur. Menstruation is the loss of the uterus lining, which is passed out of the body. The menstrual cycle takes approximately 28 days.

The role of meiosis in reproduction

The production of haploid gametes that fuse together to form new individuals means a continual blending of genetic material (DNA) across the generations. Children receive half of their chromosomes from their mother and the other half from their father. The chromosomes will be similar, in that they will have the information for the same features, such as eye colour, but the specific instructions can be different. For example, your father could have blue eyes while your mother has brown eyes. You could end up with blue, brown or maybe even hazel eyes.

Meiosis in the human female

Meiosis is a prolonged process in human females. The ova develop in structures called follicles. Many follicles fail to complete their development to release ova, but after **puberty**, once every 28 days, a single follicle will release its ovum into a fallopian tube.

This cycle of releasing an ovum every 28 days is the basis of the menstrual cycle. It is controlled by hormones but also produces hormones that are involved in the complex process of preparing a female for pregnancy.

Meiosis in the human male

Meiosis occurs in the testes of the male from the onset of puberty at about 11 or 12 years. It then occurs continuously with gradual decline only in old age. Meiosis division and differentiation into a sperm takes about 72 days.

The sperm itself has three parts: the head contains the nucleus, the mid-piece contains mitochondria to provide energy, and the hind part is a tail or flagellum that allows it to move.

Copulation

For fertilisation to occur, sperm and ovum must meet. Fertilisation in humans is internal.

Copulation is the process where the male inserts his penis into the vagina of the female. Ejaculation during copulation ensures that the sperm is deposited inside the female reproductive tract. The sperm then swim up through the uterus and into the fallopian tubes. If an ovum is present, then the sperm fertilise it to form a zygote. Even though it only takes one sperm to fertilise the ovum, millions of sperm will enter the fallopian tubes. Changes in levels of hormones occur if fertilisation and implantation of an embryo do not occur, and the female will menstruate.

Pregnancy and birth

If fertilisation does occur, the zygote (the first diploid cell that results from the fusion of sperm and ova) grows by the production of new cells from mitosis to become a blastocyst. The blastocyst implants in the lining of the wall of the uterus, then becomes an embryo with an attachment through the umbilical cord to a structure called the placenta. The embryo develops organs and limbs through cell differentiation and by 8 weeks has become a foetus. The placental connection to the mother enables the transfer of oxygen and other nutrients through a capillary network directly into the bloodstream of the foetus. The nutrients for the foetus are provided by the mother’s body systems, and most of the wastes produced by the foetus are collected and removed by the mother’s body systems.

After around 9 months of pregnancy, the foetus has developed enough to survive outside the mother’s body. During birth, the uterus involuntarily contracts and, along with a lot of active pushing from the mother, the baby is pushed out of the uterus through the vagina.

2.3 Science for better health

SOLVING REAL-WORLD PROBLEMS

Scientific research in many different fields has led to discoveries that have solved important real-world problems relating to human health. The discovery of bacteria and other pathogens that cause infectious disease has led to ways of preventing these diseases, and ways to treat our food and waste to keep us healthy.

Table 2.2 summarises how some real-world problems have been solved or at least improved with the use of scientific discoveries.

TECHNOLOGY FOR BETTER HEALTH

Technology refers to the creation of instruments, machines and devices that can improve our lives and aid science investigations. Biotechnology is the application of science to living organisms.

Traditional biotechnology is involved in making cheese and bread by using microorganisms as discussed in chapter 1. Modern biotechnology is used to develop living things that meet our requirements based on our understanding of genetics and inheritance.

The development of artificial limbs is just one way that technology has helped humans that are suffering from disease or injury.

Artificial limbs and joints

Artificial limbs, or prosthetics, are devices used to replace a missing body part (usually an amputated arm or leg) in patients that have suffered a major injury, have an illness such as cancer or were born with particular defects. Artificial joints are used to treat patients with severe cases of arthritis or joint damage.

Improvements in prosthetics are a result of developments in material sciences and electronics. Materials must be strong, lightweight and free of pathogens. Artificial limbs are made out of titanium and carbon fibre. Artificial limbs are now individually shaped and dyed to match existing limb shapes and appearances.

Electronic advances even allow robotic artificial limbs to respond to nerve messages from the patient. The nerves that would have controlled the limb before it was amputated are surgically re-directed.

Electrodes are then placed at the re-directed site and transferred to electrodes in the artificial limb.

Influencing reproduction

There are many situations in which we wish to encourage reproduction. When a human couple wants to have a baby and encounter troubles, technology can assist. If a species of plant or animal is threatened with extinction, technology can also help. When certain features or characteristics are favoured in a plant or an animal, humans may use techniques such as selective breeding (see chapter 1) and biotechnology to influence the outcome.

Technology and human reproduction

Assisted reproductive technology (ART) is the name given to any procedure that is used to help a couple have a healthy baby. In vitro fertilisation (IVF) means that an egg is fertilised by sperm in vitro or ‘in glass’, meaning a test tube. This is done so a doctor can carefully watch every step to make sure the egg gets fertilised and begins dividing as it is supposed to. The tiny embryo can then be transferred back into the mother’s uterus to go through a normal pregnancy.

Unborn babies can also be screened for health problems. The amniotic fluid that protects the growing foetus can be tested, as can the cells of the placenta. The problem with these tests is that they involve inserting a needle into the uterus, which can result in an infection or may interfere with the pregnancy. Thankfully, many issues can be spotted in an ultrasound – a moving picture of what is going on inside, complete with the baby’s heartbeat.

Biotechnology

Recently, technologies have been developed so that characteristics can be transferred from one species to another by gene technology. The desirable characteristics of plants and animals that can be transferred include resistance to disease, higher concentrations of nutrients (such as vitamin A in rice), and resistance to insects. The use of biotechnology has led to cows that produce milk with built-in medicines, and bacteria that can produce human insulin for diabetics.

There are concerns about some of these experiments and the impacts of genetically modified organisms (GMO) on the environment, including how they are owned and controlled. As well as ethical concerns, there are worries about producing resistant species or the genes escaping into other organisms. Research into these potential impacts is ongoing.

DIFFERENT APPROACHES TO TREATING DISEASE

Science and technology have greatly increased our options of avoiding certain diseases or treatment if we become diseased or injured. This section will look at case studies involving kidney disease, heart disease and diabetes, and consider the different options available for their treatment.

Kidney disease

We cannot survive without kidney function. When the nephrons in the kidneys become damaged, the kidneys can no longer function efficiently and remove toxins such as urea from the blood. While we can survive with kidneys functioning at only 20% capacity, kidney failure requires ongoing dialysis treatment or a kidney transplant. Technological advances have increased the range of options for people with kidney failure. There is now a range of dialysis treatments, some of which can be carried out in the home and with minimal equipment. These treatments rely on osmosis and the diffusion of toxins into a solution that can be removed.

Option A: Prevention. Healthy diet and lifestyle, drinking water and avoiding salty foods, not smoking and minimal alcohol consumption, management of blood glucose (if diabetic) and management of blood pressure all help to prevent kidney disease for those at risk.

Option B: Dialysis. When option A is no longer a possibility and the kidneys begin to fail, dialysis is necessary.

Haemodialysis involves the use of a machine in a clinic or hospital for 3–5 hours three times a week to filter the blood and remove toxins, and then return the filtered blood back to the body. Another form of dialysis can occur more conveniently in the home and requires less equipment, but it must be carried out for longer periods.

Option C: Compatible organ transplant. A kidney may be transplanted from a compatible donor based on blood type. The donor may be alive or deceased. This treatment is medically preferable, as patients tend to live on average 15 years longer than those on dialysis, although the need for drugs to suppress the immune system may be a complicating factor for some people. Not all patients are able to receive a donated kidney for medical reasons. Also, it may take a long time for a compatible donor kidney to become available. Australia has a very low rate of organ donation. Kidney donors can live successfully with only one healthy kidney.

Option D: Non-compatible organ transplant. A kidney may be transplanted from a non-compatible donor. Additional medical treatment is required to help prevent the immune system from rejecting the transplanted organ.

Many factors impact on the decision about the type of treatment for kidney disease. Some of these include the effectiveness of the different treatments, cost and availability. Some religious groups also do not believe in receiving blood transfusions or organ transplants.

Heart disease

A heart attack is usually caused by heart disease, which is basically fatty deposits called cholesterol blocking important blood vessels in the heart (see Figure 2.66). The ‘attack’ occurs when the vessels become completely blocked or when a bit of the fatty deposit breaks off and travels into the heart. Heart muscle cells may be killed in the process.

In 1967, history was made in South Africa when Dr. Christian Barnard transplanted a human heart from a woman who had died in a car accident into a patient. The patient survived for 18 days. Since then, much has been learnt about tissue rejection. New drugs have been developed so that heart transplantation is a viable option for some people at risk of dying from heart disease. Shortages of heart donations have resulted in efforts to develop artificial hearts. Xenotransplantation, the transplantation of an organ from a different animal, such as a pig, has even been trialled. Issues with rejection of transplanted hearts are still reducing the long-term benefits.

There is more research to be done and many ethical issues to be debated.

Other new technologies include artificial heart valves, pacemakers, replacement of blocked arteries with bypass surgery, widening blocked arteries or the placement of devices called stents inside arteries.

Many of these surgical techniques depend on the use of medical imaging techniques, such as X-rays, MRIs and CT scans. Science has also contributed to the treatment of heart disease with the discovery of risk factors linked to the incidence of heart disease.

Option A: Prevention. The likelihood of heart disease can be significantly reduced by addressing the risk factors such as obesity, high blood pressure and high levels of cholesterol in the blood, and by living a healthy lifestyle with regular exercise, keeping a balanced diet and not smoking. Diets high in foods such as salmon, sardines, cod, walnuts and flax seeds also reportedly reduce the risk of heart disease.

Option B: Use of preventative medications. Some people take aspirin as a blood thinner, and there are prescription drugs available such as cholesterol-lowering drugs, but the effectiveness of these is still being investigated.

Option C: Surgical repair. Heart valves may be repaired or sometimes replaced. Replacement heart parts can be obtained from other organs of the patient, a deceased human donor, pigs or cows, or be mechanical. A device called a pacemaker may need to be inserted to restore the heartbeat rhythm. Diseased heart arteries can be replaced during bypass surgery, or stents can be inserted to repair the blocked artery.

Option D: Compatible organ transplant. A heart may be transplanted from a compatible donor based on blood type.

Option E: Rehabilitation. There are a number of rehabilitation therapies to help survivors of heart attacks and strokes recover and regain movement and other abilities.

Many factors affect whether people and groups in society adopt preventative measures against heart disease. If heart disease does occur, the decisions involve the treatment options available and what is considered acceptable. Death rates from heart disease have been declining, but some risk factors have been increasing.

Diabetes

Diabetes is a disease where the body cannot control the level of glucose in the blood. A person with diabetes will have high blood glucose levels either because their pancreas is unable to produce insulin (which controls the blood glucose level), or because their body cannot respond properly to the insulin that is released.

There are a variety of types of diabetes and factors associated with the onset of the disease. Type 2 diabetes has been linked to obesity and a variety of lifestyle factors, and its incidence is increasing. Diabetes can also develop during pregnancy.

Currently, 1.8 million Australians suffer from some form of diabetes. Type 2 diabetes does not always show symptoms and may go undetected until serious complications arise.

Diabetes was once a fatal condition, and even now it shortens life expectancy and can cause a wide range of complications.

These include heart disease, kidney disease, poor circulation and ulcers on the hands and feet, and eye problems causing blindness. Modern treatments can be a change of diet and lifestyle in mild cases, and the monitoring of blood glucose levels and injection of insulin in more severe cases. Insulin is now available as a result of genetic engineering so that bacteria actually manufacture human insulin. Previously, animal insulin was used and dose levels were much harder to manage.

Option A: Prevention. Prevention is especially important for type 2 diabetes. If there is a family history of type 2 diabetes, an active lifestyle and well- managed diet may help prevent the disease. Blood tests may detect diabetes that has not been diagnosed or is not showing any symptoms. Some high-risk people have surgical procedures to assist weight loss.

Option B: Insulin. Insulin injections, in addition to controlling diet and lifestyle, will maintain a relatively stable concentration of sugar in the blood.

Options are limited in the treatment of diabetes. Even with the use of insulin, modification to diet and lifestyle are the key to long-term survival.

Chapter 4 Body systems and responses

4.1 Coordination systems

THE NERVOUS SYSTEM

Your five senses – hearing, sight, smell, taste and touch – help you detect changes in the world around you, including danger. To survive immediate danger, you need quick responses. For example, when you trip or slip on something you may respond by throwing your arms out and trying to stay upright. This is to prevent damage to your body.

In addition to responding to the external environment, your internal environment must also be monitored and changes responded to. Receptors in your blood vessels detect the levels of dissolved carbon dioxide and oxygen in your blood, and can causes changes to your breathing rate to maintain the correct balance.

The nervous system is integral to the detection of and response to changes in internal and external conditions in the human body. The nervous system consists of the brain, spinal cord and nerve tissue that spreads throughout all parts of the body.

Humans are constantly receiving stimuli (incoming information about internal and external conditions) that they need to respond to. The nervous system makes it possible to detect and respond very quickly to certain stimuli. The nervous system uses electrical messages to transmit information.

Your body requires many responses at every moment of the day and night that rely on the coordinated and efficient working of the two main parts of the nervous system.

Your brain and spinal cord make up the central nervous system (CNS), which is responsible for processing the information received from the peripheral nervous system (PNS), which includes all the other nerves. The peripheral nervous system is responsible for detecting stimuli and initiating the response that comes from the central nervous system.

Nerves

The basic unit of the nervous system is a specialised cell called a nerve cell, or neuron. Scientists believe we may have up to 100 billion neurons in our bodies, connected in bundles called nerves.

Neurons have many highly specialised features, as shown in Figure 4.3 (on page 138).

Each neuron has a large cell body, where the nucleus of the cell can be found, which connects to a long thin axon on one side and to the dendrites on the other. Dendrites are nerve endings that branch out of the cell body. These highly sensitive, thin branches receive information and form contacts with the axons of other neurons, allowing the message to be transmitted. The axon carries the message away from the cell body towards another neuron or effector cell. The axons connecting your spinal cord to your foot can be up to 1 metre long! The messages that travel along neurons are called impulses. An impulse involves a series of electrochemical changes and movement of charged ions across membranes.

Just like electrical wires require plastic around them to insulate them, so do neurons. A fatty layer called the myelin sheath covers the axons. This helps to speed up a nerve impulse along an axon by controlling its path. People with the disease multiple sclerosis have damaged myelin sheaths. In people with multiple sclerosis, the nerve impulse is disrupted, blocked or able to escape, which causes issues with movement and problems with the senses.

The axon usually branches at the end to increase the number of connections with other neurons. At the end of each axon branch is a small bulb called a synaptic terminal.

Neurons communicate with each other and different types of cells at the junction where the two cells meet. The nerve cell and the other cell don’t actually touch – they are separated by a tiny gap called the synaptic gap. This point of communication is called the synapse, and includes the synaptic terminal of the neuron sending the message, the synaptic gap, and the dendrites of the next neuron receiving the message.

An electrical impulse cannot cross the synaptic gap. When an electrical impulse reaches the end of a neuron at the synaptic gap, the synaptic terminals release chemicals called neurotransmitters into the gap.

These chemicals diffuse across the synaptic gap and bind to the surface of the dendrites, which triggers a new electrical impulse in the second cell to continue the message.

Different types of neuron

There are three specialised types of neuron, all with different jobs:

Sensory neurons are sensitive to various stimuli, collecting information from either the body’s internal environment or the outside world. Sensory neurons send the information they have collected to the central nervous system for processing. One example is the specialised cells in the retina, which sense the intensity and colour of light and send messages to the brain so that we can see. You can see the photoreceptors of the eye in Figure 4.6.

Other sensory neurons include chemoreceptors that can detect certain chemicals in our olfactory organs and tongue, thermoreceptors that detect heat and cold and mechanoreceptors that detect pressure, pain and position. Sensory neurons are only found in the peripheral nervous system.

Interneurons link to sensory and motor neurons, as well as to other interneurons.

Interneurons only make connections with other neurons. They are also known as connector or relay neurons and are only found within the central nervous system, the spinal cord and the brain. They process incoming information and determine the responses.

Motor neurons carry messages from the central nervous system to muscle cells or endocrine glands throughout the body, which then carry out the response. Motor neurons are also known as effector cells. Motor neurons are only found in the peripheral nervous system.

THE CENTRAL NERVOUS SYSTEM

The central nervous system is the control centre of the body. All incoming messages from your environment, and your responses to them, are processed by the central nervous system. The two main parts of the central nervous system are the brain and the spinal cord.

The brain

The brain is the processing centre of the body. Its main functions relate to our survival. The brain is a soft, heavy organ, mostly made up of neurons, and surrounded by a tough skull to protect it. The brain receives information from the sensory organs and other receptors throughout the body about what is going on inside and outside the body. The brain then makes decisions about things like internal changes and movements. It is also home to memories, personality and thought processes. The brain connects to the spinal cord and also to 12 pairs of cranial nerves that connect directly to some internal organs and the face and head.

The human brain is easily the most complex and fascinating organ of any living thing.

Neuroscientists are learning a lot about how it works. They already know the brain is divided into different parts, each of which has a specific function but works with the other parts. Neuroscientists also know the brain demonstrates ‘plasticity’, which means it can change as you grow and develop and heal – previously thought impossible.

The brains of most complex animals follow the same basic structure as the human brain. Relative sizes of the different parts of the brain can be an indication of the complexity and intelligence of that animal.

Lobes of the brain

The cerebrum is the top layer of the brain and is where most of the processing is done. The cerebrum is divided into two hemispheres. They appear to be mirror images of each other but they do have some slight differences in structure and function. The hemispheres are joined in the middle of the brain by the corpus callosum, a bundle of neurons that enable the two hemispheres to communicate. Each hemisphere is then divided into four lobes or sections. These lobes have specific functions:

•The frontal lobe is located at the front of the brain. Its functions include emotions, reasoning, movement and problem-solving.

•The parietal lobe, found in the centre of the brain, manages the perception of senses, including taste, pain, pressure, temperature and touch.

•The temporal lobe is located in the region near the ears. It deals with the recognition of sounds and smells.

•The occipital lobe is at the very back of the brain. It is responsible for vision.

The spinal cord

If you have ever accidentally touched something very hot, you will remember how quickly you snatched your hand away, so quickly that you didn’t even have time to think about it– it was automatic. This involuntary and nearly instantaneous movement in response to a stimulus is called a reflex, or reflex action.

The spinal cord is a cylinder of nerves that runs from the brain through the vertebrae of the spine and branches out into 31 pairs of spinal nerves. During a reflex action, an impulse is passed along a sensory neuron to the spinal cord, where it crosses a synapse to a motor neuron. Activating motor neurons without having to wait for signals to pass through the brain allows reflex actions to occur quickly. Of course, the message is eventually sent to the brain so the brain can record what has happened. This explains why, after a fraction of a second after you pull your hand away from a hot stove, you feel the pain in your hand.

THE PERIPHERAL NERVOUS SYSTEM

The peripheral nervous system is a large system made up of all the nerves outside the central nervous system. The peripheral nervous system carries information to and from the central nervous system to the rest of the body, such as the limbs and organs.

The peripheral nervous system is divided into two parts:

•The somatic nervous system controls voluntary skeletal muscle movements, such as waving or reaching out to take something.

•The autonomic nervous system controls involuntary actions, which happen without our conscious control. This includes heartbeat, digestion, respiration, salivation and sweating. The autonomic nervous system maintains your body’s internal environment.

The autonomic nervous system also has two parts: the sympathetic nervous system and the parasympathetic nervous system.

The sympathetic nervous system is responsible for a group of responses that are often simply called the flight or fight responses. Stimuli that trigger these responses are usually unexpected or potentially life threatening. The main response of the sympathetic nervous system is to activate the endocrine system to produce a chemical called adrenalin. Adrenalin travels around the body in the bloodstream and causes many other responses, including increased heart rate and blood pressure, and the release of stored glucose into the bloodstream.

The parasympathetic nervous system is responsible for returning the body back to its normal state after a period of stress. So the sympathetic and parasympathetic systems often have opposite effects. For example, the parasympathetic system slows down the heart rate, whereas the sympathetic system speeds up the heart rate. Together, the systems maintain a relatively stable internal environment for the body.

THE ENDOCRINE SYSTEM

The endocrine and nervous systems are the systems largely responsible for sensing and responding to the environment. Part of this important job is communication: once a change or threat has been detected, messages must be sent around the body to coordinate a response. The nervous system sends very fast electrical messages, and the endocrine system uses chemical messengers called hormones to maintain a stable internal environment and to regulate growth and normal functioning. These chemical messengers usually act more slowly than the nerve impulses sent around by the nervous system, but their effects often last for a lot longer.

The endocrine system is made up of glands that secrete (make and release) hormones. These glands are shown in Figure 4.16. The glands and organs of the endocrine system are in different places throughout the body (Table 4.1). The hormones are usually secreted into the bloodstream and then travel through the blood to arrive at a target organ.

How does the hormone know where to go? Different hormones have a different three-dimensional shape that bind to a matching receptor on the target organ or tissue, working like a lock and key. Only the hormone whose shape exactly matches the receptor can bind to it.

Types of hormone

Hormones are classified into two main types based on their chemical structure: peptide hormones and steroid hormones. One example of each type is shown in Figure 4.18. Peptide hormones are made from proteins and are produced by the anterior pituitary, parathyroid gland, placenta, thyroid gland and pancreas. Most hormones are peptide hormones. Peptide hormones travel through the bloodstream until they find and interact with specific receptors on the surface of their target cells.

Hormones at work

Hormonal effects are often controlled by **feedback mechanisms**. When a hormone (a messenger) is sent out into the body, information is received about what is going on. This then affects other responses by the body. The rate of hormone production and secretion is often regulated by a **negative feedback mechanism**. If a stimulus is received indicating that a hormone-controlled effect is happening ‘too much’, the response would be to produce less of that hormone to reduce the effects. Negative feedback also works the other way, where the stimulus detects that a condition in the body is ‘too low’, so the response is to increase that condition. The ‘negative’ part of this response really means to respond in the opposite way, rather than to always reduce it.

Steroid hormones include hormones secreted by the adrenal glands and the ovaries (women) or testes (men). Steroid hormones are produced from cholesterol and are lipid-soluble, which means they can pass thorough the cell membrane and move directly into the target cells.

**Positive feedback mechanisms** are far less common. Oxytocin is a hormone that causes uterus muscles to contract in childbirth. The contractions result in more oxytocin because of positive feedback.

When things go wrong in the endocrine system

Disorders and diseases of the endocrine system are fairly common, and are often due to imbalances in **feedback mechanisms** within hormonal systems or problems with the production of hormones or their receptors. Diabetes, thyroid goitre and some forms of obesity are all caused by imbalances in the endocrine system.

Diabetes is one of the more serious and common results of hormone imbalance. Left untreated, it can result in blindness, kidney failure, heart disease or death. Diabetes occurs when the pancreas either produces too little insulin or doesn’t properly use the insulin it does produce, resulting in high blood sugar levels. Insulin is the hormone that assists the body to process sugar in the bloodstream. A message that blood sugar is low results in less insulin being produced. The opposite happens when blood sugar is high.

A goitre occurs when the thyroid gland, which is in the neck, becomes enlarged. The thyroid gland needs iodine to produce thyroid hormones. If a person’s diet is low in iodine, the thyroid gland is not able to produce the hormones. The gland enlarges as it tries to make more thyroid hormones. An underactive thyroid gland can also produce a goitre.

4.2 Responding to change

RESPONDING TO EXTERNAL CHANGE

Our bodies are constantly responding to changes. A stimulus (the plural is stimuli) is any information an organism receives that might cause it to respond. Responding to a stimulus can prevent major changes to the internal environment that can cause the organisms to become ill or possibly die. The easiest stimuli to identify are those we detect with the major sense organs: the eyes, ears, tongue, nose and skin.

Sight

Sight tells us more about the world than any other sense. The pupil of the eye changes size to control how much light enters the eye. Light entering the eye forms an upside- down image on a layer of tissue called the retina at the back of the eye. Sensory neurons on the retina called photoreceptors transform the light into nerve signals, which are transferred to the brain via the optic nerve. The brain then interprets the information, telling you what you are seeing.

Hearing

Imagine the strumming of a guitar. This action sets off a wave of vibrating particles in the air. These vibrations enter your ear and cause the eardrum to vibrate. The vibrations are transferred along the tiny bones of the middle ear and converted into nerve impulses by sensory neurons called mechanoreceptors. The brain then interprets these impulses, telling you what you are hearing.

Taste

Your tongue is covered in thousands of tiny tastebuds, which contain a type of sensory neuron called chemoreceptors that react with chemicals in foods. Tastebuds can recognise five kinds of taste: sweet, salty, sour, bitter and a ‘savoury’ taste called umami. When eating or drinking, the information from the taste receptor cells is sent to the brain, telling you what flavours you are tasting.

Smell

Chemoreceptors in the nose detect airborne chemicals and then send messages to the brain, which interprets the message and tells us what we are smelling. Smell is closely linked to taste. If this seems strange, think about the last time you had a bad cold and a blocked nose. Did it affect your ability to taste?

Touch, temperature and pain

As well as forming a barrier between us and the outside world, skin is also able to detect touch, temperature and pain. The bottom layer of skin, called the dermis, contains sensory neurons that can detect temperature (thermorceptors), pressure (mechanoreceptors) and pain (pain receptors). Information is collected by these neurons and sent to the brain for processing.

Responses to stimuli

Here are some examples of responses caused by stimuli:

•External temperature changes result in shivering to keep us warm or sweating to cool us down.

•Pressure on a part of our body might be light and ticklish, or strong and painful; it might reduce blood circulation or simply make us look at what is causing it.

•Light might make us squint, close our eyes or look towards its source.

•Sound might makes us look towards its source, follow an instruction or cover our ears.

Responses to stimuli in other animals

Other animals also sense and respond to stimuli, often in similar ways to humans. However, some animals such as the platypus have very special and unique ways of sensing things.

Plant responses to stimuli

Humans and most animals detect changes to their external environment by using their senses of sight, hearing, taste, touch and smell. Plants also have ways of sensing and responding to their environment.

Odours may cause a plant to flower or fruit to ripen. Chemicals in bushfire smoke promote the germination of some seeds.

Flowering plants respond to the direction of incoming light by growing towards it because of the production of hormones that cause cells to divide and grow in certain areas and prevent it in others. The hairs on the leaves of a Venus flytrap plant respond to pressure when an insect brushes past and the leaves respond by closing to trap the insect.

Plant responses are often called tropisms because they affect the way the plant grows or develops. Some of the more common plant tropisms are listed in Table 4.2.

RESPONDING TO INTERNAL CHANGE

Our bodies experience internal changes every day. Requirements for oxygen and for removal of carbon dioxide from our muscle cells when we are sleeping are different to those when we are exercising. If we have eaten sugary food, the production of insulin by the pancreas may be very different to when we eat meat. Our cells can only survive in a narrow range of temperatures. Sometimes our bodies generate too much heat, and this heat has to be removed.

The process of maintaining a stable internal environment in the body is called homeostasis.

Responses to changes in our internal environment are automatic and regulated by the autonomic nervous system. However, they often involve the interactions of a range of body systems. One of the more obvious interactions is between the endocrine system and the circulatory system. Hormones are produced by glands and organs in the endocrine system, but they are carried around the body to their target tissues by the circulatory system.

Multicellular organisms have an amazing combination of cells, tissues, organs and systems working together to maintain a stable internal environment. The different systems communicate via the nervous and endocrine systems, which are constantly monitoring the functioning of all the other systems.

Flowering plants rely on coordinated responses to changes in the internal environment. The root and shoot systems need to work together to ensure that cells get their requirements for photosynthesis and respiration, wastes are removed and excess food is stored appropriately.

Body systems and nutrition

Cells require nutrients such as glucose for energy, amino acids, minerals and lipids (fats) for growth and repair, and vitamins for cell functioning. We obtain these nutrients by eating and drinking. Food and drink are broken down in the digestive system into forms that the body can use.

Hunger and thirst are impulses controlled by the nervous system. Some parts of digestion, such as chewing and swallowing, are under conscious control. Most other processes in digestion, such as peristalsis and the secretion of digestive juices, happen automatically through the coordinated action of the nervous and endocrine systems.

Nutrients from food are carried to cells by the circulatory system. The nutrients are the result of the digestive system breaking down food so it is absorbed into the blood, mainly through the small intestine. Not all nutrients are required in the proportions absorbed at the time of digestion, so our bodies have a system of processing and storage. The liver plays a large part in providing stored nutrients when we need them. Some nutrients, such as soluble vitamins, cannot be stored, and so we need to eat foods that contain them on a regular basis.

Body systems and oxygen

The respiratory system is responsible for the absorption of oxygen into the blood. The circulatory system then works in a coordinated way to deliver oxygen to the cells. Haemoglobin in the blood carries oxygen from the respiratory organs to the rest of the body. Iron is an essential part of haemoglobin, so digesting and absorbing iron from our food is very important for oxygen absorption.

Breathing and heartbeat are controlled by the coordinated functioning of the nervous and endocrine systems. We can consciously control the muscles of the ribs and diaphragm to breathe, but when we are not thinking about it or are asleep, these functions occur unconsciously because of the autonomic nervous system. Hormones released by the endocrine system can increase the rate of breathing and heartbeat in readiness to deal with sudden frights, pain and injury, or strong emotions.

Body systems and water

Water enters the body through the stomach and the small and large intestines of the digestive system. It leaves in perspiration, urine, faeces and the air we breathe out. The excretory system, through the action of the kidneys, ensures that water balance, blood pressure and blood volume are maintained. Water also makes up a large proportion of blood plasma (fluid).

If the body is dehydrated, more water is absorbed in the lower intestines, the faeces become hard and dry, and constipation may result.

Body systems and wastes

Waste products of the cells are carried away by the circulatory system. The respiratory system works with the circulatory system to ensure carbon dioxide is expelled from the lungs. The kidneys in the excretory system ensure wastes such as urea are filtered out of the blood and expelled in the urine. The kidneys rely on blood pressure to filter efficiently, so they depend on the circulatory system and the absorption of water through the digestive system to provide the pressure.

4.3 Responding to disease

INFECTIOUS DISEASE

A disease is a problem with the body that stops it from functioning as it should. There are two main types of disease: infectious and non-infectious. Non-infectious diseases are not contagious, and cannot be caught from another individual. Illness such as heart disease or diabetes, are called non-infectious diseases.

Infectious diseases are those that can be spread between individuals. Infectious agents or pathogens cause infectious diseases. There are a number of different types of pathogen, including the virus that causes the common cold and bacteria that can give you food poisoning. Virulence is a measure of how easily an infectious disease is passed on to others. The spread of some diseases can be halted or slowed by washing your hands and being careful when handling food.

Pathogens

Pathogens can vary in type and size, from large molecules to viruses, unicellular microorganisms, larger multicellular macroparasites and fungi. Prions are responsible for rare, fatal untreatable brain diseases such as kuru and Creutzfeldt-Jakob disease (mad cow disease). Prions are not organisms – they are ‘faulty’ protein molecules that convert normal cellular proteins into the infectious form as they come in contact. There is still much research to do before we fully understand prions.

Bacteria are prokaryotic (without a true nucleus or membrane-bound organelles), unicellular microorganisms. Very few bacteria are pathogenic and some bacteria are vital for the proper function of our bodies. Some bacteria live on our skin and fight ‘bad’ bacteria before they enter our bodies. These bacteria and other microorganisms that help keep us healthy are called our natural flora.

Table 4.3 shows the different types of pathogen and an example of the types of disease they cause.

Scientific research and infectious diseases

Research in many aspects of science helps society find ways of reducing or curing a variety of diseases.

Dieback in Australian plants

The fungus Phytophthora cinnamomi was accidentally introduced into Australia a long time ago. It lives in the soil and plant roots and is spread naturally in ground water, causing a condition in plants called root rot or dieback. Recent human activity such as logging has increased its rate of spread, with disastrous effects on many native plant species as well as on some ornamental plants. Plants such as the Wollemi pine, waratah and grass trees are particularly at risk.

Dieback is common in Western Australia. It is also of concern in the national parks of New South Wales. Botanists at the Royal Botanic Gardens in Sydney and officers from New South Wales National Parks and Wildlife have collaborated to run research and education programs to try to reduce its spread. These include ways to avoid spreading it through human activities such as carrying infected soil on shoes.

Avian influenza

Viruses are challenging to researchers because they can change into different forms that make vaccines obsolete (no longer effective). In recent years, the movement of viral diseases from animals to humans has become of concern.

The Spanish influenza (flu) between 1918 and 1920 killed at least 20 million people worldwide. It was the first human influenza pandemic caused by the avian (bird) virus (strain H1N1) adapting to humans. In 1997, the Hong Kong flu was the first time the deadly H5N1 strain was recognised. In 2004 and 2007, outbreaks of H5N1 resulted in the death or slaughter of hundreds of millions of chickensn across Asia.

In 2009, there was another pandemic of the H1N1 strain, called swine flu (the virus can also interchange with humans and pigs). The major concern is that the virus will remain in bird populations and suddenly change. It has the potential to become a form that can transmit directly from human to human, as happened in the case of the Spanish flu.

Quarantine reduces the risk of an outbreak of avian flu in Australia. CSIRO’s Australian Animal Health Laboratory conducted research into gene silencing, which can prevent the mechanisms that viruses use to take over their host’s cells. This research is in addition to research areas such as early detection of avian flu, development and testing of vaccines, and better understanding of the biology of the avian flu virus and its strains.

Devil facial tumour disease

The first photographs of Tasmanian devils with lesions and swelling on the face were taken in 1996. We now understand that the disease, called devil facial tumour disease, is caused by one of the few contagious cancers. Cancer is a disease caused by uncontrolled cell division.

Devil facial tumour disease is believed to be spread by biting. In areas where the disease is widespread, populations have declined by 95% and the Tasmanian devil is now protected and classified as endangered.

The total devil population is now believed to be about half of the population of 10 years ago. This disease was new to science and only occurs in Tasmanian devils. The drastic effects on the population have prompted a range of research, and there is still a lot more to learn.

Scientists are focusing on three areas in their research: monitoring the spread of the disease, learning more about the disease, and managing it to avoid extinction of the Tasmanian devils. Ways to avoid extinction include keeping infected populations separate and keeping a certain number of healthy Tasmanian devils isolated separately.

Professor Kathy Belov is a genetic researcher who has made a major breakthrough. She discovered Tasmanian devils lacked genetic diversity particularly related to helping fight infection. Her research focuses on finding populations where more diversity is found, and developing and trialling vaccines.

THE IMMUNE SYSTEM

The role of your immune system is to protect you against foreign bodies by physically stopping them from entering your body, and then identifying and attacking them if they do manage to enter. Your immune system has three lines of defence against disease, each with a different role.

First line of defence

The first line of defence against pathogens is to stop pathogens from getting inside the body. This is done by the skin and mucous membranes. Openings such as the eyes, nose and ears are natural gateways into the body and need to be protected. Hairs surround your eyes and are present in your nose and ears. They act like nets to catch pathogens as they try to enter your body. Tears, wax and mucus also line these areas, and help to trap, and in some cases kill pathogens.

Your skin itself is too thick for pathogens to pass through, and your natural flora kills most pathogens that land on it. However, any breaks or damage to your skin are prime entry zones for pathogens. Viruses, unlike bacteria, contain a protective coating that allows them to slip more easily through the first line of defence. If they or other pathogens get past the first line of defence, a second stage of defence is in place to stop them.

Second line of defence

If a pathogen manages to get inside our body, a general ‘seek and destroy’ approach is initially taken, regardless of the type or structure of the pathogen. This is a general or non-specific immune response that includes:

•blood clotting – to stop additional infection through skin damage

•inflammation – to increase the amount of blood (carrying white blood cells) reaching an infected area

•fever – to heat up the body and destroy pathogens that cannot survive in extreme heat

•phagocytosis – large white blood cells envelope pathogens and destroy them via enzymes.

White blood cells are cells produced by the body to destroy pathogens. An increase in the amount of blood reaching an infected area of the body, as a result of inflammation, means more white blood cells are available to attack the pathogen. The white blood cells may also release substances that increase the amount of fluid in the infected area, causing swelling.

White blood cells can only do their job if they can recognise the difference between body cells and pathogens. Every cell has chemical markers on its surface called antigens. Every cell in your body has the same antigens, which flag to white blood cells that they are ‘good’ cells. Pathogens will have their own specific antigens that are different from your body cells. White blood cells ‘read’ the antigens and recognise the pathogens as ‘non-self’ and attack them.

There are a few different types of white b blood cell. Each type does its own job but they all work together. Only some white blood cell types are involved in the non-specific immune response. These are called phagocytes (the term comes from Greek words meaning ‘cells that eat’). A phagocyte envelops a pathogen, and once inside the phagocyte it is destroyed with enzymes. This process is called phagocytosis and is shown below.

Third line of defence

Any pathogens remaining after a non- specific response are targeted according to their type. This is called a specific immune response.

The specific immune response creates antibodies. Antibodies are protein molecules that bind specifically to target antigens, like a key fitting in a lock. Antigens may be the pathogen itself or marker molecules on the surface of a pathogen.

When a person is infected with a pathogen, specific antibodies are produced to combat the pathogen. These antibodies remain in the blood long after the infection has been fought, sometimes for the rest of the person’s life. If the person is infected with the same pathogen again, the antibodies react immediately to attack and destroy it.

This is called natural active immunity. The body may take up to a week to make the antibodies needed to combat a new antigen. This is why recovering from an illness takes time. Once the body has learned how to make the particular antibody, it is more likely to be protected from reinfection in the future. If a person gets infected, but fights off the infection without getting sick, the person is said to be immune to that disease.

Unborn babies obtain some natural immunity by receiving some of their mother’s antibodies across the placenta. Antibodies are also passed to babies who are fed breast milk. This is called natural passive immunity. However, because the baby isn’t making those antibodies themselves, they are not replaced when those molecules break down. The baby’s immunity may drop after a few months of not breastfeeding until they make enough of their own antibodies.

One other way to acquire immunity is by ingestion (by swallowing) or injection with specific antigens. This is called vaccination, or inoculation. Vaccination is an example of acquired active immunity.

A vaccine can be:

•the dead pathogen

•a living but non-virulent (weak or non-infectious) form of the pathogen– the pathogen in this case is called attenuated

•antigens of the pathogen that have been separated from the pathogen itself

•the weakened or inactive toxin that some bacteria produce.

Through vaccination, a person is prompted to produce antibodies without the disadvantage of becoming ill, which usually leads to immunity. Vaccinations are often given as a preventive measure. For instance, the influenza vaccine is recommended for people over 65 years of age because complications from influenza can be life- threatening in older people. Vaccinations can also be given when there is an urgent need to provide immunity. Tetanus is a disease caused by a type of bacteria that lives on rusty objects. A tetanus vaccine is often given immediately after a tetanus-prone injury, such as an open wound caused by a rusty or dirty object, because tetanus can be fatal. The use of vaccinations has resulted in many diseases becoming extremely rare or, in the case of smallpox, eradicated.

Medicines

Medicines usually work in one of several ways:

•changing how cells work

•replacing substances missing from your body

•destroying microorganisms and abnormal cells

•reducing the symptoms of illness.

Before a medicine can be sold in Australia, it needs to be approved for use by the Therapeutic Goods Administration. This agency decides which medicines are available to you and whether the medicines can be sold without prescription, over the counter without having to see a doctor first.

NON-INFECTIOUS DISEASE

Invading pathogens do not cause non- infectious diseases. Non-infectious diseases are a result of things like poor diet or lifestyle choices, genetic mutations, poor mental health, ageing, cellular malfunction, or environmental factors. They are not contagious – you cannot ‘catch’ a non- infectious disease from someone. However, some may be passed down from parent to child in the case of genetic disorders like Huntington’s disease or cystic fibrosis.

Diet and lifestyle

The nutrients you supply your body can affect how well the individual cells do their job. The right balance of nutrients is very important. Too much or not enough of some nutrients can cause diseases like scurvy, type 2 diabetes, heart disease and obesity. Smoking or excessive alcohol consumption can lead to cancers, liver failure and increased likelihood of accidents and injury.

Genetic disorders

All genetic disorders are a result of a mutation in the DNA or chromosomes at some stage. If these mutations occur in the DNA of sperm and eggs, they can be passed on to future children. Some common genetic diseases and disorders include:

•haemophilia

•cystic fibrosis

•sickle-cell anaemia

•muscular dystrophy

•Down syndrome

•fragile X syndrome.

Environmental factors

We are exposed to toxins, carcinogens (cancer-causing substances) and radiation on a daily basis. Some of these are harmless in very small amounts, but with prolonged exposure or extreme exposure they can cause non-infectious disease.

Nuclear reactions release radiation such as gamma rays. We are all exposed to some background radiation, but also may be exposed to X-rays in some medical procedures. But what does radiation actually do to us?

Radiation is energy that can make and break chemical bonds, and which can interfere with the molecules in your body’s cells such as DNA.

The nucleus of each of your cells contains the instructions for every task and substance required for healthy functioning. The instructions take the form of the molecule DNA (deoxyribonucleic acid).

Any change to these instructions can result in damage, which may be major or minor depending on where the change occurs.

DNA is vital for the reproduction of all cells, not just the ones that make new organisms. Many of your other cells need to be regularly replaced and cells need to be reproduced for healing to occur.

Your body is able to protect itself from harm and repair or to destroy faulty cells. However, sometimes parts of the body’s cellular systems don’t function as they should. Tumours are faulty cells that continue to multiply, replicating the fault with each cell division. These tumours can cause physical blockages in the body or interfere with certain chemical processes. Location, size and type determine whether a particular tumour is considered malignant (cancerous) or benign (not cancerous). Tumours that continue to grow and spread, forming new tumours, are considered to be cancers and can be fatal without treatment.

Some, but certainly not all, types of cancer are caused by radiation. Ionising radiation can also be used to diagnose and treat cancer.

During radiation treatment of cancer, high doses of radiation are focused to destroy the cells of a tumour. Using radiation to diagnose and treat cancer has some risks, but it is relatively non-invasive and can give people with cancer a greater chance of successful treatment.

**Textbook 2: NSW Pearson Biology Year 11**

4.1 Cellular arrangements of organisms

Cells carry out all the functions necessary to sustain life, including obtaining nutrients and water, exchanging gases, sourcing energy, removing waste products and reproducing. In this section, you will explore the differences between unicellular, colonial and multicellular organisms, the origin of multicellularity, and the advantages and disadvantages of being multicellular.

UNICELLULAR ORGANISMS

The first life forms on Earth were unicellular (single-celled) organisms that arose more than 3.8 billion years ago. In unicellular organisms, such as the prokaryote Escherichia coli and the eukaryote Euglena (Figure 4.1.1), a single cell must carry out all functions, including obtaining nutrients, exchanging gases, removing waste and reproducing. In colonial and multicellular (many-celled) organisms, these functions are shared between different individuals or different types of specialised cells. Unicellular organisms can live together in groups; however, each cell is still capable of breaking away and living individually. Unicellular organisms are examined in more detail in Chapter 2.

MULTICELLULAR ORGANISMS

A multicellular organism is like a community of cells that work cooperatively for the survival and reproduction of the organism. All multicellular organisms consist of eukaryotic cells. There is an enormous diversity of multicellular organisms, from simple mosses through to complex flowering plants, birds and mammals.

The earliest known multicellular animals are the Ediacaran animals (Figure 4.1.2). They are named after the Ediacara Hills in the Flinders Ranges of South Australia, where their fossils were first discovered. Ediacarans evolved more than 600 million years ago. Some resemble modern sea jellies or segmented worms. Others are unlike any other known organisms.

For an organism to be considered truly multicellular, it must have multiple specialised cells that are responsible for specific functions (one of which must be reproduction). In addition, its cells must:

have the same DNA (except for the reproductive cells)

be connected and must communicate and cooperate to function as a single organism

be dependent on each other for survival.

Colonial organisms

A colonial organism is a special form of multicellular organism that consists of rnany individuals living together. There are two types of colonial organisms: those that form a facultative colony and those that form an obligate colony. Facultative colonies are usually independent organisms that aggregate together to form complex social structures that increase the chance of survival, for example honey bees. Obligate colonies consist of individuals called zooids that vary in forrn and carry out specific functions for the organism to survive. Individuals in an obligate colony are dependent on one another for survival and reproduction and are sometimes physically connected. Examples of obligate colonial organisms are parasites, which need a host to complete part of a life cycle, or sea jellies, which are made up of individuals with specialised roles (e.g. digestion or reproduction).

Prokaryotes are not multicellular organisms. However, some bacteria, such as cyanobacteria, grow in chains of cells. Others form aggregates or colonies of cells that behave in a coordinated fashion, such as species that form biofilms.

ADVANTAGES AND DISADVANTAGES OF MULTICELLULARITY

Almost all the life forms you can see around you are multicellular, eukaryotic organisms. The microscopic prokaryotes that surround you are invisible to the naked eye, yet they are incredibly abundant, diverse, survive in a remarkable range of environments and have been around for billions of years. Despite the success of prokaryotes, multicellular organisms continue to evolve and thrive, suggesting that being multicellular with specialised cells must have its advantages. Table 4.1.2 lists some advantages and disadvantages of multicellularity and cell specialisation.

The advantages of multicellularity outweigh the disadvantages. It is not surprising that evolution has favoured complex multicellular organisms that are organised into tissues, organs and organ systems.

4.2 Levels of organisation in multicellular organisms

A multicellular organism can consist of many trillions of cells, which vary in size and function. Different types of cells have different organelles in different numbers. The number and types of organelles in a cell are related to the cell's function. For example, a muscle cell that uses a lot of energy will be packed with energy-producing mitochondria. Organelles carry out functions that are necessary for the cell's survival, while cells play important roles in the structure and function of tissues, organs and systems. All these components work together to promote the survival and reproduction of the whole organism. A mammal, such as a lion or human, has hundreds of different types of specialised cells (Figure 4.2.1), including muscle cells, red blood cells, bone cells and nerve cells.

While multicellularity has many advantages, it also has several disadvantages (Table 4.1.2, page 192). An individual muscle cell can shorten, yet on its own could not possibly bring about movement in a large organism. The same muscle cell requires nutrients and oxygen and produces waste. To ensure that cells can carry out their functions correctly and maintain healthy systems, the organism must expend energy finding the resources to fuel all its cells.

In this section, you will take a closer look at multicellular organisms and learn how they .are organised to overcome these challenges and take full advantage of multicellularity.

LEVELS OF ORGANISATION

A cell must be able to obtain nutrients and remove waste, and physical conditions such as temperature, solute concentration and pH must remain within the tolerable limits of the cell. If any of these conditions are not met in a multicellular organism, cells could die.

One advantage of being a large, multicellular organism is that most body cells are isolated from the external environment by a protective outer layer called the epidermis. This outer layer provides a buffer against changes in the external environment, allowing conditions on the inside of the organism to be maintained at suitable levels for cells to function efficiently.

However, the isolation of the internal environment from the external environment means that most cells do not have direct access to their essential requirements, such as oxygen. It also means that wastes expelled from the cells need to be removed from the internal environment so that they do not accumulate. As an organism increases in size and complexity, its cells must be organised to allow greater cooperation and coordination. The term 'organism' is derived from the French word 'organisme', which means 'organise'.

Depending on their complexity, multicellular organisms can be organised into the following levels to meet the needs of the entire organism:

• organelles

• specialised cells

• tissues

• organs

• systems.

Organelles

Cells vary in the number and type of organelles they have, based on the cell's function (Figure 4.2.2). For example, animal cells that require large energy reserves will contain more mitochondria than other cells. Plant cells that require additional water to maintain their shape may possess larger vacuoles than other plant cells. And chloroplasts, the organelles that carry out photosynthesis, are only found in plant cells that photosynthesise, such as leaf cells. Organelles are covered in detail in Chapter 2.

Specialised cells

Specialised cells are cells that have a specific function. All cells are adapted to perform different jobs in a multicellular organism, and have unique structural adaptations that enable them to carry out these functions. These specialised cells are the building blocks of complex tissues and organs in multicellular organisms. Examples of specialised cells in plants are root hair cells, which absorb and transport water; leaf palisade cells, which absorb light for photosynthesis; and leaf guard cells, which open and close to regulate gas exchange. In animals, specialised cells include myocytes (muscle cells), erythrocytes (red blood cells), epithelial cells and neurons. You will learn more about specialised cells in Section 4.3.

Tissues

Specialised cells are organised into tissues. A tissue is a group of similar cells working together to carry out a specific function in a multicellular organism. For some organisms, this level of organisation is sufficient to meet all its needs. As organisms become more complex, tissues alone may not be enough to carry out all the tasks required. In these cases, tissues have evolved to group together in distinct structures called organs.

Organs

An organ consists of two or more tissues that work together to perform one or more specialised tasks. An organ is commonly recognisable as a distinct structure. Examples of organs are flowers, leaves and roots in plants; and the heart, liver and brain in mammals.

Systems

In multicellular organisms, an organ rarely functions independently of other organs. Instead, organs form systems. A system is a group of organs that work together to perform a vital task, such as the circulatory and respiratory systems in humans.

Organisms

The final level of organisation is the organism itself. In a complex animal, systems work together and contribute to the successful functioning and reproduction of the whole organism.

ORGANISATION IN SIMPLE MULTICELLULAR ORGANISMS

Some multicellular organisms are organised only at the cellular level. This includes simple multicellular organisms, such as sponges. These animals are considered tissue-less multicellular organisms, because their cells are not organised into discrete, functioning systems within the organism.

Although simple multicellular organisms are more complex than unicellular organisms, they can survive without organising their cells into true tissues and organs, because they are often only a few cells thick (Figure 4.2.6).This means that materials can easily diffuse into, out of and between cells. This lack of organisational complexity also means that many simple multicellular organisms, such as sponges, can regenerate. They can build new limbs or even an entirely new organism from just a tiny piece of their body or a single cell.

In sponges, the body is hollow and consists of two layers of eukaryotic cells separated by a jelly-like substance. The outer layer protects the sponge and contains tiny pores through which water and food can enter. Sponges are filter feeders, filtering plankton, bacteria, dinoflagellates and many other microscopic organisms from the water around them. Digestion is carried out within food vacuoles inside the cells of the sponge. The inner layer consists of several cell types, including collar cells and amoebocytes.

Sponges have some unique methods of defence against predators and disease­causing organisms. They produce toxins that prevent predators from eating them, and powerful antibiotics that fight infections from bacteria. Scientists are studying these chemicals as possible new medicines for human use. Cnidarians (sea jellies and anemones) have specialised cells for defence and capturing prey. These cells are called cnidocytes or nematocytes, and are found along the tentacles of cnidarians (Figure 4.2.7). A thread is fired from the cnidocyte, which wraps around and traps prey. Some species, such as sea jellies or anemones, have cnidocytes that contain toxins for stinging and paralysing prey.

Despite the simple organisation of these organisms, each of the different cell types found within them has a specialised function that contributes to their survival and reproduction (Table 4.2.1).

ORGANISATION IN COMPLEX PLAN TS

Complex plants are those that have specialised tissues for transporting water and nutrients. These plants are called vascular plants, and the specialised tissues are known as vascular tissues. Non-vascular plants, such as algae and mosses, do not have vascular tissue or true organs. Instead, they have simplified tissues and absorb water directly through their cell walls, transporting it between cells via osmosis.

The absence of vascular tissue in non-vascular plants also limits their size, due to the lack of structural support and limited area over which they can transport water and nutrients.

In comparison, a cellular level of organisation cannot meet the needs of larger and more complex organisms, such as vascular plants. Consequently, cells in complex plants such as angiosperms (flowering plants) and conifers are organised into higher levels of organisation: tissues, organs and systems.

Specialised cells in complex plants

Some of the most important functions in vascular plants are involved in the transport of nutrients and water and acquiring energy via photosynthesis. Many specialised cells are found within the vascular tissue of plants for these functions.

Tissues in complex plants

The characteristic tissues in vascular plants (and the basis of this type of plant's name) are the vascular tissues, which are involved in the transport of water and nutrients throughout the plant. There are two types of vascular tissue: xylem and phloem. You will learn more about the tissues of plants in Chapters 5 and 6.

Organs in complex plants

The major organs of vascular plants are roots, leaves, stems, flowers and fruits.

Roots

Roots are responsible for absorbing and storing water and nutrients (mineral ions) from the soil. Roots also support and anchor the plant to the ground. Root systems are often very complex and can be much larger than the above-ground structures of the plant. The large root systems of many trees in nutrient-poor rainforest soils do not penetrate deep into the soil layers and instead grow above ground (Figure 4.2.8).

Leaves

Leaves are the primary organ of photosynthesis. Photosynthesis is carried out to convert light energy into chemical energy, which fuels the organism's cells. A flat shape and large surface area makes leaves well-suited to absorbing sunlight for photosynthesis. The major tissues that make up a leaf are the epidermis, photosynthetic tissue and vascular tissue. The vascular tissue (xylem and phloem) is visible as veins in the leaf structure (Figure 4.2.9).

Stems

The primary functions of stems are to:

• support the plant's leaves, flowers and fruit

• store nutrients

• transport water and nutrients between the roots and the shoots

• grow new plant tissue.

The stem is made up of three tissue types: dermal tissue, ground tissue and vascular tissue. The structure of stems varies widely between different species. For example, the stems of strawberry runners are flexible and fleshy, while the stem or trunk of an oak tree is thick and woody. Some stems are even edible, such as asparagus and celery stalks.

Flowers

Flowers are the reproductive structures of angiosperms. Flowers facilitate the fertilisation of the ovules (contained within the ovary) by the sperm (contained within pollen). The structures of many flowers are highly specialised to attract pollinators, such as bees, moths and fruit bats, which disperse the pollen from one flower to another. Other flowers produce pollen that is specialised for wind dispersal. Following fertilisation, the seeds develop and the surrounding ovary grows into a fruit.

Fruits

Fruits protect developing seeds and help them disperse from the parent plant. Fruits develop from the mature ovaries of flowers and often have a fleshy outer layer that surrounds the seeds. The outer structure of the fruit is often specialised to attract animals that aid in seed dispersal. Some animals, such as birds, eat the fruit and later excrete the seeds, while others disperse seeds that have attached to their fur. Examples of fruits are nuts, legumes, berries, peaches, tomatoes and oranges (Figure 4.2.9).

Systems in complex plants

Vascular plants have 1:\vo systems: the root system and the shoot system. The root system, which is usually underground, supports the structure of the plant and absorbs water and nutrients from the soil. The shoot system is made up of two parts: the non-reproductive (vegetative) parts of the plant, such as leaves and stems, and the reproductive parts, such as flowers and fruits.

ORGANISATION IN COMPLEX ANIMALS

The animal kingdom includes the most complex types of multicellular organisms. A cellular level of organisation is not enough to meet the needs of complex animals, so their specialised cells are organised into tissues, organs and systems.

Specialised cells in complex animals

Most complex animals are made up of hundreds of different cell types that are specialised to perform different functions. The roles of these cells are critical to the healthy functioning of the tissues, organs and systems of animals. Nerve cells or neurons (Figure 4.2.10) are an example of specialised cells in complex animals. These cells play essential roles in processing and transmitting signals throughout the body.

Tissues in complex animals

As described earlier, tissues contain specialised cells working together to complete a specific function. For example, a human red blood cell is perfectly adapted to absorbing and releasing oxygen as it travels around the body. However, one red blood cell cannot possibly carry all the oxygen that a human body needs. Trillions of red blood cells need to work together to meet the needs of a human.

Cells do not need to be identical to be considered a tissue; they just need to be working together to carry out a certain function. Blood, for example, is a tissue that consists of red blood cells, white blood cells and platelets all working together.

Tissues in complex animals are grouped into four main types (Figure 4.2.11):

•muscle tissue, formed by cells that can contract (e.g. skeletal and cardiac tissue)

•nerve tissue, consisting of highly specialised cells called neurons, which sense stimuli and transmit signals (Figure 4.2.10); this is essential for communication and coordination in complex multicellular animals connective tissue, forming the supporting and connecting structures of the body (e.g. bone and blood) epithelial tissue, formed by one or more layers of cells that cover most internal and external surfaces of the organism (e.g. skin and intestinal lining).

Organs in complex animals

An organ is a structure made up of two or more tissues that perform a specific function that cannot be carried out at a tissue level. Some of the many organs in complex animals include the eye, skin and heart. The largest internal organ in the human body is the liver, but the largest organ overall is the skin. The smallest organ in the human body-the pineal gland-is located within the brain.

The eye

The function of the eye is vision. Insects have compound eyes that consist of thousands of individual units called ommatidia (Figures 4.2.12 and 4.2.13). Each ommatidium is like a single eye. Collectively, they are oriented to receive light from different directions, giving an insect a very wide angle of view. Each ommatidium consists of a lens, crystalline cone, light-sensitive visual cells and pigment cells (Figure 4.2.13). The pigment cells ensure that light hits the visual cells at the correct angle. The visual cells transfer a message to the optic nerve, which transmits information to the brain.

Insect vision is quite different from that of humans. The image generated is more like a light and dark mosaic, rather than a sharp picture. Insect eyes can also detect very fast movement from a wide range of directions, allowing them to react very quickly when something moves towards them.

The skin

The skin is the largest organ of the human body. In an average adult, the skin is more than 1.8 m2 and makes up 6-10% of the body's total weight. Skin is considered an organ because it carries out several functions, including regulating temperature, preventing water loss and sensing the environment. It also provides a protective barrier and contributes to a stable internal environment for the other cells, tissues, organs and systems that make up a human.

Skin in humans is divided into three layers: epidermis, dermis and subcutis (the subcutaneous fatty layer) (Figure 4.2.14).

The epidermis is the outermost layer and consists mostly of keratinocytes. Keratinocytes are cells that contain keratin and create a tough, waterproof layer for the body. The outer layer of the epidermis consists of dead cells, which are continually replaced by dividing cells below. The thickness of this layer of dead cells varies enormously over the body, and is 10 times thicker on the soles of the feet than on the face.

The dermis has a rich supply of blood vessels that control blood flow through the skin, regulating the body temperature of the whole organism. Fibres of collagen and elastin in the dermis give the skin strength and elasticity. The dermis also contains nerves and receptors that sense external stimuli, such as temperature, pressure and touch. Some touch receptors are attached to hair cells, while pain receptors are close to the surface of the skin in the dermis or epidermis. Sweat glands aid in cooling by releasing a watery substance (sweat) onto the epidermis via pores. Sebum is released by the sebaceous glands to help keep the skin and hair cells pliable. Sebum is also thought to have a mildly antiseptic effect on bacteria, because some of its fatty acid molecules inhibit bacterial growth.

The final layer of human skin, the subcutis, consists mainly of fat cells. These act as a food reserve for the body, provide insulation and cushion physical impact.

The heart

The heart of a complex animal beats continuously throughout the life of the organism, transporting nutrients and oxygen to cells and helping remove carbon dioxide and other waste products of cells. In a single year, the human heart beats more than 30 million times.

The human heart has two separate pumps (Figure 4.2.15). The right-hand pump receives blood from the body and pumps it to the lungs. The left-hand pump receives blood from the lungs and pumps it to the body.

Blood moves from the right ventricle to the lungs. In the lungs, oxygen binds to the haemoglobin in the red blood cells, while carbon dioxide diffuses into the lungs and is exhaled. Oxygen-rich blood then returns to the heart via the left atrium and moves to the left ventricle, which pumps the blood under high pressure to the body via the aorta. Blood eventually returns to the heart via the right atrium, completing the circuit.

The heart has several tissues. Most of the heart is composed of cardiac muscle tissue, which contracts to force blood through the heart and out to the body. Connective tissue makes up valves, which ensure blood moves through the heart in the right direction. Nerve tissue controls the heart rate. The most important nerve tissue is the sinoatrial node, located in the right atrium. This node generates the electrical impulses that sweep across the heart, causing it to contract and pump blood. It is often referred to as the natural pacemaker.

Systems in complex animals

The organisation of cells into tissues may be enough to fulfil the biological requirements of simple animals, but more complex animals require further organisation of their organs. Systems are groups of functionally similar organs working together as a unit.

The grouping of organs into systems is the highest level of biological complexity. Mammals have 11 organ systems, each with specialised roles that are essential for the correct functioning of the organism (Figure 4.2.19). The systems do not work in isolation; they have vital connections to one another, and many of their functions overlap. Each of the systems ultimately functions to maintain homeostasis (internal stability) and ensure the survival and reproduction of the organism.

4.3 Cell differentiation and specialisation

While most cells within a multicellular organism will contain the same genetic material, they differentiate and specialise to perform different functions in different locations around the organism. This allows the organism to function more efficiently and increase its chance of survival and reproduction. In this section, you will learn more about the role of cell specialisation in multicellular organisms.

CELL SPECIALISATION

All multicellular organisms begin life as a single cell that resulted from the fusion of two highly specialised cells called gametes. These gametes are called the egg (or ovum) and sperm. Gametes are unique in being able to fuse together to form a single cell, called a zygote. This one cell contains all the genetic information required to develop into a fully functional multicellular organism. The zygote develops by cell division into an embryo (Figure 4.3.1). It is through cell replication and cell differentiation that one single cell can become the trillions of highly specialised cells that make up an organism.

Cell differentiation

Cell differentiation is the process by which unspecialised cells, called stem cells, become specialised cells. It takes place in all multicellular organisms. Stem cells are present in the embryo and some adult tissues of animals, and in meristem tissue in plants. Stem cells retain the ability to divide, while specialised cells cannot usually divide.

The process of cell differentiation begins shortly after fertilisation. Five days after fertilisation, tl1e human zygote becomes a blastula (called a blastocyst in mammals). This is the early stage of embryonic development, when cell differentiation begins. Embryonic stem cells originate in the blastula and make up the primary germ layers (the ectoderm, mesoderm and endoderm) that differentiate to form specialised cells, tissues and organs in animals (Figure 4.3.2).

In plants, cell differentiation and cell specialisation derives from cells in the meristem tissue. The meristematic cells are unspecialised embryonic cells at the tips of shoots and roots (Figure 4.3.3). Organs such as leaves and flowers develop from cells in the shoot apical meristem, while root growth comes from the cells of the root apical meristem.

The internal and external structure of a cell is the basis for the functions it performs. Examples of the structures and functions of specialised cells in plants and animals are shown in Table 4.3.1. Cell specialisation is an advantage, because cells are more efficient when they have only one function rather than many. This makes multicellular organisms much more energy efficient than unicellular organisms.

Specialised cells in animals include nerve cells, which are specialised to carry signals rapidly over long distances. They could not do this if they also had to break down food to obtain nutrients or protect against disease. Another example is a red blood cell, which is essentially a bag of haemoglobin that carries oxygen around the body. It cannot also protect the organism from an invading bacterium; this is the role of specialised white blood cells. In plants, different cells are specialised for photosynthesis, exchange of substances (with other cells or the environment) and fluid transport.

Gene expression

Gene expression is the process by which the information stored in genes is used to build the different structures in a cell. It determines how a cell will differentiate and function.

All the genes required to produce every type of cell needed by an organism are present in every cell after fertilisation. However, only some of these genes will be active (expressed) at any one time in different cells. For example, in developing red blood cells, the genes for haemoglobin are expressed, while in gland cells, genes that code for different hormones, such as insulin, are expressed.

In cancer cells, the genes that regulate normal cell growth and development have been altered. Scientists can observe these changes in gene expression using a technique called fluorescence microscopy. Fluorescent tags target specific genes, with the location and brightness of the fluorescence indicating the level of gene expression in different cells (Figure 4.3.4). Fluorescence microscopy is covered in more detail in Chapter 2.

An example of animal cell specialisation: your skin

The human body consists of about 210 different types of cells. Each of these cell types differentiates from unspecialised stem cells during embryonic development.

You learnt about the skin as an organ in Section 4.2. The skin's function relies on the many highly specialised cells within it. The epidermis alone has four different cell types: keratinocytes, Langerhans cells, Merkel cells and melanocytes (Figure 4.3.5). The cells of the epidermis also interact with many other cell types. Nerve cells, red and white blood cells, muscle cells and gland cells all contribute to the correct functioning of the skin cells in their role of protection, thermoregulation and sensation.

The structures of specialised skin cells can give you clues about their function. The keratin-producing keratinocytes are the most common type of skin cell, making up around 90 to 95% of the epidermis. The flat, scale-like structure of keratinocytes (also called squamous cells) helps them maintain the structural integrity of the skin, with the tight junctions between them creating an effective barrier. Keratinocytes also interact with nerve cells, antigen-processing Langerhans cells, sensory­processing Merkel cells and melanin-producing melanocytes.

5.1 Autotroph and heterotroph requirements

In this section, you will learn how organisms are divided into groups according to how they fulfil their energy requirements, and how they use available resources to survive and thrive in their environments. You will also examine the nutrient and gas requirements of autotrophs and heterotrophs. Heterotrophs have a variety of complex feeding patterns based on their energy requirements, and the location and availability of resources.

AUTOTROPHS

Autotrophs make their own organic compounds using energy and inorganic compounds from their environment, such as carbon dioxide and water. The process of converting inorganic carbon into organic compounds is called carbon fixation, because the autotroph 'fixes' the inorganic carbon into organic molecules such as glucose (Figure 5 .1.1). These organic compounds are then consumed by heterotrophs (Figure 5.1.2). Figure 5.1.1 shows the flow of energy from autotrophs to heterotrophs in an ecosystem.

Autotrophs can be further divided into two groups according to how they obtain the energy required for carbon fixation. These two groups are:

•photosynthetic autotrophs, which use light energy (photoautotrophs)

•chemosynthetic autotrophs, which use chemical energy (chemoautotrophs).

Photosynthetic autotrophs

Photosynthetic autotrophs (photoautotrophs) are organisms that obtain the energy required for carbon fixation from light or solar energy (sunlight). They combine carbon dioxide and water using solar energy to produce organic compounds in a process known as photosynthesis (Figure 5.1.3). In plants, the organic compound produced by photosynthesis is glucose. The process of photosynthesis is covered in detail in Chapter 3.

Most autotrophs are photosynthetic. The most well-known photosynthetic organisms are green plants, but there are also other types of photosynthetic organisms, such as algae, Euglena and cyanobacteria (Figure 5 .1.4).

Carnivorous plants, such as the Venus flytrap (Dionaea muscipula) (Figure 5. 1. 5), obtain some nutrients, such as nitrogen, potassium and phosphorous, by capturing and consuming other organisms. However, because they obtain most of their organic compounds through photosynthesis, they are considered photosynthetic autotrophs.

Chemosynthetic autotrophs

Chemosynthetic autotrophs obtain the energy they need for carbon fixation from inorganic chemical reactions-a process known as chemosynthesis. All known chemosynthetic organisms are prokaryotes.

Some chemosynthetic autotrophs obtain energy by the oxidation of inorganic molecules. Some of these conversions include:

ammonium ions (NH/) to nitrite ions (NO2-)

nitrite ions (NO2 -) to nitrate (NO3 -)

sulfide ions (S2 -) to sulfate ions (SO/-).

Chemoautotrophs can live in the more extreme environments where these ions can be found. For this reason, these organisms are often referred to as extremophiles. Methanogens are chemoautotrophs that live in environments where hydrogen is more readily available. They obtain energy from a carbon-fixing reaction in which carbon dioxide (CO2) and hydrogen (H2) react to form a simple organic compound: methane (CH4). Methanogens are poisoned by oxygen and live in places with very low oxygen levels, such as in the digestive tracts of animals and in wetlands (Figure 5.1.8).

Other chemoautotrophs include:

archaea that live off the carbon in coal (Figure 5.1.9a)

bacteria that convert sulfur to sulfate in deep sea thermal vents

various nitrifying bacteria that fix nitrogen gas from the air or convert ammonia to nitrite to nitrate ions underground, helping plant growth in the process (Figure 5.1.9b)

denitrifying bacteria that return nitrogen to the air

rock-eating bacteria that obtain energy from hydrogen in rock compounds and dissolved carbon dioxide in water trapped deep underground (Figure 5 .1. 9c) iron-oxidising bacteria located in lava beds and groundwater that form a sludge of reddish-brown iron rust

bacteria that decompose crude oil.

HETEROTROPHS

Unlike autotrophs, heterotrophs cannot carry out carbon fixation using light or chemical energy, and cannot use simple inorganic substances to make organic compounds. Instead, they obtain organic compounds by consuming other organisms or their products (Figures 5 .1.13 and 5 .1.14). Heterotrophs use the nutrients they consume to obtain their energy.

All heterotrophs depend directly or indirectly on autotrophs for nutrients and energy. For example, a swamphen eating a soft plant stem is using an autotroph directly as a food source. That swamphen might also eat an insect that feeds on the plant, and so also depends indirectly on the plant for food.

All animals and fungi are heterotrophs. Some bacteria and many protists are also heterotrophs. Because most heterotrophs feed on certain types of organisms (e.g. plants or animals), they can be further subdivided into groups based on their diet.

Photoheterotrophs

Photoheterotrophs are specialised prokaryotes that use solar energy, rather than organic compounds, as a source of energy. Unlike photoautotrophs, photoheterotrophs cannot fix carbon from CO2 into organic compounds such as glucose. They use organic compounds obtained from other organisms as their carbon source for growth and renewal, not as an energy source. Photoheterotrophic organisms include green non-sulfur bacteria, purple non-sulfur bacteria and heliobacteria (Figure 5 .1.15).

Chemoheterotrophs

Most heterotrophs are chemoheterotrophs. They obtain energy from organic compounds by a chemical conversion called cellular respiration. Animals, protists, fungi and most heterotrophic bacteria are chemoheterotrophs. The chemoheterotrophs can be further divided by their source of food.

Herbivorous heterotrophs

Herbivorous heterotrophs are animals that eat only plant material, and are known as herbivores. Kangaroos, horses, parrots, caterpillars and snails are all herbivores (Figure 5.1.16). Herbivores are examined in more detail in Section 5.3.

Carnivorous heterotrophs

Carnivorous heterotrophs are animals that eat only other animals, and are known as carnivores. Dingoes, eagles, crocodiles, sharks and spiders are all carnivores (Figure 5.1.17). Carnivores are examined in more detail in Section 5.3.

Omnivorous heterotrophs

Omnivores are organisms with a broad diet that can eat a mixture of both plants and animals. This distinguishes them from carnivores, which eat only animals, and herbivores, which eat only plants. Omnivores don't tend to specialise in a food source, but instead are opportunistic eaters, eating foods that are easily available to them. Humans, bears, and lizards such as the blue-tongued skink (Figure 5 .1.18) are all omnivorous. Omnivores are examined in more detail in Section 5.3.

Saprotrophic heterotrophs

Another group of heterotrophs are the saprotrophs, which include most fungi and some bacteria. Saprotrophs eat by digesting organic material by extracellular means. This means they secrete enzymes onto dead and decaying organic material, such as carcasses, leaf litter or fruit (Figure 5 .1.19). Once the enzymes have broken down the large molecules, the saprotrophic organisms absorb the simple organic nutrients through endocytosis. This process of decomposing and recycling organic matter is essential for ecosystems to function, because the process returns nutrients back into the environment to continue driving the cycle of energy.

Parasites

Parasitic heterotrophs, or parasites, derive their energy and nutrients directly from other living organisms. They feed on the cell contents, tissues or body fluids of their host. The host is usually harmed and sometimes even killed in the process. Parasites are highly diverse and can be found in all five kingdoms. Parasites that live inside the host are called endoparasites, and include tapeworms and liver flukes. Parasites that live outside the host are called ectoparasites, and include ticks and lice (Figure 5.1.20).

COMPARING HETEROTROPHS AND AUTOTROPHS

5.2 Autotroph nutrient and gas exchange systems

This section introduces you to the structures of a plant that are involved in obtaining nutrients and exchanging gases. The structure of a plant appears quite simple at first. But by delving deeper to the microscopic level, you will see that just like animals, plants are very complex organisms. The vascular tissue of a plant contains specialised cells and vessels for transporting materials from roots to leaves and all around the plant (Figure 5.2.3). This section allows you to explore the microscopic structure of leaves, stems and roots, which are essential for nutrient exchange. Photosynthesis is also discussed, allowing you to recognise the inputs and outputs required for successful gas exchange.

GAS EXCHANGE STRUCTURES

The ability of a plant to exchange gases with the environment is a vital element for its survival. Photosynthesis depends on interconnected variables, such as the amount of sunlight, water and carbon dioxide in a plant's environment. The exchange of gases via plants and animals is a reciprocal relationship; both organisms survive because of each other. Plants have many specialised structures that allow them to efficiently exchange gases with their environment.

Stomata

Gas exchange in plants occurs through a structure called the stoma (plural stomata) (Figure 5.2.4). The stoma is the opening to an air space located in the lower epidermis of a leaf (Figure 5.2.5). Each stoma consists of two highly specialised epidermal cells called guard cells. The guard cells surround a pore, creating an opening through the epidermis and cuticle.

Stomata play an important role in regulating the exchange of gases and water between a plant's internal and external environment. They do this by changing shape, which causes the pore to open and close. When plants open their stomata to allow carbon dioxide gas in for photosynthesis, oxygen gas is released, and water is lost as water vapour during the process of transpiration. Transpiration is the passive movement of water through a plant from the roots and its evaporation as water vapour through the stomatal pores in leaves. Transpiration involves the upwards movement of water against the force of gravity. The theory that explains this phenomenon is called the **transpiration-cohesion-tension theory**.

Stomata are usually open during the day to increase the rate of photosynthesis when sunlight is available. When the guard cells are turgid, or swollen, the stomatal opening is large, allowing water and gases to enter and exit the leaf (Figure 5.2.6a). The guard cells become swollen when potassium ions (K+) accumulate and the water potential of the guard cells decreases. When guard cells lose water, the cells become flaccid and the stomatal opening closes, preventing water and gas from leaving the leaf (Figure 5.2.6b). The stomata close when light levels drop and the plants do not need any more carbon dioxide gas for photosynthesis.

Chloroplasts

In the cells of eukaryotic autotrophs, photosynthesis occurs in the chloroplasts. Each chloroplast has an outer and an inner membrane, which together regulate the movement of materials into and out of the organelle. Inside these membranes is a fluid matrix called stroma and a highly complex inner thylakoid membrane system. Figure 5.2.7 shows that the thylakoid membranes fold to form flat hollow discs, which form stacks called grana. Each granum looks like a stack of coins. Between the grana are flat membrane sheets called thylakoid lamellae (singular lamella).

Chloroplasts and photosynthesis are examined in detail in Chapter 3.

A useful means of studying gas exchange and transport is by investigating factors that affect the rate of transpiration and the rate of photosynthesis. Simple experiments can be conducted in the laboratory or by using a virtual model. Scientists can use suspensions of isolated chloroplasts or green algae to test the effect of varying different factors on the rate of photosynthesis under controlled conditions.

PHOTOSYNTHESIS INPUTS: CARBON DIOXIDE, WATER AND LIGHT ENERGY

Photosynthesis is essential for autotrophs to fulfill their nutrient and gas requirements. If we look closely at the process of photosynthesis, we can predict that the rate at which it occurs will be affected by several factors. The main requirements of photosynthesis are carbon dioxide, water and light energy. If any one of these factors is in limited supply, it is reasonable to predict that the rate of photosynthesis will also be limited. The biochemical process of photosynthesis is covered in Chapter 3 and is reviewed here in the context of the nutrient and gas requirements of autotrophs.

Carbon dioxide

The carbon dioxide level in the air remains relatively constant. Therefore, the factors that affect the amount of carbon dioxide available for photosynthesis in most terrestrial plants are the number of stomata in the leaves, and whether these stomata are open or closed. If the stomata are closed, photosynthesis will use up the carbon dioxide available, reducing the carbon dioxide concentration in the leaves. With less carbon dioxide available, the rate of photosynthesis will be limited, even in the presence of light.

In the laboratory, it is possible to control the concentration of carbon dioxide to which plants are exposed without changing other factors. Figure 5.2.8 compares the rate of photosynthesis for a plant exposed to different concentrations of carbon dioxide at different light intensities.

Water

Because the amount of water used in photosynthesis is small compared with the amount needed to keep the cells alive, a living plant cell will normally have sufficient water for photosynthesis to occur. When water availability is low and a plant is suffering from water stress (Figure 5.2.9), the stomata in the leaf close to conserve water. This limits the amount of carbon dioxide that can enter the leaves for photosynthesis.

Water is essential for all life forms. In plants, water molecules are split to form oxygen gas and hydrogen ions. Plants harness the energy involved in the process of water splitting to drive photosynthesis. The hydrogen ions go on to produce adenosine triphosphate (ATP), and can also combine with carbon to produce glucose. Water is also an output of photosynthesis. You will learn in Chapter 6 that water is transported in the xylem of vascular plants from roots to leaves, where photosynthesis takes place.

Light energy

In the laboratory, chloroplasts can be extracted from plant cells and tested in isolation. By varying the amount of light shining on isolated chloroplasts while keeping the carbon dioxide levels constant, it is possible to measure the rate at which photosynthesis occurs at different light levels. The results of this experiment are shown in Figure 5.2.10, and present what is known as a light saturation curve.

The curve shows a steady increase in the photosynthesis rate with an increase in light intensity until a plateau is reached. The plateau indicates that there is a maximum rate at which photosynthesis can occur. Assuming unlimited amounts of carbon dioxide (and water), the limit will be the point at which all the photosynthesis systems and enzymes in the chloroplasts are working at their optimum rate.

In the natural environment, the amount of light available for photosynthesis will be determined by the amount of sunlight. Trees and taller plants shade plants on the forest floor, while the amount of light available to aquatic plants depends on how far underwater they grow (Figure 5.2.11). Sunlight will also vary during the cycle of a day and will change with the seasons and the weather.

PHOTOSYNTHESIS OUTPUTS: OXYGEN AND GLUCOSE

Oxygen

Oxygen is a product of photosynthesis and is often used as a measure of photosynthetic rate. The greater the amount of oxygen produced by a plant, the greater its rate of photosynthesis. In the laboratory, it is possible to measure the amount of oxygen produced by a plant and therefore infer its rate of photosynthesis. This experiment can be conducted under different environmental conditions to understand the environmental factors required for optimal rates of photosynthesis. The oxygen output of a plant is influenced by both the amount of oxygen produced by photosynthesis and the amount used by cellular respiration in the plant's cells.

Glucose

It is possible to directly measure the rate of mineral uptake in a plant by measuring the amount of glucose the plant produces, but this can be difficult to do.

An alternative, indirect way to measure glucose production is by measuring biomass. Biomass is the weight of organic matter derived from living, or recently living, organisms. To measure glucose production in this way, the plant tissue must be completely dehydrated before weighing to ensure the change in biomass represents only organic matter, and not water. The amount of glucose in a plant can also be indirectly estimated by measuring starch levels, because glucose is stored as starch. An iodine-staining technique turns starch purple and is a useful qualitative measure. A colorimeter can then be used to obtain a quantitative result.

VASCULAR PLANTS

Vascular plants include ferns, cycads, conifers and flowering plants. They usually grow in terrestrial environments, and are characterised by the presence of vascular tissue, which is specialised for transporting fluids. Vascular tissue will be discussed in detail in Chapter 6, but includes:

•xylem-transports water and inorganic nutrients (mineral ions) absorbed from the soil up the plant

•phloem-transports dissolved sugars produced by photosynthesis from the leaves throughout the plant, and organic substances such as amino acids.

Plant tissue is also organised into organs. Two of the major organs in plants are leaves and roots (Figures 5.2.14 and 5.2.15). In vascular plants, vascular tissue is found within both these organs.

Roots

The root tissue of plants is usually located in the soil and is not visible without extracting a plant from its soil bed (Figure 5.2.14). On close inspection of a root system, the fine detail and intricate structure of the tissue is clear. Roots have a critical role in anchoring a plant to the soil, as well as absorbing water and dissolved minerals from the soil for growth and photosynthesis. Roots also store glucose produced by the plant.

Roots have structural adaptations that help them absorb water. Examination of the anatomy of a root reveals highly specialised cells. The exterior of the root is the epidermis, composed of epidermal cells. Some of these epidermal cells have long, fine extensions called root hairs, which increase surface area and maximise water and mineral uptake. The next layer of a root is the cortex. The cortex is composed of parenchyma cells, which can store nutrients and starch. The innermost central region of the root contains the vascular tissue: xylem and phloem.

Leaves

In vascular plants, a leaf is an organ composed of three distinct layers of specialised cells, or tissues (Figure 5.2.16):

•upper epidermis

•mesophyll

•lower epidermis.

The epidermis is a layer of cells covering the entire leaf. It secretes a waterproof waxy layer called the cuticle. Together the epidermis and cuticle provide a barrier that protects the cells and tissues inside the leaf and prevents excess water loss. The epidermal cells lack chloroplasts, but are transparent, allowing sunlight to reach the photosynthetic cells below. Within the lower epidermis are the stomata. The stomata regulate the exchange of carbon dioxide, oxygen and water vapour between a plant's internal and external environment.

Between the epidermal layers are the mesophyll cells, where photosynthesis takes place. The cells closest to the upper epidermis are the palisade mesophyll cells. These cells contain many chloroplasts and are tightly packed together. The spongy mesophyll cells below the palisade mesophyll cells are loosely packed together, with air spaces between them to allow gas exchange. These cells contain fewer chloroplasts.

The vascular tissue (xylem and phloem) is also located between the two layers of epidermal cells. Vascular tissue is often visible in leaves as veins.

5.3 Obtaining nutrients: heterotroph digestive systems

Mammals are heterotrophs; unlike plants, they cannot make organic molecules from inorganic materials. Therefore, they must consume other organisms or their products to obtain organic molecules. As well as needing organic molecules to provide chemical energy, heterotrophs require other organic molecules, such as vitamins, amino acids and fatty acids. Their diet must also contain minerals and water. Chapter 3 examines cell requirements in more detail.

NUTRITIONAL REQUIREMENTS OF HETEROTROPHS

Carbohydrates and lipids

Carbohydrates are an important source of immediate energy for all living organisms. The monosaccharide glucose is broken down to produce ATP during cellular respiration. Animals store carbohydrates in the form of the polysaccharide glycogen.

Lipids are also an important energy store in animals, and they are required for cell membranes, hormones and vitamins.

Amino acids

Amino acids are required for protein synthesis. Animals cannot make all the amino acids they need, but can change some amino acids into others. However, nine amino acids cannot be made in this way (Table 5.3.1). These are called the essential amino acids, because they must be included in the diet. Because amino acids are not stored, all required amino acids must be present in the blood for protein synthesis to proceed smoothly. This means that all essential amino acids should be eaten regularly to maintain their levels in the blood.

All nine essential amino acids are found in milk, eggs and meat. Wheat, corn, rice and other grains contain very little lysine. Beans, lentils and other legumes are rich in lysine but contain little methionine, so it is good to eat grains and beans together. Other sources with moderate to high amounts of amino acids are shown in Table 5.3.1.

Vitamins and minerals

Vitamins are a diverse group of organic compounds made by plants and by some simple animals and microorganisms. They are not used to supply energy, but are required in very small amounts for cellular processes. Many vitamins are important because they are needed to make certain enzymes.

Mammals need 13 vitamins. Like most animals, they must obtain vitamins from their diet. Some vitamins that are important for human health are listed in Table 5.3.2.

Minerals are also essential for cellular processes. Dietary minerals are chemical elements that are required as essential nutrients by an organism. Minerals are a major constituent of structures such as teeth and bones, and can be important components of body fluids. A deficiency in one or more dietary mineral can result in a disorder, such as lack of calcium impacting on bone mineralisation.

FOOD MUST BE DIGESTED

Organisms are composed of many different types of complex organic molecules. When eaten as food, these molecules are too large to be simply absorbed into an animal's body.

The principle function of the digestive system of any animal is the digestion and absorption of food. In other words, the digestive system breaks down organic food into molecules small enough to be able to pass through cell membranes and into cells for use in the production of energy.

Before food passes into the digestive system of a mammal, it is physically broken into pieces by the teeth. Mucus is secreted to protect the lining of the gut and to lubricate food for easier passage. The food then moves along the gut past a series of digestive enzymes that sequentially break down the various compounds for absorption. Proteins are broken down to amino acids. Fats and lipids are broken down to fatty acids. And glycerol and complex carbohydrates such as starch are broken down to simple sugars.

The food you eat does not become part of your body until it has been absorbed by the cells lining the walls of your intestine. The digested food then passes into the bloodstream and is carried throughout the body. If food is not absorbed, it continues through the intestine and is passed out again as faeces (egestion).

Do not confuse egestion with excretion. Excretion refers to the removal of substances that were once part of the body, and occurs largely in the kidneys. Excretion is covered in more detail in Section 3.3.

Physical digestion

Digestive enzymes can only act on the outside surface of food. If food is swallowed in large pieces, the enzymes have a relatively small surface area to work on. Unless the digestive system is extraordinarily long, most of the food would remain undigested. Given the relationship between surface area and volume, digestion is much faster if food is in small pieces and the enzymes have a proportionally larger area to act upon.

Therefore, it is important to have a mechanism for breaking down large food into pieces to increase its surface area. This process is called physical digestion. Animals have developed a variety of structures to break down food physically-for example, the teeth of vertebrates, which break food into pieces small enough to be swallowed. You will learn more about teeth as an adaptation to diet in Chapter 8.

To improve the efficiency of digestion, this physical breakdown should take place before chemical digestion is completed. In contrast to chemical digestion, physical breakdown does not chemically change food molecules.

Bile is important in the physical breakdown of fats, but it is not an enzyme. Bile is a fluid produced by the liver and released into the small intestine, where it acts like a detergent to emulsify fats-breaking up large fatty masses into small droplets. This increases the surface area of fats available for digestion by enzymes.

Chemical digestion

The process of breaking apart complex molecules into simple molecules is called chemical digestion, and is carried out by the action of enzymes. Enzymes are important in digestion, because they greatly increase the rate of breakdown of food molecules.

Most digestive enzymes split food molecules by the process of hydrolysis (from Greek 'hydro', meaning 'water', and 'lysis', meaning 'split'). This means the enzymes split the food molecule by adding a molecule. There are three main types of digestive enzymes (Figure 5.3.2):

amylases, which act on carbohydrates ( e.g. starch) proteases, which act on proteins lipases, which act on lipids.

Digestive enzymes are manufactured by specific cells in the gut wall, and by the salivary glands and the pancreas. Many very large food molecules can only be broken down by several enzymes acting one after the other. In this case, the different enzymes are produced at appropriate sites along the digestive system.

The importance of pH

Because enzymes are proteins, they are sensitive to changes in the pH of a solution (Figure 5.3.3). Altering the pH changes the shape of protein molecules, which in turn alters their chemical properties. The change in shape alters the way that an enzyme binds with the molecule upon which it acts. Enzymes, therefore, have certain pH ranges over which they operate best. Different regions of the gut have different pH values that are most suitable for enzymes found in that region.

Extracellular digestion

Chemical digestion can be extracellular or intracellular. Extracellular digestion occurs when, for example, cells release enzymes into the lumen (central cavity) of the small intestine. There, enzymes split the food molecules and the resulting smaller molecules are absorbed. A sea star turns its stomach inside out and releases enzymes directly onto the animal it has trapped. Carnivorous plants and fungi also release enzymes to break down their food before absorbing it. In each of these examples, digestion is extracellular, because it takes place outside cells. Sometimes, digestive enzymes are located on the actual surface of cells. As the food is digested into smaller molecules, the molecules pass immediately into the cells. Mammals and most other animals rely on some form of extracellular digestion.

In contrast, many protozoans and invertebrate animals, such as some species of mussels, sea jellies and free-living flatworms, use intracellular digestion. Their cells engulf small pieces of food into a membrane-bound food vacuole within the cell. Enzymes are released into the vacuole, the food is digested, and the resulting small molecules pass through the vacuole membrane and into the cell's cytosol.

FEATURES OF EFFECTIVE DIGESTIVE SYSTEMS

In one sense, the digestive systems of all animals must be effective, otherwise the animal would not survive. The digestive system of each animal must adequately meet their nutritional requirements to maintain normal functions at the cell, tissue, organ and system level.

Large animals require higher levels of energy and nutrients for their normal activities. Because mammals are endothermic (animals that maintain a stable body temperature, usually higher than their environment), they require a lot of energy to maintain their body temperature. They therefore need digestive systems that can efficiently extract large amounts of energy and nutrients from food resources. Characteristics of highly efficient digestive systems include:

•effective mechanisms for capture and handling of food

•appropriate physical breakdown of food

•a one-way gut with separation of tasks along its length

•efficient transport and storage of ingested food

•efficient sequential release of digestive enzymes

•an adequate surface area for maximum absorption of nutrients and water

•efficient egestion of unwanted materials.

DIGESTIVE SYSTEMS OF MAMMALS

All mammals need food and water, but different species have different food requirements, feeding behaviours and digestive systems. For example, cows are slow-moving and spend much of the day eating grass and chewing. In contrast, dingoes are energetic and active, and may spend only 5 or 10 minutes each day eating food. Dingoes and cows have many other differences that relate to their eating habits. Their teeth are very different, and cows have much larger and more complex intestines than dingoes (Figure 5.3.4).

The feeding behaviour, teeth and digestive system of a Tasmanian devil (Sarcophilus harrisii, Figure 5.3.Sa) are similar to those of a dingo (Canis lupus dingo, Figure 5.3.4), while these features in a kangaroo (Macropus species, Figure 5.3.Sb) are similar to those in a cow (Bos taurus, Figure 5.3.4). One common factor is diet: Tasmanian devils and dingoes eat meat, whereas kangaroos and cows eat plants. Dingoes, cows and humans are examples of animals with three different diets: carnivorous, herbivorous and omnivorous.

Carnivores

Animals that only eat meat are called carnivores, and include dingoes, cats and Tasmanian devils (Figure 5.3.Sa). Carnivores have strong jaws for biting and their teeth are specialised for tearing meat. Many carnivores share adaptations, such as long, sharp canine teeth. Carnivores spend much less time eating than herbivores; some animals in the wild, such as lions, may not eat for days between meals.

Animal matter has a much higher proportion of extractable energy per gram than plant matter. Digestion of animal matter is therefore quicker and more efficient than digestion of plant matter. The digestive systems of carnivores are shorter and simpler than those of herbivores, and the carnivore digestive system produces all the enzymes needed for the complete digestion of meat. Carnivores do not have a large caecum (an enlarged pouch where the small and large intestines join) like herbivores do, because bacteria are not required to break down plant matter in their diet.

Herbivores

Animals that eat only plant material are called herbivores, and include cows, rabbits, kangaroos and koalas (Figure 5. 3. Sb). They need to consume a large amount of plant matter to meet their energy requirements. The tough cellulose cell walls of plants make plant matter much harder to break down and digest than animal matter. Herbivores typically spend much of the day eating, because the plant matter must be repeatedly ground by the teeth (physical digestion) to release the contents from broken cells and increase the surface area for enzyme action ( chemical digestion). Herbivore teeth are usually flat for grinding and crushing hard plant matter. Herbivores also have specialised enzymes in their saliva to break down cellulose.

Because plant matter is more difficult to digest, herbivores require a longer digestive system than carnivores to increase the surface area over which nutrients can be absorbed. Microorganisms in the digestive tract of herbivores also play an important role in breaking down cellulose and providing essential nutrients to their animal hosts.

Herbivores use cellulose

Cellulose is the main component of plant cell walls, but its molecules are too large to be absorbed without digestion. Although many species of animals are herbivores, only a few can make the enzyme cellulase, which is needed to digest cellulose. To get around this problem, herbivores have a symbiotic partnership with bacteria that can produce cellulase. This type of partnership is called mutualism.

The cellulase-producing bacteria live in the gut of the animal, where they receive shelter and nutrients. In return, they convert cellulose into simpler molecules that can be absorbed by the gut. The bacteria also supply important vitamins, such as vitamin K and the vitamin B group.

The environment inside the gut is warm and wet, but has little or no oxygen. Therefore, the breakdown of cellulose must occur without oxygen by fermentation (using anaerobic respiration). Because of this, the part of the intestine in which the breakdown of cellulose occurs is sometimes called a fermentation chamber.

In herbivorous mammals, fermentation takes place in different parts of the intestine in different species, with varying degrees of efficiency. Generally, herbivorous mammals belong to either of two groups-hindgut or foregut fermenters.

Hindgut fermenters

In hindgut fermenters, fermentation occurs in either the caecum or the first part of the large intestine, as in the wombat (Figure 5.3.6a). In the koala, fermentation occurs in both the caecum and the first part of the large intestine (Figure 5.3.6b).

Both regions are located after the small intestine, which is the part of the digestive tract where most absorption takes place. This arrangement limits the advantage obtained from this symbiotic relationship, because the products of their digestion are not completely absorbed.

Horses are hindgut fermenters. The relative inefficiency of their system can be seen by the large amounts of undigested plant material found in horse faeces. Some hindgut fermenters, such as possums and rabbits, overcome this problem by producing two types of faeces. One type comes directly from the caecum at night, and is eaten and reingested so that it can go through the intestine again. This practice is known as coprophagy, and allows the vitamins and products of fermentation to be absorbed in the small intestine.

Foregut fermenters

In foregut fermenters, such as the kangaroo (Figure 5.3.6c), the fermentation chamber is located before the stomach. In ruminants, such as cattle and sheep, it is called the rumen. Food can be regurgitated back into the mouth for further physical breakdown (rumination), and then returned to the rumen for continued chemical breakdown by bacteria. This regurgitated food is called cud.

Foregut fermentation has the obvious advantage that the products of digestion by microorganisms are available for absorption along the entire length of the small intestine. Kangaroos and wallabies are the only marsupial foregut fermenters.

Ruminant digestion has some drawbacks. The complete digestion of plant material in the rumen by microorganisms can take a long time-hours or even days-with constant regurgitation and chewing of the cud. If the quality of food is very low (that is, mostly cellulose and not much fresh, young plant growth) an animal may be starved of food that is digested enough for absorption, even though the animal has a very full rumen.

Omnivores

Animals that eat both plant and animal matter are called omnivores, and include humans, domestic dogs and pigs. The word omnivore is from the Latin 'omnivorus', which means 'all-devouring'. Omnivore digestive tracts can break down both meat and plant matter. The variety of different animals that are classified as omnivores can be further categorised based on their feeding behaviour and preferred foods. For example, animals that consume insects and plant matter are classified as insectivores.

Omnivores can consume a diverse range of foods, providing greater food security during periods of low food availability or environmental pressures.

Digestive system of humans

Because humans are omnivorous, our teeth are unlike those of carnivores or herbivores. We are not very good at chewing bones or grass-our preferred foods include both meat and plant material, and we often cook our food first.

Humans spend about 30 to 90 minutes each day eating, although the social aspects of eating may extend this time. The human digestive system is proportionally longer than that of a carnivore, but shorter than that of a herbivore.

The main regions of the human digestive system are the mouth and mouth cavity, oesophagus, stomach, small intestine, large intestine, rectum and anus (Figure 5 .3. 7). Glands and organs that assist with digestive functions are the salivary glands, pancreas and liver.

Key steps in the process of digestion in humans occur at the following sites:

• mouth

- teeth mechanically break food into small pieces

- saliva lubricates food and enzyme amylase digests starch into maltose

• epiglottis (a flap at the entrance to the larynx)

- prevents food from entering the trachea and respiratory system, directing it down the oesophagus

- is also associated with the gag and cough reflex

oesophagus

- a tube down which food travels to the stomach, aided by muscular contractions (peristalsis)

stomach

- secretes protein-digesting enzymes (proteases) and gastric juices to aid in food digestion

- peristalsis of stomach muscles further breaks the food down and pushes it through the digestive system

• liver

- has important roles in regulating metabolism, toxin removal and processing nutrients

- stores excess glucose as glycogen (a polysaccharide or carbohydrate) for later conversion back to glucose when needed for energy

- is the site of bile production for the breakdown of fats

• gall bladder

- stores and concentrates bile before releasing it to the small intestine

• pancreas

- produces digestive enzymes that are activated when the food reaches the duodenum (first part of the small intestine)

- produces the hormones insulin and glucagon, which regulate sugar levels in the blood

- produces sodium bicarbonate, which neutralises stomach acids in the food

• small intestine

- absorbs nutrients and minerals from food

- enzymes produced in the pancreas and the small intestine and bile from the liver and gall bladder further breakdown food products to facilitate nutrient and water absorption

- has many blood vessels to absorb nutrients and waste products of digestion and deliver them to the circulatory system

• large intestine

- absorbs water with soluble compounds, such as vitamins and minerals

- undigested food leaves the body as faeces.

Structure of the small intestine

The principle organ of absorption is the small intestine. 'Small' refers to the diameter of this part of the intestine. The small intestine is long and has a large surface area, making it well suited for absorption. The internal surface area is further increased by millions of tiny folds called villi, and by the presence of many microvilli on the exposed surface of the epithelial cells lining the lumen.

Absorption in the small intestine

The epithelial lining in the small intestine is only one cell thick. This allows a rapid transfer of nutrients to the many blood and lymphatic vessels beneath the surface, which transport nutrients away to the body tissues. Nutrients pass through the lining of the small intestine by facilitated diffusion or active transport, along or against the concentration gradient.

Lipid-soluble molecules, which are the products of fat digestion (fatty acids and glycerol), diffuse easily through the membranes of the epithelial cells along a concentration gradient. They then reassemble into fats before passing into the lacteals. Lacteals are capillaries of the lymphatic system near the intestine. They have a milky appearance, because of their high fat content after a fatty meal has been eaten. Lipid-soluble vitamins also pass through the intestinal epithelium by passive diffusion.

Water-soluble molecules, including amino acids, simple sugars (monosaccharides, e.g. glucose), and water-soluble vitamins and minerals pass through the membranes of the epithelial cells by active transport and facilitated diffusion. This can occur down or against a concentration gradient, ensuring that these essential nutrients are absorbed quickly.

Most of the water (90-95%) that enters the small intestine is also absorbed. This absorption is passive. Water diffuses across the lining of the intestine osmotically as the products of digestion are absorbed.

Blood leaving the intestine passes first into the liver through the hepatic portal vein. Here, absorbed nutrients are removed and stored in the liver before the blood passes into the general venous circulation.

food and energy storage in mammals

When food is not available, an animal's body draws on its own stores to meet its nutritional and energy needs. Energy storage is essential for carnivores, which can only eat intermittently depending on the availability of prey.

Herbivores often travel considerable distances to find new and adequate supplies of edible plants when the seasons change, or if they have overgrazed an area. In winter, food generally becomes scarce for both herbivores and carnivores. In cold climates, animals need to consume more food to produce enough energy to maintain their body temperature. For example, freshly shorn sheep need to eat approximately 25% more food than normal, because without their fleece, their insulation against heat loss has been greatly reduced (Figure 5. 3 .11). In very cold climates, some mammals (usually small species) resort to hibernation to survive the winter (Figure 5.3.12). In each of these situations, the ability to store nutrients and energy reserves is essential for survival.

Energy reserves

In contrast to plants, animals have only a limited capacity to store carbohydrates. The storage carbohydrate in animals is glycogen. Like starch, glycogen is a large molecule made from glucose subunits. When needed, carbohydrate stores are used first and most easily. In humans, about 300 g of glucose is stored as glycogen in the liver and muscles. This is enough to provide the energy for about half a day at a moderate level of activity. The remainder of our energy reserves is stored as fats.

Animals use fats rather than carbohydrates as their main form of energy reserves because:

•almost 25% more ATP is produced (per carbon atom) from fats compared with carbohydrates

•fat is almost 50% lighter (per carbon atom) than carbohydrate stored carbohydrates attract and bind water molecules, increasing their weight by 200-500%; fats do not

1 g of carbohydrate or protein provides up to 1 7 kJ of energy, while 1 g of fat provides 39 kJ of energy.

The capacity for storage in fat tissue (adipose tissue) is virtually unlimited. An average 70 kg male human stores about 11 kg of fat, which provides enough energy to last about a month without eating food. The same amount of energy stored as carbohydrate could weigh more than 100 kg.

Some of the chemical processes that take place in living organisms use up energy, while other processes release energy. For energy balance, energy input (eating) must equal energy output (usage). If the amount of food eaten provides more energy than is used, the excess energy is stored as chemical energy (e.g. in fat or glycogen). If the energy content of food is less than required, the balance is made up from stored energy reserves or by breaking down body tissues.

Unlike carbohydrates and fats, amino acids cannot be stored in animal tissues. The full range of amino acids needed for building proteins must be available in an animal's diet, because cells assemble proteins by linking amino acids in a specific order. If the next amino acid required is not available, the synthesis of that protein molecule cannot continue until the required amino acid arrives.

This has consequences for strict vegetarians. Unlike meat, individual plants do not normally contain the full range of essential amino acids. However, a vegetarian can obtain a balanced diet by eating an appropriate combination of plant foods in the same meal, such as beans (which are a good source of the amino acids isoleucine and lysine, but deficient in tryptophan) and rice (which is deficient in isoleucine and lysine, but a good source of other essential amino acids). A meal of rice and beans together is as good a source of protein as eggs or meat.

Energy requirements of humans

In animals, the amount of energy needed each day depends on factors such as basal metabolic rate, body size, activity level and environmental temperature. Metabolism is the name for the sum of all these processes, and metabolic rate is a measure of the overall energy requirements of an organism.

Basal metabolic rate refers to the amount of energy required to maintain basic functions in a resting, unstressed animal per unit of time. It varies greatly between species. Mammals have a much higher basal metabolic rate than some other vertebrates, because they use energy to maintain a constant body temperature. Basal metabolic rate does not include the extra energy required for activity or for maintaining a warm body temperature in a cold climate.

In humans and other mammals, metabolic rate is affected by:

•body composition (the proportion of fat or bone to muscle)

-muscle tissue uses energy at a faster rate than does fat tissue, so more muscle means a greater energy requirement level of activity

- different levels of physical activity account for large differences in metabolic rate

- individuals vary in the amount of energy they use to carry out a particular activity

biological sex

- males generally use energy at a higher rate than females, mainly because on average they have a lower fat-to-muscle ratio than females age

-metabolic rate increases during periods of growth, such as childhood and **adolescence** and levels off during adulthood (except during pregnancy and breastfeeding, when metabolism and energy requirements increase by approximately 25%)

-metabolic rate declines during later years, mainly because of decreased physical activity and changes in body composition.

Malfunctions of the digestive system

The digestive system is complex. Many different functions must coordinate to digest food. Sometimes problems can arise in the digestive tract, inhibiting the absorption of nutrients. These problems can lead to minor or serious diseases, some of which can be treated with surgery or medication.

Coeliac disease

Coeliac disease affects both children and adults. It is a condition in which the villi of the small intestine are damaged (Figure 5.3.13) by the body's immune system in response to gluten, the protein found in wheat, rye, barley and other cereals. The damaged villi will repair themselves if affected individuals follow a gluten-free diet.

Liver disease

People who drink alcohol excessively are prone to severe and often fatal liver disease. Medical evidence indicates that the addition of vitamins to alcoholic drinks, while good for nutrition, will not prevent chronic liver damage.

Alcohol is a toxic substance. The enzymes that are needed to break it down are found in the liver. Because the biochemical pathways in the liver cells of a heavy drinker are involved with removing alcohol, the cells cannot carry out their normal levels of cellular respiration. Substances that should have been broken down for energy are converted to fats instead, and these fats accumulate in the liver.

For a while, the situation is reversible. But then the cells filled with fat start to die, causing alcoholic hepatitis. This is followed by cirrhosis, which is the formation of scar tissue in the liver (Figure 5.3.14). Finally, death may occur when the liver is unable to carry out its normal functions.

5.4 Gas exchange: heterotroph

respiratory systems

Organisms must exchange oxygen and carbon dioxide with their environments to maintain the important energy-transforming process of cellular respiration. Disruption of this exchange-for example, by respiratory illness in humans-can have serious consequences.

In organisms that use aerobic respiration, the rate at which oxygen is supplied to cells limits the amount of energy that can be released from glucose for cellular activities. Carbon dioxide, which is produced as a waste product during cellular respiration, forms a weak acid in solution with water. If carbon dioxide accumulates in the body fluids, the pH will decrease (that is, acidity will increase), with damaging effects on the structure and function of many important molecules. It is therefore important that carbon dioxide is removed efficiently.

In unicellular and very small organisms with high surface area to volume ratios, adequate levels of gas exchange occur directly with the environment. In larger animals that have a high metabolic rate and a need for highly efficient gas exchange, well-developed mechanisms to ventilate their gas exchange surfaces are required. The gas exchange surface is linked closely to blood transport systems, ensuring that gases move efficiently between cells and the environment.

DIFFUSION

Gas exchange always takes place by diffusion across a moist cell membrane. Diffusion is the passive movement of a substance along its concentration gradient from a region of high concentration to a region of low concentration.

The immediate environment of cells is the layer of fluid that surrounds them. Even for organisms that get their oxygen from air, oxygen must first dissolve in the layer of extracellular fluid covering the gas exchange surface before it can cross cell membranes and enter the body.

Small, uncharged molecules, such as oxygen and carbon dioxide, pass directly through the phospholipid bilayer of the membrane. They therefore diffuse into or out of cells along their concentration gradient. In contrast to the many nutrients that are actively taken up by organisms, neither oxygen nor carbon dioxide is actively pumped across membranes.

The rate of diffusion of a molecule across a membrane depends on the size and maintenance of the concentration gradient, and on properties of the membrane itself. The amount of a certain molecule transferred per unit time depends on the membrane's permeability to the molecule, the available surface area of the membrane, and the thickness of the membrane (the distance of diffusion).

EFFICIENT GAS EXCHANGE SURFACES

For efficient gas exchange:

•the surface area should be as large as possible. There is a greater total exchange across a large surface than across a small one

•the barrier to be crossed (such as cell membranes and fluid layers) should be as thin as possible and should consist of a material that allows the gas to pass through the barrier easily

•there should be an adequate supply of the gas being transferred. If the respiratory surface is not adequately ventilated, the rate of exchange drops

•there should be efficient removal of the substance after transfer. Oxygen is carried away from the respiratory surface, usually by blood. Inadequate blood flow past the respiratory surface will allow oxygen to accumulate, slowing further transfer.

In most large animals, energy is required to ventilate the respiratory surface and circulate blood past its inner surface. The efficient supply and removal of oxygen maintains a high concentration gradient across the exchange surface, and therefore a high rate of diffusion.

Energy expenditure is most economical when the rates of ventilation and blood flow to the respiratory tissue are matched. For example, when you begin to exercise, you need more oxygen. You breathe more heavily and your heart rate increases. Ventilation and blood flow to the lungs are still matched, but each is at a higher level to supply more oxygen.

BREATHING AIR

When you breathe air, oxygen is absorbed from the environment by the respiratory system and transferred to your cells via the circulatory system.

The key steps in the process of human respiration occur at the following sites: nasal cavity, airways and alveoli (Figure 5.4.1).

Nasal cavity-Air is drawn in through the nose and passes into the nasal cavity and pharynx (the back of the throat). Breathing through the nose is preferable to breathing through the mouth because the air is filtered, moistened and warmed in the nasal passages.

Airways-From the pharynx, air passes into the airways: the trachea, paired bronchi (singular bronchus) and branching bronchioles. The trachea and bronchi are lined with cells covered in cilia (singular cilium) and secrete mucus (Figure 5.4.2). Particles of dust or bacteria are trapped by this mucus and swept by the cilia back up to the pharynx and swallowed.

Alveoli-Air enters the terminal air sacs, called alveoli, where gas exchange takes place. A constant supply of oxygen to cells is the most critical input for endotherms, such as mammals and birds, because they use energy to warm their bodies, and therefore need oxygen at a great rate for cellular respiration.

The alveoli have many specialised features that make them efficient gas­exchange structures. Alveoli provide a large surface area for gas exchange; the total surface area in most adults is between 30 and 70m2. Each alveolus is lined with a very thin layer of flattened cells, called the alveolar epithelium (Figure 5 .4.1). This thin layer of cells is richly supplied with blood capillaries, facilitating diffusion of gases between the alveoli and the capillaries (Figure 5.4.1). Once oxygen enters the capillaries, it has entered the circulatory system, and the oxygenated blood is transported throughout the body (Figure 5.4.3).

Breathing air has advantages and disadvantages. Ventilation with air requires much less energy than breathing water, which is heavy, and much more oxygen is available in air than is available in water. But animals that breathe air must have a large, moist gas exchange surface. Because water evaporates continuously from this surface, it is a major site of water loss for all terrestrial organisms.

Enclosing the respiratory surface inside the body provides physical protection from the external environment, supports the respiratory membrane, and reduces water loss. However, it increases the need for efficient ventilation of the gas exchange surface.

Lung ventilation

Mammalian lungs are contained in the chest cavity (the thorax). The thorax is completely enclosed and under a small negative pressure that keeps the lungs expanded. The floor of the chest cavity is the muscular diaphragm.

Mammals use a **'suction pump' mechanism** to ventilate their lungs. The chest cavity is expanded by the contraction of the diaphragm downwards and the raising of the ribs. This expands the lungs and draws air in through the airways (Figure 5 .4.4). Inhalation is always an active process that requires energy. Exhalation, however, is normally the result of the elastic recoil of the thorax as it returns to its relaxed state. Forceful exhalation involves an active compression of the rib cage.

Human divers must breathe air at high pressure to inflate their lungs against the pressure of the outside water. This is because our chest muscles are not strong enough to expand the rib cage and inhale against water pressure at depths below about lm (Figure 5.4.5).

Tidal volume

Tidal volume is the volume of air moved in and out at each breath. Normal resting levels of inhalation and exhalation are much less than our vital capacity, which is the maximum volume of air that we can move into and out of our lungs. Tidal volume varies according to the need for oxygen.

Air moves tidally into and out of mammalian lungs through the same airways (Figures 5.4.4 and 5 .4.6). This is not as efficient as one-way flow, because at the end of an exhalation there is still some 'stale' air left in the airways and in the alveoli. The next inhalation draws this stale air back into the lungs. Therefore, it is impossible to fill our lungs completely with fresh air. The volume of air left in the respiratory system at the end of exhalation is referred to as the residual volume.

You will learn more about the transport of gases between the internal and external environments of an animal in Chapter 6.

MALFUNCTIONS OF THE RESPIRATORY SYSTEM

Asthma, emphysema and pneumonia are human illnesses of the respiratory system. Although these three illnesses are unrelated, some of their symptoms such as tiredness and inability to exercise-are similar. Each illness interferes with at least one of the important features responsible for efficient gas exchange in the lungs, leaving the cells unable to produce enough energy for their normal functions.

Asthma

Asthma is a condition in which the cells lining the airways (bronchi and bronchioles) are sensitive to foreign particles in the air, such as pollen. Small airways swell, fill with mucus and become constricted (Figure 5. 4. 7). This reduces the space through which air can flow, increasing the resistance to the flow of air into and out of the lungs. It is particularly difficult to breathe out. When the pressure on the lungs is increased to force air out, the narrowed airways causing the obstruction are also compressed.

Emphysema

Emphysema is caused by the breakdown of air sacs in the lungs. This reduces the lung surface area available for gas exchange, sometimes to less than one-quarter of that of healthy lung tissue (Figure 5.4.8). It occurs mostly in older people and is becoming increasingly common, most likely due to smoking (Figure 5.4.9). Like asthma, emphysema involves an increased resistance to airflow in the small airways, making breathing more difficult.

Pneumonia

Pneumonia is caused by an infection that causes the lungs to become inflamed, and the air sacs (alveoli) to fill with white blood cells and fluid (Figures 5.4.10 and 5 .4.11). This interferes with respiration, because the fluid in the alveoli reduces the area of lung surface in contact with air. The inflamed lung tissue is also swollen, so that oxygen must diffuse further before it can enter the blood.

6.1 Transport system in plants

As you learnt in Chapter 5, vascular plants-like all plants-are autotrophs, or producers. They manufacture their food from light energy by photosynthesis. For photosynthesis to occur, plants need water, carbon dioxide and sunlight for energy. Water is absorbed through the roots, and carbon dioxide is absorbed through the leaves. Photosynthesis occurs in the leaves and produces the sugars that are needed by all active cells of the plant.

In large vascular plants, the leaves can be a long way from the roots. Tall trees, such as the giant sequoia in Figure 6.1.1, need to transport water and nutrients up to 100 m from the roots to the upper branches. Transport of these substances to where they are needed is made possible by vascular tissue.

In this section you will learn about the systems and structures that plants use to transport water and nutrients.

TRANSPORT STRUCTURES: VASCULAR TISSUE

Vascular tissue is used by vascular plants to transport water and mineral ions absorbed from the soil and sugars produced in the leaves to cells throughout the plant. Vascular tissue is visible as parallel veins in grasses, branching veins in many other leaves and the stringy parts of celery.

Xylem-transports water and inorganic nutrients (mineral ions) absorbed by the roots from the soil to the aerial (above ground) parts of the plant phloem-transports organic nutrients (dissolved sugars) produced in the leaves by photosynthesis throughout the plant. Other organic substances, such as amino acids, are also transported in the phloem.

Xylem and phloem form continuous, closed tubular pathways through roots, stems and leaves (Figure 6.1.2). Fluids flow through these tubules to all parts of the plant. All cells are close to vascular tissue.

TRANSPORT OF WATER AND MINERAL IONS

Plants absorb the water and mineral ions they need for growth from the soil through their root hair cells. Potassium is needed to regulate the opening and closing of stomata; calcium is needed to build cell walls; magnesium is important in the production of chlorophyll; and nitrogen is necessary for making proteins and amino acids. Water is essential for dissolving and transporting mineral ions through the plant. Following absorption, the plant must transport water and mineral ions from the roots to where they are needed.

Xylem

Xylem is the vascular tissue that transports water and mineral ions obtained from the soil throughout the plant. It is mainly composed of xylem vessels and elongated cells called tracheids.

Xylem vessels

A mature xylem vessel (also known as a vessel element) is a long, water-filled tube consisting of elongated cells joined end to end (Figure 6.1.3). As the cells mature, the cell wall is strengthened with lignin (a polymer related to cellulose), making them stronger and more rigid. The cytoplasm and nucleus in the xylem vessel cells then disintegrate and the cells die, creating hollow lignin tubes.

Mature xylem vessels have:

cylindrical skeletons of dead cells joined end to end to form continuous tubes perforated or complete openings at each end, like a straw, so that fluid can flow directly through them

pits (unthickened areas) and perforations in the side walls that allow sideways movement of substances between neighbouring vessels in the vascular bundle no nucleus or cytoplasm.

Tracheids

Tracheids are single, large, tapering water-filled cells that form part of the xylem tissue in all vascular plants (Figure 6.1.3). When mature, tracheids lose their nucleus and cytoplasm. This leads to cell death, but creates an open structure for water to flow through. Mature tracheids have:

cylindrical skeletons of dead cells joined to form continuous tubes, like xylem vessels

pits and perforations in their lignified cell walls

no nucleus or cytoplasm.

Unlike xylem vessels, tracheids are not connected end to end. Instead, their ends overlap and water is transferred horizontally through the adjoining pits.

Root absorption

The major function of roots is to take in water and mineral ions from the soil. Water is essential for photosynthesis, nutrient transfer and transpiration, while mineral ions (e.g. nitrogen, phosphorus and potassium) are needed to manufacture a range of organic compounds, including amino acids, proteins and lipids.

Roots have a branched structure that increases both their surface area and their capacity to absorb water and mineral ions (Figure 6.1.4).

Water pathways

There are two possible pathways for movement of water and mineral ions absorbed from the soil via the roots: the extracellular pathway and the cytoplasmic pathway (Figure 6.1.5).

In the extracellular pathway, most water and some mineral ions pass in or between cell walls. In the cytoplasmic pathway, most mineral ions and some water pass through the cytoplasm of living root cells. The cytoplasmic pathway involves substances entering a root hair cell by crossing the cell's membrane, and then passing from cell to cell through plasmodesmata, which are strands of cytoplasm that connect one cell with the next.

There are three types of transport that move substances across cell membranes and along the cytoplasmic pathway: active transport, osmosis and diffusion. You learnt about these processes in Chapter 3.

Active transport-most dissolved mineral ions are selectively taken into roots by active transport. Proteins in the cell membrane of root cells, specific for each ion, are used for this purpose. As a result, the concentration of ions in the vascular tissue of roots can be more than 100 times their concentration in the water of the surrounding soil.

Osmosis-the high concentration of ions in the vascular tissues of terrestrial plants creates a very large osmotic concentration gradient. Large amounts of water move into root cells along this concentration gradient.

Diffusion-some mineral ions, such as potassium and phosphate, enter the roots by diffusion. The uptake of these nutrients therefore depends on the rate of water uptake.

Active transport-most dissolved mineral ions are selectively taken into roots by active transport. Proteins in the cell membrane of root cells, specific for each ion, are used for this purpose. As a result, the concentration of ions in the vascular tissue of roots can be more than 100 times their concentration in the water of the surrounding soil.

Osmosis-the high concentration of ions in the vascular tissues of terrestrial plants creates a very large osmotic concentration gradient. Large amounts of water move into root cells along this concentration gradient.

Diffusion-some mineral ions, such as potassium and phosphate, enter the roots by diffusion. The uptake of these nutrients therefore depends on the rate of water uptake.

Root pressure

In some plants, the osmotic gradient draws in so much water from the roots that it can travel up to 10 m up the stem. This is known as root pressure (Figure 6.1.6). In some deciduous trees, such as birch trees, root pressure causes the rising of sap (water and mineral ions) in spring when the soil is warm and rainfall is high.

In some small plants, root pressure also results in a process called guttation. This is the loss of liquid water, and sometimes other substances, from leaves-unlike transpiration, which is the loss of pure water in the form of water vapour. In guttation, Water is lost through specialised pores at the ends of leaf veins (Figure 6.1. 7).

It usually occurs at night when the air is moist. In tropical conditions, where humidity in the surrounding air is so high that little transpiration occurs, guttation helps plants survive by ensuring the continual upward movement of sap, which transports essential mineral ions from the soil to the leaves.

Entering the xylem

From either of the two pathways through the roots (the extracellular pathway or the cytoplasmic pathway), water and mineral ions must then reach the xylem tissue. Between the roots and the xylem is a waterproof layer of cells that form a barrier known as the Casparian strip (Figure 6.1.5). At this barrier, water travelling through the extracellular pathway is forced into the cytoplasm. In this way, the Casparian strip regulates the substances entering the xylem.

TRANSLOCATION OF SUGARS

Plants transport water, gases and minerals from their roots to the aerial parts of the plant. The transport of organic solutes from the leaves to other tissues in the plant is known as translocation. Leaves produce carbohydrates in the form of sugars during photosynthesis. The non-photosynthetic tissues of the plant also need these carbohydrates-and other organic compounds, such as amino acids, hormones and proteins-so these nutrients are transported from the leaves to where they are needed, e.g. roots, bulbs, stems, flowers and fruits.

Phloem

The tissue through which organic solutes move is the phloem, and the material that flows through it is known as phloem sap.

Phloem transports organic solutes, such as sugars and amino acids, from the leaves (site of photosynthesis) to the stems and roots (site of use or storage). The plant uses or stores the sugars in its cells to produce energy for growth and reproduction. Plants can store sugar in their cells as starch. Starch can be used for structural support, or as an energy source when the plant cannot photosynthesise. Examples of storage structures are bulbs, such as onions, or tubers, such as potatoes.

Phloem tissue is composed of: sieve tubes

companion cells

parenchyma cells

sclerenchyma cells.

Sieve tubes

Unlike xylem vessels, mature phloem sieve tubes are living cells with no nucleus and no lignin in the cell walls. Sieve tubes form linear rows of elongated cells. Their cell walls are thin and perforated at each end by holes or pores, forming sieve plates (Figure 6.1.9). Plasmodesmata pass through the perforations in sieve tubes, acting like straws through which sugars and other materials can move.

Sieve tube cells are usually closely associated with one or more companion cells, connected by plasmodesmata. Sieve cells are able to continue functioning without a nucleus because of their close relationship with companion cells.

Companion cells

Companion cells are a type of parenchyma cell that provide metabolic support and help load and unload materials throughout the plant. Like sieve tube cells, companion cells have thin cell walls. Companion cells retain their nuclei and carry out all the metabolic functions required by the sieve tube cells, sharing metabolic products through the plasmodesmata. Without the metabolic products from the companion cells, sieve tube cells would die, preventing the flow of phloem sap and killing the plant.

Parenchyma cells

Parenchyma cells make up the soft tissue of a plant and have many important functions. In leaves, they contain chloroplasts and make up the mesophyll-the inner layer of a leaf where photosynthesis takes place. Parenchyma cells that contain chloroplasts are called chlorenchyma cells.

In roots and tubers (e.g. potatoes), parenchyma cells have large vacuoles that store starch, fats, proteins and water. Parenchyma cells also provide buoyancy in aquatic plants and play a role in wound repair. Because of the different functions of parenchyma cells, their structure varies from elongated to spherical.

Sclerenchyma cells

Sclerenchyma cells provide strength and structural support for the plant. Mature sclerenchyma cells are dead and have very thick cell walls made of cellulose and lignin. There are two types of sclerenchyma cells: fibres and sclereids. Fibres are found in stems, roots and the vascular tissue of leaves. Sclereids are found in the outer layer of seeds and the shell of nuts. The fibres of some plants, such as flax and hemp, have important uses as textiles.

Translocation: sources and sinks

The sites where sugars are produced during photosynthesis are known as sources. The sources in a plant are the leaves. The sugars produced during photosynthesis need to be translocated to non-photosynthetic (food-producing) cells throughout the plant. The sites where sugars are translocated to are known as sinks. The sinks in a plant include the roots, bulbs, stems, flowers and fruits.

The sugars are translocated in phloem sap, which is around 90% sucrose. Sucrose is a disaccharide that dissolves easily in water, making it a good transport material. Glucose (a monosaccharide) is produced in the chloroplasts of the chlorenchyma cells and converted to sucrose in the cytosol of cells. Sucrose is then pumped into the companion cells and from there, flows into the sieve tube cells (Figure 6.1.10).

Sieve tube cells have no nuclei and reduced numbers of organelles to maximise space for the translocation of materials. Sieve tube cells also have thick and rigid cell walls to withstand hydrostatic pressure, which assists the flow of solutes. Transport in individual sieve tube cells is in one direction only, but bundles of sieve tube cells transport sap in both directions: upwards to leaves and fruit, and downwards to the roots.

Translocation is an active process. It involves the flow of cytoplasm in sieve tubes driven by a pressure gradient, and requires the expenditure of energy by the plant. The pressure gradient begins in the leaves, where sucrose is actively pumped into phloem sieve tube cells. Due to the incompressibility of water, the build-up of water in the phloem creates an osmotic gradient that draws water passively into the sieve cells. As water enters, it increases the fluid pressure (turgor) in sieve cells, which pushes fluid into the adjacent sieve cells.

While this is happening in the leaves, sucrose is being actively removed from sieve cells in roots, and used for growing shoots and developing fruit. This causes an osmotic gradient that draws water out of sieve cells and lowers their turgor pressure.

Fluid pressure is therefore high in sieve tube cells in leaves, and low in sieve tube cells in roots and growing shoots. Most of the phloem sap in sieve tubes flows along this fluid pressure gradient from sources to sinks. This allows the phloem to translocate solutes away from the source and towards the sink. Translocation stops if the cells in the stem die.

TRANSPIRATION

Transpiration is the passive movement of water through the xylem of vascular plants, from the roots to the leaves (Figure 6.1.14). Transpiration also includes the evaporation of water from the leaves via the stomatal pores. The plant uses a small amount of water for metabolic processes, but 99% of the water absorbed by the roots is lost via transpiration.

Although most of a plant's water is lost via transpiration, it is a vital process because it enables plants to:

absorb the water necessary for photosynthesis

transport mineral salts to leaf cells and fruits

cool down and not become overheated.

Transpiration is a passive process: it does not require energy expenditure by the plant. It is driven by the heat energy in sunlight, which breaks the cohesive bonds between water molecules, allowing evaporation through the stomata.

Transpiration-cohesion-tension theory

Although transpiration is a passive process, it requires the movement of water against the force of gravity. The **transpiration-cohesion-tension theory** was first proposed in 1894 by John Joly and Henry Horatio Dixon and is now the most widely accepted theory to explain the upwards movement of water through the xylem of plants. The **theory** explains the primary mechanisms of water movement in plants: cohesion between water molecules, adhesion between water molecules and plant cell walls, and the tension (differential pressure) created when water evaporates from the leaves.

Water molecules are very cohesive-they have a strong tendency to stick together. Water molecules are also adhesive, meaning they have a tendency to stick to other molecules. The adhesive quality of water allows it to stick to the hydrophilic cell walls in the xylem of the plant. It is the cohesion and adhesion of water molecules that allows water to move against the force of gravity.

Because of cohesion, water molecules evaporating from the surface of a leaf pull adjacent water molecules with them. Water in nearby xylem vessels is then drawn up to the leaves to replace the water lost via evaporation. In this way, thousands of leaf cells, each drawing water from the xylem, create tension (differential pressure) that pulls water up the xylem vessels from the roots.

This continuous one-way flow of water from roots to leaves is called the transpiration stream. The pull of transpiration can be strong enough to draw water to the top of the tallest tree, more than 100 m high.

Factors affecting transpiration rates

Water vapour is lost from leaves by transpiration through open stomata. The total surface area across which transpiration takes place is related to the degree of opening of all stomata. This is by far the most important factor affecting the rate of transpiration. The greater the number of stomata and more open they are, the more surface area there is from which water can be lost (Figure 6.1.15). The rate of transpiration is higher during the day than at night because stomata open during the day to exchange gases during photosynthesis, and close at night to minimise water loss.

Other factors that affect the rate of transpiration include:

humidity-transpiration rates decrease when there is a lot of water vapour in the air (i.e. a high level of humidity). Humidity reduces the water concentration gradient between leaf spaces and air, so fewer water molecules evaporate into the air.

temperature-transpiration rates increase as temperature increases, because heat energy increases the rate of water evaporation.

wind-air currents increase the rate of transpiration by moving water vapour away from the leaf and increasing the rate of evaporation of water.

The leaves of some plants that live in exposed conditions have developed structural features that reduce the rate of transpiration. For example, some plants have hairs on the leaf surface, which create a layer of relatively undisturbed, humid air.

6.2 Transport systems in animals

Mammals, including humans, are composed of billions or even trillions of specialised cells organised into tissues, organs and systems. There are many advantages to this level of complexity. But, as with vascular plants, there are also challenges. For example, specialised cells cannot survive independently and must rely on other cells and the survival of the whole organism. This section examines the cardiovascular and lymphatic mammalian systems, how these systems interconnect, and the consequences for the organism when a system malfunctions.

DISTRIBUTING MATERIALS: MAMMALIAN TRANSPORT SYSTEMS

The structure and function of transport systems are similar in all mammals. Mammals have two transport systems: the cardiovascular system (also known as the circulatory system) and the lymphatic system.

The cardiovascular system:

is a closed circulatory system

uses blood as the circulatory fluid

provides most of the transport needs in mammals. The lymphatic system:

is an open circulatory system

circulates colourless lymph fluid

plays vital roles in maintaining osmotic and fluid balance in tissues and supporting immune defences.

THE CARDIOVASCULAR SYSTEM

In simple multicellular organisms, nutrients are transported by diffusion between cells .You learnt about diffusion in Chapter 3.

Diffusion only works over short distances from cell to cell. In large, complex animals, diffusion is not sufficient to deliver essential nutrients to every cell. In humans, it would take more than a year for oxygen to diffuse from the lungs to the brain: but the brain cannot function if it is deprived of oxygen for more than about 6 minutes. For this reason, specialised circulatory systems with networks of pipes and chambers have evolved to transport vital nutrients to all cells in complex multicellular organisms. The cardiovascular system delivers oxygen from the lungs to the brain in less than 4 seconds-the time it takes for the heart to beat four or five times.

The mammalian cardiovascular system (Figure 6.2.1) is a closed system that transports substances throughout the body. The vital metabolic products of the body are transported via the blood. The blood, circulatory tissues and organs ensure that all cells have a ready supply of nutrients and oxygen and a means to transport away metabolic wastes. In mammals, the highly branched network of the cardiovascular system means that no cell is more than 1 mm from a capillary. This ensures efficient nourishment and waste removal for all cells in the body.

Circulation pathways

Blood circulates around the body via two sequential pathways (Figure 6.2.2):

Pulmonary circulation transports blood to and from the lungs. Deoxygenated blood is pumped from the heart to the lungs, where it is oxygenated before returning to the heart.

Systemic circulation transports blood to and from the rest of the body. This system is larger than the pulmonary circulatory system, because the heart must pump blood to all the organs in the body. Oxygenated blood is pumped from the heart to the organs, where it gives up its oxygen to the cells before returning to the heart.

Components of the cardiovascular system

The key components of the cardiovascular system are the heart, blood vessels and blood.

The human heart is a four-chambered muscular pump with two pumping chambers (ventricles) and two receiving chambers (atria). It is responsible for moving blood throughout the cardiovascular system. The right side of the heart pumps deoxygenated blood (blood with a low oxygen concentration), while the left side pumps oxygenated blood (blood with a high oxygen concentration).

Blood vessels are a network of muscular vessels carrying blood to and from the heart. They are divided into:

• pulmonary vessels, which carry blood to and from the lungs

• systemic vessels, which carry blood to and from all other parts of the body. There are three types of blood vessels: arteries, veins and capillaries.

• Arteries carry blood away from the heart. They have thick, muscular walls and carry blood under high pressure.

• Veins carry blood to the heart. They have thin walls and carry blood under low pressure.

• Capillaries connect the arteries and veins. Capillaries are fine vessels with very thin walls (one cell thick) that carry blood under low pressure. The thin walls of capillaries allow gases and nutrients to pass between the capillaries and tissues. Blood is the circulating fluid and is highly specialised for transport and immune defence.

The heart

The mammalian heart is in the centre of the chest, between the lungs, surrounded by the protective rib cage. It has several tissues, including cardiac muscle (Figure 6.2.3), connective tissue and nerve tissue. Connective tissue makes up the valves, while nerve tissue controls the heart rate. A mammalian heart can keep beating even if it is separated from the body, because it has its own electrical impulses.

This property can be used in a type of heart transplant called a 'living organ transplant', where a still-beating heart is transplanted into a patient (Figure 6.2.4).

The mammalian heart has four chambers (Figure 6.2.5). The upper receiving chambers, which have thinner walls, are the atria (singular atrium). Each atrium opens into one of the lower, thicker-walled chambers, called ventricles. Blood moves through the heart in one direction because of the presence of four one-way valves: one between each atrium and the ventricle below, and one between each ventricle and its outgoing artery.

Both sides of the heart function in a coordinated way: first both atria contract, then both ventricles contract. The right side of the heart pumps deoxygenated blood to the lungs, where it becomes oxygenated. The left side of the heart pumps oxygenated blood from the lungs around the body (Figure 6.2.5).

Blood flow through the heart

When the heart beats, it pushes blood through its chambers in a specific sequence. This ensures that deoxygenated blood is transported to the lungs, while oxygenated blood is transported to the rest of the body. One complete circuit through the cardiovascular system of the human body takes about 45 seconds.

Blood flows through the heart and body in the following sequence:

Right side of the heart:

Deoxygenated blood, returning from the body, enters the heart through two large veins (the inferior vena cava and the superior vena cava).

The deoxygenated blood flows through the vena cavae into the right atrium. As both heart chambers relax between contractions, the deoxygenated blood flows through a valve into the right ventricle.

The atrium contracts first, pushing more deoxygenated blood into the right ventricle.

•As the ventricle contracts, the rising ventricular pressure closes the valve between the atrium and ventricle (atrioventricular valve) and opens the valve between the ventricles and the opening of the pulmonary artery (semilunar valve), pushing blood into the pulmonary artery.

•Blood travels through the pulmonary artery to the lungs where it is oxygenated. Left side of the heart:

In the lungs, blood loses carbon dioxide and gains oxygen by diffusion as blood flows through the narrow capillaries around the alveoli.

Oxygenated blood returns from the lungs to the left atrium through the pulmonary vein.

The oxygenated blood is pumped by the left ventricle to the rest of the body via the aorta.

The heart is an active organ

The heart is a continuously active muscular organ, so it has a high requirement for nutrients and oxygen. The cells of the heart have their own rich blood supply via the coronary circulation.

The coronary circulation consists of vessels that spread across the surface of the heart and into the heart tissue. They include:

• arteries

• arterioles

• capillaries

• venules

• veins.

Arteries, veins and capillaries

Blood vessels (Figure 6.2.6) are named according to their structure and position in the cardiovascular system.

• Arteries transport blood away from the heart.

• Veins transport blood towards the heart.

• Capillaries are the narrow exchange vessels between arteries and veins. Arteries and veins are composed of the same layers of tissue, but arteries have more muscular walls, and veins are more easily stretched. Capillaries have very thin walls consisting of only a single layer of flattened epithelial cells.

Capillaries

Capillaries are the smallest of the blood vessels. Their internal diameter is so small that blood cells must travel through them in single file (Figure 6.2. 7). The capillaries:

connect arteries to veins

deliver oxygen, nutrients and other substances to extracellular fluids via diffusion receive carbon dioxide and other wastes.

Capillary walls are extremely thin-just one epithelial cell thick-and porous, which allows substances to pass in and out of the cardiovascular system. Because of their important role in the transport of oxygen and nutrients to tissues, capillaries are most abundant in metabolically active tissues and organs, such as muscle tissue.

Capillaries are distributed throughout the body as an enormous branched network, providing a vast surface area for the exchange of materials between the blood and extracellular fluid. The total length of blood vessels in an average human body is nearly 100 000 km. Most of this length is capillaries, which provide a total surface area of more than 1000 m2 for the exchange of nutrients, oxygen, carbon dioxide and wastes between blood and extracellular fluid. The interwoven network of blood vessels is known as a capillary bed.

A capillary has a diameter of 5-l0µm, so red blood cells (about 7-lOµm in diameter) pass very close to the capillary walls. When the wall of a red blood cell presses against a capillary wall, there is an exchange of oxygen and carbon dioxide. The flattened shape and lack of a nucleus in red blood cells are believed to improve their transport capability, by increasing the surface area available for exchange. Their membrane structure makes them very flexible, allowing them to fold and squeeze through the narrow capillaries.

Exchange between blood plasma and extracellular fluid occurs by diffusion and filtration across capillary walls. To ensure that materials are transported rapidly and efficiently, most cells are no more than 1 mm away from the nearest capillary. Ions and small molecules, such as glucose and amino acids, diffuse through the capillary wall along concentration gradients.

Filtration occurs because of two opposite forces: hydrostatic pressure (or blood pressure) and osmotic pressure (Figure 6.2.8). The pressure from these two forces pushes fluid into and out of the capillaries.

Hydrostatic pressure is a result of blood pushing outwards on the capillary walls. Osmotic pressure results from the differing solute concentrations between the blood and the extracellular fluid.

Because blood is hypertonic (more concentrated) than the extracellular fluid, water tries to move through the capillary walls into the blood, putting an inward pressure on the capillaries. The pressure varies along the length of a capillary, but overall tl1e hydrostatic pressure is greater than the osmotic pressure, so more fluid filters out of the capillary than filters in (Figure 6.2.8). This pressure results in a small amount of protein leakage through the capillary wall cells. When blood pressure increases, this leakage is higher and can result in fluid loss to tissues, causing swelling. Reabsorption allows around 85% of the fluid to return to the capillaries, while the remaining 15% enters the lymphatic system.

In some tissues, such as the gut and liver, the capillaries are more permeable and allow large molecules to cross. This helps the absorption of digested foods from tl1e gut, and enables the liver to take in materials to be broken down. In contrast, capillary permeability in the brain is very low, and access of substances to brain tissue is tightly controlled. Nerve tissue is very sensitive to its environment, so it is important that the composition of the extracellular fluid surrounding the brain and spinal cord is carefully regulated.

Blood vessels can be identified from histological slides or images according to the thickness of their walls:

Arteries have thick walls composed of three distinct layers (tunica).

Veins have thin walls but typically have wider lumen (lumen size may vary depending on the specific artery or vein).

Capillaries are very small and will not be easily detected under the same magnification as arteries and veins (Figure 6.2.9).

Blood

Mammalian blood is a fluid containing cells and cellular fragments. The fluid portion of blood is plasma, which is a pale, yellow liquid containing ions, dissolved gases, proteins, hormones, nutrients and wastes. The cellular elements of blood include red blood cells (erythrocytes), white blood cells (leukocytes) and platelets (thrombocytes) (Figure 6.2.10). They are all produced by cells located in the red bone marrow. The red bone marrow is found in the upper ends of long bones, and in flat bones, such the skull, ribs and pelvis. Blood is a tissue because it is made up of many similar cells working together (Figure 6.2.11).

Red blood cells

Red blood cells make up around 40% of the blood in humans, and a single drop of blood contains about 5 million red blood cells. Mature red blood cells are concave on each side and highly flexible. They lack a nucleus, and are full of the red pigment haemoglobin. Unlike carbon dioxide, oxygen is relatively insoluble. Haemoglobin binds to oxygen and transports it to the cells.

Red blood cells live for about 120 days. Old or damaged cells are removed and broken down by the liver or spleen, and important substances, such as iron, are retained and reused by the body. Every second, 2.5 million new red blood cells are released into your bloodstream, and another 2.5 million old red blood cells are removed and destroyed.

White blood cells

White blood cells are slightly larger than red blood cells, but there are far fewer of them (Figure 6.2.11). A drop of blood contains between 5000 and 10 000 white blood cells, but more are held in reserve in organs such as the spleen, kidney, thymus and thyroid gland.

There are several different types of white blood cells. The two most numerous are phagocytes (neutrophils) and lymphocytes, both of which are involved in defence against microorganisms.

•Phagocytes remove debris and fight infections. They are attracted to a site of infection, where they squeeze through tiny gaps in capillary walls and engulf harmful bacteria and damaged cells.

•Lymphocytes are responsible for the production of antibodies and the development of immune responses.

Platelets

Platelets are fragments of cells. They are much smaller than red and white blood cells, and contain substances that are important in preventing blood loss and promoting blood clotting

Blood pressure

Blood pressure is caused by the contraction of the ventricles. The muscular wall of the left ventricle is almost twice as thick as that of the right ventricle. This is because the left ventricle must pump blood to all the organs, while the right ventricle pumps blood only to the lungs. The right ventricle therefore contracts with less force, resulting in lower blood pressure in the right ventricle and pulmonary arteries than in the more muscular left side of the heart.

In arteries, blood pressure fluctuates with each heartbeat. This produces a pressure wave that can be felt as a pulse where arteries pass close to the surface of the skin, such as at the wrist. The higher systolic pressure occurs when the ventricle contracts, and the lower diastolic pressure occurs when it relaxes.

Malfunctions of the cardiovascular system

Problems that can occur when the cardiovascular system malfunctions include Marfan syndrome, arteriosclerosis and atherosclerosis.

Marfan syndrome

Marfan syndrome is an inherited disorder that affects connective tissue. Connective tissue occurs throughout the body, holding together and supporting other tissue (Figure 6.2.14). The cells of connective tissue are held in an extracellular matrix. The extracellular matrix varies in different connective tissues, from hard and tough (e.g. in bone) to jelly-like (e.g. in fatty tissue).

Marfan syndrome is caused by a defective glycoprotein called fibrillin-1. When functioning correctly, fibrillin-1 forms elastic fibres in connective tissue and assists in intercellular communication. When it is defective, the connective tissue tends to be weakened, affecting its function and causing a range of malfunctions in tissues and organs throughout the body.

Weakened connective tissue has the most serious consequences in the heart, lungs, joints, eyes, spinal cord, skeleton and major blood vessels, such as the aorta.

The aorta is the largest blood vessel in the body. Blood is pumped from the heart through the aorta to the body under considerable pressure. Weakened connective tissue in the aorta can lead to two major problems:

The aorta may stretch and bulge under pressure (Figure 6.2.15). This is referred to as an aneurysm. Aneurysms can cause pain, and blood flow may be slowed through the bulge. Slowed blood flow can lead to blood clots, which can break off. They can travel to the brain and cause a stroke, or to the lungs and cause a pulmonary embolism. If the aneurysm ruptures, the patient can die.

The aorta may begin to tear, so that blood leaks between the layers of the aorta. This can cause pain, lack of blood flow to the tissues and, if left untreated, death. Both conditions are treatable via surgery if diagnosed early.

Arteriosclerosis and atherosclerosis

With age, the arteries lose collagen and elastin filaments. They gradually become less elastic and begin to harden. This hardening of the arteries is referred to as arteriosclerosis. Arteriosclerosis puts stress on the heart, because it must pump harder to push the blood through the inflexible arteries.

Over time, fatty substances, cholesterol and calcium (together, known as plaque) can build up inside these hardened arteries, causing them to narrow (Figure 6.2.16). This specific type of arteriosclerosis is called atherosclerosis.

Both arteriosclerosis and atherosclerosis can affect arteries and arterioles in all parts of the body and restrict the flow of blood to tissues and organs. If atherosclerosis has developed, plaque can break away or blood clots can form around the plaque. Both these situations can cause a stroke or heart attack.

Atherosclerosis can affect the coronary blood vessels that supply blood to the heart muscle. A build-up of plaque restricts the supply of nutrients and oxygen to the heart tissue. If the coronary vessels become too narrow or completely blocked, a heart attack can result, possibly leading to the death of heart tissue.

Everyone will eventually develop some degree of arteriosclerosis, but what causes it to develop more rapidly in some individuals and progress to the more life­threatening atherosclerosis is not fully understood. What is known is that high blood pressure, along with high levels of cholesterol and triglycerides in the blood, increase the chance of developing atherosclerosis. This means that smoking, poor diet, lack of exercise and obesity are risk factors.

THE LYMPHATIC SYSTEM

The lymphatic system is the second transport system in mammals. The lymphatic system is an open system that consists of lymph vessels, lymph nodes and organs, such as the thymus and spleen. It transports a colourless liquid, called lymph, from the tissues to the heart.

The lymphatic system has several roles. One of its main roles is to return extracellular fluid containing proteins that have leaked out of the capillaries back into the cardiovascular system. Without the constant removal of leaked proteins from the extracellular fluid by the lymph capillaries, fluid would accumulate in the tissues. Once inside the lymphatic system, this fluid is called lymph.

The structure of the lymphatic system is similar to the venous part of the cardiovascular system (Figures 6.2.1 and 6.2.17). Fine lymphatic capillaries join to form increasingly larger vessels, which eventually empty into the large veins near the heart. The structures of lymph capillaries and vessels are similar to the capillaries and veins of the cardiovascular system.

Some of the larger lymph vessels can contract, but most lymph flow results from the external compression of lymph vessels by muscular activity, such as during movement and breathing. When vessels are compressed, the lymph fluid is forced in one direction because of numerous one-way valves, like those in veins, which are located along the lymph vessels. When a person stands still or sits for a long time, the fluid drainage from tissues decreases and causes swelling. This is especially so in the legs, because fluid drainage must work against gravity.

The lymphatic system also plays a vital role in the immune system. Invading pathogens are transported in the lymph to the lymph nodes (Figure 6.2.17), where bacteria, viruses and cancer cells are trapped and destroyed by phagocytes and lymphocytes. This is why your lymph nodes swell up when you have an infection.

Malfunctions of the lymphatic system

One malfunction of the lymphatic system is deep vein thrombosis.

Deep vein thrombosis

People who sit for long periods of time, such as passengers on a long flight, are encouraged to stretch and exercise regularly to assist the movement of lymph and venous blood back to the heart. If they don't, fluid accumulates in the feet, ankles and legs, which can then swell.

This fluid accumulation can lead to a condition called deep vein thrombosis, a blood clot that forms in the veins of the leg. If the clot breaks away and is carried by the bloodstream to a lung, it can lodge there, causing a pulmonary embolism: a blockage of the main artery of the lungs (Figure 6.2.18). A pulmonary embolism can cause difficulty in breathing, chest pain and heart palpitations. If the clot completely blocks an artery, the person can die. Regular exercise, a healthy diet and not smoking all reduce the risk of deep vein thrombosis.

COMPARING ANIMAL CIRCULATORY SYSTEMS

Many animals have systems that perform similar functions, but the structure can vary greatly between organisms (Figure 6.2.19). The circulatory system is an example of this. In humans and all other vertebrates, the circulatory system is a closed system; the blood flows in a continuous circuit, enclosed within a system of blood vessels and the heart. Some invertebrates, such as earthworms and octopuses, also have a closed system (Figure 6.2.19).

In general, there is a more complete separation of functions in closed circulatory systems than in open circulatory systems.

In open circulatory systems, blood is pumped by the heart but empties into an open, fluid-filled space, the haemocoel, which lies between the ectoderm and endoderm of the organism.

Three different circulatory systems that animals use to transport substances throughout the body are examined here: the open circulatory system of insects, the single closed circulatory system of fish, and the double closed circulatory system of amphibians, reptiles, birds and mammals.

Open circulatory systems

Arthropods, including insects, have an open circulatory system. An open circulatory system has a heart or heart-like structure, but no blood vessels (Figure 6.2.19). There is also no distinction between blood and extracellular fluid. This single fluid in open circulatory systems is called haemolymph. The haemolymph is in direct contact with all cells, and is kept moving by the beating of the heart and sometimes the movement of the organism itself.

Insect circulatory systems

Insects exchange oxygen and carbon dioxide between the atmosphere and their cells directly. There is no circulatory system involved. These animals, like most invertebrates, are classified as having an open circulation. Gas exchange takes place across a network of fine internal air-filled tubes called tracheae and finer tracheoles. These open to the atmosphere through spiracles that can open and close (Figure 6.2.20). The tracheoles branch into smaller and smaller tubes, reaching all tissue. Oxygen moves into the tissues and carbon dioxide enters the tracheae to be expelled from the body.

This process of gas exchange is quite slow, so some larger insects pump their abdomens to accelerate the movement of these gases. Some insects, such as grasshoppers, also have air sacs that can be pumped like bellows to move air through the system.

The structure of this type of respiratory system is one of the factors that limits the size of insects. Closed circulatory systems

In closed circulation, a heart is the main propulsive organ, pumping blood and maintaining a high blood pressure in the arteries. Animals with closed circulation can increase oxygen delivery to a tissue very rapidly. This is seen in squids, which can propel and swim very rapidly, maintaining high rates of oxygen uptake. The squid's closed system allows sufficient circulation of oxygen to muscles to support short bursts of activity.

Closed circulatory systems may be single circulatory systems with a two-chambered heart, or double circulatory systems with a four-chambered heart.

Single circulatory systems: fish

In the circulatory system of a fish, blood travels from the heart to the gills, where it absorbs oxygen and releases carbon dioxide. It then flows from the gills to the organs and tissues in the rest of the body, and back to the heart. There is just one circuit from the heart, which is classified as a single circulatory system.

Gills are the principal organs of the respiratory system in fish. Oxygen is not very soluble in water, so the respiratory system needs to be very efficient. Fish gills are composed of several gill arches on either side of the pharynx (throat). Each gill arch is composed of rows of filaments, which in turn are composed of lamellae.

The lamellae are closely packed rows of leaf-like structures in which oxygen diffuses into the blood and carbon dioxide diffuses from the blood into the surrounding water. Water is drawn into the pharynx through the mouth and then pushed between the gill arches by compressing the pharynx with the mouth closed. This forces water between individual gill lamellae. The lamellae provide a large surface area for gas exchange and are visibly red because they contain many blood vessels. Water then passes out under the operculurn, which covers and protects the fragile gills.

Double circulatory systems: birds

In the closed circulatory system of birds-as well as amphibians, reptiles and mammals-there are two circuits from the heart. It is therefore classified as a double circulatory system.

In the first circuit, blood passes from the heart to the lungs. It absorbs oxygen and releases carbon dioxide, then returns to the heart. In the second circuit, blood passes from the heart to the organs and tissues in the body, and then back to the heart. This transport system was discussed in detail earlier in this section.

Birds have the most efficient respiratory system of all animals. Most birds can fly and are usually very active. This means a bird has a high demand for oxygen and needs to be light.

As with mammals, gas exchange for birds takes place in the lungs. Bird lungs are similar to those of mammals, but instead of alveoli, they have a system of microscopic tubules called air capillaries. In the air capillaries, oxygen moves into the blood and carbon dioxide moves from the blood into the lungs to be exhaled.

Birds have relatively small lungs that do not expand and contract like those of a mammal. Unlike mammals, birds do not have a diaphragm, but instead rely on pressure changes in air sacs to move air in and out of their respiratory system (Figure 6.2.23). The respiratory system of a bird has seven, eight or nine air sacs, depending on the species.

During inhalation, air is drawn into the posterior air sacs and air from the lungs moves into the anterior air sacs. During exhalation, the air sacs collapse, which pushes air from the posterior air sacs into the lungs. At the same time, the air in the anterior air sacs is expelled via the trachea. This process of exhalation creates a one­way flow of fresh air through the bird's lungs, which is extremely efficient. The large number of air sacs also makes the bird very lightweight.

TRANSPORTING GASES

Oxygen is essential for cellular respiration and the production of energy. The respiratory and cardiovascular systems work together to bring oxygen into the body, transport oxygen to cells throughout the body and remove carbon dioxide (a waste product of cellular respiration) out of the body to the external environment. When oxygen is brought into the lungs, it is carried to cells throughout the body by the oxygen-carrying molecule haemoglobin in the blood. Different molecules in the blood have different affinities for oxygen, which determines how tightly they attach to or release oxygen. Carbon dioxide is carried by the blood to the lungs to be removed from the body via exhalation.

Carrying oxygen

Maintaining an oxygen concentration gradient across the lung surface requires efficient supply (through ventilation) and removal (by circulation) of the oxygen. But the amount of oxygen that dissolves in water (or blood, which is about 90% water) is very small.

The oxygen-carrying molecule haemoglobin increases the oxygen-carrying capacity of the blood. The most important feature of haemoglobin is that it can combine reversibly with oxygen.

Increasing the oxygen-carrying capacity of blood reduces the amount of energy that must be spent pumping blood. Because each millilitre of blood carries much more oxygen, an animal can have a much smaller volume of blood, and pump it around the body more slowly, while still supplying the same amount of oxygen to its cells.

Haemoglobin

Oxygen is relatively insoluble in blood: only 0.2 mL of oxygen gas dissolves in 100 mL of blood. The carrying capacity of mammalian blood is increased 100 times by the presence of the red respiratory protein haemoglobin, which is carried in red blood cells. Mature red blood cells are little more than cell membranes filled with haemoglobin.

Haemoglobin is a complex protein containing iron. Four oxygen molecules can combine with each haemoglobin molecule (Figure 6.2.24). In areas of high oxygen concentration, such as in the blood in vessels in the lungs, haemoglobin combines with oxygen to form oxyhaemoglobin. In areas of low oxygen concentration, such as in muscles that are exercising, oxygen is released (dissociated) from the oxyhaemoglobin. The percentage of oxygen concentration in exercising muscles, tissues and lungs therefore varies. This relationship can be seen in Figure 6.2.24.

Oxygen in the tissues

The oxygen-haemoglobin dissociation curve represents how blood carries and releases oxygen throughout the body (Figure 6.2.25). The percentage of haemoglobin (Hb) saturated with oxygen (02) indicates how readily haemoglobin binds to oxygen molecules. Haemoglobin's affinity for oxygen is affected by many factors. An increased oxygen affinity shifts the curve to the left and a decreased oxygen affinity shifts the curve to the right.

In resting humans, haemoglobin is almost 100% saturated with oxygen in the lungs, and about 75% saturated in other tissues (Figure 6.2.25). This means that only about one-quarter of the oxygen carried by the blood throughout the body is taken up from capillaries and used by cells for cellular respiration. The remaining oxygen in the blood is a reserve, available for use when oxygen demand increases for example, during exercise, when blood oxygen saturation may drop to 25%. An adequate oxygen supply is so critical to our survival that we need a considerable reserve for use during emergencies.

Our muscles are red because they also contain a form of haemoglobin called myoglobin. Myoglobin carries a reserve store of oxygen that muscles can use for a limited period if the amount of oxygen in the blood suddenly decreases to a very low level. This situation could arise if a blood vessel were temporarily blocked, or during strenuous exercise. When blood supply is restored, the myoglobin oxygen store is immediately refilled from the blood. Myoglobin has a higher affinity for oxygen than haemoglobin and therefore can take oxygen from it. This also means that haemoglobin releases large amounts of its bound oxygen to exercising muscle before the myoglobin releases its store, making it a true emergency resource.

The haemoglobin of a fetus binds oxygen with a greater affinity than adult haemoglobin, therefore extracting haemoglobin from the mother's blood in the placenta. This means that at lower partial pressures of oxygen, the fetal haemoglobin loads oxygen more easily than adult haemoglobin. This would cause a shift to the left in the oxygen-haemoglobin dissociation curve (Figure 6.2.25). Myoglobin has a stronger affinity for oxygen than does haemoglobin.

Anaemia

Anaemia is a condition in which there are insufficient red blood cells, or the quality of the red blood cells or the haemoglobin is low. The most common cause of anaemia is a deficiency of iron in the diet. Other causes include failure to absorb iron because of disease, heavy menstruation, or inherited disorders such as sickle cell disease.

Anaemia results in pale skin, tiredness, muscle weakness, headaches and problems with concentration. Treatment is determined by the underlying cause, but can involve a change in diet or taking iron supplements. In extreme cases, treatment can even involve oxygen therapy and blood transfusions.

Carrying carbon dioxide

Carbon dioxide, which is produced by cellular respiration, must be carried in body fluids to an external surface where it can be released to the environment. When it combines with water, carbon dioxide forms a weak acid called carbonic acid, which dissociates into bicarbonate and hydrogen ions. This causes a decrease in pH. As a result, carbon dioxide can be carried in solution only in limited amounts.

In mammals, about 7% of the carbon dioxide carried by blood is dissolved in the blood plasma. About 23% combines with haemoglobin molecules (forming carbamino-haemoglobin), but at a different site on the haemoglobin molecule from where oxygen binds. Carbamino-haemoglobin is still able to combine with oxygen. The remainder of the carbon dioxide produced in working tissues passes into red blood cells, where it is converted to hydrogen carbonate ions, and then passes out to be transported in the plasma.

When blood reaches the lungs, the hydrogen carbonate moves back into the red blood cells, where it is converted to carbon dioxide for release during breathing.

The Bohr effect

In 1904, Danish scientist Christian Bohr discovered that the affinity of haemoglobin for oxygen is inversely related to the acidity of blood and the concentration (or partial pressure) of carbon dioxide.

Bohr found that haemoglobin binds more tightly to oxygen in blood with a low carbon dioxide concentration (low acidity/high pH). This is because haemoglobin loses hydrogen ions as pH increases, changing its structure and increasing its oxygen-binding capacity. As haemoglobin's affinity for oxygen increases, the oxygen-haemoglobin dissociation curve shifts to the left.

The opposite occurs as carbon dioxide concentration increases (high acidity/low pH); haemoglobin picks up hydrogen ions and its affinity for oxygen decreases. As haemoglobin's affinity for oxygen decreases, the oxygen-haemoglobin dissociation curve shifts to the right. This is the **Bohr effect**.

The **Bohr effect** explains how haemoglobin picks up and releases oxygen where needed in the body. As blood nears the lungs, the concentration of carbon dioxide decreases (decreasing acidity) and the affinity of haemoglobin for oxygen increases. This allows haemoglobin to bind to the oxygen entering the blood from the lungs and transport it to cells throughout the body. The opposite occurs in tissues where oxygen needs to be released (dissociated) from haemoglobin. As blood moves away from the lungs, the concentration of carbon dioxide increases (increasing acidity) and the affinity of haemoglobin for oxygen decreases. This allows oxygen to be released from the haemoglobin in red blood cells to cells throughout the body where it is needed for cellular respiration.

**Textbook 3: Focus on life Year 7**

The Musculoskeletal System

Muscles, tendons, and bones work in a coordinated fashion to produce movement.

Real-World Reading Connection What holds up the walls and the roof of a building and protects it from the outside elements? There are beams, braces, and insulation inside the walls and under the roof that you cannot see. What structures support and protect our bodies?

The Skeletal System

The hard structures within our bodies are part of the skeletal system, which provides support, protection, and movement. Press on your wrist, ankle, knee, or elbow. Do you feel something hard under your skin? You are feeling bone, a hard tissue made mostly of cells, collagen, and calcium. Collagen is a protein that forms strong fibers. Calcium is a mineral that adds strength to the collagen fibers. The human body has over 200 bones that make up the skeleton, shown in Figure 1.

Functions of the Skeletal System

If you look at bones, you’ll probably notice that they have different sizes and shapes. The surfaces of bones are not smooth. You’ll see bumps, edges, round ends, rough spots, and many pits and holes where blood vessels and nerves enter and leave. Bones have many small, open spaces so they are not too heavy to move. These features allow the bones to perform all of their functions.

You might be wondering how your skeletal system can protect you from inside your body. The bones of your skull and vertebrae protect your brain and spinal cord. Ribs—the bones in your chest—protect the soft organs underneath, such as the heart and lungs. Without support from the skeletal system, you would be a soft mass without definite shape. The skeletal system also gives your muscles attachment points, which allow you to move. Your skeletal system stores calcium and phosphorus for later use. Both these minerals keep your bones hard. Finally, the middle of some bones, called marrow, is the place where blood cells are formed.

Bones Connect at Joints

Because bones are hard, they cannot bend. However, our bodies are flexible and we can bend, twist, and rotate. This is possible because bones connect at joints. The softer tissues of the skeletal system, shown in Figure 2, help hold bones together at joints and add to our flexibility. Ligaments connect bones. Ligaments are similar to strong rubber bands that stretch when we move.

Cartilage is a strong, yet flexible and elastic tissue that reduces friction and increases flexibility. You can twist your lower arm without moving your upper arm. Can you do the same with your leg? The structure of a joint determines the movement.

Types of Joints

1 Hinge Joint The joints in your fingers, elbows, and knees are hinge joints. Hinge joints only allow bones to move back and forth, like the hinges of a door. Table 1 shows the joints in the body, and simple machines that work similarly.

2 Saddle Joint Compare the movement of your thumb to the other fingers in your hand. The thumb has a wider range of motion. This is because the joint in the thumb is a saddle joint. In a saddle joint, both bones have ends shaped liked saddles. The thumb is the only saddle joint in the body.

3 Ball-and-Socket Joint The shoulder joints and hip joints can rotate and move in nearly every direction. Hip and shoulder joints are ball-and-socket joints. Ball-and-socket joints are made of a bone that has a round end that fits into a cuplike depression of another bone. An ellipsoid joint is similar to a ball-and-socket joint, except the end of the bone is shaped like an ellipse instead of being round. The knuckles of our hands are examples of ellipsoid joints. An ellipsoid joint cannot move in as many directions as a ball-and-socket joint.

4 Pivot Joint The cylindrical region of one bone fits into a ring- shaped structure of another bone in a pivot joint. Pivot joints only allow bones to rotate. The joint between the first two vertebrae in the neck is a pivot joint. This pivot joint allows you to turn your head from side to side. The pivot joint that connects the two bones in your forearm allows you to rotate your lower arm.

5 Gliding Joint Two bones that connect at flat surfaces are a gliding joint. The bones in a gliding joint can only move from side to side or front to back. Our ankles and wrists have gliding joints.

6 Immovable Joint Two bones held firmly together, allowing very little or no movement, form an immovable joint. You might be wondering why a joint would be immovable. Your skull contains immovable joints. When you were born, there was space between some of the bones of your skull. These spaces allowed your brain to increase in size. Eventually, the immovable joints fused the bones together. Your lower jaw is the only bone of the skull that moves after the immovable joints of the skull join.

The Muscular System

When you think of the parts of your body that allow you to move, you probably think of muscles. Muscle is tissue made of long cells that contract. There are more than 620 muscles in the human body. Figure 3 shows that muscles are made of bundles of muscle cells called muscle fibers. Muscle fibers are not like most other cells. A single muscle fiber has hundreds of nuclei and many mitchondria. Some muscle fibers are as long as the muscle, which can be up to 30 cm. Muscle fibers contain bundles of small tubes that contain bundles of two different threadlike proteins, or muscle filaments. The arrangement of muscle filaments is lengthwise, with their ends partially overlapping. During muscle contraction, the muscle filaments move closer to each other. All the cells of a muscle contract at the same time and the muscle shortens. During muscle relaxation, all the muscle filaments move away from each other.

Muscle Contractions

What makes our muscles contract? Muscles have nerve cells that receive signals from the nervous system. The nerve cells start a chemical reaction in the muscle cells that leads to contraction of the muscle. Movement of the muscle filaments during contraction requires energy. Muscle cells contain more mitochondria than other cells in order to produce the energy needed for contraction. A large network of blood vessels supplies muscles with the oxygen the mitochondria need for cellular respiration.

Types of Muscle

Your hand, arm, and leg muscles are voluntary muscles. A voluntary muscle is a muscle that you are able to control. Your heart and stomach are involuntary muscles. An involuntary muscle is one that you cannot control by thinking about it. These muscles work all day, every day, without your active involvement.

Recall from Chapter 2 that cells form tissues, tissues form organs, organs form organ systems, and organ systems form an organism. The same is true in the muscular system. Muscle cells form muscle tissue. There are three types of muscle tissue. Muscles that cause movement of your body are made up of skeletal muscle tissue. Cardiac muscle tissue is found only in your heart. Smooth muscle tissue is found in your internal organs, such as your stomach and blood vessels.

Interactions of the Musculoskeletal System

Our bones alone cannot move our bodies. Similarly, muscle contraction is only part of movement. In order for us to move, the muscular system must function with the skeletal system.

Our skeleton provides support for our muscles.

How do you move?

Usually a muscle connects to at least two different bones. Tendons connect bones to muscles and do not stretch as much as ligaments. Tendons, ligaments, and cartilage are connective tissues. The rough spots on bones are places where the tendons and ligaments attach.

The biceps muscle causes your arm to bend.

Flexion (FLEK shun) is the bending of a limb that decreases the angle between the bones of the limb. Flexion of your arm happens when the biceps muscle shortens during contraction. When the biceps muscle shortens, the lower arm moves closer to the upper arm, and the arm bends.

Opposing Muscle Groups

Muscles can contract and become shorter, but they cannot actively lengthen. Extension is the straightening of a limb that increases the angle between the bones of the limb. How does your arm straighten during extension? Arrangement of muscles is often in opposing groups. Look at the muscles of the upper arm in Figure 4. Notice that there are muscles on each side of the arm. The triceps muscle is at the back of the arm, opposite from the biceps.

Your arm bends when the biceps contract. The biceps relax and the triceps contract, resulting in an extension of the arm.

It is important to realize that opposing muscle groups, such as the biceps and triceps, may be contracting at the same time. At the same time the biceps muscle contracts, the triceps muscle is also slightly contracting. The triceps muscle contract so the lifting motion is smooth and controlled.

The Body and Levers

Muscles and bones are joined in a way that allows them to act as levers, providing force or speed advantages.

Real-World Reading Connection Can you imagine playing baseball without a bat or cutting paper without scissors? How long would it take you to dig a hole without a shovel? All of these tasks are easier when we use simple machines. However, you do not need to find any tools or common objects to see a lever in action. Your body is a living example of levers.

What is a lever?

“Give me a place to stand, and I will move the Earth” is a quote by the ancient Greek mathematician Archimedes (287–212 B.C.), usually credited with first describing the uses of simple machines called levers. A lever is a simple machine made of anything rigid that pivots around a fixed point. The fulcrum is the fixed point that a lever pivots around, also known as a pivot point. Figure 5 is an example of a lever. Archimedes used the concept of levers to devise war machines used against the Roman Empire.

We use levers to make work easier. Sometimes levers allow the operator to perform a task using less force. Other times, the task can be completed in less time or by moving a shorter distance.

By the end of this lesson, you’ll be presented with clues to help you discover why Archimedes said he could move the world.

The Three Classes of Levers

On a seesaw, the board the two people sit on is the lever. The base that the board rests upon is the fulcrum. Two forces act upon different parts of a lever. A force is a push or a pull on an object when it interacts with another object. The effort force moves an object over a distance. The resistance force opposes the effort force. In Figure 6, a child sitting on a seesaw provides the effort force needed to move the right side down. The child being pushed up on the opposite side exerts the resistance force. Note that while one child moves up when the other child moves down, they both move in the same direction around the fulcrum—either clockwise or counterclockwise.

The distance between the forces and the fulcrum determines how easy or how hard it will be to use the lever. If the effort force is very close to the fulcrum, it will take a lot of force to use the lever. The farther the effort force is from the fulcrum, the easier it will be to use. The situation is reversed for the resistance force: the closer to the fulcrum, the easier the resistance force will be to move. Likewise, the farther the resistance force is located from the fulcrum, the harder the lever will be to move.

In your body, if a bone is the lever and muscles supply the force, which part of your body is the fulcrum? The joints act as the fulcrum, the point around which the lever rotates. The connective tissues transfer the force to locations from bones to bones or from bones to muscles.

A seesaw is one of three different types of levers. Each type of lever is suited for different tasks. The location of the fulcrum, load, and applied force determines the type of lever. Table 2 shows the three classes of levers.

First-Class Levers

The resistance force and the effort force are on opposite sides of the fulcrum in a first-class lever. A seesaw, pliers, and scissors are examples of first-class levers. Scissors, shown in Table 2, are made of two first-class levers. When you open and close the scissors, the direction of the effort force changes.

The body has few first-class levers. Nodding your head uses a first-class lever. Table 2 shows that the fulcrum is the joint connecting your skull to your backbone. The weight of your head is the resistance force. Your neck muscles provide the effort force.

Second-Class Levers

The resistance force is between the fulcrum and the effort force in a second-class lever, as shown in Table 2. Backpacks and lug- gage with wheels on the bottom are examples of second-class levers. The handle is where you exert the effort force. The weight of the backpack or luggage is the resistance force. The wheels act as a fulcrum. Another example of second-class levers is a wheelbarrow.

When you lift your heels off the ground and stand on your toes, as shown in Table 2, you are using a second-class lever. The fulcrum is at your toes. The resistance force is the weight of your body. The calf muscle in your lower leg supplies the effort force.

Third-Class Levers

The effort force is between the resistance force and fulcrum in a third-class lever. This arrangement requires more effort force than the resistance force it produces. This means that using the lever to move the object is more difficult than moving the object without the lever. However, you are able to move the object farther or faster than you could without the lever. Most hand tools and sports equipment are third-class levers, such as baseball bats and rakes. Table 2 shows a person gripping the end of a shovel. One hand grips the handle, stabilizing the shovel and acting as the fulcrum point. The other hand exerts the effort force. The effort force moves the resistance force, the weight of the dirt, at the end of the shovel.

The most common levers in the body are third-class levers. An example of a third-class lever is your upper arm and lower arm. Table 2 shows that the fulcrum is the elbow joint. The lever is one of the bones of the lower arm. The resistance force is the weight of your lower arm and any object you may be lifting. The effort force is supplied by the biceps muscle.

Effort Forces and Resistance Forces in Levers

Levers in the human body can exert forces on objects, such as the gymnastic rings in Figure 7. When a lever is used, an effort force is applied to one end. This end moves as the effort force is applied. For example, if you push down on one end of a seesaw, that end moves. The distance this end moves is the effort distance.

When you push down, the other end of the seesaw moves up.

This end of the seesaw exerts the resistance force. This distance this end of the seesaw moves is the resistance distance. The effort distance and resistance distance determine how the resistance force compares to the effort force.

Levers that Increase the Effort Force For first-class levers and second-class levers, the resistance distance is less than the effort distance. This means the point where the effort force is applied moves a greater distance than the point where the resistance force is applied. When the effort distance is greater than the resistance distance, the resistance force is greater than the effort force. As a result, for first-class and second-class levers the resistance force is greater than the effort force.

Levers that Decrease the Effort Force For third-class levers, the opposite is true—the resistance distance is greater than the effort distance. For example, when you use a broom, the distance your upper hand moves is shorter than the distance the bottom of the broom moves. However, when the effort distance is less than the resistance distance, the resistance force is less than the effort force. As a result, third-class levers decrease the effort force.

Why use levers?

Some levers make it easier to lift heavy objects, others make it easier to move objects faster and farther. How is this possible? The person in Figure 8 is lifting a car with a jack. The jack makes it easier to do the work.

Mechanical Advantage

You just read that a lever could decrease the amount of force needed to do a task. Mechanical advantage (MA) is the ability of a machine to increase the amount of force put into the machine, a ratio of resistance force (FR) to effort force (FE).

Therefore, the machine tripled the force applied to it. This is sometimes measured as the ratio of the distance the resistance force is from the fulcrum to the distance the effort force is from the fulcrum. This looks like this:

Speed Advantage

Sometimes we need help moving objects quickly over long distances. Third-class levers can make it easier to move an object a long distance quickly. Recall that a baseball bat is an example of a third-class lever. Figure 10 shows when you swing a bat, the handle moves a short distance. However, the end of the bat travels a longer distance and at a greater speed.

Having bones as levers helps us do work by giving us more efficient ways of using force. This usually allows us to move faster. The end of a swinging baseball bat moves farther and faster than the end grasped by the batter. The same is true for your limbs. Your limbs contain multiple joints and therefore multiple levers. As a result, your feet move much farther and faster than your upper leg when you walk. Therefore, levers allow you to have long, quick strides. Similar to your legs, levers increase the speed of your arms and hands as well.

Levers in the Body

How can a length of a lever affect the way your body works? Consider arm wrestling. If you are facing an opponent whose arm is the same length as your own, who will win the match? Since the distance is the same, and work equals force times distance, the person who is able to produce more force will win. What if one person’s arm is shorter? In this case, the person with the shorter arm will have to produce more force to win the match. However, it will be easier for the person with the shorter arm to produce more force because the effort and resistance force are closer together on a shorter arm. Less effort is required to match the resistance provided by the opponent. This is why shorter men and women have a natural advantage in sports such as gymnastics, diving, and figure skating.

The Pulmonary-Circulatory System

The pulmonary and circulatory systems work together to transport oxygen to your cells and to remove carbon dioxide from your cells.

Real-World Reading Connection Take a deep breath. Notice how your chest expands. Every time you breathe, air enters and exits your lungs. At the same time, your heart is constantly beat- ing. How do these events happen and why are they necessary for survival?

The Pulmonary System

You may already know that we, like all animals, need oxygen to live. We get oxygen from the air we breathe. We also need to rid our bodies of carbon dioxide. Recall from Chapter 1 that carbon dioxide is a waste product of cellular respiration. How do we take in oxygen and remove carbon dioxide? Our pulmonary system contains tissues and organs specialized for taking in oxygen and removing car- bon dioxide from our bodies and for exchanging oxygen and carbon dioxide. The pulmonary system is also often referred to as the respiratory system. Figure 1 highlights the organs and tis- sues of the pulmonary system. Take a deep breath. Think about where you feel the air moving through your body. Look at Figure 1 and trace the path you think the air follows.

Breathing

The pulmonary system is open to the atmosphere, so atmospheric air can move into and out of your body. Breathing is the process of air entering and exiting the lungs. Inhalation is breathing in air, while exhalation is breathing air out. Both phases are shown in Figure 2. The muscles between your ribs play an important role, allowing your rib cage to expand and contract. This alone is responsible for 25 percent of the breathing process. When the flat muscle below your rib cage called the diaphragm contracts, inhalation begins. When it relaxes, exhalation begins.

When you inhale, air enters the pulmonary system through your nose or mouth. Air from the nose and mouth first passes through the pharynx, a tubelike passageway in the throat.

Then air moves into your larynx, the valve separating the upper and lower portions of the throat where sound is produced. Beyond the larynx food objects proceed to the digestive system. Air goes to the trachea, a 12-cm-long tube lined with C-shaped rings of cartilage to prevent it from collapsing. The trachea forks into two branches, called bronchi. Air continues its path through the bronchi.

The bronchi are connected to the lungs, the organs of the pulmonary system. Air enters the lungs through the bronchi and the bronchi then divide into branched tubes called bronchioles. These tubes continue to divide like branches of a tree getting smaller and smaller. At the end of the bronchioles, like leaves at the end of twigs, are millions of microscopic sacs called alveoli. Alveoli are like tiny balloons that fill with air as you breathe. In the alveoli, oxygen from the air that was breathed in and carbon dioxide from the cells are exchanged. The air that now has little oxygen and is high in carbon dioxide is exhaled and flows out in the reverse path.

Problems in the Pulmonary System

We could live days without water and longer than a month without food. However, we cannot live even an hour without oxy- gen. Cells combine the food and oxygen to produce energy. You have probably had nasal congestion from colds or other illnesses. Congestion from colds sometimes makes it hard to breathe because your swollen sinuses fill with fluid and mucus blocks air passageways in your lungs. Other problems in the pulmonary sys- tem, such as pneumonia, suffocation, and asthma, make it difficult to breathe and can lead to severe health problems. Problems in the pulmonary system prevent oxygen from reaching the lungs.

Pneumonia

One of the leading causes of death in the United States is pneumonia, an infection of the lungs. Viruses, bacteria, fungi, and parasites can cause pneumonia. Thick fluid may build up in the lungs of a person with pneumonia. Fluid in the lungs interferes with gas exchange. This causes less oxygen to be absorbed into the blood. X rays are often used to diagnose pneumonia. Compare the X rays in Figure 3 of the lungs of a healthy person and a person with pneumonia.

Pneumonia is caused by contact with mucus from an infected person or the introduction of bacteria or viruses normally present in the mouth and throat into the lungs. Many times, people weakened by environmental conditions, other illnesses, and infections may also contract pneumonia. This is why people who contract pneumonia in the hospital while being treated for other conditions are at a greater risk.

Suffocation Have you ever seen someone choking? Food or other objects can become stuck in a person’s airway. When this happens, oxygen cannot be supplied to the lungs. Suffocation occurs when the lungs and body do not receive enough oxygen. Figure 4 shows abdominal thrusts, used to dislodge an object from a person’s airway. Choking is not the only cause of suffocation. Infants and children can suffocate if blankets or plastic bags cover their faces.

Gases such as carbon monoxide can also cause suffocation. Car- bon monoxide takes the place of oxygen in the blood and is dangerous because it is odorless. Therefore, a person unknowingly can be exposed to high levels of carbon monoxide.

Asthma

The most common long-term disease in children is asthma, a disease of the airways to the lungs. Common substances that cause allergies, such as cigarette smoke, pollen, pet dander, insect droppings, mold, and ozone, cause a more severe reaction in people with asthma. Their airways become swollen, making them narrower than normal. This narrowing makes it difficult to take in enough oxygen, as shown in Figure 5. This causes wheezing, chest tightness, coughing, and trouble breathing. Nearly one in five children in California has asthma. The symptoms of asthma come and go with exposure to allergens. Therefore, people with asthma should be careful to avoid situations in which they are exposed to allergens.

What is the best way to relieve symptoms of asthma?

Many asthma triggers can be difficult to avoid, but it is still possible to lead a normal life with proper treatment. Air pollution, such as smog and ozone, is the primary trigger of most asthma attacks. In some cities, all residents, not just those with asthma, are warned to stay indoors on days when high levels of pollution are measured. Smog levels in California exceeded federal health limits on 109 days in 2004. Exercise can trigger asthma attacks in some people. People with this condition do not have to avoid exercise, but they should seek medical advice before beginning a fit- ness program. With a plan for prevention and treatment of asthma, athletes are unlimited by their condition. Some Olympic athletes, such as swimmer Tom Dolan and track-and-field star Jackie Joyner-Kersee, have won many gold medals with correct monitoring and treatment of their asthma.

The Circulatory System

All the cells of our bodies need oxygen, food, and other nutrients. Cells also produce waste products that need to be removed. How do substances get to and from cells? All these substances are transported by the blood in the circulatory system. The circulatory system is the system that contains the heart and blood vessels and transports blood throughout the body.

Blood

If you have seen your blood, you know that it is a dark red liquid. Blood is a tissue that contains many different substances— even cells. Blood is about 55 percent plasma, which is 90 percent water. The remaining 10 percent is composed of ions, proteins, and other substances the body produces or requires.

Platelets and White Blood Cells Platelets and white and red blood cells, listed on Table 1, make up the remaining portion of blood.

Platelets are fragments of cells contained in your blood. After an injury, proteins in the blood cause platelets to form a plug which stops the bleeding. Without platelets, even a small cut would continue bleeding because a scab would not form. White blood cells are part of the immune system, which fights infections.

Red Blood Cells

Your body contains about 25 trillion red blood cells. Red blood cells are normally shaped like a disc that is flattened on both sides. Red blood cells have iron-containing proteins called hemoglobin that carry oxygen. Because of this protein and their shape, red blood cells can transport about one billion oxygen molecules.

Red blood cells constantly wear out and your body replaces them. They last about 120 days, so you have a completely new population of red blood cells every two months. Because of this, people can donate blood about every eight weeks.

Heart

The heart is the organ of the circulatory system that pumps blood. It is hollow, muscular, about the size of your fist, and located in the middle of the chest between the lungs.

The two upper chambers of the heart that receive blood are called atria (singular, atrium). The ventricles are the two lower chambers that pump blood out of the heart. Deoxygenated blood enters the right atrium of the heart. The heart pumps this blood through the right ventricle and into the lungs. Then the blood flows back to the left atrium of the heart where it is pumped through the left ventricle and to the body.

Blood Vessels

Blood travels to and from the heart in vessels, as shown in Figure 7. The vessels of the circulatory system are like the roads of a city. A city has large interstates, smaller highways, and even smaller neighborhood streets. Similarly, the circulatory system has large blood vessels that are connected to smaller vessels. Arteries are vessels that carry blood away from the heart to organs in the body. Arteries branch into smaller vessels, and then even smaller vessels called capillaries. Capillaries are the blood vessels that deliver oxy- gen and nutrients to the organs, as shown in Figure 8. Just as oxygenated blood flows away from the heart, deoxygenated blood carrying car- bon dioxide must return to the heart. The capillaries take up carbon dioxide and other wastes before joining with larger vessels that carry the blood on its return path. These vessels connect to larger vessels called veins that carry blood to the heart.

All blood vessels have the same basic structure.

The inner lining is a thin, flat layer of cells where the blood and the vessel wall meet. Every part of the circulatory system is lined by this thin layer of cells. Capillaries sometimes only have this thin layer of cells. The next layer is of connective tissue followed by a layer of smooth muscle. The muscle layer is more highly developed in the arteries. Why do you think this is? Finally, there is another layer of connective tissue that contains the nerves and supplies the larger blood vessels with nutrients.

Problems in the Circulatory System

Problems in the circulatory system prevent oxygen from reaching the cells and can lead to serious health problems and death. Diseases of the heart and blood vessels are called cardiovascular diseases. Cardiovascular disease causes more than half the deaths in the United States.

Risk factors for cardiovascular disease include being overweight, eating a diet high in saturated fat and cholesterol, smoking, having high sugar in the blood, being physically inactive, and consuming too much alcohol. Older men are more likely to show symptoms of cardiovascular dis- ease than anyone else. Although tests are avail- able to screen for heart disease, it is instead recommended that people who do not show symptoms, such as heart attack and stroke, identify and eliminate risk factors in their lives.

Heart Attack

The arteries that supply the heart muscle with blood and oxygen are the coronary arteries. A heart attack occurs if the coronary arteries cannot supply enough blood to the heart. This happens when the coronary arteries clog, as shown in Figure 9. The heart muscle may die and the heart may stop working if the muscle does not receive enough oxygen. Symptoms of a heart attack include chest pain, pain in the arms and back, shortness of breath, and dizziness.

Stroke Without oxygen, brain tissue will die within 4–5 minutes. A stroke is the death of brain tissue. A stroke can happen if a blood vessel to the brain is blocked or if one of these blood vessels breaks. A ruptured blood vessel, or a blocked vessel, also shown in Figure 9, prevents blood flow to the brain. Once brain tissue dies, it is not replaced. A stroke may result in memory loss, loss of muscle control, or other loss of nerve function, depending on where in the brain the stroke occurs.

Stroke is the third leading cause of death in adults. When the blood vessels in the brain have degenerated enough in one place to have a stroke, the overall condition is usually not healthy.

Exchanges Between the Pulmonary and Circulatory Systems

The pulmonary and circulatory systems must both function properly for us to survive. The circulatory system cannot deliver oxygen unless the blood has been successfully oxygenated by the pulmonary system. Similarly, the muscles of the rib cage and diaphragm cannot function unless the circulatory system supplies them with oxygenated blood. The air in the alveoli and the blood in the capillaries must be able to exchange oxygen and carbon dioxide freely over the thin membrane that separates them. All the organs, tissues, and cells of both systems must function properly for the systems to function normally. Figure 10 illustrates some of the interactions of the pulmonary and circulatory systems.

Gas Exchange

Perhaps the most important exchange between the pulmonary and circulatory systems is the gas exchange in the lungs. The membrane separating the capillaries and the alveoli allows gases to move across it. No energy is needed for the exchange of oxygen and carbon dioxide—the gases move from regions where they are at a higher concentration to regions where they are at a lower concentration.

The structure of your lungs allows a great amount of oxygen and carbon dioxide to be exchanged between the air you breathe and your blood because the structure within the lungs greatly increases the surface area of the lungs.

Exchange in the Lungs

Alveoli are surrounded by capillaries. Oxygen gas is removed from the air you breathe and passes across a thin membrane between the alveoli and the capillaries. At the same time, carbon dioxide passes in the opposite direction, from the capillaries to the alveoli into the air you had breathed in. This process is shown in Figure 11. When the level of carbon dioxide in the air in your lungs becomes great enough, you exhale without thinking about it.

You may have heard the word respiration used interchangeably with the word breathing. However, they are not the same. Respiration is the use of oxygen and food and the production of carbon dioxide in your cells to produce energy, as discussed in Chapter 3. Breathing is the physical process of inhalation and exhalation.

Preventing Problems in the Pulmonary and Circulatory Systems

What can you do to reduce your risk of stroke and heart attack? Family history, or genetics, partially determines your risk. How- ever, a healthy lifestyle is the best way to prevent cardiopulmonary problems. Exercise reduces the risk of cardiovascular disease by improving the flow of blood and oxygen throughout your body and by keeping your blood vessels flexible. Choosing not to smoke and eating a diet low in saturated fat and cholesterol also helps prevent cardiovascular disease.

Pressure and the Body

Pressure allows us to breathe and keeps blood flowing throughout the circulatory system.

Real-World Reading Connection What happens when you pump air into a ball? The ball becomes more firm as it fills with air. The air pushes against the walls of the ball. In the circulatory system, blood pushes against the walls of blood vessels.

What is pressure?

Air in a ball places pressure on the inside walls of the ball, keeping it inflated. Figure 12 shows another example of pressure. Pressure is the amount of force per unit area. Many different units are used to measure pressure. Atmospheric pressure, the pressure of air on Earth’s surface, often is measured in millimeters of mercury (mmHg). So is your blood pressure. You may be familiar with pounds per square inch (psi), a common mea- sure for tire pressure. The pascal (Pa) is the SI unit of pressure.

Changing Pressure

There are two ways to change pressure, as shown in Figure 13.

First, pressure increases as you increase the amount of force applied to a constant area. For example, the force against the walls of a ball increases as more air pumps into the ball. Second, pressure increases as you decrease the size of the area to which a constant force is applied. For example, the pressure on a ballet dancer’s toes increases when she stands on her toes, as compared to standing on flat feet. This is because the dancer’s body weight is spread over the entire area of the foot. When the dancer is on her toes, the pressure is concentrated on a much smaller area. Similarly, you can decrease the pressure on a surface by applying less force to it. You can also decrease pressure by spreading a force over a larger area. The equation for finding pressure is:

Pressure in the Pulmonary System

When you inhale, do you push air into your lungs? You may think so, but air is pulled into your lungs, not pushed. Mammals, including humans, use this type of breathing to pull air into the lungs. The diaphragm and rib muscles move the chest cavity, as shown in Figure 14. During an inhalation they cause the chest cavity to expand. The volume of the lungs is expanded and the pressure in the alveoli decreases. This causes the pressure in the lungs to become lower than atmospheric pressure. Air moves from areas of high pressure to areas of low pressure, so air is pulled into the lungs. When we exhale, the chest contracts. The volume of the lungs decreases and forces the air out of the lungs and eventually through the mouth and nostrils.

Pressure in the Circulatory System

Have you ever tried to use a water hose when the water was turned on low? You probably had a hard time spraying the water. That is because the water in a hose must be under pressure in order to have a strong, far-reaching spray. Similarly, pressure is needed in the circulatory system. Without pressure, blood vessels would not be able to transport blood to all tissues.

The Heart as a Pump

You use a pump to increase the air pressure of a tire or a ball.

In the circulatory system, the heart is the pump. Recall from Chapter 9 that muscle fibers shorten when a muscle contracts. The heart is also a muscle that contracts. When the heart con- tracts, the volume inside the chamber decreases. Blood is forced out of the chamber as the chamber gets smaller. This is similar to how toothpaste is squeezed out of a tube.

Pumping in One Direction

What happens if you squeeze the middle of a tube of toothpaste?

Toothpaste moves in both directions. It is important for blood to move in one direction as the heart pumps. The heart and veins have valves that keep blood flowing in one direction through the circulatory system. Valves act like doors that open in only one direction. Figure 15 shows the valves in the heart.

The contractions of the muscles of the heart create areas of greater and lesser pressure, pumping blood through the heart. Valves, shown in Figure 15, open and close and also contribute to the different pressures in the chambers. When the muscles of a chamber contract, the pressure in that chamber increases. When the muscles of a chamber relax, there is less pressure. The heart works like two pumps—the right and left atria contract nearly simultaneously and the right and left ventricles contract simultaneously.

1 Right Atrium to Right Ventricle

Using Figure 16, trace the flow of blood through the heart. The right atrium receives blood from the body. The right ventricle relaxes and the tricuspid valve opens, blood flows into the right ventricle. When the right ventricle contracts, the tricuspid valve closes, preventing the blood from returning to the atrium.

2 Right Ventricle to Lungs

The increased pressure in the right ventricle causes the pulmonary valve to open. The blood is pumped to the lungs. The pressure of the blood in the arteries causes the pulmonary valve to close.

3 Left Atrium to Left Ventricle

After gas exchange occurs, the blood returns to the heart. This time, it flows into the left atrium. When the left ventricle relaxes, the bicuspid valve opens and blood flows into the left ventricle.

4 Left Ventricle to Body

The left ventricle contracts, the bicuspid valve closes and the aortic valve opens. This pumps blood into the aorta and to the body. The pressure in the aorta causes the aortic valve to close.

Continuous surges keep the blood moving through the arteries.

As the blood enters the veins, far from the heart, blood pressure decreases. The valves in the veins, shown in Figure 17, prevent backflow, allowing the blood to return to the heart.

Problems with Blood Pressure

Have you ever noticed what happens when water pressure to a faucet changes? The water either slows to a trickle or flows out much faster than expected. In a faucet, a short-term change in water pressure usually does not have a negative impact. However, changes in pressure can lead to life-threatening problems in the human circulatory system.

Hypertension Healthy blood vessels are elastic. When blood vessels lose elasticity, they cannot regulate blood flow as well. Some- times the walls of blood vessels harden or vessels become blocked.

This can lead to hypertension, a dangerous rise in blood pressure caused by blockages in or the hardening of blood vessels. Since all organs are linked by the circulatory system, a dangerous rise in blood pressure can damage other organs. Hyper- tension is a disease caused by genetic and environmental factors. A diet high in saturated fat, cholesterol, and salt is one environmental cause that increases a person’s risk of developing hypertension.

Shock As with high blood pressure, blood pressure that is too low can be very dangerous and life threatening. Shock is a condition in which a large amount of blood is lost, usually in a short period of time. Under certain circumstances, such as internal bleeding, a severe allergic reaction, or traumatic injury, blood leaves the vessels and shock occurs. When this happens, blood pressure decreases. The circulatory system, like a water hose, cannot function properly without pressure. The heart is not able to pump blood to all tissues without pressure. In some emergencies, shock is treated by stopping the blood loss and giving a blood transfusion, if blood loss has been severe.

Visible light is an electromagnetic wave.

Real-World Reading Connection Imagine you are standing on a beach watching giant waves rolling toward you. As they reach the beach, you hear them crashing down and feel them pound the sand. The energy that shakes the ground and creates the noise is energy transferred by the waves. Light is also a type of wave that transfers energy from one place to another.

Light Transfers Energy

Think about what happens when you throw a rock into a still pool of water, as in Figure 1. The rock hits the water and changes, or disturbs, the flat surface of the pool. This disturbance is caused by the energy transferred to the water from the moving rock. As you watch, waves move outward from the place where the rock entered the water. These waves carry energy to other parts of the pool.

Similar to water waves, light waves also carry energy from place to place. A source of light, such as the candle shown in Figure 1, or the Sun, emits light waves. These waves spread out in all directions. Sometimes, however, it is easier to think of light in a different way. A light ray is a narrow beam of light that travels in a straight line. In Figure 1, the light rays emitted by the candle are represented by arrows. You can think of a source of light as emitting light rays that travel away from the source in all directions.

Parts of a Wave

1 Imagine that you are holding one end of a rope that is attached to a wall, as shown in Figure 2. The rope is in its resting position when it is held so that it is perpendicular to the wall.

2 Now, think about what happens when you move your end of the rope in a steady, up-and- down motion. You create a wave in the rope. The wave has a shape that looks like a sideways letter S repeating many times.

The highest points of the wave are called crests. The lowest points are called troughs. The distance between any two crests or any two troughs is called the wavelength. The amplitude of the wave is the distance from a crest or trough of the wave to the resting position.

Frequency and Wavelength

As you move the rope up and down, you make crests and troughs that travel along the rope. The number of crests or troughs that pass a given point in one second is related to the frequency of the wave. The frequency of a wave is the number of wavelengths that pass a given point in one second. The frequency of the wave on the rope is also equal to the number of times each second that your hand moves up, down, and up again.

3 If you move your end of the rope more quickly, the frequency of the wave increases. Then the crests and troughs of the wave become closer together on the rope. This means that as the frequency of a wave increases, the wavelength decreases. The same is true for all waves, including light waves.

4 If you move the end of the rope more slowly, the crests and troughs become more spread out. This means that the wavelength increases as the frequency decreases. This is true for all waves, including light waves. The lower a light wave’s frequency, the greater its wavelength will be.

Electromagnetic Waves

When you think of waves, you might think of ocean waves or waves transferred along a rope. In these examples, the substance through which the wave moves is called the medium. The medium through which ocean waves move is water. The medium through which the rope wave moves is the material that makes up the rope.

Light can travel through different mediums. As shown in Figure 3, light can travel through solids, liquids, and gases. Unlike water waves or waves on a rope, however, light can travel through empty space where there is no matter. Light is an electromagnetic wave, which is a type of wave that can travel in empty space as well as in matter.

The Electromagnetic Spectrum

Like waves on a rope, electromagnetic waves have a range of wavelengths and frequencies. The entire range of electromagnetic waves of different wavelengths and frequencies is called the electromagnetic spectrum.

A Range of Wavelengths

As shown in Figure 4, the electromagnetic spectrum includes all electromagnetic waves arranged from those with the longest wave- lengths to those with the shortest wavelengths. Because frequency increases as wavelength decreases, the electromagnetic spectrum is also arranged in order of increasing frequency.

Visible Light

The light that you can see is only a very small part of the electromagnetic spectrum. Figure 4 shows the visible light spectrum, which is the range of electromagnetic waves human eyes can detect. Visible light has wavelengths that are so short they are usually measured in units of nanometers (nm). One nanometer equals one billionth of a meter. The wavelengths of visible light waves range from about 700 nm to about 400 nm, which is about 100 times smaller than the width of a human hair. Colors that you can see depend on the wavelengths of the light waves that enter the eye.

Visible Light and the Electromagnetic Spectrum

The light that human eyes can see is only a small part of an electromagnetic spectrum. The entire electromagnetic spectrum ranges from waves with wavelengths of thousands of meters to waves whose wavelength is less than the width of an atom. In fact, the electromagnetic spectrum has no upper or lower limits. All electromagnetic waves, whether part of the visible spectrum or some other part of the electromagnetic spectrum, transfer energy as they travel from one place to another.

Light and Matter

Light interacts with matter in different ways.

Real-World Reading Connection When you look around the room, light that enters your eyes comes from different objects. You might see sunlight passing through the glass in a window or your reflection in a mirror. How does the light from these objects reach your eyes?

The Interaction of Light and Matter

Have you ever seen a pinball machine? When the ball is launched, it enters a field of obstacles. It then rolls in straight lines until it hits a bumper. Then it bounces in a different direction. Sometimes the ball goes into a hole and then is shot out a short time later. Light rays behave in some similar ways. When light rays hit matter, they can be absorbed by the matter, be reflected, or pass right through the material.

All electromagnetic waves, including light, transfer energy from one place to another. Figure 5 shows an industrial laser that uses the energy carried by infrared waves to cut through steel. When light waves hit a material, some of the energy carried by the light waves is transferred to the atoms or molecules in the material. Atoms can absorb some of this energy so that the material becomes warmer. Atoms can also absorb some of the light energy and then emit new light waves.

1 Absorption of Light

On a sunny day, an asphalt parking lot can be very hot. The asphalt is hot because some of the energy carried by sunlight is absorbed by the asphalt. As shown in Figure 6, when light rays hit a material, some of the light ray’s energy is transferred to the atoms in the material. This transfer of energy can cause the temperature of the material to increase. The process of transferring light energy to the atoms or molecules in a material is called absorption.

The amount of energy absorbed when light rays strike a material depends on the types of atoms in the material. The amount of energy absorbed also depends on the wavelengths of light. The atoms in asphalt absorb more energy from sunlight than the atoms in grass. As a result, the asphalt becomes hotter than grass.

2 Transmission of Light

When you look through a window, you see objects on the other side of the glass. Light waves from these objects pass through the glass and enter your eyes. Instead of being absorbed by the glass, these light waves are transmitted through the glass. Transmission occurs when light waves strike a material and pass through it. Whether light waves are transmitted or absorbed by a material depends on the wavelength of the light waves that strike the material. Some materials, like glass, transmit only certain wavelengths.

Other materials do not transmit any light waves.

3 Scattering of Light

Have you ever noticed dust particles in a beam of sunlight? When the light waves in a sunbeam strike a dust particle, two things happen. First, they are absorbed by the dust particle, and then they are emitted. The light rays that are emitted travel in all directions. Scattering occurs when a material causes light waves traveling in one direction to travel in all directions. When the light waves in a sunbeam strike a dust particle, they are scatted in all directions. You see the dust particle as a bright speck of light when some of these scattered light waves enter your eye.

Opaque, Transparent, and Translucent Materials

The three candleholders in Figure 7 are made of different materials. These materials absorb, transmit, and scatter light in different ways. The candleholder on the left is made from an opaque material. An opaque material only absorbs and reflects light—no light is transmitted through it. The middle candleholder is translucent. A trans- lucent material allows some light to pass through, but scatters light so you cannot see clearly through it. The candleholder on the right is transparent—it transmits nearly all the light that strikes it.

The Speed of Light in Different Materials

Light waves and all electromagnetic waves travel through empty space at a speed of about 300,000 km/s. This speed is called the speed of light. No object or wave can move faster than the speed of light in empty space.

However, when light waves travel in matter, they move more slowly. Figure 8 compares the speed of light in different materials. Light waves slow down in a material because they interact with the atoms and molecules in the material.

Refraction

When light rays move from one medium to another, such as from air to water as shown in Figure 9, they can change direction.

Refraction occurs when a light ray changes direction when it moves from one material into another.

Refraction and Speed Changes

Why does the light beam in Figure 9 change direction? Light waves change direction, or refract, whenever they change speed in moving from one medium into another. The light beam bends because light waves slow down as they move from air into water. The greater the difference in speed between the two materials, the greater the amount of refraction. However, refraction does not occur for waves that are traveling perpendicular to the boundary between the materials. Figure 10 shows how the refraction of light waves causes a straw in water to look like it is broken.

Refraction and the Visible Spectrum

A prism, like the one in Figure 11, is a piece of transparent glass or plastic that is usually shaped like a wedge. When light rays pass through the prism, they change direction. Light waves slow down as they move from air into the glass prism. This causes light waves to refract when they enter the prism. Light waves speed up and refract when they move from the prism back into the air.

Refraction produces the rainbow of colors shown in Figure 11. White light is a combination of all light waves in the visible light spectrum.

When white light passes through the prism, light waves with different wavelengths are refracted by different amounts. Violet light waves have the shortest wavelengths and are bent the most. Red light waves are bent the least.

White Light

You know that passing white light through a prism separates the white light into the visible light spectrum. But does the process work in reverse? Can you combine different colors of light to make white light? Figure 12 shows that it is possible to make white light by mixing colored lights. You can also mix colors together to make other colors. For example, where the green and red light overlap, there is yellow light. Red, green, and blue light are called the primary colors of light. Almost any color of light can be made by mixing these three colors in different amounts.

Reflection

Light waves usually travel in straight lines in a material or through space. However, light waves can change direction when they speed up or slow down. Light waves also change direction when they are reflected from a surface. When light rays are reflected, the direction of the reflected ray depends on the direction of the incoming light ray that strikes the surface.

The Law of Reflection

The direction of a reflected light ray is determined by the law of reflection, as shown in Figure 13. The incoming ray and the reflected ray make an angle with a line perpendicular to the sur- face. The line perpendicular to the surface is called the normal to the surface. The angle of incidence is the angle between the incoming light ray and the normal. The angle of reflection is the angle between the reflected ray and the normal. According to the law of reflection, when a light ray is reflected from a surface, the angle of incidence equals the angle of reflection. Light rays reflected from all surfaces always obey the law of reflection.

Regular and Diffuse Reflection

Although the surface of a sheet of paper might seem smooth, it’s not as smooth as the surface of a mirror. Figure 14 shows how the rough paper surface reflects light rays in many directions. Each light ray reflected from the uneven surface of the paper obeys the law of reflection. But each one hits a surface that is at a slightly different angle. This means the light rays that are parallel before they hit the surface end up going many different directions after they strike the surface. This reflection from a rough surface is called diffuse reflection.

However, the smooth surface of a mirror reflects parallel light rays so that they remain parallel. Reflection from a mirror is called regular reflection. Whether a surface is rough or smooth, all light rays that strike the surface obey the law of reflection.

Reflection and Color

Look around the room. Notice the colors of different objects.

Why do some things look red while others appear green? Figure 15 illustrates why different objects have different colors. As white light strikes an object, some of the light is absorbed and some is reflected. The reflected light is what enters your eyes and causes you to see the object. For example, what makes the flower look red? The flower looks red because the materials in the flower absorb all wavelengths of light except red. The red light is reflected to your eyes, and you see the flower as red.

Using Lenses

Lenses form images by causing light rays to bend.

Real-World Reading Connection Have you ever used a cam- era to take a picture of a friend? All cameras record the light that is emitted or reflected by objects. Cameras, microscopes, and telescopes use lenses to form images that you can see.

What is a convex lens?

You are probably familiar with different devices that change how you see things. Eyeglasses change the way light is focused on a person’s eye. Magnifying lenses and microscopes make very small objects appear to be large. Telescopes and binoculars make objects that are far away appear to be closer. All of these devices use at least one lens to form images. A lens is a transparent object with at least one curved side that causes light waves to bend. Figure 16 shows two different types of lenses.

A lens that bulges outward, such as the one shown in the left photo in Figure 16, is called a convex lens. Parallel light rays passing through a convex lens are bent so they come together, or converge. As shown on the right of Figure 16, a con- cave lens is thinner in the middle than at the edges. Parallel light rays passing through a concave lens spread apart, or diverge.

Light’s Path Through a Convex Lens

Convex lenses are usually made out of glass or plastic. Light travels more slowly in both glass and plastic than it does in air. This means that a light ray bends when it slows down as it moves from air into the lens. The light ray bends again when it speeds up as it moves from the lens back into the air.

Focal Point and Focal Length

Figure 17 shows several beams of light shining parallel to the optical axis of a convex lens. These beams of light all bend toward the optical axis. The point where all of the beams of light converge is called the focal point. In a convex lens, all light rays traveling parallel to the optical axis are bent so that they pass through the focal point. The distance from the center of the lens to the focal point is called the focal length.

Image Formation by a Convex Lens

Figure 18 shows that the image formed by a convex lens depends on the position of an object in relation to the focal point. Notice that the images in the first two panels appear where the light rays converge. The light rays never converge in the third panel. Instead, they diverge from the lens, forming an image that is right- side up, on the same side of the lens as the flower and bigger than the flower.

Optical Instruments

Have you ever thought about why it’s so hard to see objects that are far away? An optical instrument uses lenses to focus light and create useful images. Often, an optical instrument acts as a bigger eye by collecting more light than your eyes can collect. It gathers the light and then forms an image that your eyes can see. Different optical instruments do this by combining lenses in various ways.

Cameras

A typical camera, like the one shown in Figure 19, uses several lenses to form an image. The camera is focused by moving the lenses back and forth until a sharp image is formed. The image is smaller than the object and is upside down. In some types of cam- eras, the image is formed on a section of film. In digital cameras, the image is formed on an electronic light sensor. When you take a picture, the camera shutter opens so that light enters the camera, and the film or the electronic sensor is exposed.

If too much light strikes the film or the light sensor, the image formed is overexposed and looks washed out. If too little light enters the camera, the photograph can be too dark. To control the amount of light that reaches the film or the light sensor, cameras have a device called a diaphragm or an aperture. The opening in the aperture becomes larger to let more light into the camera. The aperture opening becomes smaller to reduce the amount of light that enters the camera.

Refracting Telescopes

Why is it hard to see far-away objects clearly? As an object gets farther away, less of the light from the object enters the openings in your eyes. As a result, the object appears dimmer and less detailed.

A telescope is an optical instrument that makes far-away objects seem closer. There are two basic types of telescopes. A simple refracting telescope is a combination of two convex lenses in a tube, as shown in Figure 20. The larger lens is the objective lens. The objective lens forms an image, which is enlarged by the smaller eyepiece lens.

The objective lens in a refracting telescope is much larger than the opening in a human eye. This means that much more light from a distant object enters the objective lens than would enter an eye. This causes the image formed by the objective lens to be brighter than the image your eye would form. Because the image is brighter, more detail can be seen when the image is magnified. Making the objective lens larger lets more light pass through the lens.

Then even clearer images can be formed.

Reflecting Telescopes

The second type of telescope is a reflecting telescope. In a reflecting telescope, the objective lens is replaced with a mirror that has a curved reflecting surface. An image of a distant object is formed inside the telescope tube when light rays are reflected from the curved surface.

A simple reflecting telescope is shown in

Figure 21. Light from a distant object enters one end of the tube and strikes the curved mirror at the other end. The light is reflected from the curved mirror to a flat mirror inside the tube. The flat mirror then reflects the light to an eyepiece lens, which magnifies the image.

Large Telescopes

In order to form detailed images of very distant objects, such as planets and galaxies, the objective lens or curved mirror of a telescope must be as large as possible. Because a lens can be supported only around its edges, very large lenses tend to sag due to their weight. However, a large mirror can be supported rigidly on its back side so that it doesn’t sag. As a result, the largest telescopes are reflecting telescopes instead of refracting telescopes. Figure 22 shows one of the largest telescopes in the world, the Hale telescope at Mount Palomar Observatory in southern California.

Microscopes

A refracting telescope uses convex lenses to enable distant objects to be seen. A microscope uses convex lenses to make a small object appear larger. Figure 23 shows a simple microscope.

Light from the object passes through the objective lens. The objective lens is positioned so that it forms an enlarged image of the object. The light rays from that image then pass through the eyepiece lens. This lens is positioned so it is closer to the image than one focal length. As a result, the image is made even larger. By using more than one lens, a microscope forms a much larger image than a single lens can produce.

The Eye and Vision

The eye is a complex organ made up of different parts.

Real-World Reading Connection Cameras, telescopes, and microscopes all use lenses to form images. Did you know that your eyes also contain lenses? Like other optical instruments, the human eye uses refraction to form images.

How the Eye Forms an Image

Your eye detects light that is emitted by or reflected from objects. In some ways an eye is similar to a camera, as shown in Figure 24. In a camera, light from an object enters the lens. The lens forms an image on the film or light sensor at the back of the camera. The film or light sensor then records the image.

As light enters your eye, lenses in your eye focus light to pro- duce an image on the back of your eye. Special cells at the back of the eye convert the image into electrical signals. These signals then travel to your brain, where they are interpreted as the object you are looking at.

Cornea

Figure 25 shows the different parts of the human eye. The eye is roughly spherical and is about 2.5 cm in diameter. The outer layer of the eye is called the sclera. The front part of the sclera is clear.

Light enters your eye through the cornea (KOR nee uh), which is a clear area of the sclera. The cornea is a convex lens that causes light rays to converge as they enter the eye. Although the eye contains another convex lens that helps focus light rays, most of the refraction of light rays occurs when they enter the cornea.

Iris

After passing through the cornea, light rays then pass through the pupil. The pupil is the dark opening into the interior of your eye. The pupil is surrounded by the iris, which is the colored part of your eye behind the cornea. The pupil and the iris are shown in Figure 26.

The amount of light that enters the inside of your eye is con- trolled by the iris. When the light is dim, your iris is small and the pupil is large. This allows more light to enter the interior of your eye. When the light is bright, your iris is larger and your pupil is smaller, so that less light enters your eye.

Lens

After passing through the pupil, light rays pass through the lens. The lens in your eye is convex, like the lens in a magnifying glass.

However, instead of being made of rigid glass or plastic, the lensbin your eye is flexible. The ciliary muscles attached to the lens change its shape, as shown in Figure 27. When you look at objects that are farther away, the muscles contract. This flattens the lens. When you look at objects that are closer, the muscles relax. This makes the lens rounder. By changing its shape, the lens enables sharp images of both nearby and distant objects to be formed on the retina.

Retina

The light rays that pass through the lens form an image on the retina of the eye. The retina is a sheet of light-sensitive cells in the back of the eye. As shown in Figure 28, the retina contains two types of cells, called rods and cones. When these cells absorb light energy, chemical reactions occur. These chemical reactions pro- duce nerve impulses that are transmitted to the brain by the optic nerve. Rod cells respond to dim light. Cone cells enable you to see colors but need brighter light to function than rod cells. Table 1 summarizes the structures in the eye and their functions.

Seeing Color

How do cone cells enable you to see color? You have about seven million cone cells in each retina. Light waves reflected from objects enter the pupil and strike the retina. The response of the cone cells to different wavelengths of light causes you to see objects as having color.

Three Types of Cone Cells in the Retina

There are three types of cone cells. Each type responds to differ- ent wavelengths of light. One type of cone cells responds to the wavelengths of red and yellow light. These cells cause you to see the color red. The second type responds to yellow and green light and causes you to see the color green. The third type responds to blue and violet light and causes you to see the color blue.

Light waves that strike the retina cause the three types of cone cells to send signals to the brain. The brain interprets the combination of the signals from the cone cells as the various colors you see.

Pigment Colors

Some colors of the objects you see are caused by pigments. A pigment is a material used to change the color of other materials or objects. The color of a pigment, such as paint, depends on the wavelengths of the light it reflects. Blue paint reflects blue light and absorbs all other wavelengths. As shown in Figure 29, there are three primary pigment colors—magenta, cyan, and yellow.

Figure 29 also shows that each primary pigment color absorbs one of the primary light colors—red, green, or blue—and reflects the other primary colors. Most colors can be made by mixing different amounts of the primary pigment colors.

Color Printing

Look at a color picture in a magazine with a magnifying lens. As shown in Figure 30, the picture is formed by many tiny dots of color.

Only four colors of dots are used to make all the different colors in the picture. These four colors are usually the primary colors of pigments—magenta, cyan, and yellow—as well as black. The four colors are combined in dots that are too small for the human eye to see clearly. As a result, the light reflected by the dots combines to make all the colors you see in magazines. This book was printed using four- color printing.

Common Vision Problems

If you have normal vision, you should be able to see objects clearly when they are 25 cm or farther from your eyes. Also, you should be able to detect all colors of visible light. How- ever, some people cannot detect certain colors. Also, many people have problems seeing nearby objects or distant objects.

Color Deficiency

Take a look at Figure 31. Do you see a number? If not, you might have a red-green color deficiency. About 8 percent of males and

0.4 percent of females have a difficult time telling the difference between red and green. People with this deficiency either lack green or red cones or they have green or red cones that do not function correctly.

Nearsightedness

You probably know someone who wears glasses or contacts, or maybe you wear them yourself. Two common vision problems that glasses correct are nearsightedness and farsightedness.

A nearsighted person cannot see faraway objects clearly. As Figure 32 shows, in a nearsighted eye a sharp image is formed in front of the retina. The image on the retina is blurry. A concave lense causes light rays to diverge before they enter the eye. Then the cornea and the lens can form a sharp image on the retina.

Farsightedness

A person who is farsighted cannot see nearby objects clearly. As shown in Figure 33, a sharp image of a nearby object would be formed behind the retina. Glasses with convex lenses make light rays converge more before they enter the eye. Then a sharp image is formed on the retina.

Sound

Sound is a compression wave.

Real-World Reading Connection If you stop reading and pay attention to all of the sounds around you, you will probably hear many different noises. You might hear cars passing by on the street. You might hear voices speaking in another room. Per- haps you will hear someone playing music nearby. There might be the hum of a computer or an air conditioner.

What is sound?

All of these noises are perceived by your ear as sound. These sounds travel like a ripple travels in water. Every sound has something in common with every other sound. Vibrations make all sounds, and all sounds are transmitted by waves. Have you ever played with a coiled-spring toy like the one in Figure 1? If you stretch it out and then give one end a little push, you create a section of coils that are more tightly spaced than the coils in the rest of the spring. You can watch as a region of compressed coils travels all the way to the other side of the spring. In the same way, sound waves compress the molecules through which they travel. These waves travel through a medium, the air, to be received by your ear.

Sound Waves

In the last chapter, you learned that light is a wave. Recall that waves are disturbances that carry energy from one place to another place. Sound is also a wave. When you speak, the vocal cords in your throat vibrate. Your vocal cords move the air in your throat, and the vibrations continue as a wave through the air. The energy that moves one molecule of air is passed on to the next one. The wave moves through the air in your throat to the air sur- rounding your body and beyond. In this way, energy is transferred from one place to another.

Compression Waves Sound waves are called compression waves. In a compression wave, particles move back and forth in the same direction the sound wave moves. Figure 2 illustrates the molecules in a sound wave. In some places of the wave, the molecules are crowded closely together. These areas of high density and pressure are regions of compression. In between two regions of compression, the molecules are spread apart. This area of low density and pressure is a region of rarefaction.

Wavelength and Frequency Like other waves, a sound wave has a wavelength and a frequency. The wavelength of a sound wave is the distance between the centers of two regions of compression or the distance between the centers of two regions of rarefaction, as shown in Figure 2. Just as for other waves, the frequency is the number of wavelengths that pass a certain point in one second.

Recall that frequency is measured in hertz, which is symbolized Hz and represents 1/s. If a wave has a long wavelength, only a few wavelengths will pass a point in one second, so the wave will have a low frequency. An example of this would be the bass drum: the low notes it produces have a low frequency and a long wavelength and can be felt through your whole body.

Vibration

Have you ever put your hand on a stereo speaker? If so, you probably felt it vibrating. When an object vibrates, it puts pressure on the surrounding molecules. As the speaker moves outward, as in Figure 3, it pushes molecules together and creates a region of compression. When it moves inward, it creates a region of rarefaction, also shown in Figure 3. As the speaker continues to vibrate, a series of compressions and rarefactions forms. The compressions and rarefactions move away from the speaker in all directions, creating a sound wave.

Playing in Tune

What does it sound like when you play a musical instrument, such as a violin, that is properly tuned? It probably sounds rice and full. When you play middle C on a violin that has been properly tuned, the string vibrates in a certain pattern that makes the note middle C. However, the vibration of the string is too complex to produce just one note at a time. It also produces vibrations that match middle C in the same way that some notes on a scale match each other. This creates a rich sound that you expect from the violin.

What does it sound like when you play a violin that is out of tune? It probably doesn’t sound very good. If the primary note, such as middle C, is not quite right, the other vibrations that would normally match it do not blend together. Because the sounds are not organized, this can be considered noise.

How loud is it?

You’ve probably listened to music and had someone say to you, “Your music is too loud. I can’t hear myself think!” You’ve also probably whispered in your quietest voice to a friend and had a teacher say, “Do you want to share that with the group?” What is the difference between a loud noise and a quiet noise?

Amplitude and Loudness

Loud sound waves generally carry more energy than soft sound waves do. Loudness is a person’s perception of how much energy a sound wave carries. Because of the structure of the ear, not all sound waves with the same energy have the same loudness. Humans hear sounds with frequencies between 3,000 Hz and 4,000 Hz as louder than other sound waves with the same energy.

Loudness is measured in units called phons. Two sounds of different frequencies produced with different amounts of power will not sound equally loud. Nor will two sounds produced with the same power at different frequencies sound equally loud. The phon takes both frequency and power into account to measure loudness.

The amount of energy a wave carries depends on its amplitude.

For a sound wave, amplitude depends on how spread out the molecules in the regions of compression and rarefaction are, as shown in Figure 4. If a sound wave has compressions with molecules that are packed tightly together and rarefactions with molecules that are spaced widely apart, it has a large amplitude. The object that created the wave transferred a lot of energy to the molecules to force them tightly together or to spread them far apart. Sound waves with greater amplitudes carry more energy and sound louder. Sound waves with smaller amplitudes carry less energy and sound quieter.

The Decibel Scale

Your perception of the amount of energy in a sound wave is measured on the decibel (DES uh bel) scale, as shown in Figure 5. The symbol for a decibel is dB. Because humans can hear sounds over a large range, the decibel scale is based on powers of 10. For example, a sound of 0 decibels is the quietest sound that humans can hear. A sound of 10 decibels has ten times more energy than a sound of 0 dB. A sound that is 20 dB has 100 times more energy. A sound that is 30 dB has 1,000 times more energy.

The Decibel Scale

Your perception of the amount of energy in a sound wave is measured on the decibel (DES uh bel) scale, as shown in Figure 5. The symbol for a decibel is dB. Because humans can hear sounds over a large range, the decibel scale is based on powers of 10. For example, a sound of 0 decibels is the quietest sound that humans can hear. A sound of 10 decibels has ten times more energy than a sound of 0 dB. A sound that is 20 dB has 100 times more energy. A sound that is 30 dB has 1,000 times more energy.

Why is a 20-dB sound not twice as loud as a 10-dB sound?

Decibels that Damage

Damage to a person’s hearing can begin with sounds of about 85 dB. The type of damage depends on the loudness of the sound and the length of time that a person is exposed to the sound. For example, listening to an 85-dB sound for more than eight hours can cause hearing damage. Some everyday sounds that are about 85 dB include garbage disposals, lawn mowers, heavy traffic, and noisy restaurants. Listening to a 110-dB sound for just a minute and a half can cause hearing damage. A car horn, a video arcade, a power saw, and a crying baby all produce sounds of about 110 dB.

Hearing Protection How do you protect your hearing? You should wear earplugs or hearing protectors at concerts or when around loud machinery. Workplaces have laws about how long their employees can be exposed to noises between 90 and 115 decibels. Noises above 140 decibels are illegal for worker exposure. Because of the way the decibel scale is constructed, sounds do not add as you might expect. If three people are holding a conversation and suddenly start talking at once, one at 60 dB, another at 65 dB, and the third at 70 dB, how loud do you think it will seem? It actually adds to only

71.5 dB, not much greater than the third person talking alone. However, the effect of three people talking at once is still greater than one per- son alone.

Frequency and Pitch

Have you ever been in a car when the driver revved the engine? It makes a higher sound than when the car is idling, because the engine shaft completes more revolutions per minute. Recall that the frequency of a sound is measured by the number of cycles the object causing the sound completes per second. The faster it moves, the higher the frequency is. Pitch corresponds to the frequency of the sound.

The pitch of a sound is how high or how low it is. The higher the pitch is, the higher the frequency is. The words pitch and frequency often mean the same.

The human ear can detect sound waves with frequencies between about 64 Hz and 23,000 Hz, shown in Table 1. Some animals can hear sounds with even higher frequencies. For example, when a trainer blows a dog whistle, you do not hear anything, but a dog will respond. Dog whistles have frequencies that are higher than the human ear can detect. Other animals that can detect high-frequency sound waves include bats, whales, and dolphins.

The Ear and Hearing

The ear detects sound waves that pass through the three parts of the ear and bend small hairs in the inner ear.

Real-World Reading Connection You may have used a tape recorder to record sounds. Have you ever thought about how a tape recorder works? In some ways it is similar to your ear, as Figure 6 shows.

Functions of the Ear

The outer part of your ear collects sound waves. The micro- phone connected to a tape recorder serves a similar purpose. Your middle ear passes the sound waves you hear to your inner ear. Similarly, sound waves in digitized form are passed through some wires to the inside of the tape recorder. Finally, your inner ear processes the sound waves into electrical impulses so that your brain can interpret what you hear. A tape recorder records sounds on tape so that they can be played back at a future date.

Structures of the Ear

The human ear is made up of the outer ear, the middle ear, and the inner ear, as shown in Figure 7. The outer ear includes the external ear and the auditory canal; it collects sound waves and transfers them to the middle ear. The middle ear contains the ear- drum and three tiny bones that conduct sounds to the inner ear.

The inner ear contains intricate structures that detect different frequencies of sounds and sends signals to the brain.

The Outer Ear

If someone asked you where your ears are, you would probably point to the visible parts on either side of your head. Technically, these are the external ears, and they are just one part of the outer ear. The external ear is made up of folds of cartilage and skin.

These folds adjust the sound waves that enter your ear to help the brain interpret the origin of the sound waves.

The other part of the outer ear is called the auditory canal. The auditory canal is the part of your ear that collects sound waves from the external ear and passes them to the middle ear. You can easily find the beginning of the auditory canal. It is the opening in your ear that leads into your middle ear.

The Middle Ear

You have probably heard someone say, “Don’t listen to such loud music or you’ll burst your eardrums.” But what exactly are the eardrums? As shown in Figure 8, the first part of the middle ear that sound waves reach is called the tympanic membrane, more commonly called the eardrum. It is a thin layer of skin that vibrates when sound waves hit it.

The vibrations of the tympanic membrane cause three tiny bones to transmit sound waves through the middle ear to the inner ear, as shown in Figure 8. These bones are known by their Latin names: the malleus, also called the hammer; the incus, also called the anvil; and the stapes, also called the stirrup. Two small muscles control the tension on the eardrum and the bones of the middle ear. By changing the tension, these muscles can help protect the eardrum from large vibrations that come from loud sounds.

The malleus, the incus, and the stapes rest in a fluid-filled vessel called the eustachian tube. The eustachian tube connects the middle ear to the throat. When you swallow, the pressure is adjusted in your eustachian tube and in your middle ear. This is why it helps to chew gum and swallow or to yawn when the pressure changes on airplanes or in elevators.

The Inner Ear

If you have ever walked through a maze in a cornfield, a cathedral, an amusement park, or a formal garden, you know that a maze is full of twists and turns. Another name for a maze is a labyrinth. The inner ear is full of structures with so many twists and turns that it is also called a labyrinth. All the twists and turns in the inner ear allow many sensory cells to be packed into a small space.

When the bones of the middle ear move, they transfer vibrations from the tympanic membrane to the inner ear by way of a mem- brane called the oval window. The oval window then passes the vibration on to the fluid in the cochlea. The cochlea, shown in Figure 9, is a snail-shaped structure. The cochlea is lined with sensory cells. These cells sense different frequencies of sound. Figure 10 shows a diagram of what the cochlea would look like if it were unwound.

The sensory cells closest to the middle ear sense high-frequency sound waves. The sensory cells farther from the middle ear sense low-frequency sound waves.

Sensing Sound

When you smell or taste something, the signals that are sent to your brain come from chemical changes in your cells. Sound is different. When you hear something, it is because of a movement. As shown in Figure 11, all of the sensory cells in the cochlea have little hairs sticking out of them. When a vibration hits the hairs, they bend. The cells react to the bent hairs by sending a signal to the brain.

Hearing in Humans

Remember that a sound wave is a compression wave. When a sound wave hits the tympanic membrane, it causes a vibration. The vibration is passed to the three tiny bones in your inner ear. The last of these bones, the stapes, vibrates against a part of the cochlea called the oval window. The oval window then passes the vibration on to the fluid in the cochlea.

The vibration of the fluid in the cochlea bends the hairs in the sensory cells. When the hairs bend, a signal is sent to the brain. The brain can tell the frequency of the sound wave by determining the location of the bent hairs. The brain can also determine the loudness of a sound. The farther a hair is deflected, the louder the brain recognizes the sound to be.

Hearing Damage

Hearing damage can occur in almost any part of the ear. If something happens to prevent sound from being transmitted through the outer and middle ear, conductive hearing loss occurs.

Effect on Ear Structures

Conductive hearing loss is often caused by colds or allergies that cause a temporary buildup of fluid in the middle ear. This can lead to an infection that puts pressure on the eardrum. When the ear- drum does not vibrate properly, vibrations are not passed to the cochlea correctly, so a person cannot hear clearly.

Damage to the cochlea can occur because of aging or from exposure to loud noises. When the hairs on the sensory cells in the cochlea are bent for too long or too much, they can become dam- aged. This can happen to musicians who spend a lot of time per- forming at loud concerts, to people who ride motorcycles, or to people who work around loud machinery. Damage to the cochlea is called sensorineural hearing loss.

Correcting Hearing Damage

Conductive hearing loss can usually be treated. If fluid as a result of infection has accumulated in the middle ear, medicines can clear up the infection. Once the problem is corrected, hearing usually returns to normal.

Sensorineural hearing loss is permanent. It can be corrected only with hearing aids or other devices that increase the ability to hear. People who have badly damaged inner ears can have cochlear implants surgically inserted in their ears, as shown in Figure 12. A person with a cochlear implant hears differently from people with normal cochlea, but the implant allows for full communication.

Hearing in Other Mammals

Most mammals use a system for hearing that is similar to the hearing system of humans. However, some mammals possess qualities that allow them to hear certain sounds better than humans can.

Redirecting Sound

Have you ever watched a dog or a cat move its ears around? You might have noticed that these animals rotate their ears very often, especially when they are tracking or hunting. As shown in

Figure 13, some mammals use their external ears to redirect sound into their auditory canals. This helps them to determine the direction from which the sound is coming and also can help them to detect faint sounds, giving them the ability to hear predators and prey easily.

Echolocation

Sound waves can reflect off hard surfaces, just as a light wave reflects off a shiny surface. A reflected sound wave is called an echo. The amount of time it takes an echo to bounce back depends on how far away the reflecting surface is.

Some mammals use a method called echolocation, shown in

Figure 14, to navigate and hunt. Bats, for example, make high-frequency calls and then listen for the echoes. When a bat hears an echo, it can determine the position and identity of objects in its path. Dolphins and whales also use echolocation. By making clicks that bounce off objects in the ocean, they are able to navigate.

The Human Ear

The human ear is made up of many tiny parts that work together to interpret sound waves for the brain. Sound is con- ducted through tiny bones and fluid-filled chambers, where the deflection of tiny hairs determines the type of sound a person hears. Human hearing can be temporarily or permanently dam- aged by illness or injury to the ears. Treatments can restore permanent hearing loss only partially, so it is important to avoid damage to begin with.

Some animals are able to move their outer ears to better collect sound, and other animals are able to determine the position of objects by the reflection of the sounds the animals make.

Reproductive Systems

The structures of the human reproductive systems are specialized for the production of offspring.

Real-World Reading Connection

It’s the first day of school. You notice that some students changed a lot over the summer but others hardly changed at all. Although you and your class- mates are about the same ages, you are at different stages of physical development. Your reproductive systems are at different stages of development, too.

Male Reproductive System

Adult males have many body characteristics that differ from adult females. Men usually have more body hair, deeper voices, and larger, more muscular bodies than women do. These features develop as boys get older and their reproductive systems grow toward maturity.

As you read in Chapter 3, testes (singular, testis) are male animal organs that produce sperm. Human males have two testes, as shown in Figure 1. A human male’s testes do not begin to produce sperm until his reproductive system matures.

Male Reproductive Organs

The testes are inside a baglike structure called the scrotum. It hangs outside the male’s body cavity, which keeps the testes slightly cooler than the rest of the body. Normal human body temperature is too warm for sperm production. The cooler temperature in the scrotum enables sperm production.

Organs of Sperm Production

The testes contain tightly coiled tubes called seminiferous tubules, where sperm are produced. As shown in Figure 2, sperm travel from seminiferous tubules to a storage organ within the scrotum called the epididymis. It connects to muscular ducts or tubes called the vas deferens.

Organs of Sperm Transfer

The male organ that transfers sperm to a female’s reproductive tract is a penis. During sexual activity, sperm move from the vas deferens into a short ejaculation duct that connects to a tube called the urethra. The urethra extends to the end of the penis and carries sperm out of the body. The urethra also carries urine, but ejaculation and urination never occur at the same time.

When a male is sexually excited, the tissues of the penis fill with blood. This extra blood causes an erection, or firming of the penis. An erection is needed for the penis to enter a female’s reproductive tract. During an ejaculation, sperm leave the epididymus, enter the vas deferens, move to the urethra, travel down the urethra, and out the end of the penis. It is important to know that sperm can leave the penis without the male’s knowledge, either before ejaculation or during sexual activity that does not result in ejaculation.

Sperm Production

Males start producing sperm during **puberty**, which usually begins when they are 10–16 years of age. Sperm production occurs by meiosis in cells that line the seminiferous tubules, as shown in Figure 3. It takes 65–75 days to produce a mature sperm cell, also shown in Figure 3. A male can continue to make healthy sperm for the rest of his life.

Sperm and Semen

Each sperm consists of a head, a midpiece, and a tail. The head contains a nucleus, and the midpiece contains mitochondria that release energy. The tail, or flagellum, whips back and forth and propels the sperm forward.

A male ejaculates about 2–5 mL of semen on average. Semen contains a liquid made by glands in a male’s reproductive system and about 100 to 650 million sperm. The seminal vesicles are a pair of glands that makes most of the liquid in semen. They produce a thick, yellowish liquid that contains mucus, ascorbic acid (vitamin C), hormonelike substances that control cell activity, and an enzyme that helps thicken the semen. This liquid also contains sugar that is an energy supply for sperm. The prostate gland also makes some of the liquid in semen. It produces a thin, milky liquid containing enzymes and nutrients.

Female Reproductive System

A female’s reproductive system produces eggs. This system is also the place where a fertilized egg can grow and develop into a baby. Recall that a male begins producing sperm when he reaches **puberty**. A female begins producing eggs before she is born.

Female Reproductive Organs

Unlike a male, all the reproductive organs of a female are located inside her abdomen, as shown in Figure 4. Two folds of skin, called labia (LAY bee uh), protect the opening to a female’s reproductive system. Beyond the opening, inside the female’s body is a thin-walled chamber called the vagina. This is where semen is deposited.

Uterus Above the vagina, further inside the body, is the uterus. It is a thick, muscular organ inside which a fertilized egg can develop. A uterus is normally about the size and shape of a pear, but it enlarges during pregnancy. A tissue called the endometrium lines the uterus. The neck, or opening, of the uterus into the vagina is called the cervix. During childbirth, the cervix gets wider, or dilates. This enables the baby to move into the vagina and out of the mother’s body.

Ovaries and Fallopian Tubes A pair of organs called ovaries produces eggs. An egg released from an ovary moves into a fallopian tube or oviduct that connects the ovary to the uterus, also shown in Figure 4. Fertilization usually occurs while the egg is in a fallopian tube. An egg cell has no flagellum, so it cannot move on its own like a sperm cell can. Recall from Chapter 1 that the surface of a cell can have hairlike structures called cilia that move back and forth. The cells on the inside surface of a fallopian tube have cilia. These cilia move an egg toward the uterus.

Egg Production

Cell division by meiosis produces a human egg, as shown in Figure 5. Before a female is born, cells in her developing ovaries begin meiosis, but stop at the first phase, **prophase I**. The cells stopped at **prophase I** are called primary oocytes (OH uh sites). They remain unchanged until a female begins **puberty**.

**Puberty** in a human female usually begins between the ages of 9 and 13. At **puberty**, a female’s body begins producing chemical signals that cause primary oocytes to continue meiosis. However, meiosis stops again at the second stage of meiosis, **metaphase II**. The cells stopped at **metaphase II** are called secondary oocytes.

Secondary oocytes are the egg cells. A female usually produces only one egg cell every four weeks on average. An egg cell does not complete meiosis until fertilization occurs.

Cells of the ovary surround, protect, and nourish each egg cell. A follicle is an egg cell and its surrounding cells. A female at puberty has about 400,000 follicles. The release of an egg from a follicle into a fallopian tube is called ovulation also shown in Figure 5.

Menstrual Cycle

Before a follicle releases an egg, other changes happen in a female’s body. The changes that take place before, during, and after ovulation are called the menstrual cycle. As illustrated in Figure 6, a menstrual cycle lasts about 28 days. The first day of a menstrual cycle is the first day of menstrual bleeding, or menstrual flow.

Menstrual Flow During the menstrual cycle, the endometrium thickens and the number of blood vessels in it increases to support a fertilized egg. However, if a released egg is not fertilized, the endometrium breaks down and sloughs off. This tissue, some blood, and the unfertilized egg leave the vagina as menstrual flow. Menstrual flow usually lasts four to seven days. After menstrual flow stops, the endometrium thickens and its blood vessels regrow.

Ovulation About two weeks after the first day of menstrual flow, ovulation occurs. Usually, only one egg is released from one of a female’s ovaries during a menstrual cycle. It takes about 24 to 48 hours for an egg to move down the fallopian tube and into the uterus. If the egg is fertilized, a zygote forms, cell divisions begin, and an embryo begins to develop. When the embryo enters the uterus, it attaches to, or implants in, the endometrium. If this happens, menstrual bleeding does not occur. The absence of menstrual bleeding is usually one of the first signs of pregnancy.

Hormones Chemical messengers called hormones regulate the timing of the menstrual cycle and ovulation. Some of the glands and organs that produce hormones, including hormones that regulate menstrual cycles, are shown in Figure 7 on the following pages.

Menopause

Sometime between the ages of 46 and 54, most women stop ovulating and no longer have menstrual cycles. This stage of life is called **menopause**. Many women begin to lose the ability to reproduce before menopause, as early as their mid-30s.

As a woman gets older, the eggs she produces decrease in quality, and it becomes more difficult for her to have a successful pregnancy. An important difference between males and females is that a male’s reproductive system continues to function throughout his lifetime, but a female’s reproductive system does not.

Fertilization

Have you ever heard people say they were in the right place at the right time? The same might be said about sperm and fertilization. In humans, for a sperm to fuse with an egg cell, the sperm must swim to the right place—a fallopian tube—and at the right time—near the time of ovulation.

Sperm deposited in or near a female’s vagina can swim into her reproductive tract, as shown in Figure 8. Most of these sperm will not make it to an egg. Some sperm swim into a fallopian tube that does not contain an egg. Some sperm swim in the opposite direction, away from the fallopian tubes. Other sperm might have genetic or physical defects that prevent them from fertilizing an egg even if they reach it. These facts help explain why millions of sperm are ejaculated to fertilize just one egg.

Normally, only one sperm fertilizes an egg, as shown in Figure 8. Once a sperm attaches to an egg, chemical reactions occur that block other sperm from entering the same egg. Sperm can live inside a female’s reproductive tract for up to three days. Therefore, a female can become pregnant even if sexual inter- course occurs a couple days before she ovulates.

Development Before Birth

The normal development of a fetus depends on the good health of its mother.

Real-World Reading Connection Did you know that some- one might have taken your picture before you were born? A sonogram uses sound waves to produce a video image of a fetus. It can help a medical provider determine if the fetus is developing normally and whether it is a girl or a boy.

Fetal Development

Recall from Chapter 3 that all sexually produced organisms begin life as a zygote that forms when a sperm fertilizes an egg. Cell divisions of a human zygote begin about 24 hours after fertilization. Cells continue to divide and, after about seven days, a hollow ball of more than 100 cells has formed, as shown in Figure 9. This ball of cells is the embryo that implants into the endometrium. After two weeks of growth, the cells begin to arrange themselves into three layers, also shown in Figure 9. Different body structures eventually form from each layer. Over a period of about nine months, a human embryo develops into a baby.

Growth and Development of Body Systems

The development of a baby within a female’s uterus is called pregnancy. In humans, pregnancy usually lasts for 38 weeks after fertilization, or about 40 weeks after the beginning of the last menstrual cycle. When describing the many changes that take place during pregnancy, it is helpful to divide the nine months of pregnancy into three parts, called trimesters.

The first trimester is the first twelve weeks of pregnancy. By the end of the first trimester, an embryo has all the structures that will become the major organ systems of an adult. During the second and third trimesters, an embryo is called a fetus. A fetus changes as it continues to develop, as shown in Table 1.

During the second trimester, the pregnant female can feel the fetus’s movements. During the third trimester, the fetus grows rapidly, nearly tripling in size in preparation for birth.

Premature Babies

Sometimes, infants are born prematurely, before development is complete. Premature babies can have difficulty surviving because some of their organs are not ready to function. The lungs are among the last organs to develop fully. Premature babies must often be cared for in the hospital until their lungs develop completely. Also, premature babies usually have low birth weights. Extremely premature and low birth weight infants can have physical challenges, learning difficulties, or behavioral problems as they grow older.

Placenta and Umbilical Cord

During development, a growing fetus receives oxygen and nutrients from its mother. A pregnant woman receives carbon dioxide and other wastes from her fetus. This exchange of materials between a pregnant woman and her fetus takes place through a disk-shaped organ called the placenta. A placenta begins to form when an embryo first implants into the endometrium. It develops from tissues of both the fetus and the endometrium. The placenta contains many blood vessels from both the fetus and its mother, but they are not directly connected.

Substances enter and leave the body of a fetus through an umbilical cord, as shown in Figure 11. The umbilical cord contains two arteries and one vein that connect the fetus to the placenta. When a baby is born, its umbilical cord is cut, but a few inches of it remain attached to the baby’s body. This portion of the cord is called the umbilicus. After a few days, the umbilicus dries up and drops off. The place where it was attached to the body is called a navel, or belly button.

Fetal Health

Everything that happens in a woman’s body has an effect on her developing fetus. Anything she does that could harm her health before or during her pregnancy could also harm her fetus. It is important for any woman who might become pregnant to take good care of her health. If she is in good health before she becomes pregnant, she has a better chance of having a healthy pregnancy and a healthy baby.

Prenatal Care

Health care designed to protect the health of a pregnant woman and prevent problems in her developing fetus is called prenatal care. Research has shown that a pregnant woman who receives prenatal care from a certified health care provider has a better chance of delivering a healthy baby. A pregnant woman’s prenatal care includes advice and information about nutrition, about viral infections, and about substances that could harm her fetus.

Nutrition

All the energy and nutrients a fetus needs for normal development must come from its mother. Vitamins, minerals, proteins, fats, and carbohydrates pass from mother to fetus through the placenta. To support her growing fetus, a pregnant woman needs to eat a healthy diet that includes dairy products, proteins, fruits, vegetables, and whole grains, such as those shown in Figure 11.

Calories

A pregnant woman is usually advised to add about 300 extra calories a day to her diet. The added calories supply the extra energy needed for the development of the fetus. However, it’s best to choose healthy foods and avoid high-calorie foods that contain large amounts of sugar or fat, but few other nutrients.

Folic Acid

A fetus’s spinal cord forms during the first weeks of pregnancy. Without a certain amount of folic acid, a form of vitamin B, spinal cord formation is abnormal. Doctors often recommend that pregnant women take vitamin supplements containing folic acid, in addition to eating a balanced diet.

Caffeine A pregnant woman should avoid caffeine, or consume it only in small amounts. Caffeine can increase a woman’s blood pressure and heart rate, which can be stressful to her fetus.

Environmental Factors

A pregnant woman can encounter substances in her environment, such as those in Figure 12, that present health risks for her fetus. She might inhale harmful substances, consume them with food or water, or absorb them through her skin.

These substances can then pass through the placenta and into the fetus. For example, a pregnant woman is usually advised to avoid using pesticides or insect repellents. Chemicals in insecticides and other pesticides can cause premature birth, birth defects, or miscarriage—the loss of an embryo during the first trimester.

Lead is a chemical element sometimes found in air pollution, old paint, and electronics. It can be harmful to anyone, but is especially harmful to a fetus, an infant, or a young child. Pregnant women who have been exposed to high levels of lead have a higher risk of miscarriage, premature delivery, and low birth-weight babies.

Viruses

Viruses can pass from a pregnant woman to her fetus through the placenta or during childbirth. Nearly everyone has had an infection caused by a virus. You’ve probably had colds, flu, chicken pox, or measles. Other viral illnesses include genital herpes and AIDS. Some viruses do not cause harm to adults, but they can be very harmful to a fetus or a newborn. For example, the viruses that cause chicken pox and genital herpes can cause birth defects or even death in newborns.

A virus that is deadly to both adults and newborns is the human immunodeficiency virus (HIV) that causes AIDS. AIDS attacks a person’s immune system, limiting the infected person’s ability to fight other infections. One out of every four pregnant women infected with HIV passes HIV to her fetus. An HIV-infected pregnant woman can lower the odds of having an HIV- infected baby if she sees a medical provider early, gets good medical care, and takes HIV-fighting medicines.

Drugs and Alcohol

A pregnant woman should always consult her medical provider before taking any over-the-counter medicine or prescription drug. A medicine that is safe for an adult might not be safe for a developing fetus.

Nicotine and Alcohol Nicotine, found in cigarettes, is a drug that has serious negative effects on a fetus. Smoking cigarettes during pregnancy can damage the placenta, and then it cannot deliver normal amounts of oxygen to a fetus. Also, the amount of nutrients passed to the fetus is reduced. A pregnant woman who smokes cigarettes runs a higher risk of having a premature baby.

Even second-hand smoke can cause health problems for a fetus. A pregnant woman who drinks alcohol excessively risks having a baby with fetal alcohol syndrome (FAS), like the children shown in Figure 13.

Illegal Drugs

A pregnant woman who uses alcohol or illegal drugs puts herself and her fetus in danger. Illegal drugs, including marijuana, cocaine, and heroin, enter the placenta and then pass into the body of the fetus. Use of these substances increases the chances for miscarriage, premature birth, and low birth weight. They also increase the chances that the child will have behavior problems and learning difficulties as it grows and develops.

Some drugs, such as cocaine and heroin, can cause the death of a fetus. A pregnant woman who shares needles to inject drugs increases her risk of being infected with viruses such as HIV.

**Textbook 4: Dynamics of Life Vol. 1**

Origins of Plants

What is a plant? A plant is a multicellular eukaryote. Most plants can produce their own food in the form of glucose through the process of photosynthesis. In addition, plant cells have thick cell walls made of cellulose. The stems and leaves of most plants have a waxy waterproof coating called a cuticle.

Fossils and other geological evidence suggest that a billion years ago, plants had not yet begun to appear on land. No ferns, mosses, trees, grasses, or wildflowers existed. The land was barren except for some algae at the edges of inland seas and oceans. However, the shallow waters that covered much of Earth’s surface at that time were teeming with bacteria, algae and other protists, as well as simple animals such as corals, sponges, jellyfish, and worms. Evidence indicates that green algae eventually became adapted to life on land.

Scientists hypothesize that all plants probably evolved from filamentous green algae that lived in the ancient oceans. Some of the evidence for their relationship can be found in modern members of both groups. Green algae and plants have cell walls that contain cellulose. Both groups have the same types of chlorophyll used in photosynthesis and store food in the form of starch. All other major groups of organisms store food in the form of glycogen and other complex sugars, and/or lipids.

The first evidence of plants in the fossil record began to appear over 440 million years ago. These early plants were simple in structure and did not have leaves. They were probably instrumental in turning bare rock into rich soil. The earliest known plant fossils are those of psilophytes, such as those shown in Figure 21.1.

Adaptations in Plants

Life on land has advantages as well as challenges. All organisms need water to survive. A filamentous green alga floating in a pond does not need to conserve water. The alga is completely immersed in a bath of water and dissolved nutrients, which it can absorb directly into its cells. For most land plants, the only available supply of water and minerals is in the soil, and only the portion of the plant that penetrates the soil can absorb these nutrients.

When you studied protists, you learned that algae reproduce by releasing unprotected unicellular gametes into the water, where fertilization and development take place. Land plants evolved structural and physiological adaptations that help protect the gametes from drying out. In some plants, the sperm are released near the egg so they only have to travel a short distance. Other plants have protective structures to ensure the survival of the gametes. Land plants must also withstand the forces of wind and weather and be able to grow against the force of gravity. Over the past 443 million years or so, plants have developed a huge variety of adaptations that reflect both the challenges and advantages of living on land.

Preventing water loss

If you run your fingers over the surface of an apple, a maple leaf, or the stem of a houseplant, you’ll probably find that it is smooth and slightly slippery. Most fruits, leaves, and stems are covered with a protective, waxy layer called the cuticle. Waxes and oils are lipids, which are biomolecules that do not dissolve in water. The waxy cuticle creates a barrier that helps prevent the water in the plant’s tissues from evaporating into the atmosphere.

Carrying out photosynthesis

The leaf, like the one in Figure 21.2, is a plant organ that grows from a stem and usually is where photosynthesis occurs. Leaves differ greatly in size and shape and they can vary on the same plant. Each plant division has unique leaves or leaflike structures.

Putting down roots

Most plants depend on the soil as their primary source for water and other nutrients. Plants can take in water and nutrients from the soil with their roots. In most plants, a root is a plant organ that absorbs water and minerals usually from the soil. Roots contain tissues that transport those nutrients to the stem. Roots anchor a plant usually in the ground. Some roots, such as those of radishes or sweet potatoes, accumulate starch and function as organs of storage. Many people use these storage roots as a food source. Find out more about the uses of plants on pages 1076–1079 in the Focus On.

In the MiniLab on this page, explore and evaluate some structural adaptations of plants that allow them to survive on land. Also, practice your lab skills by using a dissecting microscope.

Transporting materials

Water moves from the roots of a tree to its leaves, and the sugars produced in the leaves move to the roots through the stem. A stem is a plant organ that provides support for growth, as shown in Figure 21.3. It contains tissues for transporting food, water, and other materials from one part of the plant to another. Stems also can serve as organs for food storage. In green stems, some cells contain chlorophyll and can carry out photosynthesis.

The stems of most plants contain vascular tissues. Vascular tissues are made up of tubelike, elongated cells through which water, food, and other materials are transported. Plants that possess vascular tissues are known as vascular plants. Most of the plants you are familiar with, including pine and maple trees, ferns, rhododendrons, rye grasses, English ivy, and sunflowers, are vascular plants.

Mosses and several other small, less familiar plants called hornworts and liverworts are usually classified as non-vascular plants. Nonvascular plants do not have vascular tissues. The bodies of nonvascular plants are usually no more than a few cells thick, and water and nutrients travel from one cell to another by the processes of osmosis and diffusion.

The evolution of vascular tissues was an important structural adaptation for plants that allowed them to survive in the many habitats on land. Vascular plants can live farther away from water than nonvascular plants. Also, because vascular tissues include thickened cells called fibers that help support growth, vascular plants can grow much larger than nonvascular plants.

Reproductive strategies

Adaptations in some land plants include the evolution of seeds. A seed is a plant organ that contains an embryo, along with a food supply, and is covered by a protective coat, as shown in Figure 21.4. A seed protects the embryo from drying out and also can aid in its dispersal. Recall that a spore consists only of a haploid cell with a hard, outer wall. Land plants reproduce by either spores or seeds.

In non-seed plants, which include mosses and ferns, the sperm require a film of water on the gametophyte plant to reach the egg. In seed plants, which include all conifers and flowering plants, sperm reach the egg without using a film of water. This difference is one reason why non-seed plants require wetter habitats than most seed plants.

Alternation of generations

As in algae, the lives of all plants include two stages, or alternating generations, as shown in Figure 21.5. The gametophyte generation of a plant results in the development of gametes. All cells of the gametophyte, including the gametes, are haploid (n). The sporophyte generation begins with fertilization. All cells of the sporophyte are diploid (2n) and are produced by mitosis and cell division. The spores are produced in the sporophyte plant body by meiosis, and are therefore haploid (n).

In non-seed vascular plants such as ferns, spores have hard outer coverings. Spores are released into the environment where they can grow into haploid gametophyte plants. These plants produce male and female gametes. Following fertilization, the sporophyte plant develops and grows from the gametophyte plant.

In seed plants, such as conifers and flowering plants, spores develop inside the sporophyte and become the gametophytes. The gametophytes consist of only a few cells. Male and female gametes are produced by these gametophytes. After fertilization, a new sporophyte develops within a seed. The seed eventually is released and the new sporophyte plant grows.

Use the Problem-Solving Lab on this page to explore further the differences between the gametophyte and sporophyte generations of plants.

Survey of the Plant Kingdom

Phylogeny of Plant

Many geological and climate changes have taken place since the first plants became adapted to life on land. Landmasses have moved from place to place over Earth’s surface, climates have changed, and bodies of water have formed and disappeared. Hundreds of thousands of plant species evolved, and countless numbers of these became extinct as conditions continually changed. These processes of evolution and extinction continue to be affected by local and global changes. As plant species evolved in this changing landscape, they retained many of their old characteristics and also developed new ones. These processes of evolution and extinction continue today.

Some botanists use plant characteristics to classify plants into divisions. Recall that a plant division is similar to a phylum in other kingdoms. The highlights of plant evolution include origins of plants from green algae, the production of a waxy cuticle, the development of vascular tissue and roots, and the production of seeds. The production of seeds can be used as a basis to separate the divisions into two groups— non-seed plants and seed plants.

Non-seed Plants

The divisions of non-seed plants are shown in Figure 21.6. These plants produce hard-walled reproductive cells called spores. Non-seed plants include vascular and nonvascular organisms.

Hepaticophyta

Hepaticophytes include small plants commonly called liverworts. Their flattened bodies resemble the lobes of an animal’s liver. Liverworts are nonvascular plants that grow only in moist environments. Water and nutrients move throughout a liverwort by osmosis and diffusion. Studies comparing the biochemistry of different plant divisions suggest that liverworts may be the ancestors of all plants.

There are two kinds of liverworts: thallose liverworts and leafy liverworts. Thallose liverworts have a broad body that looks like a lobed leaf. Leafy liverworts are creeping plants with three rows of thin leaves attached to a stem.

Anthocerophyta

Anthocerophyte are also small thallose plants. The sporophytes of these plants, which resemble the horns of an animal, give the plants their common name—hornworts. These nonvascular plants grow in damp, shady habitats and rely on osmosis and diffusion to transport nutrients.

Bryophyta

Bryophytes, the mosses, are nonvascular plants that rely on osmosis and diffusion to transport materials. However, some mosses have elongated cells that conduct water and sugars. Moss plants are usually less than 5 cm tall and have leaflike structures that are usually only one to two cells thick. Their spores are formed in capsules.

Psilophyta

Psilophytes, known as whisk ferns, consist of thin, green stems. The psilophytes are unique vascular plants because they have neither roots nor leaves. Small scales that are flat, rigid, overlapping structures cover each stem. The two known genera of psilophytes are tropical or subtropical. Only one genus is found in the southern United States.

Lycophyta

Lycophytes, the club mosses, are vascular plants adapted primarily to moist environments. Lycophytes have stems, roots, and leaves. Their leaves, although very small, contain vascular tissue. Species existing today are usually less than 25 cm high, but their ancestors grew as tall as 30 m and formed a large part of the vegetation of Paleozoic forests. The plants of these ancient forests have become part of the coal that is now used by people for fuel.

Arthrophyta

Arthrophytes, the horsetails, are vascular plants. They have hollow, jointed stems surrounded by whorls of scalelike leaves. The cells covering the stems of some arthrophytes contain large deposits of silica. Although primarily a fossil group, about 15 species of arthrophytes exist today. All modern horse-tails are small, but their fossil relatives were the size of trees.

Pterophyta

Pterophytes, ferns, are the most well-known and diverse group of non-seed vascular plants. Ferns were abundant in Paleozoic and Mesozoic forests. They have leaves called fronds that vary in length from 1 cm to 500 cm. The large size and complexity of fronds is one difference between pterophytes and other groups of seedless vascular plants. Although ferns are found nearly everywhere, most grow in the tropics.

Seed Plants

Seed plants produce seeds, which in a dry environment are a more effective means of reproduction than spores. A seed consists of an embryonic plant and a food supply covered by a hard protective seed coat. All seed plants have vascular tissues. In Problem-Solving Lab 21.2, you can compare a characteristic common to seed plants and non-seed plants.

Cycadophyta

Cycads were abundant during the Mesozoic Era. Today, there are about 100 species of cycads. They are palmlike trees with scaly trunks and can be short or more than 20 m in height. Cycads produce male and female cones on separate trees. Cones are scaly structures that support male or female reproductive structures. Cycad cones can be as long as 1 m. Seeds are produced in female cones. Male cones produce clouds of pollen.

Gnetophyta

There are three genera of gnetophytes and each has distinct characteristics. Gnetum includes about 30 species of tropical trees and climbing vines. There are about 35 Ephedra species that grow as shrubby plants in desert and arid regions. Welwitschia has only one species, which is found in the deserts of southwest Africa. Its leaves grow from the base of a short stem that resembles a large, shallow cap.

Ginkgophyta

This division has only one living species, Ginkgo biloba, a distinctive tree with small, fan-shaped leaves. Like cycads, ginkgoes have male and female reproductive structures on separate trees. The seeds produced on female trees have an unpleasant smell, so ginkgoes planted in city parks are usually male trees. Ginkgoes are hardy and resistant to insects and to air pollution.

Coniferophyta

These are the conifers, cone-bearing trees such as pine, fir, cypress, and redwood. Conifers are vascular seed plants that produce seeds in cones. Species of conifers can be identified by the characteristics of their cones or leaves that are needle-like or scaly. You can learn more about how to identify conifers in the BioLab at the end of the chapter.

Bristlecone pines, the oldest known living trees in the world, are members of this plant division. Another type of conifer, the Pacific yew, is a source of cancer-fighting drugs. Read more about medicinal plants in the Connection to Health at the end of this chapter.

Anthophyta

Anthophytes, commonly called the flowering plants, are the largest, most diverse group of seed plants living on Earth. There are approximately 250 000 species of anthophytes. Fossils of the Anthophyta date to early in the **Cretaceous Period**. Unlike conifers, anthophytes produce flowers from which fruits develop, like those in Figure 21.7. A fruit usually contains one or more seeds. This division has two classes: the monocotyledons and dicotyledons. You will learn more about the distinctions between monocots and dicots when you read about anthophyte tissues in Chapter 23. Table 21.1 lists some information about the divisions of seed plants. Do you recognize any of the common names of the plants? Can you add to the list of common names?

The Diversity of Plants

Nonvascular Plants

What is a nonvascular plant?

Nonvascular plants are not as common or as widespread in their distribution as vascular plants because life functions, including photosynthesis and reproduction, require a close association with water. Because a steady supply of water is not available everywhere, nonvascular plants are limited to moist habitats by streams and rivers or in temperate and tropical rain forests. Recall that a lack of vascular tissue also limits the size of a plant. In drier soils, there is not enough water to meet the needs of most nonvascular plants. Their long-term survival in dry environments is limited by this resource—water. However, nonvascular plants, such as the moss in Figure 22.1, are successful in habitats with adequate water.

Alternation of generations

As in all plants, the life cycle of nonvascular plants includes an alternation of generations between a diploid sporophyte and a haploid gametophyte. However, nonvascular plant divisions include the only plants that have a dominant gametophyte generation. Sporophytes grow attached to and depend on gametophytes to take in water and other substances.

Non-photosynthetic sporophytes, like those shown in Figure 22.2A, depend on their gametophytes for food.

Gametophytes of nonvascular plants produce two kinds of sexual reproductive structures. The antheridium is the male reproductive structure in which sperm are produced. The archegonium is the female reproductive structure in which eggs are produced. Fertilization, which begins the sporophyte generation, occurs in the archegonium.

Adaptations in Bryophyta

There are several divisions of nonvascular plants. The first division you’ll study are the mosses, or bryophytes. Bryophytes are the most familiar of the nonvascular plant divisions. Mosses are small plants with leafy stems. The leaves of mosses are usually one cell thick. Mosses have rhizoids, colorless multicellular structures, which help anchor the stem to the soil. Although mosses do not contain true vascular tissue, some species do have a few, long water-conducting cells in their stems.

Mosses usually grow in dense carpets of hundreds of plants, as shown in Figure 22.2B. Some have upright stems; others have creeping stems that hang from steep banks or tree branches. Some mosses form extensive mats that help retard erosion on exposed rocky slopes.

Mosses grow in a wide variety of habitats, as stated in the Problem-Solving Lab on the opposite page. They even grow in the arctic during the brief growing season where sufficient moisture is present.

A well-known moss is Sphagnum, also known as peat moss. This plant thrives in acidic bogs in northern regions of the world. It is harvested for use as fuel and is a commonly used soil additive. Dried peat moss absorbs large amounts of water, so florists and gardeners use it to increase the water-holding ability of some soils.

Adaptations in Hepaticophyta

Another division of nonvascular plants is the liverworts, or hepaticophytes. Like mosses, liverworts are small plants that usually grow in clumps or masses in moist habitats. The name of the division is derived from the word hepar, which refers to the liver. The flattened body of a liverwort gametophyte is thought to resemble the shape of the lobes of an animal’s liver. Liverworts occur in many environments worldwide.

A liverwort can be categorized as either thallose or leafy, as shown in Figure 22.3. The body of a thallose liverwort is called a thallus. It is broad and ribbonlike and resembles a fleshy, lobed leaf. Thallose liverworts like Marchantia, shown in Figure 22.3A, are usually found growing on damp soil. Leafy liverworts grow close to the ground and usually are common in tropical jungles and areas with persistent fog. Their stems have flat, thin leaves arranged in three rows—a row along each side of the stem and a row of smaller leaves on the stem’s lower surface. Liverworts have rhizoids that are composed of only one elongated cell.

Adaptations in Anthocerophyta

Anthocerophytes are the smallest division of nonvascular plants, currently consisting of only about 100 species. Also known as hornworts, these nonvascular plants are similar to liverworts in several respects. Like some liverworts, hornworts have a thallose body. The sporophyte of a hornwort resembles the horn of an animal, as shown in Figure 22.4, which is why members of this division are commonly called “hornworts.” Another feature unique to hornworts is the presence of one to several chloroplasts in each cell of the sporophyte depending upon the species. Unlike other nonvascular plants, the hornwort sporophyte, not the gametophyte, produces most of the food used by both generations.

Origins of Nonvascular Plants

Fossil and genetic evidence suggests that liverworts were the first land plants. Fossils that have been positively identified as nonvascular plants first appear in rocks from the early Paleozoic Era, more than 440 million years ago. However, paleobotanists suspect that nonvascular plants were present earlier than current fossil evidence suggests. Both nonvascular and vascular plants probably share a common ancestor that had alternating sporophyte and gametophyte generations, cellulose in their cell walls, and chlorophyll for photosynthesis.

Non-Seed Vascular Plants

What is a non-seed vascular plant?

The obvious difference between a vascular and a nonvascular plant is the presence of vascular tissue. As you may remember, vascular tissue is made up of tubelike, elongated cells through which water and sugars are transported. Vascular plants are able to adapt to changes in the availability of water, and thus are found in a variety of habitats. You will learn about three divisions of non-seed vascular plants: Lycophyta, Arthrophyta, and Pterophyta.

Alternation of generations

Vascular plants, like all plants, exhibit an alternation of generations. Unlike nonvascular plants, the spore-producing vascular sporophyte is dominant and larger in size than the gametophyte, as shown in Figure 22.5. The mature sporophyte does not depend on the gametophyte for water or nutrients.

A major advance in this group of vascular plants was the adaptation of leaves to form structures that protect the developing reproductive cells. In some non-seed vascular plants, spore-bearing leaves form a compact cluster called a strobilus. The spores are released from the strobilus and can grow to form gametophytes. A fern gametophyte is called a prothallus. Gametophytes are relatively small and live in or on the soil. Antheridia and archegonia develop on the gametophyte, as illustrated in Figure 22.6. Sperm are released from antheridia and require a continuous film of water to reach eggs in the archegonia. If fertilization occurs, a zygote can grow into a large, dominant sporophyte.

Adaptations in Lycophyta

From fossil evidence it is known that tree-sized lycophytes were members of the early forest community. Modern lycophytes, like the one in Figure 22.7, are much smaller than their early ancestors. Lycophytes are commonly called club mosses and spike mosses. Their leafy stems resemble moss gametophytes, and their reproductive structures are club or spike shaped. However, unlike mosses, the sporophyte generation of the lycophytes is dominant. It has roots, stems, and small leaflike structures. A single vein of vascular tissue runs through each leaflike structure. The stems of lycophytes may be upright or creeping and have roots growing from the base of the stem.

The club moss, Lycopodium, is commonly called ground pine because it is evergreen and resembles a miniature pine tree. Some species of ground pine have been collected for decorative uses in such numbers that the plants have become endangered.

Adaptations in Arthrophyta

Arthrophytes, or horsetails, represent a second group of ancient vascular plants. Like the lycophytes, early horsetails were tree-sized members of the forest community. Today’s arthrophytes are much smaller than their ancestors, usually growing to about 1 m tall. There are only about 15 species in existence, all of the genus Equisetum.

The name horse-tail refers to the bushy appearance of some species. These plants also are called scouring rushes because they contain silica, an abrasive substance, and were once used to scour cooking utensils. If you run your finger along a horsetail stem, you can feel how rough it is.

Most horsetails, like the ones shown in Figure 22.8, are found in marshes, in shallow ponds, on stream banks, and other areas with damp soil. Some species are common in the drier soil of fields and roadsides. The stem structure of horsetails is unlike most other vascular plants; it is ribbed and hollow, and appears jointed. At each joint, there is a whorl of tiny, scalelike leaves. Like lycophytes, arthrophyte spores are produced in strobili that form at the tips of non-photosynthetic stems. After the spores are released, they can grow into gametophytes with antheridia and archegonia.

Adaptations in Pterophyta

According to fossil records, ferns— division Pterophyta—first appeared nearly 375 million years ago during the time when club mosses and horse-tails were the predominant members of Earth’s plant population. Ancient ferns grew tall and treelike and formed vast forests. Over time, ferns evolved into many species, adapted to different environments, and today are more abundant than club mosses or horsetails.

Ferns range in size from a few meters tall, like tree ferns, to small, floating plants that are only a few centimeters in diameter, such as those in Figure 22.9. You may have seen shrub-sized ferns on damp forest floors or along stream banks. Some ferns inhabit dry areas, becoming dormant when moisture is scarce and resuming growth and reproduction only when water is available again. Explore the relationship between water and non-seed vascular plants in the Problem-Solving Lab on this page.

Fern structures

As with most vascular plants, it is the sporophyte generation of the fern that has roots, stems, and leaves. The part of the fern plant that we most commonly recognize is the sporophyte generation. The gametophyte in most ferns is a thin, flat structure that is independent of the sporophyte. In most ferns, the main stem is underground. This thick, underground stem is called a rhizome. It contains many starch-filled cells for storage. The leaves of a fern are called fronds and grow upward from the rhizome, as shown in Figure 22.10. The fronds are often divided into leaflets called pinnae, which are attached to a central rachis. Ferns are the first of the vascular plants to have evolved leaves with branching veins of vascular tissue. The branched veins in ferns transport water and food to and from all the cells.

The fern life cycle is representative of other non-seed vascular plants. Fern spores are produced in structures called sporangia. Clusters of sporangia form a structure called a sorus. Sori are usually found on the undersides of fronds, as shown in Figure 22.11, but in some ferns, spores are borne on modified fronds.

Origins of Non-Seed Vascular Plants

The earliest evidence of non-seed vascular plants is found in fossils from early in the **Devonian Period**, around 375 million years ago. Large tree-sized lycophytes, arthrophytes, and pterophytes were extremely abundant in the warm, moist forests that dominated Earth during the **Carboniferous Period**. Ancient lycophyte species grew as tall as 30 m. Many of these species of non-seed vascular plants died out about 280 million years ago—a time when Earth’s climate was cooler and drier. Today’s non-seed nonvascular plants are much smaller and less widespread in their distribution than their prehistoric ancestors.

The evolution of vascular tissue enabled these plants to live on land and to maintain larger body sizes in comparison with nonvascular plants. As you can infer from Figure 22.12, non-seed vascular plants are closely related to nonvascular plants.

Seed Plants

What is a seed plant?

Some vascular plants produce seeds in which reduced sporophyte plants are enclosed within a protective coat. The seeds may be surrounded by a fruit or carried on the scales of a cone.

Seed plants produce spores

In seed plants, as in all other plants, spores are produced by the sporophyte generation. These spores develop into the male and female gametophytes. The male gametophyte develops inside a structure called a pollen grain that includes sperm cells, nutrients, and a protective outer covering. The female gametophyte, which produces the egg cell, is contained within a sporophyte structure called an ovule.

Fertilization and reproduction

The union of the sperm and egg, called fertilization, forms the sporophyte zygote. In most seed plants, this process does not require a continuous film of water as required by nonvascular and non-seed vascular plants. Remember that in non-seed plants, the sperm must swim through a continuous film of water in order to reach eggs in the archegonia of a gametophyte. Because they do not require a continuous film of water for fertilization, seed plants are able to grow and reproduce in a wide variety of habitats that have limited water availability.

After fertilization, the zygote develops into an embryo. An embryo is an early stage of development of an organism. In plants, an embryo is the young diploid sporophyte stage of the plant. Embryos of seed plants include one or more cotyledons. Cotyledons usually store or absorb food for the developing embryo. In conifers and many flowering plants, cotyledons are the leaflike structures on the plant’s stem when the plant emerges from the soil.

Advantages of seeds

A seed consists of an embryo and its food supply enclosed in a tough, protective coat, as shown in Figure 22.13. Seed plants have several important advantages over non-seed plants. The seed contains a supply of food to nourish the young plant during the early stages of growth. This food is used by the plant until its leaves are developed enough to carry out photosynthesis. In conifers and some flowering plants, the embryo’s food supply is stored in the cotyledons. The embryo is protected during harsh conditions by a tough seed coat. The seeds of many species are also adapted for easy dispersal to new areas. Then the new plants do not have to compete with their parent plant for sunlight, water, soil nutrients, and living space. You can learn more about seed structure in MiniLab 22.2.

Diversity of seed plants

In some plants, seeds develop on the scales of woody strobili called cones. This group of plants is sometimes referred to as gymnosperms. The term gymnosperm means “naked seed” and is used with these plants because their seeds are not protected by a fruit. The gymnosperm plant divisions you will learn about are Cycadophyta, Ginkgophyta, Gnetophyta, and Coniferophyta.

Flowering plants, also called angiosperms, produce seeds enclosed within a fruit. A fruit includes the ripened ovary of a flower. The fruit provides protection for seeds and aids in seed dispersal. The Anthophyta division contains all species of flowering plants.

Adaptations in Cycadophyta

About 100 species of cycads exist today, exclusively in the tropics and subtropics. The only present-day species that grows wild in the United States is found in Florida, although you may see cycads cultivated in greenhouses or botanical gardens.

Cycads have male and female reproductive systems on separate plants, as shown in Figure 22.14. The male system includes cones that produce pollen grains, which produce motile sperm. Cycads are one of the few seed plants that produce motile sperm. The female system includes cones that produce ovules. The trunks and leaves of many cycads resemble those of palm trees, but cycads and palms are not closely related because palms are anthophytes.

Adaptations in Ginkgophyta

Today, this division is represented by only one living species, Ginkgo biloba. All ginkgoes are cultivated trees, and they are not known to exist in the wild. Like cycads, ginkgo male and female reproductive systems are on separate plants. The male ginkgo produces pollen grains in strobiluslike cones that grow from the bases of leaf clusters. Also like cycads, ginkgo pollen grains produce motile sperm. The female ginkgo produces ovules which, when fertilized, develop fleshy, apricot-colored seed coats, as shown in Figure 22.15. These soft seed coats give off a foul odor when broken or crushed. Ginkgoes often are planted in urban areas because they tolerate smog and pollution. Gardeners and landscapers usually only plant male gingkoes because they do not produce seeds with soft seed coats. Do the BioLab at the end of this chapter to explore what other trees are planted in urban areas.

Adaptations in Gnetophyta

Most living gnetophytes can be found in the deserts or mountains of Asia, Africa, North America, and Central and South America. The division Gnetophyta contains only three genera, which have different structural adaptations to their environments. The genus Gnetum is composed of tropical climbing plants. The genus Ephedra contains shrublike plants and is the only gnetophyte genus found in the United States. The third genus, Welwitschia, is a bizarre-looking plant found only in South Africa. It grows close to the ground, has a large tuberous root, and may live 1000 years. Ephedra and Welwitschia are pictured in Figure 22.16.

Adaptations in Coniferophyta

The sugar pine is one of many familiar forest trees that belong to the division Coniferophyta. The conifers are trees and shrubs with needlelike or scalelike leaves. They are abundant in forests throughout the world, and include pine, fir, spruce, juniper, cedar, redwood, yew, and larch.

The reproductive structures of most conifers are produced in cones. Most conifers have male and female cones on different branches of the same tree. The male cones produce pollen. They are small and easy to overlook. Female cones are much larger. They stay on the tree until the seeds have matured. Examples of both types of cones are shown in Figure 22.17.

Evergreen conifers

Most conifers, like those pictured in Figure 22.18, are evergreen plants— plants that retain some of their leaves for more than one year. Although individual leaves drop off as they age or are damaged, the plant never loses all of its leaves at one time.

Plants that retain some of their leaves year-round can photosynthesize whenever favorable environmental conditions exist. This is an advantage in environments where the growing season is short. Another advantage of leaf retention is that a plant’s food reserves are not depleted each spring to produce a whole set of new leaves.

Evergreen leaves usually have a heavy coating of cutin, a water-insoluble, waxy material that helps reduce water loss. For conifers, leaf shape—needlelike or scalelike—also helps reduce water loss. To learn more about conifer needles, see Figure 22.20 on the next page.

Deciduous trees lose their leaves

A few conifers, including larches and bald cypress trees, are deciduous, Figure 22.19. Deciduous plants drop all their leaves each fall or when water is scarce or unavailable as in the tundra or in deserts. Plants lose most of their water through the leaves; very little is lost through bark or roots. Dropping all leaves is an adaptation for reducing water loss. However, a tree with no leaves cannot photosynthesize and must remain dormant during this time.

Adaptations in Anthophyta

Flowering plants are classified in the division Anthophyta. They are the most well-known plants on Earth with more than 250 000 identified species. See if you are familiar with some of the plants in Figure 22.21. Like other seed plants, anthophytes have roots, stems, and leaves. But unlike the other seed plants, anthophytes produce flowers and form seeds enclosed in a fruit. Many different species of flowering plants inhabit tropical forests. As you will discover in Biology and Society at the end of this chapter, different groups of people have different viewpoints on preserving this rich habitat.

Fruit production

Anthophyta is unique among plant divisions. It is the only division in which plants have flowers and produce fruits. A fruit develops from a flower’s female reproductive structure(s). Sometimes, other flower parts become part of the fruit and, as in pineapples, the fruit develops from more than one flower. A fruit usually contains one or more seeds. One of the advantages of fruit-enclosed seeds is the added protection the fruit provides for the young embryo.

Fruits often aid in the dispersal of seeds. Animals may eat them or carry them off to store for food. Seeds of some species that are eaten pass through the animal’s digestive tract unharmed and are distributed as the animal wanders. In fact, some seeds must pass through a digestive tract before they can begin to grow a new plant. Some fruits have structural adaptations that help disperse the seed by wind or water. Some examples of fruits are illustrated in Figure 22.22.

Monocots and dicots

The division Anthophyta is divided into two classes: monocotyledons and dicotyledons. The two classes are named for the number of cotyledons in the seed. Monocotyledons have one seed leaf; dicotyledons have two seed leaves. These two classes often are called monocots and dicots. Table 22.1 compares the characteristics of monocots and dicots. About 65 000 species of monocots have been identified and include grasses, orchids, lilies, and palms. Identified dicot species number about 185 000. They include nearly all of the familiar shrubs and trees (except conifers), cacti, wild- flowers, garden flowers, vegetables, and herbs.

Life spans of anthophytes

Why do some plants live longer than people, and others live only a few weeks? The life span of a plant is genetically determined and reflects strategies for surviving periods of harsh conditions.

Annual plants live for only a year or less. They sprout from seeds, grow, reproduce, and die in a single growing season. Most annuals are herbaceous, which means their stems are green and do not contain woody tissue. Many food plants such as corn, wheat, peas, beans, and squash are annuals, as are many weeds of the temperate garden. Annuals form drought-resistant seeds that can survive the winter.

Biennial plants have life spans that last two years. Many biennials develop large storage roots, such as carrots, beets, and turnips. During the first year, biennials grow many leaves and develop a strong root system. Over the winter, the aboveground portion of the plant dies back, but the roots remain alive. Underground roots are able to survive conditions that leaves and stems cannot endure. During the second spring, food stored in the root is used to produce new shoots that produce flowers and seeds.

Perennials live for several years, producing flowers and seeds periodically—usually once each year. Some survive harsh conditions by dropping their leaves or dying back to soil level, while their woody stems or underground storage organs remain intact and dormant. Examples of plants with different lifespans are shown in Figure 22.23.

Origins of Seed Plants

Seed plants first appeared about 360 million years ago during the Paleozoic Era. Some seed plants, such as ancient relatives of cycads and ginkgoes, shared Earth’s forest with the dinosaurs during the Mesozoic Era. However, about 65 million years ago, most members of the Ginkgophyta died out along with many organisms during a mass extinction.

According to fossil evidence, the first conifers emerged around 250 million years ago. During the **Jurassic Period**, conifers became predominant forest inhabitants and remain so today. Anthophytes first appeared about 140 million years ago late in the **Jurassic Period of the Mesozoic Era**.

Plant Structure and Function

Plant Cells and Tissues

Types of Plant Cells

Like all organisms, plants are composed of cells. Plant cells are different from animal cells because they have a cell wall, a central vacuole, and can contain chloroplasts. Figure 23.1 shows a typical plant cell. Plants, just like other organisms, are composed of different cell types.

Parenchyma

Parenchyma cells are the most abundant kind of plant cell. They are found throughout the tissues of a plant. These spherical cells have thin, flexible cell walls. Most parenchyma cells usually have a large central vacuole, which sometimes contains a fluid called sap.

Parenchyma cells, as shown in Figure 23.2A, have two main functions: storage and food production. The large vacuole found in these cells can be filled with water, starch grains, or oils. The edible portions of many fruits and vegetables are composed mostly of parenchyma cells. Parenchyma cells also can contain numerous chloroplasts that produce glucose during photosynthesis.

Collenchyma

Collenchyma cells are long cells with unevenly thickened cell walls, as illustrated in Figure 23.2B. The structure of the cell wall is important because it allows the cells to grow. The walls of collenchyma cells can stretch as the cells grow while providing strength and support. These cells are arranged in tubelike strands or cylinders that provide support for surrounding tissue. The long tough strands you may have noticed in celery are composed of collenchyma.

Sclerenchyma

The walls of sclerenchyma cells are very thick and rigid. At maturity, these cells often die. Although their cytoplasm disintegrates, their strong, thick cell walls remain and provide support for the plant. Sclerenchyma cells can be seen in Figure 23.2C. Two types of sclerenchyma cells commonly found in plants are fibers and sclerids. Fibers are long, thin cells that form strands. They provide support and strength for the plant and are the source of fibers used for making linen and rope. A type of fiber is associated with vascular tissue, which you will learn about later in this section. Sclerids are irregularly shaped and usually found in clusters. They are the gritty texture of pears and a major component of the pits found in peaches and other fruits.

Plant Tissues

Recall that a tissue is a group of cells that function together to perform an activity. Tissues can be referred to as plant subsystems. There are several different tissue types in plants.

Dermal tissues

The dermal tissue, or epidermis, is composed of flattened cells that cover all parts of the plant. It functions much like the skin of an animal, covering and protecting the body of a plant. As shown in Figure 23.3, the cells that make up the epidermis are tightly packed and often fit together like a jigsaw puzzle. The epidermal cells produce the waxy cuticle that helps prevent water loss.

Another structure that helps control water loss from the plant, a stoma, is part of the epidermal layer. Stomata are openings in leaf tissue that control the exchange of gases. Stomata are found on green stems and on the surfaces of leaves. In many plants, fewer stomata are located on the upper surface of the leaf as a means of conserving water. Cells called guard cells control the opening and closing of stomata. The opening and closing of stomata regulates the flow of water vapor from leaf tissues. You can learn more about stomata in the BioLab at the end of this chapter.

The dermal tissue of roots may have root hairs. Root hairs are extensions of individual cells that help the root absorb water and dissolved minerals. On the stems and leaves of some plants, there are structures called trichomes. Trichomes are hairlike projections that give a stem or a leaf a “fuzzy” appearance. They help reduce the evaporation of water from the plant. In some cases, trichomes are glandular and secrete toxic substances that help protect the plant from predators. Stomata, root hairs, and trichomes are shown in Figure 23.4.

Vascular tissues

Food, dissolved minerals, and water are transported throughout the plant by vascular tissue. Xylem and phloem are the two types of vascular tissues. Xylem is plant tissue composed of tubular cells that transports water and dissolved minerals from the roots to the rest of the plant. In seed plants, xylem is composed of four types of cells—tracheids, vessel elements, fibers, and parenchyma.

Tracheids are tubular cells tapered at each end. The cell walls between adjoining tracheids have pits through which water and dissolved minerals flow.

Vessel elements are tubular cells that transport water throughout the plant. They are wider and shorter than tracheids and have openings in their end walls, as shown in Figure 23.5. In some plants, mature vessel elements lose their end walls and water and dissolved minerals flow freely from one cell to another.

Although almost all vascular plants have tracheids, vessel elements are most commonly found in anthophytes. Conifers have tracheids but no vessel elements in their vascular tissues. This difference in vascular tissues could be one reason why anthophytes are the most successful plants on Earth. Anthophyte vessel elements are thought to transport water more efficiently than tracheids because water can flow freely from vessel element to vessel element through the openings in their end walls.

You can learn more about vascular tissues in the MiniLab on this page. What other types of tissues are found in vascular plants? To answer this question, look at Figure 23.6 on the next page.

Sugars and other organic compounds are transported throughout a vascular plant within the phloem.

Phloem is made up of tubular cells joined end to end, as shown in Figure 23.7. It is similar to xylem because phloem also has long cylindrical cells. However these cells, called sieve tube members, are alive at maturity. Sieve tube members are unusual because they contain cytoplasm but do not have a nucleus or ribosomes. Next to each sieve tube member is a companion cell. Companion cells are nucleated cells that help with the transport of sugars and other organic compounds through the sieve tubes of the phloem. In anthophytes, the end walls between two sieve tube members are called sieve plates. The sieve plates have large pores that allow sugar and organic compounds to move from sieve tube member to sieve tube member. Phloem can transport materials from the roots to the leaves also.

The vascular phloem tissue of many plants contains fibers. Although the fibers are not used for transporting materials, they are important because they provide support for the plant.

Ground tissue is composed mostly of parenchyma cells but it may also include collenchyma and sclerenchyma cells. It is found throughout a plant and often is associated with other tissues. The functions of ground tissue include photosynthesis, storage, and support. The cells of ground tissue in leaves and some stems contain numerous chloroplasts that carry on photosynthesis. Ground tissue cells in some stems and roots contain large vacuoles that store starch grains and water. Cells, such as those shown in Figure 23.8, are often seen in ground tissue.

Meristematic tissues

A growing plant produces new cells in areas called meristems. Meristems are regions of actively dividing cells. Meristematic cells are differently shaped parenchyma cells with large nuclei. There are several types of meristems; two types are shown in Figure 23.6 on page 609.

Apical meristems are found at or near the tips of roots and stems. They produce cells that allow the roots and stems to increase in length. Lateral meristems are cylinders of dividing cells located in roots and stems. The production of cells by the lateral meristems results in an increase in root and stem diameters. Most woody plants have two kinds of lateral meristems—vascular cambium and cork cambium. The vascular cambium produces new xylem and phloem cells in the stems and roots. The cork cambium produces cells with tough cell walls. These cells cover the surface of stems and roots. The outer bark of a tree is produced by the cork cambium.

A third type of lateral meristem is found in grasses, corn, and other monocots. This meristem adds cells that lengthen the part of the stem between the leaves. These plants do not have a vascular or a cork cambium.

Roots, Stems, and Leaves

Roots

Roots are plant organs that anchor a plant, usually absorb water and dissolved minerals, and contain vascular tissues that transport materials to and from the stem. As shown in Figure 23.9, roots may be short or long, and thick and massive or thin and threadlike. The surface area of a plant’s roots can be as much as 50 times greater than the surface area of its leaves. Most roots grow in soil but some do not.

The type of root system is genetically determined but can vary because of environmental factors such as soil type, moisture, and temperature. There are two main types of root systems—taproots and fibrous roots. Carrots and beets are taproots, which are single, thick structures with smaller branching roots. Taproots accumulate and store food. Fibrous roots systems have many, small branching roots that grow from a central point.

Some plants, such as the corn in Figure 23.10, have a type of root called prop roots, which originate above ground and help support a plant. Many climbing plants have aerial roots that cling to objects such as walls and provide support for climbing stems. When bald cypress trees grow in swampy soils, they produce modified roots called pneumatophores, which are referred to as “knees.” The knees grow upward from the mud, and eventually, out of the water. Knees help supply oxygen to the roots.

The structure of roots

If you look at the diagram of a root in Figure 23.11, you can see that a root hair is a tiny extension of an epidermal cell. Root hairs increase the surface area of a root that contacts the soil. They absorb water, oxygen, and dissolved minerals. The next layer is a part of the ground tissue called the cortex, which is involved in the transport of water and dissolved minerals into the vascular tissues. The cortex is made up of parenchyma cells that sometimes store food and water.

At the inner limit of the cortex lies the endodermis, a layer of cells with waterproof cell walls that form a seal around the root’s vascular tissues. The waterproof seal of the endodermis forces water and dissolved minerals that enter the root to pass through the cells of the endodermis. Thus, the endodermis controls the flow of water and dissolved minerals into the root. Next to the endodermis is the pericycle. It is the tissue from which lateral roots arise as offshoots of older roots.

Xylem and phloem are located in the center of the root. The arrangement of xylem and phloem tissues, as shown in Figure 23.12, accounts for one of the major differences between monocots and dicots. In dicot roots, the xylem forms a central star-shaped mass with phloem cells between the rays of the star. Monocot roots usually have strands of xylem that alternate with strands of phloem. There is sometimes a central core of parenchyma cells in the monocot root called a pith.

Root growth

There are two areas of rapidly dividing cells in roots where the production of new cells initiates growth. The root apical meristem produces cells that cause a root to increase in length. As these cells begin to mature, they differentiate into different types of cells.

In dicots, the vascular cambium develops between the xylem and phloem and contributes to a root’s growth by adding cells that increase its diameter. Each layer of new cells produced by the root apical meristem is left farther behind as new cells are added and the root grows forward through the soil. The tip of each root is covered by a protective layer of parenchyma cells called the root cap. As the root grows through the soil, the cells of the root cap wear away. Replacement cells are produced by the root apical meristem so the root tip is never without its protective covering. Examine Figure 23.13 on the previous page to see if you can locate all the structures of a root.

Stems

Stems usually are the aboveground parts of plants that support leaves and flowers. They have vascular tissues that transport water, dissolved minerals, and sugars to and from roots and leaves. Their form ranges from the thin, herbaceous stems of basil plants to the massive, woody trunks of trees. Green, herbaceous stems are soft and flexible and usually carry out some photosynthesis. Petunias, impatiens, and carnations are other examples of plants with herbaceous stems. Trees, shrubs, and some other perennials have woody stems. Woody stems are hard and rigid and have cork and vascular cambriums.

Some stems are adapted to storing food. This can enable the plant to survive drought or cold, or grow from year to year. Stems that act as food-storage organs include corms, tubers, and rhizomes. A corm is a short, thickened, underground stem surrounded by leaf scales. A tuber is a swollen, underground stem that has buds from which new plants can grow. Rhizomes also are underground stems that store food. Some examples of these food-storing stems are shown in Figure 23.14.

Internal structure

Both stems and roots have vascular tissues. However, the vascular tissues in stems are arranged differently from that of roots. Stems have a bundled arrangement or circular arrangement of vascular tissues within a surrounding mass of parenchyma tissue. As you can see in Figure 23.15A and B, monocots and dicots differ in the arrangement of vascular tissues in their stems. In most dicots, xylem and phloem are in a circle of vascular bundles that form a ring in the cortex. The vascular bundles of most monocots are scattered throughout the stem.

Woody stems

Many conifers and perennial dicots produce thick, sturdy stems, as shown in Figure 23.15C, that may last several years, or even decades. As the stems of woody plants grow in height, they also grow in thickness. This added thickness, called secondary growth, results from cell divisions in the vascular cambium of the stem. The xylem tissue produced by secondary growth is also called wood. In temperate regions, a tree’s annual growth rings are the layers of vascular tissue produced each year by secondary growth. These annual growth rings can be used to estimate the age of the plant. The vascular tissues often contain sclerenchyma fibers that provide support for the growing plant.

As secondary growth continues, the outer portion of a woody stem develops bark. Bark is composed of phloem cells and the cork cambium. Bark is a tough, corky tissue that protects the stem from damage by burrowing insects and browsing herbivores.

Stems transport materials

Water, sugars, and other compounds are transported within the stem. Xylem transports water and dissolved minerals from the roots to the leaves. Water that is lost through the leaves is continually replaced by water moving in the xylem. Water forms an unbroken column within the xylem. As water moves up through the xylem, it also carries dissolved minerals to all living plant cells.

The contents of phloem are primarily dissolved sugars but phloem also can transport hormones, viruses, and other substances. The sugars originate in photosynthetic tissues that are usually in leaves. Any portion of the plant that stores these sugars is called a sink, such as the parenchyma cells that make up the cortex in the root. The movement of sugars in the phloem is called translocation. Figure 23.16 shows the movement of materials in the vascular tissues of a plant.

Growth of the stem

Primary growth in a stem is similar to primary growth in a root. This increase in length is due to the production of cells by the apical meristem, which lies at the tip of a stem. As mentioned earlier, secondary growth or an increase in diameter is the result of cell divisions in the vascular cambium or lateral meristem. Meristems located at intervals along the stem, called nodes, give rise to leaves and branches.

Leaves

The primary function of the leaves is photosynthesis. Most leaves have a relatively large surface area that receives sunlight. Sunlight passes through the transparent cuticle and epidermis into the photosynthetic tissues just beneath the leaf surface.

Leaf variation

When you think of a leaf, you probably think only of a flat, broad, green structure. This part of the leaf is called the leaf blade. Sizes, shapes, and types of leaves vary enormously. The giant Victoria water lily that grows in some of the rivers of Guyana has floating, circular leaves that can be more than two meters in diameter.

The leaves of duckweed, a common floating plant of ponds and lakes, are measured in millimeters. Some plant species commonly produce different forms of leaves on one plant.

Some leaves, such as grass blades, are joined directly to the stem. In other leaves, a stalk joins the leaf blade to the stem. This stalk, which is part of the leaf, is called the petiole. The petiole contains vascular tissues that extend from the stem into the leaf and form veins. If you look closely, you will notice these veins as lines or ridges running along the leaf blade.

Leaves vary in their shape and arrangement on the stem. A simple leaf is one with a blade that is not divided. When the blade is divided into leaflets, it is called a compound leaf. Figure 23.17 gives some examples of the variety of leaf shapes.

The arrangement of leaves on a stem can vary. Leaves can grow from opposite sides of the stem in an alternating arrangement. If two leaves grow opposite each other on a stem, the arrangement is called opposite. Three or more leaves growing around a stem at the same position is called a whorled arrangement.

Leaf structure

The internal structure of a typical leaf is shown in Figure 23.18. The vascular tissues are located in the midrib and veins of the leaf. Just beneath the epidermal layer are two layers of mesophyll. Mesophyll is the photosynthetic tissue of a leaf. It is usually made up of two types of parenchyma cells—palisade mesophyll and spongy mesophyll. The palisade mesophyll is made up of column-shaped cells containing many chloroplasts. These cells are found just under the upper epidermis and receive maximum exposure to sunlight. Most photosynthesis takes place in the palisade mesophyll. Below the palisade mesophyll is the spongy mesophyll, which is composed of loosely packed, irregularly shaped cells. These cells usually are surrounded by many air spaces that allow carbon dioxide, oxygen, and water vapor to freely flow around the cells. Gases can also move in and out of a leaf through the stomata, which are located in the upper and/or lower epidermis.

Transpiration

You read previously that leaves have an epidermis with a waxy cuticle and stomata that help reduce water loss. Guard cells are cells that surround and control the size of a stoma, as shown in Figure 23.19. The loss of water through the stomata is called transpiration. Learn more about how a plant’s surroundings may influence rate of transpiration in Problem-Solving Lab 23.2 on this page.

Plant Responses

Plant Hormones

Plants, like animals, have hormones that regulate growth and development. A hormone is a chemical that is produced in one part of an organism and transported to another part, where it causes a physiological change. Only a small amount of the hormone is needed to make this change.

Auxins cause stem elongation

The group of plant hormones called auxins promote cell elongation.

Indoleacetic acid (IAA)—a naturally occurring auxin—is produced in apical meristems of plant stems. IAA weakens the connections between the cellulose fibers in the cell wall, which allows a cell to stretch and grow longer. The combination of new cells from the apical meristem and increasing cell lengths leads to stem growth. Auxin is not transported in the vascular system. It moves from one parenchyma cell to the next by active transport.

Auxins have other effects on plant growth and development. Auxin produced in the apical meristem inhibits the growth of side branches. Removing the stem tip reduces the amount of auxin present and allows the development of branches, as shown in Figure 23.22.

Auxin also delays fruit formation and inhibits the dropping of fruit from the plant. When auxin concentrations decrease, the ripened fruits of some trees fall to the ground and deciduous trees begin to shed their leaves.

Gibberellins promote growth

The group of plant growth hormones called gibberellins cause plants to grow taller because, like auxins, they stimulate cell elongation. Unlike auxins, gibberellins are transported in vascular tissue. Many dwarf plants, such as those in Figure 23.23, are short because the plant does not produce gibberellins or its cells are not receptive to the hormone. If gibberellins are applied to the tip of a dwarf plant, it will grow taller. Gibberellins also increase the rate of seed germination and bud development. Farmers have learned to use gibberellins to enhance fruit formation. Florists often use gibberellins to induce flower buds to open.

Cytokinins stimulate cell division

The hormones called cytokinins stimulate mitosis and cell division. Cytokinins stimulate the production of proteins needed for mitosis and cell division. Most cytokinins are produced in root meristems. This hormone travels up the xylem to other parts of the plant. The effects of cytokinins are often enhanced by the presence of other hormones.

Ethylene gas promotes ripening

The plant hormone ethylene is a simple, gaseous compound composed of carbon and hydrogen. It is produced primarily by fruits, but also by leaves and stems. Ethylene is released during a specific stage of fruit ripening. It causes cell walls to weaken and become soft. Ethylene speeds the ripening of fruits and promotes the breakdown of complex carbohydrates to simple sugars. If you have ever enjoyed a ripe red apple you know that it tastes sweeter than an immature fruit.

Many farmers use ethylene to ripen green fruits or vegetables after they have been picked, as shown in Figure 23.24.

Plant Responses

Why do roots grow down and most stems grow up? Although a plant lacks a nervous system and usually cannot make quick responses to stimuli, it does have mechanisms that enable it to respond to its environment. Plants grow, reproduce, and reposition their roots, stems, and leaves in response to environmental conditions, such as gravity, light, temperature, and amount of darkness.

Tropic responses in plants

At the beginning of this section, you read that the flower heads of sunflowers slowly respond to the sun’s apparent movement across the sky. Tropism is a plant’s response to an external stimulus. The tropism is called positive if the plant grows toward the stimulus. The tropism is called negative if the plant grows away from the stimulus.

There is another tropism associated with the upward growth of stems and the downward growth of roots. Gravitropism is plant growth in response to gravity. Gravitropic responses are beneficial to plants. Roots that grow down into the soil are able to anchor the plant and can take in water and dissolved minerals. Stems usually exhibit a negative gravitropism.

Some plants exhibit another tropism called thigmotropism, which is a growth response to touch. The tendrils of the vine in Figure 23.25B have coiled around a fence after making contact during early growth.

Because tropisms involve growth, they are not reversible. The position of a stem that has grown several inches in a particular direction cannot be changed. But, if the direction of the stimulus is changed, the stem will begin growing in another direction.

Nastic responses in plants

A responsive movement of a plant that is not dependent on the direction of the stimulus is called a nastic movement. An example of a nastic movement is the movement of Mimosa pudica leaflets when they are touched, as shown in Figure 23.26A. This is caused by a change in water pressure in the cells at the base of each leaflet. A dramatic drop in pressure causes the cells to become limp and the leaflets to change orientation. Another example of a nastic response is the sudden closing of the hinged leaf of a Venus’s-flytrap, Figure 23.26B. If an insect triggers sensitive hairs on the inside of the leaf, the leaf snaps shut. Nastic responses that are due to changes in cellular water pressure are reversible because they do not involve growth. The Mimosa pudica and Venus’s- flytrap leaves return to their original positions once the stimulus ends.

Reproduction in Plants

Life Cycles of Mosses, Ferns, and Conifers

Alternation of Generations

As you learned earlier, most plant life cycles include an alternation of generations. As shown in Figure 24.1, an alternation of generations consists of a sporophyte stage and a gametophyte stage.

All cells of a sporophyte are diploid. Certain cells of a sporophyte undergo meiosis, which produces haploid spores. These spores undergo cell divisions and form a multicellular, haploid gametophyte. Some cells of a gametophyte differentiate and form haploid gametes. The female gamete is an egg and the male gamete is a sperm. When a sperm fertilizes an egg, a diploid zygote forms. This is sexual reproduction. The zygote can undergo cell divisions and form an embryo sporophyte. If the embryo develops to maturity, the cycle can begin again.

This basic life cycle pattern is the same for most plants. However, there are many variations on this pattern within the plant kingdom. For instance, recall that in mosses the gametophyte is the familiar form, not the sporophyte.

In others, such as flowering plants, the gametophyte is microscopic. Most people have never even seen the female gametophyte of a flowering plant. Botanists usually refer to the bigger, more obvious plant as the dominant generation. The dominant generation lives longer and can survive independently of the other generation. In most plant species the sporophyte is the dominant plant.

Asexual reproduction

Most plants also can reproduce asexually by a process called vegetative reproduction. In this type of reproduction, new plants are produced from existing plant organs or parts of organs. The new plants have the same genetic makeup as the original plant. For example, some thallose liverwort gametophytes can produce cuplike structures, as shown in Figure 24.2. Inside, minute pieces of tissue called gemmae develop. If gemmae fall from the cup, they can grow into other liverwort gametophytes that are genetically identical to the thallus. You can learn more about asexual reproduction in MiniLab 24.1.

Life Cycle of Mosses

The gametophyte stage is the dominant generation in mosses. A haploid moss spore can germinate and grow to form a protonema. It is a small green filament of cells that can develop into the gametophyte. In some mosses, male and female reproductive structures form on separate gametophytes, but in others, male and female reproductive structures are on the same gametophyte. Recall that the egg-producing, female reproductive structure is an archegonium, and the sperm-producing, male reproductive structure is an antheridium.

Motile sperm from an antheridium swim in a continuous film of water to an egg in an archegonium. If fertilization occurs, a diploid zygote forms. The zygote undergoes cell divisions forming the sporophyte that consists of a stalk with a capsule at the top. The sporophyte remains attached to and dependent on the gametophyte. Cells in the capsule undergo meiosis, producing haploid spores. When the capsule matures, it bursts open and releases spores. If the spores land in a favorable environment, they can germinate and the cycle repeats, as shown in Figure 24.3.

Some moss gametophytes also reproduce by vegetative reproduction. They can break into pieces when dry and brittle then, when moisture returns, each piece can grow and form a protonema then a gametophyte.

Life Cycle of Ferns

Unlike mosses, the dominant stage of the fern life cycle is the sporophyte stage. The fern sporophyte includes the familiar fronds you see in Figure 24.4A. Fern fronds grow from a rhizome, which is an underground stem. On the underside of some fronds are sori, which are clusters of sporangia. Meiosis occurs within the sporangia, producing haploid spores. When environmental conditions are right, the sporangia open and release haploid spores, as shown in Figure 24.4B.

A spore can germinate to form a heart-shaped gametophyte called a prothallus, as shown in Figure 24.4C. The prothallus produces both archegonia and antheridia on its surface. The flagellated sperm released by antheridia swim through a film of water to eggs in archegonia. If fertilization occurs, the diploid zygote can develop into the sporophyte. Initially, this developing sporophyte depends upon the gametophyte for its nutrition. However, once the sporophyte produces green fronds, it can carry on photosynthesis and survive on its own. The prothallus dies and decomposes as the sporophyte matures. The mature fern sporophyte consists of a rhizome from which roots and fronds grow. If pieces of rhizome break away, new fern plants can develop from them by vegetative reproduction. Sporangia can develop on the fronds, spores can be released, and the cycle can begin again. The life cycle of the fern is summarized in Figure 24.5.

The Life Cycle of Conifers

The dominant stage in conifers is the sporophyte generation. One of the more familiar conifer sporophytes is shown in Figure 24.6A. The adult conifer produces male and female cones on separate branches of one plant. Cones contain spore-producing structures, or sporangia, on their scales. Female cones, which are larger than the male cones, develop two ovules on the upper surface of each cone scale. Each ovule contains a sporangium with a diploid cell that undergoes meiosis and produces four megaspores. A megaspore is a female spore that eventually can become the female gametophyte. One of the four megaspores survives and grows by cell divisions into the female gametophyte. It consists of hundreds of cells and is dependent on the sporophyte for protection and nutrition. Within the female gametophyte are two or more archegonia, each containing an egg.

Male cones have sporangia that undergo meiosis to produce male spores called microspores. Each microspore can develop into a male gametophyte, or pollen grain. Each pollen grain, with its hard, water-resistant outer covering, is a male gametophyte. Examples of male and female conifer gametophytes are shown in Figure 24.7.

In conifers, pollination is the transfer of pollen grains from the male cone to the female cone. Pollination can occur when a wind-borne pollen grain falls near the opening in one of the ovules of the female cone. The opening of the ovule is called the micropyle. The pollen grain adheres to a sticky drop of fluid that covers the micropyle. As the fluid evaporates, the pollen grain is drawn closer to the micropyle. Although pollination has occurred, fertilization does not take place for at least a year. The pollen grain and the female gametophyte will mature during this time.

As the pollen grain matures, it produces a pollen tube that grows through the micropyle and into the ovule. A sperm nucleus from the male gametophyte moves through the pollen tube to the egg. If fertilization occurs, a zygote forms. It is nourished by the female gametophyte and can develop into an embryo with several cotyledons. The cotyledons will nourish the sporophyte after germination.

Flowers and Flowering

What is a flower?

The process of sexual reproduction in flowering plants takes place in a flower, which is a complex structure made up of several organs. Some organs of the flower are directly involved in fertilization and seed production. Other floral organs function in pollination. There are probably as many different shapes, sizes, colors, and configurations of flowers as there are species of flowering plants. In fact, flower characteristics are often used in plant identification.

The structure of a flower

Even though there is an almost limitless variation in flower shapes and colors, all flowers share a basic structure. A flower’s structure is genetically determined and usually made up of four kinds of organs: sepals, petals, stamens, and pistils. The flower parts you are probably most familiar with are the petals. Petals are usually the colorful structures at the top of a flower stem. The flower stem is called the peduncle. Sepals are usually leaflike and encircle the peduncle below the petals.

Inside the petals are the stamens. A stamen is the male reproductive organ of a flower. At the tip of the stamen is the anther. The anther produces pollen that eventually contains sperm.

At the center of the flower, attached to the top of the peduncle, is usually one or more pistils. The pistil is the female organ of the flower. The bottom portion of the pistil is the ovary, a structure with one or more ovules, each usually contains one egg. As you read in the previous section, the female gametophyte develops inside the ovule. You can learn more about floral structure and practice your lab skills in the BioLab at the end of this chapter.

Modifications in flower structure

A flower that has all four organs— sepals, petals, stamens, and pistils—is called a complete flower, as shown in Figure 24.9. The morning glory, shown in Figure 24.10A, is another example of a complete flower. A flower that lacks one or more organs is called an incomplete flower. For example, walnut trees have separate male and female flowers. The male flowers, as shown in Figure 24.10B, have stamens but no pistils; the female flowers bear pistils but no stamens.

The flowers of plants such as sweet corn, as shown in Figure 24.10C, and grasses, have no petals and are adapted for pollination by wind rather than by animals. You can explore flower adaptations further in the Problem-Solving Lab on the next page.

Photoperiodism

The relative lengths of daylight and darkness each day have a significant effect on the rate of growth and the timing of flower production in many species of flowering plants. For example, some chrysanthemum plants produce flowers only during the fall, when the length of daylight is getting shorter and the length of darkness is getting longer daily.

A grower who wants to produce these chrysanthemum flowers during the middle of summer must artificially increase the length of darkness. The grower can do this by draping a black cloth over and around the chrysanthemum plants before sunset each day, and then removing it the following morning. The response of flowering plants to daily daylight-darkness conditions is called photoperiodism.

Plant biologists originally thought that the length of daylight controlled flowering. However, they now know that it is the length of darkness that controls flowering, and that the darkness must be uninterrupted. Each plant species has a critical period—specific daylight-darkness conditions that will initiate flowering. Plants can be placed in one of four categories depending on the daylight-darkness conditions that they require for flower production. Plants are short-day plants, long-day plants, day-neutral plants, or intermediate day plants.

A short-day plant flowers when the number of daylight hours is shorter than that of its critical period. Short-day plants usually flower sometime during late summer, fall, winter, or spring. Examples of short-day plants include asters, poinsettias, strawberries, ragweed, and pansies, as shown in Figure 24.11A.

A long-day plant flowers when the number of daylight hours is longer than that of its critical period. Long-day plants usually flower in summer, but also will flower if lighted continually. Carnations, petunias, potatoes, spinach, and wheat are long-day plants, as well as the lettuce shown in Figure 24.11B.

As long as the proper growing conditions exist, some plants will flower over a range in the number of day-light hours. These plants are called day-neutral plants. This category includes many tropical plants, roses, cotton, dandelions and many other weeds, as well as the cucumbers shown in Figure 24.11C.

An intermediate-day plant will not flower if days are shorter or longer than its critical period. Several grasses and sugarcane are in this category.

Photoperiodism is a physiological adaptation of all flowering plants that ensures the production of flowers at a time when there is an abundant population of pollinators. This is important because pollination is a critical event in the life cycles of all flowering plants.

The Life Cycle of a Flowering Plant

The Life Cycle of an Anthophyte

The life cycle of flowering plants is similar to that of conifers in many ways. In both coniferophytes and anthophytes, the gametophyte generation is contained within the sporophyte. Many of the reproductive structures are also similar. However, anthophytes are the only plants that produce flowers and fruits. Figure 24.12 summarizes the life cycle of a flowering plant.

Development of the female gametophyte

In anthophytes, the female gametophyte is formed inside the ovule within the ovary. In the ovule, a cell undergoes meiosis and produces haploid megaspores. One of these megaspores can develop into the female gametophyte, but the other three spores usually die. In most flowering plants, the megaspore’s nucleus undergoes mitosis three times, producing eight haploid nuclei. These eight nuclei make up the female gametophyte. Cell walls form around each of six nuclei, one of which is now called the egg cell.

The two remaining nuclei, which are called polar nuclei, are enclosed in one cell. This cell, the central cell, is located at the center of the female gametophyte. The egg cell is near the micropyle, as shown in Figure 24.13. The other five cells eventually disintegrate.

Development of the male gametophyte

The formation of the male gametophyte begins in the anther, as seen in Figure 24.14. Haploid microspores are produced by meiosis within the pollen sac. The nucleus of each microspore undergoes mitosis. A thick, protective wall surrounds these two nuclei. This structure is the immature male gametophyte, or pollen grain. The nuclei within the pollen grain are the tube nucleus and the generative nucleus. When the pollen grains mature, the anther usually splits open.

Pollination

In anthophytes, pollination is the transfer of the pollen grain from the anther to the stigma. Depending on the type of flower, the pollen can be carried to the stigma by wind, water, or animals. Plant reproduction is most successful when the pollination rate is high, which means that the pistil of a flower receives enough pollen of its own species to fertilize the egg in each ovule. Although it may seem wasteful for wind-pollinated plants to produce such large amounts of pollen, it does help ensure pollination. Many anthophytes have elaborate mechanisms that help ensure that pollen grains are deposited in the right place at the right time. Some of these are shown in Figure 24.15.

Most anthophytes that are pollinated by animals produce nectar in their flowers. Nectar is a highly concentrated food sought by visitors to these flowers. It is a liquid made up of proteins and sugars and usually collects in the cuplike area at the base of petals. Animals, such as insects and birds, brush up against the anthers while trying to get to the nectar.

The pollen that attaches to them can be carried to another flower, resulting in pollination. Some insects also gather pollen to use as food. By producing nectar and attracting animal pollinators, animal-pollinated plants are able to promote pollination with lesser amounts of pollen.

Some nectar-feeding pollinators are attracted to a flower by its color or scent or both. Some of the bright, vivid flowers attract pollinators, such as butterflies and bees. Some of these flowers have markings that are invisible to the human eye but are easily seen by insects, as shown in Figure 24.15D. Flowers that are pollinated by beetles and flies have a strong scent but are often dull in color.

Many flowers have structural adaptations that favor cross-pollination— pollination between two plants of the same species. This results in greater genetic variation because a sperm from one plant fertilizes an egg from another. For example, the flowers of certain species of orchids resemble female wasps. A male wasp visits the flower and attempts to mate with it and becomes covered with pollen, which is deposited on orchids it may visit in the future.

Fertilization

Once a pollen grain has reached the stigma of the pistil, several events take place before fertilization occurs. Inside each pollen grain are two haploid nuclei, the tube nucleus and the generative nucleus. The tube nucleus directs the growth of the pollen tube down through the pistil to the ovary, as shown in Figure 24.16. The generative nucleus divides by mitosis, producing two sperm nuclei. The sperm nuclei move through the pollen tube to a tiny opening in the ovule called the micropyle.

Within the ovule is the female gametophyte. One of the sperm unites with the egg forming a diploid zygote, which begins the new sporophyte generation. The other sperm nucleus fuses with the central cell, which contains the polar nuclei, to form a cell with a triploid (3n) nucleus. This process, in which one sperm fertilizes the egg and the other sperm joins with the central cell, is called double fertilization. Double fertilization is unique to anthophytes and is illustrated in Figure 24.16. The triploid nucleus will divide many times, eventually forming the endosperm of the seed. The endosperm is food storage tissue that supports development of the embryo in anthophyte seeds.

Many flower ovaries contain more than one ovule. For each ovule to become a seed, at least one pollen grain must land on the stigma for each ovule contained in the ovary. In a watermelon plant, for example, hundreds of pollen grains are required to pollinate a flower if each ovule is to be fertilized. You are probably familiar with the hundreds of seeds in a watermelon that are the result of this process.

Seeds and Fruits

The embryo contained within a seed is the next sporophyte generation. The formation of seeds and the fruits that enclose them, as shown in Figure 24.17, help ensure the survival of the next generation.

Seed formation

After fertilization takes place, seed development begins. Inside the ovule, the zygote divides and develops into the embryo plant. The triploid central cell develops into the seed’s endosperm The wall of the ovule becomes the seed coat, which can aid in seed dispersal and help protect the embryo until it begins growing.

Fruit formation

As the seeds develop, the surrounding ovary enlarges and becomes the fruit. Sometimes other flower organs become part of the fruit. A fruit is the structure that contains the seeds of an anthophyte.

A fruit is as unique to an anthophyte as is its flower, and many anthophytes can be identified by examining their fruit. You are familiar with plants that develop fleshy fruits, such as apples, grapes, melons, tomatoes, and cucumbers. Other plants develop dry fruits such as peanuts, sunflower “seeds,” and walnuts. In dry fruits, the ovary around the seeds hardens as the fruit matures. Some plant foods that we call vegetables or grains are actually fruits, as shown in Figure 24.18.

Can you think of any vegetables that are actually fruits? For example, green peppers are fleshy fruits that are often referred to as vegetables.

Seed dispersal

A fruit not only protects the seeds inside it, but also may aid in dispersing those seeds away from the parent plant and into new habitats. The dispersal of seeds, as shown in Figure 24.19, is important because it reduces competition for sunlight, soil, and water between the parent plant and its offspring. Animals such as raccoons, deer, bears, and birds help distribute many seeds by eating fruits. They can carry the fruit some distance away from the parent plant before consuming it and spitting out the seeds. Or they may eat the fruit, seeds and all. Seeds that are eaten usually pass through the digestive system undamaged and are deposited in the animal’s wastes. Squirrels, birds, and other nut gatherers may drop and lose seeds they collect, or even bury them only to forget where. These seeds can then germinate far from the parent plant.

Plants, such as water lilies and coconut palms that grow in or near water, produce fruits or seeds with air pockets in the walls that enable them to float and drift away from the parent plant. The ripened fruits of many plants split open to release seeds with structural adaptations for dispersal by wind or by clinging to animal fur. Orchid seeds are so tiny that they can become airborne. The fruit of the poppy flower forms a seed-filled capsule that bobs about in the wind and sprinkles its tiny seeds like a salt shaker. Tumbleweed seeds are scattered as the plant rolls along the ground.

Seed germination

At maturity, seeds are fully formed. The seed coat dries and hardens, enabling the seed to survive environmental conditions that are unfavorable to the parent plant. The seeds of some plant species must germinate within a short period of time or die.

However, the seeds of some plant species can remain in the soil until conditions are favorable for growth and development of the new plant. This period of inactivity in a mature seed is called dormancy. The length of time a seed remains dormant can vary from one species to another. Some seeds, such as willow, magnolia, and maple remain dormant for only a few weeks after they mature. These seeds cannot survive harsh conditions for long periods of time. Other plants produce seeds, like those shown in Figure 24.20, that can remain dormant for remarkably long periods of time. Even under harsh conditions, the seeds of desert wildflowers and some conifers can survive dormant periods of 15 to 20 years. Scientists discovered ancient seeds of the East Indian Lotus, Nelumbo nucifera, in China, which they have radiocarbon dated to be more than a thousand years old. Imagine their amazement when these seeds germinated!

Requirements for germination

Dormancy ends when the seed is ready to germinate. Germination is the beginning of the development of the embryo into a new plant. The absorption of water and the presence of oxygen and favorable temperatures usually end dormancy, but there may be other requirements.

Water is important because it activates the embryo’s metabolic system. Once metabolism has begun, the seed must continue to receive water or it will die. Just before the seed coat breaks open, the rate of respiration in the plant embryo increases rapidly.

Many seeds germinate best at temperatures between 25°C and 30°C. At temperatures below 0°C or above 45°C, most seeds won’t germinate at all. Some seeds have specific requirements for germination. For example, some germinate more readily after they have passed through the acid environment of an animal’s digestive system. Others require a period of freezing temperatures, such as apple seeds, extensive soaking in saltwater, such as coconut seeds, or certain day lengths. The seeds of some conifers will not germinate unless they have been exposed to fire. The same is true of certain wildflower species, including lupines and gentians, as shown in Figure 24.21.

The germination of a typical dicot embryo is shown in Figure 24.22. Once the seed coat has been softened by water, the embryo starts to emerge from the seed. The first part of the embryo to appear is the embryonic root called the radicle. The radicle grows down into the soil and develops into a root. The portion of the stem nearest the seed is called the hypocotyl. In some plants, the first part of the stem to push above ground is an arched portion of the hypocotyl. As the hypocotyl continues growing, it straightens, bringing with it the cotyledons and the plant’s first leaves. In monocots, the cotyledon remains below the soil’s surface. As growth continues, the leaves turn green, and the plant produces its own food through photosynthesis.

Vegetative reproduction

The roots, stems, and leaves of plants are called vegetative structures. When these structures produce a new plant, it is called vegetative reproduction. Vegetative reproduction is common among anthophytes. Some modified stems of anthophytes, such as potato tubers, can produce a new plant from each “eye” or bud. Farmers make use of this feature when they cut potato tubers into pieces and plant them.

Although cloning animals is a relatively new phenomenon, for years gardeners have relied on cloning to reproduce plants. Using vegetative reproduction to grow numerous plants from one plant is frequently referred to as vegetative propagation. Some plants, such as begonias, can be propagated by planting cuttings, which are pieces of the stem or a leaf that has been cut off another begonia. Even smaller pieces of plants can be used to grow plants by tissue culture. Minute pieces of plant tissue are placed on nutrient agar in test tubes or petri dishes. The plants grown from cuttings and tissue cultures have the same genetic makeup as the plants from which they came and are botanical clones.

There is great diversity in the flowers, seeds, fruits, and vegetative structures of anthophytes. Anthophytes are divided into families based on these differences. The relationships among these families are shown in Figure 24.23 on the opposite page. Which of the families is part of your environment?

Invertebrates

What is an animal?

Typical Animal Characteristics

Characteristics of Animals

All animals have several characteristics in common. Animals are eukaryotic, multicellular organisms with ways of moving that help them reproduce, obtain food, and protect themselves. Most animals have specialized cells that form tissues and organs—such as nerves and muscles. Unlike plants, animals are composed of cells that do not have cell walls.

Animals obtain food

Examine the animals shown in Figure 25.1. One characteristic common to all animals is that they are heterotrophic, meaning they must consume food to obtain energy and nutrients. All animals depend either directly or indirectly on autotrophs for food.

Scientists hypothesize that animals first evolved in water. Water is denser and contains less oxygen than air, but water usually contains more food. In water, some animals, such as barnacles and oysters, do not move from place to place and have adaptations that allow them to capture food from their water environment. Organisms that are permanently attached to a surface are called sessile. They don’t expend much energy to obtain food.

Some aquatic animals, such as the corals shown in Figure 25.2A, and sponges move about only during the early stages of their lives. They hatch from fertilized eggs into free-swimming larval forms. Most adults are sessile and attach themselves to rocks or other objects.

There is little suspended food in the air. Land animals use more oxygen and expend more energy to find food. The sidewinder snake and osprey shown in Figure 25.2B and C, can move about in their environment in an active search for food.

Animals digest food

Animals are heterotrophs that ingest their food; after ingestion, they must digest it. In some animals, digestion is carried out within individual cells; in other animals, digestion takes place in an internal cavity. Some of the food that an animal consumes and digests is stored as fat or glycogen, a polysaccharide, and used when other food is not available.

Examine the digestive tracts of a flatworm and an earthworm in Figure 25.3. Notice that there is only one opening to the flatworm’s digestive tract, a pharynx. An earthworm has a digestive tract with two openings, a mouth at one end and an anus at the other.

Animal cell adaptations

Most animal cells are differentiated and carry out different functions. Animals have specialized cells that enable them to sense and seek out food and mates, and allow them to identify and protect themselves from predators. Observe the animals in the MiniLab on this page. Can you identify any specialized cells in these animals? Find out about other specialized animal cells in the Biotechnology at the end of this chapter.

Development of Animals

Most animals develop from a fertilized egg cell called a zygote. But how does a zygote develop into many different kinds of cells that make up a snail, a fish, or a human? After fertilization, the zygote of different animal species all have similar, genetically determined stages of development.

Fertilization

Most animals reproduce sexually. Male animals produce sperm cells and female animals produce egg cells. Fertilization occurs when a sperm cell penetrates the egg cell, forming a new cell called a zygote. In animals, fertilization may be internal or external.

Cell division

The zygote divides by mitosis and cell division to form two cells in a process called cleavage. Find out how important this first cell division is in frog development by studying the Problem-Solving Lab. Once cell division has begun, the organism is known as an embryo. Recall that an embryo is an organism at an early stage of growth and development. The two cells that result from cleavage then divide to form four cells and so on, until a cell-covered, fluid-filled ball called a blastula is formed. In some animals, such as a lancelet, the blastula is a single layer of cells surrounding a fluid-filled space. In other animals, such as frogs, there may be several layers of cells surrounding the space. The blastula is formed early in the development of an animal embryo. In sea urchin development, for example, the formation of a blastula is complete about ten hours after fertilization. In humans, the blastula forms about five days after fertilization.

Gastrulation

After blastula formation, cell division continues. The cells on one side of the blastula then move inward to form a gastrula—a structure made up of two layers of cells with an opening at one end.

Gastrula formation can be compared to the way a potter creates a cup or bowl from a lump of clay, as shown in Figure 25.4. First, the clay is formed into a ball. Then, the potter presses in on the top of the ball to form a cavity that becomes the interior of the bowl. In a similar way, the cells at one end of the blastula move inward, forming a cavity lined with a second layer of cells. The layer of cells on the outer surface of the gastrula is called the ectoderm. The layer of cells lining the inner surface is called the endoderm. The ectoderm cells of the gastrula continue to grow and divide, and eventually they develop into the skin and nervous tissue of the animal. The endoderm cells develop into the lining of the animal’s digestive tract and into organs associated with digestion.

Formation of mesoderm

In some animals, the development of the gastrula progresses until a layer of cells called the mesoderm is formed. Mesoderm is found in the middle of the embryo; the term meso means “middle.” The mesoderm is the third cell layer found in the developing embryo between the ectoderm and the endoderm. The mesoderm cells develop into the muscles, circulatory system, excretory system, and, in some animals, the respiratory system. Identify and review cell differentiation in the development of an animal as shown in Figure 25.5 on the next page.

When the opening in the gastrula develops into the mouth, the animal is called a protostome. Snails, earthworms, and insects are examples of protostomes.

In other animals, such as sea stars, fishes, toads, snakes, birds, and humans, the mouth does not develop from the gastrula’s opening. An animal whose mouth developed not from the opening, but from cells elsewhere on the gastrula is called a deuterostome. Scientists hypothesize that protostome animals were the first to appear in evolutionary history, and that deuterostomes followed at a later time. Biologists today often classify an unknown organism by identifying its phylogeny. Recall that phylogeny is the evolutionary history of an organism. Determining whether an animal is a protostome or deuterostome can help biologists identify its group. Even though sea urchins, for example, are invertebrates and fishes are vertebrates, both are deuterostomes and are, therefore, more closely related than you might conclude from comparing their adult body structures.

Growth and development

Cells in developing embryos continue to differentiate and become specialized to perform different functions. Most animal embryos continue to develop over time, becoming juveniles that look like smaller versions of the adult animal. In some animals, such as insects and echinoderms, the embryo develops inside an egg into an intermediate stage called a **larva**. A larva often bears little resemblance to the adult animal. Inside the egg, the larva is surrounded by a membrane formed right after fertilization. When the egg hatches, the larva breaks through this fertilization membrane. Animals that are generally sessile as adults, such as sea urchins, often have a free-swimming larval stage, as shown in Figure 25.6.

Adult animals

Once the juvenile or **larval stage** has passed, most animals continue to grow and develop into adults. This growth and development may take just a few days in some insects, or up to fourteen years in some mammals. Eventually the adult animals reach sexual maturity, mate, and the cycle begins again.

Body Plans and Adaptations

What is symmetry?

Look at the animals shown in Figure 25.7. You know that all animals share certain characteristics, but these animals don’t look like they have much in common. The sponge seems to have no particular shape, whereas the leopard has a head, body, tail, and two pairs of legs. The jelly-fish doesn’t have a head or tail, and is circular in form. Each animal can be described in terms of symmetry—a term that describes the arrangement of body structures. Different kinds of symmetry enable animals to move about in different ways.

Asymmetry

Many sponges have an irregularly shaped body, as seen in Figure 25.8A. An animal that is irregular in shape has no symmetry or an asymmetrical body plan. Animals with no symmetry often are sessile organisms that do not move from place to place. Most adult sponges do not move about.

The bodies of most sponges consist of two layers of cells. Unlike all other animals, a sponge’s embryonic development does not include the formation of an endoderm and mesoderm, or a **gastrula stage**. Fossil sponges first appeared in rocks dating back to more than 700 million years ago. They represent one of the oldest groups of animals on Earth—evidence that their two-layer body plan makes them well adapted for life in aquatic environments.

Radial symmetry

A hydra feeds on small animals it snares with its tentacles. A hydra has radial symmetry. Its tentacles radiate out from around its mouth. As shown in Figure 25.8B, animals with radial symmetry can be divided along any plane, through a central axis, into roughly equal halves.

Radial symmetry is an adaptation that enables an animal to detect and capture prey coming toward it from any direction.

Have you ever had your groceries double bagged at the store? The body plan of a hydra can be compared to a sack within a sack. These sacks are cell layers organized into tissues with distinct functions. A hydra develops from just two embryonic cell layers— ectoderm and endoderm.

Bilateral symmetry

The butterfly in Figure 25.8C has bilateral symmetry. An organism with bilateral symmetry can be divided down its length into similar right and left halves. Bilaterally symmetrical animals can be divided in half only along one plane. In contrast, radially symmetrical animals can be divided along any vertical plane.

In bilateral animals, the anterior, or head end, often has sensory organs. The posterior of these animals is the tail end. The dorsal, or upper surface, also looks different from the ventral, or lower surface. In animals that are upright or nearly so, the back is on the dorsal surface and the belly is on the ventral surface. Animals with bilateral symmetry can find food and mates and avoid predators because they have sensory organs and good muscular control.

Bilateral Symmetry and Body Plans

Animals that are bilaterally symmetrical also share other important characteristics. All bilaterally symmetrical animals developed from three embryonic cell layers—ectoderm, endoderm, and mesoderm. Some bilaterally symmetrical animals also have fluid-filled spaces inside their bodies called body cavities in which internal organs are found. The development of fluid-filled body cavities made it possible for animals to grow larger because it allowed for the efficient circulation and transport of fluids, and support for organs and organ systems.

Acoelomates

Animals that develop from three cell layers—ectoderm, endoderm, and mesoderm—but have no body cavities are called acoelomate animals. They have a digestive tract that extends throughout the body. Acoelomate animals may have been the first group of animals in which organs evolved.

Flatworms are bilaterally symmetrical animals with solid, compact bodies, as shown in Figure 25.9. Like other acoelomate animals, the organs of flatworms are embedded in the solid tissues of their bodies. A flattened body and branched digestive tract allow for the diffusion of nutrients, water, and oxygen to supply all body cells and to eliminate wastes.

Pseudocoelomate

A roundworm is an animal with bilateral symmetry. However, unlike a flatworm, the body of a roundworm has a space that develops between the endoderm and mesoderm. It is called a pseudocoelom—a fluid-filled body cavity partly lined with mesoderm.

Pseudocoelomates can move quickly. How? Think about the way your muscles work. The muscles in your arm lift your hand by pulling against your arm bones. If there were no rigid bones in your arms, your muscles would not be able to work. Although the roundworm has no bones, it does have a rigid, fluid-filled space, the pseudocoelom. Its muscles attach to the mesoderm and brace against the pseudocoelom. You can observe this movement in the MiniLab on this page.

Pseudocoelomates have a one-way digestive tract that has regions with specific functions. The mouth takes in food, the breakdown and absorption of food occurs in the middle section, and the anus expels wastes.

Coelomates

The body cavity of an earthworm develops from a coelom, a fluid-filled space that is completely surrounded by mesoderm. Humans, insects, fishes, and many other animals have a coelomate body plan. The greatest diversity of animals is found among the coelomates.

Specialized organs and organ systems develop in the coelom. In coelomate animals, the digestive tract and other internal organs are attached by double layers of mesoderm and are suspended within the coelom. Like the pseudocoelom, the coelom cushions and protects the internal organs. It provides room for them to grow and move independently within an animal’s body.

Animal Protection and Support

Over time, the development of body cavities resulted in a greater diversity of animal species. These diverse animal species became adapted to life in different environments. Some animals, such as mollusks, evolved hard shells that protected their soft bodies. Other animals, such as sponges, evolved hardened spicules between their cells that provided support.

Some animals developed exoskeletons. An exoskeleton is a hard covering on the outside of the body that provides a framework for support. Exoskeletons also protect soft body tissues, prevent water loss, and provide protection from predators. An exoskeleton is secreted by the epidermis and extends into the body, where it provides a place for muscle attachment. As an animal grows, it secretes a new exoskeleton and sheds the old one, as shown in Figure 25.10.

Exoskeletons are often found in invertebrates. An invertebrate is an animal that does not have a backbone. Many invertebrates, such as crabs, spiders, grasshoppers, dragonflies, and beetles, have exoskeletons.

Other animals have evolved different structures for support and protection. Invertebrates, such as sea urchins and sea stars, have an internal skeleton called an endoskeleton. It is covered by layers of cells and provides support for an animal’s body. The endoskeleton protects internal organs and provides an internal brace for muscles to pull against. An endoskeleton may be made of calcium carbonate, as in sea stars; cartilage, as in sharks; or bone. Bony fishes, amphibians, reptiles, birds, and mammals all have endoskeletons made of bone.

A vertebrate is an animal with an endoskeleton and a backbone. All vertebrates are bilaterally symmetrical. Examples of vertebrates include, fishes, amphibians, reptiles, birds, and mammals. Figure 25.11 shows examples of invertebrate and vertebrate animals.

Origin of Animals

Most biologists agree that animals probably evolved from aquatic, colonial protists. Scientists trace this evolution back in time to late in the Precambrian. Although evidence suggests that bilaterally symmetrical animals might have appeared much later, many scientists agree that all the major animal body plans that exist today were already in existence at the beginning of the **Cambrian Period**, 543 million years ago. Since then, many new species have evolved but all known species have variations of the animal body plans developed during the **Cambrian Period**.

Sponges, Cnidarians, Flatworms, and Roundworms

Sponges

What is a sponge?

Sponges are asymmetrical aquatic animals that have a variety of colors, shapes, and sizes. Many are bright shades of red, orange, yellow, and green. Some sponges are ball shaped; others have many branches. Sponges can be smaller than a quarter or as large as a door. Although sponges do not resemble more familiar animals, they carry on the same life processes as all animals. Figure 26.1 shows a natural sponge harvested from the ocean.

Sponges are pore-bearers

Sponges are classified in the invertebrate phylum Porifera, which means “pore bearer.” More than 5000 species of sponges have been described. Most live in marine biomes, but about 150 species can be found in freshwater environments.

Sponges are mainly sessile organisms. Because most adult sponges can’t travel in search of food, they get their food by a process called filter feeding. Filter feeding is a method in which an organism feeds by filtering small particles of food from water that pass by or through some part of the organism. How does a sponge get rid of its wastes?

Cell organization in sponges

Like all animals, sponges are multicellular, as shown in Figure 26.2 on the opposite page. Their cells are differentiated to perform functions that help the animal survive. Read the Problem-Solving Lab on this page to find out how sponges survive in different environments. The functions of the different cell types are coordinated in a sponge, but sponges do not have tissues like those found in other animals. Tissues are groups of cells that are derived from the ectoderm, endoderm, and mesoderm in the embryo. Sponge embryos do not develop endoderm or mesoderm, so they do not have cells organized into tissues.

For some sponge species, if you took a living sponge and put it through a sieve, not only would the sponge’s cells be alive and separated out, but these cells would come together to form new sponges. It can take several weeks for the sponge’s cells to reorganize themselves.

Many biologists hypothesize that sponges evolved directly from colonial, flagellated protists, such as Volvox, described in Chapter 19. More importantly, sponges exhibit a major step in the evolution of animals—the change from a unicellular life to a division of labor among groups of organized cells.

Reproduction in sponges

Sponges can reproduce asexually and sexually. Depending on the species, asexual reproduction can be by budding, fragmentation, or the formation of gemmules. An external growth, called a bud, can form on a sponge. If a bud drops off, it can float away, settle, and grow into a sponge. Sometimes, buds do not break off. When this occurs, a colony of sponges forms, as shown in Figure 26.3. Often, fragments of a sponge break off and grow into new sponges.

Some freshwater sponges produce seedlike particles, called gemmules, in the fall when waters cool. The adult sponges die over the winter, but the gemmules survive and grow into new sponges in the spring when waters warm.

Most sponges reproduce sexually. Some sponges have separate sexes, but most sponges are hermaphrodites. A hermaphrodite is an animal that can produce both eggs and sperm. Hermaphrodism increases the likelihood that fertilization will occur in sessile or slow-moving animals. Eggs and sperm form from amoebocytes. During reproduction, sperm released from one sponge can be carried by water currents to another sponge, where fertilization can occur.

Fertilization in sponges may be either external or internal. A few sponges have external fertilization— fertilization that occurs outside the animal’s body. Most sponges have internal fertilization, in which eggs inside the animal’s body are fertilized by sperm carried into the sponge with water. In sponges, the collar cells collect and transfer sperm to amoebocytes. The amoebocytes then transport the sperm to ripe eggs. Fertilization occurs and the result is the development of free-swimming, flagellated larvae, shown in Figure 26.4.

Support and defense systems in sponges

Sponges are soft-bodied invertebrates, that can be found at depths to about 8500 m. Their internal structure gives them support and can help protect them from predators. Some sponges have sharp, hard spicules located between the cell layers. Spicules may be made of glasslike material or of calcium carbonate. Some species, such as the lake sponges shown in Figure 26.5, have thousands of tiny, sharp, needlelike spicules that make them hard for animals to eat. Other sponges have an internal framework made of silica or of spongin, a fibrous protein-like material. Sponges can be classified according to the shape and makeup of their spicules and/or frameworks.

Besides sharp spicules, some sponges may have other methods of defense. Some sponges contain chemicals that are toxic to fishes and to other predators. Scientists are studying sponge toxins to identify those that possibly could be used as medicines.

Cnidarians

What is a cnidarian?

Cnidarians are a group of invertebrates made up of more than 9000 species of jellyfishes, corals, sea anemones, and hydras. They can be found worldwide, and all but a few cnidarians live in marine biomes.

Body structure

Cnidarians are a diverse group of organisms but all have the same basic body structure, as shown in Figure 26.6. A cnidarian’s body is radially symmetrical. It has one body opening and is made up of two layers of cells. The protective outer layer of cells develops from the ectoderm of the cnidarian embryo. The endoderm of the cnidarian embryo develops into the inner layer of cells. The two cell layers are organized into tissues with specific functions. For example, the inner layer is adapted mainly to assist in digestion.

Because a cnidarian’s body is only two layers of cells, no cell is ever far from water. Oxygen dissolved in water can diffuse directly into body cells. Carbon dioxide and other wastes can move out of a cnidarian’s body cells directly into the surrounding water.

Body form

Most cnidarians undergo a change in body form during their life cycles. There are two body forms, the polyp and the medusa, as shown in Figure 26.7. A polyp has a tube-shaped body with a mouth surrounded by tentacles. A medusa has an umbrella-shaped body, called a bell, with tentacles that hang down. Its mouth is on the underside of the bell.

In cnidarians, one body form may be more observable than the other. In jellyfishes, the medusa is the body form usually observed. The jellyfish polyp is small and not easily seen. The polyp is the familiar body form of hydras. Its medusa form is small and delicate. Corals and sea anemones have only polyp forms.

Reproduction in cnidarians

All cnidarians have the ability to reproduce sexually and asexually. Sexual reproduction occurs in only one phase of the life cycle. It usually occurs in the **medusa stage**, unless there is no medusa stage then the polyp can reproduce sexually.

The most common form of reproduction in cnidarians can be illustrated by the life cycle of a jellyfish, as shown in Figure 26.7. As you can see, the sexual **medusa stage** alternates with the asexual **polyp stage**, from generation to generation. Male medusae release sperm, and female medusae release eggs into the water where fertilization occurs. The resulting zygotes develop into embryos, and then into larvae. Recall that a larva is an intermediate stage in animal development. The free-swimming larva eventually settles and grows into a polyp that reproduces asexually to form new medusae. Even though these two stages alternate in a cnidarian’s life cycle, this form of reproduction is not alternation of generations as in plants. In plants, one generation is diploid and the other is haploid. However, both cnidarian medusae and polyps are diploid animals.

Asexual reproduction can occur in either the **polyp or medusa stage**. Polyps reproduce asexually by a process known as budding, as shown in Figure 26.8. Cnidarians that remain in the **polyp stage**, such as hydras, corals and sea anemones, also can reproduce sexually as polyps.

Digestion in cnidarians

Cnidarians are predators that capture or poison their prey using nematocysts. A nematocyst is a capsule that contains a coiled, threadlike tube. The tube may be sticky or barbed, and it may contain toxic substances. Nematocysts are located in stinging cells that are on tentacles. In response to touch or chemicals in the environment, nematocysts are discharged like toy popguns, but much faster. Prey organisms are then taken in for digestion.

The origins of a digestive process, which is similar to that of animals that evolved later, are found in cnidarians. Once captured by nematocysts, prey is brought to the mouth by contraction of the tentacles.

As shown in Figure 26.9 on the previous page, the inner cell layer of cnidarians surrounds a space called a gastrovascular cavity. Cells adapted for digestion line the gastrovascular cavity and release enzymes over captured prey. Any undigested materials are ejected back out through the mouth. You can observe a cnidarian feeding in the MiniLab on this page. Cnidarians are classified into groups partly based on whether or not there are divisions within the gastrovascular cavity, and if there are, how many divisions are present.

Nervous system in cnidarians

A cnidarian has a simple nervous system without a control center, such as a brain like that of other animals. In cnidarians, the nervous system consists of a nerve net that conducts impulses to and from all parts of the body. The impulses from the nerve net cause contractions of musclelike cells in the two cell layers. For example, the movement of tentacles when a cnidarian captures prey is the result of contractions of these musclelike cells.

Diversity of Cnidarians

There are four classes of cnidarians: Hydrozoa, Scyphozoa, Cubozoa, and Anthozoa. Cubozoans once were classified as scyphozoans.

Most hydrozoans form colonies

The class Hydrozoa includes two groups—the hydroids, such as hydra, and the siphonophores, including the Portuguese man-of-war. Most hydroids are marine animals that consist of branching polyp colonies formed by budding, and are found attached to pilings, shells, and other surfaces. The siphonophores include floating colonies that drift about on the ocean’s surface. Hydrozoans have open gastrovascular cavities with no internal divisions.

It’s difficult to understand how the organism shown in Figure 26.10 could be a closely associated group of individual animals. The Portuguese man-of-war, Physalia, is an example of a siphonophore hydrozoan colony. Each individual in a Physalia colony has a function that helps the entire organism survive. For example, just one individual forms a large, blue, gas-filled float. Regulation of gases in the float allows the colony to sink to lower depths or rise to the water’s surface. Other polyps hanging from the float have functions, such as reproduction and feeding. The polyps all function together for the survival of the colony.

Scyphozoans are the jellyfishes

Have you ever seen a jellyfish like the one shown in Figure 26.11? The fragile and sometimes luminescent bodies of jellyfishes can be beautiful. Some jellyfishes are transparent, but others are pink, blue, or orange. The medusa form is the dominant stage in this class.

The gastrovascular cavity of scyphozoans has four internal divisions. Like other cnidarians, scyphozoans have musclelike cells in their outer cell layer that can contract. When these cells contract together, the bell contracts, which propels the animal through the water.

Jellyfishes can be found everywhere in the oceans, from arctic to tropical waters. They have been seen at depths of more than 3000 m. Swimmers should avoid jellyfishes because of their painful stings.

Most anthozoans build coral reefs

Anthozoans are cnidarians that exhibit only the polyp form. All anthozoans have many incomplete divisions in their gastrovascular cavities.

Sea anemones are anthozoans that live as individual animals, and are thought to live for centuries. They can be found in tropical, temperate, and arctic seas. Some tropical sea anemones may have a diameter of more than a meter.

Corals are anthozoans that live in colonies of polyps in warm ocean waters around the world. They secrete protective, cuplike calcium carbonate shelters around their soft bodies. Colonies of many coral species build the beautiful coral reefs that provide food and shelter for many other marine species. Corals that form reefs are known as hard corals. Other corals are known as soft corals because they do not build such structures. When a coral polyp dies, its shelter is left behind, which adds to the coral reef’s structure.

The living portion of a coral reef is a thin, fragile layer that grows on top of the shelters left behind by previous generations. Coral reefs form slowly. It took thousands of years to form the reefs found today in tropical and subtropical waters. Find out more about the fragility of coral reefs by reading the Biology and Society feature at the end of this chapter.

A coral polyp extends its tentacles to feed, as shown in Figure 26.12. Although corals are often found in relatively shallow, nutrient-poor waters, they thrive because of their symbiotic relationship with microscopic, photosynthetic protists called zooxanthellae. The zooxanthellae produce oxygen and food that the corals use, while using carbon dioxide and waste materials produced by the corals. These protists are primarily responsible for the bright colors found in coral reefs. Because the zooxanthellae are free-swimming, they sometimes leave the corals. Corals without these protists often die. You can find out how corals respond to changing environmental conditions in Problem-Solving Lab 26.2.

Origins of Sponges and Cnidarians

As shown in Figure 26.13, sponges represent an old animal phylum. The earliest fossil evidence for sponges dates this group to late in the Precambrian, about 650 million years ago. Scientists infer that sponges may have evolved directly from a group of flagellated protists that today resemble the collar cells of sponges.

The earliest known cnidarians also date to the Precambrian, about 630 million years ago. Because cnidarians are soft-bodied animals, they do not preserve well as fossils, and their origins are not well understood. The earliest coral species were not reef builders, so reefs cannot be used to date early cnidarians. The larval form of cnidarians resembles protists, and because of this, scientists consider cnidarians to have evolved from protists.

Flatworms

What is a flatworm?

To most people, the word worm describes a spaghetti-shaped animal. Many animals have this general appearance, but are classified into different phyla. The least complex worms belong to the phylum Platyhelminthes, as shown in Figure 26.14. These flatworms are acoelomates with thin, solid bodies. They range in size from 1 mm up to several meters. There are approximately 14 500 species of flatworms found in marine and freshwater environments and in moist habitats on land.

The most well-known members of this phylum are the parasitic tapeworms and flukes, which cause diseases in other animals, among them frogs and humans. The most commonly studied flatworms in biology classes are the free-living planarians.

Nervous control in planarians

Most of a planarian’s nervous system is located in its head—a characteristic common to other bilaterally symmetrical animals. The nervous system enables a planarian to respond to stimuli in its environment. Some flatworms have a nerve net, and others have the beginnings of a central nervous system. A planarian’s nervous system includes two nerve cords that run the length of the body, as shown in Figure 26.15. It also includes eyespots that can detect the presence or absence of light and sensory cells that can detect chemicals and movement in water. At the anterior end of the nerve cord is a small swelling called a ganglion. The ganglion receives messages from the eyespots and sensory pits, then communicates with the rest of the body along the nerve cords. Messages from the nerve cords trigger responses in a planarian’s muscle cells.

Reproduction in planarians

Like many of the organisms studied in this chapter, most flatworms including planarians, are hermaphrodites. During sexual reproduction, individual planarians exchange sperm, which travel along special tubes to reach the eggs. Fertilization occurs internally. The zygotes are released in capsules into the water, where they hatch into tiny planarians.

Planarians also can reproduce asexually. When a planarian is damaged, it has the ability to regenerate, or regrow, new body parts. Regeneration is the replacement or regrowth of missing body parts. Missing body parts are replaced through cell divisions. If a planarian is cut horizontally, the section containing the head will grow a new tail, and the tail section will grow a new head. Thus, a planarian that is damaged or cut into two pieces may grow into two new organisms—a form of asexual reproduction. The BioLab at the end of this chapter is about regeneration in planarians.

Feeding and digestion in planarians

A planarian feeds on dead or slow-moving organisms. It extends a tube-like, muscular organ, called the pharynx, out of its mouth, as shown in Figure 26.16. Enzymes released by the pharynx begin digesting food outside the animal’s body. Food particles are sucked into the digestive tract, where they are broken up. Cells lining the digestive tract obtain food by endocytosis. Food is thus digested in individual cells.

Feeding and digestion in parasitic flatworms

A parasitic flatworm is adapted to obtaining nutrients from inside the bodies of one or two hosts. Recall that a parasite is an organism that lives on or in another organism and depends upon that host organism for its food. Parasitic flatworms have mouthparts with hooks that keep the flatworm firmly attached inside its host. Because they are surrounded by nutrients, they do not need to move to seek out or find food. Parasitic flat-worms do not have complex nervous or muscular tissue.

Tapeworm bodies have sections

The body of a tapeworm is made up of a knob-shaped head called a scolex, and detachable, individual sections called proglottids, as shown in Figure 26.17. A proglottid contains muscles, nerves, flame cells, and male and female reproductive organs. Each proglottid can contain up to 100 000 eggs. Some adult tapeworms that live in animal intestines can be more than 10 m in length and consist of 2000 proglottids.

The life cycle of a fluke

A fluke is a parasitic flatworm that spends part of its life in the internal organs of a vertebrate, such as a human or a sheep. It obtains its nutrition by feeding on cells, blood, and other fluids of the host organism. Flukes have complex life cycles that can include one, two, or more invertebrate and/or vertebrate hosts.

Blood flukes of the genus Schistosoma, as shown in Figure 26.18, cause a disease in humans known as schistosomiasis. Schistosomiasis is common in countries where rice is grown. Farmers must work in standing water in rice fields during planting and harvesting. Blood flukes are common where the secondary host, snails, also are found.

Roundworms

What is a roundworm?

Roundworms belong to the phylum Nematoda. They are widely distributed, living in soil, animals, and both freshwater and marine environments. More than 12 000 species of roundworms are known to scientists. Most roundworm species are free-living, but many are parasitic. Nearly all plant and animal species are affected by parasitic roundworms.

Roundworms are tapered at both ends. They have a thick outer covering, which they shed four times as they grow, that protects them in harsh environments. Roundworms look like tiny, wriggling bits of thread. They lack circular muscles but have lengthwise muscles. As one muscle contracts, another muscle relaxes. This alternating contraction and relaxation of muscles causes roundworms to move in a thrashing fashion.

Roundworms have a pseudocoelom and are the simplest animals with a tubelike digestive system. Unlike flatworms, roundworms have two body openings—a mouth and an anus. The free-living species have well-developed sense organs, such as eyespots, although these are reduced in parasitic forms.

Diversity of Roundworms

Approximately half of the described roundworm species are parasites, and about 50 species infect humans.

Roundworm parasites of humans

Infection by Ascaris, shown in Figure 26.19, is the most common roundworm infection in humans. It occurs worldwide but is more common in subtropical or tropical areas. Children become infected more often than adults do. Eggs of Ascaris are found in soil and enter a human’s body through the mouth. The eggs hatch in the intestines, move into the bloodstream, and eventually to the lungs, where they are coughed up, swallowed, and begin the cycle again.

Pinworms are the most common human roundworm parasites in the United States. The highest incidence of infection is in children. Pinworms are highly contagious because eggs can survive for up to two weeks on surfaces. Its life cycle begins when live eggs are ingested. They mature in the host’s intestinal tract. Female pinworms exit the host’s anus—usually as the host sleeps—and lay eggs on nearby skin. These eggs fall onto bedding or other surfaces.

Trichinella causes a disease called trichinosis. This roundworm can be ingested in raw or undercooked pork, pork products, or wild game. Trichinosis can be controlled by properly cooking meat. Find out what these roundworms look like in MiniLab 26.2.

Hookworm infections are common in humans in warm climates where they walk on contaminated soil in bare feet. Hookworms cause people to feel weak and tired due to blood loss.

Roundworm parasites of other organisms

About 1200 species of nematodes cause diseases in plants. Nematodes can infect and kill pine trees, cereal crops, and food plants such as potatoes. They are particularly attracted to plant roots, as shown in Figure 26.20, and cause a slow decline of the plant. They also can infect fungi and can form symbiotic associations with bacteria. Nematodes also can be used to control pests, as described in Problem-Solving Lab 26.4.

Mollusks and Segmented Worms

Mollusks

What is a mollusk?

Slugs, snails, squids, and some animals that live in shells in the ocean or on the beach are all mollusks. These organisms belong to the phylum Mollusca. Members of this phylum range from the slow moving slug to the jet-propelled squid. Although most species live in the ocean, others live in freshwater and moist terrestrial habitats. Some aquatic mollusks, such as oysters, live much of their lives firmly attached to the ocean floor or to submerged docks or parts of boats. Others, such as the octopus, swim freely in the ocean. Land-dwelling slugs and snails can be found slowly moving over leaves on the forest floor. Examples of mollusks are shown in Figure 27.1.

Some mollusks have shells, and others, including slugs and squids, are adapted to life without a hard covering. All mollusks have bilateral symmetry, a coelom, a digestive tract with two openings, a muscular foot, and a mantle. The mantle is a membrane that surrounds the internal organs of the mollusk. In shelled mollusks, the mantle secretes the shell.

Although mollusks look different from one another on the outside, they share many similarities on the inside. You can see the similarities and the differences in these body areas in Figure 27.2 as you compare a snail and a squid.

How mollusks obtain food

Have you ever watched a snail clean algae from the sides of an aquarium? Snails, like many mollusks, use a rasping structure called a radula to obtain food. A radula, located within the mouth of a mollusk, is a tonguelike organ with rows of teeth. The radula is used to drill, scrape, grate, or cut food. Figure 27.3 shows the results of the use of a radula. How does a radula cut food? Find out in Figure 27.4. Octopuses and squids are predators that use their radulas to tear up the food that they capture with their tentacles. Other mollusks are grazers and some are filter feeders. Bivalves do not have radulas; they filter food from the water.

Reproduction in mollusks

Mollusks reproduce sexually and most have separate sexes. In most aquatic species, eggs and sperm are released at the same time into the water, where external fertilization takes place. Many gastropods that live on land, and a few bivalves, are hermaphrodites and produce both eggs and sperm. Fertilization is internal.

Although members of the phylum Mollusca have different appearances as adults, they all share similar developmental patterns. One larval stage of most mollusks is similar, as you can see in Figure 27.5.

Some marine mollusks have free-swimming larvae that propel themselves by cilia. Most marine snails and bivalves have another developmental stage called a **veliger** in which the beginnings of a foot, shell, and mantle can be seen.

Nervous control in mollusks

Mollusks have simple nervous systems that coordinate their movement and behavior. Some more advanced mollusks have a brain. Most mollusks have paired eyes that range from simple cups that detect light to the complex eyes of octopuses that have irises, pupils, and retinas similar to the eyes of humans.

Circulation in mollusks

Mollusks have a well-developed circulatory system that usually includes a two or three-chambered heart. In most mollusks, the heart pumps blood through an open circulatory system. In an open circulatory system, the blood moves through vessels and into open spaces around the body organs. This adaptation exposes body organs directly to blood that contains nutrients and oxygen, and removes metabolic wastes. Some mollusks, such as octopuses, move nutrients and oxygen through a closed circulatory system. In a closed circulatory system, blood moves through the body enclosed entirely in a series of blood vessels. A closed system provides an efficient means of gas exchange within the body.

Respiration in mollusks

Most mollusks have respiratory structures called gills. Gills are specialized parts of the mantle that consist of a system of filamentous projections that contain a rich supply of blood for the transport of gases. Gills increase the surface area through which gases can diffuse. In land snails and slugs, the mantle cavity appears to have evolved into a primitive lung.

Excretion in mollusks

Mollusks are the oldest known animals to have evolved excretory structures called nephridia. Nephridia are organs that remove metabolic wastes from an animal’s body. Mollusks have one or two nephridia that collect wastes from the coelom, which is located around the heart only. Wastes are discharged into the mantle cavity, and expelled from the body by the pumping of the gills.

Diversity of Mollusks

Phylum Mollusca is large and diverse. Three mollusk classes— Gastropoda, Bivalvia, and Cephalopoda—include the most common and well-known species.

Gastropods:

One-shelled mollusks

The largest class of mollusks is Gastropoda, or the stomach-footed mollusks. The name comes from the way the animal’s large foot is positioned under the rest of its body. Most species of gastropods have a shell. Other gastropod species, such as slugs, have no shell.

Shelled gastropods include snails, abalones, conches, periwinkles, whelks, limpets, cowries, and cones. They can be found in freshwater, saltwater, or moist terrestrial habitats. Shelled gastropods may be plant eaters, predators, or parasites. Figure 27.6 shows two examples of shelled gastropods.

Instead of being protected by a shell, the body of a slug is protected by a thick layer of mucus. Colorful sea slugs, also called nudibranchs, are protected in another way. When certain species of sea slugs feed on jellyfishes, they incorporate the poisonous nematocysts of the jellyfish into their own tissues without causing these cells to discharge. Any fishes trying to eat the sea slugs are repelled when the nematocysts discharge into the unlucky predator. The bright colors of these gastropods warn predators of the potential danger, as shown in Figure 27.7.

Bivalves: Two-shelled mollusks

Two-shelled mollusks such as clams, oysters, and scallops belong to the class Bivalvia, illustrated in Figure 27.8. Most bivalves are marine, but a few species live in freshwater habitats. Bivalves occur in a range of sizes. Some are less than 1 mm in length and others, such as the tropical giant clam, may be 1.5 m long. Bivalves have no distinct head or radula. Most use their large, muscular foot for burrowing in the mud or sand at the bottom of the ocean or a lake. A ligament, like a hinge, connects their two shells, called valves; strong muscles allow the valves to open and close over the soft body. See if you can identify the shells pictured in the MiniLab by using the dichotomous key given.

One of the main differences between gastropods and bivalves is that bivalves are filter feeders that obtain food by filtering small particles from the surrounding water. Bivalve mollusks have several adaptations for filter feeding, including gill cilia that beat to draw water in through an incurrent siphon. As water moves over the gills, food and sediments become trapped in mucus. Cilia that line the gills push food particles to the mouth. Cilia also act as sorting devices. Large particles, sediment, and anything else that is rejected is transported to the mantle where it is expelled through the excurrent siphon, or to the foot, where it is eliminated from the animal’s body.

Cephalopods:

Head-footed mollusks

The head-footed mollusks are marine organisms in the class Cephalopoda. This class includes the octopus, squid, cuttlefish, and chambered nautilus, as shown in Figure 27.9. The only cephalopod with a shell is the chambered nautilus, but some species, such as the cuttlefish, have a reduced internal shell. Scientists consider the cephalopods to have the most complex structures and to be the most recently evolved of all mollusks.

In cephalopods, the foot has evolved into tentacles with suckers, hooks, or adhesive structures. Cephalopods swim or walk over the ocean floor in pursuit of their prey, capturing it with their tentacles. Once tentacles have captured prey, it is brought to the mouth and bitten with beaklike jaws. Then the food is torn and pulled into the mouth by the radula.

Like bivalves, cephalopods have siphons that expel water. These mollusks can expel water forcefully in any direction, and move quickly by jet propulsion. Squids can attain speeds of 20 m per second using this system of movement. You may be aware that cephalopods use jet propulsion to escape from danger. Squids and octopuses also can release a dark fluid to cloud the water. This “ink” helps to confuse their predators so they can make a quick escape.

Segmented Worms

What is a segmented worm?

Segmented worms are classified in the phylum Annelida. They include leeches and bristleworms, shown in Figure 27.10, as well as earthworms. Segmented worms are bilaterally symmetrical and have a coelom and two body openings. Some have a larval stage that is similar to the larval stages of certain mollusks, suggesting a common ancestor.

The basic body plan of segmented worms is a tube within a tube. The internal tube, suspended within the coelom, is the digestive tract. Food is taken in by the mouth, an opening in the anterior end of the worm, and wastes are released through the anus, an opening at the posterior end.

Most segmented worms have tiny bristles called setae on each segment. The setae help segmented worms move by providing a way to anchor their bodies in the soil so each segment can move the animal along. Segmented worms can be found in most environments, except in the frozen soil of the polar regions and the dry sand and soil of the deserts.

Earthworms are just one of about 15 000 species of segmented worms that live in soil, freshwater, and the sea. Can you identify a segmented worm? Find out by reading the Problem-Solving Lab on this page.

Segmentation supports diversified functions

The most distinguishing characteristic of segmented worms is their cylindrical bodies that are divided into ringed segments, as seen in the worm in Figure 27.11. In most species, this segmentation continues internally as each segment is separated from the others by a body partition. Segmentation is an important adaptation for movement because each segment has its own muscles, allowing shortening and lengthening of the body.

Segmentation also allows for specialization of body tissues. Groups of segments may be adapted for a particular function. Certain segments have modifications for functions such as sensing and reproduction.

Nervous system

Segmented worms have simple nervous systems in which organs in anterior segments have become modified for sensing the environment. Some sensory organs are sensitive to light, and eyes with lenses and retinas have evolved in certain species. In some species there is a brain located in an anterior segment. Nerve cords connect the brain to nerve centers called ganglia, located in each segment.

Circulation and respiration

Segmented worms have a closed circulatory system. Blood carrying oxygen to and carbon dioxide from body cells flows through vessels to reach all parts of the body. Segmented worms must live in water or in wet areas on land because they also exchange gases directly through their moist skin.

Digestion and excretion

Segmented worms have a complete internal digestive tract that runs the length of the body. Food and soil taken in by the mouth eventually pass to the gizzard. In the gizzard, a muscular sac and hard particles help grind soil and food before they pass into the intestine. Undigested material and solid wastes pass out the worm’s body through the anus. Segmented worms have two nephridia in almost every segment that collect waste products and transport them through the coelom and out of the body. Find out what an earthworm eats in figure.

Reproduction in segmented worms

Earthworms and leeches are hermaphrodites, producing both eggs and sperm. During mating, two worms exchange sperm. Each worm forms a capsule for the eggs and sperm. The eggs are fertilized in the capsule, then the capsule slips off the worm and is left behind in the soil. In two to three weeks, young worms emerge from the eggs.

Bristleworms and their relatives have separate sexes and reproduce sexually. Usually eggs and sperm are released into the seawater, where fertilization takes place. Bristleworm larvae hatch in the sea and become part of the plankton. Once segment development begins, the worm settles to the bottom.

Diversity of Segmented Worms

The phylum Annelida includes three classes: class Oligochaeta, earthworms; class Polychaeta, bristle- worms and their relatives; and class Hirudinea, leeches.

Earthworms

Earthworms are the most well-known annelids because they can be seen easily by most people. Although earthworms have a definite anterior and posterior section, they do not have a distinct head. Earthworms have only a few setae on each segment. What does an earthworm look like internally?

Earthworms eat their way through soil. As they eat, they create spaces for air and water to flow through soil. As soil passes through their digestive tracts, nutrients are extracted and undigested materials pass out of the worms. Castings, the wastes of an earthworm, help fertilize soil.

Bristleworms and their relatives

The class Polychaeta includes bristleworms and their relatives— fanworms, shown in Figure 27.13, lug worms, plumed worms, and sea mice. Polychaetes are primarily marine organisms. Most body segments of a polychaete have many setae, hence the name. Polychaete means “many bristles.” Most body segments of a polychaete also have a pair of appendages called parapodia, which can be used for swimming or crawling over corals and the bottom of the sea. Parapodia also function in gas exchange. A polychaete has a head with well-developed sense organs, including eyes.

Leeches

Leeches are segmented worms with flattened bodies and usually no setae. Most leeches live in freshwater streams or rivers. Unlike earthworms, many species are parasites that suck blood or other body fluids from the bodies of their hosts, which include ducks, turtles, fishes, and humans. Front and rear suckers enable leeches to attach themselves to their hosts.

You may cringe at the thought of being bitten by a leech, but the bite is not painful. This is because the saliva of the leech contains chemicals that act as an anesthetic. Other chemicals prevent the blood from clotting. A leech can ingest two to five times its own weight in one meal. Once fed, a leech will drop off its host. It may not eat again for months.

Origins of Mollusks and Segmented Worms

Fossil records show that mollusks lived in great numbers as long as 500 million years ago. Gastropod, bivalve, and cephalopod fossils have been found in Precambrian deposits. Some species, such as the chambered nautilus, appear to have changed very little from related species that lived long ago. Find out how fossil mollusks are used to determine the age of rocks in the Connection to Earth Science at the end of this chapter.

Annelids probably evolved in the sea, perhaps from larvae of ancestral flatworms. The fossil record for segmented worms is limited because segmented worms have almost no hard body parts. Tubes constructed by polychaetes are the most common fossils of this phylum. Some of these tubes appear in the fossil record as early as 540 million years ago, as you can see in Figure 27.14.

Arthropods

Characteristics of Arthropods

What is an arthropod?

Arthropods pollinate many of the flowering plants on Earth. Some arthropods spread plant and animal diseases. Despite the enormous diversity of arthropods, they all share some common characteristics.

A typical arthropod is a segmented, coelomate invertebrate animal with bilateral symmetry, an exoskeleton, and jointed structures called appendages. An appendage is any structure, such as a leg or an antenna, that grows out of the body of an animal. In arthropods, appendages are adapted for a variety of purposes including sensing, walking, feeding, and mating. Figure 28.1 shows some of these adaptations.

Arthropods are the earliest known invertebrates to exhibit jointed appendages. Joints are advantageous because they allow more flexibility, especially in animals that have hard, rigid exoskeletons. Joints also allow powerful movements of appendages, and enable an appendage to be used in many different ways. For example, the second pair of appendages is used for mating in spiders and for seizing prey in scorpions.

Arthropod exoskeletons provide protection

The success of arthropods as a group can be attributed in part to the presence of an exoskeleton. The exoskeleton is a hard, thick, outer covering made of protein and chitin. Chitin also is found in the cell walls of fungi and in other animals. In some species, the exoskeleton is a continuous covering over most of the body. In other species, the exoskeleton is made of separate plates held together by hinges. The exoskeleton protects and supports internal tissues and provides places for attachment of muscles. In many species that live on land, the exoskeleton is covered by a waxy layer that provides additional protection against water loss. In many aquatic species, the exoskeletons are reinforced with calcium carbonate.

Why arthropods must molt

Exoskeletons are an important adaptation for arthropods, but they also have their disadvantages. First, they are relatively heavy structures. The larger an arthropod is, the thicker and heavier its exoskeleton must be to support its larger muscles. Thus, the weight of the exoskeleton limits the size of arthropods. However, many terrestrial and flying arthropods have adapted to their habitats by having a thinner, lighter- weight exoskeleton, which offers less protection but allows the animal more freedom to fly and jump.

A second and more important disadvantage is that exoskeletons cannot grow, so they must be shed periodically. Shedding the old exoskeleton is called molting. Before an arthropod molts, a new, soft exoskeleton is formed beneath the old one. When the new exoskeleton is ready, the animal contracts muscles and takes in air or water. This causes the animal’s body to swell until the old exoskeleton splits open, usually along the back. The animal then sheds its old exoskeleton, as shown in Figure 28.2. Before the new exoskeleton hardens, the animal puffs up as a result of increased blood circulation to all parts of its body. Many insects and spiders increase in size by also taking in air. Thus, the new exoskeleton hardens in a larger size, allowing some room for the animal to continue to grow.

Most arthropods molt four to seven times in their lives before they become adults. During molting, they are particularly vulnerable to predators. When the new exoskeleton is soft, arthropods cannot protect themselves from danger because they move by bracing muscles against the rigid exoskeleton. Therefore, many species hide or remain motionless for a few hours or days until the new exoskeleton hardens.

Segmentation in arthropods

Most arthropods are segmented, but they do not have as many segments as you have seen in segmented worms. In most groups of arthropods, segments have become fused into three body sections—head, thorax, and abdomen. In other groups, even these segments may be fused. Some arthropods have a head and a fused thorax and abdomen. In other groups, there is an abdomen and a fused head and thorax called a cephalothorax, as shown in Figure 28.3B.

Fusion of the body segments is related to movement and protection. Beetles and some other arthropod groups that have separate head and thorax regions are more flexible than those with fused regions. Many species, such as shrimps, lobsters, and crayfishes, have a cephalothorax, which protects the animal but which limits movement of the body regions. Take a closer look at the fused body segments of a lobster in the MiniLab on this page.

Arthropods have efficient gas exchange

Arthropods are generally quick, active animals. They crawl, run, climb, dig, swim, and fly. As you would expect, arthropods have efficient respiratory structures that ensure rapid oxygen delivery to cells. This large oxygen demand is needed to sustain the high levels of metabolism required for rapid movements.

Three types of respiratory structures have evolved in arthropods: gills, tracheal tubes, and book lungs. In some arthropods, air diffuses across the exoskeleton and body wall. For this to happen, the exoskeleton must be thin and permeable. Aquatic arthropods exchange gases through gills, which extract oxygen from water and release carbon dioxide into the water. Land arthropods have either a system of tracheal tubes or book lungs. Most insects have tracheal tubes, branching networks of hollow air passages that carry air throughout the body. Muscle activity helps pump the air through the tracheal tubes. Air enters and leaves the tracheal tubes through openings on the thorax and abdomen called spiracles.

Most spiders and their relatives have book lungs, air-filled chambers that contain leaflike plates. The stacked plates of a book lung are arranged like pages of a book. All three types of respiration in arthropods are illustrated in Figure 28.4.

Arthropods have acute senses

Quick movements that are the result of strong muscular contractions enable arthropods to respond to a variety of stimuli. Movement, sound, and chemicals can be detected with great sensitivity by antennae, stalklike structures that detect changes in the environment.

Antennae are also used for sound and odor communication among animals. Have you ever watched as a group of ants carried home a small piece of food? The ants were able to work together as a group because they were communicating with each other by pheromones, chemical odor signals given off by animals. Antennae sense the odors of pheromones, which signal animals to engage in a variety of behaviors. Some pheromones are used as scent trails, such as in the group-feeding behavior of ants, and many are important in the mating behavior of arthropods.

Accurate vision is also important to the active lives of arthropods. Most arthropods have one pair of large compound eyes and three to eight simple eyes. A simple eye is a visual structure with only one lens that is used for detecting light. A compound eye is a visual structure with many lenses. Each lens registers light from a tiny portion of the field of view. The total image that is formed is made up of thousands of parts. The multiple lenses of a flying arthropod, such as the dragonfly shown in Figure 28.5, enable it to analyze a fast-changing landscape during flight. Compound eyes can detect the movements of prey, mates, or predators, and can also detect colors.

Arthropod nervous systems are well developed

Arthropods have well-developed nervous systems that process information coming in from the sense organs. The nervous system consists of a double ventral nerve cord, an anterior brain, and several ganglia. Arthropods have ganglia that have become fused. These ganglia act as control centers for the body section in which they are located.

Arthropods have other complex body systems

Arthropod blood is pumped by a heart in an open circulatory system with vessels that carry blood away from the heart. The blood flows out of the vessels, bathes the tissues of the body, and returns to the heart through open body spaces.

Arthropods have a complete digestive system with a mouth, stomach, intestine, and anus, together with various glands that produce digestive enzymes. The mouthparts of most arthropod groups include one pair of jaws called mandibles. The mandibles, together with other mouthparts, illustrated in Figure 28.6, are adapted for holding, chewing, sucking, or biting the various foods eaten by arthropods.

Most terrestrial arthropods excrete wastes through Malpighian tubules. In insects, the tubules are all located in the abdomen rather than in each segment, as you have seen with nephridia in segmented worms. Malpighian tubules are attached to and empty into the intestine.

Another well-developed system in arthropods is the muscular system. In a human limb, muscles are attached to the outer surfaces of internal bones. In an arthropod limb, the muscles are attached to the inner surface of the exoskeleton. An arthropod muscle is attached to the exoskeleton on both sides of the joint.

Arthropods reproduce sexually

Most arthropod species have separate males and females and reproduce sexually. Fertilization is usually internal in land species but is often external in aquatic species. A few species, such as barnacles, are hermaphrodites, animals with both male and female reproductive organs. Some species, including bees, ants, aphids, and wasps, exhibit parthenogenesis, a form of asexual reproduction in which a new individual develops from an unfertilized egg.

Reproductive diversity is one reason there are more arthropod species than all other animal species combined.

Diversity of Arthropods

Arachnids

Do you remember the last time you saw a spider? Did you draw back with a quick, fearful breath, or did you move a little closer, curious to see what it would do next? Of all 35 000 species of spiders, only about a dozen are dangerous to humans. In North America, you need to watch out for only the two species illustrated in Figure 28.7—the female black widow and the brown recluse.

What is an arachnid?

Spiders, scorpions, mites, and ticks belong to the class Arachnida. Spiders are the largest group of arachnids. Spiders and other arachnids have only two body regions—the cephalothorax and the abdomen. Arachnids have six pairs of jointed appendages.

The first pair of appendages, called chelicerae, is located near the mouth. Chelicerae are often modified into pincers or fangs. Pincers are used to hold food, and fangs inject prey with poison. Spiders have no mandibles for chewing. Using a process of extracellular digestion, digestive enzymes from the spider’s mouth liquefy the internal organs of the captured prey. The spider then sucks up the liquefied food.

The second pair of appendages, called the pedipalps, are adapted for handling food and for sensing. In male spiders, pedipalps are further modified to carry sperm during reproduction. The four remaining appendages in arachnids are adapted as legs for locomotion. Arachnids have no antennae.

Most people know spiders for their ability to make elaborate webs. Although all spiders spin silk, not all make webs. Spider silk is secreted by silk glands in the abdomen. As silk is secreted, it is spun into thread by structures called spinnerets, located at the rear of the spider. How does a spider catch its prey? Find out in Figure 28.9 on the opposite page.

Ticks, mites, and scorpions: Spider relatives

Arachnida also includes ticks, mites, and scorpions. Ticks and mites differ from spiders in that they have only one body section, as shown in Figure 28.8. The head, thorax, and abdomen are completely fused. Ticks feed on blood from reptiles, birds, and mammals. They are small but capable of expanding up to 3 times or more of their original size after a blood meal. Ticks also can spread diseases.

Mites feed on fungi, plants, and animals. They are so small that they often are not visible to the unaided human eye. However, you can certainly feel their irritating bites. Like ticks, mites can transmit diseases.

Scorpions are easily recognized by their many abdominal body segments and enlarged pincers. They have a long tail with a venomous stinger at the tip. Scorpions live in warm, dry climates and eat insects and spiders. They use the poison in their stingers to paralyze large prey organisms.

Crustaceans

Crustaceans are the only arthropods that have two pairs of antennae for sensing. Some crustaceans have three body sections, and others have only two. All crustaceans have mandibles for crushing food and typically have two compound eyes, often located on movable stalks. Unlike the up-and- down movement of your jaws, crustacean mandibles open and close from side to side. Many crustaceans have five pairs of walking legs. A crustacean’s walking legs are used for walking, seizing prey, and cleaning other appendages. The first pair of walking legs are often modified into strong claws for defense.

Members of the class Crustacea include crabs, lobsters, shrimps, cray-fishes, water fleas, pill bugs, and barnacles, shown in Figure 28.10. Most crustaceans are aquatic and exchange gases as water flows over feathery gills. Sow bugs and pill bugs, two of the few land crustaceans, must live where there is moisture, which aids in gas exchange. They are frequently found in damp areas around building foundations. You can observe crustaceans in the BioLab at the end of this chapter.

Centipedes and Millipedes

Centipedes, which belong to the class Chilopoda, and millipedes, members of the class Diplopoda, are shown in Figure 28.11. Like spiders, millipedes and centipedes have Malpighian tubules for excreting wastes. In contrast to spiders, centipedes and millipedes have tracheal tubes rather than book lungs for gas exchange.

If you have ever turned over a rock on a damp forest floor, you may have seen the flattened bodies of centipedes wriggling along on their many tiny, jointed legs. Centipedes are carnivorous and eat soil arthropods, snails, slugs, and worms. The bites of some centipedes are painful to humans.

A millipede eats mostly plants and dead material on damp forest floors. Millipedes do not bite, but they can spray foul-smelling fluids from their defensive stink glands. You may have seen their cylindrical bodies walking with a slow, graceful motion.

Horseshoe Crabs: Living Fossils

Horseshoe crabs are members of the class Merostomata. Of the three living genera, one, Limulus, is found along North America’s East Coast, and two are found in the Asian tropics. Horseshoe crabs are considered to be living fossils; Limulus fossils have remained relatively unchanged since the **Triassic Period** about 220 million years ago.

Horseshoe crabs are heavily protected by an extensive exoskeleton and live in deep coasta waters. Shown	in Figure 28.12, they forage on sandy or muddy ocean bottoms for algae, annelids, and mollusks. These arthropods migrate to shallow water in the spring, mating at night during high tide.

Insects

Have you ever launched an ambush on a fly with your rolled-up news-paper? You swat with great accuracy and speed, yet your prey is now firmly attached upside down on the kitchen ceiling. How does a fly do this?

The fly approaches the ceiling right-side up at a steep angle. Just before impact, it reaches up with its front legs. The forelegs grip the ceiling with tiny claws and sticky hairs, while the other legs swing up into position. The flight mechanism shuts off, and the fly is safely out of swatting distance. Adaptations that enable flies to land on ceilings are among the many that make insects the most successful arthropod group. How is the ability to fly an adaptive advantage to insects? Find out in Figure 28.13 on the next page.

Flies, grasshoppers, lice, butterflies, bees, and beetles are just a few members of the class Insecta, by far the largest group of arthropods. Insects have three body segments and six legs. There are more species of insects than all other classes of animals combined.

Insect reproduction

Insects usually mate once during their lifetime. The eggs usually are fertilized internally. Some insects exhibit parthenogenesis, reproducing from unfertilized eggs. In aphids, parthenogenesis produces all-female generations. Most insects lay a large number of eggs, which increases the chances that some offspring will survive long enough to reproduce. Many female insects are equipped with an appendage that can pierce through the surface of the ground or into wood. The female lays eggs in the hole.

Metamorphosis: Change in body shape and form

After eggs are laid, the insect embryo develops and the eggs hatch. In some wingless insects, such as silverfish, development is direct; the eggs hatch into miniature forms that look just like tiny adults. These insects go through successive molts until the adult size is reached. Many other species of insects undergo a series of major changes in body structure as they develop. In some cases, the adult insect bears little resemblance to its juvenile stage. This series of changes, controlled by chemical substances in the animal, is called metamorphosis.

Insects that undergo metamorphosis usually go through four stages on their way to adulthood: egg, larva, pupa, and adult. The larva is the free-living, wormlike stage of an insect, often called a caterpillar. As the larva eats and grows, it molts several times.

The **pupa stage** of insects is a period of reorganization in which the tissues and organs of the larva are broken down and replaced by adult tissues. Usually the insect does not move or feed during the **pupa stage**. After a period of time, a fully formed adult emerges from the pupa.

The series of changes that occur as an insect goes through the egg, larva, pupa, and adult stages is known as complete metamorphosis. In winged insects that undergo complete metamorphosis, the wings do not appear until the adult stage. More than 90 percent of insects undergo complete metamorphosis. The complete metamorphosis of a butterfly is illustrated in Figure 28.14. Other insects that undergo complete metamorphosis include ants, beetles, flies, and wasps.

Complete metamorphosis is an advantage for arthropods because larvae do not compete with adults for the same food. For example, butterfly larvae (caterpillars) feed on leaves, but adult butterflies feed on nectar from flowers. You can find out how destructive a caterpillar’s diet can be in the Biology and Society feature at the end of this chapter.

Incomplete metamorphosis has three stages

Many insect species, as well as other arthropods, undergo a gradual or incomplete metamorphosis, in which the insect goes through only three stages of development.

These three stages are egg, nymph, and adult, as shown in Figure 28.15. A nymph, which hatches from an egg, has the same general appearance as the adult but is smaller. Nymphs may lack certain appendages, or have appendages not seen in adults, and they cannot reproduce. As the nymph eats and grows, it molts several times. With each molt, it begins to resemble the adult more. Wings begin to form, and an internal reproductive system develops. Gradually, the nymph becomes an adult. Grasshoppers and cockroaches are insects that undergo incomplete metamorphosis.

Origins of Arthropods

Arthropods have been enormously successful in establishing themselves over the entire surface of Earth. Their ability to survive in just about every habitat is unequaled in the animal kingdom. The success of arthropods can be attributed in part to their varied life cycles, high reproductive output, and structural adaptations, such as small size, a hard exoskeleton, and jointed appendages. How did arthropods evolve?

Arthropods most likely evolved from an ancestor of the annelids. As arthropods evolved, body segments fused and became adapted for certain functions such as locomotion, feeding, and sensing the environment. Segments in arthropods are more complex than in annelids, and arthropods have more developed nerve tissue and sensory organs, such as eyes.

The exoskeleton of arthropods provides protection for their soft bodies. Muscles in arthropods are arranged in bands associated with particular segments and portions of appendages. The circular muscles of annelids do not exist in arthropods. Because arthropods have many hard parts, much is known about their evolutionary history. The trilobites shown in Figure 28.16 were once an important group of ancient arthropods, but they have been extinct for 248 million years.

Echinoderms and Invertebrate Chordates

Echinoderms

What is an echinoderm?

Members of the phylum Echinodermata have a number of unusual characteristics that easily distinguish them from members of any other animal phylum. Echinoderms move by means of hundreds of hydraulic, suction- cup-tipped appendages and have skin covered with tiny, jawlike pincers. Echinoderms are found in all the oceans of the world.

Echinoderms have endoskeletons

If you were to examine the skin of several different echinoderms, you would find that they all have a hard, spiny, or bumpy endoskeleton covered by a thin epidermis. The long, pointed spines on a sea urchin are obvious. Sea stars, sometimes called starfishes, may not appear spiny at first glance, but a close look reveals that their long, tapering arms, called rays, are covered with short, rounded spines. The spiny skin of a sea cucumber consists of soft tissue embedded with small, platelike structures that barely resemble spines. The endoskeleton of all echinoderms is made primarily of calcium carbonate, the compound that makes up limestone. Some of the spines found on sea stars and sea urchins have become modified into pincerlike appendages called pedicellariae. An echinoderm uses its jawlike pedicellariae for protection and for cleaning the surface of its body.

Echinoderms have radial symmetry

You may remember that radial symmetry is an advantage to animals that are stationary or move slowly. Radial symmetry enables these animals to sense potential food, predators, and other aspects of their environment from all directions. Observe the radial symmetry, as well as the various sizes and shapes of spines, of each echinoderm pictured in Figure 29.1.

The water vascular system

Another characteristic unique to echinoderms is the water vascular system that enables them to move, exchange gases, capture food, and excrete wastes. The water vascular system is a hydraulic system that operates under water pressure. Water enters and leaves the water vascular system of a sea star through the madreporite, a sievelike, disk-shaped opening on the upper surface of the echinoderm’s body. This disk functions like the strainer that fits into a sink drain and keeps large particles out of the pipes.

Look at the close-up of the under-side of a sea star in Figure 29.2. You can see that tube feet run along a groove on the underside of each ray. Tube feet are hollow, thin-walled tubes that end in a suction cup. Tube feet look somewhat like miniature droppers. The round, muscular structure called the ampulla works something like the bulb of a dropper. The end of a tube foot works like a tiny suction cup. You can find out how tube feet help a sea star eat by looking at Figure 29.4 on the next page. Each tube foot works independently of the others, and the animal moves along slowly by alternately pushing out and pulling in its tube feet. You can learn more about the operation of the water vascular system in the Connection to Physics at the end of this chapter.

Tube feet also function in gas exchange and excretion. Gases are exchanged and wastes are eliminated by diffusion through the thin walls of the tube feet.

Echinoderms have varied nutrition

All echinoderms have a mouth, stomach, and intestines, but their methods of obtaining food vary. Sea stars are carnivorous and prey on worms or on mollusks such as clams. Most sea urchins are herbivores and graze on algae. Brittle stars, sea lilies, and sea cucumbers feed on dead and decaying matter that drifts down to the ocean floor.

Echinoderms have a simple nervous system

Echinoderms have no head or brain, but they do have a central nerve ring that surrounds the mouth. Nerves extend from the nerve ring down each ray. Each radial nerve then branches into a nerve net that provides sensory information to the animal. Echinoderms have cells that detect light and touch, but most do not have sensory organs. Sea stars are an exception. A sea star’s body consists of long, tapering rays that extend from the animal’s central disk. A sensory organ known as an eyespot and consisting of a cluster of light-detecting cells is located at the tip of each arm, on the underside. Eyespots enable sea stars to detect the intensity of light. Most sea stars move toward light. Sea stars also have chemical receptors on their tube feet. When a sea star detects a chemical signal from a prey animal, it moves in the direction of the arm receiving the strongest signal.

Echinoderms have bilaterally symmetrical larvae

If you examine the larval stages of echinoderms, you will find that they have bilateral symmetry. The ciliated larva that develops from the fertilized egg of an echinoderm is shown in Figure 29.3. Through metamorphosis, the free-swimming larvae make dramatic changes in both body parts and in symmetry.

Echinoderms are deuterostomes

Recall that most invertebrates show protostome development. Echinoderms are deuterostomes. This pattern of development indicates a close relationship to chordates, which are also deuterostomes.

Diversity of Echinoderms

Approximately 6000 species of echinoderms exist today. About one-fourth of these species are in the class Asteroidea, to which the sea stars belong. The five other classes of living echinoderms are Ophiuroidea, the brittle stars; Echinoidea, the sea urchins and sand dollars; Holothuroidea, the sea cucumbers; Crinoidea, the sea lilies and feather stars; and Concentricycloidea, the sea daisies. You can compare different echinoderms in the BioLab at the end of this chapter.

Sea stars

Sea stars may be the most familiar echinoderms. Most species of sea stars have five rays, but some have more. Some species may have more than 40 rays. You have already read about the characteristics of sea stars that make them a typical example of echinoderms.

Brittle stars

As their name implies, brittle stars are extremely fragile, Figure 29.5. If you try to pick up a brittle star, parts of its rays will break off in your hand. This adaptation helps the brittle star survive an attack by a predator. While the predator is busy with the broken-off ray, the brittle star can escape. A new ray will regenerate.

Brittle stars do not use their tube feet for locomotion. Instead, they propel themselves with the snake-like, slithering motion of their flexible rays. They use their tube feet to pass particles of food along the rays and into the mouth in the central disk.

Sea urchins and sand dollars

Sea urchins and sand dollars are globe-or disk-shaped animals covered with spines, as Figure 29.5 shows. They do not have rays. The circular, flat skeletons of sand dollars have a five-petaled flower pattern on the surface. A living sand dollar is covered with minute, hair-like spines that are lost when the animal dies. A sand dollar has tube feet that protrude from the petal-like markings on its upper surface. These tube feet are modified into gills and are used for respiration. Tube feet on the animal’s bottom surface aid in bringing food particles to the mouth.

Sea urchins look like living pincushions, bristling with long, usually pointed spines. The sea urchin’s spines protect it from predators. Sea urchins have long, slender tube feet that, along with the spines, aid the animal in locomotion.

Sea cucumbers

Sea cucumbers are so called because of their vegetablelike appearance, shown in Figure 29.6A. Their leathery covering allows them flexibility as they move along the ocean floor. When sea cucumbers are threatened, they may expel a tangled, sticky mass of tubes through the anus, or they may rupture, releasing some internal organs that are regenerated in a few weeks. These actions confuse their predators, giving the sea cucumber an opportunity to move away. Sea cucumbers reproduce by shedding eggs and sperm into the water, where fertilization occurs. You can find out more about sea cucumber reproduction in the Problem-Solving Lab on this page.

Sea lilies and feather stars

Sea lilies and feather stars resemble plants in some ways, as shown in Figure 29.6B. Sea lilies are the only sessile echinoderms. Feather stars are sessile only in larval form. The adult feather star uses its feathery arms to swim from place to place.

Sea daisies

Two species of sea daisies were discovered in 1986 in deep waters off New Zealand. They are flat, disk-shaped animals less than 1 cm in diameter. Their tube feet are located around the edge of the disk rather than along radial lines, as in other echinoderms.

Origins of Echinoderms

The earliest echinoderms may have been bilaterally symmetrical as adults, and probably were attached to the ocean floor by stalks. Another view of the earliest echinoderms is that they were bilateral and free-swimming. Most invertebrates show protostome development, whereas deuterostome development appears mainly in chordates. The echinoderms represent the only major group of deuterostome invertebrates. This pattern of development is one piece of evidence biologists have for placing echinoderms as the closest invertebrate relatives of the chordates.

Because the endoskeletons of echinoderms easily fossilize, there is a good record of this phylum. Echinoderms, as a group, date from the Paleozoic Era, as shown in Figure 29.7. More than 13 000 fossil species have been identified.

Invertebrate Chordates

What is an invertebrate chordate?

The chordates most familiar to you are the vertebrate chordates—chordates that have backbones, such as birds, fishes, and mammals, including humans. But the phylum Chordata includes three subphyla: Urochordata, the tunicates (sea squirts); Cephalochordata, the lancelets; and Vertebrata, the vertebrates. In this section you will examine the tunicates and lancelets—invertebrate chordates that have no backbones. You will study the vertebrate chordates in the next unit.

Invertebrate chordates may not look much like fishes, reptiles, or humans, but like all other chordates, they have a notochord, a dorsal hollow	nerve	cord, pharyngeal pouches, and a postanal tail at some time during their development. In addition, all chordates have bilateral symmetry, a well-developed coelom, and segmentation. The features shared by invertebrate and vertebrate chordates are illustrated in Figure 29.8.

All chordates have a notochord

The embryos of all chordates have a notochord—a long, semirigid, rodlike structure located between the digestive system and the dorsal hollow nerve cord. The notochord is made up of large, fluid-filled cells held within stiff, fibrous tissues. In invertebrate chordates, the notochord may be retained into adulthood. But in vertebrate chordates, this structure is replaced by a backbone. Invertebrate chordates do not develop a backbone.

The notochord develops just after the formation of a gastrula from mesoderm on what will be the dorsal side of the embryo. The notochord anchors internal muscles and enables invertebrate chordates to make rapid movements of the body. These movements propel the animal through the water at a great speed.

All chordates have a dorsal hollow nerve cord

The dorsal hollow nerve cord in chordates develops from a plate of ectoderm that rolls into a hollow tube. The sequence of development of the dorsal hollow nerve cord is illustrated in Figure 29.9. This tube is composed of cells surrounding a fluid-filled canal that lies above the notochord. In most adult chordates, the cells in the posterior portion of the dorsal hollow nerve cord develop into the spinal cord. The cells in the anterior portion develop into a brain. A pair of nerves connects the nerve cord to each block of muscles.

All chordates have pharyngeal pouches

The pharyngeal pouches of a chordate embryo are paired openings located in the pharynx, behind the mouth. Many chordates have pharyngeal pouches only during embryonic development. In aquatic chordates, pharyngeal pouches develop openings called gill slits. Food is filtered out and gas exchange occurs as water flows through gill slits. In terrestrial chordates, pharyngeal pouches develop into other structures.

All chordates have a postanal tail

At some point in development, all chordates have a postanal tail. As you know, humans are chordates, and during the early development of the human embryo, there is a postanal tail that disappears as development continues. In most animals that have tails, the digestive system extends to the tip of the tail, where the anus is located. Chordates, however, usually have a tail that extends beyond the anus.

Muscle blocks aid in movement of the tail. Muscle blocks are modified body segments that consist of stacked muscle layers. Muscle blocks are anchored by the notochord, which gives the muscles a firm structure to pull against. As a result, chordates tend to be more muscular than members of other phyla. You can observe many of the chordate traits in a lancelet in the MiniLab on the next page.

Homeotic genes control development

Homeotic genes specify body organization and direct the development of tissues and organs in an embryo. Studies of chordate homeotic genes have helped scientists understand the process of development and the relationship of invertebrate chordates to vertebrate chordates.

Diversity of Invertebrate Chordates

The invertebrate chordates belong to two subphyla of the phylum chordata: subphylum Urochordata, the tunicates, also called sea squirts, and subphylum Cephalochordata, the lancelets.

Tunicates are sea squirts

Members of the subphylum Urochordata are commonly called tunicates, or sea squirts. Although adult tunicates do not appear to have any shared chordate features, the larval stage, as shown in Figure 29.10, has a tail that makes it look similar to a tadpole. Tunicate larvae do not feed and are free swimming after hatching. They soon settle and attach themselves with a sucker to boats, rocks, and the ocean bottom. Many adult tunicates secrete a tunic, a tough sac made of cellulose, around their bodies. Colonies of tunicates sometimes secrete just one big tunic that has a common opening to the outside.

Only the gill slits in adult tunicates indicate their chordate relationship. Adult tunicates are small, tubular animals that range in size from microscopic to several centimeters long. If you remove a tunicate from its sea home, it might squirt out a jet of water—hence the name sea squirt.

Lancelets are similar to fishes

Lancelets belong to the subphylum Cephalochordata. They are small, streamlined, and common marine animals, usually about 5 cm long, as Figure 29.11 shows. They spend most of their time buried in the sand with only their heads sticking out. Like tunicates, lancelets are filter feeders. Unlike tunicates, however, lancelets retain all their chordate features throughout life.

Although lancelets look somewhat similar to fishes, they have only one layer of skin, with no pigment and no scales. Lancelets do not have a distinct head, but they do have light sensitive cells on the anterior end. They also have a hood that covers the mouth and the sensory tentacles surrounding it. The tentacles direct the water current and food particles toward the animal’s mouth.

Origins of Invertebrate Chordates

Because sea squirts and lancelets have no bones, shells, or other hard parts, their fossil record is incomplete. Biologists are not sure where sea squirts and lancelets fit in the phylogeny of chordates. According to one hypothesis, echinoderms, invertebrate chordates, and vertebrates all arose from ancestral sessile animals that fed by capturing food in tentacles. Modern vertebrates probably arose from the free-swimming **larval stages** of ancestral invertebrate chordates. Recent discoveries of fossil forms of organisms that are similar to living lancelets in rocks 550 million years old show that invertebrate chordates probably existed before vertebrate chordates.

Vertebrates

Fishes and Amphibians

Fishes

What is a fish?

Fishes, like all vertebrates, are classified in the phylum Chordata. This phylum includes three subphyla: Urochordata, the tunicates; Cephalochordata, the lancelets; and Vertebrata, the vertebrates. Fishes belong to the subphylum Vertebrata. In addition to fishes, subphylum Vertebrata includes amphibians, reptiles, birds, and mammals. Recall from the previous chapter that all chordates have four traits in common—a notochord, pharyngeal pouches, postanal tail, and a dorsal hollow nerve cord. In vertebrates, the embryo’s notochord is replaced by a backbone in adult animals. All vertebrates are bilaterally symmetrical coelomates that have endoskeletons, closed circulatory systems, nervous systems with complex brains and sense organs, and efficient respiratory systems.

Classes of fishes

Fishes can be grouped into four classes of the subphylum Vertebrata. The jawless fishes belong to the superclass Agnatha, which means “without jaws.” Superclass Agnatha consists of two classes: class Myxini, hagfishes, and class Cephalaspidomorphi, lampreys.

Class Chondrichthyes is comprised of cartilaginous fishes, such as sharks and rays; and class Osteichthyes contains the bony fishes. Examples from the different classes are shown in Figure 30.1.

Fishes inhabit nearly every type of aquatic environment on Earth. They are found in freshwater and salt water and adapted to living in shallow, warm water and deeper, cold and lightless water.

Fishes breathe using gills

Fishes have gills made up of feathery gill filaments that contain tiny blood vessels. Gills are an important adaptation for fishes and other vertebrates that live in water. As a fish takes water in through its mouth, water passes over the gills and then out through slits at the side of the fish. Oxygen and carbon dioxide are exchanged through the capillaries in the gill filaments. You can find out more about the structure and function of gills in fishes in the MiniLab on the next page.

Fishes have two-chambered hearts

All fishes have two-chambered hearts, as shown in Figure 30.2. One chamber receives deoxygenated blood from the body tissues, and the second chamber pumps blood directly to the capillaries of the gills, where oxygen is picked up and carbon dioxide released. Oxygenated blood is carried from the gills to body tissues. Blood flow through the body of a fish is relatively slow because most of the heart’s pumping action is used to push blood through the gills.

Fishes reproduce sexually

Although the method may vary, all fishes reproduce sexually. Fertilization and development is external in most fishes. Eggs and sperm can be released directly into the water, or deposited in more protected areas, such as on floating aquatic plants. Although most fishes produce large numbers of eggs at one time, hagfishes produce small numbers of relatively large eggs.

Cartilaginous fishes have internal fertilization. Skates deposit fertilized eggs on the ocean floor. Some female sharks and rays carry developing young inside their bodies. Because these young fishes are well developed when they are born, they have an increased chance of survival.

Most bony fishes have external fertilization and development. This type of external reproduction in fishes and some other animals is called spawning. During spawning, some female bony fishes, such as cod, produce as many as 9 million eggs, of which only a small percentage survive. In some bony fishes, such as guppies and mollies, fertilization and development is internal. Most fishes that pro- duce millions of eggs provide no care for their offspring after spawning. In these species, only a few of the young survive to adulthood. Some fishes, such as the mouth-brooding cichlids, stay with their young after they hatch. When their young are threatened by predators, the parent fishes scoop them into their mouths for protection.

Most fishes have paired fins

Fishes in the classes Chondrichthyes and Osteichthyes have paired fins. Fins are fan-shaped membranes that are used for balance, swimming, and steering. Fins are attached to and supported by the endoskeleton and are important in locomotion. The paired fins of fishes, illustrated in Figure 30.3, foreshadowed the development of limbs for movement on land and ultimately of wings for flying.

Fishes have developed sensory systems

All fishes have highly developed sensory systems. Cartilaginous and bony fishes have an adaptation called the lateral line system that enables them to sense objects and changes in their environment. The lateral line system is a line of fluid-filled canals running along the sides of a fish that enable it to detect movement and vibrations in the water.

Fishes have eyes that allow them to see objects and contrasts between light and dark in the water as well. The amount of vision varies greatly among fishes. Some fishes that live in areas of the ocean where there is no light may have reduced, almost non-functional eyes.

Some fishes also have an extremely sensitive sense of smell and can detect small amounts of chemicals in the water. Sharks can follow a trail of blood through the water for several hundred meters. This ability helps them locate their prey.

Most fishes have scales

Cartilaginous and bony fishes have skin covered by intermittent or overlapping rows of scales. Scales are thin bony plates formed from the skin. Scales, shown in Figure 30.4, can be toothlike, diamond-shaped, cone-shaped, or round. Shark scales are similar to teeth found in other vertebrates. The age of some species of fishes can be estimated by counting annual growth rings in their scales.

Jaws evolved in fishes

An important event in vertebrate evolution was the development of jaws in ancestral fishes. The advantage of jaws is that they enable an animal to grasp and crush its prey with great force. Jaws also allowed early fishes to prey on a greater variety of organisms. This, among other factors, explains why some early fishes were able to grow to such great size. Figure 30.5 shows the evolution of jaws in fishes.

When you think of a shark, do you imagine gaping jaws and rows of razor-sharp teeth? Sharks have up to 20 rows of teeth that are continually replaced. Their teeth point backwards to prevent prey from escaping once caught. Sharks are among the most streamlined of all fishes and are well adapted for life as predators.

Most fishes have bony skeletons

The majority of the world’s fishes belong to the class Osteichthyes, the bony fishes. Bony fishes, a successful and widely distributed class, differ greatly in habitat, size, feeding behavior, and shape. All bony fishes have skeletons made of bone rather than cartilage as found in other classes of fishes. Bone is the hard, mineralized, living tissue that makes up the endoskeleton of most vertebrates.

In general, the development of bone was important for the evolution of fishes and vertebrates. It allowed fishes to adapt to a variety of aquatic environments, as shown in Figure 30.6, and eventually to land.

Bony fishes have separate vertebrae that provide flexibility

The evolution of a backbone composed of separate, hard segments called vertebrae was significant in providing the major support structure of the vertebrate skeleton. Separate vertebrae provide great flexibility. This is especially important for fish locomotion, which involves continuous flexing of the backbone. You can see how modern bony fishes propel themselves through water in Figure 30.7. Some fishes are effective predators, in part because of the fast speeds they can attain as a result of having a flexible skeleton.

Bony fishes evolved swim bladders

Another key to the evolutionary success of bony fishes was the evolution of the swim bladder. A swim bladder is a thin-walled, internal sac found just below the backbone in most bony fishes. It can be filled with mostly oxygen or nitrogenous gases that diffuse out of a fish’s blood. A fish with a swim bladder can control its depth by regulating the amount of gas in the bladder. The gas works like the gas in a blimp that allows the blimp to change its height above the ground.

Some fishes remove gases from the swim bladder by expelling them through a special duct that attaches the swim bladder to the esophagus. In fishes that do not have this duct their swim bladders empty when gases diffuse back into the blood.

Diversity of Fishes

Fishes range in size from the tiny dwarf goby that is less than 1 cm long, to the huge whale shark that can reach a length of about 15 m—the length of two school buses.

Agnathans are jawless fishes

Lampreys and hagfishes belong to the superclass Agnatha. The skeletons of agnathans, as well as of sharks and their relatives, are made of a tough, flexible material called cartilage. Though they do not have jaws, they are voracious feeders. A hagfish, Figure 30.8, has a toothed mouth and feeds on dead or dying fishes.

It can drill a hole into a fish and suck out the blood and insides. Parasitic lampreys use their suckerlike mouths to attack other fishes. They use their sharp teeth to scrape away the flesh and then suck out the prey’s blood.

Sharks and rays are cartilaginous fishes

Sharks, skates, and rays belong to the class Chondrichthyes, shown in Figure 30.9. These fishes, like agnathans, possess skeletons composed entirely of cartilage. Because living sharks, skates, and rays are similar to species that swam the seas more than 100 000 years ago, they are considered living fossils. Sharks are perhaps the most well-known predators of the oceans.

Like sharks, most rays are predators and feed on or near the ocean floor. Rays have flat bodies and broad pectoral fins on their sides. By slowly flapping their fins up and down, rays can glide as they search for mollusks and crustaceans along the ocean floor. Some species of rays have sharp spines with poison glands on their long tails that they can use for defense. Other species of rays have organs that generate electricity, which can stun or kill both prey and predators.

Subclasses of bony fishes

Scientists recognize two subclasses of bony fishes—the lobe-finned fishes, including lungfishes, and the ray-finned fishes. Figure 30.10 shows examples of these subclasses. The lobe-finned fishes are represented by seven living species: six species of lungfishes, which have both gills and lungs, and the coelacanth, shown in Figure 30.10B. In the ray-finned fishes, such as catfish, perch, salmon, and cod, fins are fan-shaped membranes supported by stiff spines called rays.

Origins of Fishes

Scientists have identified fossils of fishes that existed during the late **Cambrian Period**, 500 million years ago. At this time, ostracoderms, early jawless fishes, were the dominant vertebrates on Earth. Most ostracoderms became extinct at the end of the **Devonian Period**, about 354 million years ago. Present-day agnathans appear to be the direct descendants of ostracoderms.

Weighed down by heavy, bony external armor, ostracoderms, shown in Figure 30.12, were fearsome-looking animals that swam sluggishly over the murky seafloor. Although all ostracoderms had cartilaginous skeletons, they also had shields of bone covering their heads and necks. The development of bone in early vertebrates was an important evolutionary step because bone provides a place for muscle attachment, which improves locomotion. In ancestral fishes, bone that formed into plates provided protection as well.

Scientists hypothesize that the jawless ostracoderms were the common ancestors of all fishes. Modern cartilaginous and bony fishes evolved during the mid-**Devonian Period**. Lobe-finned fishes, such as coelacanths, are another ancient group, appearing in the fossil record about 395 million years ago. They are characterized by lobelike, fleshy fins and live at great depths where they are difficult to find. The limblike skeletal structure of fleshy fins is thought to be an ancestral condition of all tetrapods (animals with four limbs). The earliest tetrapods discovered also had gills and therefore were still aquatic.

Amphibians

What is an amphibian?

The striking transition from a completely aquatic larva to an airbreathing, semiterrestrial adult gives the class Amphibia its name, which means “double life.” The class Amphibia includes three orders: Caudata—salamanders and newts, Anura— frogs and toads, and Apoda—legless caecilians, shown in

Figure 30.13. Amphibians have thin, moist skin and most have four legs. Although most adult amphibians are capable of a terrestrial existence, nearly all of them rely on water for reproduction. Fertilization in most amphibians is external, and water is needed as a medium for transporting sperm. Amphibian eggs lack protective membranes and shells and must be laid in water or other moist areas. Review some adaptations that allow frogs to live a “double life” in Figure 30.14 on the next page.

Amphibians are ectotherms

Amphibians are more common in regions that have warm temperatures all year because they are ectotherms. An ectotherm is an animal that has a variable body temperature and gets its heat from external sources. Because many biological processes require particular temperature ranges in order to occur, amphibians become dormant in regions that are too hot or cold for part of the year. During such times, many amphibians burrow into the mud and stay there until suitable conditions return.

Amphibians undergo metamorphosis

Unlike fishes, most amphibians go through the process of metamorphosis. Fertilized eggs hatch into tadpoles, the aquatic stage of most amphibians. You can compare tadpoles with adult frogs in the MiniLab on the next page. Tadpoles possess fins, gills, and a two-chambered heart as seen in fishes. As tadpoles grow into adult frogs and toads, they develop legs, lungs, and a three-chambered heart. Figure 30.15 shows this life cycle.

Young salamanders resemble adults, but, as aquatic larvae, they have gills and usually have a tail fin. Most adult salamanders lack gills and fins. They breathe through their moist skin or with lungs. Salamanders in the family Plethodontidae have no lungs and breathe only through their skin. Completely terrestrial salamander species do not have a **larval stage**; the young hatch as smaller versions of adults. Most salamanders have four legs for moving about, but a few have only two front legs.

Walking requires more energy

The laborious walking of early amphibians required a great deal of energy from food and large amounts of oxygen for aerobic respiration. The evolution of the three-chambered heart in amphibians ensured that cells received the proper amount of oxygen. This heart was an important evolutionary transition from the simple circulatory system of fishes.

In the three-chambered heart of amphibians, one chamber receives oxygen-rich blood from the lungs and skin, and another chamber receives oxygen-poor blood from the body tissues. Blood from both chambers then moves to the third chamber, which pumps oxygen-rich blood to body tissues and oxygen-poor blood back to the lungs and skin so it can pick up more oxygen. This results in some mixing of oxygen-rich and oxygen-poor blood in the amphibian heart and in blood vessels leading away from the heart. Thus, in amphibians, the skin is much more important than the lungs as an organ for gas exchange.

Because the skin of an amphibian must stay moist to exchange gases, most amphibians are limited to life on the water’s edge or other moist areas. Some newts and salamanders remain totally aquatic. Amphibians, such as toads, have thicker skin, and although they live primarily on land, they still must return to water to reproduce.

Amphibian Diversity

Because most amphibians still complete part of their life cycle in water, they are limited to the edges of ponds, lakes, streams, and rivers or to areas that remain damp during part of the year. Although they may not be easily seen, amphibian species are numerous worldwide.

Frogs and toads belong to the order Anura

Frogs and toads are amphibians with no tails. Frogs have long hind legs and smooth, moist skin. Toads have short legs and bumpy, dry skin. Like fishes, frogs and toads have jaws and teeth. Adult frogs and toads are predators that eat invertebrates, such as insects and worms. Many species of frogs and toads secrete chemicals through their skin as a defense against predators. You can find out more about poisonous frogs in the Connection to Chemistry at the end of this chapter.

Frogs and toads also have vocal cords that are capable of producing a wide range of sounds. Vocal cords are sound-producing bands of tissue in the throat. As air moves over the vocal cords, they vibrate and cause molecules in the air to vibrate. In many male frogs, air passes over the vocal cords, then passes into a pair of vocal sacs lying underneath the throat, as shown in Figure 30.16.

Most frogs and toads spend part of their life cycle in water and part on land. They breathe through lungs or through their thin skins. As a result, frogs and toads often are among the first organisms to be exposed to pollutants in the air, on land, or in the water. Declining numbers of frog species, or deformities in local frogs, sometimes indicate the presence of pollutants in the environment.

Salamanders belong to the order Caudata

Unlike a frog or toad, a salamander has a long, slender body with a neck and tail. Salamanders resemble lizards, but have smooth, moist skin and lack claws. Some salamanders are totally aquatic, and others live in damp places on land. They range in size from a few centimeters in length up to 1.5 m. The young hatch from eggs, look like small salamander adults, and are carnivorous.

Caecilians are limbless amphibians

Caecilians are burrowing amphibians, have no limbs, and have a short, or no, tail. Caecilians are primarily tropical animals with small eyes that often are blind. They eat earthworms and other invertebrates found in the soil. All caecilians have internal fertilization.

Origins of Amphibians

Imagine a time 360 million years ago when the inland, freshwater seas were filled with carnivorous fishes. One type of tetrapod had evolved that retained gills for breathing and a finned tail for swimming. Early tetrapods may have used their limbs to move on the bottom of marshlands filled with plants. In later fossils, the four limbs are found further below the body to lift it off the ground.

Most likely, amphibians arose as their ability to breathe air through well-developed lungs evolved. The success of inhabiting the land depended on adaptations that would provide support, protect membranes involved in respiration, and provide efficient circulation.

Challenges of life on land

Life on land held many advantages for early amphibians. There was a large food supply, shelter, and no predators. In addition, there was much more oxygen in air than in water. However, land life also held many dangers. Unlike the temperature of water, which remains fairly constant, air temperatures can vary greatly. In addition, without the support of water, the body was clumsy and heavy. Some of the efforts by early amphibians to move on land probably were like movements of present-day salamanders. The legs of salamanders are set at right angles to the body. You can see in Figure 30.17 why the bellies of these animals may have dragged on the ground.

Amphibians first appeared about 360 million years ago. Amphibians probably evolved from an aquatic tetrapod, as shown in Figure 30.18, around the middle of the Paleozoic Era. At that time, the climate on Earth is known to have become warm and wet, ideally suited for an adaptive radiation of amphibians. Able to breathe through their lungs, gills, or skin, amphibians became, for a time, the dominant vertebrates on land.

Recall that early vertebrates evolved from swimming fishes to aquatic tetrapods. Scientists have found fossil evidence that supports the hypothesis that limbs first evolved in aquatic animals. Some of these aquatic vertebrates had lungs and evolved into animals that crawled on the bottom of marshlands, and finally evolved to fully developed amphibians that could live on land. Although the fossil record for fishes and amphibians is incomplete, most scientists agree that the relationships shown in Figure 30.19 represent the best fit for the available evidence.

Reptiles and Birds

Reptiles

What is a reptile?

At first glance, it may be difficult to determine how a legless snake is related to a tortoise. Snakes, turtles, alligators, and lizards are an extremely diverse group of animals, yet all share certain traits that place them in the class Reptilia.

Early reptiles, called the stem reptiles, as shown in Figure 31.1, were the first animals to become adapted to life on land. All reptiles have adaptations that enable them to complete their life cycles entirely on land. These adaptations released the stem reptiles and other reptiles from the need to return to swamps, lakes, rivers, ponds, or oceans for reproduction.

Reptiles have scaly skin

Unlike the moist, thin skin of amphibians, reptiles have a dry, thick skin covered with scales. Scaly skin, as shown in Figure 31.2, prevents the loss of body moisture and provides additional protection from predators. Because gas exchange cannot occur through scaly skin, reptiles are dependent on lungs as their primary organ of gas exchange.

Reptiles reproduce on land

Most reptiles reproduce by laying eggs on land, as shown in Figure 31.3. Some snakes give live birth to well-developed young. Unlike amphibians, reptiles have no aquatic **larval stage**, so reptile young are not as vulnerable to water-dwelling predators.

Although all of these adaptations enabled reptiles to live successfully on land, the evolution of the amniotic egg was the adaptation that liberated reptiles from a dependence on water for reproduction. An amniotic egg provides nourishment to the embryo and contains membranes that protect it while it develops in a terrestrial environment. The egg functions as the embryo’s total life-support system.

Skeletal changes in reptiles

Look again at Figure 31.1. This early reptile had legs that were placed more directly under the body rather than at right angles to the body as in early amphibians. This positioning of the legs provides greater body support and makes walking and running on land easier for most reptiles. They have a better chance of catching prey or avoiding other predators. Reptiles that have legs also have claws that help them obtain food and protect themselves. Additional evolutionary changes in the structure of the jaws and teeth of early reptiles allowed them to use other resources and niches on land.

Some reptiles have four-chambered hearts

Most reptiles, like amphibians, have three-chambered hearts. Some reptiles, notably the crocodilians, have a four-chambered heart that completely separates the supply of blood with oxygen from blood without oxygen. The separation enables more oxygen to reach body tissues. This separation is an adaptation that supports the higher level of energy use required by land animals.

All reptiles have internal fertilization. In most cases, the eggs are laid after fertilization and embryos develop after eggs are laid. Most reptiles lay their eggs under rocks, bark, grasses, or other surface materials, but a few dig holes or collect materials for a nest. Most reptiles provide no care for hatchlings, but female crocodiles have been observed guarding their nests from predators.

Reptiles are ectotherms

Even though reptiles are different from amphibians in many ways, they are similar in one way. Both amphibians and reptiles are ectotherms. They depend on an external heat source and behavior to maintain their body temperature within the range needed to perform life functions, such as digestion. In the cool morning, a turtle might pull itself out of the pond or swamp and bask on a log in the sunlight until noon. Then, when the temperature gets a little too warm, the turtle may slip back into the cool water. Figure 31.4 shows other examples of behavioral adjustment of body temperature in reptiles.

Because reptiles are dependent on heat from the environment, they do not inhabit extremely cold regions. Reptiles are common in temperate and tropical regions, where climates are warm, and in hot desert climates. Many species of reptiles become dormant during cold periods in moderately cold environments such as in the northern United States.

How reptiles obtain food

Like other animals, reptiles have adaptations that enable them to find food and to sense the world around them. Most turtles and tortoises are too slow to be effective predators, but that doesn’t mean they go hungry. Most are herbivores, and those that are predators prey on worms and mollusks. Snapping turtles, however, are extremely aggressive, attacking fishes and amphibians, and even pulling ducklings under water.

Lizards primarily eat insects. The marine iguana of the Galápagos Islands is one of the few herbivorous lizards, feeding on marine algae. The Komodo dragon, the largest lizard, is found on several islands in Indonesia, north of Australia. It is an efficient predator, sometimes even of humans. Although lizards such as the Komodo dragon may look slow, they are capable of bursts of speed, which they use to catch their prey.

Snakes are also effective predators. Some, like the rattlesnake, have poison fangs that they use to subdue or kill their prey. A constrictor wraps its body around its prey, tightening its grip each time the prey animal exhales. Two predatory reptiles are shown in Figure 31.5.

How reptiles use their sense organs

Reptiles have a variety of sense organs that help them detect danger or potential prey. How does a rattle-snake know that you are nearby?

The heads of some snakes, as shown in Figure 31.6A, have heat-sensitive organs or pits that enable them to detect tiny variations in air temperature brought about by the presence of warm-blooded animals.

Snakes and lizards are equipped with a keen sense of smell. Have you ever seen a snake flick out its tongue? The tongue is picking up molecules in the air. The snake draws its tongue back into its mouth and moves it past or inserts it into a pitlike structure called Jacobson’s organ, described in Figure 31.6B.

Diversity of Reptiles

Gracefully gliding snakes and quickly darting lizards are grouped together in the order Squamata. Turtles, slowly plodding and carrying heavy shells, belong to the order Chelonia. Basking crocodiles and alligators, classified in the order Crocodilia, may look clumsy but are surprisingly quick hunters. Tuataras are lizardlike reptiles. They make up the fourth order of reptiles, Rhynochocephalia.

Turtles have shells

Turtles are the only reptiles protected by a shell made up of two parts. The dorsal part of the shell is the carapace, and the ventral part of the shell is the plastron. The vertebrae and expanded ribs of turtles are fused to the inside of the carapace. Most turtles have a two-layer shell—a hard, bony inner layer and an outer layer of horny keratin. In a few species, the shell is a covering made of tough, leathery skin. Most turtles can draw their limbs, tail, and head into their shells for protection against predators. Although turtles have no teeth, they do have powerful jaws with a beaklike structure that is used to crush food.

Some turtles are aquatic, and some live on land. Turtles that live on land are called tortoises. Tortoises forage for fruit, berries, and insects. The largest tortoises in the world, shown in Figure 31.7, are found on the Galápagos Islands off the coast of Ecuador.

Some adult marine turtles swim enormously long distances to lay their eggs. Like salmon, these turtles return from their feeding grounds to the place where they hatched. For example, green turtles travel from the coast of Brazil to Ascension Island in the Atlantic Ocean, a distance of more than 4000 km.

Crocodiles include the largest living reptiles

In contrast to marine turtles, crocodiles don’t migrate to reproduce. They may spend their days alternately basking in the sun on a river-bank and floating like motionless logs. Only their eyes and nostrils remain above water. Crocodiles can be identified by their long, slender snouts, whereas alligators have short, broad snouts. Both animals have powerful jaws with sharp teeth that can drag prey underwater and hold it there until it drowns. Another feature that makes these animals efficient predators is that, unlike other reptiles, they can continue to breathe air with their mouths full of food and water. The American alligator is found throughout many of the fresh-water habitats of the southeastern United States. The American crocodile can be found only in salt water and estuarine habitats in southern Florida. The American alligator can reach a length of 5 m. Other crocodilians, such as the Nile crocodile of Africa, can grow even longer.

Both alligators and crocodiles lay eggs in nests on the ground. Unlike other reptiles, these animals stay close to their nests and guard them from predators. Several crocodilian species have been observed holding their newly hatched offspring gently in their mouths as they carried them to the safety of the water.

Snakes and lizards have diverse forms

Lizards, like the ones shown in Figure 31.8, are found in many types of habitats throughout the world. Some live on the ground; some burrow; some live in trees; and some are aquatic. Many are adapted to hot, dry climates. Although a few species of lizards are limbless, most lizards have four legs.

Snakes, in contrast to most vertebrates, have no limbs and lack the bones to support limbs. Exceptions are pythons and boas, which retain bones of the pelvis. The many vertebrae of snakes permit fast undulations through grass and over rough terrain. Some snakes even swim and climb trees.

Snakes usually kill their prey in one of three ways. Remember that constrictors wrap themselves around their prey. Common constrictors include boas, pythons, and anacondas. Venomous snakes use poison to paralyze or kill their prey. These include rattlesnakes, cobras, and vipers, which inject poison from venom glands.

Most snakes are neither constrictors nor poisonous. They get food by grabbing it with their mouths and swallowing it whole. Snakes eat rodents, amphibians, insects, fishes, eggs, and other reptiles, as shown in Figure 31.9.

The fourth order of reptiles, Rhynchocephalia, is represented by two living species of tuatara, one of which is shown in Figure 31.10. Tuataras are the only survivors of a primitive group of reptiles, most of which died out 100 million years ago.

Origins of Reptiles

You may have marveled at dinosaurs ever since you were very young. These animals were the most numerous land vertebrates during the Mesozoic Era. Some were the size of chickens, and others were the largest land dwellers that ever lived. Learn more about dinosaurs on pages 1084–1085 in the Focus On.

The ancestors of snakes and lizards are traced to a group of early reptiles, called scaly reptiles, that branched off from the ancient stem reptiles. The name “scaly reptiles” may be misleading because it implies that other reptiles lacked scales—which is not true. Although the evolutionary history of turtles is incomplete, scientists have suggested that they may also be descendants of stem reptiles. Dinosaurs and crocodiles are the third group to descend from stem reptiles, as you can see in Figure 31.11.

Although scientists used to think that birds arose as a separate group from this third branch, there is now much fossil evidence that leads biologists to suggest that birds are the living descendants of the dinosaurs.

Birds

What is a bird?

After conquering the sea and land, vertebrates took to the air, where there was a huge source of insect food and a refuge from land-dwelling predators. The existence of more than 8600 species of modern birds, class Aves, shows that flight was a successful adaptation for survival. Birds inhabit a variety of environments around the world, including Antarctica, deserts, and tropical rain forests.

Biologists sometimes refer to birds as feathered dinosaurs. Fossil evidence seems to indicate that birds have evolved from small, two-legged dinosaurs called theropods, illustrated in Figure 31.12. Like reptiles, birds have clawed toes and protein scales on their feet. Fertilization is internal and shelled amniotic eggs are produced in both groups. Although some birds are flightless, all birds have feathers and wings.

Birds have feathers

A feather, shown in Figure 31.13, is a lightweight, modified protein scale that provides insulation and enables flight. You may have seen a bird running its bill or beak through its feathers while sitting on a tree branch or on the shore of a pond. This process, called preening, keeps the feathers in good condition for flight. During preening, a bird also uses its bill or beak to rub oil from a gland near the tail onto the feathers. This is especially important for water birds as a way to waterproof the feathers. You can compare types of bird feathers in the MiniLab on this page.

Even with good care, feathers wear out and must be replaced. The shedding of old feathers and the growth of new ones is called molting. Most birds molt in late summer. However, most do not lose their feathers all at once and are able to fly while they are molting. Wing and tail feathers are usually lost in pairs so that the bird can maintain its balance in flight.

Birds have wings

A second adaptation for flight in birds is the modification of the front limbs into wings. Powerful flight muscles are attached to a large breastbone called the sternum and to the upper bone of each wing. The sternum looks like the keel of a sailing boat and is important because it supports the enormous thrust and power produced by the muscles as they move to generate the lift needed for flight.

Flight requires energy

Flight requires high levels of energy. Several factors are involved in maintaining these high energy levels. First, a bird’s four-chambered, rapidly beating heart moves oxygenated blood quickly throughout the body. While sleeping, a chickadee’s heart, for example, beats 500 times a minute. Compare this to an average human heart, which beats 70 times a minute. This efficient circulation supplies cells with the oxygen needed to produce energy.

Second, a bird’s respiratory system supplies oxygenated air to the lungs when it inhales as well as when it exhales. A bird’s respiratory system consists of lungs and anterior and posterior air sacs. You can see the path air follows in a bird’s respiratory system in Figure 31.14. During inhalation, oxygenated air passes through the trachea and into the lungs, where gas exchange occurs. Most of the air, however, passes directly into the posterior air sacs. When a bird exhales deoxygenated air from the lungs, oxygenated air returns to the lungs from the posterior air sacs. At the next inhalation, deoxygenated air in the lungs passes into the anterior air sacs. Finally, at the next exhalation, air passes from the anterior air sacs out of the trachea. Thus, air follows a one-way path in a bird. Find out more about bird flight in Figure 31.15.

Birds are endotherms

Birds are able to maintain the high energy levels needed for flight because they are endotherms. An endotherm is an animal that maintains a nearly constant body temperature that is not dependent on the environmental temperature.

Birds have a variety of ways to save or give off their body heat in order to maintain a nearly constant body temperature. Feathers reduce heat loss in cold temperatures. The feathers fluff up and trap a layer of air that limits the amount of heat lost. Responses to high temperatures include flattening the feathers and holding the wings away from the body. Birds also pant to increase respiratory heat loss.

A major advantage of being endothermic is that birds can live in all environments, from the hot tropics to the frigid Antarctic. However, birds and other endotherms must eat large amounts of food to sustain these higher levels of energy.

Reproduction in birds

Birds, like reptiles, reproduce by internal fertilization and lay amniotic eggs usually inside a nest. Bird eggs are encased in a hard shell, unlike the leathery shell of a reptile. Bird nests may be made out of bits of straw and twigs, may consist of a depression scratched into the sand, or may be elaborate structures that are added to yearly. Whatever the type of nest, birds do not leave the eggs to hatch on their own. Instead, birds incubate or sit on their eggs to keep them warm. The eggs are turned periodically so that they develop properly.

In some species of birds, both parents take turns incubating eggs; in others, only one parent does so. Bird eggs are distinctive, and often the species of bird can be identified just by the color, size, and shape of an egg.

Diversity of Birds

Unlike reptiles, which take on a wide variety of forms from legless snakes to shelled turtles, birds are all very much alike in their basic form and structure. You have no difficulty recognizing a bird.

In spite of the basic uniformity of birds, they do exhibit specific adaptations, depending on the environment in which they live and the food they eat. As shown in Figure 31.16, ptarmigans have feathered legs and feet that serve as snowshoes in the winter, making it easier for the birds to walk in the snow. Penguins are flightless birds with wings and feet modified for swimming and a body surrounded with a thick layer of insulating fat. Large eyes, an acute sense of hearing, and sharp claws make owls well-adapted, nocturnal predators able to swoop with absolute precision onto their prey.

The shape of a bird’s beak or bill gives clues to the kind of food the bird eats. Hummingbirds, for example, have long beaks that are used for obtaining nectar from flowers. Hawks, like the one shown in Figure 31.17, have curved beaks that are adapted for tearing apart their prey. Pelicans have huge bills with pouches that they use as nets for capturing fish. The short, stout beak of a cardinal is adapted to cracking seeds. Many bird species are now threatened with extinction due to changes in their habitats.

Origins of Birds

Current thoughts about bird evolution are illustrated in Figure 31.18. Scientists hypothesize that today’s birds are derived from an evolutionary line of dinosaurs that did not become extinct. Figure 31.19 shows the earliest known bird in the fossil record, Archaeopteryx. At first, scientists thought that Archaeopteryx was a direct ancestor of modern birds; however, some paleontologists now think that it most likely did not give rise to any other bird groups. Archaeopteryx was about the size of a crow and had feathers and wings like a modern bird. But it also had teeth, a long tail, and clawed front toes, much like a reptile.

Fossil finds in China support the idea that birds evolved from a theropod dinosaur. As illustrated in Figure 31.19, it was flightless and ran to capture prey. It was about 1 m tall and had feathers similar to those of modern birds. Scientists hypothesize that their feathers helped insulate the animal, or perhaps were adapted for camouflage or courtship behavior. Most scientists studying the origins of birds hypothesize that feathers evolved before flight.

But feathers aren’t the only features shared by modern birds and some theropod dinosaurs. Both also have a sternum, a wishbone, shoulder blades, flexible wrists, and three fingers on each hand.

Mammals

Mammal Characteristics

What is a mammal?

Mammals, like birds, are endotherms. The ability to maintain a fairly constant body temperature enables mammals to live in almost every possible environment on Earth. Mammals have important characteristics not found in other animals. They have hair and produce milk to nurse their young. Mammals also have diaphragms, four-chambered hearts, specialized teeth, modified limbs, and highly developed brains.

Mammals have hair

Have you ever heard someone complain about a pet that is shedding its hair? There’s no doubt that such a pet is a mammal because hair is present on all mammals at some point in their lives. Like feathers, mammalian hair, made out of the protein keratin, is also thought to have evolved from scales. The arrangement of hair provides insulation and waterproofing and thereby conserves body heat. If you have ever worn a wool sweater made from the hair of a sheep, you know how warm wool can be on a cold day. As shown in Figure 32.1 on the next page, hair also serves other functions.

Although hair helps retain body heat, mammals also have **internal feedback mechanisms** that signal the body to cool off when it gets too warm. Mammals cool off by panting and through the action of sweat glands.

Panting releases water from the nose and mouth, which results in a loss of body heat. Sweat glands help regulate body temperature by secreting moisture onto the surface of the skin. As the moisture evaporates, it transfers heat from the body to the surrounding air.

Mammals nurse their young

Mammals have several types of glands, which are a group of cells that secrete fluids. They include glands that produce saliva, sweat, oil, digestive enzymes, hormones, milk, and scent. You have already learned how sweat glands help keep a mammal cool.

Mammals also feed their young from mammary glands, possibly modified sweat glands, which produce and secrete milk, a liquid that is rich in fats, sugars, proteins, minerals, and vitamins. Mammals nurse their young until they are able to digest and absorb nutrients from solid foods. Figure 32.2 shows that the number of young each mother has and the length of time she nurses her young vary among species.

Respiration and circulation in mammals

Mammals need a high level of energy to maintain their endothermic metabolism. This energy level is sustained when large amounts of nutrients and oxygen enter the body and reach the cells.

The mammals’ diaphragm helps expand the chest cavity to aid the flow of oxygen into their lungs. A diaphragm is the sheet of muscle located beneath the lungs that separates the chest cavity from the abdominal cavity, where other organs are located. Once in the lungs, oxygen diffuses into the blood. As the chest cavity returns to its resting position, air is released.

Mammals, like birds, have four-chambered hearts in which oxygenated blood is kept entirely separate from deoxygenated blood. This ensures that a good supply of nutrients and oxygen are delivered to cells which supports their endothermic metabolism. Circulation also removes waste products from cells and helps regulate body temperature. Blood helps keep a constant cellular environment, which maintains homeostasis.

Most mammals have specialized teeth

Teeth are a distinguishing feature of most mammals. Although fishes and reptiles have teeth, their teeth are relatively uniform and are used primarily for tearing, grasping, and holding prey.

Mammals with teeth have different kinds that are adapted to the type of food the animal eats. Think of the different tools you might use to build a piece of furniture, such as a chisel for scraping or a saw for cutting. Like a cabinetmaker’s tools, teeth are shaped to match the types of jobs they do.

The pointed incisors of moles grasp and hold small prey. The chisel-like incisors of beavers are modified for gnawing. A lion’s canines puncture and tear the flesh of its prey. Premolars and molars are used for slicing or shearing, crushing, and grinding. You can get a closer look at mammalian teeth in the MiniLab on this page. By examining the teeth of a mammal, a scientist can determine what kind of food it eats.

Many hoofed mammals have an adaptation called cud chewing that enables the cellulose in plant cell walls to be broken down into nutrients they can absorb and use.

Have you ever seen cows slowly chewing while lying in a pasture? When plant material is swallowed, it moves into the first two of four pouches in the stomach where cellulose in the cell walls is broken down by bacteria. The partially digested food, called cud, is repeatedly brought back up into the mouth. After more chewing, the cud is swallowed again and when the food particles are small enough they are passed to the other stomach areas, where digestion continues. You can learn more about digestion in mammals in the Problem-Solving Lab on this page. The various types of teeth in different mammals can be seen in Figure 32.3.

Mammals have modified limbs

Mammals have several adaptations that help them meet their energy needs. For example, mammal limbs are adapted for a variety of methods of food gathering. Recall that primates use their opposable thumb to grasp objects—including fruits and other foods. Figure 32.4 illustrates other limb modifications in mammals. You can see the bones of mammal limbs and other parts of mammalian skeletons in the MiniLab on this page. Refer to Figure 32.5 on the next page for a summary of mammalian characteristics.

Mammals can learn

One reason mammals are successful is that they guard their young fiercely and teach them survival skills. Mammals can accomplish complex behaviors, such as learning and remembering what they have learned.

Diversity of Mammals

Mammal Classification

Living in the United States, you are probably familiar with only one of the three subclasses, of the class Mammalia— placental mammals. Scientists place mammals into one of three subclasses based on their method of reproduction.

Placental mammals: A great success

Placental mammals give birth to young that have developed inside the mother’s uterus until their body systems are fully functional and they can live independently of their mother’s body. The uterus is a hollow, muscular organ in which offspring develop. Nourishment of the young inside the uterus occurs through an organ called the placenta, which develops during pregnancy. The placenta passes nutrients and oxygen to and removes wastes from the developing embryo. The time during which placental mammals develop inside the uterus is called gestation. The kits shown in Figure 32.7 were born after a period of gestation.

Development inside the mother’s body is an adaptation that played a major role in the success of mammals. It ensures that the offspring are protected from predators and the environment during the early stages of development.

About 90 percent of all mammals are placentals. You can learn more about placental mammals on pages 1086–1089 in the Focus On.

Pouched mammals:

The marsupials

Marsupials make up the second subclass of mammals. A marsupial is a mammal in which the young have a short period of development within the mother’s body, followed by a period of development inside a pouch made of skin and hair on the outside of the mother’s body. You may have seen the only North American marsupial, the opossum. Most marsupials are found in Australia and surrounding islands. **The theory of plate tectonics** explains why most marsupials are found in Australia today. Scientists have found fossil marsupials on the continents that once made up Gondwana. These fossils support the idea that marsupials originated in South America, moved across Antarctica, and populated Australia before Gondwana broke up.

Ancestors of today’s marsupials were able to populate the landmass that became Australia without having to share the area with the competitive placental mammals that evolved in other places. They successfully spread out and filled niches similar to those that placental mammals filled in all other parts of the world, as you can see in Figure 32.8. In fact, since humans introduced sheep, rabbits, and other placental mammals to Australia, many of the native marsupial species have become threatened, endangered, or even extinct.

Monotremes: The egg layers

Do you think the animal shown in Figure 32.9B is a mammal? It has hair and mammary glands, yet it lays eggs. The duck-billed platypus is a monotreme, a mammal that reproduces by laying eggs. Spiny anteaters, also called echidnas, belong to this subclass as well. Monotremes are found only in Australia, Tasmania, and New Guinea. One of the two species of spiny anteaters can be found only in New Guinea. Only three species of monotremes are alive today.

The platypus, a mostly aquatic animal, has a broad, flat tail, much like that of a beaver. Its rubbery snout resembles the bill of a duck. The platypus has webbed front feet for swimming through water, but it also has sharp claws on its front and hind feet for digging and burrowing into the soil. Much of its body is covered with thick, brown fur.

The spiny anteater has coarse, brown hair, and its back and sides are covered with sharp spines that it can erect for defensive purposes when threatened by enemies. From its mouth, the anteater extends its long, sticky tongue to catch insects.

Origins of Mammals

Present-day mammal orders are shown in Figure 32.10. The first placental mammals appeared in the fossil record about 125 million years ago. Scientists trace the origins of placental mammals from a group of mouse-sized animals, such as Eomaia represented in Figure 32.11A, to a group of reptilian ancestors called therapsids. Therapsids, represented in Figure 32.11B, had features of both reptiles and mammals. They existed between 270 and 180 million years ago. The mass extinction of the dinosaurs at the end of the Mesozoic Era, along with the breaking apart of Pangaea and changes in climate, opened up new niches for early mammals to fill. The appearance of flowering plants at the end of this era supplied new living areas, food sources, and shelter. Some mammals that moved into the drier grasslands became fast-running grazers, browsers, and predators. The Cenozoic Era is sometimes called the golden age of mammals because of the dramatic increase in their numbers and diversity.

Animal Behavior

Innate Behavior

What is behavior?

A peacock displaying his colorful tail, a whale spending the winter months in the ocean off the coast of southern California, and a lizard seeking shade from the hot desert sun are all examples of animal behavior. Behavior is anything an animal does in response to a stimulus. A stimulus is an environmental change that directly influences the activity of an organism. The presence of a peahen stimulates a peacock to open its tail feathers and strut. Environmental cues, such as a change in day length, might be the stimulus that causes the whale to leave its summertime arctic habitat. Heat stimulates the lizard to seek shade. Figure 33.1 shows two examples of stimuli that affect animal behavior.

Animals carry on many activities— such as getting food, avoiding predators, caring for young, finding shelter, and attracting mates—that enable them to survive and reproduce. These behavior patterns, therefore, have adaptive value. For example, a parent gull that is not incubating eggs or caring for chicks joins a noisy flock of gulls to dive for fishes. If the parent cannot catch a lot of fishes, not only will it die, but its chicks will not survive either. Therefore, this feeding behavior has adaptive value for the gull.

Inherited Behavior

Inheritance plays an important role in the ways animals behave. You don’t expect a hummingbird to tunnel underground or a mouse to fly. Yet, why does a mouse run away when a cat appears? Why does a humming bird fly south for the winter? These behavior patterns are genetically programmed. An animal’s genetic make-up determines how that animal reacts to certain stimuli.

Natural selection favors certain behaviors

Often, a behavior exhibited by an animal species is the result of natural selection. The variability of behavior among individuals affects their ability to survive and reproduce. Individuals with behavior that makes them more successful at surviving and reproducing tend to produce more offspring than individuals without the behavior. These offspring will inherit the genetic basis for the successful behavior.

Inherited behavior of animals is called innate behavior. A toad captures prey by flipping out its sticky tongue. To capture prey, a toad must first be able to detect and follow its movement. Toads have “insect detector” cells in the retinas of their eyes. As an insect moves across a toad’s line of sight, the “insect detector” cells signal the brain of the prey’s changing position, thus initiating an innate response; the toad’s tongue flips out. Figure 33.2 shows a toad that captured its prey using an innate behavior known as a fixed-action pattern. A fixed-action pattern is an unchangeable behavior pattern that, once initiated, continues until completed.

Genes form the basis of innate behavior

Through experiments, scientists have found that an animal’s hormonal balance and its nervous system—especially the sense organs responsible for sight, touch, sound, or odor identification—affect how sensitive the individual is to certain stimuli. In fire ant colonies, a single gene influences the acceptance or rejection of the ant queen, thereby controlling the colony’s social structure. Innate behavior includes fixed-action patterns, automatic responses, and instincts. You can observe the response of animals to certain stimuli in the BioLab at the end of this chapter.

Automatic Responses

What happens if something quickly passes in front of your eyes or if something is thrown at your face? Your first reaction is to blink and jerk back your head. Even if a protective clear shield is placed in front of you, you can’t stop yourself from behaving this way when the object is thrown. This reaction is an example of the simplest form of innate behavior, called a reflex. A reflex is a simple, automatic response to a stimulus that involves no conscious control. Figure 33.3 shows an example of a reflex.

The adaptive value of another automatic response is obvious. Think about a time when you were suddenly scared. Immediately, your heart began to beat faster. Your skin got cold and clammy, your respiration increased, and maybe you trembled. You were having a fight-or-flight response. A fight-or-flight response mobilizes the body for greater activity. Your body is being prepared to either fight or run from the danger. A fight-or-flight response is automatic and controlled by hormones and the nervous system.

Instinctive Behavior

Compare the fixed-action pattern of a toad capturing prey with a fight- or-flight response. Both are quick, automatic responses to stimuli. But some behaviors take a longer time because they involve more complex actions. An instinct is a complex pattern of innate behavior. Instinctive behavior begins when the animal recognizes a stimulus and continues until all parts of the behavior have been performed.

As shown in Figure 33.4, greylag geese instinctively retrieve eggs that have rolled from the nest. They will go through the motions of egg retrieval even if the eggs roll or are taken away until they are comfortably back on their nest. If they see the egg has not been retrieved they begin the process again.

Courtship behavior ensures reproduction

Much of an animal’s courtship behavior is instinctive. Courtship behavior is the behavior that males and females of a species carry out before mating. Like other instinctive behaviors, courtship has evolved through natural selection. Imagine what would happen to the survival of a species if members were unable to recognize other members of that same species. Individuals often can recognize one another by the behavior patterns each performs. In courtship, behavior ensures that members of the same species find each other and mate. Obviously, such behavior has an adaptive value for the species. Different species of fireflies, for example, can be seen at dusk flashing distinct light patterns. However, female fireflies of one species respond only to those males exhibiting the species-correct flashing pattern.

Some courtship behaviors help prevent females from killing males before they have had the opportunity to mate. For example, in some spiders, the male is smaller than the female and risks the chance of being eaten if he approaches her. Before mating, the male in some species presents the female with an object, such as an insect wrapped in a silk web. While the female is unwrapping and eating the insect, the male is able to mate with her without being attacked. After mating, however, the male may be eaten by the female anyway.

In some species, such objects play an important role in allowing the female to exercise a choice as to which male to choose for a mating partner. The hanging fly, shown in Figure 33.5, is such a species.

Territoriality reduces competition

You may have seen a chipmunk chase another chipmunk away from seeds on the ground under a bird feeder. The chipmunk was defending its territory. A territory is a physical space an animal defends against other members of its species. It may contain the animal’s breeding area, feeding area, and potential mates, or all three. Animals that have territories will defend their space by driving away other individuals of the same species. For example, a male sea lion patrols the area of beach where his harem of female sea lions rests. He does not bother a neighboring male that has a harem of his own because both have marked their territories, and each respects the common boundaries. But if an unattached, young male tries to enter the sea lion’s territory, the owner of the territory will attack and drive the intruder away from his harem.

Although it may not appear so, setting up territories actually reduces conflicts, controls population growth, and provides for efficient use of environmental resources. When animals space themselves out, they don’t compete for the same resources within a limited space. This behavior improves the chances of survival of the young, and, therefore, survival of the species. If the male has selected an appropriate site and the young survive, they may inherit his ability to select an appropriate territory. Therefore, territorial behavior has survival value, not only for individuals, but also for the species. The male stickleback shown in Figure 33.6 is another animal that exhibits territoriality, especially during breeding season.

Recall that pheromones are chemicals that communicate information among individuals of the same species. Many animals produce pheromones to mark territorial boundaries. For example, wolf urine contains pheromones that warn other wolves to stay away. The male prong-horn antelope uses a pheromone secreted from facial glands. One advantage of using pheromones is that they work both day and night, and whether or not the animal that made the mark is present.

Aggressive behavior threatens other animals

Animals occasionally engage in aggression. Aggressive behavior is used to intimidate another animal of the same species. Animals fight or threaten one another in order to defend their young, their territory, or a resource such as food. Aggressive behaviors, such as bird calling, teeth baring, or growling, deliver the message to keep away.

When a male bighorn sheep is threatened by another male moving into his territory, for example, he does not kill the invader. Animals of the same species rarely fight to the death. The fights are usually symbolic, as shown in Figure 33.7. Male bighorn sheep do not usually even injure one another. Why does aggressive behavior rarely result in serious injury? One answer is that the defeated individual shows signs of submission to the victor. These signs inhibit further aggression by the victor. Continued fighting might result in serious injury for the victor; thus, its best interests are served by stopping the fight.

Submission leads to dominance hierarchies

Do you have an older or younger sibling? Who wins when you argue? In animals, usually the oldest or strongest wins the argument. But what happens when several individuals are involved in the argument? Sometimes, aggressive behavior among several individuals results in a grouping in which there are different levels of dominant and submissive animals. A dominance hierarchy is a form of social ranking within a group in which some individuals are more subordinate than others. Usually, one animal is the top-ranking, dominant individual. This animal might lead others to food, water, and shelter. A dominant male often sires most or all of the offspring. There might be several levels in the hierarchy, with individuals in each level subordinate to the one above. The ability to form a dominance hierarchy is innate, but the position each animal assumes may be learned.

The term pecking order comes from a dominance hierarchy that is formed by chickens. The top-ranking chicken can peck any other chicken. The chicken lowest in the hierarchy is pecked at by all the other chickens in the group.

Behavior resulting from internal and external cues

Some instinctive behavior is exhibited in animals in response to internal, biological rhythms. Behavior based on a 24-hour day/night cycle is one example. Many animals, humans included, sleep at night and are awake during the day. Other animals, such as owls, reverse this pattern and are awake at night. A 24-hour, light-regulated, sleep/wake cycle of behavior is called a circadian rhythm. Circadian rhythms keep you alert during the day and help you relax at night. They may even wake you if you forget to set your alarm clock or on days that you could sleep in. Circadian rhythms are controlled by genes, yet are also influenced by factors such as jet lag and shift work.

Rhythms also can occur on a yearly or seasonal cycle. Migration, for example, occurs on a seasonal cycle. Migration is the instinctive, seasonal movement of animals, shown in Figure 33.8. In North America, about two-thirds of bird species fly south in the fall to areas such as South America, where food is available during the winter. The birds fly north in the spring to areas where they breed during the summer. Whales migrate seasonally, as well. Change in day length is thought to stimulate the onset of migration in the same way that it controls the flowering of plants. You can find out how migrating turtles are tracked in the Biotechnology at the end of this chapter.

Migration calls for remarkable strength and endurance. The arctic tern migrates between the arctic circle and the Antarctic, a one-way flight of almost 18 000 km.

Animals navigate in a variety of ways. Some use the positions of the sun and stars to navigate. They may use geographic clues, such as mountain ranges. Some bird species seem to be guided by Earth’s magnetic field. You might think of this as being guided by an internal compass.

Animals that migrate might be responding to colder temperatures and shorter days, as well as to hormones. Young animals may learn when and where to migrate by following their parents. You can easily see why animals migrate from a cold place to a warmer place, yet most animals do not migrate. How many animals cope with winter is another example of instinctive behavior.

You know that many animals store food in burrows and nests. But other animals survive the winter by undergoing physiological changes that reduce their need for energy. Some mammals, such as bats and chipmunks, and a few other types of animals go into a deep sleep during parts of the cold winter months. This period of inactivity is called hibernation. Hibernation is a state in which the body temperature drops substantially, oxygen consumption decreases, and breathing rates decline to a few breaths per minute. Hibernation conserves energy. Animals that hibernate typically eat vast amounts of food to build up body fat before entering hibernation. This fat fuels the animal’s body while it is in this state. The golden-mantled ground squirrel shown in Figure 33.9 is an example of an animal that hibernates. You can find out more about hibernation in the Problem- Solving Lab on the next page.

What happens to animals that live year-round in hot environments? Some of these animals respond in a way that is similar to hibernation. Estivation is a state of reduced metabolism that occurs in animals living in conditions of intense heat. Desert animals appear to estivate sometimes in response to lack of food or periods of drought.

Learned Behavior

What is learned behavior?

Learning, or learned behavior, takes place when behavior changes through practice or experience. The more complex an animal’s brain, the more elaborate the patterns of its learned behavior. As you can see in Figure 33.11, innate behaviors are more common in invertebrates, and learned behaviors are more common in vertebrates. In humans, many behaviors are learned.

Learning has survival value for all animals in changing environments because it permits behavior to change in response to varied conditions. Learning allows an animal to adapt to change, an ability that is especially important for animals with long life spans. The longer that an animal lives, the greater the chance that its environment will change.

Kinds of Learned Behavior

Just as there are several types of innate behavior, there are several types of learned behavior. Some learned behavior is simple and some is complex. Which group of animals do you think carries out the most complex type of learned behavior?

Habituation: A simple form of learning

Horses normally shy away from an object that suddenly appears from the trees or bushes, yet after a while they disregard noisy cars that speed by the pasture honking their horns. This lack of response is called habituation. Habituation illustrated in Figure 33.12, occurs when an animal is repeatedly given a stimulus that is not associated with any punishment or reward. An animal has become habituated to a stimulus when it finally ceases to respond to the stimulus.

Imprinting:

A permanent attachment

Have you ever seen young ducklings following their mother? This behavior is the result of imprinting. Imprinting is a form of learning in which an animal, at a specific critical time of its life, forms a social attachment to another object. Many kinds of birds and mammals do not innately know how to recognize members of their own species. Instead, they learn to make this distinction early in life. Imprinting takes place only during a specific period of time in the animal’s life and is usually irreversible. For example, birds that leave the nest immediately after hatching, such as geese, imprint on their mother. They learn to recognize and follow her within a day of hatching.

In birds	such as	ducks, imprinting takes place during the first day or two after hatching. A duckling rapidly learns to recognize and follow the first conspicuous moving object it sees. Normally, that object is the duckling’s mother. Learning to recognize their mother and follow her ensures that food and protection will always be nearby.

Learning by trial and error

Do you remember when you first learned how to ride a bicycle? You probably tried many times before being able to successfully complete the task. Nest building, like riding a bicycle, may be a learning experience. The first time a jackdaw builds a nest, it uses grass, bits of glass, stones, empty cans, old lightbulbs, and anything else it can find. With experience, the bird finds that grasses and twigs make a better nest than do lightbulbs. The jackdaw has used trial-and-error learning in which an animal receives a reward for making a particular response. When an animal tries one solution and then another in the course of obtaining a reward, in this case a suitable nest, it is learning by trial and error.

Learning happens more quickly if there is a reason to learn or be successful. Motivation is an internal need that causes an animal to act, and it is necessary for learning to take place. In most animals, motivation often involves satisfying a physical need, such as hunger or thirst. If an animal isn’t motivated, it won’t learn. Animals that aren’t hungry won’t respond to a food reward. Mice living in a barn, shown in Figure 33.13, discover that they can eat all the grain they like if they first chew through the container in which the grain is stored.

Classical conditioning: Learning by association

Suppose that when you first got a new kitten, it would meow as soon as it smelled the aroma of cat food in the can you were opening. After a few weeks, the sound of the can opener alone attracted your kitten, causing it to meow. Your kitten had become conditioned to respond to a stimulus other than the smell of food. Classical conditioning is learning by association. A well-known example of an early experiment in classical conditioning is illustrated in Figure 33.14.

Insight: The most complex type of learning

In a classic study of animal behavior, a chimpanzee was given two bamboo poles, neither of which was long enough to reach some fruit placed outside its cage. By connecting the two tapering short pieces to make one longer pole, the chimpanzee learned to solve the problem of how to reach the fruit. This type of learning is called insight. Insight is learning in which an animal uses previous experience to respond to a new situation.

Much of human learning is based on insight. When you were a baby, you learned a great deal by trial and error. As you grew older, you relied more on insight. Solving math problems is a daily instance of using insight. Probably your first experience with mathematics was when you learned to count. Based on your concept of numbers, you then learned to add, subtract, multiply, and divide. Years later, you continue to solve problems in mathematics based on your past experiences. When you encounter a problem you have never experienced before, you solve the problem through insight.

The Role of Communication

When you think about interactions among animals as a result of their behavior, you realize that some sort of communication has taken place. Communication is an exchange of information that results in a change of behavior. Black-headed gulls visually communicate their availability for mating with instinctive courtship behavior. The pat on the head from a dog’s owner after the dog retrieves a stick signals a job well done.

Most animals communicate

Animals have several channels of communication open to them. They signal each other by sounds, sights, touches, or smells. Sounds vibrate in all directions and can be heard a long way from their sources. Sounds such as songs, roars, and calls communicate a lot of information quickly. For example, the song of a male cricket tells his sex, his location, his social status, and, because communication by sound is usually species specific, his species.

Signals that involve odors may be broadcast widely and carry a general message. Ants, shown in Figure 33.15, leave odor trails that are followed by other members of their nest. These odors are species specific. As you know, pheromones, such as those of moths, may be used to attract mates. Because only small amounts of pheromones are needed, other animals, especially predators, may be unable to detect the odor.

Using both innate and learned behavior

Some communication is a combination of both innate and learned behavior. In some species of songbirds, such as the one shown in Figure 33.16, males automatically sing when they reach sexual maturity. Their songs are specific to their species, and singing is innate behavior. Yet members of the same species that live in different regions learn different variations of the song. They learn to sing with a regional dialect. In other species, birds raised in isolation never learn to sing their species song.

Some animals use language

Language, the use of symbols to represent ideas, is present primarily in animals with complex nervous systems, memory, and insight. Humans, with the help of spoken and written language, can benefit from what other people and cultures have learned and don’t have to experience everything for themselves. People can use the accumulated knowledge in the books shown in Figure 33.17 to build new knowledge.

The Human Body

Protection, Support, and Locomotion

Skin: The Body’s Protection

Structure and Functions of the Integumentary System

Skin, the main organ of the integumentary system, is composed of layers of the four types of body tissues: epithelial, connective, muscle, and nervous. Epithelial tissue, found in the outer layer of the skin, functions to cover surfaces of the body. Connective tissue, which consists of both tough and flexible protein fibers, serves as a sort of organic glue, holding your body together. Muscle tissues interact with hairs on the skin to respond to stimuli, such as cold and fright. Nervous tissue helps us detect external stimuli, such as pain or pressure. The skin is a flexible and responsive organ. Skin is composed of two principal layers—the epidermis and dermis. Each layer has a unique structure and performs a different function in the body.

Epidermis: The outer layer of skin

The epidermis is the outermost layer of the skin, and is made up of two parts—an exterior and interior portion. The exterior layer of the epidermis consists of 25 to 30 layers of dead, flattened cells that are continually being shed. Although dead, these cells still serve an important function as they contain a protein called keratin. Keratin helps protect the living cell layers underneath from exposure to bacteria, heat, and chemicals.

The interior layer of the epidermis contains living cells that continually divide to replace the dead cells. Some of these cells contain melanin, a pigment that colors the skin and helps protect body cells from damage by solar radiation. As the newly formed cells are pushed toward the skin’s surface, the nuclei degenerate and the cells die. Once they reach the outer-most epidermal layer, the cells are shed. This entire process takes about 28 days. Therefore, every four weeks, all cells of the epidermis are replaced by new cells.

Look at your fingertips. The epidermis on the fingers and palms of your hands, and on the toes and soles of your feet, contains ridges and grooves that are formed before birth. These epidermal ridges are important for gripping as they increase friction. As shown in Figure 34.2, footprints, as well as fingerprints, are often used to identify individuals as each person’s pattern is unique.

Dermis: The inner layer of skin

The second principal layer of the skin is the dermis. The dermis is the inner, thicker portion of the skin. The thickness of the dermis varies in different parts of the body, depending on the function of that part.

The dermis contains structures such as blood vessels, nerves, nerve endings, hair follicles, sweat glands, and oil glands. Why do some people have dark skin while others are pale? Find out by examining Figure 34.1. Beneath the dermis, the skin is attached to underlying tissues by the subcutaneous layer, which consists of fat and connective tissue. These fat deposits also help the body absorb impact, retain heat, and store food.

Hair, another structure of the integumentary system, grows out of narrow cavities in the dermis called hair follicles, as shown in Figure 34.3. As hair follicles develop, they are supplied with blood vessels and nerves and become attached to muscle tissue. Most hair follicles have an oil gland associated with them. When oil and dead cells block the opening of the hair follicle, pimples may form.

Functions of the integumentary system

One function of skin is to help maintain homeostasis by regulating your internal body temperature. When your body temperature rises, the many small blood vessels in the dermis dilate, blood flow increases, and body heat is lost by radiation. This mechanism also works in reverse. When you are cold, the blood vessels in the skin constrict and heat is conserved.

Another noticeable thing that happens to your skin as your body heats up is that it becomes wet. Glands in the dermis produce sweat in response to an increase in body temperature. As sweat evaporates, water changes state from liquid to vapor and heat is lost. The body cools as a result of the heat loss. Investigate further the role of skin in cooling the body by carrying out the Problem-Solving Lab on this page.

Of course, anyone who has ever stepped on a sharp object or been burned by a hot pot handle knows that skin also functions as a sense organ. Nerve cells in the dermis receive stimuli from the external environment and relay information about pressure, pain, and temperature to the brain.

Skin also plays a role in producing essential vitamins. When exposed to ultraviolet light, skin cells produce vitamin D, a nutrient that aids the absorption of calcium into the blood-stream. As a person’s exposure to sunlight varies, daily intake of vitamin D from dietary sources or supplements may be needed to meet requirements. Skin also serves as a protective layer to underlying tissues. It shields the body from physical and chemical damage and from invasion by microbes. Cuts or other openings in the skin surface allow bacteria to enter the body, so they must be repaired quickly. Figure 34.4 shows the stages involved in skin repair.

Bones: The Body’s Support

Skeletal System Structure

The adult human skeleton contains about 206 bones. Its two main parts are shown in Figure 34.6 on the next page. The axial skeleton includes the skull and the bones that support it, such as the vertebral column, the ribs, and the sternum. The appendicular skeleton includes the bones of the arms and legs and structures associated with them, such as the shoulder and hip bones, wrists, ankles, fingers, and toes.

Joints: Where bones meet

Next time you open a door, notice how it is connected to the door frame. A metal joint positioned where the door and frame meet allows the door to move easily back and forth. In vertebrates, joints are found where two or more bones meet. Most joints facilitate the movement of bones in relation to one another. The joints of the skull, on the other hand, are fixed, as the bones of the skull don’t move. These immovable joints are actually held together by the intergrowth of bone, or by fibrous cartilage.

Joints are often held together by ligaments. A ligament is a tough band of connective tissue that attaches one bone to another. Joints with large ranges of motion, such as the knee, typically have more ligaments surrounding them. In movable joints, the ends of bones are covered by cartilage.

This layer of cartilage allows for smooth movement between the bones. In addition, joints such as those of the shoulder and knee have fluid-filled sacs called bursae located on the outside of the joints. The bursae act to decrease friction and keep bones and tendons from rubbing against each other. Tendons, which are thick bands of connective tissue, attach muscles to bones. Figure 34.7 shows the different movable joints in the skeleton.

Forcible twisting of a joint, called a sprain, can result in injury to the bursae, ligaments, or tendons. A sprain most often occurs at joints with large ranges of motion such as the wrist, ankle, and knee.

Besides injury, joints are also subject to disease. One common joint disease is arthritis, an inflammation of the joints. It can be caused by infections, aging, or injury. One kind of arthritis results in bone spurs, or outgrowths of bone, inside the joints. Such arthritis is especially painful, and often limits a person’s ability to move his or her joints.

Compact and spongy bone

Although bones may appear uniform, they are actually composed of two different types of bone tissue: compact bone and spongy bone.

Surrounding every bone is a layer of hard bone, or compact bone. Running the length of compact bone are tubular structures known as osteon or Haversian systems, shown in Figure 34.8. Compact bone is made up of repeating units of osteon systems. Living bone cells, or osteocytes, receive oxygen and nutrients from small blood vessels running within the osteon systems. Nerves in the canals conduct impulses to and from each bone cell. Compact bone surrounds less dense bone known as spongy bone because, like a sponge, it contains many holes and spaces.

Formation of Bone

The skeleton of a vertebrate embryo is made of cartilage. By the ninth week of human development, bone begins to replace cartilage. Blood vessels penetrate the membrane covering the cartilage and stimulate its cells to become potential bone cells called osteoblasts. These potential bone cells secrete a protein called collagen in which minerals in the bloodstream begin to be deposited. The deposition of calcium salts and other ions hardens and the newly formed bone cells, now called osteocytes, are trapped. The adult skeleton is almost all bone, with cartilage found only in places where flexibility is needed—regions such as the nose tip, external ears, discs between vertebrae, and movable joint linings.

Bone growth

Your bones grow in both length and diameter. Growth in length occurs at the ends of bones in cartilage plates. Growth in diameter occurs on the outer surface of the bone. The increased production of sex hormones during your teen years causes the osteoblasts to divide more rapidly, resulting in a growth spurt. However, these same hormones will also cause the growth centers at the ends of your bones to degenerate. As these cells die, your growth will slow. After growth stops, bone-forming cells are involved in repair and maintenance of bone.

Skeletal System Functions

The primary function of your skeleton is to provide a framework for the tissues of your body. The skeleton also protects your internal organs, including your heart, lungs, and brain.

The arrangement of the human skeleton allows for efficient body movement. Muscles that move the body need firm points of attachment to pull against so they can work effectively. The skeleton provides these attachment points.

Bones also produce blood cells. Red marrow—found in the humerus, femur, sternum, ribs, vertebrae, and pelvis—is the production site for red blood cells, white blood cells, and cell fragments involved in blood clotting. Yellow marrow, found in many other bones, consists of stored fat as shown in Figure 34.9.

Bones store minerals

Finally, your bones serve as store-houses for minerals, including calcium and phosphate. Calcium is needed to form strong, healthy bones and is therefore an important part of your diet. Sources of calcium include milk, yogurt, cheese, lettuce, spinach, and other assorted leafy vegetables.

Bone injury and disease

Bones tend to become more brittle as their composition changes with age. For example, a disease called osteoporosis involves a loss of bone volume and mineral content, causing the bones to become more porous and brittle. Osteoporosis is most common in older women because they produce lesser amounts of estrogen—a hormone that aids in bone formation.

When bones are broken, as shown by the X-ray images in Figure 34.10, a doctor moves them back into position and immobilizes them with a cast or splint until the bone tissue regrows. Read more about the use of X rays in the diagnosis of broken bones in the Connection to Physics at the end of this chapter.

Muscles for Locomotion

Three Types of Muscles

Nearly half of your body mass is muscle. A muscle consists of groups of fibers, or cells, bound together. Almost all of the muscle fibers you will ever have were present at birth. Figure 34.11 shows the three main kinds of muscles in your body. One type of tissue, smooth muscle, is found in the walls of your internal organs and blood vessels.

Smooth muscle is made up of sheets of cells that are ideally shaped to form a lining for organs, such as the digestive tract and the reproductive tract. The most common function of smooth muscle is to squeeze, exerting pressure on the space inside the tube or organ it surrounds in order to move material through it. Examples include the movement of food through the digestive system and the movement of gametes through the reproductive system. Because contractions of smooth muscle are not under conscious control, smooth muscle is considered an involuntary muscle.

Another type of involuntary muscle is the cardiac muscle, which makes up your heart. Cardiac muscle fibers are interconnected and form a network that helps the heart muscle contract efficiently. Cardiac muscle is found only in the heart and is adapted to generate and conduct electrical impulses necessary for its rhythmic contraction. The third type of muscle tissue, skeletal muscle, is the type that is attached to and moves your bones. The majority of the muscles in your body are skeletal muscles, and, as you know, you can control their contractions. A muscle that contracts under conscious control is called a voluntary muscle.

Skeletal Muscle Contraction

Whether you are playing tennis, pushing a lawn mower, or writing, some muscles contract while others relax as the action is performed. Figure 34.12 shows the movement of the lower arm as controlled by opposing muscles in the upper arm. The majority of skeletal muscles work in opposing pairs.

Muscle tissue is made up of muscle fibers, which are actually just very long, fused muscle cells. Each fiber is made up of smaller units called myofibrils. Myofibrils are themselves composed of even smaller protein filaments that can be either thick or thin. The thicker filaments are made of the protein myosin, and the thinner filaments are made of the protein actin. Each myofibril can be divided into sections called sarcomeres, the functional units of muscle. How do nerves signal muscles to contract? Find out in Figure 34.13 on the next page.

The **sliding filament theory** currently offers the best explanation for how muscle contraction occurs. The **sliding filament theory** states that, when signaled, the actin filaments within each sarcomere slide toward one another, shortening the sarcomeres in a fiber and causing the muscle to contract. The myosin filaments, on the other hand, do not move.

Muscle Strength and Exercise

How can you increase the strength of your muscles? Muscle strength does not depend on the number of fibers in a muscle. It has been shown that this number is basically fixed before you are born. Rather, muscle strength depends on the thickness of the fibers and on how many of them contract at one time. Regular exercise stresses muscle fibers slightly; to compensate for this added workload, the fibers increase in diameter by adding myofibrils.

Recall that ATP is produced during cellular respiration. Muscle cells are continually supplied with ATP from both aerobic and anaerobic processes. However, the aerobic respiration process dominates when adequate oxygen is delivered to muscle cells, such as when a muscle is at rest or during moderate activity. When an adequate supply of oxygen is unavailable, such as during vigorous activity, an anaerobic process—specifically lactic acid fermentation—becomes the primary source of ATP production.

The Digestive and Endocrine Systems

Following Digestion of a Meal

Functions of the Digestive System

The main function of the digestive system is to disassemble the food you eat into its component molecules so that it can be used as energy for your body. In this sense, your digestive system can be thought of as a sort of disassembly line.

Digestion is accomplished through a number of steps. First, the system takes ingested food and begins moving it through the digestive tract. As it does so, it digests—or breaks down mechanically and chemically—the complex food molecules. Then, the system absorbs the digested food and distributes it to your cells. Finally, it eliminates undigested materials from your body. As you read about each digestive organ, use Figure 35.1 on the next page to locate its position within the system.

The Mouth

The first stop along the digestive disassembly line is your mouth. Suppose it is lunchtime and you have just prepared a bacon, lettuce, and tomato sandwich. The first thing you do is bite off a piece and chew it.

What happens as you chew?

As you chew, your tongue moves the food around and helps position it between your teeth. Chewing is a form of mechanical digestion, the physical process of breaking food into smaller pieces. Mechanical digestion prepares food particles for chemical digestion. Chemical digestion is the process of changing food on a molecular level through the action of enzymes. What purpose do the different structures inside your mouth serve? Find out by examining Figure 35.2.

Chemical digestion begins in the mouth

Some of the nutrients in your sandwich are starches, large molecules known as polysaccharides. As you chew your bite of sandwich, salivary glands in your mouth secrete saliva. Saliva contains a digestive enzyme, called amylase, which breaks down starch into smaller molecules such as di-or monosaccharides. In the stomach, amylase continues to digest starch in the swallowed food for about 30 minutes. Table 35.1 lists some digestive enzymes that act to break food molecules apart.

Swallowing your food

Once you’ve thoroughly chewed your bite of sandwich, your tongue shapes it into a ball and moves it to the back of your mouth to be swallowed. Swallowing forces food from your mouth into your throat and from there into your esophagus, a muscular tube that connects your mouth to your stomach. Food moves down the esophagus by way of peristalsis. Peristalsis is a series of involuntary smooth muscle contractions along the walls of the digestive tract.

The Stomach

When the chewed food reaches the end of your esophagus, it enters the stomach. The stomach is a muscular, pouchlike enlargement of the digestive tract. Both physical and chemical digestion take place in the stomach.

Muscular churning

Three layers of involuntary muscles, lying across one another, are located within the wall of the stomach. When these muscles contract, as shown in Figure 35.4, they work to physically break down the swallowed food, creating smaller pieces. As the muscles continue to work the food pieces, they mix them with digestive juices produced by the stomach.

Chemical digestion in the stomach

The inner lining of the stomach contains millions of glands that secrete a mixture of chemicals called gastric juice. Gastric juice contains pepsin and hydrochloric acid. Pepsin is an enzyme that begins the chemical digestion of proteins in food. Pepsin works best in the acidic environment provided by hydrochloric acid, which increases the acidity of the stomach contents to pH 2.

How is the stomach lining protected from powerful digestive enzymes and strong acids? The stomach lining secretes mucus that forms a protective layer between it and the acidic environment of the stomach.

Food remains in your stomach for approximately two to four hours. When food is ready to leave the stomach, it is about the consistency of tomato soup. Peristaltic waves gradually become more vigorous and begin to force small amounts of liquid out of the lower end of the stomach and into the small intestine.

The Small Intestine

From your stomach, the liquid food moves into your small intestine, a muscular tube about 6 m long. This section of the intestine is called small not because of its length, but because of its narrow diameter—only 2.5 cm. Digestion of your meal is completed within the small intestine. Muscle contractions contribute to further mechanical breakdown of the food. At the same time, carbohydrates and proteins undergo further chemical digestion with the help of enzymes produced and secreted by the pancreas and liver.

Chemical action

The first 25 cm of the small intestine is called the duodenum. Most of the enzymes and chemicals that function in the duodenum enter it through ducts that collect juices from the pancreas, liver, and gallbladder. These organs, shown in Figure 35.5, play important roles in digestion, even though food does not pass directly through them.

Secretions of the pancreas

The pancreas is a soft, flattened gland that secretes both digestive enzymes and hormones, which you will learn more about in the last section of this chapter. The mixture of enzymes it secretes breaks down carbohydrates, proteins, and fats. Alkaline pancreatic juices also help to neutralize the acidity of the liquid food, stopping any further action of pepsin.

Secretions of the liver

The liver is a large, complex organ that has many functions. One of its functions is to produce bile. Bile is a chemical substance that helps break down fats. Once made in the liver, bile is stored in a small organ called the gallbladder.

From the gallbladder, bile passes into the duodenum. Bile causes further mechanical digestion by breaking apart large drops of fat into smaller droplets. If bile becomes too concentrated due to high levels of cholesterol in the diet, or if the gallbladder becomes inflamed, gallstones can form, as seen in Figure 35.6. Can a person live without a gallbladder?

Absorption of food

Liquid food stays in your small intestine for three to five hours and is slowly moved along its length by peristalsis. As digested food moves through the intestine, it passes over thousands of tiny fingerlike structures called villi. A villus is a single projection on the lining of the small intestine that functions in the absorption of digested food. The villi greatly increase the surface area of the small intestine, allowing for a greater absorption rate. Because the digested food is now in the form of small molecules, it can be absorbed directly into the cells of the villi, as shown in Figure 35.7. The food molecules then diffuse into the blood vessels of the villus and enter the bloodstream. The villi are the link between the digestive system and the circulatory system.

The Large Intestine

The indigestible material from your meal now passes into your large intestine, a muscular tube that is also called the colon. Although the large intestine is only about 1.5 m long, it is much wider than the small intestine—about 6.5 cm in diameter. The appendix, a tubelike extension off the large intestine thought to be an evolutionary remnant from our herbivorous ancestors, seems to serve no function in human digestion.

Water absorption

As the indigestible mixture passes through the large intestine, water and salts are absorbed by the intestinal walls, leaving behind a more solid material. In this way, the water is not wasted. A secondary function of the large intestine is vitamin synthesis. Anaerobic bacteria in the large intestine synthesize some B vitamins and vitamin K, which are absorbed as needed by the body. The presence of certain bacteria in the large intestine is beneficial in another way. Under normal conditions, these bacteria stop harmful bacteria from colonizing, reducing the risk of intestinal infections.

Elimination of wastes

After 18 to 24 hours in the large intestine, the remaining indigestible material, now called feces, reaches the rectum. The rectum is the last part of the digestive system. Feces are eliminated from the rectum through the anus. Your meal’s entire journey through the digestive tract has taken between 24 and 33 hours.

Nutrition

The Vital Nutrients

Six basic kinds of nutrients can be found in foods: carbohydrates, fats, proteins, minerals, vitamins, and water. These substances are essential to proper body function. You supply your body with these nutrients when you eat foods from the five main food groups shown in Figure 35.8.

Carbohydrates

Perhaps your favorite food is pasta, fresh-baked bread, or corn on the cob. If so, your favorite food contains carbohydrates, important sources of energy for your body cells. Recall that carbohydrates are starches and sugars. Starches are complex carbohydrates found in bread, cereal, potatoes, rice, corn, beans, and pasta. Sugars are simple carbohydrates found mainly in fruits, such as plums, strawberries, and oranges.

During digestion, complex carbohydrates are broken down into simple sugars, such as glucose, fructose, and galactose. Absorbed into the bloodstream through the villi of the small intestine, these sugar molecules circulate to fuel body functions. Some sugar is carried to the liver where it is stored as glycogen.

Cellulose, another complex carbohydrate, is found in all plant cell walls and is not digestible by humans. However, cellulose (also known as fiber) is still an important item to include in the diet as it helps in the elimination of wastes. Sources of fiber include bran, beans, and lettuce.

Fats

Many people think that eating fat means getting fat; however, fats are an essential nutrient. They provide energy for your body and are also used as building materials. Recall that fats are essential building blocks of the cell membrane. They are also needed to synthesize hormones, protect body organs against injury, and insulate the body from cold.

Sources of fat in the diet include meats, nuts, and dairy products, as well as cooking oils. In the digestive system, fats are broken down into fatty acids and glycerol and absorbed by the villi of the small intestine. Eventually, some of these fatty acids end up in the liver. The liver converts them to glycogen or stores them as fat throughout your body.

Proteins

Your body has many uses for proteins. Enzymes, antibodies, many hormones, and substances that help the blood to clot, are all proteins. Proteins form part of muscles and many cell structures, including the cell membrane.

During digestion, proteins are broken down into amino acids. After the amino acids have been absorbed by the small intestine, they enter the blood-stream and are carried to the liver. The liver can convert amino acids to fats or glucose, both of which can be used for energy. However, your body uses amino acids for energy only if other energy sources are depleted. Most amino acids are absorbed by cells and used for protein synthesis. The human body needs 20 different amino acids to carry out protein synthesis, but it can make only 12 of them. The remaining 8 must be consumed in the diet and so are called essential amino acids. Sources of essential amino acids include meats, dried beans, whole grains, eggs, and dairy products.

Minerals and vitamins

When you think of minerals, you may picture substances that people mine, or extract from Earth. As shown in Figure 35.9 on the previous page, the same minerals can also be extracted from foods and put to use by your body.

A mineral is an inorganic substance that serves as a building material or takes part in a chemical reaction in the body. Minerals make up about four percent of your total body weight, most of it in your skeleton. Although they serve many different functions within the body, minerals are not used as an energy source.

Unlike minerals, vitamins are organic nutrients that are required in small amounts to maintain growth and metabolism. The two main groups of vitamins are fat-soluble and water-soluble, as shown in Table 35.2. Although fat-soluble vitamins can be stored in the liver, the accumulation of excess amounts can prove toxic. Water-soluble vitamins cannot be stored in the body and so must be included regularly in the diet. Table 35.2 lists foods that contain fat-soluble and water-soluble vitamins.

Vitamin D, a fat-soluble vitamin, is synthesized in your skin. Vitamin K and some B vitamins are made by bacteria in your large intestine. The rest of the vitamins must be consumed in your diet.

Water

Water is the most abundant substance in your body—between 45 and 75 percent of your total body mass. Water facilitates the chemical reactions in your body and is necessary for the breakdown of foods during digestion. Water is also an excellent solvent; oxygen and nutrients from food could not enter your cells if they did not first dissolve in water.

Recall that water absorbs and releases heat slowly. It is this characteristic that helps water maintain your body’s internal temperature. A large amount of heat is needed to raise the temperature of water. Because the body contains so much water, it takes a lot of added energy to raise its internal temperature. Your body loses about 2.5 L of water per day through exhalation, sweat, and urine. As a result, water must be replaced constantly.

Calories and Metabolism

The energy content of food is measured in units of heat called Calories, each of which represents a kilocalorie, or 1000 calories (written with a small c). A calorie is the amount of heat required to raise the temperature of 1 mL of water by 1°C. Some foods, especially those with fats, contain more Calories than others. In general, 1 g of fat contains nine Calories, while 1 g of carbohydrate or protein contains four Calories. To learn more about Calories in meals, complete the MiniLab on this page.

The number of Calories needed each day varies from person to person, depending on metabolism, or rate at which energy is burned. As you will see in the next section, a major regulator of metabolic rate is a hormone from the thyroid gland.

A person’s body mass, age, gender, and level of physical activity also affect metabolic rate. Generally, males need more Calories per day than females, teenagers need more than adults, and active people need more than inactive people.

Calories and health

What happens if a person consumes more Calories than his or her body can metabolize? When the energy taken in is greater than the energy expended, the extra energy is stored as body fat and a person gains weight. However, if a person eats fewer Calories than the body can metabolize, some of the body’s stored energy is used and weight is lost.

Physicians have determined that many Americans are overweight. Being overweight or obese increases a person’s risk for developing health problems such as high blood pressure, diabetes, and heart disease. Being underweight is also associated with health problems such as anemia, fatigue, and decreased ability to fight infection and disease. A simple way to determine if a person is at a healthy weight is to calculate his or her Body Mass Index (BMI). Calculate a sample BMI by doing the Problem-Solving Lab on this page.

Millions of people put themselves on diets every year in hopes of losing weight. While many diets are nutritionally sound, others prescribe eating habits that are not sensible and usually fail to produce the desired result. Read more about weight-loss products in the Biology and Society section at the end of this chapter.

The Endocrine System

Control of the Body

Internal control of the body is directed by two systems: the nervous system, which you will learn more about later, and the endocrine system. The endocrine system is made up of a series of glands, called endocrine glands, that release chemicals directly into the bloodstream. These chemicals act as messengers, relaying information to other parts of the body. Ultimately, the functions of all body systems are controlled by the interaction between the nervous and endocrine systems.

Interaction of the nervous system and endocrine system

Much of the time, the endocrine system and the nervous system work together to maintain homeostasis within the body. Because there are two control systems within the body, coordination between the two systems is needed. The hypothalamus is the portion of the brain that connects the endocrine and nervous systems. The hypothalamus receives messages from other areas of the brain and from internal organs. When a change in homeostasis is detected, the hypothalamus stimulates the pituitary gland. The pituitary gland, the main gland of the endocrine system, is located in the skull just beneath the hypothalamus. The pituitary gland is controlled by the hypothalamus, and the two are connected by nerves and blood vessels. In response to messages received by the hypothalamus, the pituitary gland releases its own chemicals or stimulates other glands to release theirs. Other endocrine glands under the control of the pituitary include the thyroid gland, the adrenal glands, and glands associated with reproduction.

Endocrine control of the body

The chemicals secreted by endocrine glands into the bloodstream are called hormones. Recall that a hormone is a chemical released in one part of an organism that affects another part. Hormones convey information to other cells in your body, giving them instructions regarding your metabolism, growth, development, and behavior. Once released by the glands, the hormones travel in the bloodstream and then attach to specific binding sites found on the plasma membranes, or in the nuclei, of target cells. These binding sites on cells are called receptors. Figure 35.10 summarizes the action of different endocrine glands.

Example of endocrine control

Human growth hormone (hGH) is a good example of an endocrine system hormone. When your body is actively growing, blood glucose levels are slightly lowered as the growing cells use up the sugar. This low blood glucose level is detected by the hypothalamus, which stimulates the production and release of hGH from the pituitary into the bloodstream. hGH binds to receptors on the plasma membranes of liver cells, stimulating the liver cells to release glucose into your blood. Your cells need the glucose in order to continue growing. Figure 35.11 summarizes the control of hGH by the pituitary gland.

Negative Feedback Control

If homeostasis is disrupted, endocrine glands can be stimulated by the nervous system, changes in blood chemistry, or by other hormones. Regulation of the endocrine system is controlled most often through one type of internal feedback mechanism called a **negative feedback system**. In a **negative feedback system**, the hormones, or their effects, are fed back to inhibit the original signal. Once homeostasis is reached, the signal is stopped and the hormone is no longer released. The thermostat in your home is controlled by a similar negative feedback system. It maintains the room at a set temperature. When the temperature drops, the thermostat senses the reduction of thermal energy and signals the heater to increase its output. When the thermal energy of the room rises again to a certain point, the thermostat no longer stimulates the heater, which shuts off. When the temperature drops again, the process repeats itself. In this negative feedback system, the increase in temperature “feeds back” to signal the thermostat to stop stimulating thermal energy production.

Feedback control of hormones

The majority of endocrine glands operate under negative feedback systems. A gland synthesizes and secretes its hormone, which travels in the blood to target cells where the appropriate response occurs. Information regarding the hormone level or its effect on target cells is fed back, usually to the hypothalamus or pituitary gland, to regulate the gland’s production of the hormone.

Control of blood water levels

Let’s look at an example of a hormone that is controlled by a negative feedback system. After working out in the gym and building up a sweat, you are thirsty. This is because the water content of your blood has been reduced. The hypothalamus, which is able to sense the concentration of water in your blood, determines that your body is dehydrated. In response, it stimulates the pituitary gland to release antidiuretic hormone (ADH).

ADH reduces the amount of water in your urine. It binds to receptors in kidney cells, promoting the reabsorption of water and reducing the amount of water excreted in urine. Information about blood water levels is constantly fed back to the hypothalamus so it can regulate the pituitary’s release of ADH. If the body becomes overhydrated, the hypothalamus stops stimulating release of ADH.

Control of blood glucose levels

Another example of a negative feedback system involves the regulation of blood glucose levels. Unlike most other endocrine glands, the pancreas is not controlled by the pituitary gland. When you have just eaten and your blood glucose levels are high, your pancreas releases the hormone insulin. Then, insulin signals liver and muscle cells to take in glucose, thus lowering blood glucose levels. When blood glucose levels become too low, another pancreatic hormone, glucagon, is released. Glucagon binds to liver cells, signaling them to release stored glycogen as glucose.

Hormone Action

Once hormones are released by an endocrine gland, they travel to target cells and cause a change. Hormones can be grouped into two basic types according to how they act on their target cells: steroid hormones and amino acid hormones.

Action of steroid hormones

Hormones that are made from lipids are called steroid hormones. Steroid hormones are lipid-soluble and therefore diffuse freely into cells through their plasma membranes, as shown in Figure 35.12. There they bind to a hormone receptor inside the cell. The hormone-receptor complex then travels to the nucleus where it activates the synthesis of specific messenger RNA molecules. The mRNA molecules move out to the cytoplasm where they guide the synthesis of the required proteins.

Action of amino acid hormones

The second group of hormones is made from amino acids. Recall that amino acids can be strung together in chains and that proteins are made from long chains of amino acids. Some hormones are short chains of amino acids and others are large chains. These amino acid hormones, once secreted into the bloodstream, bind to receptors embedded in the plasma membrane of the target cell, as shown in Figure 35.13. From there, they open ion channels in the membrane, or route signals down from the surface of the membrane to activate enzymes inside the cell. The enzymes, in turn, alter the behavior of other molecules inside the cell. In both of these ways, the hormone is able to control what goes on inside the target cell.

Adrenal Hormones and Stress

You are sitting in math class and the teacher is about to hand out the semester test. Because this test is an important one, you have spent many hours studying for it. Like most of your classmates, you are a little nervous as the test is being passed down the row. Your heart is beating fast and your hands are a little sweaty. As you review the first problem, however, you begin to calm down because you know how to solve it. The adrenal glands play an important role in preparing your body for stressful situations. The adrenal glands are located on top of the kidneys and consist of two parts—an inner portion and an outer portion.

The outer portion secretes steroid hormones, including glucocorticoids and aldosterone.

These steroid hormones cause an increase in available glucose and raise blood pressure. In this way, they help the body combat stresses such as fright, temperature extremes, bleeding, infection, disease, and even test anxiety.

The inner portion of the adrenal gland secretes two amino acid hormones: epinephrine—often called adrenaline—and norepinephrine. Recall the fight-or-flight response discussed in the animal behavior chapter. During such a response, the hypothalamus relays impulses to the nervous system, which in turn stimulates the adrenal glands to increase their output of epinephrine and norepinephrine. These hormones increase heart rate, blood pressure, and rate of respiration; increase efficiency of muscle contractions; and increase blood sugar levels. If you have ever had to perform in front of a large audience, you may have experienced these symptoms, often referred to collectively as an “adrenaline rush.” This is how the body prepares itself to face or flee a stressful situation.

Thyroid and Parathyroid Hormones

The thyroid gland, located in the neck, regulates metabolism, growth, and development. The main metabolic and growth hormone of the thyroid is thyroxine. This hormone affects the rate at which the body uses energy and determines your food intake requirements.

The thyroid gland also secretes calcitonin—a hormone that regulates calcium levels in the blood. Calcium is a mineral the body needs for blood clotting, formation of bones and teeth, and normal nerve and muscle function. Calcitonin binds to the membranes of kidney cells and causes an increase in calcium excretion. Calcitonin also binds to bone-forming cells, causing them to increase calcium absorption and synthesize new bone.

Another hormone involved in mineral regulation, parathyroid hormone (PTH), is produced by the parathyroid glands, which are attached to the thyroid gland. The release of PTH leads to an increase in the rate of calcium, phosphate, and magnesium absorption in the intestines. PTH causes the release of calcium and phosphate from bone tissue. It also increases the rate at which the kidneys remove calcium and magnesium from urine and return them to the blood.

The overall effect of parathyroid hormone and calcitonin hormone interaction in the body is shown in Figure 35.14. Take a closer look at thyroid and parathyroid tissue by completing the MiniLab on the previous page.

As you can see, hormones associated with the endocrine system are responsible for controlling many different functions in your body. Different hormones may play more important roles during some periods in your life than others. In any case, they remain the principal biological influence on your behavior and development.

The Nervous System

Neurons: Basic Units of the Nervous System

The basic unit of structure and function in the nervous system is the neuron, or nerve cell. Neurons conduct impulses throughout the nervous system. As shown in Figure 36.1, a neuron is a long cell that consists of three regions: a cell body, dendrites, and an axon.

Dendrites are branchlike extensions of the neuron that receive impulses and carry them toward the cell body. The axon is an extension of the neuron that carries impulses away from the cell body and toward other neurons, muscles, or glands.

Neurons fall into three categories: sensory neurons, motor neurons, and interneurons. Sensory neurons carry impulses from the body to the spinal cord and brain.

Interneurons are found within the brain and spinal cord. They process incoming impulses and pass response impulses on to motor neurons. Motor neurons carry the response impulses away from the brain and spinal cord to a muscle or gland.

Relaying an impulse

Suppose you’re in a crowded, noisy store and you feel a tap on your shoulder. Turning your head, you see the smiling face of a good friend. How did the shoulder tap get your attention? The touch stimulated sensory receptors located in the skin of your shoulder to produce an impulse. The sensory impulse was carried to the spinal cord and then up to your brain. From your brain, an impulse was sent out to your motor neurons, which then transmitted the impulse to muscles in your neck. Your neck muscles then turned your head. Figure 36.2 shows how a stimulus, such as a tap on the shoulder, is transmitted through your nervous system.

A neuron at rest

First, let’s look at a resting neuron— one that is not transmitting an impulse. You have learned that the plasma membrane controls the concentration of ions in a cell. Because the plasma membrane of a neuron is more permeable to potassium ions (K+) than it is to sodium ions (Na+), more potassium ions exist inside of the cell membrane than outside it. Similarly, more sodium ions exist outside the cell membrane than inside of it.

The neuron membrane also contains an active transport system, called the sodium/potassium (Na+/K+) pump, which uses ATP to pump three sodium ions out of the cell for every two potassium ions it pumps in. As you can see in Figure 36.3, the action of the pump increases the concentration of positive charges on the outside of the membrane. In addition, the presence of many negatively charged proteins and organic phosphates means that the inside of the membrane is more negatively charged than the outside. Under these conditions, which exist when the cell is at rest, the plasma membrane is said to be polarized. A polarized membrane has the potential to transmit an impulse.

How an impulse is transmitted

When a stimulus excites a neuron, gated sodium channels in the membrane open up and sodium ions rush into the cell. As the positive sodium ions build up inside the membrane, the inside of the cell becomes more positively charged than the outside. This change in charge, called depolarization, moves like a wave down the length of the axon, as seen in Figure 36.4. As the wave passes, gated channels and the Na+/K+ pump act to return the neuron to its resting state, with the inside of the cell negatively charged and the outside positively charged.

An impulse can move down the complete length of an axon only when stimulation of the neuron is strong enough. If the threshold level—the level at which depolarization occurs—is not reached, the impulse quickly dies out.

White matter and gray matter

Most axons are surrounded by a white covering of cells called the myelin sheath, shown previously in Figure 36.1. Like the plastic coating on an electric wire, the myelin sheath insulates the axon, hindering the movement of ions across its plasma membrane. The ions move quickly down the axon until they reach a gap in the sheath. Here, the ions pass through the plasma membrane of the nerve cell and depolarization occurs. As a result, the impulse jumps from gap to gap, greatly increasing the speed at which it travels.

The myelin sheath gives axons a white appearance. In the brain and spinal cord, masses of myelinated axons make up what is called “white matter.” The absence of myelin in masses of neurons accounts for the grayish color of “gray matter” in the brain.

Connections between neurons

Although neurons lie end to end— axons to dendrites—they don’t actually touch. A tiny space lies between one neuron’s axon and another neuron’s dendrites. This junction between neurons is called a synapse. Impulses traveling to and from the brain must move across the synaptic space that separates the axon and dendrites. How do they make this leap?

As an impulse reaches the end of an axon, calcium channels open, allowing calcium to enter the end of the axon. As shown in Figure 36.5, the calcium causes vesicles in the axon to fuse with the plasma membrane, releasing their chemicals into the synaptic space by exocytosis. These chemicals, called neurotransmitters, diffuse across the space to the dendrites of the next neuron. As the neurotransmitters reach the dendrites, they signal receptor sites to open the ion channels. These open channels change the polarity in the neuron, initiating a new impulse. Enzymes in the synapse typically break down the neurotransmitters shortly after transmission, preventing the continual firing of impulses.

The Central Nervous System

When you make a call to a friend, your call travels through wires to a control center where it is switched over to wires that connect with your friend’s telephone. In the same manner, an impulse traveling through neurons in your body usually reaches the control center of the nervous system—your brain—before being rerouted. The brain and the spinal cord together make up the central nervous system, which coordinates all your body’s activities.

Two systems work together

Another division of your nervous system, called the peripheral nervous system, is made up of all the nerves that carry messages to and from the central nervous system. It is similar to the telephone wires that run between a phone system’s control center and the phones in individual homes. Together, the central nervous system (CNS) and the peripheral nervous system (PNS), shown in Figure 36.6, respond to stimuli from the external environment.

Anatomy of the brain

The brain is the control center of the entire nervous system. For descriptive purposes, it is useful to divide the brain into three main sections: the cerebrum, the cerebellum, and the brain stem.

The cerebrum is divided into two halves, called hemispheres, that are connected by bundles of nerves. Your conscious activities, intelligence, memory, language, skeletal muscle movements, and senses are all controlled by the cerebrum. The outer surface of the cerebrum, called the cerebral cortex, is made up of gray matter. The cerebral cortex contains countless folds and grooves that increase its total surface area. This increase in surface area played an important role in the evolution of human intelligence as greater surface area allowed more and more complex thought processes.

The cerebellum, located at the back of your brain, controls your balance, posture, and coordination. If the cerebellum is injured, your movements become jerky.

The brain stem is made up of the medulla oblongata, the pons, and the midbrain. The medulla oblongata is the part of the brain that controls involuntary activities such as breathing and heart rate. The pons and midbrain act as pathways connecting various parts of the brain with each other. Read more about how the brain evolved on pages 1090–1091 in the Focus On. For the latest on technological advances in brain imaging, check out the Biotechnology section at the end of the chapter.

The Peripheral Nervous System

Remember that the peripheral nervous system carries impulses between the body and the central nervous system. For example, when a stimulus is picked up by receptors in your skin, it initiates an impulse in the sensory neurons. The impulse is carried to the CNS. There, the impulse transfers to motor neurons that carry the impulse to a muscle.

The peripheral nervous system can be separated into two divisions—the somatic nervous system and the autonomic nervous system.

The somatic nervous system

The somatic nervous system is made up of 12 pairs of cranial nerves from the brain, 31 pairs of spinal nerves from the spinal cord, and all of their branches. These nerves are actually bundles of neuron axons bound together by connective tissue. The cell bodies of the neurons are found in clusters along the spinal column. Most nerves contain both sensory and motor axons.

The nerves of the somatic system relay information mainly between your skin, the CNS, and skeletal muscles. This pathway is voluntary, meaning that you can decide whether or not to move body parts under the control of this system.

Reflexes in the somatic system

Sometimes a stimulus results in an automatic, unconscious response within the somatic system. When you touch something hot, you automatically jerk your hand away. Such an action is a reflex, an automatic response to a stimulus. Rather than proceeding to the cerebrum or cerebellum for interpretation, a reflex impulse travels to the spinal column or brain stem where it causes an impulse to be sent directly back to a muscle. The brain becomes aware of the reflex only after it occurs. Figure 36.7 on the next page shows the shortened route of a reflex impulse.

The autonomic nervous system

Imagine that you are spending the night alone in a creepy old house. Suddenly, a creak comes from the attic and you think you hear footsteps. Your heart begins to pound. Your breathing becomes rapid. Your thoughts race wildly as you try to figure out what to do—stay and confront the unknown, or run out of the house!

Your internal reactions to this scary situation are being controlled by your autonomic nervous system. The autonomic nervous system carries impulses from the CNS to internal organs. These impulses produce responses that are involuntary, or not under conscious control.

There are two divisions of the autonomic nervous system—the sympathetic nervous system and the parasympathetic nervous system. The sympathetic nervous system controls many internal functions during times of stress. When something frightens you, such as the rattlesnake shown in Figure 36.8, the sympathetic nervous system causes the release of hormones, such as epinephrine and norepinephrine, that results in the fight-or-flight response.

The parasympathetic nervous system on the other hand, controls many of the body’s internal functions when it is at rest. It is in control when you are relaxing after a picnic or reading quietly in your room. Both the sympathetic and parasympathetic systems send signals to the same internal organs. The resulting activity of the organ depends on the intensities of the opposing signals.

The different divisions and subsystems of your nervous system are summarized in Figure 36.9. Each division plays a key role in communication and control within your body.

Note that the sympathetic and parasympathetic systems are part of the autonomic nervous system. The autonomic and somatic systems are part of the PNS. The peripheral nervous system carries information to and from the CNS. Together, these two systems respond to stimuli from the external and internal environment.

The Senses

Sensing Chemicals

How are you able to smell and taste an orange? Chemical molecules of the orange contact receptors in your nose and mouth as you sniff and eat the fruit. The receptors for smell are hairlike nerve endings located in the upper portion of your nose, as shown in Figure 36.10. Chemicals acting on these nerve endings initiate impulses in the olfactory nerve, which is connected to your brain. In the brain, this signal is interpreted as a particular odor.

The senses of taste and smell are closely linked. Think about what your sense of taste is like when your nose is stuffed up and you can smell little, if anything. Because much of what you taste depends on your sense of smell, your sense of taste may also be dulled.

You taste something when chemicals dissolved in saliva contact sensory receptors on your tongue called taste buds. Tastes that you experience can be divided into four basic categories: sour, salty, bitter, and sweet. As seen with the sequence of electrochemical changes a neuron undergoes as it is depolarized, each of the different tastes produces a similar change in the cells of taste buds. As these cells are depolarized, signals from your taste buds are sent to the cerebrum. There, the signal is interpreted and you notice a particular taste. A young adult has approximately 10 000 taste buds. As a person ages, his or her sense of smell becomes less sharp and taste buds may decrease in number or become less sensitive. This can result in a decreased sense of taste.

Sensing Light

How are you able to see? Your sense of sight depends on receptors in your eyes that respond to light energy. The retina, found at the back of the eye, is a thin layer of tissue made up of light receptors and sensory neurons. Light enters the eye through the pupil and is focused by the lens onto the back of the eye, where it strikes the retina. Follow the pathway of light to the retina in Figure 36.11.

The retina contains two types of light receptor cells—rods and cones. Rods are receptor cells adapted for vision in dim light. They help you detect shape and movement. Cones are receptor cells adapted for sharp vision in bright light. They also help you detect color.

At the back of the eye, retinal tissue comes together to form the optic nerve, which leads to the brain, where images are interpreted. Can you see as well with one eye as with two?

Sensing Mechanical Stimulation

How are you able to hear the leaves rustle and feel the grass as you relax in the park? These senses, hearing and touch, depend on receptors that respond to mechanical stimulation.

Your sense of hearing

Every sound causes the air around it to vibrate. These vibrations travel outward from the source in waves, called sound waves. Sound waves enter your outer ear and travel down to the end of the ear canal, where they strike a membrane called the eardrum and cause it to vibrate. The vibrations then pass to three small bones in the middle ear—the malleus, the incus, and the stapes. As the stapes vibrates, it causes the membrane of the oval window, a structure between the middle and inner ear, to move back and forth.

From here, the vibrations continue to travel deeper into the ear. The movement from the oval window causes fluid in the cochlea, a snail-shaped structure in the inner ear, to move. Inside the circular walls of the cochlea are structures that are lined with hair cells. The fluid in the cochlea moves like a wave against the hair cells causing them to bend.

The movement of the hairs produces electrical impulses, which travel along the auditory nerve to the sides of the cerebrum, where they are interpreted as sound. Trace the pathway of sound waves in Figure 36.13. Hearing loss can occur if the auditory nerve or the hair cells in the cochlea are damaged. To find out what impact loud sounds have on your hearing, do the Problem-Solving Lab on this page.

Your sense of balance

The inner ear also converts information about the position of your head into nerve impulses which travel to your brain, informing it about your body’s equilibrium.

Maintaining balance is the function of your semicircular canals. Like the cochlea, the semicircular canals are also filled with a thick fluid and lined with hair cells. When you tilt your head, the fluid moves, causing the hairs to bend. This movement stimulates the hair cells to produce impulses. Neurons from the semicircular canals carry the impulses to the brain, which sends an impulse to stimulate your neck muscles and readjust the position of your head.

Your sense of touch

Like the ear, your skin also responds to mechanical stimulation with receptors that convert the stimulus into a nerve impulse. Receptors in the dermis of the skin respond to changes in temperature, pressure, and pain. It is with the help of these receptors, shown in Figure 36.14, that your body is able to respond to its external environment.

Although some receptors are found all over your body, those responsible for responding to particular stimuli are usually concentrated within certain areas of your body. For example, receptors that respond to light pressure are numerous in the dermis of your finger-tips, eyelids, lips, the tip of your tongue, and the palms of your hands. When these receptors are stimulated, you perceive sensations of light touch. Receptors that respond to heavier pressure are found inside your joints, in muscle tissue, and in certain organs. They are also abundant on the skin of your palms and fingers and on the soles of your feet. When these receptors are stimulated, you perceive heavy pressure.

Free nerve endings extend into the lower layers of the epidermis. Free nerve endings act as receptors for itch, tickle, hot and cold, and pain sensations. Heat receptors are found deep in the dermis, while cold receptors are found closer to the surface of your skin. Pain receptors can be found in all tissues of the body except those in the brain.

The Effects of Drugs

Drugs Act on the Body

You probably hear the word drug used often, maybe even every day. A drug is a chemical that affects the body’s functions. Most drugs interact with receptor sites on cells, probably the same ones used by neurotransmitters of the nervous system or hormones of the endocrine system. Some drugs increase the rate at which neurotransmitters are synthesized and released, or slow the rate at which they are broken down, as illustrated in Figure 36.15. Other drugs interfere with a neurotransmitter’s ability to interact with its receptor. Explore how these different drugs work on neurotransmitters by doing the Problem-Solving Lab on the next page.

Medicinal Uses of Drugs

A medicine is a drug that, when taken into the body, helps prevent, cure, or relieve a medical problem. Some of the many kinds of medicines used to relieve medical conditions are discussed below.

Relieving pain

Headache, muscle ache, cramps— all are common pain sensations. You just studied how pain receptors in your body send signals to your brain. Medicines that relieve pain manipulate either the receptors that initiate the impulses or the central nervous system that receives them.

Pain relievers that do not cause a loss of consciousness are called analgesics. Some analgesics, like aspirin, work by inhibiting receptors at the site of pain from producing nerve impulses. Analgesics that work on the central nervous system are called narcotics. Many narcotics are made from the opium poppy flower, shown in Figure 36.16. Opiates, as they are called, can be useful in controlled medical therapy because these drugs are able to relieve severe pain from illness or injury.

Treating circulatory problems

Many drugs have been developed to treat heart and circulatory problems such as high blood pressure. These medicines are called cardiovascular drugs. In addition to treating high blood pressure, cardiovascular drugs may be used to normalize an irregular heartbeat, increase the heart’s pumping capacity, or enlarge small blood vessels. Discover how various types of drugs can affect heart rate by doing the BioLab at the end of this chapter.

Treating nervous disorders

Several kinds of medicines are used to help relieve symptoms of nervous system problems. Among these medicines are stimulants and depressants. Drugs that increase the activity of the central and sympathetic nervous systems are called stimulants.

Amphetamines are synthetic stimulants that increase the output of CNS neurotransmitters. Amphetamines are seldom prescribed because they can lead to dependence. However, because they increase wakefulness and alertness, amphetamines are sometimes used to treat patients with sleep disorders.

Drugs that lower, or depress, the activity of the nervous system are called depressants, or sedatives. The primary medicinal uses of depressants are to encourage calmness and produce sleep. For some people, the symptoms of anxiety are so extreme that they interfere with the person’s ability to function effectively. By slowing down the activities of the CNS, a depressant can temporarily relieve some of this anxiety.

The Misuse and Abuse of Drugs

The misuse or abuse of drugs can cause serious health problems—even death. Drug misuse occurs when a medicine is taken for an unintended use. For example, giving your prescription medicine to someone else, not following the prescribed dosage by taking too much or too little, and mixing medicines, are all instances of drug misuse. You must pay careful attention to the specific instructions given on the label of a drug you are taking. The MiniLab on the next page shows you how to analyze such a label.

Drug abuse is the inappropriate self-administration of a drug for non-medical purposes. Drug abuse may involve use of an illegal drug, such as cocaine; use of an illegally obtained medicine, such as someone else’s prescribed drugs; or excessive use of a legal drug, such as alcohol or nicotine. Drugs abused in this way can have powerful effects on the nervous system and other systems of the body, as described in Figure 36.17.

Addiction to drugs

When a person believes he or she needs a drug in order to feel good or function normally, that person is psychologically dependent on the drug. When a person’s body develops a chemical need for the drug in order to function normally, the person is physiologically dependent. Psychological and physiological dependence are both forms of addiction.

Tolerance and withdrawal

When a drug user experiences tolerance to or withdrawal from a frequently used drug, that person is addicted to the drug. Tolerance occurs when a person needs larger or more frequent doses of a drug to achieve the same effect. The dosage increases are necessary because the body becomes less responsive to the drug. Withdrawal occurs when the person stops taking the drug and actually becomes ill.

Classes of Commonly Abused Drugs

Each class of drug produces its own special effect on the body, and its own particular symptoms of withdrawal. Table 36.1 summarizes the health effects of some commonly abused drugs.

Stimulants: Cocaine, amphetamines, caffeine, and nicotine

You already know that stimulants increase the activity of the central nervous system and the sympathetic nervous system. Increased CNS stimulation can result in mild elevation of alertness, increased nervousness, anxiety, or even convulsions.

Cocaine stimulates the CNS by working on the part of the inner brain that governs emotions and basic drives, such as hunger and thirst. When these needs are met under normal circumstances, neurotransmitters—such as dopamine—are released to reward centers and the person experiences pleasure. Cocaine artificially increases levels of these neurotransmitters in the brain. As a result, false messages are sent to reward centers indicating that a basic drive has been satisfied. The user quickly feels a euphoric high called a rush. This sense of intense pleasure and satisfaction cannot be maintained, however, and soon the effects of the drug change. Physical hyperactivity follows. Often, anxiety and depression set in.

Cocaine also disrupts the body’s circulatory system by interfering with the sympathetic nervous system. Although initially causing a slowing of the heart rate, it soon produces a great increase in heart rate and a narrowing of blood vessels, known as vasoconstriction. The result is high blood pressure. Heavy use of this drug compromises the immune system and often leads to heart abnormalities. Cocaine may affect more than just the individual who uses it. As Figure 36.18 shows, babies of addicted mothers are sometimes born already dependent on this drug.

Amphetamines are stimulants that increase levels of CNS neurotransmitters. Like cocaine, amphetamines also cause vasoconstriction, a racing heart, and increased blood pressure. Other adverse side effects of amphetamine abuse include irregular heartbeat, chest pain, paranoia, hallucinations, and convulsions.

Not all stimulants are illegal. As shown in Figure 36.19, one stimulant in particular is as close as the nearest coffee maker or candy machine. Caffeine—a substance found in coffee, some carbonated soft drinks, cocoa, and tea—is a CNS stimulant. Its effects include increased alertness and some mood elevation. Caffeine also causes an increase in heart rate and urine production, which can lead to dehydration.

Nicotine, a substance found in tobacco, is also a stimulant. By increasing the release of the hormone epinephrine, nicotine increases heart rate, blood pressure, breathing rate, and stomach acid secretion. Although nicotine is the addictive substance in tobacco, there are many other harmful chemicals found in tobacco products. Smoking cigarettes leads to an increased risk of lung cancer and cardiovascular disease. Use of chewing tobacco is associated with oral and throat cancers.

Depressants: Alcohol and barbiturates

As you already know, depressants slow down the activities of the CNS. All CNS depressants relieve anxiety, but most produce noticeable sedation.

One of the most widely abused drugs in the world today is alcohol. Easily produced from various grains and fruits, this depressant is distributed throughout a person’s body via the bloodstream. Like other drugs, alcohol affects cellular communication by influencing the release of or interacting with receptors for several important neurotransmitters in the brain. Alcohol also appears to block the movement of sodium and calcium ions across the cell membrane, a process that is important in the transmission of impulses and the release of neurotransmitters.

Tolerance to the effects of alcohol develops as a result of heavy alcohol consumption. Addiction to alcohol— alcoholism—can cause the destruction of nerve cells and brain damage. A number of organ diseases are directly attributable to chronic alcohol use. For example, cirrhosis, a hardening of the tissues of the liver, is a common affliction of alcoholics.

Barbiturates are sedatives and anti-anxiety drugs. When barbiturates are used in excess, the user’s respiratory and circulatory systems become depressed. Chronic use results in addiction.

Narcotics: Opiates

Most narcotics are opiates, derived from the opium poppy. They act directly on the brain. The most abused narcotic in the United States is heroin. It depresses the CNS, slows breathing, and lowers heart rate. Tolerance develops quickly, and withdrawal from heroin is painful.

Hallucinogens: Natural and synthetic

Natural hallucinogens have been known and used for thousands of years, but the abuse of hallucinogenic drugs did not become widespread in the United States until the 1960s, when new synthetic versions became widely available.

Hallucinogens stimulate the CNS—altering moods, thoughts, and sensory perceptions. The user sees, hears, feels, tastes, or smells things that are not actually there. This disorientation can impair the user’s judgment and place him or her in a potentially dangerous situation. Hallucinogens also increase heart rate, blood pressure, respiratory rate, and body temperature, and sometimes cause sweating, salivation, nausea, and vomiting. After large enough doses, convulsions of the body may even occur.

Unlike the hallucinogens shown in Figure 36.20, LSD—or acid—is a synthetic drug. The mechanism by which LSD produces hallucinations is still debated, but it may involve the blocking of a CNS neurotransmitter.

Anabolic steroids

Anabolic steroids are synthetic drugs that are similar to the hormone testosterone. Like testosterone, anabolic steroids stimulate muscles to increase in size. Physicians use anabolic steroids in the treatment of hormone imbalances or diseases that result in a loss of muscle mass. Abuse of anabolic steroids is associated with infertility in men, high cholesterol, and extreme mood swings.

Breaking the Habit

Once a person has become addicted to a drug, breaking the habit can be very difficult. Recall that an addiction can involve both physiological and psychological dependencies. Besides the desire to break the addiction, studies have shown that people usually need both medical and psychological therapy—such as counseling—to be successful in their treatment. Support groups such as Alcoholics Anonymous allow addicts to share their experiences in an effort to maintain sobriety. Often, people going through the same recovery are able to offer the best support.

Nicotine replacement therapy

Nicotine replacement therapy is one example of a relatively successful drug treatment approach. People who are trying to break their addiction to tobacco often go through stressful withdrawals when they stop smoking cigarettes. To ease the intensity of the withdrawal symptoms, patients wear adhesive patches that slowly release small amounts of nicotine into their bloodstream, as shown in Figure 36.21. Alternatively, pieces of nicotine-containing gum are chewed periodically to temporarily relieve cravings.

Respiration, Circulation, and Excretion

The Respiratory System

Passageways and Lungs

Your respiratory system is made of a pair of lungs and a series of passageways, each one extending deeper into your body. These passageways include the nasal passages, the throat, the windpipe, and the bronchi. When you hear the term respiratory system, you probably think of breathing. However, breathing is just one of the functions that the respiratory system carries out. Respiration, the process of gas exchange, is another important function performed by the respiratory system. Respiration includes all of the mechanisms involved in getting oxygen to the cells of your body and getting rid of carbon dioxide. Recall that cellular respiration also involves the formation of ATP within the cells.

The path air takes

The first step in the process of respiration involves taking air into your body through your nose or mouth. Air flows into the pharynx, or throat, passes the epiglottis, and moves through the larynx. It then travels down the windpipe, or trachea, a tubelike passageway that leads to two tubes, or bronchi, which lead into the lungs. When you swallow food, the epiglottis covers the entrance to the trachea, which prevents food from getting into the air passages.

Cleaning dirty air

The air you breathe is far from clean. An individual living in an urban area breathes in millions of particles of foreign matter each day. To prevent most of this material from reaching the lungs, the nasal cavity, trachea, and bronchi are lined with ciliated cells that secrete mucus. The cilia constantly beat upward in the direction of your throat, where foreign material can be swallowed or expelled by coughing or sneezing. Follow the passage of air through the respiratory system in Figure 37.1.

Alveoli: The place of gas exchange

Like the branches of a tree, each bronchus branches into bronchioles, which in turn branch into numerous microscopic tubules that eventually open into thousands of thin-walled sacs called alveoli. Alveoli are the sacs of the lungs where oxygen and carbon dioxide are exchanged by diffusion between the air and blood. The clusters of alveoli are surrounded by networks of tiny blood vessels, or capillaries. Blood in these vessels has come from the cells of the body and contains wastes from cellular respiration. Diffusion of gases takes place easily because the wall of each alveolus, and the walls of each capillary, are only one cell thick. External respiration involves the exchange of oxygen or carbon dioxide between the air in the alveoli and the blood that circulates through the walls of the alveoli, and is shown in the inset portion of Figure 37.1.

Blood transport of gases

Once oxygen from the air diffuses into the blood vessels surrounding the alveoli, it is pumped by the heart to the body cells, where it is used for cellular respiration. In an earlier chapter, you learned that cellular respiration is the process by which cells use oxygen to break down glucose and release energy in the form of ATP. Carbon dioxide is a waste product of this process. The carbon dioxide diffuses into the blood, which carries it back to the lungs.

As a result, the blood that comes to the alveoli from the body’s cells is high in carbon dioxide and low in oxygen. Carbon dioxide from the body diffuses from the blood into the air spaces in the alveoli. During exhalation, this carbon dioxide is removed from your body. At the same time, oxygen diffuses from the air in the alveoli into the blood, making the blood rich in oxygen. Use the Problem-Solving Lab on this page to find out more about how the composition of air changes as it passes through the lungs.

The Mechanics of Breathing

The action of your diaphragm and the muscles between your ribs enable you to breathe in and breathe out. Figure 37.2 shows how air is drawn in or forced out of the lungs as a result of the diaphragm’s position.

When you inhale, the muscles between your ribs contract and your rib cage rises. At the same time, the diaphragm muscle contracts, becomes flattened, and moves lower in the chest cavity. These actions increase the space in the chest cavity, which creates a slight vacuum. Air rushes into your lungs because the air pressure outside your body is greater than the air pressure inside your lungs.

When you exhale, the muscles associated with the ribs relax, and your ribs drop down in the chest cavity. Your diaphragm relaxes, returning to its resting position. The relaxation of these muscles decreases the volume of the chest cavity and forces most of the air out of the alveoli.

The alveoli in healthy lungs are elastic, they stretch as you inhale and return to their original size as you exhale. The alveoli still contain a small amount of air after you exhale. Measure your breathing rate in the BioLab at the end of this chapter.

Control of Respiration

Breathing is usually an involuntary process. It is partially controlled by an internal feedback mechanism that involves signals being sent to the medulla oblongata about the chemistry of your blood. The medulla oblongata helps maintain homeostasis. It responds to higher levels of carbon dioxide in your blood by sending nerve signals to the rib muscles and diaphragm. The nerve signals cause these muscles to contract, and you inhale. When breathing becomes more rapid, as during exercise, a more rapid exchange of gases between air and blood occurs.

The Circulatory System

Your Blood: Fluid Transport

Your blood is a tissue composed of fluid, cells, and fragments of cells. Table 37.1 summarizes information about the components of human blood. The fluid portion of blood is called plasma. Plasma is straw colored and makes up about 55 percent of the total volume of blood. Blood cells—both red and white—and cell fragments are suspended in plasma.

Red blood cells: Oxygen carriers

The round, disk-shaped cells in blood are red blood cells. Red blood cells carry oxygen to body cells. They make up 44 percent of the total volume of your blood, and are produced in the red bone marrow of your ribs, humerus, femur, sternum, and other long bones.

Red blood cells in humans have nuclei only during an early stage in each cell’s development. The nucleus is lost before the cell enters the bloodstream.

Red blood cells remain active in the bloodstream for about 120 days, then they break down and are removed as waste. Old red blood cells are destroyed in your spleen, an organ of the lymphatic system, and in your liver.

Oxygen in the blood

How is oxygen carried by the blood? Red blood cells like those shown in Figure 37.3 are equipped with an iron-containing protein molecule called hemoglobin. Oxygen becomes loosely bound to the hemoglobin in blood cells that have entered the lungs. These oxygenated blood cells carry oxygen from the lungs to the body’s cells. As blood passes through body tissues with low oxygen concentrations, oxygen is released from the hemoglobin and diffuses into the tissues.

Carbon dioxide in the blood

Hemoglobin carries some carbon dioxide as well as oxygen. You have already learned that, once biological work has been done in a cell, wastes in the form of carbon dioxide diffuse into the blood and are carried in the bloodstream to the lungs. About 70 percent of this carbon dioxide combines with water in the blood plasma to form bicarbonate. The remaining 30 percent travels back to the lungs dissolved in the plasma or attached to hemoglobin molecules that have already released their oxygen into the tissues.

White blood cells: Infection fighters

White blood cells, shown in Figure 37.4, play a major role in protecting your body from foreign substances and from microscopic organisms that cause disease. They make up only one percent of the total volume of your blood.

Blood clotting

Think about what happens when you cut yourself. If the cut is slight, you usually bleed for a short while, until the blood clots. That’s because, in addition to red and white blood cells, your blood also contains small cell fragments called platelets, which help blood clot after an injury, as shown in Figure 37.5. Platelets help link together a sticky network of protein fibers called fibrin, which forms a web over the wound that traps escaping blood cells. Eventually, a dry, leathery scab forms. Platelets are produced from cells in bone marrow and have a short life span. They are removed from the blood by the spleen and liver after only about one week.

ABO Blood Groups

If a person is injured so severely that a massive amount of blood is lost, a transfusion of blood from a second person may be necessary. Whenever blood is transfused from one person to another, it is important to know to which blood group each person belongs. You have already learned about the four human blood groups— A, B, AB, and O. You inherited the characteristics of one of these blood groups from your parents. Sometimes, the term blood type is used to describe the blood group to which a person belongs. If your blood falls into group O, for example, you are said to have type O blood.

Blood surface antigens determine blood group

Differences in blood groups are due to the presence or absence of proteins, called antigens, on the membranes of red blood cells. Antigens are substances that stimulate an immune response in the body. As you’ll learn in a later chapter, an immune response defends the body against foreign proteins. The letters A and B stand for the types of blood surface antigens found on human red blood cells.

Blood plasma contains proteins, called antibodies, that are shaped to correspond with the different blood surface antigens. The antibody in the blood plasma reacts with its matching antigen on red blood cells if they are brought into contact with one another. This reaction results in clumped blood cells that can no longer function. Each blood group contains antibodies for the blood surface antigens found only in the other blood groups—not for antigens found on its own red blood cells.

For example, if you have type A blood, you have the A antigen on your red blood cells and the anti-B antibody in your plasma. If you had anti-A anti-bodies, they would react with your own type A red blood cells. What would happen if you had type A blood and anti-A was added to it by way of a transfusion of type B blood?

An antigen-antibody reaction would occur, resulting in clumped blood cells like those shown in Figure 37.6B. Clumped blood cells cannot carry oxygen or nutrients to body cells. Similarly, if you have type B blood, you have the B antigen on your red blood cells and the anti-A antibody in your blood plasma. Figure 37.6C illustrates the antigens and antibodies present in each blood group.

Rh factor

Another characteristic of red blood cells involves the presence or absence of an antigen called Rh, or Rhesus factor. Rh factor is an inherited characteristic. People are Rh positive (Rh+) if they have the Rh antigen factor on their red blood cells. They are Rh negative (Rh—) if they don’t.

Rh factor can cause complications in some pregnancies. The problem begins when an Rh— mother becomes pregnant with an Rh+ baby. At birth, the Rh+ baby’s blood mixes with the Rh— blood of the mother, as Figure 37.7A illustrates. Upon exposure to the baby’s Rh+ antigen factor, the mother will make anti-Rh+ antibodies like those shown in Figure 37.7B. If the mother becomes pregnant again, these anti- bodies can cross the placenta. If the new fetus is Rh+, the anti-Rh+ antibodies from the mother will destroy red blood cells in the fetus, as shown in Figure 37.7C.

Prevention of this problem is possible. When the Rh+ fetus is 28 weeks old, and again shortly after the Rh+ baby is born, the Rh— mother is given a substance that prevents the production of Rh antibodies in her blood. As a result, the next fetus will not be in danger.

Your Blood Vessels: Pathways of Circulation

Because blood is fluid, it must be channeled through blood vessels like those shown in Figure 37.8. The three main types of blood vessels are arteries, capillaries, and veins. Each is different in structure and function.

Arteries are large, thick-walled, muscular, elastic blood vessels that carry blood away from the heart.

The blood that they carry is under great pressure. As the heart contracts, it pushes blood through the arteries. Each artery’s elastic walls expand slightly. As the heart relaxes, the artery shrinks a bit, which also helps push the blood forward. As a result, blood surges through the arteries in pulses that correspond with the rhythm of the heartbeat.

After the arteries branch off from the heart, they divide into smaller arteries that, in turn, divide into even smaller vessels called arterioles. Arterioles enter tissues, where they branch into the smallest blood vessels, the capillaries. Capillaries are microscopic blood vessels with walls that are only one cell thick. These vessels are so tiny that red blood cells must move through them in single file. Capillaries form a dense network that reaches virtually every cell in the body. Thin capillary walls enable nutrients and gases to diffuse easily between blood cells and surrounding tissue cells.

As blood leaves the tissues, the capillaries join to form slightly larger vessels called venules. The venules merge to form veins, the large blood vessels that carry blood from the tissues back toward the heart. Blood in veins is not under pressure as great as that in the arteries. In some veins, especially those in your arms and legs, blood travels uphill against gravity. These veins are equipped with valves that prevent blood from flowing backward. Figure

37.9 shows how these valves function. When your skeletal muscles contract, the top valves open, and blood is forced toward the heart. When the skeletal muscles relax, the top valves close to prevent blood from flowing backward, away from the heart.

Your Heart:

The Vital Pump

The thousands of blood vessels in your body would be of little use if there were not a way to move blood through them. The main function of the heart is to keep blood moving constantly throughout the body. Well adapted for its job, the heart is a large organ made of cardiac muscle cells that are rich in energy-producing mitochondria.

All mammalian hearts, including yours, have four chambers. The two upper chambers of the heart are the atria. The two lower chambers are the ventricles. The walls of each atrium are thinner and less muscular than those of each ventricle. As you will see, the ventricles perform more work than the atria, a factor that helps explain the thickness of their muscles. Each atrium pumps blood into the corresponding ventricle. The left ventricle pumps blood to the entire body, so its muscles are thicker than those of the right ventricle, which pumps blood to the lungs. As a result, your heart is somewhat lopsided.

Blood’s path through the heart

Blood enters the heart through the atria and leaves it through the ventricles. Both atria fill up with blood at the same time. The right atrium receives oxygen-poor blood from the head and body through two large veins called the venae cavae. The left atrium receives oxygen-rich blood from the lungs through four pulmonary veins. These veins are the only veins that carry blood rich in oxygen. After they have filled with blood, the two atria then contract, pushing the blood down into the two ventricles.

After the ventricles have filled with blood, they contract simultaneously. When the right ventricle contracts, it pushes the oxygen-poor blood from the right ventricle out of the heart and toward the lungs through the pulmonary arteries. These arteries are the only arteries that carry blood poor in oxygen. At the same time, the left ventricle forcefully pushes oxygen-rich blood from the left ventricle out of the heart through the aorta to the arteries of the body. The aorta is the largest blood vessel in the body. See Figure 37.10 on the next page to learn how a drop of blood moves through the heart.

Heartbeat regulation

Each time the heart beats, a surge of blood flows from the left ventricle into the aorta and then into the arteries. Because the radial artery in the arm and carotid arteries near the jaw are fairly close to the surface of the body, the surge of blood can be felt as it moves through them. This surge of blood through an artery is called a pulse. Find out more about how the pulse is used to measure heart rate by conducting the MiniLab on this page.

The heart rate is set by the pace-maker, a bundle of nerve cells located at the top of the right atrium. This pacemaker generates an electrical impulse that spreads over both atria. The impulse signals the two atria to contract at almost the same time. The impulse also triggers a second set of cells at the base of the right atrium to send the same electrical impulse over the ventricles, causing them to contract. These electrical signals can be measured and recorded by a machine called an electrocardiograph. This recording, shown in Figure 37.11, is called an electrocardiogram (ECG).

The ECG is an important tool used in diagnosing abnormal heart rhythms or patterns. Each peak or valley in the ECG tracing represents a particular electrical activity that takes place during a heartbeat. You can learn how ECG tracings are analyzed by carrying out the Problem-Solving Lab on this page.

Blood pressure

A pulse beat represents the pressure that blood exerts as it pushes against the walls of an artery. Blood pressure is the force that the blood exerts on the blood vessels. As Figure 37.12 shows, blood pressure rises and falls as the heart contracts and then relaxes.

Blood pressure rises sharply when the ventricles contract, pushing blood through the arteries. The high pressure is called systolic pressure. Blood pressure then drops dramatically as the ventricles relax. The lowest pressure occurs just before the ventricles contract again and is called diastolic pressure.

Whereas the pacemaker controls the heartbeat, a portion of the brain called the medulla oblongata regulates the rate of the pacemaker, speeding or slowing its nerve impulses. If the heart beats too fast, sensory cells in arteries near the heart become stretched. Via the nervous system, these cells send a signal to the medulla oblongata, which in turn sends signals that slow the pacemaker. If the heart slows down too much, blood pressure in the arteries drops, signalling the medulla oblongata to speed up the pacemaker and increase the heart rate.

The Urinary System

Kidneys: Structure and Function

The urinary system is made up of two kidneys, a pair of ureters, the urinary bladder, and the urethra, which you can see in Figure 37.13. The kidneys filter the blood to remove wastes from it, thus maintaining the homeostasis of body fluids. Your kidneys are located just above the waist, behind the stomach. One kidney lies on each side of the spine, partially surrounded by ribs. Each kidney is connected to a tube called a ureter, which leads to the urinary bladder. The urinary bladder is a smooth muscle bag that stores a solution of wastes.

Nephron: The unit of the kidney

Each kidney is made up of about one million tiny filters. A filter is a device that removes impurities from a solution. Each filtering unit of the kidney is called a nephron. A nephron is shown in Figure 37.14.

Blood entering a nephron carries wastes produced by body cells. As blood enters the nephron, it is under high pressure and immediately flows into a bed of capillaries called the glomerulus. Because of the pressure, water, glucose, vitamins, amino acids, protein waste products (called urea), salts, and ions from the blood pass out of the capillaries into a part of the nephron called the Bowman’s capsule. Blood cells and most proteins are too large to pass through the walls of a capillary, so these components stay within the blood vessels.

The liquid forced into the Bowman’s capsule passes through a narrow, U-shaped tubule. As the liquid moves along the tubule, most of the ions and water, and all of the glucose and amino acids, are reabsorbed into the bloodstream. This reabsorption of substances is the process by which the body’s water is conserved and homeostasis is maintained. Small molecules, including water, move back into the capillaries by diffusion. Other molecules and ions move back into the capillaries by active transport.

The formation of urine

The liquid that remains in the tubules—composed of waste molecules and excess water and ions—is urine. The production of urine is shown in Figure 37.15. You produce about 2 L of urine a day. This waste fluid flows out of the kidneys, through the ureter, and into the urinary bladder, where it may be stored. Urine passes from the urinary bladder out of the body through a tube called the urethra.

The Urinary System and Homeostasis

The major waste products of cells are nitrogenous wastes, which come from the breakdown of proteins. These wastes include ammonia and urea. Both compounds are toxic to your body and, therefore, must be removed from the blood regularly.

In addition to removing these wastes, the kidneys control the level of sodium in blood by removing and reabsorbing sodium ions. This helps control the osmotic pressure of the blood. The kidneys also regulate the pH of blood by filtering out hydrogen ions and allowing bicarbonate to be reabsorbed back into the blood. Glucose is a sugar that is not usually filtered out of the blood by the kidneys. Individuals who have the disease known as diabetes have excess levels of glucose in their blood. The MiniLab on this page shows how urine is used to test for diabetes.

Reproduction and Development

Human Reproductive Systems

Human Male Anatomy

The ultimate result of the reproductive process is the formation and union of egg and sperm, development of the fetus, and birth of the infant. The organs, glands, and hormones of the male reproductive system are instrumental in meeting this goal. Their main functions are the production of sperm—the male sex cells—and their delivery to the female.

Where sperm form

Sperm production takes place in the testes, which are located in the scrotum. The scrotum is a sac that contains the testes and is suspended directly behind the base of the penis. Before birth, the testes form in the embryo’s abdomen and then descend into the scrotum. Because sperm can develop only in an environment with a temperature about 2–3°C lower than normal body temperature, the scrotum is positioned outside the abdomen. Muscles in the walls of the scrotum help maintain the proper temperature. The muscles contract in response to cold temperatures, pulling the scrotum closer to the body for warmth. The muscles relax in response to warm temperatures, lowering the scrotum to allow air to circulate and cool both testes and sperm. Figure 38.1 on the next page shows the organs and glands of the male reproductive system.

Within each testis is a fine network of highly coiled tubes. Sperm are produced by meiosis of the cells that line these tubes. Recall that meiosis produces haploid cells. When a single cell in the testis divides by meiosis, it produces four haploid cells. All four of these cells develop into mature sperm over a period of about 74 days. A sexually mature human male can produce about 300 million mature sperm per day, each day of his life.

As you can see in Figure 38.2, a sperm is highly adapted for reaching and entering the female egg. The head portion of a sperm contains the nucleus and is covered by a cap containing enzymes that help penetrate the egg. A number of mitochondria are found in the midpiece of the sperm; they provide energy for locomotion. The tail is a typical flagellum that propels the sperm along its way. Sperm usually live for about 48 hours inside the female reproductive tract.

How sperm leave the testes

Before the sperm mature, they move out of the testes through a series of coiled ducts that empty into a single tube called the epididymis. The epididymis is a coiled tube within the scrotum in which the sperm complete their maturation.

When sperm are released from the epididymis, they enter the vas deferens, where they are stored for as long as two or three months until they are released from the body.

The vas deferens is a duct that transports sperm from the epididymis toward the ejaculatory ducts and the urethra. Peristaltic contractions of the vas deferens force the sperm along. The urethra is a tube in the penis that transports sperm out of the male’s body. The urethra also transports urine from the urinary bladder. A muscle located at the base of the bladder prevents urine and sperm from mixing.

Fluids that help transport sperm

As sperm travel from the testes, they mix with fluids that are secreted by several different glands. The seminal vesicles are a pair of glands located at the base of the urinary bladder. They secrete a mucouslike fluid into the vas deferens. The fluid is rich in the sugar fructose, which provides energy for the sperm cells.

The prostate gland is a single, doughnut-shaped structure that lies below the urinary bladder and surrounds the top portion of the urethra. The prostate secretes a thinner, alkaline fluid that helps sperm move and survive. Two tiny bulbourethral glands are located beneath the prostate. These glands secrete a clear, sticky, alkaline fluid that protects sperm by neutralizing the acidic environment of the male urethra and female vagina. The combination of sperm and all of these fluids is called semen.

Puberty in Males

In an earlier chapter, you learned that the glands of the endocrine system release hormones, which play a key role in the regulation of body functions, metabolism, and homeostasis. Hormones also control the development and activity of the male reproductive system.

Hormones and male puberty

In the early teen years, changes to a child’s body begin to occur. Puberty begins. **Puberty** refers to the time when secondary sex characteristics begin to develop so that sexual maturity—the potential for sexual reproduction—is reached. Figure 38.3 shows several males at a variety of ages. The physical transition of the body from child to young adult occurs during **puberty**. The changes associated with puberty are controlled by sex hormones secreted by the endocrine system.

Hormones and the male reproductive system

In males, the onset of puberty causes the hypothalamus to produce several kinds of hormones that interact with the pituitary gland, which influences many physiological processes of the body. As shown in Figure 38.4A, the hypothalamus secretes a hormone that causes the pituitary gland to re- lease two other hormones: follicle-stimulating hormone (FSH) and luteinizing hormone (LH). When released into the bloodstream, FSH and LH are transported to the testes. In the testes, FSH causes the production of sperm cells. LH causes endocrine cells in the testes to produce the male hormone, testosterone, which in turn influences sperm cell production.

The levels of these hormones in the body are regulated by a negative-feed-back system. As the testosterone levels in the blood increase, the production of FSH and LH is inhibited, or decreased. Increased production of sperm in the testis also feeds back into the system to inhibit production of FSH and LH, as Figure 38.4B illustrates. When testosterone levels in the blood drop, production of FSH and LH increases.

Testosterone is the steroid hormone responsible for the growth and development of secondary sex characteristics in a male. These characteristics include growth and maintenance of male sex organs; the production of sperm; an increase in body hair, especially on the face, under the arms, and in the pubic area; an increase in muscle mass; increased growth of the long bones of the arms and legs; and deepening of the voice.

Human Female Anatomy

The main functions of the female reproductive system are to produce eggs, which are the female sex cells, to receive sperm, and to provide an environment in which a fertilized egg can develop. Egg production takes place in the two ovaries. Each ovary is about the size and shape of an almond. One ovary is located on each side of the lower part of the abdomen. As you can see in Figure 38.5, the open end of an oviduct is located close to each ovary. The oviduct is a tube that transports eggs from the ovary to the uterus. Peristaltic contractions of the muscles in the wall of the oviduct combine with beating cilia to move the egg through the tube.

You learned earlier that female mammals have a uterus in which the fetus develops during pregnancy. The human uterus is situated between the urinary bladder and the rectum and is the size and shape of an inverted pear. The uterine wall is composed of three layers: an outer layer of connective tissue; a thick, muscular middle layer; and a thin, inner lining called the endometrium. The lower end of the uterus, called the cervix, tapers to a narrow opening into the vagina, which is a passageway to the outside of the female’s body.

Puberty in Females

As in males, puberty in females begins when the hypothalamus signals the pituitary to produce and release the hormones FSH and LH. These are the same hormones that are produced in males; however, in females, FSH stimulates the development of follicles in the ovary. A follicle is a group of epithelial cells that surround a developing egg cell. FSH also causes the release of the hormone estrogen from the ovary. Estrogen is the steroid hormone responsible for the secondary sex characteristics of females. These characteristics include the growth and maintenance of female sex organs, an increase in the growth rates of the long bones of the arms and legs, and a broadening of the hips.

Other changes that take place include an increase in body hair, especially under the arms and in the pubic area; an increase in fat deposits in the breasts, buttocks, and thighs; and the onset of the menstrual cycle.

Production of eggs

Recall that sperm production does not begin in males until they reach **puberty**, after which time it continues for the rest of their lives. Egg production is different. Even before a female is born, her body begins to develop eggs. During this prenatal period, cells in her ovaries divide until the first stage of meiosis, **prophase I**, is reached. At this point, the cells go into a resting stage. At birth, a female’s ovaries contain about two million of these potential eggs, which are called primary oocytes. Many of these break down, or degenerate. At **puberty**, a female’s ovaries contain about 40 000 primary oocytes. How does the production of sperm differ from the production of egg cells? To find out, look at Figure 38.7.

How eggs are released

About once a month, beginning at **puberty**, the process of meiosis starts up again in several of the prophase I cells. Each cell completes meiosis I and begins meiosis II. During meiosis II, one of the egg cells ruptures from the ovary and passes into the oviduct. The process of the egg rupturing through the ovary wall and moving into the oviduct is called ovulation. A total of about 400 eggs are ovulated during the reproductive life of a female. Fertilization, if it takes place, usually occurs in the oviduct. Figure 38.6 shows the process leading to ovulation.

The Menstrual Cycle

The series of changes in the female reproductive system that includes producing an egg and preparing the uterus for receiving it is known as the menstrual cycle. The entire menstrual cycle repeats about once a month. Once an egg has been released during ovulation, the part of the follicle that remains in the ovary develops into a structure called the corpus luteum. The corpus luteum secretes the hormones estrogen and progesterone. Progesterone causes changes to occur in the lining of the uterus that prepare it for receiving a fertilized egg. The menstrual cycle begins during **puberty** and continues for 30 to 40 years, until menopause. At menopause, the female stops releasing eggs and the secretion of female hormones decreases.

The length of each menstrual cycle varies from female to female, but the average is 28 days. If the egg released at ovulation is not fertilized, the lining of the uterus is shed, causing some bleeding for a few days. The entire menstrual cycle can be divided into three phases: **the flow phase**, the **follicular phase**, and the **luteal phase**, illustrated in Figure 38.8. The timing of each phase of the menstrual cycle correlates with hormone output from the pituitary gland, changes in the ovary, and changes in the uterus. Figure 38.9 shows how internal feed-back controls hormone secretion during the menstrual cycle. Carry out the Problem-Solving Lab to find out how the phases of the menstrual cycle can vary in length.

Flow phase

Day 1 of the menstrual cycle is the day menstrual flow begins. Menstrual flow is the shedding of blood, tissue fluid, mucus, and epithelial cells that made up the lining of the uterus, the endometrium. This flow passes from the uterus through the cervix and the vagina to the outside of the body. Contractions of the uterine muscle help expel the uterine lining and can cause discomfort in some females.

Generally, menstrual flow ends by day 5 of the cycle. During the **flow phase**, the level of FSH in the blood begins to rise, and another follicle in one of the ovaries begins to mature as meiosis of the prophase I cell proceeds.

Follicular phase

The second phase of the menstrual cycle is more varied in length than the other phases. In a 28-day cycle, it lasts from about day 6 to day 14. As the follicle containing a primary oocyte continues to develop, it secretes estrogen, which stimulates the repair of the endometrial lining of the uterus. The endometrial cells undergo mitosis, and the lining thickens. The steady increase in estrogen also feeds back to the hypothalamus and pituitary gland, which slows the production of FSH and LH. Just before ovulation, estrogen levels peak, stimulating a sudden, sharp increase in the release of LH.

Ovulation occurs at about day 14. The sharp increase in LH causes the follicle to rupture, releasing the egg into the oviduct. At this time, the female’s body temperature rises about 0.5°C.

In addition, the cells of the cervix produce large amounts of mucus. Some females also experience discomfort in the area of one or both ovaries around the time of ovulation.

Luteal phase

The last stage of the menstrual cycle, from days 15 to 28, is named the **luteal phase**, for the corpus luteum. During the **luteal phase**, LH stimulates the corpus luteum to develop from the ruptured follicle. The corpus luteum produces progesterone and some estrogen. Progesterone increases the blood supply of the endometrium, causing it to accumulate lipids and tissue fluid. These changes correspond to the arrival of a fertilized egg. Through negative feedback, progesterone prevents the production of LH.

If the egg is not fertilized, the rising levels of progesterone and estrogen from the corpus luteum cause the hypothalamus to inhibit the release of FSH and LH. The corpus luteum degenerates and stops secreting progesterone or estrogen. As hormone levels drop, the thick lining of the uterus begins to shed. If fertilization occurs, as shown in Figure 38.10, the endometrium begins secreting a fluid rich in nutrients for the embryo.

Development Before Birth

Fertilization and Implantation

After an egg ruptures from a follicle, it is able to stay alive for about 24 hours. For fertilization to occur, sperm must be present in the oviduct at some point during those first hours after ovulation. Sperm enter the vagina of the female’s reproductive system when strong, muscular contractions ejaculate semen from the male’s penis. Between 300 and 500 million sperm are forced out of the male’s penis and into the female’s vagina during intercourse. Because sperm can live for 48 hours after ejaculation, fertilization can occur if intercourse occurs anywhere from a few days before to a day after ovulation.

One sperm plus one egg

How is it possible that, of the millions of sperm released into the vagina during ejaculation, only one fertilizes the mature egg? One reason is that the fluids secreted by the vagina are acidic and destroy most of the delicate sperm. Yet, some sperm survive because of the neutralizing effect of semen. The surviving sperm swim up the vagina into the uterus. Of the sperm that reach the uterus, only a few hundred pass into the two oviducts. The egg is present in one of them.

The head of the sperm contains enzymes that help the sperm penetrate the egg. Once one sperm has entered the egg, the electrical charge of the egg’s membrane changes, preventing other sperm from entering the egg.

The sperm’s nucleus then combines with the egg’s nucleus to form a zygote that contains a complete array of genetic information. Examine the structures of a sperm and egg cell by completing the MiniLab on this page.

The fertilized egg travels to the uterus

As the zygote passes down the oviduct, it begins to divide by repeated mitotic division. During its journey pictured in Figure 38.11, the zygote obtains nutrients from fluids secreted by the mother. By the sixth day, the zygote passes into the uterus. Continuous cell divisions result in the formation of a hollow ball of cells called a blastocyst. Blastocyst is the term used when discussing human embryonic development. Recall that the term blastula is used for the embryonic development of other animals.

The blastocyst attaches to the uterine lining six days after fertilization. Attachment of the blastocyst to the lining of the uterus is called implantation. A small, inner mass of cells within the blastocyst will soon become a human embryo.

Embryonic Membranes and the Placenta

You have already learned about the importance of the amniotic egg to the evolutionary advancement of animals. Membranes that are similar to those of the amniotic egg form around the human embryo, protecting and nourishing it. The amnion is a thin, inner membrane filled with a clear, watery amniotic fluid. Amniotic fluid serves as a shock absorber and helps regulate the body temperature of the developing embryo.

The allantois membrane is an outgrowth of the digestive tract of the embryo. Blood vessels of the allantois form the umbilical cord, a ropelike structure that attaches the embryo to the wall of the uterus. The chorion is the outer membrane that surrounds the amniotic sac and the embryo within it. About 12 days after fertilization, fingerlike projections of the chorion, called chorionic villi, begin to grow into the uterine wall. The chorionic villi combine with part of the uterine lining to form the placenta, as shown in Figure 38.12.

Exchange between embryo and mother

To survive and grow, the embryo must obtain the proper nutrients and eliminate the wastes its cells produce. The placenta delivers nutrients to the embryo and carries wastes away.

In the placenta, blood vessels from the mother’s uterine wall lie close to the blood vessels of the embryo’s chorionic villi. Although they are close together, they are not directly connected to one another. Instead, oxygen and nutrients transported by the mother’s blood diffuse into the blood vessels of the chorionic villi in the placenta. These vital substances are then carried by the blood in the umbilical cord to the embryo. In turn, waste products from the embryo travel in the umbilical blood vessels to the placenta. Here they diffuse out of the vessels in the chorionic villi into the blood of the mother. These waste products are then removed by the mother’s excretory system.

Hormonal maintenance of pregnancy

Remember that estrogen, and especially progesterone, cause the uterine lining to thicken in preparation for implantation. Once the blastocyst implants, the chorionic membrane of the embryo starts to secrete the hormone human chorionic gonadotropin (hCG). This hormone keeps the corpus luteum alive so that it continues to secrete progesterone. Learn how this hormone is an indicator of pregnancy in the BioLab at the end of this chapter. By the third or fourth month, the placenta takes over for the corpus luteum, secreting enough estrogen and progesterone to maintain the pregnancy.

Fetal Development

When you think of an embryo growing within the mother’s body, you may not realize that its development involves three different processes: growth, development, and cellular differentiation. Growth refers to the actual increase in the number of cells. Development follows as the multiplying cells move and arrange themselves into specific organs. In addition, each cell differentiates to perform specific tasks and functions. All three processes begin with fertilization.

Pregnancy in humans usually lasts about 280 days, calculated from the first day of the mother’s last menstrual period. The baby actually develops for about 266 days, calculated from the time of fertilization to birth. This time span is divided into three trimesters, each about three months in length. Each trimester brings significant advancement in the development of the embryo and fetus.

First trimester:

Organ systems form

During the first trimester, all the organ systems of the embryo begin to form. A five-week embryo is shown in Figure 38.13A. During this time of development, the woman may not even realize she is pregnant. Yet, the first seven weeks following fertilization are critical because during this time, the embryo is more sensitive to outside influences—such as alcohol, tobacco, and other drugs that cause malformations—than at any other time. Other environmental factors that can adversely affect the health of a developing embryo include exposure to toxins, such as pesticides, or maternal infections such as chicken pox or measles. Lack of adequate amounts of folic acid, a vitamin found in green leafy vegetables, broccoli, and dried beans, is associated with neural tube defects.

By the eighth week, all the organ systems have been formed, and the embryo is now referred to as a fetus. You can see this stage of fetal development in Figure 38.13B. At the end of the first trimester, the fetus weighs about 28 g and is about 7.5 cm long from the top of its head to its buttocks. The gender of the fetus can be determined by the appearance of the external sex organs when viewed by ultrasound.

Second trimester:

A time of growth

For the most part, fetal development during the next three months is limited to body growth. Growth is rapid during the fourth month, but then slows by the beginning of the fifth month. During the fifth month, fetal movements can be felt by the mother. In the sixth month of development, the fetus’s eyes open and eyelashes form.

At this point, it is possible for the fetus to survive outside the uterus, but it would require a great amount of medical assistance, and the mortality rate is high. The fetus’s metabolism cannot yet maintain a constant body temperature, and its lungs have not matured enough to provide a regular respiratory rate. Figure 38.13C shows a fetus during the second trimester. By the end of the second trimester, the fetus weighs about 650 g and is about 34 cm long.

Third trimester: Continued growth

During the last trimester, the mass of the fetus more than triples. The fetus continues to kick, stretch, and move freely within the amniotic cavity. During the eighth month, fat is deposited beneath the skin, which will help insulate the newborn.

During the final weeks of pregnancy, the fetus grows large enough to fill the space within the embryonic membranes. By the end of the third trimester, the fetus weighs about 3300 g and is about 51 cm long. All of its body systems have developed, and it can now survive independently outside the uterus.

Genetic disorders can be predicted

With our increasing knowledge of human heredity and advancing technology, medical science is now better able to determine the risk that certain genetic disorders will occur in individuals. Advances in the field of genetics have allowed scientists to identify genes that carry certain genetic disorders, such as cystic fibrosis, Huntington’s disease, and Tay-Sachs disease. Early identification or detection of certain genetic disorders, such as phenylketonuria (PKU) and cystic fibrosis, is essential to the treatment of these disorders. As services, such as genetic counseling, become more available, some people may consider visiting a genetic counselor to discover additional information about their genetic makeup before having children.

A genetic counselor, like the one shown in Figure 38.14, has a medical background with additional training in genetics. Sometimes, a team of professionals works with potential parents. The team may include geneticists, clinical psychologists, social workers, and other consultants. A genetic counselor will start by recording the medical histories of both parents and their families. These histories may include pedigrees, biochemical analyses of blood, karyotypes, and DNA analysis. Once a counselor has collected and analyzed all the available information, he or she explains to the couple their risk factors for giving birth to children with genetic disorders.

Birth, Growth, and Aging

Birth

Birth is the process by which a fetus is pushed out of the uterus and the mother’s body and into the outside world, like the newborn in Figure 38.15. What triggers the onset of birth is not fully understood. Different hormones released from the pituitary gland, uterus, and placenta may all be involved in stimulating the uterus. Birth occurs in three recognizable stages: **dilation**, **expulsion**, and the **placental stage**.

Dilation of the cervix

The physiological and physical changes a female goes through to give birth are called labor. Labor begins with a series of mild contractions of the uterine muscles. These contractions are stimulated by oxytocin, a hormone released by the pituitary. The contractions open, or dilate, the cervix to allow for passage of the baby, as shown in Figure 38.16A. As labor progresses, the contractions begin to occur at regular intervals and intensify as the time between them shortens. When the opening of the cervix is about 10 cm, it is fully dilated. Usually, the amniotic sac ruptures and the amniotic fluid is released through the vagina, which is also referred to as the birth canal.

Expulsion of the baby

Expulsion occurs when the involuntary uterine contractions become so forceful that they push the baby through the cervix into the birth canal. The mother assists with expelling the baby by contracting her abdominal muscles in time with the uterine contractions. As shown in Figure 38.16B, the baby moves from the uterus, through the birth canal, and out of the mother’s body. The **expulsion stage** usually lasts from 20 minutes to an hour.

Placental stage

As shown in Figure 38.16C, within ten to 15 minutes after the birth of the baby, the placenta separates from the uterine wall and is expelled with the remains of the embryonic membranes. Collectively, these materials are known as the afterbirth. The uterine muscles continue to contract forcefully, constricting uterine blood vessels to prevent the mother from bleeding excessively. After the baby is born, the umbilical cord is clamped and cut near the baby’s abdomen. The bit of cord that is left eventually dries up and falls off, leaving an abdominal scar called the navel.

Growth and Aging

Once a baby is born, growth continues and learning begins. Human growth varies with age and is somewhat gender dependent.

A hormone controls growth

Human growth is regulated by human growth hormone (hGH), a protein secreted by the pituitary gland. Although hGH causes all body cells to grow, it acts principally on the skeleton and skeletal muscles. The hormone works by increasing the rate of protein synthesis and the metabolism of fat molecules. Other hormones that influence growth include thyroxin, estrogen, and testosterone.

The first stage of growth: Infancy

The first two years of life are known as infancy. During infancy, a child shows tremendous growth as well as an increase in physical coordination and mental development. Generally, an infant will double its birth weight by the time it is five months of age, and triple its weight in a year. By two years of age, most infants weigh approximately four times their birth weight. During this time, the infant learns to control its limbs, roll over, sit, crawl, and walk. By the end of infancy, the child also utters his or her first words.

From child to adult

Childhood is the period of growth and development that extends from infancy to adolescence, when **puberty** begins. Physically, the childhood years are a period of relatively steady growth. Mentally, a child develops the ability to reason and to solve problems.

**Adolescence** follows childhood. At **puberty**, the onset of adolescence, there is often a growth spurt, sometimes quite a dramatic one. Increases of 5 to 8 cm of height in one year are not uncommon in teenage boys. During the teen years, adolescents reach their maximum physical stature, which is determined by heredity, nutrition, and their environment. By the time a young person reaches adulthood, his or her organs have reached their maximum mass, and physical growth is complete. You can see in Figure 38.17 how the physical appearance of a person changes from birth to adulthood.

An adult ages

As an adult ages, his or her body undergoes many distinct changes. Metabolism and digestion become slower. The skin loses some of its elasticity, and less pigment is produced in the hair follicles; that is, the hair turns white. Bones often become thinner and more brittle, resulting in an increased risk of fracture. Stature may shorten because the disks between the vertebrae become compressed. Vision and hearing might diminish, but, as Figure 38.18 shows, many people continue to be both intellectually and physically active as they grow older.

Immunity from Disease

The Nature of Disease

What is an infectious disease?

The cold virus causes a disease—a change that disrupts the homeostasis in the body. Disease-producing agents such as bacteria, protozoans, fungi, viruses, and other parasites are called pathogens. The main sources of pathogens are soil, contaminated water, and infected animals, including other people.

Not all microorganisms are pathogenic. In fact, the presence of some microorganisms in your body is beneficial. At birth, microorganisms establish themselves on your skin and in your upper respiratory system, lower urinary and reproductive tracts, and lower intestinal tract. Figure 39.1 shows some common bacteria that live on your skin.

These microorganisms have a symbiotic relationship with your body. Microorganisms that colonize the body help maintain equilibrium within the body by keeping harmful bacteria and other microorganisms from growing. If conditions change and the beneficial organisms are eliminated, pathogens can establish themselves and cause infection and disease. If the beneficial organisms enter areas of the body where they are not normally found or if a person becomes weakened or injured, these formerly harmless organisms can become potential pathogens.

Any disease caused by the presence of pathogens in the body is called an infectious disease. Table 39.1 lists some of the infectious diseases that occur in humans.

Determining What Causes a Disease

One of the first problems scientists face when studying a disease is finding out what causes the disease. Not all diseases are caused by pathogens. Some disorders are inherited, such as hemophilia, which is caused by a recessive allele on the X chromosome, and sickle-cell anemia. Others, such as osteoarthritis, may be caused by wear and tear on the body as it ages. Some diseases, such as cirrhosis, are caused by exposure to chemicals or toxins such as alcohol that destroy liver cells. Other diseases can be caused by malnutrition. Scurvy, which results in poor wound healing, swollen gums, and loosening of teeth, is caused by a deficiency of vitamin C. Pathogens cause infectious diseases and some cancers. In order to determine which pathogen causes a specific disease, scientists follow a standard set of procedures.

First pathogen identified

The first proof that pathogens actually cause disease came from the work of Robert Koch (KAHK) in 1876. Koch, a German physician, was looking for the cause of anthrax, a deadly disease that affects mainly cattle and sheep but can also occur in humans. Koch discovered a rod-shaped bacterium in the blood of cattle that had died of anthrax. He cultured the bacteria on nutrients and then injected samples of the culture into healthy animals. When these animals became sick and died, Koch isolated the bacteria in their blood and compared them with the bacteria he had originally isolated from anthrax victims. He found that the two sets of blood cultures contained the same bacteria.

A procedure to establish the cause of a disease

Koch established experimental steps, shown in Figure 39.2, for directly relating a specific pathogen to a specific disease. These steps, first published in 1884, are known today as Koch’s postulates:

1. The pathogen must be found in the host in every case of the disease.

2. The pathogen must be isolated from the host and grown in a pure culture—that is, a culture containing no other organisms.

3. When the pathogen from the pure culture is placed in a healthy host, it must cause the disease.

4. The pathogen must be isolated from the new host and be shown to be the original pathogen.

Exceptions to Koch’s postulates

Although Koch’s postulates are useful in determining the cause of most diseases, some exceptions exist. Some organisms, such as the pathogenic bacterium that causes the sexually transmitted disease syphilis, have never been grown on an artificial medium. Viral pathogens also cannot be cultured this way because they multiply only within cells. As a result, living tissue must be used as a culture medium for viruses.

The Spread of Infectious Diseases

For a disease to continue and spread, there must be a continual source of the disease organisms. This source can be either a living organism or an inanimate object on which the pathogen can survive.

Reservoirs of pathogens

The main source of human disease pathogens is the human body itself. In fact, the body can be a reservoir of disease-causing organisms. People may transmit pathogens directly or indirectly to other people. Sometimes, people can harbor pathogens without exhibiting any signs of the illness and unknowingly transmit the pathogens to others. These people are called carriers and are a significant reservoir of infectious diseases.

Other people may unknowingly pass on a disease during its first stage, before they begin to experience symptoms. This **symptom-free period**, while the pathogens are multiplying within the body, is called an **incubation period**. Humans can unknowingly pass on the pathogens that cause colds, streptococcal throat infections, and sexually transmitted diseases (STDs) such as gonorrhea and AIDS during the incubation periods of these diseases.

Animals are other living reservoirs of microorganisms that cause disease in humans. For example, some types of influenza, commonly known as the flu, rabies, and Lyme disease are transmitted to humans from animals. The major nonliving reservoirs of infectious diseases are soil and water. Soil harbors pathogens such as fungi and the bacterium that causes botulism, a type of food poisoning. Water contaminated by feces of humans and other animals is a reservoir for several pathogens, especially those responsible for intestinal diseases.

Transmission of disease

Pathogens can be transmitted to a host from reservoirs in four main ways: by direct contact, by an object, through the air, or by an intermediate organism called a vector. Figure 39.3 illustrates several routes of transmission.

The common cold, influenza, and STDs are spread by direct contact. STDs, such as genital herpes and the virus that causes AIDS, are usually transmitted by the exchange of body fluids, especially during sexual intercourse.

Bacteria and other microorganisms can be present on nonliving objects such as money, toys, or towels. Transmission occurs when people unknowingly handle contaminated objects. This type of transmission can be prevented by thoroughly cleaning objects such as eating utensils and countertops that can harbor pathogens, and by washing your hands often throughout each day.

Airborne transmission of a disease can occur when a person coughs or sneezes, spreading pathogens contained in droplets of mucus into the air. Streptococcus, the bacterium that causes strep throat infections, and the virus that causes measles are two examples of disease-causing organisms that can be spread through the air.

Diseases transmitted by vectors are most commonly spread by insects and arthropods. Diseases such as malaria and the West Nile virus are transmitted by mosquitoes. Lyme disease and Rocky Mountain spotted fever are diseases that are transmitted by ticks. The bubonic plague—a disease that swept through Europe in the 1400s, killing up to one-quarter of the population—was caused by a bacterium that was transmitted from infected rats to humans by fleas. Flies also are significant vectors of disease. They transmit pathogens when they land on infected materials, such as animal wastes, and then land on fresh food that is eaten by humans. A current international concern is the intentional spreading of disease organisms such as anthrax spores, as occurred in the fall of 2001 in the United States through contaminated mail. To learn more about how diseases are spread, refer to the Problem-Solving Lab here and the MiniLab on the next page.

What causes the symptoms of a disease?

When a pathogen invades your body, it encounters your immune system, which consists of individual cells, tissues, and organs that work together to protect the body against organisms that may cause infection or disease. If the pathogen overcomes the defenses of your immune system, it can metabolize and multiply, causing damage to the tissues it has invaded, and even killing host cells.

Damage to the host by viruses and bacteria

You already know that viruses cause damage by taking over a host cell’s genetic and metabolic machinery. Many viruses also cause the eventual death of the cells they invade.

Most of the damage done to host cells by bacteria is inflicted by toxins. Toxins are poisonous substances that are sometimes produced by microorganisms. These poisons are transported by the blood and can cause serious and sometimes fatal effects. Toxins can inhibit protein synthesis in the host cell, destroy blood cells and blood vessels, produce fever, or cause spasms by disrupting the nervous system.

For example, the toxin produced by tetanus bacteria affects nerve cells and produces uncontrollable muscle contractions. If the condition is left untreated, paralysis and death occur. Tetanus bacteria are normally present in soil, and as Figure 39.4 illustrates, a soldier could become infected with tetanus as a result of wounds received on the battlefield. If dirt transfers the bacteria into a deep wound on the body, the bacteria begin to produce the toxin in the wounded area. A small amount of this toxin, about the same amount as the ink used to make a period on this page, could kill 30 people.

Patterns of Diseases

In today’s highly mobile world, diseases such as influenza can spread rapidly as people fly from one part of the country or world to another. Therefore, identifying a pathogen, its method of transmission, and the geographic distribution of the disease it causes are major concerns of government health departments. The Centers for Disease Control and Prevention, the central source of disease information in the United States, publishes a weekly report about the incidence of specific diseases.

Some diseases, such as typhoid fever, occur only occasionally in the United States. These periodic outbreaks often occur because someone traveling in a foreign country has brought the disease back home. On the other hand, many diseases are constantly present in the population. Such a disease is called an endemic disease. The common cold is an endemic disease.

Sometimes, an epidemic breaks out. An epidemic occurs when many people in a given area are afflicted with the same disease at about the same time. Influenza is a disease that often achieves epidemic status, sometimes spreading to many parts of the world. During the early 1950s, a polio epidemic spread across the United States. Victims of this disease were paralyzed or died when the polio virus attacked the nerve cells of the brain and spinal cord. Many survived only after being placed in an iron lung—a machine that allowed the patient to continue to breathe, as shown in Figure 39.5.

Treating Diseases

A person who becomes sick often can be treated with medicinal drugs, such as antibiotics. An antibiotic is a substance produced by a microorganism that, in small amounts, will kill or inhibit the growth and reproduction of other microorganisms, especially bacteria. Antibiotics are produced naturally by various species of bacteria and fungi. Although antibiotics can be used to cure some bacterial infections, antibiotics do not have an affect on viruses.

With the continued use of antibiotics, bacteria can become resistant to the drugs. That means the drugs become ineffective. Penicillin, an antibiotic produced by a fungus, was used for the first time in the 1940s and is still one of the most effective antibiotics known. However, penicillin has now been in use for more than 50 years, and more and more types of bacteria have evolved that are resistant to it. Bacteria that are resistant to penicillin produce an enzyme that breaks down this antibiotic. Examples of penicillin-resistant bacteria include Streptococcus pneumoniae, shown in Figure 39.6, and the organism that causes the STD gonorrhea. The resistance of Streptococcus pneumoniae to penicillin is a problem because penicillin is the primary drug used to treat pneumonia, ear infections, and meningitis, all of which can be caused by this organism.

The use of antibiotics is only one way to fight infections. Your body also has its own built-in defense system—the immune system—that works to keep you healthy.

Defense against Infectious Diseases

Innate Immunity

Your body produces a variety of white blood cells that defend it against invasion by pathogens that are constantly bombarding you. No matter what pathogens are present, your immune system is always ready. The body’s earliest lines of defense against any and all pathogens make up your nonspecific, innate immunity.

Skin and body secretions

When a potential pathogen contacts your body, often the first barrier it must penetrate is your skin. Like the walls of a castle, intact skin is a formidable physical barrier to the entrance of microorganisms.

In addition to the skin, pathogens also encounter your body’s secretions of mucus, oil, sweat, tears, and saliva. The main function of mucus is to prevent various areas of the body from drying out. Because mucus is slightly viscous (thick), it also traps many microorganisms and other foreign substances that enter the respiratory and digestive tracts. Mucus is continually swallowed and passed to the stomach, where acidic gastric juice destroys most bacteria and their toxins. Sweat, tears, and saliva all contain the enzyme lysozyme, which is capable of breaking down the cell walls of some bacteria.

Inflammation of body tissues

If a pathogen manages to get past the skin and body secretions, your body has several other nonspecific defense mechanisms that can destroy the invader and restore homeostasis. Think about what happens when you get a splinter. If bacteria or other pathogens enter and damage body tissues, inflammation results. Inflammation is characterized by four symptoms—redness, swelling, pain, and heat. As Figure 39.7 shows, inflammation begins when damaged tissue cells called mast cells, and white blood cells called basophils release histamine. Histamine causes blood vessels in the injured area to dilate, which makes them more permeable to tissue fluid. These dilated blood vessels cause the redness of an inflamed area. Fluid that leaks from the vessels into the injured tissue helps the body destroy toxic agents and restore homeostasis. This increase in tissue fluid causes swelling and pain, and may also cause a local temperature increase. Inflammation can occur as a reaction to other types of injury as well as infections. Physical force, chemical substances, extreme temperatures, and radiation may also inflame body tissues.

Phagocytosis of pathogens

Pathogens that enter your body may encounter cells that carry on phagocytosis. Recall that phagocytosis occurs when a cell engulfs a particle. Phagocytes are white blood cells that destroy pathogens by surrounding and engulfing them. Phagocytes include monocytes, which develop into macrophages, neutrophils, and eosinophils. Macrophages are present in body tissues. The other types of phagocytes circulate in the blood.

Macrophages are white blood cells that provide the first defense against pathogens that have managed to enter the tissues. Macrophages, shown in Figure 39.8, are found in the tissues of the body. They are sometimes called giant scavengers, or big eaters, because of the manner in which they engulf pathogens or damaged cells. Lysosomal enzymes inside the macrophage digest the particles it has engulfed.

If the infection is not stopped by the tissue macrophages, another type of phagocyte, called a neutrophil, is attracted to the site. They also destroy pathogens by engulfing and digesting them.

If the infection is not stopped by tissue macrophages and neutrophils, there is a third method of defense. A different type of phagocyte begins to arrive on the scene. Monocytes are small, immature macrophages that circulate in the bloodstream. These cells squeeze through blood vessel walls to move into the infected area. Once they reach the site of the infection they mature and are now called macrophages. They then begin consuming pathogens and dead neutrophils by phagocytosis. Once the infection is over, some monocytes mature into tissue macrophages that remain in the area, pre- pared to fend off a new infection.

After a macrophage has destroyed large numbers of pathogens, dead neutrophils, and damaged tissue cells, it eventually dies. After a few days, infected tissue harbors a collection of live and dead white blood cells, multiplying and dead pathogens, and body fluids called pus. Pus formation usually continues until the infection subsides. Eventually, the pus is cleared away by macrophages.

Which white blood cells are involved in the body’s defense against pathogens?

Protective proteins

When an infection is caused by a virus, your body faces a problem. Phagocytes alone cannot destroy viruses. Recall that a virus multiplies within a host cell. A phagocyte that engulfs a virus will itself be destroyed if the virus multiplies within it. One way your body can counteract viral infections is with interferons. Interferons are proteins that protect cells from viruses. Interferons are host-cell specific. This means that human interferons will protect human cells from viruses but cannot protect cells of other species from the same virus.

Interferon is produced by a body cell that has been infected by the virus. The interferon diffuses to uninfected neighboring cells, which then produce antiviral proteins that can prevent the virus from multiplying.

Acquired Immunity

The cells of your innate immune system continually survey your body for foreign invaders. When a pathogen is detected, these cells begin defending your body right away. Meanwhile, as the infection continues, another type of immune response that counteracts the invading pathogen is also mobilized. Certain white blood cells gradually develop the ability to recognize a specific foreign substance. This acquired immune response enables these white blood cells to inactivate or destroy the pathogen. Defending against a specific pathogen by gradually building up a resistance to it is called acquired immunity.

Normally, the immune system recognizes components of the body as self, and foreign substances, called antigens, as nonself. Antigens are usually proteins present on the surfaces of whole organisms, such as bacteria, or on parts of organisms, such as the pollen grains of plants. An acquired immune response occurs when the immune system recognizes an antigen and responds to it by producing antibodies against it. Antigens are foreign substances that stimulate an immune response, and antibodies are proteins in the blood that correspond specifically to each antigen.

The development of acquired immunity is the job of the lymphatic system. The process of acquiring immunity to a specific disease can take days or weeks.

The lymphatic system

Your lymphatic system not only helps the body defend itself against disease, but also maintains homeostasis by keeping body fluids at a constant level.

Your body’s cells are constantly bathed with fluid. This tissue fluid is composed of water and dissolved substances that diffuse from the blood into the spaces between the cells that make up the surrounding tissues. This tissue fluid collects in open-ended lymph capillaries. Once the tissue fluid enters the lymph vessels, it is called lymph.

Lymph capillaries meet to form larger vessels called lymph veins. The flow of lymph is only toward the heart, so there are no lymph arteries. The lymph veins converge to form two major lymph ducts. These ducts return the lymph to the bloodstream in the shoulder area, after it has been filtered through various lymph glands.

Glands of the lymphatic system

At locations along the lymphatic system, the lymph vessels pass through lymph nodes. A lymph node is a small mass of tissue that contains lymphocytes and filters pathogens from the lymph, as shown in Figure 39.11. Lymph nodes are made of an interlaced network of connective tissue fibers that holds lymphocytes. A lymphocyte is a type of white blood cell that defends the body against foreign substances.

The tonsils are large clusters of lymph tissue located at the back of the mouth cavity and at the back of the throat. They form a protective ring around the openings of the nasal and oral cavities. Tonsils provide protection against bacteria and other pathogens that enter your nose and mouth.

The spleen is an organ that stores certain types of lymphocytes. It also filters out and destroys bacteria and worn-out red blood cells, and acts as a blood reservoir. Unlike lymph nodes, the spleen does not filter lymph.

Another important component of the lymphatic system is the thymus gland, which is located above the heart. The thymus gland stores immature lymphocytes until they mature and are released into the body’s defense system.

Antibody Immunity

Acquired immunity involves the production of two kinds of immune responses: antibody immunity and cellular immunity. Antibody immunity is a type of chemical warfare within your body that involves several types of cells. Follow the steps of antibody immunity illustrated in Figure 39.12. When a pathogen invades your body, it is first attacked by the cells of your innate immune system, as shown in Figure 39.12A, B, and C. If the infection is not controlled, then your body builds up acquired immunity to the antigen by producing anti-bodies to it. A type of lymphocyte called a T cell becomes involved. A T cell is a lymphocyte that is produced in bone marrow and processed in the thymus gland. Two kinds of T cells play different roles in immunity. One kind of T cell, called a helper T cell, interacts with B cells, shown in Figure 39.12D, E, and F. A B cell is a lymphocyte that, when activated by a T cell, becomes a plasma cell and produces antibodies. B cells are produced in the bone marrow. Plasma cells, shown in Figure 39.12G and H, release antibodies into the blood-stream and tissue spaces. Some activated B cells do not become plasma cells but remain in the bloodstream as memory B cells. Memory B cells are ready and armed to respond rapidly if the same pathogen invades the body at a later time. The response to a second invasion is immediate and rapid, usually without any symptoms.

Cellular Immunity

Like antibody immunity, cellular immunity also involves T cells with antigens on their surfaces. The T cells involved in cellular immunity are cytotoxic, or killer, T cells. T cells stored in the lymph nodes, spleen, and tonsils transform into cytotoxic T cells that are specific for a single antigen. However, unlike B cells, they do not form antibodies. Cytotoxic T cells differentiate and produce identical clones. They travel to the infection site and release enzymes directly into the pathogens, causing them to lyse and die. The steps in cellular immunity are illustrated in Figure 39.13.

The same cells that protect the body against pathogens can sometimes cause problems within the body. Sometimes the immune system overreacts to a harmless substance such as pollen. Mast cells release histamines in large amounts, causing the symptoms of an allergic reaction: sneezing, increased mucus production in the nasal passages, and redness. The immune system also can recognize its own cells as foreign and mistakenly attack the body’s own tissues in what is referred to as an autoimmune disorder, such as lupus or rheumatoid arthritis. T cells and antibodies also can attack transplanted tissue, such as a transplanted kidney, that comes from a source outside the body.

Passive and Active Immunity

Acquired immunity to a disease may be either passive or active. Passive acquired immunity develops as a result of acquiring antibodies that are generated in another host. For example, prior to birth and during nursing, a human infant acquires passive immunity to disease from its mother.

Active acquired immunity develops when your body is directly exposed to antigens and produces antibodies in response to those antigens.

Passive immunity

Passive immunity may develop in two ways. Natural passive immunity develops when antibodies are transferred from a mother to her unborn baby through the placenta or to a newborn infant through the mother’s milk. Artificial passive immunity involves injecting into the body anti-bodies that come from an animal or a human who is already immune to the disease. For example, a person who is bitten by a snake might be injected with antibodies from a horse that is immune to the snake venom.

Active immunity

Active immunity is obtained naturally when a person is exposed to antigens. The body produces antibodies that correspond specifically to these antigens. Once the person recovers from the infection, he or she will usually be immune if exposed to the pathogen again.

Active immunity can be induced artificially by vaccines. A vaccine is a substance consisting of weakened, dead, or incomplete portions of pathogens or antigens that, when injected into the body, cause an immune response. Vaccines produce immunity because they prompt the body to react as if it were naturally infected. Table 39.2 lists some common vaccines.

In the late 1790s, Edward Jenner, an English country doctor shown in Figure 39.14, demonstrated the first safe vaccination procedure. Jenner knew that dairy workers who acquired cowpox from infected cows were resistant to catching smallpox during epidemics. Cowpox is a disease similar to, but milder than, smallpox. To test whether immunity to cowpox also caused immunity to smallpox, Jenner infected a young boy with cowpox. The boy developed a mild cowpox infection. Six weeks later, Jenner scratched the skin of the boy with viruses from a smallpox victim.

**Textbook 5: Dynamics of Life Vol.2**

Plant Evolution and Adaptations

Plant Evolution

Plants are vital to our survival. The oxygen we breathe, the food we eat, and many of the things that make our lives comfortable, such as clothing, furniture, and our homes, come from or are parts of plants. If you were asked to describe a plant, would you describe a tree, a garden f lower, or a houseplant? Biologists describe plants as multicellular eukaryotes with tissues and organs that have specialized structures and functions. For example, most plants have photosynthetic tissues, and organs that anchor them in soil or to an object or another plant. However, does this description apply to ancient plants?

You read in Chapter 14 that Earth is about 4.6 billion years old. Can you imagine ancient Earth without land plants? That was the case until about 400 million years ago when primitive land plants appeared. However, fossil evidence from about 500 million years ago indicates that the shallow waters of ancient Earth were filled with a variety of organisms—bacteria, algae and other protists, and animals, such as sponges, corals, and worms.

There is strong evidence, including biochemical and fossil evidence, that multicellular land plants and present-day green algae share a common ancestor, as diagrammed in the evolutionary tree in Figure 21.1. This common ancestor might have been able to survive periods of drought. Through natural selection, drought-resistant adaptations in that ancestor, such as protected embryos and other survival characteristics, might have passed to future generations.

When scientists compare present-day plants and present-day green algae, they find the following common characteristics:

•	cell walls composed of cellulose

•	cell division that includes the formation of a cell plate

•	the same type of chlorophyll used in photosynthesis

•	similar genes for ribosomal RNA

•	food stored as starch

•	the same types of enzymes in cellular vesicles

Plant Adaptations to Land Environments

While living on land might seem advantageous for many organisms, there are challenges for land organisms that aquatic organisms do not face. Over time, plants that inhabited land developed adaptations that helped them survive limited water resources as well as other environmental factors.

Cuticle

Have you ever noticed that some plant leaves appear shinier than others, or that some leaves have a grayish appearance, such as those of the agave in Figure 21.2? An adaptation found on most above-ground plant parts is a fatty coating called the cuticle on the outer surface of their cells. Wax also can be a component of the cuticle, giving it a grayish appearance. Fats and waxes are lipids and are insoluble in water, as you learned in Chapter 6. Because of this, the cuticle helps prevent the evaporation of water from plant tissues and also can act as a barrier to invading microorganisms.

Stomata Like algae, most plants carry on photosynthesis that produces glucose and oxygen from carbon dioxide and water. The exchange of gases between plant tissues and the environment is necessary for photosynthesis to occur. If the cuticle reduces water loss, it also might prevent the exchange of gases between a plant and its environment. Stomata are adaptations that enable the exchange of gases even with the presence of a cuticle on a plant. Stomata are openings in the outer cell layer of leaves and some stems, as shown in Figure 21.3.

Although photosynthesis can occur in some green stems, plant leaves usually are the sites of photosynthesis and are where most stomata are found. You will read more about the structures and functions of stomata and leaves in Chapter 22.

Vascular tissues

Another plant adaptation to land environments is vascular tissue—specialized transport tissues. Recall from Chapter 7 that many substances slowly move into and out of cells and from cell to cell by osmosis or diffusion. However, vascular tissue enables faster movement of substances than by osmosis and diffusion, and over greater distances. Plants with vascular tissues are called vascular plants, like those in Figure 21.4. In some plants, substances slowly move from cell to cell by osmosis and diffusion. They are the nonvascular plants and lack specialized transport tissues.

Vascular tissues also provide structure and support. The presence of thickened cell walls in some vascular tissue provides additional support. Therefore, vascular plants can grow larger than nonvascular plants. You will read more about vascular tissues in Chapter 22.

Reproductive strategies

You learned in Chapter 19 that a spore is a haploid cell capable of producing an organism. Some land plants reproduce by spores that have waterproof protective coverings. However, the gametophytes of those land plants must have a film of water covering them for sperm to swim to eggs. Water is a **limiting factor** in the environments of these plants. In Chapter 23 you will read about adaptations of seed plants that enable a sperm to reach an egg without the presence of water.

Seeds

The evolution of the seed was another important adaptation that helped ensure the success of some vascular plants. A seed, as shown in Figure 21.5, is a plant structure that contains an embryo, contains nutrients for the embryo, and is covered with a protective coat. These features enable seeds to survive harsh environmental conditions and then sprout when favorable conditions exist. Seeds also can have different structural adaptations that help scatter them. You will read more about these structural adaptations of seeds in Section 4 of this chapter.

Alternation of Generations

You read in Chapter 19 that the life cycles of some organisms include an alternation of generations—a haploid gametophyte generation and a diploid sporophyte generation. The gametophyte generation produces gametes—sperm and eggs. Some plants produce sperm and eggs on separate gametophytes while others produce them on one gametophyte. When a sperm fertilizes an egg, a diploid zygote forms that can undergo countless mitotic cell divisions to form a multicellular sporophyte. The sporophyte generation produces spores that can grow to form the next gametophyte generation.

Depending on the type of plant, one generation is dominant over the other. This means it is larger and, therefore, more noticeable and lasts longer. Most of the plants you see—houseplants, grasses, garden plants, and trees—are the diploid sporophyte generation for those plants. During plant evolution, the trend was from dominant gametophytes to dominant sporophytes that contain vascular tissue. In land plants, the gametophyte generation of vascular plants is microscopic, as shown in Figure 21.6, but is larger in nonvascular plants and can be observed without using a magnifying device. You will see more examples of gametophytes and sporophytes later in this chapter.

Nonvascular Plants

Diversity of Nonvascular Plants

As shown in the evolutionary tree in Figure 21.8, nonvascular plants make up one of the four major groups of plants that evolved along with green algae from a common ancestor. In general, nonvascular plants usually are small, which enables materials to move within them easily. These plants often are found growing in damp, shady areas—an environment that provides the water needed by nonvascular plants for nutrient transport and reproduction.

Division Bryophyta

The most familiar bryophytes are the mosses. You might have seen these small, nonvascular plants growing on a damp log or along a stream. Although they do not have true leaves, mosses have structures that are similar to leaves. Their photosynthetic, leaf like structures usually consist of a layer of cells that is only one cell thick.

Mosses produce rootlike, multicellular rhizoids that anchor them to soil or another surface, as shown in Figure 21.8. Water and dissolved minerals can diffuse into a moss’s rhizoids. Although mosses have some tissue that transports water and food, these plants do not have true vascular tissues. Water and other substances move throughout a moss by osmosis and diffusion, processes explained in Chapter 7.

Mosses exhibit variety in structure and growth. Some mosses have stems that grow upright and others have trailing vinelike stems. Other mosses form extensive mats that help slow erosion on rocky slopes. Over time, Sphagnum (a type of moss) and other plant matter accumulated, decayed, and formed deep deposits called peat. Peat can be cut into blocks and burned as a fuel. Gardeners and florists often add peat moss to soil to help it retain moisture.

Scientists estimate that as much as one percent of Earth’s surface might be covered by bryophytes. Many mosses, like those in Figure 21.8, grow in temperate regions and freeze and thaw without damage. Other mosses can survive an extreme loss of water and then resume growth when moisture returns.

Division Anthocerophyta

The smallest division of nonvascular plants is division Anthocerophyta. Anthocerophytes are called hornworts because of their hornlike sporophytes, as shown in Figure 21.9. Water, nutrients, and other substances move in hornworts by osmosis and diffusion. Only about 100 species of hornworts have been identified.

An identifying feature of these plants is the presence of one large chloroplast in each cell of the gametophyte and sporophyte. This feature can be observed under a microscope. However, the hornwort sporophyte produces much of the food used by its sporophyte and gametophyte generations.

While examining hornwort tissue under a microscope, besides the large chloroplast in each cell, you also might observe that the spaces around cells are filled with mucilage, or slime, rather than air. Cyanobacteria in the genus Nostoc often grow in this slime. The cyanobacteria and hornwort exhibit mutualism.

Division Hepaticophyta

Because of their appearance and use as a medicine to treat liver ailments during medieval times, hepaticophytes are referred to as liverworts. This division of nonvascular plants contains more than 6000 species. They are found in a variety of habitats ranging from the tropics to the arctic. Liverworts tend to grow close to the ground and in areas where moisture is plentiful, such as damp soil, near water, or on damp decaying logs. A few species even can survive in relatively dry areas. Like other nonvascular plants, water, nutrients, and other substances are transported throughout liverworts by osmosis and diffusion.

Liverworts are classified as either thallose or leafy, as shown in Figure 21.10. A thallose liverwort has a body that resembles a fleshy, lobed structure. Leafy liverworts have stems with flat, thin leaf-like structures arranged in three rows—a row on each side of the stem and a row of smaller leaves on the undersurface. Liverworts have unicellular rhizoids, unlike mosses that have multicellular rhizoids.

DNA analysis has shown that liverworts lack DNA sequences that most other land plants contain. This suggests that liverworts are the most primitive of land plants.

Seedless Vascular Plants

Diversity of Seedless Vascular Plants

Club mosses, also known as spike mosses, and the fern group make up the seedless vascular plant group. As indicated in Figure 21.11, this plant group is one of the three plant groups with vascular tissues. Seedless vascular plants exhibit a great diversity of form and size. Most club mosses are small plants, usually less than 30 cm in height. However, tropical tree ferns can grow to 25 m.

Regardless of their size, an adaptation seen in some seedless vascular plant sporophytes is the strobilus. A strobilus is a compact cluster of spore-bearing structures. The tiny spores produced in the strobilus often are carried by the wind. If a spore lands in a favorable environment, it can grow to form the gametophyte.

Division Lycophyta

Present-day lycophytes or club mosses are descendants of the oldest group of vascular plants. Fossil evidence suggests that ancient lycophytes were tree-sized plants—some as tall as 30 m. They formed a large part of the vegetation of Paleozoic forests. After this vegetation died, its remains changed over time and eventually became part of the coal that humans mine for fuel.

Unlike true mosses, the sporophyte generation of lycophytes is dominant. They resemble moss gametophytes, and their reproductive structures that produce spores are club-shaped or spike-shaped, as shown in Figure 21.11.

Lycophytes have roots, stems, and small, scaly, leaflike structures.

Another name for some lycophytes is ground pines because they resemble miniature pine trees. Stems are either branched or unbranched and grow either upright or creep along the soil’s surface. Roots grow from the base of a stem. Extending down the middle of each scaly leaflike structure is a vein of vascular tissue.

Reading Check Identify the contribution of ancient lycophytes to present-day economies.

Most of the club mosses belong to two genera—Lycopodium and Selanginella—like the examples shown in Figure 21.11 and Figure 21.12. Many tropical lycophyte species are epiphytes. An epiphyte is a plant that lives anchored to an object or another plant. When, anchored in treetops, they create another habitat in the forest canopy.

Division Pterophyta This plant division includes ferns and horse-tails. The horsetails once were in their own plant division. However, recent biochemical studies reveal that they are closely related to ferns and should be grouped with them.

During the **Carboniferous Period**, about 300–359 million years ago, ferns were the most abundant land plants.

Vast forests of treelike ferns existed, and some of them produced seedlike structures. Ferns grow in many different environments. Although ferns are most common in moist environments, they can survive dry conditions. When water is scarce, the life processes of some ferns slow so much that the fern appears to be lifeless. When water becomes available, the fern resumes growth. Examples of ferns growing in diverse habitats are shown in Figure 21.13.

It is unlikely that you have seen a fern gametophyte like the one in Figure 21.14. This tiny, thin structure is smaller than a pin. It grows from a spore and has male and female reproductive structures. Following fertilization, the sporophyte grows from and is briefly dependent on the gametophyte. One adaptation of some ferns that live in dry areas is that they can produce sporophytes without fertilization. Eventually, the sporophyte produces roots and a thick underground stem called a rhizome. The rhizome is a food-storage organ. The aboveground structures of some ferns die at the end of a growing season. The breakdown of the rhizome’s stored food releases energy when growth resumes.

The familiar parts of a fern are its photosynthetic leafy structures, or fronds, shown in Figure 21.14. The frond is part of the sporophyte generation of ferns. Fronds have branched vascular tissue and vary greatly in size.

Fern spores form in a structure called a sporangium, and clusters of sporangia form a sorus. Sori usually are located on the undersides of fronds, as shown in Figure 21.15.

Figure 21.15 also shows the typical structure of horsetails—ribbed, hollow stems with circles of scalelike leaves. Like lycophytes, horsetails produce spores in strobili at the tips of reproductive stems. When released into a favorable environment, horsetail spores can develop into gametophytes.

Another common name for horsetails is scouring rushes because in colonial days, they often were used to scrub pots and pans. Horsetails contain a scratchy substance called silica. You can feel it when you rub your finger along a horsetail stem.

Present-day horsetail species are much smaller than their ancient ancestors. Most horsetails grow in wet areas, such as marshes, swamps, and stream banks. Some species grow in the drier soil of fields and roadsides only because their roots grow into underlying, water-saturated soil.

Vascular Seed Plants

Diversity of Seed Plants

Vascular seed plants produce seeds. Each seed usually contains a tiny sporophyte surrounded by protective tissue. Seeds have one or more cotyledons—structures that either store food or help absorb food for the tiny sporophyte. Plants whose seeds are part of fruits are called angiosperms. Those plants whose seeds are not part of fruits are called gymnosperms. The word gymnosperm comes from two Greek words that together mean naked seed.

Seed plants have a variety of adaptations for the dispersal or scattering of their seeds throughout their environments, like those shown in Figure 21.16. Dispersal is important because it limits competition between the new plant and its parent and/or other offspring.

The sporophyte is dominant in seed plants and produces spores. These spores divide by meiosis to form male gametophytes (pollen grains) and female gametophytes. Each female gametophyte consists of one or more eggs surrounded by protective tissues. Both gametophytes are dependent on the sporophyte generation for their survival.

Earlier in this chapter you read that water must be present for a sperm to reach an egg in both nonvascular plants and seedless vascular plants. Most seed plants do not require a film of water for this process. This is an important difference between seed plants and other plants. This adaptation enables seed plants to thrive in different environments, including areas where water is scarce.

Division Cycadophyta

Cones contain male or female reproductive structures of cycads and other gymnosperm plants. As shown in the evolutionary tree in Figure 21.17, plants with cones—the gymnosperms—evolved before plants with flowers—the angiosperms. A male cone produces clouds of pollen grains that produce male gametophytes. Female cones contain female gametophytes. Cycad cones can be as long as 1 m and weigh as much as 35 kg. Male and female cones grow on separate cycad plants.

Because cycads have large divided leaves and some grow more than

18 m tall, people often think that cycads are related to palm trees. However, cycads have structural differences and different reproductive strategies than do palms. While cycads might resemble woody trees, they actually have a soft stem or trunk consisting mostly of storage tissue.

The natural habitats for cycads are the tropics or subtropics. There is only one species native to the United States. Its native habitat is southern Florida. Cycads grew in abundance 200 million years ago, but today there are only about 11 genera and 250 species.

Division Gnetophyta Plants in division Gnetophyta can live as long as 1500–2000 years. There are just three genera of gnetophytes and each exhibits unusual structural adaptations to its environment.

If you have taken medicines for colds or allergies, they might have contained ephedrine—a compound found naturally in gnetophytes in the genus Ephedra. It is the only gnetophyte genus that grows in the United States. The genus Gnetum includes about 30 species of tropical trees and climbing vines. The remaining genus, Welwitschia, has only one species—a bizarre-looking plant shown in Figure 21.18—found exclusively in the deserts of southwest Africa. It has a large storage root, and two continuously growing leaves that eventually can exceed 6 m in length. Welwitschia takes in available moisture from fog, dew, or rain through its two leaves.

Division Ginkgophyta Only one living species, Ginkgo biloba, represents division Ginkgophyta. Early in the 19th century, fossil remains of Ginkgo biloba were discovered in the state of Washington. The ginkgo disappeared from North America during the Ice Age.

However, it survived in China where it was grown for its seeds—a food delicacy only eaten at weddings and during holidays.

This distinctive tree has small, fan-shaped leaves. Like cycads, male and female reproductive systems are on separate plants. The male tree produces pollen grains in strobiluslike cones growing from the bases of leaf clusters, as shown in Figure 21.19. The female tree produces cones, also shown in Figure 21.19, which, when fertilized, develop foul-smelling, fleshy seed coats. Because they tolerate smog and pollution, ginkgoes are popular with gardeners and urban landscapers. However, male trees usually are favored because they do not produce foul-smelling fleshy cones.

Division Coniferophyta Conifers range in size from low-growing shrubs that are several centimeters tall to towering trees over 50 m in height. Pines, firs, cypresses, and redwoods are examples of conifers.

Conifers are the most economically important gymnosperms. They are sources of lumber, paper pulp, and the resins used to make turpentine, rosin, and other products.

Reproductive structures of most conifers develop in cones. Most conifers have male and female cones on different branches of the same tree or shrub. The small male cones produce pollen. Larger female cones remain on the plants until the seeds have matured. The characteristics of female cones, such as those shown in Figure 21.20, can be used to identify conifers.

Conifers like all plants exhibit adaptations to their environments. What connection can you make between the facts that most conifers have drooping branches and that many conifers grow in snowy climates? Another adaptation is a waxlike coating called cutin that covers conifer needlelike or scalelike leaves and reduces water loss.

When you hear the word evergreen, do you think of a pine or another conifer? Most plants in northern temperate regions called evergreen are conifers. However, in subtropical and tropical regions, other plants, such as palms, also are evergreen. Botanists define an evergreen plant as one with some green leaves throughout the year. This adaptation enables it to undergo photosynthesis whenever conditions are favorable. A plant that loses its leaves at the end of the growing season or when moisture is scarce is called deciduous. Some conifers are deciduous, such as larches and bald cypresses. Whether deciduous or evergreen, you can identify a conifer species by its leaves, as demonstrated in MiniLab 21.2.

Division Anthophyta Flowering plants, or anthophytes, are the most widely distributed plants because of adaptations that enable them to grow in terrestrial and aquatic environments. Anthophytes, also known as angiosperms, first appeared in the fossil record about 130 million years ago after the appearance of gymnosperms, as shown in Figure 21.21. Today, flowering plants make up more than 75 percent of the plant kingdom.

Traditionally, botanists classified anthophytes as monocots or dicots. The names refer to the number of cotyledons in their seeds: monocot— one cotyledon, dicot—two cotyledons. However, botanists now classify dicots as eudicots or dicots, based on the structure of their pollen. About 75 percent of anthophytes are eudicots and include many trees, shrubs, and garden plants. There are few dicots and examples include maples, oaks, and sycamores. Monocots are the second largest group and include palms, lilies, onions, and grasses. You will read more about monocots and eudicots in Chapters 22 and 23.

Life spans A few weeks or years describe the life spans of anthophytes. An annual plant completes its life span—sprouts from a seed, grows, produces new seeds, and dies—in one growing season or less. This group includes many garden plants and most weeds.

A biennial plant’s life spans two years. During the first year, it produces leaves and a strong root system. Refer to Figure 21.22. Some biennials, like carrots, beets, and turnips, develop fleshy storage roots that are harvested after the first growing season. If the biennial is not harvested, the aboveground tissues die. However, roots and other underground parts remain alive for biennials that are adapted to their environments. In the second year, stems, leaves, f lowers, and seeds grow. The plant’s life ends the second year.

Perennial plants can live for several years and usually produce flowers and seeds yearly. Some perennials respond to harsh conditions by dropping leaves; otherwise their aboveground structures die. They resume growth when favorable growing conditions return. Fruit and shade trees, shrubs, irises, peonies, roses, and many types of berries are perennial plants.

The life spans of all plants are determined genetically and reflect adaptations for surviving harsh conditions. However, all plant life spans are affected by environmental conditions.

Plant Cells and Tissues

Plant Cells

Recall from Chapters 7 and 21 that you can identify a typical plant cell, like the one in Figure 22.1, by the presence of a cell wall and large central vacuole. Also, plant cells can have chloroplasts. However, there are many different types of plant cells—each with one or more adaptations that enable it to carry out a specific function. Three types of plant cells form most plant tissues. Together they provide storage and food production, strength, flexibility, and support.

Parenchyma cells

Most flexible, thin-walled cells found throughout a plant are parenchyma cells. They are the basis for many plant structures and are capable of a wide range of functions, including storage, photosynthesis, gas exchange, and protection. These cells are spherical in shape and their cell walls flatten when they are packed tightly together, as shown in Table 22.1. An important trait of parenchyma cells is that they can undergo cell division when mature. When a plant is damaged, parenchyma cells divide to help repair it.

Depending on their function, parenchyma cells can have special features. Some parenchyma cells have many chloroplasts, also shown in Table 22.1. These cells often are found in leaves and green stems, and can carry on photosynthesis, producing glucose. Some parenchyma cells, such as those found in roots and fruits, have large central vacuoles that can store substances, such as starch, water, or oils.

Collenchyma cells

If you have eaten celery, you might be familiar with collenchyma cells. These cells make up those long strings that you can pull from a celery stalk. Collenchyma cells are plant cells that often are elongated and occur in long strands or cylinders that provide support for the surrounding cells. As shown in Table 22.1, collenchyma cells can have unevenly thickened cell walls. As a collenchyma cell grows, the thinner portions of its cell wall can expand. Because of this growth pattern, collenchyma cells are flexible and can stretch, which enables plants to bend without breaking. Like parenchyma cells, collenchyma cells retain the ability to undergo cell division when mature.

Sclerenchyma cells

Unlike parenchyma and collenchyma cells, sclerenchyma cells are plant cells that lack cytoplasm and other living components when they mature, but their thick, rigid cell walls remain. These cells provide support for a plant, and some are used for transporting materials within the plant. Sclerenchyma cells make up most of the wood we use for shelter, fuel, and paper products.

There are two types of sclerenchyma cells—sclereids and fibers— also shown in Table 22.1. You might have eaten sclereids—they create the gritty texture of pears. Sclereids, also called stone cells, can be distributed randomly throughout a plant, are shorter than fibers, and are somewhat irregularly shaped. The toughness of seed coats and nut shells results from the presence of sclereids. Sclereids also function in transport, which you will learn more about later in this section. A fiber cell is needle-shaped, has a thick cell wall, and has a small interior space. When stacked end-to-end, fibers form a tough, elastic tissue.

Humans have used these fibers for making ropes and linen, canvas, and other textiles for centuries, as shown in Figure 22.2.

Plant Tissues

You learned in Chapter 9 that a tissue is a group of cells that work together to perform a function. Depending on its function, a plant tissue can be composed of one or many types of cells. There are four different tissue types found in plants—meristematic, dermal, vascular, and ground. These are illustrated in Figure 22.3.

Meristematic tissue

Throughout their lives, plants can continue to produce new cells in their meristematic tissues. Meristematic tissues make up meristems—regions of rapidly dividing cells. Cells in meristems have large nuclei and small vacuoles or, in some cases, no vacuoles at all. As these cells mature, they can develop into many different kinds of plant cells. Meristematic tissues are located in different regions of a plant.

Apical meristems Meristematic tissues at the tips of roots and stems, which produce cells that result in an increase in length, are apical meristems, as shown in Figure 22.3. This growth is called primary growth. Since plants usually are stationary, it enables stems and roots to enter different environments or different areas of the same environments.

Intercalary meristems

Another type of meristem, called intercalary meristem, is related to a summer job you might have had—mowing grass. This meristem is found in one or more locations along the stems of many monocots. Intercalary meristem produces new cells that result in an increase in stem or leaf length. If grasses only had apical meristems, they would stop growing after the first mowing. They continue to grow because they have more than one type of meristematic tissue.

Lateral meristems Increases in root and stem diameters result from secondary growth produced by two types of lateral meristems. Only nonflowering seed plants, eudicots or dicots, and a few monocots have secondary growth.

The vascular cambium, also shown in Figure 22.3, is a thin cylinder of meristematic tissue that can run the entire length of roots and stems. It produces new transport cells in some roots and stems.

In some plants, another lateral meristem, the cork cambium, produces cells that develop tough cell walls. These cells form a protective outside layer on stems and roots. Cork tissues make up the outer bark on a woody plant like an oak tree. Recall that cells of cork tissue are what Robert Hooke observed when he looked through his microscope.

Dermal tissue—the epidermis

The layer of cells that makes up the outer covering on a plant is dermal tissue, also called the epidermis. Cells of the epidermis resemble pieces of a jigsaw puzzle with interlocking ridges and dips, as shown in Figure 22.4. Most epidermal cells can secrete a fatty substance that forms the cuticle. You might recall from Chapter 21 that the cuticle helps reduce water loss from plants by slowing evaporation. The cuticle also can help prevent bacteria and other disease-causing organisms from entering a plant.

Stomata Plants can have several adaptations of their epidermis. Recall from Chapter 21 that the epidermis of most leaves and some green stems have stomata—small openings through which carbon dioxide, water, oxygen, and other gases pass. The two cells that form a stoma are guard cells. Changes in the shapes of guard cells result in the opening and closing of stomata, as shown in Figure 22.4.

Trichomes

Some epidermal cells on leaves and stems produce hairlike projections called trichomes, shown in Figure 22.5. Trichomes can give leaves a fuzzy appearance and can help protect the plant from insect and animal predators. Some trichomes even release toxic substances when touched. Trichomes help keep some plants cool by reflecting light.

Root hairs

Some roots have root hairs—fragile extensions of root epidermal cells. Root hairs, as shown in Figure 22.5, increase a root’s surface area and enable the root to take in a greater volume of materials than it can without root hairs.

Vascular tissues Food, water, and other substances are carried throughout your body in your blood vessels. In a plant, the transportation of water, food, and dissolved substances is the main function of two types of vascular tissue—xylem and phloem.

Xylem Water that contains dissolved minerals enters a plant through its roots. Some of the water is used in photosynthesis. The dissolved minerals have many functions in cells. This water with dissolved minerals is transported throughout a plant within a system of xylem that flows continuously from the roots to the leaves. Xylem is the water-carrying vascular tissue composed of specialized cells—vessel elements and tracheids. When mature, each vessel element and tracheid consists of just its cell wall. This lack of cytoplasm at maturity allows water to flow freely through these cells.

Vessel elements are tubular cells that are stacked end-to-end, forming strands of xylem called vessels. Vessel elements are open at each end with barlike strips across the openings. In some plants, mature vessel elements lose their end walls. This enables the free movement of water and dissolved substances from one vessel element to another.

Tracheids are long, cylindrical cells with pitted ends. The cells are found end-to-end and form a tubelike strand. Unlike some mature vessel elements, mature tracheids have end walls. For this reason, tracheids are less efficient than vessel elements at transporting materials. Compare the structure of tracheids to vessel elements in Figure 22.6.

In gymnosperms or nonflowering seed plants, xylem is composed almost entirely of tracheids. However, in f lowering seed plants, xylem consists of tracheids and vessels. Because vessels are more efficient at transporting water and materials, scientists propose that this might explain why f lowering plants inhabit many different environments.

Phloem The main food-carrying tissue is phloem. It transports dissolved sugars and other organic compounds throughout a plant. Recall that xylem only transports materials away from the roots. Phloem, however, transports substances from the leaves and stems to the roots and from the roots to the leaves and stems. Although not used for transport, there are sclereids and fibers associated with the phloem. These sturdy sclerenchyma cells provide support for the plant.

Phloem consists of two types of cells—sieve tube members and companion cells, shown in Figure 22.7. Each sieve tube member contains cytoplasm but lacks a nucleus and ribosomes when it is mature. Next to sieve tube members are companion cells, each with a nucleus. Scientists hypothesize that this nucleus functions for both the companion cell and the mature sieve tube member. In f lowering plants, structures called cell plates are at the end of the sieve tube members. The cell plates have large pores through which dissolved substances can f low.

Some of the glucose produced in leaves and other photosynthetic tissue is metabolized by the plant. However, some is converted to other carbohydrates and transported and stored in regions of the plant called sinks. Examples of sinks are the parenchyma storage cells in the root cortex, which are described in the next section of this chapter. The transport in phloem of dissolved carbohydrates from sources to sinks and other dissolved substances is translocation.

Ground tissue

The category for plant tissues that are not meristematic tissues, dermal tissues, or vascular tissues is ground tissue. Ground tissues consist of parenchyma, collenchyma, and sclerenchyma cells and have diverse functions, including photosynthesis, storage, and support. Most of a plant consists of ground tissue. The ground tissue of leaves and green stems contains cells with numerous chloroplasts that produce glucose for the plant. In some stems, roots, and seeds, cells of ground tissue have large vacuoles that store sugars, starch, oils, or other substances. Ground tissues also provide support when they grow between other types of tissue.

Roots, Stems, and Leaves

Roots

If you ever have eaten a carrot, a radish, or a sweet potato, then you have eaten part of a plant root. The root usually is the first structure to grow out of the seed when it sprouts. For most plants, roots take in water and dissolved minerals that are transported to the rest of the plant. If you have tried to pull a weed, you experienced another function of roots—they anchor a plant in soil or to some other plant or object. Roots also support a plant against the effects of gravity, extreme wind, and moving water.

In some plants, the root system is so vast that it makes up more than half of the plant’s mass. The roots of most plants grow 0.5 to 5 m down into the soil. However, some plants, such as the mesquite that grows in the dry southwestern part of the United States, have roots that grow downward as deep as 50 m toward available water. Other plants, such as some cacti, have many, relatively shallow branching roots that grow out from the stem in all directions as far as 15 m. Both root types are adaptations to limited water resources.

Root structure and growth

The tip of a root is covered by the root cap, as shown in Figure 22.8. It consists of parenchyma cells that help protect root tissues as the root grows. The cells of the root cap produce a slimy substance that, together with the outside layer of cells, form a lubricant that reduces friction as the root grows through the soil, a crack in a sidewalk, or some other material. Cells of the root cap that are rubbed off as the root grows are replaced by new cells produced in the root’s apical meristem. Recall from Section 22.1 that the root’s apical meristem also produces cells that increase the root’s length.

These cells develop into the numerous types of root tissues that perform different functions.

You also learned in Section 22.1 that an epidermal layer covers the root. Some root epidermal cells produce root hairs that absorb water and dissolved minerals. The layer below this epidermal layer is the cortex. It is composed of ground tissues made of parenchyma cells that are involved in transport and storage of plant substances. The cortex is between the epidermis and the vascular tissues of the root. To reach vascular tissues, all water and nutrients that are taken in by the epidermal cells must move through the cortex.

At the inner boundary of the cortex is a layer of cells called the endodermis, as illustrated in Figure 22.9. Encircling each cell of the endodermis as part of the cell wall is a waterproof strip called a Casparian strip. Its location is similar to that of mortar that surrounds bricks in a wall. The Casparian strip creates a barrier that forces water and dissolved minerals to pass through endodermal cells rather than around them. Therefore, the plasma membranes of endodermal cells regulate the material that enters the vascular tissues.

The layer of cells directly next to the endodermis toward the center of the root is called the pericycle. It is the tissue that produces lateral roots. In dicots, most eudicots, and some monocots, a vascular cambium develops from part of the pericycle. Recall that the vascular cambium produces vascular tissues that contribute to an increase in the root’s diameter. The vascular tissues—xylem and phloem—are in the center of a root. Monocots and eudicots or dicots can be distinguished by the pattern of the xylem and phloem in their roots, as shown in Figure 22.10.

Types of roots

The two major types of root systems are taproots and fibrous roots. A taproot system consists of a thick root with few smaller, lateral-branching roots. Some plants, such as radishes, beets, and carrots, as shown in Table 22.2, store food in the parenchyma cells of a taproot. Other taproots, like those of poison ivy plants, grow deep into the soil toward available water.

Fibrous root systems, also shown in Table 22.2, have numerous branching roots that are about the same size and grow from a central point, similar to the way that the spokes of a bicycle wheel are arranged. Plants also can store food in fibrous roots systems. For example, sweet potatoes develop on fibrous roots.

Other root types, also shown in Table 22.2, are adapted to diverse environments. In arid regions, some plants produce huge water-storage roots. Cypress, mangrove, and some other trees that live in water develop modified roots that help supply oxygen to the roots called pneumatophores. Adventitious roots form where roots normally do not grow and can have different functions. For example, some tropical trees have adventitious roots that help support their branches. As these roots develop, they resemble trunks.

Stems

While you might know that asparagus spears are stems, you might be surprised to learn that there are many types of plant stems. Some stems, like asparagus, are soft, flexible, and green due to the presence of chloroplasts, and therefore can perform photosynthesis. These stems are called herbaceous and most annual plants have this type of stem. Palms and bamboos have rigid, fibrous stems. Trees, shrubs, and many perennials have sturdy, woody stems that do not carry on photosynthesis. Some older plants have stems that are covered with bark. This tough, corky tissue can protect the stem from physical damage and insect invasion. Some trees even have survived a forest fire with minimal damage because of the bark that covers their trunks.

Stem structure and function

The main function of a plant’s stem is support of a plant’s leaves and reproductive structures. Vascular tissues in stems transport water and dissolved substances throughout the plant and provide support. These tissues are arranged in bundles, or groups, that are surrounded by parenchyma cells. As is true for roots, the pattern of these tissues can be used to distinguish between monocots and eudicots and dicots, as shown in Figure 22.11.

Growth of a stem Cells produced by the apical meristem result in an increase in the length of the stem. As the plant grows taller, an increase in stem diameter provides additional support. In annual plants, an increase in stem diameter mostly is due to an increase in cell size. The increase in stem diameter in plants, such as perennial eudicots and conifers, is due to the production of cells by the vascular cambium. The production of xylem and phloem throughout the year can produce annual growth rings. The age of a tree can be estimated by counting the annual growth rings at the base of its trunk, like those of the white oak shown in Figure 22.12.

Types of stems

All stems have adaptations that help plants survive. In some plants, these adaptations enable stems to store excess food, and in other plants, they help withstand drought, cold, or heat. While you easily might identify stems of tomatoes and oaks, other plants have stems that do not resemble typical stems.

For example, a white potato is a type of stem called a tuber—a swollen, underground stem with buds from which new potato plants can grow. The stem of an onion, a tulip, or a tiger lily is part of a bulb. A bulb is a shortened, compressed stem surrounded by fleshy leaves.

Irises and some ferns have rhizomes—underground horizontal stems.

Some rhizomes store food. Runners, or stolons, are horizontal stems that grow along the soil’s surface in nature, like those of strawberry plants and some grasses. Crocuses and gladiolas are examples of plants that form corms. A corm is composed almost entirely of stem tissue with some scaly leaves at its top. Examples of some of these stem types are shown in Table 22.3.

Leaves

There are many shapes and colors of leaves, and their arrangements on plants are different for different species. Also, the sizes of leaves can range from as large as 2 m in diameter to less than 1 mm in length. In a growing season, the number of leaves that a plant can produce varies from a few, such as for a daffodil, to over five million produced by a mature American elm tree.

Leaf structure

The main function of leaves is photosynthesis, and their structure is well-adapted for this function. Most leaves have a flattened portion called the blade that has a relatively large surface area.

Depending on the plant species, the blade might be attached to the stem by a stalk called a petiole. The petiole’s vascular tissue connects the stem’s vascular tissues to the leaf’s vascular tissue or veins. Plants such as grasses lack petioles, and their leaf blades are attached directly to the stem.

The internal structure of most leaves is well-adapted for photosynthesis. Figure 22.13 shows tightly packed cells directly below a leaf’s upper epidermis. This location has the maximum exposure to light, and therefore, most photosynthesis takes place in these column-shaped cells. They contain many chloroplasts and make up the tissue called the palisade mesophyll, or palisade layer.

Below the palisade mesophyll is the spongy mesophyll, consisting of irregularly-shaped, loosely packed cells with spaces surrounding them.

Oxygen, carbon dioxide, and water vapor move through the spaces in the spongy mesophyll. Cells of the spongy mesophyll also contain chloroplasts, but have fewer per cell than in the palisade mesophyll.

Gas exchange and transpiration

The epidermis covers a leaf. Except for submerged leaves of aquatic plants, the epidermis contains stomata. There usually are more stomata on the underside of leaves than on the upper side. Recall that two guard cells border a stoma. When more water diffuses into the guard cells than out of them, their shapes change in such a way that the stoma opens. Conversely, when more water diffuses out of the guard cells than into them, their shapes change in such a way that the stoma closes. In Chapter 8, you learned that carbon dioxide is used in photosynthesis, and that oxygen gas is a by-product of photosynthesis. The diffusion of these and other gases into and out of a plant also occurs through stomata.

In most plants, water travels from the roots up through the stems and into the leaves, replacing the water used in photosynthesis and lost from the plant by evaporation. Water evaporates from the inside of a leaf to the outside through stomata in a process called transpiration that helps pull the water column upward.

Characteristics of leaves Can you identify a maple tree by looking at its leaves? Some people can use differences in the size, shape, color, and texture of leaves to help them identify types of plants. Some leaves are simple, which means the leaf blade is not divided into smaller parts. Compound leaves have leaf blades that are divided into two or more smaller parts called leaflets, as shown in Figure 22.14.

The arrangement of leaves on the stem, also shown in Figure 22.14, also can be used to distinguish between types of plants. If two leaves are directly opposite of each other on a stem, the growth arrangement is called opposite. An alternate growth arrangement is when the positions of leaves alternate on opposite sides of the stem. A third arrangement, called whorled, is when three or more leaves are evenly spaced around a stem at the same position.

The arrangement of veins in a leaf, or the venation pattern, also can be used to identify leaves. Monocots usually have parallel venation and eudicots and dicots usually have branched or netlike venation.

Leaf modifications

Although the primary function of leaves is photosynthesis, there are many chemical and structural leaf modifications related to other functions. Many succulents, like the cacti in Figure 22.15, have modified leaves called spines. In addition to reducing water loss, the spines help protect cacti from being eaten by animals. Other succulents have leaves used as water storage sites. The leaves swell with water when water is available, and when water is scarce, these reserves can help ensure the long-term survival of plants.

In plants like poinsettias, leaves called bracts at the tips of stems change from green to another color in response to the number of hours of darkness in their environments. These plants usually have tiny flowers at the center of the colored leaves, shown in Figure 22.16. The leaves look like flower petals and attract pollinators.

The leaves of the sundew plant produce a sticky substance that traps insects. The pitcher plant, also shown in Figure 22.16, has cylinderlike modified leaves that fill with water and can trap and drown insects and small animals. Both of these adaptations enable the plants to get nutrients, especially nitrogen, from the insects they capture.

You might be familiar with poison ivy or poison oak that can cause severe skin irritation for some people. These are examples of leaves that contain toxic chemicals that deter organisms from touching them.

Some leaves have modifications that deter herbivores from eating them. For example, the epidermis of tomato and squash leaves and stems have tiny hairs with glands at their tips. The glands contain substances that repel insects and other herbivores.

When you read about stems, you learned that bulbs were shortened stems with leaves. A bulb’s leaves are modified food storage structures. They provide the dormant bulb with necessary energy resources when favorable growth conditions exist.

Plant Hormones and Responses

Plant Hormones

You read in Chapter 6 that hormones are organic compounds that are made in one part of an organism, and then are transported to another part where they have an effect. It takes only a tiny amount of a hormone to cause a change in an organism. Were you surprised to read that plants produce hormones? Plant hormones can affect cell division, growth, or differentiation. Research results indicate that plant hormones work by chemically binding to the plasma membrane at specific sites called receptor proteins. These receptors can affect the expression of a gene, the activity of enzymes, or the permeability of the plasma membrane. You will learn more about human hormones in Chapter 35.

Auxin

One of the first plant hormones to be identified was auxin.

There are different kinds of auxins, but indoleacetic acid (IAA) is the most widely studied. IAA is produced in apical meristems, buds, young leaves, and other rapidly growing tissues. It moves throughout a plant from one parenchyma cell to the next by a type of active transport. The rate of this movement has been measured at 1 cm per hour. Some auxins also move in the phloem. Also, an auxin moves in only one direction—away from where it was produced.

Auxin usually stimulates the lengthening, or elongation, of cells. Research indicates that in young cells this is an indirect process. Auxin promotes a flow of hydrogen ions through proton pumps from the cytoplasm into the cell wall. This creates a more acidic environment, which weakens the connections between the cellulose fibers in the cell wall. It also activates certain enzymes that help to break down the cell wall. Due to the loss of hydrogen ions in the cytoplasm, water enters the cell, as shown in Figure 22.17. The combination of weakened cell walls and increased internal pressure results in cell elongation.

The effect of auxin in a plant varies greatly depending on its concentration and location. For example, in some plants the concentration of auxin that promotes stem growth can inhibit root growth. Low concentrations of auxin usually stimulate cell elongation. However, at higher concentrations, auxin can have the reverse effect. The presence of other hormones can modify the effects of an auxin.

The presence of auxin also creates a phenomenon called apical dominance, which is when plant growth is mostly upward with few or no side branches. The auxin produced by an apical meristem inhibits the growth of side or lateral branches. Removing a plant’s apical meristem, however, decreases the amount of auxin present. This promotes the growth of side branches. Figure 22.18 shows the difference this makes.

Auxins affect fruit formation and inhibit the dropping of fruit. Research results show that the production of auxin slows as cells mature. At the end of the growing season, the decreased amount of auxin in some trees and shrubs causes ripened fruits to fall to the ground and leaves to fall before winter.

Gibberellins The group of plant hormones called gibberellins causes cell elongation, stimulates cell division, and affects seed growth. Gibberellins are transported in vascular tissue. Dwarf plants often lack either the genes for gibberellins production or the genes for gibberellins protein receptors. When treated with gibberellins, plants that lack the genes for gibberellins but have gibberellins receptors grow taller. Applying gibberellins to a plant can cause an increase in height, as shown in Figure 22.19.

Ethylene The only known gaseous hormone is ethylene. It is a simple compound composed of two carbon and four hydrogen atoms.

Ethylene is found in plant tissues such as ripening fruits, dying leaves, and flowers. Since ethylene is a gas, it can diffuse through the spaces between cells. It also is transported within the phloem.

Although ethylene can affect other parts of plants, it primarily affects the ripening of fruits. Ethylene causes cell walls of unripe fruit to weaken and complex carbohydrates to break down into simple sugars.

The results of ethylene exposure are fruits that are softer and sweeter than unripe fruits.

Because ripe fruits and vegetables are bruised easily during shipping, growers often pick and ship unripe fruits and vegetables. Once they reach their destinations, a treatment with ethylene speeds up the ripening process.

Cytokinins

Growth-inducing cytokinins are produced in rapidly dividing cells. They travel to other parts of the plant within xylem. Cytokinins promote cell division by stimulating the production of the proteins needed for mitosis and cytokinesis.

Because cytokinins increase the rate of growth, they often are added to the growth media used for plant tissue culture—a laboratory technique for growing plants from pieces of plant tissues. The presence of other hormones, especially auxins, influences the effects of cytokinins. For example, IAA alone stimulates cell elongation, but combined with a cytokinin, it promotes rapid cell division and results in rapid growth.

Plant Responses

Have you ever wondered why the leaves of a houseplant grow toward a window, or how a vine can climb a pole? These and other events— roots growing down, stems growing upward, trees dropping their leaves, and leaves of some plants trapping an insect—are due to responses of plants to their environment.

Nastic responses

A response of a plant that causes movement independent of the direction of the stimulus is a nastic response. It is not a growth response, is reversible, and can be repeated many times.

Solar tracking that is exhibited by some plants, such as a sunflower, is a nastic response. As Earth rotates, the flower of a sunflower plant moves in relation to the apparent changing position of the Sun. It can be inferred that this response provides maximum light exposure.

Another example of a nastic response is the closing of a Venus flytrap’s leaves. Recent research shows that this results from a movement of water within each half of the leaf trap. The movement results in uneven expansion until the leaf’s curved shape suddenly changes and snaps the trap shut.

Tropic responses

What do you notice about the plants in Table 22.4? These are examples of tropic responses, or tropisms. A tropism is a plant’s growth response to an external stimulus. If resulting plant growth is toward the stimulus, it is called a positive tropism. If the resulting plant growth is away from the stimulus, it is called a negative tropism. There are several different types of tropisms, including phototropism, gravitropism, and thigmotropism.

Phototropism is a plant growth response to light caused by an unequal distribution of auxin. There is less auxin on the side of the plant toward the light source and more auxin on the side away from the light source. Because auxin can cause cell elongation, the cells on the side away from the light elongate, making that side of the stem longer. This results in the stem curving toward the direction of the light.

Gravitropism is a plant growth response to gravity.

Roots generally show a positive gravitropism. The downward growth of roots into soil helps to anchor the plant and brings roots in contact with water and minerals. However, a stem exhibits a negative gravitropism when it grows upward, away from gravity. This growth positions leaves for maximum exposure to light.

Another tropism found in some plants is thigmotropism. This is a growth response to mechanical stimuli, such as contact with an object, another organism, or even wind. Thigmotropism is evident in vining plants that twist around a nearby structure such as a fence or tree.

Introduction to Plant Reproduction

Vegetative Reproduction

Recall from Chapter 9 that reproduction without the joining of an egg and a sperm is called asexual reproduction. Vegetative reproduction is a form of asexual reproduction in which new plants grow from parts of an existing plant. The new plants are clones of the original plant because their genetic makeups are identical to the original plant.

There are several advantages of vegetative reproduction. It usually is a faster way to grow plants than from a spore or a seed. In Chapter 10 you read that an organism produced sexually will have a combination of features from its parents. However, plants produced vegetatively are more uniform than those that result from sexual reproduction. Also, some fruits do not produce seeds, and vegetative reproduction is the only way to reproduce them.

Naturally occurring vegetative reproduction

There are many examples of natural vegetative reproduction. When conditions are dry, some mosses dry out, become brittle, and easily are broken and scattered by animals or wind. When conditions improve and water is available, some of these fragments can resume growth. Liverworts reproduce asexually by producing small, cuplike structures on the gametophyte thallus, as shown in Figure 23.1. Strawberry plants produce horizontal stems called stolons. A new strawberry plant can grow at the end of a stolon, and if the stolon is cut, the plant can continue to grow.

Humans use vegetative reproduction Farmers, horticulturists, and scientists have been using vegetative reproduction for years. Leaves, roots, or stems, when cut from certain plants, can grow and become new plants if kept under proper environmental conditions. For example, white potatoes can be cut into sections. As long as each section contains an eye or bud and is planted in a favorable environment, a new plant can grow from the section and produce new potatoes.

Some plants can be grown from a few cells of plant tissue using a technique called tissue culture. The plant tissue is grown on nutrient agar in sterile conditions, as shown in Figure 23.2. Eventually, hundreds of identical plants can be produced.

Alternation of Generations

As you read in Chapter 21, the life cycle of a plant includes an alternation of generations that has a **diploid (2n) sporophyte stage**, and **a haploid (n) gametophyte stage**. As shown in Figure 23.3, the **sporophyte stage** produces haploid spores that divide by mitosis and cell division and form the gametophyte generation. Depending on the plant species, the size of a gametophyte can be tiny or a larger structure. In the plant kingdom, there is an evolutionary trend for smaller gametophytes as plants become more complex.

The **gametophyte stage** produces gametes—eggs and sperm. One distinguishing characteristic among plants is how a sperm gets to an egg. Sperm of nonvascular plants and some of the vascular plants must have at least a film of water to reach an egg. Sperm of flowering plants do not need water for sperm to reach the egg.

Fertilization of an egg by a sperm forms a zygote that is the first cell of the sporophyte stage. As plants evolved and became more complex, sporophytes became larger. In addition to the size of the sporophyte, another distinguishing feature among plants is the growth pattern of the sporophyte. Flowering plants and other vascular plants have sporophytes that live completely independent of the gametophyte. Most nonvascular plants have sporophytes that depend on the gametophyte for support and food.

Moss Reproduction and Life Cycle

The reproduction and life cycle of mosses, as shown in Figure 23.4, exhibits alternation of generations and is typical of most nonvascular plants. The dominant stage is the gametophyte stage that you might see growing in damp shady places or on rocks along a stream. Recall from Chapter 21 that gametophytes can produce archegonia and antheridia. These structures can be on the same moss plant or, as is often the case, on separate plants. Depending on the moss species, an archegonium produces one or more eggs. The tissues of the archegonium surround the egg or eggs with a protective layer.

Antheridia produce flagellated sperm that need water to get to the archegonia. If a film of water covers the moss, sperm can move toward archegonia. This is a response to chemicals produced by archegonia and is called chemotaxis. When a sperm fertilizes an egg, it forms the first cell of the sporophyte stage called the zygote. Tissues of the archegonium protect the new sporophyte. The new sporophyte absorbs nutrients from the archegonium as it grows and matures. Because most mature moss sporophytes cannot undergo photosynthesis, they are dependent upon their gametophytes for nutrition and support.

A mature sporophyte consists of a stalk with a capsule at its tip.

Certain cells within the capsule undergo meiosis and produce spores. Some species produce up to 50 million spores per capsule. When conditions are favorable, the capsule opens, releasing the spores. If a spore lands in a suitable place, mitotic cell divisions begin. The resulting growth forms a protonema—a small, threadlike structure—that can develop into the gametophyte plant, and the cycle repeats.

Fern Reproduction and Life Cycle

When you visit a forest or a plant conservatory, you might see the lacy fronds of ferns. Fronds are part of a fern’s sporophyte stage. If you look closely at a frond, you might find spore-producing structures called sori on it. Each sorus consists of sporangia. Certain cells in a sporangium undergo meiosis and the resulting spores are the beginning of the new gametophyte generation.

If a fern spore lands on damp, rich soil, it can grow and form a tiny heart-shaped gametophyte called a prothallus, as shown in Figure 23.5. Cells of the prothallus contain chloroplasts; therefore, photosynthesis can occur. Most prothalli develop both antheridia and archegonia. Antheridia produce flagellated sperm that need water to move to archegonia. Each archegonium contains one egg. If fertilization occurs, the resulting zygote is the first cell of the sporophyte generation. Chemical reactions between sperm and eggs of the same prothallus can prevent fertilization.

The zygote undergoes mitotic cell divisions and forms a photosynthetic, multicellular sporophyte. Initially, the sporophyte grows on the prothallus and receives support and nutrition. Later, the prothallus disintegrates and the sporophyte develops fronds and a rhizome—a thick underground stem that produces roots and supports the photosynthetic fronds.

Conifer Reproduction and Life Cycle

Have you ever seen the surface of a car or a pond covered with fine yellow dust? It’s possible that this dust came from one or several plants called conifers. The tree or shrub that you might recognize as a pine or other conifer is that plant’s sporophyte generation. Conifers, like a few lycophytes and pterophytes, are heterosporous—they produce two types of spores that develop into male or female gametophytes.

Female cones

Each female cone is composed of many scales. At the base of each scale are two ovules. Within each ovule, meiosis of a cell in the megasporangium produces four megaspores. Three of these megaspores disintegrate. The remaining megaspore undergoes mitotic cell divisions and becomes the female gametophyte. When fully developed, the female gametophyte consists of hundreds of cells and contains two to six archegonia. Each archegonium eventually contains an egg.

Male cones

The pollen-producing cone, commonly called the male cone, consists of small reproductive scales that have hundreds of sporangia. Certain cells in these sporangia undergo meiosis and form microspores.

A pollen grain—the male gametophyte— consists of four cells and develops from a microspore. Pollen grains are transported on air currents.

Reading Check Compare the sizes of a moss gametophyte and a pine gametophyte.

Pollination

When a pollen grain from one species of seed plants lands on the female reproductive structure of a plant of the same species, pollination occurs. If a conifer pollen grain lands near the micropyle, or the opening of the ovule, it can be trapped in a sticky substance called a pollen drop. As the pollen drop slowly evaporates or is absorbed into the ovule, the pollen grain is pulled closer to the micropyle. Over the next year, the pollen grain will continue to develop.

Seed development

Following pollination, the pollen grain generates a pollen tube. It grows through the micropyle, and into the ovule. This process can take a year or longer. One of the four cells in the pollen grain undergoes mitosis, forming two nonflagellated sperm. The sperm travel in the pollen tube to an egg, as shown in Figure 23.6.

Fertilization occurs when an egg and a sperm join to form the zygote. The remaining sperm and the pollen tube disintegrate. The zygote is dependent on the female gametophyte for nutrition as it undergoes mitotic cell divisions that result in the formation of an embryo with one or more cotyledons. These undergo photosynthesis and provide nutrition for the embryo when the seed sprouts.

As the embryo develops, the outside layer of the ovule forms a seed coat. Seed development can take as long as three years. When seeds mature, the female cone opens and releases them.

Flowers

Flower Organs

Vivid orange, deep purple, ghostly white, fragrant, rancid, spectacular, and inconspicuous—these all are terms that can be used to describe flowers. The colors, shapes, and sizes of flowers are determined by each species’ genetic makeup. It is important to remember that flowers can vary in structure and form from species to species.

Flowers have several organs. Some organs provide support or protection, while others can be involved directly in the reproductive process. In general, flowers have four organs—sepals, petals, stamens, and one or more pistils, illustrated in Figure 23.7. Sepals protect the flower bud and can look like small leaves or even resemble the flower’s petals.

Petals usually are colorful structures that can both attract pollinators and provide them with a landing platform. Sepals and petals, if present, are attached to a flower stalk, called a peduncle.

Most flowers have several stamens—the male reproductive organs. A stamen is composed of two parts—the filament and the anther. The filament, or stalk, supports the anther. Inside the anther are cells that undergo meiosis and then mitotic cell divisions, forming pollen grains. Two sperm eventually form inside each pollen grain.

The female reproductive structure of a flower is the pistil. In the center of a flower is one or more pistils. A pistil usually has three parts—the stigma, the style, and the ovary. The stigma is the tip of the pistil and is where pollination occurs. The style is the part that connects the stigma to the ovary that contains one or more ovules. A female gametophyte develops in each ovule, and an egg forms inside each female gametophyte.

Flower Adaptations

The flower organs described in the previous paragraphs are typical of most flowers. However, many flowers can have modifications to one or more organs. Scientists categorize flowers using these modifications.

Structural differences Flowers that have sepals, petals, stamens, and one or more pistils are called complete flowers. If a flower is missing one or more of these organs, it is an incomplete flower. For example, wild ginger flowers are called incomplete because they have no petals. Other descriptive terms relating to flower organs are perfect and imperfect.

Flowers that have both stamens and pistils are called perfect flowers. Some plants, such as cucumbers and squash, have imperfect flowers. An imperfect flower has either functional stamens or pistils. The stamen-containing, or male, flowers release pollen grains. Following fertilization, a fruit forms from the pistil-containing, or female, flowers.

The number of each flower organ varies from species to species. However, the number of flower organs distinguishes dicots and eudicots from monocots. When the petal number for a flower is a multiple of four or five, the plant usually is a dicot or eudicot. The number of other organs—the sepals, pistils, and stamens—often is the same multiple of four or five. For example, the members of the mustard family of plants have flowers with four sepals and petals, as shown in Figure 23.8. Monocots generally have flower organs in multiples of three. The daylily, also shown in Figure 23.8, has three sepals and petals and six stamens.

Pollination mechanisms Different anthophyte species have flowers of distinctive sizes, shapes, colors, and petal arrangements. Many of these adaptations relate to pollination.

Animal pollination

As shown in Figure 23.9, many animal-pollinated flowers are brightly colored, have strong scents, or produce a sweet liquid called nectar. When insects and other small animals move from flower to flower searching for nectar, they can carry pollen from one flower to another flower. Other insects collect pollen for food. The bright colors and sweet scents of peonies, roses, and lilacs attract insects such as bees, butterflies, beetles, and wasps. White or pale yellow flowers are more visible at dusk and at night, and attract nocturnal animals, such as moths and bats. The fruity smell of some flowers attracts fruit-eating bats that act as the flowers’ pollinators. On the first page of this chapter, you read about Rafflesia, a flower. It gives off the odor of rotting meat. Flowers with this trait attract fly pollinators.

Bird-pollinated flowers often give off little or no aroma. A bird generally has a poor sense of smell, so it usually locates flowers by sight.

Wind pollination Flowers that generally lack showy or fragrant floral parts, also shown in Figure 23.9, usually are wind-pollinated. They produce huge amounts of lightweight pollen. This helps to ensure that some pollen grains will land on the stigma of a flower of the same species. Also, the stamens of wind-pollinated flowers often hang below the petals, exposing them to the wind. The stigma of a wind-pollinated flower often is large, which helps to ensure that a pollen grain might land on it. Wind-pollinated plants include most trees and grasses.

Self pollination

Recall from Chapter 10 that Mendel chose to experiment with pea plants during his genetic experiments. He knew that pea flowers tend to self-pollinate, but also can be cross-pollinated. Self-pollinating flowers can pollinate themselves or another flower on the same plant. Cross-pollinated flowers receive pollen from another plant. Some flowers must be cross-pollinated. This is one reason that pollinators play important roles in anthophyte reproduction. Pollinators provide a way to transfer pollen for flowers that must be cross-pollinated. Pollinators also ensure that reproduction can occur for imperfect flowers, like squash blossoms, as shown in Figure 23.10.

Photoperiodism After noticing that certain plants only flowered at certain times of the year, plant biologists conducted experiments to explain this observation. The research initially focused on the number of hours of daylight to which the plants were exposed. However, researchers discovered that the critical factor that influenced flowering was the number of hours of uninterrupted darkness, not the number of hours of daylight. This flowering response is known as photoperiodism. Scientists also learned that the beginning of flower development for each plant species was a response to a range in the number of hours of darkness. This range of hours is called the plant’s critical period.

Botanists classify flowering plants into one of four different groups— short-day plants, long-day plants, intermediate-day plants, or day-neutral plants. This classification is based on the critical period. The names reflect the researchers’ original focus—the number of hours of daylight. It is important to remember that a more accurate term for a short-day plant, for example, would be a long-night plant. As you read the descriptions of these plants, refer to Figure 23.11.

Short-day photoperiodism

A short-day plant flowers when exposed daily to a number of hours of darkness that is greater than its critical period. For example, a short-day plant could flower when exposed to 16 hours of darkness. Short-day plants flower during the winter, spring, or fall, when the number of hours of darkness is greater than the number of hours of light. Some short-day plants you might recognize are pansies, poinsettias, tulips, and chrysanthemums.

Long-day photoperiodism

A long-day plant flowers when the number of hours of darkness is less than its critical period. These plants flower during the summer. Examples of long-day plants are lettuce, asters, coneflowers, spinach, and potatoes.

Intermediate-day photoperiodism

Many plants that are native to tropical regions are intermediate-day plants. This means that they will flower as long as the number of hours of darkness is neither too great nor too few. Sugarcane and some grasses are examples of intermediate-day plants.

Day-neutral photoperiodism

Some plants will flower regardless of the number of hours of darkness as long as they receive enough light for photosynthesis that supports growth. A plant that flowers over a range in the number of hours of darkness is a day-neutral plant. Buckwheat, corn, cotton, tomatoes, and roses are examples of day-neutral plants.

Flowering Plants

In anthophytes, seeds and fruits can develop from flowers after fertilization.

Real-World Reading Link In 1893, the U.S. Supreme Court ruled that a tomato is legally a vegetable and not a fruit. The justices argued that a tomato is not a fruit because it is not sweet. As you read this section, decide whether this ruling is scientifically accurate.

Life Cycle

Anthophytes are the most diverse and widespread group of plants.

They are unique because they have flowers. Anthophytes have distinctive life cycles and, like all plants, exhibit an alternation of generations.

Like conifers, the sporophyte generation of anthophytes is dominant and supports the gametophyte generation. However, there are many variations of the anthophyte reproductive process.

Gametophyte development

In anthophytes, the development of male and female gametophytes begins in an undeveloped f lower.

Anthophytes are heterosporous—pistils produce megaspores, and stamens produce microspores. A specialized cell in the ovule of a pistil’s ovary undergoes meiosis, producing four megaspores. Usually, three of these megaspores disintegrate and disappear. The nucleus of the functional megaspore undergoes mitosis. Mitotic division continues and the megaspore grows until there is one large cell with eight nuclei. As shown in Figure 23.12, two nuclei migrate toward the center and membranes form around the other six nuclei. The result is three nuclei at each end of the cell and two nuclei in the center called polar nuclei. One of the three nuclei at the end closest to the micropyle becomes the egg. The cell that contains the egg and seven nuclei is the female gametophyte.

The development of the female gametophyte and the male gametophyte might or might not occur at the same time. Within the anther, specialized cells undergo meiosis and produce microspores. As shown in Figure 23.13, the nucleus in each microspore undergoes mitosis that forms two nuclei called the tube nucleus and the generative nucleus. A thick, protective cell wall forms around a microspore. At this point, the microspore is an immature male gametophyte, or pollen grain.

Scientists can identify the family or genus of a pollen grain by the distinctive outer layer of its cell wall called the exine. This characteristic is useful to paleontologists and forensic investigators. Paleontologists can trace the agricultural history of certain regions using pollen fossils. For over 50 years, forensic scientists have used pollen evidence to help determine where and when some crimes were committed.

Pollination and fertilization

Earlier in this chapter, you learned that various flower adaptations help to ensure the successful transfer of pollen from the anther to the stigma of the pistil. Once pollination occurs, the pollen grain can form a pollen tube—an extension of the pollen grain. Usually, the pollen tube grows down through the style to the ovary and the two nuclei travel in the pollen tube toward the ovule.

The pollen grain’s exine can contain compounds that react with compounds of the pistil’s stigma. These reactions can stimulate or inhibit the growth of the pollen tube. For example, in some poppies, a chemical reaction disrupts the formation of the pollen grain’s cytoskeleton. This inhibits the pollen tube’s growth. Different mechanisms prevent incompatible pollen from producing a functional pollen tube.

When a compatible pollen grain lands on a stigma, the pollen grain absorbs substances from the stigma and a pollen tube starts to form, also shown in Figure 23.13. The tube nucleus directs the growth of the pollen tube. However, recent research suggests that the growth of the pollen tube toward the ovule is a chemotaxic response. In some plants, it has been found that calcium affects the direction of the pollen tube’s growth.

The length of a pollen tube depends on the length of the pistil, and can vary from a few centimeters or less to over 50 cm in some corn plants. As the pollen tube grows, the generative nucleus undergoes mitosis, forming two nonflagellated sperm nuclei. The pollen grain is now a mature male gametophyte. When the pollen tube reaches the ovule, it grows through the micropyle and releases the two sperm nuclei. One sperm nucleus fuses with the egg, forming the zygote—the new sporophyte. The other sperm nucleus and the two polar nuclei in the center of the ovule fuse, forming a triploid or 3n cell.

Because two fertilizations occur in an anthophyte egg, this is called double fertilization, shown in Figure 23.14. Double ferilization occurs only in anthophytes. After fertilization, the ovule and the ovary begin to develop into the seed and fruit, respectively.

Results of Reproduction

Fertilization is only the beginning of a long process that finally ends with the formation of a seed. In anthophytes, a seed is part of a fruit that develops from the ovary and sometimes other flower organs.

Seed and fruit development

The sporophyte begins as a zygote—or a 2n cell. Numerous cell divisions produce a cluster of cells that eventually develops into an elongated embryo with one cotyledon in monocots or two cotyledons in dicots and eudicots. The 3n cell formed as a result of double fertilization undergoes cell divisions.

A tissue called the endosperm forms as a result of these divisions and provides nourishment for the embryo. Initially, these cell divisions occur rapidly without cell wall formation. As the endosperm matures, cell walls form. In some monocots, the endosperm is the major component of the seed and makes up most of the seed’s mass. For example, the coconut palm is a monocot. The liquid inside a fresh coconut is liquid endosperm—cells without cell walls.

In eudicots, the cotyledons absorb most of the endosperm tissue as the seed matures. Therefore, the cotyledons of eudicot seeds provide much of the nourishment for the embryo. Examples of eudicot and monocot seeds are shown in Figure 23.15.

As the endosperm matures, the outside layers of the ovule harden and form a protective tissue called the seed coat. You might notice the seed coats of beans or peas when you eat them. The seed coat is the thin, outer covering that often comes off or loosens as seeds are cooked.

Have you ever eaten a tomato or cucumber and noticed the number of seeds inside? Depending on the plant, the ovary can contain one ovule or hundreds. As the ovule develops into a seed, changes occur in the ovary that lead to the formation of a fruit.

Fruits form primarily from the ovary wall. In some cases, the fruit consists of the ovary wall and other flower organs. For example, the seeds of the apple are within the core that develops from the ovary. The juicy tissue that we eat develops from other flower parts.

Besides the apple, other fruits, such as peaches and oranges, are fleshy, while some are dry and hard, such as walnuts and grains.

Seed dispersal In addition to providing some protection for seeds, fruits also help disperse seeds. Dispersal of seeds away from the parent plant increases the survival rate of offspring. For example, when many plants are growing in one area, there is competition for light, water, and soil nutrients. Seeds sprouting next to parent plants and with other offspring compete for these resources.

Fruits that are attractive to animals can be transported great distances away from the parent plant. Animals that gather and bury or store fruits usually do not recover all of them, so the seeds might sprout. Some of the animals, such as deer, bears, and birds, consume fruits. The seeds pass through their digestive tracts undamaged and then are deposited on the ground along with the animals’ wastes. Some seeds have structural modifications that enable them to be transported by water, animals, or wind. You can review seed dispersal in Chapter 21.

Seed germination

When the embryo in a seed starts to grow, the process is called germination. There are a number of factors that affect germination, including the presence of water and/or oxygen, temperature, and those described in Data Analysis Lab 23.1. Most seeds have an optimum temperature for germination. For example, some seeds can germinate when soil is cool, but others need the warmer soils.

Germination begins when a seed absorbs water, either as a liquid or gas. As cells take in water, the seed swells; this can break the seed coat. Water also transports materials to the growing regions of the seed.

Within the seed, digestive enzymes help start the breakdown of stored food. This broken-down food and oxygen are the raw materials for cellular respiration, which results in the release of energy for growth.

The first part of the embryo to appear outside the seed is a structure called the radicle that starts absorbing water and nutrients from its environment. The radicle, as shown in Figure 23.16, will develop into the plant’s root. The hypocotyl is the region of the stem nearest the seed and, in many plants, it is the first part of the seedling to appear above the soil. In some eudicots, as the hypocotyl grows, it pulls the cotyledons and the embryonic leaves out of the soil. Photosynthesis begins as soon as the seedling’s cells that contain chloroplasts are above ground and exposed to light. In monocots, seedling growth is slightly different because the cotyledon usually stays in the ground when the stem emerges from the soil.

Some seeds can survive harsh environmental conditions, such as drought and cold. Other seeds germinate soon after dispersal and others can germinate after long periods. Some maple seeds must germinate within two weeks after dispersal or they will not germinate at all. Most seeds produced at the end of a growing season enter dormancy— a period of little or no growth. Dormancy is an adaptation that increases the survival rate of seeds exposed to harsh conditions. The length of dormancy varies from species to species.

Animal Characteristics

Animals are multicellular, eukaryotic heterotrophs that have evolved to live in many different habitats.

General Animal Features

Recall from Chapter 17 that biologists have created an evolutionary tree to organize the great diversity of living things. The ancestral animals at the beginning of the evolutionary tree are eukaryotic and multicellular—they are made up of many cells. The tiger in Figure 24.1 and all other present-day animals might have evolved from choano-flagellates, which are protists that formed colonies in the sea 570 million years ago. Choanoflagellates, such as the ones shown in Figure 24.1, might have been the earliest true animals. As animals evolved from this multicellular ancestor, they developed adaptations in structure that enabled them to function in numerous habitats. These features mark the branching points of the evolutionary tree and are discussed in the next section. In this section, you will learn about the characteristics that all animals have in common.

Feeding and Digestion

Animals are heterotrophic, so they must feed on other organisms to obtain nutrients. A sea star obtains its food from a clam it has pried open, and a butterfly feeds on nectar from a flower. The structure or form of an animal’s mouth parts determines how its mouth functions. You can investigate how some animals obtain food by performing MiniLab 24.1. After obtaining their food, animals must digest it. Some animals, such as sponges, digest their food inside specific cells. Others, such as earthworms and humans, digest their food in internal body cavities or organs.

Support

Just as animals digest their food in different ways, they support their bodies in different ways. Between 95 and 99 percent of animal species are invertebrates—animals without backbones. The bodies of many invertebrates are covered with exoskeletons, which are hard or tough outer coverings that provide a framework of support. Exoskeletons also protect soft body tissues, prevent water loss, and provide protection from predators. As the animal grows, like the cicada in Figure 24.2, it must shed the old exoskeleton and make a new one.

Some invertebrates, such as sea urchins and sea stars, have internal skeletons called endoskeletons. If an animal has an endoskeleton and a backbone, it is called a vertebrate. An endoskeleton grows with the animal, like the squirrel in Figure 24.2. The material making up the endoskeleton varies. Sea urchins and sea stars have endoskeletons made of calcium carbonate, sharks have endoskeletons made of cartilage, and fishes, amphibians, reptiles, birds, and mammals have endoskeletons made of bone.

An endoskeleton protects internal organs, provides support for the body, and can provide an internal brace for muscles to pull against. You will learn more about the human skeletal and muscular systems in Unit 9.

Habitats

Animal bodies have a variety of adaptations, such as those for feeding, digestion, and support. These body variations enable animals to live in numerous habitats. Vertebrates and invertebrates live in oceans, in freshwater, and on land. They can be found in deserts, grasslands, rain forests, polar regions, and all other land biomes and aquatic ecosystems.

Animal Cell Structure

No matter where an animal lives or what adaptations it has, its cells do not have cell walls. Recall from Chapter 7 that plants also are multicellular organisms, but their cells have cell walls. The cells of all animals, except sponges, are organized into structural and functional units called tissues. Recall from Chapter 9 that a tissue is a group of cells that is specialized to perform a specific function.

For example, nerve tissue is involved in the transmission of nerve impulses throughout the body and muscle tissue enables the body to move.

Beginning with Aristotle in the fourth century B.C. and continuing into the nineteenth century, living organisms were classified into two kingdoms—Animalia (animals) and Plantae (plants). In 1866, Ernst Haeckel, a German scientist, proposed adding a third kingdom called Protista. The organisms in this kingdom are mainly unicellular eukaryotes. Some protists have cell walls, while others do not, making them neither plant nor animal. During the 1960s, as more was learned about cell structure, bacteria and fungi were placed into their own kingdoms. Figure 24.3 illustrates how the classification of living things continues to develop.

Movement

The evolution of nerve and muscle tissues enables animals to move in ways that are more complex and faster than organisms in other kingdoms. This is one notable characteristic of the animal kingdom. A gecko running across a ceiling, a mosquito buzzing around your ear, and a school of minnows swimming against the current are all exhibiting movements unique to animals. Some animals are stationary as adults, yet most have a body form that can move during some stage of development.

Reproduction

Most animals reproduce sexually, although some species can reproduce asexually. Most commonly in sexual reproduction, male animals produce sperm and female animals produce eggs. Some animals, such as earth-worms, are hermaphrodites, which produce both eggs and sperm in the same animal body. In general, hermaphrodites produce eggs and sperm at different times, so another individual of the same species still is needed for sexual reproduction.

Fertilization occurs when the sperm penetrates the egg to form a fertilized egg cell called the zygote. Fertilization can be internal or external. Internal fertilization occurs when the sperm and egg combine inside the animal’s body. For example, male turtles fertilize the eggs of the female internally. External fertilization occurs when egg and sperm combine outside the animal’s body. This process requires an aquatic environment for the sperm to swim to the egg. In many fishes, the female lays eggs in the water and the male sheds sperm over the eggs, as shown in Figure 24.4.

Recall that asexual reproduction means that a single parent produces offspring that are genetically identical to itself. Although few animal species reproduce asexually, when they do, they use one or more methods to do so. Some of the common methods of asexual reproduction include:

•budding—an offspring develops as a growth on the body of the parent

•fragmentation—the parent breaks into pieces and each piece can develop into an adult animal

•regeneration—a new organism can regenerate, or regrow, from the lost body part if the part contains enough genetic information

•parthenogenesis—a female animal produces eggs that develop without being fertilized

Early development

In most animals, the zygote undergoes mitosis and a series of cell divisions to form new cells. After the first cell division, in which the zygote forms two cells, the developing animal is called an embryo. The embryo continues to undergo mitosis and cell division, forming a solid ball of cells. These cells continue to divide, forming a fluid-filled ball of cells called the blastula, as shown in Figure 24.5. During these early stages of development, the number of cells increases, but the total amount of cytoplasm in the embryo remains the same as that in the original cell. Therefore, the total size of the embryo does not increase during early development.

In animals such as lancelets, the outer blastula is a single layer of cells, while in animals such as frogs, there might be several layers of cells surrounding the fluid. The blastula continues to undergo cell division. Some cells move inward to form a gastrula—a two-cell-layer sac with an opening at one end. A gastrula looks like a double bubble—one bubble inside another bubble.

Look again at Figure 24.5. Notice how the diagrams of the two-cell stage, the 16-cell stage, and the blastula differ from the photographs of these same stages. The diagrams illustrate early development in embryos that develop inside the adult animal. The photographs illustrate early development in embryos that develop outside of the adult animal. The large ball that does not divide is the yolk sac. It provides food for the developing embryo.

Tissue development Notice in Figure 24.6 that the inner layer of cells in the gastrula is called the endoderm. The endoderm cells develop into the digestive organs and the lining of the digestive tract. The outer layer of cells in the gastrula is called the ectoderm. The ectoderm cells in the gastrula continue to grow and become the nervous tissue and skin.

Cell division in some animals continues in the gastrula until another layer of cells, called the mesoderm, forms between the endoderm and the ectoderm. In some animals, the mesoderm forms from cells that break away from the endoderm near the opening of the gastrula. In more highly evolved animals, the mesoderm forms from pouches of endoderm cells on the inside of the gastrula. As development continues, mesoderm cells become muscle tissue, the circulatory system, the excretory system, and, in some species of animals, the respiratory system.

Recall from Chapter 12 that Hox genes might be expressed in ways that give proteins new properties that cause variations in animals. Much of the variation in animal bodies is the result of changes in location, number, or time of expression of Hox developmental genes during the course of tissue development.

Animal Body Plans

Evolution of Animal Body Plans

Recall that the evolutionary tree is organized like a family tree, and the phylogeny of animals is represented by the branches. For example, all of the mammals in Figure 24.7 belong on the chordate branch of the tree. The trunk represents the earliest animals and the branches represent the probable evolution of the major phyla of animals from a common ancestor, as shown in Figure 24.8.

Anatomical features in animals’ body plans mark the branching points on the evolutionary tree. For example, animals without tissues are grouped separately from animals with tissues, and animals without segments are grouped separately from animals with segments. Recall from Chapter 17 that the relationships among animals on this tree are inferred by studying similarities in embryological development and shared anatomical features. This traditional phylogeny, with animals classified into 35 phyla, is still used by most taxonomists. However, molecular data suggest other relationships among animals. Recent molecular findings, based on comparisons of DNA, ribosomal RNA, and proteins, indicate that the relationships between arthropods and nematodes and between flatworms and rotifers might be closer than anatomical features suggest.

Development of Tissues

As animals evolved from the first multicellular forms, the first anatomical feature to indicate a major change in body plan was the development of tissues. Therefore, tissues mark the first branching point on the evolutionary tree. Notice in Figure 24.8 that the only animals without tissues are sponges. These animals descended from a common ancestor that lacked tissues, and they are on the no-true-tissue branch of the evolutionary tree. Follow the tissue branch of the evolutionary tree, and you will see that all other phyla have tissues.

Symmetry

Move along the tissue branch on the evolutionary tree in Figure 24.8 and you will find the next branching point to be symmetry. Symmetry describes the similarity or balance among body structures of organisms. The type of symmetry an animal has enables it to move in certain ways.

Asymmetry The sponge in Figure 24.9 has no tissue and has asymmetry—it is irregular in shape and has no symmetry or balance in its body structures. In contrast, animals with tissues have either radial or bilateral symmetry.

Radial symmetry

An animal with radial symmetry can be divided along any plane, through a central axis, into roughly equal halves. The jellyfish in Figure 24.9 has radial symmetry. Its tentacles radiate from its mouth in all directions—a body plan adapted to detecting and capturing prey moving in from any direction. Jellyfishes and most other animals with radial symmetry develop from only two embryonic cell layers—the ectoderm and the endoderm.

Bilateral symmetry

The bird in Figure 24.9 has bilateral symmetry. In contrast to radial symmetry, bilateral symmetry means the animal can be divided into mirror image halves only along one plane through the central axis. All animals with bilateral symmetry develop from three embryonic cell layers—the ectoderm, the endoderm, and the mesoderm.

Cephalization Animals with bilateral symmetry also have an anterior, or head end, and a posterior, or tail end. This body plan is called cephalization—the tendency to concentrate nervous tissue and sensory organs at the anterior end of the animal. Most animals with cephalization move through their environments with the anterior end first, encountering food and other stimuli. In addition to cephalization, animals with bilateral symmetry have a dorsal surface, also called the backside, and a ventral surface, also called the underside or belly.

Body Cavities

In order to understand the next branching point on the evolutionary tree, it is important to know about certain features of animals with bilateral symmetry.

Body plans of animals with bilateral symmetry include the gut, which is either a sac inside the body or a tube that runs through the body, where food is digested. A saclike gut has one opening—a mouth— for taking in food and disposing of wastes. A tube-like gut has an opening at both ends—a mouth and an anus—and is a complete digestive system that digests, absorbs, stores, and disposes of unused food.

Coelomates Between the gut and the outside body wall of most animals with bilateral symmetry is a fluid-filled body cavity. One type of fluid-filled cavity, the coelom, shown in Figure 24.10, has tissue formed from mesoderm that lines and encloses the organs in the coelom. You have a coelom, as do insects, fishes, and many other animals, therefore, you are a coelomate. The coelom was a key adaptation in the evolution of larger and more specialized body structures. Specialized organs and body systems that formed from mesoderm developed in the coelom. As more efficient organ systems evolved, such as the circulatory system and muscular system, animals could increase in size and become more active.

Pseudocoelomates Follow the body cavity branch on the evolutionary tree in Figure 24.8 until you come to the pseudocoelomates—animals with pseudocoeloms. A pseudocoelom is a fluid-filled body cavity that develops between the mesoderm and the endoderm rather than developing entirely within the mesoderm as in coelomates. Therefore, the pseudocoelom, as shown in Figure 24.10, is lined only partially with mesoderm. The body cavity of pseudocoelomates separates mesoderm and endoderm, which limits tissue, organ, and system development.

Acoelomates Before the body cavity branch on the evolutionary tree in Figure 24.8, notice that the branch to the left takes you to the acoelomate animals. Acoelomates, such as the flat-worm in Figure 24.10, are animals that do not have a coelom. The body plan of acoelomates is derived from ectoderm, endoderm, and mesoderm—the same as in coelomates and pseudocoelomates. However, acoelomates have solid bodies without a fluid-filled body cavity between the gut and the body wall. Nutrients and wastes diffuse from one cell to another because there is no circulatory system.

Development in Coelomate Animals

The evolutionary tree in Figure 24.11 begins at the coelomate branch. Notice that two major lines of development have been identified in coelomate animals. One is protostome development, which occurs in animals such as snails, earthworms, and spiders. The other is deuterostome development, which occurs in animals such as sea urchins, dogs, and birds. Biologists can tell if animals are closely related based on their patterns of embryonic development.

Protostomes In organisms that are protostomes, the mouth develops from the first opening in the gastrula. As protostomes develop, the final outcome for each cell in the embryo cannot be altered. If one cell of the embryo is removed, the embryo will not develop into a normal larva, as shown in Figure 24.12. In addition, in the eight-cell stage of embryonic development, the top four cells are offset from the bottom four cells, giving the embryo a spiral appearance. As the embryo continues to develop, the mesoderm splits down the middle. The cavity between the two pieces of mesoderm becomes the coelom.

Deuterostomes In organisms that are deuterostomes, the anus develops from the first opening in the gastrula. The mouth develops later from another opening of the gastrula. During the development of deuterostomes, the final outcome for each cell in the embryo can be altered. In fact, each cell in the early embryo, if removed, can form a new embryo, as shown in Figure 24.12. In contrast to protostome development, in the eight-cell stage of embryonic deuterostome development the top four cells are directly aligned on the bottom four cells. As the embryo develops, the coelom forms from two pouches of mesoderm.

Segmentation

Examine the next branching point on the evolutionary tree in Figure 24.13. Segmentation is an important feature in the evolution of coelomate animals. Just as a chain is constructed from a series of links, segmented animals can be “put together” from a succession of similar parts.

The segmentation, such as that seen in scorpions, has two advantages. First, segmented animals can survive damage to one segment because other segments might be able to carry out the damaged section’s function. Second, movement is more effective because segments can move independently. Therefore, the scorpion in Figure 24.13 has more flexibility and can move in ways that are very complex. Segments allow the scorpion to arch its tail over its back to sting prey.

Sponges and Cnidarians

Sponges

If you examine a living sponge, you might wonder how these animals do so much with so little. They have no tissues, no organs, and most have no symmetry. You can break apart a sponge into its individual cells and those cells will come together again to form a sponge. Other animals cannot do this.

Locate sponges on the evolutionary tree in Figure 24.14. They are in the phylum Porifera, which contains between 5000 and 10,000 members. Most live in marine environments. Biologists hypothesize that sponges evolved from the colonial choanoflagellates because sponges have cells that look similar to these protist cells.

Body structure Notice the asymmetrical appearance and bright colors of the sponge in Figure 24.14. It is difficult to think that these are animals, especially if you see one washed up on a beach where it might appear as a black blob. Recall that tissues form from ectoderm, endoderm, and mesoderm in a developing embryo. Sponge embryos do not develop endoderm or mesoderm, and, therefore, sponges do not develop tissues. How does a sponge’s body function without tissues?

Two layers of independent cells with a jellylike substance between the layers accomplish all of the life functions of sponges. As illustrated in Figure 24.15, epithelial-like cells cover the sponge and protect it.

Collar cells with flagella line the inside of the sponge. As collar-cell flagella whip back and forth, water is drawn into the body of the sponge through pores. These pores give sponges their phylum name Porifera, which means “pore-bearer.” Water and waste materials are expelled from the sponge through the osculum, which is the mouthlike opening at the top of the sponge.

Feeding and digestion

When an organism such as a sponge gets its food by filtering small particles from water, it is called a filter feeder. Even though this might sound like a process that is not very active, consider that a sponge only 10 cm tall can filter as much as 100 L of water each day. Although sponges have free-swimming larvae, the adults move very little. Adaptations for filter-feeding are common in animals that are sessile—meaning they are attached to and stay in one place. As nutrients and oxygen dissolved in water enter through the pores in a sponge’s body, food particles cling to the cells. Digestion of nutrients takes place within each cell.

Support Within the jellylike material that lies between the two cell layers of a sponge are amoeba-like cells—cells that can move and change shape. These amoeba-like cells are called archaeocytes and are illustrated in Figure 24.15. These cells are involved in digestion, production of eggs and sperm, and excretion. Archaeocytes also can become specialized cells that secrete spicules—the support structures of sponges. Spicules are small, needlelike structures made of calcium carbonate, silica, or a tough fibrous protein called spongin.

Sponge diversity Biologists place sponges into three classes based on the type of support system they have. Most sponges belong to class Demospongiae, the demosponges, and have spicules composed of spongin fibers, silica, or both. Natural bath sponges, like the ones in Figure 24.16, have spongin support. Class Calcarea consists of sponges with spicules composed of calcium carbonate. Calcareous sponges, like the one in Figure 24.17, often have a rough texture because the calcium carbonate spicules can extend through the outer covering of the sponge. The sponges in class Hexactinellida are called glass sponges and have spicules composed of silica. These spicules join together to form a netlike skeleton that often looks like spun glass, as illustrated in Figure 24.17.

Response to stimuli

A sponge does not have a nervous system. They do have epithelial-like cells that detect external stimuli, such as touch or chemical signals, and respond by closing their pores to stop water flow.

Reproduction Sponges can reproduce asexually by fragmentation, through budding, or by producing gemmules. In fragmentation, a piece of sponge that is broken off due to a storm or other event develops into a new adult sponge. In budding, a small growth, called a bud, forms on a sponge, drops off, and settles in a spot where it grows into a new sponge. Some freshwater sponges form seedlike particles called gemmules during adverse conditions like droughts or freezing temperatures. Gemmules contain sponge cells protected by spicules that will survive and grow again when favorable conditions occur.

Most sponges reproduce sexually, illustrated in Figure 24.18. Some sponges have separate sexes, but most sponges are hermaphrodites.

Recall that a hermaphrodite is an animal that can produce both eggs and sperm. During reproduction, eggs remain within a sponge, while sperm are released into the water. Sperm released from one sponge can be carried by water currents to the collar cells of another sponge. The collar cells then change into specialized cells that carry the sperm to an egg. After fertilization occurs, the zygote develops into a larva that is free-swimming and has flagella. The larva eventually attaches to a surface, then develops into an adult.

Sponge ecology

Although spicules and toxic or distasteful compounds in sponges discourage most potential predators, sponges are food for some tropical fishes and turtles. Sponges also are common habitats for a variety of worms, fishes, shrimp, and colonies of symbiotic green algae. Some sponges even live on and provide camouflage for mollusks, as shown in Figure 24.19.

Sponges also are beneficial to humans. Sponges with spicules made of sponging fibers often are used for household scrubbing purposes. Medical research is focusing on sponge chemicals that appear to discourage prey and prevent infection. Ongoing studies of these sponge chemicals as possible pharmaceutical agents have shown that they might have antibiotic, anti-inflammatory, or antitumor possibilities. They also might have potential importance as respiratory, cardiovascular, and gastrointestinal medicines.

For example, researchers discovered a powerful antitumor substance in the deep water sponge shown in Figure 24.20. This substance, discodermolide, stops cancer cells from dividing by breaking down the nucleus and rearranging the microtubule network. Recall from Chapter 7 that microtubules are part of a cell’s skeleton and help the cell maintain its shape. Note the differences in the nuclei and microtubules between the untreated and treated cancer cells in Figure 24.20.

Cnidarians

Imagine that you go snorkeling around a coral reef, and you wear a bodysuit to protect yourself from the stings of jelly-fishes that float on the water. Later, when you go ashore to visit a tidepool, you might see colorful sea anemones that look somewhat like flowers. The jellyfish and sea anemone in Figure 24.21 belong to phylum Cnidaria. This phylum consists of about 10,000 species, most of which are marine.

Body structure

Like sponges, cnidarians have one body opening and most have two layers of cells.

However, in cnidarians, the two cell layers are organized into tissues with specific functions. The outer layer functions in protecting the internal body, while the inner layer functions mainly in digestion. Because cnidarians have tissues, they also have symmetry. As shown in Figure 24.21, cnidarian bodies have radial symmetry. Recall that radial symmetry enables slow moving or sessile animals to detect and capture prey from any direction. Cnidarians are adapted to aquatic floating or sessile attachment to surfaces under the water.

Feeding and digestion Cnidarian tentacles are armed with stinging cells called cnidocytes. Cnidarians get their name from these stinging cells. Cnidocytes contain nematocysts, as shown in Figure 24.22. A nematocyst is a capsule that holds a coiled, threadlike tube containing poison and barbs.

A nematocyst works like a tiny but very powerful harpoon. Recall from Chapter 7 that osmosis is the diffusion of water through a selectively permeable membrane. The pressure provided by this flow of water is called osmotic pressure. The water inside an undischarged nematocyst is under an osmotic pressure of more than 150 atmospheres. This pressure is about 20 times the pressure in an inflated bicycle tire.

In response to being touched or to a chemical stimulus, the permeability of the nematocyst membrane increases, allowing more water to rush in. As the osmotic pressure increases, the nematocyst discharges forcefully. A barb is capable of penetrating a crab shell.

Nematocyst discharge is one of the fastest cellular processes in nature. It happens so quickly—in just 3/1000ths of a second—that it is impossible to escape after touching these cells. After capture by nematocysts and tentacles, the prey is brought to the mouth of the cnidarian.

The inner cell layer of cnidarians surrounds a space called the gastrovascular cavity, illustrated in Figure 24.23. Cells lining the gastrovascular cavity release digestive enzymes over captured prey. Undigested materials are ejected through the mouth. Recall that digestion occurs within each cell of a sponge. However, in cnidarians, digestion takes place in the gut cavity—a major evolutionary adaptation.

Response to stimuli In addition to cells adapted for digestion, cnidarians have a nervous system consisting of a nerve net that conducts impulses to and from all parts of the body. The impulses from the nerve net cause contractions of musclelike cells in the two cell layers. The movement of tentacles during prey capture is the result of contractions of these musclelike cells. Cnidarians have no blood vessels, respiratory systems, or excretory organs. Look at Table 24.1 to compare the structures and functions of sponges and cnidarians.

Reproduction In addition to stinging cells, cnidarians have another adaptation not seen in most animals of recent origin. Most cnidarians have two body forms: a polyp with a tube-shaped body and a mouth surrounded by tentacles, and a medusa with an umbrella-shaped body and tentacles that hang down. The mouth of a medusa is on the ventral surface between the tentacles.

The two body forms of cnidarians can be observed in the life cycle of jellyfishes, illustrated in Figure 24.24. To reproduce, jellyfishes in the **medusa stage** release eggs and sperm into the water where fertilization occurs. The resulting zygotes eventually develop into free-swimming larvae that settle and grow into polyps. These polyps reproduce asexually to form new medusae. It would be easy to confuse the life cycle of cnidarians with the alternation of generations you studied in plants. However, in plants, one generation is diploid and the other is haploid. In cnidarians, both the medusae and polyps are diploid animals.

Cnidarian diversity There are four main classes of cnidarians: Hydrozoa, the hydroids; two classes of jellyfishes—Scyphozoa and Cubozoa; and Anthozoa, the sea anemones and corals.

Hydroids Most of the approximately 2700 species of hydroids have both **polyp and medusa stages** in their life cycles. Most hydroids form colonies, such as the Portuguese man-of-war in Figure 24.25. Another well-known hydroid is the freshwater hydra, which is unusual because it has only a **polyp stage**.

Jellyfishes There are about 200 species of jellyfishes. They are transparent or translucent in appearance and float near the water’s surface. The medusa is the dominant body form, although a **polyp stage** does exist. They are called jellyfishes because the substance between the outer body covering and the inner body wall is jellylike. The structure of the inner and outer body layers with the jellylike structure between can be compared to a jelly sandwich. The box jellyfishes take their name from the boxlike medusae that are their dominant form. The stings of some box jellyfish species can be fatal to humans.

Sea anemones and corals

Generally colorful and inviting, sea anemones and corals still possess stinging cells like all cnidarians. The 6200 species of sea anemones and corals are different from the jellyfishes because the polyp stage is the dominant stage of their life cycles. Recent research indicates that these anthozoans actually might have bilateral symmetry. This would alter the evolutionary tree because this adaptation usually is seen only in animal groups that evolved later than cnidarians.

Sea anemones live as individual animals, while corals live in colonies of polyps. Corals secrete protective calcium carbonate shelters around their soft bodies. The living portion of a coral reef is a thin, fragile layer growing on top of the shelters left behind by previous generations. Coral reefs form from these shelters over thousands of years.

Coral polyps extend their tentacles to feed, as shown in Figure 24.26. They also harbor symbiotic photosynthetic protists called zooxanthellae. The zooxanthellae produce oxygen and food that corals use, while using carbon dioxide and waste materials produced by the corals. These protists are primarily responsible for the bright colors found on healthy coral reefs.

The health of a coral reef depends on proper water temperature, adequate light, and appropriate water depth. If these environmental conditions deteriorate in areas where there are reefs, the health of the reef might also deteriorate. You can examine this problem in Data Analysis Lab 24.1.

Cnidarian ecology Mutualism, a relationship in which both organisms benefit, is common in cnidarians. One species of sea anemone wraps itself around hermit crabs’ shells; the anemones obtain food scraps and the crabs are protected. Some sea slugs feed on cnidarians and incorporate the unfired nematocysts into their bodies for their own defense. As shown in the photo at the beginning of the chapter, clown fishes are protected by the tentacles of anemones. One theory as to how clown fishes are protected from the tentacles of anemones is that the fish incorporates mucus from an anemone into its own mucous coating, which prevents the nematocysts from discharging.

People benefit from cnidarians in many ways. Some people enjoy visiting a coral reef. In the medical field, some stony coral species are used in surgical procedures. A calcium phosphate mineral in coral called hydroxyapatite can be treated so that it has the same structure and chemical composition as human bone.

Small pieces of coral are implanted as bone grafts, especially in face and jaw reconstruction and in arm and leg surgery. The grafts anchor to the adjacent bone, as shown in Figure 24.27, and eventually are replaced by new human bone growth.

Evolution of cnidarians

There are two major interpretations of the phylogeny of cnidarians. The fact that cnidarians have two body forms—medusa and polyp—raises the question of whether the ancestral cnidarian had a medusa or a polyp body form. The cladograms in Figure 24.28 present both interpretations.

In the cladogram on the left, the ancestral cnidarian has a medusa body form. As cnidarians evolved, a **polyp stage** developed. The life cycles of hydrozoans have both **polyp and medusa stages**. As the scyphozoans and cubozoans developed, the medusa stage became the dominant stage in their life cycles. The most highly evolved cnidarians, the anthozoans, have no **medusa stage**.

In the cladogram on the right, the ancestral cnidarian has a polyp body form. The anthozoans evolved first, and the polyp stage is the dominant stage of their life cycles. The **medusa stage** evolved independently in hydrozoans and in scyphozoans and cubozoans. As you examine the cladograms, notice how the classes of cnidarians are arranged in each.

Flatworms

Body Structure

The evolutionary tree in Figure 25.1, shows that flatworms are on the acoelomate branch of the tree, while roundworms are on the pseudocoelomate branch. However, flatworms and roundworms both have bilateral symmetry—they can be divided along only one plane into mirror-image halves. Bilateral symmetry is a major evolutionary step that allows parts of the body to evolve different organs. Animals that have bilateral symmetry also have more efficient movement than animals with radial symmetry.

Phylum Platyhelminthes—the flatworms— consists of about 20,000 species. Figure 25.1 shows some of the variety seen in flatworms. Flatworms range in length from many meters to 1 mm or less. They have thin, flat bodies that resemble a ribbon. Unlike sponges and cnidarians, flatworms have a definite head region and body organs. Flatworms are acoelomates. They lack a coelom—their bodies have no cavities.

Most flatworms are parasites living in the bodies of a variety of animals, while others are free-living in marine, freshwater, or moist land habitats. Freshwater planarians often are seen on the underside of rocks in swiftly flowing streams.

Feeding and digestion

Free-living flatworms feed on dead or slow-moving organisms. They extend a tubelike muscular organ, called the pharynx, out of their mouths. The pharynx, shown in Figure 25.2, releases enzymes that begin the digestion of prey. Then food particles are sucked into the digestive tract where digestion continues. Because flatworms have only one body opening, wastes are ejected through the mouth.

Parasitic flatworms have modified feeding structures called hooks and suckers, which enable them to stay attached to their hosts. Some parasitic flatworms have a reduced digestive system and feed on blood and other body tissues. Other parasitic flatworms lack a digestive system. Because they are so thin, like a single layer of cloth, and are surrounded by nutrients in their host’s intestines, these parasites can absorb directly through their body walls partially or completely digested food eaten by the host.

Respiration, circulation, and excretion

Like sponges and cnidarians, flatworms do not have circulatory organs or respiratory organs. Because flatworms are so thin, their cells can use the process of diffusion to move dissolved oxygen and nutrients to all parts of their bodies. Carbon dioxide and other wastes also are removed from flatworm cells by diffusion.

Unlike sponges and cnidarians, flatworms have an excretory system that consists of a network of small tubes that run through the body. On side branches of the tubes, as shown in Figure 25.2, bulblike flame cells lined with cilia sweep water and excretory substances into tubules. These substances then exit through pores to the outside of the body. Flame cells were named because the flickering movements of the cilia inside the cells look like the light of a candle flame. Because flame cells move water out of the body, they keep flatworm cells from becoming waterlogged. In addition to the action of flame cells, flatworms also excrete waste products and maintain homeostatic water balance through their mouths.

Response to stimuli

The nervous system regulates the body’s response to stimuli. In most flatworms, the nervous system consists of two nerve cords with connecting nerve tissue that run the length of the body. In most flatworms, the connecting nerve tissue looks like the rungs of a ladder, as illustrated in Figure 25.2. At the anterior end of the nerve cords is a small swelling composed of ganglia that send nerve signals to and from the rest of the body. A ganglion is a group of nerve cell bodies that coordinates incoming and outgoing nerve signals.

Movement

Some flatworms move by contracting muscles in the body wall. To escape predators and to find food, most free-living flatworms glide by using cilia located on their undersides. Mucus lubricates the worms and improves the gliding motion, while muscular action lets the animals twist and turn. If you ever have tried to loosen planaria worms from the bottoms of rocks, you know that their outer mucus covering enables them to stick tightly—an important adaptation in a swiftly moving stream.

Reproduction Flatworms are hermaphrodites because they produce both eggs and sperm. During sexual reproduction, two different flatworms exchange sperm, and the eggs are fertilized internally. In marine flatworms, zygotes in cocoons are released into the water where they hatch within a few weeks.

Free-living flatworms can reproduce asexually by regeneration—a process in which body parts that are missing due to damage or predation can be regrown. A planarian that is cut in half horizontally can grow a new head on the tail end and a new tail on the head end, forming two new organisms, as shown in Figure 25.3.

Diversity of Flatworms

There are three main classes of flatworms: Turbellaria, Trematoda, and Cestoda. Class Turbellaria consists of the free-living flatworms. Class Trematoda and class Cestoda consist of parasitic flatworms.

Turbellarians Members of the class Turbellaria are called turbellarians. Most turbellarians, like planarians, live in marine or freshwater habitats, while some live in moist soils. They vary in size, color, and body shape. As shown in Figure 25.4, turbellarians have eyespots that can detect the presence or absence of light. They also have sensory cells that help them identify chemicals and water movement.

The cells sensitive to chemicals are concentrated on small projections called auricles at the anterior end of the worm. When a planarian hunts, it might wave its head back and forth as it crawls forward, exposing the auricles to chemical stimuli coming from food. At the same time, its eyespots might help it perceive light conditions that would protect it from predators.

Trematodes Flukes belong to class Trematoda—the trematodes. They are parasites that infect the blood or body organs of their hosts. The life cycle of the parasitic fluke Schistosoma is shown in Figure 25.5. Notice that this parasite requires two hosts to complete its life cycle.

When humans contract schistosomiasis, the fluke eggs clog blood vessels, causing swelling and eventual tissue damage. Schistosomiasis can be prevented by proper sewage treatment and by wearing protective clothing when wading or swimming in infested water. Schistosomiasis infections are not common in the United States.

Cestodes All tapeworms are members of class Cestoda—the cestodes. They are parasites adapted to life in the intestines of their hosts. Look at the anterior end, or head, of the tapeworm in Figure 25.6. This is the scolex, a knob-shaped structure with hooks and suckers that attach to the intestinal lining of a host such as a cow or a human.

Behind the scolex of the worm are a series of individual sections called proglottids, each of which contains muscles, nerves, flame cells, and male and female reproductive organs. Proglottids form continuously; as new ones form near the scolex, older proglottids move farther back and mature. After eggs in the mature proglottids are fertilized, the last segments with developing embryos break off and pass out of the intestines of their hosts. Animals such as cattle might feed on vegetation or drink water contaminated by the tapeworm proglottids, and then the cycle of tapeworm growth is repeated.

When eaten by cattle, tapeworms can burrow through intestinal walls, entering blood and eventually muscle. If this infected beef is eaten rare or undercooked, human infection by tapeworms is likely. Tapeworm infections are uncommon in developed countries because these countries require beef inspections.

Roundworms and Rotifers

Body Structure of Roundworms

Roundworms are in phylum Nematoda and often are called nematodes. Locate roundworms on the evolutionary tree in Figure 25.7. Notice that they have a body cavity in the form of a pseudocoelom. Roundworms have bilateral symmetry and are cylindrical, unsegmented worms that are tapered at both ends. Roundworms come in many sizes, as shown in Figure 25.7. Most are less than 1 mm long. However, the longest known roundworm, living in certain whales, can grow to 9 m in length.

Roundworms are found in both marine and freshwater habitats and on land. Some are parasites on plants and animals. A spadeful of garden soil might contain one million roundworms. One study revealed that a rotting apple contained 1074 roundworms! Dogs and cats can be plagued by roundworms if they are not wormed when they are young and at regular intervals during adulthood. Roundworms have adaptations that enable them to live in many places.

Feeding and digestion

Most roundworms are free-living, but some are parasites. Some free-living roundworms are predators of other tiny invertebrates, while others feed on decaying plant and animal matter. Free-living forms have a key evolutionary adaptation in their digestive systems. Recall from Chapter 24 that in the course of evolution, pseudocoelomate animals were the first to have a body cavity. The pseudocoelom of a nematode separates the endoderm-lined gut from the rest of the body. The movement of food through the gut, or digestive tract, is one-way—food enters through the mouth, and undigested food leaves through an opening at the end of the digestive tract called the anus.

Respiration, circulation, excretion, and response to stimuli

Like flatworms, roundworms have no circulatory organs or respiratory organs, and they depend on diffusion to move nutrients and gases throughout their bodies. Most roundworms exchange gases and excrete metabolic wastes through their moist outer body coverings. More complex forms have excretory ducts that enable them to conserve water for living on land, while others have flame cells.

Ganglia and associated nerve cords coordinate nematode responses.

Nematodes are sensitive to touch and to chemicals. Some have structures that might detect differences between light and dark.

Movement Roundworms have muscles that run the length of their bodies. These muscles cause their bodies to move in a thrashing manner as one muscle contracts and another relaxes. These muscles also pull against the outside body wall and the pseudocoelom. The pseudocoelom acts as a hydrostatic skeleton—fluid within a closed space that provides rigid support for muscles to work against. If you were to observe a roundworm moving, it might resemble a tiny piece of wriggling thread. Learn more about worm movement in Data Analysis Lab 25.1.

Reproduction Roundworms reproduce sexually. The females produce eggs, and the males, which often are smaller than the females, produce sperm. Fertilization is internal. In free-living roundworms, larvae hatch from the fertilized eggs, then grow into adults. In parasitic roundworms, development often is more complicated, involving one or more hosts or different locations in the host’s body.

The adult roundworm Caenorhabditis elegans (C. elegans), shown in Figure 25.8, contains only 959 cells; zygotes mature to adults in just three days. These characteristics make it an extremely important subject of research on development, aging, and genetics. C. elegans was the first multicellular organism to have its entire genome sequenced. The

C. elegans genome contains 97 million DNA bases encoding over 19,000 different genes. See the BioDiscoveries feature at the end of this chapter to learn more about C. elegans.

Diversity of Roundworms

Of the 20,000 known roundworm species, approximately half are parasites. These parasitic roundworms cause a variety of diseases in plants and animals, including humans. Many of these diseases in humans are the result of carelessness, a lack of personal hygiene, or poor sanitation.

Trichinella worms

A disease called trichinosis can be contracted by eating raw or undercooked pork and pork products, or wild game infected with the larvae of Trichinella, like the one shown in Figure 25.9. After ingestion by a host organism, the worms mature in one to two days. Female worms with fertilized eggs burrow into the intestinal walls of humans, pigs, and other mammals. After the eggs hatch, the larvae burrow into muscles where they form cysts, causing muscle pain. Trichinosis can be prevented by cooking meat properly.

Hookworms Hookworm infections are common in warm climates when people go barefoot on contaminated soil. When a hookworm, shown in Figure 25.10, contacts bare human skin, it cuts its way inside, travels in the bloodstream to the lungs, and then to the windpipe, or esophagus, where it is coughed up and swallowed. The parasite then moves to the small intestine, where it attaches to intestinal walls and feeds on blood and other tissue. Hookworm infection can be prevented by wearing shoes.

Ascarid worms

The most common worm infection in humans is ascariais, which is caused by ascarid worms like the ones shown in Figure 25.10. Eggs of ascarid worms are found in soil in subtropical and tropical areas. They enter the human body through the mouth and live in the intestine. Infection can result when unwashed vegetables from contaminated soil are eaten or when hands contaminated with infected soil are put in the mouth, as young children are likely to do. Infection by ascarid worms can be controlled by carefully washing vegetables and hands.

Pinworms Figure 25.10 shows a pinworm—the most common nematode parasite in humans in the United States. The highest incidence of infection occurs in children. At night, female pinworms living in the intestine move out of the anus and lay eggs on nearby skin. When the skin is scratched because of the itching caused by pinworm activity, the eggs are transferred to hands and then to any surface that is touched. These eggs can survive for up to two weeks on surfaces and are ready to hatch if another person ingests them. This infection can spread quickly among children who put toys and other objects in their mouths.

Filarial worms Elephantiasis is a disease caused by filarial worms—roundworm parasites that live in tropical areas. A mosquito is the intermediate host of filarial worms. When a mosquito sucks blood from a person who is infected with this roundworm, worm embryos are passed into the insect’s bloodstream. The embryos grow into larvae, which then are passed to another person when the mosquito bites again. Adult worms accumulate in the lymphatic system and obstruct the flow of lymph—the tissue fluid in the spaces between cells. This fluid builds up in tissue, causing legs and other body regions to enlarge. Controlling mosquitoes and using mosquito netting at night can aid in preventing this disease.

Another disease caused by a filarial worm—heartworm—is found in dogs and cats throughout the United States. Heartworms are transmitted to dogs and cats through mosquito bites. Once in the bloodstream, the worms travel to the heart and block the flow of blood. Regular doses of oral medications prevent heartworm in dogs and cats.

Nematodes in plants

Some species of roundworms cause diseases in plants. Nematodes can infect and kill pine trees, soybean crops, and food plants such as tomatoes. When they infect plant roots, as shown in Figure 25.11, they damage the plant.

Most species of nematodes are either harmless or beneficial to plants. Certain nematodes are used to control the spread of cabbage worm caterpillars, Japanese beetle grubs, and many other pests of crop plants. Spraying a solution of nematodes and water on areas that are infested with crop pests is most effective when the targeted pest is at the stage in its life cycle when it lives in the soil.

In addition to treating plant pests, nematodes are used to control pests of humans and animals. Nematodes eat flea larvae, controlling the flea population in yards. This reduces or eliminates exposure of humans and animals to traditional chemicals used to treat flea infestations.

Rotifers

Rotifers are tiny animals only about 0.1 mm to 0.5 mm in length. The phylum Rotifera, meaning wheel-bearer, gets its name from the rings of cilia around the mouths of the animals. About 1800 species of rotifers have been studied, mostly from freshwater habitats including ponds, streams, and lakes. It would not be unusual to find 40 to 500 rotifers in a liter of pond water. A few species are marine. Refer back to the evolutionary tree in Figure 25.7 and see that, although rotifers and round-worms occupy separate branches, both are pseudocoelomates.

Rotifer features and movement Rotifers are similar to roundworms because they have bilateral symmetry and are pseudocoelomates with a gut open at both ends. Unlike round-worms, rotifers move through the water by means of their ciliated wheel-like structures, which are shown in Figure 25.12. The posterior end of a rotifer generally has “toes” and glands that secrete an adhesive material that enables a rotifer to attach itself to a surface in the water.

Organ systems of rotifers Rotifers feed by using cilia to gather protists and organic materials into a complete digestive tract, which includes a mouth and an anus. Like other pseudocoelomates, rotifers exchange gases and excrete metabolic wastes by diffusion through body walls. Sensory structures include sensory bristles and eyespots on the head. Some rotifers reproduce sexually, while others have complex life cycles involving diploid eggs producing diploid females and haploid eggs producing haploid males. These life cycles are dependent on environmental conditions, such as spring rains, frost, or the presence of stagnant water.

Mollusks

Body Structure

Mollusks are members of the phylum Mollusca. They range from the slow-moving slug to the jet-propelled squid, from scallops and cuttle-fish to chitons and nudibranchs. Mollusks range in size from almost microscopic snails to giant squids, which can grow to be 21 m long.

Look at the evolutionary tree in Figure 25.13. Mollusks, such as the nudibranch and the octopus in Figure 25.13, undergo protostome development and might have been the first animals in the course of evolution to have a coelom, which allowed for the development of more complex tissues and organs. There are more than 110,000 species of mollusks. Many are marine, some live in freshwater, and others live in moist land environments.

Mollusks are coelomate animals with bilateral symmetry, a soft internal body, a digestive tract with two openings, a muscular foot, and a mantle. The mantle is a membrane that surrounds the internal organs of the mollusk. In mollusks with shells, the mantle secretes calcium carbonate to form the shell. Other mollusks, including slugs and squids, are adapted to life without a hard outer covering.

Compare the bodies of the snail and the squid in Figure 25.14. Their external features are very different from each other. However, both have coelomate body plans and highly evolved body systems, such as the digestive system, respiratory system, circulatory system, and nervous system.

Feeding and digestion Many mollusks use a rasping structure called a radula to scrape food into their mouths. Located in a mollusk’s mouth, a radula is a tonguelike organ with rows of teeth, as shown in Figure 25.15. Herbivorous mollusks use their radulas to scrape algae off rocks. Carnivorous mollusks use their radulas to drill into other mollusks and feed on their internal body parts. Some of these predators, such as octopuses and squids, use their radulas to tear up the food they capture with their tentacles. Other mollusks, such as clams, are filter feeders and do not have radulas.

Mollusks have complete guts with digestive glands, stomachs, and intestines, as shown in Figure 25.16. As in roundworms, the digestive system has two openings—a mouth and anus.

Respiration

Most mollusks have respiratory structures called gills. Gills, shown in Figure 25.16, are parts of the mantle that consist of a system of filamentous projections like the fringes of a blanket. Gills contain a rich supply of blood for the transport of oxygen to the blood and for the removal of carbon dioxide from the blood. Gills move water into and through the mantle cavity in a continuous stream. They are highly branched structures, which increase the surface area through which gases can diffuse. This enables the gills to take in more oxygen from water. Land snails and slugs remove oxygen from the air using the lining of their mantle cavities. In some mollusks, the gills also function in filter feeding.

Circulation Mollusks have a well-developed circulatory system that includes a chambered heart. Most mollusks have an open circulatory system, in which the blood is pumped out of vessels into open spaces surrounding the body organs. This adaptation enables animals to diffuse oxygen and nutrients into tissues that are bathed in blood and also to move carbon dioxide from tissues into the blood. Slow-moving animals, such as snails and clams, utilize this system effectively because they do not need rapid delivery of oxygen and nutrients for quick movements.

Some mollusks, such as squids, move nutrients and oxygen through a closed circulatory system, which was a major adaptation in the evolution of animals. In a closed circulatory system, blood is confined to vessels as it moves through the body. A closed system efficiently transports oxygen and nutrients to cells where they are converted to usable forms of energy. Mollusks that move quickly, such as the octopus and squid, need more energy than slow-moving mollusks, and the closed circulatory system quickly delivers nutrients and oxygen. A closed circulatory system is like the heating ducts in some houses. A furnace is efficient at delivering warm air to the rooms in a house because the air travels through a series of ducts or pipes. Rooms of a house would not be evenly heated if the furnace did not have a delivery system.

Excretion

Most mollusks get rid of metabolic wastes from cellular processes through structures called nephridia, shown in Figure 25.16. After nephridia filter the blood, waste is passed out through the mantle cavity. Nephridia are an evolutionary adaptation enabling mollusks to efficiently maintain homeostasis in their body fluids.

Response to stimuli Mollusks have nervous systems that coordinate their movements and behavior. Mollusks that are more highly evolved, such as octopuses, have a brain. In addition, octopuses have complex eyes similar to human eyes with irises, pupils, and retinas.

Most mollusks have simple structures in the eyes that reflect light.

Movement The muscular foot of a clam enables it to burrow into wet sand. Mollusks with two shells can clap their shells together for short bursts of rapid swimming. Most slugs and snails creep along moist areas on a slime trail of mucus secreted by glands in the foot. Octopuses and squids take water into the mantle cavity and expel it through a tube called a siphon. When threatened, they can eject the water so rapidly that they appear to be jet-propelled. Figure 25.17 illustrates the ways mollusks move.

Reproduction Mollusks reproduce sexually, as illustrated in Figure 25.18. The males and females of most aquatic species release their eggs and sperm into the water at the same time, and fertilization is external. A few bivalves and many gastropods that live on land are hermaphrodites, in which fertilization takes place internally.

All members of the phylum Mollusca share similar developmental patterns, even though their adult forms vary widely. One larval stage of most mollusks—the trochophore—looks very similar to the larval stage of the next group of animals you will study—the segmented worms. Because the larval forms are similar in both segmented worms and mollusks, scientists hypothesize that segmented worms and mollusks are closely related.

Diversity of Mollusks

Animals in the three major classes of mollusks—gastropods, bivalves, and cephalopods—are grouped based on differences in their shell and foot structures.

Gastropods

The largest class of mollusks is Gastropoda, the stomach-footed mollusks. The name comes from the way the animal’s large foot is positioned under the stomach on the ventral surface. Most species of gastropods have a single shell, like the abalone in Figure 25.19. Single-shelled gastropods also include snails, conches, periwinkles, limpets, cowries, whelks, and cones. They can be found in aquatic habitats and in moist terrestrial habitats, and they can quickly draw their bodies into their shells for protection when threatened.

Slugs and nudibranchs do not have shells, but secrete a thick mucus that covers their bodies. To protect themselves, land slugs hide in dark locations under forest or garden litter. Nudibranchs incorporate into their own tissues the poisonous nematocysts of the jellyfishes they eat. The presence of nematocysts is advertised to predator fishes by the bright colors of the nudibranchs.

Bivalves One word—slow—best describes most behavior of the class Bivalvia—the two-shelled mollusks. Bivalves, such as clams, mussels, oysters, and the scallop shown in Figure 25.19, are all aquatic animals. Most are marine, but some are found in freshwater habitats. Bivalves might seem to be inactive even though they are continuously filter feeding and carrying on all bodily functions.

If you have ever been clamming or have seen people clamming, you know that you might have to dig deeply to find the clams because they use a muscular foot to burrow far down into wet sand. Mussels attach to rocks with a sticky, gluelike substance called byssal threads. Scallops are more active than other bivalves because they can clap their shells together to move more quickly through water.

Cephalopods

Quick is a word that best describes some behaviors of the class Cephalopoda. Cephalopods are the head-footed mollusks, which includes the squid, octopus, chambered nautilus, and the cuttle-fish in Figure 25.20. The chambered nautilus is the only cephalopod with an external shell. Squids and cuttlefishes have an internal shell, while octopuses do not have a shell. The foot of a cephalopod is divided into arms and tentacles with suckers, which are used to capture prey.

Protection

Although most cephalopods don’t have a hard external shell, they have evolved other protective mechanisms. Octopuses forcefully expel water to propel themselves away from threat. They hide in crevices or caves in the daytime. At night, they creep about in search of prey.

When threatened, an octopus shoots out an inky substance that forms a cloud. Scientists hypothesize that the ink visually confuses predators, and it also might act as a narcotic. Octopuses can change color to blend in with their surroundings. Squids and cuttlefishes also use ink and camouflage to escape predators. A chambered nautilus can pull into its shell for protection. It also uses its shell as camouflage. The dark top of the shell blends in with the ocean bottom when seen from above, while the white bottom of the shell blends in with the water above when seen from below.

Learning Octopuses are considered to be the most intelligent mollusks. They are capable of complex learning, such as being trained to select an object of a certain shape, color, or texture.

Ecology of Mollusks

Mollusks play important roles in aquatic and terrestrial food chains as herbivores, predators, scavengers, and filter feeders. In many areas, certain mollusks are considered keystone species. A keystone is the stone at the top of an arch that holds the arch together, so a keystone species is one whose health influences the health of the entire ecosystem. For example, the hard clam is a keystone species for the Great South Bay in Long Island, New York. These clams filter water, which cleans the ecosystem. If the hard clam population declines, the water isn’t filtered.

This disrupts the food web, causing algal blooms and a decline in water quality.

The ability of some mussels to accumulate toxins in their body tissues can be useful to scientists who are monitoring water quality. By examining these mollusks, scientists can find out more about water quality than they could by testing the water alone.

Cone snails, as shown in Figure 25.21, are highly prized by collectors for the beauty of their shells and, as a result, might be close to extinction.

Certain cone snails produce powerful venom to kill prey. These venoms are being studied as potential treatments for pain, heart disease, clinical depression, and brain diseases, such as

Alzheimer’s disease, Parkinson’s disease, and epilepsy.

Some mollusks cause damage, while others benefit humans. Some marine bivalve species, such as the shipworm, burrow into wood, causing much damage to wooden marinas and boats. On the other hand, people enjoy beautiful pearls that come from oysters. Pearls result when a grain of sand or a tiny parasite becomes trapped in an oyster. The mantle of the mollusk secretes a coating around the object to protect the mollusk, resulting in a pearl. Pearl producers implant pieces of shell or tiny plastic spheres in oysters and harvest cultured pearls in about five to seven years.

Segmented Worms

Body Structure

Earthworms are annelids and belong to phylum Annelida, which is characterized by animals with a body plan consisting of segments. As you can see on the evolutionary tree in Figure 25.22, both mollusks and annelids undergo protostome development and, therefore, are considered close relatives.

There are over 11,000 species of annelids, most of which live in the sea. Most of the remaining species are earthworms. Annelids live just about everywhere except in the frozen soil of the polar regions and in the sand of dry deserts.

Annelids include earthworms, marine worms, such as the ones shown in Figure 25.22, and parasitic leeches. All of these worms are different from flatworms and roundworms because they are segmented and have a coelom. Most annelids also have a **larval stage** that is similar to that of certain mollusks, suggesting a common ancestor. Annelids have bilateral symmetry like flatworms and roundworms and have two body openings like roundworms.

Even though annelids have the same cylindrical body shape as roundworms, annelid bodies are divided into segments. Externally, the segments look like a stack of thick coins or a stack of donuts. Inside the worm, the segments are divided almost completely from each other by walls of tissue, similar to the way walls separate the segments of a submarine. Each segment contains structures for digestion, excretion, and locomotion. The fluid within the coelom of each segment makes a rigid support system for the worm similar to the rigidity of a filled water balloon.

This rigidity in annelid segments creates a hydrostatic skeleton that muscles can push against. Segmentation also permits segments to move independently of each other and enables a worm to survive damage to a segment because other segments with the same functions exist.

Segments can be specialized, and groups of segments might be adapted to a particular function. For example, some segments might be adapted to sensing, while others are adapted to reproduction. As you continue to study annelids, earthworms will be used to show examples of typical annelid features.

Feeding and digestion

Running through all earthworm segments from the mouth to the anus is the digestive tract, a tube within a tube. Locate the digestive tract in the earthworm in Figure 25.23. Food and soil taken in by the mouth pass through the pharynx into the crop, where they are stored until they pass to the gizzard. The gizzard is a muscular sac containing hard particles that help grind soil and food before they pass into the intestine. Nutrients are absorbed from the intestine, then undigested material passes out of the worm’s body through the anus. Parasitic annelids have pouches along the digestive tract that hold enough food to last for months.

Circulation Unlike most mollusks, most annelids have a closed circulatory system. Oxygen and nutrients move to various parts of their bodies through their blood vessels. At the same time carbon dioxide and metabolic wastes are removed from the blood and excreted. Some of the vessels at the anterior end, or head, are large and muscular, as shown in Figure 25.24, and serve as hearts that pump the blood. The blood moves toward the anterior end of the worm in the dorsal blood vessel and toward the posterior end in the ventral blood vessel.

Respiration and excretion Earthworms take in oxygen and give off carbon dioxide through their moist skin. Some aquatic annelids have gills for the exchange of gases in the water. Segmented worms have two nephridia—similar to those in mollusks—in almost every segment. Cellular waste products are collected in the nephridia and are transported in tubes through the coelom and out of the body. Nephridia also function in maintaining homeostasis of the body fluids of annelids, ensuring that the volume and composition of body fluids are kept constant.

Response to stimuli

In most annelids, such as the earthworm, the anterior segments are modified for sensing the environment. The brain and nerve cords composed of ganglia are shown in the earthworm in

Figure 25.23. You might have seen an earthworm quickly withdraw into its burrow when you shine a flash-light on it or step close to it. These observations show that earthworms can detect both light and vibrations.

Movement When an earthworm moves, it contracts circular muscles running around each segment. This squeezes the segment and causes the fluid in the coelom to press outward like paste in a tube of toothpaste being squeezed. Because the fluid in the coelom is confined by the tissues between segments, the fluid pressure causes the segment to get longer and thinner. Next, the earth-worm contracts the longitudinal muscles that run the length of its body. This causes the segment to shorten and return to its original shape, pulling its posterior end forward and resulting in movement.

Many annelids have setae on each segment.

Setae, as shown in Figure 25.25, are tiny bristles that push into the soil and anchor the worm during movement. By anchoring some segments and retracting others, earthworms can move their bodies forward and backward segment by segment.

Reproduction Annelids can reproduce both sexually and asexually. Most annelids have separate sexes, but some, such as earthworms and leeches, are hermaphrodites. Sperm are passed between two worms near segments called the clitellum. Refer back to Figure 25.23 and notice that the clitellum is a thickened band of segments. It produces a cocoon from which young earthworms hatch, as shown in Figure 25.26. Sperm and eggs pass into the cocoon as it slips forward off the body of the worm. After fertilization, the young are protected in the cocoon as they develop. Some annelids reproduce asexually by fragmentation. If a worm breaks apart, the missing parts can be regenerated.

Diversity of Annelids

The phylum Annelida is divided into three classes: class Oligochaeta—the earthworms and their relatives, class Polychaeta—the bristleworms and their relatives, and class Hirudinea—the leeches.

Earthworms and their relatives Earthworms probably are the best-known annelids. They are used as bait for fishing and are found in garden soil. An earthworm can eat its own mass in soil every day. Earth-worms ingest soil to extract nutrients. In this way, earthworms aerate the soil—they break up the soil to allow air and water to move through it.

In addition to earthworms, class Oligochaeta—the oligochaetes—includes tubifex worms and lumbriculid worms.

Tubifex worms are small, threadlike aquatic annelids that are common in areas of high pollution. Lumbriculid worms are freshwater oligochaetes that are about 6 cm long and live at the edges of lakes and ponds.

Ecology of Annelids

Segmented worms play important roles in the ecology of ecosystems. Some are beneficial to plants and animals, while others benefit humans.

Earthworms Many different animals, including frogs and birds, eat earthworms as a part of their diets. Earthworms also mix leaf litter into soil, aerating it so that roots can grow easily and water can move through the soil efficiently. Both functions are important for a healthy ecosystem. However, nonnative earthworms are moving into areas, especially northern forests, where they are altering ecosystems in harmful ways. The disappearance of leaf litter on forest floors due to nonnative earthworms removes the shelter and moisture needed by many native plants and animals. Earthworms can be introduced into new habitats inadvertently by fishers and gardeners when the earthworms left from a day’s fishing are dumped on the ground, or when a nursery plant from another part of the country is planted in a home garden.

Polychaetes Marine polychaetes help convert the organic debris of the ocean floor into carbon dioxide. Marine plant plankton take in carbon dioxide and use it during photosynthesis. Marine polychaetes are an important part of the diet of many marine predators.

Leeches have been used as medical treatments for centuries. They were used to suck blood out of patients who were believed to be ill because of an excess of blood. Today, leeches are used after microsurgical procedures to prevent blood from accumulating in the surgical area. However, there are drawbacks to using leeches. They have a small feeding capacity and must be replaced often. They can cause bacterial infections, and many people cringe at the thought of having a leech attached to them. To solve these problems, a nonliving mechanical leech has been designed to do the work of the animal. Table 25.1 summarizes the ecological benefits of earthworms, polychaetes, and leeches.

Evolution of Mollusks and Annelids

In Figure 25.29, the cladogram on the left is one interpretation of the evolution of mollusks. As shown, gastropods have more shell coiling than cephalopods. In shell coiling, the shell grows in a circular manner, making it more compact and more stable than an uncoiled shell.

Cephalopods have a reduced shell. Bivalves are considered to have evolved later than gastropods and cephalopods because they lack a radula.

The cladogram on the right in Figure 25.29 is one interpretation of the evolution of annelids. In this interpretation, early segmented worms— the polychaestes—developed parapodia in the course of their evolution.

Later annelids—the oligochaetes and leeches—developed clitella. Leeches developed posterior body suckers even later.

Arthropod Characteristics

Arthropod Features

Copepods belong to phylum Arthropoda. Between 70 and 85 percent of all named animal species are arthropods. As shown in Figure 26.1, the majority of arthropods are insects, which includes beetles, butterflies, moths, flies, bees, and wasps.

Find arthropods on the evolutionary tree shown in Figure 26.1.

Follow the branches and you will see that, like annelids, arthropods are segmented invertebrates with bilateral symmetry, coelomate body cavities, and protostome development. Unlike annelids, arthropods have exoskeletons with jointed appendages that enable them to move in complex ways. All three of these features—segmentation, exoskeletons, and jointed appendages—are important keys to their success.

Segmentation Like annelids, arthropods are segmented, which allows for efficient and complex movements. Notice in Figure 26.2 that the praying mantid’s segments are fused into three main body regions—a head, a thorax, and an abdomen. The heads of arthropods have mouthparts for feeding and various types of eyes. Many have antennae. Antennae are long sensory structures that contain receptors for the senses of smell and touch. The thorax is the middle body region, consisting of three fused main segments to which, in many arthropods, the legs and wings are attached. The abdomen, which also contains fused segments and is at the posterior end of the arthropod, bears additional legs and contains digestive structures and the reproductive organs. Some arthropods, such as the crayfish in Figure 26.2, have the thorax region fused with the head into a structure called a cephalothorax.

In some groups of arthropods, segmentation is more obvious during early development. For example, a caterpillar has many obvious segments, while the adult butterfly has only three body segments.

Exoskeleton In addition to segmented bodies, arthropods have hard exoskeletons on the outside of their bodies, similar to a light-weight suit of armor. Recall from Chapter 24 that the exoskeleton provides a framework for support, protects soft body tissues, and slows water loss in animals that live on land. It also provides a place for muscle attachment.

Some segments in arthropods are fused. The praying mantid shows fusion of segments into its head, thorax, and abdomen. The crayfish shows a different fusion of segments into its cephalothorax and abdomen.

The exoskeleton of an arthropod is made of chitin. Recall from Chapter 20 that chitin is a nitrogen-containing polysaccharide bound with protein. While the exoskeleton of a grasshopper is leathery, the exoskeletons of some crustaceans, such as lobsters, incorporate calcium salts that harden them to such an extent that a hammer would be needed to crush them. An arthropod’s exoskeleton can be “hard as nails” in some places and thin and flexible in others, providing for movable joints between body segments and within appendages.

There is a limit to how hard and thick an exoskeleton can be. It is thin in small arthropods, such as the copepod, because it bears the pull of tiny muscles; it is thicker in larger arthropods, such as crabs and lobsters, to bear the pull of larger muscles. Imagine a fly as large as a bird. The fly’s exoskeleton would have to be so thick to bear the pull of the large muscles that the fly would not be able to move under the weight of the exoskeleton.

Body Structure of Arthropods

Arthropods have complex organ systems that enable them to live in many diverse habitats. Adaptations in several organ systems, such as the respiratory system and the nervous system, have contributed to the success of these animals.

Feeding and digestion

The great diversity of arthropods is reflected in their enormous variety of feeding habits and structures. The mouthparts of most arthropods include a pair of appendages called mandibles that can be adapted for biting and chewing, as shown in Figure 26.5. Depending on their feeding habits, other arthropods have mouthparts modified like feathery strainers, stabbing needles, cutting swords, or sucking straws. Arthropods can be herbivores, carnivores, filter feeders, omnivores, or parasites. To digest food, arthropods have a complete, one-way digestive system with a mouth, gut, and an anus, along with various glands that produce digestive enzymes.

Respiration Arthropods obtain oxygen by using one of three structures—gills, tracheal tubes, or book lungs. Recall from Chapter 25 that maintaining a certain homeostatic balance of oxygen in body tissues enables animals to have energy for a variety of functions. Most aquatic arthropods have gills, like those shown in Figure 26.6, that function in the same way as the gills in mollusks. All terrestrial arthropod body tissues need to be near airways to obtain oxygen.

Terrestrial arthropods depend on respiratory systems rather than circulatory systems to carry oxygen to cells. Most terrestrial arthropods have a system of branching tubes called tracheal tubes, shown in Figure 26.6, that branch into smaller and smaller tubules.

These tubules carry oxygen throughout the body.

Some arthropods, including spiders, have book lungs, saclike pockets with highly folded walls for respiration. In Figure 26.6, notice how the membranes in book lungs are like the pages in a book. The folded walls increase the surface area of the lungs and allow an efficient exchange of gases. You also can see how both tracheae and book lungs open to the outside of the body of the arthropod in openings called spiracles.

Circulation Even though most arthropods do not rely on their circulatory systems to deliver oxygen, they do rely on their circulatory systems to transport nutrients and remove wastes. Arthropod blood is pumped by a heart into vessels that carry the blood to body tissues. The tissues are flooded with blood, which returns to the heart through open body spaces. The blood maintains homeostasis in tissues by delivering nutrients and removing wastes.

Excretion In most arthropods, cellular wastes are removed from the blood through Malpighian tubules. These tubules also help terrestrial arthropods preserve water in their bodies to maintain homeostatic water balance. In insects, the tubules, as shown in Figure 26.7, are located in the abdomen, unlike in segmented worms, where nephridia exist in each segment. Malpighian tubules are attached to and empty into the gut, which contains the undigested food wastes to be eliminated from the body. Crustaceans and some other arthropods do not have Malpighian tubules. They have modified nephridia, similar to those in annelids, to remove cellular wastes.

Response to stimuli Most arthropods have a double chain of ganglia throughout their bodies, on the ventral surface. Fused pairs of ganglia in the head make up the brain. Although most behaviors, such as feeding and locomotion, are controlled by the ganglia in each segment, the brain can inhibit these actions.

Vision

Have you ever tried to swat a fly with a flyswatter? The fly’s accurate vision allows the fly to spot even the slightest movement, and the fly often escapes. Most arthropods have one pair of large compound eyes. A compound eye, as shown in Figure 26.8, has many facets, which are hexagonal in shape. Each facet sees part of an image. The brain combines the images into a mosaic. The compound eyes of flying arthropods, such as dragonflies, enable them to analyze a fast-changing landscape during flight. Compound eyes can detect the movements of prey, mates, or predators, and also can detect colors. In addition, many arthropods have three to eight simple eyes. A simple eye has one lens and functions by distinguishing light from dark. In locusts and some other flying insects, simple eyes act as horizon detectors that help stabilize flight.

Hearing In addition to having eyes that detect movement and distinguish light from dark, many arthropods also have another sense organ called a tympanum. A tympanum is a flat membrane used for hearing. It vibrates in response to sound waves. Arthropod tympanums can be located on the forelegs as in crickets, on the abdomen as in some grasshoppers, or on the thorax as in some moths.

Chemicals Imagine ants carrying off potato chip pieces, following each other like soldiers marching in formation. Ants communicate with each other by pheromones, chemicals secreted by many animal species that influence the behavior of other animals of the same species. The ants use their antennae to sense the odor of pheromones and to follow the scent trail. Arthropods give off a variety of pheromones that signal behaviors such as mating and feeding.

Movement

Think again about the ants carrying the potato chip pieces and how fast they were moving. Arthropods generally are quick, active animals. They are able to crawl, run, climb, dig, swim, and fly because of their well-developed muscular systems. Refer to Figure 26.9 to compare muscle attachment in human and arthropod limbs. The muscles in a human leg are attached to the outer surfaces of the bones.

The muscles in an arthropod limb are attached to the inner surface of the exoskeleton on both sides of the joint. The strength of muscle contraction in arthropods depends on the rate at which nerve impulses stimulate muscles. In contrast, in vertebrates, the strength of muscle contraction depends on the number of muscle fibers contracting.

Reproduction Most arthropods reproduce sexually and have a variety of adaptations for reproduction. Most arthropods have separate sexes, but a few, such as barnacles, are hermaphrodites and undergo cross-fertilization. Most crustaceans brood, or incubate, their eggs in some way, but they do not care for their hatched offspring. Some spiders and insects also incubate their eggs, and some, such as bees, care for their young.

Arthropod Diversity

Arthropod Groups

Spiders, pill bugs, and ants are arthropods. In the previous section, you learned why they all are considered arthropods. In the next two sections, you will learn how they differ from one another. Arthropods are classified into groups based on shared similarities, such as the structure of their body segments, appendages, and mouthparts. Taxonomists continue to debate the classification of arthropods. In this section, you will learn about two of the major groups—the crustaceans, such as crabs and lobsters, and the arachnids, such as spiders and their relatives. In the next section, you will learn about the third major group—the insects and their relatives. Table 26.1 summarizes the common characteristics of the three main groups of arthropods.

Crustaceans

Crabs, shrimps, lobsters, crayfishes, barnacles, water fleas, and pill bugs are crustaceans, and they live in marine, freshwater, and terrestrial habitats. Class Crustacea consists of about 35,000 named species. Most are aquatic and have two pairs of antennae, two compound eyes that often are on the tips of slender movable stalks, and mandibles for chewing.

Crustacean mandibles open and close from side to side, instead of in an up-and-down movement like human jaws. Crustaceans possess branched appendages and have a free-swimming larval stage called a nauplius larva. A larva is an immature form of an animal that is markedly different in form and appearance from the adult.

Most crustaceans, such as crayfishes, lobsters, and crabs, have five pairs of legs. The first pair of legs—the chelipeds, shown in Figure 26.10—has large claws adapted to catch and crush food. Behind the next four pairs of walking legs are the swimmerets, appendages that are used for reproduction and as flippers during swimming. If you have ever seen a lobster swim, you might have been surprised at how it can snap its tail beneath its body and move backward quickly. Some crustaceans, such as barnacles, are sessile, and use their legs to kick food into their mouths.

Unlike their aquatic relatives, sow bugs and pill bugs are terrestrial crustaceans that live in damp places, such as under logs. They have seven pairs of legs.

Spiders and Their Relatives

Spiders belong to class Arachnida in which there are about 57,000 named species. Arachnids include spiders, ticks, mites, and scorpions.

Most arachnids have two body sections—a cephalothorax and an abdomen—and six pairs of jointed appendages. They do not have antennae. An arachnid’s most anterior pair of appendages is modified into mouthparts called chelicerae. Chelicerae are adapted to function as fangs or pincers and often are connected to a poison gland. Most spiders in the United States are not poisonous to humans. Exceptions include the black widow and the brown recluse shown in Figure 26.11.

In arachnids, the second pair of appendages is called the pedipalps. These appendages are used for sensing and holding prey. The pedipalps also are used for reproduction in male spiders and as large pincers in scorpions. The remaining four pairs of appendages in arachnids are used for locomotion.

Spiders

All spiders are carnivores. Some spiders, such as wolf spiders and tarantulas, hunt prey. Other spiders catch prey in silken webs. Silk is made from a fluid protein secreted by glands and spun into silk by structures called spinnerets, located at the end of a spider’s abdomen.

Have you ever watched a spider weave a web? If you have, you might have wondered how the spider seemed to know just what to do and where it managed to get the training to do such intricate work.

Spiders are capable of constructing only specific kinds of webs. This instinctive behavior enables them to do this efficiently and effectively time after time. Figure 26.12 shows the stages of construction of an orb web.

After catching an insect or other arthropod in their webs, many spiders wrap their prey in a silken cocoon until they are ready to feed.

Digestion begins externally, when a spider secretes digestive enzymes onto its prey. After liquification occurs, the spider ingests the softened food. The remaining nutrients are digested internally.

To reproduce, a male spider deposits sperm on a small web he has built, picks up the sperm, and stores it in a cavity on his pedipalps. After a courtship ritual, the male inserts the sperm into the female. The female lays her eggs in a cocoon spun of spider silk. There can be as many as 100 eggs in one cocoon. The young hatch after about two weeks, then molt between five and ten times before reaching their adult size.

Ticks, mites, and scorpions

Other members of class Arachnida—ticks, mites, and scorpions—are shown in Figure 26.13. Most mites are less than 1 mm long, with the cephalothorax and abdomen fused into one oval-shaped body section. They can be predators or parasites of other animals. Ticks are parasites that feed on blood after attaching themselves to the surface of their hosts. Ticks also harbor disease-causing agents, such as viruses, bacteria, and protozoa, and introduce them to their hosts when they bite. Some of these diseases, such as Lyme disease and Rocky Mountain spotted fever, affect humans.

Scorpions feed on insects, spiders, and small vertebrates that they capture with their pedipalps and tear apart with their chelicerae. They generally are nocturnal, hiding under logs or in burrows during the day. When you think of a scorpion, you might think of the stinger at the end of the abdomen. Most scorpions that live in the United States are not considered to be dangerous, but their sting can be quite painful.

Horseshoe crabs Horseshoe crabs are an ancient group of marine animals, related to the arachnids, that have remained basically unchanged since the **Triassic Period** more than 200 million years ago. They have unsegmented heavy exoskeletons in the shape of a horse-shoe. The chelicerae, pedipalps, and the next three pairs of legs are used for walking and getting food from the bottom of the sea. The animals feed on annelids, mollusks, and other invertebrates, which they capture with their chelicerae. The posterior appendages are modified with leaf-like plates at their tips and can be used for digging or swimming.

Horseshoe crabs, shown in Figure 26.14, come to shore to reproduce at high tide. The female burrows into the sand to lay her eggs. A male follows behind and adds sperm before the female covers the eggs with sand. Young larvae hatch after a period of being warmed by the Sun and then return to the ocean during another high tide.

Insects and Their Relatives

Diversity of Insects

Scientists estimate that there are as many as 30 million insect species, which is more species than all other animals combined. Recall that arthropods make up about three-fourths of all named animal species. About 80 percent of arthropods are insects. They are the most abundant and widespread of all terrestrial animals. You can find insects in soil, in forests and deserts, on mountaintops, and even in polar regions.

Insects live in many habitats because of their ability to fly and their ability to adapt. Their small size enables them to be moved easily by wind or water. Diversity of insects also is enhanced by the hard exoskeleton that protects them and keeps them from drying out in deserts and other dry areas. In addition, the reproductive capacity of insects ensures that they are successful in any areas they inhabit. Insects produce a large number of eggs, most of the eggs hatch, and the offspring have short life cycles, all of which can lead to huge insect populations.

External Features

Insects have three body areas—the head, thorax, and abdomen, shown in Figure 26.15. Head structures include antennae, compound eyes, simple eyes, and mouthparts. Insects have three pairs of legs and generally two pairs of wings on the thorax. Some only have one pair of wings, and others do not have wings at all.

Insect Adaptations

Structural adaptations to legs, mouthparts, wings, and sense organs have led to increased diversity in insects. These adaptations enable insects to utilize all kinds of food and to live in many different types of environments. Taking advantage of a variety of food sources, insects might be parasites, predators, or plant-sap suckers.

Legs Insect legs are adapted to a variety of functions. Beetles have walking legs with claws that enable them to dig in soil or crawl under bark. Flies have walking legs with sticky pads on the ends that enable them to walk upside down. Honeybee legs have adaptations for collecting pollen, while the hind legs of grasshoppers and crickets are adapted to jumping. Water striders have legs adapted to skimming over the surface of water. On its footpads, a water strider has water-repellent hairs that do not break the surface tension of the water. As it skates over the water, this insect propels itself with its back legs and steers with its front legs, like a rear-wheel-drive car.

Mouthparts Insects’ mouthparts are adapted to the food they eat, as shown in Table 26.2. Butterflies and moths have a long tube through which they draw nectar from flowers in a motion similar to sipping through a straw. Different types of flies, such as houseflies and fruit flies, have sponging and lapping mouthparts that take up liquids. Some insects, such as leafhoppers and mosquitoes, have piercing mouthparts for feeding on plant juices or prey. Insects such as beetles and ants cut animal skin or plant tissue with their mandibles to reach the nutrients inside.

Wings Insects are the only invertebrates that can fly. Unlike bird and mammal wings that are modified limbs, insect wings are outgrowths of the body wall. Wings are formed of a thin double membrane of chitin, which is the same material that makes up the exoskeleton, and they have rigid veins that give them strength. Wings can be thin, as in flies, or thick, as in beetles. The wings of butterflies and moths are covered with fine scales, as shown in Figure 26.16. Investigate how butterflies might use their wing scales to attract mates in Data Analysis Lab 26.1. Flying requires complex movements of the wings. Forward thrust, upward lift, balance, and steering are all important. Most insects rotate their wings in a figure-eight pattern, as shown in Figure 26.16.

Sense organs Along with leg, mouthpart, and wing adaptations, insects have a variety of adaptations in their sense organs. Recall how arthropods use their antennae and eyes to sense their environment. Insects also have hairlike structures that are sensitive to touch, pressure, vibration, and odor. In addition to visually detecting motion, a fly detects changes in airflow using the hundreds of hairs that cover its body. It’s no wonder that a fly often is long gone before the flyswatter can strike.

Some insects detect airborne sounds with their tympanic organs, while others can detect vibrations coming from the ground. These sensory cells often are located on the legs.

Most insects have keen chemical senses. Chemical receptors, or chemoreceptors, for taste and smell are located on mouthparts, antennae, or legs. Some insects, such as moths, can detect odors several kilometers away. Chemical signals in the form of pheromones enable insects to communicate with one another to attract mates or to gather members in large colonies to migrate or survive periods of cold weather.

Metamorphosis Insects that do not care for their young lay many more eggs than insects that do care for their young. Most insects lay their eggs in a specific habitat where the young can survive. For example, a monarch butterfly lays its eggs on milkweed plants, which the young feed on after they hatch. After hatching, most insects undergo metamorphosis, a series of major changes from a larval form to an adult form.

Complete metamorphosis Most insects develop through the four stages of complete metamorphosis—egg, larva, pupa, and adult. As shown in Figure 26.17, when the egg of a butterfly hatches, the worm-like larva that appears commonly is called a caterpillar. At this stage, the larva usually has chewing mouthparts and behaves like a feeding machine. The larva molts several times as it grows. A pupa is a nonfeeding stage of metamorphosis in which the animal changes from the larval form into the adult form. The adult stage of metamorphosis generally is specialized for dispersal and reproduction. If adults feed, they generally do not use the same food source as the larvae, which eliminates competition for the same food and increases chances for survival if food is scarce.

Incomplete metamorphosis Insects that undergo incomplete metamorphosis, as shown in Figure 26.17, hatch from eggs as nymphs—the immature form of insects that look like small adults without fully developed wings. After several molts, young nymphs become winged adults.

Insect societies

The players on a basketball team work together to win the game. Insects such as honeybees, ants, and termites organize into social groups and cooperate in activities necessary for their survival.

Honeybees have a complex society, with as many as 70,000 bees in one hive. There are three castes in a hive. A caste is a group of individuals within a society that perform specific tasks. Workers are females that do not reproduce. They gather nectar and pollen, build the honeycomb, manufacture honey, care for young, and guard the hive. Drones are the reproductive males. The queen is the only reproductive female.

Communication methods Honeybees have evolved an efficient system of communication, using bodily movements to indicate the location of food sources. One of the movements by which honeybees communicate is called the waggle dance. This dance is performed when a bee returns to the hive from a faraway food source. First, the returning bee makes a circle with a diameter about three times the bee’s length. The bee then moves in a straight line while waggling its abdomen from side to side. The orientation of the line indicates the direction to the food source. Finally, the bee makes another circle in the opposite direction from the first circle. It traces this figure-eight pattern many times. The duration of the dance indicates the distance to the food source.

The most significant part of the waggle dance is the straight line because it tells the other bees where the food is in relation to the hive. The direction of the line relative to the vertical indicates the direction of the food relative to the Sun, as shown in Figure 26.18. If food is located 70 degrees to the right of the Sun, the straight line of the dance will be 70 degrees to the right of vertical.

Round dances also convey information about food sources and are used only if the food is close to the hive. In a round dance, the bee traces a clockwise circle followed by a counterclockwise circle and repeats this dance many times. The dance does not indicate distance or direction.

Ants also have evolved various societal behaviors for living in colonies. Females that do not reproduce gather food, care for young, and protect the colony from predators. Like honeybees, the males die after mating with the queen, whose sole function is to lay eggs.

Insects and humans It might be difficult to think of insects as beneficial when a mosquito buzzes around your head or when a bee stings you, but insects are an integral part of all ecosystems on Earth. Most insect species are not harmful to humans. Insects pollinate most flowering plants, including almost ten billion dollars’ worth of food crops in the United States. They produce honey and silk used by humans and serve as food for many birds, fishes, and other animals. Insect predators, such as praying mantids and ladybird beetles, feed on plant pests such as aphids and mites, as shown in Figure 26.19.

Insects also can be harmful to humans. Lice and bloodsucking flies are human parasites. Fleas can carry plague, houseflies can carry typhoid fever, and mosquitoes can carry malaria, yellow fever, and filariasis. Weevils, cockroaches, ants, and termites cause much property destruction.

Grasshoppers, corn borers, and boll weevils destroy agricultural crops. Bark beetles, spruce budworms, and gypsy moths can destroy whole portions of forests.

How is all this insect damage kept in check? In the past, chemicals were used indiscriminately to control insects. However, the overuse of chemicals disrupted food chains, reduced numbers of beneficial insects, and insects developed resistance to the insecticides. Use of biological controls has become increasingly important. Integrated pest management, a technique used by many farmers today, offers long-term control of pests. This strategy employs resistant plant varieties, crop rotation, and critical timing of planting and other agricultural practices along with small amounts of chemicals at critical times to control insect pests.

Centipedes and Millipedes

The centipedes of class Chilopoda and the millipedes of class Diplopoda are close relatives of insects. Centipedes move quickly and live in moist places under logs, bark, and stones. They have long, segmented bodies, and each segment has one pair of jointed legs. The first pair of appendages is modified to form poison claws, which a centipede uses to kill prey. Most species of centipedes are not harmful to humans.

Millipedes have two pairs of appendages on their abdominal segments and one pair on their thorax segments. Millipedes are herbivorous and live, as centipedes do, in moist places under logs or stones. Unlike centipedes, they do not wriggle quickly, but walk with a slow, graceful motion. Millipedes do not have poison claws and feed primarily on damp and decaying vegetation. Compare the centipede and millipede in Figure 26.20.

Evolution of Arthropods

The relationships of tardigrades, trilobites, and arthropods have been under close scrutiny as new evidence is discovered. Fossil records show that trilobites, abundant in the mid-Cambrian but now extinct, were early arthropods. Trilobites, like the one shown in Figure 26.21, were oval, flattened, and divided into three body sections like some modern arthropods. The large number of identical segments of these ancestral arthropods evolved to more specialized appendages and fewer segments in modern arthropods.

Tardigrades also are related to arthropods, but they appear to be related less closely to arthropods than trilobites are. The tardigrade shown in Figure 26.21 illustrates why these tiny animals are known commonly as water bears. The largest are 1.5 mm long with four pairs of stubby legs.

They feed on algae, decaying matter, nematodes, and other soil animals. They inhabit freshwater, marine, and land habitats. During temperature extremes and drought, tardigrades can survive for years in a completely dry state with reduced metabolism until favorable conditions return.

Echinoderm Characteristics

Echinoderms Are Deuterostomes

As shown in the evolutionary tree in Figure 27.1, echinoderms are deuterostomes—a major transition in the phylogeny of animals. Notice how the evolutionary tree branches at deuterostome development.

The mollusks, annelids, and arthropods you studied in previous chapters are protostomes. Recall that during development, a protostome’s mouth develops from the opening on the gastrula, while a deuterostome’s mouth develops from elsewhere on the gastrula. This might not seem important, but consider that only echinoderms and the chordates that evolved after echinoderms have this kind of development.

Echinoderms and chordates are related more closely than groups that do not develop in this way. You are related more closely to the sea star in the opening photo than you are to a beetle or a clam.

The approximately 6000 living species of echinoderms are marine animals and include sea stars, sea urchins and sand dollars, sea cucumbers, brittle stars, sea lilies and feather stars, and sea daisies. Two echinoderms are shown in Figure 27.1.

Body Structure

The brittle star is an example of an echinoderm with the spiny endoskeleton that is characteristic of the organisms in this phylum. Echinoderms are the first group of animals in evolutionary history to have endoskeletons. In echinoderms, the endoskeleton consists of calcium carbonate plates, often with spines attached, and is covered by a thin layer of skin. On the skin are pedicellariae, small pincers that aid in catching food and in removing foreign materials from the skin.

All echinoderms have radial symmetry as adults. In Figure 27.2, you can see this feature in the five arms of the brittle star radiating out from a central disk. However, echinoderm larvae have bilateral symmetry, as shown in Figure 27.2. In the next chapter, you will learn how bilateral symmetry shows an embryonic link to the vertebrate animals that evolved later.

No other animals with the complex organ systems of echinoderms have radial symmetry. Scientists theorize that the ancestors of echinoderms did not have radial symmetry. Primitive echinoderms might have been sessile, and radial symmetry developed, to enable them to carry on a successful stationary existence. Free-moving echinoderms might have evolved from the sessile animals.

Water-vascular system

Another feature of echinoderms is their water-vascular system—a system of fluid-filled, closed tubes that work together to enable echinoderms to move and get food. The strainerlike opening to the water-vascular system, shown in Figure 27.3, is called the madreporite. Water is drawn into the madreporite, then moves through the stone canal to the ring canal. From there, the water moves to the radial canals and eventually to the tube feet.

Tube feet are small, muscular, fluid-filled tubes that end in suction-cup-like structures and are used in movement, food collection, and respiration. The opposite end of the tube foot is a muscular sac, called the ampulla. When muscles contract in the ampulla, water is forced into the tube foot and it extends. Imagine holding a small, partly inflated balloon in your hand and squeezing it. The balloon will extend from between your thumb and forefinger, which is similar to the way the tube foot extends. The suction-cup-like structure on the end of the tube foot attaches it to the surface. This hydraulic suction enables all echinoderms to move and some, such as sea stars, to apply a force strong enough to open the shells of mollusks, as illustrated in Figure 27.4.

Feeding and digestion Echinoderms use a great variety of feeding strategies in addition to tube feet. Sea lilies and feather stars extend their arms and trap food. Sea stars prey on a variety of mollusks, coral, and other invertebrates. Many species of sea stars can push their stomachs out of their mouths and onto their prey. They then spread digestive enzymes over the food and use cilia to bring the digested material to their mouths. Brittle stars can be active predators or scavengers, and they can trap organic materials in mucus on their arms. Most sea urchins use teethlike plates, shown in Figure 27.3, to scrape algae off surfaces or feed on other animals. Many sea cucumbers extend their branched, mucus-covered tentacles to trap floating food.

Respiration, circulation, and excretion Echinoderms also use their tube feet in respiration. Oxygen diffuses from the water through the thin membranes of the tube feet. Some echinoderms carry out diffusion of oxygen through all thin body membranes in contact with water. Others have thin-walled skin gills that are small pouches extending from the body. Many sea cucumbers have branched tubes, called respiratory trees, through which water passes and oxygen moves into the body.

Circulation takes place in the body coelom and the water-vascular system, while excretion of cellular wastes occurs by diffusion through thin body membranes. Cilia move water and body fluids throughout these systems aided by pumping action in some echinoderms. In spite of the simplicity of these organs and systems, echinoderms maintain homeostasis effectively with adaptations that are suited to their way of life.

Response to stimuli Echinoderms have both sensory and motor neurons with varying degrees of complexity in different species. In general, a nerve ring surrounds the mouth with branching nerve cords connecting to other body areas.

Sensory neurons respond to touch, chemicals dissolved in the water, water currents, and light. At the tips of the arms of sea stars are eyespots, clusters of light-sensitive cells, illustrated in Figure 27.5. Many echinoderms also sense the direction of gravity. For example, a sea star will return to an upright position after being overturned by a wave or current.

Movement Echinoderm locomotion is as varied as echinoderm body shapes. The structure of the endoskeleton is important for determining the type of movement an echinoderm can undertake. The movable bony plates in the endoskeletons of echinoderms enable them to move easily. Feather stars move by grasping the soft sediments of the ocean bottom with their cirri—long, thin appendages on their ventral sides—or by swimming with up-and-down movements of their arms. Brittle stars use their tube feet and their arms in snakelike movements for locomotion.

Sea stars use their arms and tube feet for crawling. Sea urchins move by using tube feet and burrowing with their movable spines. Sea cucumbers crawl using their tube feet and body wall muscles.

Reproduction and development

Most echinoderms reproduce sexually. The females shed eggs and the males shed sperm into the water where fertilization takes place. The fertilized eggs develop into free-swimming larvae with bilateral symmetry. After going through a series of changes, the larvae develop into adults with radial symmetry.

Recall that echinoderms have deuterostome development, making them an important evolutionary connection to vertebrates.

The sea star in Figure 27.6 illustrates an echinoderm regenerating a lost body part. Many echinoderms can drop off an arm when they are attacked, enabling them to flee while the predator is distracted. Others can expel part of their internal organ systems when threatened, an action that might surprise and deter predators. All of these body parts can be regenerated.

Echinoderm Diversity

The major classes of living echinoderms include Asteroidea, the sea stars; Ophiuroidea, the brittle stars; Echinoidea, the sea urchins and sand dollars; Crinoidea, the sea lilies and feather stars; Holothuroidea, the sea cucumbers; and Concentricycloidea, the sea daisies. Recall that they all are marine animals with radial symmetry as adults, a water-vascular system with tube feet, endoskeletons often bearing spines, and larvae with bilateral symmetry. The classes of echinoderms are summarized in Table 27.1.

Sea stars

If you ever have seen an echinoderm, it probably was a sea star. Most species of sea stars have five arms arranged around a central disk. Some, such as the one in Figure 27.7, have more than five arms. Sea stars can be found in shallow water near the shore and in tide pools when the tide recedes. They can be found in groups clinging to rocks by means of their tube feet. A single tube foot can exert a pull of 0.25–0.30 N. This is equal to the force required to lift 25–30 large paper clips. Because a sea star might have as many as 2000 tube feet, it can exert quite a large force as it crawls or opens mollusks for food. Sea stars are important predators in marine eco-systems, feeding on clams and other bivalves. Because of their spiny skin, sea stars usually are not food for other marine predators.

Brittle stars

Like sea stars, most brittle stars have five arms, but the brittle star’s arms are thin and very flexible, as shown in Figure 27.8. They do not have suckers on their tube feet, so they cannot use them for movement as sea stars do. Brittle stars move by rowing themselves quickly over the bottom rocks and sediments or by snakelike movements of their arms. When attacked by a predator, a brittle star can release an arm and make a quick getaway. The missing arm will be regenerated later. Brittle stars hide in the crevices of rocks by day and feed at night. They feed on small particles suspended in the water or catch suspended materials on mucous strands between their spines.

Some brittle stars respond to light. The spherical structures covering the body of these brittle stars might function as light-gathering lenses. Brittle stars are more abundant and have more numbers of species than any other class of echinoderm.

Reading Check Compare and contrast the locomotion of sea stars and brittle stars.

Sea urchins and sand dollars Burrowing is a key characteristic of sea urchins and sand dollars. Sand dollars can be found in shallow water burrowing into the sand, while sea urchins burrow into rocky areas. These echinoderms each have a compact body enclosed in a hard endoskeleton, called a test, that looks like a shell. The tube feet extend through pores in the test. Closely fitting plates of calcium carbonate make up the test. Sea urchins and sand dollars lack arms, but their tests reflect the five-part pattern of arms in sea stars and brittle stars. Spines also are an important feature of this class, as seen in Figure 27.9. Some sea urchin spines and pedicellariae contain venom and are used for fending off predators. The poison in pedicellariae can paralyze prey.

Sea urchins also can be herbivorous grazers, scraping algae from rocks, while sand dollars filter organic particles from the sand in which they are partially buried.

Most sea urchins have a chewing apparatus inside their mouths consisting of five hard plates, similar to teeth. This structure, shown in Figure 27.10, is called Aristotle’s lantern. It was named after a description written by Aristotle, a Greek philosopher, in his book Historia Animalium. In the fourth century B.C., which is when Aristotle lived, people used five-sided lanterns with side panels made of thin, translucent horn, called horn lanterns. Aristotle thought the mouth of a sea urchin looked like a horn lantern without the panels.

Sea lilies and feather stars Fossil records show that sea lilies and feather stars are the most ancient of the echinoderms and were abundant before other echinoderms evolved. They are different from other echinoderms in that they are sessile for part of their lives. As shown in Figure 27.11, sea lilies have a flower-shaped body at the top of a long stalk, while the long-branched arms of feather stars radiate upward from a central area. Though they might stay in one place for a long time, sea lilies and feather stars can detach themselves and move elsewhere. Both sea lilies and feather stars capture food by extending their tube feet and arms into the water, where they catch suspended organic materials.

Sea cucumbers

Sea cucumbers don’t look like other echinoderms. Some might say they don’t even look like animals. Can you guess why they are called sea cucumbers? They look like cartoon cucumbers creeping over the ocean floor. Their elongated bodies move sluggishly by means of tube feet assisted by contractions in their muscular body wall. Their calcium carbonate plates are reduced in size and do not connect as they do in other echinoderms, so their outer bodies generally appear leathery.

Some of their tube feet are modified to form tentacles which extend from around their mouths to trap suspended food particles, as shown in Figure 27.12. The tentacles are covered with mucus, which increases their ability to trap food. Once food has been trapped on a tentacle, it is drawn into the mouth where the food is sucked off. This process is similar to licking your finger after putting it in a bowl of pudding.

Sea cucumbers are the only echinoderms to have respiratory organs in the form of respiratory trees. These many-branched tubes pump in seawater through the anus for oxygen extraction. The respiratory tree also functions in excretion by removing cellular wastes.

When a sea cucumber is threatened, it can cast out some of its internal organs through its anus. A potential predator might be confused by this action and move on. The sea cucumber can regenerate its lost parts. Even though the sea cucumber’s adaptations might seem odd, it is important to remember that this animal maintains homeostasis with adaptations that fit its way of life in its particular habitat.

Sea daisies Discovered in 1986 off the coast of New Zealand, sea daisies have been difficult to classify and study, because so few have been found. They are less than 1 cm in diameter and are disc-shaped with no arms. Their tube feet are located around the edge of the disc. Figure 27.13 shows that they have five-part radial symmetry, as do other echinoderms. Notice the daisy pattern of petals, or tube feet, around the edges of the disc.

Ecology of Echinoderms

Sea cucumbers and sea urchins are sources of food for people in some Asian countries. The muscles of certain sea cucumbers are eaten as sushi, and dried sea cucumbers are added to flavor soups, vegetables, and meat. The egg masses of sea urchins are eaten raw or slightly cooked.

Commensal relationships exist between some echinoderms and other marine animals. Recall from Chapter 2 that commensalism is a relationship in which one organism benefits and the other organism is neither helped nor harmed. For example, some species of brittle stars live inside sponges. The brittle star leaves the protective interior of the sponge and feeds on materials that have settled on the sponge.

Echinoderm benefits Marine ecosystems also depend on some echinoderms. When populations of echinoderms decline, a change in the ecosystem often is noted. For example, a sea urchin species that lives in the Caribbean and the Florida keys declined in numbers by more than 95 percent in 1983 due to disease. After this, algae increased greatly on the coral reefs and virtually destroyed the reefs in many areas. Sea urchins and sea cucumbers are bioturbinators, organisms that stir up sediment on the ocean floor. This action is important to the entire marine ecosystem, as it makes nutrients in the seafloor available to other organisms.

Echinoderm harm

Some echinoderms can harm marine ecosystems. The crown-of-thorns sea star shown in the photo at the beginning of this chapter feeds on coral polyps. When these sea stars increase in numbers, coral reefs are destroyed. Although the causes of the population explosions of these sea stars continue to be debated, the numbers seem to decline on their own. At a later time, they might increase again with no apparent explanation. Sea urchins are a favorite food of sea otters, as shown in Figure 27.14. The number of sea otters in California has declined in recent years, leading to an increase in the number of sea urchins. The sea urchins are eating the kelp forests, destroying the habitat of fish, snails, and crabs.

Invertebrate Chordates

Invertebrate Chordate Features

Look at Figure 27.15, the evolutionary tree of animal phylogeny. Notice that invertebrate chordates, such as lancelets and tunicates, are deuterostomes like echinoderms, but as you will learn, they have additional chordate features that echinoderms do not have.

If you were to guess the most celebrated and famous animal among zoologists—scientists who study animals—you might not guess that it is one of the animals in Figure 27.15. This small, eel-like animal is an amphioxus, also called a lancelet, and spends most of its life buried in sand. If you go to a beach and carefully dig in the sand at the shoreline, it would be unlikely for you to find an amphioxus. They are inconspicuous creatures with translucent, fish-shaped bodies about 5 cm long. With its headless body half-burrowed in the sand, the amphioxus quietly filter feeds, not at all aware of its fame. Fossil evidence and recent molecular data show that the amphioxus is one of the closest living relatives of vertebrates. You are related more closely to this fishlike creature than you are to any other invertebrate—yet related very distantly compared to other vertebrates.

Chordates are animals belonging to the phylum Chordata that have four distinctive features—a dorsal tubular nerve cord, a notochord, pharyngeal pouches, and a postanal tail—at some point during their development. Recent evidence suggests that all chordates also might have some form of a thyroid gland. In addition, they have a coelom and segmentation. Study Figure 27.16 to see the main features of chordates. Recall that vertebrates are animals with backbones. Most chordates are vertebrates. Invertebrate chordates, which belong to two of the subphyla of chordates—Cephalochordata and Urochordata, also have a dorsal tubular nerve cord, a notochord, pharyngeal pouches, a postanal tail, and, possibly, an ancestral thyroid gland. They have no backbone, however.

Notochord The notochord is a flexible, rodlike structure that extends the length of the body. It is located just below the dorsal tubular nerve cord. In most vertebrates, the notochord eventually is replaced by bone or cartilage. In invertebrate chordates, the notochord remains. The flexibility of the notochord enables the body to bend, rather than shorten, during contractions of the muscle segments. An animal with a notochord can make side-to-side movements of the body and tail, the first time in the course of evolution that fishlike swimming is made possible.

Postanal tail

A free-swimming animal moves efficiently by using a postanal tail. A postanal tail is a structure used primarily for locomotion and is located behind the digestive system and anus. In most chordates, the postanal tail extends beyond the anus. Tails in nonchordates have parts of the digestive system inside and the anus is located at the end of the tail. The postanal tail with its muscle segments can propel an animal with more powerful movements than the body structure of invertebrates without a postanal tail.

Dorsal tubular nerve cord

In nonchordates, the nerve cords are ventral to, or below, the digestive system and are solid. Chordates have a dorsal tubular nerve cord that is located dorsal to, or above, the digestive organs and is a tube shape. The anterior end of this cord becomes the brain and the posterior end becomes the spinal cord during development of most chordates.

Pharyngeal pouches

In all embryos, paired structures called pharyngeal pouches connect the muscular tube that links the mouth cavity and the esophagus. In aquatic chordates, the pouches contain slits that lead to the outside. These structures were used first for filter feeding and later evolved into gills for gas exchange in water. In terrestrial chordates, the pharyngeal pouches do not contain slits and develop into other structures, such as the tonsils and the thymus gland. Pharyngeal pouches are thought to be evidence of the aquatic ancestry of all vertebrates.

Ancestral thyroid gland

The thyroid gland is a structure that regulates metabolism, growth, and development. An early form of a thyroid gland had its origins in cells of early chordates that secreted mucus as an aid in filter feeding. Invertebrate chordates have an endostyle—cells in this same area that secrete proteins similar to those secreted by the thyroid gland.

Only vertebrate chordates have a thyroid gland.

Iodine is concentrated in the endostyle and plays an important role in thyroid gland function. It is essential for the production of thyroid hormones. In the United States, iodine is added to salt to prevent iodine deficiency. Other sources of iodine include fish, dairy products, and vegetables grown in iodine-rich soil.

Diversity of Invertebrate Chordates

Like echinoderms, all invertebrate chordates are marine animals. There are about 23 species of lancelets belonging to subphylum Cephalochordata. Tunicates, in subphylum Urochordata, consist of about 1250 species.

Lancelets

Recall the amphioxus at the beginning of this section. Most lancelets belong to the genus

Branchiostoma (formerly Amphioxus). They are small, fishlike animals without scales. As shown in Figure 27.17, lancelets burrow their bodies into the sand in shallow seas.

Lancelets lack color in their skin, which is only one cell layer deep, enabling an observer to view some body functions and structures. Water flowing through the body can be observed as a lancelet filter feeds. To get food, water enters the mouth of the lancelet and passes through pharyngeal gill slits. Food is trapped and passed on to a stomachlike structure to be digested. Water exits through the gill slits.

Just as filter feeding can be observed in the amphioxus, its muscles also can be seen. Observe the internal structures of a lancelet in Figure 27.17. The arrangement of segmented muscle blocks is similar to that in vertebrates, enabling lancelets to swim with a fishlike motion. Unlike vertebrates, they have no heads or sensory structures other than light receptors and small sensory tentacles near the mouth. The nervous system consists of main branching nerves and a simple brain at the anterior end of the animal. Blood passes through the body by the action of pumping blood vessels, as there is no true heart. Lancelets have separate sexes, and fertilization is external.

Tunicates

Often called sea squirts, tunicates are named for the thick outer covering, called a tunic, that covers their small, saclike bodies. Most tunicates live in shallow water; some live in masses on the ocean floor. In general, tunicates are sessile, and only in the larval stages do they show typical chordate features. Locate the notochord, postanal tail, dorsal tubular nerve cord, pharyngeal pouches, and ancestral thyroid gland on the tunicate larva in Figure 27.18.

Water is drawn into the saclike body of an adult tunicate through the incurrent siphon, as shown in Figure 27.19, by the action of beating cilia. Food particles are trapped in a mucous net and moved into the stomach where digestion takes place. In the meantime, water leaves the body, first through gill slits in the pharynx and then out through the excurrent siphon.

Circulation in the body of the tunicate is performed by a heart and blood vessels that deliver nutrients and oxygen to body organs. The nervous system consists of a main nerve complex and branching neurons. Tunicates are hermaphrodites—they produce both eggs and sperm—with external fertilization.

Why are tunicates called sea squirts? When they are threatened, they can eject a stream of water with force through the excurrent siphon, possibly distracting a potential predator.

Evolution of Echinoderms and Invertebrate Chordates

Biologists are studying fossil and molecular evidence to learn how echinoderms and invertebrate chordates are related to the vertebrates that evolved later.

Phylogeny of echinoderms The fossil record of echinoderms extends back to the Cambrian. Scientists think that they evolved from ancestors with bilateral symmetry because echinoderms have bilaterally symmetrical larvae. Their radial symmetry develops later in the adult stage. Many biologists think that ancient echinoderms were sessile and attached to the ocean floor by a long stalk, just as the sea lily does today.

Echinoderms also undergo deuterostome development. Recall that this type of development links them phylogenetically to chordates, which also have deuterostome development. The cladogram in Figure 27.20 shows one interpretation of the evolution of echinoderms.

Phylogeny of invertebrate chordates How could sleek, burrowing, fishlike lancelets be close relatives of saclike, sessile sea squirts? They are related because of their dorsal tubular nerve cords, notochords, pharyngeal gill slits, and postanal tails, even if sea squirts have all these features only in their larvae. Beyond this answer, scientists debate about the evolution of these animals and raise still unanswered questions. For example, from which invertebrate chordate did the fishlike tadpole larvae arise? What was the original form of that first fishlike animal?

One thing is certain: the notochord, that flexible tough rod, provided support for the animal, and it also provided a place for muscles to attach. With this arrangement, chordates could swing their backs from side to side and swim through the water, a key development in the evolution of chordates. This advance also led to the first large animals. Examine the cladogram in Figure 27.21 to see one interpretation of how chordates are related.

Fishes

Characteristics of Vertebrates

Until now you have been studying sponges, worms, and sea stars, which are all invertebrates. Recall that the four main characteristics of chordates are that they have a dorsal nerve cord, a notochord, pharyngeal pouches, and a postanal tail. Animals belonging to subphylum Vertebrata are called vertebrates. Vertebrates have a vertebral column and specialized cells that develop from the nerve cord. The vertebral column, also called a spinal column, is the hallmark feature of vertebrates. Classes of vertebrates include fishes, amphibians, reptiles, birds, and mammals.

Vertebral column

In most vertebrates, the notochord is replaced by a vertebral column that surrounds and protects the dorsal nerve cord. The replacement of the notochord happens during embryonic development. Cartilage or bone is the building material of most vertebrate endoskeletons. Cartilage is a tough, flexible material making up the skeletons or parts of skeletons of vertebrates.

The vertebral columns, shown in Figure 28.1, are important structures in terms of the evolution of animals. The vertebral column functions as a strong, flexible rod that muscles can pull against during swimming or running. Separate vertebrae enhance an animal’s ability to move quickly and easily. Bones enable forceful contraction of muscles, improving the strength of an animal.

Neural crest

As the nerve cord forms during embryonic development in vertebrates, another important process occurs: a neural crest forms. A neural crest is a group of cells that develop from the nerve cord in vertebrates. The process of neural crest formation is shown in Figure 28.2. Even though this group of cells is small, it is significant in the development of vertebrates because many important vertebrate features develop from the neural crest. These features include portions of the brain and skull, certain sense organs, parts of pharyngeal pouches, some nerve fibers, insulation for nerve fibers, and certain gland cells.

Other features that are characteristic of vertebrates include internal organs, such as kidneys and a liver. A heart and closed circulatory system also are features of all vertebrates.

Characteristics of Fishes

Fishes live in most aquatic habitats on Earth—seas, lakes, ponds, streams, and marshes. Some fishes live in complete darkness at the bottom of the deep ocean. Others live in the freezing waters of the polar regions and have special proteins in their blood to keep the blood from freezing. There are about 24,600 species of living fishes, more than all other vertebrates combined. They range in size from whale sharks that can be 18 m long to tiny cichlids that are the size of a human fingernail.

The features of fishes provided the structural basis for the development of land animals during the course of evolution. Important characteristics of fishes include the development of jaws and, in some fishes, lungs. As shown in the evolutionary tree in Figure 28.3, there are three groups of fishes, all of which are vertebrates. Although fishes’ body shapes and structures vary a great deal, they all have several characteristics in common. Most fishes have vertebral columns, jaws, paired fins, scales, gills, and single-loop blood circulation, and they are not able to synthesize certain amino acids.

Jaws Most fishes have jaws. The evolution of jaws is shown in Figure 28.4, where you can see that the anterior gill arches evolved to form jaws in ancient fishes. The development of jaws allowed ancient fishes to prey on a larger range of animals. This included being able to prey on fish that were larger in size and more active. Fishes grasp prey with their teeth and quickly crush them using powerful jaw muscles. Jaws also allow for a biting defense against predators.

Reading Check Describe why the evolution of jaws in fishes was important.

Paired fins

At the same time jaws were evolving, paired fins also were appearing in fishes. A fin is a paddle-shaped structure on a fish or other aquatic animal that is used for balance, steering, and propulsion. Pelvic fins and pectoral fins, like the ones shown in Figure 28.5, give fishes more stability. Most fishes have paired fins. Paired fins reduce the chance of rolling to the side and allow for better steering during swimming.

While fishes in ancient seas moved with precision and skill, they also were able to use their jaws in new ways. Both jaws and paired fins contributed to the evolution of a predatory way of life for some fishes and also enabled them to live in new habitats and produce more offspring.

Scales Most fishes have at least one of four different types of scales. A scale is small, f lat, platelike structure near the surface of the skin of most fishes. There are four types of fish scales. Look again at one type, ctenoid scales, at the beginning of the chapter. Ctenoid scales and a second type of scale, cycloid scales, are made of bone and skin. Cycloid scales are shown in Figure 28.6.

Ctenoid and cycloid scales are thin and flexible. The scales of a shark, called placoid scales, are made of toothlike materials and are rough and heavy. You can see a picture of placoid scales in

Figure 28.15 in the next section. The thick ganoid scales of a garfish, shown in Figure 28.6, are diamond-shaped and made of both enamel and bone.

Gills

Another adaptation that allows fishes to live in aquatic environments is their ability to get oxygen from the water. Fishes get oxygen when water that enters their mouths f lows across their gills, where oxygen from the water diffuses into the blood. Gills are composed of thin filaments that are covered with highly-folded, platelike lamellae. The gill structure of most fishes is shown in

Figure 28.7. The lamellae have many blood vessels that can take in oxygen and give off carbon dioxide.

The flow of blood in the gill is opposite to the flow of water over the gill surface. This countercurrent flow is an efficient mechanism by which oxygen can be removed from water. Up to 85 percent of oxygen dissolved in water is removed as water flows over the gills in one direction and blood in the other. Some fishes have an operculum, a movable flap that covers the gills and protects them. An operculum also aids in pumping water coming in the mouth and over the gills. Some fishes, such as lungfishes, can live out of water for short times by using structures resembling lungs. Eels can breathe through their moist skin when they are out of water.

Circulation Vertebrates have a closed circulatory system in which the heart pumps blood through blood vessels. The circulatory system of fishes is shown in Figure 28.8. In most fishes, the blood is passed through the heart in a one-way loop. From the heart, the blood goes to the gills, and then through the body, delivering oxygenated blood to tissues. The blood then returns to the heart. From the heart, blood is pumped back to the gills and then to the body again. Because this system is a complete and uninterrupted circuit, it is called a single-loop circulatory system.

In most fishes, the heart consists of two main chambers that are analogous to parts of your own heart—an atrium and a ventricle. The atrium is the chamber of the heart that receives blood from the body. From there, blood is passed to the ventricle, a chamber of the heart that pumps blood from the heart to the gills. Once the blood passes over the gills, it travels to the rest of the body.

Feeding and digestion Ancient fishes most likely were filter feeders or scavengers, sucking up organic debris on the ocean floor. With the evolution of jaws, fishes became efficient predators, and the diets of fishes changed dramatically. The digestive tract of fishes, illustrated in

Figure 28.9, consists of organs similar to those of other vertebrates.

Most fishes swallow their food whole, passing it through a tube called the esophagus to the stomach, where digestion begins. Food then passes to the intestine, where most digestion occurs. Some fishes have pyloric ceca, which are small pouches at the junction of the stomach and the intestine that secrete enzymes for digestion and absorb nutrients into the bloodstream. The liver, pancreas, and gallbladder add digestive juices that complete digestion.

Fishes are described not only by structures and their functions, but also by one important thing that they cannot do. They are not able to synthesize certain amino acids. Therefore, not only fishes, but all the vertebrates that evolved from them must get these same amino acids from the foods they eat.

Excretion

Cellular wastes are filtered from fishes’ blood by organs called kidneys. The main functional unit of the kidney is the nephron. A nephron is a filtering unit within the kidney that helps to maintain the salt and water balance of the body and to remove cellular waste products from the blood. Some cellular wastes are excreted by the gills.

The bodies of freshwater fishes take in water by osmosis because the surrounding water is hypotonic—the water contains more water molecules than the fishes’ tissues. The opposite occurs in saltwater bony fishes. Because the surrounding water is hypertonic—the water contains fewer water molecules than their tissues—their bodies tend to lose water. Kidneys, gills, and other internal mechanisms adjust the water and salt balance in the bodies of freshwater and saltwater fishes.

The brain and senses As in other vertebrates, the nervous system of fishes consists of a spinal cord and a brain. A fish brain is shown in Figure 28.10. The cerebellum is involved in coordinating movement and controlling balance. Fishes have receptors for the sense of smell that enable them to detect chemicals in the water. The olfactory bulbs record and respond to incoming chemical input. Fishes also have color vision. The optic lobes are responsible for visual input. The cerebrum coordinates input from other parts of the brain. Internal organs are under the control of the medulla oblongata.

If you have spent any time fishing, you know that fishes can detect the slightest movement in the water. Fishes can do this because they have special receptors called the lateral line system. The lateral line system enables fishes to detect movement in the water and also helps to keep them upright and balanced. You can see the lateral line system of a fish in Figure 28.10.

Reproduction

The majority of fishes reproduce through external fertilization. Male and female fishes release their gametes near each other in the water in a process called spawning. Developing embryos get nutrition from food stored in the yolk of the egg. Some fishes, such as sharks, reproduce through internal fertilization. Although fertilization takes place internally, development of the embryo of some fish species might occur outside of the female’s body when fertilized eggs are laid. Some species of fishes have internal fertilization as well as internal development of offspring. In this case, the developing embryos get nutrition from the female’s body.

Fishes that reproduce through external fertilization can produce millions of eggs in a single season. Most fishes do not protect or care for their eggs or offspring. As a result, many eggs and juvenile fishes are prey to other animals. The production of large numbers of eggs ensures that some offspring develop and survive to reproduce. One exception is the male sergeant major fish, shown in Figure 28.11. The male fish guards the fertilized eggs from predators until they hatch.

Movement Fishes are well adapted to swimming in the water. Most fishes have a streamlined shape. Most also have mucus that lubricates their body surface and reduces friction between the fish and the water.

Fins enable fishes to steer and maneuver in a variety of ways. The buoyant force of water reduces the effect of gravity on fishes. In addition, the swim bladder, which is shown in Figure 28.9, is a gas-filled space, like a balloon, found in bony fishes that allows a fish to control its depth. When gases diffuse out of the swim bladder, a fish can sink.

When gases from the blood diffuse into the swim bladder, a fish can rise in the water column.

Examine Figure 28.12. Fishes move through the water by contracting muscle groups on either side of their bodies. The arrangement of muscle in a fish allows muscle contraction to bend a large portion of the fish’s body. As the body of a fish bends, it pushes against the water, creating an opposing force that moves the fish forward, but at an angle. Alternate contraction of muscles, first on one side of the body and then on the other, keep the fish moving in an S-shaped pattern.

Diversity of Today’s Fishes

Classes of Fishes

You have read about jellyfish, crayfish, and various shellfish, but none of these are true fishes. True fishes belong to three groups based on their body structure. Hagfishes and lampreys are jawless fishes; sharks, skates, and rays are cartilaginous fishes; and the bony fishes include both ray-finned and lobe-finned fishes.

Jawless fishes Hagfishes, as shown in Figure 28.13, are jawless, eel-shaped fishes that do not have scales, paired fins, or a bony skeleton. Members of class Myxini, hagfishes have a notochord throughout life. Although they do not develop a vertebral column, they do have gills and many other characteristics of fishes. They live on the seafloor and feed on soft-bodied invertebrates and dead or dying fishes. Even though they are almost blind, their keen chemical sense enables them to locate food. They either enter the body of the fish through the mouth or they scrape an opening into the fish with toothlike structures on their tongues. After eating the internal parts of the fish, the hagfish leaves only a sac of skin and bones.

Hagfishes are known for their ability to produce slime. If threatened, they secrete fluid from glands in their skin. The fluid, when in contact with seawater, forms a slime that is slippery enough to prevent the hagfishes from being caught by predators.

Lampreys, like hagfishes, also are jawless, eel-shaped fishes that lack scales, paired fins, or a bony skeleton. Lampreys, members of class Cephalaspidomorphi, retain a notochord throughout life, as do hagfishes. Lampreys have gills and other characteristics of fishes. Adult lamprey, shown in Figure 28.13, are parasites that feed by attaching themselves to other fishes. They use their sucker-like mouth and tongue with toothlike structures to feed on the blood and bodily fluids of their hosts.

Reading Check List the characteristics of jawless fishes.

Cartilaginous fishes

When you hear the word shark, the first thing that might come to mind is a large fish with many sharp teeth. In spite of being famous for teeth, a shark’s main distinguishing feature is its skeleton. All cartilaginous fishes have skeletons made of cartilage.

The skeleton of a shark also is made of cartilage, which gives the skeleton flexibility, and calcium carbonate, which gives it strength.

Sharks belong to class Chondrichthyes. Some species of sharks have several rows of sharp teeth as shown in Figure 28.14. As teeth are broken or lost, new ones move forward to replace them. Most sharks also have a streamlined shape, with a pointed head and a tail that turns up at the end, as shown in Figure 28.15.

These streamlining features, along with strong swimming muscles and sharp teeth, make sharks one of the top predators in the sea. They can sense chemicals in the water, allowing them to detect prey from a distance of one kilometer. As they move in closer, their lateral line systems can detect vibrations in the water. Finally, when they are in the last stages of pursuit, they use their vision and other receptors to detect the bioelectrical fields given off by all animals. An additional adaptation to a predatory life includes tough skin with placoid scales, shown in Figure 28.15.

Not all sharks have rows of teeth. Whale sharks, the largest living sharks, are filter-feeders with specialized straining structures in their mouths. Other sharks have mouths adapted to feeding on shelled mollusks.

Skates and rays are cartilaginous fishes adapted to life on the bottom of the sea. In addition to their flattened bodies, their pectoral fins, shown in Figure 28.16, are enlarged and attached to their heads. Their winglike fins flap slowly as they swim along the seafloor in search of mollusks and crustaceans, which they crush with their teeth.

Bony fishes Class Osteichthyes contains two groups of bony fishes: the ray-finned fishes, belonging to subclass Acti-nopterygii, and the lobe-finned fishes, belonging to the subclass Sarcopterygii. Modern ray-finned fishes have a bony skeleton, ctenoid or cycloid scales, an operculum covering the gills, and a swim bladder. The most distinguishing feature of ray-finned fishes is in their name. The thin mem- branes of these fishes’ fins are supported by thin, spinelike rays, which are shown in Figure 28.17. Most fishes alive today, including salmon and trout, are ray-finned fishes.

There are only eight species of lobe-finned fishes living today. Their fins, shown in Figure 28.17, have muscular lobes and joints similar to those of land vertebrates. This makes the fins more flexible than those of ray-finned fishes. Lobe-finned fishes, such as the lungfish, usually have lungs for gas exchange. When drought occurs, a lungfish can burrow with its fleshy fins into the mud and breathe air. When rain returns, lungfishes come out of their burrows.

Coelacanths are another small group of lobe-finned fishes that many people thought had become extinct about 70 million years ago. However, in 1938, some people fishing off the coast of South Africa caught a coelacanth. Since that time, other coelacanths have been caught. A third group of lobe-finned fishes, now extinct, is thought to be the ancestor of tetrapods. A tetrapod, shown in Figure 28.17, is a four-footed animal with legs that have feet and toes that have joints.

Evolution of Fishes

In the ancient seas of the Cambrian, the first vertebrates wriggled through the water. They were jawless and toothless, but they did have gills, heads, and tails that moved them through the water. The cladogram shown in Figure 28.18 is one interpretation of the phylogeny of fishes. As you examine the cladogram, notice the characteristics of fishes that developed during the course of their evolution.

First fishes

These first jawless, toothless fishes sucked up organic materials from the ocean floor as if they were miniature vacuum cleaners. Ostracoderms were the next group of fishes to appear in the fossil record of the **Ordovician Period**. Ostracoderms had head shields made of bone, a bony outer covering, and paired fins. They were jawless filter-feeders, many of which rested on the bottom of ancient seas.

The bony armor of ostracoderms was an evolutionary milestone in the development of vertebrates. Stronger movement is possible when muscle is attached to bone. Even though ostracoderms became extinct, scientists hypothesize that modern fishes share an ancestry with ostracoderms.

Age of fishes During the **Devonian Period**, modern fishes had their beginnings. Some were jawless, while some, such as the placoderms, had jaws, a covering of bony plates, paired fins, and an internal skeleton. A fossil of a placoderm fish is shown in Figure 28.19. Recall that three of these features are characteristics of the fishes that eventually replaced the placoderms as they became extinct. The **Devonian Period** often is referred to as the Age of Fishes because of the adaptive radiation of fishes that occurred at that time.

Ecology of Fishes

Fishes are an important source of food in all aquatic ecosystems. Yet, their freshwater habitats and saltwater habitats are being changed by human activities, such as damming rivers or pollution. Fishes are good bioindicators of the environmental health of an aquatic system. When noncommercial fish populations decrease, the main cause often is habitat alteration. When fishes decline in numbers, not only are there negative human economic impacts, ecosystems also can become unbalanced as well.

Habitat alteration

Some fishes, such as salmon, migrate. Salmon spend their adult lives in the ocean but return to freshwater to spawn in the streams where they hatched. In the Pacific Northwest, river and stream habitats have been changed by the construction of dams. Dams interfere with the upstream and downstream migration of salmon, as shown in Figure 28.20. The end result in the Pacific Northwest, for example, is that the number of salmon swimming upstream now is only about three percent of the 10 to 16 million salmon that swam up the rivers 150 years ago.

Pollution

The habitats of fishes can be changed by pollution which can reduce the quality of water in lakes, rivers, and streams. This can result in a decline in both the number and diversity of fishes in an area. In some cases, when the cause of habitat alteration is stopped and suitable conditions return, fishes also return. For example, Atlantic salmon were not observed in the Penobscot River in Maine for ten years during a time when intense pollution altered water quality. When the pollution was stopped, the salmon returned.

Amphibians

Evolution of Tetrapods

Tetrapods first appeared on Earth about 360 million years ago. The first amphibians to crawl onto land were different from today’s amphibians. Examine the evolutionary tree in Figure 28.21 to see how amphibians are related to other vertebrates. As millions of years passed, animals adapted to the conditions of life on land.

The move to land Animals faced several physical challenges in the move from water to land. Table 28.1 lists some of the differences between conditions of life in the water and life on land. These differences include buoyancy, oxygen concentration, and temperature.

Table 28.1 also gives examples of how terrestrial vertebrates adapted to life on land.

In addition to the differences listed in Table 28.1, another difference between conditions in water and on land is that sound travels more quickly through water. Fishes use lateral line systems to sense vibrations, or sound waves, in water. A lateral line system is not effective in air. The ears of terrestrial vertebrates developed to sense sound waves traveling through air.

Terrestrial habitats In spite of the challenges associated with terrestrial life, there are many habitats available to animals on land. The different biomes on land, including tropical rain forests, temperate forests, grass-lands, deserts, taiga, and tundra, provide suitable habitats for animals with appropriate adaptations.

Characteristics of Amphibians

Have you ever watched a tadpole in a jar of pond water? Examine and describe the tadpole in Figure 28.22. A tadpole is the limbless, gill-breathing, fishlike larva of a frog. Day by day, the tadpole undergoes a metamorphosis—hind legs form and grow longer, the tail shortens, gills are replaced by lungs, and forelimbs sprout. In just a few weeks or months, depending on the species, the tadpole becomes an adult frog. Most amphibians begin life as aquatic organisms. After metamorphosis, they are equipped to live life on land.

Modern amphibians include frogs, toads, salamanders, newts, and legless caecilians. Most amphibians are characterized by having four legs, moist skin with no scales, gas exchange through skin, lungs, a double-loop circulatory system, and aquatic larvae.

Feeding and digestion

Most frog larvae are herbivores, whereas salamander larvae are carnivores. However, as adults their diets are similar as both groups become predators and feed on a variety of invertebrates and small vertebrates. Some salamanders and legless amphibians use just their jaws to catch prey. Others, such as frogs and toads, can flick out their long, sticky tongues with great speed and accuracy to catch flying prey.

Food moves from the mouth through the esophagus to the stomach, where digestion begins. From the stomach, food moves to the small intestine, which receives enzymes from the pancreas to digest food.

From the intestine, food is absorbed into the blood-stream and delivered to body cells. Food moves from the small intestine into the large intestine before waste material is eliminated. At the end of the intestine is a chamber called the cloaca. The cloaca is a chamber that receives the digestive wastes, urinary waste, and eggs or sperm before they leave the body.

Excretion Amphibians filter wastes from the blood through their kidneys, and excrete either ammonia or urea as the waste product of cellular metabolism. Ammonia is the end product of protein metabolism and is excreted by amphibians that live in the water. Amphibians that live on land excrete urea that is made from ammonia in the liver.

Unlike ammonia, urea is stored in the urinary bladder until it is eliminated from the body through the cloaca.

Respiration and circulation As larvae, most amphibians exchange gases through their skin and gills. As adults, most breathe through lungs, their thin, moist skin, and the lining of the mouth cavities. Frogs can breathe through their skin either in or out of water. This ability enables them to spend the winter protected from the cold in the mud at the bottom of a pond.

The circulatory system of amphibians is shown in Figure 28.23. It consists of a double loop instead of the single loop you learned about in fishes. The first loop moves oxygen-poor blood from the heart to pick up oxygen in the lungs and skin, and then moves the oxygen-filled blood back to the heart. During circulation in the second loop, blood filled with oxygen moves from the heart through vessels to the body, where the oxygen diffuses into cells.

Amphibians have three-chambered hearts. The atrium is completely separated into two atria by tissue. The right atrium receives deoxygenated blood from the body, while the left atrium receives oxygenated blood from the lungs. The ventricle of amphibians remains undivided.

The brain and senses

Like fishes, the nervous systems of amphibians are well developed. The differences in conditions between life in the water and life on land are reflected in the differences between the brains of fishes and those of amphibians. For example, the forebrain of frogs contains an area that is involved with the detection of odors in the air. The cerebellum, which is important in maintaining balance in fishes, is not as well developed in terrestrial amphibians that stay close to the ground.

Vision is an important sense for most amphibians. They use sight to locate and capture prey that fly at high speeds and to escape predators. Frogs’ eyes have structures called nictitating membranes. The nictitating membrane is a transparent eyelid that can move across the eye to protect it underwater and keep it from drying out on land.

The amphibian ear also shows adaptation to life on land. The tympanic membrane is an eardrum. In frogs, it is a thin external membrane on the side of the head, as shown in Figure 28.24.

Frogs use their tympanic membrane to hear high-pitched sounds and to amplify sounds from the vocal cords. Other senses in amphibians include touch, chemical receptors in skin, taste buds on the tongue, and sense of smell in the nasal cavity.

It is important for amphibians to sense the temperature of their environment because they are ectotherms. Ectotherms are animals that obtain their body heat from the external environment. Ectotherms cannot regulate their body temperatures through their metabolism, so they must be able to sense where they can go to get warm or to cool down. For example, if it is cold, a toad can find a warm, moist rock on which to bask and warm itself.

Reproduction and development

Like many amphibians, female frogs lay eggs to be fertilized by males in the water. The eggs do not have shells or protective coverings to keep them from drying out. The eggs, like the ones shown in Figure 28.25, are covered with a sticky, jelly-like substance that helps them stay anchored to vegetation in the water. After fertilization, the developing embryo uses the yolk in the egg for nourishment until it hatches into a tadpole. A tadpole, like the one shown in Figure 28.22, changes from a gill-breathing, legless herbivore with fins and a two-chambered heart into a lung-breathing, four-legged carnivore with a three-chambered heart. The stages of metamorphosis are primarily under the control of chemicals released within the tadpole’s body.

Amphibian Diversity

Biologists classify modern amphibians into three orders. Order Anura contains 4200 species of frogs and toads. Order Caudata has about 400 species of salamanders and newts. One hundred and fifty species of wormlike caecilians make up order Gymnophiona. Frogs, toads, and salamanders live in moist areas in a variety of habitats, while newts are aquatic. Caecilians are tropical burrowing animals.

Frogs and toads Frogs and toads, shown in Figure 28.26, lack tails and have long legs enabling them to jump. Frogs have longer and more powerful legs than toads and are able to make more powerful jumps than the small hops of toads. Frogs have moist, smooth skin, while the skin of toads tends to be bumpy and dry. Though both need to be near water to carry out reproduction, toads generally live farther away from water than do frogs. Another difference between frogs and toads is that toads have kidney-bean-shaped glands near the back of their heads that release a bad-tasting poison. The poison discourages predators from eating them.

Salamanders and Newts Unlike frogs and toads, salamanders and newts have long, slim bodies with necks and tails, as shown in Figure 28.27. Most salamanders have four legs and thin, moist skin and cannot live far from water. Like frogs, most salamanders lay their eggs in water. The larvae look like miniature salamanders, except that they have gills. Newts, like the one in Figure 28.27, generally are aquatic throughout their lives, while most salamanders, as adults, live in moist areas such as under logs or in leaf litter. Salamanders range in size from about 15 cm long to the giant salamander that is 1.5 m long. An adult salamander’s diet consists of worms, frog eggs, insects, and other invertebrates.

Caecilians Caecilians are different from other amphibians because they are legless and wormlike, as shown in Figure 28.28.

They burrow in the soil and feed on worms and other invertebrates. Skin covers the eyes of many caecilians, so they might be nearly blind. All caecilians have internal fertilization. They lay their eggs in moist soil located near water. Caecilians can be found in tropical forests of South America, Africa, and Asia.

Evolution of Amphibians

Fossil evidence shows that the first tetrapods evolved limbs in water before they moved to land. Many adaptations that are useful on land first evolved in water. For example, legs with feet and toes could be helpful in moving through bottom vegetation. Ankles and wrists might have increased maneuverability. The attachment of hip bones to the vertebral column might have helped predators attack prey more easily.

The cladogram shown in Figure 28.29 is one interpretation of the evolution of amphibians. Many scientists believe that early tetrapods are most closely related to a group of now extinct, lobe-finned fishes called rhipidistians. Characteristics that both early tetrapods and rhipidistians share include similar bone structure in the skull and limbs, nostril-like openings in the tops of their mouths, and a similar tooth structure.

Early tetrapods had defined legs with feet, but the construction of the legs was too weak for these animals to walk easily on land. Ichthyostegans, shown in Figure 28.17, had more support in the shoulder bones, heavier leg bones, and more muscular features that enabled them to pull themselves onto land and move a little more easily. The skull had the same general shape as the skulls of lobe-finned fishes. Tetrapod groups branched out to produce the three major groups of amphibians alive today, as well as reptiles, birds, and mammals.

Ecology of Amphibians

In recent decades, amphibian populations have been declining worldwide. Scientists have been collecting data to determine possible causes for the decline. The results have varied. In some cases, the cause can be isolated to a local condition. In other cases, the cause might be the result of several factors occurring on a large scale.

Local factors

In some cases, such as that of the California red-legged frog, the decline is due to habitat destruction. When wetlands are drained and buildings are built in the areas instead, these areas of water are no longer available to amphibians that must lay eggs in or near water to reproduce successfully. In other areas, the introduction of exotic species— species that are not found in that area naturally—has affected amphibian populations. The exotic species compete with the amphibians for food and habitat space, or they are predators of amphibians. The introduction of trout, which prey on tadpoles, into the high-altitude lakes of California’s Sierra Nevada Mountains is thought to have contributed to the near extinction of the mountain yellow-legged frog found in that region.

Global factors In addition to local factors, various global factors might be at work causing amphibian decline. Aspects of global climate change, such as increased temperature, decreased soil moisture, increased length of the dry season, and changes in rainfall can cause either death or stress to the bodies of amphibians, making them more susceptible to disease.

Figure 28.30 compares healthy toad eggs versus those infected by a fungus. Some scientists believe that global climate changes that have led to a decreased amount of rainfall leave developing amphibians’ eggs in shallow pond water. Because the depth of the water is reduced, the eggs are exposed to more ultraviolet light. Laboratory experiments have shown that increased exposure to UV light leads to an increased risk of fungal infection in amphibian eggs.

Reptiles

Characteristics of Reptiles

In Chapter 28, you learned that vertebrates with well-developed limbs, circulatory and respiratory systems, and other adaptations that equipped them for life on land moved from water to land. However, amphibians were left vulnerable to the drying effects of life on land with their shell- less eggs and larvae that breathed through gills. In contrast, reptiles, like the Western fence lizard shown in Figure 29.1, are fully adapted to life on land and were the first completely terrestrial vertebrates. Char- acteristics that allow reptiles to succeed on land include a shelled egg, scaly skin, and more efficient circulatory and respiratory systems.

Amniotic eggs

As you can see in the evolutionary tree in Figure 29.1, reptiles have characteristics in common with other groups that have an amnion and other membranes that surround the embryo as it develops.

An amnion is a membrane that surrounds a developing embryo. It is filled with fluid that protects the embryo during development. Animals that undergo this type of development are called amniotes and include reptiles, birds, and mammals.

An amniotic egg, like the one shown in Figure 29.2, is covered with a protective shell and has several internal membranes with fluids contained between the membranes. Inside the egg, the embryo is self-sufficient because it gets its nutrition from food in the yolk sac inside the egg. Bathing the embryo within the amnion is amniotic fluid.

Amniotic fluid mimics the aquatic environments of fish and amphibian embryos. The allantois is a membrane that forms a sac that contains wastes produced by the embryo. The outer-most membrane of the egg is the chorion, which allows oxygen to enter and keeps fluid inside the egg. In reptiles, the leathery shell protects the internal fluids and embryo, and prevents the egg from drying out on land. In birds, the shell is hard instead of leathery.

Dry, scaly skin In addition to keeping fluid in their eggs, reptiles also must keep fluids in their bodies. The dry skin of reptiles keeps them from losing internal fluids to the air. A layer of scales on the exterior of many reptiles also keeps them from drying out. However, one problem with having a tough outer covering is that an organism could have difficulty growing larger. In order to grow, some reptiles, like the snake in Figure 29.3, periodically must shed their skins in a process called molting. You might have seen the molt of a snake’s skin while hiking a nature trail.

Respiration Most reptiles, except for some aquatic turtles, depend primarily on lungs for gas exchange. Recall that when amphibians breathe, they squeeze their throats to force air into their lungs. Reptiles are able to suck air into their lungs, or inhale, by contracting muscles of the rib cage and body wall to expand the upper part of the body cavity

in which the lungs are held. They exhale by relaxing these same muscle groups. Reptiles exchange gases in lungs that have larger surface areas for gas exchange than the lungs of amphibians. With more oxygen, more energy can be released through metabolic reactions and made available for more complex movements.

Circulation In most reptiles, oxygen from the lungs enters into a circulatory system that is similar to that of amphibians. Most reptiles have two separate atria and one ventricle that is partially divided by an incomplete septum, as shown in Figure 29.4. In crocodiles, however, the septum in the ventricles is complete, thereby resulting in a four-chambered heart. The separation into two ventricles keeps oxygen-rich blood separate from the oxygen-poor blood throughout the heart.

Because reptiles generally are larger than amphibians, they need to pump blood forcefully enough to reach parts of the body far away from the heart. In an example from the past, the dinosaur Brachiosaurus had to pump blood more than 6 m from the heart to the head!

Feeding and digestion

The organs of the digestive system of reptiles, shown in Figure 29.4, are similar to those of fish and amphibians. Reptiles have a variety of feeding methods and diets. Most reptiles are carnivores, but some, such as iguanas and tortoises, are herbivores that feed on plants, and some turtles are omnivores. Turtles and crocodiles have tongues that help them swallow. Some lizards, such as the chameleon, have long, sticky tongues for catching insects.

Snakes have the ability to ingest prey much larger than themselves. The bones of the skull and jaws of snakes are joined loosely so that they can spread apart when taking in large food materials, as shown in Figure 29.5. To swallow, the opposite sides of the upper and lower jaws can alternately thrust forward and retract to draw in the food. Some snakes have venom that can paralyze and begin digestion of their prey.

Excretion The excretory system of reptiles is adapted to life on land. The kidneys, such as the one shown in Figure 29.4, filter the blood to remove waste products. When urine enters the cloaca, water is reabsorbed to form uric acid, which is a semisolid excretion. This method of water reabsorption enables reptiles to conserve water and maintain homeostasis of water and minerals in their bodies.

The brain and senses Reptile brains are similar to amphibian brains, except that the cerebrum of reptiles is larger. Because vision and muscle function are more complex, the optic lobes and cerebellum portions in the brain of reptiles also are larger than those of amphibians.

Vision is the main sense for most reptiles, and some reptiles even have color vision. Hearing varies in reptiles. Some reptiles have tympanic membranes similar to those of amphibians, while others, such as snakes, detect vibrations through their jaw bones.

The sense of smell is more highly evolved in reptiles than it is in amphibians. You might have seen video of snakes flicking their forked tongues. They do this to smell odors. The odor molecules stick to the tongue, which the snake places in its mouth. The odor molecules are transferred to a pair of saclike structures called Jacobson’s organs, which are shown in Figure 29.6. These structures are on the roof of a snake’s mouth and they sense odors. Experiments have shown that without Jacobson’s organs, snakes have difficulty finding prey and mates.

Temperature control

Like amphibians, reptiles are ectotherms that cannot generate their own body heat. They regulate their body temperatures behaviorally. You might have seen turtles basking in the Sun on top of rocks. This raises body temperature. Body temperature can be lowered by moving into the shade or a cool burrow. Some reptiles in temperate regions survive winter by burrowing or going into a state of inactivity with lower body metabolism and lower body temperature. Others, such as some snakes, gather together in masses of hundreds during the winter. Heat loss is reduced when the snakes are covering each other.

Movement Compare the leg position of the salamander to the leg position of the crocodile shown in Figure 29.7. Note that the salamander’s belly is on the ground, while the crocodile’s belly is above the ground. Like amphibians, some reptiles move with limbs sprawled to their sides and push against the ground while swinging their bodies from side to side. Crocodiles, however, have their limbs rotated farther under the body and, as a result, can bear more weight and move faster. To bear more body weight on land, reptiles’ skeletons are stronger with heavier bone structure. Reptiles also have claws on their toes which aid in digging, climbing, and gripping the ground for traction.

Reproduction Reptiles have internal fertilization. In most reptiles, after fertilization, the egg develops to form the new embryo and all of the membranes associated with an amniotic egg to help ensure safe development. The female reproductive system produces a leathery shell around the eggs that will be laid. The developing embryo is nourished from the yolk of the egg. The female usually digs a hole and lays eggs in the ground or in plant debris. After laying their eggs, most females leave them alone to hatch. Alligators and crocodiles build a nest in which to lay eggs and tend to young after they hatch. Some snakes and lizards keep their eggs in their bodies until they hatch. In this way, the eggs are protected in the mother’s body until they are fully developed young.

Diversity of Modern Reptiles

From the ancient Age of Reptiles, in which an enormous variety of dinosaurs ruled Earth, four living orders of reptiles remain—snakes and lizards belonging to order Squamata, crocodiles and alligators belonging to order Crocodilia, turtles and tortoises belonging to order Testudinata, and tuataras belonging to order Sphenodonta.

Lizards and snakes Lizards commonly have legs with clawed toes. Also, lizards usually have movable eyelids, a lower jaw with a movable hinge joint allowing for flexibility in jaw movement, and tympanic membranes. Common lizards include iguanas, chameleons, geckos, and anoles. An iguana is shown in Figure 29.8.

Snakes are legless and have shorter tails than lizards. Snakes lack movable eyelids and tympanic membranes. Like lizards, however, snakes have joints in their jaws enabling them to eat prey larger than their heads. Some snakes, such as the rattlesnake shown at the beginning of the chapter, have venom that can slow down or even kill their prey. Other snakes, such as the python shown in Figure 29.8, anacondas, and boas, are constrictors. Constrictors generally are very large snakes. They suffocate their prey by wrapping around the prey’s body and tightening until the prey dies because it no longer can breathe.

Turtles

Observe the turtle shown in Figure 29.9. The protective shell in which a turtle’s body is encased makes it different from other reptiles. The dorsal part of the shell is called the carapace and the ventral part of the shell is called the plastron. The vertebrae and ribs of most turtles are fused to the inside of the carapace. Many turtles can pull their head and legs inside their shells to protect themselves against predators. Some turtles are aquatic, and some live on land. Turtles that live on land are called tortoises. Tortoises and turtles do not have teeth, but they have a sharp beak that can deliver a powerful bite.

Crocodiles and alligators Order Crocodilia includes crocodiles, alligators, and caimans. Crocodilians have a four-chambered heart, another adaptation which is also a characteristic of birds and mammals. Because a four-chambered heart can deliver oxygen more efficiently, powerful muscles enable crocodilians to move quickly and aggressively, both in and out of the water, attacking animals as large as cattle, deer, and humans.

Crocodiles have a long snout, sharp teeth, and powerful jaws. The teeth of crocodiles are similar to those of dinosaurs and the earliest birds. This type of tooth shows the close relationship between modern crocodilians and the dinosaurs that gave rise to living birds.

Alligators, like the one in Figure 29.9, generally have a broader snout than crocodiles. The upper jaw of an alligator is wider than the lower jaw. When an alligator closes its mouth, the upper jaw overlaps the lower jaw and its teeth are almost completely covered. The upper and lower jaws of a crocodile are about the same width. So when a crocodile closes its mouth, some teeth in the lower jaw are easily visible.

Tuataras Tuataras look like large lizards, as shown in Figure 29.10, and are found only on islands off the coast of New Zealand. There are only two species of tuataras. Tuataras have a spiny crest that runs down the back and a “third eye” on top of the head. This structure is covered with scales but can sense sunlight. Biologists think that it might keep the tuatara from overheating in the Sun. One distinguishing feature of tuataras is that they have unique teeth compared to those of other reptiles. Two rows of teeth on the upper jaw shear against one row in the lower jaw, making them effective predators of small vertebrates.

Evolution of Reptiles

The cladogram in Figure 29.11 shows one interpretation of how early amniotes underwent adaptive radiation, giving rise to reptiles as well as modern birds and mammals. Recall that amniotes are vertebrates in which the embryo is encased in an amniotic membrane. As shown in the cladogram, early amniotes separated into three lines, each having a different skull structure. Anapsids, which might have given rise to turtles, have a skull that has no openings behind the eye sockets. Diapsids, which gave rise to crocodiles, dinosaurs, modern birds, tuataras, snakes, and lizards, have a skull with two pairs of openings behind each eye socket. Synapsids, which gave rise to modern mammals, have one opening behind each eye socket.

Dinosaurs

For 165 million years, dinosaurs dominated Earth. Some, such as Tyrannosaurus rex, stood almost 6 m high, were 14.5 m long, weighed more than 7 tonnes, and were predatory. Others, such as Triceratops, had massive horns and were herbivores. Despite their diversity, dinosaurs can be divided into two groups based on the structure of their hips. A comparison of the two groups is shown in Figure 29.12. Saurischians had hip bones that radiated out from the center of the hip area. In Ornithischians, some bones projected back toward the tail.

Like birds and crocodiles, some dinosaurs built nests and cared for eggs and young. Some dinosaurs might have had the ability to regulate their body temperatures. Fossil evidence shows that one group of dinosaurs had feathers and evolved into today’s birds.

The **Cretaceous Period** is known for worldwide mass extinction of many species, including all dinosaurs. Some scientists hypothesize that a meteorite crashed to Earth and caused this extinction. Clouds of dust might have blocked the Sun, causing a much cooler climate to develop. This change, along with fires, toxic dust, and gases, could have caused the death of many plants and animals at this time. When dinosaurs disappeared, the niches they had occupied were made available for other vertebrates to evolve.

Ecology of Reptiles

Reptiles are important parts of food chains both as prey and as predators. The balance of an ecosystem can be disrupted when a reptile species is removed. For example, when certain snakes continually are removed from an environment, rodent populations can increase. Loss of habitat and the introduction of exotic species are factors that contribute to the decline in population of some reptile species.

Habitat loss Both the American alligator (Alligator mississippiensis) and the American crocodile (Crocodylus acutus) have been affected by habitat loss in the Florida Everglades. The destruction and fragmentation of wetlands for building development has led to reduced numbers of these reptiles. The American crocodile remains endangered, with only 500-1200 remaining in Florida. With the passage of laws to protect wetlands in certain areas, the American alligator population has rebounded enough so that its status has been changed from endangered to threatened.

Introduction of exotic species

When species that are not naturally found in an area, exotic species, are introduced, the local animals might suffer due to predation or competition for resources. For example, when the mongoose, a small mammal, was introduced into Jamaica to kill rats in sugarcane fields, the mongoose fed mostly on several lizard species which are now endangered. This includes the Jamaican iguana, which was thought to have been extinct due to the introduction of the mongoose. In 1990, a small population was discovered in a remote area of Jamaica.

Some species, such as the San Francisco garter snake shown in Figure 29.13, have suffered a population decline due to both habitat loss and the introduction of exotic species. The use of land for building houses, other buildings, and agriculture has led to habitat loss for this snake. The introduction of the American bullfrog, which is not native to California, also has impacted the population number of this snake. The bullfrog eats both the garter snake as well as the red-legged frog, a food source of the garter snake.

Birds

Characteristics of Birds

Suppose your teacher asked you to describe a bird. You might respond that birds have feathers and that they fly—two characteristics that distinguish them from other vertebrates. Birds belong to class Aves and include about 8600 species, making them the most diverse of all terrestrial vertebrates. Birds range in size from tiny hummingbirds hovering over bright flowers to large flightless ostriches running across the

African plains. Birds are found in deserts, forests, mountains, prairies, and on all seas.

As shown on the evolutionary tree in Figure 29.14, birds and reptiles have a common ancestor. Birds have many characteristics that demonstrate their reptilian roots. For example, birds lay amniotic eggs. In addition, scales similar to those of reptiles cover the legs of birds.

You can think of a bird as a collection of adaptations to a lifestyle that includes flight. The adaptations include being able to generate their own body heat internally, or endothermy, feathers, and light-weight bones. The respiratory and circulatory systems of birds also are adapted to provide more oxygen to working muscles to support flight.

Endotherms Unlike reptiles, birds are endotherms. An endotherm is an organism that generates its body heat internally by its own metabolism. The high metabolic rate associated with endothermy generates a large amount of ATP that can be used to power flight muscles or for other purposes. Endothermy might have evolved in dinosaurs and been passed along to birds. Endotherms generate heat due to their normal body metabolism. The body temperature of a bird is about 41°C. Your body temperature is about 37°C. A high body temperature enables the cells in a bird’s flight muscles to use the large amounts of ATP needed for rapid muscle contraction during flight.

Feathers Birds are the only living animals to have feathers. Feathers are specialized outgrowths of the skin of birds. They are made of keratin, a protein in the skin that also makes up hair, nails, and horns of other animals. Feathers have two main functions: flight and insulation. Feathers keep heat generated during metabolism from escaping from the body of the bird. When a bird fluffs its feathers, it creates a dead air space that traps the heat. Similarly, if you are covered with a quilt while you sleep, the quilt creates dead air space between you and the cool air in the room so that you do not lose body heat.

Feathers that cover the body, wings, and tail of a bird are called contour feathers. Examine the contour feathers shown in Figure 29.15. Contour feathers consist of a shaft with barbs that branch off. Barbules branch off barbs and are held together by hooks. If two adjoining barbs become separated, they can be rejoined like the teeth of a zipper. Birds repair broken links when they preen their feathers. They use their bills to preen their feathers, drawing the length of the feather through the bill to zip up broken links. Birds spend a large amount of time maintaining their feathers. Many birds have a preen gland, a gland located near the base of the tail that secretes oil. During preening, birds spread oil from the preen gland over their feathers, thereby adding a waterproofing coating. Down feathers, shown in Figure 29.15, are soft feathers located beneath contour feathers. Down feathers do not have hooks to hold barbs together. As a result, the looser structure of down feathers can trap air that acts as insulation.

Lightweight bones

Another adaptation of birds that allows flight is their strong, lightweight skeletons. The bones of birds are unique because they contain cavities of air. Figure 29.15 shows the internal structure of a bird bone. Despite the fact that the bones are filled with air, they are still strong.

Have you ever found the wishbone in a piece of chicken or turkey? The wishbone is formed from fused collarbones, as shown in Figure 29.15.

Fusion of bones in the skeleton of a bird makes the skeleton sturdier, another adaptation for flight. Large breast muscles, which can make up 30 percent of a bird’s total weight, provide the power for flight. These muscles connect the wing to the breastbone, called the sternum, also shown in Figure 29.15. The sternum is large and has a keel to which the muscles attach.

Respiration Flight muscles use a large amount of oxygen, and the respiratory systems of birds are well-adapted to provide it. Not only do birds have much more space for air in their respiratory system than reptiles, birds also have one-way air circulation. When a bird inhales, oxygenated air moves through the trachea into posterior air sacs, shown in Figure 29.16. Other air already within the respiratory system is drawn out of the lungs, where gas exchange occurs, and into the anterior air sacs. When a bird exhales, the deoxygenated air in the anterior air sacs is expelled from the respiratory system and oxygenated air from the posterior air sacs is sent to the lungs. The net result is that only oxygenated air is moved through the lungs, and it is moved in a single direction relative to blood flow.

Circulation

A bird’s circulatory system also helps it maintain high levels of energy by efficient delivery of oxygenated blood to the body. Recall that crocodiles are the only reptiles to have a heart ventricle completely divided by a septum. Birds also have a four-chambered heart, as shown in Figure 29.17. Having two ventricles keeps the oxygenated and deoxygenated blood separated and makes delivery of oxygenated blood more efficient. The left atrium receives blood from the lungs. This blood is pumped into the left ventricle and out to the body. Blood returning from the body is delivered to the right atrium, then moves into the right ventricle and on to the lungs where it will pick up more oxygen.

Reproduction

The reproductive activities of birds are complex. They include establishing territories, locating mates, courtship behavior, mating, constructing nests, incubating eggs, and feeding young. During breeding season, many birds gather in large colonies where they breed and take care of young. All birds have internal fertilization. Generally, after fertilization, the amniotic egg develops and is encased within a hard shell while still within the body of the female. After the shell forms, the egg or eggs are released through the cloaca to a nest, where the male or female or both birds incubate the egg or eggs and feed the young after hatching. To incubate means to maintain favorable conditions for hatching. Birds sit on their eggs to incubate them.

Diversity of Modern Birds

Modern birds are divided into about 27 living orders, depending on the classification system used. Anatomical differences, specific behaviors, songs, and the habitats occupied distinguish the orders. In Table 29.1, you will study the most common orders of birds and their adaptations. The largest order of birds is the Passeriformes, which often are called perching birds or songbirds. There are more than 5000 species in order Passeriformes. Flightless birds, including ostriches, emus, and kiwis, have reduced or no wings. The kiwi, a bird about the size of a chicken found in New Zealand, lays an egg that is extremely large given the size of the bird. Some birds, such as penguins, geese, and ducks, have adaptations that allow them to swim. Penguins use their wings as paddles to swim through the water. Ducks and geese have webbed feet.

Evolution of Birds

Fossil evidence shows that birds descended from archosaurs, the same line from which crocodiles and dinosaurs evolved, as you saw in Figure 29.11. Similarities between birds and reptiles are apparent.

They have similar skeletal features, kidney and liver function, amniotic eggs, and behaviors such as nesting and caring for young. Discoveries of fossils of feathered dinosaurs in China support the idea that birds evolved from a group called theropod dinosaurs.

Feathered dinosaurs

Three different species of birdlike dinosaurs from the Chinese fossil beds have been carefully studied. Sinosauropteryx had a coat of downy, featherlike fibers. Protoarchaeopteryx and Caudipteryx, illustrated in Figure 29.20, had long feathers on their front appendages and on their tails. The downy dinosaur feathers might have functioned as insulation, and the front appendage feathers might have served as balancing devices as the dinosaurs ran along the ground.

In 1861, in southern Germany, paleontologist Hermann von Meyer discovered what is now known to be the oldest bird fossil—Archaeopteryx. Archaeopteryx, illustrated in Figure 29.20, lived about 150 million years ago. This ancient bird had a long reptile-like tail, clawed fingers in the wings, and teeth. These are features that modern birds do not have. Yet, like modern birds, its body was covered with feathers. The feathers were asymmetrical, like those found only in modern birds that fly. Recent fossil evidence also shows that the brain of Archeopteryx was much like that of modern birds.

Recent discoveries

Two recent fossil discoveries of ancient birds, Sinornis and Eoalulavis, show that these birds had more features of modern birds. These birds lived between 135 and 115 million years ago. Eoalulavis had features that most likely enabled slow, hovering flight.

Ecology of Birds

Birds are important parts of food chains as predators of small mammals, arthropods, and other invertebrates. You probably have seen a robin pulling a worm out of the ground. Birds also are prey of larger birds and mammals. Therefore, birds are important parts of food chains and food webs as prey within an ecosystem.

Birds also play an important role in the dispersal of seeds. Birds eat seeds or fruits and berries, and, after digestion, eliminate them in a different location. Seeds also get caught on the feathers of birds and drop off as birds move from one location to another. Some birds, such as hummingbirds, feed on the nectar of flowers, and pollinate the flowers as they feed.

Habitat destruction

Many birds are threatened with extinction as the habitat they require either disappears or is degraded by pesticides and other chemical pollutants. Waterfowl populations depend on wetlands—a habitat that is disappearing rapidly as wetlands are drained for development. Deforestation of tropical rain forests also has led to some species of birds being endangered.

Illegal trade Illegal pet bird trade is increasing. Many pet birds are raised in captivity, but other exotic birds are taken from the wild in a multibillion dollar industry. In some cases illegal capture has led to the disappearance of rare birds in the wild. The little blue macaw, shown in Figure 29.21, only exists in captivity. An international trade agreement was enacted in 1975 to ensure that the buying and selling of wild animals does not endanger their survival. Currently, 160 countries participate in the agreement; however, illegal wildlife trade continues.

Mammalian Characteristics

Hair and Mammary Glands

Two characteristics that distinguish members of class Mammalia from other vertebrate animals are hair and mammary glands.

Mammary glands produce and secrete milk that nourishes developing young. Recall that if an animal has feathers, it is a bird. In a similar way, if an animal has hair, it is a mammal. As you can see on the evolutionary tree in Figure 30.1, mammals have their own branch labeled hair and mammary glands.

Functions of hair Mammals’ hair has several functions, including:

1.Insulation—One of the most important functions of hair is to insulate against the cold. Mammals benefit from having fur or hair that traps their body heat and prevents it from escaping.

2.Camouflage—The striped coat of a Bengal tiger allows it to blend into its natural habitat—the jungle.

3.Sensory devices—In some cases, hair has been modified into sensitive whiskers. Seals use the whiskers on their snouts to track prey in murky water by sensing changes in water movements when a fish is nearby.

4.Waterproofing—You might know how cool it feels when you come out of a swimming pool on a hot day. As the water evaporates from your skin, your body loses heat. Many aquatic mammals, such as the sea otter shown in Figure 30.2, have hair that keeps water from reaching their skin. This helps them maintain their body temperature.

5.Signaling—Hair can function as a signaling device. The white-tailed deer in Figure 30.3 raise their tails, the undersides of which are white, when they run so that other deer can follow.

6.Defense—Hair also can function as a defense against predators. The porcupine in Figure 30.3 has sharp quills, which are modified hairs, that are easily detached when the animal is threatened by a predator. The quills stick to and stab predators that touch the porcupine.

Structure of hair

The hair in a bushy fox tail and the hair on your head contains a tough, fibrous protein called keratin, which is a protein that also makes up nails, claws, and hooves. A coat of hair usually consists of two kinds of hair—long guard hairs that protect a dense layer of shorter insulating underhair. The air trapped in the thick underhair layer provides insulation against the cold and retains body heat.

Other Characteristics

In addition to having hair and mammary glands, mammals share other characteristics. These include a high metabolic rate, which supports endothermy, specialized teeth and digestive systems, a diaphragm to aid in respiration, a four-chambered heart, and a highly developed brain.

Endothermy Mammals are endotherms, which means they produce their body heat internally. The source of body heat is internal—the result of heat produced by a high metabolic rate. Body temperature is regulated by **internal feedback mechanisms** that send signals between the brain and sensors throughout the body.

For example, when some mammals become warm as a result of exertion or because the air is warm, sweat glands in the skin are stimulated to secrete sweat that evaporates from the skin. As the sweat evaporates, it draws heat away from the body and cools it. When body temperature lowers, sweating stops. For other mammals that do not sweat, panting cools the body. You might have seen a dog panting in the summer’s heat. During panting, water evaporates from the mouth and nose. Because mammals can regulate their body temperatures internally to maintain homeostasis, they can live in a range of ecosystems from the frigid temperatures of polar regions to the sweltering heat of deserts and the tropics.

Feeding and digestion

Maintaining an endothermic metabolism requires large amounts of energy. Mammals get the energy they need from the breakdown of food. Much of an endotherm’s daily intake of food is used to generate heat to maintain a constant body temperature.

Examine the graph in Figure 30.4. The graph shows the relationship of a mammal’s metabolic rate to its body mass. Small mammals, including shrews, bats, and mice, have a high metabolic rate relative to their body mass. As a result, these small mammals must hunt and eat food almost constantly in order to fuel their metabolisms.

Trophic categories Mammalogists divide mammals into four trophic categories based on what they eat:

1.	Insectivores, such as moles and shrews, eat insects and other small invertebrates.

2.	Herbivores, such as rabbits and deer, feed on vegetation.

3.	Carnivores, such as foxes and lions, mostly feed on herbivores.

4.	Omnivores, such as raccoons and most primates, feed on both plants and animals.

A mammal’s adaptations for finding, capturing, chewing, swallowing, and digesting food all influence the mammal’s structure and life habits. The fibers of plants are more difficult to digest and take longer than the digestion of meat. As a result, mammals that eat plant material have a larger cecum and longer digestive tracts than those that eat meat, as shown in Figure 30.5.

Ruminant herbivores Cellulose, a component of the cell walls of plants, can be a source of nutrition and energy. However, the enzymes in the digestive system of mammals cannot digest cellulose. Instead, some herbivores have bacteria in the cecum—a pouch where the small intestine meets the large intestine. Other herbivores have bacteria in their stomachs that break down the cellulose and release nutrients the animals can use. These mammals, called ruminants, have large, four-chambered stomachs. Cattle, sheep, and buffalo are all ruminants. As a ruminant feeds, plant material passes into the first and second stomach chambers. Plants are partially digested by bacteria into a material called cud. The ruminant brings the cud back up into the mouth and chews the cud for a long period of time. This further crushes the grass fibers. Once the cud is swallowed, it eventually reaches the fourth chamber of the stomach where digestion continues.

Respiration The food a mammal eats is used to maintain high energy levels. High levels of oxygen also are required to maintain a high level of metabolism. Oxygen is taken into the lungs of mammals during respiration. Although other animals, such as birds and reptiles, have lungs, mammals are the only animals that have a diaphragm. A diaphragm is a sheet of muscle located beneath the lungs that separates the chest cavity from the abdominal cavity where other organs are located. As the diaphragm contracts, it flattens, causing the chest cavity to enlarge, as shown in Figure 30.6. Once air enters the lungs, oxygen in the air moves by diffusion into blood vessels. When the diaphragm relaxes, the chest cavity becomes smaller and air is exhaled.

Circulation

Once oxygen is in the blood, vessels carry it to the heart, which pumps it out to the body. Like birds, mammals have a four-chambered heart. Also as in birds, oxygenated blood is kept entirely separate from deoxygenated blood in mammals. This is illustrated in Figure 30.7. Because most mammals are physically active and all are endotherms—they require a consistent supply of nutrients and oxygen to maintain homeostasis. Keeping oxygenated and deoxygenated blood separate makes the delivery of nutrients and oxygen more efficient.

The circulatory system of a mammal also functions to help maintain a constant internal temperature. When body temperature increases, the blood vessels near the surface of the skin dilate, or expand, and deliver more blood than usual. Heat moves from the blood to the surface of the skin by conduction. At the skin’s surface, heat is lost from the body by radiation and the evaporation of sweat. When body temperature decreases, blood vessels near the surface of the skin contract and do not deliver as much blood as usual.

This action reduces the loss of body heat.

The brain and senses Mammals have highly developed brains, especially the cerebrum. The cerebral cortex, shown in Figure 30.8, is the highly folded outer layer of the cerebrum. The foldings allow the brain to have a larger surface area for nerve connections while allowing it to still fit inside the skull. The cerebral cortex is responsible for coordinating conscious activities, memory, and the ability to learn.

Another area of the mammalian brain that is well developed is the cerebellum. The cerebellum is responsible for balance and coordinating movement. Compare the size and structure of the cerebellums of a reptile, a bird, and a mammal that are shown in Figure 30.8.

A well-developed cerebellum allows an animal to have precise motor movements and to make complex movements in three dimensional space.

Complex behavior

The mother fox referred to at the beginning of the chapter eventually will teach her young to hunt. Because mammals can learn and teach their young survival skills, they have an increased chance of survival. Mammals can carry out complex behaviors, such as learning and remembering what they have learned. Many mammals can get information about their environment and retain it. This information can then be used later. For example, mice that have had a chance to explore a habitat are able to avoid predators better than mice that have not had a chance to explore and learn about the same habitat.

Senses

The importance of the senses varies from one group of mammals to the next. In some mammals, such as humans, vision is extremely important, while hearing is most important to mammals such as bats. Bats produce high-frequency sounds that bounce off objects and return to them. In this way a bat can detect objects in its path. This process is called echolocation. If you have seen how a dog sniffs people and objects in its surroundings, you recognize the importance of the sense of smell to this mammal. In some cases, a dog’s sense of smell is one million times more sensitive than a human’s sense of smell.

Glands

A system of glands secretes a variety of fluids that helps to regulate a mammal’s internal environment. A gland is a group of cells that secrete fluid to be used elsewhere in the body. Sweat glands help maintain body temperature. Mammary glands produce and secrete milk that nourishes developing young. Milk contains water, carbohydrates in the form of the sugar lactose, fat, and protein. The proportion of these nutrients differs according to species.

Examine Table 30.1 to see the proportions of nutrients in the milk of various mammals. The proportion of nutrients is highly variable among different species of mammals. For example, fat amounts can range from one percent to 50 percent. Aquatic mammals, which use a layer of fat to help keep warm, usually have the highest percent of fat in their milk.

Scent glands produce substances that mammals use to mark their territories or attract mates. Oil glands in the skin maintain the quality of the animal’s hair and skin. Other glands produce hormones that regulate internal processes, such as growth and release of eggs from ovaries.

Reproduction In mammals, the egg is fertilized internally. In most mammals, development of the embryo takes place in the female uterus. The uterus is a saclike muscular organ in which embryos develop. In most mammals, the developing embryo is nourished by the placenta, an organ that provides food and oxygen to and removes waste from the developing young. The **gestation period** is specific for particular species. Gestation is the amount of time the young stay in the uterus before they are born. **Gestation periods** in mammals can vary—the shortest being that of the Virginian opossum which can be only 12 days. The longest **gestation period** occurs in the African elephant, which is an average of 660 days and can be as long as 760 days. In general, the larger the mammal, the longer the gestation period. After birth, the offspring of mammals drink milk for nourishment from the mother’s mammary glands.

Movement Mammals must find food, shelter, and escape from predators. They have evolved a variety of limb types that enable them to carry out these essential behaviors. Some mammals, such as coyotes and foxes, run. The fastest land mammal—the cheetah—can reach speeds as fast as 110 km/h.

Other mammals, such as kangaroos, leap. Some mammals, including dolphins, swim. Bats are the only mammals that fly. The structure of the skeletal and muscular systems in animals reflects the type of movement that an animal uses. Examine Figure 30.9, which shows the forelimbs of a mole and those of a bat. How does the structure of these limbs reflect the habitat and behavior of these animals?

Diversity of Mammals

Mammal Classification

Class Mammalia is divided into three subgroups based on methods of reproduction—monotremes, marsupials, and placental mammals.

Monotremes

The animal shown in Figure 30.10 with its duck bill and webbed feet might not look like any mammal you have seen. However, it has hair and mammary glands, which makes it a mammal. The duck-billed platypus is a monotreme that lays eggs similar to those of reptiles. Monotremes are mammals that reproduce by laying eggs. The only other living monotremes besides the duck-billed platypus are echidnas. An adult echidna and an echidna egg are shown in Figure 30.10. Both the duck-billed platypus and the echidna only live in Australia, Tasmania, and New Guinea. Besides laying eggs, other reptilian features of these mammals include similarities in bone structure in the shoulder area, lower body temperature than most mammals, and a unique mix of normal-sized chromosomes, mammal-sized chromosomes, and small, reptilelike chromosomes.

Marsupials Pouched mammals that have a very short period of development in the uterus are marsupials. Immediately following birth, the offspring crawl into a pouch made of skin and hair on the outside of the mother’s body. Within the pouch, the offspring continue development while being nourished by milk from the mother’s mammary glands. In some species of marsupials, offspring are born and crawl into the mother’s pouch only eight days after fertilization occurs.

The only North American marsupial is the opossum, shown hanging by its tail in Figure 30.11. Other marsupials include the koala, wallaby, kangaroo, and cuscus, some of which are shown in Figure 30.12. Australia and its nearby islands are home to most marsupials.

Why marsupials are limited mostly to Australia is still a subject for debate among scientists. Based on fossil evidence, marsupials originated in North America, then spread to South America and Europe while the continents still were connected as one giant land mass. From South America, marsupials moved across

Africa to Antarctica then to Australia. Then, about 200 million years ago, the continents separated due to the movement of Earth’s plates. This isolated the ancestors of today’s marsupials to Australia and nearby islands.

Australian marsupials thrived because they were isolated from competing placental mammals. In North and South America, however, placental mammals had competitive adaptive advantages. For example, placental mammals evolved highly social behavior, had more variety in food sources, and evolved greater diversity in form and function than marsupials.

Marsupials in Australia and New Guinea fill the niches occupied by placental mammals elsewhere in the world. For example, kangaroos are the grazers in Australia. They fill the niche that deer, antelope, and buffalo fill in other areas of the world.

Placental mammals Most mammals living today, including humans, are placentals. The placental mammals are mammals that have a placenta, the organ that provides food and oxygen to and removes waste from developing young. Placental mammals give birth to young that do not need further development within a pouch.

Placental mammals are represented by 18 orders. Some orders are represented only by a few species. For example, there are only two species of flying lemurs in order Dermoptera. Flying lemurs can glide through the air because a flap of skin connects their arms and legs. The aardvark, a termite-eating mammal found in Africa, is the only species in its order. Other orders, such as Rodentia, which includes squirrels and rats, have almost 2000 species. Sizes of placental mammals range from 1.5-g pygmy shrews to 100,000-kg whales, both of which are shown in Figure 30.13. Their forms range from marine dolphins with adaptations for swimming to moles adapted for subterranean life and bats equipped with wings and ultrasonic echolocation for flying in the dark.

Scientists have hypothesized several reasons why there are greater numbers and kinds of placental mammals compared to marsupials.

One hypothesis is that marsupial young must cling to their mother’s fur at birth. Limbs, therefore, are limited in their ability to evolve into structures such as the flippers and wings of some placental mammals. Another hypothesis that explains the success of placental mammals points out that the cerebral cortex of placental mammals is larger and more complex than that of marsupials. This might be due to the more stable, oxygen-rich environment they experience inside the uterus.

According to this hypothesis, this might have enabled placental mammals to develop more complex social behaviors that led to their success.

Order Insectivora

As the name implies, these mammals’ main food source is insects. The shrew, shown in Figure 30.14, is an insectivore, as are hedgehogs and moles. Members of order Insectivora usually are small and have pointed snouts that allow them to capture insects easily.

Shrews include the smallest of all mammals. Shrews can be found in almost all parts of the world and spend most of their lives underground.

Order Chiroptera There are about 925 species in order Chiroptera—all of which are bats. As mentioned previously, bats are the only mammals that truly can fly. Their wings are thin membranes supported by modified forelimbs. Bats feed on a variety of foods. Some eat insects, some eat fruit, and others feed on blood. The most common North American bat is the little brown bat which you might have seen swooping and darting at dusk to catch insects. The flying fox, shown in Figure 30.14, is the largest of all bats. It lives in tropical regions worldwide and feeds on fruit.

Order Primates Monkeys, apes, and humans are all examples of primates. Primates’ brains, with large cerebral hemispheres, are the most developed brains of all mammals. Most primates are tree dwellers. This leads scientists to hypothesize that the need to perform complex movements while in trees, such as those involved in capturing food and avoiding enemies, led to advances in the brain structure of primates.

Primate forelimbs often are adapted for grasping, and most have nails instead of claws. The golden lion tamarin in Figure 30.15 is grasping with its hands the branch on which it is sitting.

Order Xenarthra Animals in order Xenarthra either have no teeth or have simple, peglike teeth. Anteaters, like the one shown in Figure 30.16, are toothless. Anteaters have a spiny tongue and sticky saliva that allow them to easily capture ants and termites in their nests. Sloths and armadillos both have peglike teeth. Sloths mostly feed on leaves, and armadillos feed on insects. Most mammals in this order live in Central America and South America with the exception of the armadillo, which also can be found in the southern United States.

Order Rodentia These gnawing mammals in order Rodentia, called rodents, include the beaver, which is shown in Figure 30.16, rats, wood-chucks, marmots, squirrels, hamsters, and gerbils. Rodents make up nearly 40 percent of all mammalian species. Two pairs of razor-sharp incisor teeth continue to grow throughout the life of a rodent. They use their sharp teeth to gnaw through wood, seed pods, or shells to get food. The ability of rodents to invade all land habitats and their successful reproductive behavior have made them ecologically important in all terrestrial ecosystems.

Order Lagomorpha Like rodents, members of the the order Lagomorpha—rabbits, pikas, and hares—have long sharp incisors that continue to grow. Lagomorphs also have a pair of peglike incisors that grow behind the first pair. These mammals are herbivores that eat grasses, herbs, fruits, and seeds. The pika shown in Figure 30.17 lives in high latitude or high altitude environments in which the ground is covered with snow for parts of the year. The grasseaters adapt to these conditions by harvesting grass during the warm months and storing it. The pikas then eat the grass during the winter when no fresh vegetation is available.

Order Carnivora You might have a pet dog or cat. It, along with wolves, bears, seals, walruses, coyotes, skunks, otters, minks, and weasels, belongs to the order Carnivora. All of these carnivores are predators with teeth adapted to tearing f lesh. The lioness, shown in Figure 30.17, feeds on antelope, giraffes, and even crocodiles. After she captures her prey, she uses her incisors to tear off chunks of meat.

Order Proboscidea Elephants are the largest living land mammals. They have f lexible trunks adapted for gathering plants and taking in water. Two upper incisors are modified as tusks for digging up roots and tearing bark from trees. Some elephants are trained to help lift heavy objects. The elephant shown in Figure 30.18 is helping remove debris that washed ashore during a tsunami in Indonesia on December 26, 2004. Ancient mastodons and mammoths are the extinct relatives of today’s African and Asian elephants.

Order Sirenia Manatees and dugongs—members of the order Sirenia—are large, slow-moving mammals with big heads and no hind limbs. Their forelimbs are modified into flippers that aid in swimming. These animals are herbivores, feeding on seagrasses, algae, and other aquatic plants. Depending on their size, manatees can consume as much as 50 kg of vegetation per day. Sirenians can be found cruising the surface of warm tropical rivers and lagoons. Because they are so slow and prefer the surface of the water, they often are injured or killed by the propellers of speedboats. Notice the scars on the back of the manatee in Figure 30.19.

Order Perissodactyla

These hoofed mammals include horses, zebras, and rhinoceroses. Members of this order have an odd number of toes, either one or three on each foot. These mammals are herbivores and have teeth that are adapted for grinding plant material.

Perissodactyls can be found on all continents except Antarctica.

Order Artiodactyla Members of the order Artiodactyla also are hoofed mammals. They differ from perissodactyls in that they have an even number of toes, either two or four, on each limb. Deer, antelopes, cattle, sheep, pigs, goats and hippopotamuses are all artiodactyls. Many cattle, sheep, and deer have horns or antlers. Mammals in this order are herbivorous and most chew their cud.

Order Cetacea Whales, dolphins, and porpoises have front limbs modified into flippers that aid in swimming. They have no hind limbs and the tail consists of fleshy flukes. Nostrils are modified into a single or double blowhole on top of the head. Except for a few muzzle hairs, their bodies are hairless. Some whales are predators. Others, like the blue whale, have a specialized structure inside their mouths called a baleen that is used to filter plankton for food. The baleen of a humpback whale is shown in Figure 30.19.

Evolution of Mammals

The first mammals probably evolved from reptiles in the mid-**Triassic period** about 220 million years ago. A few lived side by side with the dinosaurs, but mammals did not become common until the dinosaurs disappeared. The cladogram in Figure 30.20 shows one interpretation of the evolution of mammals.

Therapsids Fossil evidence indicates that the first mammals probably arose from a group of mammal-like reptiles called therapsids. A therapsid is an extinct vertebrate with both mammalian and reptilian features. Therapsids had some characteristics of mammals today, including a pair of holes in the roof of the skull that allowed for the attachment of jaw muscles. Therapsids also had limbs positioned beneath their bodies that allowed for more efficient movement.

Evidence shows that therapsids might have been endotherms. They ate more food than their ancestors, which might have provided them with the energy to produce their own body heat. Being endothermic would have given therapsids an advantage over other ectothermic vertebrates in that they would have been able to be more active during the winter. Therapsids went extinct about 170 million years ago. One group of therapsids called cynodonts continued to evolve more mammalian characteristics, including a high metabolic rate, stronger jaws, and a structure in the mouth that allowed them to breathe while holding food or nursing. A cynodont is shown in Figure 30.21.

The Age of Mammals According to fossil evidence, the first placental mammals might have been mouse-sized animals such as Eomaia, shown in Figure 30.21. During the time when dinosaurs ruled Earth, mammals might have scurried about the forest f loor at night and remained hidden during the day. When dinosaurs disappeared at the end of the Mesozoic Era, mammals underwent extraordinary adaptations to the environment. As flowering plants flourished, new sources of nutrition and new habitats became available. Mammals had new environments to fill. For example, fast-moving herbivores and their predators evolved to fill the niches in the drier prairies. The huge expansion in mammalian diversity and numbers led some scientists to call the Cenozoic Era the “Age of Mammals.

Basic Behaviors

Animal behaviors are innate and learned behaviors that evolve by natural selection.

Behavior

You might have seen a lizard lying on a rock in the sunlight. The lizard is regulating its body temperature through its behavior. In order to raise its body temperature, the lizard absorbs the Sun’s heat. If the lizard’s body temperature starts to get too high, it will move into the shade. When it moves into or out of the sunlight, the lizard is responding to stimuli from its nervous system that its temperature is too low or too high. This is an example of behavior. Behavior is the way an animal responds to a stimulus. A stimulus is an environmental change that directly influences the activity of an organism.

Behavior can occur in response to an internal stimulus, which is a stimulus that comes from inside the body, as in the case of the lizard. Behavior also can be caused by an external stimulus—a stimulus that comes from outside the body. An external stimulus could be the smell of food, someone calling your name, or the sight of a predator.

What influences behavior? For many years, scientists asked the question about whether behavior was genetically based or based on experiences. Studies have shown that some behavior is based solely on genetics and is not influenced by experience. Other behaviors, such as a finch learning the song of its species, are known to result from a combination of genetics and environmental influences. Today, many behaviors are considered to be the result of both genes and experience. In many cases, behavior results from the interaction of genetically based behaviors and behaviors based on experience. Figure 31.1 shows some important discoveries about animal behavior.

The evolution of behavior

Two general questions are asked when studying animal behavior. The first question focuses on what triggers an animal to react to specific stimuli. For example, what triggers a male bird, like the one shown in Figure 31.2, to sing during breeding season? The answer usually is found by studying the internal biology of an animal. Scientists now know that some male birds sing during breeding season in response to the internal stimulus of increased levels of the hormone testosterone.

The second question focuses on what advantages certain behaviors provide animals. The answers to this question are tied to the evolution of behavior through natural selection. What advantage does singing during breeding season provide the male bird? Perhaps the singing helps the male bird keep other male birds away. Perhaps the singing helps the male attract a mate.

You already have learned that animals with traits giving them a competitive advantage over other animals that do not possess those traits are more likely to reproduce and pass their genes on to future generations. In the past, birds that sang tended to have more offspring than birds that did not sing. Over a number of generations, birds that sang became the only birds contributing to the population’s gene pool. The behavior has been naturally selected.

Innate Behavior

Behaviors that are genetically based and not linked to past experiences are called innate behaviors. However, you might say that all animal behaviors occur in and are influenced by the environment. Behaviors are referred to as innate, or instinct, when the same behavior commonly is observed among a large number of individuals within a population, even if the environments are different. For example, in some species, newly-hatched birds will make innate chirping sounds while opening their mouths in an upward direction when a parent lands in the nest. As part of an innate response to the chick’s open mouth, the parent will feed the chirping bird. In addition, members of a particular group of mammals typically begin to walk at the same age, depending on their species.

Therefore, walking generally is considered an innate behavior.

Fixed action patterns The goose in Figure 31.3 is exhibiting innate behavior. It is carrying out a specific set of actions, in sequence, in response to a stimulus. This behavior is a fixed action pattern. The goose is responding to the stimulus of an egg that is out of the nest. The set of actions carried out usually is the same and usually in the same order. The goose will extend its neck toward the egg and then stand up. It will then roll the egg back to the nest with a side-to-side motion of its neck with the egg held beneath its bill. The stimulus—finding that the egg is out of the nest—triggers the innate behavior, and the entire sequence of actions is carried out. Even if the egg is removed midway through the retrieval process, the goose will continue the behavior without the egg. This is the key to a fixed action pattern—the stimulus triggers an innate response that the animal does not control and is not directly influenced by environmental conditions or past experiences. Another example of a fixed action pattern is shown in Figure 31.4.

Learned Behavior

Which activities do you enjoy—playing a sport, driving a car, playing video games or a musical instrument? These activities are examples of learned behaviors. Learned behaviors result from an interaction between innate behaviors and past experiences within a particular environment. Examples of learned behavior include habituation, conditioning, imprinting, and cognitive behavior.

Habituation Sometimes, animals learn over time that a potentially important stimulus deserves little or no attention. For example, baby birds in a nest see many types of objects moving overhead. At first, they might respond to these stimuli by crouching down and staying still.

Some of the objects, such as falling leaves or members of their own species flying by, often are seen and have no positive or negative effects to the birds. Over time, the birds will stop responding to these stimuli.

This is referred to as habituation, which is a decrease in an animal’s response after repeatedly being exposed to a stimulus that has no positive or negative effects.

The horses shown in Figure 31.5 have become habituated to street and crowd noise. Habituation can be thought of as learning not to respond to a stimulus. Habituation is important to an animal’s success because it allows an animal to ignore unimportant stimuli and focus on and respond to important stimuli, such as the presence of food, a mate, or a predator. Another example of habituation is shown in Figure 31.4. Birds often become habituated to a scarecrow because they learn that it has no positive or negative effects.

Classical conditioning Ivan Pavlov, a Russian scientist who conducted experiments in the late 1890s and early 1900s, noticed that after he presented meat powder to a dog, the animal produced saliva. Later, Pavlov rang a bell each time he presented the meat powder. After repeated trials, the dog salivated when it heard the bell alone, without smelling or tasting meat powder.

Pavlov concluded that the dog related the sound of the ringing bell with the meat powder. Animal behaviorists refer to this type of learning as classical conditioning, which is illustrated in Figure 31.6.

Classical conditioning occurs when an association is made between two different kinds of stimuli. In Pavlov’s experiment, the dog learned to associate the sound of the bell with the unrelated stimulus of meat powder. The sound of the bell could produce the response of salivation.

Operant conditioning B.F. Skinner, an American psychologist, carried out experiments on operant conditioning. In operant conditioning, an animal learns to associate its response to a stimulus with a reward or a punishment. In Skinner’s experiment, a rat was placed in a box. As the rat explored the box, it accidentally would hit a lever, causing a food pellet to be released into the box. At first, the rat ignored the lever. It would eat the pellet and continue to move around the box. Eventually, the rat learned to associate pressing the lever with getting food. The animal was rewarded positively (receiving the food pellet) for its response (pressing the lever) to the stimulus (the lever).

In some cases, animals learn to associate their response with a negative reward. Monarch butterflies, which have a bright orange color pattern, are toxic to many predators. When a young blue jay eats a monarch butterfly for the first time, the bird becomes ill and vomits the butterfly. The bird quickly associates eating the butterfly with illness. In the future, the bird avoids eating monarch butterflies and other butterflies with a similar color pattern.

Operant conditioning is a more powerful, long-lasting kind of learning that dominates much of everyday learning of humans and other vertebrates. For example, animals, including humans, learn ways of finding food by exploring a variety of locations. When certain locations prove to be a good source of food, animals are positively reinforced.

Research shows that such animals are more likely to seek food the next time in the same location or locations that appear similar.

Imprinting Learning that can only occur within a specific time period in an animal’s life and is permanent is called imprinting. The time during which an animal imprints is called the sensitive period. In some animals, the sensitive period occurs immediately after birth.

Newborn offspring can form a strong bond with another animal, such as a parent, during this time. Some animals, such as whooping cranes, form a social attachment to the first object that they see after birth.

Other animals, such as salmon, imprint on the chemical composition of the water in which they are hatched. The salmon use this imprint to return to this location when it is time for them to spawn.

Evidence of the influence of genetics on imprinting comes from experiments with newly hatched birds. In nature, the first object the offspring sees most likely will be its parent. This ensures that the offspring will have a higher chance of success by being nurtured by a parent. Experiments have shown that newly hatched birds will imprint on whatever object they see first, whether it is an animal of a different species, such as a human, or an inanimate object, such as a box.

In 1999, only one flock of 180 migratory whooping cranes existed naturally. Scientists created a plan to introduce a second migratory flock of cranes to help ensure the species would not become extinct. Crane chicks were hatched in Wisconsin at the northernmost point of their migratory path. The chicks were imprinted using an ultralight plane like the one shown in Figure 31.7. Each year since 2001, a group of newly hatched chicks is imprinted by an ultralight. They follow it to the winter migration site in Florida and back to Wisconsin in the spring. In doing this, a second population of migratory cranes has been established successfully.

Cognitive behavior Thinking, reasoning, and processing information to understand complex concepts and solve problems are cognitive behaviors. Humans exhibit cognitive behaviors when they solve problems, make decisions, and plan for the future. Some experimental evidence supports the idea that other animals, such as chimpanzees and ravens, exhibit cognitive behavior. The raven shown in Figure 31.8 appears to be using problem-solving skills to reach a piece of food.

Observations made by scientists of animals in their natural habitats also seem to show examples of cognitive behavior. Chimpanzees, like the one shown in Figure 31.8, have been observed using rocks to break open nuts. This behavior suggests that the chimpanzees are thinking and using tools to solve problems. Experiments are being conducted to find out if some primates purposely deceive, or lie to, other animals in their group—another sign of cognitive behavior.

Ecological Behaviors

Types of Behaviors

All animal behaviors are somewhat ecologically based. Ecology is the study of the interactions of living things with each other and with their environment. These interactions can occur between members of the same species or between members of different species. Animals that engage in complex behaviors survive and reproduce because they have inherited genes that allow them to be successful in a particular environment.

Examine Figure 31.9, which shows two bighorn sheep fighting over a mate. Although it looks painful, the thick horns of the sheep protect them from injury when they butt heads. One of the sheep eventually will give up the contest, leaving the other the winner. What are the survival and reproductive advantages and disadvantages of this behavior? The winner is able to court and mate with a female without interference from the other male. The genes of the winner most likely will be passed on to future generations. Genes that provide adaptive advantages will increase in relative frequency according to the principles of evolution by natural selection. Genes that do not help an individual animal survive and produce offspring are likely to decrease in frequency in the gene pool of future generations. As you read about different types of behavior in this section, think about why a particular behavior might have evolved.

Competitive behaviors Competition for food, space, mates, and other resources occurs between individuals within a population. Competitive behaviors, like the example shown in Figure 31.9, allow individuals to establish dominance or control of an area or resource.

Animals that are successful at competitive behaviors are more likely to obtain resources needed for survival and reproduction. Animals usually do not critically injure or kill one another when competing for food, mates, or other resources. Types of competitive behaviors include agonistic behavior, dominance hierarchies, and territorial behavior.

Agonistic behavior

The polar bears in Figure 31.10 are engaging in behavior in which one bear will be the winner and will have control over resources such as food or potential mates. This type of threatening or combative interaction between two individuals of the same species is called agonistic behavior. Although the bears look as though they might hurt each other, agonistic behavior usually does not result in injury or death to either individual. The challenge will end when one animal eventually stops participating and leaves.

Dominance hierarchies

A hierarchy is a grouping in which objects or individual animals are ranked in order from highest to lowest. Some animals living in groups develop dominance hierarchies in which a top-ranked animal gets access to resources without conflict from other animals in the group. This ranking system helps reduce hostile behaviors among animals. These hostile behaviors would take time and energy away from finding food or a mate, or caring for offspring. Higher-ranked animals are more likely to get what they need to survive and reproduce. Female wolves, baboons, some song birds, and the chickens shown in Figure 31.11, establish dominance hierarchies.

Territorial behaviors Many animals establish a territory. A territory is a specific area that contains resources, such as food or potential mates, that an individual continually defends against other individuals of the same species. The size of territories varies widely, depending on the animal and the particular environment. Territorial behaviors are attempts to adopt and control a physical area against the other animals of the same species. Territorial behaviors include verbal signals, such as the singing of birds or chattering of squirrels, as well as chemical signals, such as a male cheetah’s urine. Birds, such as the North American gannets shown in Figure 31.12, that gather in large colonies to breed engage in territorial behavior by fighting and jabbing to maintain space in the nesting colony. Territories usually are defended by males in order to increase their chance of obtaining adequate food, mates, and places to rear their offspring.

Foraging behaviors

Finding and eating food are examples of foraging behaviors. These behaviors have obvious advantages for animals. Foraging successfully means obtaining needed nutrients, while avoiding predators and poisonous foods. Foraging involves a trade-off between a food’s energy content and the cost of finding, pursuing, and eating it. Scientists theorize that natural selection favors individual animals whose foraging behaviors use the least amount of energy to obtain the maximum amount of energy possible. These are the animals that will be most able to reproduce successfully and pass genes on to future generations.

Migratory behaviors

Some animals, such as birds and grazing mammals, move long distances seasonally to new locations. These animals are engaging in migratory behaviors that increase their chances of survival. Land animals, like the wildebeest and zebra in East Africa, migrate almost continuously as different areas receive the rain needed for their food sources to grow. Each fall in North America, about two-thirds of bird species fly south to areas such as South America where food is available during the North American winter. The birds fly north in the spring to areas where they feed and breed during the summer.

How do the snow geese, shown in Figure 31.13, and other birds know which direction to fly, sometimes over thousands of kilometers each year, with seemingly little navigational information? Recent studies show that the first migration of some birds is guided innately by both the position of the stars and Earth’s magnetic field. Future migrations are influenced by external cues that the bird learns while flying that help it navigate more precisely.

Biological rhythms Many animals, including humans, repeat behaviors in a rhythmic cycle. A circadian rhythm is a cycle, such as sleeping and waking, that occurs daily. Other biological cycles are seasonal or yearly. These cycles are influenced by environmental factors such as temperature changes, the increase or decrease of daylight hours, and the availability of food and water. All of these factors act as cues for animals to move into another phase of the cycle.

The daily cycle of sleeping and waking is influenced by external cues in animals. However, experiments have shown that many animals have an internal clock, often referred to as a **biological clock**, that maintains the daily rhythm of the sleep/wake cycle of about 24 hours. The graphs in Figure 31.14 show the results of an experiment in which the activity level of nocturnal squirrels was monitored under two sets of conditions for 23 days—one in which a squirrel was exposed to a light cycle of 12 hours of light followed by 12 hours of darkness, and one in which a squirrel was kept in continual darkness. The biological clock of the squirrel maintained a sleep/wake cycle of 24 hours and

21 minutes in the absence of an external light and dark cycle. Controlled experiments show that the human biological clock has a cycle length of about 24 hours and 11 minutes.

Communication Behaviors

Dogs bark, birds chirp, wolves howl, and lions growl and snarl. These are all examples of animal communication. Wolves howl to communicate information over long distances, including letting other wolves know their location, attracting mates, and signaling the presence of a predator. Such communication behaviors are critical to the survival and reproductive success of animals. Animals have several types of communication behaviors.

Pheromones Recall from Chapter 26 that some animals communicate by spreading highly specific chemicals called pheromones. These chemicals are specific to species, ensuring that individuals within a population receive important information. An advantage of species-specific pheromones is that predators cannot detect them, unlike other more noticeable communication behaviors, such as barks or howls. Pheromones often are used to relay messages between males and females about reproduction. For example, female silk moths produce a pheromone that is used to attract male moths for mating. Pheromones also can be used to relay messages of alarm in response to a predator attack. The cheetah in Figure 31.15 is leaving its scent to communicate with other cheetahs.

Auditory communication If you ever have spent an evening outside in a park or a forest, you might have heard many animals using auditory communication. Howls, hoots, barks, and chirps are just a few of the sounds you might have heard. Auditory communication permits animals to send and receive sound messages that move faster than chemical messages. Male crickets, frogs, birds, and the howler monkey shown in Figure 31.15 communicate information about mating, predators, and territory to others in the population using auditory communication. Humans use language to communicate complex information. Language is a form of auditory communication in which animals use vocal organs to produce groups of sounds that have shared meanings.

Courting and Nurturing Behaviors

Certain behaviors displayed by animals are directly related to the reproductive success of an individual animal. Attracting a mate and caring for offspring are important aspects of reproductive success.

Courting behaviors An animal engages in courting behaviors in order to attract a mate. An example of courting behavior is shown in Figure 31.16. The male frigate bird has inflated its bright red throat sac and is displaying it to attract the attention of female frigate birds.

Courtship signals, whether they are a display of brightly colored feathers or a series of movements or sounds, are species specific. This is important in ensuring the reproductive success of a species. Courting behavior can last for minutes or months, depending on the species.

Selecting a male often is the female’s role in the courtship process. Females often choose to mate with males that appear relatively larger and healthier than others. Thus, males with desired traits have a competitive advantage over other males and typically have a better chance of mating and successfully producing offspring.

Nurturing behaviors

When parents provide care to their offspring in the early stages of development, they are engaging in nurturing behaviors. This includes providing food, protection, and skills needed for survival.

Nurturing behaviors cost parents energy because of the extra work required to sustain offspring until they can take care of themselves. Animal species that spend time nurturing young often produce fewer offspring than animals that do not nurture. Reproductive energy can be spent producing millions of eggs, with very little if any energy spent on nurturing.

For example, a female cod can produce as many as nine million eggs during a single reproductive period. Only a small percentage of these eggs will survive. In contrast to the cod, animals that nurture, such as primates, produce far fewer eggs and offspring. A female chimpanzee, like the one shown in Figure 31.17, will give birth to one baby which she will nurse for up to three years. The baby will stay with the mother for five to seven years. In this case, more energy is spent nurturing young after birth to ensure they successfully reach a reproductive age. Although each of the reproductive strategies uses energy differently, each can be selected for because ultimately each results in there being at least one offspring that reaches maturity to successfully reproduce.

Cooperative Behaviors

Cooperative behaviors can exist in groups of same-species animals. Cooperative behaviors, such as those of the honeybee described in Chapter 26, can benefit all members of the group. However, some examples of cooperative behavior lead to an individual animal performing altruistic, or self-sacrificing, behaviors.

Altruistic behavior

Sometimes an animal will perform an action that benefits another individual at a cost to itself. This type of behavior is called altruistic behavior. One example of altruistic behavior occurs in naked mole rats. Naked mole rats live underground in colonies. Each colony consists of one female that reproduces, called the queen, several males with whom the queen mates, called kings, and between 75–250 other males and females that do not reproduce. The nonreproductive members of the colony forage for food and care for and protect the queen, kings, and newborn offspring. Figure 31.18 shows the nonreproductive individuals of a colony surrounding a queen and her offspring.

By huddling around her, they are helping to keep the offspring warm.

Scientists have wondered what the advantage of altruistic behavior would be to an individual animal. Why should behavior that might hurt the individual animal ever be selected?

Kin selection

One theory that has been presented to explain some types of altruistic behavior is **kin selection**. According to the idea of **kin selection**, altruistic behavior evolves because it increases the number of copies of a gene that is common to a population. It does not matter which individual passes the gene on to future generations. In the case of the mole rats, scientists have discovered through DNA analysis that all of the individuals in a colony of naked mole rats are closely related.

The nonreproductive members of the colony will not pass on their genes. However, genes that are similar, if not identical, to their own will be passed on by the queen. As the nonreproductive members work to protect the queen and bring her food, they are ensuring that genes similar to their own will be passed on to future generations.

The Integumentary System

The Structure of Skin

The integumentary system is the organ system that covers and protects the body. Skin is the main organ of the integumentary system and is composed of four types of tissues: epithelial tissue, connective tissue, muscle tissue, and nerve tissue. Epithelial tissue covers body surfaces, and connective tissue provides support and protection. Muscle tissue is involved in body movement. Nerve tissue forms the body’s communication network. You will learn more about muscle tissue in Section 32.3, and you will learn about nerve tissue in Chapter 33.

The epidermis Refer to Figure 32.1, which illustrates the two main layers of skin as seen through a microscope. The outer superficial layer of skin is the epidermis. The epidermis consists of epithelial cells and is about 10 to 30 cells thick, or about as thick as this page. The outer layers of epidermal cells contain keratin, a protein which waterproofs and protects the cells and tissues that lie underneath. These dead, outer cells are constantly shed. Figure 32.2 shows that some of the dust in a house actually is dead skin cells. As much as an entire layer of skin cells can be lost each month.

The inner layer of the epidermis contains cells that continually are dividing by mitosis to replace cells that are lost or die. Some cells in the inner layer of the epidermis provide protection from harmful ultraviolet radiation by making a pigment called melanin. Melanin is a pigment that absorbs light energy, which protects deeper cells from the damaging effects of ultraviolet rays of sunlight. The amount of melanin that is produced also influences the color of a person’s skin. A suntan results when melanin is produced in response to exposure to the ultraviolet radiation in sunlight.

The dermis

Directly beneath the epidermis is the dermis, the second layer of skin. The thickness of the dermis varies but usually is 15–40 times thicker than the epidermis. The dermis consists of connective tissue, a type of tissue that prevents the skin from tearing and also enables the skin to return to its normal state after being stretched.

This layer contains other structures including nerve cells, muscle fibers, sweat glands, oil glands, and hair follicles. Beneath the dermis is the subcutaneous layer, a layer of connective tissue that stores fat and helps the body retain heat.

Hair and nails Hair, fingernails, and toenails also are parts of the integumentary system. Both hair and nails contain keratin and develop from epithelial cells. Hair cells grow out of narrow cavities in the dermis called hair follicles. Cells at the base of a hair follicle divide and push cells away from the follicle, causing hair to grow.

Hair follicles usually have sebaceous or oil glands associated with them, as shown in Figure 32.3. Sebaceous glands lubricate skin and hair. When glands produce too much oil, the follicles can become blocked. The blockage can close the opening of a follicle, causing a white-head, blackhead, or acne—an inflammation of the sebaceous glands.

Fingernails and toenails grow from specialized epithelial cells at the base of each nail. As cells at the base of a nail divide, older dead cells are compacted and pushed out. Nails grow about 0.5 to 1.2 mm per day. You might have heard that nails and hair continue to grow for several days after death. This is a myth; cells surrounding the nail and hair cells dehydrate causing the cells to shrink and pull away from nails and hair. This makes both appear longer.

Functions of the Integumentary System

Skin serves several important functions including regulation of body temperature, production of vitamin D, protection, and perception of one’s surroundings.

Temperature regulation

What happens when a person is working outside on a hot summer day? In order to regulate body temperature, the person sweats. As sweat evaporates it absorbs body heat, thereby cooling the body. What happens to skin when a person gets cold or frightened? “Goose bumps” are caused by the contraction of muscle cells in the dermis. In other mammals, when these muscles contract, the hair (fur) stands on end.

Notice the frightened cat in Figure 32.4. The cat appears larger, perhaps as a way to scare off enemies. This also is a mechanism for trapping air, which insulates or warms the mammal. Humans do not have as much hair as most other mammals, but “goose bumps” are caused by the same type of muscles that make a cat’s fur stand on end. Humans rely on fat in the subcutaneous layer instead of hair to keep warm.

Vitamin production Skin also responds to exposure to ultraviolet light rays from the Sun by producing vitamin D. Vitamin D increases absorption of calcium into the bloodstream and is essential for proper bone formation. Many food products are now fortified with vitamin D.

Protection and senses

Intact skin prevents the entry of micro-organisms and other foreign substances. Skin helps maintain body temperature by preventing excessive water loss. Melanin in the skin protects against ultraviolet rays. Information about changes in the environment, such as pain, pressure, and temperature changes, is relayed to the brain.

Damage to the Skin

Skin has remarkable abilities to repair itself. Without a repair mechanism, the body would be subject to invasion by microbes through breaks in the skin.

Cuts and scrapes

Sometimes, as in the case of a minor scrape, only the epidermis is injured. Cells deep in the epidermis divide to replace the lost or injured cells. When the injury is deep, blood vessels might be injured, resulting in bleeding. Blood f lows out of the wound and a clot is formed. Blood clots form a scab to close the wound, and cells beneath the scab multiply and fill in the wound. At the same time, infection-fighting white blood cells will help get rid of any bacteria that might have entered the wound.

Effects of the Sun and burns

As people age, the elasticity of their skin decreases and they start to get wrinkles. Exposure to ultraviolet rays from the Sun might accelerate this process and can result in burning of the skin.

Burns, whether caused by the Sun, heat, or chemicals, usually are classified according to their severity. The types of burns are summarized in Table 32.1. First-degree burns generally are mild and involve only cells in the epidermis. A burn that blisters or leaves a scar is a second-degree burn and involves damage to both the epidermis and dermis. Third-degree burns are the most severe. Muscle tissue and nerve cells in both the epidermis and dermis might be destroyed, and skin function is lost. Healthy skin might have to be transplanted from another place on the body in order to restore the protective layer of the body.

Skin cancer Exposure to ultraviolet radiation, whether it is from the Sun or from artificial sources such as tanning beds, is recognized as an important risk factor for the development of skin cancer. Ultraviolet radiation can damage the DNA in skin cells, causing those cells to grow and divide uncontrollably. When this happens, skin cancer results.

Refer to Figure 32.5 to see some warning signs of skin cancer.

Skin cancer is the most common cancer in the United States. There are two main categories of skin cancer: melanoma and nonmelanoma. Melanoma begins in melanocytes, the cells that produce the pigment melanin. Melanoma is the deadliest form of skin cancer. Melanoma can spread to internal organs and the lymphatic system. It is estimated that one person dies from melanoma every hour in the United States. Teens are at greater risk for melanoma because as they grow, their skin cells divide more rapidly than they will when they reach adulthood.

Anyone can get skin cancer. However, individuals with light skin, light-colored eyes, light hair color, and a tendency to burn or freckle are at the greatest risk. Everyone should try to avoid prolonged exposure to the Sun, especially between 10 A.M. and 4 P.M. when the Sun’s rays are the strongest. Other preventative measures include wearing protective clothing or sunscreen with a Sun Protection Factor (SPF) of at least 15.

The Skeletal System

Structure of the Skeletal System

Notice all the bones in the adult skeleton pictured in Figure 32.6. If you counted them, you would find there are 206 bones. The human skeleton consists of two divisions—the axial skeleton and the appendicular skeleton. The axial skeleton includes the skull, the vertebral column, the ribs, and the sternum. The appendicular skeleton includes the bones of the shoulders, arms, hands, hips, legs, and feet.

Compact and spongy bone Bone is a connective tissue that has many shapes and sizes. Bones are classified as long, short, flat, or irregular. Refer back to Figure 32.6. Arm and leg bones are examples of long bones, and wrist bones are examples of short bones. Flat bones make up the skull. Facial bones and vertebrae are irregular bones.

Regardless of their shape, all bones have the same basic structure.

The outer layers of all bones are composed of compact bone.

Compact bone is dense and strong; it provides strength and protection. Running the length of compact bones are tubelike structures called osteons, or Haversian systems, which contain blood vessels and nerves. The blood vessels provide oxygen and nutrients to osteocytes—living bone cells. The centers of bones can differ greatly, as illustrated in Figure 32.7.

As the name suggests, spongy bone is less dense and has many cavities that contain bone marrow. Spongy bone is found in the center of short or flat bones and at the end of long bones. Spongy bone is surrounded by compact bone and does not contain Haversian systems.

There are two types of bone marrow—red and yellow. Red and white blood cells and platelets are produced in red bone marrow. Red bone marrow is found in the humerus bone of the arm, the femur bone of the leg, the sternum and ribs, the vertebrae, and the pelvis. The cavities of an infant’s bones are composed of red marrow. Children’s bones have more red marrow than adult bones. Yellow bone marrow, found in many other bones, consists of stored fat.

Formation of bone

The skeletons of embryos are composed of cartilage. During fetal development, cells in fetal cartilage develop into bone-forming cells called osteoblasts. The formation of bone from osteoblasts is called ossification. Except for the tip of the nose, outer ears, discs between vertebrae, and the lining of movable joints, the human adult skeleton is all bone. Osteoblasts also are the cells responsible for the growth and repair of bones.

Remodeling of bone Bones constantly are being remodeled, which involves replacing old cells with new cells. This process is continual throughout life and is important in the growth of an individual. Cells called osteoclasts break down bone cells, which are then replaced by new bone tissue. Bone growth involves several factors, including nutrition and physical exercise. For example, a person with insufficient calcium can develop a condition known as osteoporosis that results in weak, fragile bones that break easily.

Repair of bone Fractures are very common bone injuries. When a bone breaks but does not come through the skin, it is a simple fracture. A compound fracture is one in which the bone protrudes through the skin. A stress fracture is a thin crack in the bone. When a bone is fractured, repair begins immediately.

Refer to Figure 32.8, which illustrates the steps in the repair of a broken bone.

Fracture Upon injury, endorphins, chemicals produced in the brain and sometimes called “the body’s natural painkillers,” flood the area of the injury to reduce the amount of pain temporarily. The injured area quickly becomes inflamed, or swollen. The swelling can last for two or three weeks.

Within about eight hours, a blood clot forms between the broken ends of the bone and new bone begins to form. First, a soft callus, or mass, of cartilage forms at the location of the break. This tissue is weak, so the broken bone must remain in place.

Callus formation

About three weeks later, osteoblasts form a callus made of spongy bone that surrounds the fracture. The spongy bone is then replaced by compact bone. Osteoclasts remove the spongy bone while osteoblasts produce stronger, compact bone.

Splints, casts, and sometimes traction can ensure that the broken bone remains in place until new bone tissue has formed. Broken fingers often are kept in place by being taped to an adjacent finger.

Remodeling Bones require different amounts of time to heal. Age, nutrition, location, and severity of the break are all factors. A lack of calcium in a person’s diet will slow down bone repair. Bones of younger people usually heal more quickly than bones of older people. For example, a fracture might take only four to six weeks to be repaired in a toddler, but it might take six months in an adult.

Joints

Joints occur where two or more bones meet. Except for the joints in the skull, they can be classified according to the movement they allow and the shapes of their parts. Table 32.2 identifies five kinds of joints— ball-and-socket, pivot, hinge, gliding, and sutures. Study Table 32.2 to identify the type of movement that each kind of joint allows and also the bones involved in each example.

Not all joints are movable. The joints between some skull bones are fixed. At birth, however, skull bones are not all fused together. They become fused by the time a baby is about three months old. Gliding joints, like those found in the hand, have limited movement. Other joints, such as the hinge joint of the elbow and the pivot joint in the lower arm, allow back-and-forth movement and twisting. The ball-and-socket joints of the hips and shoulders have the widest range of motion.

The bones of joints are held together by ligaments. Ligaments are tough bands of connective tissue that attach one bone to another. You will learn more about ligaments and tendons, which attach muscle to bone, in the following section.

Osteoarthritis

The ends of bones in movable joints, such as the knee, are covered by cartilage, which serves as a cushion and allows smooth movement of the joint. Osteoarthritis is a painful condition that affects joints and results from the deterioration of the cartilage. It is a very common condition in knees and hips and also affects the neck and back. Osteoarthritis affects about ten percent of Americans and the frequency increases with age. A young person who has a joint injury is at risk to develop osteoarthritis later in life.

Rheumatoid arthritis Rheumatoid arthritis is another form of arthritis that affects joints. Rheumatoid arthritis is not the result of cartilage deterioration or of wear and tear on the joint.

Affected joints lose strength and function and are inflamed, swollen, and painful. Fingers can look deformed, as illustrated in Figure 32.9.

Bursitis Shoulders and knees also have fluid-filled sacs called bursae that surround these joints. Bursae decrease friction and act as a cushion between bones and tendons. Bursitis is an inflamation of the bursae and can reduce joint movement and cause pain and swelling. Perhaps you have heard of “tennis elbow” which is a form of bursitis. Treatment usually involves resting the joint involved.

Sprains A sprain involves damage to the ligaments that hold joints together. It is caused when a joint is twisted or overstretched and usually causes the joint to swell and be tender and painful.

Functions of the Skeletal System

You might think that the only purpose of a skeleton is to serve as a framework to support the body. The bones of the legs, pelvis, and the vertebral column hold up the body. The mandible supports the teeth, and almost all bones support muscles. Many soft organs are directly or indirectly supported by nearby bones.

The skeletal system serves other functions besides support, as shown in Table 32.3. The skull protects the brain, vertebrae protect the spinal cord, and the rib cage protects the heart, lungs, and other organs.

The outer layers of bone tissue also protect the bone marrow found inside bones. In addition to forming red blood cells and white blood cells, red bone marrow forms platelets, which are involved in blood clotting. Red blood cells are produced at the rate of over two million per second.

Until a person reaches about seven years of age, all bone marrow is red bone marrow. Then, fat tissue replaces some red marrow and gives the marrow a yellowish appearance, which gives it its name. Fat is an important source of energy.

Bones are reservoirs for the storage of minerals such as calcium and phosphorus. When blood calcium levels are too low, calcium is released from bones. When blood calcium levels are high, excess calcium is stored in bone tissue. In this way, the skeletal system helps to maintain homeostasis.

Bones that have muscles attached to them allow movement of the body. For example, as muscles pull on the bones of the arms and legs, they cause movement. Muscles that are attached to your ribs allow you to breathe normally.

The Muscular System

Three Types of Muscle

A muscle consists of groups of fibers or muscle cells that are bound together. When the word muscle is used, many people immediately think of skeletal muscle. Examine Figure 32.10 to see that there are three types of muscle: skeletal muscle, smooth muscle, and cardiac muscle. Muscles are classified according to their structure and function.

Smooth muscle

Many hollow internal organs such as the stomach, intestines, bladder, and uterus are lined with smooth muscle. Smooth muscle is called involuntary muscle because it cannot be controlled consciously. For example, food moves through the digestive tract because of the action of smooth muscles that line the esophagus, stomach, and small and large intestines. Under a microscope, smooth muscle does not appear striated, or striped, and each cell has one nucleus.

Cardiac muscle

The involuntary muscle present only in the heart is called cardiac muscle. Cardiac muscle cells are arranged in a network, or web, that allows the heart muscle to contract efficiently and rhythmically. This arrangement gives strength to the heart. Cardiac muscle is striped, or striated, with light and dark bands of cells with many nuclei. Cells usually have one nucleus and are connected by gap junctions.

Skeletal muscle Most of the muscles in the body are skeletal muscles. Skeletal muscles are muscles attached to bones by tendons and when tightened, or contracted, cause movement. Skeletal muscles are voluntary muscles that are consciously controlled to move bones. Tendons, which are tough bands of connective tissue, connect muscles to bones. Under a microscope, skeletal muscles also appear striated.

Skeletal Muscle Contraction

Most skeletal muscles are arranged in opposing, or antagonistic pairs. Refer to Figure 32.11, which illustrates muscles that you use to raise your arm and opposing muscles that you use to lower your arm. Skeletal muscle is arranged into fibers, which are fused muscle cells. Muscle fibers consist of many smaller units called myofibrils. Myofibrils consist of even smaller units, myosin and actin, which are protein filaments.

Myofibrils are arranged in sections called sarcomeres. A sarcomere is the functional unit of a muscle and the part of the muscle that contracts as illustrated in Figure 32.12. The striations of skeletal muscles are a result of the sarcomeres, which run Z line to Z line. Z lines are where actin filaments attach within a myofibril. The overlap of actin and myosin filaments results in a dark band called the A band. The M line consists of only myosin filaments. The arrangement of the components of a sarcomere causes a muscle to shorten and then relax.

Sliding filament theory Figure 32.12 also illustrates the **sliding filament theory**. This theory states that once a nerve signal reaches a muscle, the actin filaments slide toward one another, causing the muscle to contract. Notice that the myosin filaments do not move.

There are many skeletal muscles involved in a simple motion, such as turning this page.

When the nerve impulse reaches the muscle, calcium is released into the myofibrils. Calcium causes the myosin and actin to attach to each other. The actin filaments then are pulled toward the center of the sarcomere, which causes muscle contraction. ATP produced in the mitochondria is necessary for this step of muscle contraction. When the muscle relaxes, the filaments slide back into their original positions.

Energy for muscle contraction

All muscle cells metabolize aerobically and anaerobically. When sufficient oxygen is available, aerobic cellular respiration occurs in muscle cells.

Recall from Chapter 8 that cellular respiration process provides ATP for energy. After a period of intense exercise, muscles might not get enough oxygen to sustain cellular respiration, limiting the amount of ATP that is available. Muscles, like those of the athlete in Figure 32.13, then must rely on the anaerobic process of lactic acid fermentation for energy.

During exercise, lactic acid builds up in muscle cells, causing fatigue. Excess lactic acid enters the bloodstream and this stimulates rapid breathing. After resting for a short time, adequate amounts of oxygen are restored and lactic acid is broken down.

You probably have seen a dead animal along the side of the road. When an animal dies, rigor mortis sets in. Rigor mortis is a state of prolonged muscular contraction. ATP is required to pump the calcium back out of the myofibrils, which causes the muscles to relax. In rigor mortis, the dead animal cannot produce ATP, so the calcium remains in the myofibrils and the muscle remains contracted. After 24 h, cells and tissues begin degrading and the muscle fibers cannot remain contracted.

Skeletal Muscle Strength

Many people do not develop the physiques of champion bodybuilders, no matter how often they work out in the weight room. A person might be the fastest sprinter on the track team, but quickly becomes fatigued in a long-distance race. What might be the reason for these differences?

The reason in both cases is the ratio of slow-twitch muscle fibers to fast-twitch muscle fibers. Both slow-twitch and fast-twitch fibers are present in every person’s muscles.

Slow-twitch muscles Muscles vary in the speeds at which they contract. Slow-twitch muscles contract more slowly than fast-twitch muscle fibers. Slow-twitch muscle fibers have more endurance than fast-twitch muscle fibers. The body of the triathlete in Figure 32.14 has many slow-twitch fibers. These kinds of muscle fibers function well in long distance running or swimming because they resist fatigue more than fast-twitch muscle fibers do.

Slow-twitch muscle fibers have many mitochondria needed for cellular respiration. They also contain myoglobin, a respiratory molecule that stores oxygen and serves as an oxygen reserve. Myoglobin causes the muscles to have a dark appearance. Exercise increases the number of mitochondria in these fibers but the overall increase in the size of the muscle is minimal.

Fast-twitch muscles

Fast-twitch muscle fibers fatigue easily but provide great strength for rapid, short movements. Fast-twitch muscle fibers are adapted for strength. They function well in exercises requiring short bursts of energy such as sprinting or weightlifting, as illustrated in Figure 32.14.

Fast-twitch fibers are lighter in color because they lack myoglobin. Because they have fewer mitochondria, they rely on anaerobic metabolism, which causes a buildup of lactic acid. This causes these muscles to fatigue easily. Exercise increases the number of myofibrils in a muscle, thereby increasing the diameter of the entire muscle.

Most skeletal muscles contain a mixture of slow-twitch and fast-twitch muscles fibers. The ratio of these fibers is determined genetically. If there is a very high ratio of slow-twitch to fast-twitch, a person might be a champion cross-country runner. Champion sprinters have a high proportion of fast-twitch muscle fibers. Most people are somewhere in between.

Structure of the Nervous System

Neurons

Electricity and chemistry were both involved as your brain received the message that you stubbed your toe. Neurons are specialized cells that help you gather information about your environment, interpret the information, and react to it. Neurons make up an enormous communication network in your body called the nervous system. You will learn more about how this communication works electrically and chemically later in the chapter.

Figure 33.1 shows that a neuron consists of three main regions: the dendrites, a cell body, and an axon. Dendrites receive signals called impulses from other neurons and conduct the impulses to the cell body. A single neuron might have many dendrites. The nucleus of the neuron and many of the cell organelles are found in the cell body.

Lastly, an axon carries the nerve impulse from the cell body to other neurons and muscles.

There are three kinds of neurons: sensory neurons, interneurons, and motor neurons. Sensory neurons send impulses from receptors in the skin and sense organs to the brain and spinal cord. Sensory neurons signal interneurons, which are found in the spinal cord and brain. Interneurons carry the impulse to motor neurons, which carry impulses away from the brain and spinal cord to a gland or muscle, which results in a response.

Refer to Figure 33.2 to follow the path of an impulse for a simple, involuntary reflex. The nerve impulse completes what is called a reflex arc. A reflex arc is a nerve pathway that consists of a sensory neuron, an interneuron, and a motor neuron. Notice that the brain is not involved. A reflex arc is a basic structure of the nervous system.

A Nerve Impulse

A nerve impulse is an electrical charge traveling the length of a neuron. An impulse results from a stimulus, such as a touch or perhaps a loud bang that causes you to jump.

A neuron at rest Figure 33.3 shows a neuron at rest—it is not conducting an impulse. Notice that there are more sodium ions (Na+) outside the cell than inside the cell. The reverse is true for potassium ions (K+)—there are more potassium ions inside the cell than outside the cell.

Recall from Chapter 7 that ions tend to diffuse across the plasma membrane from an area of high concentration of ions to an area of low concentration of ions. Proteins found in the plasma membrane work to counteract the diffusion of the sodium ions and potassium ions.

These proteins, called the sodium-potassium pump, actively transport sodium ions out of the cell and potassium ions into the cell.

For every two potassium ions pumped into a neuron, three sodium ions are pumped out. This maintains an unequal distribution of positively charged ions, resulting in a positive charge outside the neuron and a negatively-charged cytoplasm inside the neuron.

An action potential

Another name for a nerve impulse is an action potential. The minimum stimulus to cause an action potential to be produced is a threshold. However, a stronger stimulus does not generate a stronger action potential. Action potentials are described as being “all or nothing,” meaning a nerve impulse is either strong enough to travel along the neuron or it is not strong enough.

When a stimulus reaches threshold, channels in the plasma membrane open. Sodium ions rapidly move into the cytoplasm of the neuron through these channels, causing a temporary reversal in electrical charges. The inside of the cell now has a positive charge, which causes other channels to open. Potassium ions leave the cell through these channels, restoring a positive charge outside the cell. Figure 33.4 shows that this change in charge moves like a wave along the length of the axon.

Speed of an action potential

The speed of an action potential varies. Many axons have a covering of a lipid called myelin, which forms an insulating layer called a sheath around the axon. The myelin sheath has many gaps, called nodes, along the length of the axon, as shown in Figure 33.5. Sodium ions and potassium ions cannot diffuse through myelin, but they can reach the plasma membrane at these nodes. This allows the action potential to jump from node to node, greatly increasing the speed of the impulse as it travels the length of the axon.

In the human body, there are neurons that have myelin, and neurons that do not have myelin. Neurons with myelin carry impulses that are associated with sharp pain; neurons that lack myelin carry impulses associated with dull, throbbing pain. The action potentials in these neurons travel much more slowly than they do in neurons with myelin.

When you stubbed your toe, which kind of neurons were involved?

The synapse

A small gap exists between the axon of one neuron and the dendrite of another neuron. This gap is called a synapse. When an action potential reaches the end of an axon, small sacs called vesicles carrying neurotransmitters fuse with the plasma membrane and release a neurotransmitter by exocytosis. When a motor neuron synapses with a muscle cell, as illustrated in Figure 33.6, the released neurotransmitter crosses the synapse and causes a muscle to contract.

A neurotransmitter is a chemical that diffuses across a synapse and binds to receptors on the dendrite of a neighboring neuron. This causes channels to open on the neighboring cell and creates a new action potential.

There are over 25 known neurotransmitters. Once a neurotransmitter has been released into a synapse, it does not remain there for long. Depending on the neurotransmitter, it might simply diffuse away from the synapse, or enzymes might break it down. Some neurotransmitters are recycled and used again. Figure 33.7 shows that a single neuron can communicate with many other neurons.

Organization of the Nervous System

The Central Nervous System

The nervous system consists of two major divisions: the central nervous system (CNS) and the peripheral nervous system (PNS). The brain and the spinal cord make up the central nervous system. The peripheral nervous system consists of the sensory neurons and motor neurons that carry information to and from the CNS.

The CNS is made up mostly of interneurons. Its function is to coordinate all of the body’s activities. The CNS relays messages, processes information, and analyzes responses. Sensory neurons carry information about the environment to the spinal cord. Interneurons in the spinal cord might respond via a reflex arc, or they might relay this information to the brain, where it is processed in at least two ways.

Some brain neurons send a message by way of the spinal cord to motor neurons, and the body responds appropriately. Other neurons in the brain might store the information to be recalled later.

The brain Over 100 billion neurons are found in the brain. Because the brain maintains homeostasis and is involved with almost all of the body’s activities, it is sometimes called the control center of the body. Refer to Figure 33.8 to learn about important events that have led to understanding of the functions of the brain.

Refer to Figure 33.9. The cerebrum is the largest part of the brain and is divided into two halves called hemispheres. The two hemispheres are not independent of each other—they are connected by a bundle of nerves. The cerebrum carries out thought processes involved with learning, memory, language, speech, voluntary body movements, and sensory perception. Most of these higher thought processes occur near the surface of the brain. The folds and grooves on the surface of the cerebrum, as shown in Figure 33.9, increase the surface area and allow more complicated thought processes.

The cerebellum controls balance, posture, and coordination, and is located at the back of the brain. The cerebellum controls the smooth and coordinated movement of skeletal muscles and also is involved with motor skills, such as playing the piano or riding a bike.

The brain stem connects the brain to the spinal cord and is made up of two regions called the medulla oblongata and the pons. The medulla oblongata relays signals between the brain and the spinal cord. It also helps control breathing rate, heart rate, and blood pressure. The pons relays signals between the cerebrum and the cerebellum.

The pons also helps control the rate of breathing. Have you ever felt a gagging sensation when your doctor put a tongue depressor in your mouth? The interneurons that form a reflex center for swallowing, vomiting, coughing, and sneezing are located in the medulla oblongata.

Located between the brain stem and the cerebrum, the hypothalamus is essential for maintaining homeostasis. The hypothalamus regulates body temperature, thirst, appetite, and water balance. It also partially regulates blood pressure, sleep, aggression, fear, and sexual behavior. It is about the size of a fingernail and performs more functions than any other brain structure of its size.

The spinal cord

The spinal cord is a nerve column that extends from the brain to the lower back. It is protected by the vertebrae.

Spinal nerves extend from the spinal cord to parts of the body and connect them to the central nervous system. Reflexes are processed in the spinal cord.

The Peripheral Nervous System

When you hear the word nerve, you might initially think of a neuron. However, a nerve is actually a bundle of axons. Many nerves contain both sensory and motor neurons. For example, there are 12 cranial nerves that lead to and from the brain and 31 spinal nerves (and their branches), as shown in Figure 33.10, that lead to and from the spinal cord. You could think of these as two-way streets.

Information travels to and from the brain through these sensory and motor neurons.

Refer to Figure 33.11 as you read about the peripheral nervous system. This system includes all neurons that are not part of the central nervous system, including sensory neurons and motor neurons. Neurons in the peripheral nervous system can be classified further as being either part of the somatic nervous system or part of the autonomic nervous system.

The somatic nervous system Nerves in the somatic nervous system relay information from external sensory receptors to the central nervous system, and motor nerves relay information from the central nervous system to skeletal muscles. Usually, this is voluntary. However, not all reactions of the central nervous system are voluntary. Some responses are the result of a reflex, which is a fast response to a change in the environment. Reflexes do not require conscious thought and are involuntary. Most signals in reflexes go only to the spinal cord, and not to the brain. Remember the example of stubbing your toe? Refer back to Figure 33.2 and note that the illustrated reflex is part of the somatic nervous system.

The autonomic nervous system Remember the last time you had a scary dream? You might have awakened and realized that your heart was pounding. This type of reaction is the result of the action of the autonomic nervous system. The autonomic nervous system carries impulses from the central nervous system to the heart and other internal organs. The body responds involuntarily, not under conscious control. The autonomic nervous system is important in two different kinds of situations. When you have a bad nightmare or perhaps find yourself in a scary situation, your body responds with what is known as a fight-or-flight response. When everything is calm, your body rests and digests.

There are two branches of the autonomic nervous system and they act together. The sympathetic nervous system is most active in times of emergency or stress when the heart rate and breathing rate increase. The parasympathetic nervous system is most active when the body is relaxed. It counterbalances the effects of the sympathetic system and restores the body to a resting state after a stressful experience. Table 33.1 compares and contrasts the two systems.

Both the sympathetic and parasympathetic systems relay impulses to the same organs, but the overall response depends on the intensities of the opposing signals.

The Senses

Taste and Smell

Specialized neurons in your body called sensory receptors enable you to taste, smell, hear, see, and touch, and to detect motion and temperature.

The senses of taste and smell are stimulated by chemicals and often function together. Specialized receptors located high in the nose respond to chemicals in the air and send the information to the olfactory bulb in the brain. Taste buds are areas of specialized chemical receptors on the tongue that detect the tastes of sweet, sour, salty, and bitter. These receptors detect the different combinations of chemicals in food and send this information to another part of the brain. The receptors associated with taste and smell are shown in Figure 33.12. Signals from these receptors work together to create a combined effect in the brain. Try eating while holding your nose. You will find that your food loses much of its flavor.

Sight

Figure 33.13 shows the path of light as it travels through the eye. Light first enters the eye through a transparent, yet durable, layer of cells called the cornea. The cornea helps to focus the light through an opening called the pupil. The size of the pupil is regulated by muscles in the iris—the colored part of the eye. Behind the iris is the lens, which inverts the image and projects it onto the retina. The image travels through the vitreous humor, which is a colorless, gelatinlike liquid between the lens and the retina. The retina contains numerous receptor cells called rods and cones. Rods are light-sensitive cells that are excited by low levels of light. Cones function in bright light and provide information about color to the brain. These receptors send action potentials to the brain via the neurons in the optic nerve. The brain then interprets the specific combination of signals received from the retina and forms a visual image.

Hearing and Balance

Hearing and balance are the two major functions of the ear. From a soft sound, like whispering, to a loud sound, such as a crowd cheering at a sporting event, specialized receptors in the ear can detect both the volume and the highness and lowness of sounds. Canals in the inner ear are responsible for your sense of balance, or equilibrium.

Hearing Vibrations called sound waves cause particles in the air to vibrate. Figure 33.14 illustrates the path of sound waves as they travel through the ear.

Sound waves enter the auditory, or ear, canal and cause a membrane, called the eardrum or tympanum, at the end of the ear canal to vibrate. These vibrations travel through three bones in the middle ear—the malleus (also called the hammer), the incus (anvil), and stapes (stirrup). As the stapes vibrates, it causes the oval window—a membrane that separates the middle ear from the inner ear—to move back and forth. In the inner ear, a snail-shaped structure called the cochlea is filled with fluid and lined with tiny hair cells. Vibrations cause the fluid inside the cochlea to move like a wave against the hair cells. The hairs cells respond by generating nerve impulses in the auditory nerve and transmitting them to the brain.

Balance The inner ear also contains organs for balance, including three semicircular canals. Semicircular canals transmit information about body position and balance to the brain. The three canals are positioned at right angles to one another and, like the cochlea, they are fluid-filled and lined with hair cells. When the position of your head changes, fluid moves through the canals. This causes the hair cells to bend, which in turn sends nerve impulses to the brain. The brain then is able to determine your position and whether your body is still or in motion.

Touch

Many types of sensory receptors that respond to temperature, pressure, and pain are found in the epidermis and dermis layers of the skin.

Figure 33.15 illustrates the different types of receptors—some that respond to light touches, and others that respond to heavy pressure.

Distribution of receptors is not uniform in all areas of the body. The tips of the fingers have many receptors that detect light touch. The soles of the feet have many receptors that respond to heavy pressure. Pain receptors are very simple, consisting of free nerve endings that are found in all tissues of the body except the brain. The brain constantly receives signals from these receptors and responds appropriately.

Effects of Drugs

How Drugs Work

A drug is a substance, natural or artificial, that alters the function of the body. Some types of drugs are shown in Table 33.2. Antibiotics are prescription drugs that fight bacterial infections. Cocaine and marijuana are illegal drugs. There are over-the-counter drugs for pain relief. There are other common substances that people often do not think of as drugs, such as caffeine, nicotine, and alcohol. However, they are classified as drugs.

Drugs can affect a person’s body in many different ways. Not all drugs affect the nervous system. However, those that cause changes in the nervous system work in one or more of the following ways:

•a drug can cause an increase in the amount of a neurotransmitter that is released into a synapse

•a drug can block a receptor site on a dendrite, preventing a neurotransmitter from binding

•a drug can prevent a neurotransmitter from leaving a synapse

•a drug can imitate a neurotransmitter

Many drugs that affect the nervous system influence the level of a neurotransmitter called dopamine. Dopamine is a neurotransmitter found in the brain that is involved with the control of body movements and other functions. Dopamine also is strongly involved with feelings of pleasure or reward. Dopamine normally is removed from a synapse by being reabsorbed by the neuron that released it, as illustrated in Figure 33.16.

Classes of Commonly Abused Drugs

Drug abuse does not necessarily involve the use of illegal drugs. Any use of a drug for reasons other than legitimate medical purposes, whether deliberate or unintentional, can be considered abuse of that drug.

Stimulants Drugs that increase alertness and physical activity are stimulants. Figure 33.17 indicates some common stimulants.

Nicotine Nicotine in cigarette and cigar smoke increases the amount of dopamine released into a synapse. Nicotine also constricts blood vessels, raising blood pressure and causing the heart to work harder than normal. Cigarette smoking has been linked to about 90 percent of all lung cancer cases.

Caffeine

The most commonly used, and often abused, stimulant probably is caffeine. Caffeine is found in coffee, tea, some soft drinks, and even some foods like chocolate. Caffeine works by binding to adenosine receptors on neurons in the brain. Adenosine slows down neural activity, causing drowsiness. When caffeine binds to these receptors, it has the opposite effect. It makes users feel awake and alert. Caffeine also temporarily raises epinephrine (adrenaline) levels in the body, giving a quick burst of energy that soon wears off.

Depressants Drugs that tend to slow down the central nervous system are depressants. These drugs can lower blood pressure, interrupt breathing, and slow the heart rate. Depressants can relieve anxiety, but they also can cause the noticeable effect of sedation.

Alcohol Alcohol is a depressant. It affects the central nervous system and is one of the most widely abused drugs in the world today. It is produced by the fermentation of grains and fruits. Alcohol is known to affect at least four different neurotransmitters, resulting in a feeling of relaxation and sluggishness. Short-term alcohol use impairs judgment, coordination, and reaction time. Long-term effects of alcohol abuse include a reduction in brain mass, liver damage, stomach and intestinal ulcers, and high blood pressure. Consumption of alcohol during pregnancy is the number-one cause of fetal alcohol syndrome, which can result in damage to a baby’s brain and nervous system.

Inhalants Inhalants are chemical fumes that have an influence on the nervous system. Exposure to inhalants might be accidental due to poor ventilation. Inhalants generally work by acting as a depressant on the central nervous system. Inhalants might produce a short-term effect of intoxication, as well as nausea and vomiting. Death can occur. Long- term exposure to inhalants can cause memory loss, hearing loss, vision problems, peripheral nerve damage, and brain damage.

Illegal drugs Amphetamines and cocaine both increase dopamine levels and both prevent dopamine from being reabsorbed, so it remains in the synapse. This ultimately increases the levels of dopamine in the brain, which results in a feeling of pleasure and well-being.

The use of cocaine and amphetamines has short-term and long-term effects. Cocaine abuse might result in disturbances in heart rhythm, heart attacks, chest pain, respiratory failure, strokes, seizures, headaches, abdominal pain, and nausea. Abuse of amphetamines might result in rapid heart rate, irregular heartbeat, increased blood pressure, and irreversible, stroke-producing damage to small blood vessels in the brain. Elevated body temperature, called hyperthermia, and convulsions can result from an amphetamine or cocaine overdose, and if not treated immediately, can result in death. Abusers also can experience episodes of violent behavior, paranoia, anxiety, confusion, and insomnia. It can take a year or longer for users of methamphetamine—the strongest type of amphetamine—to recover after quitting the drug.

Marijuana is the most-used illegal drug in the United States. The active chemical in marijuana is tetrahydrocannabinol, or THC. Smoking marijuana quickly gets THC into the bloodstream where it is carried to the brain. THC binds to receptors on neurons in the brain, which produces the effect of intense pleasure. These receptors are found on neurons associated with many body activities. Short-term effects of marijuana use include problems with memory and learning, loss of coordination, increased heart rate, anxiety, paranoia, and panic attacks. Long-term smoking of marijuana might also cause lung cancer.

Tolerance and Addiction

Drug tolerance can lead to addiction. Tolerance occurs when a person needs more and more of the same drug to get the same effect. The dosage increases because the body becomes less responsive to the drug.

Addiction The psychological and/or physiological dependence on a drug is addiction. Current research suggests that the neurotransmitter dopamine is involved with most types of physiological addiction. Recall that dopamine normally is removed from a synapse as it is reabsorbed by the neuron that released it. However, certain drugs prevent that reabsorption, which results in an increase of dopamine in the brain.

A person addicted to drugs derives pleasure from increased levels of dopamine and builds up a tolerance to the drug. As a result, the person takes more of the drug. When people who are addicted try to quit, the levels of dopamine decrease, making it difficult to resist going back to the drug.

Addictions can also be psychological. An individual with a psychological dependence on a drug such as marijuana has a strong desire to use the drug for emotional reasons. Both physiological and psychological dependence can affect emotional and physical health. Both types are strong, making it difficult to quit a drug.

Treatment People who are either psychologically or physiologically dependent on a drug experience serious withdrawal symptoms without it. It is very difficult for dependent users to quit on their own. They might be able to quit for short periods of time, but they are likely to use the drug again. Medical supervision is necessary when people who are psychologically and physiologically dependent on a drug try to quit.

The best way to avoid an addiction is never to use drugs in the first place, even when pressured to use them. Encourage people who abuse drugs to seek treatment for drug dependency. Physicians, nurses, counselors, clergy, and social workers are trained to direct people to the resources they need to get help, as illustrated in Figure 33.18.

Circulatory System

Functions of the Circulatory System

Cells must have oxygen and nutrients and must get rid of waste products. This exchange is done by the circulatory system—the body’s transport system. The circulatory system consists of blood, the heart, blood vessels, and the lymphatic system. Blood carries important substances to all parts of the body. The heart pumps blood through a vast network of tubes inside your body called blood vessels. The lymphatic system is considered part of the circulatory and immune systems. You will learn about the lymphatic system in Chapter 37. All of these components work together to maintain homeostasis in the body.

The circulatory system transports many important substances, such as oxygen and nutrients. The blood also carries disease-fighting materials produced by the immune system. The blood contains cell fragments and proteins for blood clotting. Finally, the circulatory system distributes heat throughout the body to help regulate body temperature.

Blood Vessels

Highways have lanes that separate traffic. They also have access ramps that take vehicles to and from roads. Similarly, the body also has a network of channels—the blood vessels. Blood vessels circulate blood throughout the body and help keep the blood flowing to and from the heart. The fact that there are different kinds of blood vessels was first observed by Greek physician Praxagoras, as noted in Figure 34.1. The three major blood vessels are arteries, capillaries, and veins, as illustrated in Figure 34.2.

Arteries Oxygen-rich blood, or oxygenated blood, is carried away from the heart in large blood vessels called arteries. These strong, thick-walled vessels are elastic and durable. They are capable of withstanding high pressures exerted by blood as it is pumped by the heart.

As shown in Figure 34.2, arteries are composed of three layers: an outer layer of connective tissue, a middle layer of smooth muscle, and an inner layer of endothelial tissue. The endothelial layer of the artery is thicker than that of the other blood vessels. The endothelial layer of arteries needs to be thicker because blood is under higher pressure when it is pumped from the heart into the arteries.

Capillaries Arteries branch through the body like the branches of a tree, becoming smaller in diameter as they grow farther away from the main vessel. The smallest branches are capillaries. Capillaries are microscopic blood vessels where the exchange of important substances and wastes occurs. Capillary walls are only one cell thick, as illustrated in Figure 34.2. This permits easy exchange of materials between the blood and body cells, through the process of diffusion. These tubes are so small that red blood cells move single-file through these vessels.

The diameter of blood vessels changes in response to the needs of the body. For example, when you are exercising, muscle capillaries will expand, or dilate. This increases blood flow to working muscles, which brings more oxygen to cells and removes extra wastes from cells.

Veins After blood moves through the tiny capillaries, it enters the largest blood vessels, called veins. Veins carry oxygen-poor blood, or deoxygenated blood, back to the heart. The endothelial walls of veins are much thinner than the walls of arteries. Pressure of the blood decreases when the blood flows through capillaries before it enters the veins. By the time blood f lows into the veins, the heart’s original pushing force has less effect on making the blood move. So how does the blood keep moving? Because many veins are located near skeletal muscles, the contraction of these muscles helps keep the blood moving. Larger veins in your body also have f laps of tissue called valves, such as the one in Figure 34.3, that prevent blood from f lowing backward. Lastly, breathing movements exert a squeezing pressure against veins in the chest, forcing blood back to the heart.

The Heart

The heart is a muscular organ that is about as large as your fist and is located at the center of your chest. This hollow organ pumps blood throughout the body. The heart performs two pumping functions at the same time. The heart pumps oxygenated blood to the body, and it pumps deoxygenated blood to the lungs.

Structure of the heart Recall from Chapter 32 that the heart is made of cardiac muscle. It is capable of conducting electrical impulses for muscular contractions. The heart is divided into four compartments called chambers, as illustrated in Figure 34.4. The two chambers in the top half of the heart—the right atrium and the left atrium—receive blood returning to the heart.

Below the atria are the right and left ventricles, which pump blood away from the heart. A strong muscular wall separates the left side of the heart from the right side of the heart. The right and left atria have thinner muscular walls and do less work than the ventricles. Notice the valves in Figure 34.4 that separate the atria from the ventricles and keep blood f lowing in one direction. Valves also are found in between each ventricle and the large blood vessels that carry blood away from the heart, such as the aortic valve shown in a closed position in Figure 34.4.

How the heart beats

The heart acts in two main phases. In the first phase, the atria fill with blood. The atria contract, filling the ventricles with blood. In the second phase, the ventricles contract to pump blood out of the heart, into the lungs, and forward into the body.

The heart works in a regular rhythm. A group of cells found in the right atrium, called the pacemaker or sinoatrial (SA) node, send out signals that tell the heart muscle to contract. The SA node receives internal stimuli about the body’s oxygen needs, and then responds by adjusting the heart rate. The signal initiated by the

SA node causes both atria to contract. This signal then travels to another area in the heart called the atrioventricular (AV) node, illustrated in Figure 34.5. This signal travels through fibers, causing both ventricles to contract. This two-step contraction makes up one complete heartbeat.

Pulse The heart pulses about 70 times each minute. If you touch the inside of your wrist just below your thumb, you can feel a pulse in the artery in your wrist rise and fall. This pulse is the alternating expansion and relaxation of the artery wall caused by contraction of the left ventricle. The number of times the artery pulses is the number of times your heart beats.

Blood pressure Blood pressure is a measure of how much pressure is exerted against the vessel walls by the blood. Blood-pressure readings can provide information about the condition of arteries. The contraction of the heart, or systole, causes the blood pressure to rise to its highest point, and the relaxation of the heart, or diastole brings the pressure down to its lowest point. A normal blood-pressure reading for a healthy adult is a reading below 120 (systolic pressure)/80 (diastolic pressure).

Blood flow in the body

If you follow the flow of blood shown in Figure 34.6, you might notice that it flows in two loops. First, the blood travels from the heart to the lungs and back to the heart. Then, the blood is pumped in another loop from the heart through the body and back. The right side of the heart pumps deoxygenated blood to the lungs, and the left side of the heart pumps oxygenated blood to the rest of the body.

To the lungs and back When blood from the body flows into the right atrium, it has a low concentration of oxygen but a high concentration of carbon dioxide. This deoxygenated blood is dark red. The blood flows from the right atrium into the right ventricle and is pumped into the pulmonary arteries that lead to the lungs, as shown in Figure 34.6.

Eventually, blood flows into capillaries in the lungs that are in close contact with the air that comes into the lungs. The air in the lungs has a greater concentration of oxygen than the blood in the capillaries does, so oxygen diffuses from the lungs into the blood. At the same time, carbon dioxide diffuses in the opposite direction—from the blood into the air space in the lungs. Oxygenated blood, which is now bright red, flows to the left atrium of the heart to be pumped out to the body.

To the body and back The left atrium fills with oxygenated blood from the lungs, beginning the second loop of the figure eight. As shown in Figure 34.6, the blood then moves from the left atrium into the left ventricle. The left ventricle pumps the blood into the largest artery in the body called the aorta. Eventually, blood flows into the capillaries that branch throughout the body. Importantly, the capillaries are in close contact with body cells. Oxygen is released from the blood into the body cells by diffusion, and carbon dioxide moves from the cells to the blood by diffusion. The deoxygenated blood then flows back to the right atrium through veins.

Blood Components

Blood is the fluid of life because it transports important substances throughout the body and contains living cells. Blood is made up of a liquid medium called plasma, red and white blood cells, and cell fragments called platelets.

Plasma The clear, yellowish fluid portion of blood is the plasma. Over fifty percent of blood is plasma. Ninety percent of plasma is water and nearly ten percent is dissolved materials. Plasma carries the breakdown products of digested food, such as glucose and fats. Plasma also transports vitamins, minerals, and chemical messengers such as hormones that signal body activities, such as the uptake of glucose by the cells. In addition, waste products from the cells are carried away by plasma.

There are three groups of plasma proteins that give plasma its yellow color. One group helps to regulate the amount of water in blood. The second group, produced by white blood cells, helps fight disease.

The third group helps to form blood clots.

Red blood cells

The red blood cells carry oxygen to all of the body’s cells. Red blood cells resemble discs with pinched-in centers, as shown in Figure 34.7. Recall that red blood cells develop in the marrow—the center portion of large bones. Red blood cells have no nuclei and only live for about 120 days.

Red blood cells mostly consist of an iron-containing protein called hemoglobin. Hemoglobin chemically binds with oxygen molecules and carries oxygen to the body’s cells. Some carbon dioxide is carried by the hemoglobin, but most carbon dioxide is carried in plasma.

Platelets

Have you ever cut your finger? You probably have noticed that in a short while, the blood flowing from the cut slows and then stops as a blood clot forms a scab. Platelets are cell fragments that play an important part in forming blood clots.

When a blood vessel is cut, platelets collect and stick to the vessel at the site of the wound. The platelets then release chemicals that produce a protein called fibrin. Fibrin is a protein, also known as a clotting factor, which weaves a network of fibers across the cut that traps blood platelets and red blood cells, as shown in Figure 34.8. As more and more platelets and blood cells get trapped, a blood clot forms.

Blood is composed of liquid plasma, red blood cells (dimpled discs), white blood cells (irregularly shaped cells), and platelets (flat fragments).

White blood cells

The body’s disease fighters are the white blood cells. Like red blood cells, white blood cells are produced in bone marrow. Some white blood cells recognize disease-causing organisms, such as bacteria, and alert the body that it has been invaded. Other white blood cells produce chemicals to fight the invaders. Still, other white blood cells surround and kill the invaders. You will learn more about white blood cells in Chapter 37.

White blood cells are different from red blood cells in important ways. First, many white blood cells move from the marrow to other sites in the body to mature. Unlike red blood cells, there are fewer white blood cells—only about one white blood cell for every 500 to 1000 red blood cells. Also, white blood cells have nuclei. Finally, most white blood cells live for months or years.

Blood Types

How do you know what type of blood you have? There are marker molecules attached to red blood cells. These markers determine blood type.

ABO blood groups There are four types of blood—A, B, AB, and

O. If your blood type is A, you have A markers on your blood cells. If your blood type is B, you have B markers on your blood cells. People with blood type AB have both A and B markers. If your blood type is O, you do not have A or B markers.

Importance of blood type

If you ever need a blood transfusion, you only will be able to receive certain blood types, as shown in

Table 34.1. This is because plasma contains proteins called antibodies that recognize red blood cells with foreign markers and cause those cells to clump together. For example, if you have blood type B, your blood contains antibodies that cause cells with A markers to clump. If you received a transfusion of type-A blood, your clumping proteins would make the type-A cells clump together. Clumping of blood cells can be dangerous because it can block blood flow.

Rh factor Another marker found on the surface of red blood cells is called the Rh factor. The Rh marker can cause a problem when an Rh-negative person, someone without the Rh factor, receives a transfusion of Rh-positive blood that has the Rh marker. This can result in clumping of red blood cells because Rh-negative blood contains Rh antibodies against Rh-positive cells.

The Rh factor can cause complications during some pregnancies. If the Rh-positive blood of a fetus mixes with the mother’s Rh-negative blood, the mother will make anti-Rh antibodies. If the mother becomes pregnant again, these antibodies can cross the placenta and can destroy red blood cells if the fetus has Rh-positive blood. Rh-negative mothers are given a substance that prevents the production of Rh antibodies in the blood, so these problems can be avoided.

Circulatory System Disorders

Several disorders of the blood vessels, heart, and brain are associated with the circulatory system. Blood clots and other matter, such as fat deposits, can reduce the flow of oxygen-rich and nutrient-rich blood traveling through arteries. Physicians refer to the condition of blocked arteries as atherosclerosis. Signs of clogged arteries include high blood pressure and high cholesterol levels. When blood flow is reduced or blocked, the heart must work even harder to pump blood, and vessels can burst.

Atherosclerosis can lead to a heart attack or stroke. Heart attacks occur when blood does not reach the heart muscle. This can result in damage to the heart, or could even result in death if not treated. Strokes occur when clots form in blood vessels supplying oxygen to the brain. This can lead to ruptured blood vessels and internal bleeding, as shown in Figure 34.9.

Respiratory System

The Importance of Respiration

Your body’s cells require oxygen. Recall from Chapter 8 that oxygen and glucose are used by cells to produce energy-rich ATP molecules needed to maintain cellular metabolism. This process is called cellular respiration. In addition to releasing energy, cellular respiration releases carbon dioxide and water.

Breathing and respiration

The function of the respiratory system is to sustain cellular respiration by supplying oxygen to body cells and removing carbon dioxide waste from cells. The respiratory system can be divided into two processes: breathing and respiration. First, air must enter the body through breathing. Breathing is the mechanical movement of air into and out of your lungs. Figure 34.10 illustrates air being released from the lungs into the air. Second, gases are exchanged in the body. External respiration is the exchange of gases between the atmosphere and the blood, which occurs in the lungs. Internal respiration is the exchange of gases between the blood and the body’s cells.

The Path of Air

The respiratory system is made up of the nasal passages, pharynx, larynx, epiglottis, trachea, lungs, bronchi, bronchioles, alveoli, and diaphragm. Air travels from the outside environment to the lungs where it passes through the alveoli, as shown in Figure 34.11.

First, air enters your mouth or nose. Hairs in the nose filter out dust and other large particles in the air. Hairlike structures called cilia, shown in Figure 34.12, also line the nasal passages, as well as other respiratory tubes. Cilia trap foreign particles from the air and sweep them toward the throat so that they do not enter the lungs. Mucous membranes beneath the cilia in the nasal passages, also shown in Figure 34.12, warm and moisten the air while trapping foreign materials.

Filtered air then passes through the upper throat called the pharynx. A flap of tissue called the epiglottis, which covers the opening to the larynx, prevents food particles from entering the respiratory tubes. The epiglottis allows air to pass from the larynx to a long tube in the chest cavity called the trachea, or windpipe. The trachea branches into two large tubes, called bronchi, which lead to the lungs. The lungs are the largest organs in the respiratory system, and gas exchange takes place in the lungs. Each bronchus branches into smaller tubes called bronchioles. Each of these small tubes continues to branch into even smaller passageways, each of which ends in an individual air sac called an alveolus. Each alveolus has a thin wall— only one cell thick—and is surrounded by very thin capillaries.

Gas exchange in the lungs Air travels to individual alveoli where oxygen diffuses across the moist, thin walls into capillaries and then into red blood cells, as shown in Figure 34.11. The oxygen is then transported by the blood to be released to tissue cells in the body during internal respiration. Meanwhile, carbon dioxide moves in the opposite direction in the alveoli. Carbon dioxide in the blood crosses capillary walls, and then diffuses into the alveoli to be returned to the atmosphere during external respiration.

Breathing

The brain directs the rate of breathing by responding to internal stimuli that indicate how much oxygen the body needs. When the concentration of carbon dioxide in the blood is high, the breathing rate increases because cells need more oxygen.

Inhalation is the act of taking air into the lungs. During inhalation, as shown in Figure 34.13, the diaphragm contracts. This causes the chest cavity to expand as the diaphragm moves down, allowing air to move into the lungs. During exhalation, the diaphragm relaxes and returns to its normal resting position. This reduces the size of the chest cavity as the diaphragm moves up. Air naturally flows out from the greater pressure of the lungs. Follow Figure 34.14 to learn how circulation and respiration work together to supply the needed oxygen and to get rid of carbon dioxide.

Excretory System

Parts of the Excretory System

The body collects wastes, such as toxins, waste products, and carbon dioxide, that result from metabolism in the body. The excretory system removes these toxins and wastes from the body. In addition, the excretory system regulates the amount of fluid and salts in the body, and maintains the pH of the blood. All of these functions help to maintain homeostasis.

The components that make up the excretory system include the lungs, skin, and kidneys, as illustrated in Figure 34.15. The lungs primarily excrete carbon dioxide. The skin primarily excretes water and salts contained in sweat. The kidneys, however, are the major excretory organ in the body.

The Kidneys

As shown in Figure 34.16, the kidneys are bean-shaped organs that filter out wastes, water, and salts from the blood. The kidneys are divided into two distinct regions, also illustrated in Figure 34.16. The outer portion is called the renal cortex and the inner region is called the renal medulla. Each of these regions contains microscopic tubes and blood vessels. In the center of the kidney is a region called the renal pelvis, where the body’s filters are found. Follow Figure 34.16 as you read about how the kidneys function.

Nephron filtration

Each kidney contains approximately one million filtering units called nephrons. Blood enters each nephron through a long tube that is surrounded by a ball of capillaries called the glomerulus. The glomerulus is surrounded by a structure called the Bowman’s capsule.

The renal artery transports nutrients and wastes to the kidney and branches into smaller and smaller blood vessels, eventually reaching the tiny capillaries in the glomerulus. The walls of the capillaries are very thin and the blood is under great pressure. As a result, water and substances dissolved in the water, such as the nitrogenous waste product called urea, are pushed through the capillary walls into the Bowman’s capsule. Larger molecules, such as red blood cells and proteins, remain in the bloodstream.

Reabsorption and the formation of urine

The filtrate collected in the Bowman’s capsule flows through the renal tubule that consists of the convoluted tubule, the loop of Henle, and the collecting tubule, as illustrated in Figure 34.16. Much of the lost water and useful substances, such as glucose and minerals, are reabsorbed back into the capillaries surrounding the renal tubule. This process is called reabsorption. At the same time, excess fluids and toxic substances in the capillaries are passed to the collecting tubules. This waste product is called urine. Urine leaves the kidney through ducts called the ureters, shown in Figure 34.16. Urine is then stored in the urinary bladder and exits the body through the urethra.

Each kidney filters about 180 L of blood each day in adults, but produces only about 1.5 L of urine. The process of filtration and reabsorption from the blood requires large amounts of energy.

Although kidneys account for only one percent of body weight, they use 20 to 25 percent of the body’s oxygen intake for their internal energy requirements.

The kidney can help maintain a normal pH in the blood by adjusting the acid-base balance. Recall from Chapter 6 that low pH results when there is an abundance of H+. When the blood pH is too low, the kidney can increase pH levels in the body by excreting hydrogen (H+) ions and ammonia into the renal tubules. The kidney can decrease pH levels by reabsorbing buffers such as bicarbonate (HCO —) and sodium (Na+) ions. Because biological processes normally require pH between 6.5 and 7.5, the kidneys help to maintain homeostasis by keeping pH levels within the normal range.

Kidney Disorders

Sometimes kidney function can be inhibited or impaired by infections or disorders. When kidney function is impaired, the body cannot rid itself of wastes and homeostasis might be disrupted.

Infections Symptoms of a kidney infection include fever, chills, and mid-to low-back pain. Kidney infections often start as urinary bladder infections that spread to the kidney. Obstructions in the kidney also can cause an infection. If the infection is not treated, the kidneys can become scarred and their function might be permanently impaired.

Antibiotics usually are effective in treating a bacterial infection.

Nephritis Another common kidney problem is nephritis, which often is due to inflammation or painful swelling of some of the glomeruli, as listed in Table 34.3. This occurs for many reasons, such as when large particles in the bloodstream become lodged in some of the glomeruli. Symptoms of this condition include blood in the urine, swelling of body tissues, and protein in the urine. If this condition does not improve on its own, the patient may need a special diet or prescription drugs to treat the infection.

Kidney stones Kidney stones are another type of kidney disorder, as listed in Table 34.3 and shown in Figure 34.17. A kidney stone is a crystallized solid, such as calcium compounds, that forms in the kidney. Small stones can pass out of the body in urine; this can be quite painful. Larger stones often are broken into small pieces by ultrasonic sound waves. The smaller stones then can pass out of the body. In some cases, surgery might be required to remove large stones.

Kidneys also can be damaged by other diseases present in the body. Diabetes and high blood pressure are the two most common reasons for reduced kidney function and kidney failure. In addition, kidneys can be damaged by prescription and illegal drug use.

Kidney Treatments

A large percentage of kidney function can be lost before kidney failure becomes apparent. If kidney problems are left untreated, the buildup of waste products in the body can lead to seizures, a comatose state, or death. However, modern medicine offers two possible treatments for reduced kidney function or complete kidney failure.

Dialysis Dialysis is a procedure in which an artificial kidney machine filters out wastes and toxins from a patient’s blood. There are two different types of dialysis, and one is illustrated in Figure 34.18. Blood is passed through a machine that temporarily filters and cleanses the blood. The filtered blood is then returned to the patient’s body. The procedure lasts about three to four hours and requires three session per week.

In the second type of dialysis, the membrane lining the abdomen acts as an artificial kidney. The abdominal cavity is injected with a special fluid through a small tube attached to the body. The patient’s fluid, that contains wastes from the blood, is drained. This procedure is performed on a daily schedule for thirty to forty minutes.

Kidney transplant

A kidney transplant is the surgical placement of a healthy kidney from another person into the patient’s body. Kidney transplants have shown increasing success in recent years. However, there is a limited supply of donated kidneys. The number of patients waiting for kidney transplants far exceeds the organs available for transplant.

The major complication of a transplant is possible rejection of the donated organ. Rejection is prevented with medications such as steroids and cyclosporine. Cyclosporine is a drug given to transplant recipients to help prevent organ rejection. Many transplant patients also need blood pressure medication and other drugs to prevent infections.

The Digestive System

Functions of the Digestive System

There are three main functions of the digestive system. The digestive system ingests food, breaks it down so nutrients can be absorbed, and eliminates what cannot be digested. Refer to Figure 35.1 and Figure 35.2 as you learn about the structure and function of the digestive system.

Ingestion On Friday night, you and your friends go out for pizza. When the pizza arrives at the table, you bite into a slice and begin to chew. Why do you need to chew each bite?

Mechanical digestion involves chewing food to break it down into smaller pieces. It also includes the action of smooth muscles in the stomach and small intestine that churn the food. Once a piece of pizza is chewed into smaller pieces, a digestive enzyme in saliva begins to break down carbohydrates. Chemical digestion is the action of enzymes—discussed in Chapter 6—in breaking down large molecules into smaller molecules that then can be absorbed by cells. Amylase, an enzyme found in saliva, begins the process of chemical digestion by breaking down starches into sugars.

Esophagus

When the tongue pushes chewed food to the back of the mouth, the swallowing reflex is stimulated. The food is forced by the action of the tongue into the upper portion of the esophagus. The esophagus is a muscular tube that connects the pharynx, or throat, to the stomach, illustrated in Figure 35.2. The wall of the esophagus is lined with smooth muscles that contract rhythmically to move the food through the digestive system in a process called peristalsis. Peristalsis continues throughout the digestive tract. Even if a person were upside down, food would still move toward the stomach.

When a person swallows, the small plate of cartilage called the epiglottis covers the trachea. If this opening is not closed, food can enter the trachea and cause a person to choke. The body responds to this by initiating the coughing reflex in an attempt to expel the food to keep the food from entering the lungs.

Stomach When food leaves the esophagus, it passes through a circular muscle called a sphincter, and then it moves into the stomach. The sphincter between the esophagus and stomach is the cardiac sphincter. The walls of the stomach are composed of three overlapping layers of smooth muscle that are involved with mechanical digestion. As the muscles contract, they further break down the food and mix it with the secretions of glands that line the inner wall of the stomach.

Recall from Chapter 6 that pH is a measure of a solution’s acidity. The environment inside the stomach is very acidic. Stomach glands, called gastric glands, secrete an acidic solution, which lowers the pH in the stomach to a pH of about 2. This is about the same acidity as lemon juice. If the sphincter in the upper portion of the stomach allows any leakage, some of this acid might move back into the esophagus, causing what is commonly known as heartburn.

The acidic environment in the stomach is favorable to the action of pepsin, an enzyme involved in the process of the chemical digestion of proteins. Cells in the lining of the stomach secrete mucus to help prevent damage from pepsin and the acidic environment. Although most absorption occurs in the small intestine, some substances, such as alcohol and aspirin, are absorbed by cells that line the stomach. While empty, the capacity of the stomach is about 50 mL. When full, it can expand to 2–4 L.

The muscular walls of the stomach contract and push food farther along the digestive tract. The consistency of the food resembles tomato soup as it passes through the pyloric sphincter at the lower end of the stomach into the small intestine. Figure 35.3 illustrates peristalsis in the small intestine.

Small intestine

The small intestine is approximately 7 m in length and is the longest part of the digestive tract. It is called small because it has a diameter of 2.5 cm compared to the 6.5 cm diameter of the large intestine. The smooth muscles in the wall of the small intestine continue the process of mechanical digestion and push the food farther through the digestive tract by peristalsis.

The completion of chemical digestion in the small intestine depends on three accessory organs—the pancreas, liver, and gallbladder, illustrated in Figure 35.4. The pancreas serves two main functions. One is to produce enzymes that digest carbohydrates, proteins, and fats. The other is to produce hormones, which will be discussed later in this chapter. The pancreas secretes an alkaline fluid to raise the pH in the small intestine to slightly above 7, which creates a favorable environment for the action of intestinal enzymes.

The liver is the largest internal organ of the body and produces bile, which helps to break down fats. About 1 L of bile is produced every day, and excess bile is stored in the gallbladder to be released into the small intestine when needed. Figure 35.5 shows gallstones, which are cholesterol crystals that can form in the gallbladder.

Chemical digestion is completed and most of the nutrients from food are absorbed from the small intestine into the bloodstream through fingerlike structures called villi. Villi, illustrated in Figure 35.6, increase the surface area of the small intestine, giving the small intestine approximately the same surface area as a tennis court.

Refer again to Figure 35.1 and Figure 35.2 to follow the movement of digested food through the digestive system. Once digestion is complete, the remaining food, now in a semiliquid form called chyme, moves into the large intestine. Chyme is made up of materials that cannot be digested or absorbed by villi in the small intestine.

Large intestine

The large intestine is the end portion of the digestive tract. It is about 1.5 m long and includes the colon, the rectum, and a small saclike appendage called the appendix. Although the appendix has no known function, it can become inflamed and swollen, resulting in appendicitis. If inflamed, the appendix likely will have to be removed surgically.

Some kinds of bacteria are normal in the colon. These bacteria produce vitamin K and some B vitamins that then become available to the body.

A primary function of the colon is to absorb water from the chyme. The indigestible material then becomes more solid and is called feces. Peristalsis continues to move feces toward the rectum, causing the walls of the rectum to stretch. This initiates a reflex that causes the final sphincter muscle to relax, and the feces are eliminated from the body through the anus. Refer to Table 35.1 to review the primary function of each structure of the digestive system and how long food usually remains in each structure as it is being digested.

Nutrition

Calories

Nutrition is the process by which a person takes in and uses food. Foods supply the building blocks and energy to maintain body mass. The daily input of energy from food should equal the amount of energy a person uses daily. A Calorie (with an uppercase C) is the unit used to measure the energy content of foods. A Calorie is equal to 1 kilocalorie, or 1000 calories (with a lowercase c). A calorie is the amount of heat needed to raise the temperature of 1 mL of water by 1°C.

The energy content of a food can be measured by burning the food and converting the stored energy to heat. Not all foods have the same energy content. The same mass of different foods does not always equal the same number of Calories. For example, one gram of carbohydrate or protein contains four Calories. One gram of fat contains nine Calories. Choosing foods wisely is important. To lose weight, more Calories must be used than consumed. The opposite is true to gain weight. In 2005, the United States Department of Agriculture released new guidelines for nutrition and suggested that people should become more active and use more Calories. Table 35.2 compares Calorie usage with different activities.

Carbohydrates

Cereal, pasta, potatoes, strawberries, and rice all contain a high proportion of carbohydrates. Recall from Chapter 6 that sugars, such as glucose, fructose, and sucrose, are simple carbohydrates that are found in fruits, soda pop, and candy. Complex carbohydrates are macromolecules such as starches, which are long chains of sugars. Foods such as those shown in Figure 35.7 have a high starch content, as do some vegetables.

Complex carbohydrates are broken down into simple sugars in the digestive tract. Simple sugars are absorbed through villi in the small intestine into blood capillaries and circulated throughout the body to provide energy for cells. Excess glucose is stored in the liver in the form of glycogen. Cellulose, sometimes called dietary fiber, is another complex carbohydrate found in plant foods. Although humans cannot digest fiber, it is important because fiber helps keep food moving through the digestive tract and helps with the elimination of wastes. Bran, whole-grain breads, and beans are good sources of fiber.

Reading Check Compare simple and complex carbohydrates.

Fats

In proper amounts, fats are an essential part of a healthful diet. Fats are the most concentrated energy source available to the body, and they are building blocks for the body. Fats also protect some internal organs and help maintain homeostasis by providing energy, and storing and transporting certain vitamins. However, not all fats are beneficial.

Recall from Chapter 6 that fats are classified according to their chemical structure as saturated or unsaturated.

Meats, cheeses, and other dairy products are sources of saturated fats. A diet high in saturated fats might result in high blood levels of cholesterol, which can lead to high blood pressure and other heart problems. Plants are the main source of unsaturated fats. They are not associated with heart disease, although excessive consumption of any type of fat can lead to weight gain.

A general rule is that saturated fats are solid and unsaturated fats are liquid at room temperature. The margarine in Figure 35.8 contains less saturated fat than butter. Fats are digested in the small intestine and broken down into fatty acids and glycerol. Fatty acids can be absorbed through the villi and circulated in the blood throughout the body.

Proteins

You have learned that proteins are basic structural components of all cells, and that amino acids are the building blocks of proteins. Enzymes, hormones, neurotransmitters, and membrane receptors are just a few important proteins in the body.

During the process of digestion, proteins in foods are broken down to their subunit amino acids. The amino acids are absorbed into the bloodstream and carried to various body cells. These body cells, through the process of protein synthesis, assemble the amino acids into proteins needed for body structures and functions.

Humans require 20 different amino acids for protein synthesis. The human body can synthesize 12 of the 20 amino acids needed for cellular function. Essential amino acids are the eight amino acids that must be included in a person’s diet. Animal products, such as meats, fish, poultry, eggs, and dairy products, are sources of all eight essential amino acids.

Vegetables, fruits, and grains contain amino acids, but no single plant food source contains all eight essential amino acids. However, certain combinations, such as the beans and rice shown in Figure 35.9, provide all of the essential amino acids.

Food Pyramid

In 2005, the United States Department of Agriculture published a new food pyramid, MyPyramid, which replaces the old pyramid that had been a symbol of good nutrition since 1992. Figure 35.10 shows the new pyramid. Notice that the orange and green sections are wider than the purple and yellow sections. The message of the pyramid is that a person needs more nutrients from grains and vegetables than from meats and oils.

Vitamins and Minerals

In addition to carbohydrates, fats, and proteins, your body needs vitamins and minerals to function properly. Vitamins are organic compounds that are needed in small amounts for metabolic activities.

Many vitamins help enzymes function well. Vitamin D is made by cells in your skin. Some B vitamins and vitamin K are produced by bacteria living in the large intestine. Sufficient quantities of most vitamins cannot be made by the body, but a well-balanced diet can provide the vitamins that are needed. Some vitamins that are fat-soluble can be stored in small quantities in the liver and fatty tissues of the body. Other vitamins are water-soluble and cannot be stored in the body. Foods providing an adequate level of these vitamins should be included in a person’s diet on a regular basis.

Minerals are inorganic compounds used by the body as building material, and they are involved with metabolic functions. For example, the mineral iron is needed to make hemoglobin. Recall from Chapter 34 that oxygen binds to hemoglobin in red blood cells and is delivered to body cells as blood circulates in the body. Calcium, another mineral, is an important component of bones and is involved with muscle and nerve functions.

Vitamins and minerals are essential parts of a healthy diet. Table 35.3 on the next page lists some important vitamins and minerals, their benefits, and some food sources that can provide these necessary nutrients.

Over-the-counter vitamins also are available. Taking more than the recommended daily allowance, however, can be dangerous and should not be done without consulting a doctor.

Nutrition Labels

Nutrition labels are provided on commercially packaged foods like those shown in Figure 35.11. These labels are based on a 2000-Calorie per day diet. Labels can be especially useful for monitoring fat and sodium intake, two nutrients that need to be consumed in moderation. The FDA requires that food labels list the following information:

•name of the food

•net weight or volume

•name and address of manufacturer, distributor, or packager

•ingredients

•nutrient content

The Endocrine System

Action of Hormones

The endocrine system is composed of glands and functions as a communication system. Endocrine glands produce hormones, which are released into the bloodstream and distributed to body cells. A hormone is a substance that acts on certain target cells and tissues to produce a specific response. Hormones are classified as steroid hormones and nonsteroid or amino acid hormones, based on their structure and mechanism of action.

Steroid hormones Estrogen and testosterone are two examples of steroid hormones. You will learn how each of these affect the human reproductive systems in Chapter 36. All steroid hormones work by causing the target cells to initiate protein synthesis, as illustrated in Figure 35.12.

Steroid hormones are soluble in lipids and therefore can diffuse through the plasma membrane of a target cell. Once inside a target cell, they bind to a receptor in the cell. The hormone and the receptor that are bound together bind to DNA in the nucleus, which activates specific genes.

Amino acid hormones Insulin and growth hormones are two examples of nonsteroid, or amino acid, hormones. As the name implies, these hormones are composed of amino acids. Amino acid hormones must bind to receptors found on the plasma membrane of a target cell because they cannot diffuse through the plasma membrane. Once the hormone binds to the receptor, the receptor activates an enzyme found on the inside of the membrane. This usually initiates a biochemical pathway, eventually causing the cell to produce the desired response, as illustrated in Figure 35.13.

Negative Feedback

Homeostasis in the body is maintained by internal feedback mechanisms called **negative feedback**. Negative feedback returns a system to a set point once it deviates sufficiently from that set point. As a consequence, the system varies within a particular range. You already might be familiar with an example of a negative feedback system in your own home, illustrated in Figure 35.14.

For example, the temperature in a house might be maintained at 21°C. The thermostat in the house detects the temperature, and when the temperature drops below 21°C, the thermostat sends a signal to the heat source, which turns it on and produces more heat. Soon the temperature rises above 21°C, and the thermostat sends a signal to the heat source to shut off. The heat source will not turn on again until the room temperature drops below 21°C and is detected by the thermostat. Because this process can go on indefinitely, negative feedback often is described as a “loop.”

Endocrine Glands and Their Hormones

The endocrine system includes all the glands that secrete hormones— pituitary, thyroid, parathyroid, and adrenal glands, the pancreas, ovaries, testes, pineal gland, and the thymus gland—illustrated in Figure 35.15.

Pituitary gland

The pituitary gland is situated at the base of the brain as illustrated in Figure 35.16. This gland is sometimes called the “master gland” because it regulates so many body functions. Despite its size, it is the most important endocrine gland. The pituitary gland secretes hormones that not only regulate many body functions but also regulates other endocrine glands, such as the thyroid gland, the adrenal glands, the testes, and the ovaries.

A few pituitary hormones act on tissues rather than on specific organs. Human growth hormone regulates the body’s physical growth by stimulating cell division in muscle and bone tissue. This hormone is especially active during childhood and **adolescence**.

Thyroid and parathyroid glands

Identify the thyroid and parathyroid glands in Figure 35.17. One hormone produced by the thyroid gland is thyroxine. Like hGH, thyroxine does not act on specific organs; rather, it causes cells of the body to have a higher rate of metabolism. The thyroid gland also produces calcitonin. Calcitonin is a hormone that is partly responsible for the regulation of calcium, an important mineral for bone formation, blood clotting, nerve function, and muscle contraction. Calcitonin lowers blood calcium levels by signaling bones to increase calcium absorption and also signaling the kidneys to excrete more calcium.

When blood calcium levels are too low, the parathyroid glands increase production of parathyroid hormone. Parathyroid hormone increases blood calcium levels by stimulating the bones to release calcium. The action of this hormone also causes the kidneys to reabsorb more calcium and the intestines to absorb more calcium from food.

The thyroid and parathyroid glands have opposite effects on blood calcium levels. However, as they work together, they maintain homeostasis.

Pancreas As discussed in Section 1, the pancreas has a crucial role in the production of enzymes that digest carbohydrates, proteins, and fats. The pancreas also secretes the hormones insulin and glucagon, which work together to maintain homeostasis, as illustrated in Figure 35.18. When blood glucose levels are high, the pancreas releases insulin. Insulin signals body cells, especially liver and muscle cells, to accelerate the conversion of glucose to glycogen, which is stored in the liver. When blood glucose levels are low, glucagon is released from the pancreas. Glucagon binds to liver cells, signaling them to convert glycogen to glucose and release the glucose into the blood.

Diabetes is a disease that results from the body not producing enough insulin or not properly using insulin. Type 1 diabetes, which usually appears in people by the age of 20, occurs when the body cannot produce insulin. Type 2 diabetes occurs in 70–80 percent of people diagnosed with diabetes, and usually occurs after the age of 40. It results from the cells of the body becoming insensitive to insulin. Complications from diabetes include coronary heart disease, retinal and nerve damage, and acidosis, or low blood pH. In either type of diabetes, the blood glucose levels must be monitored and maintained to prevent complications from the disease.

Adrenal glands Refer again to Figure 35.15. The adrenal glands are located just above the kidneys. The outer part of the adrenals is called the cortex, which manufactures the steroid hormone aldosterone and a group of hormones called glucocorticoids. Aldosterone primarily affects the kidneys and is important for reabsorbing sodium.

Cortisol, another glucocorticoid, raises blood glucose levels and also reduces inflammation.

The body has different mechanisms for responding to stress, such as those discussed in Chapter 33 concerning the role of the nervous system and the “fight or flight response.” The endocrine system also is involved with these types of responses. An “adrenaline rush” occurs when there seems to be a sudden burst of energy during a stressful situation. The inner portions of the adrenal glands secrete epinephrine, also called adrenaline, and norepinephrine. Together, these hormones increase heart rate, blood pressure, breathing rate, and blood sugar levels, all of which are important in increasing the activity of body cells.

Reproductive Systems

Human Male Reproductive System

Reproduction is necessary to ensure continuation of a species. The result of the human reproductive process is the union of an egg cell and a sperm cell, development of the fetus, and the birth of an infant. The organs, glands, and hormones of the male and female reproductive systems are instrumental in meeting this goal.

Figure 36.1 illustrates the male reproductive structures. The male reproductive glands are called the testes and are located outside of the body cavity in a pouch called the scrotum. A temperature lower than 37°C—the average body temperature—is required for the development of sperm.

Because the scrotum is located outside of the body cavity, it is several degrees cooler. This makes the environment suitable for the normal development of sperm.

Sperm cells

The male reproductive cells, called sperm cells, are produced in the testes. Follow the path that sperm travel in Figure 36.1 as you read about the structures in the male reproductive system. Sperm, like the one shown in Figure 36.2, develop in the testes in the seminiferous tubules. These tubules produce 100–200 million sperm each day. Next, sperm travel to the epididymis, a structure located on top of each testis where sperm mature and are stored. When the sperm are released from the body, they travel through the vas deferens, a duct leading away from the testis. There are two vas deferens, one leading away from each testis. The two vas deferens join together and enter the urethra, the tube that carries both semen and urine outside of the body through the penis.

Sperm require a nourishing fluid to survive long enough to fertilize an egg. Semen refers to the fluid that contains sperm, the nourishment, and other fluids from the male reproductive glands. The seminal vesicles contribute over half of the semen and secrete sugar into the f luid, which provides energy, other nutrients, proteins, and enzymes for the sperm. The prostate gland and bulbourethral glands contribute an alkaline solution to the fluid to neutralize acidic conditions sperm might encounter in the urethra and the female reproductive tract.

Male hormones Testosterone, which is made in the testes, is a steroid hormone that is necessary for the production of sperm. It also influences the development of male secondary sex characteristics that begin to appear at **puberty**, the period of growth when sexual maturity is reached. These characteristics include hair on the face and chest, broad shoulders, increased muscle development, and a deeper voice. Recall from Chapter 34 that the larynx contains the vocal cords. Because the vocal cords are longer in males than in females, the male voice is deeper. Later in life, testosterone might lead to a receding hairline or baldness.

Three hormones influence testosterone production.

Figure 36.3 indicates that the hypothalamus, discussed in Chapter 33, produces gonadotropin-releasing hormone (GnRH) that acts on the anterior pituitary gland. GnRH increases the production of follicle-stimulating hormone (FSH) and luteinizing hormone (LH). Both FSH and LH travel from the anterior pituitary gland through the bloodstream and to the testes. In the testes, FSH promotes the production of sperm, and LH stimulates the production and secretion of testosterone.

Levels of the male hormones are regulated by a negative feedback system that starts with the hypothalamus. Increased levels of testosterone in the blood are detected by cells in the hypothalamus and anterior pituitary, and the production of LH and FSH is decreased. When testosterone levels in the blood drop, the body responds by making more LH and FSH, as shown in Figure 36.3.

Human Female Reproductive System

A female’s reproductive system is specialized to produce egg cells, receive sperm, and provide an environment that is right for fertilization of an egg and the development of an embryo. Refer to Figure 36.4 as you read about the structures of the female reproductive system.

Egg cells The female reproductive cells, called egg cells, are produced in the ovaries, also illustrated in Figure 36.4. Each ovary is about the size of an almond. Inside each ovary are oocytes, which are immature eggs. Approximately once every 28 days, oocyte development is stimulated and an egg, called an ovum, is formed. The ovum is surrounded by follicle cells that provide protection and nourishment.

After the egg is released from the ovary, it travels through an oviduct, a tube that connects to the uterus. The uterus, or womb, is about the size of an average human fist and is where a baby develops before birth. The cervix at the lower end of the uterus has a narrow opening into the vagina, which leads to the outside of the female’s body.

Female hormones Estrogen and progesterone are steroid hormones made by cells in the ovaries. A female’s anterior pituitary gland also produces LH and FSH, which influence estrogen and progesterone levels in a negative feedback loop. Effects of LH and FSH are different in males and females. During **puberty**, an increase in estrogen levels causes a female’s breasts to develop, her hips to widen, and her amount of fat tissue to increase. During **puberty**, a female also will experience her first menstrual cycle—the events that take place each month in the human female to help prepare the female body for pregnancy.

Sex Cell Production

In Chapter 10, you learned that through meiosis, one cell in the male or female gonads—called testes and ovaries in humans—gives rise to four sex cells called gametes.

In the human male, sperm are produced from primary spermatocytes daily beginning at **puberty** and continuing throughout a male’s lifetime.

The production of eggs in the human female differs, as illustrated in Figure 36.5. A female is born with all of her eggs already beginning to develop. The genetic material has replicated in primary oocytes before birth, and the process of meiosis stops before the first meiotic division is completed. Then, once each menstrual cycle during the reproductive years, meiosis continues for a single developing oocyte. The resulting structures at the end of the first meiotic division of the oocyte are of unequal size. The smaller of the two structures is called a polar body. The chromosomes have segregated, but there is an unequal division of the cytoplasm. Most of the cytoplasm from the original cell goes to the cell that eventually will become the egg, and the polar body disintegrates.

During the second meiotic division, a similar process takes place. During **metaphase** of the second meiotic division, an egg ruptures through the ovary wall in a process called ovulation. The second meiotic division is completed only if fertilization takes place. Then, the zygote and the second polar body are formed, as shown in Figure 36.5. The second polar body also disintegrates.

Thus, the two meiotic divisions have yielded only one egg instead of four. If four eggs were formed and released midway through a female’s menstrual cycle, more multiple births would be expected.

The Menstrual Cycle

The length of the menstrual cycle can vary from 23 to 35 days, but it typically lasts around 28 days. The entire menstrual cycle can be divided into three phases: the flow phase, the follicular phase, and the luteal phase.

Flow phase Day one of the menstrual cycle is when menstrual flow begins. Menstrual flow is the shedding of blood, tissue fluid, mucus, and epithelial cells from the endometrium—the tissue that lines the uterus. The endometrium is where the embryo will implant if fertilization of the egg occurs. Because an embryo will need oxygen and nutrients, the endometrium has a good supply of blood. During menstruation, bleeding occurs because the outer layers of the endometrium tear away, and blood vessels that supply the endometrium are ruptured. Around day five, repair of the endometrial lining begins, and it becomes thicker as the cycle continues.

Follicular phase

During the menstrual cycle, changes also occur in the ovaries as a result of changing hormone levels, as illustrated in Table 36.1. At the beginning of a menstrual cycle, when estrogen levels are low, the anterior pituitary begins to increase production of LH and FSH. This stimulates a few follicles to begin to mature in the ovary.

Cells in the follicles then begin to produce estrogen and a small amount of progesterone. Inside each follicle is an immature egg—the oocyte. After about a week, usually only one of the growing follicles remains. This remaining follicle continues to grow and secrete estrogen, which keeps levels of LH and FSH low—an example of negative feedback.

On day 12, the high level of estrogen causes the anterior pituitary gland to release a surge of LH. This rapid release of a large quantity of LH causes the follicle to rupture, and ovulation occurs.

Luteal phase

After ovulation, the cells of the follicle change, and the follicle is transformed into a structure called the corpus luteum, illustrated in Figure 36.6. The corpus luteum slowly degenerates as the menstrual cycle continues. The corpus luteum produces high amounts of progesterone and some estrogen, which keep levels of LH and FSH low through negative feedback. Recall that FSH and LH stimulate new follicles to develop, but when these hormones are kept at low levels, new follicles are temporarily prevented from maturing. Toward the end of the cycle, the corpus luteum breaks down, no longer producing progesterone and estrogen. This results in a rapid decrease in progesterone and estrogen levels. A rapid decrease in hormones triggers detachment of the endometrium, and the flow phase of a new menstrual cycle will begin.

Human Development before Birth

Fertilization

Figure 36.7 shows the process of a sperm joining with an egg, which is called fertilization. Fertilization usually occurs in the upper portion of an oviduct near the ovary. In humans, sperm and eggs each are haploid, and each normally has 23 chromosomes. Fertilization brings these chromosomes together, restoring the diploid number of 46 chromosomes.

Sperm enter the vagina of the female’s reproductive system when strong muscular contractions ejaculate semen from the male’s penis during intercourse. Some sperm can exit through the penis before ejaculation without the male’s knowledge. As a result, sexual activity that does not result in ejaculation can lead to the release of sperm, fertilization, and pregnancy.

Sperm can survive for 48 hours in the female reproductive tract, but an unfertilized egg can survive for only 24 hours. Fertilization can happen if intercourse occurs anytime from a few days before ovulation to a day after ovulation. Overall, there is a relatively short time when fertilization can occur sucessfully. But, it is important to remember that the length of the menstrual cycle can vary and ovulation can occur at any time.

About 300 million sperm are released into the vagina during intercourse. Only several hundred of them will successfully complete the journey to the egg. Many never make it out of the vagina, some are attacked by white blood cells, and many simply die along the way. Only one sperm can fertilize an egg, but it takes several hundred to participate in the process.

A single sperm cannot penetrate the plasma membrane that surrounds the human egg. Recall from Chapter 7 that lysosomes are organelles that contain digestive enzymes. Notice in Figure 36.7 the tip of each sperm is a specialized lysosome called an acrosome. As each of several hundred sperm bombard the egg, the enzymes inside of the acrosome weaken the plasma membrane surrounding the egg. Eventually the plasma membrane becomes weak enough that one sperm can penetrate the egg. Immediately following this penetration, the egg forms a barrier to prevent other sperm from entering the now-fertilized egg.

Reading Check Explain why hundreds of sperm are necessary for fertilization to take place.

Early Development

Figure 36.8 illustrates the first week of human development. The fertilized egg, which is called a zygote, moves through the oviduct propelled by involuntary smooth muscle contractions and by the cilia lining the oviduct. Around 30 hours after fertilization, the zygote undergoes its first mitosis and cell division. Cell division continues, and by the third day, the embryo leaves the oviduct and enters the uterus. At this point, the embryo is described as a morula—a solid ball of cells.

By the fifth day, the morula has developed into a blastocyst, which can be described as a hollow ball of cells. The blastocyst attaches to the endometrium around the sixth day and is fully implanted by Day 10.

Figure 36.8 shows that the blastocyst is not completely hollow. Inside the blastocyst is a group of cells called the inner cell mass. The inner cell mass eventually will become the embryo. Sometimes, the inner cell mass splits, and identical twins might form.

Extraembryonic membranes In previous chapters, you learned the importance of the membranes that extend beyond an embryo called the extraembryonic membranes. You also learned about the development of the amniotic egg, and how this enabled animals to reproduce on land. Developing humans have these membranes, shown in Figure 36.9, but because humans and most other mammals develop inside the mother’s body, these membranes have somewhat different functions.

Early in human development, four extraembryonic membranes form.

These membranes are the amnion, the chorion, the yolk sac, and the allantois, as illustrated in Figure 36.9. The amnion is a thin layer that forms a sac around the embryo. Inside this sac is the amniotic fluid, which protects, cushions, and insulates the embryo. Outside of the amnion is the chorion, which, together with the allantois, contributes to the formation of the placenta. The yolk sac in humans does not contain any yolk but serves as the first site of red blood cell formation for the embryo.

The placenta

About two weeks after fertilization, tiny fingerlike projections of the chorion, called chorionic villi, begin to grow into the wall of the uterus. The placenta, the organ that provides food and oxygen and removes waste, begins to form and is fully formed by the tenth week. The placenta has two surfaces—a fetal side that forms from the chorion and faces the fetus, and a maternal side that forms from uterine tissue. When completely formed, the placenta is 15–20 cm in diameter, 2.5 cm thick, and has a mass of about 0.45 kg. The umbilical cord, a tube containing blood vessels, serves as the connection between the fetus and the mother. Figure 36.10 illustrates the connection between the mother and fetus.

The placenta regulates what passes from the mother to the fetus and from the fetus to the mother. Oxygen and nutrients can travel from the mother to the fetus. Alcohol, drugs, various other substances, and the human immunodeficiency virus (HIV) also can pass through the placenta to the developing fetus.

Metabolic waste products and carbon dioxide travel from the fetus to the mother. Because the mother and the fetus have their own separate circulatory systems, blood cells do not pass through the placenta. However, the mother’s antibodies pass to the fetus and help protect the newborn until its immune system is functioning.

Hormonal regulation during pregnancy

During the first week of development, the embryo begins to secrete a hormone, called human chorionic gonadotropin (hCG), which keeps the corpus luteum from degenerating. If the corpus luteum remains active, progesterone levels, and to a lesser extent estrogen levels, remain high. Remember from the previous section that the decline of progesterone triggers a new menstrual cycle. If levels of these hormones remain high, a new menstrual cycle will not begin. Two to three months into development, the placenta secretes enough progesterone and estrogen to maintain the proper conditions for pregnancy.

Three Trimesters of Development

On average, human development takes around 266 days from fertilization to birth. This time span is divided into three trimesters, each around three months long. During this time, many events take place. The zygote grows from a single cell into a baby that has trillions of cells. These cells develop into tissues and organs with specialized functions. Follow Figure 36.11, which shows different stages of human development during the first trimester.

The first trimester

In the first trimester, all tissues, organs, and organ systems begin to develop. During this time of development, the embryo is especially vulnerable to the effects of alcohol, tobacco, drugs, and other environmental influences, such as environmental pollutants. During the first two weeks of development, the mother might not realize she is pregnant because she has not missed a menstrual period yet. A lack of certain essential nutrients during this time might cause irreversible damage to the developing embryo. A few of the major causes of preventable birth defects are listed in Table 36.2.

At the end of eight weeks, the embryo is called a fetus. All of the organ systems have begun to form. By the end of the first trimester, the fetus can move its arms, fingers, and toes and make facial expressions. Fingerprints also are present.

Diagnosis in the Fetus

Many conditions can be diagnosed before a baby is born. Identifying certain conditions as early as possible increases the chance for proper medical treatment to help a newborn baby have the highest quality of life possible.

Ultrasound One way to identify conditions in the fetus is by using ultrasound, a procedure in which sound waves are bounced off the fetus. These sound waves are converted into light images that can be seen on a video monitor. Ultrasound can be used to determine if the fetus is growing properly, the position of the fetus in the uterus, and the gender of the fetus.

Amniocentesis and chorionic villus sampling Amniocentesis and chorionic villus sampling are prenatal tests. During amniocentesis—usually performed in the second trimester—a needle is inserted through the abdomen of the pregnant female, illustrated in Figure 36.12. Fluid from the amniotic sac is removed and analyzed. Tests that measure enzyme levels associated with certain conditions can be performed. Fetal cells can be examined by a karyotype or even by DNA analysis. Recall from Chapter 11 that a karyotype is a chart of chromosome pairs that is valuable in identifying unusual chromosome numbers or the sex of the fetus.

In chorionic villus sampling—usually performed during the first trimester—a small tube, called a catheter, is inserted through the vagina and cervix of the mother, illustrated in Figure 36.13. Cells from the chorion are removed and analyzed by karyotyping. The chromosomes in the cells of the chorion are identical to those of the cells in the fetus.

Birth, Growth, and Aging

Birth

Birth occurs in three stages: **dilation, expulsion, and the placental stage**, as shown in Figure 36.14. Just before giving birth, the posterior pituitary gland releases the hormone oxytocin, which stimulates involuntary muscles in the wall of the uterus to contract. This is the beginning of the birthing process called labor.

Another sign that the baby is going to be born is the dilation, or opening, of the cervix. The cervix must open to allow the baby to leave the uterus. Contractions of the uterus become stronger and more frequent, and at some point the amniotic sac tears. The amniotic fluid flows out of the vagina, which is sometimes described as the “water breaking.”

After a period of time that could be as short as a few hours or as long as a couple of days, the cervix fully dilates to around 10 cm. The uterine contractions are now very strong. The mother consciously will contract her abdominal muscles to help push the baby, usually head first, through the vagina in the **expulsion stage**. When the baby is out of the mother’s body, the umbilical cord is clamped and cut. A small piece of the cord still attached to the baby soon will dry up and fall off, forming the navel, or belly button.

Shortly after the baby is delivered, the placenta detaches from the uterus and leaves the mother’s body along with extraembryonic membranes. This is the placental stage of the birthing process.

Sometimes, complications prevent the baby from being born through the vagina. In these cases, an incision is made through the muscles of the mother’s abdomen and uterus, and the baby is removed from the mother’s body. This process is called a cesarean section.

During the first four weeks of life, the baby is called a newborn.

Human newborns vary in size. However, on average, a newborn human baby has a mass of 3300 g and is 51 cm long.

Growth and Aging

Humans go through many stages of growth during their lives. After you were born, you were in your infancy, but soon you will enter adulthood. You now are in a major development phase called **adolescence** that began with puberty and ends at adulthood.

Hormones, such as human growth hormone, thyroxine, and steroids, influence growth. Human growth hormone stimulates most areas of the body to grow as cells replicate by the process of mitosis. This hormone works by increasing the rates of protein synthesis and breakdown of fats. Thyroxine from the thyroid increases the overall metabolic rate, and is essential for growth to occur. Steroid hormones, such as estrogen and testosterone, also are important for growth. Recall from Chapter 35 that testosterone and estrogen pass through the plasma membrane and into the nucleus of a target cell. The hormones activate certain genes that promote the formation of proteins. In this way, testosterone, and to a lesser extent, estrogen, cause an increase in the size of cells.

Infancy

The first two years of life are known as infancy. Many changes take place during these years. An infant learns how to roll and crawl, grasp objects, and perform simple tasks. By the end of the first year, the infant likely is walking and might be uttering a few words. An enormous amount of mental development also occurs during these first two years.

In the first year, a baby typically grows about 25 cm in length and weighs three times more than when the baby was born. The child’s growth slows during the second year; children grow at a rate of around 6 cm per year until the beginning of puberty.

Childhood and adolescence Childhood is the period of growth and development that extends from infancy to adolescence. The child’s ability to reason and solve problems develops progressively during childhood. **Puberty** marks the beginning of **adolescence**, the period of growth between childhood and adulthood. **Puberty** usually begins between ages 8 to 13 in girls and ages 10 to 15 in boys.

In addition to the hormonal and sexual development that takes place during this time, other physical changes take place as well. An adolescent experiences a growth spurt—girls grow approximately 6–11 cm and boys grow approximately 7–13 cm—in one year. In girls, the hips become wider and the waist might become narrower. In boys, the shoulders usually become broader. At the end of **adolescence**, physical growth is complete, marking the beginning of adulthood. The transition between adolescence and adulthood can be hard to define because of physical, emotional, and behavioral changes.

Adulthood There are a number of theories on why people age, however, scientists do agree that the body goes through many changes as it ages, as shown in Figure 36.15. Physical changes perhaps are the most noticeable signs of aging, such as hair turning gray or white as pigment production declines. An individual might lose as much as two centimeters of height during the aging process because the discs between the vertebrae in the spine become flattened. Other changes include a decrease in muscle mass, a slowing of overall metabolism, and a decreased pumping ability of the heart. The skin loses its elasticity and sensory perceptions might diminish somewhat. In women, the ability to have children ends with menopause, and sperm production decreases in men.

Despite all of the potential changes of aging, many people continue to be very active physically and mentally as they grow older. Some older adults might even begin new careers. Anna Mary Robertson Moses, known as Grandma Moses, became a famous artist when she was in her late 70s.

Infectious Diseases

Pathogens Cause Infectious Disease

What do a cold and athlete’s foot have in common? They are both examples of an infectious disease. An infectious disease is a disease that is caused when a pathogen is passed from one organism to another, disrupting homeostasis in the organism’s body. Agents called pathogens are the cause of infectious disease. Some but not all types of bacteria, viruses, protozoans, fungi, and parasites are included in this group.

Recall from Chapter 18 that many types of these organisms are present in the world around us without causing infectious diseases. Your body benefits from organisms, such as certain types of bacteria and protozoans, that normally live in your intestinal and reproductive tracts. Other bacteria live on your skin, especially in the shafts of your hair follicles. These organisms keep pathogens from thriving and multiplying on your body.

Germ Theory and Koch’s Experiments

Before the invention of the microscope, people thought “something” passed from a sick person to a well person to cause an illness. Then, scientists discovered microorganisms and Louis Pasteur demonstrated that microorganisms from the air are able to grow in nutrient solutions, as discussed in Chapter 14. With the knowledge gained from these and other discoveries, doctors and scientists began to develop the germ theory. The **germ theory** states that some microorganisms are pathogens. However, scientists were not able to prove this theory until Robert Koch developed his postulates.

Identification of the first disease pathogen

In the late 1800s Robert Koch, a German physician, was studying anthrax— a deadly disease that affects cattle and sheep and can also affect people. Koch isolated bacteria, like those in Figure 37.1, from the blood of cattle that had died from anthrax. After growing the bacteria in the laboratory, Koch injected the bacteria into healthy cattle. These animals developed the disease anthrax. He then isolated bacteria from the blood of newly infected cattle and grew the bacteria in the laboratory. The characteristics of the two sets of cultures were identical indicating that the same type of bacteria caused the illness in both sets of cattle. Thus, Koch demonstrated that the bacteria he originally isolated were the cause of anthrax.

Koch’s postulates Koch established and published experimental steps known as Koch’s postulates, which are rules for demonstrating that an organism causes a disease. These steps are followed today to identify a specific pathogen as the agent of a specific disease. Follow the steps in Figure 37.2 as you read each of the four postulates.

Postulate 1: The suspected pathogen must be isolated from the diseased host in every case of the disease.

Postulate 2: The suspected pathogen must be grown in pure culture on artificial media in the laboratory. A pure culture is a culture that contains no other types of microorganisms—only the suspected pathogen.

Postulate 3: The suspected pathogen from the pure culture must cause the disease when placed in a healthy new host.

Postulate 4: The suspected pathogen must be isolated from the new host, grown again in pure culture, and shown to have the same characteristics as the original pathogen.

Some exceptions to Koch’s postulates do exist. Some pathogens, such as the pathogen that is believed to cause syphilis, cannot be grown in pure culture on artificial media. Artificial media are the nutrients that the bacteria need to survive and reproduce.

Pathogens are grown on this media in the laboratory. Also, in the case of viruses, cultured cells are needed because viruses cannot be grown on artificial media.

Spread of Disease

Of the large number of microorganisms that coexist with humans, only a few cause disease. The pathogens vary as much as the diseases themselves. Some might cause mild diseases, such as the common cold.

Others cause serious diseases, such as meningitis, an infection of the coverings of the brain and spinal cord. Table 37.1 lists some of the human infectious diseases you might know.

For a pathogen to spread, it must have both a reservoir and a way to spread. A disease reservoir is a source of the pathogen in the environment. Reservoirs might be animals, people, or inanimate objects, such as soil.

Human reservoirs Humans are the main reservoir for pathogens that affect humans. They might pass the pathogen directly or indirectly to other humans. Many pathogens might be passed on to other hosts before the person even knows he or she has the disease. An individual that is symptom-free but capable of passing the pathogen is called a carrier. Pathogens that cause colds, influenza (commonly referred to as the flu), and sexually transmitted diseases, such as human immunodeficiency virus (HIV), can be passed on without the person knowing he or she is infected.

Animal reservoirs

Other animals also are reservoirs of pathogens that can be passed to humans. Influenza and rabies are examples of human diseases listed in Table 37.1 that are caused by pathogens passed to humans from other animals. Influenza can infect pigs and various types of birds. Rabies is found in domestic dogs and many wild animals, such as bats, foxes, skunks, and raccoons.

Other reservoirs

Some bacteria normally found in the soil, such as tetanus bacteria, can cause disease in humans. The tetanus bacteria can cause a serious infection if it contaminates a deep wound in the body. Contamination of wounds by bacteria was a major cause of death during wars before the development of antibiotics and vaccinations.

Contaminated water or food is another reservoir of pathogens for human disease. One of the main purposes of sewage treatment plants is the safe disposal of human feces, which prevents contamination of the water supply by pathogens. Contaminated water used in growing or preparing food can transfer pathogens. Food also can become contaminated through contact with humans or insects such as flies.

Transmission of pathogens Pathogens mainly are transmitted to humans in four ways: direct contact, indirectly through the air, indirectly through touching contaminated objects, or by organisms called vectors that carry pathogens. Study Figure 37.3, which illustrates some of the ways pathogens can be transmitted to humans.

Direct Contact Direct contact with other humans is one of the major modes of transmission of pathogens. Diseases such as colds, infectious mononucleosis (commonly referred to as mono, or the “kissing disease”), herpes, and sexually transmitted diseases are caused by pathogens passed through direct contact.

Indirect contact

Some pathogens can be passed through the air. When a person with an infectious disease sneezes or coughs, pathogens can be passed along with the tiny mucus droplets. These droplets then can spread pathogens to another person or to an object.

Many organisms can survive on objects handled by humans. Cleansing of dishes, utensils, and countertops with detergents as well as careful hand-washing help prevent the spread of diseases that are passed in this manner. As a result, there are various food rules that restaurants must abide by that are based on preventing the spread of disease.

Vectors

Certain diseases can be transmitted by vectors. The most common vectors are arthropods, which include biting insects such as mosquitoes and ticks. Recall from Table 37.1 that Lyme disease, malaria, and West Nile virus are diseases that are passed to humans by vectors. The West Nile virus, which is currently spreading across the United States, is transmitted from horses and other mammals to humans by mosquitoes. Flies can transmit pathogens by landing on infected materials, such as feces, and then landing on materials handled or eaten by humans.

Reading Check Describe how diseases are spread to humans.

Symptoms of Disease

When you become ill with a disease such as the flu, why do you feel aches and pains, and why do you cough and sneeze? The pathogen, such as an influenza virus or bacteria, has invaded some of the cells of your body. The virus multiplies in the cells and leaves the cells either by exocytosis or by causing the cell to burst. Thus, the virus damages tissues and even kills some cells. When pathogenic bacteria invade the body, harmful chemicals or toxins might be produced. The toxins can be carried throughout the body via the bloodstream and damage various parts of the body.

Toxins produced by pathogens can affect specific organ systems. The tetanus bacteria produce a potent toxin that causes spasms in the voluntary muscles. The disease botulism usually is caused when a person consumes food in which the botulism bacteria have grown and produced a toxin. This toxin paralyzes nerves. The toxin from the botulism bacteria can cause disease in humans even when no bacteria are present.

Some types of bacteria, some protozoans, and all viruses invade and live inside cells, causing damage. Because the cells are damaged, they might die, causing symptoms in the host. Some disease symptoms, such as coughing and sneezing, are triggered by the immune system, as discussed later in this chapter. For a closer look at research on the immune system, examine Figure 37.4.

Disease Patterns

As outbreaks of diseases spread, certain patterns are observed. Agencies such as the community health departments, the Centers for Disease Control and Prevention (CDC), and the World Health Organization (WHO) continually monitor disease patterns to help control the spread of diseases. The CDC, with headquarters in Atlanta, Georgia, receives information from doctors and medical clinics and publishes a weekly report about the incidence of specific diseases, as shown in Figure 37.5. The WHO similarly watches disease incidence throughout the world.

Some diseases, such as the common cold, are known as endemic diseases because they continually are found in small amounts within the population. Sometimes, a particular disease will have a large outbreak in an area and afflict many people, causing an epidemic. If an epidemic is widespread throughout a large region, such as a country, continent, or the entire globe, it is described as pandemic.

Treating and Fighting Diseases

A medical professional may prescribe a drug to help the body fight a disease. One type of prescription drug is an antibiotic, which is a substance that can kill or inhibit the growth of other microorganisms. Recall from Chapter 20 that penicillin is secreted by the fungus Penicillium, which is shown in Figure 37.6. This fungus secretes the chemical penicillin to kill competing bacteria that grow on the fungal food source. Penicillin was isolated, purified, and first used in humans during World War II. Many other fungal secretions are used as antibiotics, such as erythromycin, neomycin, and gentamicin. Synthetic antibiotics also have been developed by pharmaceutical companies.

Chemical agents also are used in the treatment of protozoan and fungal diseases. Some antiviral drugs are used to treat herpes infections, influenza in the elderly, and HIV infections. Most viral diseases are handled by the body’s built-in defense system—the immune system.

Over the last 60 years, the wide-spread use of antibiotics has caused many bacteria to become resistant to particular antibiotics. Recall from Chapter 15 that natural selection occurs when organisms with favorable variations survive, reproduce, and pass their variations to the next generation. Bacteria in a population might have a trait that enables them to survive when a particular antibiotic is present. These bacteria can reproduce quickly and pass on the variation. Because reproduction can occur so rapidly in bacteria, the number of antibiotic-resistant bacteria in a population can increase quickly, too.

Antibiotic resistance of bacteria has presented the medical community with some problems with treating certain diseases. For example, penicillin was used effectively for many years to treat gonorrhea, a sexually transmitted disease, but now most strains of gonorrhea bacteria are resistant to penicillin. As a result, new drug therapies are needed to treat gonorrhea. Figure 37.7 shows the increase in gonorrhea resistance as the bacteria have gained resistance to treatment with antibiotics.

Another treatment problem is with staphylococcal disease: it is acquired in a hospital, which can result in skin infections, pneumonia, or meningitis. These staphylococci often are strains of bacteria that are resistant to many current antibiotics and can be difficult to treat.

The Immune System

Nonspecific Immunity

At the time of birth, the body has a number of defenses in the immune system that fight off pathogens. These defenses are nonspecific because they are not aimed at a specific pathogen. They protect the body from any pathogen that the body encounters.

The nonspecific immunity provided by the body helps to prevent disease. Nonspecific immunity also helps to slow the progression of the disease while the specific immunity begins to develop its defenses. Specific immunity is the most effective immune response, but nonspecific immunity is the first line of defense.

Barriers Like the strong walls of a fort, barriers are used by the body to protect against pathogens. These barriers are found in areas of the body where pathogens might enter.

Skin barrier

One of the simplest ways that the body avoids infectious disease is by preventing foreign organisms from entering the body. This major line of defense is the unbroken skin and its secretions. Recall that the skin contains layers of living cells covered by many layers of dead skin cells. By forming a barrier, the layers of dead skin cells help protect against invasion by microorganisms.

Many of the bacteria that live symbiotically on the skin digest skin oils to produce acids that inhibit many pathogens. Figure 37.8 shows some normal bacteria found on the skin that protect the skin from attack.

Chemical barriers Saliva, tears, and nasal secretions contain the enzyme lysozyme. Lysozyme breaks down bacterial cell walls, which kills pathogens.

Another chemical defense is mucus, which is secreted by many inner surfaces of the body. It acts as a protective barrier, blocking bacteria from sticking to the inner epithelial cells. Cilia, discussed in Chapter 7, also line the airway. Their beating motion sends any bacteria caught in the mucus away from the lungs. When the airway becomes infected, extra mucus is secreted, which triggers coughing and sneezing to help move the infected mucus out of the body.

A third chemical defense is the hydrochloric acid secreted in your stomach. In addition to its purpose in digestion, stomach acid kills many microorganisms found in food that could cause disease.

Nonspecific responses to invasion Even if an enemy gets through the walls of a town’s fort, defense doesn’t end. Similarly, the body has nonspecific immune responses to pathogens that get beyond its barriers.

Cellular defense

If foreign microorganisms enter the body, the cells of the immune system, shown in Table 37.2, defend the body. One method of defense is phagocytosis. White blood cells, especially neutrophils and macrophages, are phagocytic. Recall from Chapter 7 and Chapter 34 that phagocytosis is the process by which phagocytic cells surround and internalize the foreign microorganisms. The phagocytes then release digestive enzymes and other harmful chemicals from their lysosomes, destroying the microorganism.

A series of about 20 proteins that are found in the blood plasma are involved in phagocytosis. These proteins are called complement proteins. Complement proteins enhance phagocytosis by helping the phagocytic cells bind better to pathogens, activating the phagocytes and enhancing the destruction of the pathogen’s membrane, as illustrated in Figure 37.9. They are activated by materials that are in the cell wall of bacteria.

Interferon

When a virus enters the body, another cellular defense helps prevent the virus from spreading. Virus-infected cells secrete a protein called interferon. Interferon binds to neighboring cells and stimulates these cells to produce antiviral proteins which can prevent viral replication in these cells.

Inflammatory response

Another nonspecific response—the inflammatory response—is a complex series of events that involves many chemicals and immune cells that help enhance the overall immune response.

When pathogens damage tissue, chemicals are released by both the invader and cells of the body. These chemicals attract phagocytes to the area, increase blood flow to the infected area, and make blood vessels more permeable to allow white blood cells to escape into the infected area. This response aids in the accumulation of white blood cells in the area. Some of the pain, heat, and redness experienced in an infectious disease are the result of the inflammatory response.

Specific Immunity

Pathogens sometimes get past the nonspecific defense mechanisms. The body has a second line of defense that attacks these pathogens. Specific immunity is more effective, but takes time to develop. This specific response involves the tissues and organs found in the lymphatic system.

Lymphatic system

The lymphatic system, illustrated in Figure 37.10, includes organs and cells that filter lymph and blood and destroy foreign microorganisms. The lymphatic system also absorbs fat. Lymph is the fluid that leaks out of capillaries to bathe body cells. This fluid circulates among the tissue cells, is collected by lymphatic vessels, and is returned to the veins near the heart.

Lymphatic organs

The organs of the lymphatic system contain lymphatic tissue, lymphocytes, a few other cell types, and connective tissue. Lymphocytes are a type of white blood cell that is produced in red bone marrow. These lymphatic organs include the lymph nodes, tonsils, spleen, thymus gland, and diffused lymphatic tissue found in mucous membranes of the intestinal, respiratory, urinary, and genital tracts.

The lymph nodes filter the lymph and remove foreign materials from the lymph. The tonsils form a protective ring of lymphatic tissue between the nasal and oral cavities. This helps protect against bacteria and other harmful materials in the nose and mouth. The spleen stores blood and destroys damaged red blood cells. It also contains lymphatic tissue that responds to foreign substances in the blood. The thymus gland, which is located above the heart, plays a role in activating a special kind of lymphocyte called T cells. T cells are produced in the bone marrow, but they mature in the thymus gland.

B Cell Response

Antibodies are proteins produced by B lymphocytes that specifically react with a foreign antigen. An antigen is a substance foreign to the body that causes an imune response; it can bind to an anti-body or T cell. B lymphocytes, often called B cells, are located in all lymphatic tissues and can be thought of as antibody factories. When a portion of a pathogen is presented by a macrophage, B cells produce antibodies. Follow along in Figure 37.11, as you learn about how B cells are activated to produce antibodies.

When a macrophage surrounds, internalizes, and digests a pathogen, it takes a piece of the pathogen, which is called a processed antigen, and displays it on its membrane, as illustrated in Figure 37.11. In the lymphatic tissues, such as the lymph nodes, the macrophage, with the processed antigen on its surface, binds to a type of lymphocyte called a helper T cell. This process activates the helper T cell. This lymphocyte is called a “helper” because it activates antibody secretion in B cells and another type of T cell, which will be discussed later, that aids in killing microorganisms:

The activated helper T cell reproduces, binds processed antigens, and attaches to a B cell.

•The new helper T cells continue the process of binding anti-gens, attaching to B cells, and reproducing.

•Once an activated helper T cell binds to a B cell holding an antigen, the B cell begins to manufacture antibodies that specifically bind to the antigen.

•The antibodies can enhance the immune response by binding to microorganisms, making them more susceptible to phagocytosis and by initiating the inflammatory response, helping promote the nonspecific response.

B cells make many combinations of antibodies by using DNA that codes for the production of various heavy and light protein chains that make up antibodies as shown in Figure 37.12. Any heavy chain can combine with any light chain. If a B cell can make 16,000 different kinds of heavy chains and 1200 kinds of light chains, it can make 19,200,000 different types of antibodies (1200 × 16,000).

T Cell Response

Once helper T cells are activated by the presentation of an antigen by macrophages, helper T cells can also bind to and activate a group of lymphocytes called cytotoxic T cells. Activated cytotoxic T cells destroy pathogens and release chemicals called cytokines. Cytokines stimulate the cells of the immune system to divide and recruit immune cells to an area of infection. Cytotoxic T cells bind to pathogens, release a chemical attack, and destroy the pathogens. Multiple target cells can be destroyed by a single cytotoxic T cell. Figure 37.11 summarizes the activation of cytotoxic T cells.

Passive and Active Immunity

The body’s first response to an invasion by a pathogen is called the primary response. For example, if the viral pathogen that causes chicken pox enters the body, nonspecific and specific immune responses eventually defeat the foreign virus and the body is cleared of the pathogen.

One result of the specific immune response is the production of memory B and T cells. Memory cells are long-living cells that are exposed to the antigen during the primary immune response. These cells are ready to respond rapidly if the body encounters the same pathogen later. Memory cells protect the body by reducing the likelihood of developing the disease if exposed again to the same pathogen.

Passive immunity

Sometimes temporary protection against an infectious disease is needed. This type of temporary protection occurs when antibodies are made by other people or animals and are transferred or injected into the body. For example, passive immunity occurs between a mother and her child. Antibodies produced by the mother are passed through the placenta to the developing fetus and through breast milk to the infant child. These antibodies can protect the child until the infant’s immune system matures.

Antibodies developed in humans and animals that are already immune to a specific infectious disease are used to treat some infectious diseases in others. These antibodies are injected into people who have been exposed to that particular infectious disease. Passive immune therapy is available for people who have been exposed to hepatitis A and B, tetanus, and rabies.

Antibodies also are available to inactivate snake and scorpion venoms.

Active immunity Active immunity occurs after the immune system is exposed to disease antigens and memory cells are produced.

Active immunity can result from having an infectious disease or immunization. Immunization, also called vaccination, is the deliberate exposure of the body to an antigen so that a primary response and immune memory cells will develop. Table 37.3 lists some of the common immunizations offered in the United States. Immunizations contain killed or weakened pathogens, which are incapable of causing the disease.

Most immunizations include more than one stimulus to the immune system, given after the first immunization. These booster shots increase the immune response, providing further protection from the disease-causing organism.

Why are immunizations effective in preventing disease?

The characteristics of the secondary immune response, which is the response to a second exposure to an antigen, enable immunizations to be effective in preventing disease. Study the graph in Figure 37.13. Note that the secondary response to the antigen has a number of different characteristics. First, the response is more rapid than the primary response, as shown by the greater steepness in the portion of the curves plotted in red. Second, the overall response, both B and T cell response, is greater during the second exposure. Lastly, the overall memory lasts longer after the second exposure.

Immune System Failure

Defects in the immune system can result in an increased likelihood of developing infectious diseases as well as certain types of cancers. Some diseases can affect the immune system’s effectiveness. One such disease called acquired immunodeficiency syndrome (AIDS) results from an infection by human immunodeficiency virus (HIV). AIDS is a serious health problem worldwide.

In 2003, approximately 43,171 AIDS cases were diagnosed in the U.S. In 2003, 18,017 people died of AIDS in the U.S. In 2004, an estimated 40 million people globally were living with HIV infection.

Recall the important role that helper T cells play in specific immunity. HIV infects mainly helper T cells, also called CD4+ cells because these cells have a receptor on the outside of their plasma membrane. This CD4+ receptor is used by medical professionals to identify these cells, as illustrated in Figure 37.14.

In Chapter 18, you learned that HIV is an RNA virus that infects helper T cells. The helper T cells become HIV factories, producing new viruses that are released and infect other helper T cells. Over time, the number of helper T cells in an infected person decreases, making the person less able to fight disease. HIV infection usually has an early phase during the first six to twelve weeks while viruses are replicating in helper T cells.

The patient suffers symptoms such as night sweats and fever, but these symptoms are reduced after about eight to ten weeks. Then, the patient exhibits few symptoms for a period of time as long as ten years, but is capable of passing the infection through sexual intercourse or blood products. HIV is a secondary immunodeficiency disease, which means that the immune system of a previously healthy person fails.

Without antiviral drug therapy, the patient usually dies from a secondary infection from another pathogen after about ten years of being infected with HIV. Current antiviral drug therapy is aimed at controlling the replication of HIV in the body. The therapy is expensive and its long-term results are not known.

Noninfectious Disorders

Genetic Disorders

Not all diseases or body disorders are caused by pathogens. In Chapter 11 you read about some diseases caused by the inheritance of genes that do not function properly in the body, such as albinism, sickle cell anemia, Huntington disease, and hemophilia. There also are chromosomal disorders that result from abnormal chromosome numbers, such as Down syndrome. Many diseases are complex and have both an environmental and a genetic cause.

Coronary artery disease (CAD) is an example of a condition with environmental and genetic origin. This cardiovascular disease can result in blockage of arteries, as shown in Figure 37.15, that deliver oxygenated blood to the heart muscle. There is a genetic component that increases a person’s risk of developing CAD. Environmental factors such as diet contribute to the development of this complex disease.

Families with a history of CAD have a two to seven times greater risk of having CAD than families without a history of CAD. The exact genetic factors, however, are not known.

Reading Check Summarize the factors that cause coronary artery disease.

Degenerative Diseases

Some diseases called degenerative diseases are the result of a part of the body wearing out. This can be due to the natural aging process. However, in many cases, degenerative conditions occur sooner than would be expected in a person’s lifetime. Degenerative arthritis and arteriosclerosis, which is also referred to as hardening of the arteries, are examples of degenerative diseases. Degenerative arthritis is common. Most people have the disease by the age of 70 years, and the disease is found in almost all vertebrate animals.

Because many degenerative diseases also have a genetic component, some individuals might be more likely to develop a disease because of their genetic makeup.

Metabolic Diseases

Metabolic disease results from an error in a biochemical pathway. Some metabolic diseases result in the inability to digest specific amino acids or to regulate body processes. When the pancreas does not make the proper amount of insulin and glucose does not enter body cells normally, the condition known as Type 1 diabetes results. This results in high glucose levels in the bloodstream, which causes damage to many organs including the kidneys and the retinas of the eyes. Metabolic diseases can have a genetic component but also can involve environmental factors such as diet.

Cancer

In Chapter 9, you learned about cancer. Cancer is characterized by abnormal cell growth. Normally, certain regulatory molecules in the body control the beginning and end of the cell cycle. If this control is lost, abnormal cell growth results that could lead to various types of tumors, as shown in Figure 37.16. The abnormal cells can interfere with normal body functions and can travel throughout the body.

Cancer can develop in any body tissue or organ, including the blood cells. Cancer in the blood cells is called leukemia. Both genetic and environmental factors have been shown to cause cancer.

Cancer has been a disease that affects humans since ancient times. Egyptian mummies show evidence of bone cancer, and ancient Greek scientists described different kinds of cancer. Medieval manuscripts have reported details about cancer.

Inflammatory Diseases

Inflammatory diseases, such as allergies and autoimmunity, are diseases in which the body produces an inflammatory response to a common substance. Recall from Section 2 that infectious diseases also result in an inflammatory response. However, the inflammatory response in an infectious disease enhances the overall immune response. This inflammatory response is a result of the immune system removing bacteria or other microorganisms from the body. In inflammatory disease, the inflammatory response is not helpful to the body.

Allergies Certain individuals might have an abnormal reaction to environmental antigens. A response to environmental antigens is called an allergy. These antigens are called allergens and include things such as plant pollens, dust, dust mites, and various foods, as illustrated in Table 37.4. An individual becomes sensitized to the allergen and has localized inflammatory response with swollen itchy eyes, stuffy nose, sneezing, and sometimes a skin rash. These symptoms are the result of a chemical called histamine that is released by certain white blood cells. Antihistamine medications can help alleviate some of these symptoms.

Severe allergic reactions to particular allergens can result in anaphylactic shock, which causes a massive release of histamine. In anaphylactic shock, the smooth muscles in the bronchioles contract, which restricts air f low into and out of the lungs.

Common allergens that cause severe allergic reactions are bee stings, penicillin, peanuts, and latex, which is used to make balloons and surgical gloves. People who are extremely sensitive to these allergens require prompt medical treatment if exposed to these agents because anaphylactic reactions are life-threatening.

Allergies and anaphylactic reactions are known to have an inherited component.

Autoimmunity During the development of the immune system, the immune system learns not to attack proteins produced by the body.

However, some people develop autoimmunity and do form antibodies to their own proteins, which injures their cells.

Figure 37.17 shows the hands of a person with rheumatoid arthritis—a form of arthritis in which antibodies attack the joints. Degenerative arthritis, the form of arthritis that you read about earlier in the section on degenerative diseases, is not caused by autoimmunity. Rheumatic fever and lupus are other examples of autoimmune disorders. Rheumatic fever is an inflammation in which anti-bodies attack the valves of the heart. This can lead to damage to the heart valves and cause the valves to leak or not close properly as blood moves through the heart. Lupus is a disorder in which antibodies against cell nuclei, called antinuclear antibodies, are formed. As a result, many organs are vulnerable to attack by the body’s own immune system.
